# Supplementary material for: Application of New Efficient Hoveyda–Grubbs Catalysts Comprising an N→Ru Coordinate Bond in a Six-Membered Ring for the Synthesis of Natural Product-Like Cyclopenta[b]furo[2,3-c]pyrroles
Source: Molecules. 2020 Nov 17;25(22):5379. doi: 10.3390/molecules25225379 (PMC7709010; doi:10.3390/molecules25225379)
Supplement: Supplementary file 1 [file molecules-25-05379-s001.zip › ESI.pdf]

molecules

MDPI

Article

Application of new efficient Hoveyda–Grubbs catalysts comprising an N→Ru coordinate bond in a six-membered ring for the synthesis of natural product-like cyclopenta[*b*]furo[2,3-*c*]pyrroles

Alexandra S. Antonova [1], Marina A. Vinokurova [1], Pavel A. Kumandin [1], Natalia L. Merkulova [1], Anna A. Sinelshchikova [2], Mikhail S. Grigoriev [2], Roman A. Novikov [3], Vladimir V. Kouznetsov [4], Kirill B. Polyanskii*[,1] and Fedor I. Zubkov *[,1]

[1] Organic Chemistry Department, Faculty of Science, Peoples' Friendship University of Russia (RUDN University), Miklukho-Maklaya St., 6, Moscow, 117198, Russian Federation; alexandrasantonova@gmail.com (A.S.A.); marina.vin1999@yandex.ru (M.A.V.); pakumandin@gmail.com (P.A.K.); fraumerk@gmail.com (N.L.M.); 1236tgp@mail.ru (K.B.P.); fzubkov@sci.pfu.edu.ru (F.I.Z.)

[2] A. N. Frumkin Institute of Physical Chemistry and Electrochemistry, Russian Academy of Sciences, Leninsky pr. 31, bld. 4, Moscow, 119071, Russian Federation; asinelshchikova@gmail.com (A.A.S.); mickgrig@mail.ru (M.S.G.)

[3] V. A. Engelhardt Institute of Molecular Biology, Russian Academy of Sciences, Vavilov Street, 32, Moscow, 119991, Russian Federation; novikovfff@bk.ru (R.A.N.)

[4] Laboratorio de Química Orgánica y Biomolecular, CMN, Universidad Industrial de Santander, Parque Tecnológico Guatiguara, Km 2 vía refugio, Piedecuesta, A.A. 681011, Colombia; kouznet@uis.edu.co (V.V.K.)

* Correspondence: fzubkov@sci.pfu.edu.ru (F.I.Z.); 1236tgp@mail.ru (K.B.P.)

Contents

General remarks S2

1. *Typical procedure for preparation of 3-allyl-3a,6-epoxyisoindole-7-carboxylic acids* **5c** and **5h** S2

2. *Studying of conditions influence on 3-allyl-3a,6-epoxyisoindole-7-carboxylic acids* **(5a, 5b, 5g, 5i and 5m)** *isomer ratio*. S3

3. NMR spectrum data for previously obtained carboxylic acids 5k *and* **5p** S5

4. *Typical synthesis technique of methyl 3-allyl-3a,6-epoxyisoindole-7-carboxylates* **(6a–h)** *and 2-methylallyl -3a,6-epoxyisoindole-7-carboxylates* **(6i–p)** S6

5. *Technique for isomer separation of methyl 3-allyl-3a,6-epoxyisoindole-7-carboxylates* **6a, 6e, 6f** *and* **6p** S12

6. *General method for synthesis of methyl cyclopenta[b]furo[2,3-c]pyrrole-3-carboxylates* **7a, 7e, 7f** *and* **7p** S12

7. *General method for synthesis of methyl cyclopenta[b]furo[2,3-c]pyrrole-3-carboxylates* **(7a–h)** S12

8. ***General method for synthesis of methyl 7-methylcyclopenta[b]furo[2,3-c]pyrrole-3-carboxylate* 7p** S15

9. ***General method for synthesis of methyl 7-methylcyclopenta[b]furo[2,3-c]pyrrole-3-carboxylates* (7i–p)** S15

10. ***General method for synthesis of methyl 2,6a-divinylhexahydro-2H-furo[2,3-c]pyrrole-3-carboxylates* 8a *and* 8b** S17

11. ***Experimental part for X-ray analysis*** S18

12. ***NMR spectrum data*** S27

13. ***Refences*** S27

General remarks

All reagents were purchased from commercial suppliers (Acros Organics and Merck) and used without further purification. Metathesis reactions required solvents (CH_2Cl_2 and $CHCl_3$) pre-dried over anhydrous P_2O_5 and an inert atmosphere (dry Ar). Thin layer chromatography was carried out on aluminum backed silica pre-coated plates «Sorbfil» and «Alugram». The plates were visualized using a water solution of $KMnO_4$ or UV (254 nm). After extraction, organic layers were dried over anhydrous $MgSO_4$. IR spectra were obtained in KBr pellets or in thin films using an Infralum FT-801 IR-Fourier spectrometer. NMR spectra were run in deuterated solvents (>99.5 atom % D) on Jeol JNM-ECA 600 (600.1 MHz for ^{1}H and 150.9 MHz for ^{13}C) spectrometer for 2–5 % solutions in $CDCl_3$ or DMSO-d_6 at 22–23 °C using residual solvent signals (7.26/77.0 ppm for ^{1}H/^{13}C in $CDCl_3$ and 2.50/39.5 ppm for ^{1}H/^{13}C in DMSO-d_6) or TMS as an internal standard. $CFCl_3$ was used as an internal standard for ^{19}F NMR spectra.

Experimental Procedures

The initial homoallylamines **4a–p** were synthesized according to the previously described procedures [1,2]. Carboxylic acids **5a–p** were synthesized using the known methods [3–6]. Two acids **5c** and **5h**, that were not previously described, were obtained according to the following technique.

1. *Typical procedure for preparation of 3-allyl-3a,6-epoxyisoindole-7-carboxylic acids* 5c *and* 5h

A mixture of maleic anhydride (0.1 mol) and homoallylamine *a*) **5c** (0.1 mol) was stirred in CH_2Cl_2 (100 mL) for 3 d at −16 °C; *b*) **5h** was boiled in toluene (100 mL) for 3 h. The crystalline precipitate was filtered off, washed with toluene (2×50 mL), and dried in a vacuum desiccator to a constant mass.

1.1 *(3aRS,6RS,7RS)-3-Allyl-2-benzyl-1-oxo-1,2,3,6,7,7a-hexahydro-3a,6-epoxyisoindole-7-carboxylic acid* **(5c)**

White crystals, 57% (*trans*-**5cA**). M.p 143.7−145.8 °C. ^{1}H NMR (600.2 MHz, $CDCl_3$): *δ* 7.32 (2H, br.t, *J* = 7.5 Hz, H-3,5-Ph), 7.26-7.24 (3H, m, H-2,6-Ph and H-4-Ph), 6.52 (1H, d, *J* = 6.1 Hz, H-4), 6.39 (1H, dd, *J* = 1.5, 6.1 Hz, H-5), 5.76-5.29 (1H, m, H-3-Allyl), 5.23 (1H, d, *J* = 1.5 Hz, H-6), 5.21-5.17 (2H, m, H-3-Allyl), 5.06 (1H, d, *J* = 15.6 Hz, NCH_2*A*Ph), 4.13 (1H, d, *J* = 15.6 Hz, NCH_2*B*Ph), 3.88 (1H, dd, *J* = 4.0, 6.1 Hz, H-3), 2.98 and 2.83 (1H and 1H, br.d, *J* = 9.1 Hz, H-7 and H-7a), 2.61-2.51 (2H, m, H-1-Allyl) ppm. ^{13}C NMR (150.9 MHz, $CDCl_3$): *δ* 174.4, 172.2, 171.8, 171.2, 137.5, 136.0, 135.8, 135.6, 134.7, 133.6, 132.6, 132.2, 131.7, 131.3, 129.4, 129.1, 127.1, 125.3, 120.5, 119.3, 91.2, 90.4, 81.7, 81.5, 77.3, 77.1, 76.9, 60.9, 59.7, 51.2, 51.1, 46.1, 33.8, 32.6 ppm. IR spectrum: ν (CO_2H) =1748 cm^{-1}, ν (NC=O) =1687 cm^{-1}. HRMS (ESI-TOF) $[M+H]^+$ calcd for $C_{19}H_{20}NO_4$: 326.31314, found: 326.1325.

1.2 *(3aRS,6RS,7RS)-3-Allyl-2-(3-chloro-4-fluorophenyl)-1-oxo-1,2,3,6,7,7a-hexahydro-3a,6-epoxyisoindole-7-carboxylic acid* (**5h**)

White crystals, 90% (*trans*-**5hA**:*cis*-**5hB** = 57:43). M.p 148.5−150.3 °C. For *trans*-**5hA**: ^{1}H NMR (600.2 MHz, $CDCl_3$): *δ* 7.60 (1H, dd, *J* = 2.5, 6.1 Hz, H-2), 7.30 (1H, ddd, *J* = 2.5, 4.0, 8.6 Hz, H-6), 7.18-7.14 (1H, m, H-5), 6.65 (1H, d, *J* = 5.6 Hz, H-4), 6.53 (1H, dd, *J* = 2.0, 5.6 Hz, H-5), 5.79-5.68 (2H, m, H-2-Allyl), 5.26 (1H, d, *J* = 2.0 Hz, H-6), 5.23-5.17 (2H, m, H-3-Allyl), 4.59 (1H, dd, *J* = 4.0, 5.6 Hz, H-3), 3.08 and 2.83 (1H and 1H, d and d, *J* = 9.1 Hz, H-7 and H-7a), 2.65-2.62 (2H, m, H-1-Allyl) ppm. ^{19}F NMR (564.7 MHz, $CDCl_3$): −117.2 ppm. For *cis*-**5hB**: ^{1}H NMR (600.2 MHz, $CDCl_3$): *δ* 7.37 (1H, dd, *J* = 1.5, 7.1 Hz, H-2), 7.18-7.14 (2H, m, H-5, H-6), 6.49 (1H, d, *J* =5.8 Hz, H-5), 6.44 (1H, dd, *J* = 1.8, 5.8 Hz, H-5), 5.79-5.68 (2H, m, H-2-Allyl), 5.32 (1H, d, *J* = 1.8 Hz, H-6), 5.12-5.07 (2H, m, H-3-Allyl), 4.56 (1H, dd, *J* = 4.5, 11.1 Hz, H-3), 3.00 and 2.87 (1H and 1H, d and d, *J* = 9.1 Hz, H-7 and H-7a), 2.48-2.44 (1H, m, H*A*-1-Allyl), 2.38-2.35 (1H, m, H*B*-1-Allyl) ppm. ^{19}F NMR (564.73 MHz, $CDCl_3$): −116.4 ppm. ^{13}C NMR (150.9 MHz, $CDCl_3$): *δ* 174.3, 174.1, 171.8, 171.2, 157.7, 155.4, 137.6, 136.0, 135.9, 133.7, 133.6, 132.8, 132.0, 131.1, 128.2, 126.5, 125.9, 125.8, 123.9, 123.8, 121.7, 121.6, 121.5, 120.6, 119.5, 117.1, 117.0, 116.9, 91.2, 90.4, 81.7, 81.6, 61.1, 59.9, 51.1, 51.0, 46.1, 46.0, 33.9, 32.7 ppm. IR spectrum: ν (COOH) =1763 cm^{-1}, ν (NC=O) =1692 cm^{-1}. HRMS (ESI-TOF) $[M+H]^+$ calcd for $C_{18}H_{16}FClNO_4$: 364.0674, found: 364.0662.

2. *Studying of conditions influence on 3-allyl-3a,6-epoxyisoindole-7-carboxylic acids* (5a, 5b, 5g, 5i and 5m) *isomer ratio.*

A mixture of maleic anhydride (0.1 mol) and amine **4a**, **4b**, **4g**, **4i** or **4m** (0.1 mol) was *a*) stirred in dichloromethane (100 mL) for 72 h at −16 °C; *b*) stirred in dichloromethane (100 mL) for 72 h at 25 °C; *c*) stirred in toluene (100 mL) for 72 h at 25 °C; *d*) boiled in toluene (100 mL) for 1 h; *e*) stirred in acetonitrile (100 mL) for 72 h at 25 °C; *f*) boiled in acetonitrile (100 mL) for 1 h; *g*) boiled in benzene (100 mL) for 1 h. In all cases, the formed crystalline precipitates were filtered off, washed with toluene (2 × 50 mL) and dried at 80 °C to a constant mass. The isomer ratio was detected using ^{1}H NMR spectroscopy (see Table 2 of the main part). All obtained acids **5a**, **5b**, **5g**, **5i** and **5m** were described previously [3−6].

2.1 *(3aRS,6SR,7RS)-3-Allyl-1-oxo-2-phenyl-1,2,3,6,7,7a-hexahydro-3a,6-epoxyisoindole-7-carboxylic acid* (**5a**)

White crystals, M.p 184.5−185.3 °C. For *trans*-**5aA**: ^{1}H NMR (600.2 MHz, $CDCl_3$): *δ* 7.44-7.37 (3H, m, H-Ar), 7.29-7.22 (2H, m, H-Ar), 6.62 (1H, d, *J* = 6.1 Hz, H-4), 6.49 (1H, dd, *J* = 1.5, 6.1 Hz, H-5), 5.81-5.68 (1H, m, H-2-Allyl), 5.25 (1H, d, *J* = 1.5 Hz, H-6), 5.20-5.04 (2H, m, H-3-Allyl), 4.64 (1H, *J* = 4.0, 6.1 Hz, H-3), 3.11 and 2.84 (1H and 1H, d and d, *J* = 9.1 Hz, H-7 and H-7a), 2.63-2.61 (2H, m, H-1-Allyl)

ppm. For *cis*-**5aB**: ^{1}H NMR (600.2 MHz, $CDCl_3$): δ 7.44-7.37 (3H, m, H-Ar), 7.29-7.22 (2H, m, H-Ar), 6.46 (1H, d, *J* = 6.1 Hz, H-4), 6.38 (1H, dd, *J* = 1.5, 6.1 Hz, H-5), 5.81-5.68 (1H, m, H-2-Allyl), 5.31 (1H, d, *J* = 1.5 Hz, H-6), 5.20-5.04 (2H, m, H-3-Allyl), 4.61 (1H, dd, *J* = 4.0, 10.6 Hz, H-3), 3.04 and 2.86 (1H and 1H, d and d, *J* = 9.1 Hz, H-7 and H-7a), 2.47-2.35 (2H, m, H-1-Allyl) ppm. ^{13}C NMR (150.9 MHz, $CDCl_3$): δ 174.1, 173.9, 172.1, 171.4, 137.4, 136.8, 136.0, 135.7, 133.6, 132.3, 131.4, 129.2, 129.1, 127.1, 126.5, 125.8, 124.3, 120.2, 119.0, 91.3, 90.5, 81.7, 81.6, 61.1, 59.9, 51.0, 50.9, 46.2, 33.7, 32.6 ppm. IR spectrum: ν (CO_2H) =1753 cm^{-1}, ν (NC=O) =1676 cm^{-1}. HRMS (ESI-TOF) $[M+H]^+$ calcd for $C_{18}H_{18}NO_4$: 312.3370, found: 312.3359.

2.2 *(3aRS,6SR,7RS)-3-Allyl-1-oxo-2-(m-tolyl)-1,2,3,6,7,7a-hexahydro-3a,6-epoxyisoindole-7-carboxylic acid* **(5b)**

White crystals, M.p 140.5–141.5 °C. For *trans*-**5bA**: ^{1}H NMR (600.2 MHz, $CDCl_3$): δ 7.30-7.18 (3H, m, H-Ar), 7.11-7.03 (2H, m, H-Ar), 6.64 (1H, *J* = 5.6 Hz, H-4), 6.51 (1H, dd, *J* = 1.6, 5.6 Hz, H-5), 5.83-5.77 (1H, m, H-2-Allyl), 5.28 (1H, d, *J* = 1.6 Hz, H-6), 5.22-5.18 (2H, m, H-3-Allyl), 4.61 (1H, dd, *J* = 4.0, 6.1 Hz, H-3), 3.09 and 2.86 (1H and 1H, d and d, *J* = 9.1 Hz, H-7 and H-7a), 2.66-2.59 (2H, m, H-1-Allyl), 2.35 (3H, s, Me-Ar) ppm. For *cis*-**5bB**: ^{1}H NMR (600.2 MHz, $CDCl_3$): δ 7.30-7.18 (3H, m, H-Ar), 7.11-7.03 (2H, m, H-Ar), 6.47 (1H, d, *J* = 5.6 Hz, H-4), 6.41 (1H, dd, *J* = 1.6, 5.6 Hz, H-5), 5.83-5.77 (1H, m, H-2-Allyl), 5.35 (1H, d, *J* = 1.6 Hz, H-6), 5.10-5.04 (2H, m, H-3-Allyl), 4.58 (1H, dd, *J* = 4.5, 11.0 Hz, H-3), 3.03 and 2.89 (1H and 1H, d and d, *J* = 9.1 Hz, H-7 and H-7a), 2.47-2.38 (2H, m, H-1-Allyl), 2.37 (3H, s, Me-Ar) ppm. ^{13}C NMR (150.9 MHz, $CDCl_3$): δ 174.0, 173.2, 172.7, 137.0, 136.0, 135.5, 135.3, 133.7, 132.5, 131.8, 128.8, 127.7, 127.5, 119.8, 118.9, 91.8, 90.5, 81.4, 58.1, 57.7, 50.9, 50.3, 45.7, 45.5, 44.8, 44.4, 33.6, 32.3 ppm. IR spectrum: ν (CO_2H) =1764 cm^{-1}, ν (NC=O) =1682 cm^{-1}. HRMS (ESI-TOF) $[M+H]^+$ calcd for $C_{19}H_{20}NO_4$: 326.3640, found: 326.3648.

2.3 *(3aRS,6SR,7RS)-3-Allyl-2-(4-iodophenyl)-1-oxo-1,2,3,6,7,7a-hexahydro-3a,6-epoxyisoindole-7-carboxylic acid* **(5g)**

White crystals, M.p 146.6–148.3 °C. For *trans*-**5gA**: ^{1}H NMR (600.2 MHz, $CDCl_3$): δ 7.72-7.68 (2H, m, H-Ar), 7.24-7.22 (2H, m, H-Ar), 6.63 (1H, d, *J* = 5.6 Hz, H-4), 6.51 (1H, dd, *J* = 1.4, 5.6 Hz, H-5), 5.78-5.68 (1H, m, H-2-Allyl), 5.25 (1H, d, *J* = 1.4 Hz, H-6), 5.21-5.15 (2H, m H-3-Allyl), 4.63 (1H, dd, *J* = 4.0, 6.1 Hz, H-3), 3.07 and 2.83 (1H and 1H, d and d, *J* = 9.1 Hz, H-7 and H-7a), 2.65-2.58 (2H, m, H-1-Allyl) ppm. For *cis*-**5gB**: ^{1}H NMR (600.2 MHz, $CDCl_3$): δ 7.72-7.68 (2H, m, H-Ar), 7.05-7.03 (2H, m, H-Ar), 6.47 (1H, d, *J* = 5.6 Hz, H-4), 6.41 (1H, dd, *J* = 1.4, 5.6 Hz, H-5), 5.78-5.68 (1H, m, H-2-Allyl), 5.31 (1H, d, *J* = 1.4 Hz, H-6), 5.11-5.06 (2H, m, H-3-Allyl), 4.58 (1H, dd, *J* = 4.5, 10.1 Hz, H-3), 2.98 and 2.85 (1H and 1H, d and d, *J* = 9.1 Hz, H-7 and H-7a), 2.46-2.37 (2H, m, H-1-Allyl) ppm. ^{13}C NMR (150.9 MHz, $CDCl_3$): δ 174.1, 173.9, 171.6, 171.1, 138.3, 138.2, 137.5, 136.7, 136.0, 135.8, 133.5, 132.1, 131.1, 127.4, 125.6, 120.4, 119.3, 91.7, 91.1, 90.7, 90.3, 81.7, 81.5, 60.6, 59.5, 51.1, 51.0, 46.1, 33.7, 32.5 ppm. IR spectrum: ν (CO_2H) =1752 cm^{-1}, ν (NC=O) =1676 cm^{-1}. HRMS (ESI-TOF) $[M+H]^+$ calcd for $C_{18}H_{17}NO_4I$: 438.2335, found: 438.2342.

2.4 *(3aRS,6SR,7RS)-3-(2-Methylallyl)-1-oxo-2-phenyl-1,2,3,6,7,7a-hexahydro-3a,6-epoxyisoindole-7-carboxylic acid* **(5i)**

White crystals, M.p 184.5–185.3 °C. For *trans*-**5iA**: ^{1}H NMR (600.2 MHz, $CDCl_3$): δ 7.45-7.36 (3H, m, H-Ar), 7.31-7.23 (2H, m, H-Ar), 6.59 (1H, d, *J* = 5.6 Hz, H-4), 6.47 (1H, dd, *J* = 1.5, 5.6 Hz, H-5), 5.30 (1H, d, *J* = 1.5 Hz, H-6), 4.92 (2H, br.s, H-3-Allyl), 4.75 (1H, dd, *J* = 3.5, 10.6 Hz, H-3), 3.16 and 2.89 (1H and 1H, d and d, *J* = 9.1 Hz, H-7 and H-7a), 2.60 (1H, dd, *J* = 2.5, 15.6 Hz, H-1-Allyl-*A*), 2.45-2.39 (1H, m, H-1-Allyl-*B*), 1.73 (3H, s, Me-2) ppm. For *cis*-**5iB**: ^{1}H NMR (600.2 MHz, $CDCl_3$): δ 7.45-7.36 (3H,

m, H-Ar), 7.31-7.23 (2H, m, H-Ar), 6.47 (1H, d, *J* = 5.6 Hz, H-4), 6.40 (1H, dd, *J* = 1.5, 5.6 Hz, H-5), 5.38 (1H, d, *J* = 1.5 Hz, H-6), 4.84 (2H, br.s, H-3-Allyl), 4.79 (1H, dd, *J* = 4.0, 11.1 Hz, H-3), 3.02 and 2.90 (1H and 1H, d and d, *J* = 9.1 Hz, H-7 and H-7a), 2.45-2.39 (1H, m, H-1-Allyl-*A*), 2.32 (1H, dd, *J* = 3.5, 13.5 Hz, H-1-Allyl-*B*), 1.73 (3H, s, Me-2) ppm. ^{13}C NMR (150.9 MHz, $CDCl_3$): δ 173.4, 172.3, 171.5, 140.4, 136.9, 136.7, 135.9, 135.5, 134.1, 129.3, 129.2, 127.4, 126.8, 125.8, 124.2, 114.5, 113.9, 91.7, 90.8, 82.0, 81.8, 59.8, 59.1, 50.8, 50.2, 46.8, 46.6, 37.8, 34.9, 23.2, 23.0 ppm. IR spectrum: ν (CO_2H) =1765 cm^{-1}, ν (NC=O) =1677 cm^{-1}. HRMS (ESI-TOF) [M+H]$^+$ calcd for $C_{19}H_{20}NO_4$: 326.3640, found: 326.3652.

2.5 *(3aRS,6SR,7RS)-2-Benzyl-3-(2-methylallyl)-1-oxo-1,2,3,6,7,7a-hexahydro-3a,6-epoxyisoindole-7-carboxylic acid* **(5m)**

White crystals, M.p 194.4–196.0 °C. For *trans*-**5mA**: ^{1}H NMR (600.2 MHz, $CDCl_3$): δ 7.35-7.22 (4H, m, H-Ar), 6.48 (1H, d, *J* = 5.6 Hz, H-4), 6.35 (1H, dd, *J* = 1.5, 5.6 Hz, H-5), 5.22 (1H, d, *J* = 1.5 Hz, H-6), 5.10 (1H, d, *J* = 15.6 Hz, CH_2*A*-Ph), 4.92 and 4.82 (1H and 1H, br.s and br.s, H-3-Allyl), 4.09 (1H, d, *J* = 15.6 Hz, CH_2*B*-Ph), 3.97 (1H, dd, *J* = 5.6, 8.1 Hz, H-3), 3.07 and 2.84 (1H and 1H, d and d, *J* = 9.1 Hz, H-7 and H-7a), 2.54-2.36 (2H, m, H-1-Allyl), 1.71 (3H, s, Me-2) ppm. For *cis*-**5iB**: ^{1}H NMR (600.2 MHz, $CDCl_3$): δ 7.35-7.22 (4H, m, H-Ar), 6.30 (1H, d, *J* = 5.6 Hz, H-4), 6.27 (1H, dd, *J* = 1.5, 5.6 Hz, H-5), 5.27 (1H, d, *J* = 1.5 Hz, H-6), 4.96 (1H, d, *J* = 15.6 Hz, CH_2*A*-Ph), 4.80 (2H, br.s, H-3-Allyl), 4.21 (1H, d, *J* = 15.6 Hz, CH_2*B*-Ph), 4.11 (1H, dd, *J* = 5.6, 9.1 Hz), 2.94 and 2.82 (1H and 1H, d and d, *J* = 9.1 Hz, H-7 and H-7a), 2.54-2.36 (2H, m, H-1-Allyl), 1.58 (3H, s, Me-2) ppm. ^{13}C NMR (150.9 MHz, $CDCl_3$): δ 173.7, 173.5, 172.9, 140.7, 140.0, 136.7, 136.0, 135.8, 135.5, 135.3, 133.9, 128.9, 128.9, 128.9, 127.7, 127.7, 114.8, 114.3, 92.4, 90.9, 81.9, 81.7, 55.7, 56.5, 50.9, 49.5, 46.1, 45.2, 44.6, 38.4, 34.8, 22.9, 22.7 ppm. IR spectrum: ν (CO_2H) =1759 cm^{-1}, ν (NC=O) =1680 cm^{-1}. HRMS (ESI-TOF) [M+H]$^+$ calcd for $C_{19}H_{20}NO_4$: 340.3910, found: 340.3927.

3. *NMR spectrum data for previously obtained carboxylic acids* 5k *and* 5p

3.1 (3a*RS*,6*SR*,7*RS*)-3-(2-Methylallyl)-1-oxo-2-(p-tolyl)-1,2,3,6,7,7a-hexahydro-3a,6-epoxyisoindole-7-carboxylic acid **(5k)**

White crystals (*trans*-**5kA**:*cis*-**5kB** = 51:49). M.p 167.4–168.3 °C. For *trans*-**5kA**: ^{1}H NMR (600.2 MHz, $CDCl_3$): δ 7.20-7.13 (4H, m, H-Ar), 6.64 (1H, d, *J* = 6.1 Hz, H-4), 6.45 (1H, dd, *J* = 1.5, 6.1 Hz, H-5), 5.18 (1H, d, *J* = 1.5 Hz, H-6), 4.72-4.68 (1H, m, H-3), 4.92 and 4.84 (1H and 1H, br.s, H-3-MethAllyl), 3.74 (3H, s, OMe), 3.07 and 2.79 (1H and 1H, d and d, *J* = 9.1 Hz, H-7 and H-7a), 2.59 (1H, br.d, *J* = 16.2 Hz, H-1-Allyl), 2.41 (1H, dd, *J* = 10.6, 16.2 Hz, H-1-MethAllyl), 2.32 (3H, s, Me-4), 1.74 (3H, s, Me-2) ppm. For *cis*-**5kB**: ^{1}H NMR (600.2 MHz, $CDCl_3$): δ 7.31-7.30 (2H, m, 2,6-Ph), 7.20-7.13 (2H, m, 3,5-Ph), 6.50 (1H, d, *J* = 6.1 Hz, H-4), 6.37 (1H, dd, *J* = 1.5, 6.1 Hz, H-5), 5.23 (1H, d, *J* = 1.5 Hz, H-6), 4.72-4.68 (1H, m, H-3), 4.92 and 4.80 (1H and 1H, br.s, H-3-MethAllyl), 3.80 (3H, s, OMe), 2.92 and 2.80 (1H and 1H, d and d, *J* = 9.1 Hz, H-7 and H-7a), 2.48 (1H, dd, *J* = 11.0, 13.6 Hz, H-1-MethAllyl), 2.34 (3H, s, Me-4), 2.31-2.30 (1H, m, H-1-MethAllyl), 1.74 (3H, s, Me-2) ppm. ^{13}C NMR (150.9 MHz, $CDCl_3$): δ 172.4, 170.4, 140.7, 140.4, 136.5, 136.4, 135.0, 134.5, 134.0, 129.8, 129.7, 125.7, 123.9, 114.3, 113.6, 90.3, 80.9, 80.8, 59.2, 58.5, 52.2, 51.4, 50.4, 45.5, 37.8, 35.0, 23.2, 22.9, 21.1, 21.0 ppm.

3.2 *(3aRS,6SR,7RS)-2-(4-Bromophenyl)-3-(2-methylallyl)-1-oxo-2-(p-tolyl)-1,2,3,6,7,7a-hexahydro-3a,6-epoxyisoindole-7-carboxylic acid* **(5p)**

White crystals (*trans*-**5pA**:*cis*-**5pB** = 76:24). M.p 170.0–171.9 °C. For *trans*-**5pA**: ^{1}H NMR (600.2 MHz, $CDCl_3$): δ 7.49-7.47 (2H, m, 3,5-Ph), 7.37-7.35 (2H, m, 2,6-Ph), 6.62 (1H, d, *J* = 6.1 Hz, H-4), 6.47 (1H,

dd, *J* = 1.6, 6.1 Hz, H-5), 5.18 (1H, d, *J* = 1.6 Hz, H-6), 4.94 and 4.85 (1H and 1H, br.s, H-3-MethAllyl), 4.75-4.70 (1H, m, H-3), 3.75 (3H, s, OMe), 3.07 and 2.81 (1H and 1H, d and d, *J* = 9.1 Hz, H-7 and H-7a), 2.57 (1H, dd, *J* = 3.0, 16.1 Hz, H-1-MethAllyl), 2.48-2.39 (1H, m, H-1-MethAllyl), 1.75 (3H, br.s, Me-2) ppm. For *cis*-**5pB**: ^{1}H NMR (600.2 MHz, $CDCl_3$): δ 7.53-7.51 (2H, m, 3,5-Ph), 7.17-7.15 (2H, m, 2,6-Ph), 6.50 (1H, d, *J* = 6.1 Hz, H-4), 6.39 (1H, dd, *J* = 1.6, 6.1 Hz, H-5), 5.22 (1H, d, *J* = 1.6 Hz, H-6), 4.86 and 4.81 (1H and 1H, br.s, H-3-MethAllyl), 4.75-4.70 (1H, m, H-3), 3.80 (3H, s, OMe), 2.91 and 2.81 (1H and 1H, d and d, *J* = 9.1 Hz, H-7 and H-7a), 2.48-2.39 (1H, m, H-1-MethAllyl), 2.34 (1H, dd, *J* = 3.5, 14.1 Hz, H-1-MethAllyl), 1.75 (3H, br.s, Me-2) ppm. ^{13}C NMR (150.9 MHz, $CDCl_3$): δ 172.4, 172.2, 170.5, 170.1, 140.4, 140.1, 136.7, 136.3, 135.8, 134.3, 132.3, 132.2, 127.2, 125.0, 120.1, 119.1, 114.5, 114.0, 99.1, 90.3, 81.1, 81.0, 59.0, 58.3, 52.4, 51.4, 50.6, 45.7, 45.7, 37.9, 34.9, 23.3, 23.1 ppm.

4. *Typical synthesis technique of methyl 3-allyl-3a,6-epoxyisoindole-7-carboxylates* **(6a–h)** *and 2-methylallyl-3a,6-epoxyisoindole-7-carboxylates* **(6i–p)**

Crystalline substances **5a–p** (10.0 mmol) dissolved in methanol (100 mL) were boiled with a catalytic amount of concentrated sulfuric acid (0.05 mL) for 3 h. After cooling, the reaction mixtures were poured into water (150 mL) and the obtained precipitates were filtered off, washed with water (3 × 100 mL) and dried in the air to give the pure target esters **6a**–**p**. The obtained solids were washed with Et_2O (2 × 30 mL) and dried in the air to give the pure esters **6a**–**h, 6j**–**k, 6m**–**p**. In two cases **6i** and **6m** the solids were not formed after addition of water, then the reaction mixtures were extracted with CH_2Cl_2 (2 × 150 mL). The combined organic layers were dried over anhydrous magnesium sulfate and the solvent was removed under lower pressure (see Table 2 for the yields and the isomer ratio).

4.1 *Methyl (3aRS,6SR,7RS)-3-allyl-1-oxo-2-phenyl-1,2,3,6,7,7a-hexahydro-3a,6-epoxyisoindole-7-carboxylate* **(6a)**

White crystals, 85% (*trans*-**6aA**:*cis*-**6aB** = 79:21). M.p 128.5–129.8 °C. For *trans*-**6aA**: ^{1}H NMR (600.2 MHz, $CDCl_3$): δ 7.44-7.37 (4H, m, H-2,6,3,5-Ph), 7.27 (1H, br.t, *J* = 7.3 Hz, H-4-Ph), 6.67 (1H, d, *J* = 5.6 Hz, H-4), 6.52 (1H, dd, *J* = 1.5, 5.6 Hz, H-5), 5.83-5.71 (1H, m, H-2-Allyl), 5.20 (1H, d, *J* = 1.5 Hz, H-6), 5.12-5.05 (2H, m, H-3-Allyl), 4.66 (1H, dd, *J* = 3.5, 6.1 Hz, H-3), 3.74 (3H, s, OMe), 3.05 and 2.80 (1H and 1H, d and d, *J* = 9.1 Hz, H-7 and H-7a), 2.67-2.58 (2H, m, H-1-Allyl) ppm. For *cis*-**6aB**: ^{1}H NMR (600.2 MHz, $CDCl_3$): δ 7.44-7.37 (4H, m, H-2,6,3,5-Ph), 7.22 (1H, br.t, *J* = 7.3 Hz, H-4-Ph), 6.53 (1H, d, H-4), 6.42 (1H, dd, H-5), 5.83-5.71 (1H, m, H-2-Allyl), 5.24 (1H, d, *J* = 1.5 Hz, H-6), 5.12-5.05 (2H, m, H-3-Allyl), 4.59 (1H, dd, *J* = 4.5, 11.1 Hz, H-3), 3.81 (3H, s, OMe), 2.94 and 2.82 (1H and 1H, d and d, *J* = 9.1 Hz, H-7 and H-7a), 2.50-2.39 (2H, m, H-1-Allyl) ppm. ^{13}C NMR (150.9 MHz, $CDCl_3$): δ 172.3, 172.1, 170.3, 170.1, 137.3, 137.2, 136.6, 136.4, 135.5, 133.8, 132.8, 132.6, 131.6, 129.2, 129.0, 126.6, 125.7, 123.9, 120.0, 118.9, 91.0, 90.2, 81.0, 80.9, 60.5, 59.4, 52.2, 51.2, 45.6, 45.5, 33.8, 32.8 ppm. IR spectrum: ν (CO_2Me) =1768 cm^{-1}, ν (NC=O) =1687 cm^{-1}. HRMS (ESI-TOF) $[M+H]^+$ calcd for $C_{19}H_{20}NO_4$: 326.1314, found: 326.1327.

4.2 *Methyl (3aRS,6RS,7RS)-3-allyl-1-oxo-2-(m-tolyl)-1,2,3,6,7,7a-hexahydro-3a,6-epoxyisoindole-7-carboxylate* **(6b)**

White crystals, 63% (*trans*-**6bA**:*cis*-**6bB** = 69:31). M.p 128.5–129.8 °C. For *trans*-**6bA**: ^{1}H NMR (600.2 MHz, $CDCl_3$): δ 7.09-7.03 (3H, m, H-2,4,6-Ph), 6.67 (1H, d, *J* = 6.1 Hz, H-4), 6.51 (1H, dd, *J* = 1.5, 6.1

Hz, H-5), 5.84-5.80 (1H, m, H-2-Allyl), 5.20 (1H, d, *J* = 1.5 Hz, H-6), 5.20-5.19 (2H, m, H-3-Allyl), 4.61 (1H, dd, *J* = 3.5, 6.6 Hz, H-3), 3.74 (3H, s, OMe), 3.03 and 2.78 (1H and 1H, d and d, *J* = 9.1 Hz, H-7 and H-7a), 2.63-2.60 (1H, m, H*A*-1-Allyl), 2.39-2.38 (1H, m, H*B*-1-Allyl), 2.34 (3H, s, Me) ppm. For *cis*-**6bB**: ^{1}H NMR (600.2 MHz, $CDCl_3$): δ 7.28 (1H, t, *J* = 7.6 Hz, H-5-Ph), 7.09-7.03 (3H, m, H-2,4,6-Ph), 6.51 (1H, d, *J* = 5.6 Hz, H-4), 6.42 (1H, dd, *J* = 1.5, 5.6 Hz, H-5), 5.78-5.71 (1H, m, H-2-Allyl), 5.24 (1H, d, *J* = 1.5 Hz, H-6), 5.11-5.06 (2H, m, H-3-Allyl), 4.55 (1H, dd, *J* = 4.0, 10.6 Hz, H-3), 3.81 (3h, s, OMe), 2.92 and 2.81 (1H and 1H, d and d, *J* = 9.1 Hz, H-7 and H-7a), 2.49-2.41 (1H, m, H*A*-1-Allyl), 2.41-2.39 (1H, m, H*B*-1-Allyl), 2.36 (3H, s, Me) ppm. ^{13}C NMR (150.9 MHz, $CDCl_3$): δ 172.3, 172.1, 170.3, 170.2, 139.2, 139.0, 137.1, 136.5, 135.5, 133.9, 132.7, 131.7, 129.0, 128.8, 127.6, 127.1, 126.8, 125.0, 122.7, 121.3, 119.9, 188.8, 91.1, 60.2, 81.0, 80.1, 60.7, 59.5, 52.2, 51.2, 51.1, 45.6, 45.4, 33.8, 32.8, 21.5, 21.4 ppm. IR spectrum: ν (CO_2Me) =1767 cm^{-1}, ν (NC=O) =1679 cm^{-1}. HRMS (ESI-TOF) $[M+H]^+$ calcd for $C_{20}H_{22}NO_4$: 340.1471, found: 340.1483.

4.3 *Methyl (3aRS,6RS,7RS)-3-allyl-2-benzyl-1-oxo-1,2,3,6,7,7a-hexahydro-3a,6-epoxyisoindole-7-carboxylate* **(6c)**

White crystals, 85% (*trans*-**6cA**:*cis*-**6cB** = 85:15). M.p 127.3–128.9 °C. For *trans*-**6cA**: ^{1}H NMR (600.2 MHz, $CDCl_3$): δ 7.35-7.32 (2H, m, H-3,5-Ph), 7.28-7.24 (3H, m, H-2,4,6-Ph), 6.59 (1H, d, *J* = 6.1 Hz, H-4), 6.44 (1H, dd, *J* = 1.8, 6.1 Hz, H-5), 5.76-5.67 (1H, m, H-2-Allyl), 5.19-5.16 (2H, m, H-3-Allyl), 5.14 (1H, d, *J* = 1.8 Hz, H-6), 5.02 (1H, d, *J* = 15.6 Hz, N-CH_2*A*Ph), 4.14 (1H, d, *J* = 15.6 Hz, N-CH_2*B*Ph), 3.87 (1H, dd, *J* = 3.8, 6.2 Hz, H-3), 3.78 (3H, s, OMe), 2.87 and 2.76 (1H and 1H, br.d and d, *J* = 9.1 Hz, H-7 and H-7a), 2.60-2.52 (2H, m, H-1-Allyl) ppm. For *cis*-**6cB**: ^{1}H NMR (600.2 MHz, $CDCl_3$): δ 7.35-7.32 (2H, m, H-3,5-Ph), 7.28-7.24 (3H, m, H-2,6-Ph, H-4-Ph), 6.39 (1H, d, *J* = 5.8 Hz, H-4), 6.36 (1H, dd, *J* = 1.8, 5.8 Hz, H-5), 5.76-5.67 (1H, m, H-2-Allyl), 5.21 (1H, d, *J* = 1.8 Hz, H-6), 5.10-5.03 (2H, m, H-3-Allyl), 4.96 (1H, d, *J* = 15.6 Hz, N-CH_2*A*Ph), 4.14 (1H, d, *J* = 15.6 Hz, N-CH_2*B*Ph), 3.92 (1H, dd, *J* = 5.1, 10.1 Hz, H-3), 3.81 (3H, s, OMe), 2.80 and 2.77 (1H and 1H, br.d and d, *J* = 9.1 Hz, H-7 and H-7a), 2.60-2.52 (2H, m, H-1-Allyl) ppm. ^{13}C NMR (150.9 MHz, $CDCl_3$): δ 172.4, 171.3, 137.0, 135.9, 134.4, 132.1, 128.8, 127.8, 127.6, 119.8, 91.9, 80.9, 57.84, 52.2, 50.4, 45.5, 44.7, 33.9 ppm. IR spectrum: ν (CO_2Me) =1770 cm^{-1}, ν (NC=O) =1668 cm^{-1}. HRMS (ESI-TOF) $[M+H]^+$ calcd for $C_{20}H_{22}NO_4$: 340.1471, found: 340.1485.

4.4 *Methyl (3aRS,6RS,7RS)-3-allyl-2-(4-chlorophenyl)-1-oxo-1,2,3,6,7,7a-hexahydro-3a,6-epoxyisoindole-7-carboxylate* **(6d)**

White crystals, 80% (*trans*-**6dA**:*cis*-**6dB** = 70:30). M.p 128.5–130.7 °C. For *trans*-**6dA**: ^{1}H NMR (600.2 MHz, $CDCl_3$): δ 7.54 (1H, t, *J* = 2.0 Hz, H-2-Ph), 7.35-7.30 (2H, m, H-5,6-Ph), 7.20-7.17 (1H, m, H-4-Ph), 6.67 (1H, d, *J* = 6.1 Hz, H-4), 6.53 (1H, dd, *J* = 1.5, 6.1 Hz, H-5), 5.81-5.72 (1H, m, H-2-Allyl), 5.24-5.07 (3H, m, H-3-Allyl and H-6), 4.65 (1H, dd, *J* = 3.5, 6.6 Hz, H-3), 3.75 (3H, s, OMe), 3.04 and 2.81 (1H and 1H, d and d, *J* = 9.1 Hz, H-7 and H-7a), 2.69-2.60 (2H, m, H-1-Allyl) ppm. For *cis*-**6dB**: ^{1}H NMR (600.2 MHz, $CDCl_3$): δ 7.35-7.30 (1H, m, H-5-Ph), 7.24 (1H, ddd, *J* = 1.0, 2.0, 8.1 Hz, H-6-Ph), 7.20-7.17 (1H, m, H-4-Ph), 6.53-6.52 (1H, m, H-4), 6.43 (1H, dd, *J* = 2.0, 6.1 Hz, H-5), 5.81-5.72 (1H, m, H-2-Allyl), 5.24-5.07 (3H, m, H-3-Allyl and H-6), 4.58 (1H, dd, *J* = 4.5, 10.1 Hz, H-3), 3.81 (3H, s, OMe), 2.92 and 2.83 (1H and 1H, d and d, *J* = 9.1 Hz, H-7 and H-7a), 2.50-2.42 (2H, m, H-1-Allyl) ppm. ^{13}C NMR (150.9 MHz, $CDCl_3$): δ 172.2, 172.0, 170.3, 138.5, 137.8, 136.3, 135.6, 134.9, 134.6, 133.6, 132.3, 131.3, 131.3, 130.2, 130.0, 126.8, 126.1, 125.7, 123.8, 121.5, 120.5, 120.3, 119.2, 90.9, 90.1, 81.1, 80.9, 60.3, 59.3, 52.3, 51.2, 51.1, 45.7, 45.5, 33.7, 32.6 ppm. IR spectrum: ν (CO_2Me) =1765 cm^{-1}, ν (NC=O) =1666 cm^{-1}. HRMS (ESI-TOF) $[M+H]^+$ calcd for $C_{19}H_{19}ClNO_4$: 360.0924, found: 360.0938.

4.5 *Methyl (3aRS,6RS,7RS)-3-allyl-2-(4-chlorophenyl)-1-oxo-1,2,3,6,7,7a-hexahydro-3a,6-epoxyisoindole-7-carboxylate* **(6e)**

White crystals, 72% (*trans*-**6eA**:*cis*-**6eB** = 70:30). M.p 130.3–132.7 °C. For *trans*-**6eA**: ^{1}H NMR (600.2 MHz, $CDCl_3$): δ 7.41-7.34 (2H, m, H-3,5-Ph), 7.23-7.21 (2H, m, H-2,6-Ph), 6.66 (1H, d, J = 6.1 Hz, H-4), 6.52 (1H, d, J = 6.1 Hz, H-5), 5.78-5.71 (1H, m, H-2-Allyl), 5.21-5.19 (2H, m, H-3-Allyl), 4.63 (1H, dd, J = 4.5, 10.6 Hz, H-3), 3.74 (3H, s, OMe), 3.03 and 2.82 (1H and 1H, d and d, J = 9.1 Hz, H-7 and H-7a), 2.47-2.41 (2H, m, H-1-Allyl) ppm. For *cis*-**6eB**: ^{1}H NMR (600.2 MHz, $CDCl_3$): δ 7.41-7.34 (2H, m, H-3,5-Ph), 7.23-7.21 (2H, m, H-2,6-Ph), 6.52 (1H, d, J = 6.1 Hz, H-4), 6.43 (1H, dd, J = 1.5, 6.1 Hz, H-5), 5.78-5.71 (1H, m, H-2-Allyl), 5.24 (1H, d, J = 1.5 Hz, H-6), 5.12-5.06 (2H, m, H-3-Allyl), 4.57 (1H, dd, J = 4.5, 10.6 Hz, H-3), 3.80 (3H, s, OMe), 2.92 and 2.79 (1H and 1H, d and d, J = 9.1 Hz, H-7 and H-7a), 2.64-2.61 (2H, m, H-1-Allyl) ppm. ^{13}C NMR (150.9 MHz, $CDCl_3$): δ 136.3, 135.6, 135.1, 132.4, 132.1, 129.2, 126.8, 119.1, 90.1, 81.1, 59.3, 52.3, 51.1, 45.5, 32.7 ppm. IR spectrum: ν (CO_2Me) =1769 cm^{-1}, ν (NC=O) =1670 cm^{-1}. HRMS (ESI-TOF) $[M+H]^+$ calcd for $C_{19}H_{19}ClNO_4$: 360.0924, found: 360.0938.

4.6 *Methyl (3aRS,6RS,7RS)-3-allyl-2-(4-bromophenyl)-1-oxo-1,2,3,6,7,7a-hexahydro-3a,6-epoxyisoindole-7-carboxylate* **(6f)**

White crystals, 70% (*trans*-**6fA**:*cis*-**6fB** =72/28). M.p 132.4–134.3 °C. For *trans*-**6fA**: ^{1}H NMR (600.2 MHz, $CDCl_3$): δ 7.51 (2H, d, J = 8.6 Hz, H-3,5-Ph), 7.36 (2H, d, J = 8.6 Hz, H-2,6-Ph), 6.66 (1H, d, J = 6.1 Hz, H-4), 6.52-6.51 (1H, m, H-5), 5.79-5.71 (1H, m, H-2-Allyl), 5.19 (1H, d, J = 1.5 Hz, H-6), 5.15-5.06 (2H, m, H-2-Allyl), 4.63 (1H, dd, J = 3.5, 6.1 Hz, H-3), 3.74 (3H, s, OMe), 3.02 and 2.79 (1H and 1H, d and d, J = 9.1 Hz, H-7 and H-7a), 2.66-2.58 (2H, m, H-1-Allyl) ppm. For *cis*-**6fB**: ^{1}H NMR (600.2 MHz, $CDCl_3$): δ 7.53 (2H, d, J = 8.6 Hz, H-3,5-Ph), 7.17 (2H, d, J = 8.6 Hz, H-2,6-Ph), 6.52-6.51 (1H, m, H-4), 6.43 (1H, dd, J = 1.5, 6.1 Hz, H-5), 5.79-5.71 (1H, m, H-2-Allyl), 5.23 (1H, d, J = 1.5 Hz, H-6), 5.15-5.06 (2H, m, H-2-Allyl), 4.57 (1H, dd, J = 4.5, 10.6 Hz, H-3), 3.80 (3H, s, OMe), 2.91 and 2.82 (1H and 1H, d and d, J = 9.1 Hz, H-7 and H-7a), 2.49-2.39 (2H, m, H-1-Allyl) ppm. ^{13}C NMR (150.9 MHz, $CDCl_3$): δ 137.5, 136.4, 133.7, 132.3, 131.3, 125.2, 120.3, 119.1, 90.9, 80.9, 60.2, 52.3, 51.2, 45.7, 33.7 ppm. IR spectrum: ν (CO_2Me) =1771 cm^{-1}, ν (NC=O) =1674 cm^{-1}. HRMS (ESI-TOF) $[M+H]^+$ calcd for $C_{19}H_{19}BrNO_4$: 404.0419, found: 404.0427.

4.7 *Methyl (3aRS,6RS,7RS)-3-allyl-2-(4-iodophenyl)-1-oxo-1,2,3,6,7,7a-hexahydro-3a,6-epoxyisoindole-7-carboxylate* **(6g)**

White crystals, 63% (*trans*-**6gA**:*cis*-**6gB** = 59:41). M.p 133.5–135.0 °C. For *trans*-**6gA**: ^{1}H NMR (600.2 MHz, $CDCl_3$): δ 7.71-7.67 (2H, m, H-3,5-Ph), 7.22-7.20 (2H, m, H-2,6-Ph), 6.64 (1H, d, J = 5.6 Hz, H-4), 6.51-6.50 (1H, m, H-5), 5.78-5.71 (1H, m, H-2-Allyl), 5.19-5.11 (2H, m, H-2-Allyl), 5.18 (1H, d, J = 1.8 Hz, H-6), 4.62 (1H, dd, J = 3.5, 6.1 Hz, H-3), 3.73 (3H, s, OMe), 3.01 and 2.79 (1H and 1H, d and d, J = 9.1 Hz, H-7 and H-7a), 2.66-2.59 (1H, m, H*A*-1-Allyl), 2.47-2.39 (1H, m, H*B*-1-Allyl) ppm. For *cis*-**6gB**: ^{1}H NMR (600.2 MHz, $CDCl_3$): δ 7.71-7.67 (2H, m, H-3,5-Ph), 7.04-7.02 (2H, m, H-2,6-Ph), 6.51-6.50 (1H, m, H-4), 6.42 (1H, dd, J = 1.8, 6.1 Hz, H-5), 5.19-5.11 (2H, m, H-2-Allyl), 5.22 (1H, d, J = 1.8 Hz, H-6), 5.19-5.11 (2H, m, H-2-Allyl), 4.56 (1H, dd, J = 4.5, 10.1 Hz), 3.79 (3H, s, OMe), 2.89 and 2.80 (1H and 1H, d and d, J = 9.1 Hz, H-7 and H-7a), 2.66-2.59 (1H, m, H*A*-1-Allyl), 2.47-2.39 (1H, m, H*B*-1-Allyl) ppm. ^{13}C NMR (150.9 MHz, $CDCl_3$): δ 172.2, 172.0, 170.2, 170.1,138.2, 138.1, 137.5, 137.1, 136.4, 136.3, 135.6, 133.6, 132.4, 131.3, 127.3, 125.3, 120.3, 119.1, 91.1, 90.1, 90.0, 81.0, 80.9, 60.1, 59.1, 52.3, 51.2, 45.7, 45.5, 33.7, 32.6 ppm. IR spectrum: ν (CO_2Me) =1765 cm^{-1}, ν (NC=O) =1674 cm^{-1}. HRMS (ESI-TOF) $[M+H]^+$ calcd for $C_{19}H_{19}INO_4$: 452.0281, found: 452.0295.

4.8 *Methyl (3aRS,6RS,7RS)-3-allyl-2-(3-chloro-4-fluorophenyl)-1-oxo-1,2,3,6,7,7a-hexahydro-3a,6-epoxyisoindole-7-carboxylate* **(6h)**

White crystals, 86% (*trans*-**6hA**:*cis*-**6hB** = 66:34). M.p 134.4–136.2 °C. For *trans*-**6hA**: ^{1}H NMR (600.2 MHz, $CDCl_3$): δ 7.58 (1H, dd, *J* = 3.0, 6.6 Hz, H-2-Ph), 7.27 (1H, ddd, *J* = 2.5, 4.0, 9.1 Hz, H-6-Ph), 7.19-7.14 (1H, m, H-5-Ph), 6.66 (1H, d, *J* = 6.1 Hz, H-4), 6.53-6.51 (1H, m, H-5), 5.79-5.71 (1H, m, H-2-Allyl), 5.21-5.17 (2H, m, H-3-Allyl), 5.19 (1H, d, *J* = 1.8 Hz, H-6), 4.58 (1H, dd, *J* = 3.5, 6.1 Hz, H-3), 3.75 (3H, s, OMe), 3.02 and 2.81 (1H and 1H, d and d, *J* = 9.1 Hz, H-7 and H-7a), 2.67-2.62 (2H, m, H-1-Allyl) ppm. ^{19}F NMR (564.73 MHz, $CDCl_3$): −117.7 ppm. For *cis*-**6hB**: ^{1}H NMR (600.2 MHz, $CDCl_3$): δ 7.36 (1H, dd, *J* = 2.0, 6.6 Hz, H-2-Ph), 7.19-7.14 (2H, m, H-5,6-Ph), 6.53-6.51 (1H, m, H-4), 6.44 (1H, dd, *J* = 1.8, 6.1 Hz, H-5), 5.79-5.71 (1H, m, H-2-Allyl), 5.24 (1H, d, *J* = 1.8 Hz, H-6), 5.13-5.07 (2H, m, H-3-Allyl), 4.54 (1H, dd, *J* = 4.5, 10.6 Hz, H-3), 3.80 (3H, s, OMe), 2.91 and 2.82 (1H and 1H, d and d, *J* = 9.1 Hz, H-7 and H-7a), 2.50-2.45 (1H, m, H*A*-1-Allyl), 2.41-2.37 (1H, m, H*B*-1-Allyl) ppm. ^{19}F NMR (564.73 MHz, $CDCl_3$): −117.1 ppm. ^{13}C NMR (150.9 MHz, $CDCl_3$): δ 172.1, 172.0, 170.5, 170.4, 156.8, 155.7, 155.3, 155.1, 137.5, 136.2, 135.7, 133.9, 133.6, 133.2, 132.2, 131.2, 128.0, 126.3, 125.7, 123.6, 121.6, 121.5, 121.4, 120.4, 119.3, 117.0, 166.8, 166.7, 90.9, 90.1, 81.1, 80.1, 60.7, 59.5, 52.3, 51.0, 45.7, 45.5, 33.9, 32.7 ppm. IR spectrum: ν (CO_2Me) =1766 cm^{-1}, ν (NC=O) =1675 cm^{-1}. HRMS (ESI-TOF) [M+H]$^+$ calcd for $C_{19}H_{18}FClNO_4$: 378.0830, found: 378.0839.

4.9 *Methyl (3aRS,6RS,7RS)-3-(2-methylallyl)-1-oxo-2-phenyl-1,2,3,6,7,7a-hexahydro-3a,6-epoxyisoindole-7-carboxylate* **(6i)**

White crystals, 53% (*trans*-**6iA**:*cis*-**6iB** = 55:45). M.p 135.8–136.9 °C. For *trans*-**6iA**: ^{1}H NMR (600.2 MHz, $CDCl_3$): δ 7.46-7.35 (3H, m, H-Ar), 7.28-7.18 (2H, m, H-Ar), 6.63 (1H, d, *J* = 5.6 Hz, H-4), 6.45 (1H, dd, *J* = 1.5, 5.6 Hz, H-5), 5.18 (1H, d, *J* = 1.5 Hz, H-6), 4.92 and 4.85 (1H and 1H, br.s and br.s, H-3-Allyl), 4.77-4.73 (1H, m, H-3), 3.74 (3H, s, OMe), 3.09 and 2.80 (1H and 1H, d and d, *J* = 9.1 Hz, H-7 and H-7a), 2.60 (1H, br.d, *J* = 15.6 Hz, H*A*-1-Allyl), 2.49-2.39 (1H, m, H*B*-1-Allyl), 1.74 (3H, s, Me-2-Allyl) ppm. For *cis*-**6iB**: ^{1}H NMR (600.2 MHz, $CDCl_3$): δ 7.46-7.35 (3H, m, H-Ar), 7.28-7.18 (2H, m, H-Ar), 6.51 (1H, d, *J* = 5.6 Hz, H-4), 6.37 (1H, dd, *J* = 1.5, 5.6 Hz, H-5), 5.23 (1H, d, *J* = 1.5 Hz, H-6), 4.92 and 4.85 (1H and 1H, br.s and br.s, H-3-Allyl), 4.77-4.73 (1H, m, H-3), 3.81 (3H, s, OMe), 2.93 and 2.80 (1H and 1H, d and d, *J* = 9.1 Hz, H-7 and H-7a), 2.49-2.39 (1H, m, H*A*-1-Allyl), 2.35 (1H, dd, H*B*-1-Allyl), 1.74 (3H, s, Me-2-Allyl) ppm. ^{13}C NMR (150.9 MHz, $CDCl_3$): δ 172.5, 172.3, 170.4, 170.0, 140.7, 140.3, 137.5, 136.7, 136.5, 136.4, 135.2, 134.2, 129.2, 129.1, 126.6, 129.1, 126.6, 126.0, 123.7, 114.3, 113.8, 91.2, 90.4, 81.0, 80.9, 59.1, 58.5, 52.3, 51.5, 50.6, 45.6, 37.9, 35.1, 23.3, 23.0 ppm. IR spectrum: ν (CO_2Me) =1769 cm^{-1}, ν (NC=O) =1670 cm^{-1}. HRMS (ESI-TOF) [M+H]$^+$ calcd for $C_{20}H_{22}NO_4$: 340.1471, found: 340.1462.

4.10 *Methyl (3aRS,6RS,7RS)-3-(2-methylallyl)-1-oxo-2-(m-tolyl)-1,2,3,6,7,7a-hexahydro-3a,6-epoxyisoindole-7-carboxylate* **(6j)**

White crystals, 73% (*trans*-**6jA**:*cis*-**6jB** = 45:55). M.p 133.7–135.6 °C. For *trans*-**6jA**: ^{1}H NMR (600.2 MHz, $CDCl_3$): δ 7.29-7.01 (4H, m, H-Ar), 6.64 (1H, d, *J* = 6.1 Hz, H-4), 6.45 (1H, dd, *J* = 1.5, 6.1 Hz, H-5), 5.18 (1H, d, *J* = 1.5 Hz, H-6), 4.92 (1H, br.s, H-3-Allyl), 3.75 (3H, s, OMe), 3.07 and 2.79 (1H and 1H, d and d, *J* = 8.9 Hz, H-7 and H-7a), 2.59 (1H, dd, *J* = 11.1, 14.1 Hz, H*A*-1-Allyl), 2.41 (1H, dd, *J* = 10.6, 16.2 Hz, H*B*-1-Allyl), 2.34 (3H, s, Me-Ar), 1.65 (3H, br.s, H-2-Allyl) ppm. For *cis*-**6jB**: ^{1}H NMR (600.2 MHz, $CDCl_3$): δ 7.29-7.01 (4H, m, H-Ar), 6.50 (1H, d, *J* = 6.1 Hz, H-4), 6.38 (1H, dd, *J* = 1.5, 6.1 Hz, H-5), 5.23 (1H, d, *J* = 1.5 Hz, H-6), 4.84 (1H, br.s, H-3-Allyl), 4.73-4.69 (1H, m, H-3), 3.81 (3H, s, OMe), 2.92 and 2.80 (1H and 1H, d and d, *J* = 8.9 Hz, H-7 and H-7a), 2.48 (1H, dd, *J* = 11.1, 14.1 Hz, H*A*-1-

Allyl), 2.36 (3H, s, Me-Ar), 2.33-2.30 (1H, dd, *J* = 3.5, 14.1 Hz, H*B*-1-Allyl), 1.74 (3H, s, H-2-Allyl) ppm. ^{13}C NMR (150.9 MHz, $CDCl_3$): δ 172.5, 172.3, 170.4, 170.0, 140.8, 140.5, 139.2, 139.1, 137.3, 136.6, 136.5, 135.1, 134.5, 129.0, 128.9, 127.7, 127.1, 126.9, 124.9, 122.8, 121.1, 114.2, 113.7, 91.3, 90.4, 81.0, 80.9, 59.3, 58.7, 52.3, 51.5, 50.6, 45.6, 37.9, 35.1 ppm. IR spectrum: ν (CO_2Me) =1772 cm^{-1}, ν (NC=O) =1663 cm^{-1}. HRMS (ESI-TOF) $[M+H]^+$ calcd for $C_{21}H_{24}O_4$: 354.4180, found: 354.4193.

4.11 *Methyl (3aRS,6RS,7RS)-3-(2-methylallyl)-1-oxo-2-(p-tolyl)-1,2,3,6,7,7a-hexahydro-3a,6-epoxyisoindole-7-carboxylate* **(6k)**

White crystals, 98% (*trans*-**6kA**:*cis*-**6kB** = 50:50). M.p 134.5–136.9 °C. For *trans*-**6kA**: ^{1}H NMR (600.2 MHz, $CDCl_3$): δ 7.31 (2H, br.d, *J* = 8.1 Hz, 2,6-Ph), 7.20-7.13 (2H, m, 3,5-Ph), 6.64 (1H, d, *J* = 6.1 Hz, H-4), 6.45 (1H, dd, *J* = 1.5, 6.1 Hz, H-5), 5.18 (1H, d, *J* = 1.5 Hz, H-6), 4.92 and 4.80 (1H and 1H, br.s, H-3-Methallyl), 4.72-4.68 (1H, m, H-3), 3.74 (3H, s, OMe), 3.07 and 2.79 (1H and 1H, d and d, *J* = 9.1 Hz, H-7 and H-7a), 2.59 (1H, br.d, *J* = 16.2 Hz, H-1-Methallyl), 2.41 (1H, dd, *J* = 10.6, 16.2 Hz, H-1-Methallyl), 2.32 (3H, s, Me-4-C_6H_4), 1.74 (3H, br.s, H-2-Methallyl) ppm. For *cis*-**6kB**: ^{1}H NMR (600.2 MHz, $CDCl_3$): δ 7.20-7.13 (4H, m, 2,3,5,6-Ph), 6.50 (1H, d, *J* = 6.1 Hz, H-4), 6.37 (1H, dd, *J* = 1.5, 6.1 Hz, H-5), 5.23 (1H, d, *J* = 1.5 Hz, H-6), 4.92 and 4.84 (1H and 1H, br.s, H-3-Methallyl), 4.72-4.68 (1H, m, H-3), 3.80 (3H, s, OMe), 2.92 and 2.80 (1H and 1H, d and d, *J* = 9.1 Hz, H-7 and H-7a), 2.48 (1H, dd, *J* = 11.0, 13.6 Hz, H-1-Methallyl), 2.34 (3H, s, Me-4-C_6H_4), 2.31-2.30 (1H, m, H-1-Allyl), 1.74 (3H, br.s, H-2-Methallyl) ppm. ^{13}C NMR (150.9 MHz, $CDCl_3$): δ 172.4, 170.4, 140.7, 140.4, 136.5, 136.4, 135.0, 129.8, 129.7, 125.7, 123.9, 114.1, 113.6, 90.3, 80.9, 80.8, 59.2, 58.5, 52.2, 51.4, 50.4, 45.5, 50.4, 45.5, 37.8, 35.0 ppm. IR spectrum: ν (CO_2Me) =1776 cm^{-1}, ν (NC=O) =1669 cm^{-1}. HRMS (ESI-TOF) $[M+H]^+$ calcd for $C_{21}H_{24}NO_4$: 354.4180, found: 354.4189.

4.12 *Methyl (3aRS,6RS,7RS)-2-(4-isopropylphenyl)-3-(2-methylallyl)-1-oxo-1,2,3,6,7,7a-hexahydro-3a,6-epoxyisoindole-7-carboxylate* **(6l)**

White crystals, 82% (*trans*-**6lA**:*cis*-**6lB** = 87:13). M.p 132.2–134.0 °C. For *trans*-**6lA**: ^{1}H NMR (600.2 MHz, $CDCl_3$): δ 7.34 (2H, d, *J* = 7.6 Hz, H-2,6-Ph), 7.26-7.21 (2H, m, H-2,6-Ph), 6.63 (1H, dd, *J* = 1.2, 5.5 Hz, H-5), 6.44 (1H, br.d, *J* = 5.5 Hz, H-4), 5.18 (1H, br.s, H-6), 4.92 (1H, br.s, H-3-Allyl), 4.83 (1H, br.s, H-3-Allyl), 4.69 (1H, dd, *J* = 3.0, 10.6 Hz, H-3), 3.74 (3H, s, OMe), 3.07 and 2.80 (1H and 1H, d and d, *J* = 9.1 Hz, H-7 and H-7a), 2.93-2.86 (1H, m, C*H*Me2), 2.61-2.58 (1H, m, H*B*-1-Allyl), 2.40-2.34 (1H, m, H*A*-1-Allyl), 1.74 (3H, br.s, H-2-Allyl), 1.22 (6H, d, *J* = 7.1 Hz, CH*Me*2) ppm. For *cis*-**6lB**: ^{1}H NMR (600.2 MHz, $CDCl_3$): δ 7.26-7.21 (2H, m, H-2,6-Ph), 7.17 (2H, d, *J* = 7.6 Hz, H-3,5-Ph), 6.50 (1H, dd, *J* = 1.2, 5.5 Hz, H-5), 6.37 (1H, br.d, *J* = 5.5 Hz, H-4), 4.83 (1H, br.s, H-3-Allyl), 4.72 (1H, dd, *J* = 3.5, 10.6 Hz, H-3), 3.80 (3H, s, OMe), 2.93-2.86 (1H, m, C*H*Me2), 2.80 (2H, dd, *J* = 1.0, 9.1 Hz, H-7 and H-7a), 2.45 (1H, dd, *J* = 11.6, 9.1 Hz, H*B*-1-Allyl), 2.34 (1H, dd, *J* = 3.0, 13.6 Hz, H*A*-1-Allyl), 1.74 (3H, br.s, H-2-Allyl), 1.24 (6H, d, *J* = 7.1 Hz, CH*Me*2) ppm. ^{13}C NMR (150.9 MHz, $CDCl_3$): δ 172.4, 172.2, 147.4, 146.8, 140.7, 140.4, 137.1, 136.4, 135.0, 134.5, 134.2, 133.6, 127.2, 127.1, 125.7, 123.9, 114.1, 113.5, 91.2, 90.3, 80.9, 59.3, 58.6, 56.7, 52.2, 51.4, 50.5, 49.8, 45.9, 45.5, 41.6, 37.7, 35.1, 33.8 ppm. IR spectrum: ν (CO_2Me) =1769 cm^{-1}, ν (NC=O) =1672 cm^{-1}. HRMS (ESI-TOF) $[M+H]^+$ calcd for $C_{23}H_{28}NO_4$: 382.1940, found: 382.1953.

4.13 *Methyl (3aRS,6RS,7RS)-2-benzyl-3-(2-methylallyl)-1-oxo-1,2,3,6,7,7a-hexahydro-3a,6-epoxyisoindole-7-carboxylate* **(6m)**

White crystals, 57% (*trans*-**6mA**:*cis*-**6mB** = 42:58). M.p 135.3–136.0 °C. For *trans*-**6mA**: ^{1}H NMR (600.2 MHz, $CDCl_3$): δ 7.34 (2H, t, *J* = 7.5 Hz, H-3,5-Ph), 7.28-7.23 (3H, m, H-2,4,6-Ph), 6.57 (1H, d, *J* = 6.1 Hz, H-4), 6.56 (1H, dd, *J* = 6.1, 1.5 Hz, H-5), 5.15 (1H, d, *J* = 1.5 Hz, H-6), 5.06 (1H, d, *J* = 15.6 Hz, N-CH*A*-Ph), 4.81 (1H, br.s, H-3-Methallyl), 4.73 (1H, br.s, H-3-Methallyl), 4.08 (1H, d, *J* = 15.6 Hz, N-CH*B*-Ph),

3.96 (1H, dd, *J* = 5.5, 8.6 Hz, H-3), 3.78 (3H, s, OMe), 2.94 and 2.79-2.75 (1H and 1H, d and m, *J* = 9.1 Hz, H-7 and H-7a), 2.52-2.45 (1H, m, H-1-Methallyl), 2.39 (1H, dd, *J* = 8.6, 15.6 Hz, H-1-Methallyl), 1.72 (3H, br.s, H-2-Methallyl) ppm. For *cis*-**6mB**: ^{1}H NMR (600.2 MHz, $CDCl_3$): δ 7.34 (2H, t, *J* = 7.5 Hz, H-3,5-Ph), 7.28-7.23 (3H, m, H-2,4,6-Ph), 6.36 (1H, d, *J* = 6.1 Hz, H-4), 6.33 (1H, dd, *J* = 6.1, 1.5 Hz, H-5), 5.20 (1H, d, *J* = 1.5 Hz, H-6), 4.97 (1H, d, *J* = 15.6 Hz, N-CH*A*-Ph), 4.73 (1H, br.s, H-3-Methallyl), 4.15 (1H, d, *J* = 15.6 Hz, N-CH*B*-Ph), 4.06 (1H, dd, *J* = 5.0, 9.6 Hz, H-3), 3.81 (3H, s, OMe), 2.79-2.75 (2H, m, H-7 and H-7a), 2.52-2.45 (2H, m, H-1-Methallyl), 1.61 (3H, br.s, H-2-Methallyl) ppm. ^{13}C NMR (150.9 MHz, $CDCl_3$): δ 172.4, 171.1, 140.9, 136.4, 135.8, 135.1, 134.4, 128.8, 128.7, 127.7, 127.6, 127.4, 114.6, 114.2, 92.1, 81.0, 80.9, 56.0, 52.2, 49.7, 45.3, 44.8, 44.2, 38.6, 34.9 ppm. IR spectrum: ν (CO_2Me) =1765 cm^{-1}, ν (NC=O) =1667 cm^{-1}. HRMS (ESI-TOF) [M+H]$^+$ calcd for $C_{20}H_{23}NO_4$: 355.4180, found: 355.4173.

4.14 *Methyl (3aRS,6RS,7RS)-2-(2-chlorophenyl)-3-(2-methylallyl)-1-oxo-1,2,3,6,7,7a-hexahydro-3a,6-epoxyisoindole-7-carboxylate* **(6n)**

White crystals, 95% (*trans*-**6nA**:*cis*-**6nB** = 75:25). M.p 136.4–137.8 °C. For *trans*-**6nA**: ^{1}H NMR (600.2 MHz, $CDCl_3$): δ 7.50-7.27 (4H, m, H-Ar), 6.52 (1H, d, *J* = 6.1 Hz, H-4), 6.40 (1H, dd, *J* = 2.0, 5.1 Hz, H-5), 5.24 (1H, d, *J* = 2.0 Hz, H-6), 4.91 (1H, dd, *J* = 5.1, 11.1 Hz, H-3), 4.84-4.74 and 4.67-4.65 (1H and 1H, m and m, H-3-Allyl), 3.78 (3H, s, OMe), 3.00 and 2.82 (1H and 1H, d and d, *J* = 9.1 Hz, H-7 and H-7a), 2.52 (1H, dd, *J* = 3.0, 13.3 Hz, H*A*-1-Allyl), 2.06 (1H, dd, *J* = 4.0, 13.2 Hz, H*B*-1-Allyl), 1.71 (3H, s, H-2-Allyl) ppm. For *cis*-**6nB**: ^{1}H NMR (600.2 MHz, $CDCl_3$): δ 7.50-7.27 (4H, m, H-Ar), 6.63 (1H, d *J* = 6.1 Hz, H-4), 6.45 (1H, dd, *J* = 2.0, 5.1 Hz, H-5), 5.22 (1H, d, *J* = 2.0 Hz, H-6), 4.84-4.74 and 4.67-4.65 (1H and 1H, m and m, H-3-Allyl), 4.62 (1H, dd, *J* = 6.1, 9.1 Hz, H-3), 3.73 (3H, s, OMe), 3.06 and 2.82 (1H and 1H, d and d, *J* = 9.1 Hz, H-7 and H-7a), 2.66 (1H, dd, *J* = 10.1, 13.1 Hz, H*A*-1-Allyl), 2.48 (1H, dd, *J* = 10.1, 13.1 Hz, H*B*-1-Allyl), 1.71 (3H, s, H-2-Allyl) ppm. ^{13}C NMR (150.9 MHz, $CDCl_3$): δ 172.5, 171.2, 140.4, 139.8, 136.2, 136.0, 135.3, 134.6, 133.9, 132.6, 132.3, 130.6, 130.1, 129.5, 127.9, 92.1, 91.11, 81.0, 80.9, 60.1, 57.9, 52.3, 50.9, 49.4, 45.7, 45.5, 38.23, 35.8, 34.74, 23.0, 22.6 ppm. IR spectrum: ν (CO_2Me) =1763 cm^{-1}, ν (NC=O) =1670 cm^{-1}. HRMS (ESI-TOF) [M+H]$^+$ calcd for $C_{20}H_{22}ClNO_4$: 375.1081, found: 375.1092.

4.15 *Methyl (3aRS,6RS,7RS)-2-(3-chlorophenyl)-3-(2-methylallyl)-1-oxo-1,2,3,6,7,7a-hexahydro-3a,6-epoxyisoindole-7-carboxylate* **(6o)**

White crystals, 78% (*trans*-**6oA**:*cis*-**6oB** = 83:17). M.p 138.3–139.7 °C. For *trans*-**6oA**: ^{1}H NMR (600.2 MHz, $CDCl_3$): δ 7.57 (1H, br.s, H-6-Ph), 7.34-7.16 (4H, m, H-Ar), 6.63 (1H, d, *J* = 5.6 Hz, H-4), 6.47 (1H, dd, *J* = 0.9, 5.6 Hz, H-5), 5.18 (1H, br.s, H-6), 4.94 (1H, s, H*A*-3-Allyl), 4.81-4.72 (2H, m, H*B*-3-Allyl and H-3), 3.75 (3H, s, OMe), 3.08 and 2.81 (1H and 1H, d and d, *J* = 9.1 Hz, H-7 and H-7a), 2.58 (1H, dd, *J* = 1.6, 16.2 Hz, H*A*-1-Allyl), 2.42 (1H, dd, *J* = 10.1, 16.2 Hz, H*B*-1-Allyl), 1.76 (3H, s, H-2-Allyl) ppm. For *cis*-**6oB**: ^{1}H NMR (600.2 MHz, $CDCl_3$): δ 7.34-7.16 (4H, m, H-Ar), 6.50 (1H, d, *J* = 5.6 Hz, H-4), 6.39 (1H, dd, *J* = 0.9, 5.6 Hz, H-5), 5.22 (1H, br.s, H-6), 4.86 (1H, s, H*A*-3-Allyl), 4.81-4.72 (2H, m, H*B*-3-Allyl and H-3), 3.81 (3H, s, OMe), 2.91 and 2.81 (1H and 1H, d and d, *J* = 9.1 Hz, H-7 and H-7a), 2.49 (1H, dd, *J* = 11.0, 13.9 Hz, H*A*-1-Allyl), 2.35 (1H, dd, *J* = 3.0, 13.9 Hz, H*B*-1-Allyl), 1.76 (3H, s, H-2-Allyl) ppm. ^{13}C NMR (150.9 MHz, $CDCl_3$): δ 172.3, 170.4, 170.1, 140.3, 140.1, 137.9, 136.6, 136.2, 135.2, 134.5, 134.5, 134.2, 130.1, 129.9, 126.7, 125.9, 125.7, 123.7, 123.6, 123.7, 123.6, 121.0, 114.4, 114.0, 91.0, 90.2, 81.0, 58.9, 58.3, 52.3, 51.3, 50.5, 45.6, 37.8, 34.9, 23.2, 23.0 ppm. IR spectrum: ν (CO_2Me) =1775 cm^{-1}, ν (NC=O) =1668 cm^{-1}. HRMS (ESI-TOF) [M+H]$^+$ calcd for $C_{20}H_{22}ClNO_4$: 375.1081, found: 375.1075.

4.16 *Methyl (3aRS,6RS,7RS)-2-(4-bromophenyl)-3-(2-methylallyl)-1-oxo-1,2,3,6,7,7a-hexahydro-3a,6-epoxyisoindole-7-carboxylate* **(6p)**

White crystals, 56% (*trans*-**6pA**:*cis*-**6pB** = 57:43). M.p 135.0–136.3 °C. For *trans*-**6pA**: ^{1}H NMR (600.2 MHz, $CDCl_3$): *δ* 7.47 (2H, d, *J* ~ 9.1 Hz, 3,5-Ph), 7.37 (2H, d, *J* ~ 9.1 Hz, 2,6-Ph), 6.62 (1H, d, *J* = 6.1 Hz, H-4), 6.47 (1H, dd, *J* = 1.6, 6.1 Hz, H-5), 5.18 (1H, d, *J* = 6.1 Hz, H-6), 4.94 (1H, br.s, H-3-Methallyl), 4.75-4.70 (1H, m, H-3), 3.75 (3H, s, OMe), 3.07 and 2.81 (1H and 1H, d and d, *J* = 9.1 Hz, H-7 and H-7a), 2.57 and 2.48-2.40 (2H, dd and m, *J* = 3.0, 16.1 Hz, H-1-Methallyl), 1.75 (3H, br.s, H-2-Methallyl) ppm. For *cis*-**6pB**: ^{1}H NMR (600.2 MHz, $CDCl_3$): *δ* 7.53-7.49 (2H, m, 3,5-Ph), 7.18 (2H, d, *J* = 9.1 Hz, 2,6-Ph), 6.50 (1H, d, *J* = 6.1 Hz, H-4), 6.39 (1H, dd, *J* = 1.6, 6.1 Hz, H-5), 5.22 (1H, d, *J* = 6.1 Hz, H-6), 4.86 (1H, br.s, H-3-Methallyl), 4.75-4.70 (1H, m, H-3), 3.80 (3H, s, OMe), 2.91 and 2.81 (1H and 1H, d and d, *J* = 9.1 Hz, H-7 and H-7a), 2.48-2.40 and 2.34 (1H, m and dd, *J* = 3.5, 14.1 Hz, H-1-Methallyl), 1.75 (3H, br.s, H-2-Methallyl) ppm. ^{13}C NMR (150.9 MHz, $CDCl_3$): *δ* 172.4, 172.2, 170.5, 170.1, 140.4, 140.1, 136.3, 135.8, 135.3, 134.3, 132.3, 132.2, 127.2, 125.0, 120.1, 119.1, 114.5, 114.0, 94.1, 90.3, 81.1, 81.0, 59.0, 58.3, 52.4, 51.4, 50.6, 45.7, 37.9, 34.9 ppm. IR spectrum: ν (CO_2Me) =1769 cm^{-1}, ν (NC=O) =1673 cm^{-1}. HRMS (ESI-TOF) $[M+H]^+$ calcd for $C_{20}H_{22}BrNO_4$: 420.2870, found: 420.2884.

5. *Technique for isomer separation of methyl 3-allyl-3a,6-epoxyisoindole-7-carboxylates* **6a, 6e, 6f** *and* **6p**

Mixture of isomers **6a**, **6e**, **6f** or **6p** (3 mmol) was stirred in methanol (50 mL) at 45 °C for 30 min. After that, the insoluble substance (*cis*-isomer, **6aA**, **6eA**, **6fA** or **6pA**) was separated from the solution by filtration. Crystals of the *trans*-isomer (**6aB**, **6eB**, **6fB** or **6pB**) precipitated from the mother liquid after cooling to 4°C. According to the ^{1}H NMR data, both obtained portions of the isomers contained admixtures of opposite isomers (5–8 %). The repeat of the mentioned above procedure using 30 mL of MeOH allows to achieve more than a 99% resolution of the isomers.

6. *General method for synthesis of methyl cyclopenta[b]furo[2,3-c]pyrrole-3-carboxylates* **7a, 7e, 7f** *and* **7p**

Catalyst 1 (0.005 mmol, 3.3 mg) was added to the solution of ester **6aA, 6eA** or **6fA** (1 mmol) in chloroform (25 mL) in an argon stream. Reaction mixture in a Schlenk flask was boiled in an atmosphere of argon for 30 min (TLC monitoring, ethyl acetate : hexane = 1:3). After cooling and removing of the solvent under reduced pressure, the target tricycles **7aA, 7eA** or **7fA** were purified by flash-chromatography on silica gel (ethyl acetate:hexane = 12:88) providing the desired substances as colourless powders with yields 83, 89 and 87 % correspondently, their physical and chemical properties see below. The reaction with compounds **6aB, 6eB, 6fB** leads to polymerization.

7. *General method for synthesis of methyl cyclopenta[b]furo[2,3-c]pyrrole-3-carboxylates* **(7a–h)**

Catalyst 1 (0.005 mmol, 3.3 mg) was added to the solution of ester **6a–h** (1 mmol) in chloroform (25 mL) in an argon stream. Reaction mixture in a Schlenk flask was boiled in an atmosphere of argon for 30 min (TLC monitoring, ethyl acetate : hexane = 1:3). After cooling and removing of the solvent

under reduced pressure, the target tricycles **7a–h** were purified by flash-chromatography on silica gel (ethyl acetate:hexane = 12:88) providing the desired substances as colorless powders.

7.1 *Methyl (2RS,3SR,3aRS,5aRS,8aSR)-4-oxo-5-phenyl-2-vinyl-3,3a,4,5,5a,6-hexahydro-2H-cyclopenta[b]furo[2,3-c]pyrrole-3-carboxylate* **(7a)**

The mixture of isomers **6aA/6aB** = 79/21 was used. White crystals, 68% yield. M.p 120.5–122.3 °C. ^{1}H NMR (600.2 MHz, $CDCl_3$): δ 7.54-7.53 (2H, m, H-2,6-Ph), 7.40-7.37 (2H, m, H-3,5-Ph), 7.21-7.18 (1H, m, H-4-Ph), 5.98 (1H, dt, *J* = 2.5, 6.0 Hz, H-8), 5.90 (1H, ddd, *J* = 6.6, 10.6, 17.1 Hz, H-1-Vinyl), 5.79 (1H, dt, *J* = 2.5, 5.6 Hz, H-7), 5.46 (1H, dt, *J* = 1.0, 17.1 Hz, H-2-*trans*-Vinyl), 5.28 (1H, dt, *J* = 1.0, 10.6 Hz, H-2-*cis*-Vinyl), 4.72 (1H, br.dd, *J* = 6.6, 10.3 Hz, H-2), 4.68 (1H, dd, *J* = 2.5, 7.1 Hz, H-5a), 3.73 (3H, s, OMe), 3.44 (1H, d, *J* = 10.3 Hz, H-3a), 3.19 (1H, t, *J* = 10.3 Hz, H-3), 2.97 (1H, ddt, *J* = 2.5, 7.1, 17.7 Hz, H-6A), 2.31 (1H, dq, *J* = 2.5, 17.7 Hz, H-6B) ppm. ^{13}C NMR (150.9 MHz, $CDCl_3$): δ 170.1, 170.0, 137.7, 134.6, 130.5, 129.1, 125.6, 122.2, 118.9, 95.9, 81.1, 67.4, 54.5, 53.2, 52.2, 38.4, 29.7 ppm. IR spectrum: ν (CO_2Me) = 1762 cm^{-1}, ν (NC=O) = 1663 cm^{-1}. HRMS (ESI-TOF) $[M+H]^+$ calcd for $C_{19}H_{21}NO_4$: 327.1314, found: 327.1332.

7.2 *Methyl (2RS,3SR,3aRS,5aRS,8aSR)-4-oxo-5-(m-tolyl)-2-vinyl-3,3a,4,5,5a,6-hexahydro-2H-cyclopenta[b]furo[2,3-c]pyrrole-3-carboxylate* **(7b)**

The mixture of isomers **6bA/6bB** = 69/31 was used. White crystals, 57%. M.p 125.8–127.0 °C. ^{1}H NMR (600.2 MHz, $CDCl_3$): δ 7.37 (1H, br.s, H-2-Ph), 7.27-7.26 (2H, m, H-2,6-Ph), 7.02-7.01 (1H, m, H-5-Ph), 5.97 (1H, m, H-7), 5.90 (1H, ddd, *J* = 6.6, 10.6, 17.1 Hz, H-1-Vinyl), 5.45 (1H, d, *J* = 17.1 Hz, H-2-*trans*-Vinyl), 5.27 (1H, d, *J* = 10.5 Hz, H-2-*cis*-Vinyl), 4.72 (1H, dd, *J* = 6.6, 10.5 Hz, H-2), 4.67 (1H, dd, *J* = 2.5, 7.1 Hz, H-5a), 3.73 (3H, s, OMe), 3.42 (1H, d, *J* = 10.5 Hz, H-3a), 3.18 (1H, t, *J* = 10.5 Hz, H-3), 2.94-2.89 (1H, m, H-6A), 2.36 (3H, s, Me-3-Ph), 2.30-2.26 (1H, m, H-6B) ppm. ^{13}C NMR (150.9 MHz, $CDCl_3$): δ 170.1, 170.0, 139.1, 137.6, 134.7, 134.6, 130.6, 129.0, 126.7, 123.4, 119.6, 118.9, 96.0, 81.2, 67.6, 54.5, 53.2, 52.3, 38.4, 29.5 ppm. IR spectrum: ν (CO_2Me) = 1768 cm^{-1}, ν (NC=O) =1670 cm^{-1}. HRMS (ESI-TOF) $[M+H]^+$ calcd for $C_{20}H_{23}NO_4$: 341.1471, found: 341.1487.

7.3 *Methyl (2RS,3SR,3aRS,5aRS,8aSR)-5-benzyl-4-oxo-2-vinyl-3,3a,4,5,5a,6-hexahydro-2H-cyclopenta[b]furo[2,3-c]pyrrole-3-carboxylate* **(7c)**

The mixture of isomers **6cA/6cB** = 85/15 was used. White crystals, 65%. M.p 121.6–123.4 °C. ^{1}H NMR (600.2 MHz, $CDCl_3$): δ 7.38 (2H, br.t, H-3,5-Ph), 7.34-7.30 (3H, m, H-2,4,6-Ph), 5.92-5.91 (1H, m, H-7), 5.83 (1H, ddd, *J* = 6.9, 10.3, 17.2 Hz, H-1-Vinyl), 5.71-5.70 (1H, m, H-8), 5.38 (1H, dd, *J* = 1.0, 17.2 Hz, H-2-*trans*-Vinyl), 5.23 (1H, dd, *J* = 1.0, 10.3 Hz, H-2-*cis*-Vinyl), 5.15 (1H, d, *J* = 14.6 Hz, NCH_2*A*-Ph), 4.46 (1H, dd, *J* = 6.9, 10.5 Hz, H-2), 3.90 (1H, d, *J* = 14.6 Hz, NCH_2*B*-Ph), 3.83 (1H, dd, *J* = 2.6, 7.6 Hz, H-5a), 3.81 (3H, s, OMe), 3.28 (1H, d, *J* = 10.5 Hz, H-3a), 3.12 (1H, t, *J* = 10.5 Hz, H-3), 2.68 (1H, ddt, *J* = 1.0, 7.6, 17.7 Hz, H-6A), 2.31 (1H, dq, *J* = 2.6, 17.7 Hz, H-6B) ppm. ^{13}C NMR (150.9 MHz, $CDCl_3$): δ 170.7, 170.1, 135.6, 134.5, 134.2, 130.5, 128.9, 128.4, 127.9, 118.7, 96.6, 80.9, 65.1, 53.8, 53.1, 52.2, 45.5, 36.5 ppm. IR spectrum: ν (CO_2Me) = 1765 cm^{-1}, ν (NC=O) = 1667 cm^{-1}. HRMS (ESI-TOF) $[M+H]^+$ calcd for $C_{20}H_{23}NO_4$: 341.1471, found: 341.1458.

7.4 *Methyl (2RS,3SR,3aRS,5aRS,8aSR)-5-(3-chlorophenyl)-4-oxo-2-vinyl-3,3a,4,5,5a,6-hexahydro-2H-cyclopenta[b]furo[2,3-c]pyrrole-3-carboxylate* **(7d)**

The mixture of isomers **6dA/6dB** = 70/30 was used. White crystals, 57%. M.p 123.4–126.7 °C. ^{1}H NMR (600.2 MHz, $CDCl_3$): δ 7.65 (1H, t, *J* = 2.0 Hz, H-2-Ph), 7.43-7.42 and 7.18-7.16 (1H and 1H, m and m, H-4,6-Ph), 7.32 (1H, t, *J* = 8.1 Hz, H-5-Ph), 5.98-5.97 (1H, m, H-7), 5.89 (1H, dt, *J* = 6.6, 10.6, 17.2 Hz, H-1-Vinyl), 5.78-5.77 (1H, m, H-8), 5.45 (1H, dt, *J* = 1.0, 17.6 Hz, H-2-*trans*-Vinyl), 5.28 (1H, dt, *J* = 1.5, 10.1

Hz, H-2-*cis*-Vinyl), 4.70 (1H, br.dd, *J* = 6.6, 10.1 Hz, H-2), 4.65 (1H, dd, *J* = 2.5, 7.6 Hz, H-5a), 3.73 (3H, s, OMe), 3.43 (1H, d, *J* = 10.6 Hz, H-3a), 3.20 (1H, t, *J* = 10.1 Hz, H-3), 3.01 (1H, ddt, *J* = 2.5, 7.6, 17.7 Hz, H-6A), 2.28 (1H, dq, *J* = 2.5, 18.2 Hz, H-6B) ppm. ^{13}C NMR (150.9 MHz, $CDCl_3$): δ 170.4, 169.9, 138.9, 135.6, 134.8, 134.4, 130.5, 130.0, 125.6, 122.1, 119.8, 95.8, 81.2, 67.2, 54.4, 53.1, 52.3, 38.4 ppm. IR spectrum: ν (CO_2Me) = 1772 cm^{-1}, ν (NC=O) = 1659 cm^{-1}. HRMS (ESI-TOF) $[M+H]^+$ calcd for $C_{19}H_{20}ClNO_4$: 361.0924, found: 361.0942.

7.5 *Methyl (2RS,3SR,3aRS,5aRS,8aSR)-5-(4-chlorophenyl)-4-oxo-2-vinyl-3,3a,4,5,5a,6-hexahydro-2H-cyclopenta[b]furo[2,3-c]pyrrole-3-carboxylate* **(7e)**

The mixture of isomers **6eA/6eB** = 70/30 was used. White crystals, 73%. M.p 124.7–127.1 °C. ^{1}H NMR (600.2 MHz, $CDCl_3$): δ 7.52-7.50 (2H, m, H-3,5-Ph), 7.36-7.34 (2H, m, H-2,6-Ph), 5.98 (br.dt, *J* = 2.3, 5.6 Hz, H-8), 5.89 (1H, ddd, *J* = 6.4, 10.1, 17.4 Hz, H-1-Vinyl), 5.79 (1H, br.dt, *J* = 2.3, 5.6 Hz, H-7), 5.45 (1H, dt, *J* = 1.5, 17.4 Hz, H-2-*trans*-Vinyl), 5.27 (1H, dt, *J* = 1.5, 10.1 Hz, H-2-*cis*-Vinyl), 4.70 (1H, br.dd, *J* = 6.4, 10.1 Hz, H-2), 4.64 (1H, dd, *J* = 2.3, 7.1 Hz, H-5a), 3.72 (3H, s, OMe), 3.43 (1H, d, *J* = 10.6 Hz, H-3a), 3.20 (1H, t, *J* = 10.6 Hz, H-3), 2.98 (1H, ddt, *J* = 2.3, 7.1, 17.9 Hz, H-6A), 2.28 (1H, dq, *J* = 2.3, 17.9 Hz, H-6B) ppm. ^{13}C NMR (150.9 MHz, $CDCl_3$): δ 170.3, 169.9, 136.2, 134.5, 130.8, 130.5, 129.2, 123.2, 118.9, 95.9, 81.2, 67.2, 54.4, 53.2, 52.3, 38.3, 30.3 ppm. IR spectrum: ν (CO_2Me) = 1763 cm^{-1}, ν (NC=O) = 1670 cm^{-1}. HRMS (ESI-TOF) $[M+H]^+$ calcd for $C_{19}H_{20}ClNO_4$: 361.0924, found: 361.0937.

7.6 *Methyl (2RS,3SR,3aRS,5aRS,8aSR)-5-(4-bromophenyl)-4-oxo-2-vinyl-3,3a,4,5,5a,6-hexahydro-2H-cyclopenta[b]furo[2,3-c]pyrrole-3-carboxylate* **(7f)**

The mixture of isomers **6fA/6fB** = 72/28 was used. White crystals, 63%. M.p 127.2–128.8 °C. ^{1}H NMR (600.2 MHz, $CDCl_3$): δ 7.43-7.37 (4H, m, H-Ar), 5.90-5.89 (1H, m, H-7), 5.81 (1H, ddd, *J* = 6.6, 10.6, 17.2 Hz, H-1-Vinyl), 5.71-5.69 (1H, m, H-8), 5.37 (1H, dt, *J* = 1.3, 17.2 Hz, H-2-*trans*-Vinyl), 5.20 (1H, dt, *J* = 1.3, 10.6 Hz, H-2-*cis*-Vinyl), 4.62 (1H, dd, *J* = 6.6, 10.6 Hz, H-2), 4.56 (1H, dd, *J* = 2.5, 7.1 Hz, H-5a), 3.65 (3H, s, OMe), 3.55 (1H, d, *J* = 10.6 Hz, H-3a), 3.12 (1H, t, *J* = 10.6 Hz, H-3), 2.91 (1H, ddt, *J* = 2.4, 7.1, 17.7 Hz, H-6*A*), 2.20 (1H, dq, *J* = 2.5, 17.7 Hz, H-6*B*) ppm. ^{13}C NMR (150.9 MHz, $CDCl_3$): δ 170.3, 170.0, 136.8, 134.5, 132.2, 130.6, 123.5, 119.0, 118.8, 95.9, 81.3, 77.3, 77.1, 76.9, 67.2, 54.5, 53.2, 52.4, 29.8 ppm. IR spectrum: ν (CO_2Me) = 1765 cm^{-1}, ν (NC=O) = 1667 cm^{-1}. HRMS (ESI-TOF) $[M+H]^+$ calcd for $C_{19}H_{20}BrNO_4$: 405.0419, found: 405.0430.

7.7 *Methyl (2RS,3SR,3aRS,5aRS,8aSR)-5-(4-iodophenyl)-4-oxo-2-vinyl-3,3a,4,5,5a,6-hexahydro-2H-cyclopenta[b]furo[2,3-c]pyrrole-3-carboxylate* **(7g)**

The mixture of isomers **6gA/6gB** = 59/41 was used. White crystals, 48%. M.p 121.3–124.0 °C. ^{1}H NMR (600.2 MHz, $CDCl_3$): δ 7.70-7.68 (2H, m, H-3,5-Ph), 7.35-7.33 (2H, m, H-2,6-Ph), 5.98-5.96 (1H, m, H-7), 5.89 (1H, dt, *J* = 2.3, 5.5 Hz, H-1-Vinyl), 5.78-5.77 (1H, m, H-8), 5.44 (1H, dt, *J* = 1.2, 16.8 Hz, H-2-*trans*-Vinyl), 5.27 (1H, dt, *J* = 1.2, 10.1 Hz, H-2-*cis*-Vinyl), 4.69 (1H, dd, *J* = 6.1, 10.1 Hz, H-2), 4.63 (1H, dd, *J* = 2.2, 7.6 Hz, H-5a), 3.72 (3H, s, OMe), 3.42 (1H, d, *J* = 10.1 Hz, H-3a), 3.19 (1H, t, *J* = 10.1 Hz, H-3), 2.99 (1H, ddt, *J* = 2.2, 7.6, 18.2 Hz, H-6*A*), 2.27 (1H, dq, *J* = 2.2, 18.2 Hz, H-6*B*) ppm. ^{13}C NMR (150.9 MHz, $CDCl_3$): δ 170.3, 169.9, 138.1, 137.4, 130.5, 123.6, 119.0, 95.8, 89.5, 81.2, 67.1, 54.4, 53.2, 52.3, 29.7 ppm. IR spectrum: ν (CO_2Me) = 1772 cm^{-1}, ν (NC=O) = 1668 cm^{-1}. HRMS (ESI-TOF) $[M+H]^+$ calcd for $C_{19}H_{20}INO_4$: 453.0281, found: 453.0272.

7.8 *Methyl (2RS,3SR,3aRS,5aRS,8aSR)-5-(3-chloro-4-fluorophenyl)-4-oxo-2-vinyl-3,3a,4,5,5a,6-hexahydro-2H-cyclopenta[b]furo[2,3-c]pyrrole-3-carboxylate* **(7h)**

The mixture of isomers **6hA/6hB** = 66/34 was used. White crystals, 54%. M.p 122.4–123.9 °C. ^{1}H NMR (600.2 MHz, $CDCl_3$): δ 7.70 (1H, dd, *J* = 3.0, 6.6 Hz, H-6-Ph), 7.71-7.38 (1H, m, H-2-Ph), 7.17 (1H, t, *J* =

9.1 Hz, H-3-Ph), 5.98-5.97 (1H, m, H-7), 5.88 (1H, dt, *J* = 6.6, 10.5, 17.1 Hz, H-1-Vinyl), 5.78-5.77 (1H, m, H-8), 5.45 (1H, dd, *J* = 1.0, 17.2 Hz, H-2-*trans*-Vinyl), 5.28 (1H, dd, *J* = 1.0, 10.6 Hz, H-2-*cis*-Vinyl), 4.70 (1H, br.dd, *J* = 6.6, 10.1 Hz, H-2), 4.61 (1H, dd, *J* = 2.5, 7.6 Hz, H-5a), 3.73 (3H, s, OMe), 3.42 (1H, d, *J* = 10.1 Hz, H-3a), 3.21 (1H, t, *J* = 10.6 Hz, H-3), 2.99 (1H, ddt, *J* = 2.5, 7.6, 18.2 Hz, H-6*A*), 2.27 (1H, dq, *J* = 3.0, 7.6 Hz, H-6*B*) ppm. ^{13}C NMR (150.9 MHz, $CDCl_3$): δ 170.3, 169.9, 156.4, 154.8, 134.4, 130.5, 124.5, 121.8, 119.0, 116.8, 95.9, 81.4, 67.3, 54.2, 53.1, 52.3, 38.2, 30.3 ppm. IR spectrum: ν (CO_2Me) = 1772 cm^{-1}, ν (NC=O) = 1654 cm^{-1}. HRMS (ESI-TOF) [M+H]$^+$ calcd for $C_{19}H_{19}FClNO_4$: 379.0830, found: 379.0842.

8. *General method for synthesis of methyl 7-methylcyclopenta[b]furo[2,3-c]pyrrole-3-carboxylate* 7p

The catalyst 2 (0.005 mmol, 3.1 mg) was added to the solution of ester **6pA** in CH_2Cl_2 (1 mmol in 20 mL). Reaction mixture was stirred at 120 °C for 10 min under microwave radiation (200 W). After cooling, the solvent was evaporated under reduced pressure, the target tricycle **7p** was purified by flash-chromatography on silica gel (ethyl acetate:hexane = 12:88) giving white powder (61% yield, see Table 2). Control of the purity of the obtained compounds was carried out using TLC (ethyl acetate : hexane = 1:3).

9. *General method for synthesis of methyl 7-methylcyclopenta[b]furo[2,3-c]pyrrole-3-carboxylates* (7i–p)

The catalyst 2 (0.005 mmol, 3.1 mg) was added to the solution of ester **6i–p** in CH_2Cl_2 (1 mmol in 20 mL). Reaction mixture was stirred at 120 °C for 10 min under microwave radiation (200 W). After cooling, the solvent was evaporated under reduced pressure, the target tricycles **7i–p** were purified by flash-chromatography on silica gel (ethyl acetate:hexane = 12:88) giving white powders. Control of the purity of the obtained compounds was carried out using TLC (ethyl acetate : hexane = 1:3).

9.1 *Methyl (2RS,3SR,3aRS,5aRS,8aSR)-7-methyl-4-oxo-5-phenyl-2-vinyl-3,3a,4,5,5a,6-hexahydro-2H-cyclopenta[b]furo[2,3-c]pyrrole-3-carboxylate* **(7i)**

The mixture of isomers **6iA/6iB** = 55/45 was used. White crystals, 50%. M.p 125.5–127.3 °C. ^{1}H NMR (600.2 MHz, $CDCl_3$): δ 7.54-7.53 (2H, m, H-2,6-Ar), 7.39-7.35 (2H, m, H-3,5-Ar), 7.20-7.18 (1H, m, H-4-Ar), 5.88 (1H, ddd, *J* = 6.6, 10.6, 17.2 Hz, H-1-Vinyl), 5.43-5.41 (2H, m, H-8 and H-2-*trans*-Vinyl), 5.26 (1H, dd, *J* = 1.0, 10.6 Hz, H-2-*cis*-Vinyl), 4.68-4.65 (2H, m, H-2 and H-5a), 3.72 (3H, s, OMe), 3.40 (1H, d, *J* = 10.6 Hz, H-3a), 3.17 (1H, t, *J* = 10.6 Hz, H-3), 2.88 (1H, br.dd, *J* = 7.6, 17.7 Hz, H-6*A*), 2.18 (1H, br.d, *J* = 17.7 Hz, H-6*B*), 1.76 (3H, s, Me-7) ppm. ^{13}C NMR (150.9 MHz, $CDCl_3$): δ δ170.4, 170.1, 145.4, 137.7, 134.6, 129.1, 125.5, 124.7, 124.0, 122.1, 118.7, 96.0, 80.7, 68.1, 54.5, 53.3, 52.2, 42.3, 29.7 ppm. IR spectrum: ν (CO_2Me) = 1769 cm^{-1}, ν (NC=O) = 1665 cm^{-1}. HRMS (ESI-TOF) [M+H]$^+$ calcd for $C_{20}H_{23}NO_4$: 341.1471, found: 341.1461.

9.2 *Methyl (2RS,3SR,3aRS,5aRS,8aSR)-7-methyl-4-oxo-5-(m-tolyl)-2-vinyl-3,3a,4,5,5a,6-hexahydro-2H-cyclopenta[b]furo[2,3-c]pyrrole-3-carboxylate* **(7j)**

The mixture of isomers **6jA/6jB** = 45/55 was used. White crystals, 38%. M.p 126.3–128.4 °C. ^{1}H NMR (600.2 MHz, $CDCl_3$): δ 7.37 (1H, s, H-2-Ph), 7.28-7.24 (2H, m, H-Ph), 7.01 (1H, m, H-Ph), 5.89 (1H, ddd, *J* = 6.1, 10.1, 16.7 Hz, H-1-Vinyl), 5.44-5.41 (2H, m, H-8 and H-2-*trans*-Vinyl), 5.26 (1H, dd, *J* = 1.0, 10.6 Hz, H-2-*cis*-Vinyl), 4.68-4.65 (2H, m, H-2 and H-5a), 3.72 (3H, s, OMe), 3.39 (1H, d, *J* = 10.6 Hz,

H-6*A*), 3.16 (1H, t, *J* = 10.6 Hz, H-3), 2.86 (1H, br.dd, *J* = 7.1, 17.7 Hz, H-6*B*), 2.36 (3H, s, Me-Ph), 1.76 (3H, s, Me-7) ppm. ^{13}C NMR (150.9 MHz, $CDCl_3$): δ 170.4, 145.6, 139.0, 137.6, 134.8, 129.0, 126.6, 124.7, 123.2, 119.5, 118.7, 96.1, 80.8, 68.3, 54.6, 53.3, 52.3, 42.3, 21.7, 16.8 ppm. IR spectrum: ν (CO_2Me) = 1767 cm^{-1}, ν (NC=O) = 1670 cm^{-1}. HRMS (ESI-TOF) $[M+H]^+$ calcd for $C_{21}H_{25}NO_4$: 355.4180, found: 355.4187.

9.3 *Methyl (2RS,3SR,3aRS,5aRS,8aSR)-7-methyl-4-oxo-5-(p-tolyl)-2-vinyl-3,3a,4,5,5a,6-hexahydro-2H-cyclopenta[b]furo[2,3-c]pyrrole-3-carboxylate* **(7k)**

The mixture of isomers **6kA/6kB** = 50/50 was used. White crystals, 44%. M.p 123.6–125.7 °C. ^1H NMR (600.2 MHz, $CDCl_3$): δ 7.41 (2H, d, *J* = 9.1 Hz, H-3,5-Ph), 7.19 (2H, d, *J* = 8.1 Hz, H-2,6-Ph), 5.89 (1H, ddd, *J* = 6.1, 10.1, 17.2 Hz, H-1-Vinyl), 5.43-5.40 (2H, m, H-8 and H-2-*trans*-Vinyl), 5.25 (1H, dd, *J* = 3.0, 10.1 Hz, H-2-*cis*-Vinyl), 4.68 (2H, m, H-2 and H-5a), 3.71 (3H, s, OMe), 3.38 (1H, d, *J* = 11.1 Hz, H-3a), 3.15 (1H, t, *J* = 10.1 Hz, H-3), 2.85 (1H, br.dd, *J* = 7.1, 18.2 Hz, H-6*A*), 2.33 (3H, s, Me-Ph), 2.15 (1H, d, *J* = 17.2 Hz, H-6*B*) ppm. ^{13}C NMR (150.9 MHz, $CDCl_3$): δ 170.2, 170.1, 145.5, 136.0, 135.4, 134.7, 129.6, 125.9, 124.7, 122.3, 118.6, 117.6, 115.9, 114.0, 96.1, 80.7, 80.6, 68.2, 65.6, 55.3, 54.5, 53.3, 52.2, 50.1, 35.4, 29.7, 20.9, 16.7 ppm. IR spectrum: ν (CO_2Me) = 1768 cm^{-1}, ν (NC=O) = 1673 cm^{-1}. HRMS (ESI-TOF) $[M+H]^+$ calcd for $C_{21}H_{25}NO_4$: 355.4180, found: 355.4193.

9.4 *Methyl (2RS,3SR,3aRS,5aRS,8aSR)-5-(4-isopropylphenyl)-7-methyl-4-oxo-2-vinyl-3,3a,4,5,5a,6-hexahydro-2H-cyclopenta[b]furo[2,3-c]pyrrole-3-carboxylate* **(7l)**

The mixture of isomers **6lA/6lB** = 87/13 was used. White crystals, 62%. M.p 124.5–126.2 °C. ^1H NMR (600.2 MHz, $CDCl_3$): δ 7.44 (2H, dd, *J* = 2.0, 6.6 Hz, H-2,6-Ph), 7.24 (2H, *J* = 1.5, 6.6 Hz, H-3,5-Ph), 5.86 (1H, ddd, *J* = 6.1, 10.1, 16.7 Hz, H-1-Vinyl), 5.43-5.40 (2H, m, H-8 and H-2-*trans*-Vinyl), 5.25 (1H, dd, *J* = 1.5, 9.6 Hz, H-2-*cis*-Vinyl), 4.67-4.64 (2H, m, H-2 and H-5a), 3.72 (3H, s, OMe), 3.39 (1H, d, *J* = 10.6 Hz, H-3a), 3.15 (1H, t, *J* = 10.6 Hz, H-3), 2.85 (1H, br.dd, *J* = 6.6, 13.6 Hz, H-6*A*), 1.76 (3H, s, Me-7), 1.25-1.22 (6H, m, CH*Me*2) ppm. ^{13}C NMR (150.9 MHz, $CDCl_3$): δ 170.2, 170.1, 146.3, 145.5, 135.3, 134.7, 127.0, 124.7, 124.0, 122.3, 118.6, 96.1, 80.7, 68.2, 54.5, 53.2, 52.2, 52.0, 42.3, 33.7 ppm. IR spectrum: ν (CO_2Me) = 1773 cm^{-1}, ν (NC=O) = 1663 cm^{-1}. HRMS (ESI-TOF) $[M+H]^+$ calcd for $C_{23}H_{29}NO_4$: 383.1940, found: 383.1957.

9.5 *Methyl (2RS,3SR,3aRS,5aRS,8aSR)-5-benzyl-7-methyl-4-oxo-2-vinyl-3,3a,4,5,5a,6-hexahydro-2H-cyclopenta[b]furo[2,3-c]pyrrole-3-carboxylate* **(7m)**

The mixture of isomers **6mA/6mB** = 42/58 was used. White crystals, 39%. M.p 123.7–125.3 °C. ^1H NMR (600.2 MHz, $CDCl_3$): δ 7.39 (2H, t, *J* = 7.6 Hz, H-3,5-Ph), 7.33-7.31 (3H, m, H-2,4,6-Ph), 5.82 (1H, ddd, *J* = 6.6, 10.6, 17.2 Hz, H-1-Vinyl), 5.35-5.33 (2H, m, H-8 and H-2-*trans*-Vinyl), 5.21 (1H, dd, *J* = 1.0, 10.6 Hz, H-2-*cis*-Vinyl), 5.17 (1H, d, *J* = 14.6 Hz, N-CH*A*-Ph), 4.42 (1H, dd, *J* = 6.6, 10.6 Hz, H-6*A*), 3.87 (1H, d, *J* = 14.6 Hz, N-CH*B*-Ph), 3.80 (3H, s, OMe), 3.24 (1H, d, *J* = 10.6 Hz, H-3a), 3.10 (1H, t, *J* = 10.6 Hz, H-3), 2.58 (1H, br.dd, *J* = 7.6, 17.7 Hz, H-6*B*), 1.75 (3H, s, Me-7) ppm. ^{13}C NMR (150.9 MHz, $CDCl_3$): δ 170.9, 170.3, 145.0, 135.6, 128.8, 128.4, 127.8, 124.7, 118.5, 96.8, 80.6, 65.8, 53.9, 53.2, 51.2, 45.4, 40.3 ppm. IR spectrum: ν (CO_2Me) = 1771 cm^{-1}, ν (NC=O) = 1664 cm^{-1}. HRMS (ESI-TOF) $[M+H]^+$ calcd for $C_{21}H_{24}NO_4$: 354.4180, found: 354.4193.

9.6 *Methyl (2RS,3SR,3aRS,5aRS,8aSR)-5-(2-chlorophenyl)-7-methyl-4-oxo-2-vinyl-3,3a,4,5,5a,6-hexahydro-2H-cyclopenta[b]furo[2,3-c]pyrrole-3-carboxylate* **(7n)**

The mixture of isomers **6nA/6nB** = 75/25 was used. White crystals, 59%. M.p 123.7–125.3 °C. ^1H NMR (600.2 MHz, $CDCl_3$): δ 7.34-7.29 (3H, m, H-Ar), 5.92 (1H, ddd, *J* = 6.6, 10.6, 17.2 Hz), 5.47-5.44 (2H, m, H-8 and H-2-*trans*-Vinyl), 5.26 (1H, dd, *J* = 1.0, 10.6 Hz, H-2-*cis*-Vinyl), 4.84 (1H, dd, *J* = 6.6 Hz, H-2),

4.63 (1H, dd, *J* = 2.0, 7.8 Hz, H-5a), 3.70 (3H, s, OMe), 3.36 (1H, d, *J* = 10.6 Hz, H-3a), 3.18 (1H, t, *J* = 10.6 Hz, H-3), 2.59 (1H, br.dd, *J* = 7.1, 17.7 Hz, H-6*A*), 2.17 (1H, br.d, *J* = 17.7 Hz, H-6*B*), 1.79 (3H, br.s, Me-7) ppm. ^{13}C NMR (150.9 MHz, $CDCl_3$): δ 170.2, 170.1, 146.3, 145.5, 135.3, 134.7, 127.0, 124.6, 122.3, 118.7, 96.1, 80.7, 68.2, 54.5, 53.2, 52.2, 42.3, 33.7, 29.7, 24.0, 22.7, 16.7 ppm. IR spectrum: ν (CO_2Me) = 1772 cm^{-1}, ν (NC=O) = 1665 cm^{-1}. HRMS (ESI-TOF) $[M+H]^+$ calcd for $C_{20}H_{21}ClNO_4$: 374.1081, found: 374.1093.

9.7 *Methyl (2RS,3SR,3aRS,5aRS,8aSR)-5-(3-chlorophenyl)-7-methyl-4-oxo-2-vinyl-3,3a,4,5,5a,6-hexahydro-2H-cyclopenta[b]furo[2,3-c]pyrrole-3-carboxylate* **(7o)**

The mixture of isomers **6oA/6oB** = 83/17 was used. White crystals, 61%. M.p 124.5–125.9 °C. ^{1}H NMR (600.2 MHz, $CDCl_3$): δ 7.44-7.24 (4H, m, H-Ar), 5.88 (1H, ddd, *J* = 7.1, 10.1, 17.2 Hz, H-1-Vinyl), 5.43-5.41 (2H, m, H-8 and H-2-*trans*-Vinyl), 5.25 (1H, d, *J* = 10.1 Hz, H-2-*cis*-Vinyl), 4.67-4.64 (2H, m, H-2 and H-5a), 3.72 (3H, s, OMe), 3.38 (1H, d, *J* = 10.1 Hz, H-3a), 3.15 (1H, t, *J* = 10.1 Hz, H-3), 2.87 (1H, dd, *J* = 7.6, 17.7 Hz, H-6*A*), 2.20 (1H, br.d, *J* = 17.7 Hz, H-6*B*), 1.76 (3H, s, Me-7) ppm. ^{13}C NMR (150.9 MHz, $CDCl_3$): δ 170.2, 146.3, 145.5, 134.7, 127.0, 124.6, 122.3, 118.7, 96.1, 80.7, 68.2, 54.5, 53.2, 52.2, 42.3, 33.7, 24.0, 16.7 ppm. IR spectrum: ν (CO_2Me) = 1771 cm^{-1}, ν (NC=O) = 1664 cm^{-1}. HRMS (ESI-TOF) $[M+H]^+$ calcd for $C_{20}H_{21}ClNO_4$: 374.1081, found: 374.1089.

9.8 *Methyl (2RS,3SR,3aRS,5aRS,8aSR)-5-(4-bromophenyl)-7-methyl-4-oxo-2-vinyl-3,3a,4,5,5a,6-hexahydro-2H-cyclopenta[b]furo[2,3-c]pyrrole-3-carboxylate* **(7p)**

The mixture of isomers **6pA/6pB** = 57/43 was used. White crystals, 52%. M.p 127.3–129.8 °C. ^{1}H NMR (600.2 MHz, $CDCl_3$): δ 7.75-7.44 (4H, m, H-Ar), 5.88 (1H, ddd, *J* = 6.6, 10.6, 17.2 Hz), 5.43-5.40 (2H, m, H-8 and H-2-*trans*-Vinyl), 5.26 (1H, dd, *J* = 1.0, 10.6 Hz, H-2-*cis*-Vinyl), 4.65-4.62 (2H, m, H-2 and H-5a), 3.72 (3H, s, OMe), 3.38 (1H, d, *J* = 10.6 Hz, H-3a), 3.17 (1H, t, *J* = 10.6 Hz, H-3), 2.90 (1H, br.dd, *J* = 7.6, 17.7 Hz, H-6*A*), 2.17 (1H, br.d, *J* = 17.7 Hz, H-6*B*), 1.77 (3H, br.s, Me-7) ppm. ^{13}C NMR (150.9 MHz, $CDCl_3$): δ 170.6, 145.4, 136.9, 134.6, 132.2, 130.2, 129.2, 126.4, 124.8, 123.4, 118.9, 118.5, 96.1, 80.9, 68.0, 54.6, 53.4, 52.3, 44.4, 42.4, 31.7, 29.8 ppm. IR spectrum: ν (CO_2Me) = 1773 cm^{-1}, ν (NC=O) = 1674 cm^{-1}. HRMS (ESI-TOF) $[M+H]^+$ calcd for $C_{20}H_{21}BrNO_4$: 419.2870, found: 419.2889.

10. *General method for synthesis of methyl 2,6a-divinylhexahydro-2H-furo[2,3-c]pyrrole-3-carboxylates* 8a *and* 8b

Catalyst 1 (0.02 mmol, 3.3 mg) was added to the solution of ester **6eA or 6fA** (4 mmol) in chloroform (100 mL). Reaction mixture in a Schlenk flask was boiled in an atmosphere of argon for 15 min. After cooling and separation of the solvent, substance **8a** or **8b** correspondently were purified by column chromatography on silica gel (eluent ethyl acetate : hexane = 1:5) providing the goal substances as white crystals.

10.1 *Methyl (2RS,3SR,6RS,6aRS)-6-allyl-5-(4-chlorophenyl)-4-oxo-2,6a-divinylhexahydro-2H-cyclopenta[b]furan-3-carboxylate* **(8a)**

The mixture of isomers **6eA/6eB** = 70/30 was used. White crystals, 30 mg, 2%. M.p 110.5–111.3 °C. ^{1}H NMR (600.2 MHz, $CDCl_3$): δ 7.43-7.41 (2H, m, H-3,5-Ar), 7.35-7.32 (2H, m, H-2,6-Ar), 6.08 (1H, dd, *J* = 11.1, 17.2 Hz, H-1-Vinyl-6a), 5.85 (1H, ddd, *J* = 7.1, 10.1, 17.2 Hz, H-1-Vinyl-2), 5.65 (1H, dd, *J* = 1.5, 17.2 Hz, H-2-*trans*-Vinyl-6a), 5.61 (1H, ddt, *J* = 6.6, 10.1, 18.0 Hz, H-2-Allyl), 5.44-5.41 (1H, m, H-2-*trans*-Vinyl-2), 5.39 (1H, dd, *J* = 1.5, 10.1 Hz, H-2-*cis*-Vinyl-6a), 5.23-5.21 (1H, m, H-2-*cis*-Vinyl-2), 5.12 (1H, br.d, *J* = 10.6 Hz, H-3-*cis*-Allyl), 5.03 (1H, dq, *J* = 1.5, 17.2 Hz, H-3-*trans*-Allyl), 4.76 (1H, dd, *J* =

7.1, 10.1 Hz, H-2), 4.33 (1H, dd, *J* = 3.5, 6.1 Hz, H-6), 3.77 (3H, s, OMe), 3.48 (1H, d, *J* = 7.1 Hz, H-3a), 3.07 (1H, dd, *J* = 7.1, 10.1 Hz, H-3), 2.44-2.40 (1H, m, H-1*A*-Allyl), 2.31-2.27 (1H, m, H-1*B*-Allyl) ppm. ^{13}C NMR (150.9 MHz, $CDCl_3$): δ 170.0, 169.5, 136.2, 135.6, 135.1, 131.4, 131.2, 129.3, 124.7, 120.2, 118.5, 118.1, 87.3, 80.3, 68.2, 54.1, 52.1, 50.3, 33.9 ppm. HRMS (ESI-TOF) $[M+H]^+$ calcd for $C_{20}H_{21}ClNO_{44}$: 387.1208, found: 387.1217.

10.2 *Methyl (2RS,3SR,6RS,6aRS)-6-allyl-5-(4-bromophenyl)-4-oxo-2,6a-divinylhexahydro-2H-cyclopenta[b]furan-3-carboxylate* **(8b)**

The mixture of isomers **6fA/6fB** = 72/28 was used. White crystals, 50 mg, 3%. M.p 113.1–114.4 °C. ^{1}H NMR (600.2 MHz, $CDCl_3$): δ (2H, m, H-3,5-Ar), 7.15 (2H, m, H-2,6-Ar), 5.99 (1H, dd, *J* = 10.6, 16.7 Hz, H-1-6-Vinyl), 5.92 (1H, ddd, *J* = 7.1, 10.3, 17.1 Hz, H-1-2-Vinyl), 5.71 (1H, ddd, *J* = 6.8, 10.3, 18.0 Hz, H-2-Allyl), 5.57-5.49 (2H, m, H-2-*trans*-Vinyl and H-3-*trans*-Allyl), 5.31-5.25 (2H, m, H-2-*cis*-Vinyl), 4.92-4.89 (2H, m, H-3-*cis*-Allyl and H-2-*trans*-Vinyl), 4.79 (1H, dd, *J* = 7.3, 10.3 Hz, H-2), 4.10 (1H, dd, *J* = 3.5, 6.0 Hz, H-5), 3.76 (3H, s, OMe), 3.39 (1H, d, *J* = 7.6 Hz, H-3a), 3.04 (1H, dd, *J* = 7.6, 11.1 Hz, H-3), 2.51-2.46 (1H, m, H-1*A*-Allyl), 2.33-2.28 (1H, m, H-1*B*-Allyl) ppm. ^{13}C NMR (150.9 MHz, $CDCl_3$): δ 170.1, 169.6, 139.0, 137.2, 135.6, 133.8, 132.2, 127.0, 123.9, 118.2, 117.9, 116.5, 85.9, 81.0, 66.6, 55.4, 52.1, 50.1, 32.7 ppm. HRMS (ESI-TOF) $[M+H]^+$ calcd for $C_{21}H_{22}BrNO_4$: 432.3170, found: 432.3163.

11. *Experimental part for X-ray analysis*

The X-ray diffraction data were collected on a Bruker Kappa Apex II automatic four-circle diffractometer equipped with an area detector (Mo-Kα sealed-tube X-ray source, λ = 0.71073 Å, graphite monochromator) at room temperature.

The data frames were collected using the program APEX2 and processed using the program SAINT routine within APEX2. The unit cell parameters were refined over the whole dataset [7]. The data were corrected for absorption on the multi-scan technique as implemented in SADABS [8]. The structures were solved by direct methods using SHELXS and refined by full-matrix least-squares on F^2 using SHELXL software [9] in the anisotropic approximation for all non hydrogen atoms. Hydrogen atoms on carbon were calculated in ideal positions with isotropic displacement parameters set to 1.2×U_{eq} (CH and CH_2 groups) or 1.5×U_{eq} (CH_3 groups) of the attached atoms. Tables and pictures for structures were generated using Olex2 [10] as GUI. Crystallographic data and structural refinements are summarized in Table S1.

X-ray diffraction experiments were performed at the Center for Shared Use of Physical Methods of Investigation at the Frumkin Institute of Physical Chemistry and Electrochemistry, RAS (CKP FMI IPCE RAS). Atomic coordinates for compounds **6eA**, **6eB** and **8b** have been deposited with the Cambridge Crystallographic Data Centre. CCDC numbers are 2023634, 2023635 and 2025199 respectively. The supplementary crystallographic data can be obtained free of charge from the Cambridge Crystallographic Data Centre via www.ccdc.cam.ac.uk/data_request/cif.

Table S1. Crystal data and structure refinement for **6eA** and **6eB**.

Identification code	**6eA**	**6eB**
CCDC number	2023634	2023635
Empirical formula	$C_{19}H_{18}ClNO_4$	$C_{19}H_{18}ClNO_4$
Formula weight	359.79	359.79
Temperature/K	296(2)	296(2)
Crystal system	triclinic	orthorhombic
Space group	P-1	$P2_12_12_1$
a/Å	7.7160(4)	7.1657(3)
b/Å	10.7213(6)	9.8809(5)
c/Å	11.2953(6)	24.4894(12)
α/°	106.864(2)	90
β/°	97.702(2)	90
γ/°	98.449(2)	90
Volume/Å^3	868.98(8)	1733.94(14)
Z	2	4
ρ_{calc}g/cm^3	1.375	1.378
μ/mm^{-1}	0.243	0.244
F(000)	376.0	752.0
Crystal size/mm^3	0.400 × 0.220 × 0.040	0.400 × 0.180 × 0.060
Radiation	MoKα (λ = 0.71073)	MoKα (λ = 0.71073)
2Θ range for data collection/°	8.538 to 59.996	7.568 to 59.982
Index ranges	$-10 \le h \le 10$, $-15 \le k \le 15$, $-15 \le l \le 15$	$-10 \le h \le 10$, $-13 \le k \le 13$, $-34 \le l \le 34$
Reflections collected	12339	24511
Independent reflections	5027 [R_{int} = 0.0225, R_{sigma} = 0.0369]	5024 [R_{int} = 0.0475, R_{sigma} = 0.0483]
Data/restraints/parameters	5027/0/227	5024/0/228
Goodness-of-fit on F^2	1.031	1.007
Final R indexes [I>=2σ (I)]	R_1 = 0.0451, wR_2 = 0.1033	R_1 = 0.0479, wR_2 = 0.0925
Final R indexes [all data]	R_1 = 0.0806, wR_2 = 0.1179	R_1 = 0.0992, wR_2 = 0.1103
Largest diff. peak/hole / e Å^{-3}	0.25/-0.32	0.17/-0.22

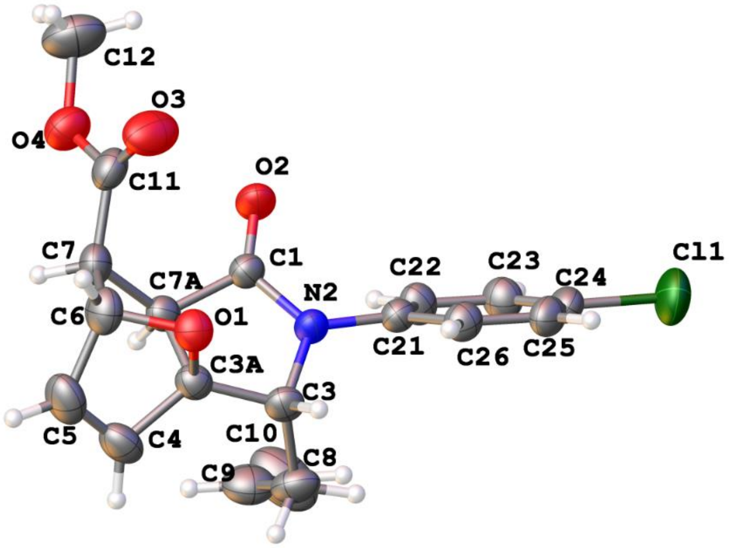

Figure S1. Structure of **6eA** (296 K) $C_{19}H_{18}ClNO_4$ (CCDC number 2023634).

Table S2. Bond Lengths for **6eA.**

Atom	Atom	Length/Å	Atom	Atom	Length/Å
Cl(1)	C(24)	1.7407(16)	C(3A)	C(4)	1.515(2)
O(1)	C(3A)	1.4429(18)	C(3)	C(8)	1.532(2)
O(1)	C(6)	1.4335(19)	C(8)	C(9)	1.486(3)
O(2)	C(1)	1.2152(16)	C(9)	C(10)	1.289(3)
O(3)	C(11)	1.195(2)	C(4)	C(5)	1.317(3)
O(4)	C(11)	1.3311(19)	C(5)	C(6)	1.513(3)
O(4)	C(12)	1.443(2)	C(6)	C(7)	1.563(2)
N(2)	C(1)	1.3664(17)	C(7)	C(11)	1.505(2)
N(2)	C(3)	1.4776(17)	C(21)	C(22)	1.388(2)
N(2)	C(21)	1.4266(18)	C(21)	C(26)	1.387(2)
C(1)	C(7A)	1.519(2)	C(22)	C(23)	1.380(2)
C(7A)	C(3A)	1.5550(18)	C(23)	C(24)	1.378(2)
C(7A)	C(7)	1.548(2)	C(24)	C(25)	1.377(2)
C(3A)	C(3)	1.509(2)	C(25)	C(26)	1.380(2)

Table S3. Bond Angles for **6eA.**

Atom	Atom	Atom	Angle/°	Atom	Atom	Atom	Angle/°
C(6)	O(1)	C(3A)	96.08(11)	C(5)	C(4)	C(3A)	105.30(15)
C(11)	O(4)	C(12)	116.19(15)	C(4)	C(5)	C(6)	106.21(15)
C(1)	N(2)	C(3)	115.13(11)	O(1)	C(6)	C(5)	101.46(14)
C(1)	N(2)	C(21)	124.19(11)	O(1)	C(6)	C(7)	101.46(12)
C(21)	N(2)	C(3)	120.55(11)	C(5)	C(6)	C(7)	106.76(14)
O(2)	C(1)	N(2)	125.60(13)	C(7A)	C(7)	C(6)	99.95(11)
O(2)	C(1)	C(7A)	126.85(12)	C(11)	C(7)	C(7A)	115.12(12)
N(2)	C(1)	C(7A)	107.50(11)	C(11)	C(7)	C(6)	113.31(13)
C(1)	C(7A)	C(3A)	101.76(11)	O(3)	C(11)	O(4)	123.52(16)
C(1)	C(7A)	C(7)	120.56(12)	O(3)	C(11)	C(7)	126.04(15)
C(7)	C(7A)	C(3A)	101.85(11)	O(4)	C(11)	C(7)	110.38(14)
O(1)	C(3A)	C(7A)	99.31(11)	C(22)	C(21)	N(2)	120.40(12)
O(1)	C(3A)	C(3)	109.69(11)	C(26)	C(21)	N(2)	119.88(13)
O(1)	C(3A)	C(4)	101.36(12)	C(26)	C(21)	C(22)	119.61(14)
C(3)	C(3A)	C(7A)	106.68(11)	C(23)	C(22)	C(21)	119.80(14)
C(3)	C(3A)	C(4)	127.35(13)	C(24)	C(23)	C(22)	119.66(14)
C(4)	C(3A)	C(7A)	108.95(12)	C(23)	C(24)	Cl(1)	119.51(12)
N(2)	C(3)	C(3A)	101.45(10)	C(25)	C(24)	Cl(1)	119.11(12)
N(2)	C(3)	C(8)	112.53(12)	C(25)	C(24)	C(23)	121.38(14)
C(3A)	C(3)	C(8)	115.56(13)	C(24)	C(25)	C(26)	118.80(14)
C(9)	C(8)	C(3)	114.31(14)	C(25)	C(26)	C(21)	120.75(14)
C(10)	C(9)	C(8)	124.9(2)				

Table S4. Hydrogen Bonds for **6eA.**

D	H	A	d(D-H)/Å	d(H-A)/Å	d(D-A)/Å	D-H-A/°
C(25)	H(25)	O(3)[1]	0.93	2.44	3.3496(19)	164.7

[1]1-X,1-Y,1-Z

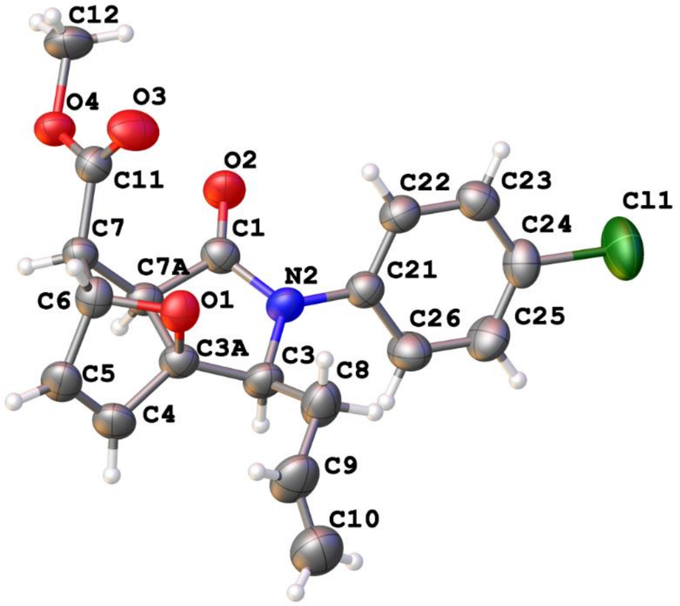

Figure S2. Structure of **6eB** (296 K) $C_{19}H_{18}ClNO_4$ (CCDC number 2023635).

Table S5. Bond Lengths for **6eB.**

Atom	Atom	Length/Å	Atom	Atom	Length/Å
Cl(1)	C(24)	1.742(3)	C(3A)	C(4)	1.504(4)
O(1)	C(3A)	1.446(3)	C(3)	C(8)	1.509(4)
O(1)	C(6)	1.439(4)	C(8)	C(9)	1.497(5)
O(2)	C(1)	1.215(3)	C(9)	C(10)	1.265(6)
O(3)	C(11)	1.190(4)	C(4)	C(5)	1.318(5)
O(4)	C(11)	1.332(4)	C(5)	C(6)	1.507(4)
O(4)	C(12)	1.444(3)	C(6)	C(7)	1.569(4)
N(2)	C(1)	1.379(4)	C(7)	C(11)	1.505(4)
N(2)	C(3)	1.479(4)	C(21)	C(22)	1.385(4)
N(2)	C(21)	1.420(4)	C(21)	C(26)	1.393(4)
C(1)	C(7A)	1.514(4)	C(22)	C(23)	1.374(5)
C(7A)	C(3A)	1.544(4)	C(23)	C(24)	1.380(4)
C(7A)	C(7)	1.548(4)	C(24)	C(25)	1.372(5)
C(3A)	C(3)	1.513(4)	C(25)	C(26)	1.375(5)

Table S6. Bond Angles for **6eB.**

Atom	Atom	Atom	Angle/°	Atom	Atom	Atom	Angle/°
C(6)	O(1)	C(3A)	95.7(2)	C(5)	C(4)	C(3A)	105.5(3)
C(11)	O(4)	C(12)	115.3(3)	C(4)	C(5)	C(6)	106.1(3)
C(1)	N(2)	C(3)	113.9(2)	O(1)	C(6)	C(5)	101.8(2)
C(1)	N(2)	C(21)	123.8(2)	O(1)	C(6)	C(7)	101.5(2)
C(21)	N(2)	C(3)	122.1(2)	C(5)	C(6)	C(7)	106.3(2)
O(2)	C(1)	N(2)	125.1(3)	C(7A)	C(7)	C(6)	99.3(2)
O(2)	C(1)	C(7A)	127.1(3)	C(11)	C(7)	C(7A)	113.3(2)
N(2)	C(1)	C(7A)	107.7(2)	C(11)	C(7)	C(6)	112.9(2)
C(1)	C(7A)	C(3A)	101.7(2)	O(3)	C(11)	O(4)	124.5(3)
C(1)	C(7A)	C(7)	120.0(2)	O(3)	C(11)	C(7)	125.6(3)
C(3A)	C(7A)	C(7)	102.5(2)	O(4)	C(11)	C(7)	109.9(2)
O(1)	C(3A)	C(7A)	99.2(2)	C(22)	C(21)	N(2)	121.2(3)
O(1)	C(3A)	C(3)	111.8(3)	C(22)	C(21)	C(26)	118.9(3)
O(1)	C(3A)	C(4)	101.8(2)	C(26)	C(21)	N(2)	119.9(3)
C(3)	C(3A)	C(7A)	106.0(2)	C(23)	C(22)	C(21)	120.6(3)
C(4)	C(3A)	C(7A)	108.6(3)	C(22)	C(23)	C(24)	119.5(3)
C(4)	C(3A)	C(3)	126.2(2)	C(23)	C(24)	Cl(1)	119.5(3)
N(2)	C(3)	C(3A)	101.3(2)	C(25)	C(24)	Cl(1)	119.4(2)
N(2)	C(3)	C(8)	114.0(3)	C(25)	C(24)	C(23)	121.1(3)
C(8)	C(3)	C(3A)	114.2(3)	C(24)	C(25)	C(26)	119.3(3)
C(9)	C(8)	C(3)	111.7(3)	C(25)	C(26)	C(21)	120.7(3)
C(10)	C(9)	C(8)	128.1(4)				

Table S7. Hydrogen Bonds for **6eB.**

D	H	A	d(D-H)/Å	d(H-A)/Å	d(D-A)/Å	D-H-A/°
C(7)	H(7)	O(3)[1]	0.98	2.66	3.228(4)	117.5
C(25)	H(25)	O(2)[2]	0.93	2.54	3.370(4)	149.1

[1]-1/2+X,3/2-Y,1-Z; [2]1-X,-1/2+Y,1/2-Z

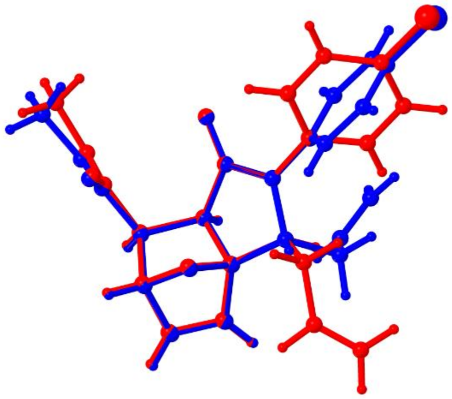

Figure S3. The overlay of molecules of *trans*-**6eA** (blue) and *cis*-**6eB** (red) methyl 3-allyl-2-(4-chlorophenyl)-1-oxo-1,2,3,6,7,7a-hexahydro-3a,6-epoxyisoindole-7-carboxylates according to the single crystal XRD.

Crystallographic data and structural refinements for compound **8b** are summarized in Table S8 (CCDC number is 2025199).

Table S8. Crystal data and structure refinement for **8b.**

Empirical formula	$C_{21}H_{22}BrNO_4$
Formula weight	432.30
Temperature/K	296(2)
Crystal system	monoclinic
Space group	$P2_1/n$
a/Å	8.0675(9)
b/Å	10.3049(10)
c/Å	24.629(2)
α/°	90
β/°	98.915(4)
γ/°	90
Volume/Å^3	2022.8(3)
Z	4
ρ_{calc}g/cm^3	1.420
μ/mm^{-1}	2.058
F(000)	888.0
Crystal size/mm^3	0.400 × 0.320 × 0.300
Radiation	MoKα (λ = 0.71073)
2Θ range for data collection/°	8.592 to 54.994
Index ranges	-10 ≤ h ≤ 10, -6 ≤ k ≤ 13, -31 ≤ l ≤ 31
Reflections collected	17096
Independent reflections	4626 [R_{int} = 0.1006, R_{sigma} = 0.1241]

Data/restraints/parameters	4626/0/245
Goodness-of-fit on F^2	1.015
Final R indexes [I>=2σ (I)]	R_1 = 0.0629, wR_2 = 0.1300
Final R indexes [all data]	R_1 = 0.1733, wR_2 = 0.1621
Largest diff. peak/hole / e Å^{-3}	0.57/-0.33

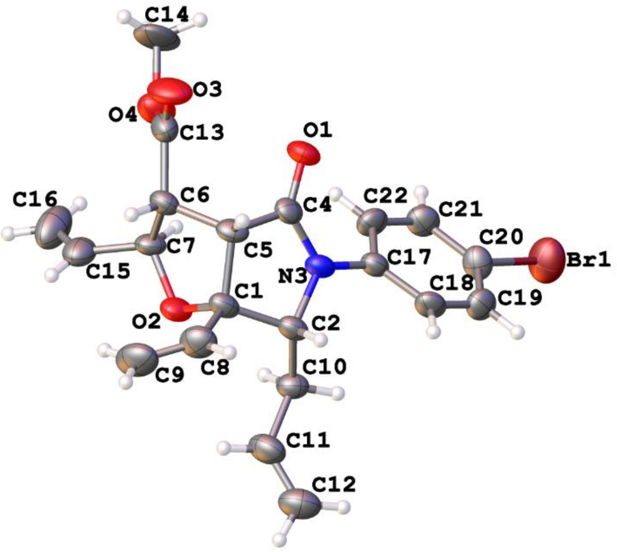

Figure S4. Structure of **8b** (CCDC number is 2025199).

Table S9. Bond Lengths for **8b.**

Atom	Atom	Length/Å	Atom	Atom	Length/Å
Br(1)	C(20)	1.893(4)	C(5)	C(6)	1.539(6)
O(1)	C(4)	1.229(5)	C(6)	C(13)	1.512(6)
O(2)	C(1)	1.437(5)	C(6)	C(7)	1.517(6)
O(2)	C(7)	1.445(5)	C(7)	C(15)	1.483(6)
O(3)	C(13)	1.191(6)	C(8)	C(9)	1.299(7)
O(4)	C(13)	1.328(6)	C(10)	C(11)	1.515(6)
O(4)	C(14)	1.449(5)	C(11)	C(12)	1.285(6)
C(1)	C(8)	1.511(6)	C(15)	C(16)	1.299(7)
C(1)	C(5)	1.530(6)	C(17)	C(22)	1.381(6)
C(1)	C(2)	1.533(6)	C(17)	C(18)	1.388(6)
C(2)	N(3)	1.489(5)	C(18)	C(19)	1.372(6)
C(2)	C(10)	1.515(6)	C(19)	C(20)	1.361(6)
N(3)	C(4)	1.354(5)	C(20)	C(21)	1.394(6)
N(3)	C(17)	1.424(5)	C(21)	C(22)	1.371(6)
C(4)	C(5)	1.495(6)			

Table S10. Bond Angles for **8b.**

Atom	Atom	Atom	Angle/°	Atom	Atom	Atom	Angle/°
C(1)	O(2)	C(7)	111.7(3)	C(7)	C(6)	C(5)	101.1(3)
C(13)	O(4)	C(14)	115.4(5)	O(2)	C(7)	C(15)	108.6(3)
O(2)	C(1)	C(8)	109.8(4)	O(2)	C(7)	C(6)	103.5(3)
O(2)	C(1)	C(5)	104.4(3)	C(15)	C(7)	C(6)	116.3(4)
C(8)	C(1)	C(5)	112.5(4)	C(9)	C(8)	C(1)	125.8(5)
O(2)	C(1)	C(2)	110.7(3)	C(2)	C(10)	C(11)	111.4(4)
C(8)	C(1)	C(2)	114.6(4)	C(12)	C(11)	C(10)	126.5(5)
C(5)	C(1)	C(2)	104.2(3)	O(3)	C(13)	O(4)	125.3(5)
N(3)	C(2)	C(10)	112.2(3)	O(3)	C(13)	C(6)	122.5(5)
N(3)	C(2)	C(1)	102.0(3)	O(4)	C(13)	C(6)	112.1(5)
C(10)	C(2)	C(1)	115.2(3)	C(16)	C(15)	C(7)	124.5(5)
C(4)	N(3)	C(17)	123.3(3)	C(22)	C(17)	C(18)	119.5(4)
C(4)	N(3)	C(2)	112.5(3)	C(22)	C(17)	N(3)	119.0(4)
C(17)	N(3)	C(2)	124.1(3)	C(18)	C(17)	N(3)	121.5(4)
O(1)	C(4)	N(3)	125.8(4)	C(19)	C(18)	C(17)	120.1(4)
O(1)	C(4)	C(5)	125.0(4)	C(20)	C(19)	C(18)	119.9(4)
N(3)	C(4)	C(5)	109.2(4)	C(19)	C(20)	C(21)	121.0(4)
C(4)	C(5)	C(1)	104.2(3)	C(19)	C(20)	Br(1)	121.1(4)
C(4)	C(5)	C(6)	113.5(4)	C(21)	C(20)	Br(1)	117.9(3)
C(1)	C(5)	C(6)	102.4(3)	C(22)	C(21)	C(20)	118.9(4)
C(13)	C(6)	C(7)	118.4(4)	C(21)	C(22)	C(17)	120.6(4)
C(13)	C(6)	C(5)	112.8(3)				

Table S11. Hydrogen Bonds for **8b.**

D	H	A	d(D-H)/Å	d(H-A)/Å	d(D-A)/Å	D-H-A/°
C(18)	H(18A)	O(1)[1]	0.93	2.48	3.410(5)	176.1
C(19)	H(19A)	O(3)[1]	0.93	2.54	3.313(6)	140.4
C(21)	H(21A)	O(2)[2]	0.93	2.56	3.408(5)	152.2

[1]3/2-X,1/2+Y,1/2-Z; [2]1/2-X,-1/2+Y,1/2-Z

12. *NMR spectrum data*

Table S12. Selected ^{1}H NMR chemical shifts for *trans*- and *cis*-isomers.

**Compound**	*δ* (^{1}H) ***trans / cis***			
	CH_2 (All, aliph.), 2×dd or m (2×ddd or m for **6e**) **δ (*tr*) > δ (*cis*)**	H(7a), d **δ (*tr*) > δ (*cis*)**	H(3), dd **δ (*tr*) < δ (*cis*)**	H(4), d **δ (*tr*) > δ (*cis*)**
**5k**	2.58+2.40 / 2.21	3.20 / 2.95	4.72 / 5.00	6.59 / 6.54
**5m**	2.58 / 2.40+2.19	3.02 / 2.86	3.75 / 4.23	6.58 / 6.47
**5p**	2.59+2.41 / 2.22	3.24 / 2.97	4.82 / 5.05	6.59 / 6.54
**6e**	2.62 / 2.36+2.2	3.17 / 3.05	4.76 / 4.88	6.76 / 6.58
**6k**	2.58+2.39 / 2.19	3.28 / 3.00	4.73 / 5.00	6.60 / 6.55
**6m**	2.54 / 2.40+2.18	3.09 / 2.92	3.75 / 4.26	6.59 / 6.48
**6p**	2.60+2.42 / 2.21	3.32 / 3.03	4.83 / 5.07	6.61 / 6.55

Table S13. Selected ^{13}C NMR chemical shifts for *trans*- and *cis*-isomers.

**Compound**	δ (^{13}C) ***trans / cis***		
	CH_2 (All, aliph.) **δ (*tr*) > δ (*cis*)**	CH(3) **δ (*tr*) > δ (*cis*)** (except **5m,6m**)	C(3a) **δ (*tr*) > δ (*cis*)**
**5k**	37.5 / 35.3	58.3 / 57.2	91.2 / 90.3
**5m**	37.9 / 35.6	56.0 / 56.0	92.1 / 90.8
**5p**	37.6 / 35.2	58.0 / 57.0	91.1 / 90.3
**6e**	33.7 / 32.6	59.5 / 58.0	91.1 / 90.4
**6k**	37.6 / 35.3	58.3 / 57.3	91.4 / 90.5
**6m**	37.9 / 35.5	56.0 / 56.0	92.2 / 91.0
**6p**	37.7 / 35.2	58.1 / 57.1	91.3 / 90.4

13. *References*

1. Kouznetsov, V.; Öcal, N.; Turgut, Z.; Zubkov, F.; Kaban,S.; Varlamov, A. Allylation and Heterocycloaddition reactions of Aldimines: Furan- and Quinolinecarboxaldhydes. *Monatsh. Chem*. **1998**, *129*, 671-677.
2. Urbina, J.M.; Cortés, J.C.; Palma, A.; López, S.N.; Zacchino, S.A.; Enriz, R.D.; Ribas, C.; Kouznetsov, V.V. Inhibitors of the fungal cell wall. Synthesis of 4-aryl-4-N-arylamine-1-butenes and related compounds with inhibitory activities on β(1–3) glucan and chitin synthases. *Bioorg. Med. Chem*. **2000**, *8*, 691–698.
3. Zubkov, F.I.; Boltukhina, E.V.; Turchin, K.F.; Borisov, R.S.; Varlamov, A.V. New synthetic approach to substituted isoindolo[2,1-*a*]quinoline carboxylic acids *via* intramolecular Diels–Alder reaction of 4-(N-furyl-2)-4-arylaminobutenes-1 with maleic anhydride. *Tetrahedron* **2005**, *61*, 4099–4113.
4. Boltukhina, E.V.; Zubkov, F.I.; Nikitina, E.V.; Varlamov, A.V. Novel approach to isoindolo[2,1-*a*]quinolines: synthesis of 1- and 3-halo-substituted 11-oxo-5,6,6a,11-tetrahydroisoindolo[2,1-*a*]quinoline-10-carboxylic acids. *Synthesis* **2005**, 1859–1875.
5. Zubkov, F.I.; Boltukhina, E.V.; Nikitina, E.V.; Varlamov, A.V. Study of regioselectivity of intramolecular cyclization of N-(*m*-R-phenyl)- and N-(α-naphthyl)-2-allyl(methallyl)-6-carboxy-4-oxo-3-aza-10-oxatricyclo[5.2.1.0^{1,5}]dec-8-enes. *Russ. Chem. Bull*. **2004**, *53*, 2816–2829.

6. Varlamov, A.V.; Boltukhina, E.V.; Zubkov, F.I.; Nikitina, E.V.; Turchin K.F. Intramolecular [4+2] cycloaddition of furfurylsubstituted homoallylamines to allylhalides, acryloyl chloride and maleic anhydride. *J. Heterocyclic Chem.* **2006**, *43*, 1479–1495.
7. SAINT-Plus, Version 7.68., **2007**, Bruker AXS Inc.: Madison, Wisconsin, USA.
8. SADABS; **2008**, Bruker AXS Inc.: Madison, Wisconsin, USA.
9. Sheldrick, G.M. Crystal structure refinement with SHELXL. *Acta Crystallogr. C* **2015**, *71*, 3–8.
10. Dolomanov, O.V.; Bourhis, L.J.; Gildea, R.J.; Howard, J.A.K.; Puschmann, H. OLEX2: A Complete Structure Solution, Refinement and Analysis Program *J. Appl. Cryst*. **2009**, *42*, 339–341.

Publisher's Note: MDPI stays neutral with regard to jurisdictional claims in published maps and institutional affiliations.

© 2020 by the authors. Submitted for possible open access publication under the terms and conditions of the Creative Commons Attribution (CC BY) license (http://creativecommons.org/licenses/by/4.0/).
