# Supplementary material for: Application of New Efficient Hoveyda–Grubbs Catalysts Comprising an N→Ru Coordinate Bond in a Six-Membered Ring for the Synthesis of Natural Product-Like Cyclopenta[b]furo[2,3-c]pyrroles
Source: Molecules. 2020 Nov 17;25(22):5379. doi: 10.3390/molecules25225379 (PMC7709010; doi:10.3390/molecules25225379)

NMR spectrum data for compound 5a

Acquisition Time (sec)	1.9818	***Comment***	single_pulse	***Date***	23 May 1990 04:15:55		
Date Stamp	02 Aug 2019 10:19:37			***File Name***	C:\Users\Fedor\Desktop\01.08.19\FZ7627-1.jdf	***Frequency (MHz)***	600.17
Nucleus	1H	***Number of Transients***	8	***Origin***	ECA 600	***Original Points Count***	32768
Owner	delta						
Points Count	32768	***Pulse Sequence***	single_pulse.ex2	***Receiver Gain***	38.00	***Solvent***	CHLOROFORM-d
Spectrum Offset (Hz)	5410.6274	***Sweep Width (Hz)***	16534.39	***Temperature (degree C)***	20.700		

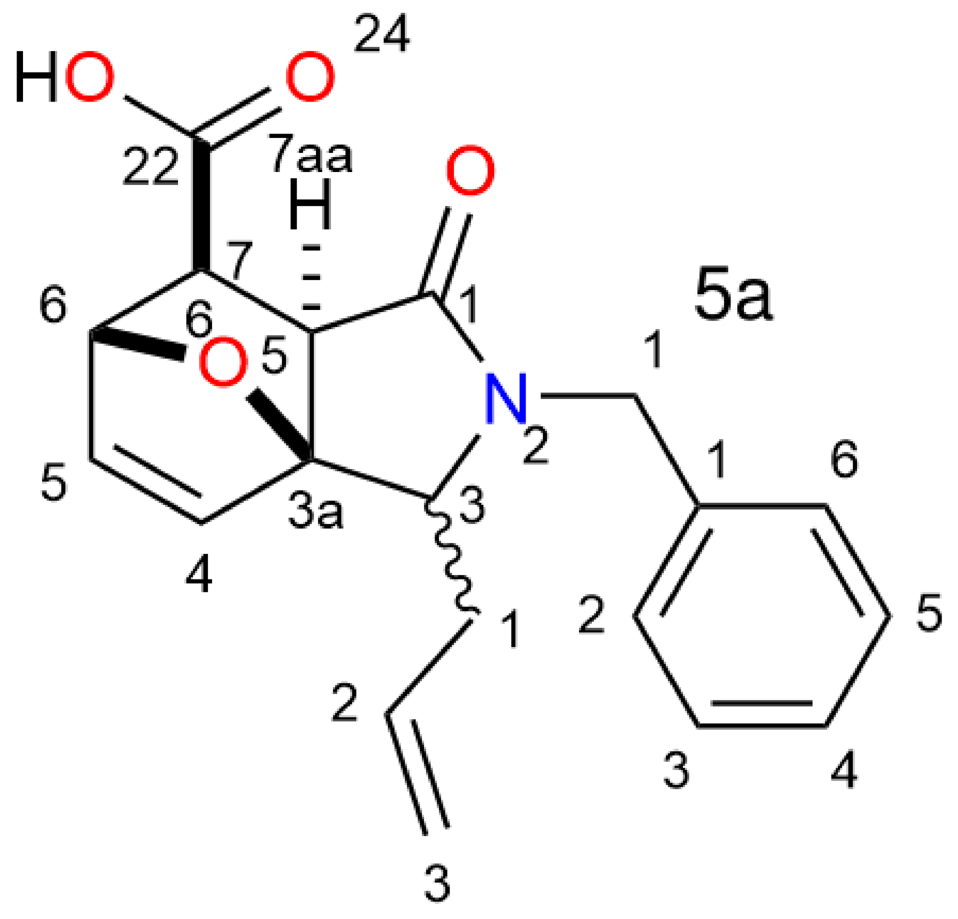

FZ7627-1.jdf

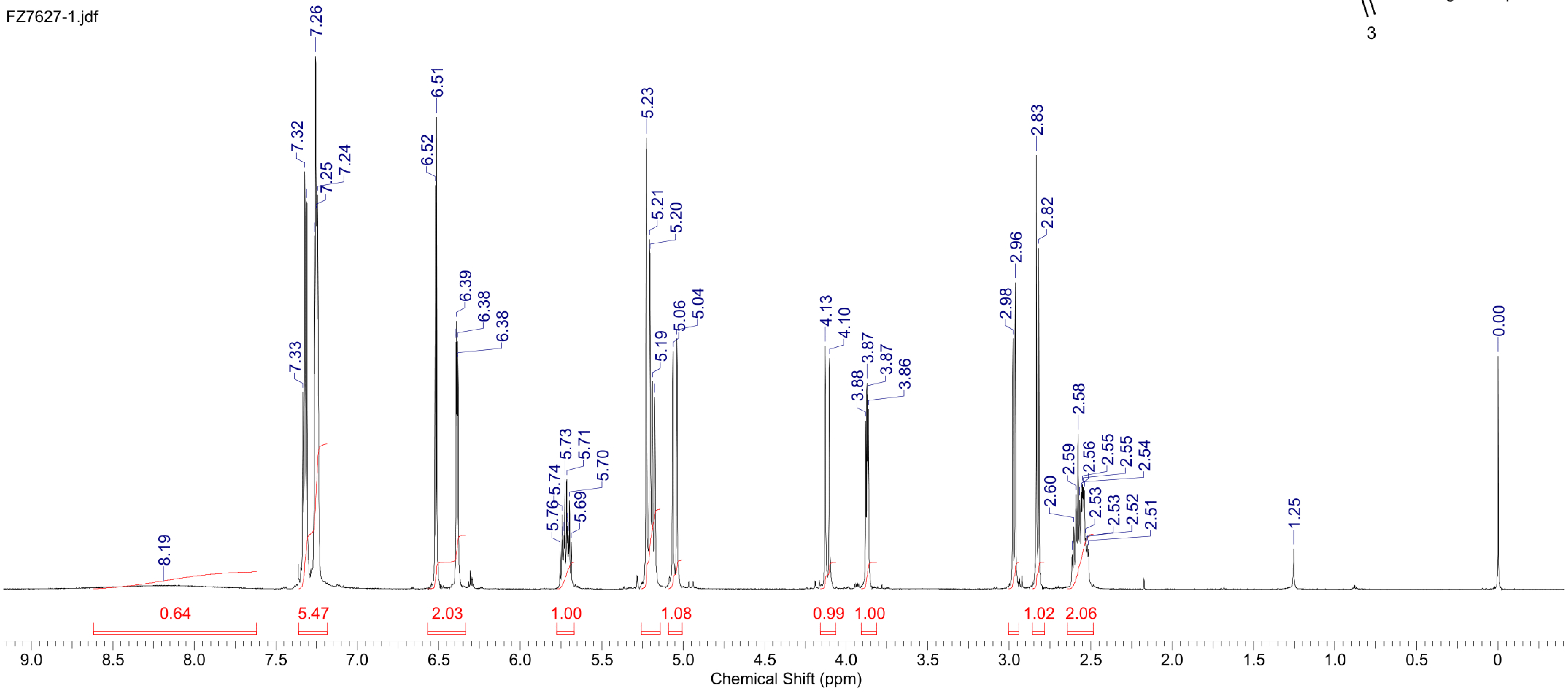

Acquisition Time (sec)	1.9818	***Comment***	single_pulse	***Date***	23 May 1990 04:15:55				
Date Stamp	02 Aug 2019 10:19:37			***File Name***	C:\Users\Fedor\Desktop\01.08.19\FZ7627-1.jdf			***Frequency (MHz)***	600.17
Nucleus	1H	***Number of Transients***	8	***Origin***	ECA 600	***Original Points Count***	32768	***Owner***	delta
Points Count	32768	***Pulse Sequence***	single_pulse.ex2			***Receiver Gain***	38.00	***Solvent***	CHLOROFORM-d
Spectrum Offset (Hz)	5410.6274	***Sweep Width (Hz)***	16534.39	***Temperature (degree C)***	20.700				

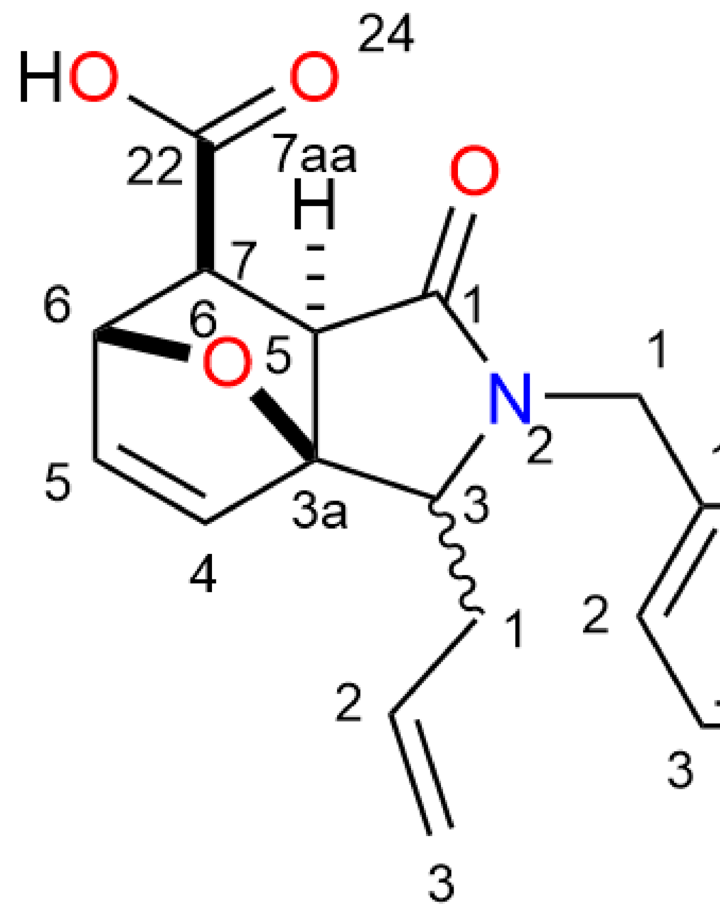

5a

FZ7627-1.jdf

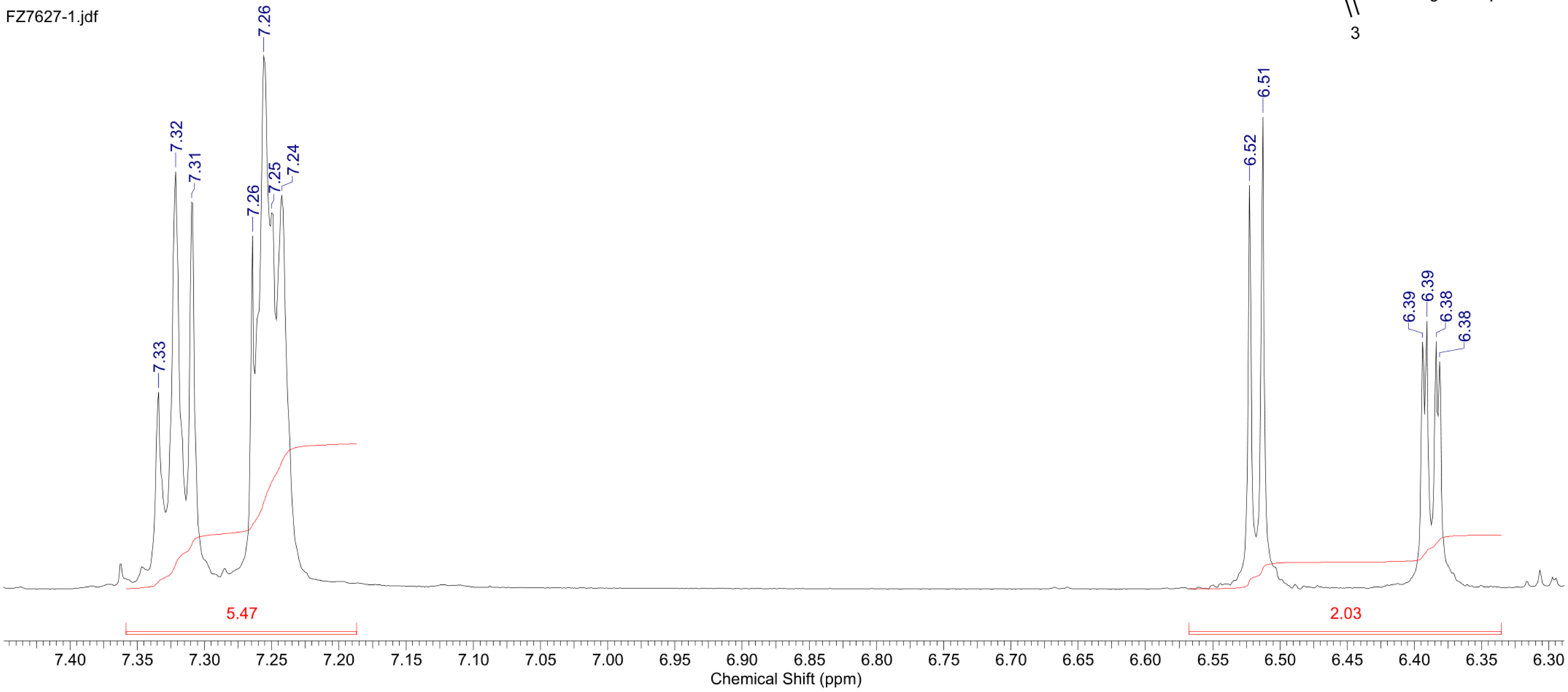

Acquisition Time (sec)	1.9818	***Comment***	single_pulse	***Date***	23 May 1990 04:15:55		
Date Stamp	02 Aug 2019 10:19:37			***File Name***	C:\Users\Fedor\Desktop\01.08.19\FZ7627-1.jdf	***Frequency (MHz)***	600.17
Nucleus	1H	***Number of Transients***	8	***Origin***	ECA 600	***Original Points Count***	32768
						Owner	delta
Points Count	32768	***Pulse Sequence***	single_pulse.ex2	***Receiver Gain***	38.00	***Solvent***	CHLOROFORM-d
Spectrum Offset (Hz)	5410.6274	***Sweep Width (Hz)***	16534.39	***Temperature (degree C)***	20.700		

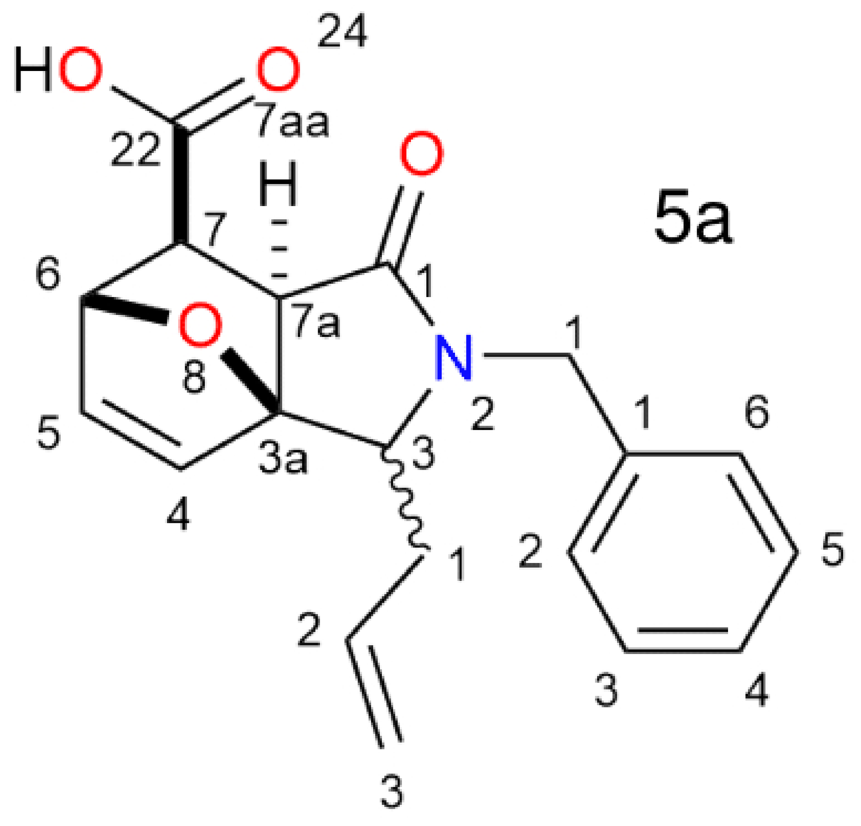

5a

FZ7627-1.jdf

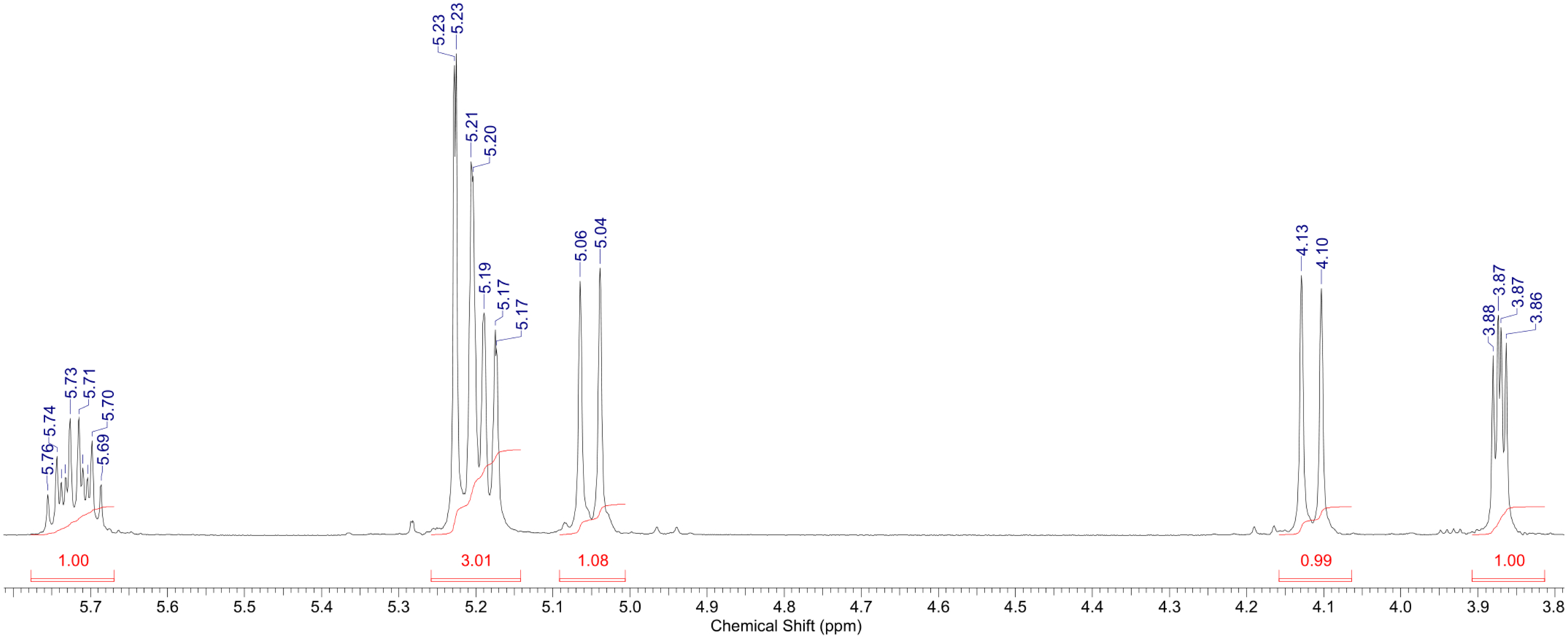

Acquisition Time (sec)	1.9818	***Comment***	single_pulse	***Date***	23 May 1990 04:15:55		
Date Stamp	02 Aug 2019 10:19:37			***File Name***	C:\Users\Fedor\Desktop\01.08.19\FZ7627-1.jdf	***Frequency (MHz)***	600.17
Nucleus	1H	***Number of Transients***	8	***Origin***	ECA 600	***Original Points Count*** 32768	***Owner*** delta
Points Count	32768	***Pulse Sequence***	single_pulse.ex2			***Receiver Gain*** 38.00	***Solvent*** CHLOROFORM-d
Spectrum Offset (Hz)	5410.6274	***Sweep Width (Hz)***	16534.39	***Temperature (degree C)***	20.700		

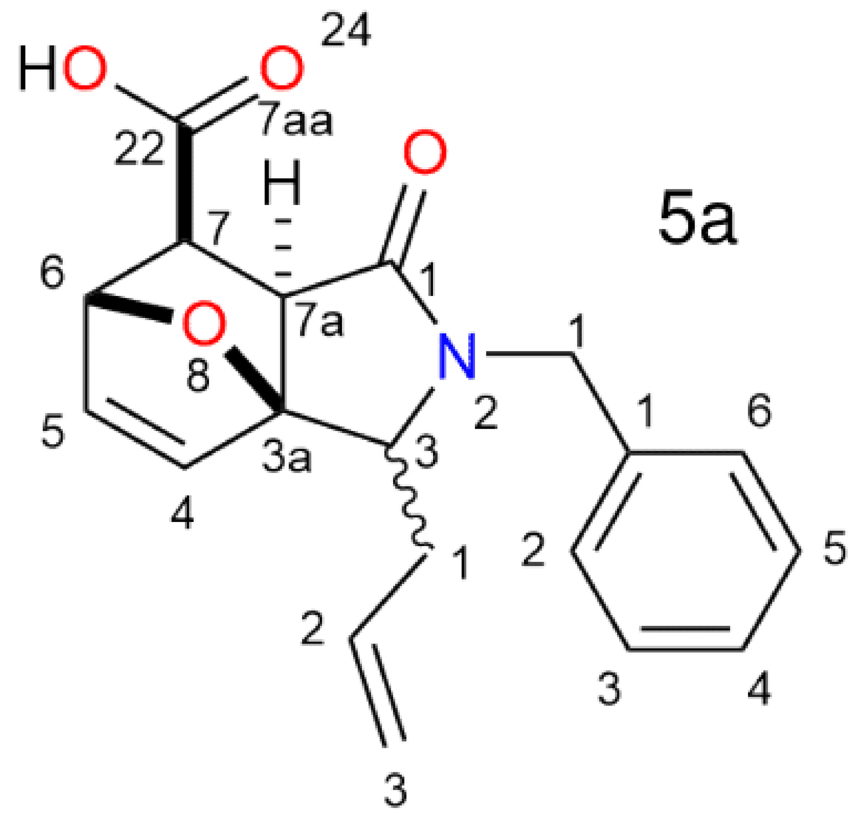

5a

FZ7627-1.jdf

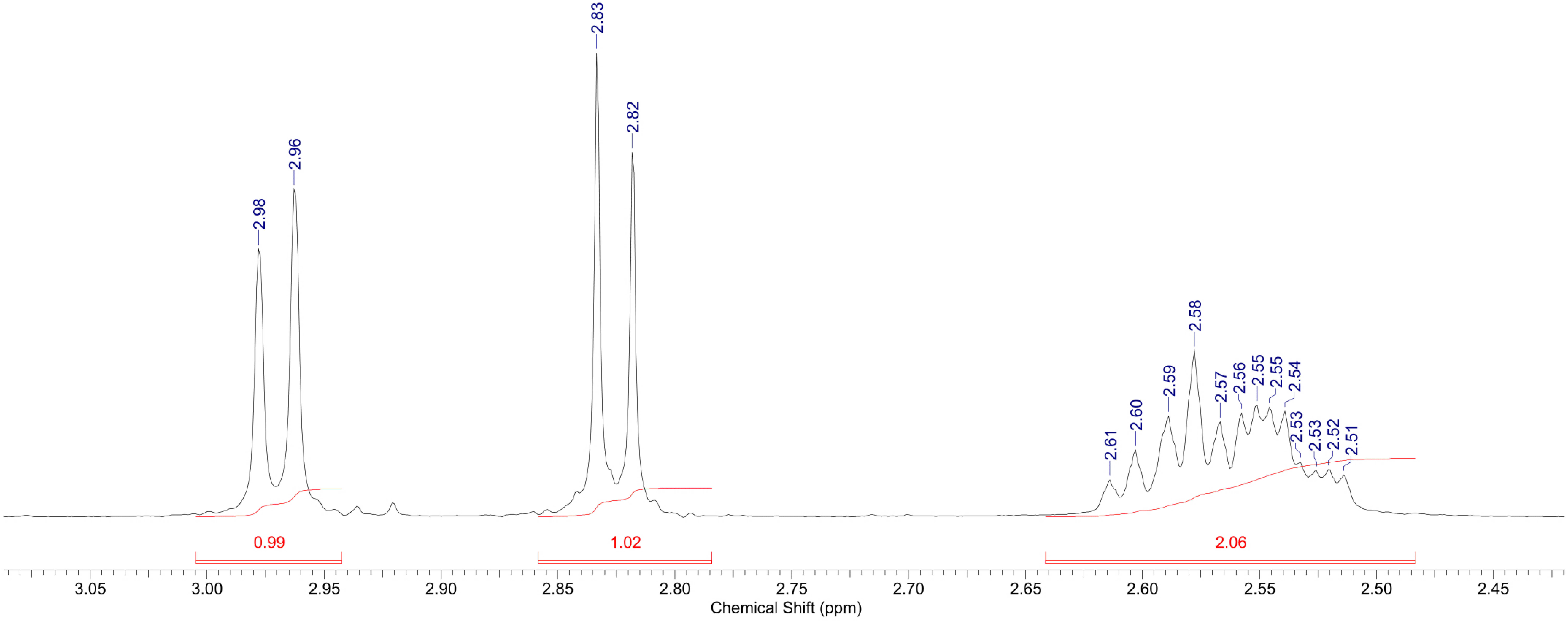

Acquisition Time (sec)	0.6921	*Comment*	single pulse decoupled gated NOE			*Date*	08 May 1990 07:03:01
Date Stamp	18 Jul 2019 13:06:40			*File Name*	C:\Users\Fedor\Desktop\18.07.19\FZ7507_13C-1.jdf		
Frequency (MHz)	150.91	*Nucleus*	13C	*Number of Transients*	800	*Origin*	ECA 600
Original Points Count	32768	*Owner*	delta	*Points Count*	32768	*Pulse Sequence*	single_pulse_dec
Receiver Gain	58.00	*Solvent*	CHLOROFORM-d			*Spectrum Offset (Hz)*	15072.1279
Sweep Width (Hz)	47348.49	*Temperature (degree C)*	23.900				

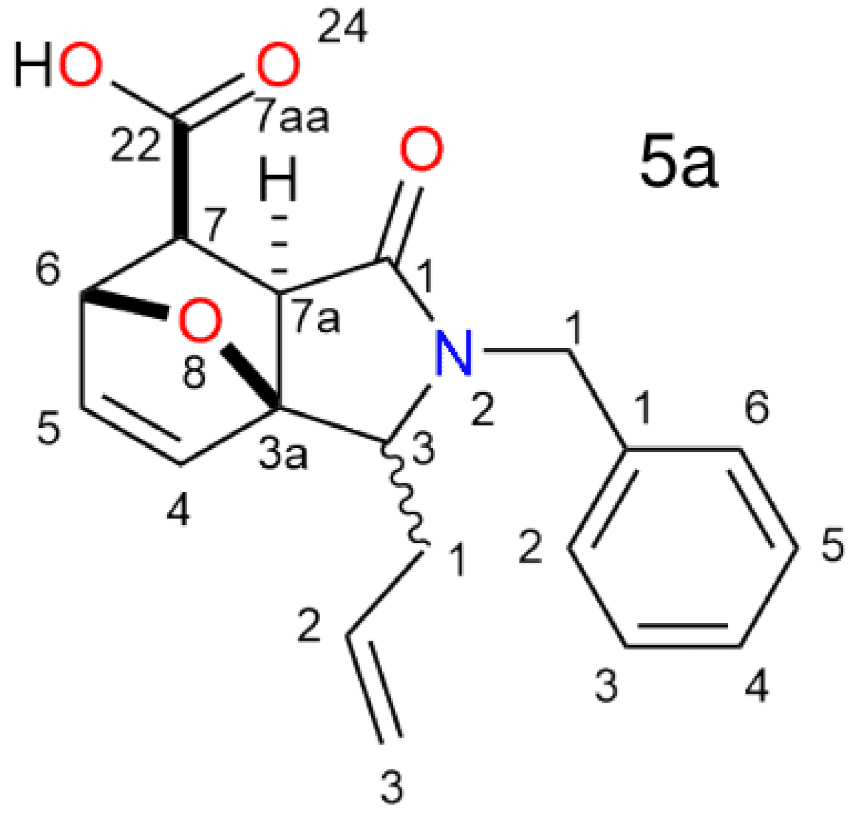

5a

FZ7507_13C-1.jdf

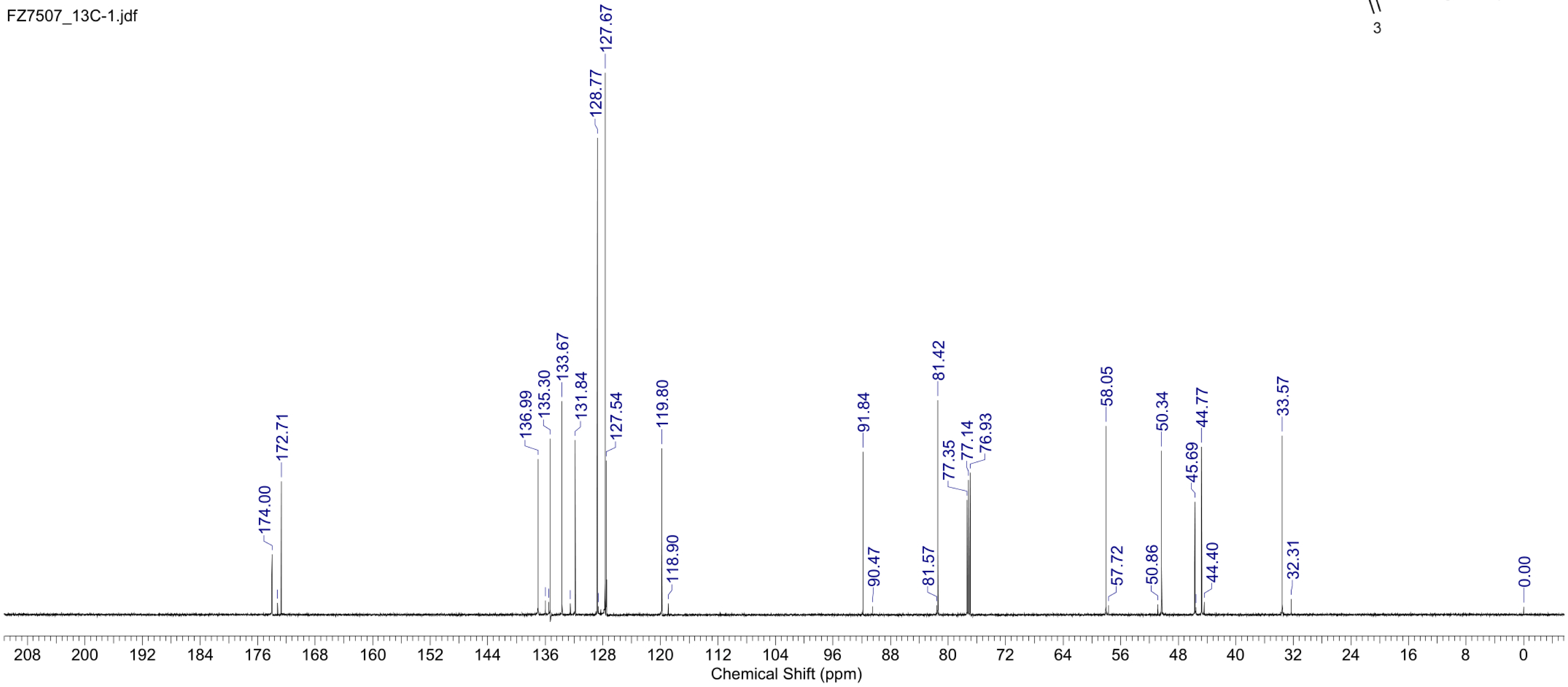

Acquisition Time (sec)	0.6921	***Comment***	single pulse decoupled gated NOE			***Date***	08 May 1990 07:03:01
Date Stamp	18 Jul 2019 13:06:40			***File Name***	C:\Users\Fedor\Desktop\18.07.19\FZ7507_13C-1.jdf		
Frequency (MHz)	150.91	***Nucleus***	13C	***Number of Transients***	800	***Origin***	ECA 600
Original Points Count	32768	***Owner***	delta	***Points Count***	32768	***Pulse Sequence***	single_pulse_dec
Receiver Gain	58.00	***Solvent***	CHLOROFORM-d			***Spectrum Offset (Hz)***	15072.1279
Sweep Width (Hz)	47348.49	***Temperature (degree C)***	23.900				

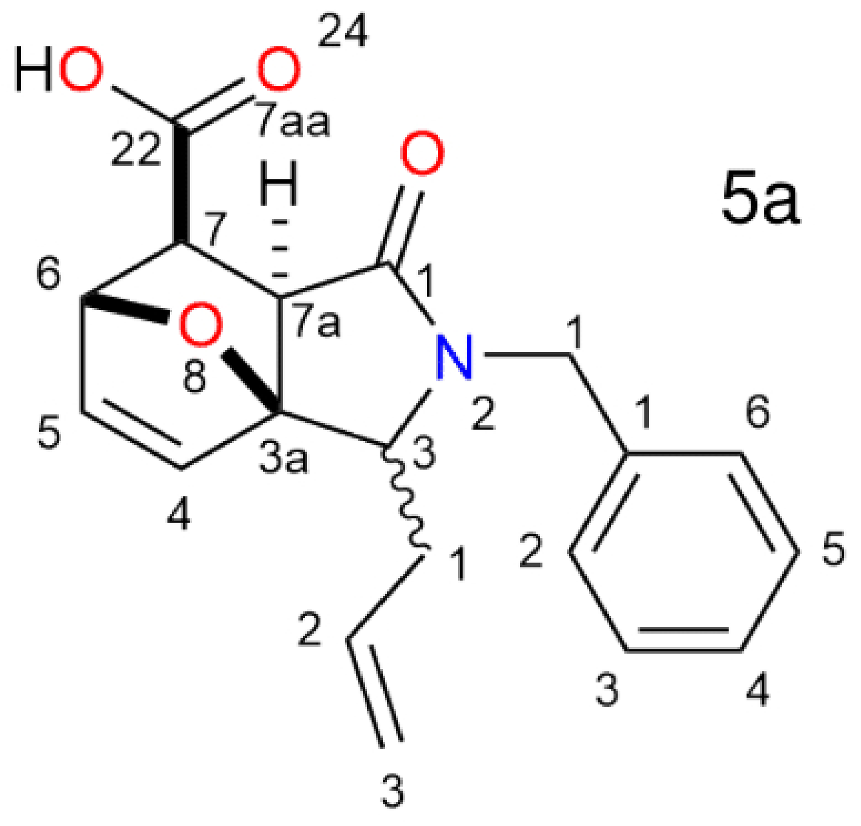

5a

FZ7507_13C-1.jdf

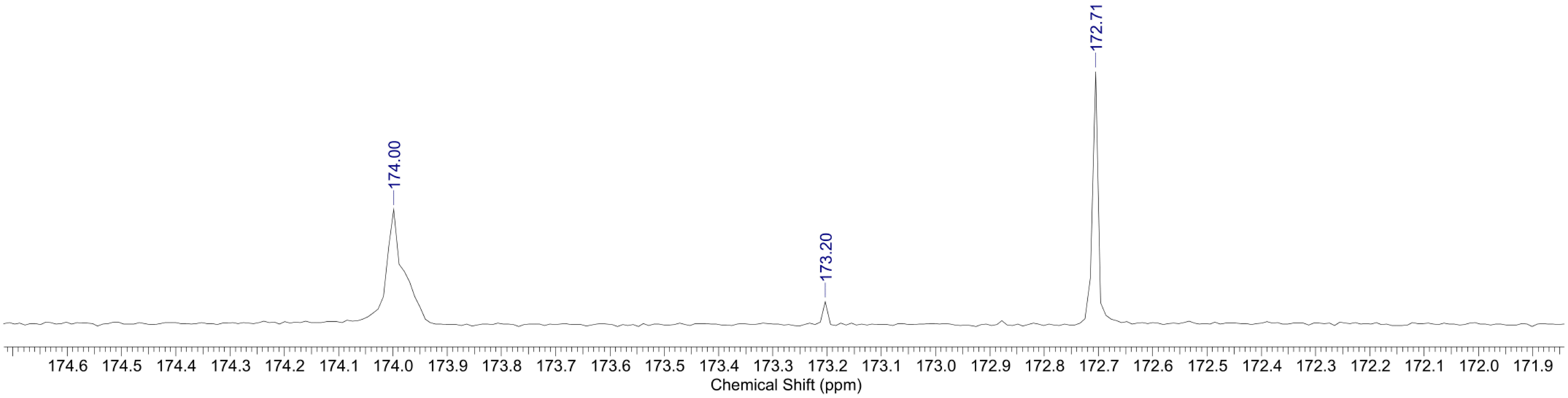

Acquisition Time (sec)	0.6921	***Comment***	single pulse decoupled gated NOE			***Date***	08 May 1990 07:03:01
Date Stamp	18 Jul 2019 13:06:40			***File Name***	C:\Users\Fedor\Desktop\18.07.19\FZ7507_13C-1.jdf		
Frequency (MHz)	150.91	***Nucleus***	13C	***Number of Transients***	800	***Origin***	ECA 600
Original Points Count	32768	***Owner***	delta	***Points Count***	32768	***Pulse Sequence***	single_pulse_dec
Receiver Gain	58.00	***Solvent***	CHLOROFORM-d			***Spectrum Offset (Hz)***	15072.1279
Sweep Width (Hz)	47348.49	***Temperature (degree C)***	23.900				

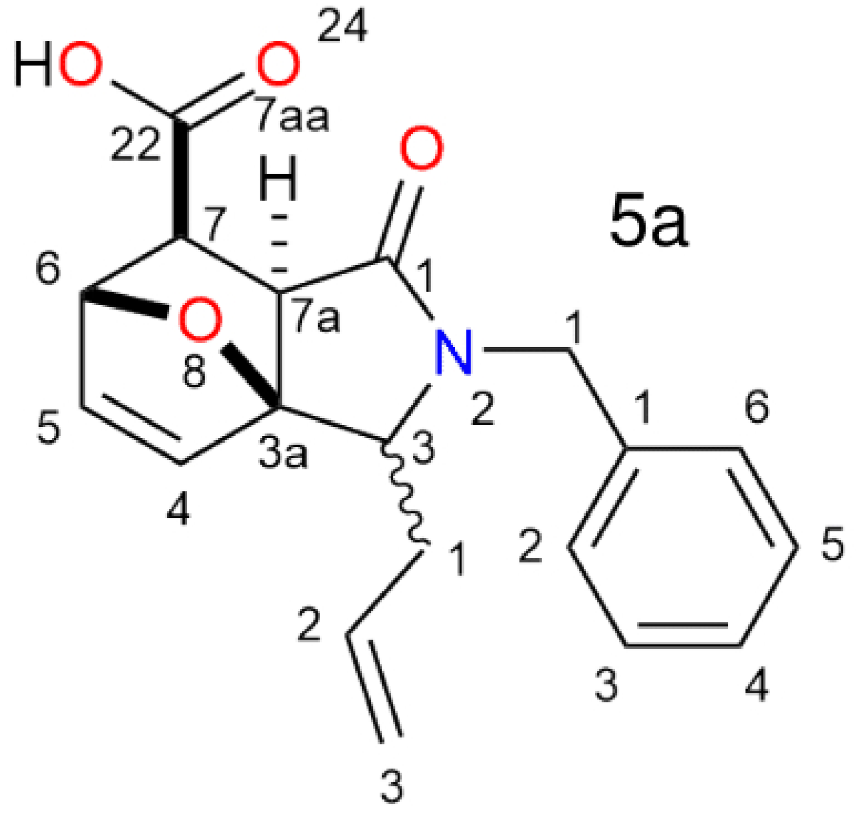

5a

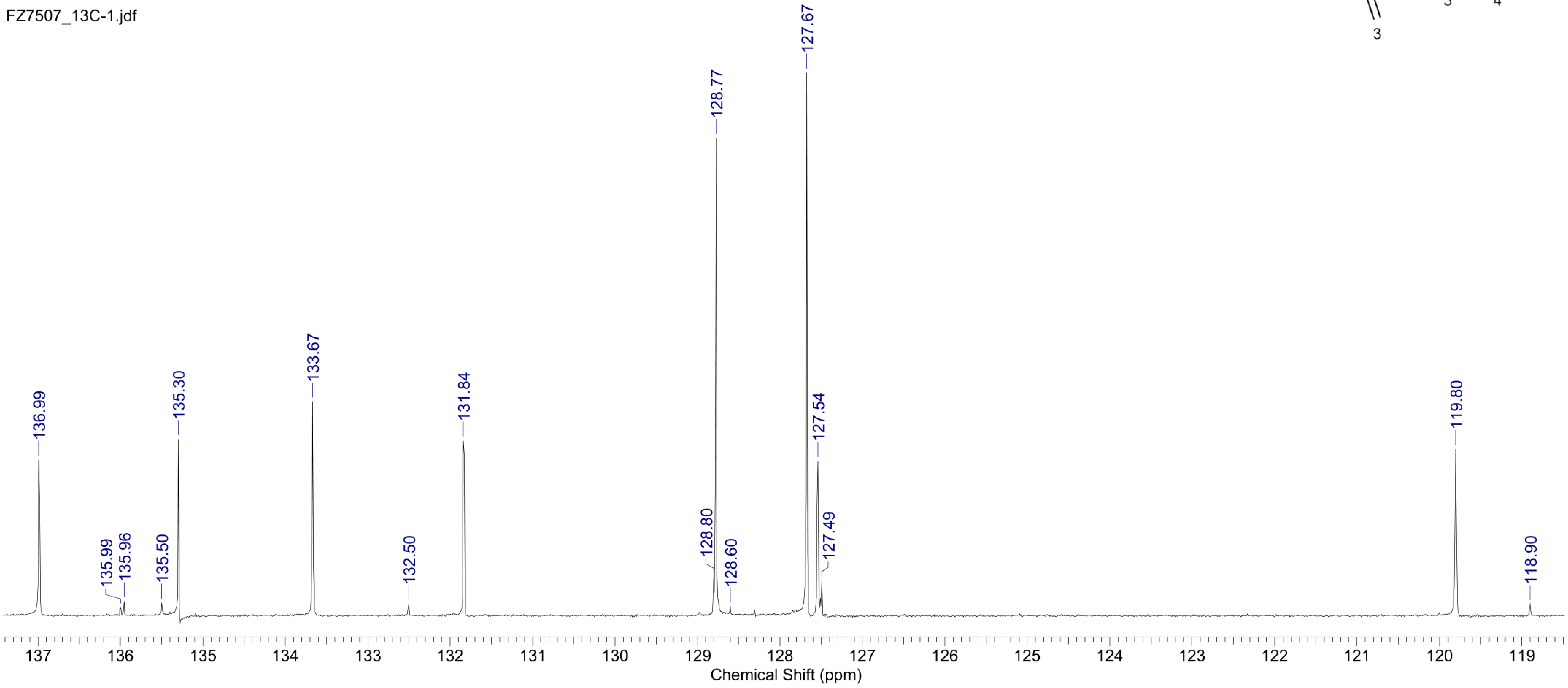

Acquisition Time (sec)	0.6921	***Comment***	single pulse decoupled gated NOE			***Date***	08 May 1990 07:03:01
Date Stamp	18 Jul 2019 13:06:40			***File Name***	C:\Users\Fedor\Desktop\18.07.19\FZ7507_13C-1.jdf		
Frequency (MHz)	150.91	***Nucleus***	13C	***Number of Transients***	800	***Origin***	ECA 600
Original Points Count	32768	***Owner***	delta	***Points Count***	32768	***Pulse Sequence***	single_pulse_dec
Receiver Gain	58.00	***Solvent***	CHLOROFORM-d			***Spectrum Offset (Hz)***	15072.1279
Sweep Width (Hz)	47348.49	***Temperature (degree C)***	23.900				

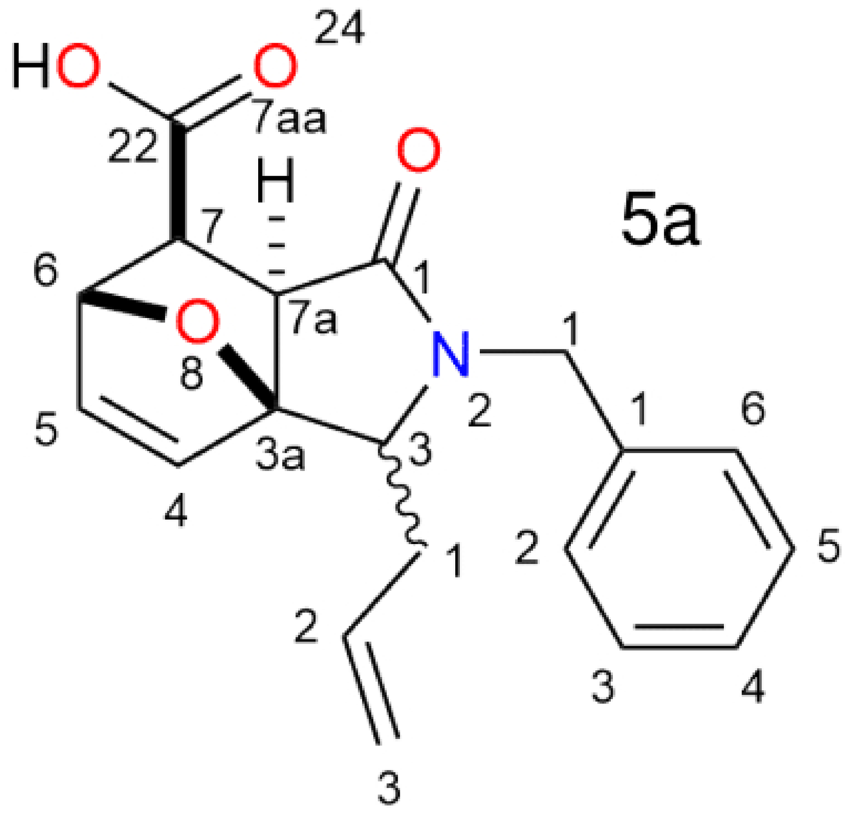

FZ7507_13C-1.jdf

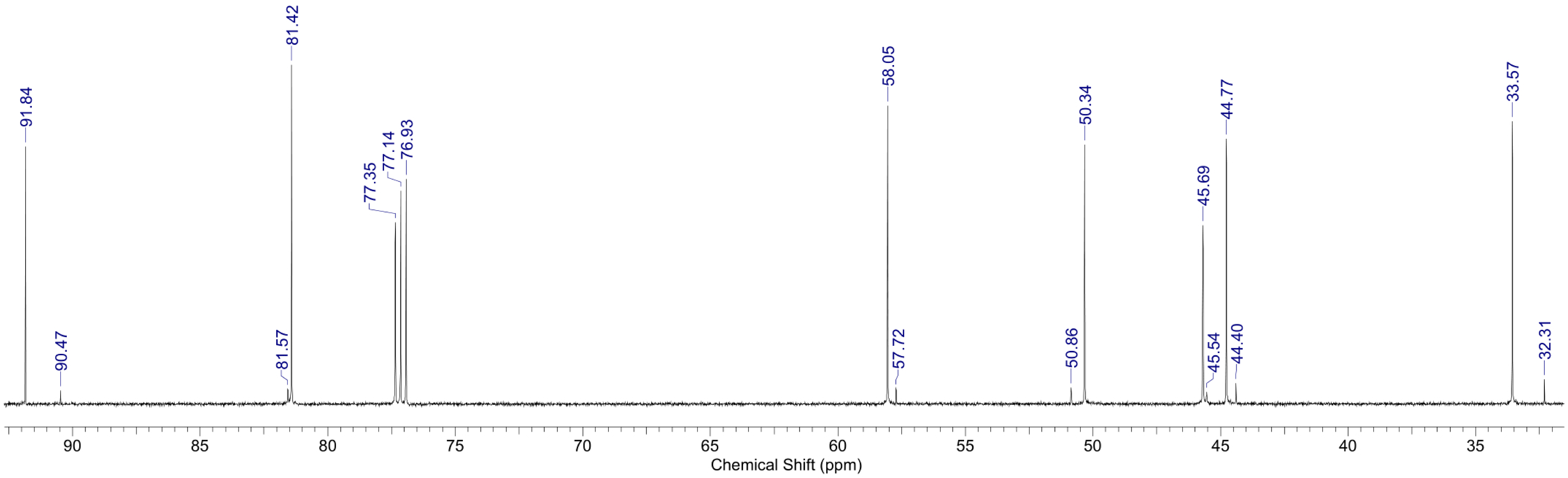

NMR spectrum data for compound 5f

**Acquisition Time (sec)**	1.9818	**Comment**	single_pulse	**Date**	07 May 1990 04:04:33		
**Date Stamp**	17 Jul 2019 10:08:12			**File Name**	C:\Users\Fedor\Desktop\15.07.19\FZ7543-1.jdf	**Frequency (MHz)**	600.17
**Nucleus**	1H	**Number of Transients**	8	**Origin**	ECA 600	**Original Points Count** 32768	**Owner** delta
**Points Count**	32768	**Pulse Sequence**	single_pulse.ex2			**Receiver Gain** 48.00	**Solvent** CHLOROFORM-d
**Spectrum Offset (Hz)**	5409.6182	**Sweep Width (Hz)**	16534.39	**Temperature (degree C)**	22.200		

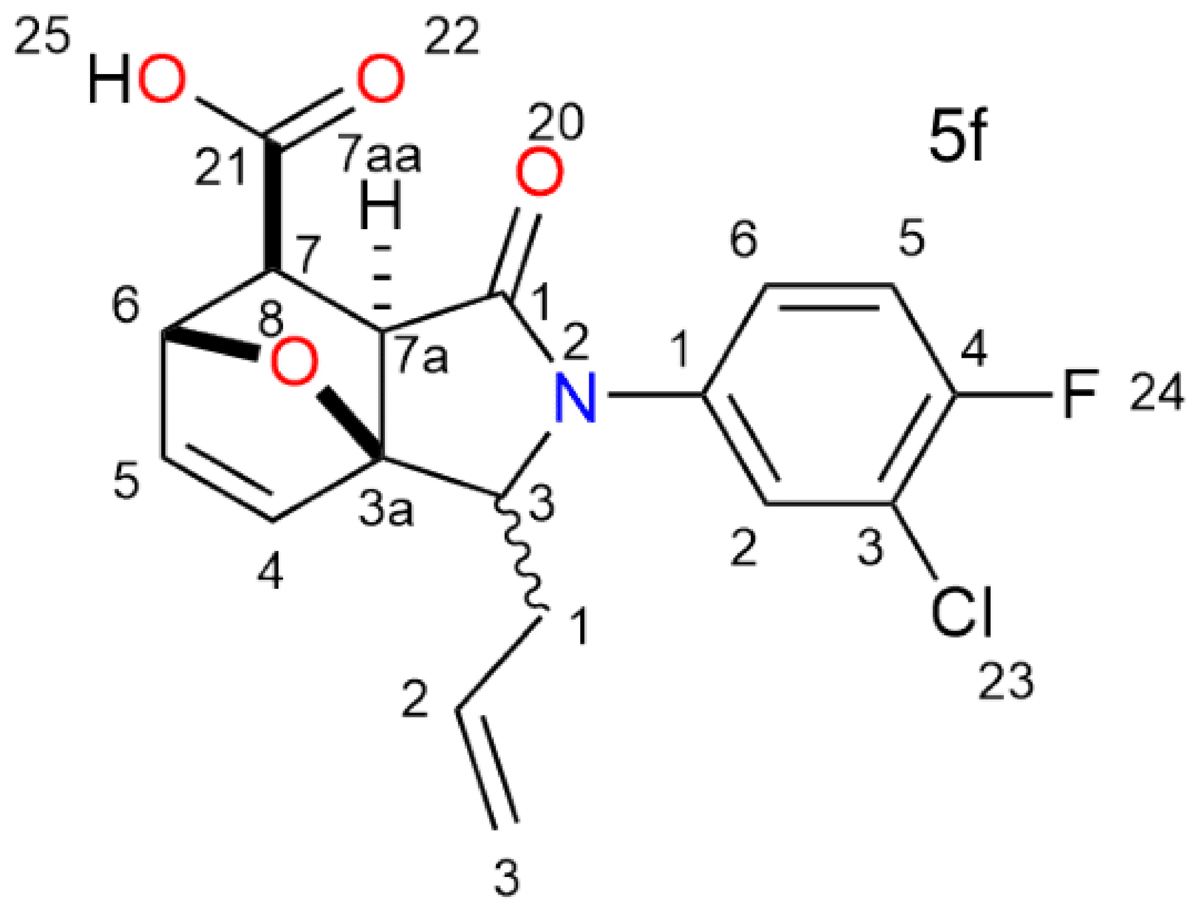

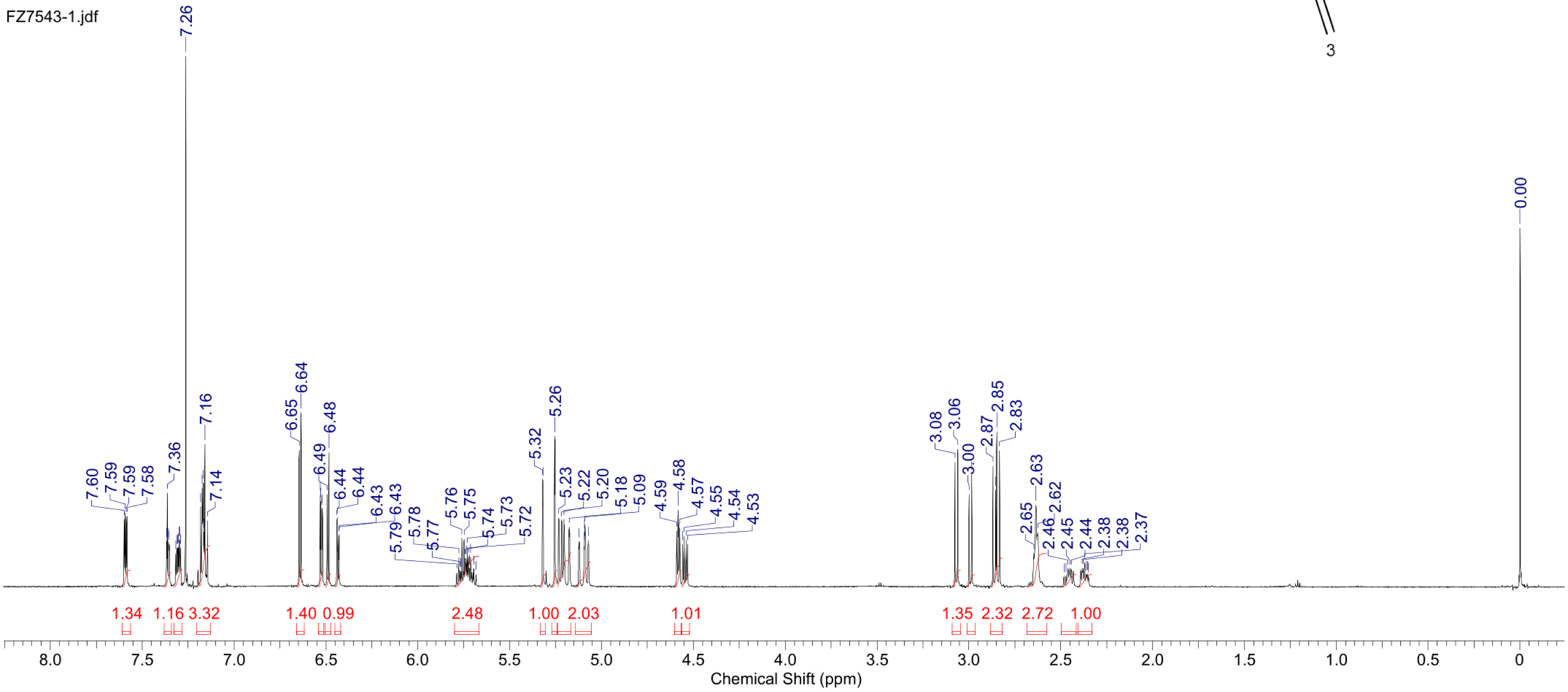

Acquisition Time (sec)	1.9818	***Comment***	single_pulse	***Date***	07 May 1990 04:04:33		
Date Stamp	17 Jul 2019 10:08:12			***File Name***	C:\Users\Fedor\Desktop\15.07.19\FZ7543-1.jdf	***Frequency (MHz)***	600.17
Nucleus	1H	***Number of Transients***	8	***Origin***	ECA 600	***Original Points Count*** 32768	***Owner*** delta
Points Count	32768	***Pulse Sequence***	single_pulse.ex2			***Receiver Gain*** 48.00	***Solvent*** CHLOROFORM-d
Spectrum Offset (Hz)	5409.6182	***Sweep Width (Hz)***	16534.39	***Temperature (degree C)***	22.200		

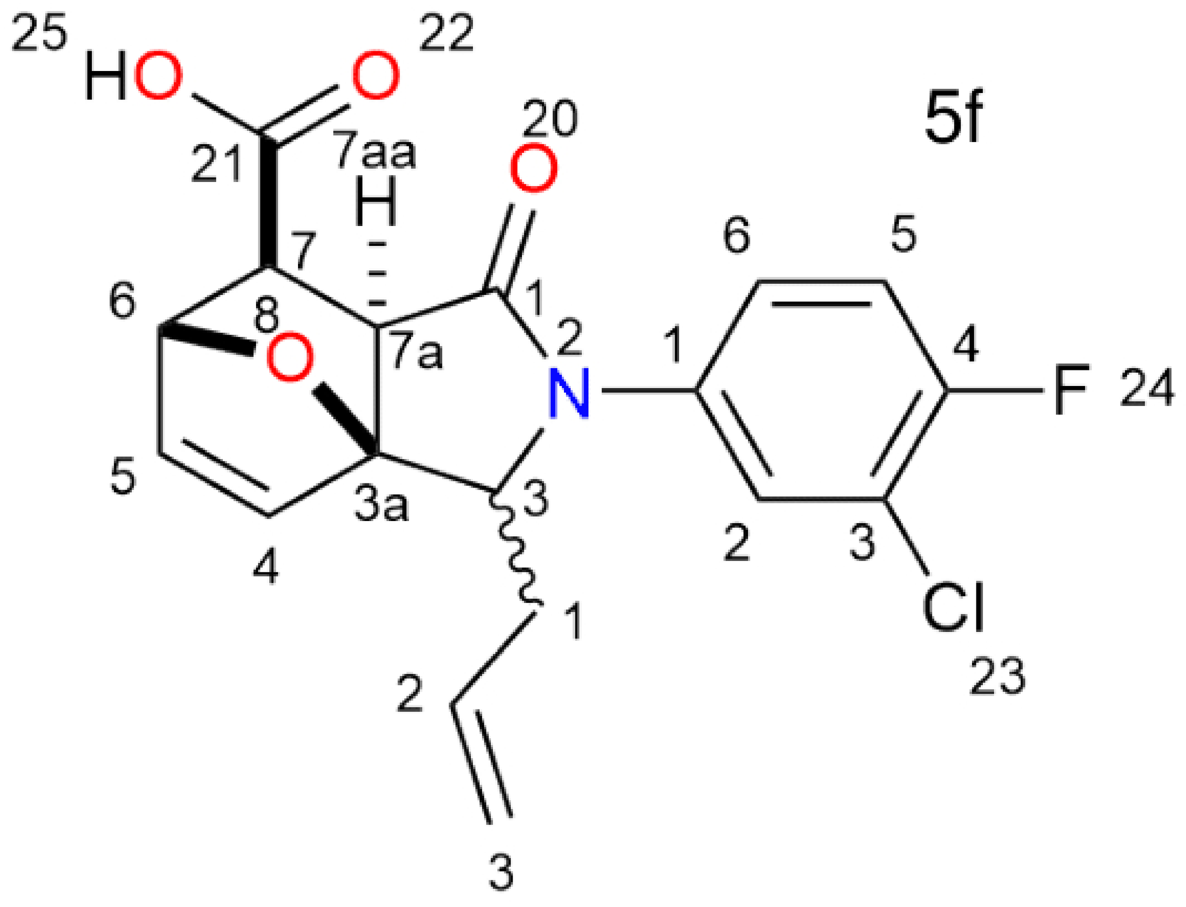

5f

FZ7543-1.jdf

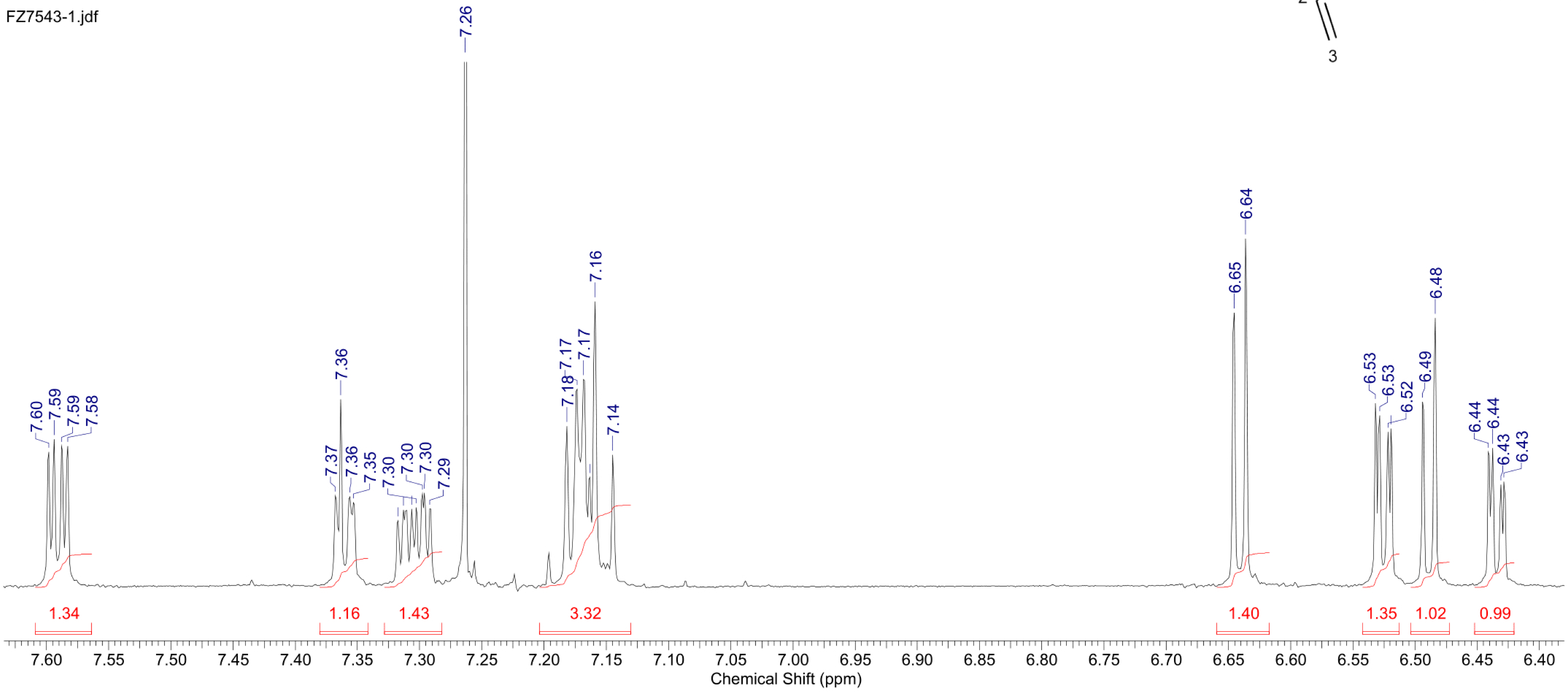

Acquisition Time (sec)	1.9818	***Comment***	single_pulse	***Date***	07 May 1990 04:04:33				
Date Stamp	17 Jul 2019 10:08:12			***File Name***	C:\Users\Fedor\Desktop\15.07.19\FZ7543-1.jdf			***Frequency (MHz)***	600.17
Nucleus	1H	***Number of Transients***	8	***Origin***	ECA 600	***Original Points Count***	32768	***Owner***	delta
Points Count	32768	***Pulse Sequence***	single_pulse.ex2			***Receiver Gain***	48.00	***Solvent***	CHLOROFORM-d
Spectrum Offset (Hz)	5409.6182	***Sweep Width (Hz)***	16534.39	***Temperature (degree C)***	22.200				

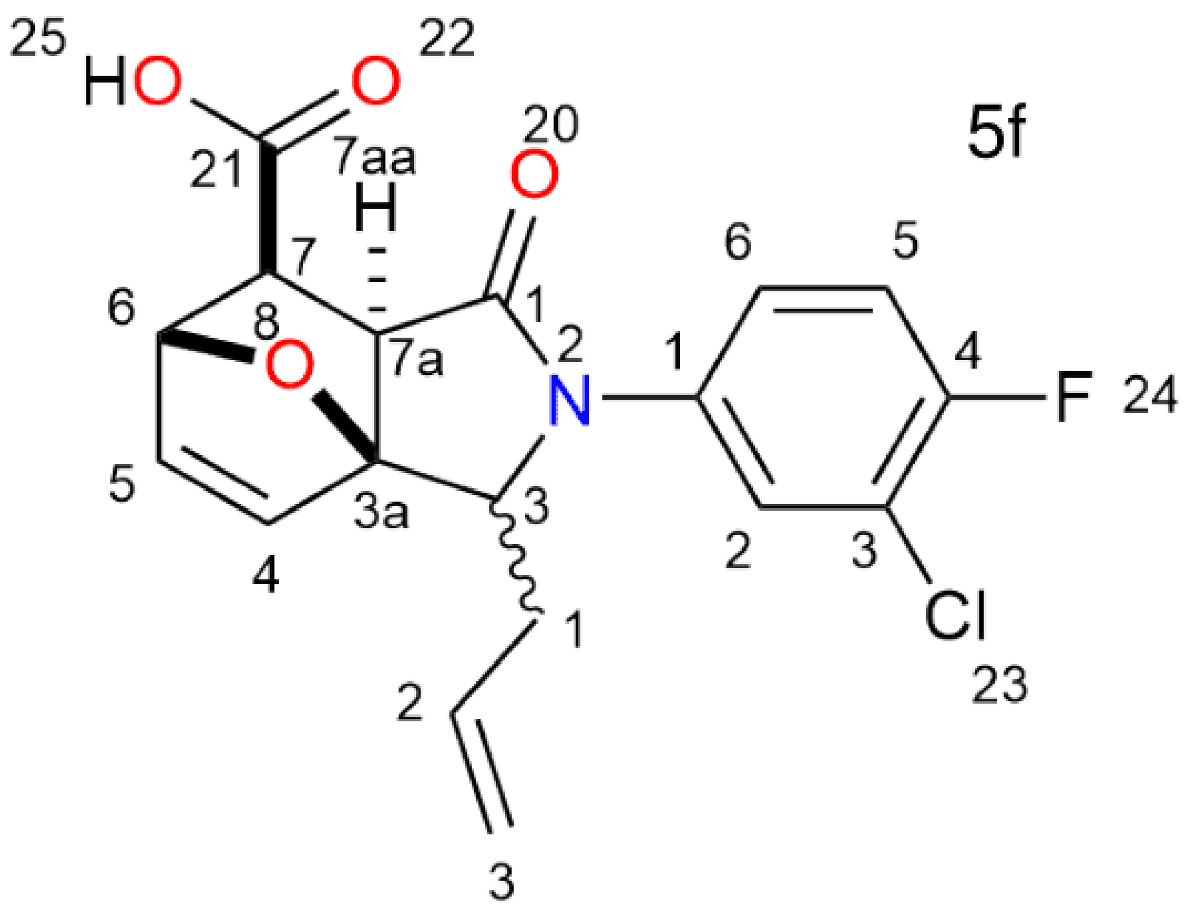

5f

FZ7543-1.jdf

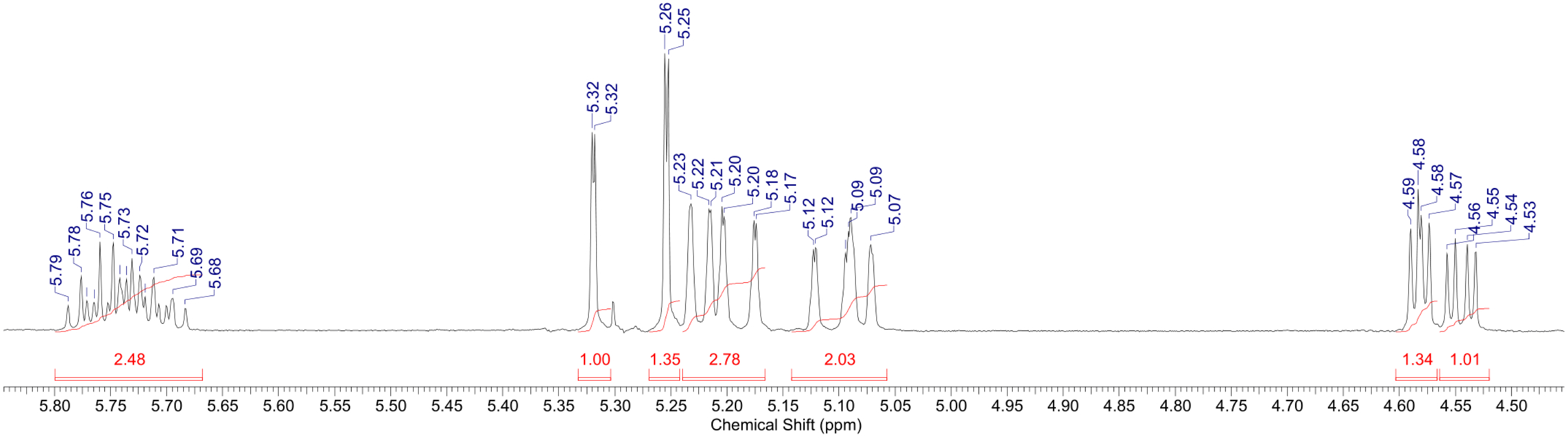

**Acquisition Time (sec)**	1.9818	**Comment**	single_pulse	**Date**	07 May 1990 04:04:33		
**Date Stamp**	17 Jul 2019 10:08:12			**File Name**	C:\Users\Fedor\Desktop\15.07.19\FZ7543-1.jdf	**Frequency (MHz)**	600.17
**Nucleus**	1H	**Number of Transients**	8	**Origin**	ECA 600	**Original Points Count** 32768	**Owner** delta
**Points Count**	32768	**Pulse Sequence**	single_pulse.ex2			**Receiver Gain** 48.00	**Solvent** CHLOROFORM-d
**Spectrum Offset (Hz)**	5409.6182	**Sweep Width (Hz)**	16534.39	**Temperature (degree C)**	22.200		

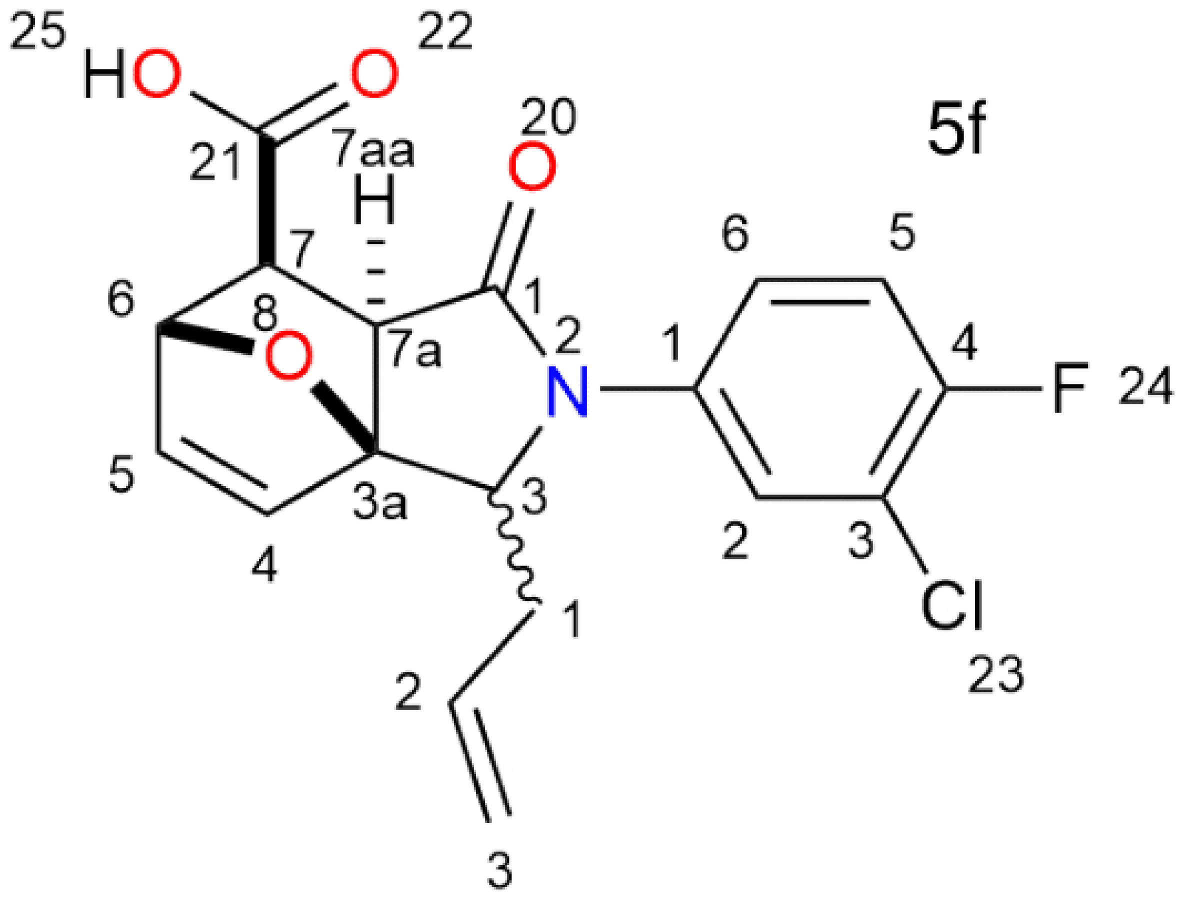

FZ7543-1.jdf

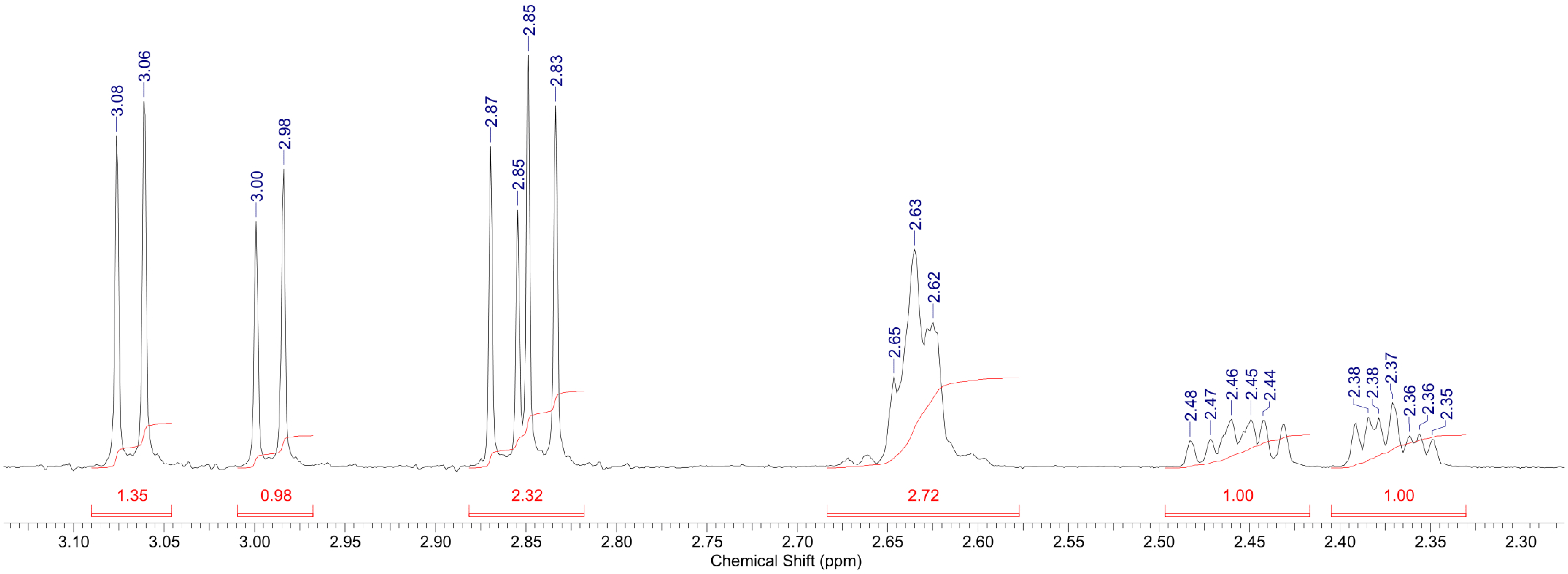

Acquisition Time (sec)	0.6921	***Comment***	single pulse decoupled gated NOE			***Date***	13 May 1990 12:30:32
Date Stamp	24 Jul 2019 00:21:51			***File Name***	C:\Users\Fedor\Desktop\22.07.19\FZ7581-1.jdf		
Frequency (MHz)	150.91	***Nucleus***	13C	***Number of Transients***	800	***Origin***	ECA 600
Original Points Count	32768	***Owner***	delta	***Points Count***	32768	***Pulse Sequence***	single_pulse_dec
Receiver Gain	56.00	***Solvent***	CHLOROFORM-d			***Spectrum Offset (Hz)***	15091.3428
Sweep Width (Hz)	47348.49	***Temperature (degree C)***	23.800				

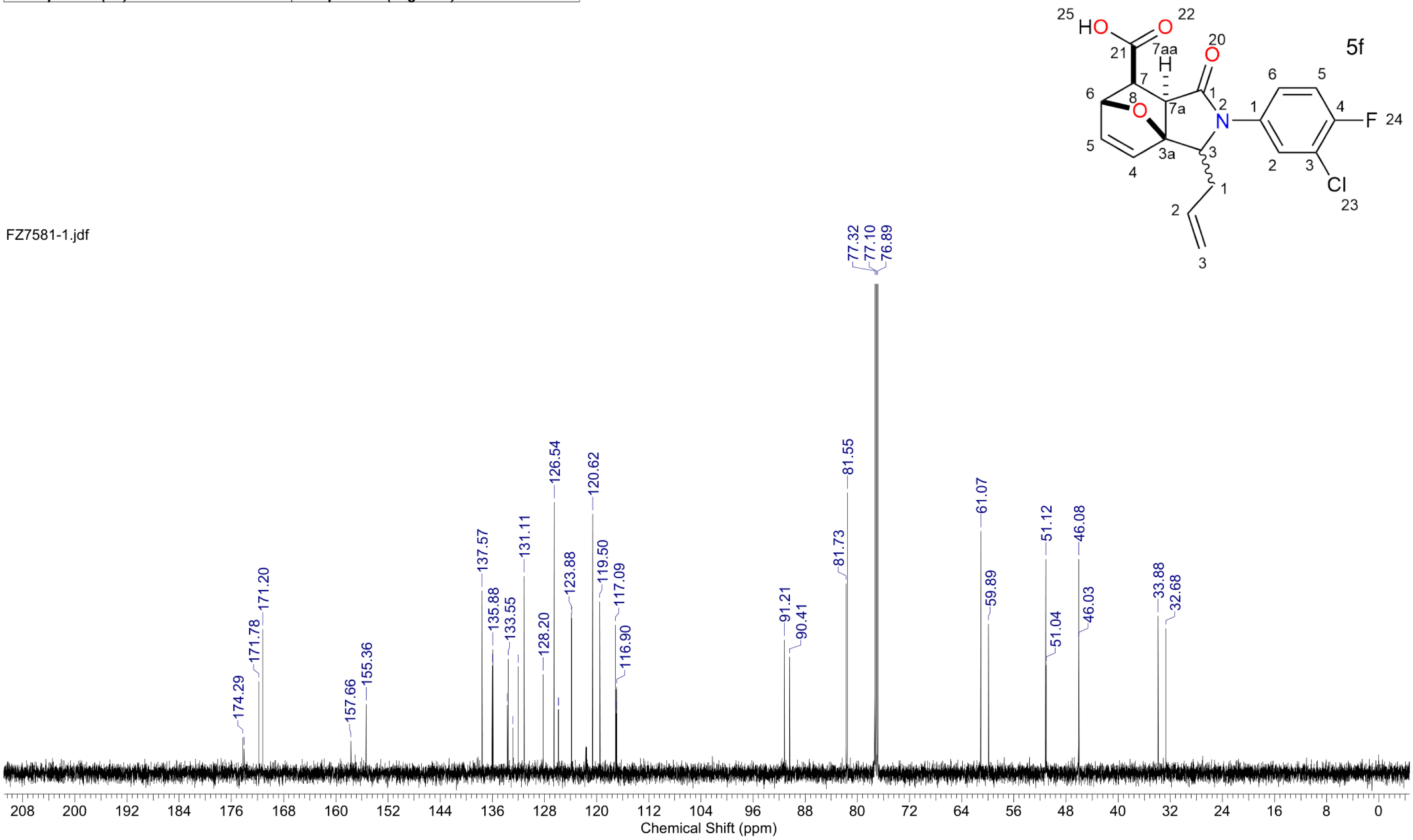

Acquisition Time (sec)	0.6921	***Comment***	single pulse decoupled gated NOE			***Date***	13 May 1990 12:30:32
Date Stamp	24 Jul 2019 00:21:51			***File Name***	C:\Users\Fedor\Desktop\22.07.19\FZ7581-1.jdf		
Frequency (MHz)	150.91	***Nucleus***	13C	***Number of Transients***	800	***Origin***	ECA 600
Original Points Count	32768	***Owner***	delta	***Points Count***	32768	***Pulse Sequence***	single_pulse_dec
Receiver Gain	56.00	***Solvent***	CHLOROFORM-d			***Spectrum Offset (Hz)***	15091.3428
Sweep Width (Hz)	47348.49	***Temperature (degree C)***	23.800				

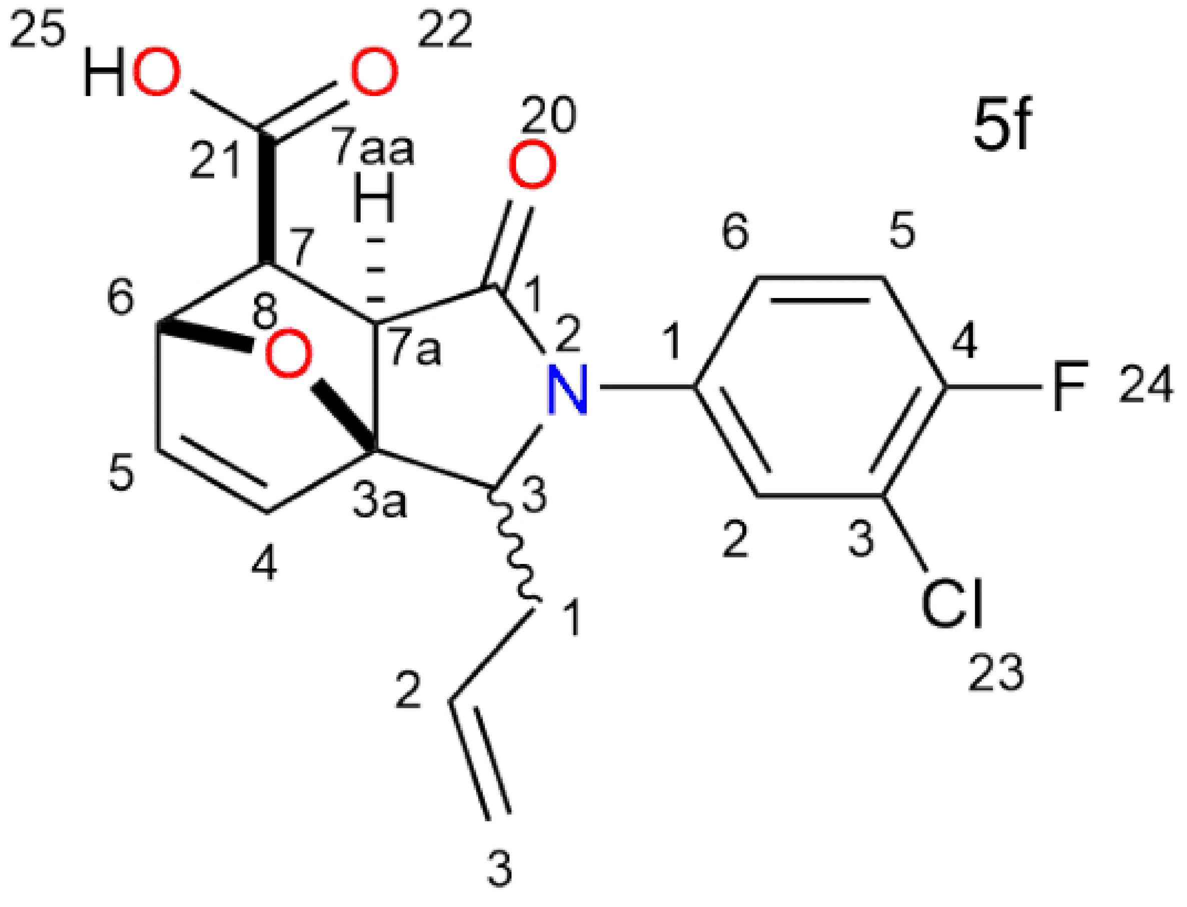

5f

FZ7581-1.jdf

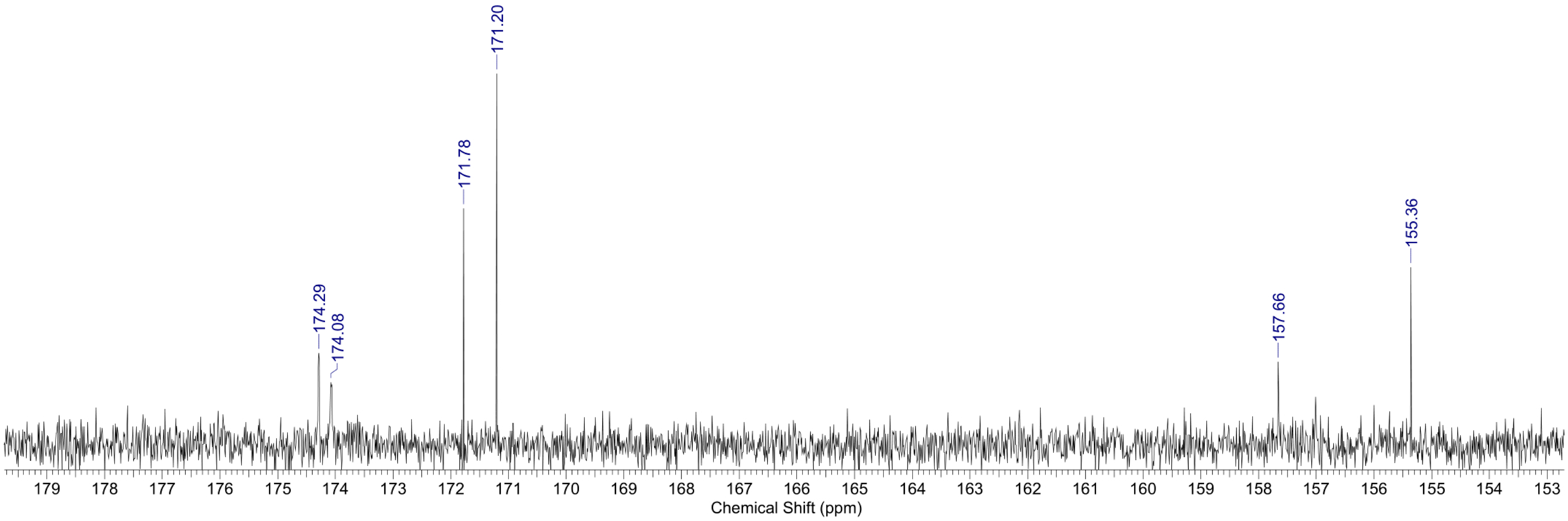

Acquisition Time (sec)	0.6921	***Comment***	single pulse decoupled gated NOE	***Date***	13 May 1990 12:30:32
Date Stamp	24 Jul 2019 00:21:51	***File Name***	C:\Users\Fedor\Desktop\22.07.19\FZ7581-1.jdf		
Frequency (MHz)	150.91	***Nucleus***	13C	***Number of Transients***	800
Origin	ECA 600	***Original Points Count***	32768	***Owner***	delta
Points Count	32768	***Pulse Sequence***	single_pulse_dec	***Receiver Gain***	56.00
Solvent	CHLOROFORM-d	***Spectrum Offset (Hz)***	15091.3428	***Sweep Width (Hz)***	47348.49
Temperature (degree C)	23.800				

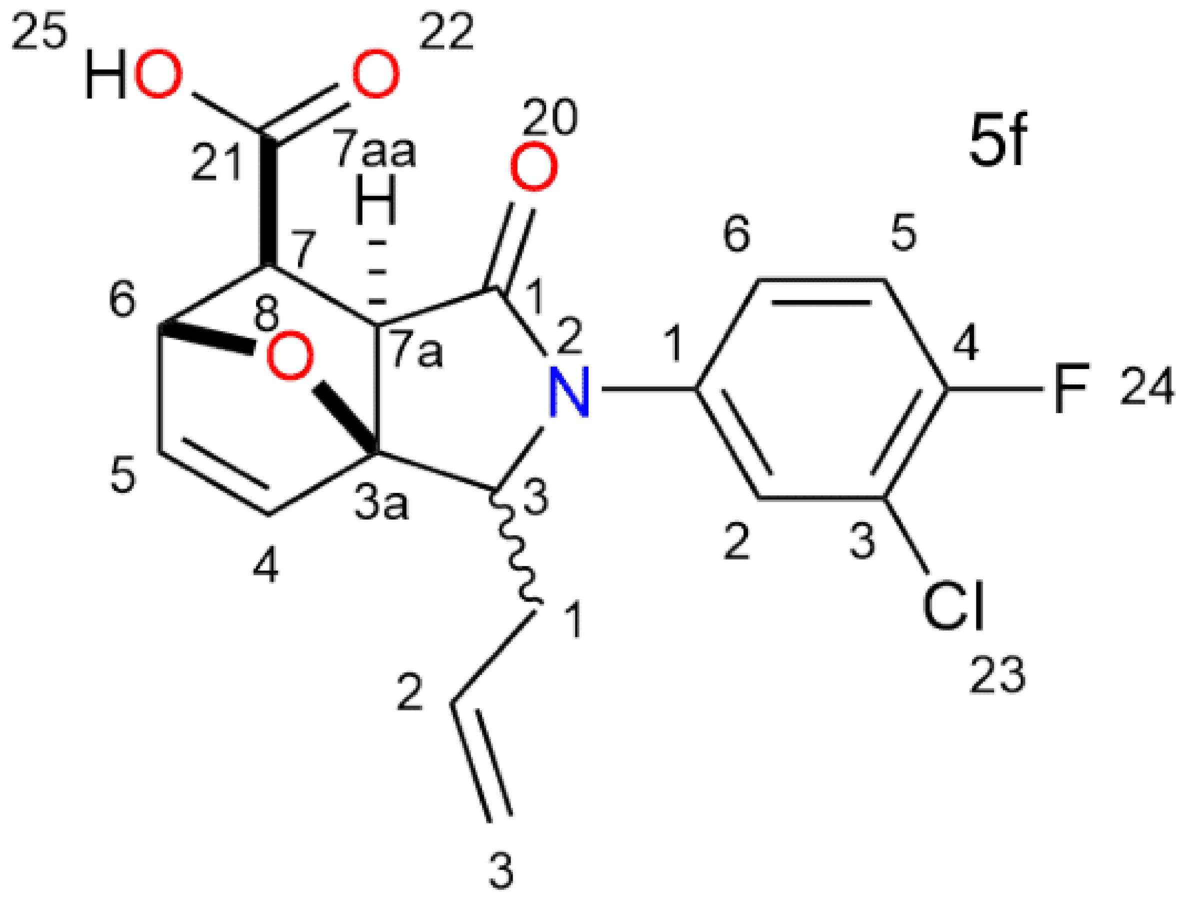

5f

FZ7581-1.jdf

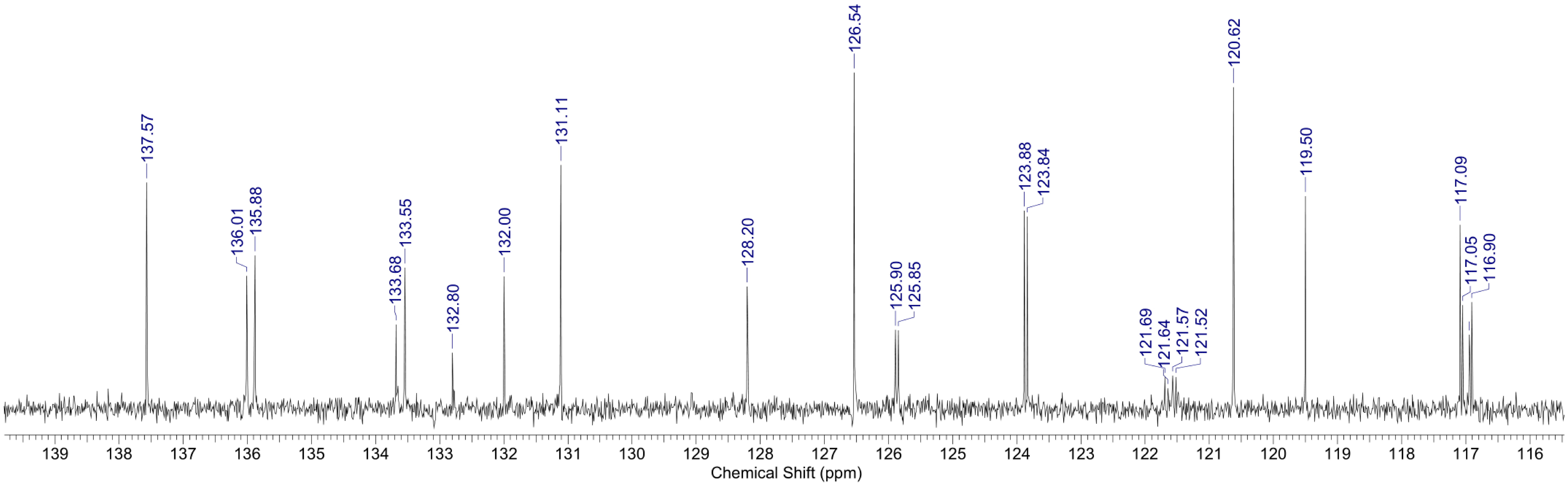

Acquisition Time (sec)	0.6921	***Comment***	single pulse decoupled gated NOE			***Date***	13 May 1990 12:30:32
Date Stamp	24 Jul 2019 00:21:51			***File Name***	C:\Users\Fedor\Desktop\22.07.19\FZ7581-1.jdf		
Frequency (MHz)	150.91	***Nucleus***	13C	***Number of Transients***	800	***Origin***	ECA 600
Original Points Count	32768	***Owner***	delta	***Points Count***	32768	***Pulse Sequence***	single_pulse_dec
Receiver Gain	56.00	***Solvent***	CHLOROFORM-d			***Spectrum Offset (Hz)***	15091.3428
Sweep Width (Hz)	47348.49	***Temperature (degree C)***	23.800				

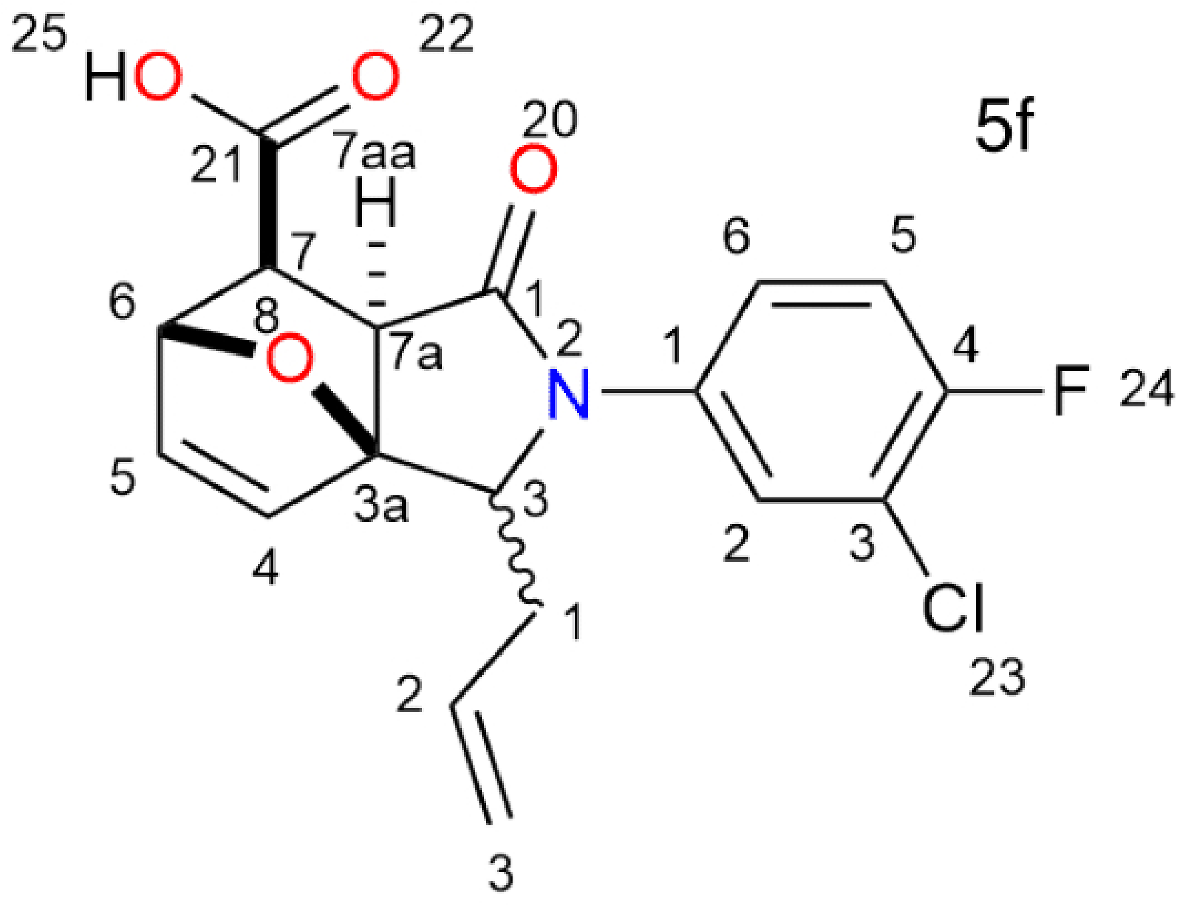

5f

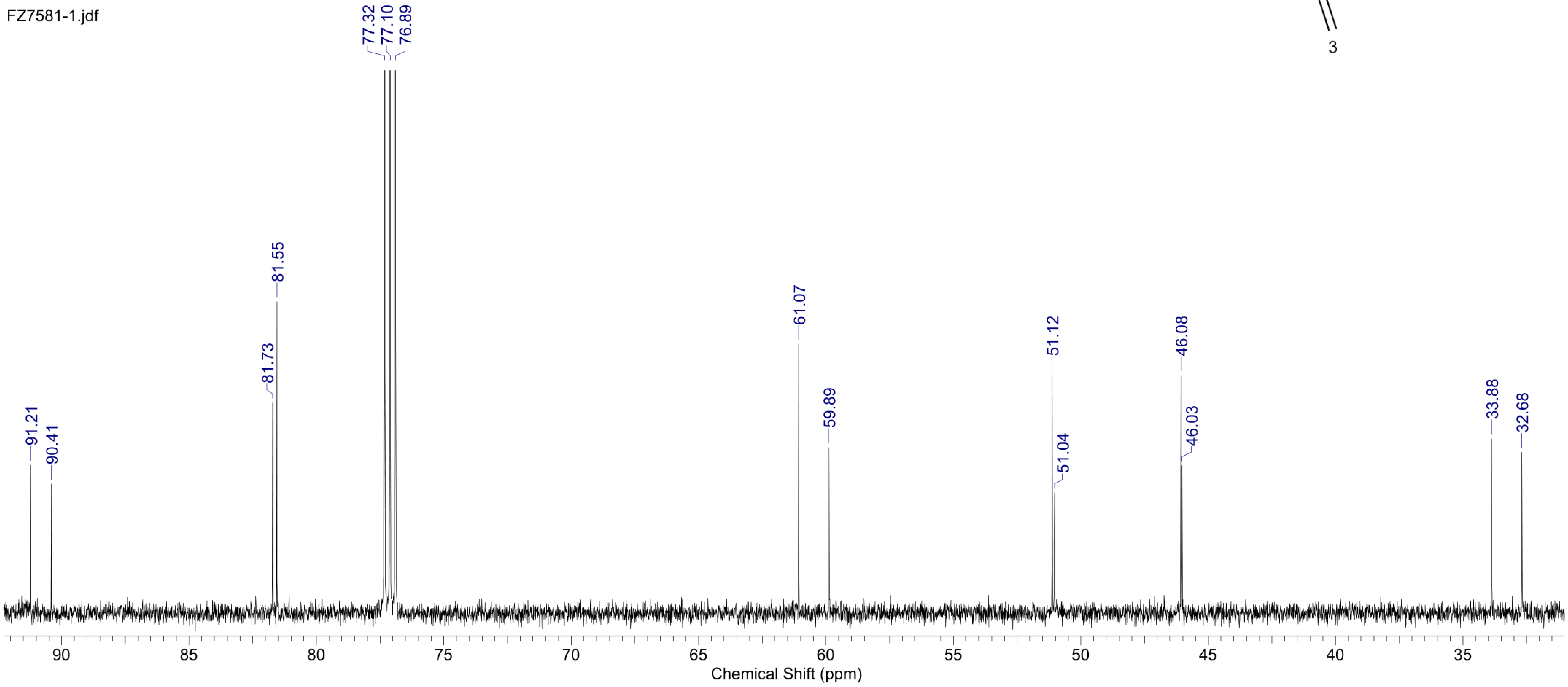

Acquisition Time (sec)	0.4614	***Comment***	single_pulse	***Date***	06 Mar 2020 11:31:05			***Date Stamp***	06 Mar 2020 10:50:59
File Name	E:\FZ8559-1.JDF			***Frequency (MHz)***	564.73	***Nucleus***	19F	***Number of Transients***	8
Origin	ECA 600	***Original Points Count***	65536	***Owner***	CKP	***Points Count***	65536	***Pulse Sequence***	single_pulse.ex2
Receiver Gain	44.00	***Solvent***	CHLOROFORM-d			***Spectrum Offset (Hz)***	-56472.6094		
Sweep Width (Hz)	142045.45	***Temperature (degree C)***	23.400						

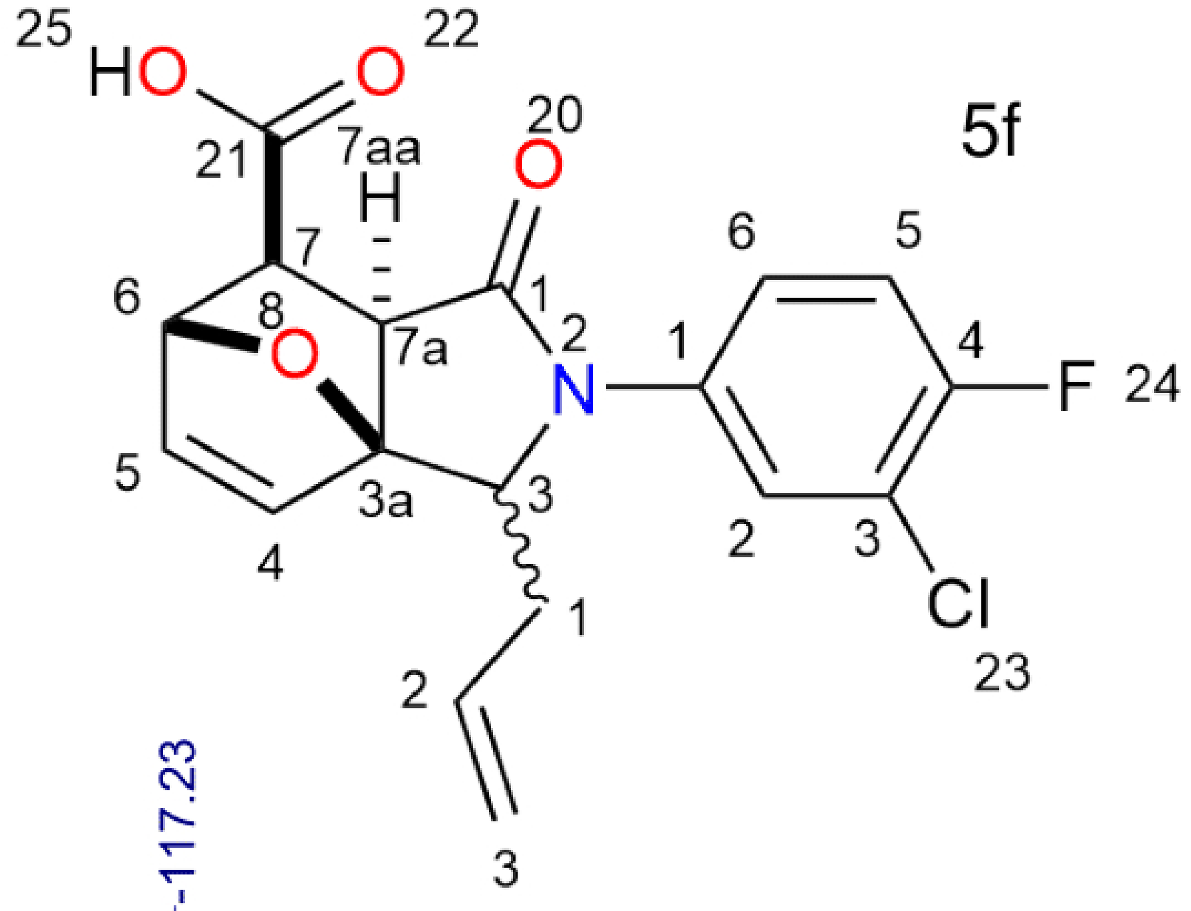

5f

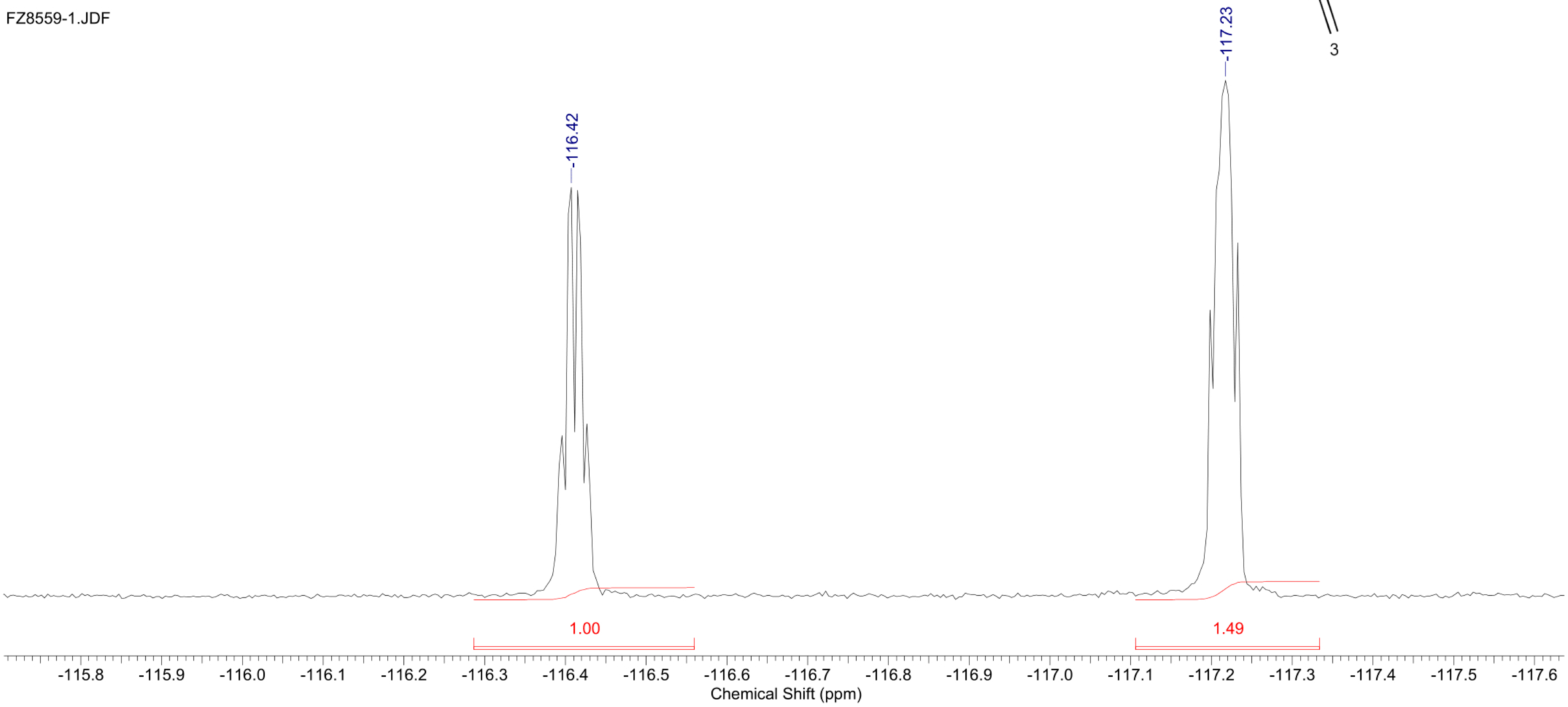

NMR spectrum data for compound 5k

Acquisition Time (sec)	1.9818	***Comment***	single_pulse	***Date***	23 May 1990 04:22:13				
Date Stamp	02 Aug 2019 10:25:55			***File Name***	C:\Users\Fedor\Desktop\01.08.19\FZ7628-1.jdf			***Frequency (MHz)***	600.17
Nucleus	1H	***Number of Transients***	8	***Origin***	ECA 600	***Original Points Count***	32768	***Owner***	delta
Points Count	32768	***Pulse Sequence***	single_pulse.ex2			***Receiver Gain***	42.00	***Solvent***	CHLOROFORM-d
Spectrum Offset (Hz)	5410.6274	***Sweep Width (Hz)***	16534.39	***Temperature (degree C)***	20.600				

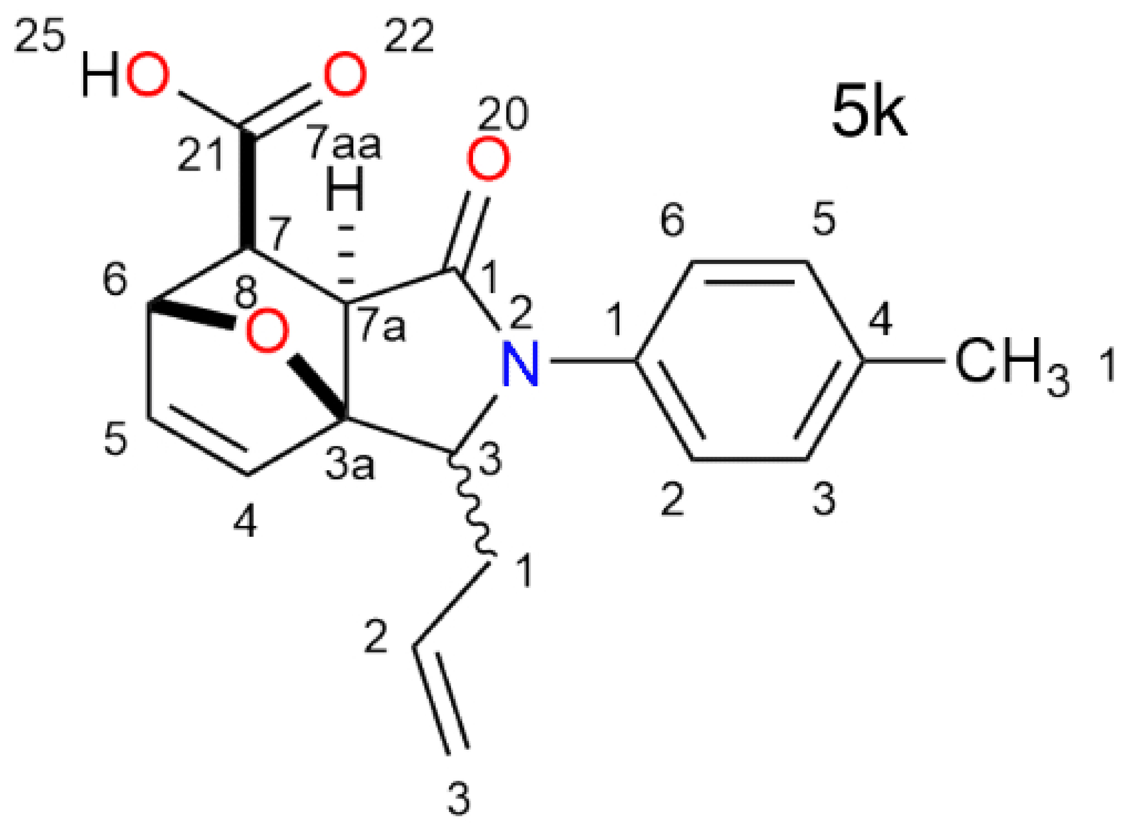

5k

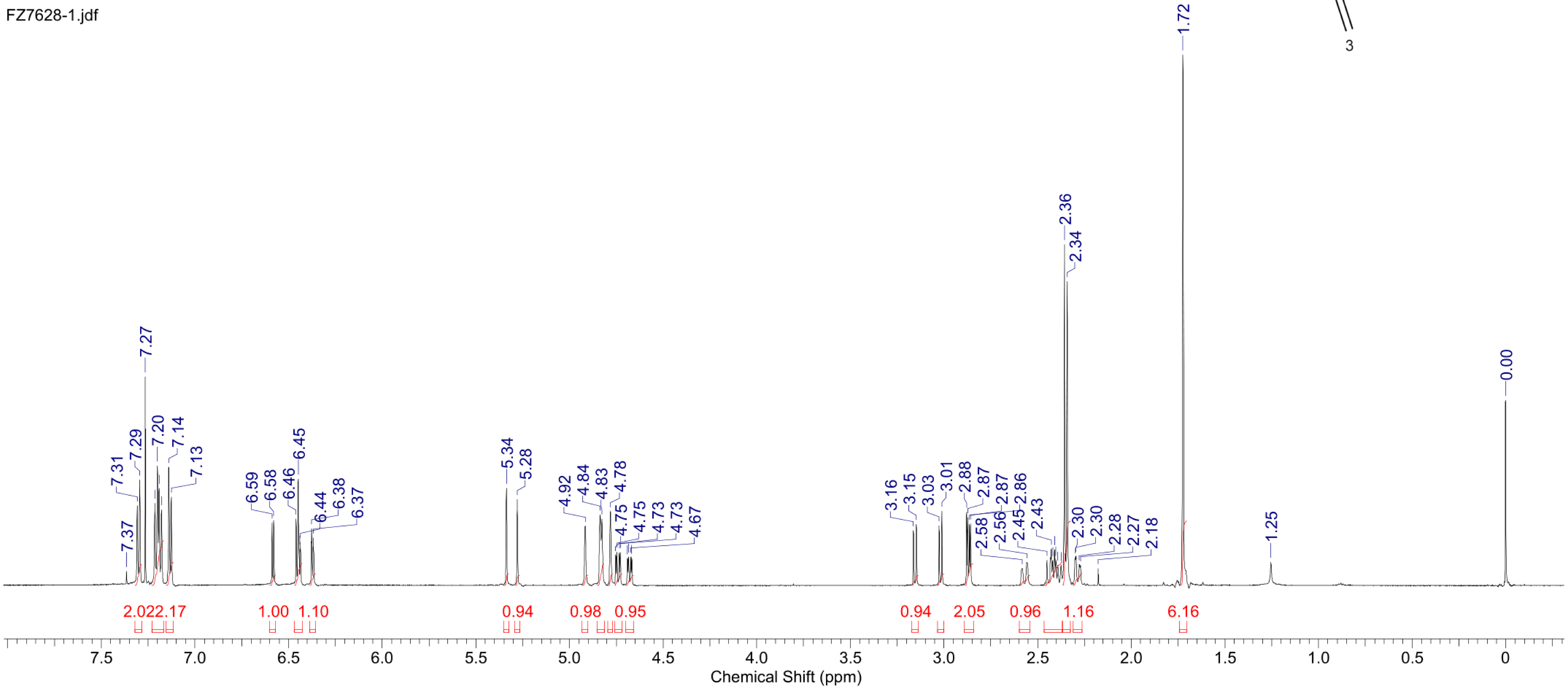

Acquisition Time (sec)	1.9818	***Comment***	single_pulse	***Date***	23 May 1990 04:22:13				
Date Stamp	02 Aug 2019 10:25:55			***File Name***	C:\Users\Fedor\Desktop\01.08.19\FZ7628-1.jdf			***Frequency (MHz)***	600.17
Nucleus	1H	***Number of Transients***	8	***Origin***	ECA 600	***Original Points Count***	32768	***Owner***	delta
Points Count	32768	***Pulse Sequence***	single_pulse.ex2			***Receiver Gain***	42.00	***Solvent***	CHLOROFORM-d
Spectrum Offset (Hz)	5410.6274	***Sweep Width (Hz)***	16534.39	***Temperature (degree C)***	20.600				

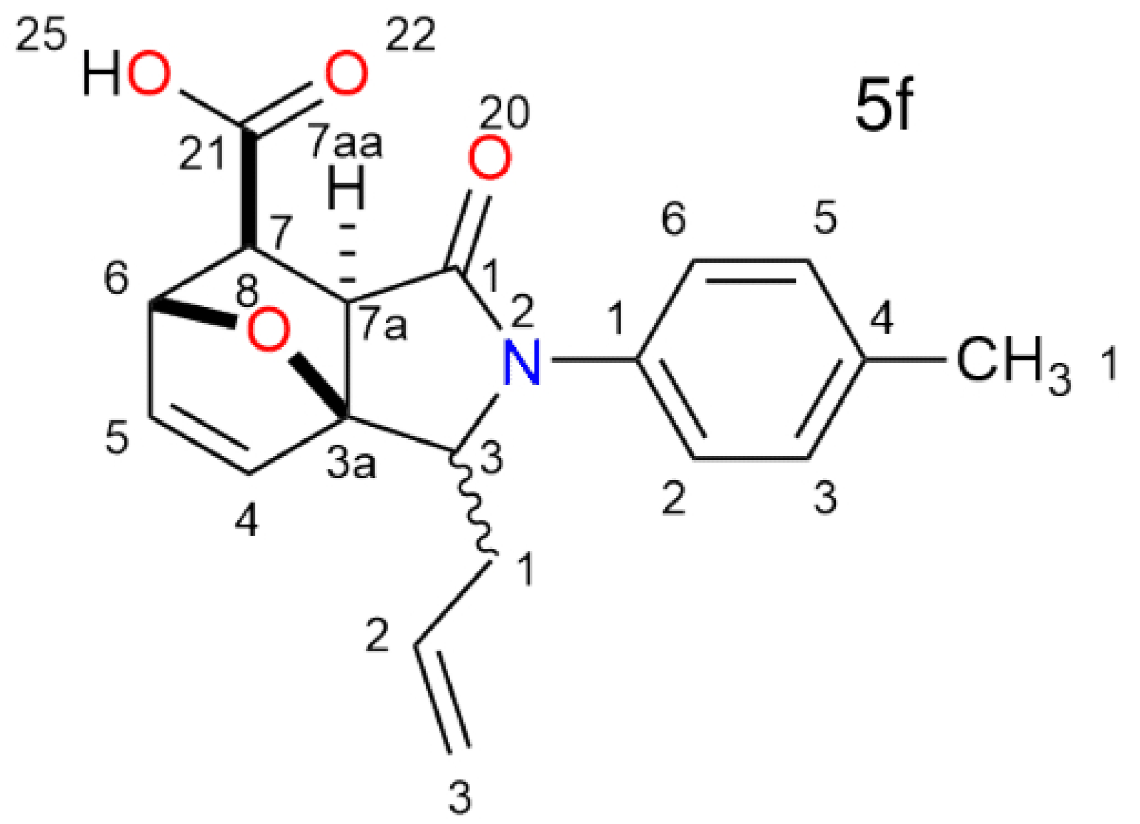

5f

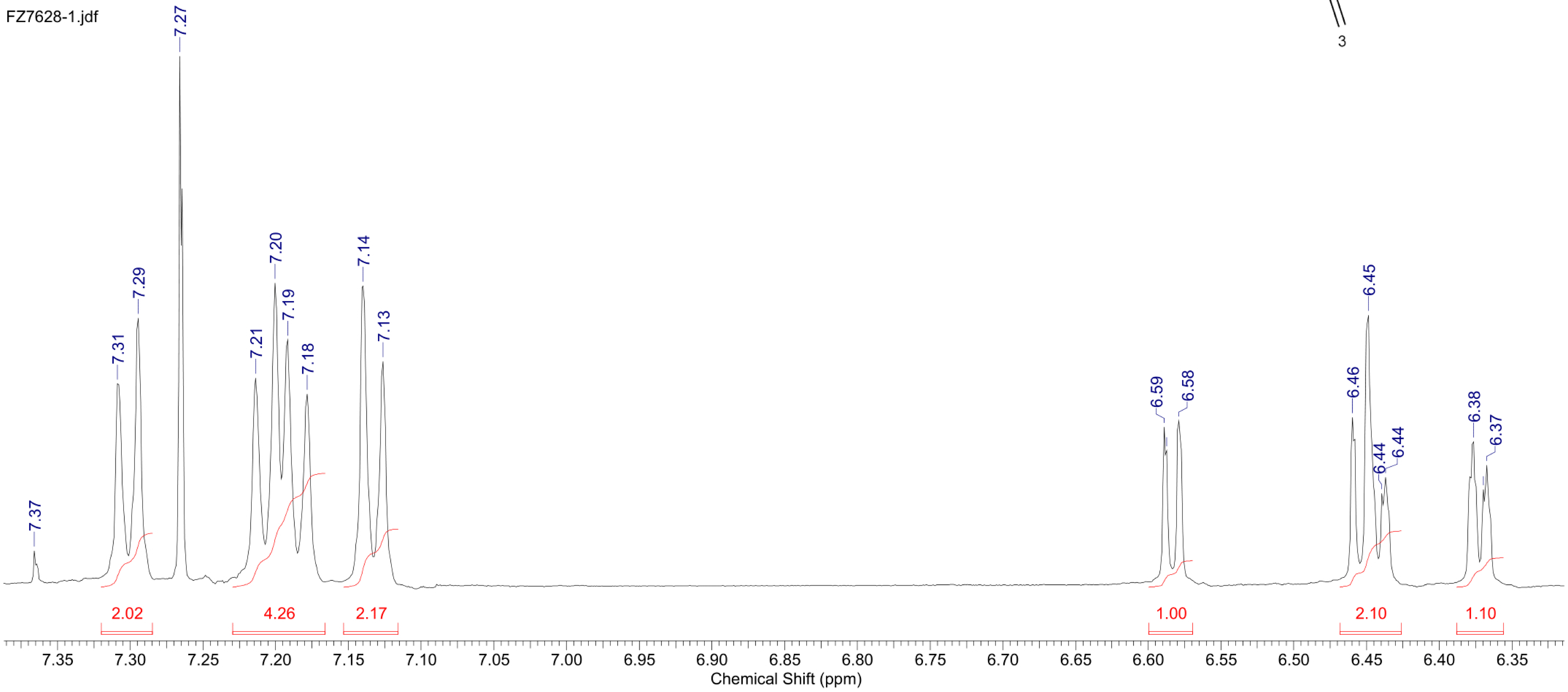

Acquisition Time (sec)	1.9818	***Comment***	single_pulse	***Date***	23 May 1990 04:22:13		
Date Stamp	02 Aug 2019 10:25:55			***File Name***	C:\Users\Fedor\Desktop\01.08.19\FZ7628-1.jdf	***Frequency (MHz)***	600.17
Nucleus	1H	***Number of Transients***	8	***Origin***	ECA 600	***Original Points Count***	32768
						Owner	delta
Points Count	32768	***Pulse Sequence***	single_pulse.ex2	***Receiver Gain***	42.00	***Solvent***	CHLOROFORM-d
Spectrum Offset (Hz)	5410.6274	***Sweep Width (Hz)***	16534.39	***Temperature (degree C)***	20.600		

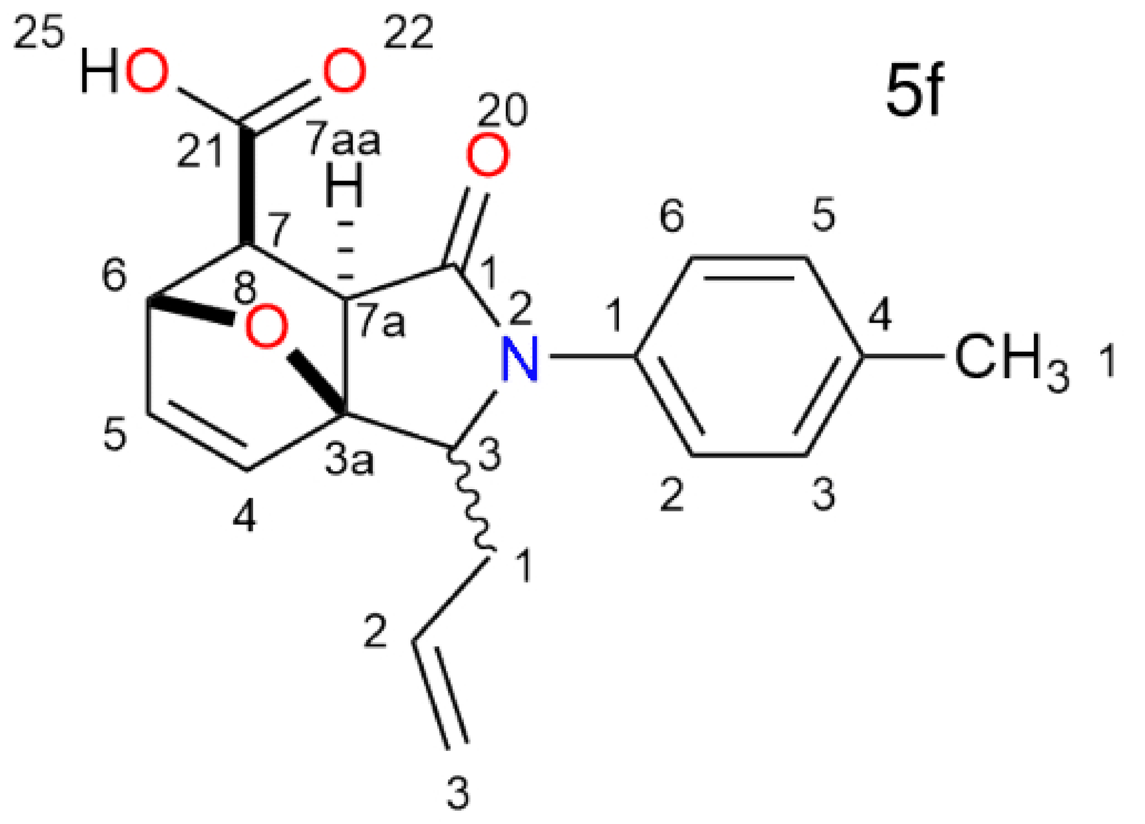

5f

FZ7628-1.jdf

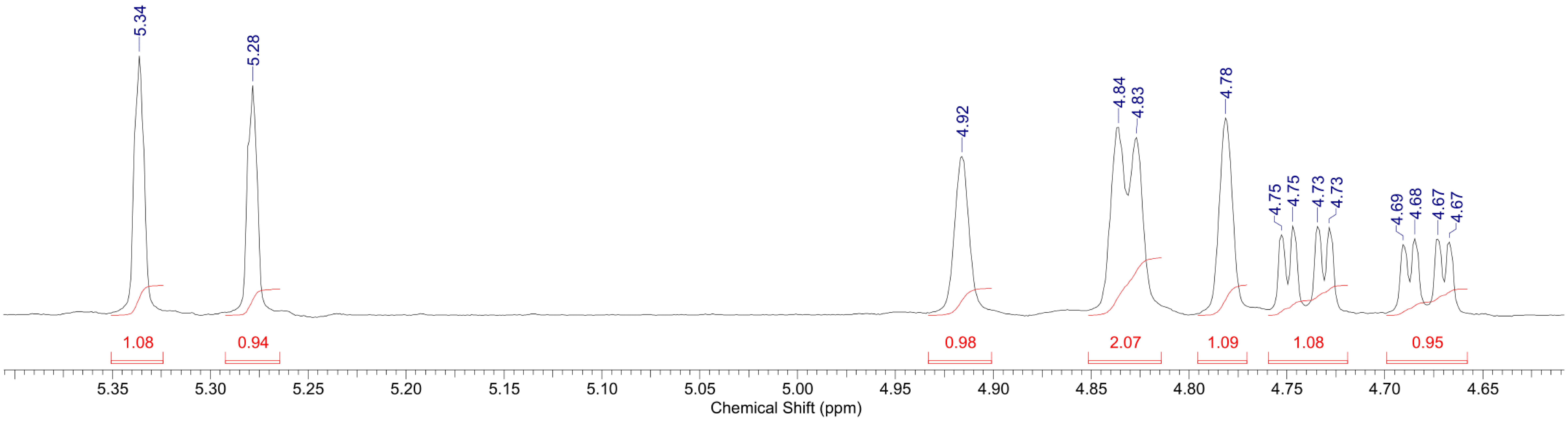

Acquisition Time (sec)	1.9818	Comment	single_pulse	Date	23 May 1990 04:22:13		
Date Stamp	02 Aug 2019 10:25:55			File Name	C:\Users\Fedor\Desktop\01.08.19\FZ7628-1.jdf	Frequency (MHz)	600.17
Nucleus	1H	Number of Transients	8	Origin	ECA 600	Original Points Count	32768	Owner	delta
Points Count	32768	Pulse Sequence	single_pulse.ex2			Receiver Gain	42.00	Solvent	CHLOROFORM-d
Spectrum Offset (Hz)	5410.6274	Sweep Width (Hz)	16534.39	Temperature (degree C)	20.600		

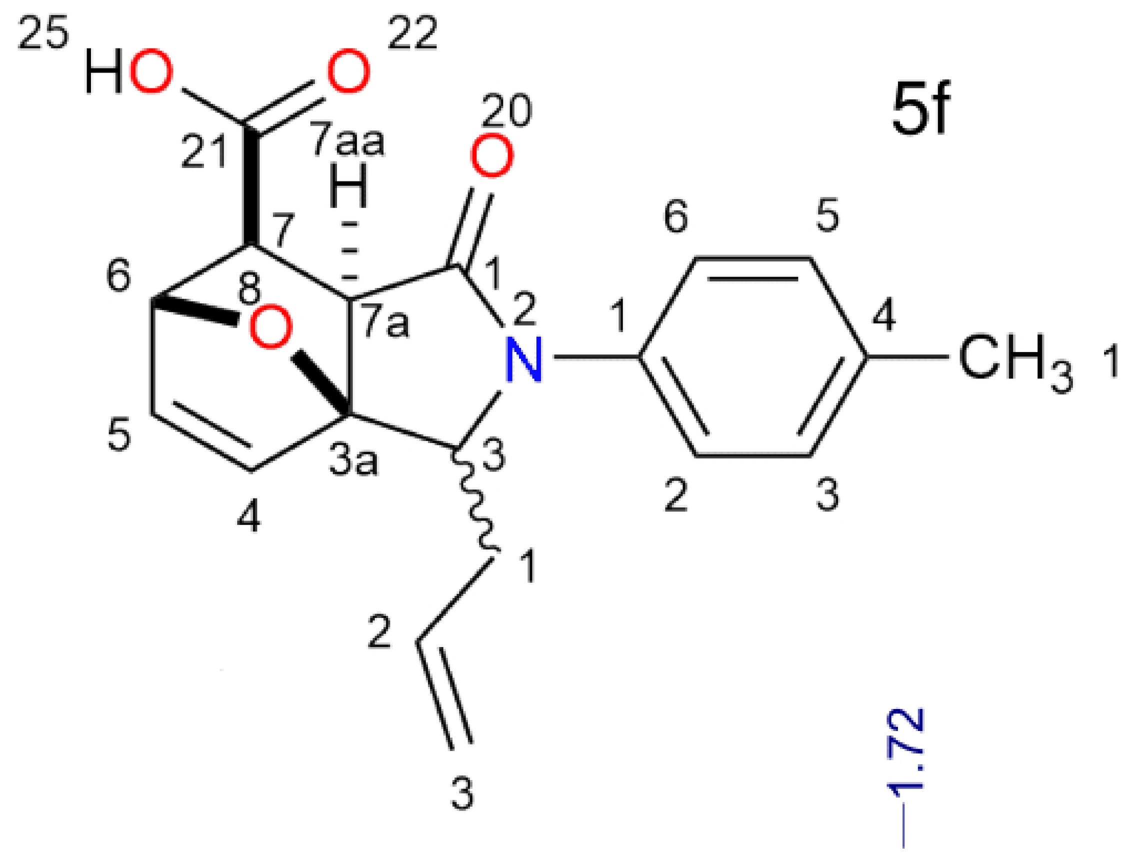

5f

FZ7628-1.jdf

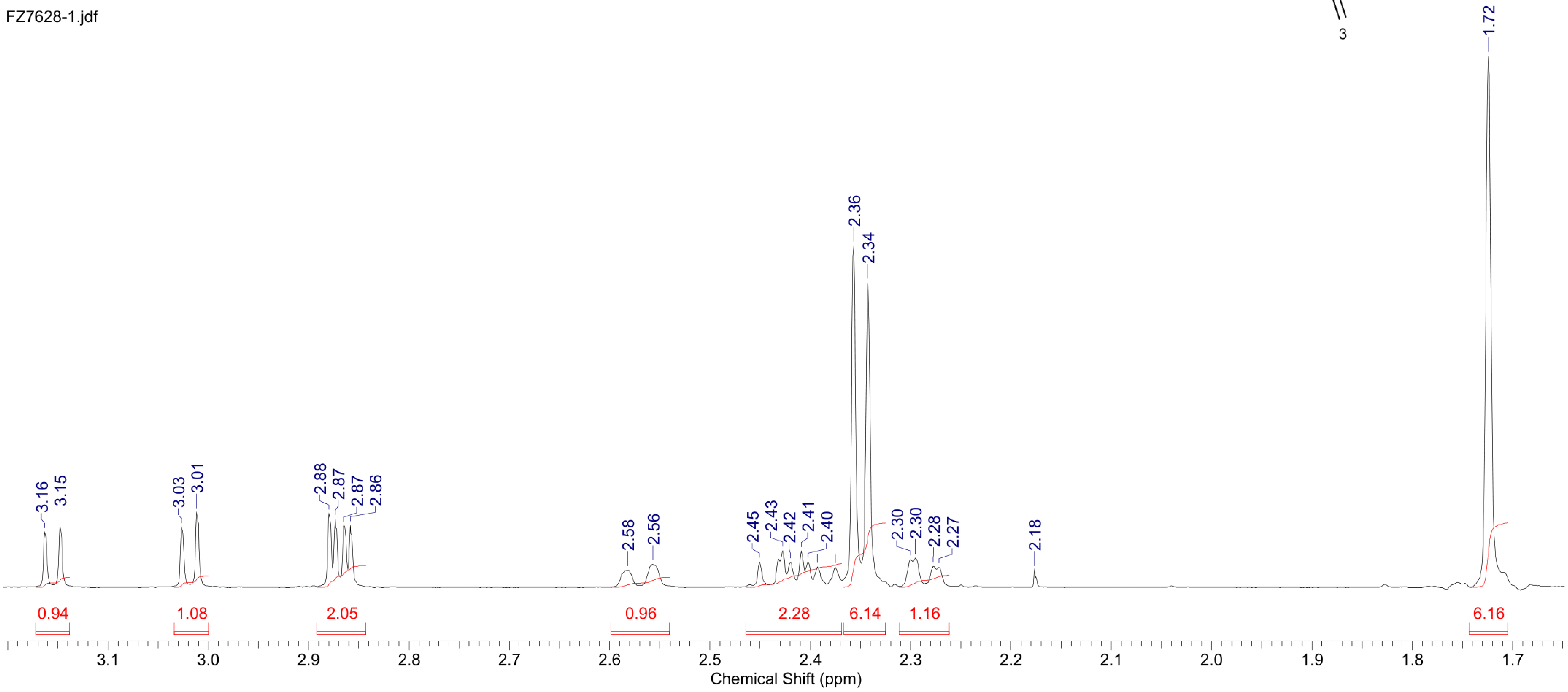

Acquisition Time (sec)	0.6921	***Comment***	single pulse decoupled gated NOE	***Date***	20 Jun 1990 07:19:45
Date Stamp	30 Aug 2019 13:22:56	***File Name***	C:\Users\Fedor\Desktop\30.08.19\FZ7747-1.jdf		
Frequency (MHz)	150.91	***Nucleus***	13C	***Number of Transients***	800
Origin	ECA 600	***Original Points Count***	32768	***Owner***	delta
Points Count	32768	***Pulse Sequence***	single_pulse_dec	***Receiver Gain***	56.00
Solvent	CHLOROFORM-d	***Spectrum Offset (Hz)***	15079.3525	***Sweep Width (Hz)***	47348.49
Temperature (degree C)	24.200				

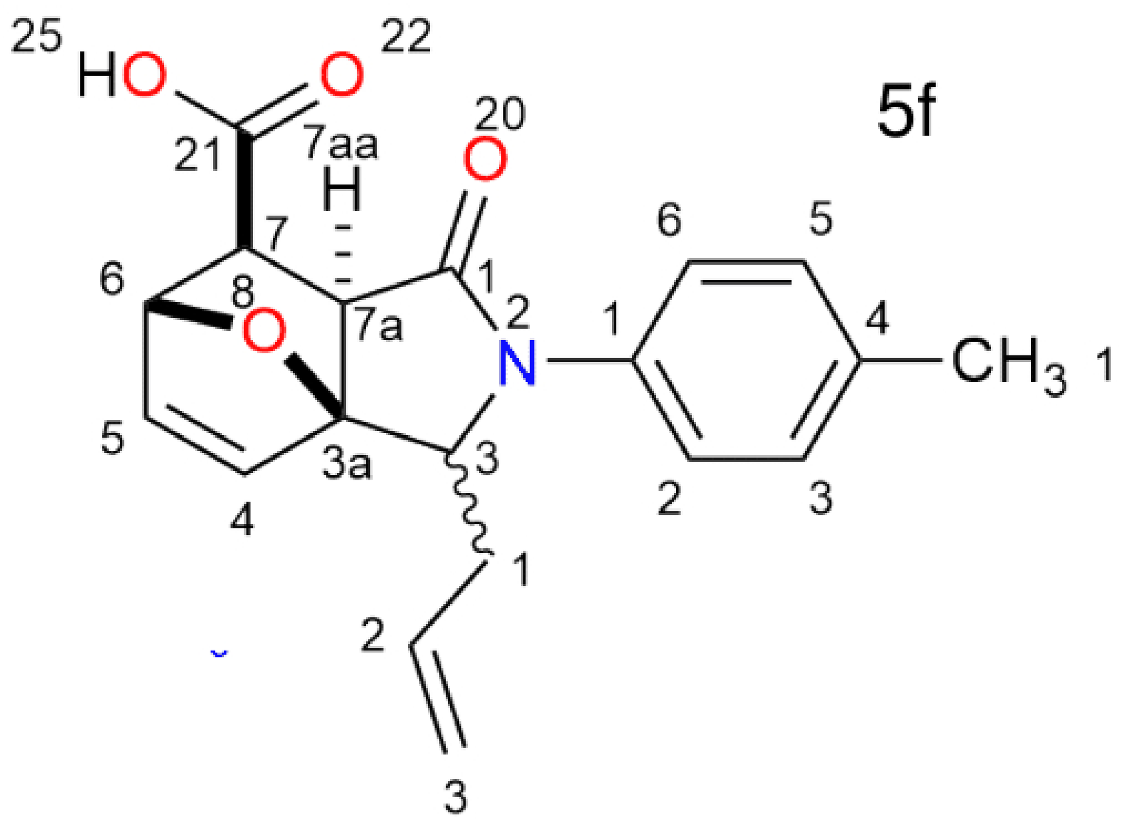

5f

FZ7747-1.esp

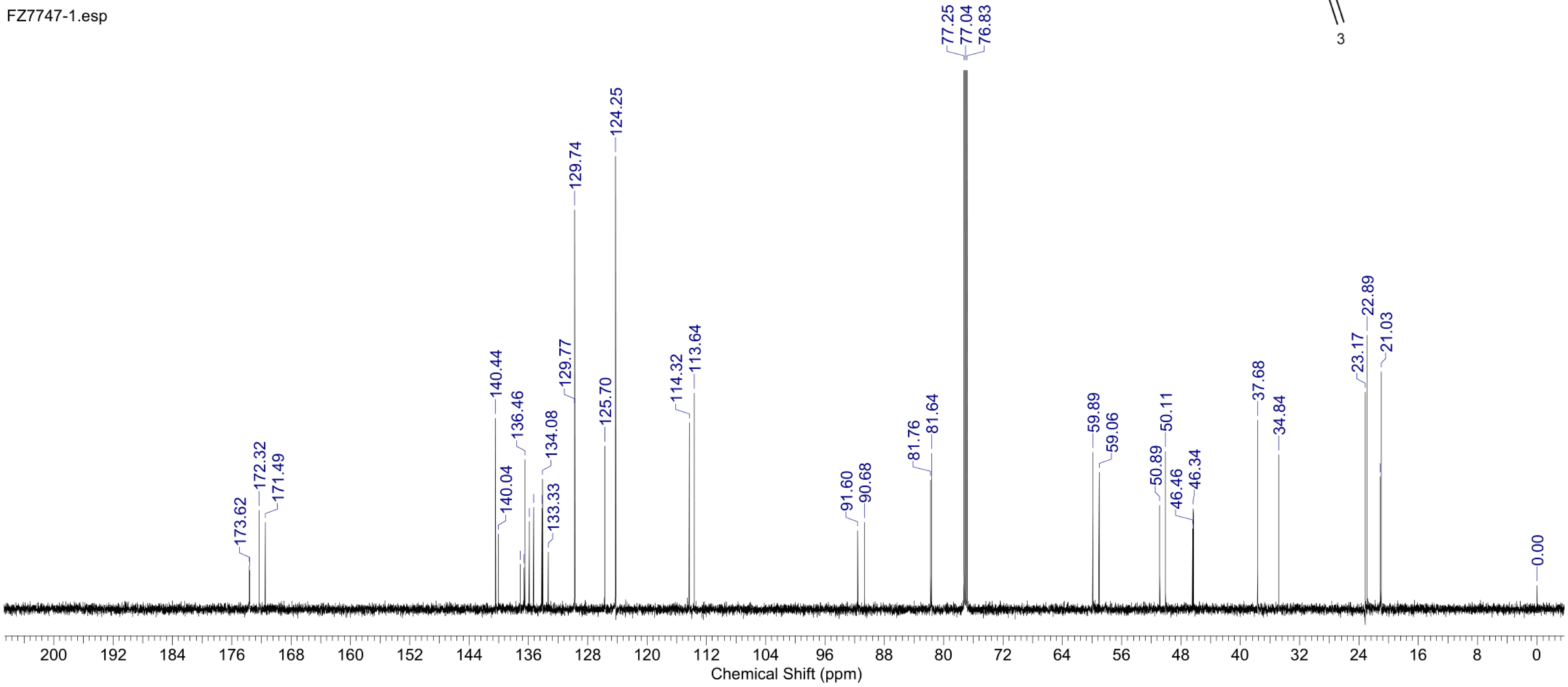

Acquisition Time (sec)	0.6921	***Comment***	single pulse decoupled gated NOE			***Date***	20 Jun 1990 07:19:45
Date Stamp	30 Aug 2019 13:22:56			***File Name***	C:\Users\Fedor\Desktop\30.08.19\FZ7747-1.jdf		
Frequency (MHz)	150.91	***Nucleus***	13C	***Number of Transients***	800	***Origin***	ECA 600
Original Points Count	32768	***Owner***	delta	***Points Count***	32768	***Pulse Sequence***	single_pulse_dec
Receiver Gain	56.00	***Solvent***	CHLOROFORM-d			***Spectrum Offset (Hz)***	15079.3525
Sweep Width (Hz)	47348.49	***Temperature (degree C)***	24.200				

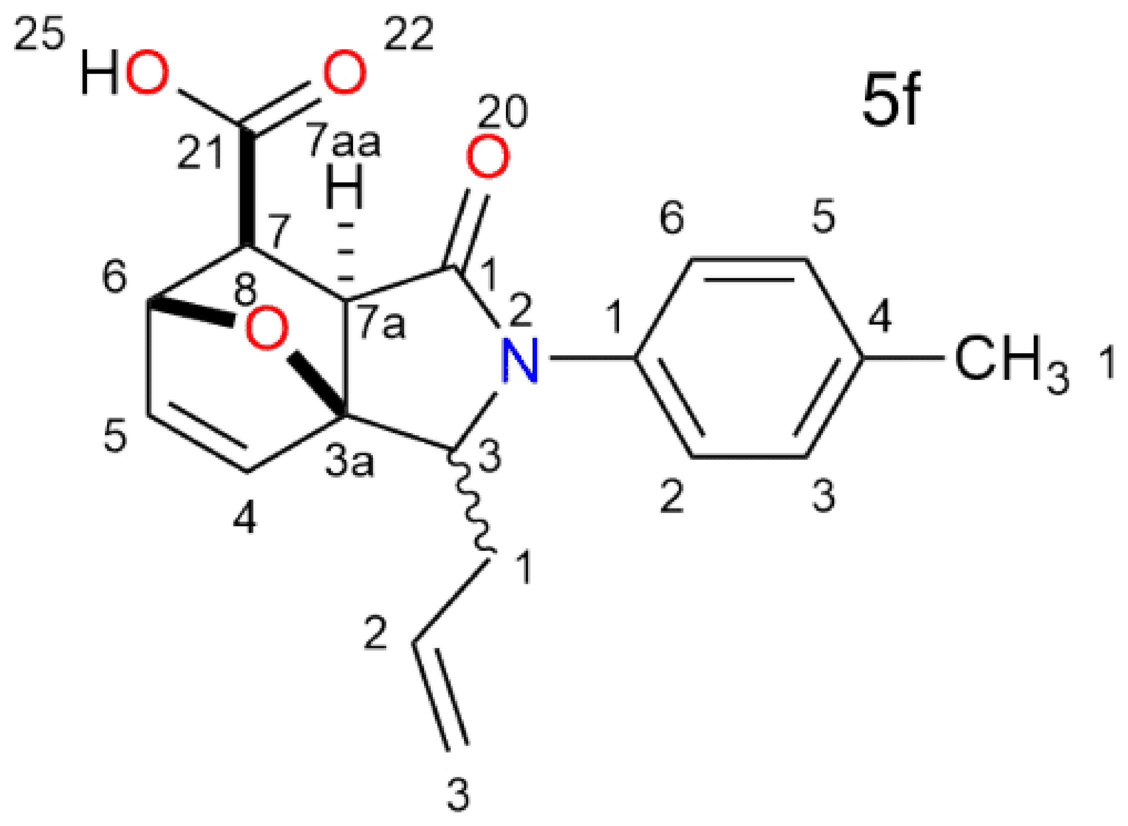

5f

FZ7747-1.esp

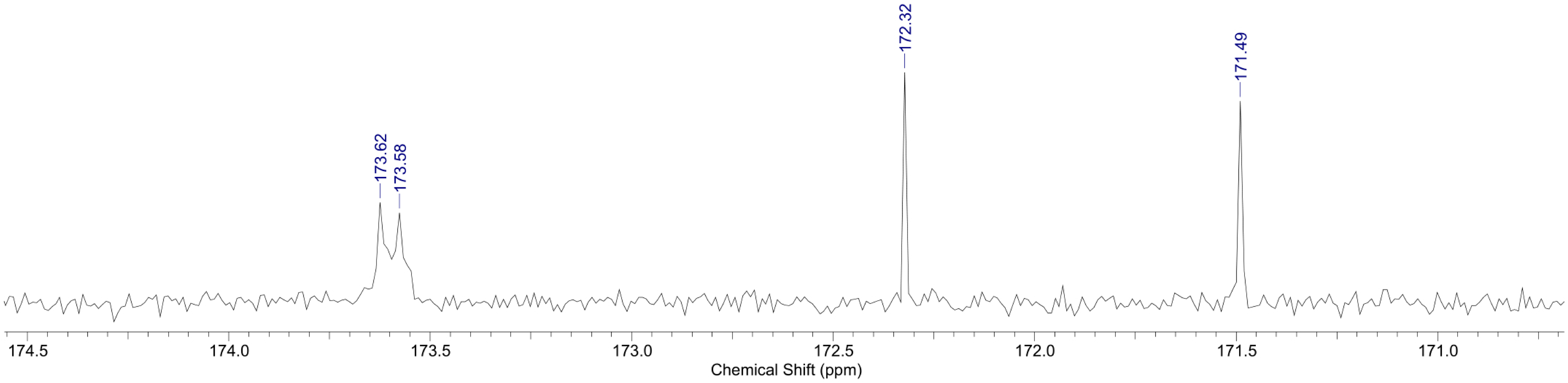

Acquisition Time (sec)	0.6921	***Comment***	single pulse decoupled gated NOE	***Date***	20 Jun 1990 07:19:45
Date Stamp	30 Aug 2019 13:22:56	***File Name***	C:\Users\Fedor\Desktop\30.08.19\FZ7747-1.jdf		
Frequency (MHz)	150.91	***Nucleus***	13C	***Number of Transients***	800
Origin	ECA 600	***Original Points Count***	32768	***Owner***	delta
Points Count	32768	***Pulse Sequence***	single_pulse_dec	***Receiver Gain***	56.00
Solvent	CHLOROFORM-d	***Spectrum Offset (Hz)***	15079.3525	***Sweep Width (Hz)***	47348.49
Temperature (degree C)	24.200				

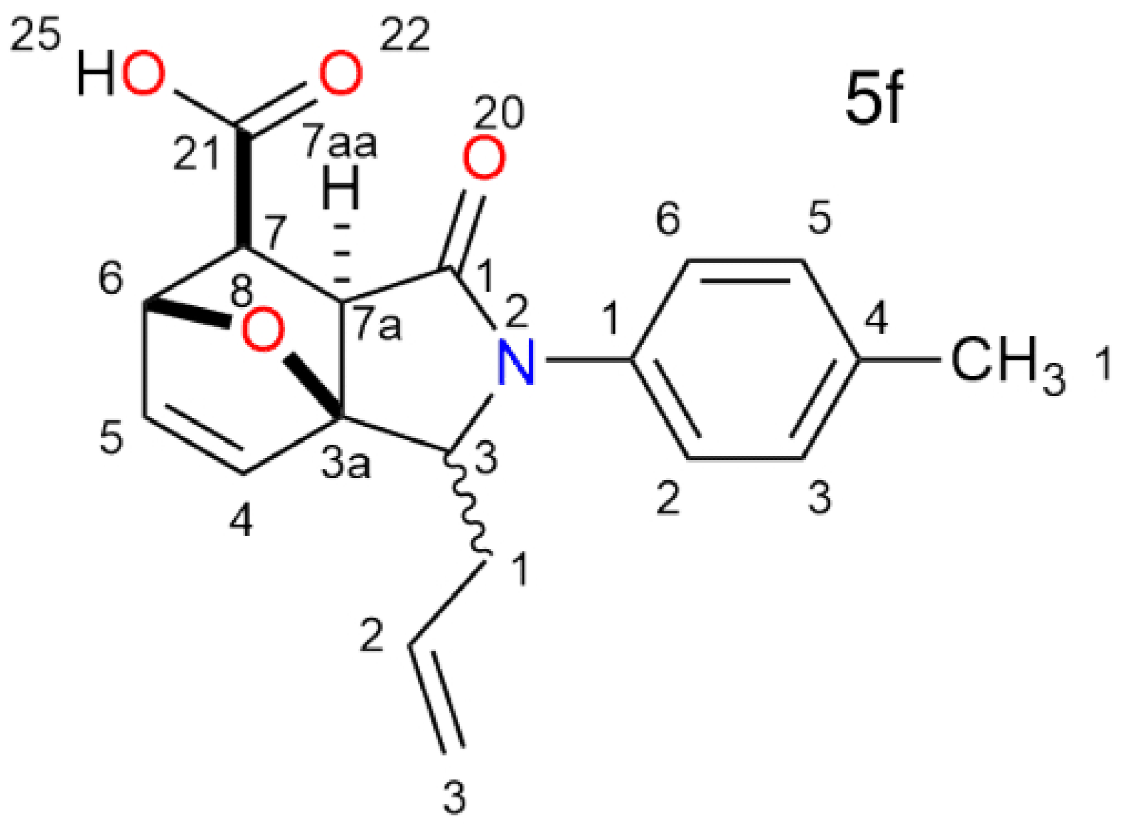

5f

FZ7747-1.esp

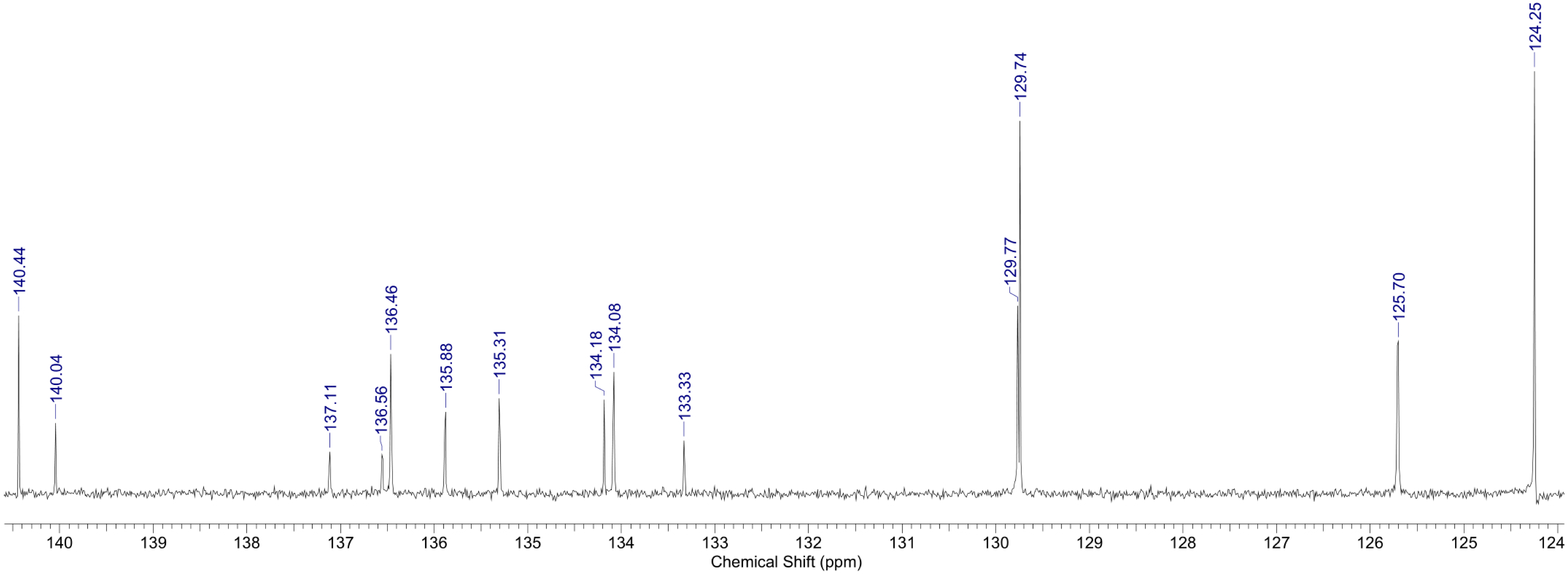

Acquisition Time (sec)	0.6921	***Comment***	single pulse decoupled gated NOE			***Date***	20 Jun 1990 07:19:45
Date Stamp	30 Aug 2019 13:22:56			***File Name***	C:\Users\Fedor\Desktop\30.08.19\FZ7747-1.jdf		
Frequency (MHz)	150.91	***Nucleus***	13C	***Number of Transients***	800	***Origin***	ECA 600
Original Points Count	32768	***Owner***	delta	***Points Count***	32768	***Pulse Sequence***	single_pulse_dec
Receiver Gain	56.00	***Solvent***	CHLOROFORM-d			***Spectrum Offset (Hz)***	15079.3525
Sweep Width (Hz)	47348.49	***Temperature (degree C)***	24.200				

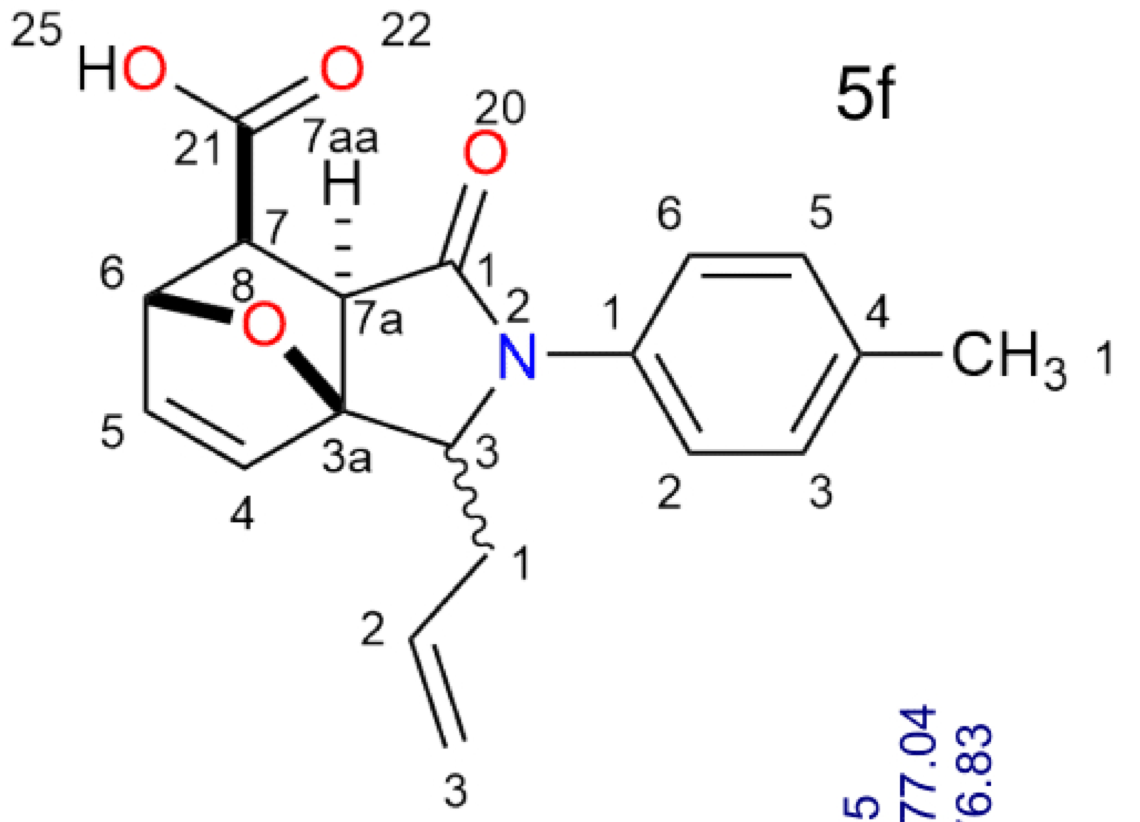

5f

FZ7747-1.esp

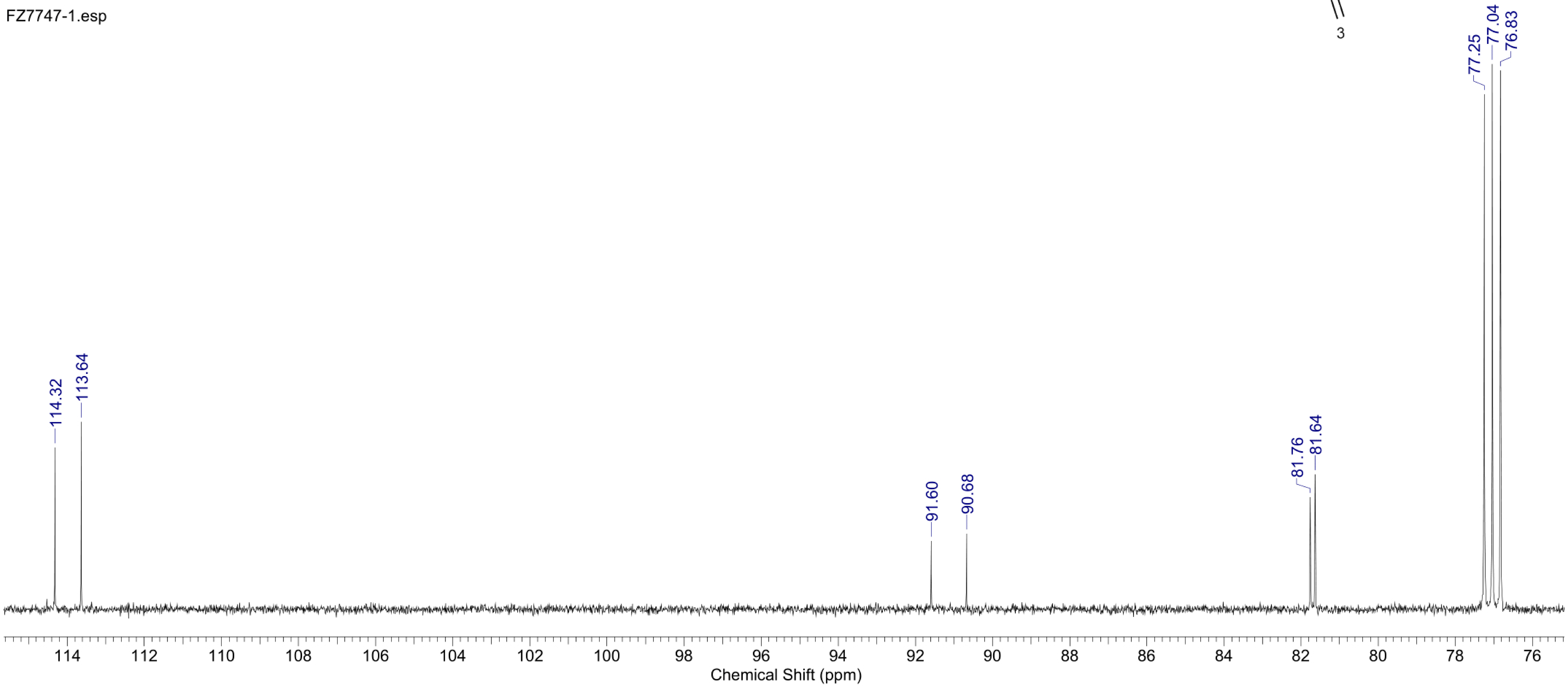

Acquisition Time (sec)	0.6921	***Comment***	single pulse decoupled gated NOE			***Date***	20 Jun 1990 07:19:45
Date Stamp	30 Aug 2019 13:22:56			***File Name***	C:\Users\Fedor\Desktop\30.08.19\FZ7747-1.jdf		
Frequency (MHz)	150.91	***Nucleus***	13C	***Number of Transients***	800	***Origin***	ECA 600
Original Points Count	32768	***Owner***	delta	***Points Count***	32768	***Pulse Sequence***	single_pulse_dec
Receiver Gain	56.00	***Solvent***	CHLOROFORM-d			***Spectrum Offset (Hz)***	15079.3525
Sweep Width (Hz)	47348.49	***Temperature (degree C)***	24.200				

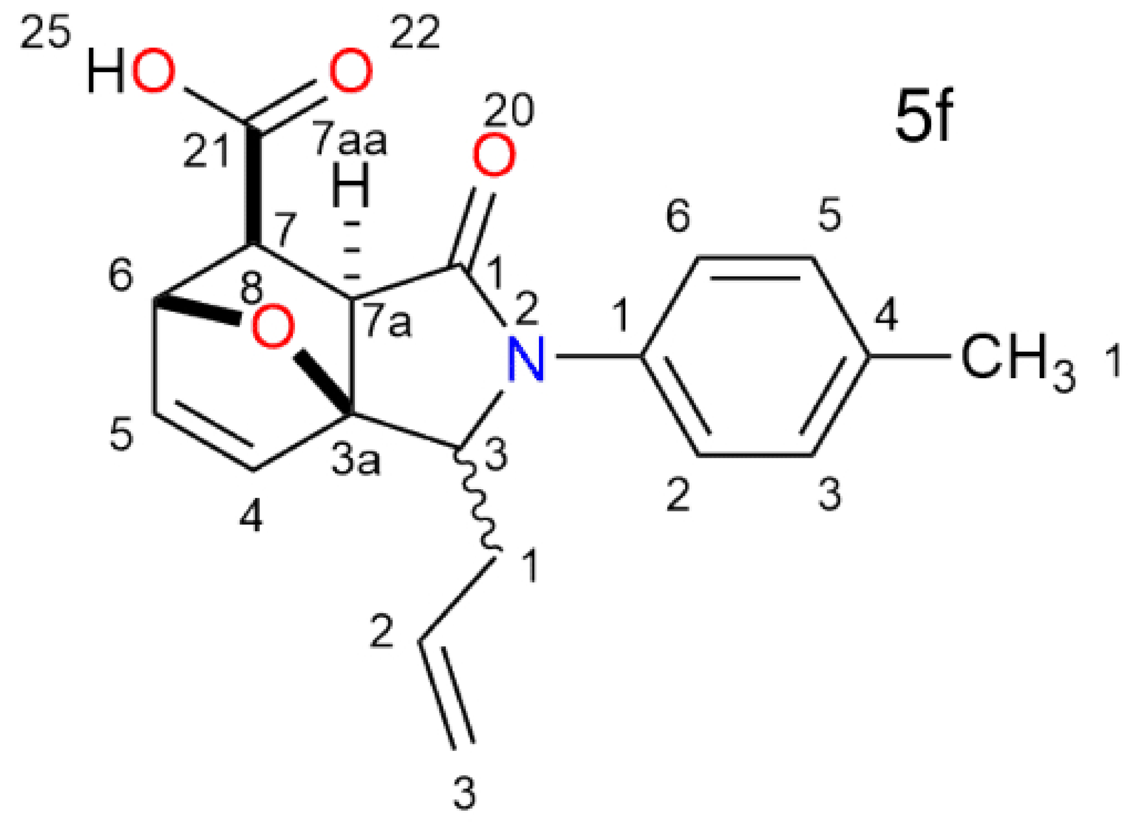

5f

FZ7747-1.esp

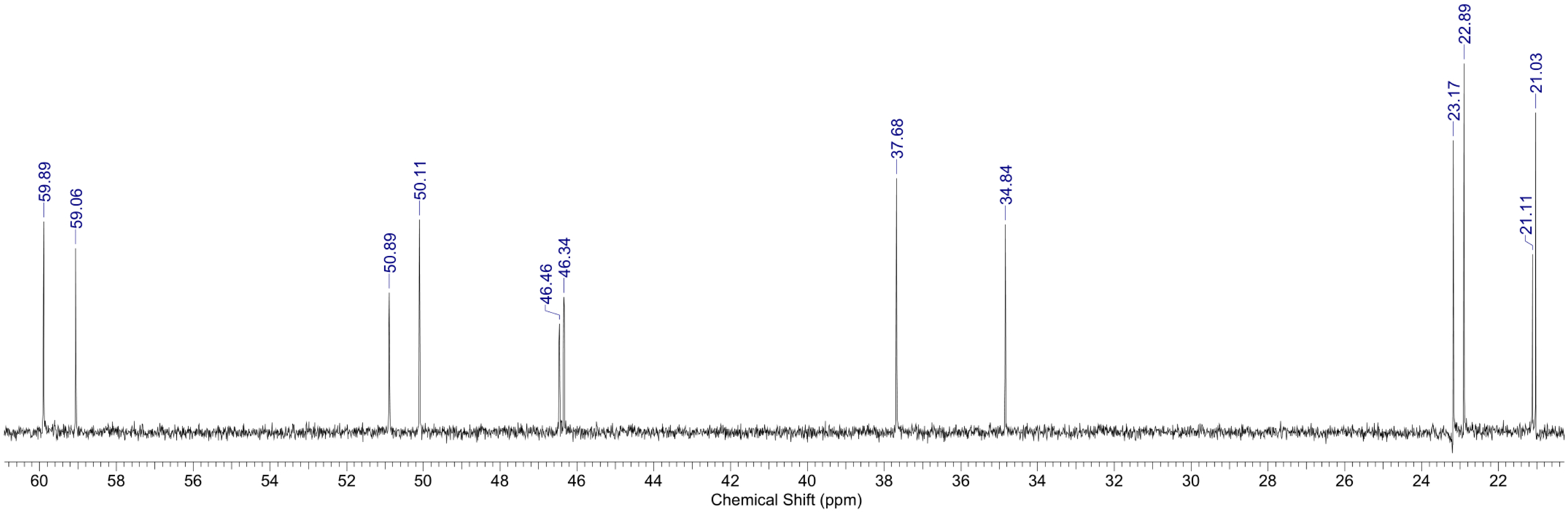

DEPT NMR spectrum for compound 5k

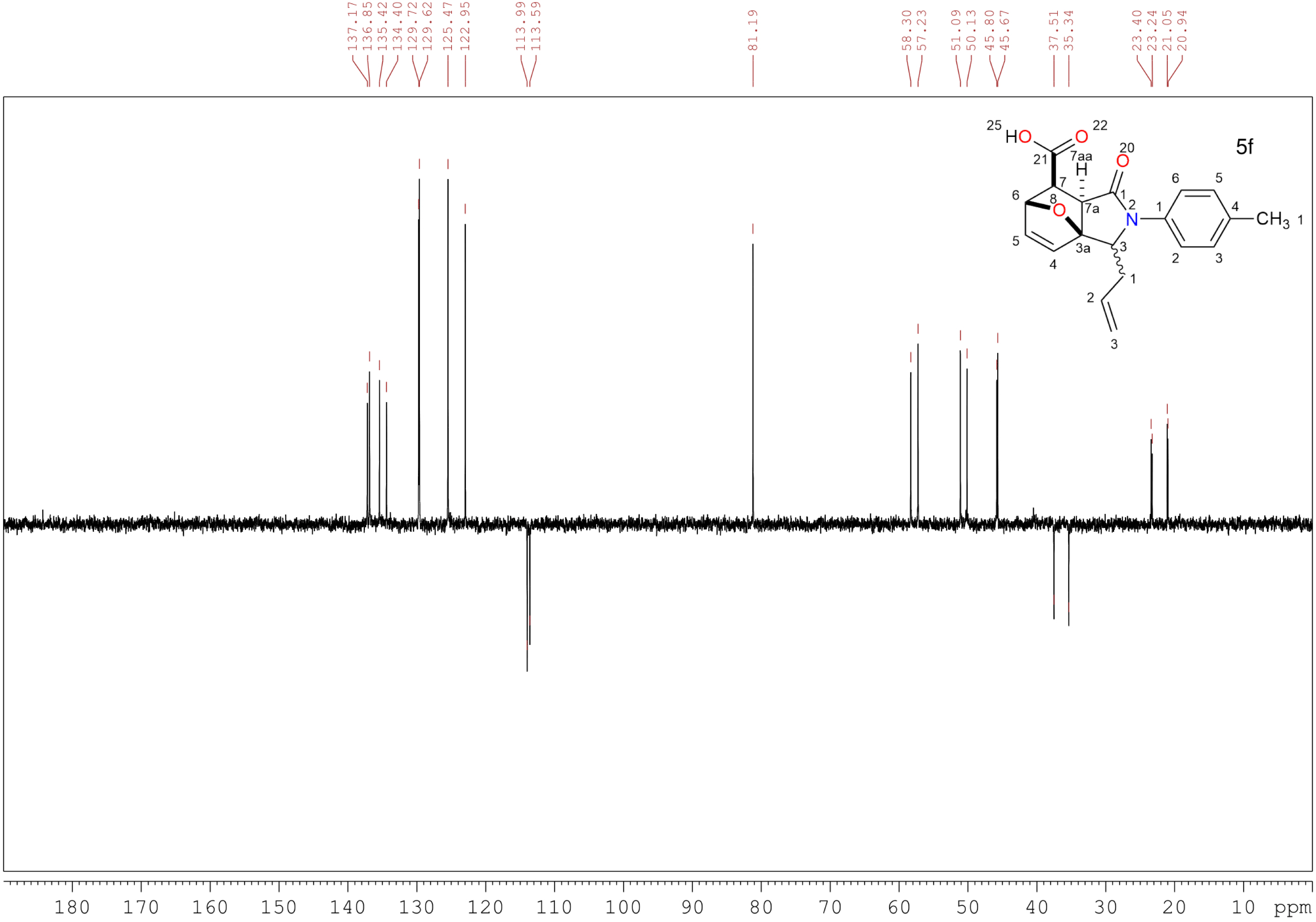

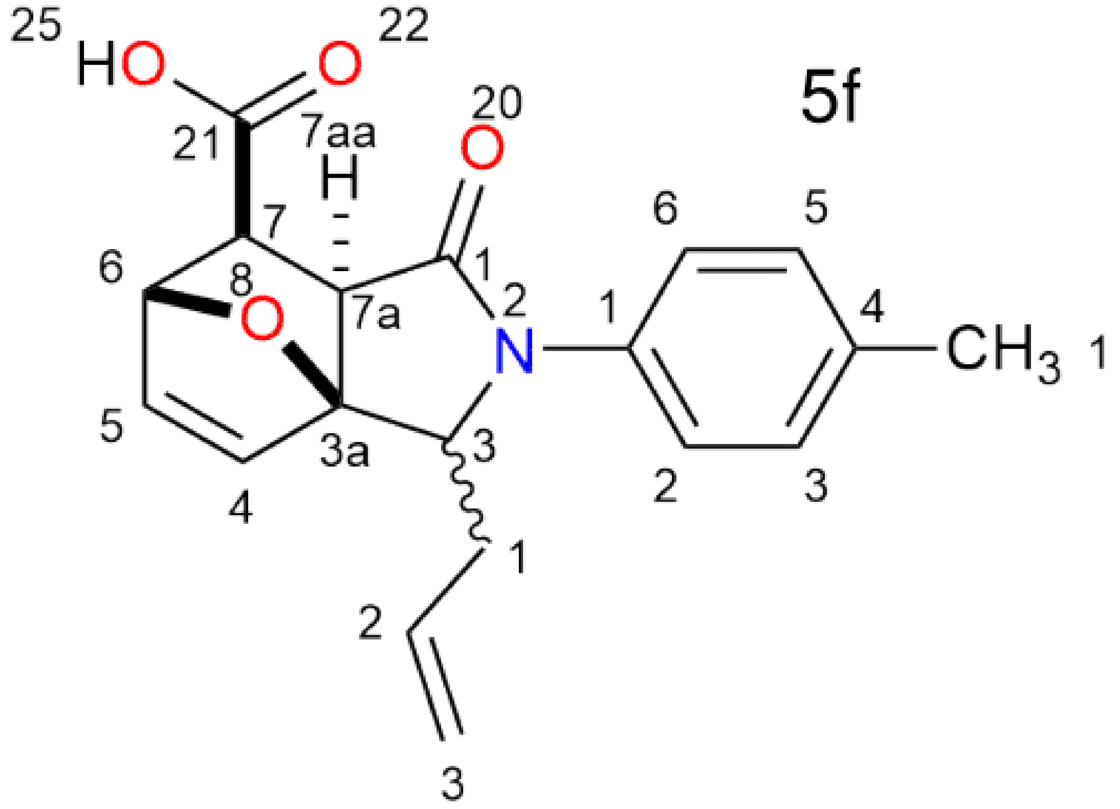

5f

COSY NMR spectrum for compound 5k

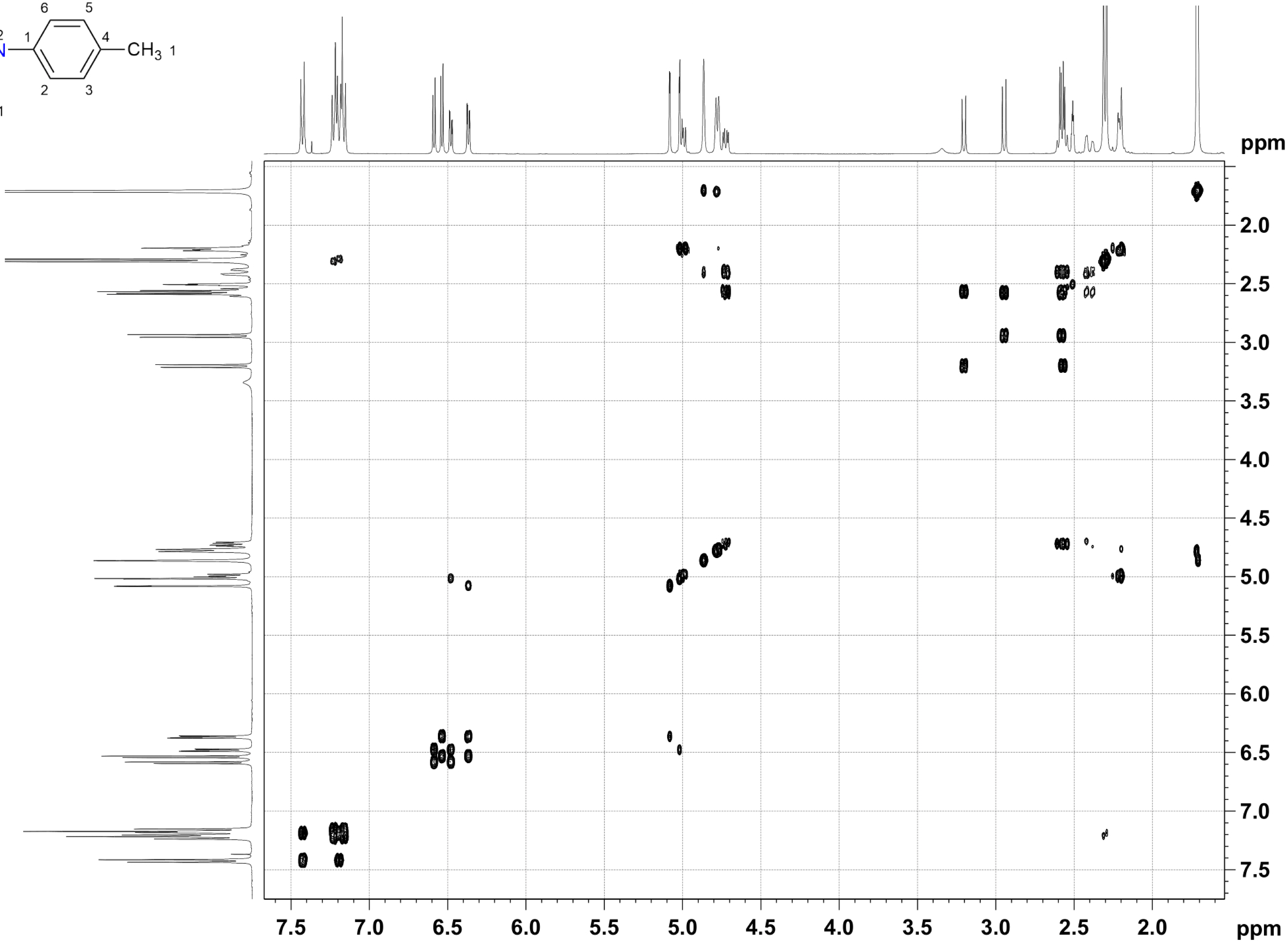

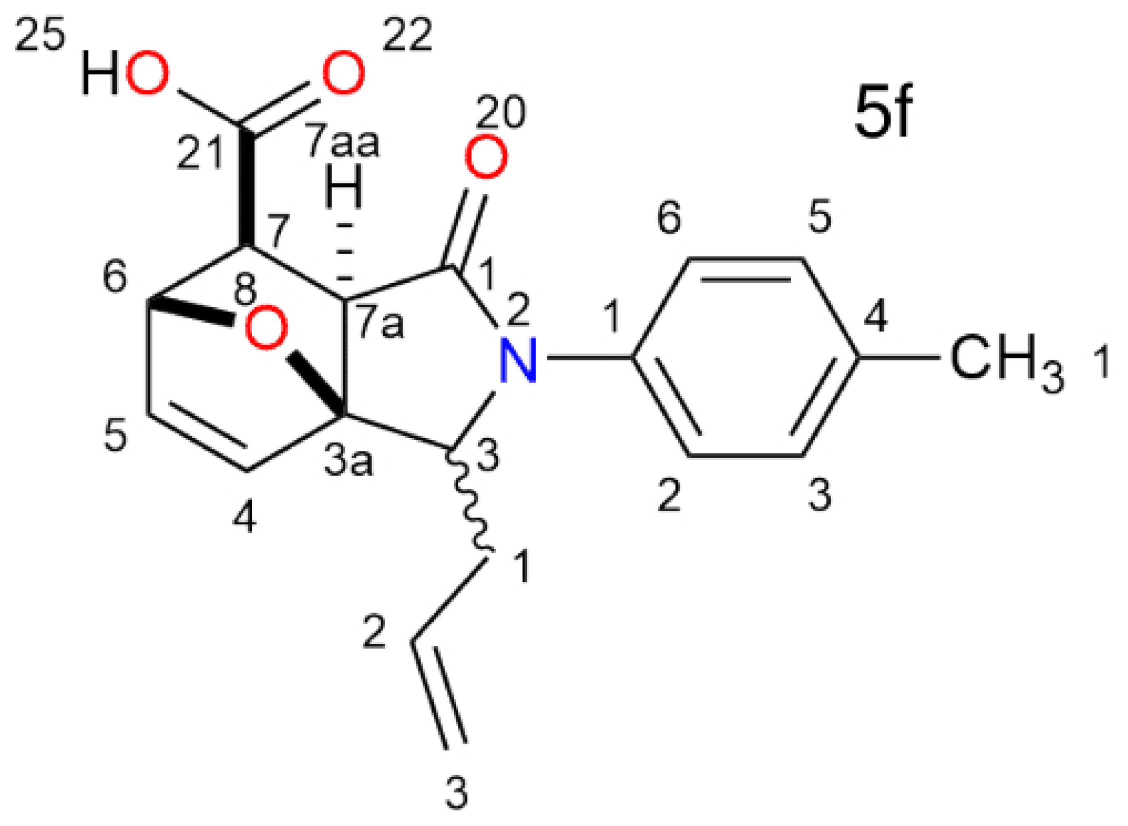

HO
O
O
H
N
CH3
O

5f

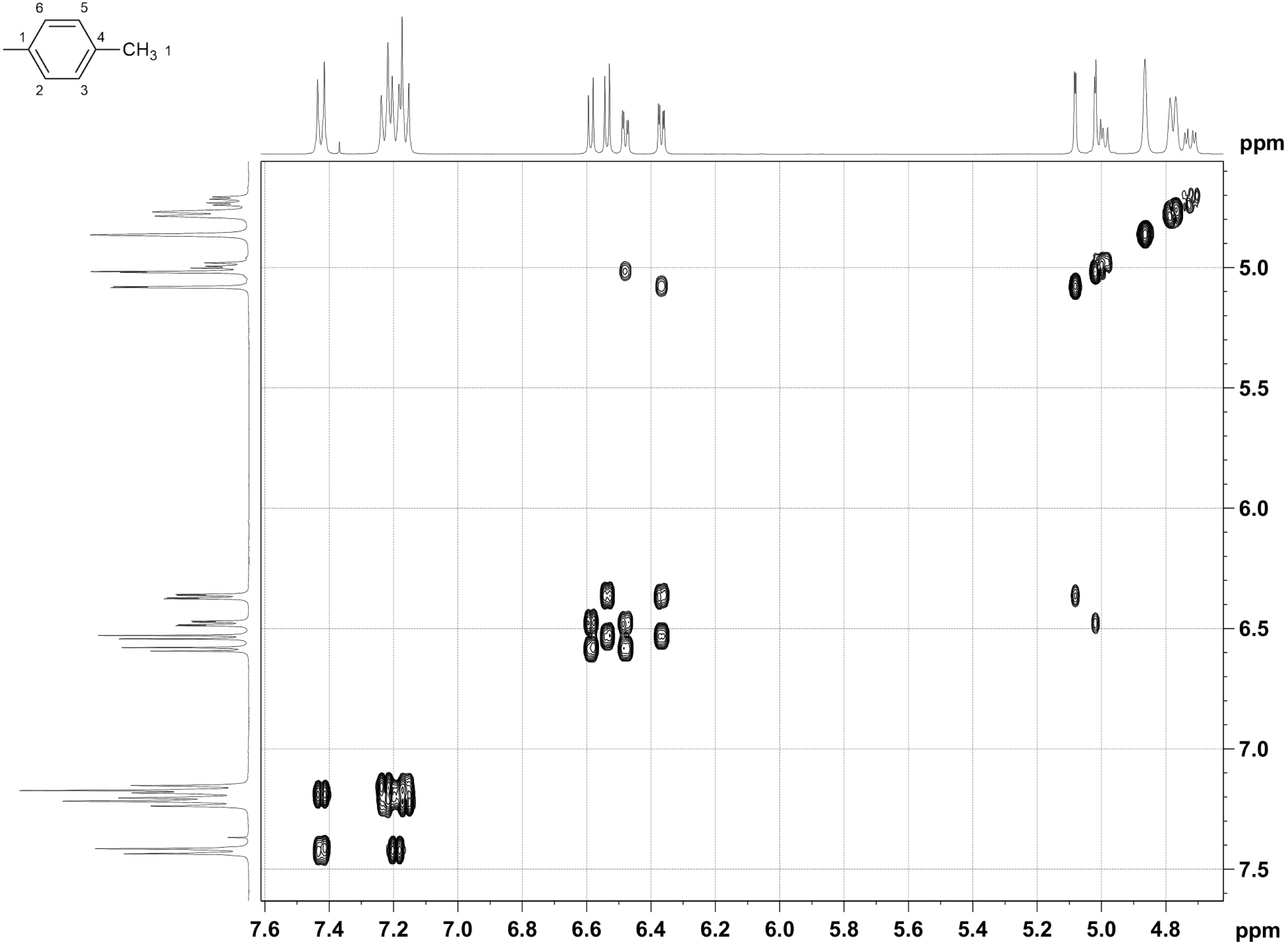

ppm
5.0
5.5
6.0
6.5
7.0
7.5
7.6
7.4
7.2
7.0
6.8
6.6
6.4
6.2
6.0
5.8
5.6
5.4
5.2
5.0
4.8
ppm

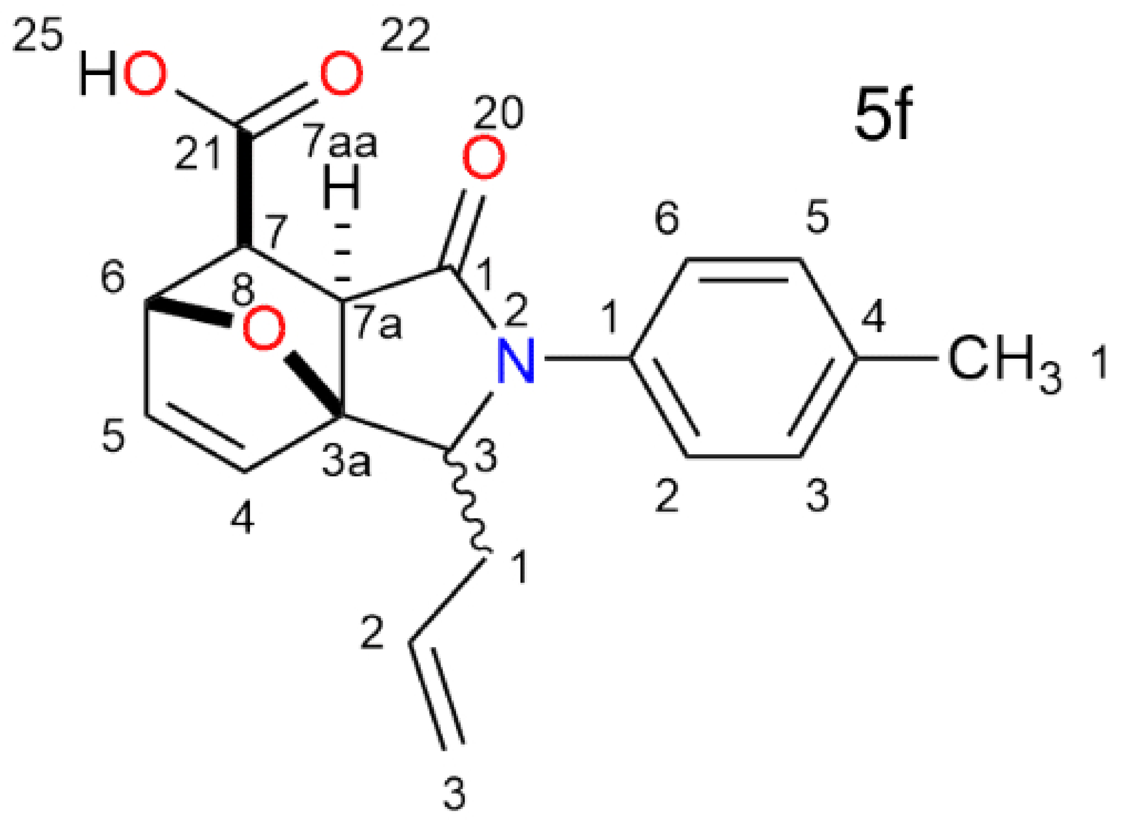
25
HO
O
22
20
O
21
7aa
H
5f
6
5
7
6
8
O
7a
1
2
N
1
4
CH3
1
5
3a
3
2
3
4
1
2
3

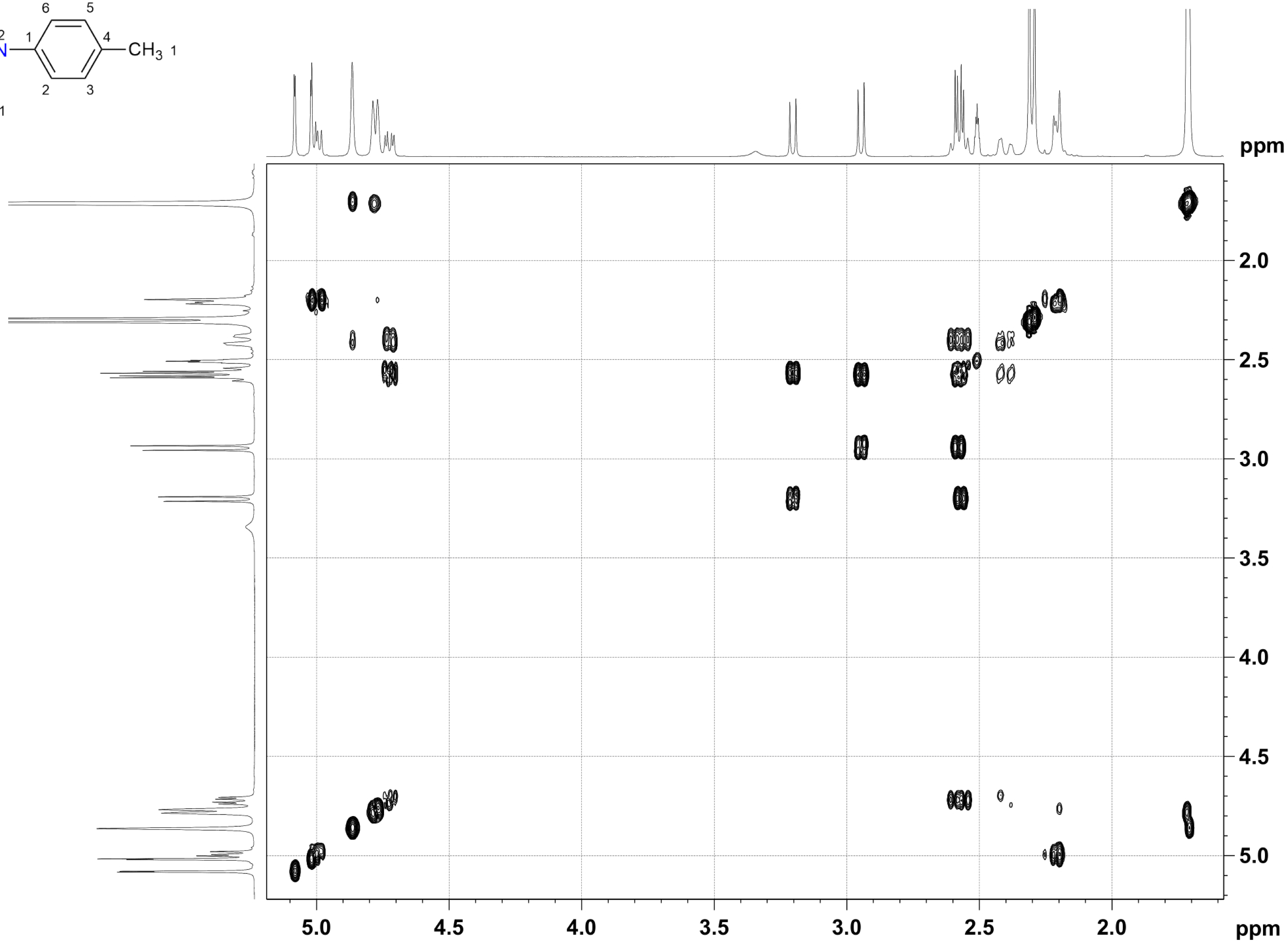
ppm
2.0
2.5
3.0
3.5
4.0
4.5
5.0
5.0
4.5
4.0
3.5
3.0
2.5
2.0
ppm

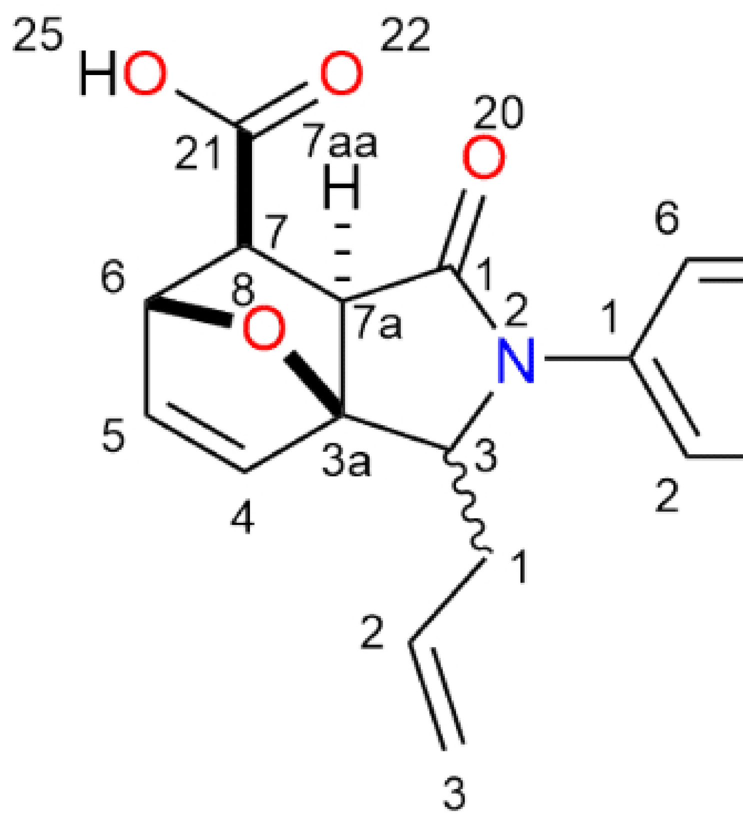

5f

NOESY NMR spectrum for compound 5k

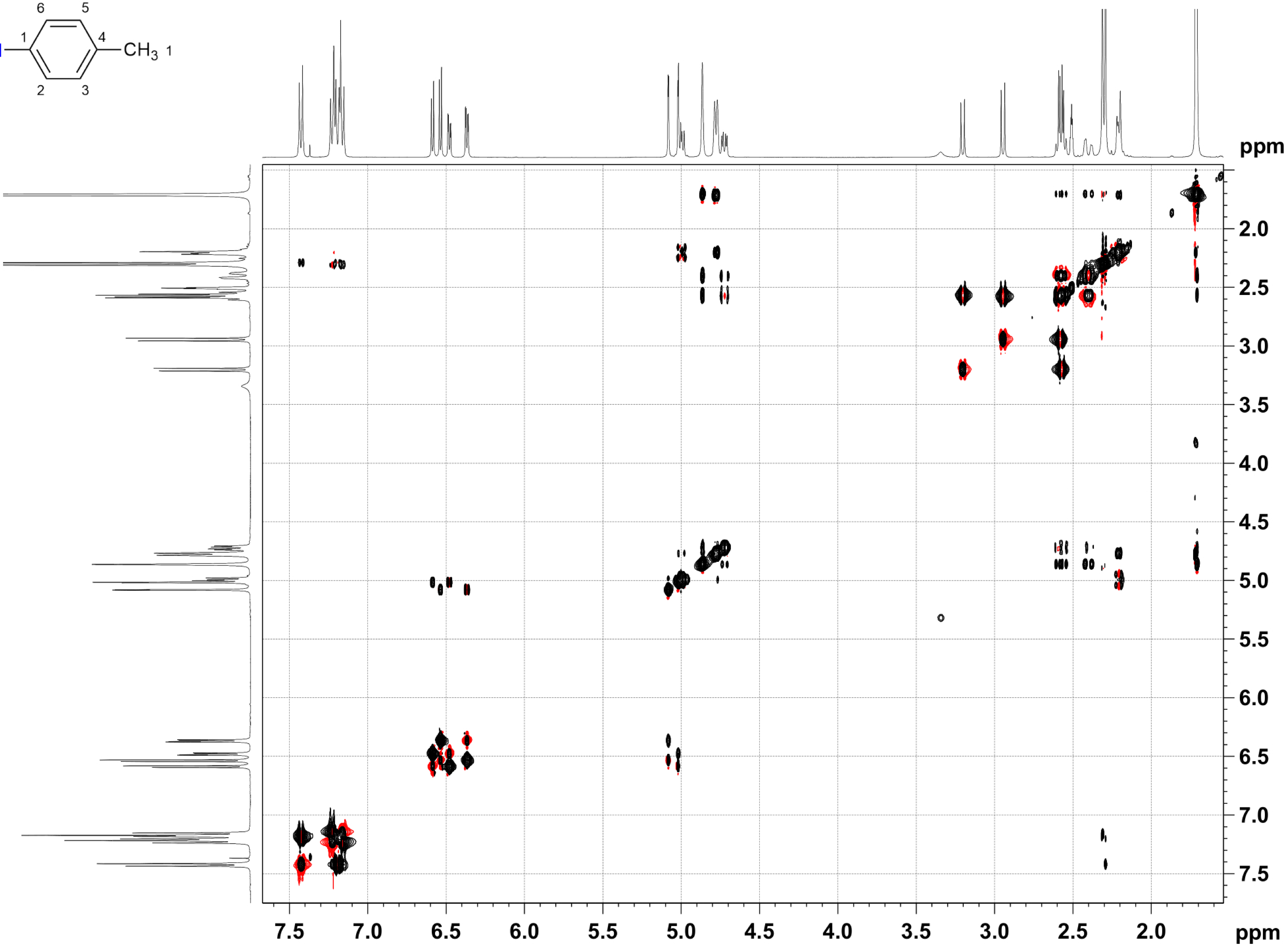

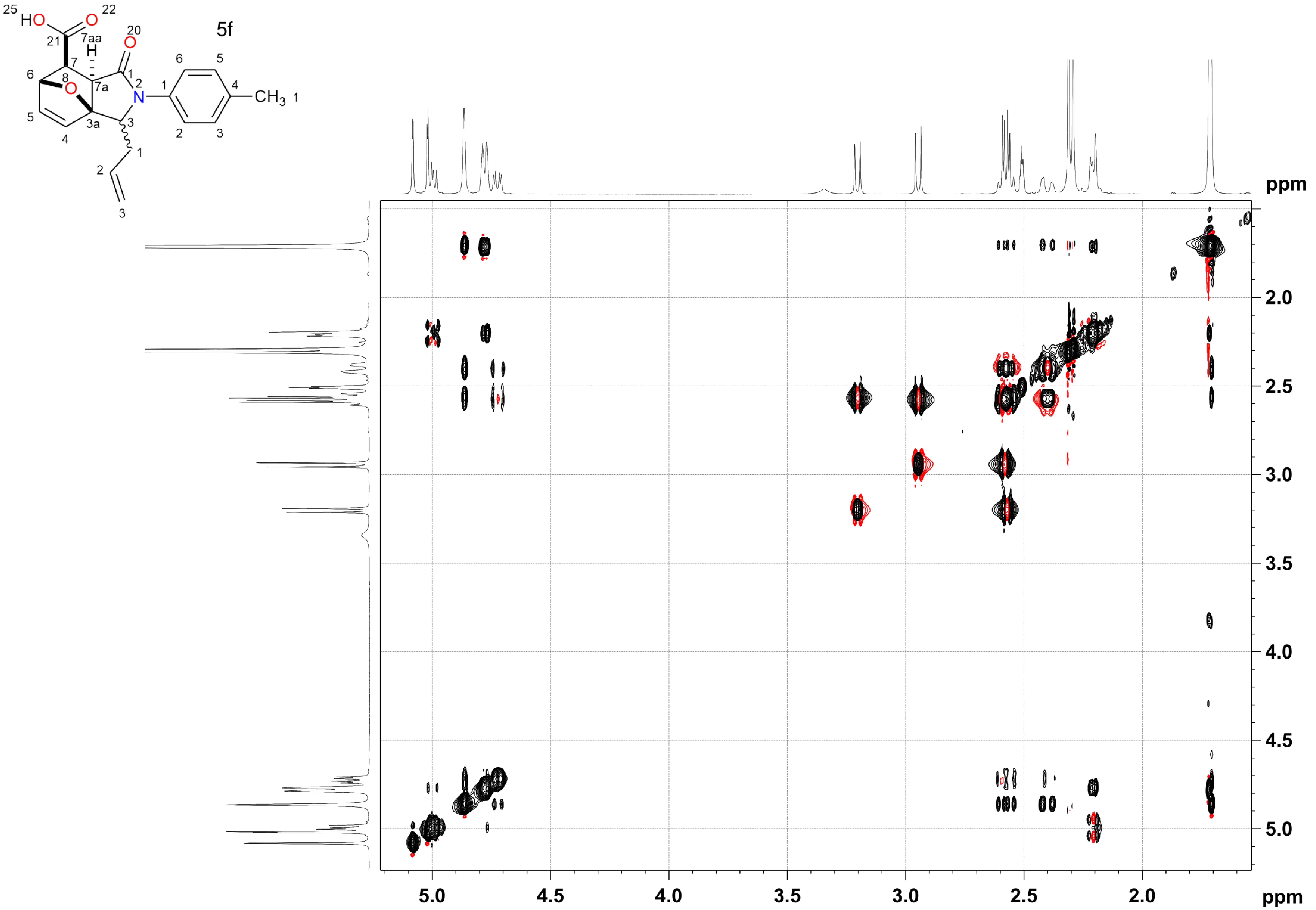

HO
O
21
7aa
H
O
7
1
2
N
CH3
6
8
O
7a
5
3a
3
4
1
2
3
25
22
20
6
5
1
4
1
2
3
ppm
2.0
2.5
3.0
3.5
4.0
4.5
5.0
5.0
4.5
4.0
3.5
3.0
2.5
2.0
ppm

5f

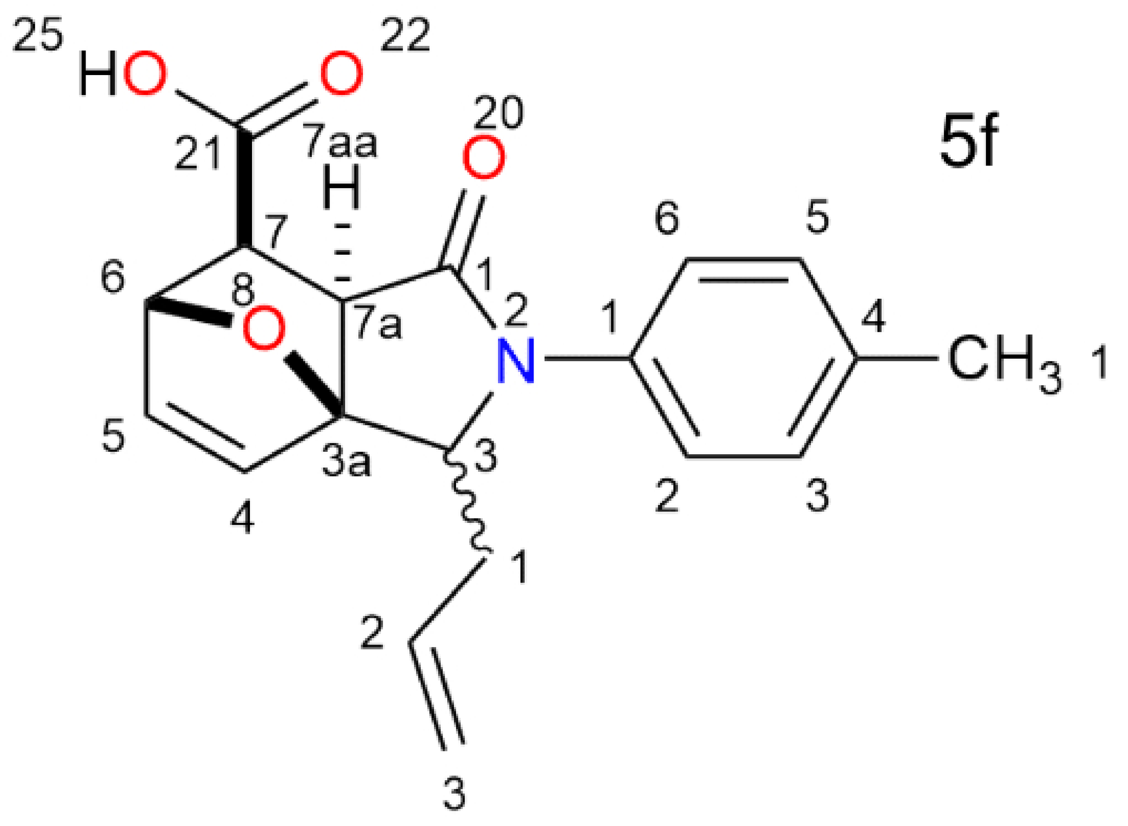

5f

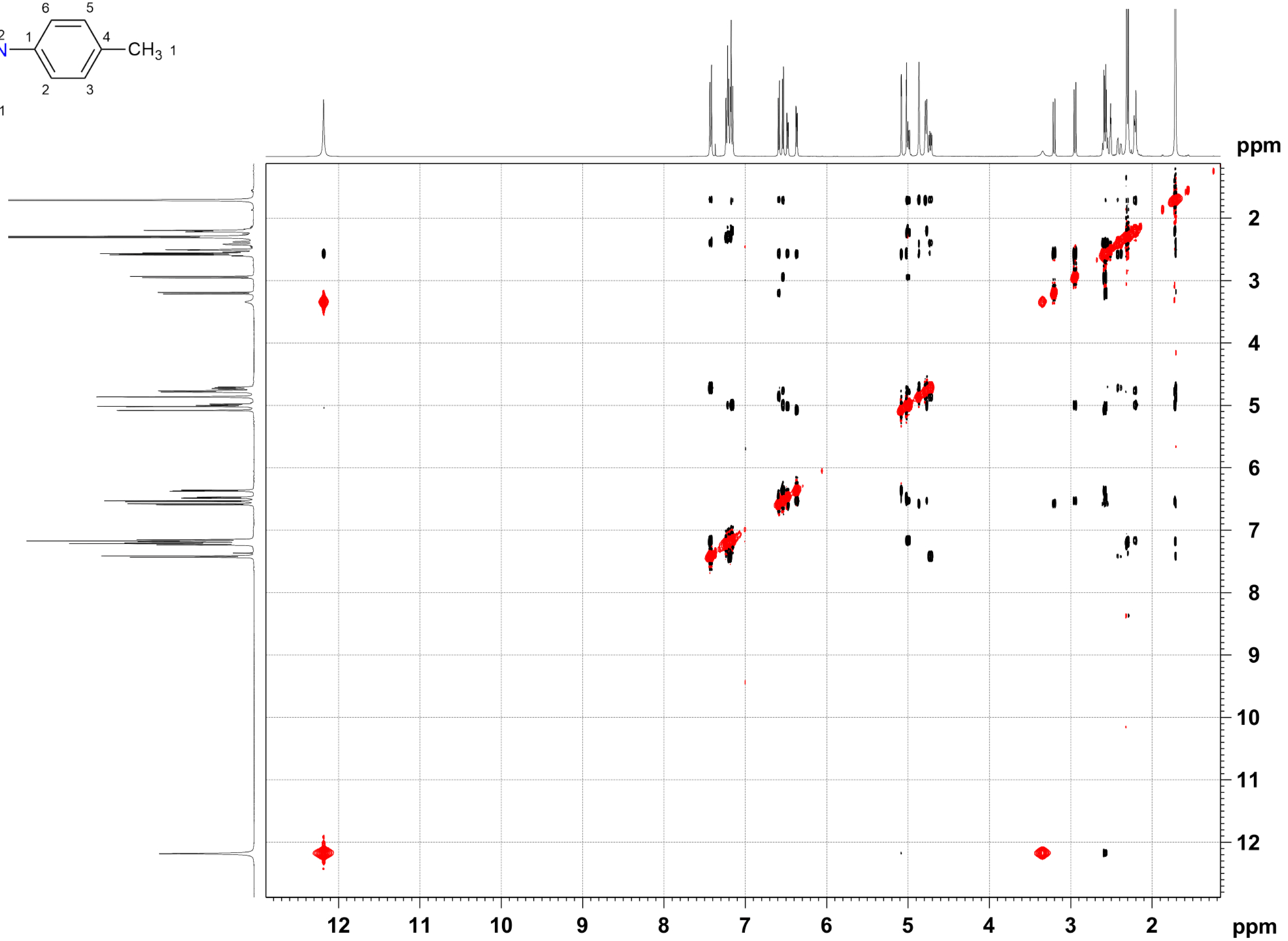

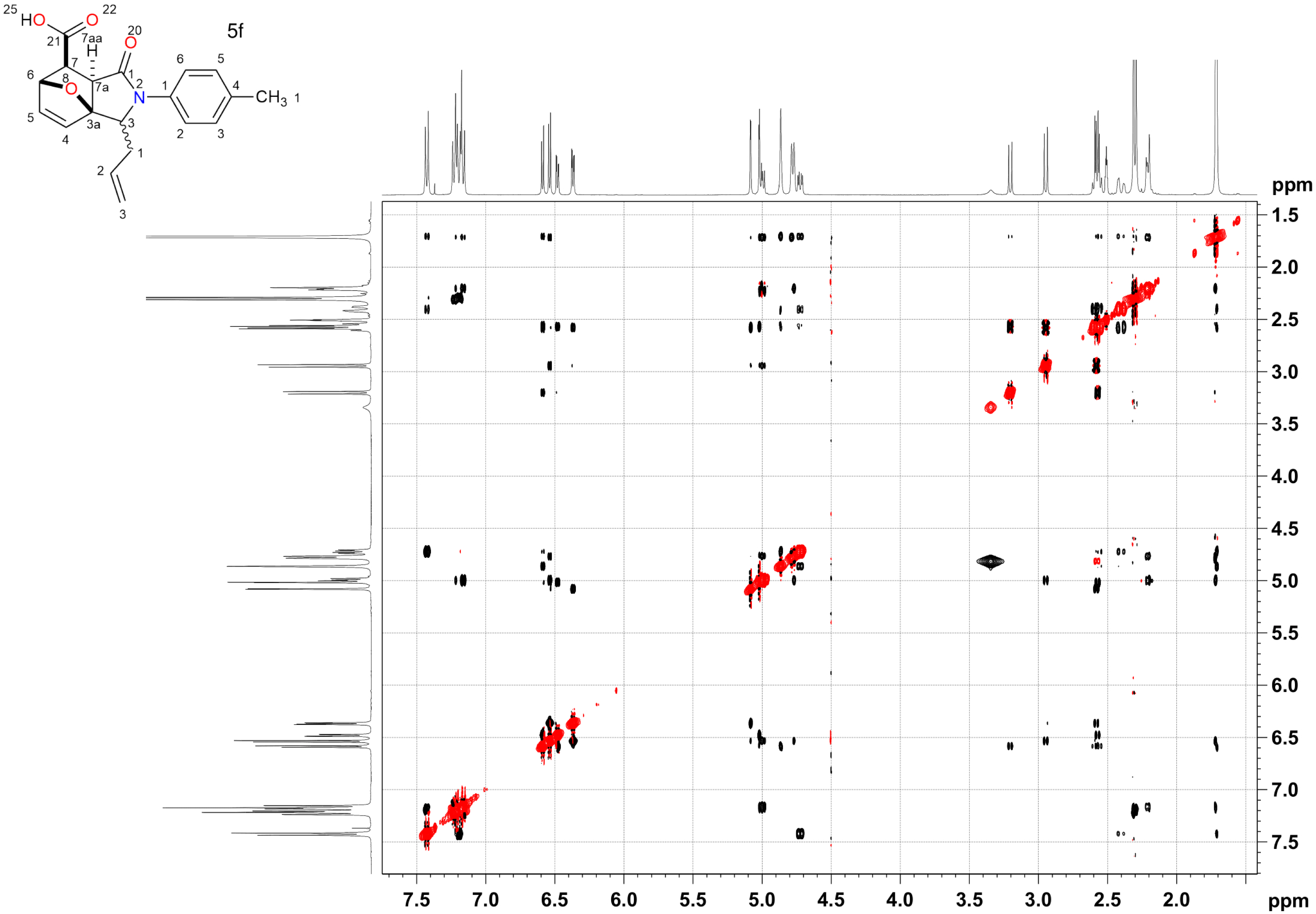

5f
HO
O
O
H
N
CH3
ppm
1.5
2.0
2.5
3.0
3.5
4.0
4.5
5.0
5.5
6.0
6.5
7.0
7.5
ppm

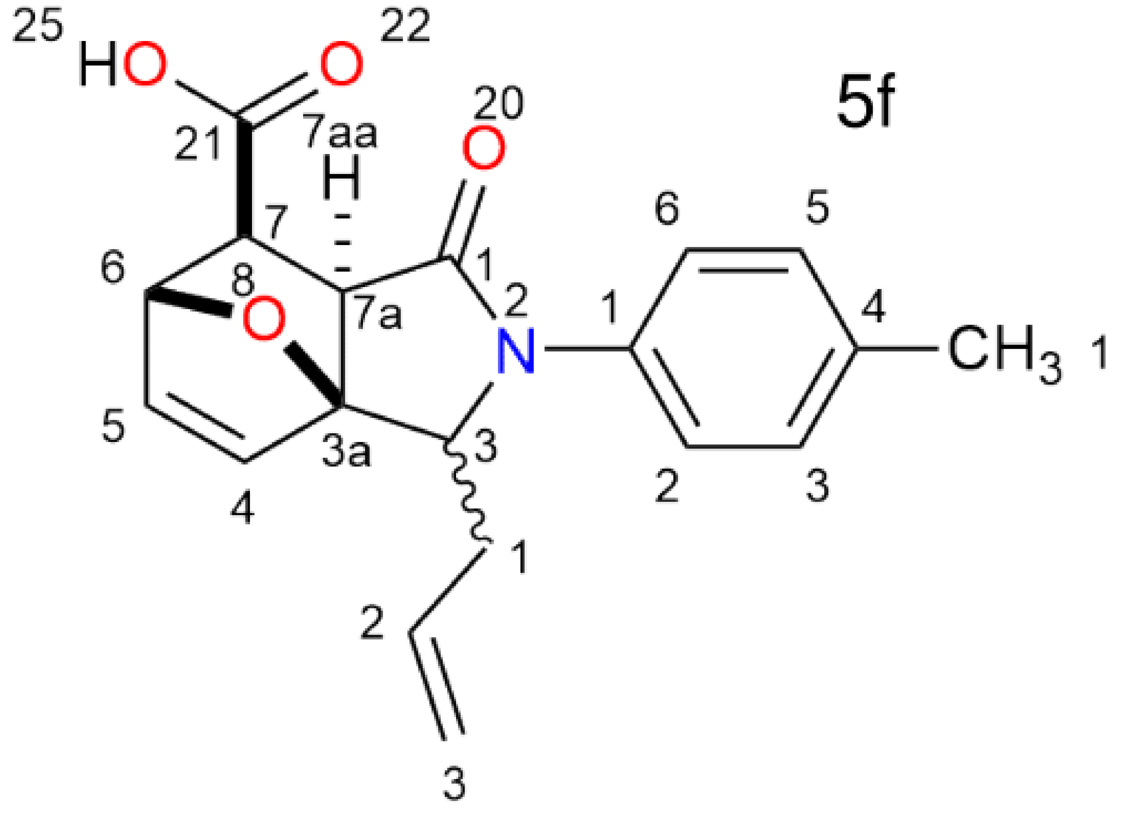

5f

HSQC NMR spectrum for compound 5k

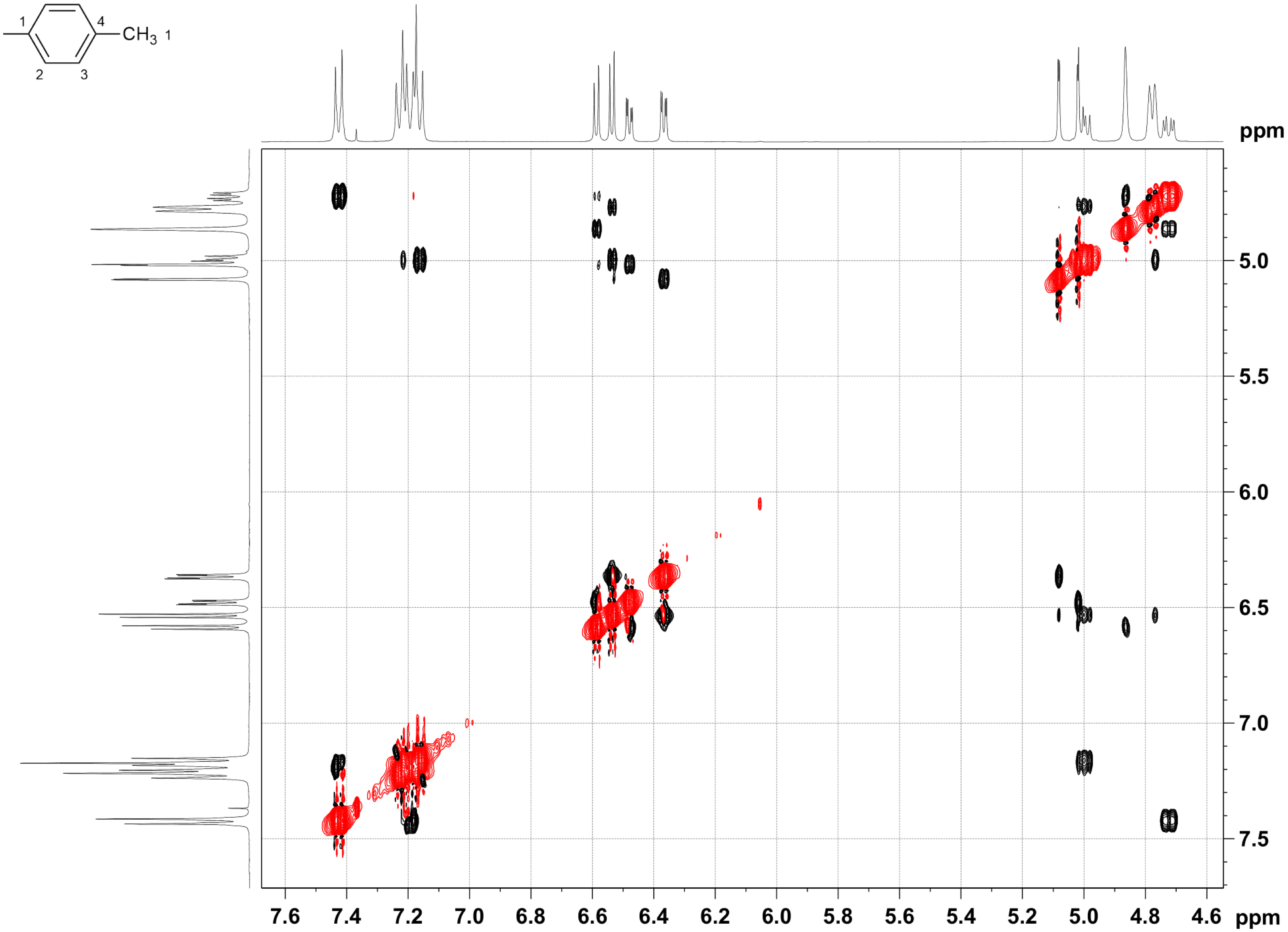

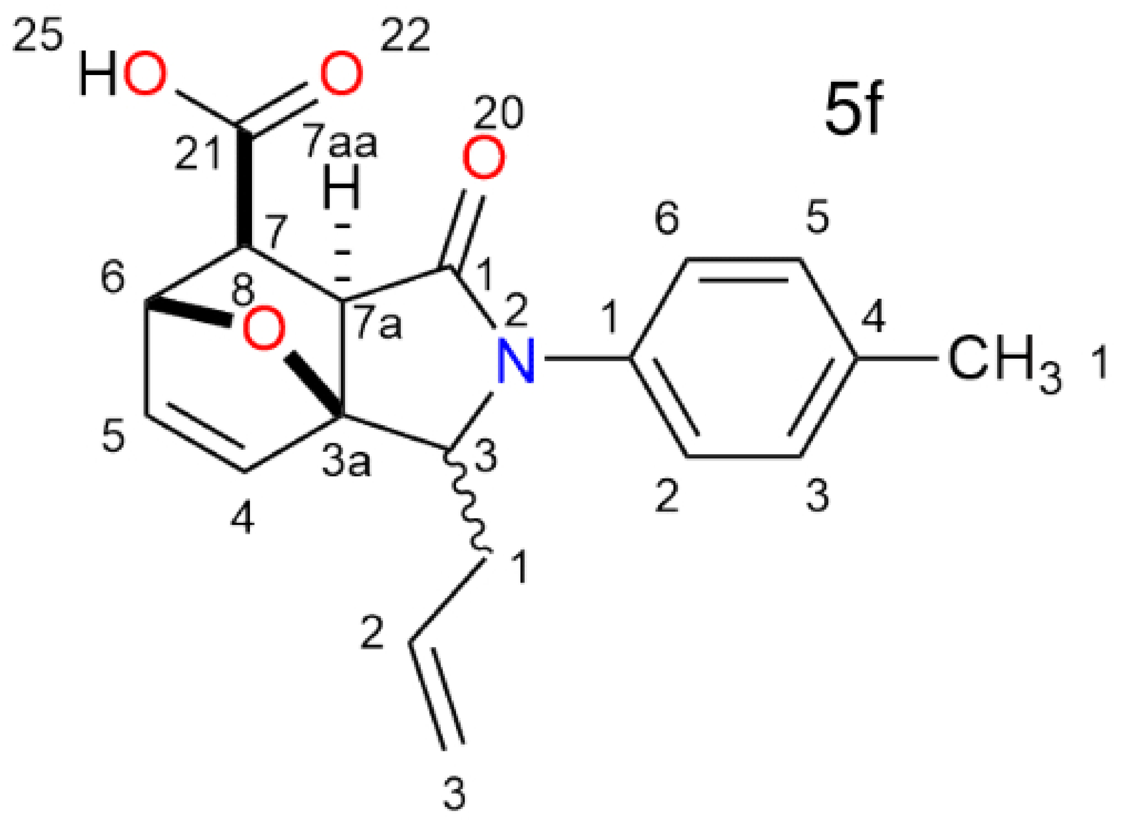
HO
O
N
CH3
H
5f

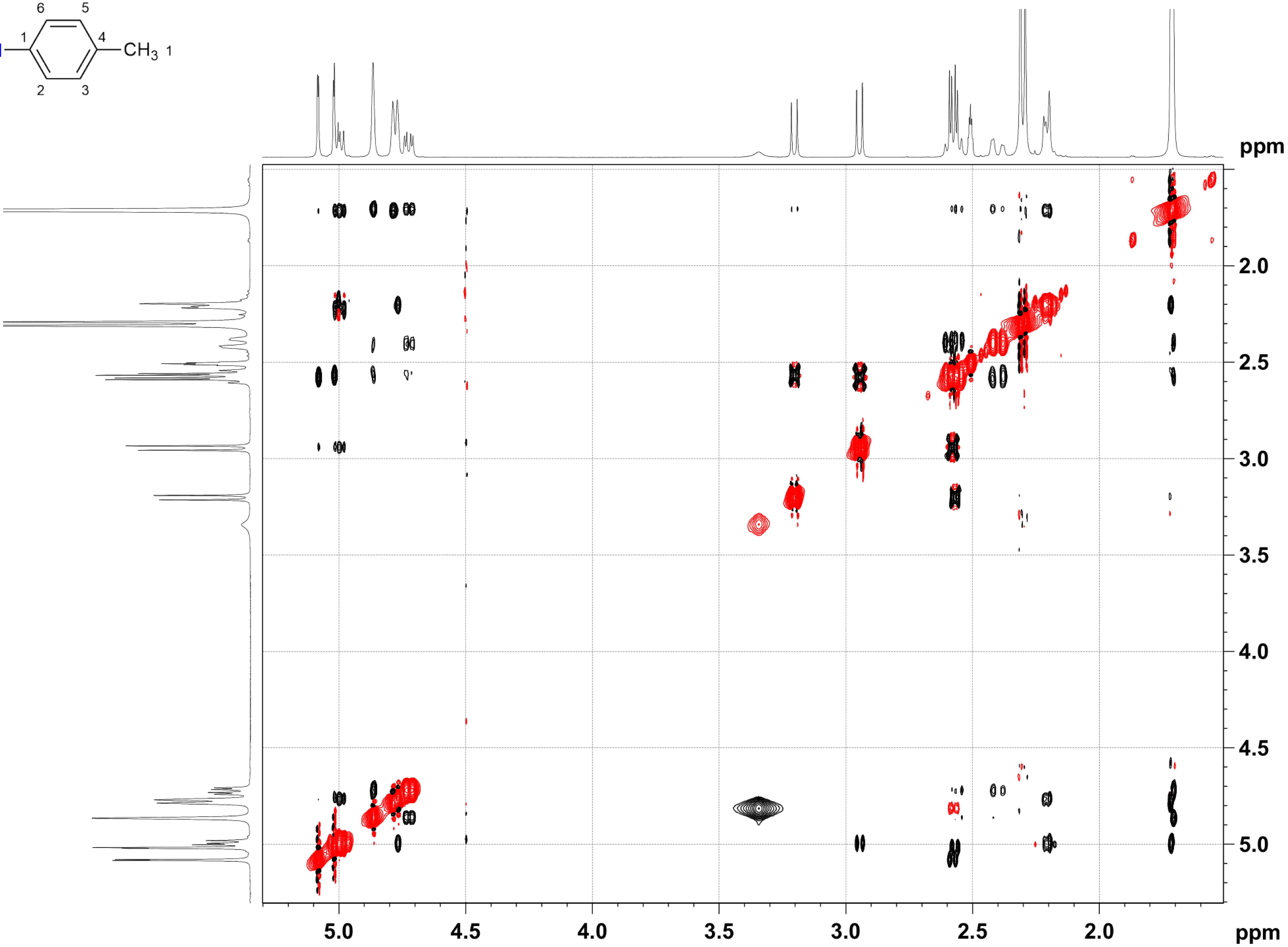
ppm
5.0
4.5
4.0
3.5
3.0
2.5
2.0
ppm
2.0
2.5
3.0
3.5
4.0
4.5
5.0

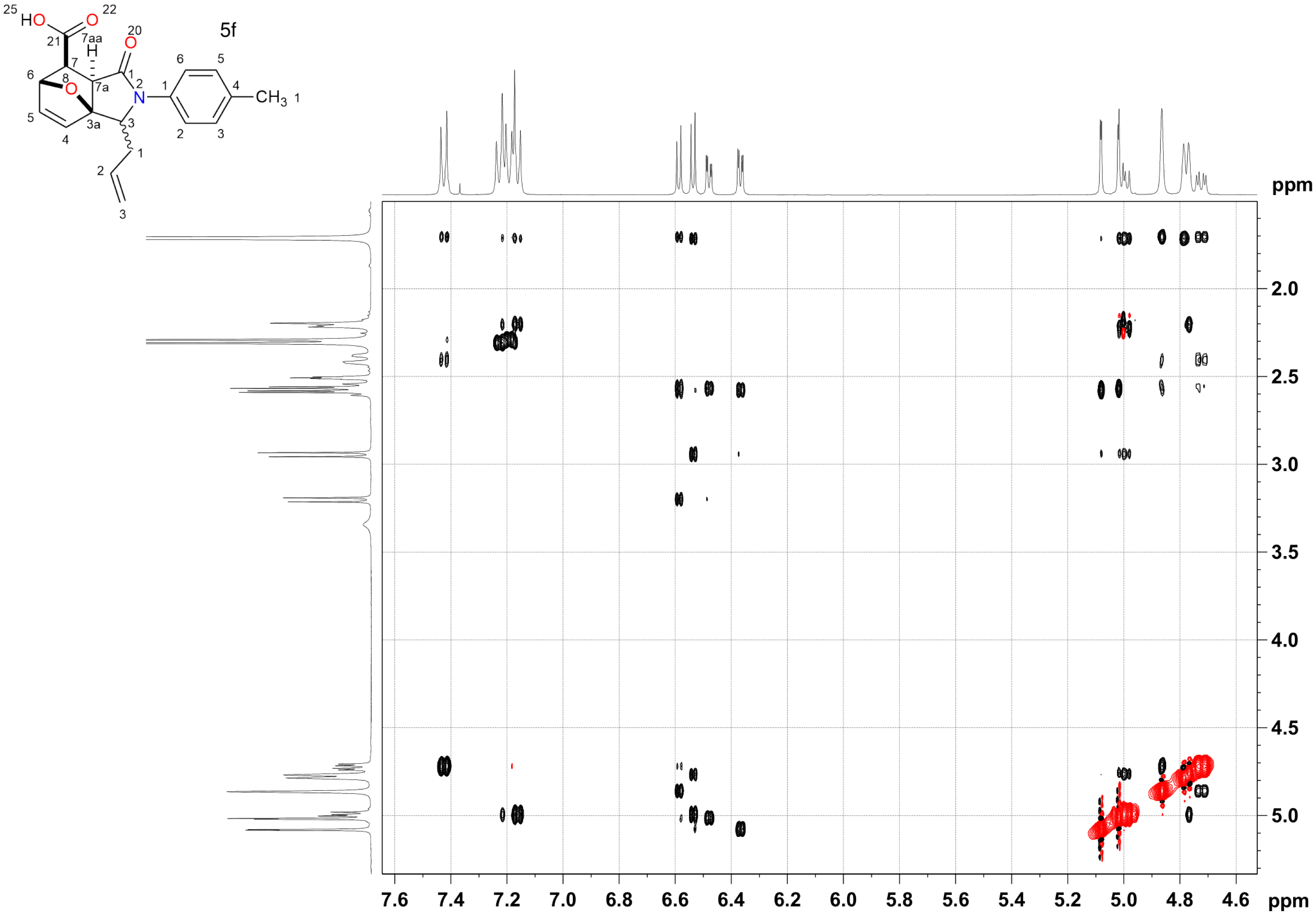

5f
HO
O
O
N
O
CH3
ppm
7.6
7.4
7.2
7.0
6.8
6.6
6.4
6.2
6.0
5.8
5.6
5.4
5.2
5.0
4.8
4.6
2.0
2.5
3.0
3.5
4.0
4.5
5.0

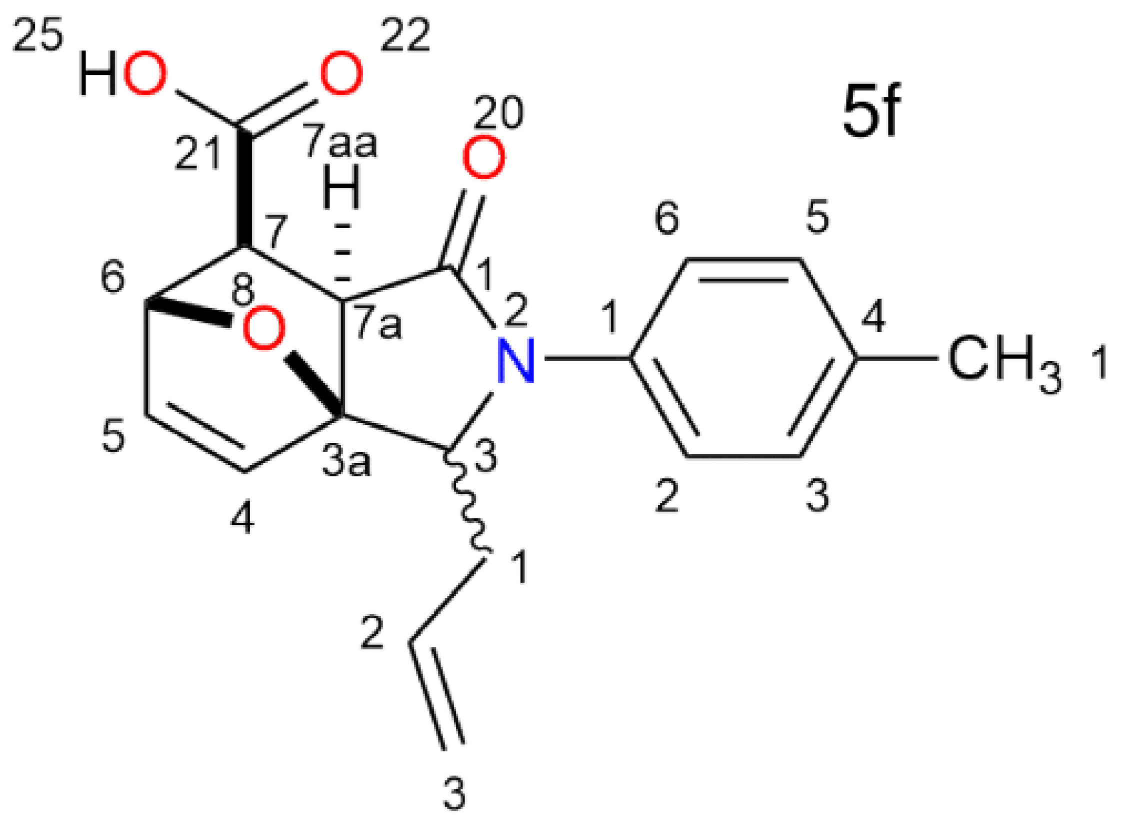
25
HO
O
22
21
7aa
H
20
O
7
1
6
8
O
7a
2
N
5
3a
3
4
1
2
3
6
5
4
CH3
1
2
3
1

5f

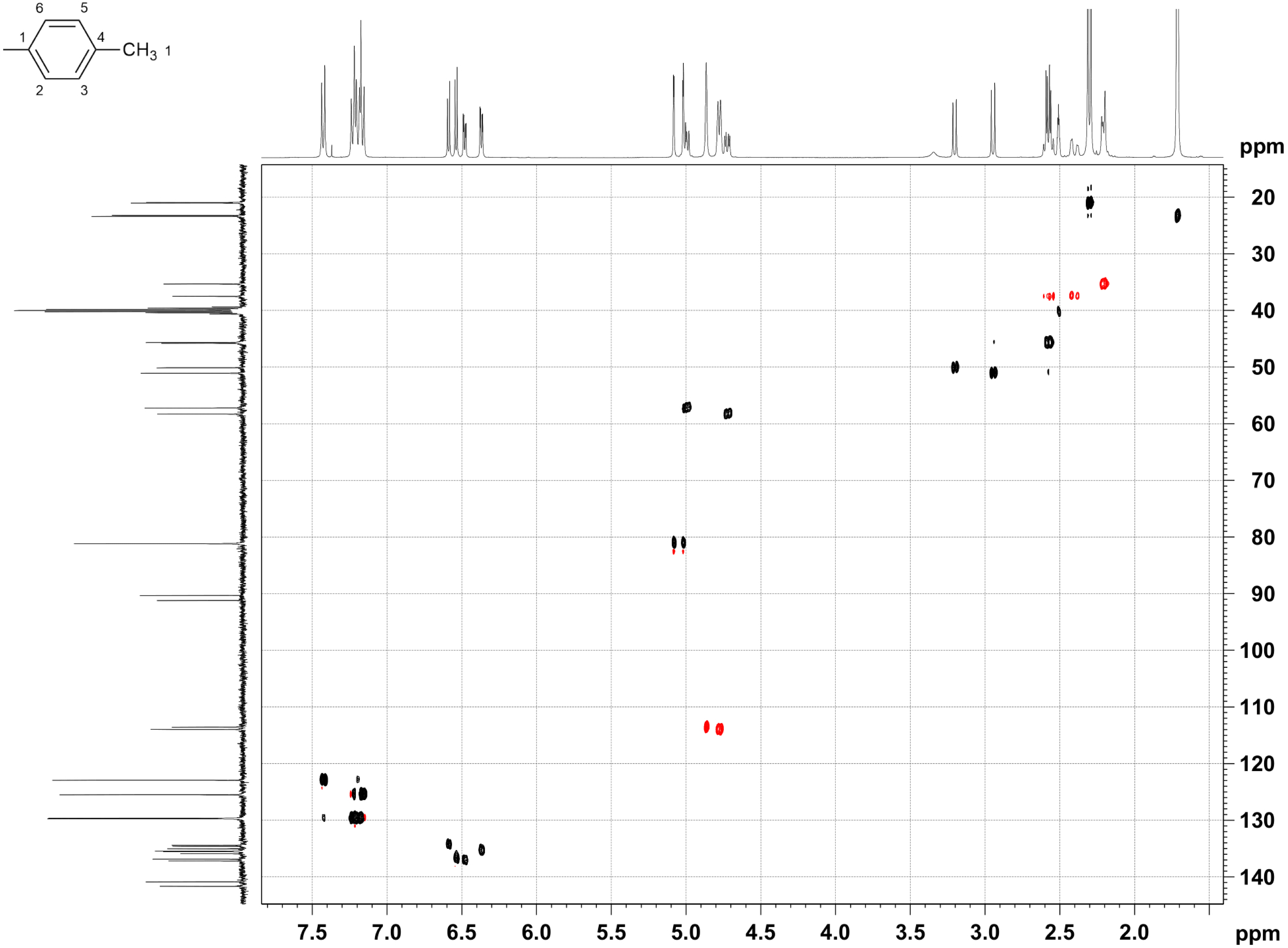
ppm
20
30
40
50
60
70
80
90
100
110
120
130
140
7.5
7.0
6.5
6.0
5.5
5.0
4.5
4.0
3.5
3.0
2.5
2.0
ppm

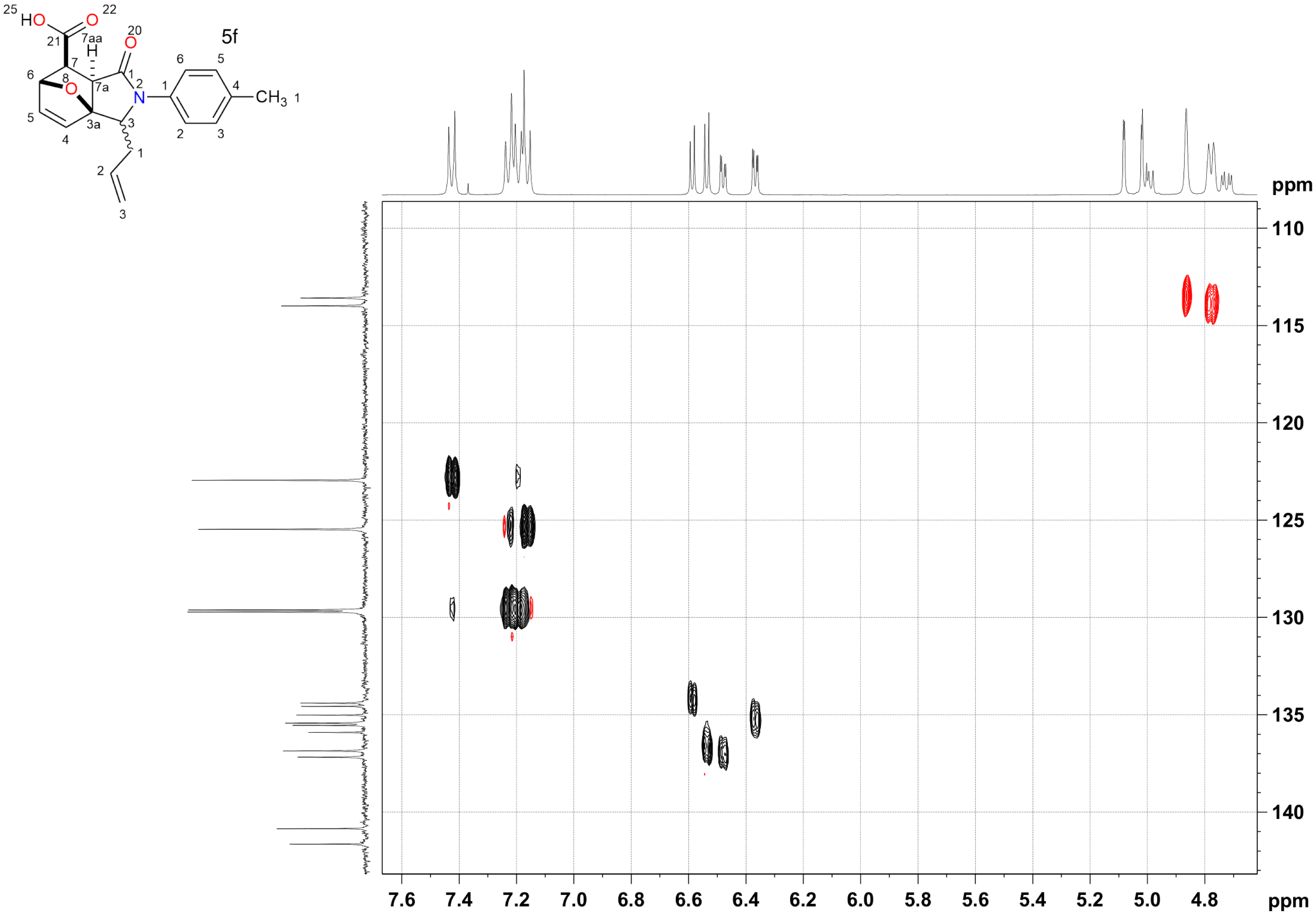

5f
ppm
110
115
120
125
130
135
140
7.6
7.4
7.2
7.0
6.8
6.6
6.4
6.2
6.0
5.8
5.6
5.4
5.2
5.0
4.8
ppm

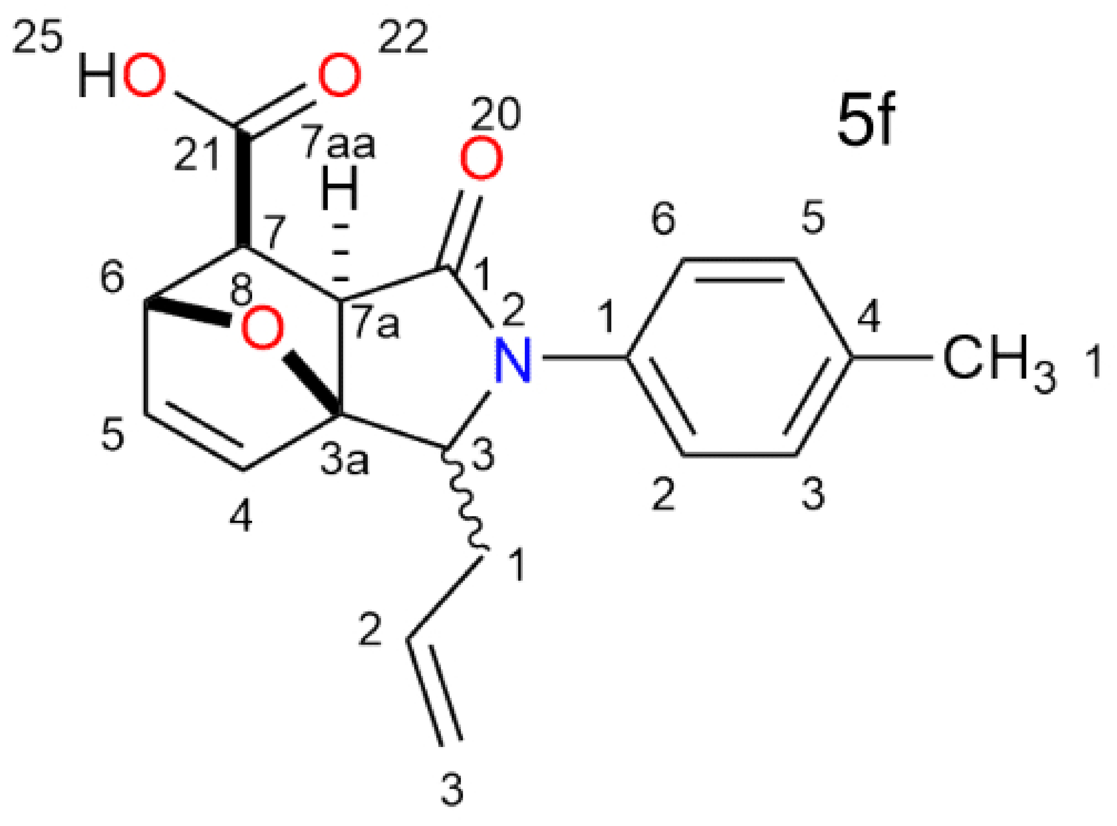

25
HO
O
22
20
O
7aa
H
21
7
6
8
O
7a
1
2
N
1
6
5
4
CH3
1
5
4
3a
3
2
3
1
2
3

5f

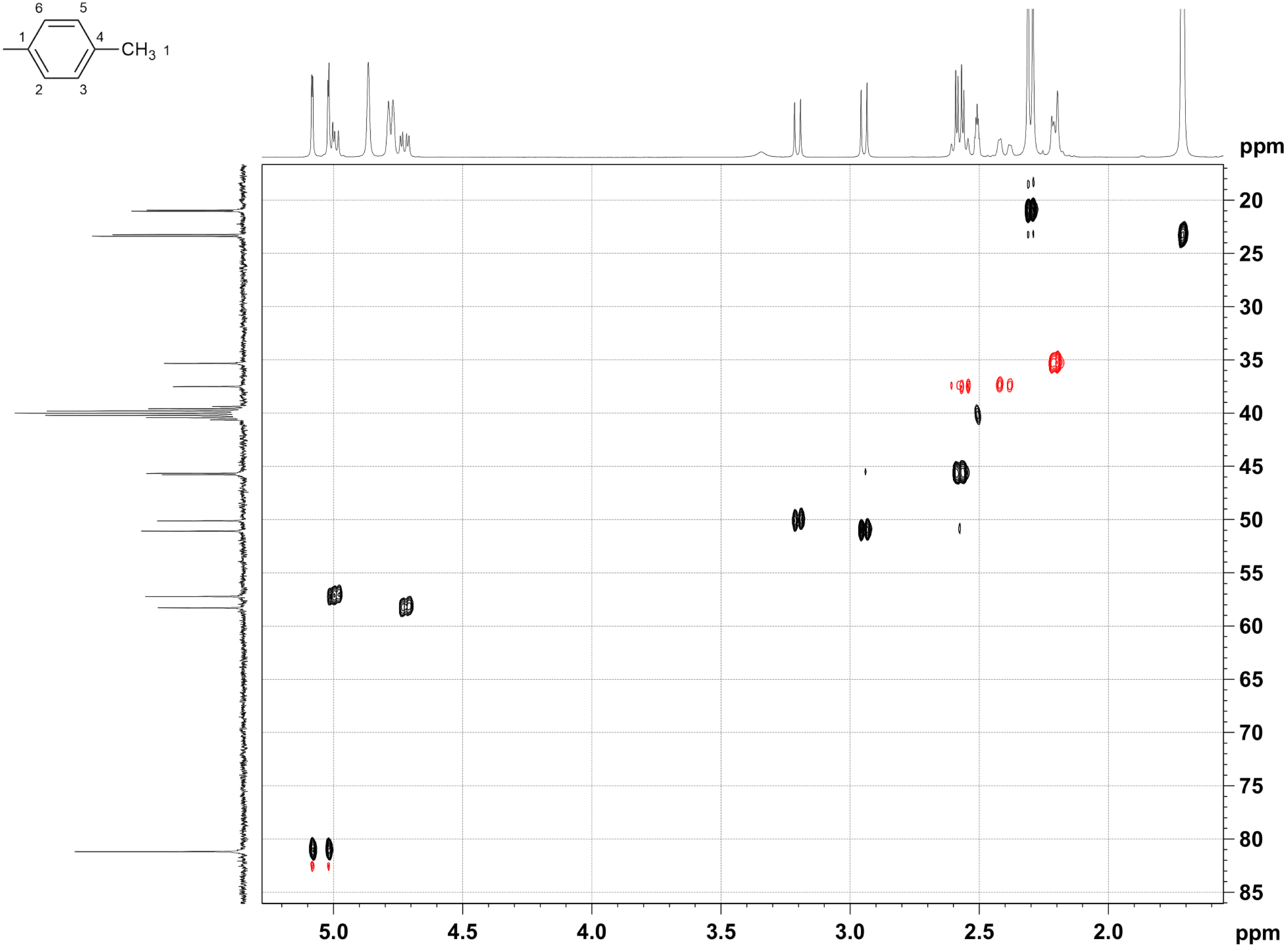

ppm
20
25
30
35
40
45
50
55
60
65
70
75
80
85
5.0
4.5
4.0
3.5
3.0
2.5
2.0
ppm

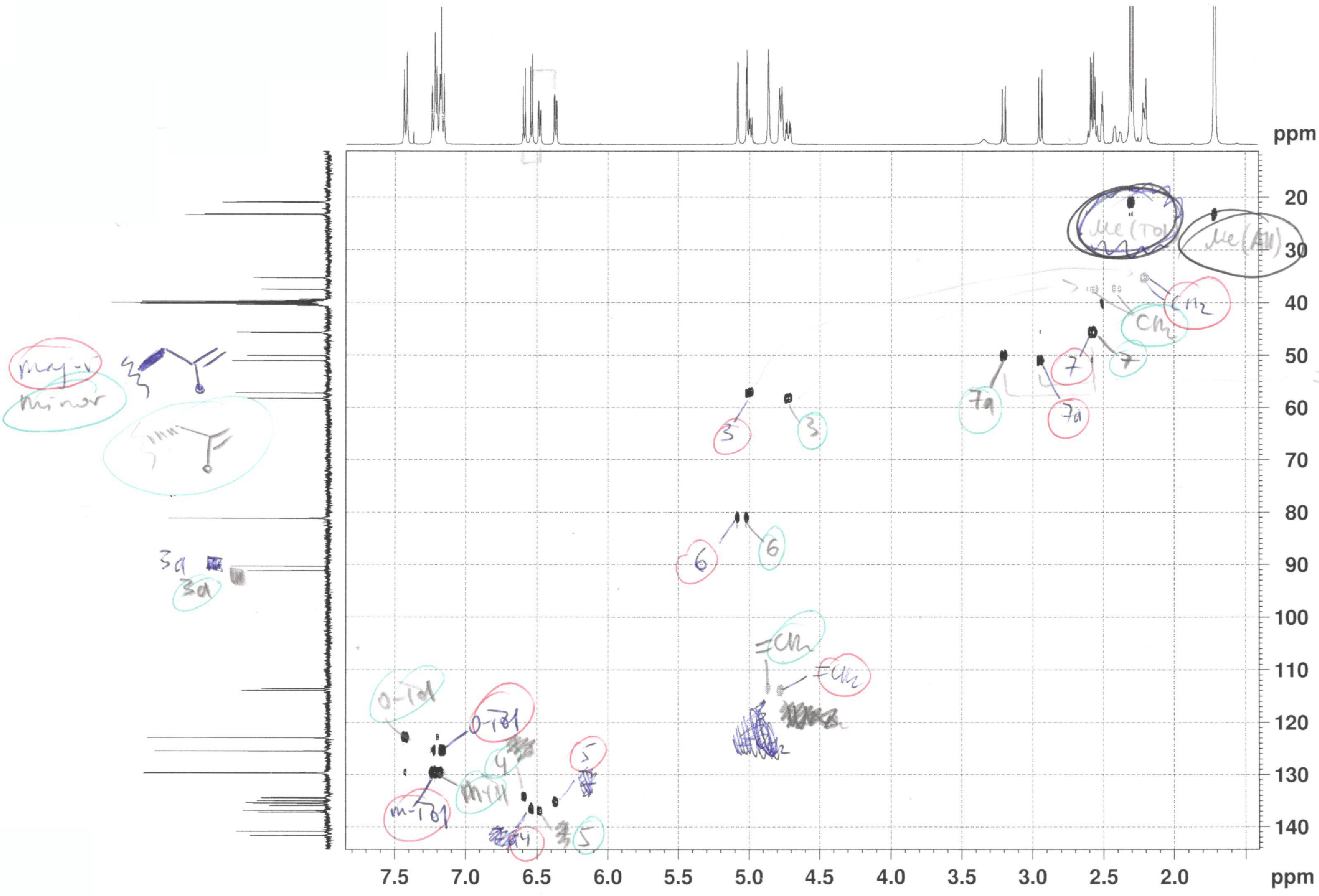

ppm
7.5
7.0
6.5
6.0
5.5
5.0
4.5
4.0
3.5
3.0
2.5
2.0
20
30
40
50
60
70
80
90
100
110
120
130
140
major
minor
3a
Me (Tol)
CH2
7
7a
3
6
=CH2
o-Tol
m-Tol
4
5

NMR spectrum for compound 5m

Acquisition Time (sec)	1.9818	***Comment***	single_pulse	***Date***	29 May 1990 08:13:49				
Date Stamp	08 Aug 2019 14:17:16			***File Name***	C:\Users\Fedor\Desktop\07.08.19\FZ7646-3.jdf			***Frequency (MHz)***	600.17
Nucleus	1H	***Number of Transients***	8	***Origin***	ECA 600	***Original Points Count***	32768	***Owner***	delta
Points Count	32768	***Pulse Sequence***	single_pulse.ex2			***Receiver Gain***	36.00	***Solvent***	CHLOROFORM-d
Spectrum Offset (Hz)	5409.6182	***Sweep Width (Hz)***	16534.39	***Temperature (degree C)***	23.100				

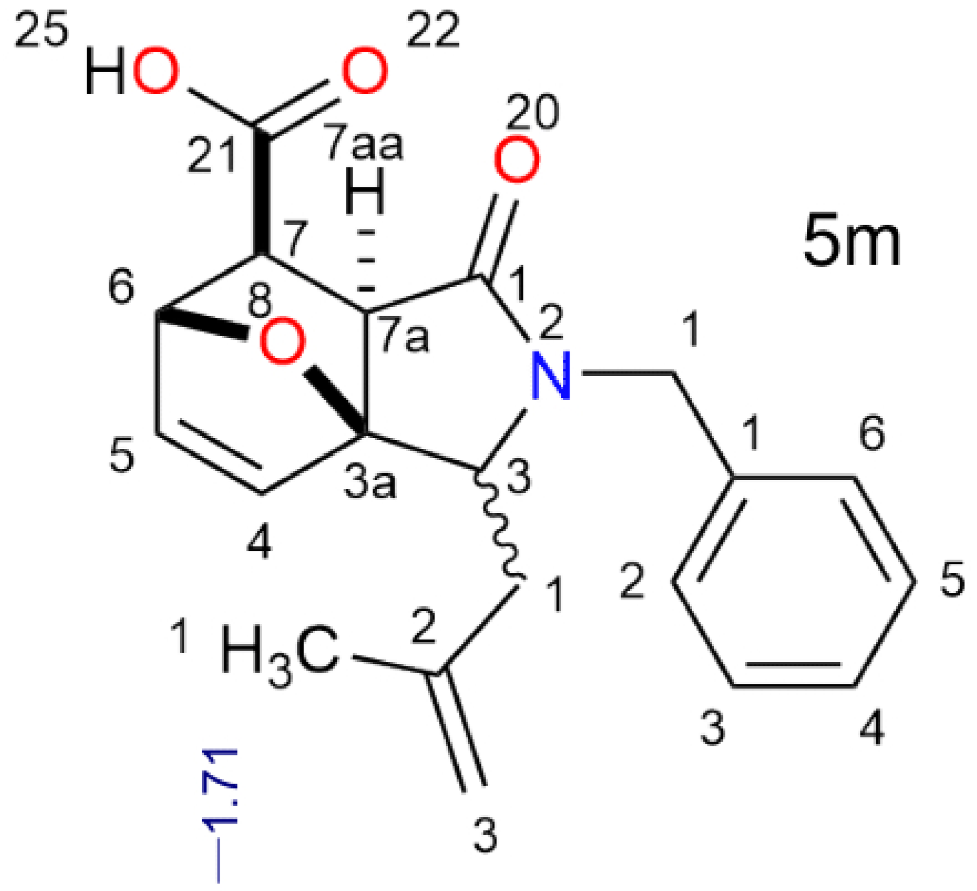

5m

FZ7646-3.esp

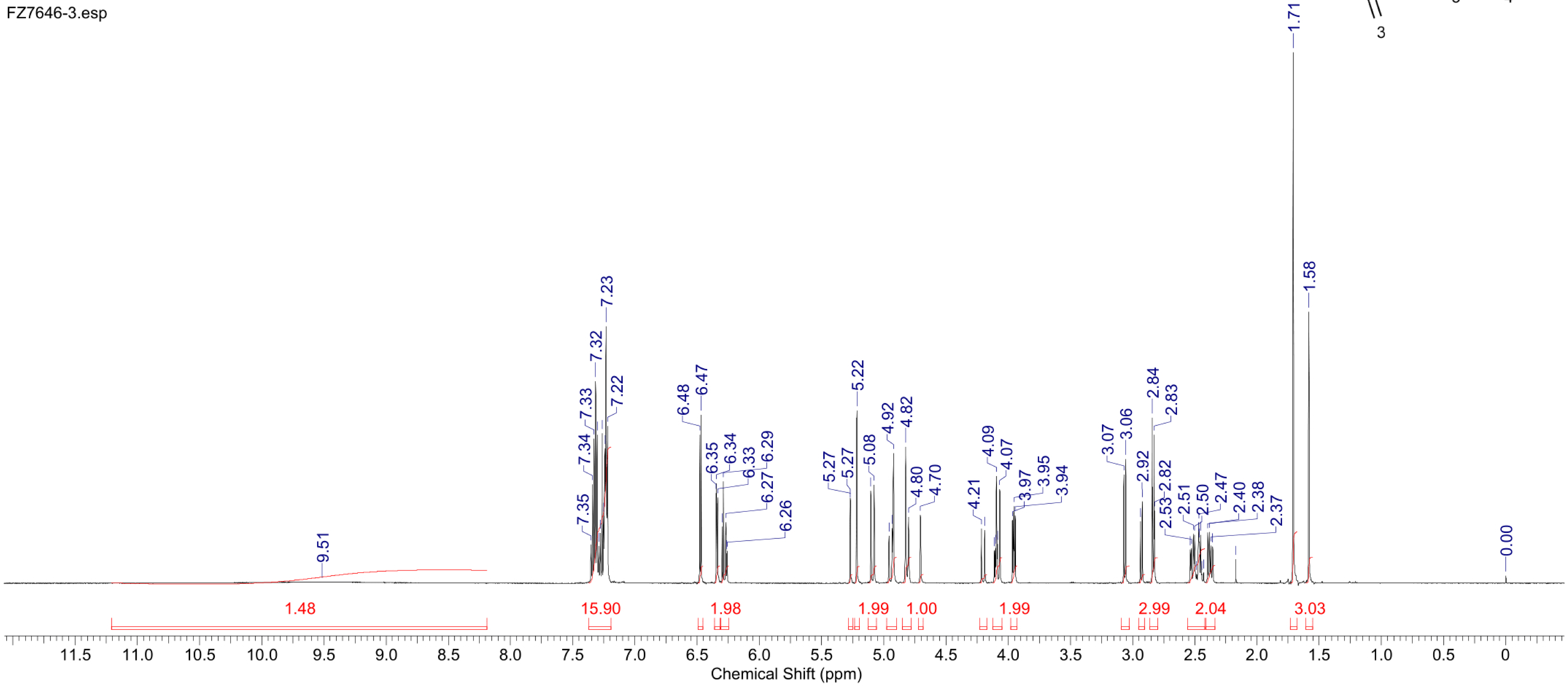

Acquisition Time (sec)	1.9818	***Comment***	single_pulse	***Date***	29 May 1990 08:13:49				
Date Stamp	08 Aug 2019 14:17:16			***File Name***	C:\Users\Fedor\Desktop\07.08.19\FZ7646-3.jdf			***Frequency (MHz)***	600.17
Nucleus	1H	***Number of Transients***	8	***Origin***	ECA 600	***Original Points Count***	32768	***Owner***	delta
Points Count	32768	***Pulse Sequence***	single_pulse.ex2			***Receiver Gain***	36.00	***Solvent***	CHLOROFORM-d
Spectrum Offset (Hz)	5409.6182	***Sweep Width (Hz)***	16534.39	***Temperature (degree C)***	23.100				

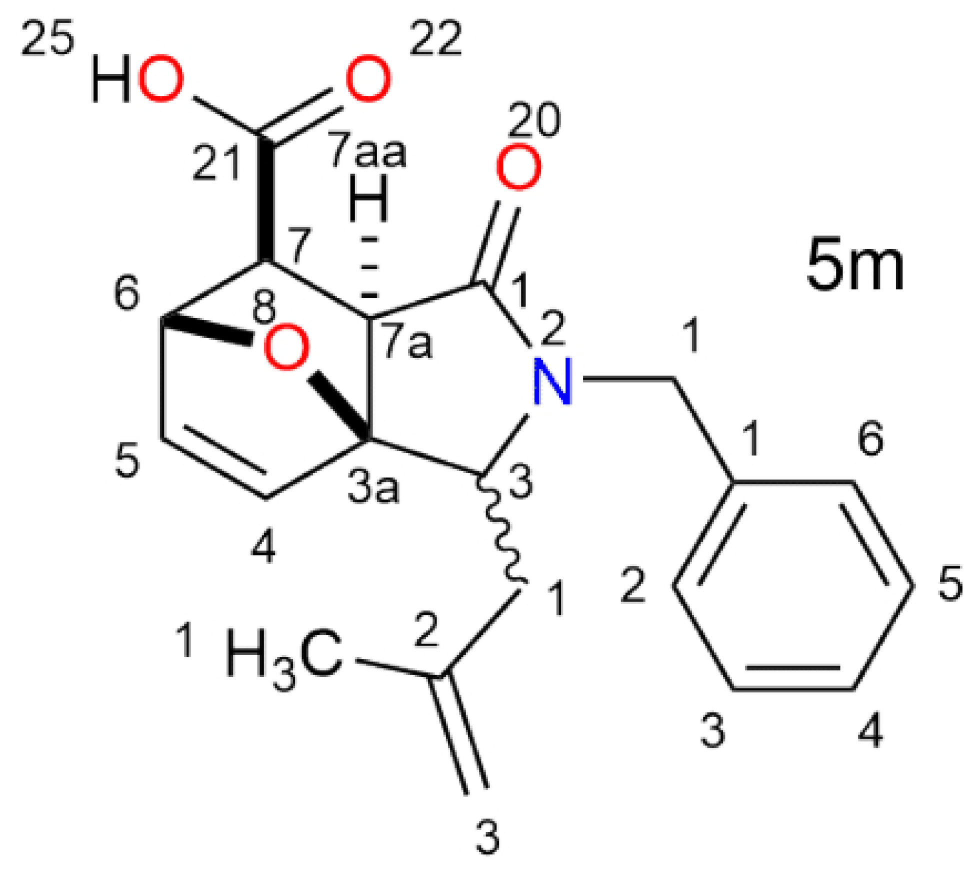

5m

FZ7646-3.esp

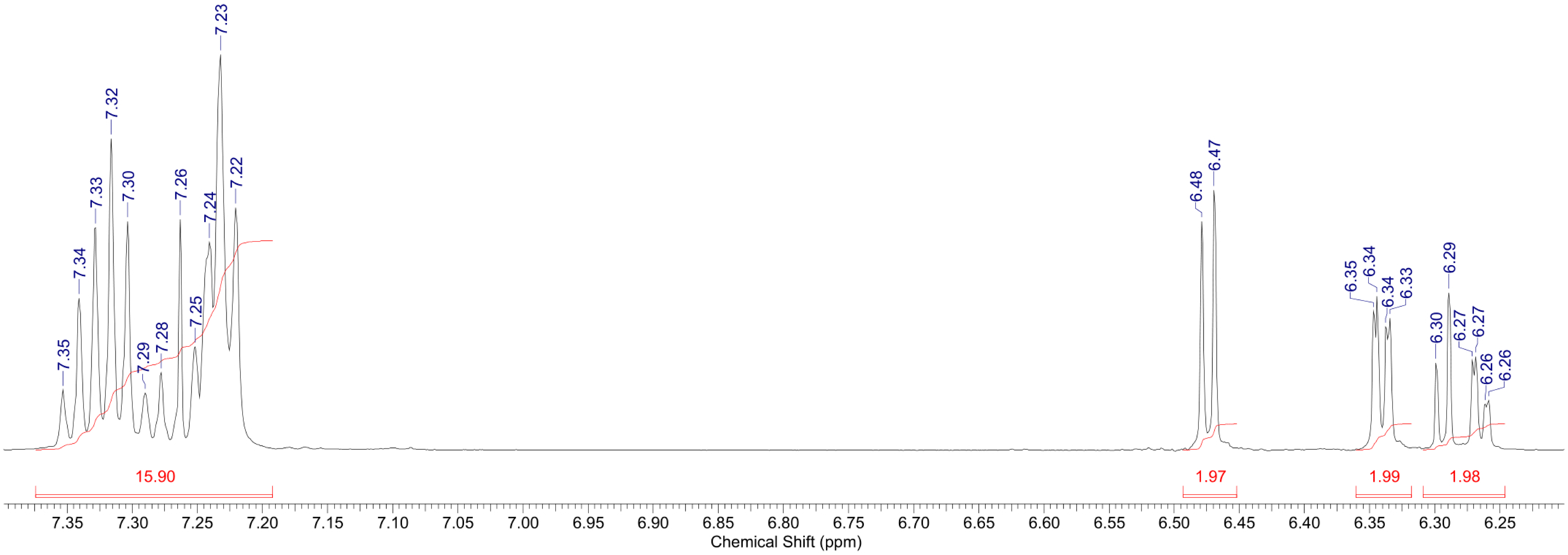

Acquisition Time (sec)	1.9818
Comment	single_pulse
Date	29 May 1990 08:13:49
Date Stamp	08 Aug 2019 14:17:16
File Name	C:\Users\Fedor\Desktop\07.08.19\FZ7646-3.jdf
Frequency (MHz)	600.17
Nucleus	1H
Number of Transients	8
Origin	ECA 600
Original Points Count	32768
Owner	delta
Points Count	32768
Pulse Sequence	single_pulse.ex2
Receiver Gain	36.00
Solvent	CHLOROFORM-d
Spectrum Offset (Hz)	5409.6182
Sweep Width (Hz)	16534.39
Temperature (degree C)	23.100

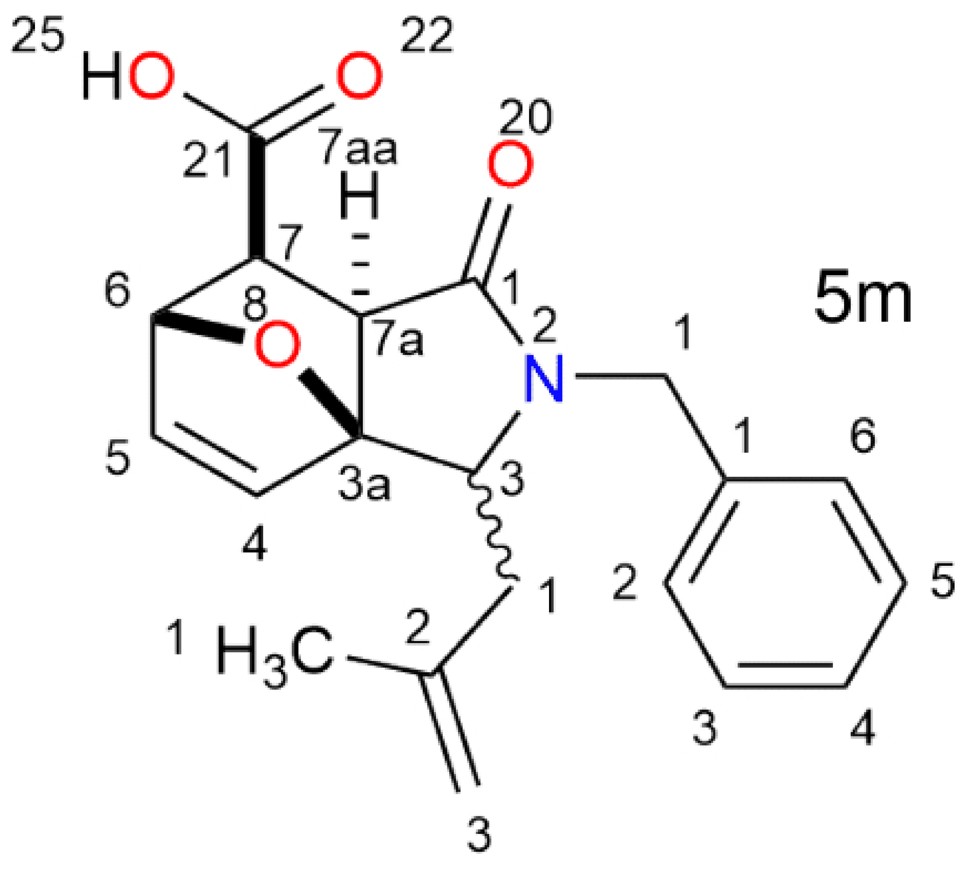

5m

FZ7646-3.esp

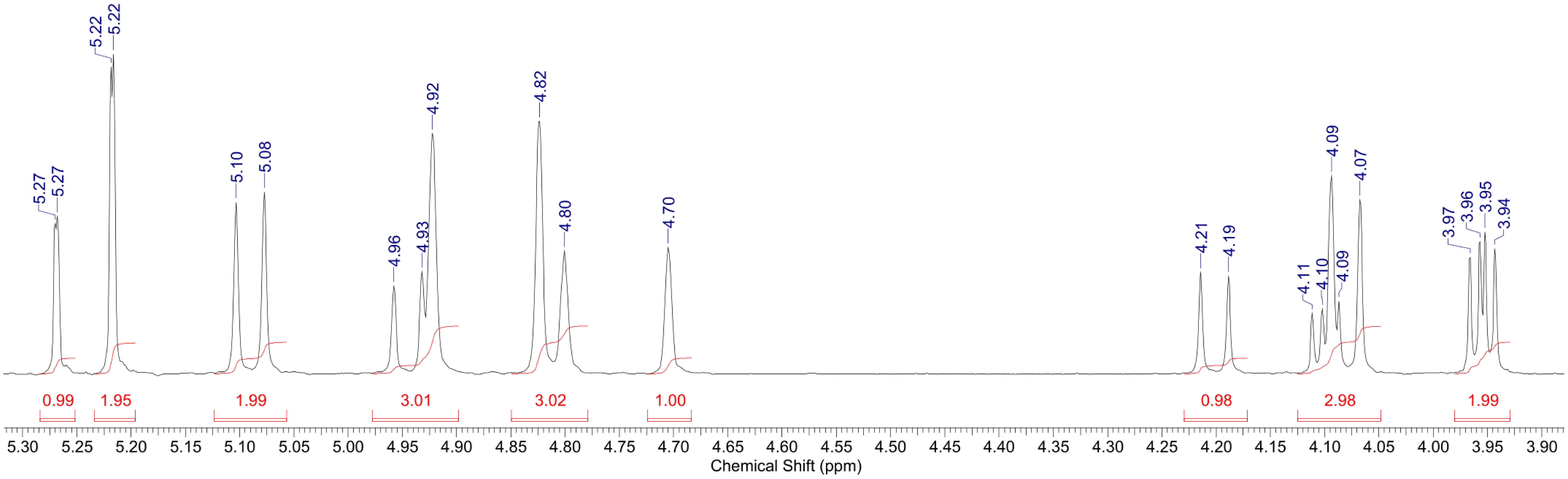

**Acquisition Time (sec)**	1.9818	**Comment**	single_pulse	**Date**	29 May 1990 08:13:49		
**Date Stamp**	08 Aug 2019 14:17:16			**File Name**	C:\Users\Fedor\Desktop\07.08.19\FZ7646-3.jdf	**Frequency (MHz)**	600.17
**Nucleus**	1H	**Number of Transients**	8	**Origin**	ECA 600	**Original Points Count**	32768
**Owner**	delta						
**Points Count**	32768	**Pulse Sequence**	single_pulse.ex2	**Receiver Gain**	36.00	**Solvent**	CHLOROFORM-d
**Spectrum Offset (Hz)**	5409.6182	**Sweep Width (Hz)**	16534.39	**Temperature (degree C)**	23.100		

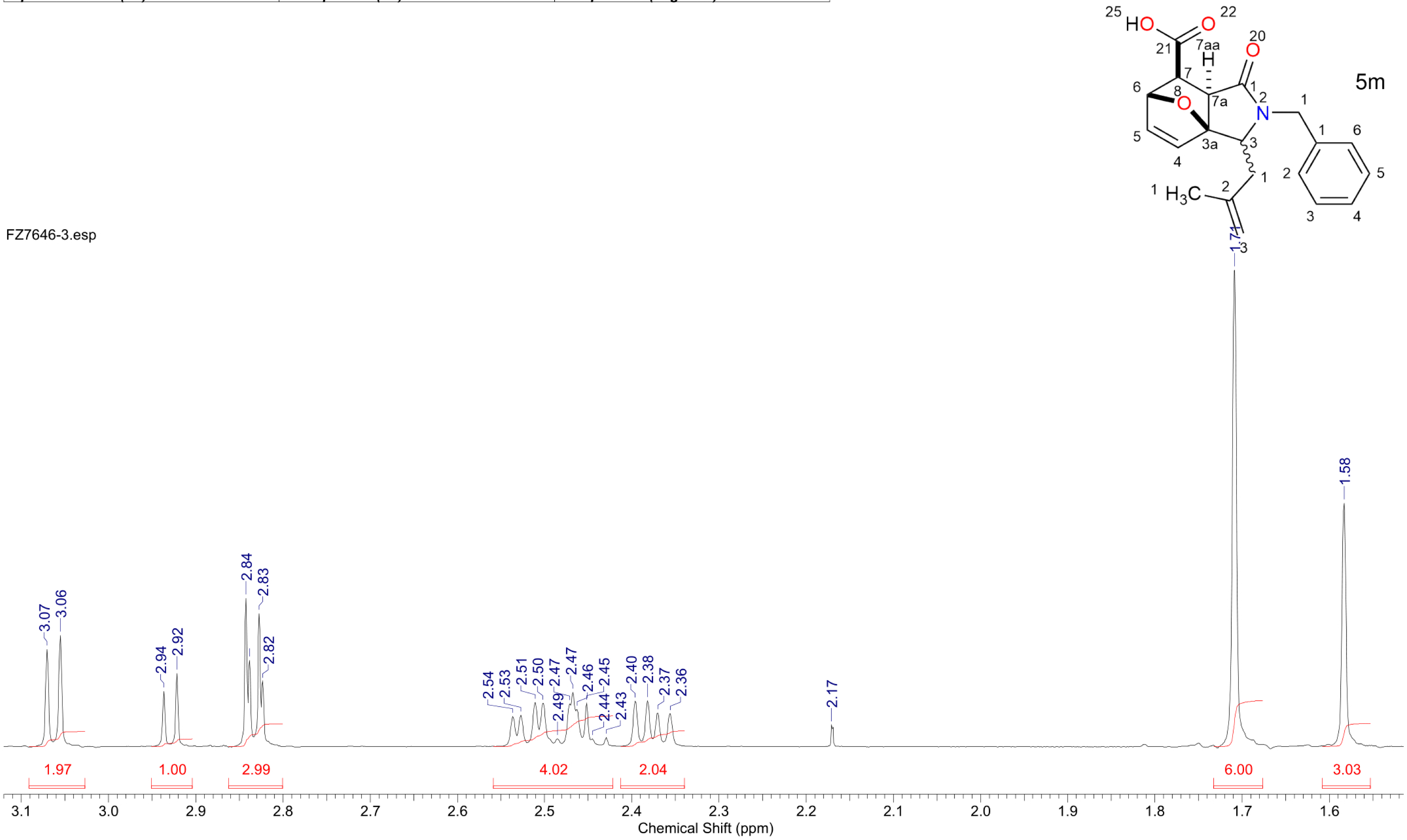

Acquisition Time (sec)	0.6921	Comment	single pulse decoupled gated NOE			Date	29 May 1990 08:12:28
Date Stamp	08 Aug 2019 14:15:55			File Name	C:\Users\Fedor\Desktop\07.08.19\FZ7646-1.jdf		
Frequency (MHz)	150.91	Nucleus	13C	Number of Transients	800	Origin	ECA 600
Original Points Count	32768	Owner	delta	Points Count	32768	Pulse Sequence	single_pulse_dec
Receiver Gain	58.00	Solvent	CHLOROFORM-d			Spectrum Offset (Hz)	15091.3428
Sweep Width (Hz)	47348.49	Temperature (degree C)	23.600				

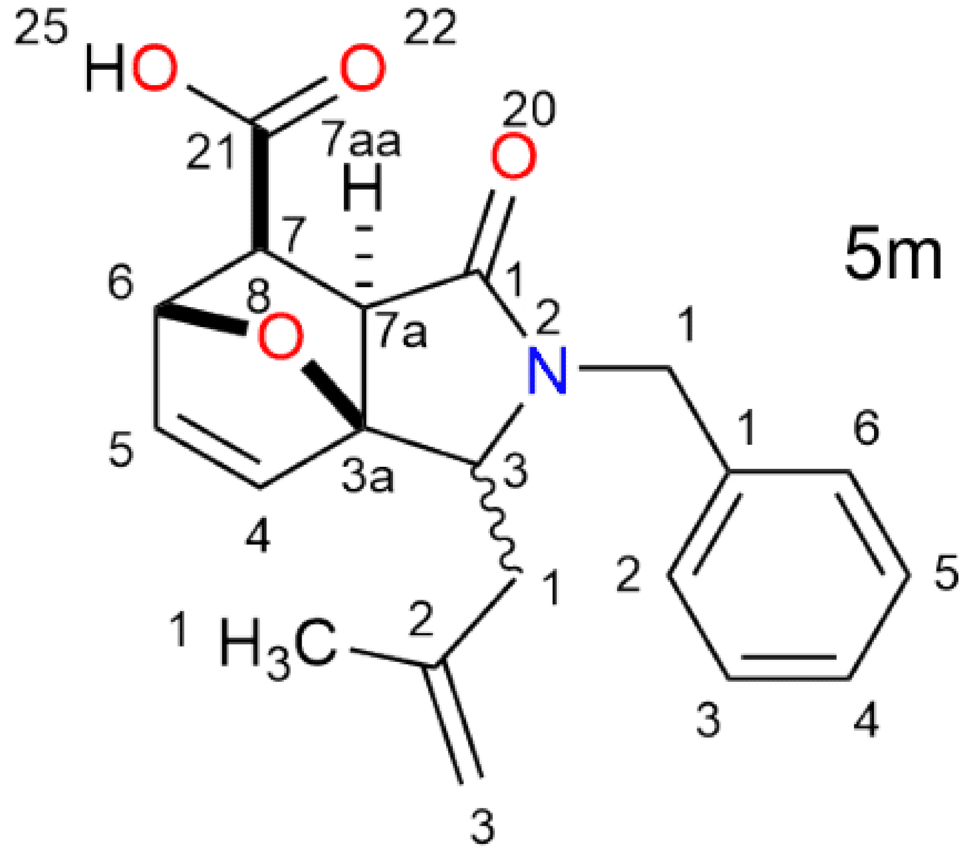

5m

FZ7646-1.jdf

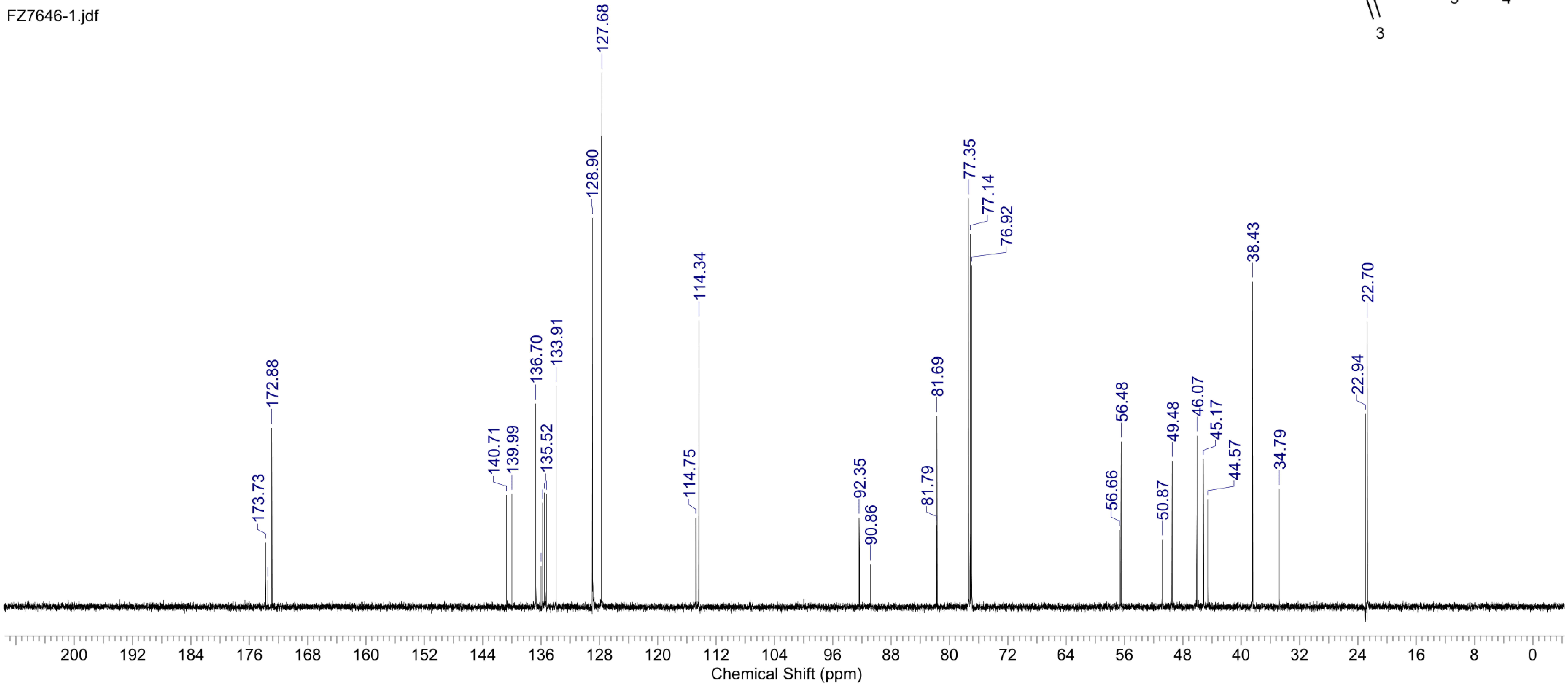

Acquisition Time (sec)	0.6921	***Comment***	single pulse decoupled gated NOE			***Date***	29 May 1990 08:12:28
Date Stamp	08 Aug 2019 14:15:55			***File Name***	C:\Users\Fedor\Desktop\07.08.19\FZ7646-1.jdf		
Frequency (MHz)	150.91	***Nucleus***	13C	***Number of Transients***	800	***Origin***	ECA 600
Original Points Count	32768	***Owner***	delta	***Points Count***	32768	***Pulse Sequence***	single_pulse_dec
Receiver Gain	58.00	***Solvent***	CHLOROFORM-d			***Spectrum Offset (Hz)***	15091.3428
Sweep Width (Hz)	47348.49	***Temperature (degree C)***	23.600				

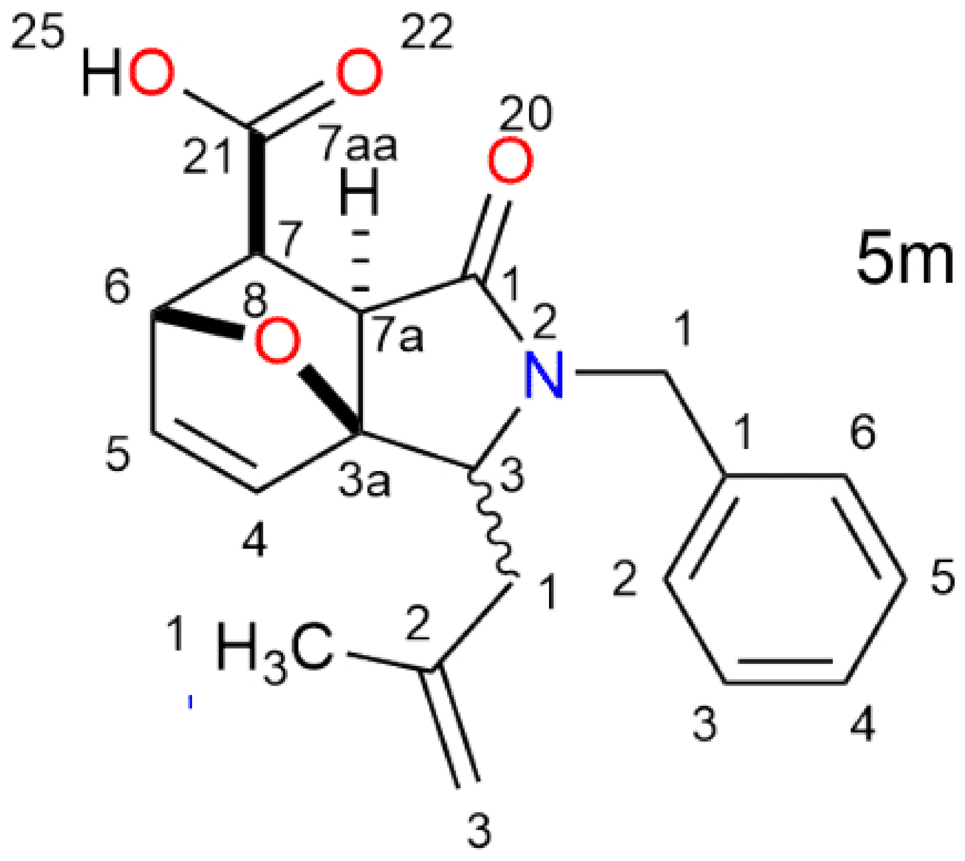

5m

FZ7646-1.jdf

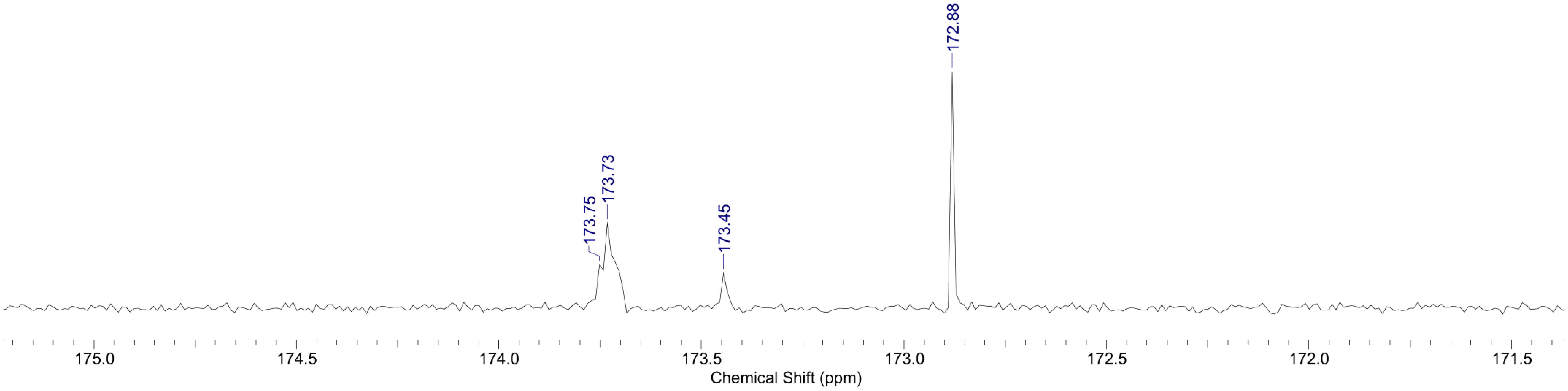

Acquisition Time (sec)	0.6921	***Comment***	single pulse decoupled gated NOE			***Date***	29 May 1990 08:12:28
Date Stamp	08 Aug 2019 14:15:55			***File Name***	C:\Users\Fedor\Desktop\07.08.19\FZ7646-1.jdf		
Frequency (MHz)	150.91	***Nucleus***	13C	***Number of Transients***	800	***Origin***	ECA 600
Original Points Count	32768	***Owner***	delta	***Points Count***	32768	***Pulse Sequence***	single_pulse_dec
Receiver Gain	58.00	***Solvent***	CHLOROFORM-d			***Spectrum Offset (Hz)***	15091.3428
Sweep Width (Hz)	47348.49	***Temperature (degree C)***	23.600				

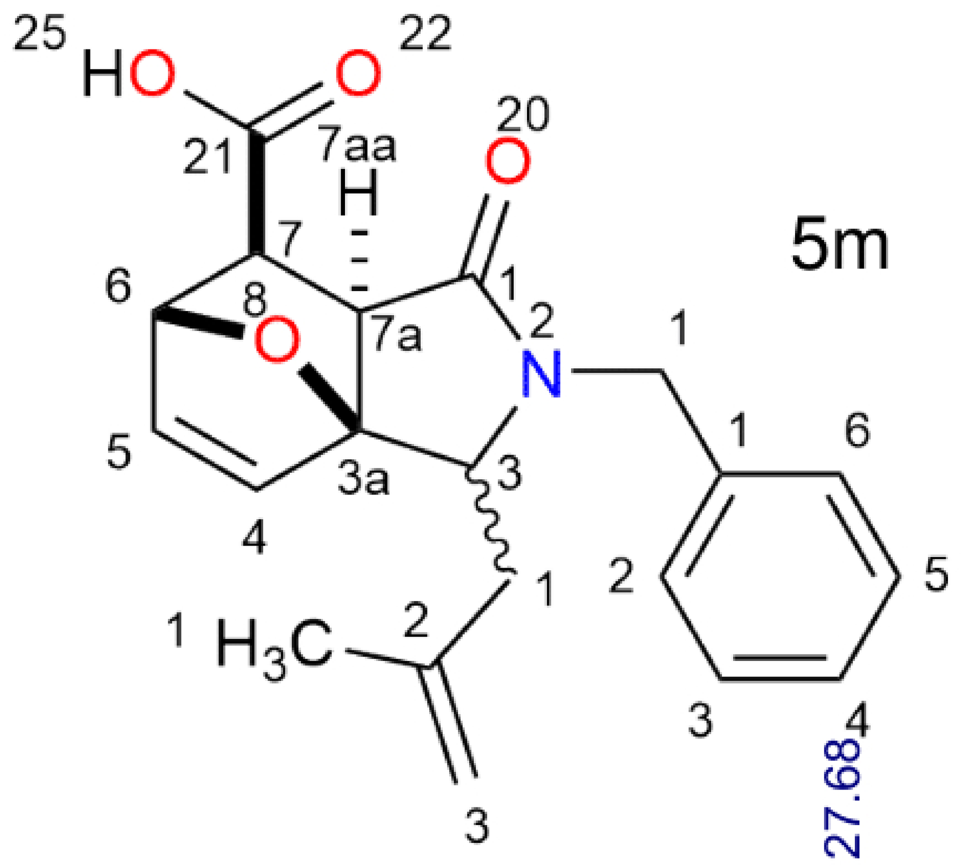

5m

FZ7646-1.jdf

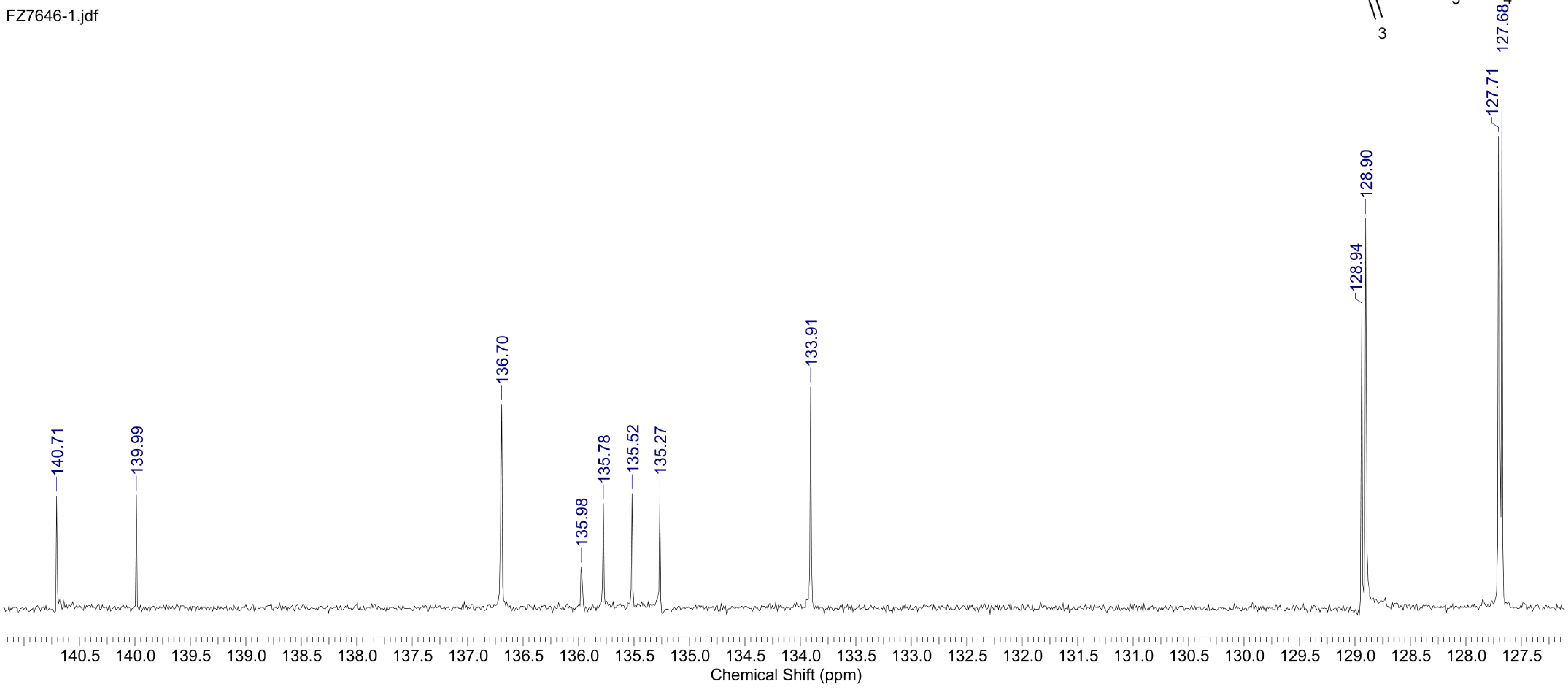

Acquisition Time (sec)	0.6921	***Comment***	single pulse decoupled gated NOE	***Date***	29 May 1990 08:12:28
Date Stamp	08 Aug 2019 14:15:55	***File Name***	C:\Users\Fedor\Desktop\07.08.19\FZ7646-1.jdf		
Frequency (MHz)	150.91	***Nucleus***	13C	***Number of Transients***	800
Origin	ECA 600	***Original Points Count***	32768	***Owner***	delta
Points Count	32768	***Pulse Sequence***	single_pulse_dec	***Receiver Gain***	58.00
Solvent	CHLOROFORM-d	***Spectrum Offset (Hz)***	15091.3428	***Sweep Width (Hz)***	47348.49
Temperature (degree C)	23.600				

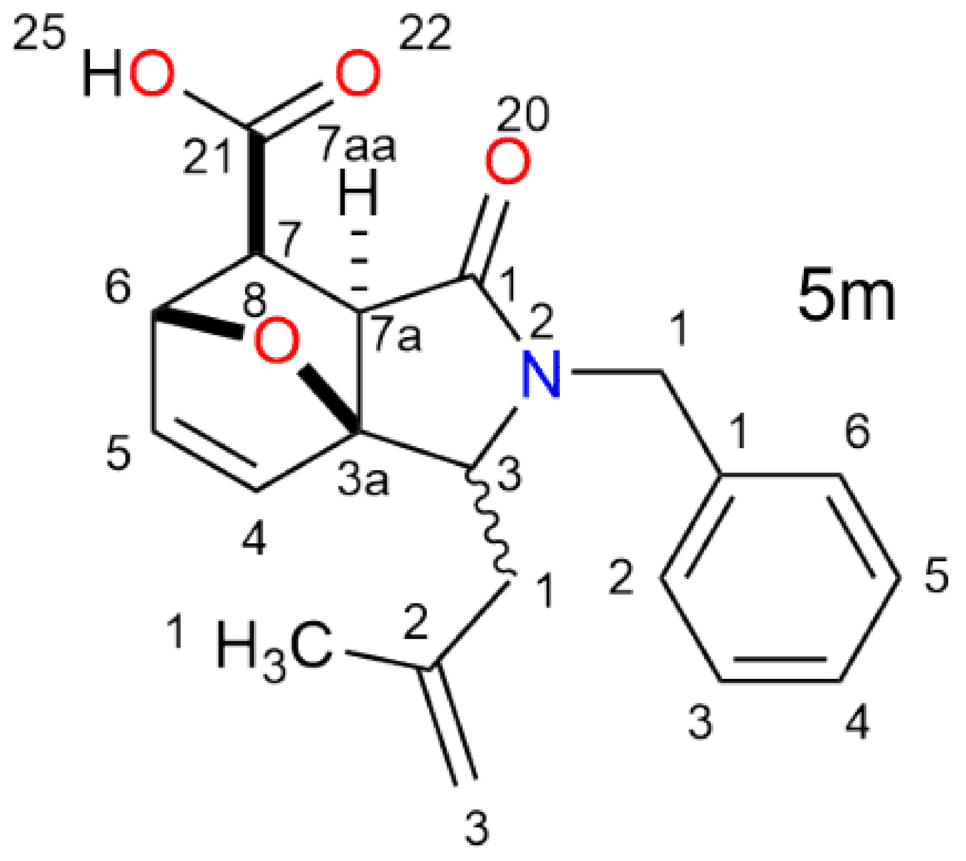

5m

FZ7646-1.jdf

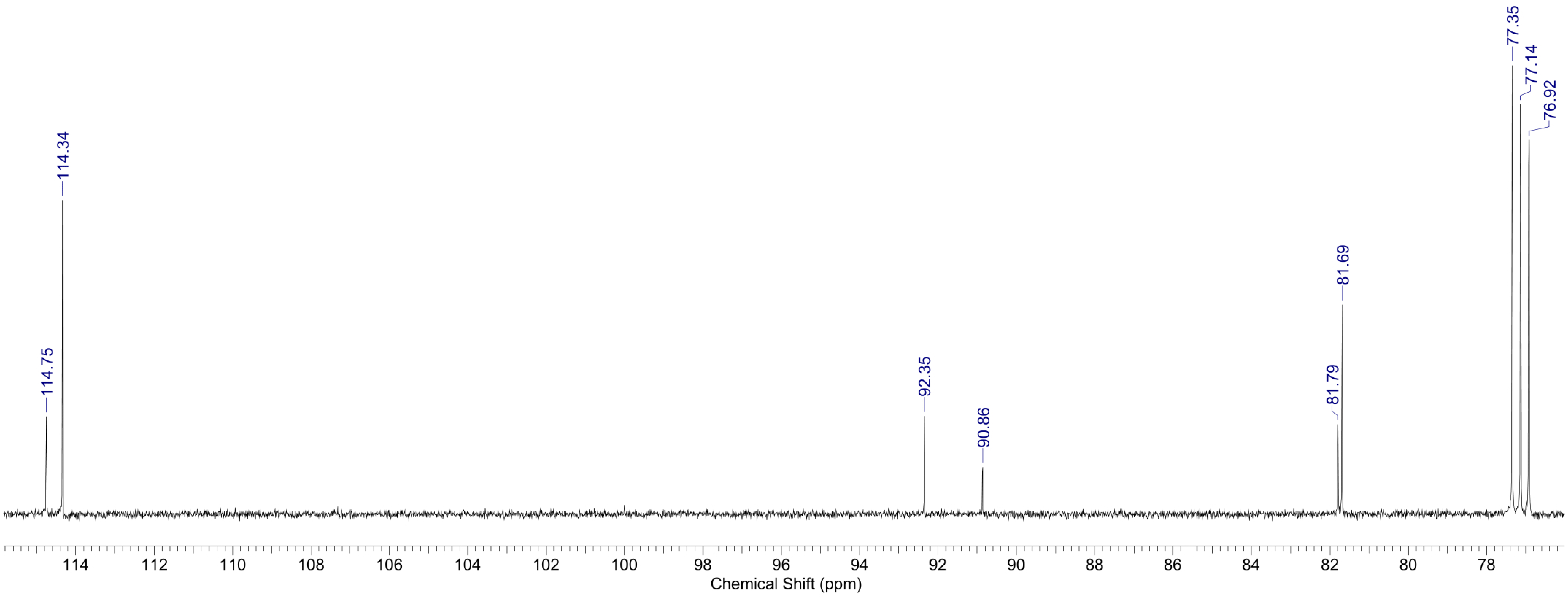

Acquisition Time (sec)	0.6921	***Comment***	single pulse decoupled gated NOE			***Date***	29 May 1990 08:12:28
Date Stamp	08 Aug 2019 14:15:55			***File Name***	C:\Users\Fedor\Desktop\07.08.19\FZ7646-1.jdf		
Frequency (MHz)	150.91	***Nucleus***	13C	***Number of Transients***	800	***Origin***	ECA 600
Original Points Count	32768	***Owner***	delta	***Points Count***	32768	***Pulse Sequence***	single_pulse_dec
Receiver Gain	58.00	***Solvent***	CHLOROFORM-d			***Spectrum Offset (Hz)***	15091.3428
Sweep Width (Hz)	47348.49	***Temperature (degree C)***	23.600				

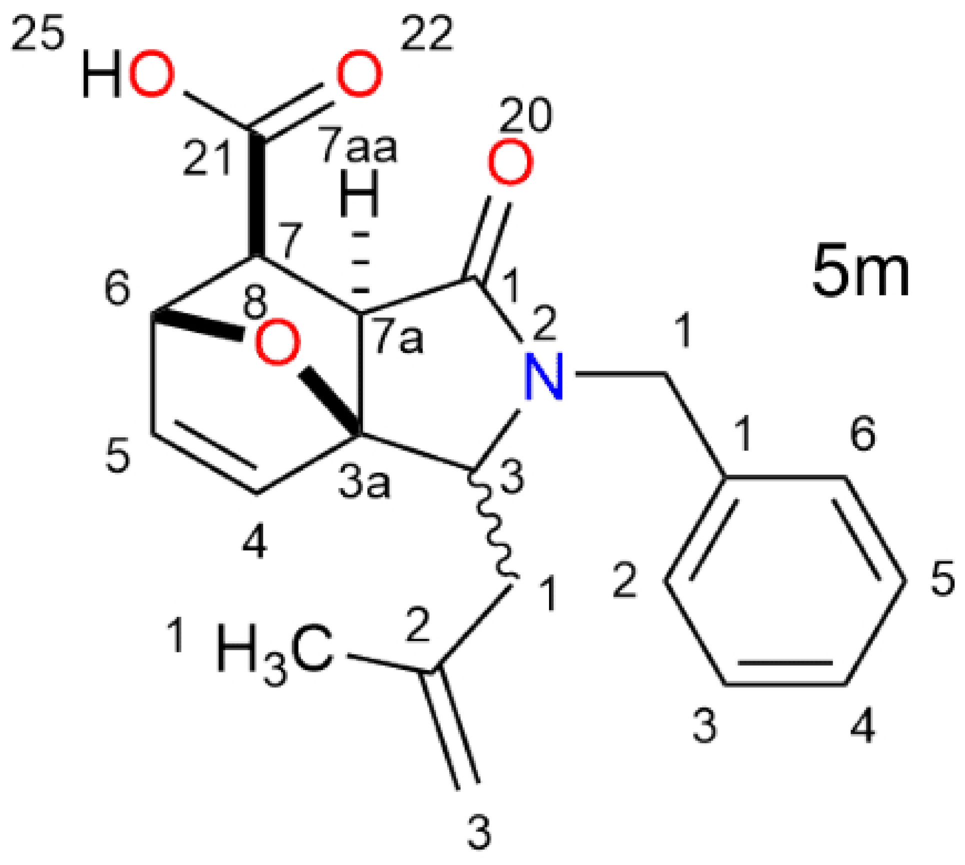

5m

FZ7646-1.jdf

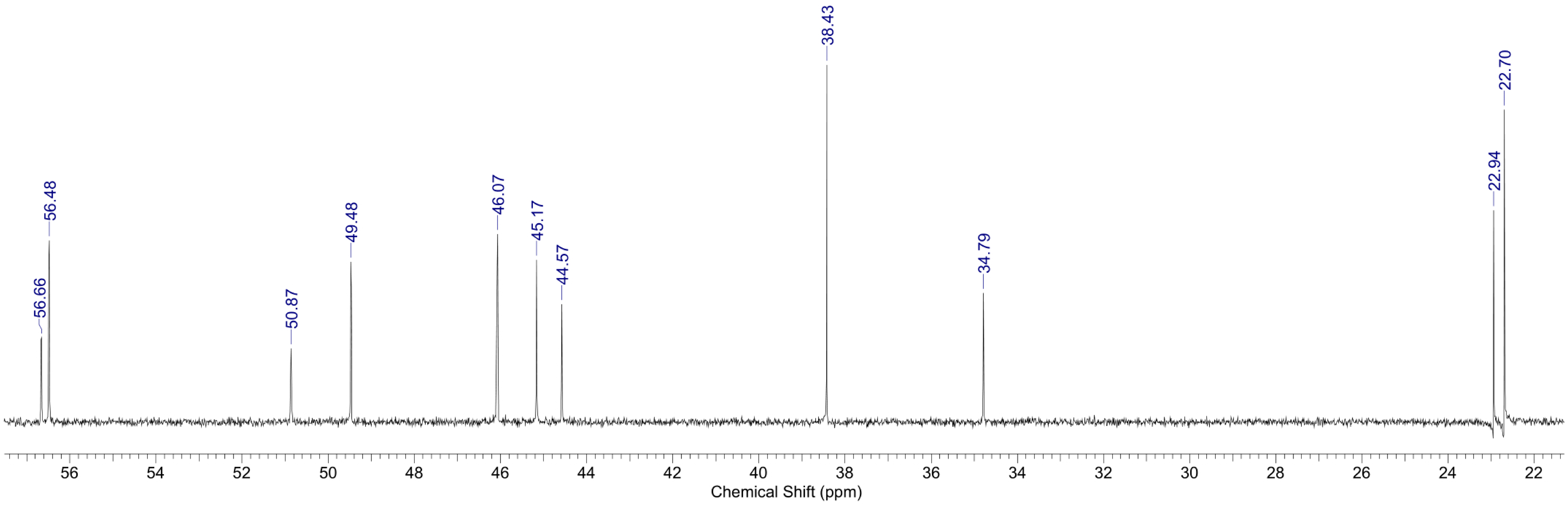

DEPT NMR spectrum for compound 5m

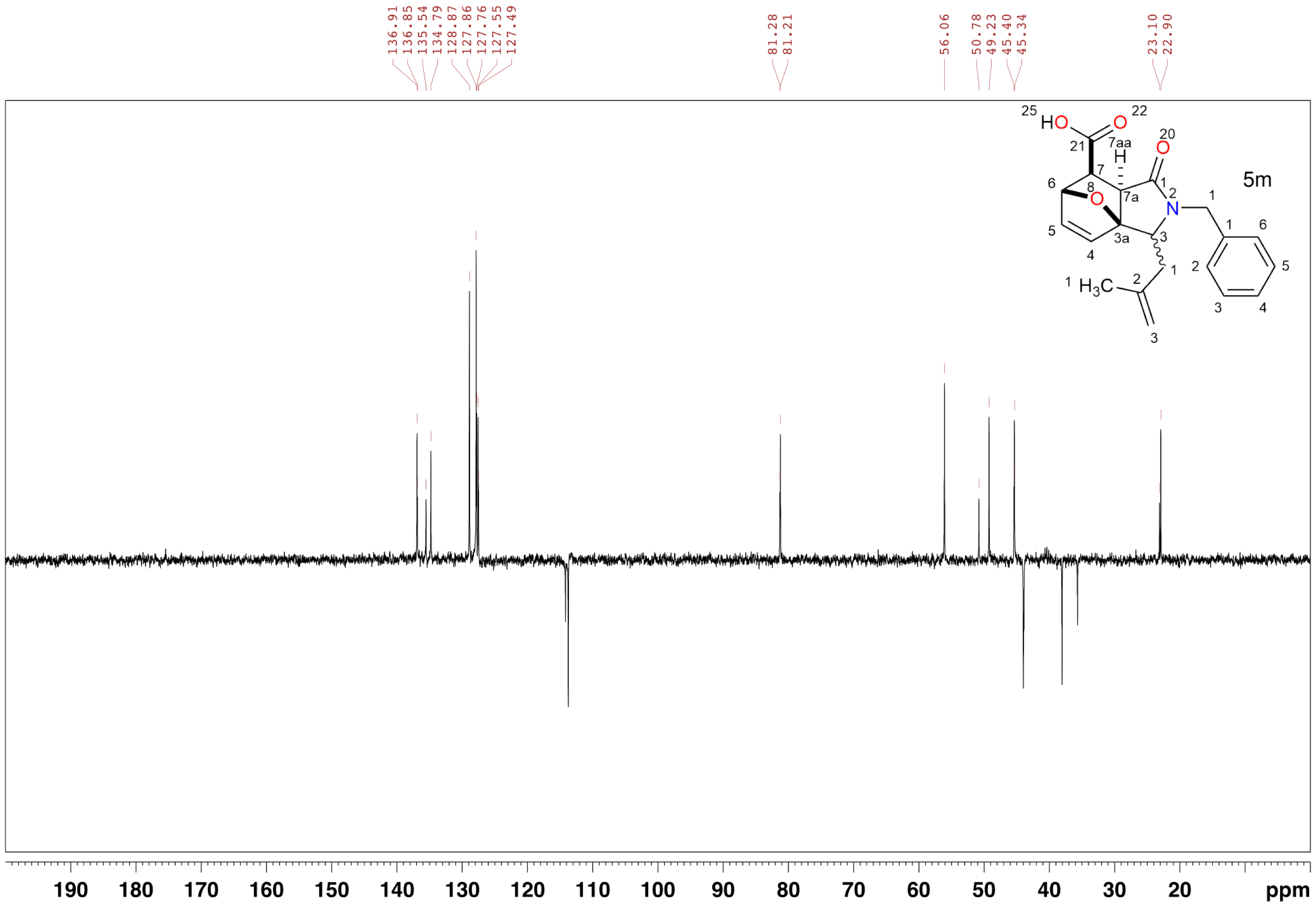

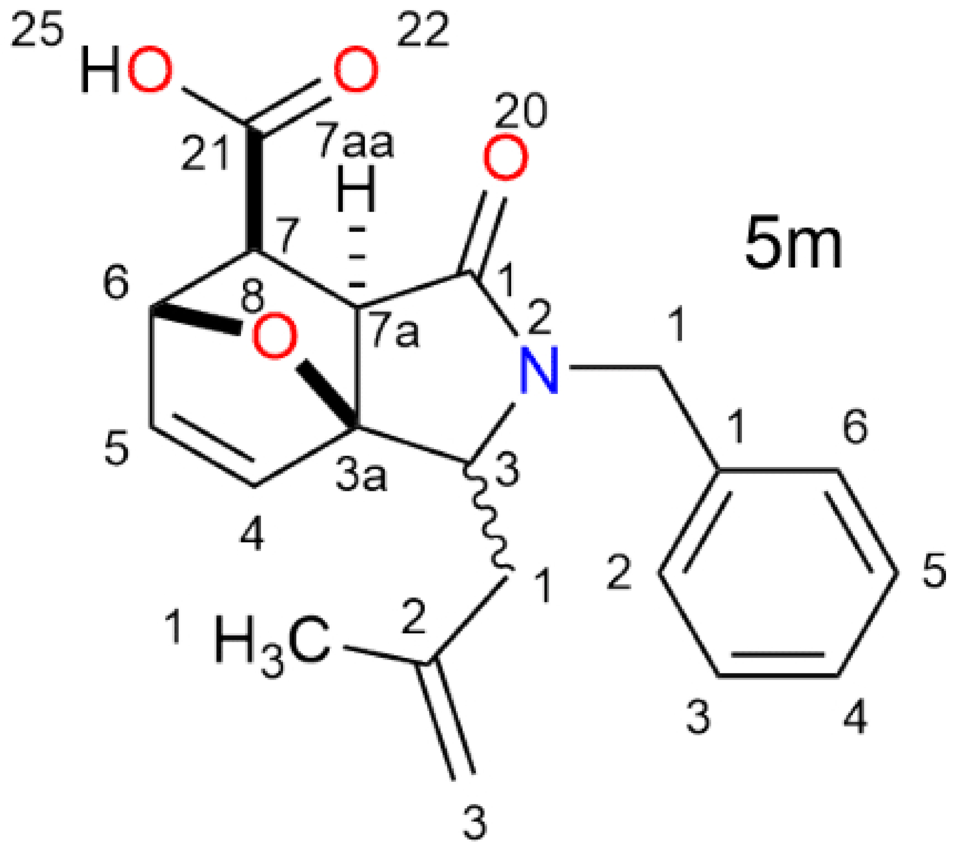

5m

COSY NMR spectrum for compound 5k

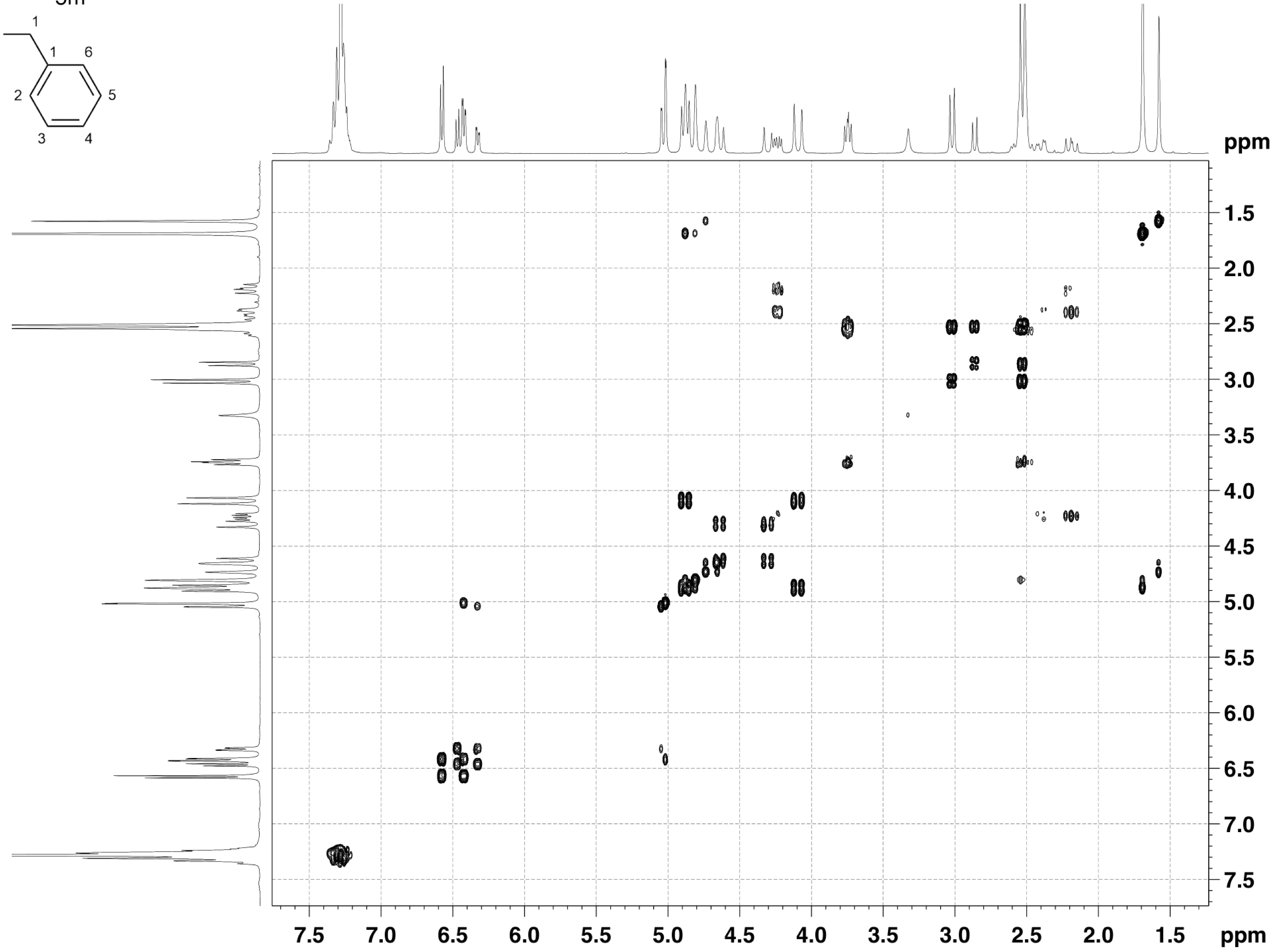

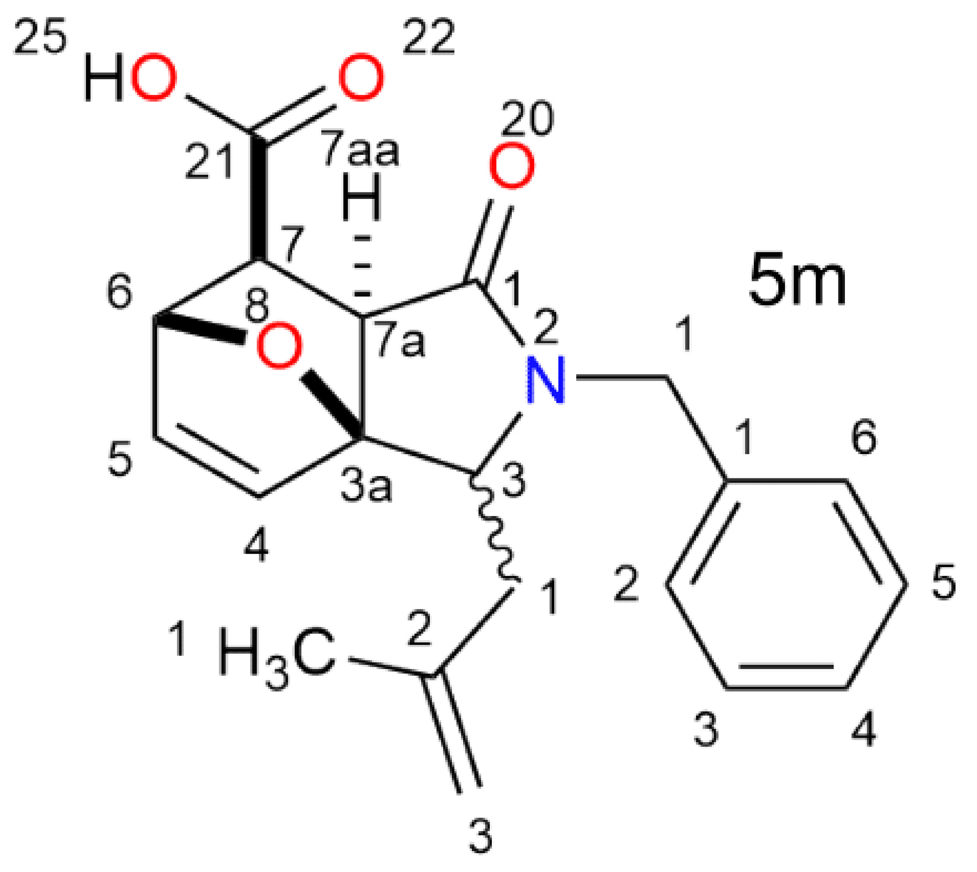

5m

NOESY NMR spectrum for compound 5m

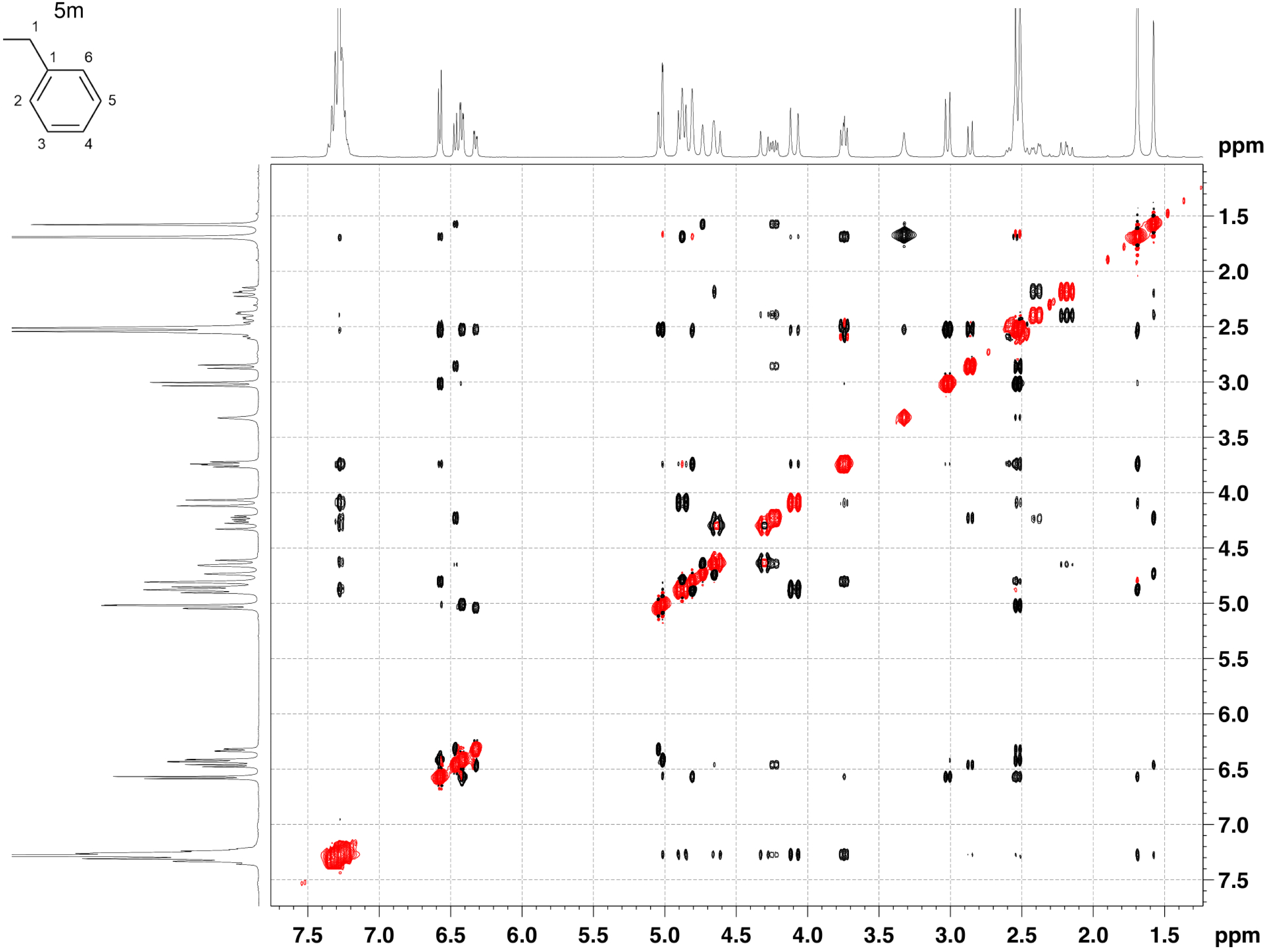

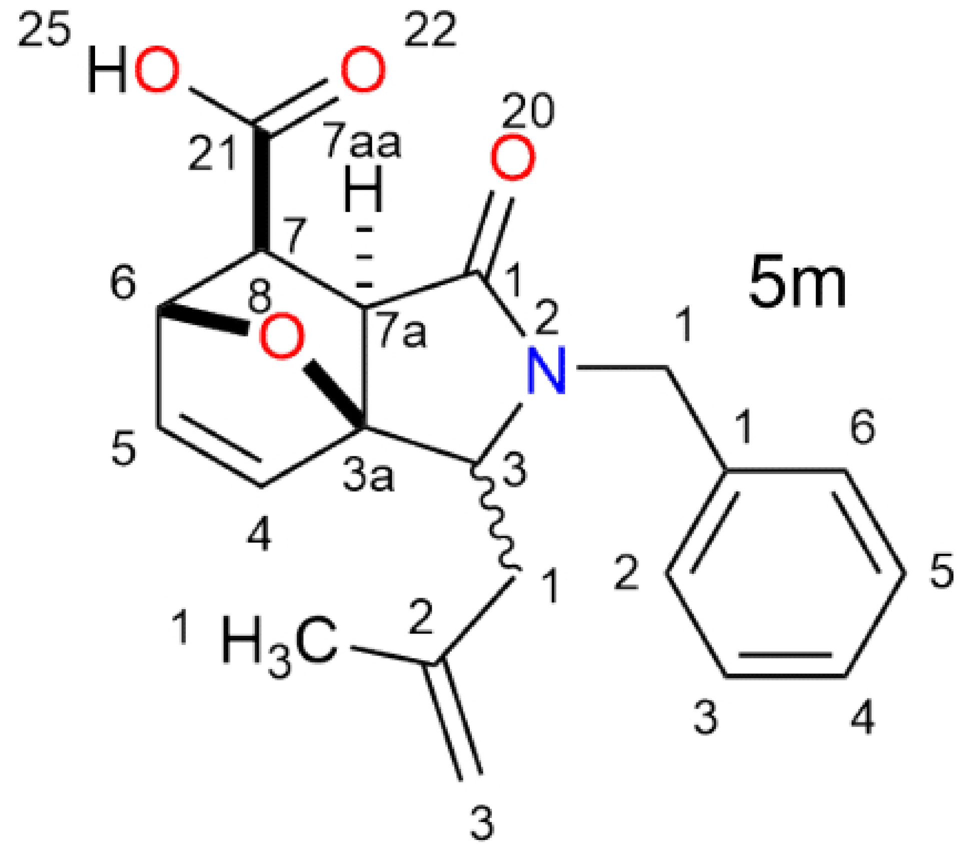

NOESY NMR spectrum for compound 5k

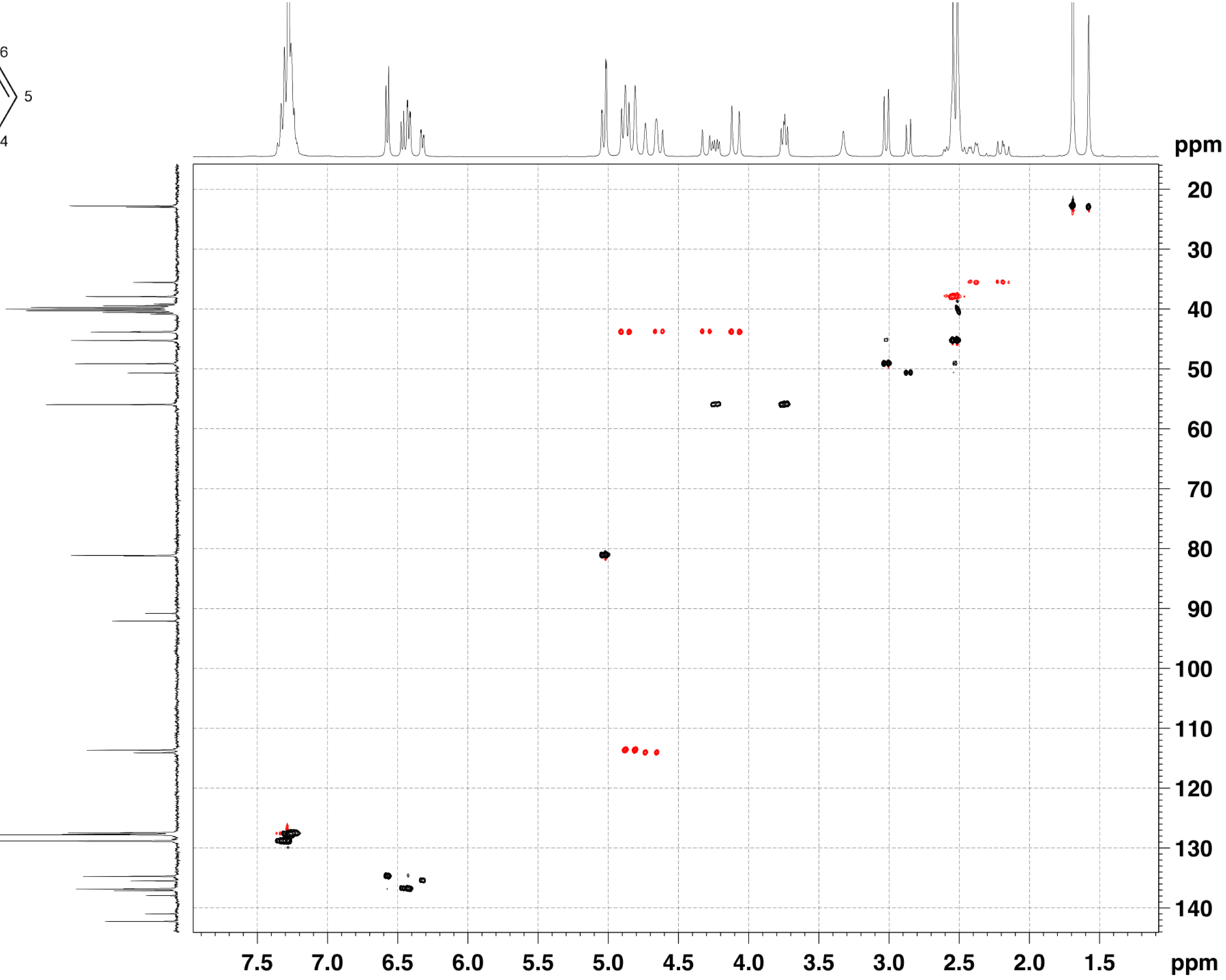

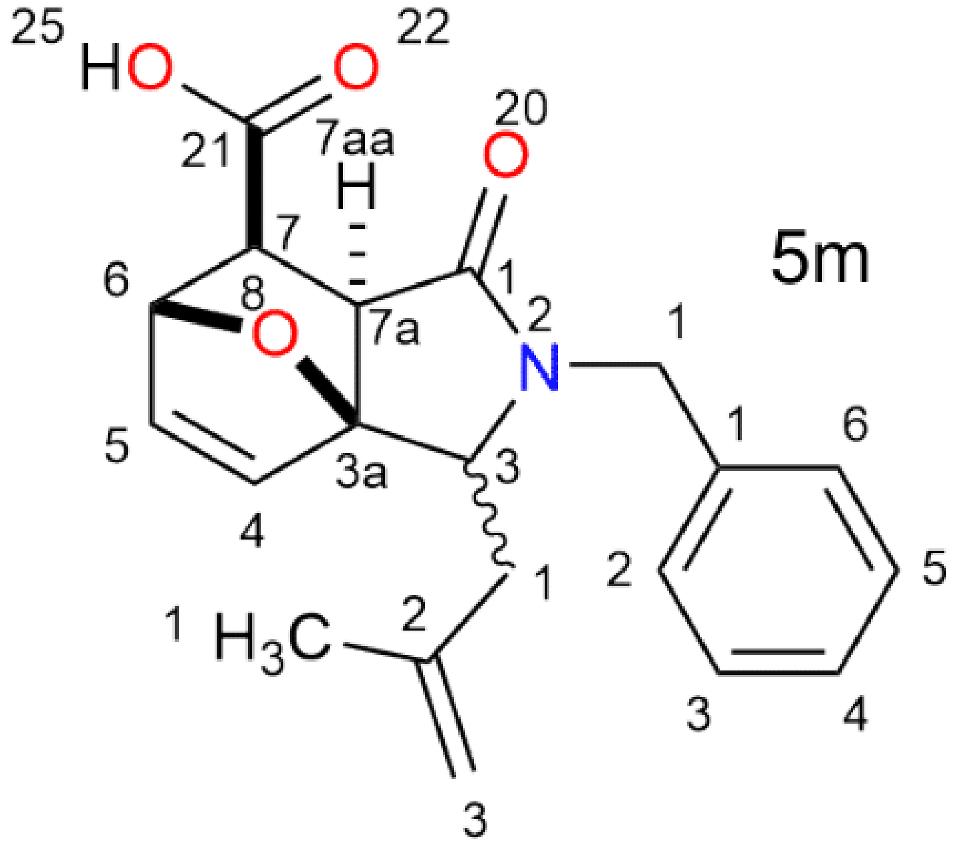
5m

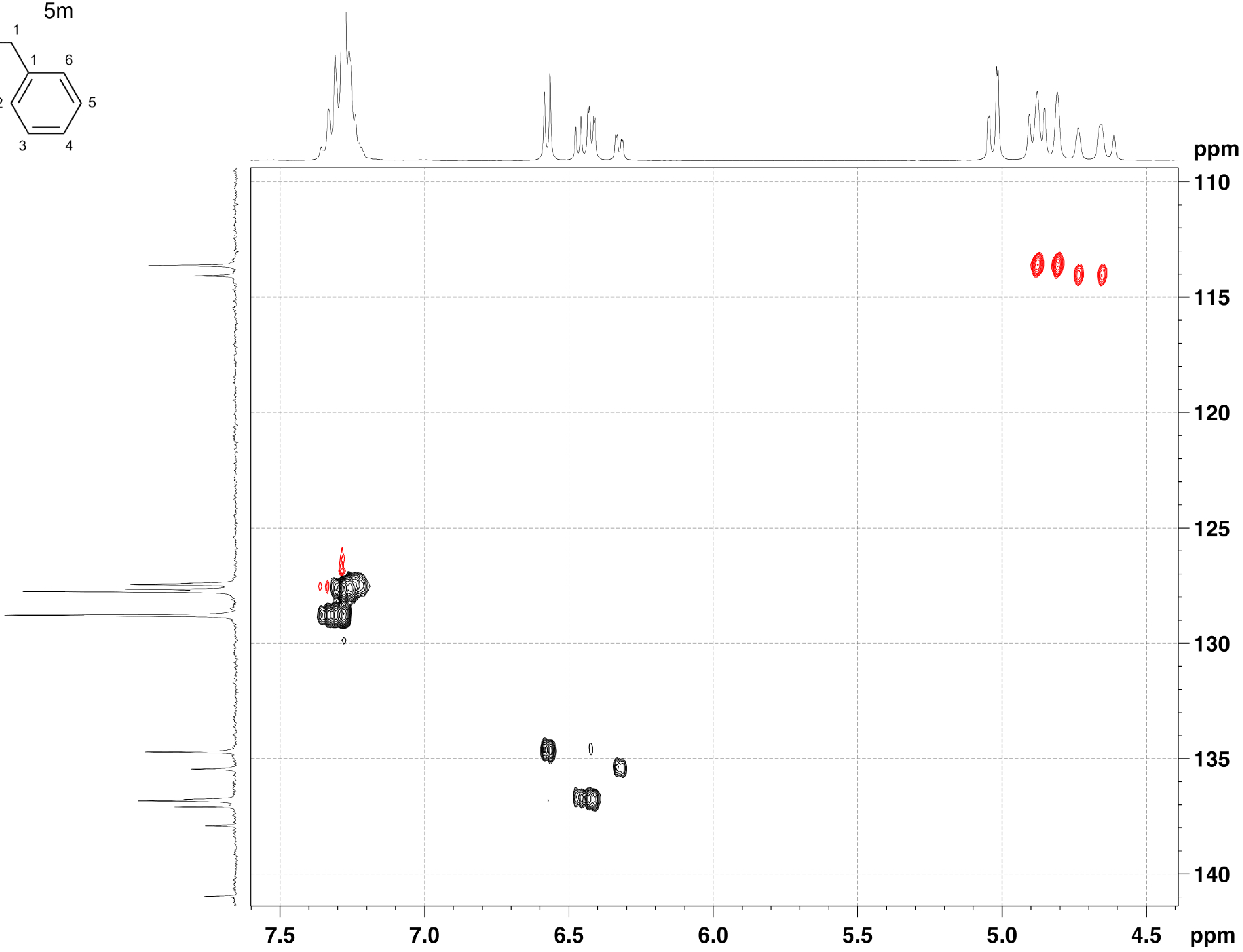
ppm
110
115
120
125
130
135
140
7.5
7.0
6.5
6.0
5.5
5.0
4.5
ppm

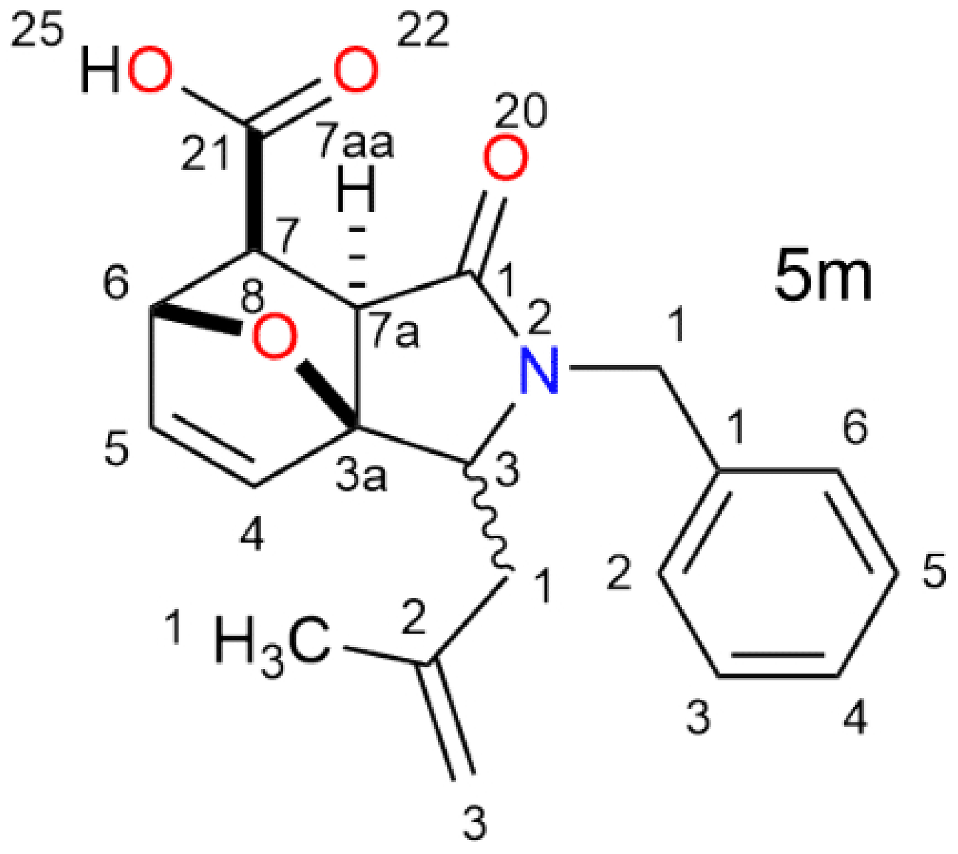
5m

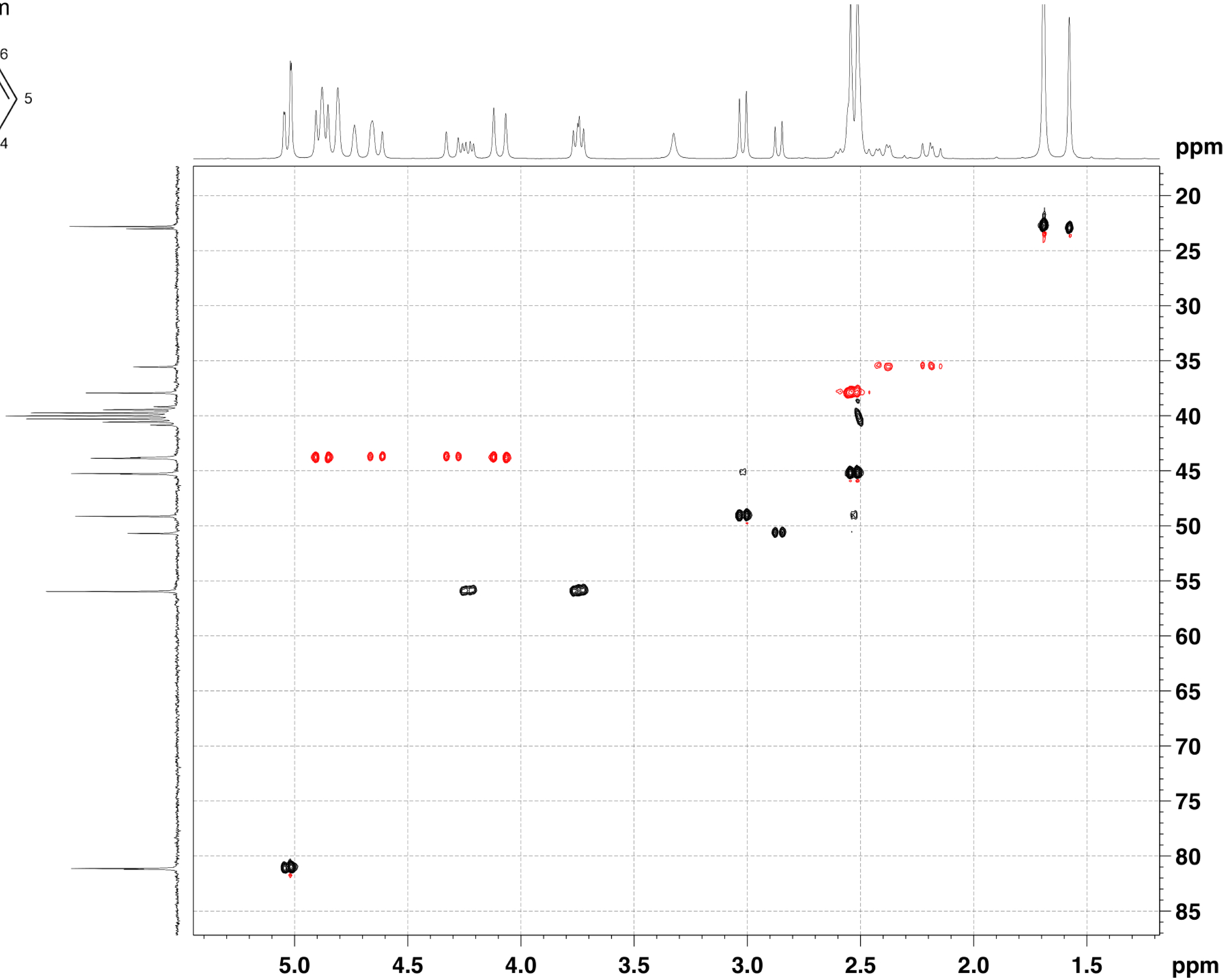
ppm
20
25
30
35
40
45
50
55
60
65
70
75
80
85
5.0
4.5
4.0
3.5
3.0
2.5
2.0
1.5
ppm

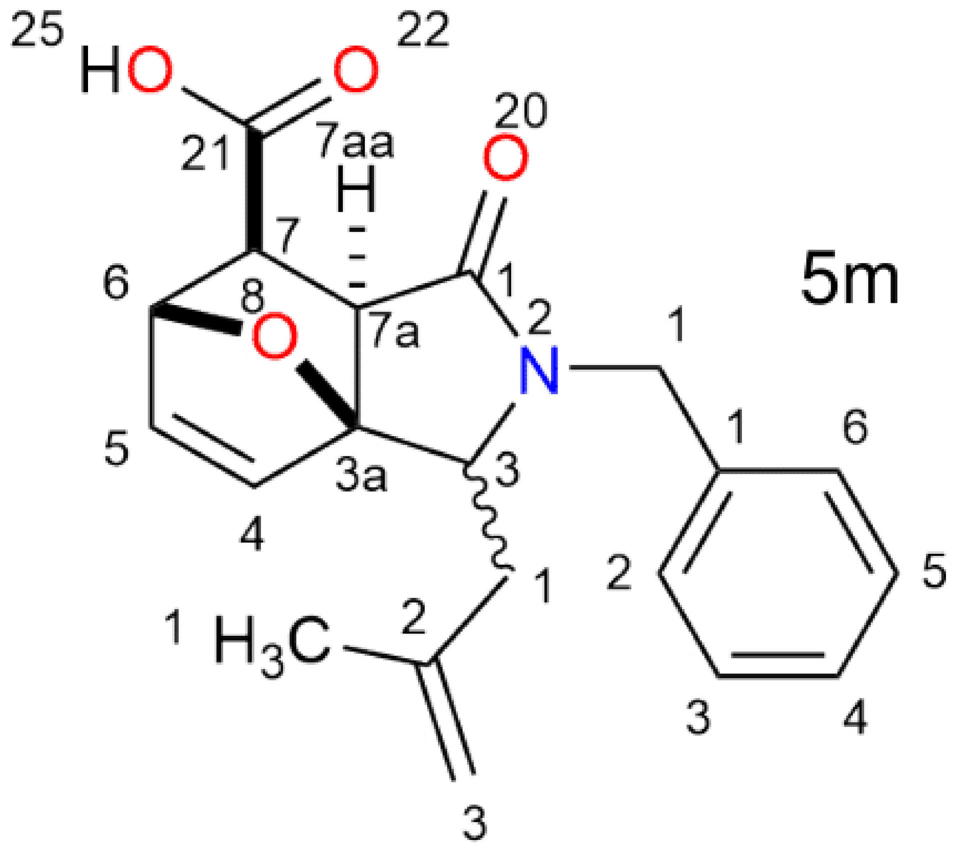

5m

HMBC NMR spectrum for compound 5m

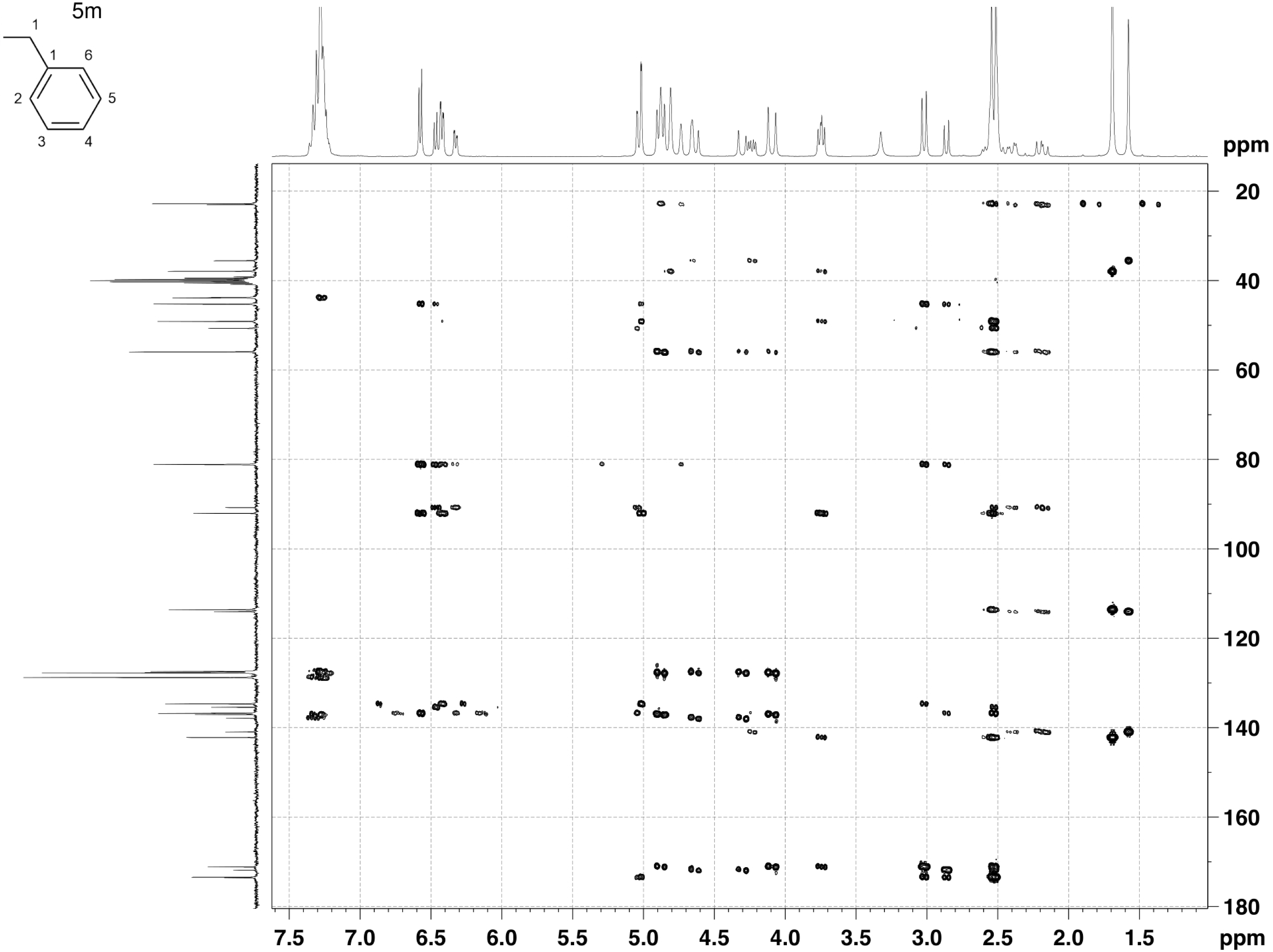

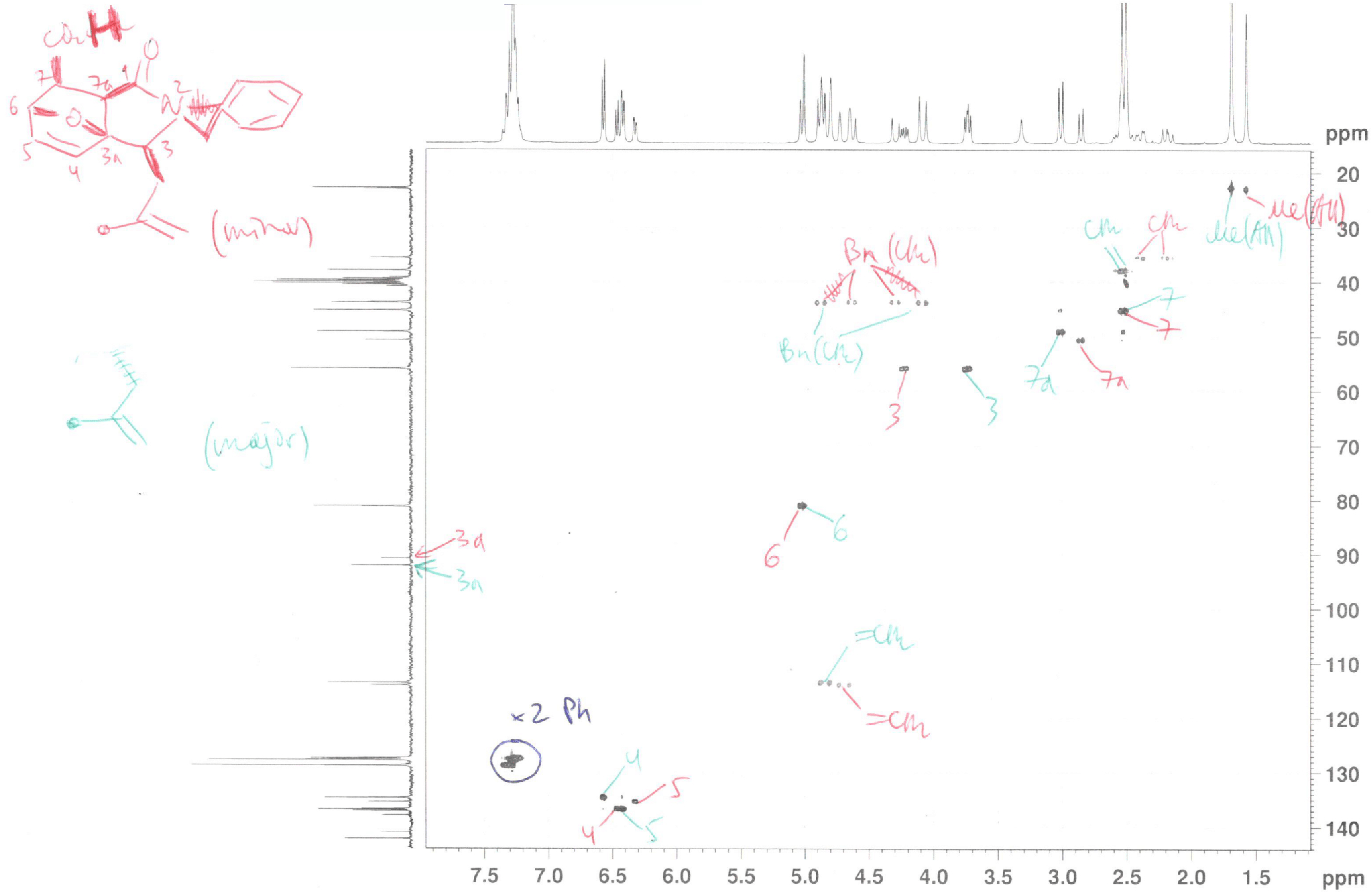

ppm
20
30
40
50
60
70
80
90
100
110
120
130
140
7.5
7.0
6.5
6.0
5.5
5.0
4.5
4.0
3.5
3.0
2.5
2.0
1.5
ppm
(minor)
(major)
×2 Ph
Bn (CH2)
=CH2
3a

1H NMR spectrum for compound 5p

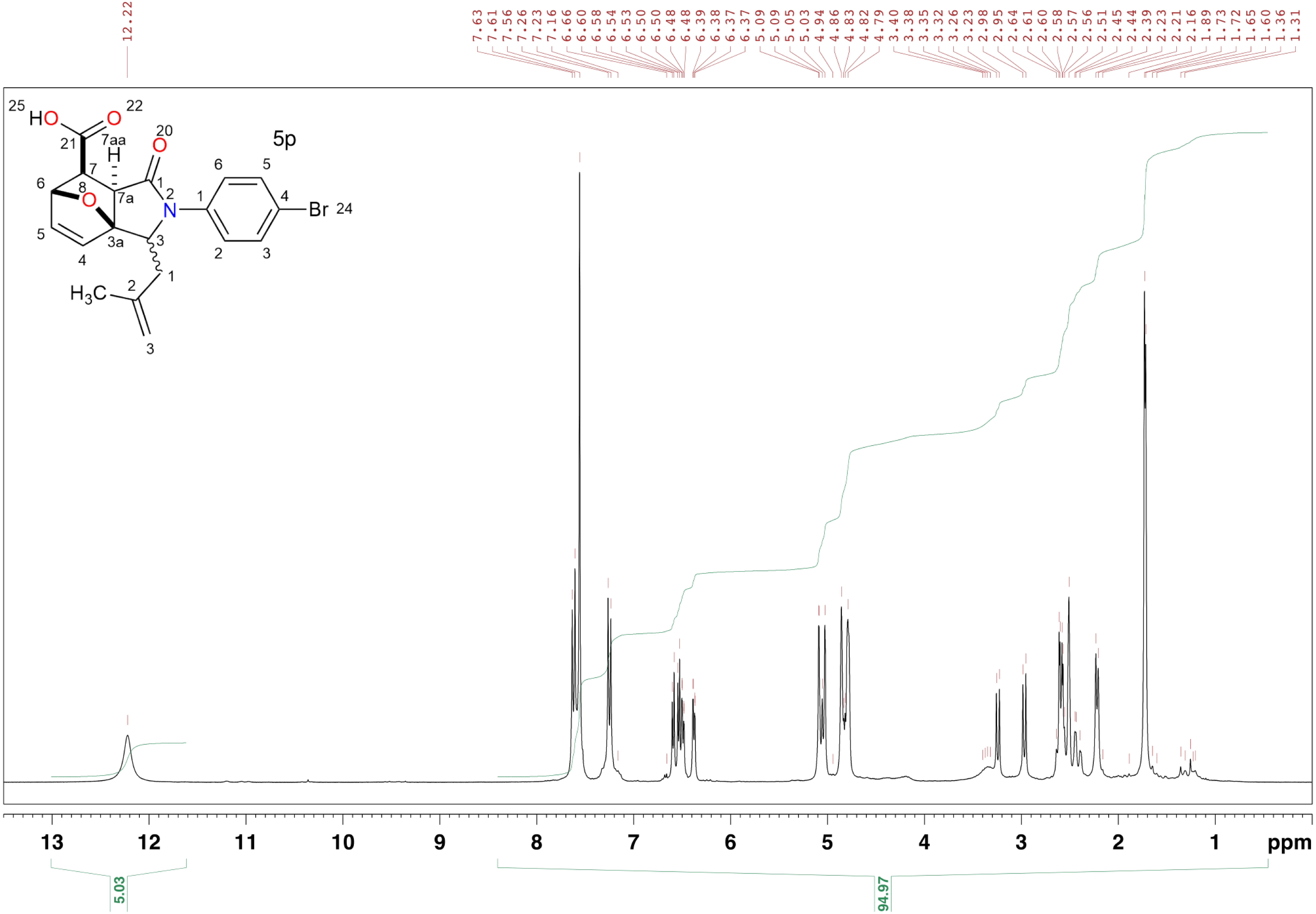

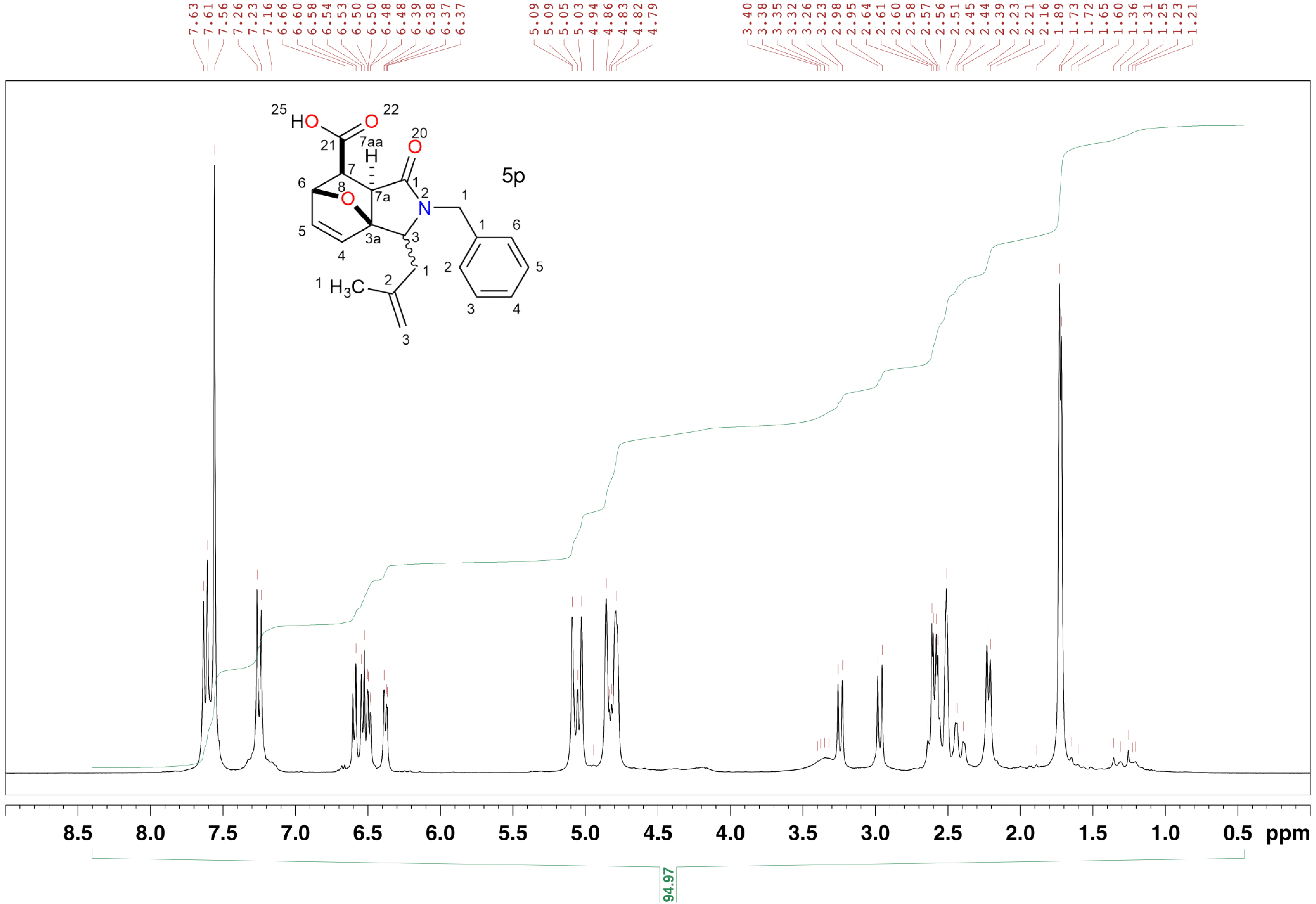

7.63 7.61 7.56 7.26 7.23 7.16 6.66 6.60 6.58 6.54 6.53 6.50 6.50 6.48 6.48 6.39 6.38 6.37 6.37
5.09 5.09 5.05 5.03 4.94 4.86 4.83 4.82 4.79
3.40 3.38 3.35 3.32 3.26 3.23 2.98 2.95 2.64 2.61 2.60 2.58 2.57 2.56 2.51 2.45 2.44 2.39 2.23 2.21 2.16 1.89 1.73 1.72 1.65 1.60 1.36 1.31 1.25 1.23 1.21
5p
HO
O
N
H_3C
8.5 8.0 7.5 7.0 6.5 6.0 5.5 5.0 4.5 4.0 3.5 3.0 2.5 2.0 1.5 1.0 0.5 ppm
94.97

13C NMR spectrum for compound 6p

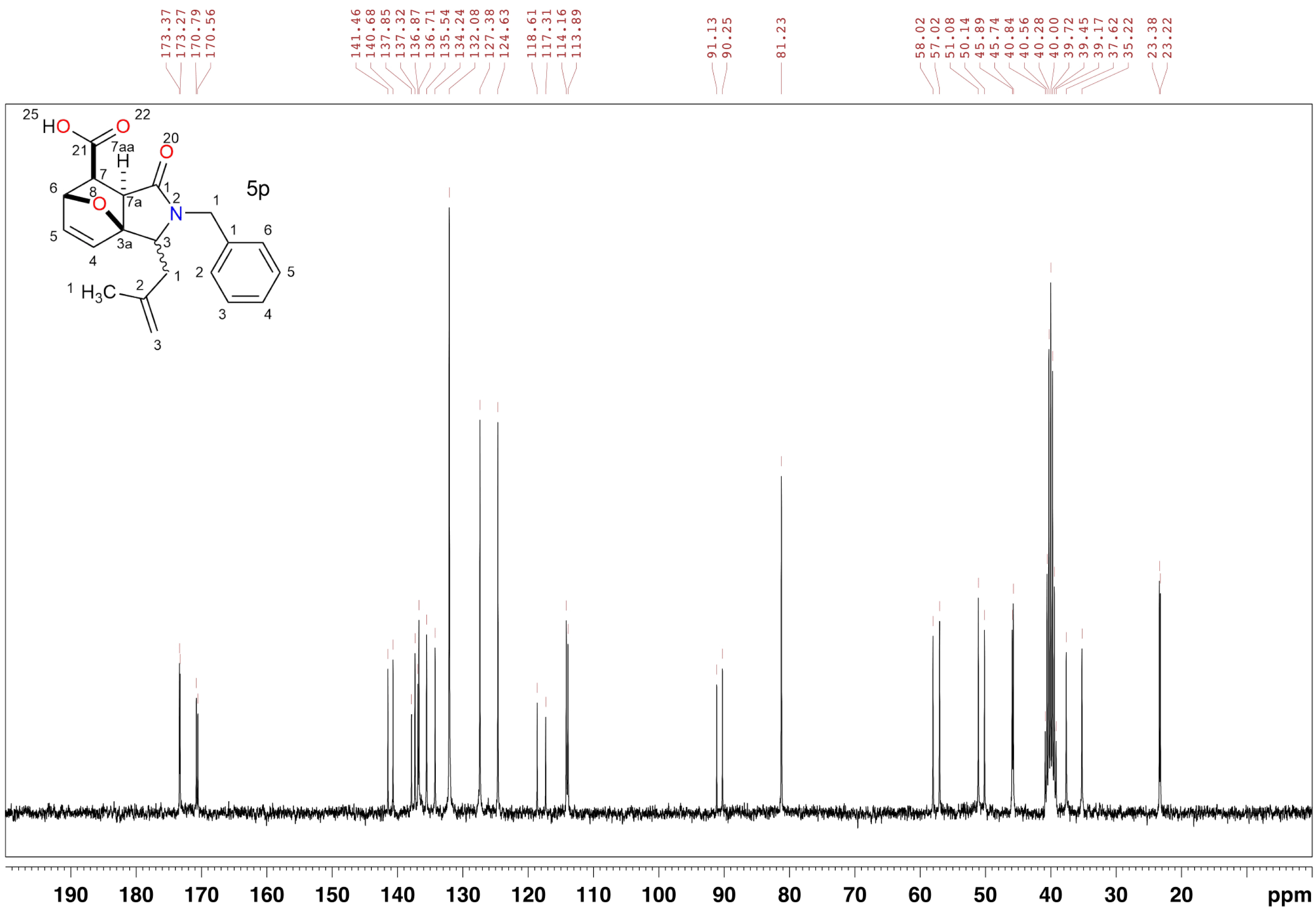

DEPT-135 NMR spectrum for compound 5p

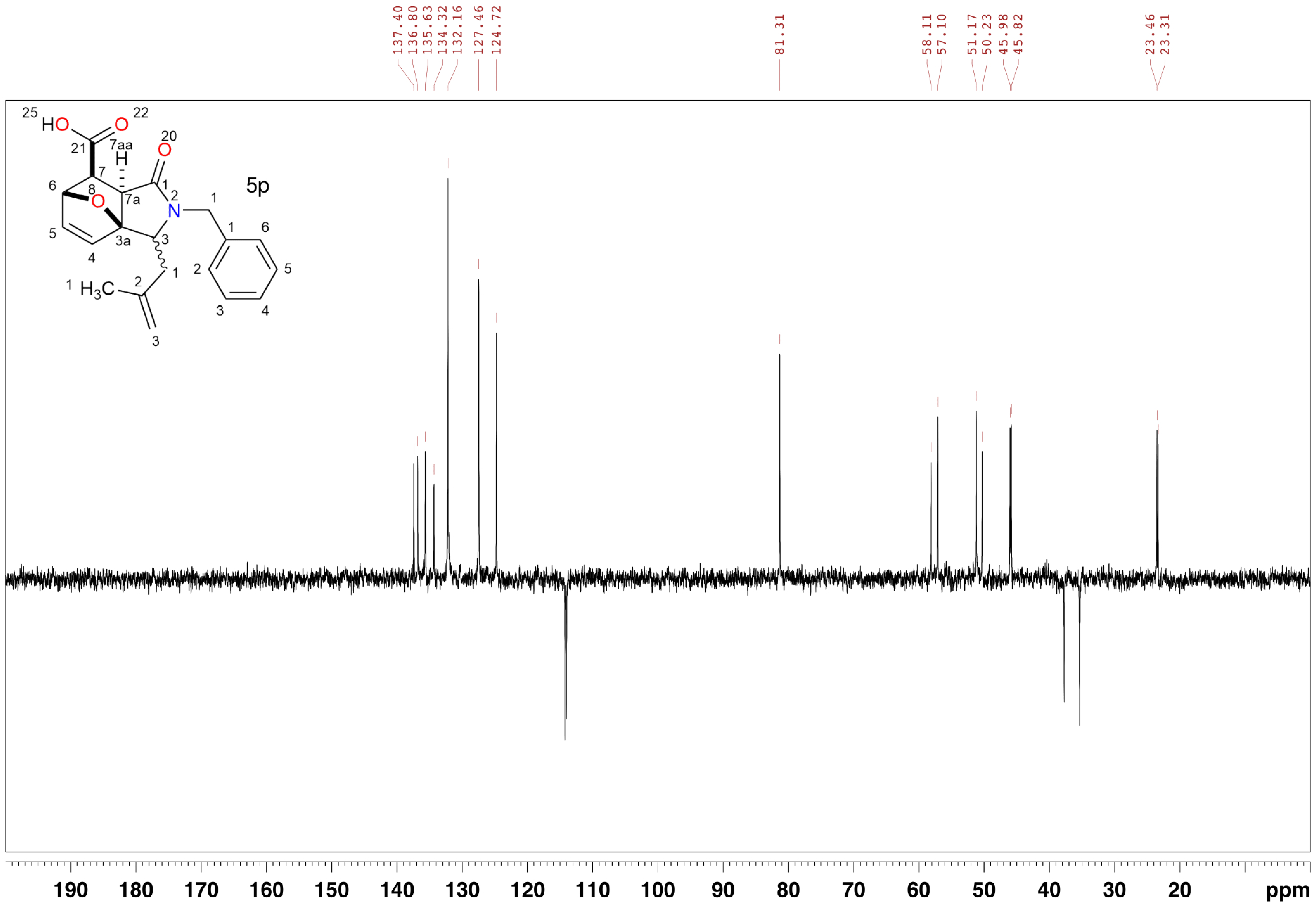

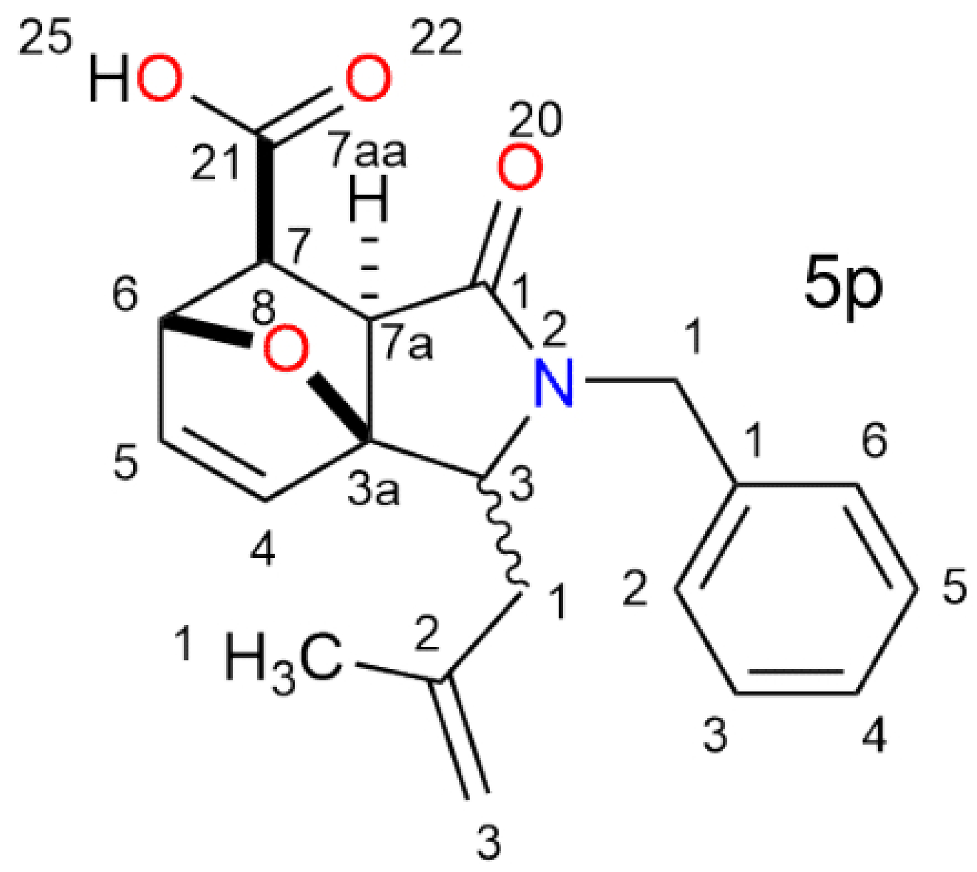

NOESY NMR spectrum for compound 5p

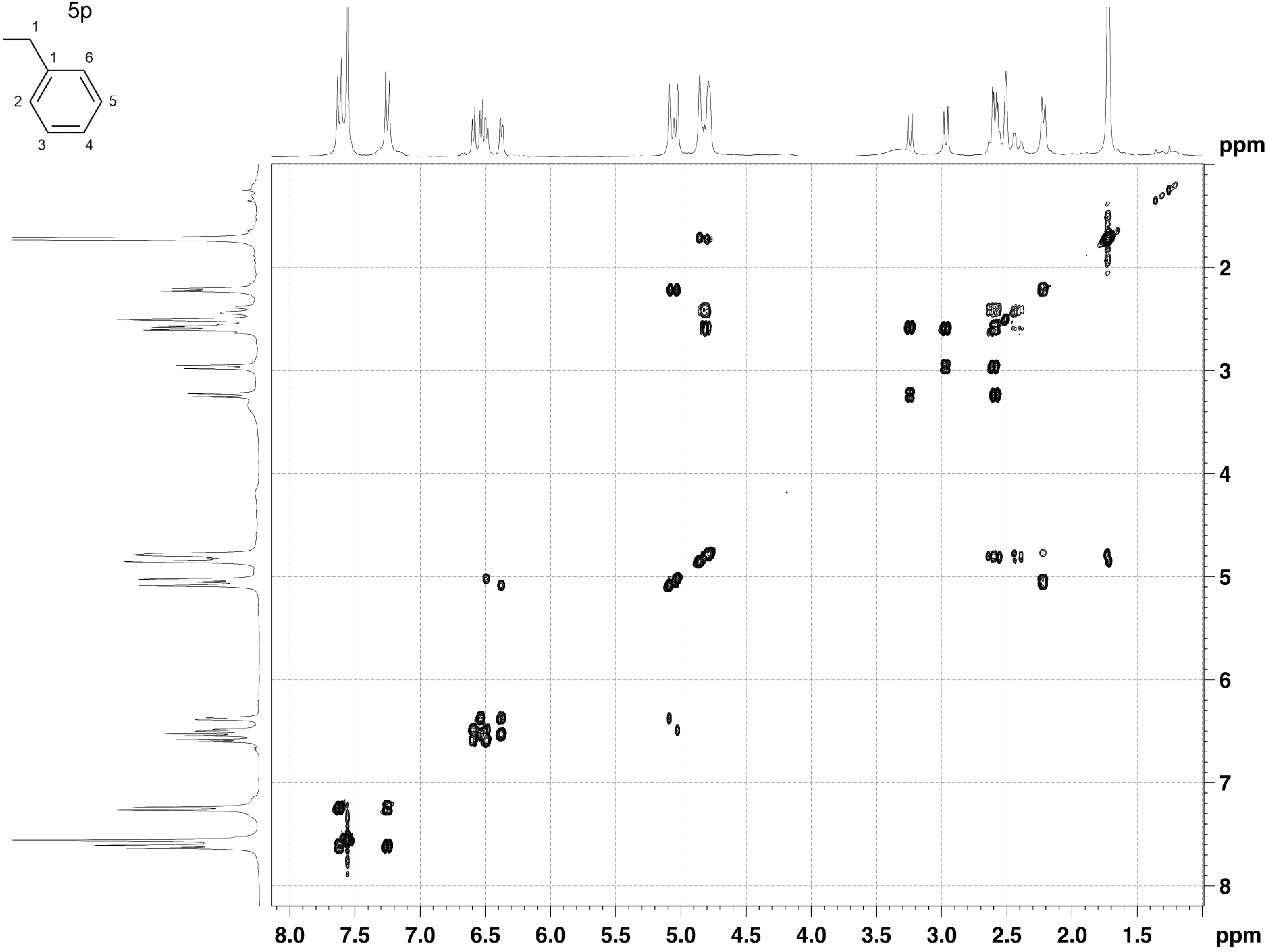

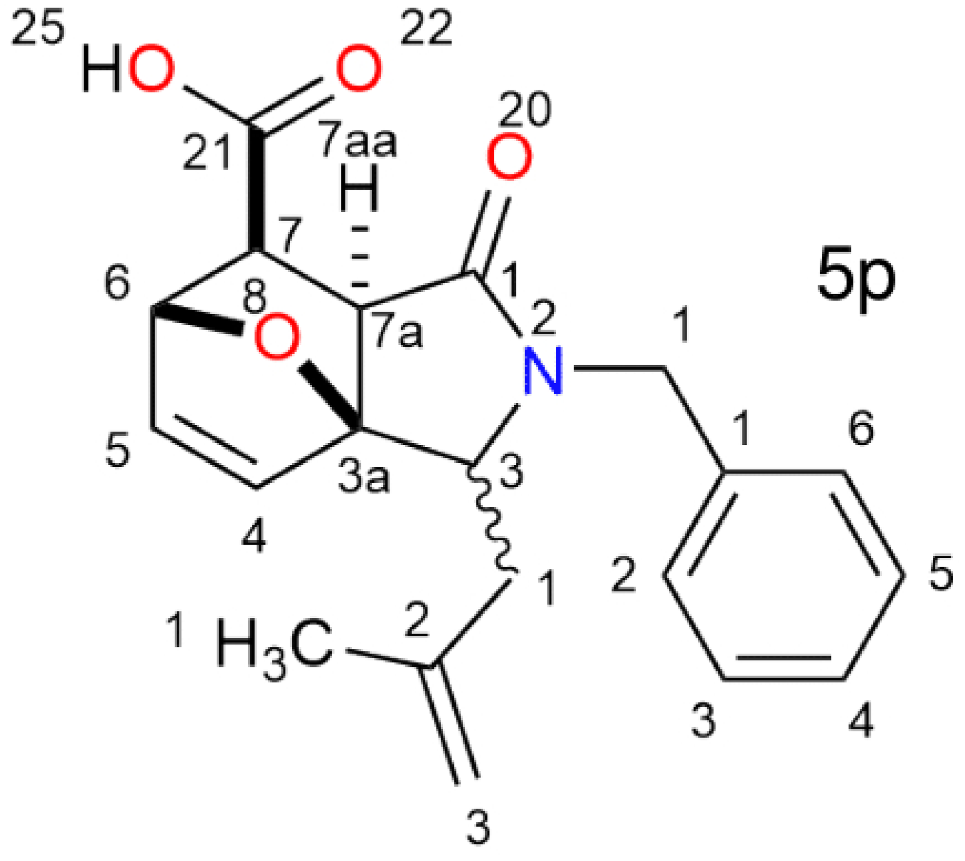

HSQC NMR spectrum for compound 5p

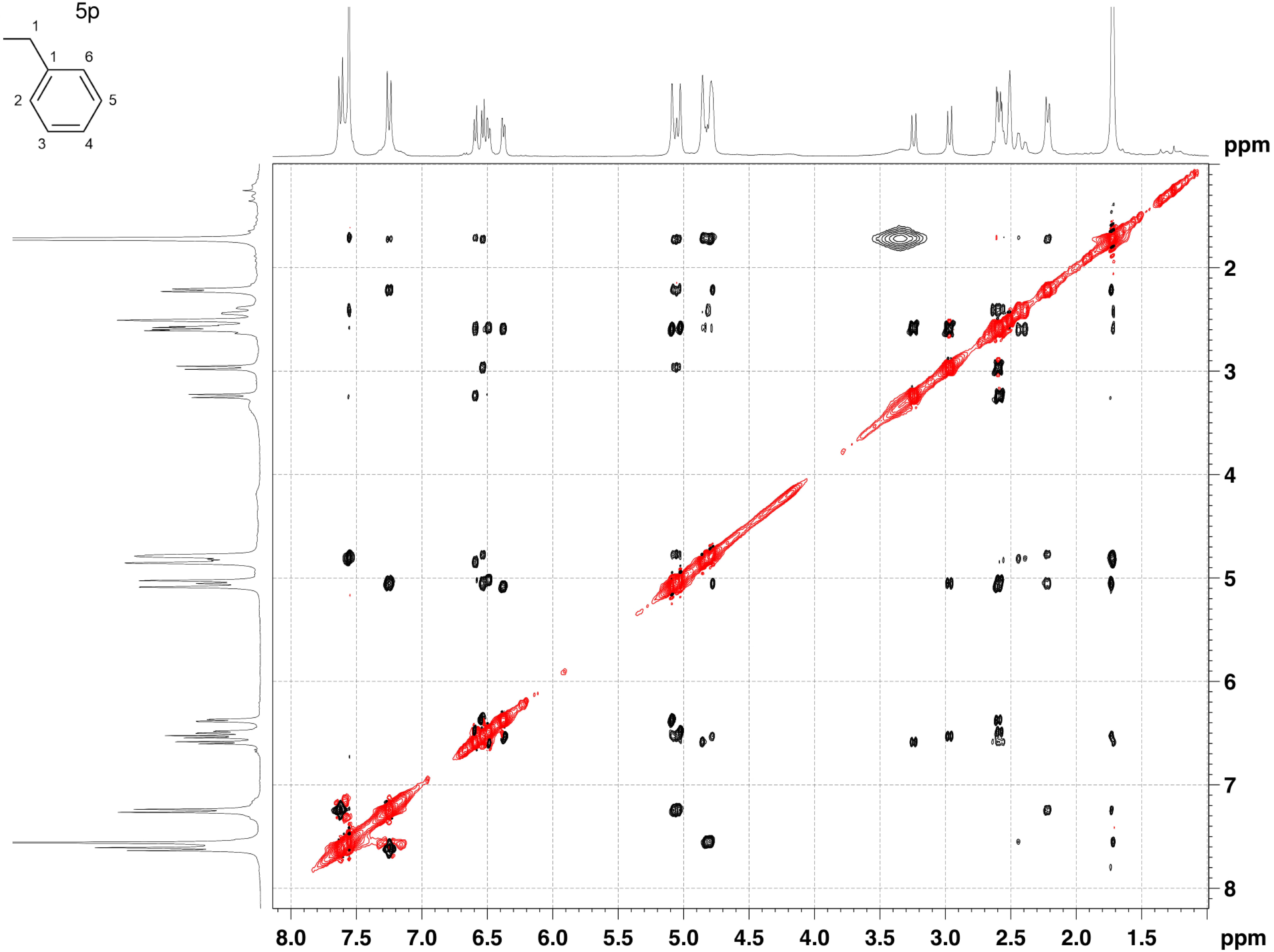

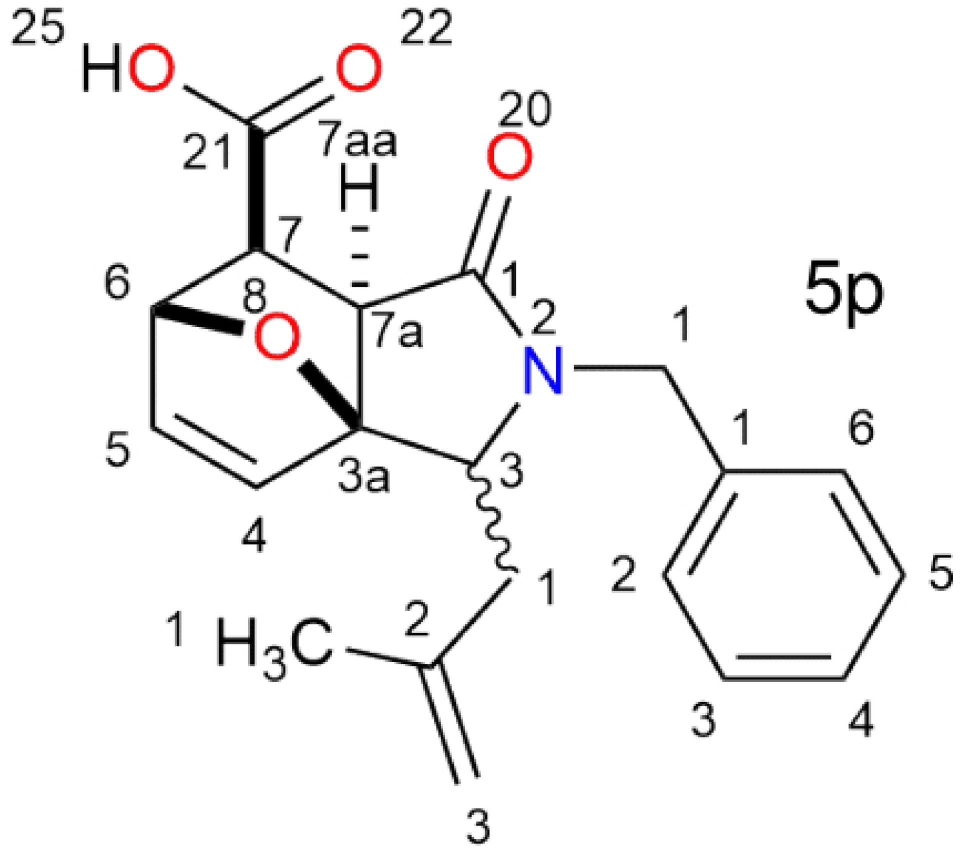

HO
O
O
N
H
H3C

5p

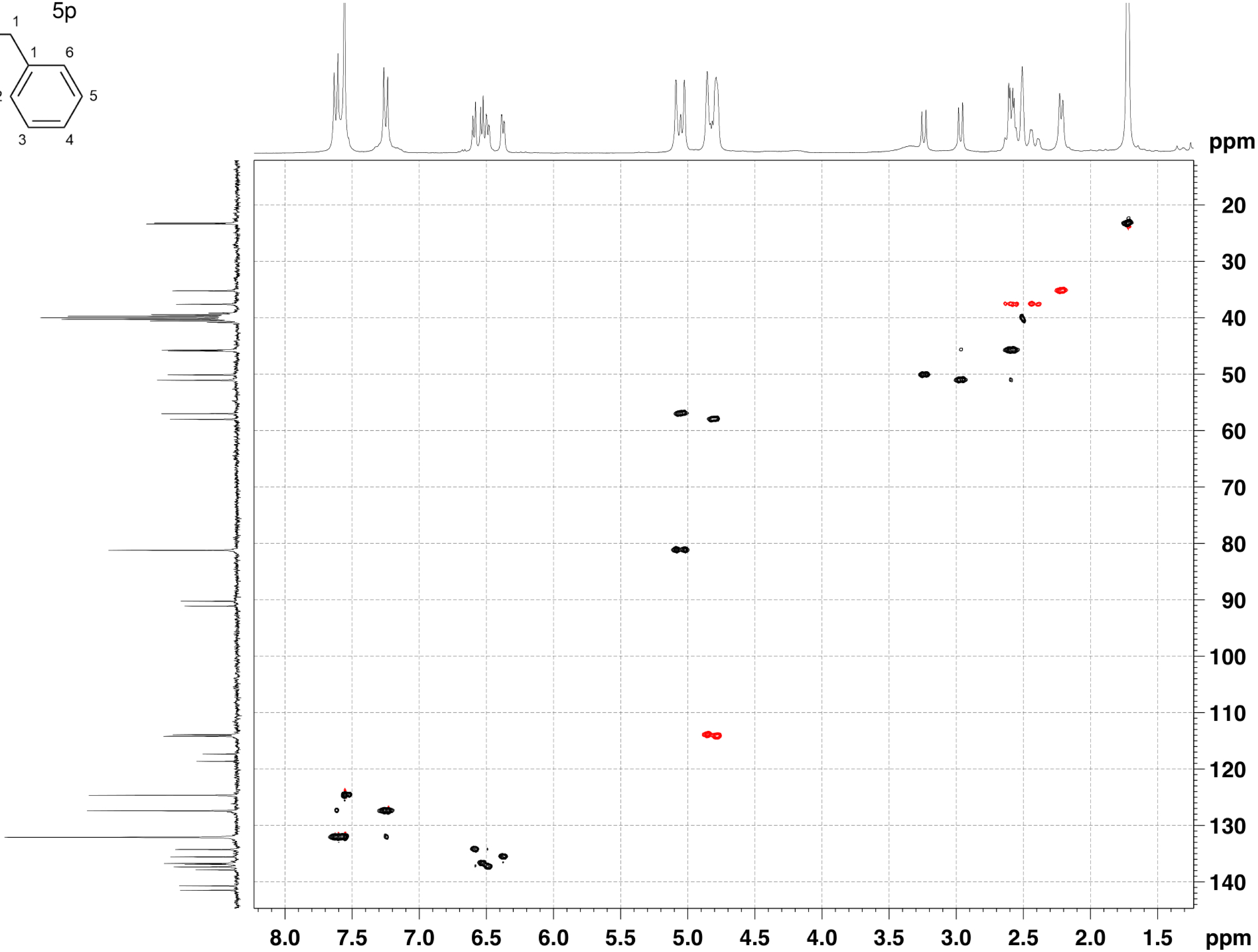

ppm
8.0
7.5
7.0
6.5
6.0
5.5
5.0
4.5
4.0
3.5
3.0
2.5
2.0
1.5
ppm
20
30
40
50
60
70
80
90
100
110
120
130
140

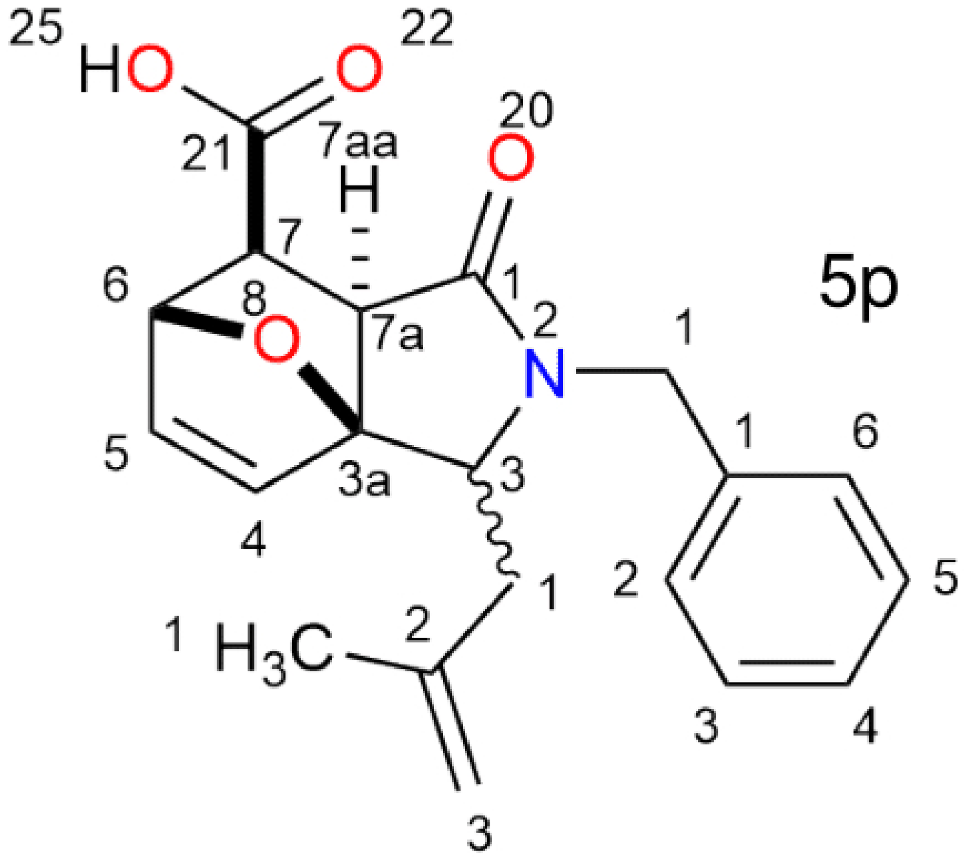

25 HO
22 O
20 O
21
7aa H
7
6
8 O
7a
1
2 N
1
5
3a
3
4
1
2
1 H_3C
3
1
6
2
5
3
4

5p

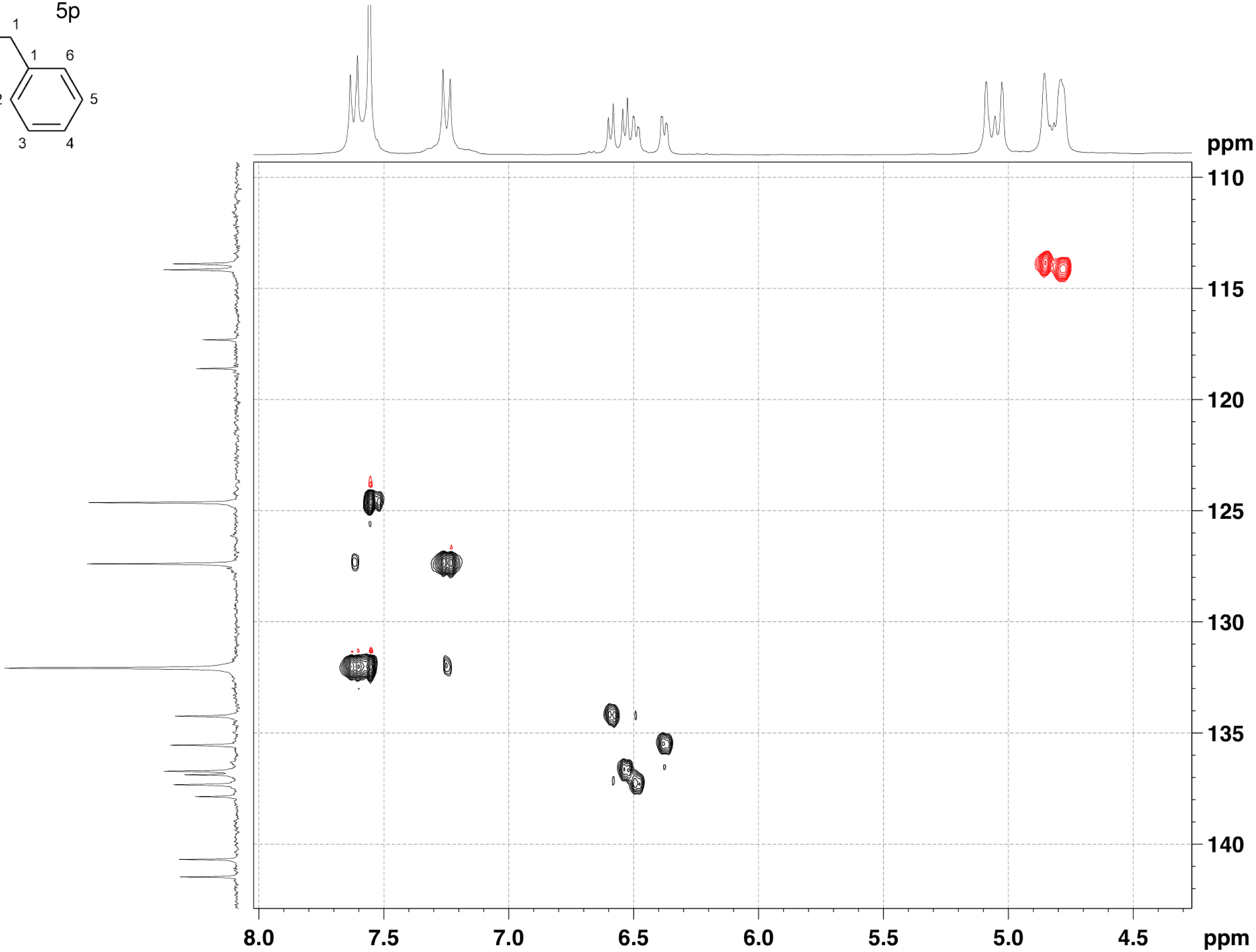

ppm
110
115
120
125
130
135
140
8.0
7.5
7.0
6.5
6.0
5.5
5.0
4.5
ppm

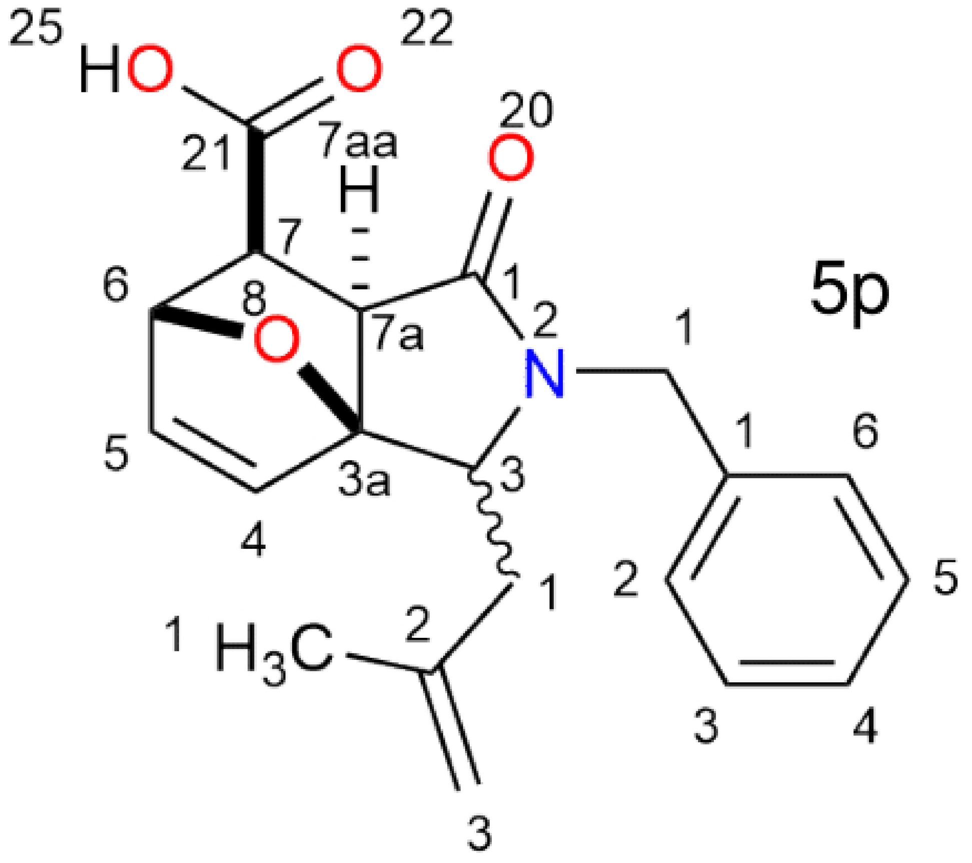
HO
O
O
H
N
O
H3C

5p

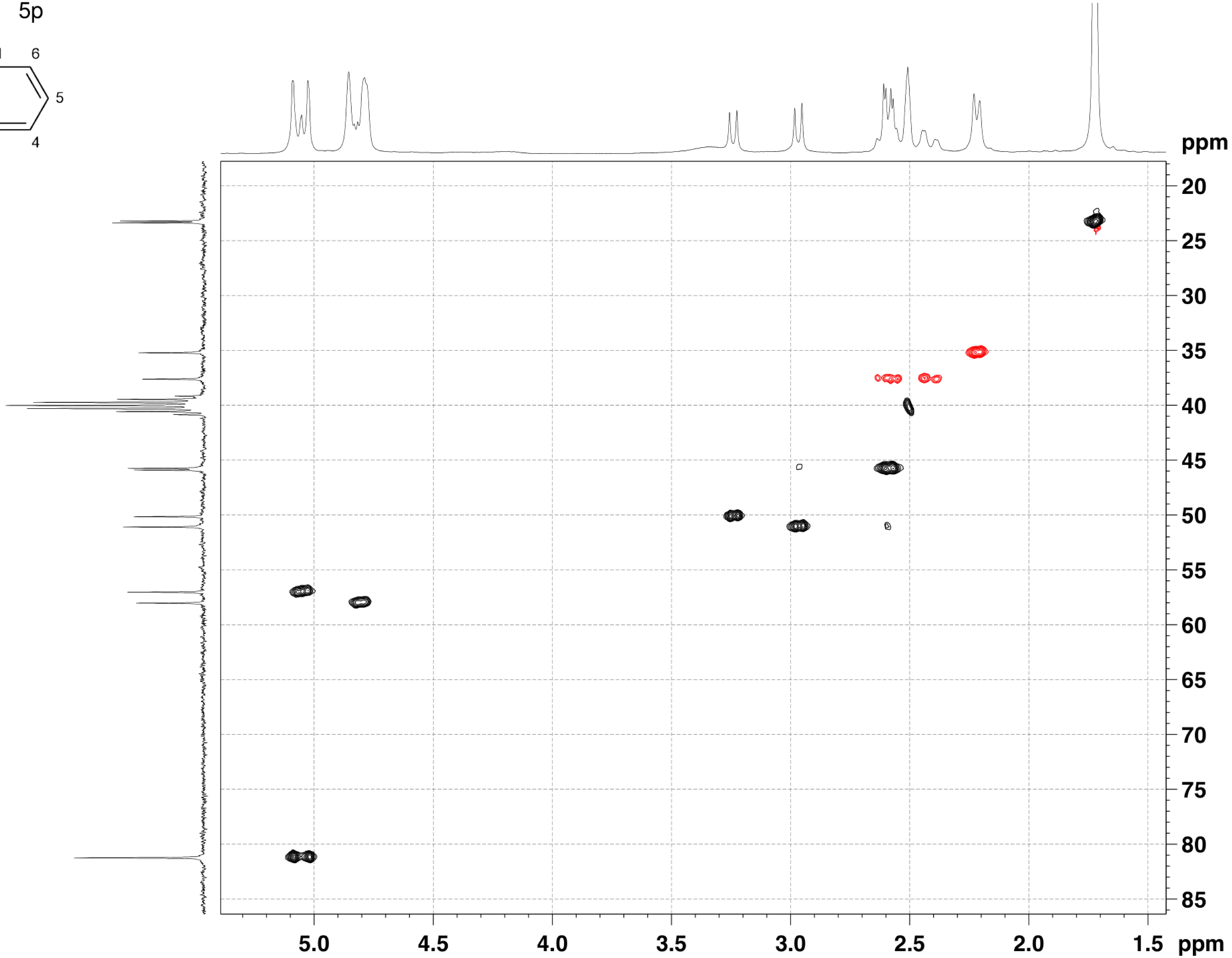
ppm
20
25
30
35
40
45
50
55
60
65
70
75
80
85
5.0
4.5
4.0
3.5
3.0
2.5
2.0
1.5
ppm

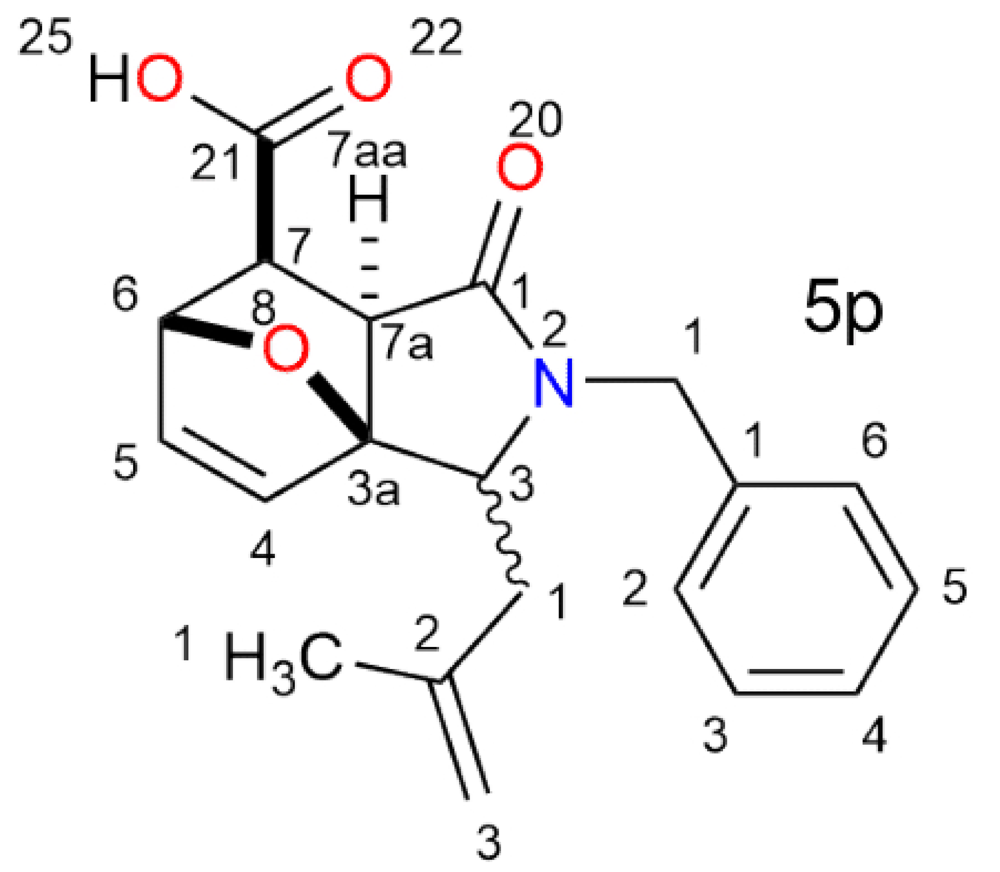

5p

HMBC NMR spectrum for compound 5p

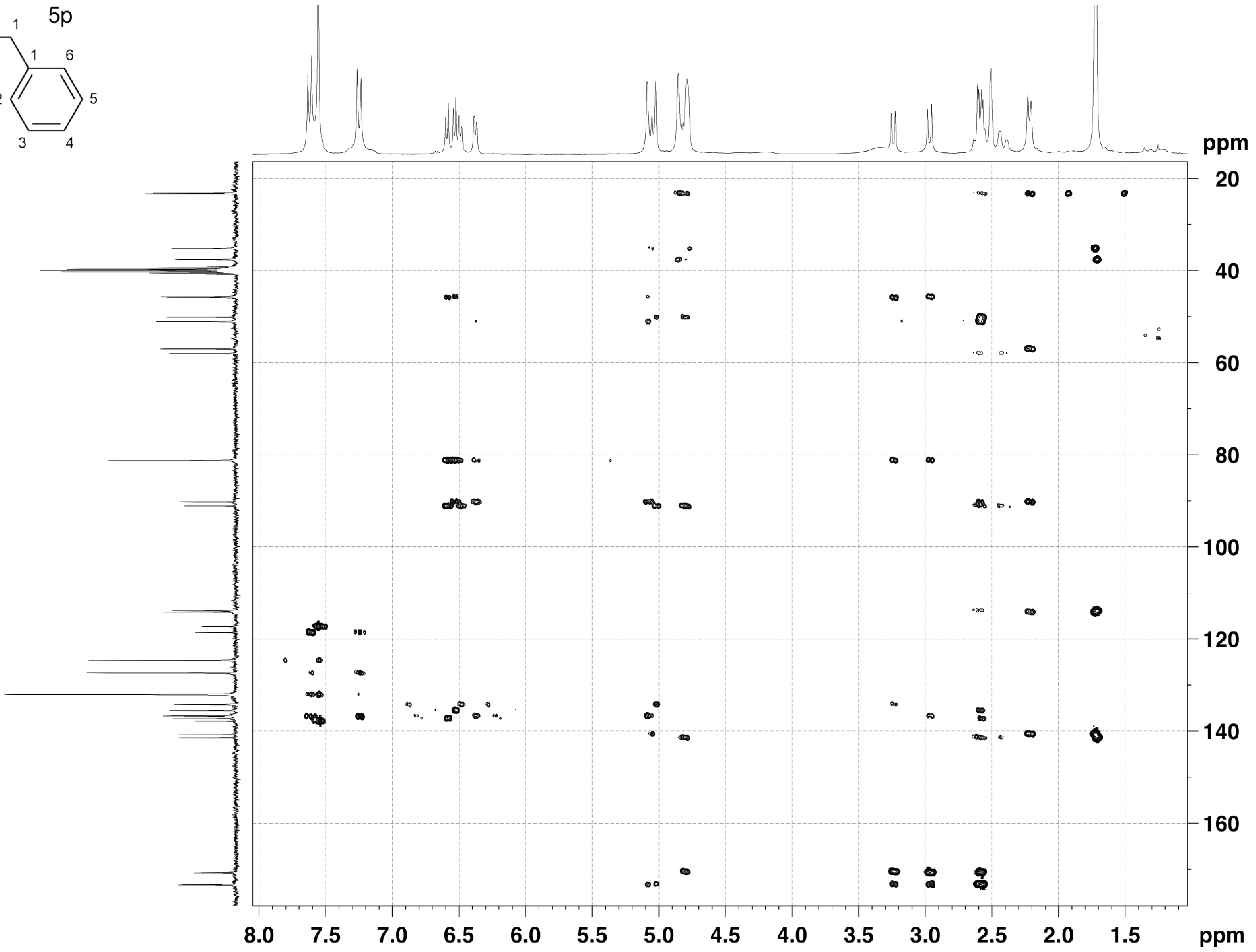

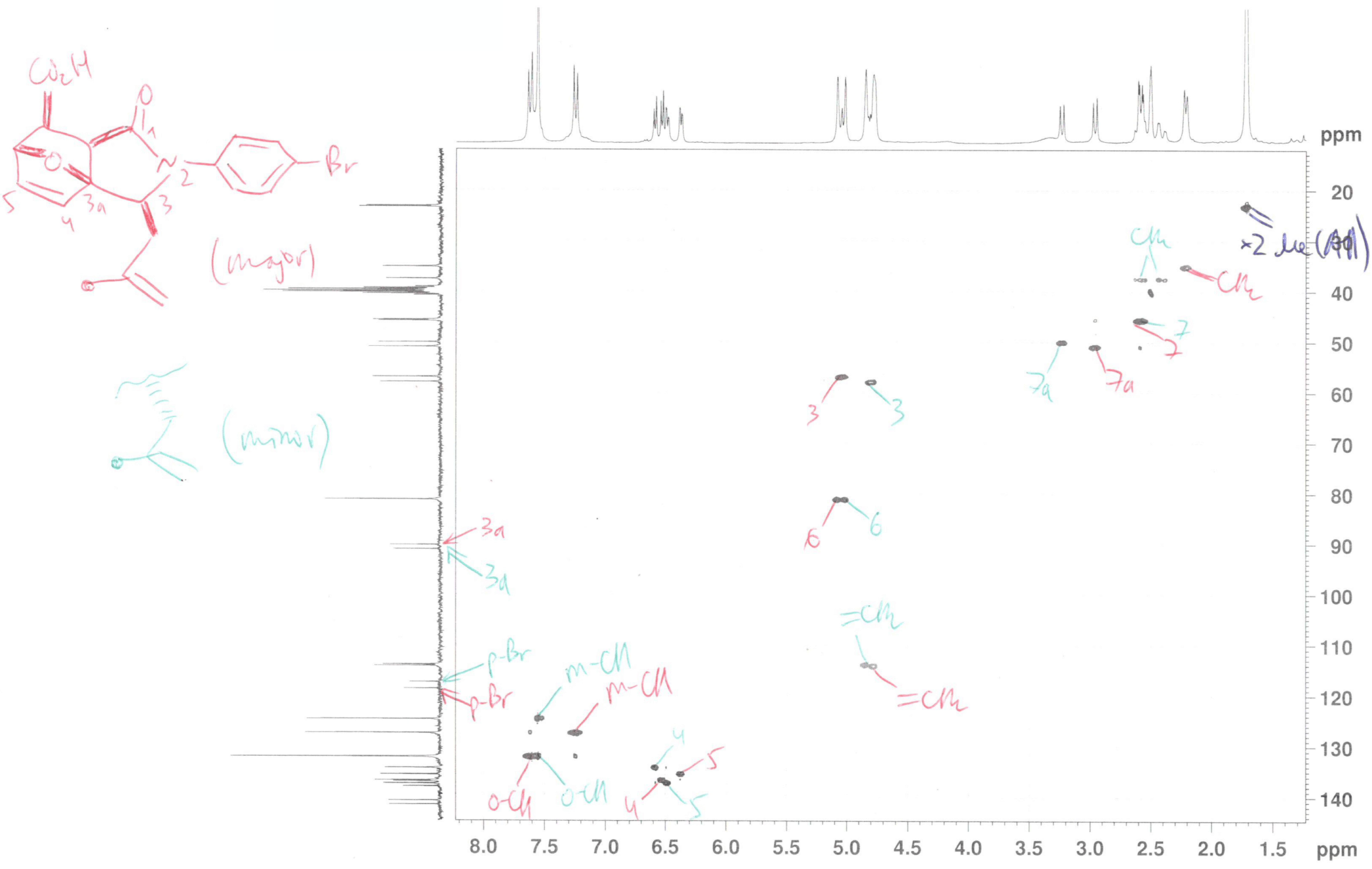
ppm
20
30
40
50
60
70
80
90
100
110
120
130
140
8.0
7.5
7.0
6.5
6.0
5.5
5.0
4.5
4.0
3.5
3.0
2.5
2.0
1.5
ppm

NMR spectrum data for compound 5a (DCM, −16 °C, 3 d)

**Formula**	$C_{18}H_{17}NO_4$	**FW**	311.3319

**Acquisition Time (sec)**	1.9818	**Comment**	single_pulse	**Date**	07 Jul 2020 11:42:25		
**Date Stamp**	07 Jul 2020 11:43:56			**File Name**	F:\NMR FIDS ACIDS\PN ALLYL\FZ8740 1.PH.JDF		
**Frequency (MHz)**	600.17	**Nucleus**	1H	**Number of Transients**	8	**Origin**	ECA 600
**Original Points Count**	32768	**Owner**	CKP	**Points Count**	32768	**Pulse Sequence**	single_pulse.ex2
**Receiver Gain**	42.00	**Solvent**	CHLOROFORM-d			**Spectrum Offset (Hz)**	5401.5503
**Sweep Width (Hz)**	16534.39	**Temperature (degree C)**	22.900				

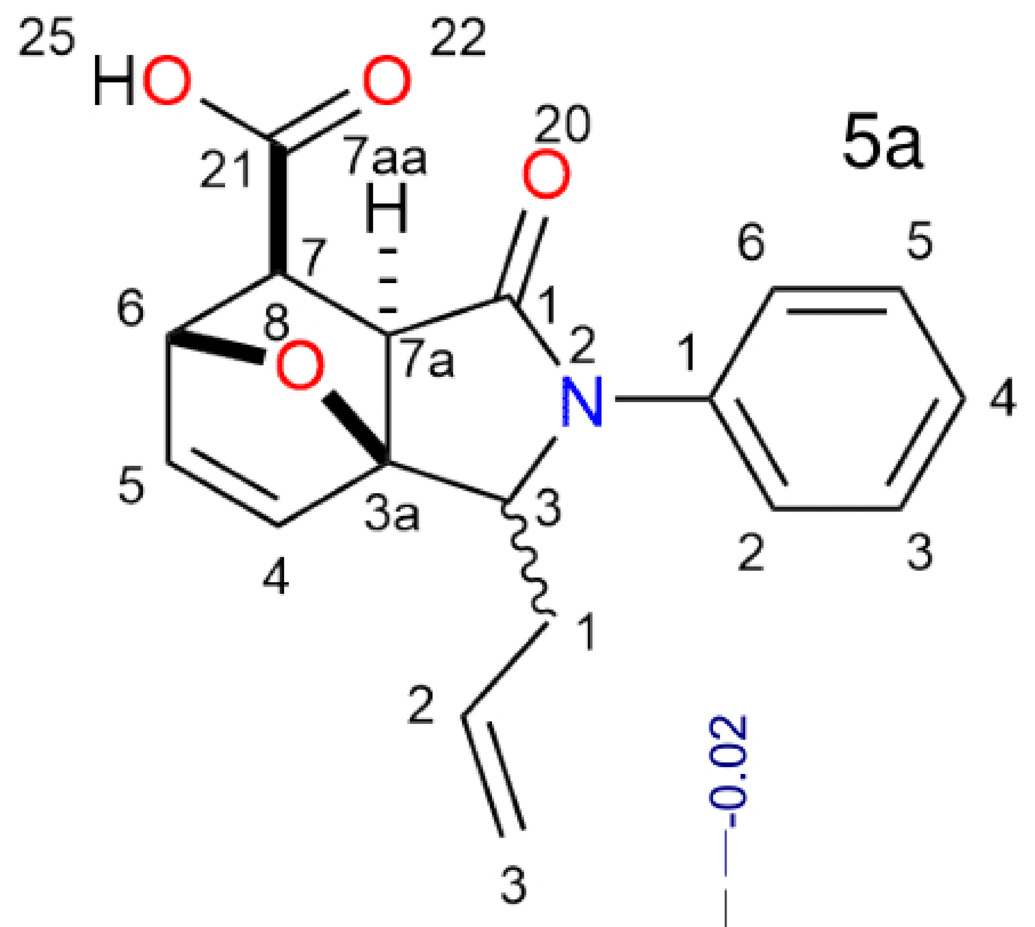

FZ8740 1.PH.JDF

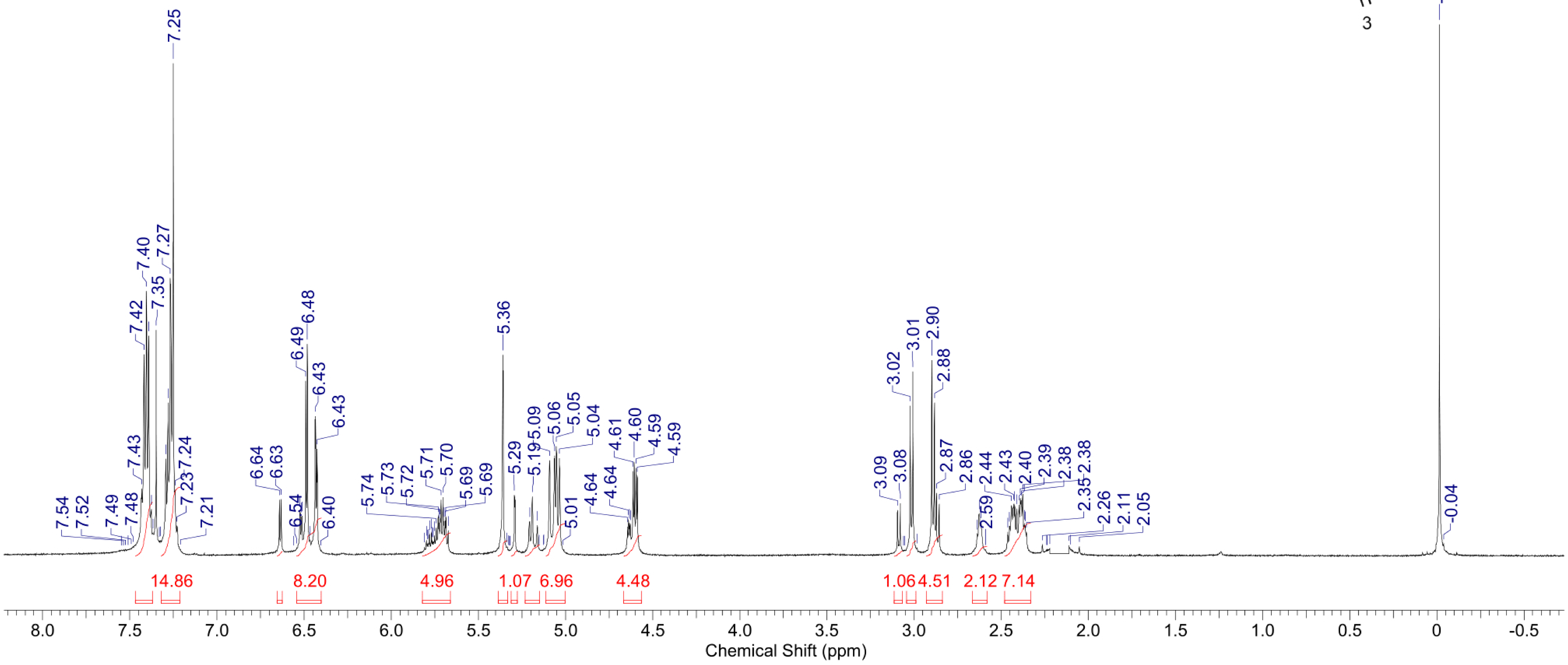

Formula $C_{18}H_{17}NO_4$	***FW***	311.3319	

Acquisition Time (sec)	1.9818	***Comment***	single_pulse	***Date***	07 Jul 2020 11:42:25		
Date Stamp	07 Jul 2020 11:43:56			***File Name***	F:\NMR FIDS ACIDS\PN ALLYL\FZ8740 1.PH.JDF		
Frequency (MHz)	600.17	***Nucleus***	1H	***Number of Transients***	8	***Origin***	ECA 600
Original Points Count	32768	***Owner***	CKP	***Points Count***	32768	***Pulse Sequence***	single_pulse.ex2
Receiver Gain	42.00	***Solvent***	CHLOROFORM-d			***Spectrum Offset (Hz)***	5401.5503
Sweep Width (Hz)	16534.39	***Temperature (degree C)***	22.900				

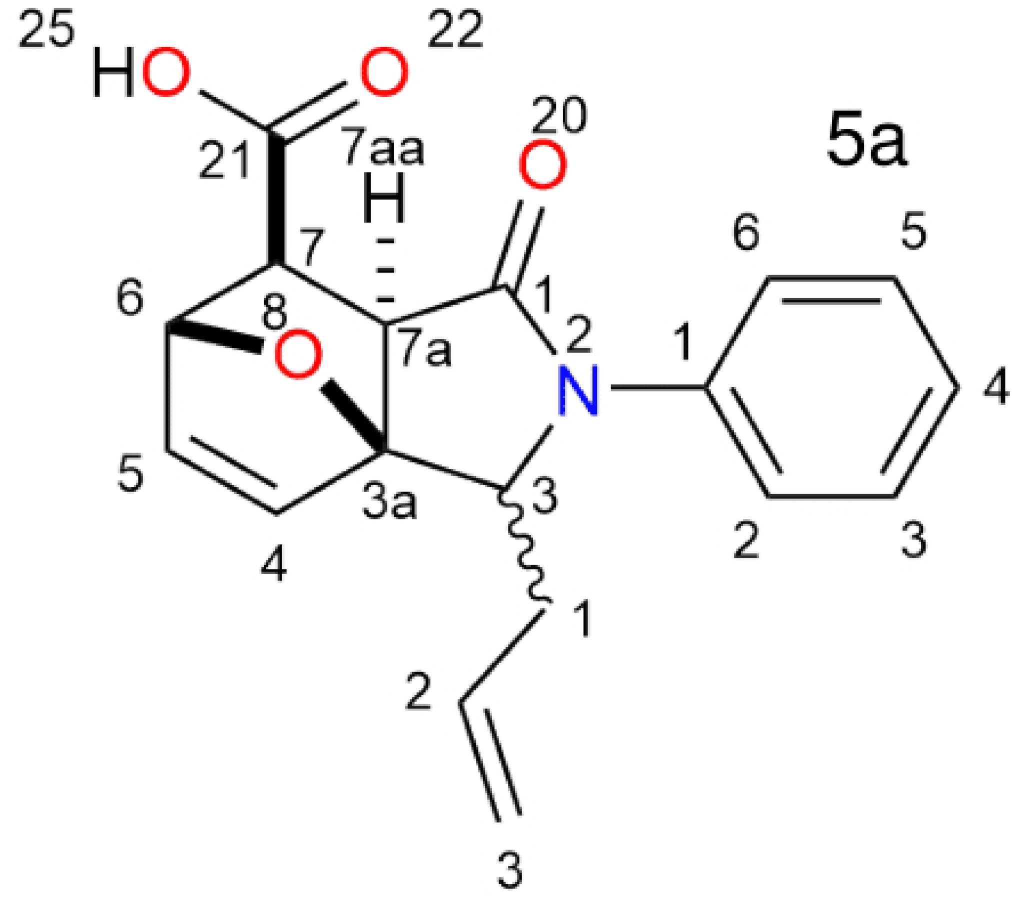

5a

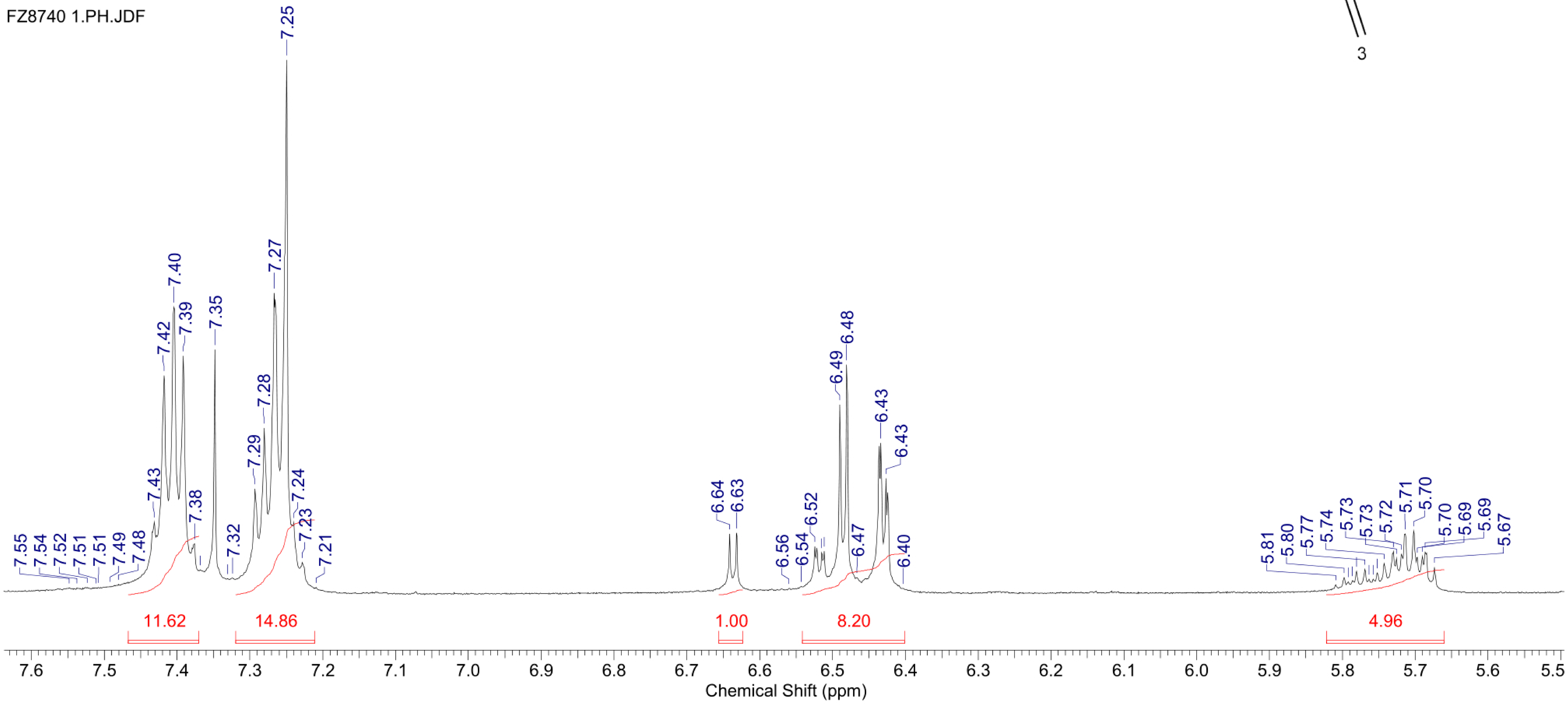

Formula $C_{18}H_{17}NO_4$	FW 311.3319

Acquisition Time (sec)	1.9818	***Comment***	single_pulse	***Date***	07 Jul 2020 11:42:25		
Date Stamp	07 Jul 2020 11:43:56			***File Name***	F:\NMR FIDS ACIDS\PN ALLYL\FZ8740 1.PH.JDF		
Frequency (MHz)	600.17	***Nucleus***	1H	***Number of Transients***	8	***Origin***	ECA 600
Original Points Count	32768	***Owner***	CKP	***Points Count***	32768	***Pulse Sequence***	single_pulse.ex2
Receiver Gain	42.00	***Solvent***	CHLOROFORM-d			***Spectrum Offset (Hz)***	5401.5503
Sweep Width (Hz)	16534.39	***Temperature (degree C)***	22.900				

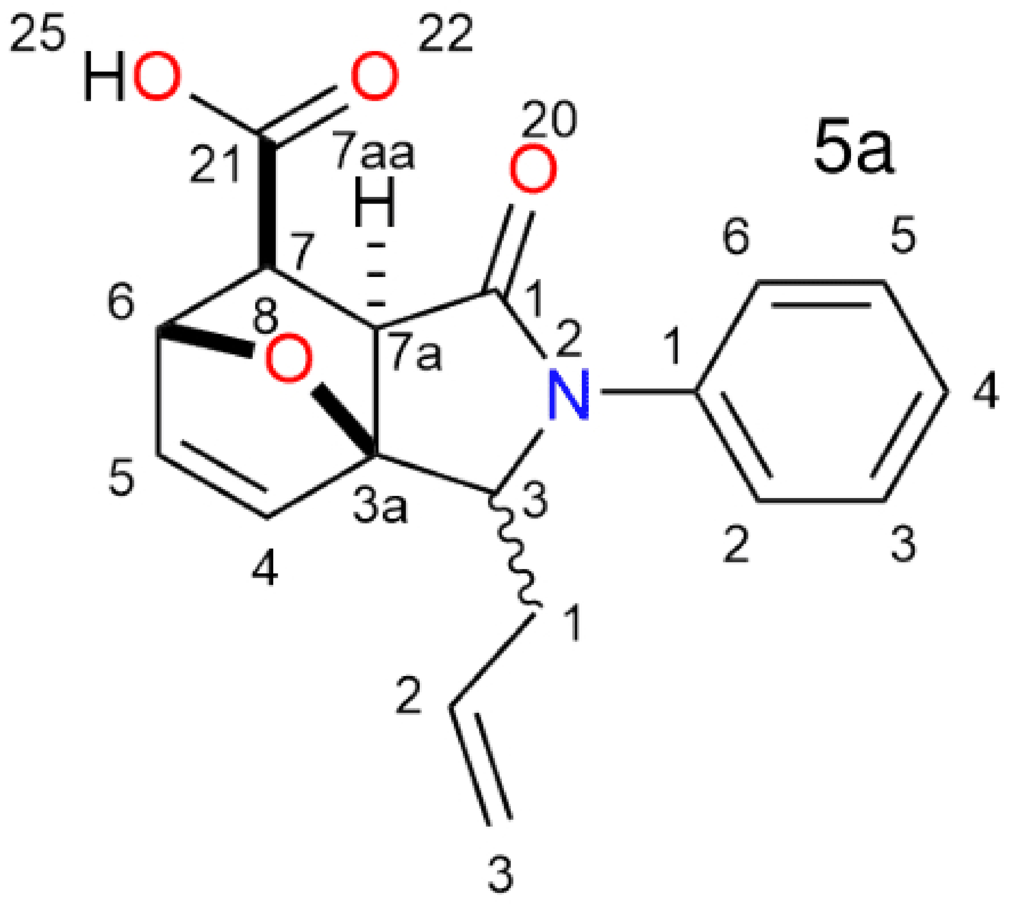

FZ8740 1.PH.JDF

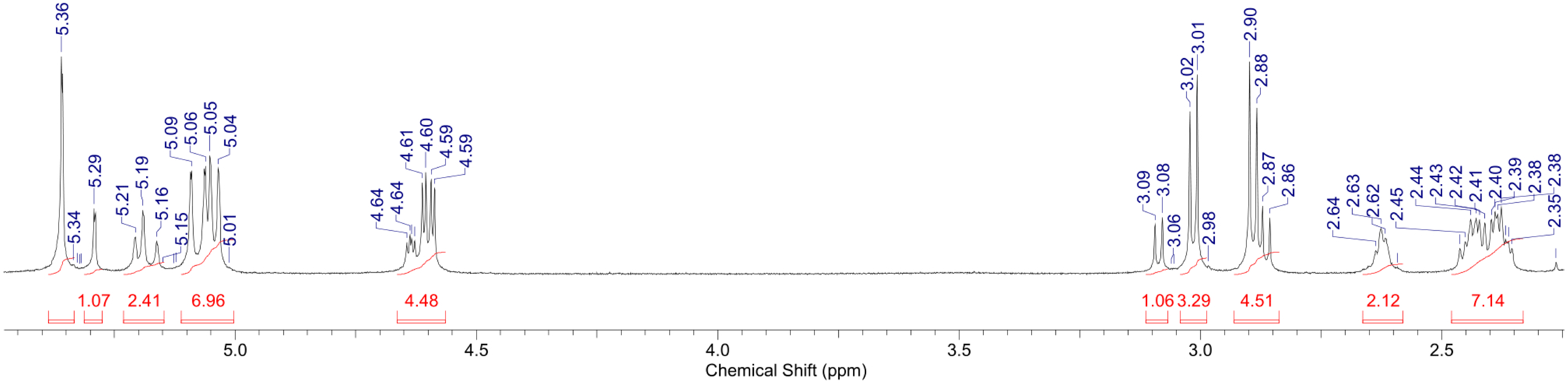

NMR spectrum data for compound 5a (DCM, r.t, 3 d)

**Formula** $C_{18}H_{17}NO_4$	**FW** 311.3319

Acquisition Time (sec)	1.9818	***Comment***	single_pulse	***Date***	07 Jul 2020 11:48:28		
Date Stamp	07 Jul 2020 11:50:00			***File Name***	F:\NMR FIDS ACIDS\PN ALLYL\FZ8741 2.PH.JDF		
Frequency (MHz)	600.17	***Nucleus***	1H	***Number of Transients***	8	***Origin***	ECA 600
Original Points Count	32768	***Owner***	CKP	***Points Count***	32768	***Pulse Sequence***	single_pulse.ex2
Receiver Gain	48.00	***Solvent***	CHLOROFORM-d			***Spectrum Offset (Hz)***	5410.1226
Sweep Width (Hz)	16534.39	***Temperature (degree C)***	22.900				

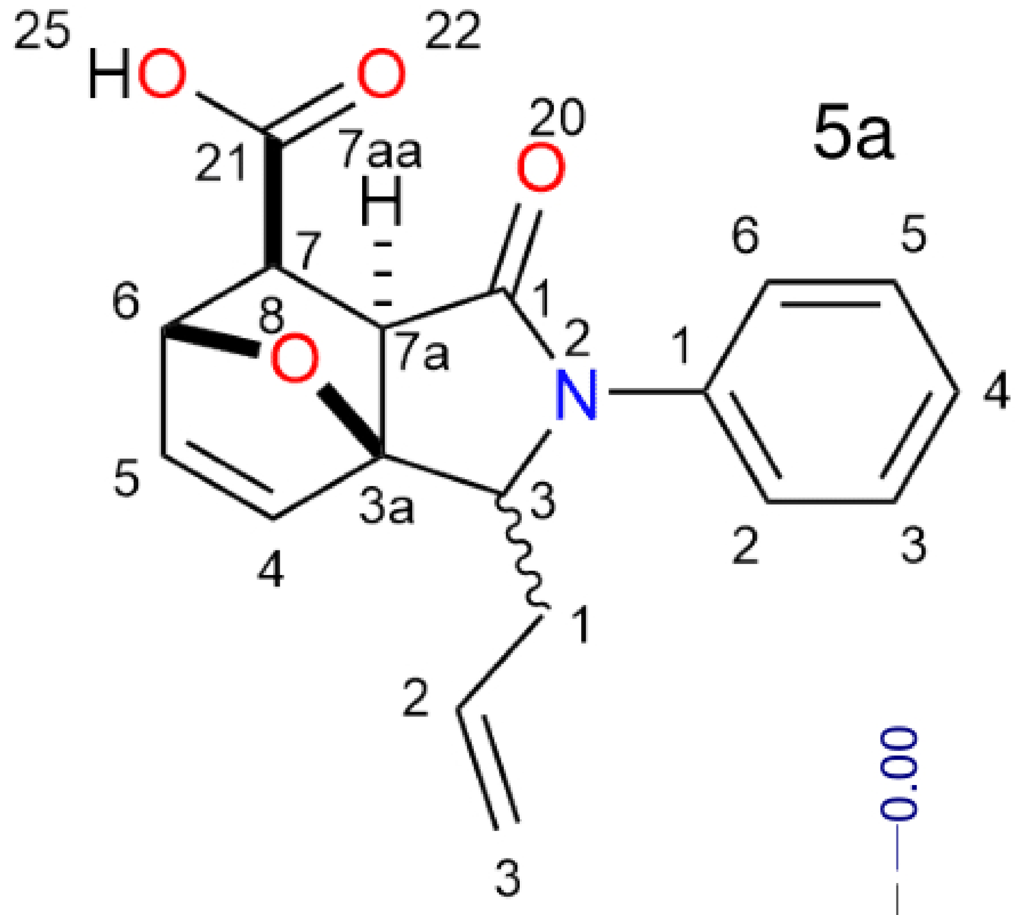

5a

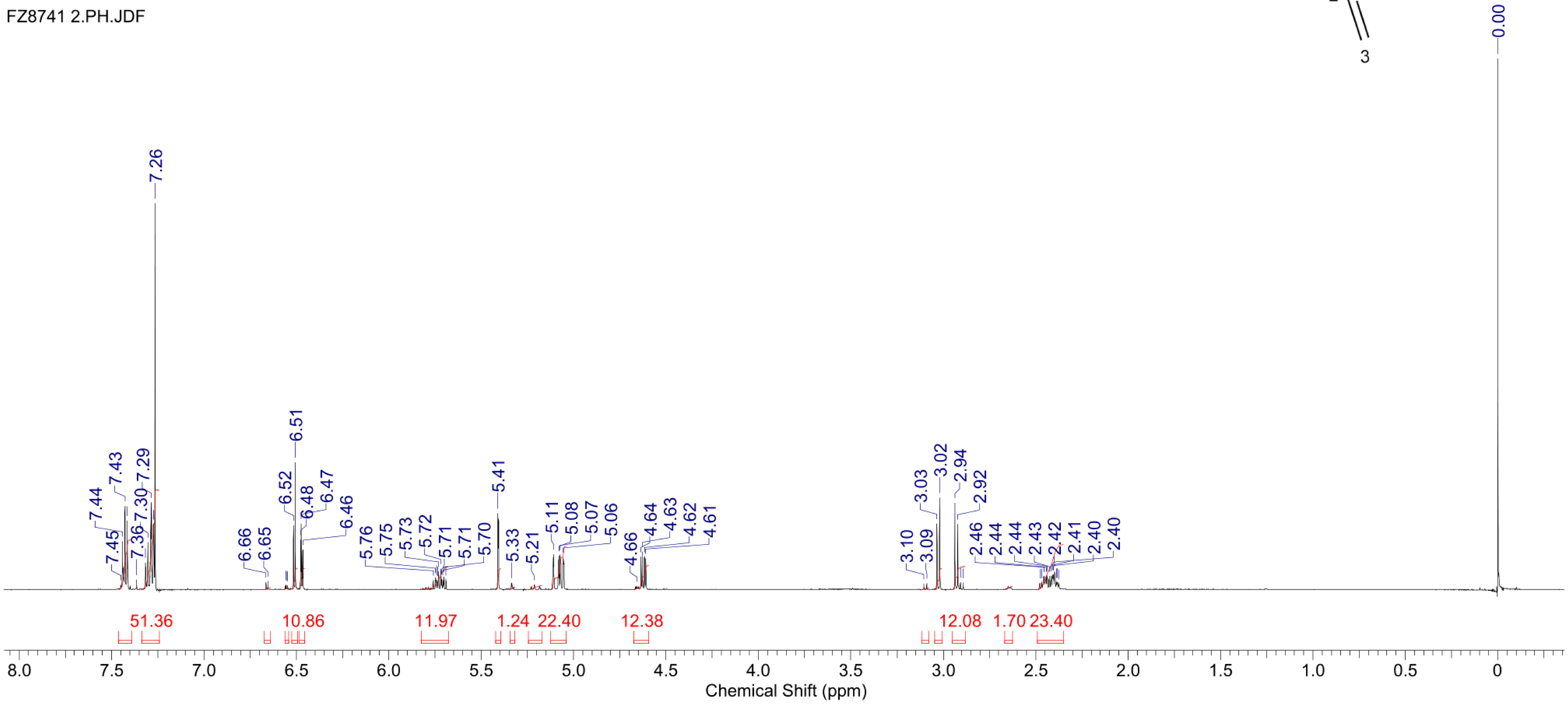

**Formula** $C_{18}H_{17}NO_4$	**FW** 311.3319

**Acquisition Time (sec)**	1.9818	**Comment**	single_pulse	**Date**	07 Jul 2020 11:48:28		
**Date Stamp**	07 Jul 2020 11:50:00			**File Name**	F:\NMR FIDS ACIDS\PN ALLYL\FZ8741 2.PH.JDF		
**Frequency (MHz)**	600.17	**Nucleus**	1H	**Number of Transients**	8	**Origin**	ECA 600
**Original Points Count**	32768	**Owner**	CKP	**Points Count**	32768	**Pulse Sequence**	single_pulse.ex2
**Receiver Gain**	48.00	**Solvent**	CHLOROFORM-d			**Spectrum Offset (Hz)**	5410.1226
**Sweep Width (Hz)**	16534.39	**Temperature (degree C)**	22.900				

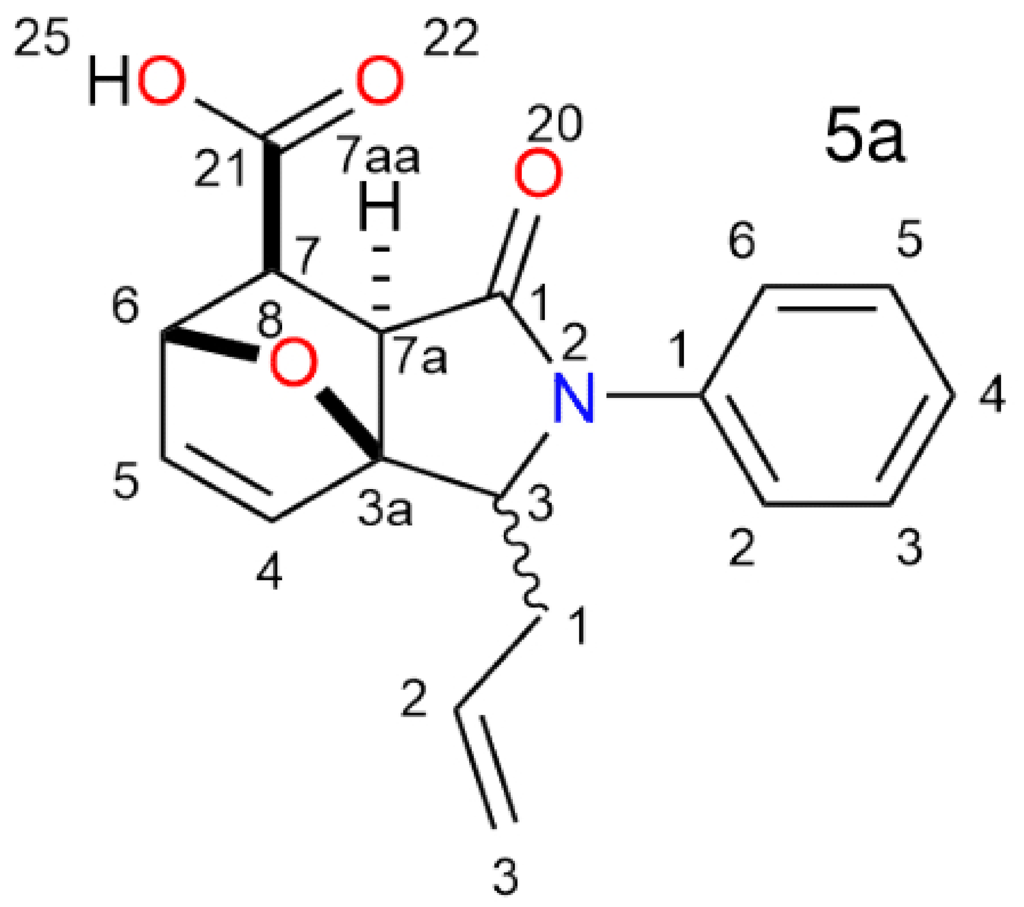

5a

FZ8741 2.PH.JDF

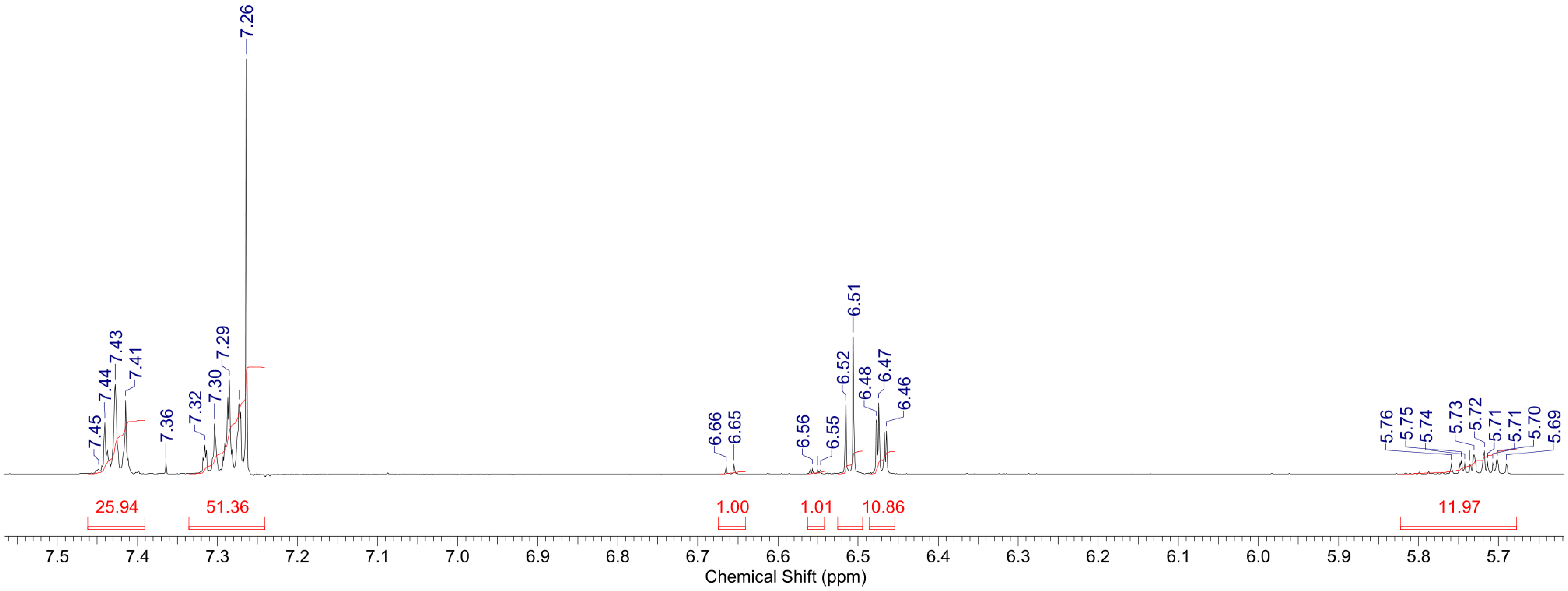

Formula $C_{18}H_{17}NO_4$	FW 311.3319

Acquisition Time (sec)	1.9818	***Comment***	single_pulse	***Date***	07 Jul 2020 11:48:28		
Date Stamp	07 Jul 2020 11:50:00			***File Name***	F:\NMR FIDS ACIDS\PN ALLYL\FZ8741 2.PH.JDF		
Frequency (MHz)	600.17	***Nucleus***	1H	***Number of Transients***	8	***Origin***	ECA 600
Original Points Count	32768	***Owner***	CKP	***Points Count***	32768	***Pulse Sequence***	single_pulse.ex2
Receiver Gain	48.00	***Solvent***	CHLOROFORM-d			***Spectrum Offset (Hz)***	5410.1226
Sweep Width (Hz)	16534.39	***Temperature (degree C)***	22.900				

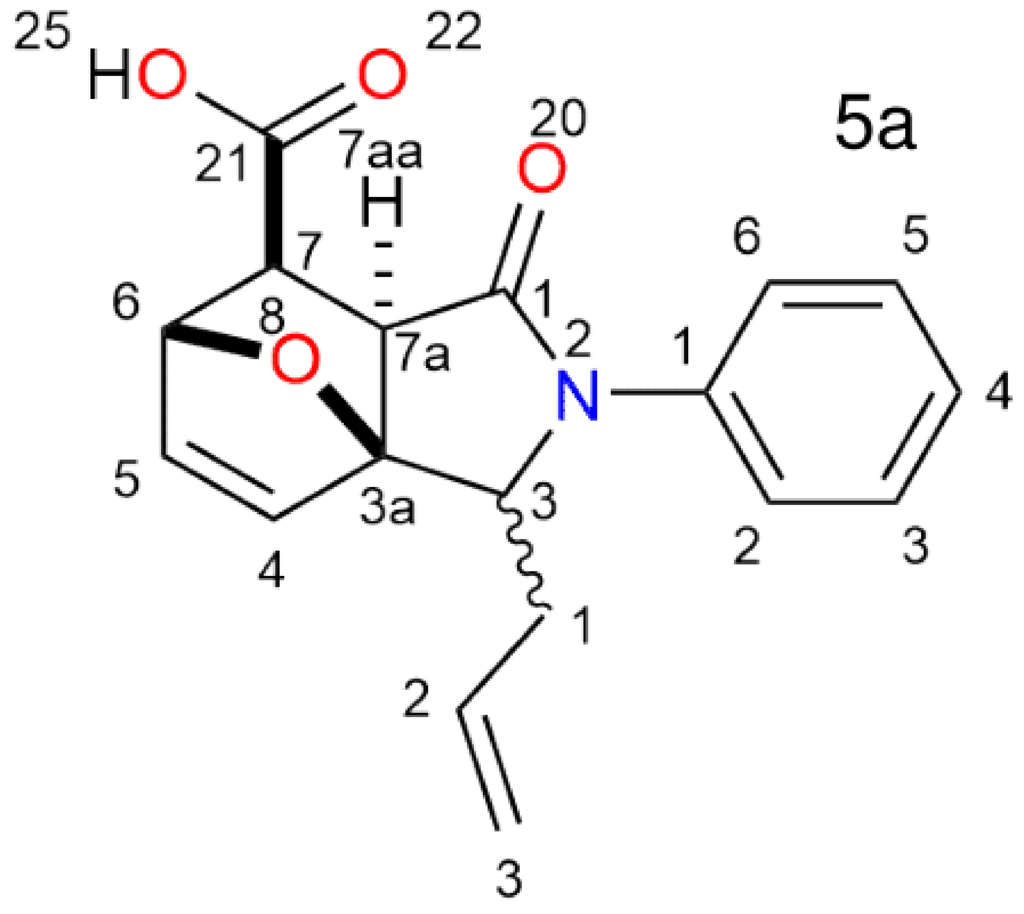

5a

FZ8741 2.PH.JDF

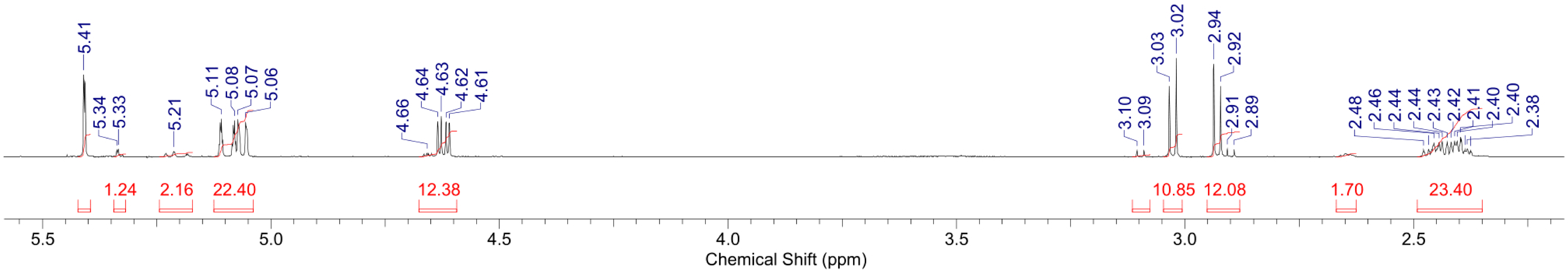

NMR spectrum data for compound 5a (MeCN, r.t, 3 d)

Formula $C_{18}H_{17}NO_4$	***FW*** 311.3319		

Acquisition Time (sec)	1.9818	***Comment***	single_pulse	***Date***	07 Jul 2020 12:52:25		
Date Stamp	07 Jul 2020 12:53:56			***File Name***	F:\NMR FIDS ACIDS\PN ALLYL\FZ8744 5.PH.JDF		
Frequency (MHz)	600.17	***Nucleus***	1H	***Number of Transients***	8	***Origin***	ECA 600
Original Points Count	32768	***Owner***	CKP	***Points Count***	32768	***Pulse Sequence***	single_pulse.ex2
Receiver Gain	44.00	***Solvent***	CHLOROFORM-d			***Spectrum Offset (Hz)***	5410.1226
Sweep Width (Hz)	16534.39	***Temperature (degree C)***	23.200				

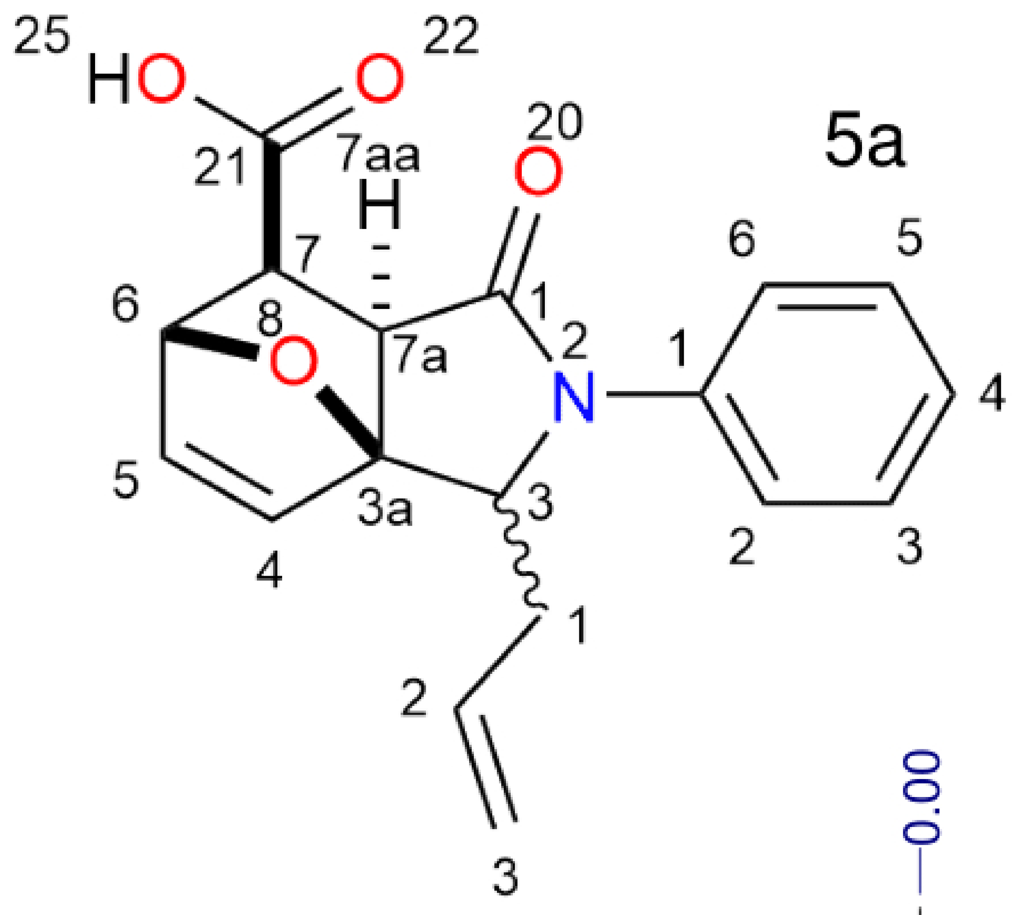

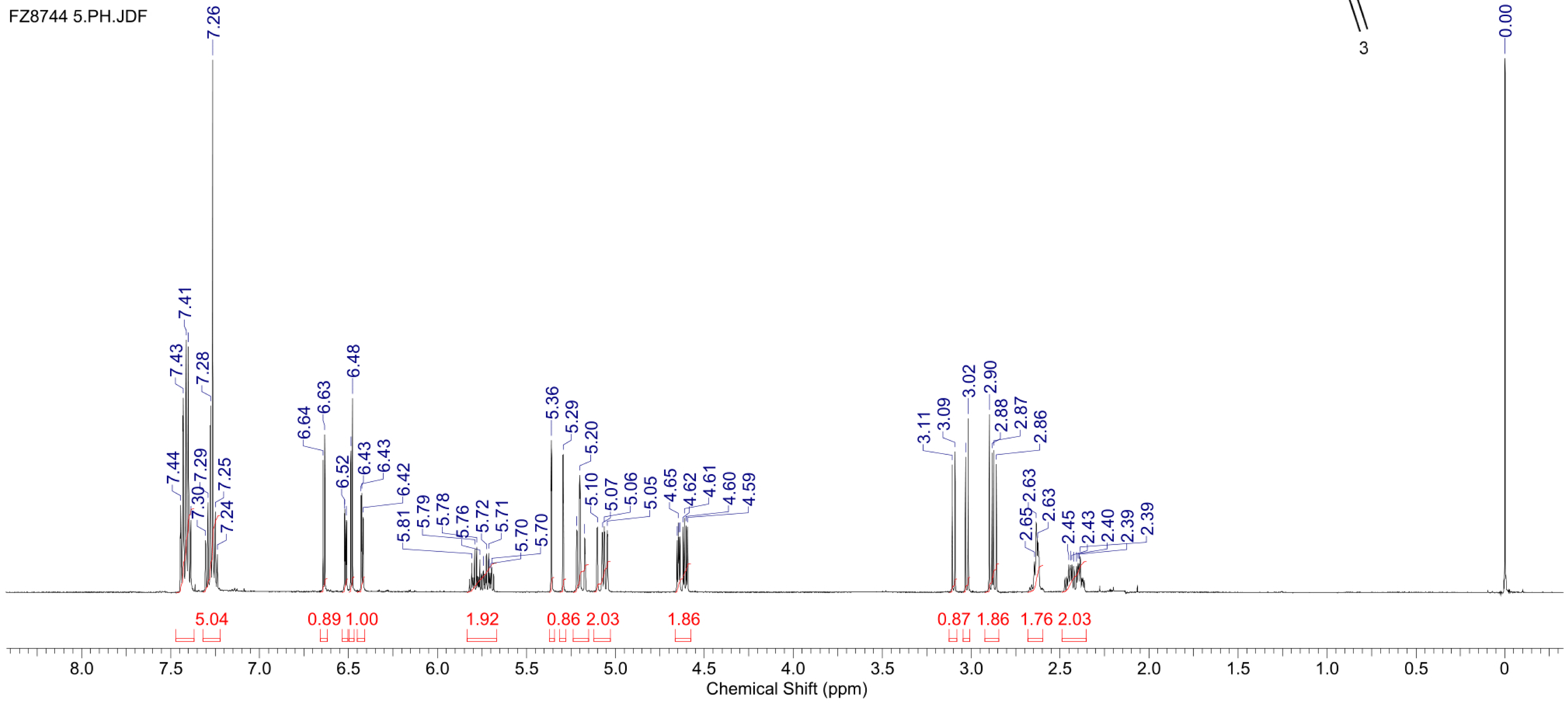

Formula $C_{18}H_{17}NO_4$	*FW* 311.3319

Acquisition Time (sec)	1.9818	***Comment***	single_pulse	***Date***	07 Jul 2020 12:52:25		
Date Stamp	07 Jul 2020 12:53:56			***File Name***	F:\NMR FIDS ACIDS\PN ALLYL\FZ8744 5.PH.JDF		
Frequency (MHz)	600.17	***Nucleus***	1H	***Number of Transients***	8	***Origin***	ECA 600
Original Points Count	32768	***Owner***	CKP	***Points Count***	32768	***Pulse Sequence***	single_pulse.ex2
Receiver Gain	44.00	***Solvent***	CHLOROFORM-d			***Spectrum Offset (Hz)***	5410.1226
Sweep Width (Hz)	16534.39	***Temperature (degree C)***	23.200				

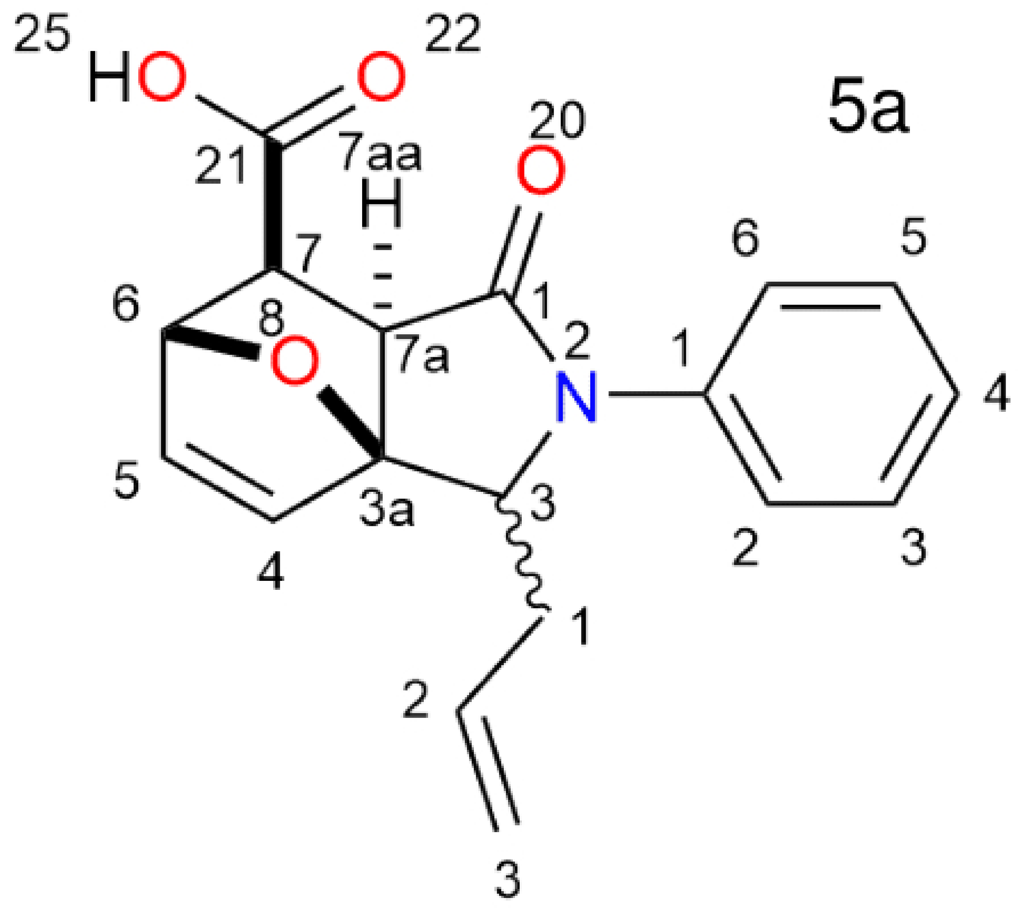

5a

FZ8744 5.PH.JDF

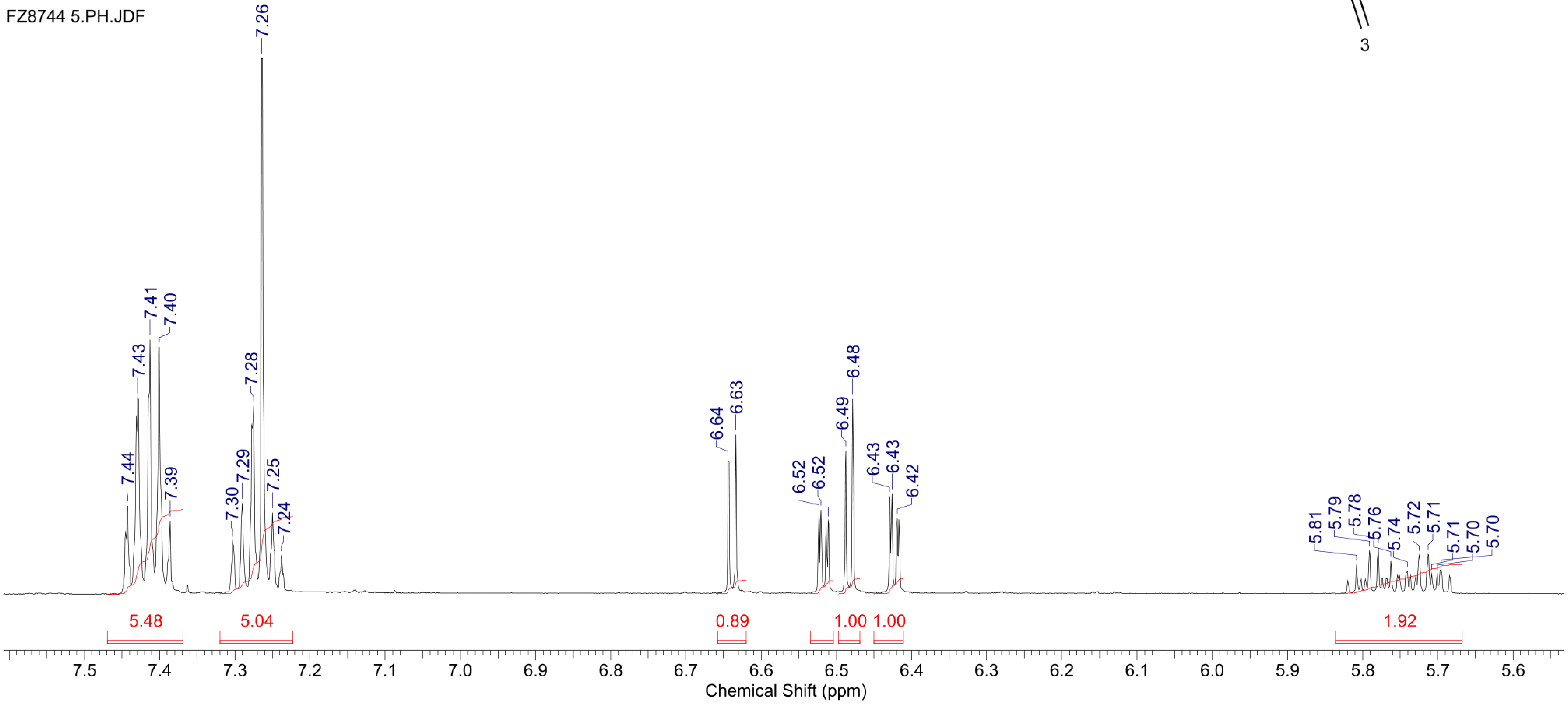

Formula $C_{18}H_{17}NO_4$	FW 311.3319

**Acquisition Time (sec)**	1.9818	**Comment**	single_pulse	**Date**	07 Jul 2020 12:52:25		
**Date Stamp**	07 Jul 2020 12:53:56			**File Name**	F:\NMR FIDS ACIDS\PN ALLYL\FZ8744 5.PH.JDF		
**Frequency (MHz)**	600.17	**Nucleus**	1H	**Number of Transients**	8	**Origin**	ECA 600
**Original Points Count**	32768	**Owner**	CKP	**Points Count**	32768	**Pulse Sequence**	single_pulse.ex2
**Receiver Gain**	44.00	**Solvent**	CHLOROFORM-d			**Spectrum Offset (Hz)**	5410.1226
**Sweep Width (Hz)**	16534.39	**Temperature (degree C)**	23.200				

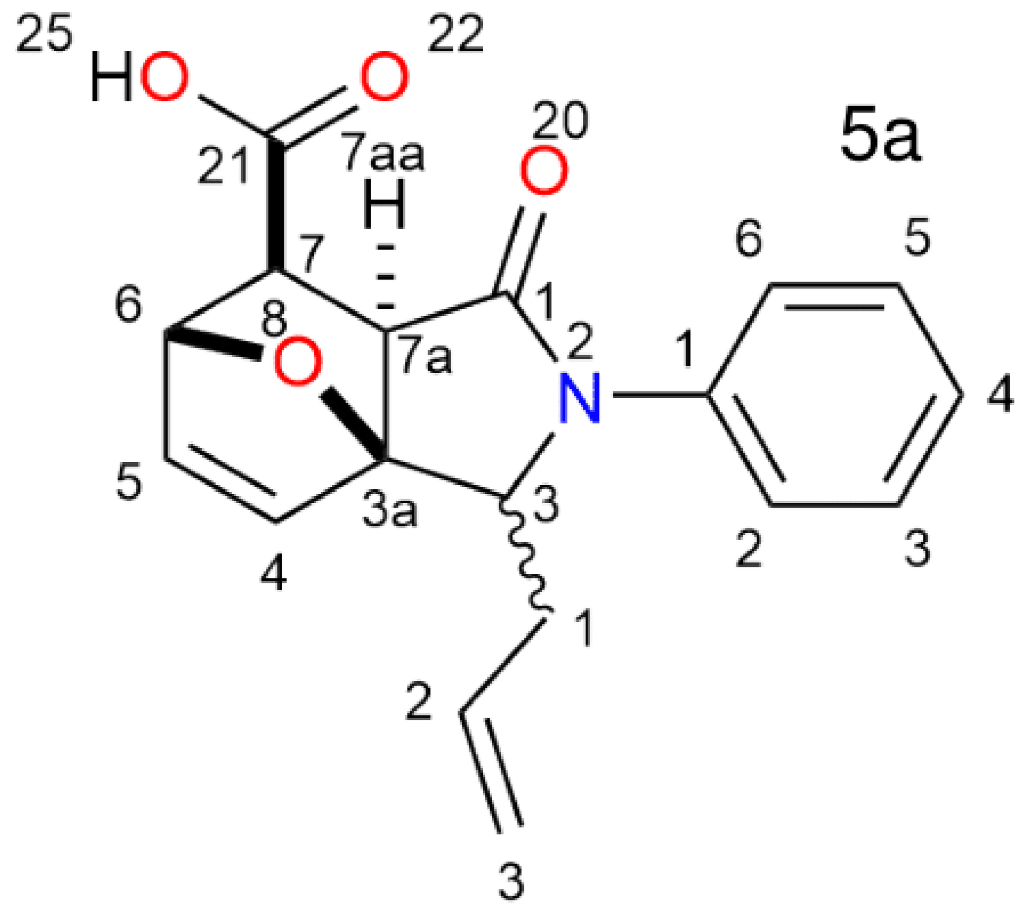

FZ8744 5.PH.JDF

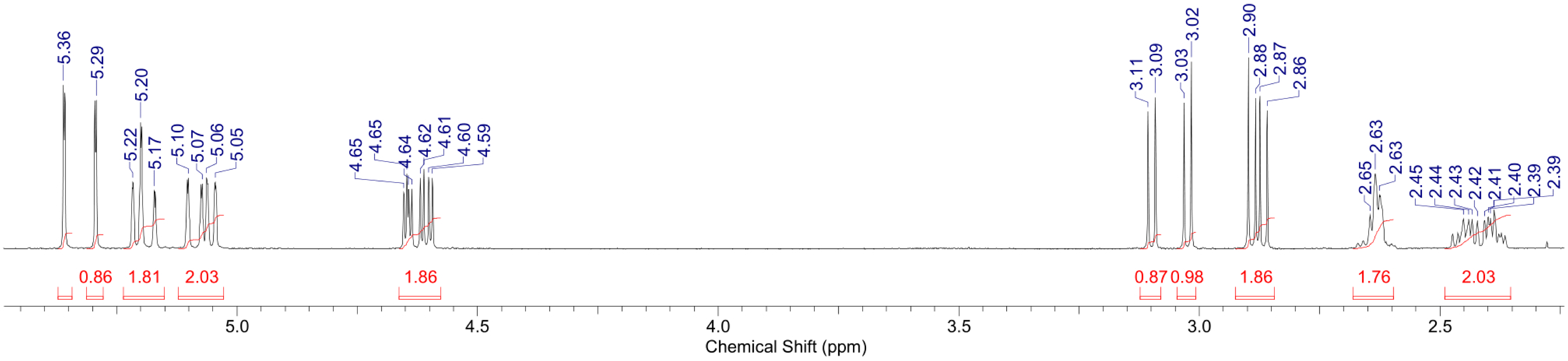

NMR spectrum data for compound 5a (MeCN, Δ, 3 h)

**Formula**	$C_{18}H_{17}NO_4$	**FW**	311.3319

**Acquisition Time (sec)**	1.9818	**Comment**	single_pulse	**Date**	07 Jul 2020 12:52:25		
**Date Stamp**	07 Jul 2020 12:53:56			**File Name**	C:\USERS\лаба 534\DOWNLOADS\FZ8744-1.JDF		
**Frequency (MHz)**	600.17	**Nucleus**	1H	**Number of Transients**	8	**Origin**	ECA 600
**Original Points Count**	32768	**Owner**	CKP	**Points Count**	32768	**Pulse Sequence**	single_pulse.ex2
**Receiver Gain**	44.00	**Solvent**	CHLOROFORM-d			**Spectrum Offset (Hz)**	5410.1226
**Sweep Width (Hz)**	16534.39	**Temperature (degree C)**	23.200				

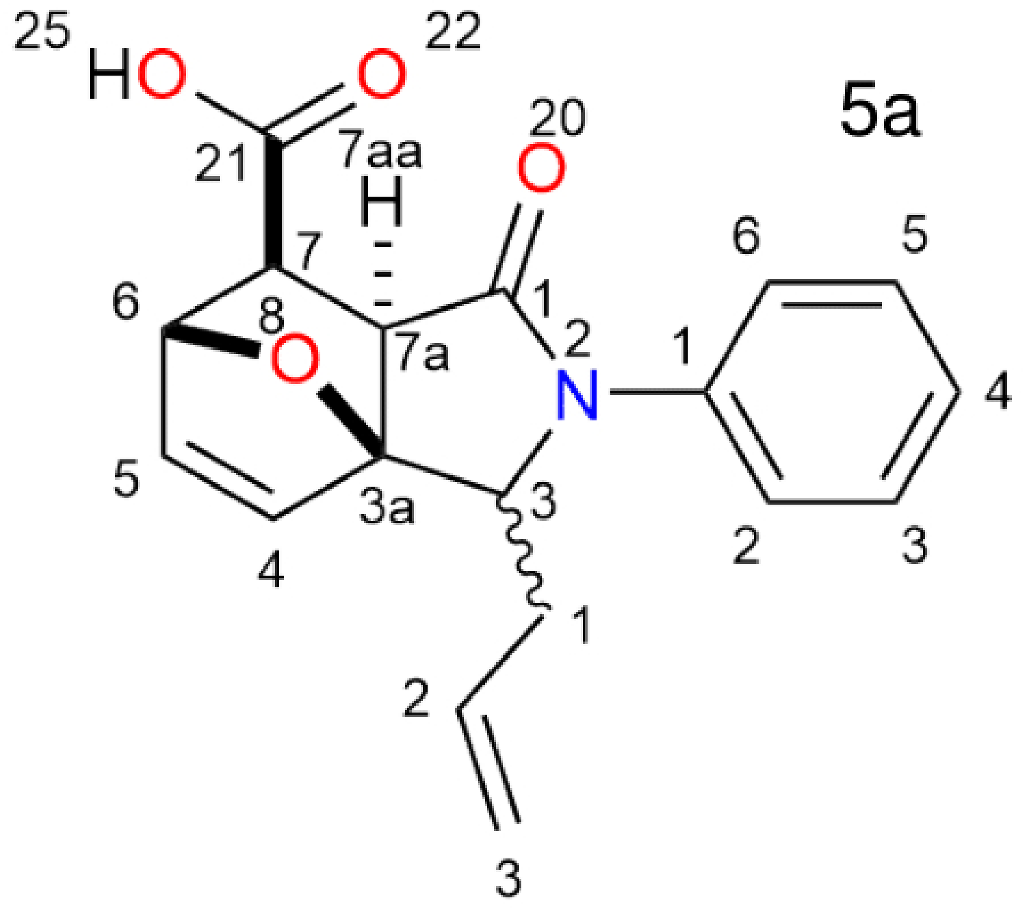

FZ8744-1.JDF

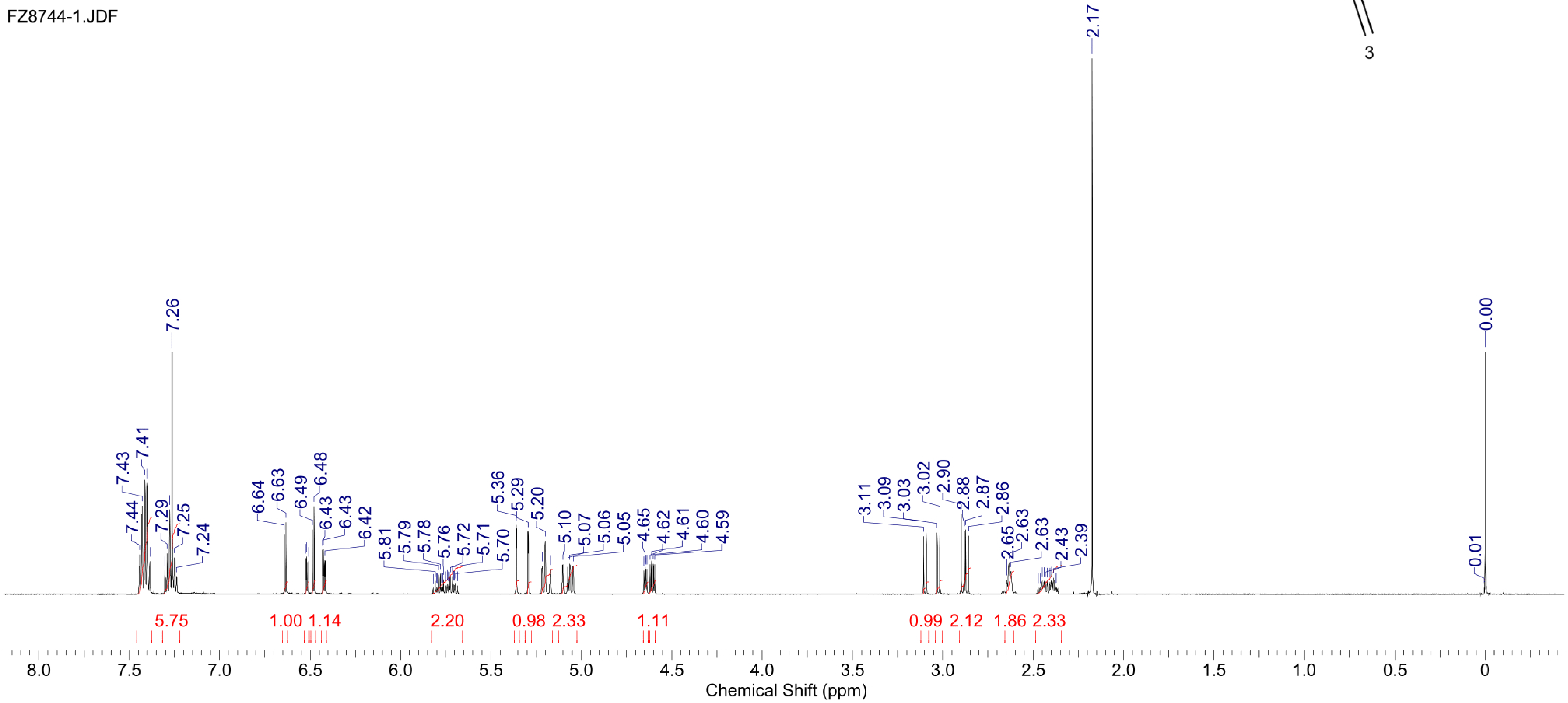

**Formula** $C_{18}H_{17}NO_4$	**FW** 311.3319

**Acquisition Time (sec)**	1.9818	**Comment**	single_pulse	**Date**	07 Jul 2020 12:52:25		
**Date Stamp**	07 Jul 2020 12:53:56			**File Name**	C:\USERS\па6а 534\DOWNLOADS\FZ8744-1.JDF		
**Frequency (MHz)**	600.17	**Nucleus**	1H	**Number of Transients**	8	**Origin**	ECA 600
**Original Points Count**	32768	**Owner**	CKP	**Points Count**	32768	**Pulse Sequence**	single_pulse.ex2
**Receiver Gain**	44.00	**Solvent**	CHLOROFORM-d			**Spectrum Offset (Hz)**	5410.1226
**Sweep Width (Hz)**	16534.39	**Temperature (degree C)**	23.200				

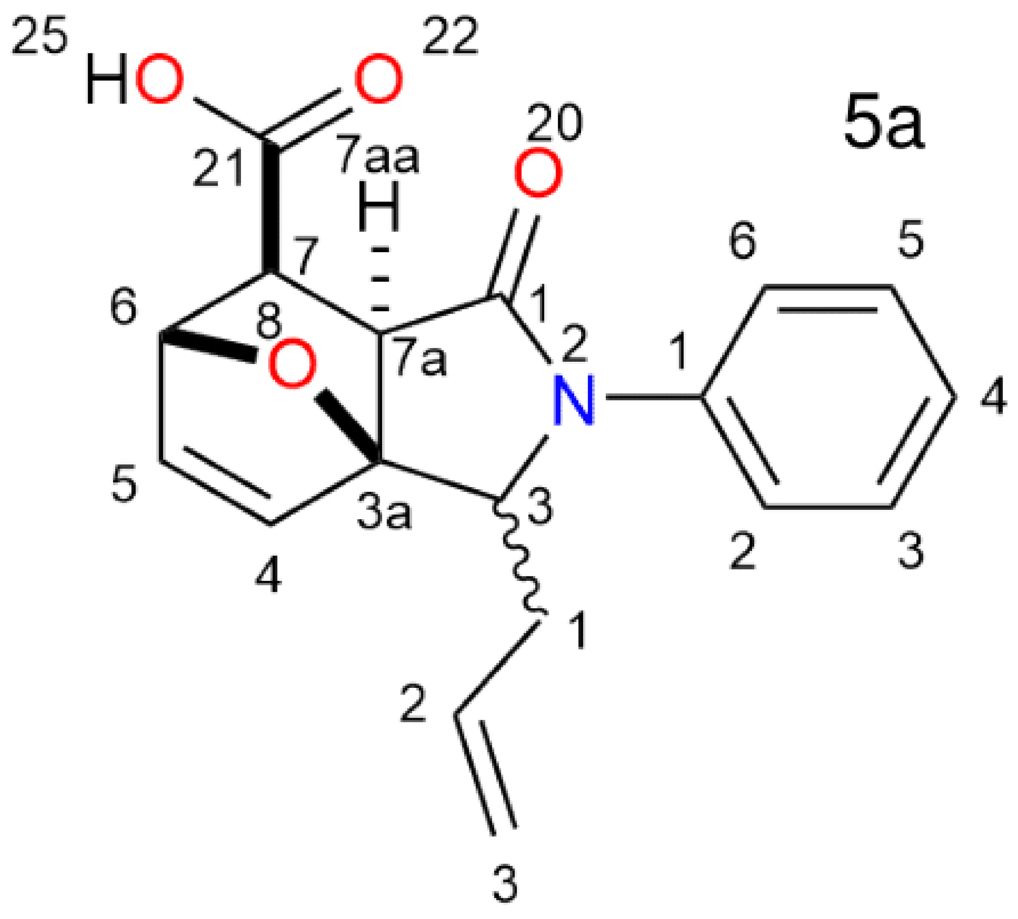

5a

FZ8744-1.JDF

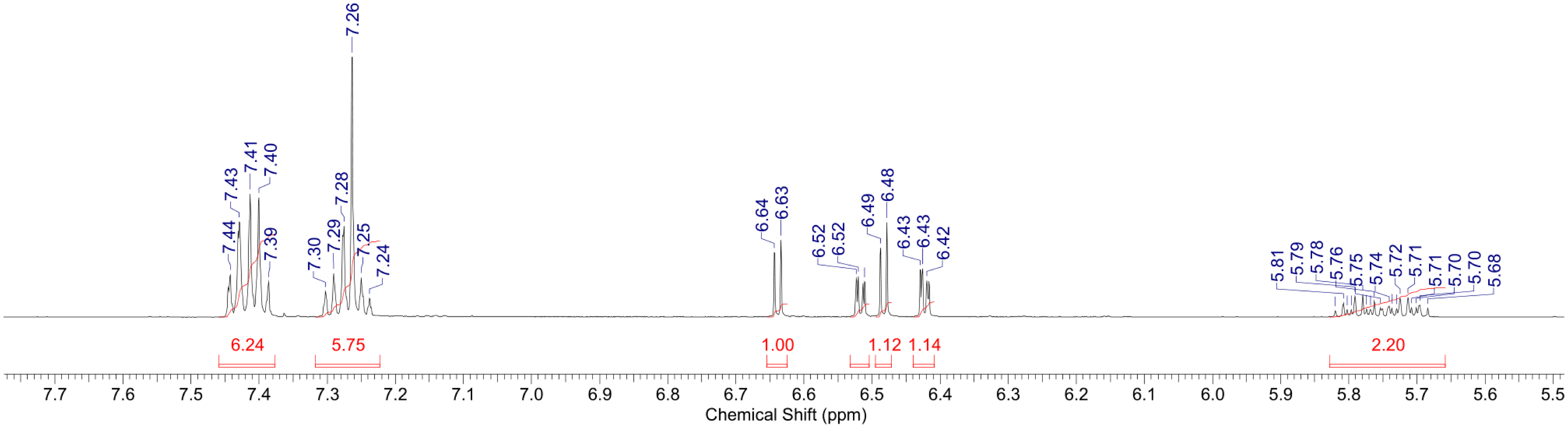

**Formula** $C_{18}H_{17}NO_4$	**FW** 311.3319

Acquisition Time (sec)	1.9818	***Comment***	single_pulse	***Date***	07 Jul 2020 12:52:25		
Date Stamp	07 Jul 2020 12:53:56			***File Name***	C:\USERS\лаба 534\DOWNLOADS\FZ8744-1.JDF		
Frequency (MHz)	600.17	***Nucleus***	1H	***Number of Transients***	8	***Origin***	ECA 600
Original Points Count	32768	***Owner***	CKP	***Points Count***	32768	***Pulse Sequence***	single_pulse.ex2
Receiver Gain	44.00	***Solvent***	CHLOROFORM-d			***Spectrum Offset (Hz)***	5410.1226
Sweep Width (Hz)	16534.39	***Temperature (degree C)***	23.200				

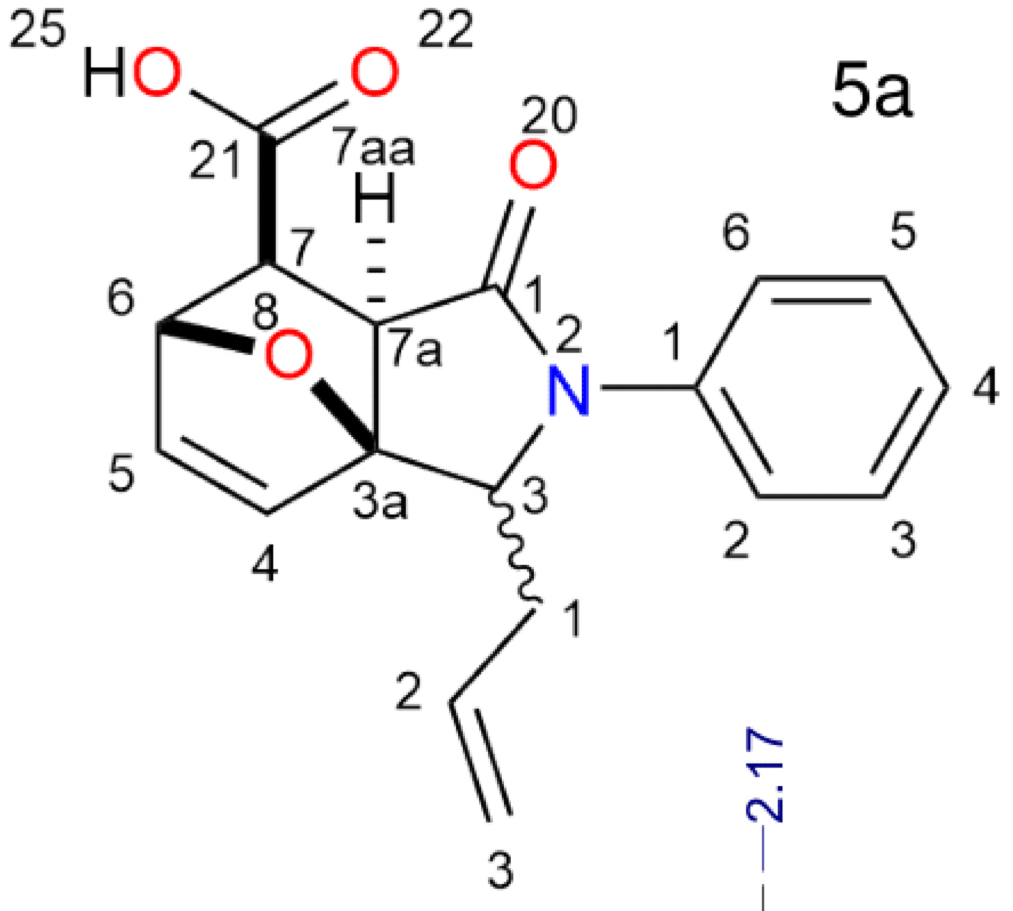

5a

FZ8744-1.JDF

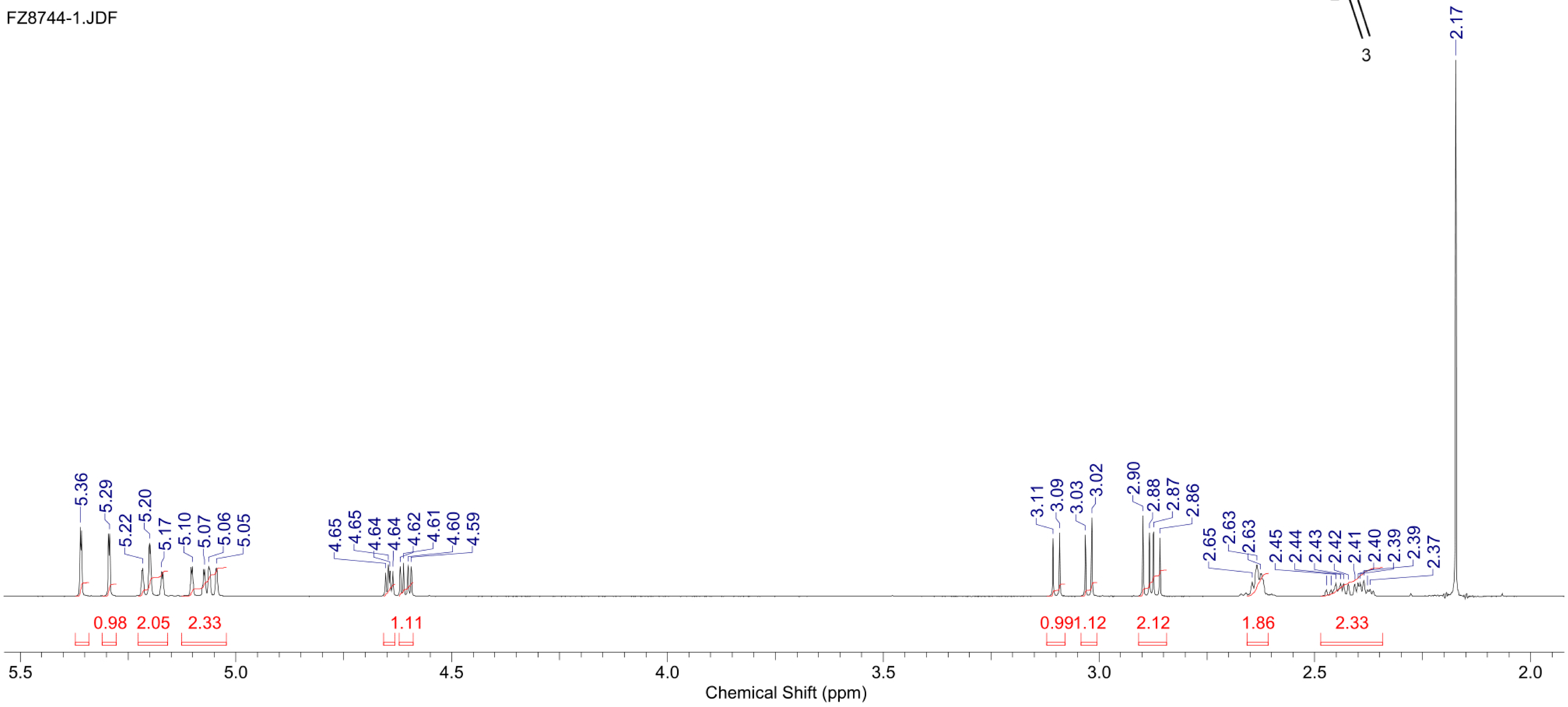

NMR spectrum data for compound 5a (PhH, Δ, 3 h)

**Formula**	$C_{18}H_{17}NO_4$	**FW**	311.3319

Acquisition Time (sec)	1.9818	***Comment***	single_pulse	***Date***	07 Jul 2020 12:18:18		
Date Stamp	07 Jul 2020 12:19:50			***File Name***	F:\NMR FIDS ACIDS\PN ALLYL\FZ8746 7.PH.JDF		
Frequency (MHz)	600.17	***Nucleus***	1H	***Number of Transients***	8	***Origin***	ECA 600
Original Points Count	32768	***Owner***	CKP	***Points Count***	32768	***Pulse Sequence***	single_pulse.ex2
Receiver Gain	42.00	***Solvent***	CHLOROFORM-d			***Spectrum Offset (Hz)***	5410.6274
Sweep Width (Hz)	16534.39	***Temperature (degree C)***	23.100				

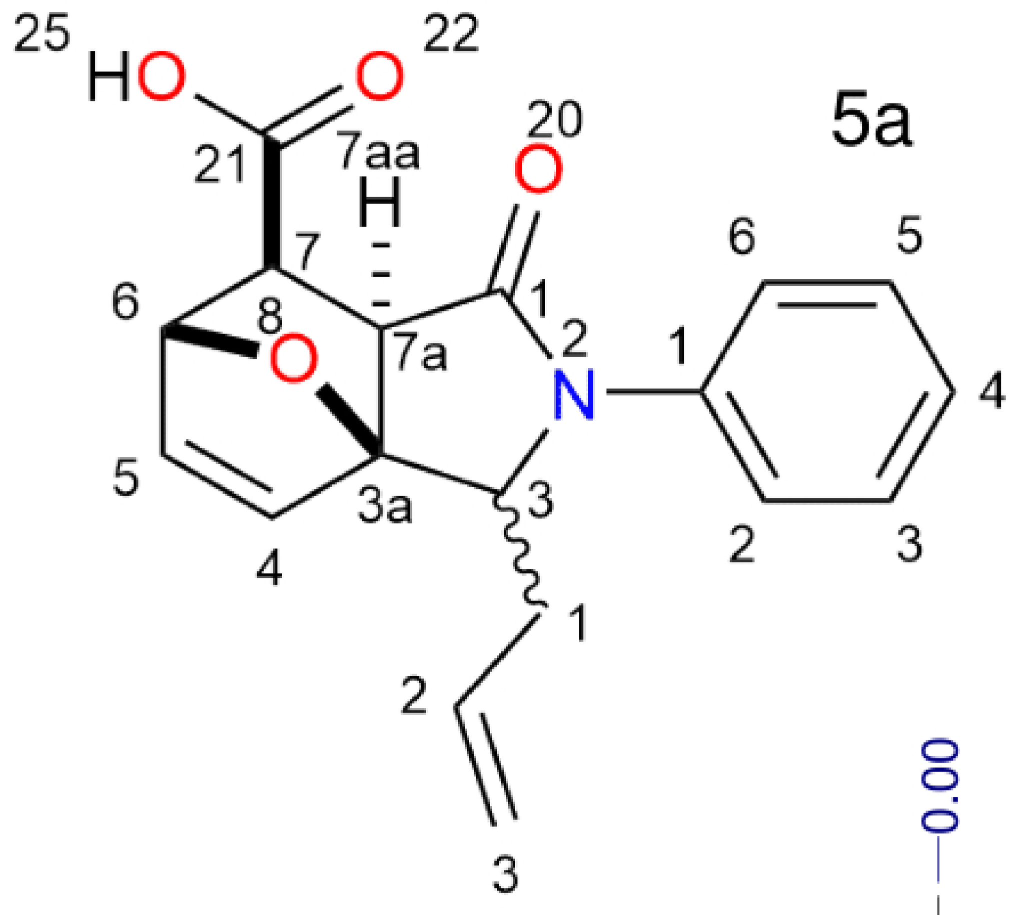

5a

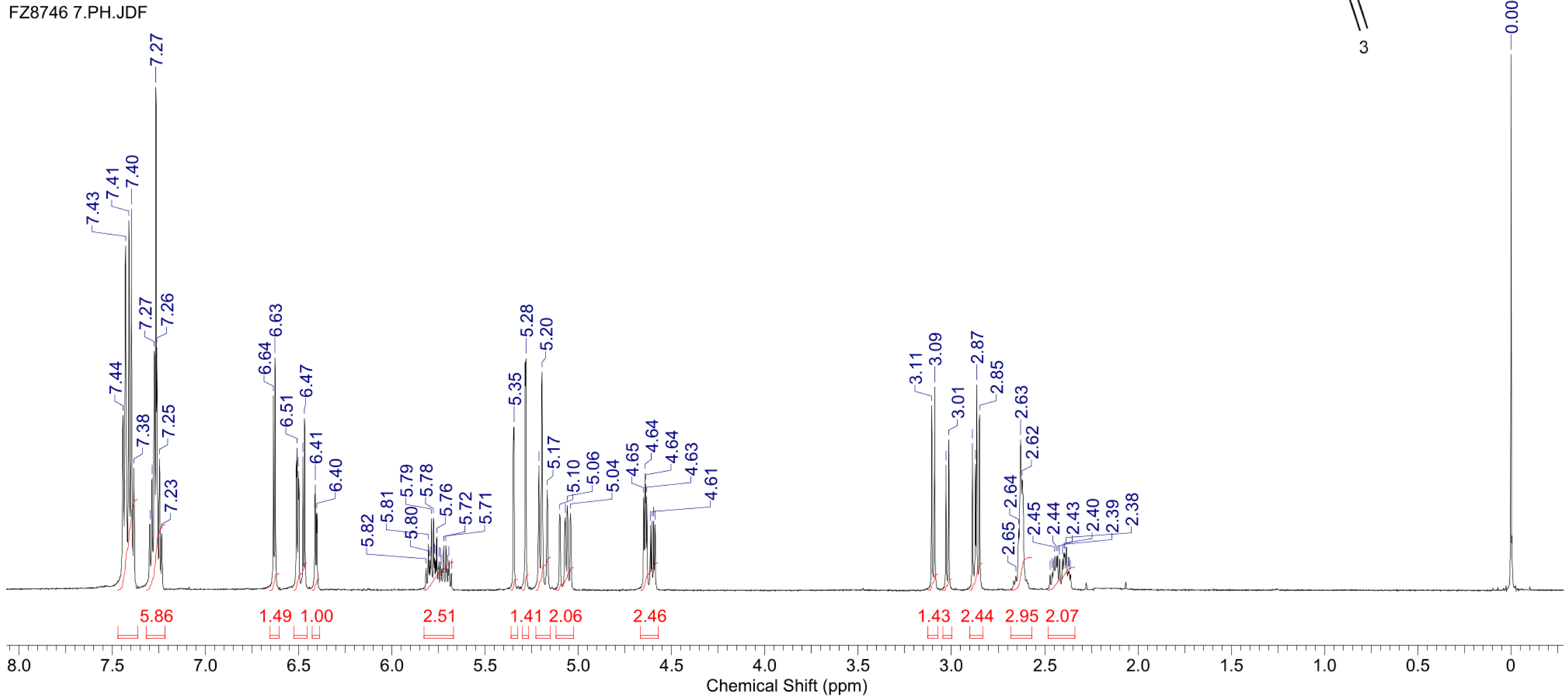

Formula $C_{18}H_{17}NO_4$	FW 311.3319

**Acquisition Time (sec)**	1.9818	**Comment**	single_pulse	**Date**	07 Jul 2020 12:18:18		
**Date Stamp**	07 Jul 2020 12:19:50			**File Name**	F:\NMR FIDS ACIDS\PN ALLYL\FZ8746 7.PH.JDF		
**Frequency (MHz)**	600.17	**Nucleus**	1H	**Number of Transients**	8	**Origin**	ECA 600
**Original Points Count**	32768	**Owner**	CKP	**Points Count**	32768	**Pulse Sequence**	single_pulse.ex2
**Receiver Gain**	42.00	**Solvent**	CHLOROFORM-d			**Spectrum Offset (Hz)**	5410.6274
**Sweep Width (Hz)**	16534.39	**Temperature (degree C)**	23.100				

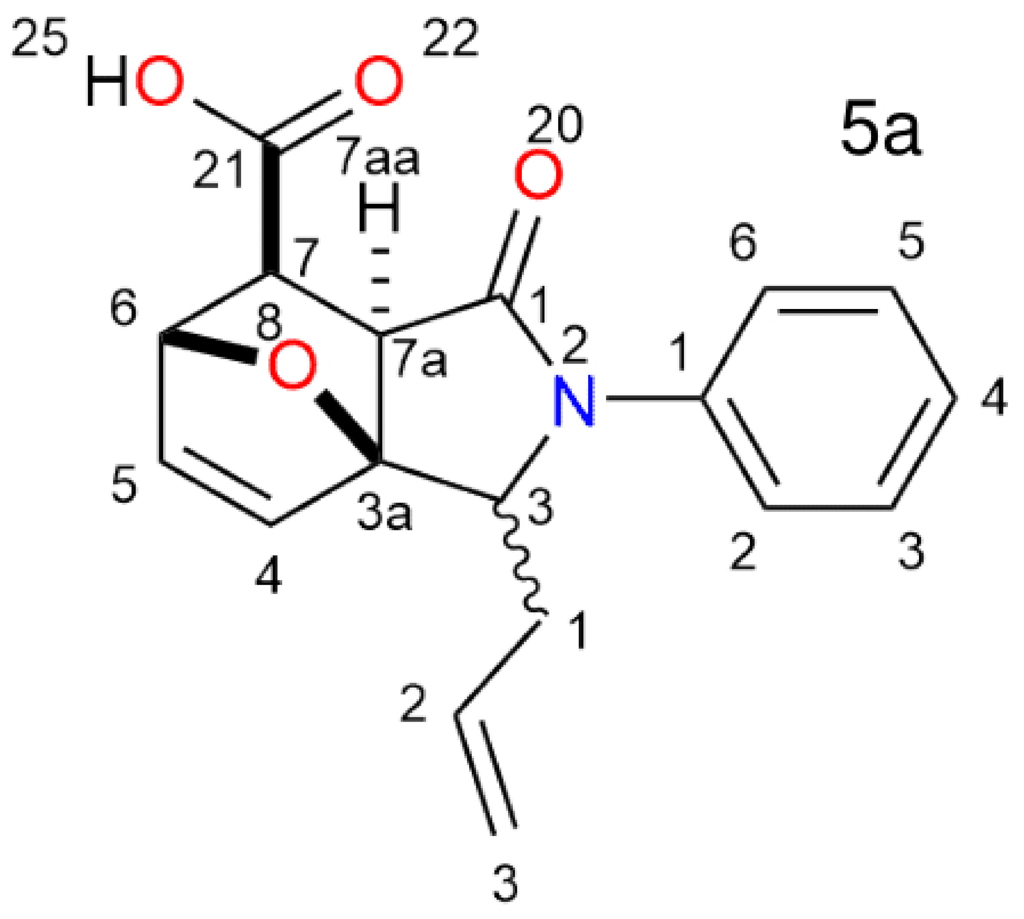

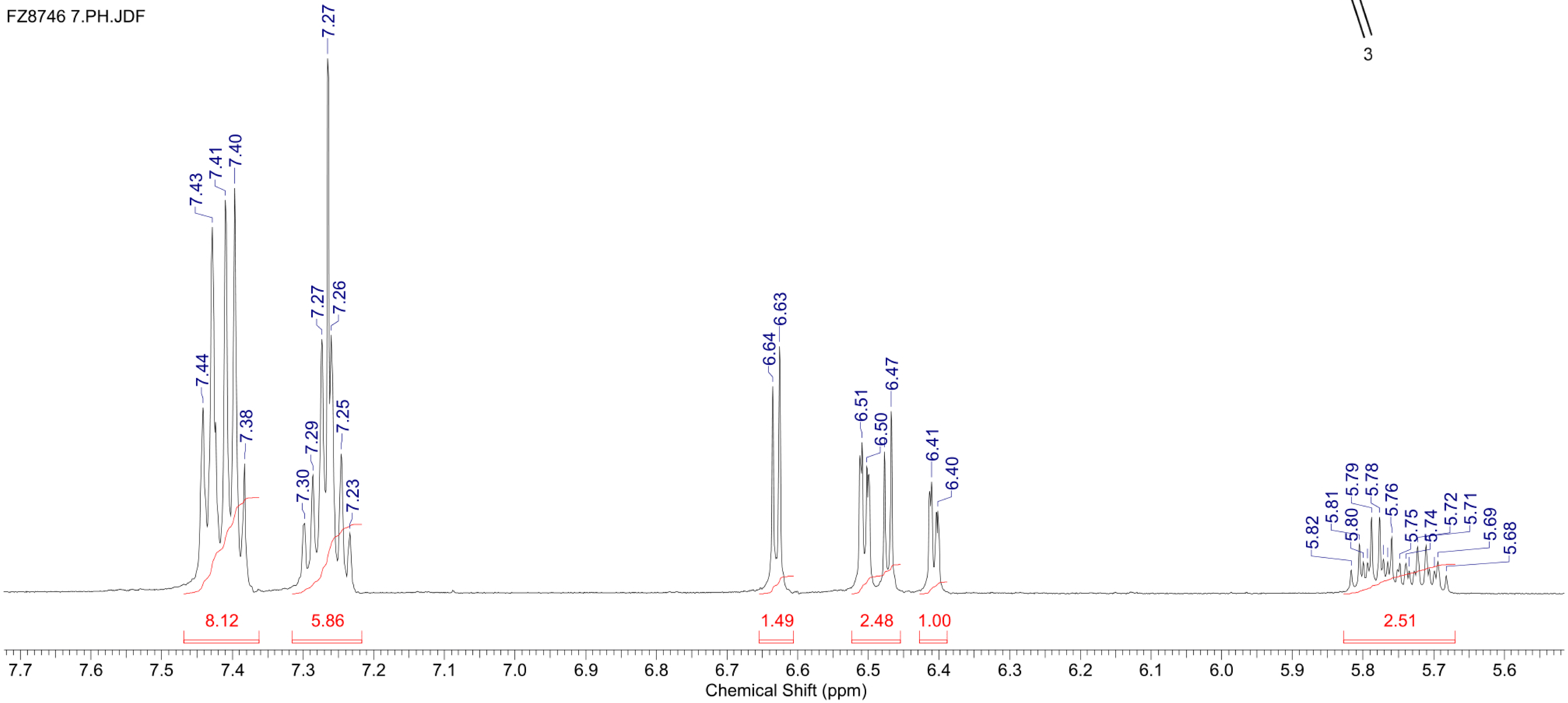

Formula $C_{18}H_{17}NO_4$	***FW*** 311.3319

Acquisition Time (sec)	1.9818	***Comment***	single_pulse	***Date***	07 Jul 2020 12:18:18		
Date Stamp	07 Jul 2020 12:19:50			***File Name***	F:\NMR FIDS ACIDS\PN ALLYL\FZ8746 7.PH.JDF		
Frequency (MHz)	600.17	***Nucleus***	1H	***Number of Transients***	8	***Origin***	ECA 600
Original Points Count	32768	***Owner***	CKP	***Points Count***	32768	***Pulse Sequence***	single_pulse.ex2
Receiver Gain	42.00	***Solvent***	CHLOROFORM-d			***Spectrum Offset (Hz)***	5410.6274
Sweep Width (Hz)	16534.39	***Temperature (degree C)***	23.100				

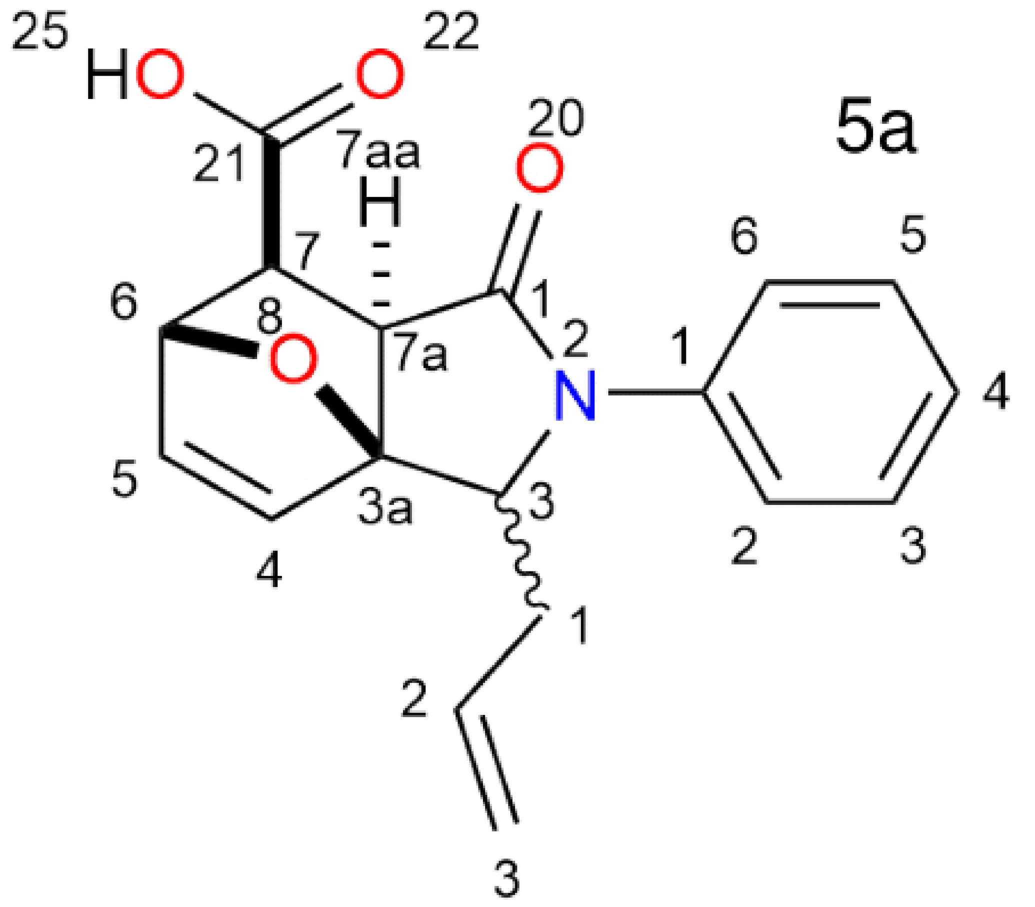

5a

FZ8746 7.PH.JDF

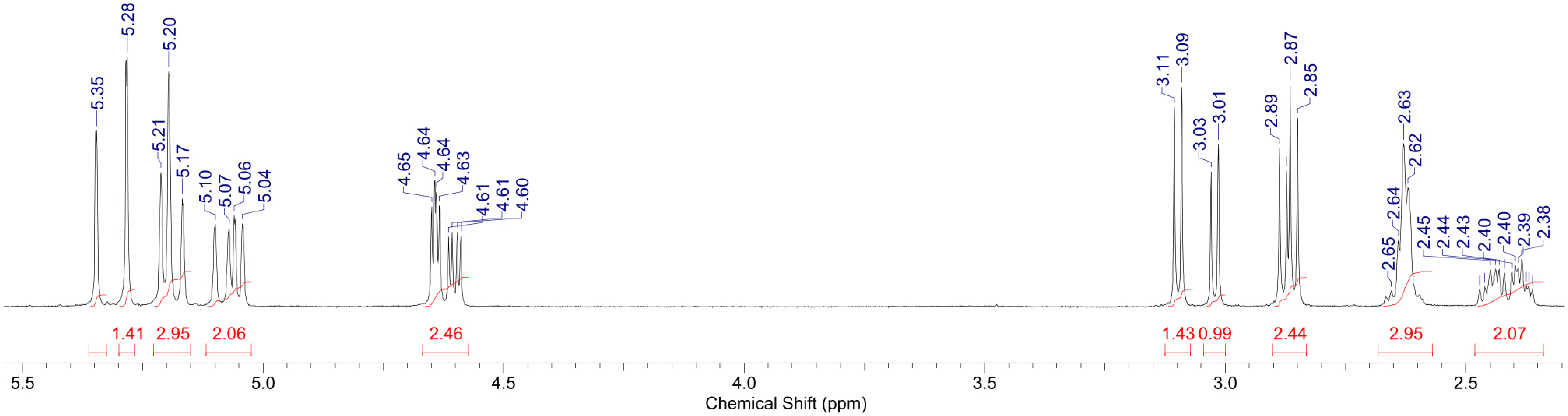

NMR spectrum data for compound 5a (PhMe, r.t, 3 d)

**Formula**	$C_{18}H_{17}NO_4$	**FW**	311.3319

**Acquisition Time (sec)**	1.9818	**Comment**	single_pulse	**Date**	07 Jul 2020 11:54:31		
**Date Stamp**	07 Jul 2020 11:56:02			**File Name**	F:\NMR FIDS ACIDS\PN ALLYL\FZ8742 3.PH.JDF		
**Frequency (MHz)**	600.17	**Nucleus**	1H	**Number of Transients**	8	**Origin**	ECA 600
**Original Points Count**	32768	**Owner**	CKP	**Points Count**	32768	**Pulse Sequence**	single_pulse.ex2
**Receiver Gain**	40.00	**Solvent**	CHLOROFORM-d			**Spectrum Offset (Hz)**	5410.6274
**Sweep Width (Hz)**	16534.39	**Temperature (degree C)**	22.900				

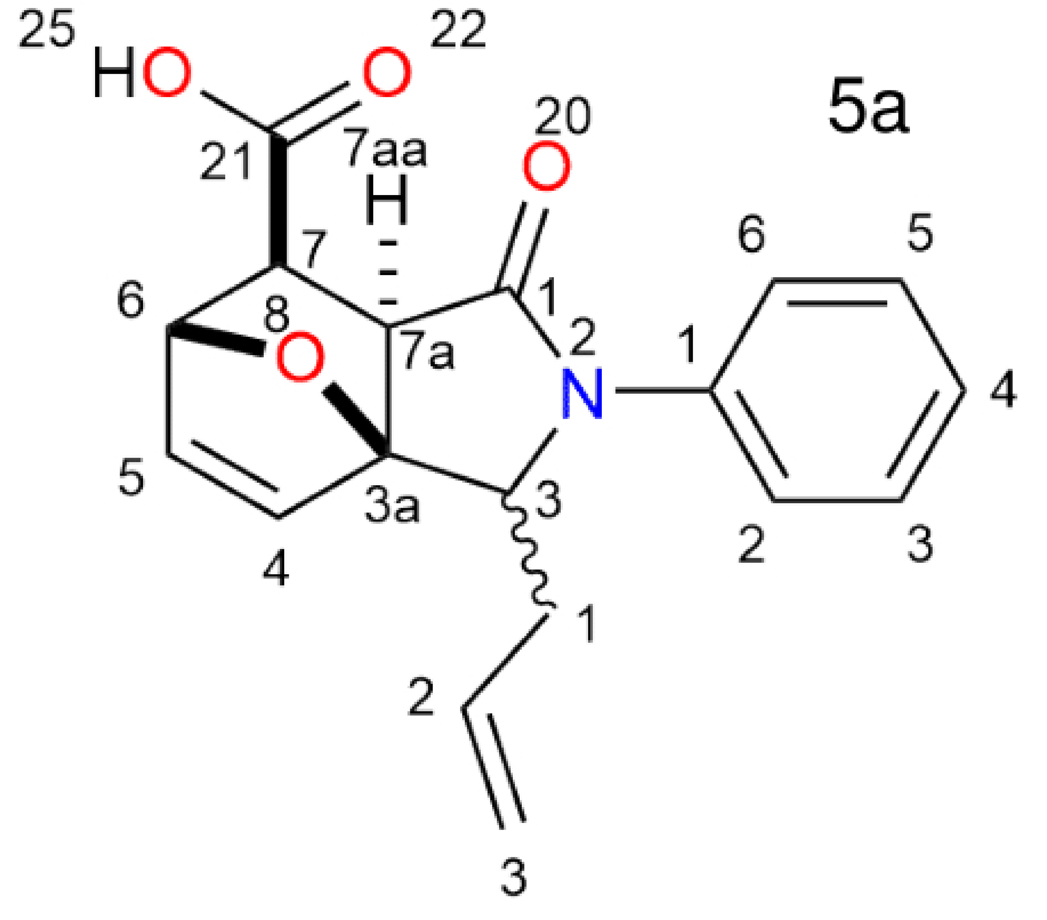

5a

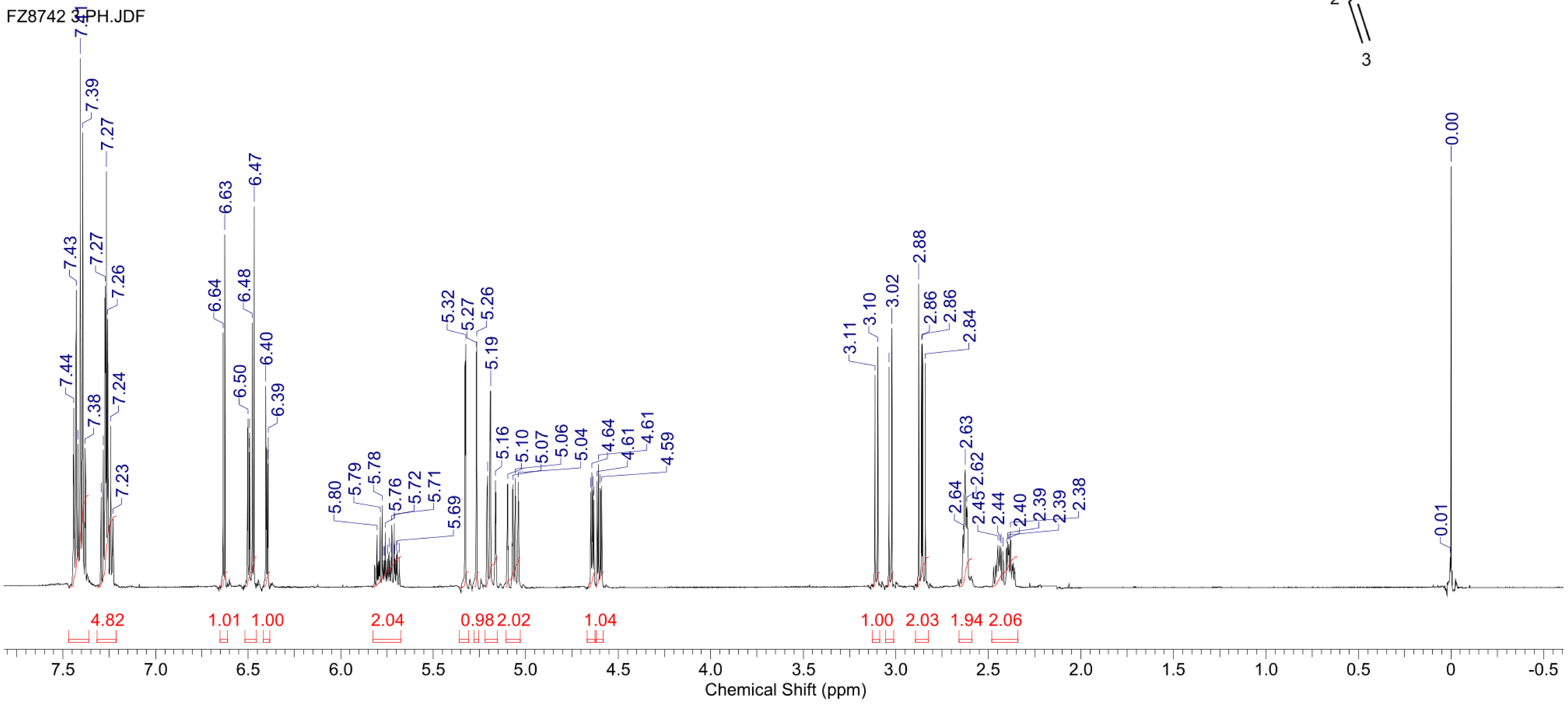

**Formula** $C_{18}H_{17}NO_4$	**FW** 311.3319

**Acquisition Time (sec)**	1.9818	**Comment**	single_pulse	**Date**	07 Jul 2020 11:54:31		
**Date Stamp**	07 Jul 2020 11:56:02			**File Name**	F:\NMR FIDS ACIDS\PN ALLYL\FZ8742 3.PH.JDF		
**Frequency (MHz)**	600.17	**Nucleus**	1H	**Number of Transients**	8	**Origin**	ECA 600
**Original Points Count**	32768	**Owner**	CKP	**Points Count**	32768	**Pulse Sequence**	single_pulse.ex2
**Receiver Gain**	40.00	**Solvent**	CHLOROFORM-d			**Spectrum Offset (Hz)**	5410.6274
**Sweep Width (Hz)**	16534.39	**Temperature (degree C)**	22.900				

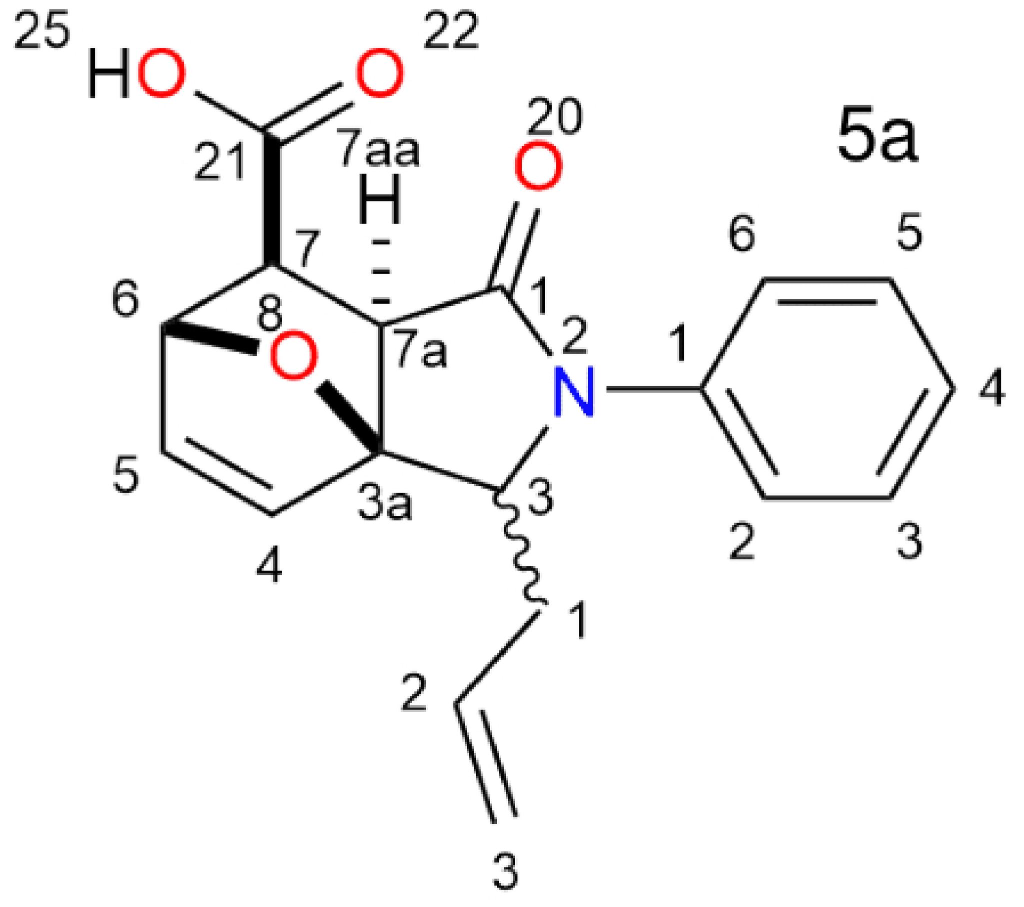

5a

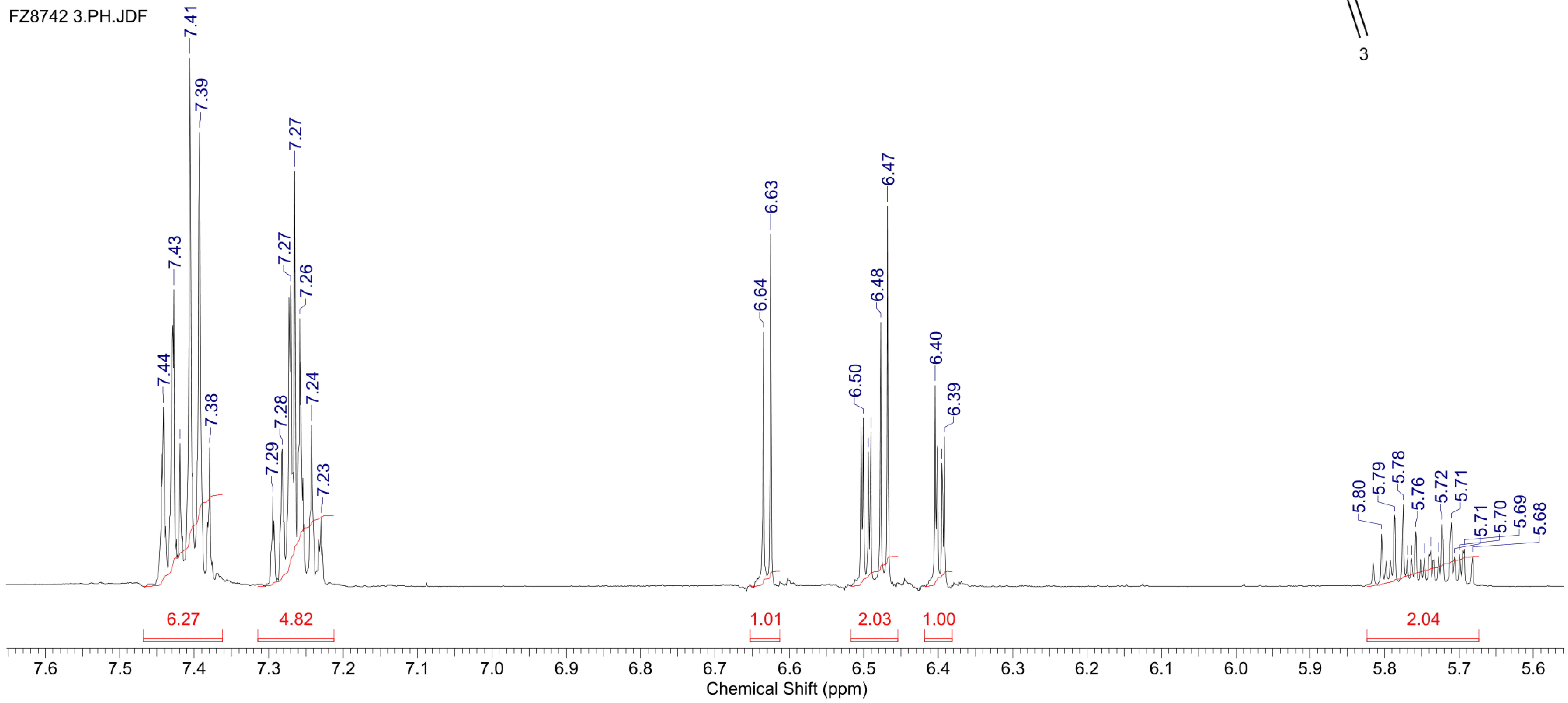

Formula	$C_{18}H_{17}NO_4$	FW	311.3319

Acquisition Time (sec)	1.9818	***Comment***	single_pulse	***Date***	07 Jul 2020 11:54:31		
Date Stamp	07 Jul 2020 11:56:02			***File Name***	F:\NMR FIDS ACIDS\PN ALLYL\FZ8742 3.PH.JDF		
Frequency (MHz)	600.17	***Nucleus***	1H	***Number of Transients***	8	***Origin***	ECA 600
Original Points Count	32768	***Owner***	CKP	***Points Count***	32768	***Pulse Sequence***	single_pulse.ex2
Receiver Gain	40.00	***Solvent***	CHLOROFORM-d			***Spectrum Offset (Hz)***	5410.6274
Sweep Width (Hz)	16534.39	***Temperature (degree C)***	22.900				

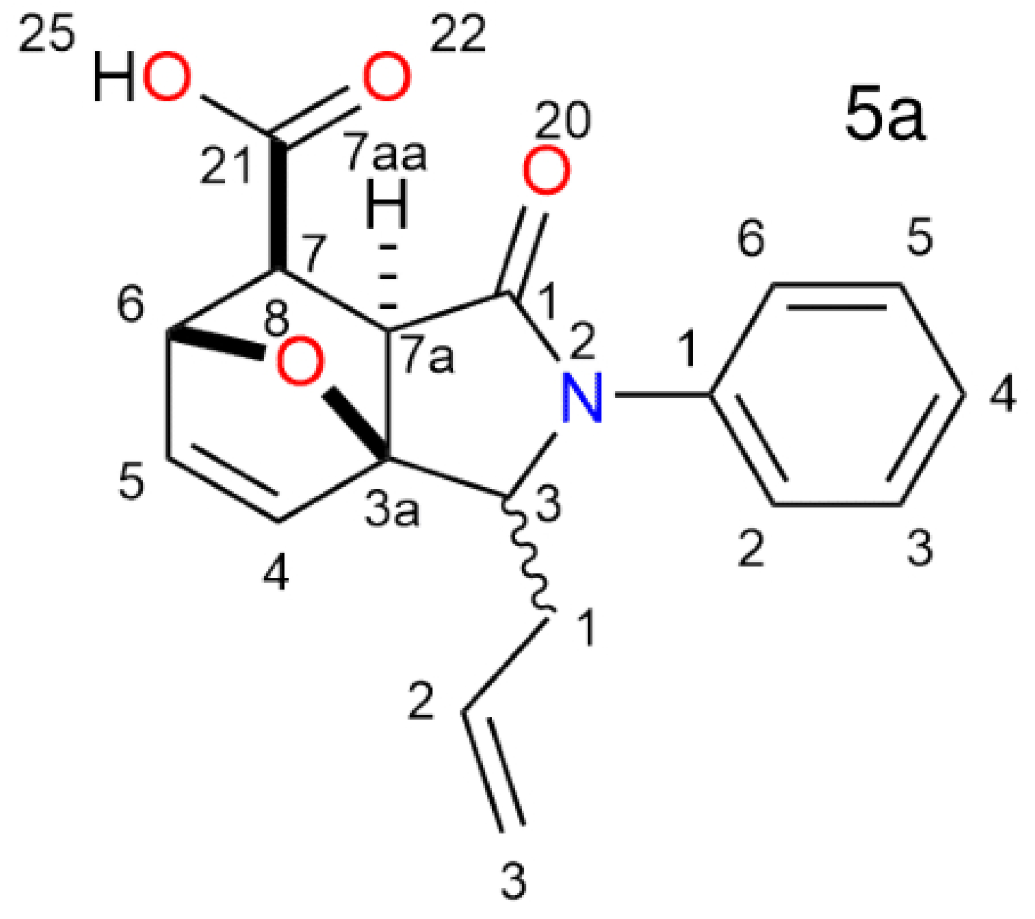

5a

FZ8742 3.PH.JDF

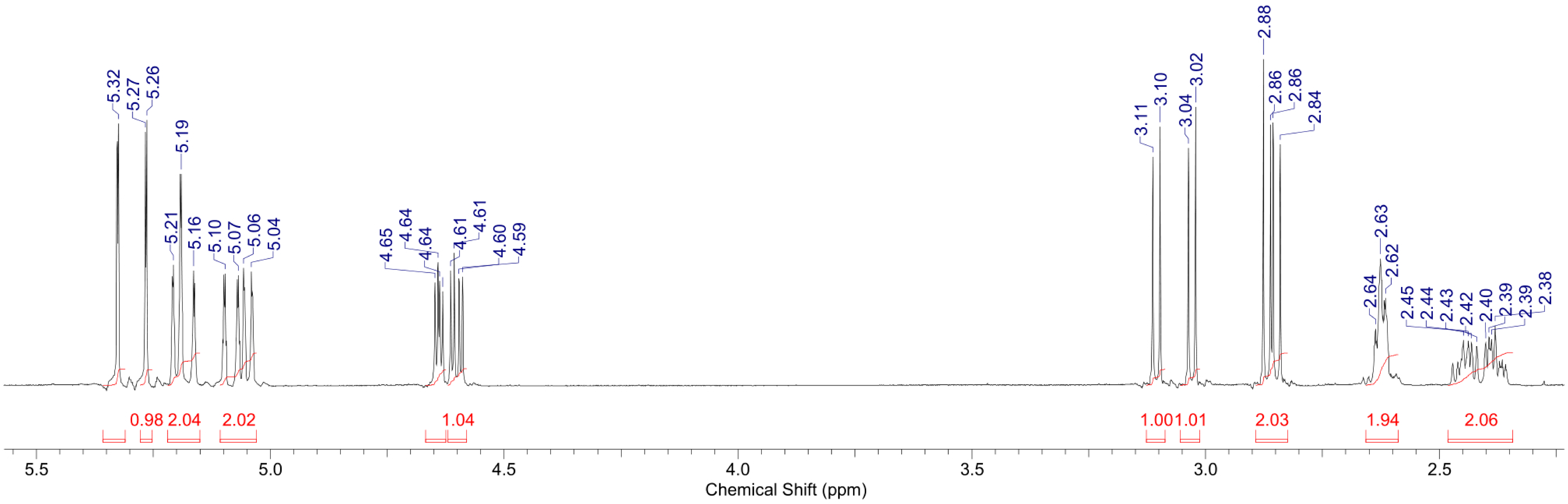

NMR spectrum data for compound 5a (PhMe, Δ, 3 h)

**Formula**	$C_{18}H_{17}NO_4$	**FW**	311.3319

**Acquisition Time (sec)**	1.9818	**Comment**	single_pulse	**Date**	07 Jul 2020 12:00:45		
**Date Stamp**	07 Jul 2020 12:02:17			**File Name**	F:\NMR FIDS ACIDS\PN ALLYL\FZ8743 4.PH.JDF		
**Frequency (MHz)**	600.17	**Nucleus**	1H	**Number of Transients**	8	**Origin**	ECA 600
**Original Points Count**	32768	**Owner**	CKP	**Points Count**	32768	**Pulse Sequence**	single_pulse.ex2
**Receiver Gain**	44.00	**Solvent**	CHLOROFORM-d			**Spectrum Offset (Hz)**	5410.6274
**Sweep Width (Hz)**	16534.39	**Temperature (degree C)**	22.900				

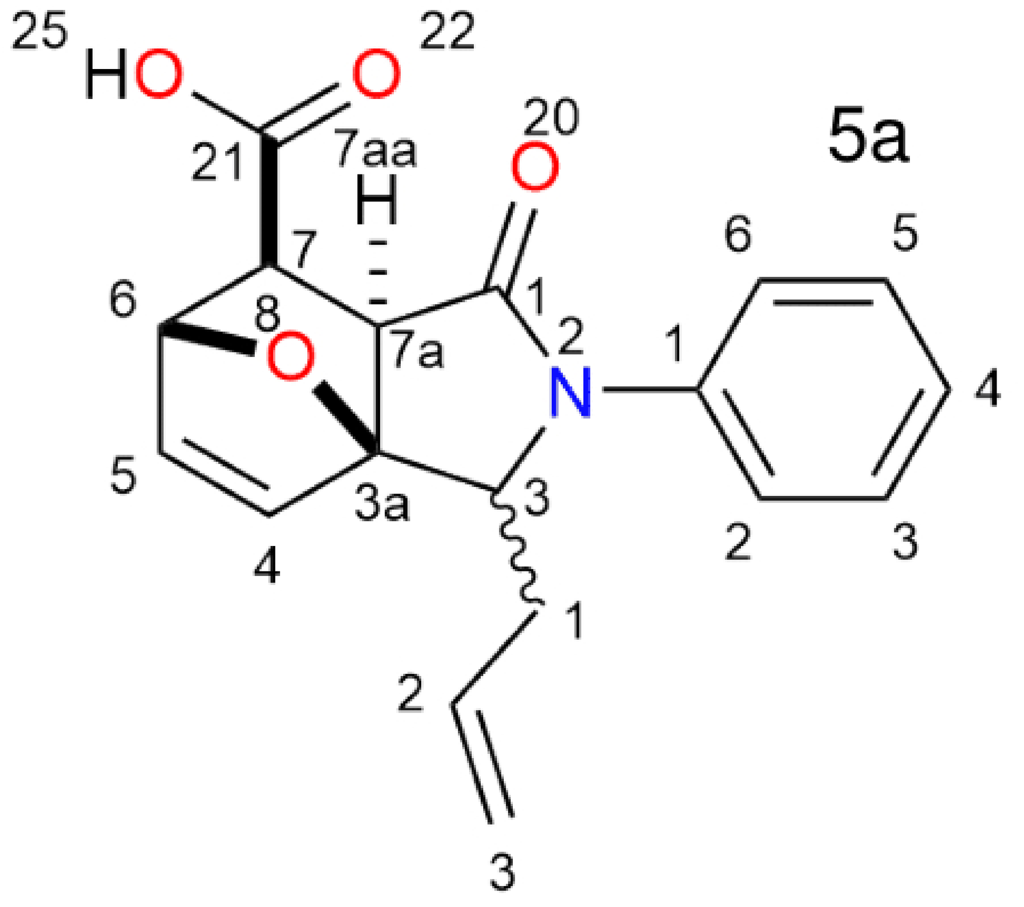

5a

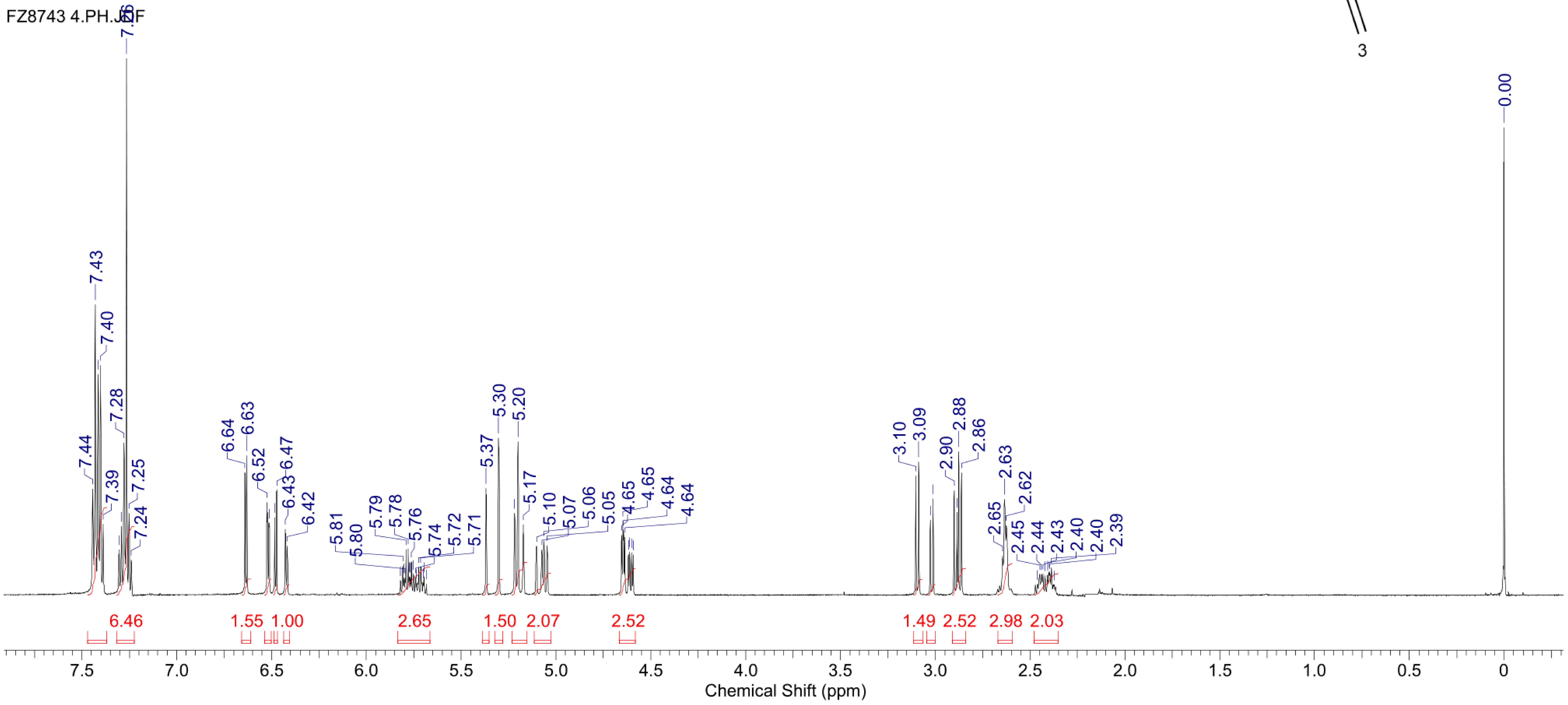

Formula $C_{18}H_{17}NO_4$	FW 311.3319

**Acquisition Time (sec)**	1.9818	**Comment**	single_pulse	**Date**	07 Jul 2020 12:00:45		
**Date Stamp**	07 Jul 2020 12:02:17			**File Name**	F:\NMR FIDS ACIDS\PN ALLYL\FZ8743 4.PH.JDF		
**Frequency (MHz)**	600.17	**Nucleus**	1H	**Number of Transients**	8	**Origin**	ECA 600
**Original Points Count**	32768	**Owner**	CKP	**Points Count**	32768	**Pulse Sequence**	single_pulse.ex2
**Receiver Gain**	44.00	**Solvent**	CHLOROFORM-d			**Spectrum Offset (Hz)**	5410.6274
**Sweep Width (Hz)**	16534.39	**Temperature (degree C)**	22.900				

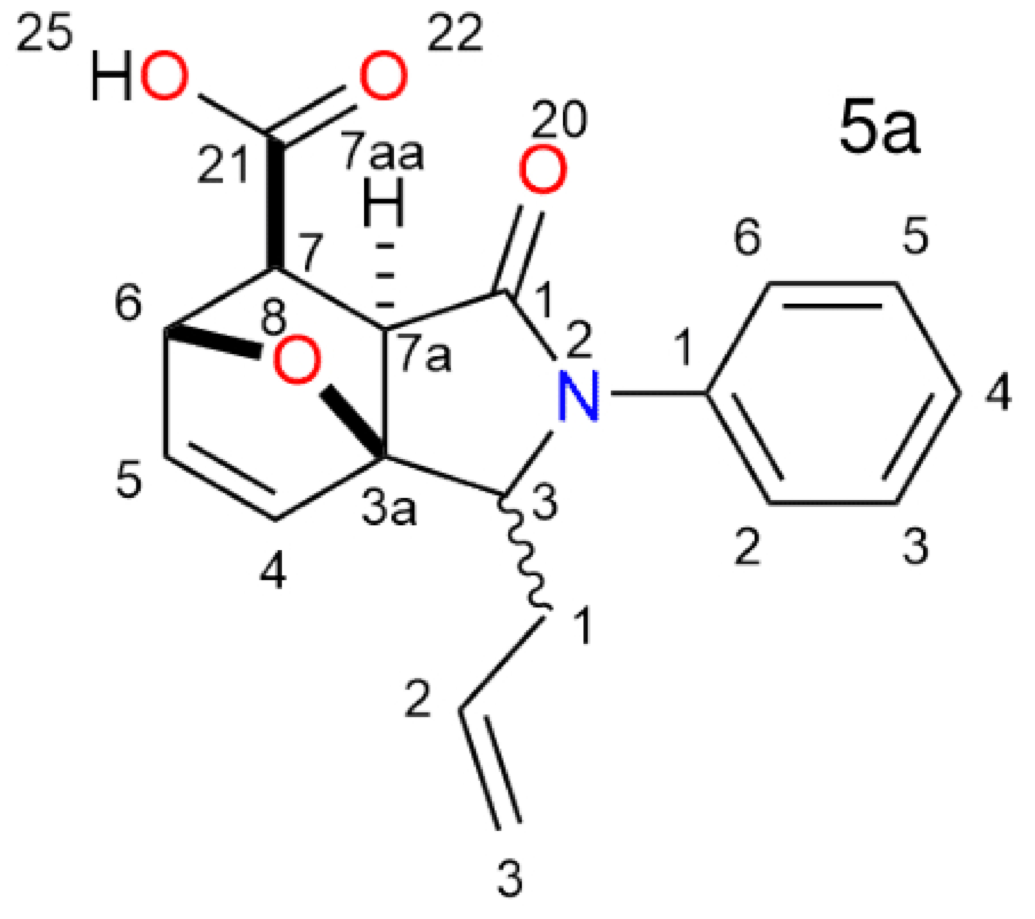

5a

FZ8743 4.PH.JDF

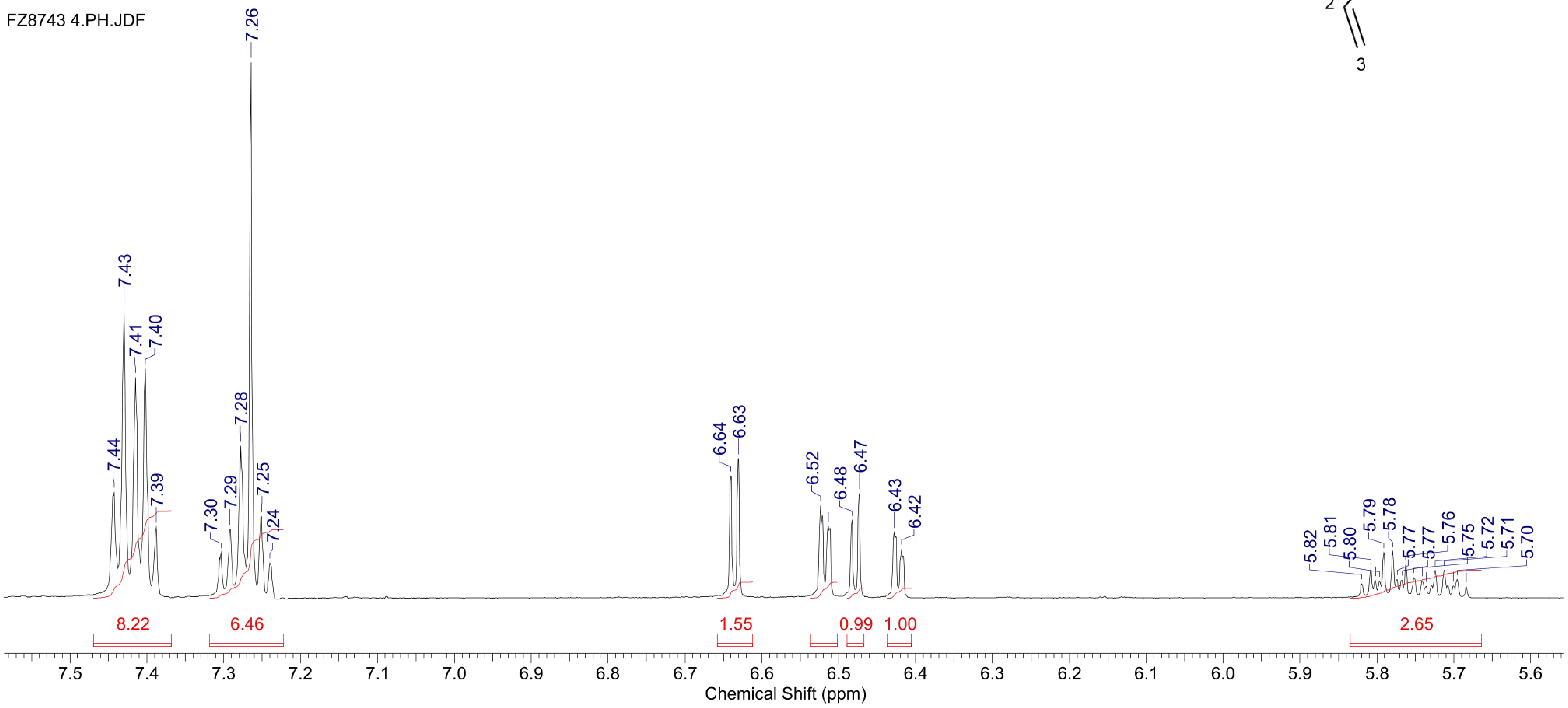

Formula $C_{18}H_{17}NO_4$	FW 311.3319

Acquisition Time (sec)	1.9818	***Comment***	single_pulse	***Date***	07 Jul 2020 12:00:45		
Date Stamp	07 Jul 2020 12:02:17			***File Name***	F:\NMR FIDS ACIDS\PN ALLYL\FZ8743 4.PH.JDF		
Frequency (MHz)	600.17	***Nucleus***	1H	***Number of Transients***	8	***Origin***	ECA 600
Original Points Count	32768	***Owner***	CKP	***Points Count***	32768	***Pulse Sequence***	single_pulse.ex2
Receiver Gain	44.00	***Solvent***	CHLOROFORM-d			***Spectrum Offset (Hz)***	5410.6274
Sweep Width (Hz)	16534.39	***Temperature (degree C)***	22.900				

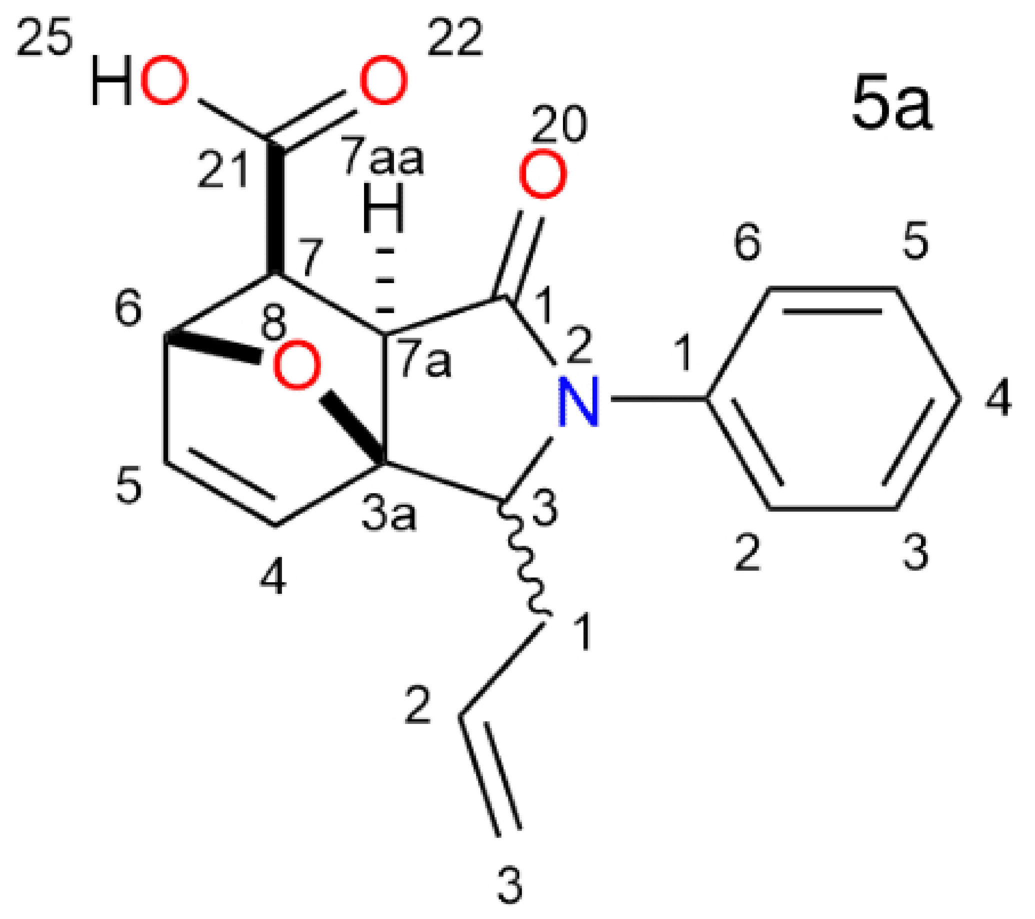

5a

FZ8743 4.PH.JDF

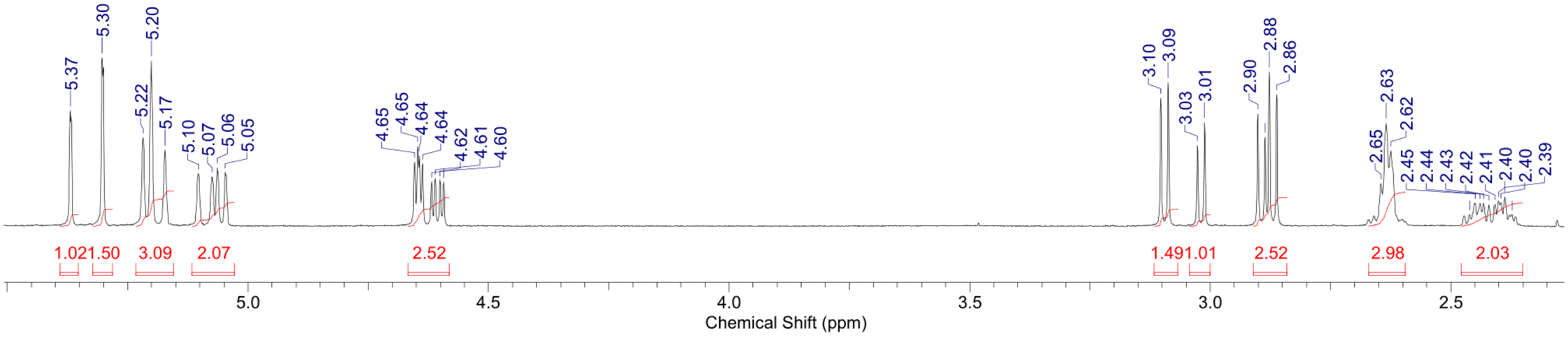

NMR spectrum data for compound 5a

26.07.2019 11:58:26

**Formula**	$C_{18}H_{17}NO_4$	**FW**	311.3319

**Acquisition Time (sec)**	0.6921	**Comment**	single pulse decoupled gated NOE			**Date**	13 May 1990 12:08:10
**Date Stamp**	23 Jul 2019 18:11:44	**File Name**	D:\Desktop\FZ7590-3.jdf			**Frequency (MHz)**	150.91
**Nucleus**	13C	**Number of Transients**	800	**Origin**	ECA 600	**Original Points Count**	32768
**Owner**	delta	**Points Count**	32768	**Pulse Sequence**	single_pulse_dec	**Receiver Gain**	56.00
**Solvent**	CHLOROFORM-d	**Spectrum Offset (Hz)**	15077.9072	**Sweep Width (Hz)**	47348.49	**Temperature (degree C)**	23.800

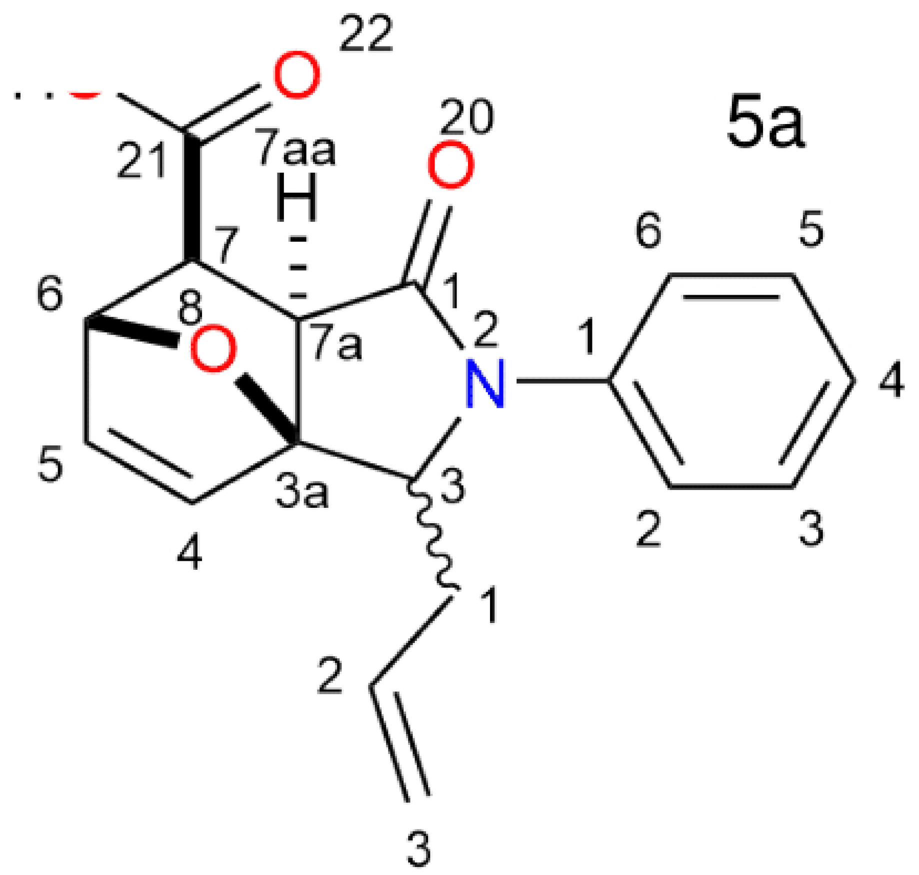

5a

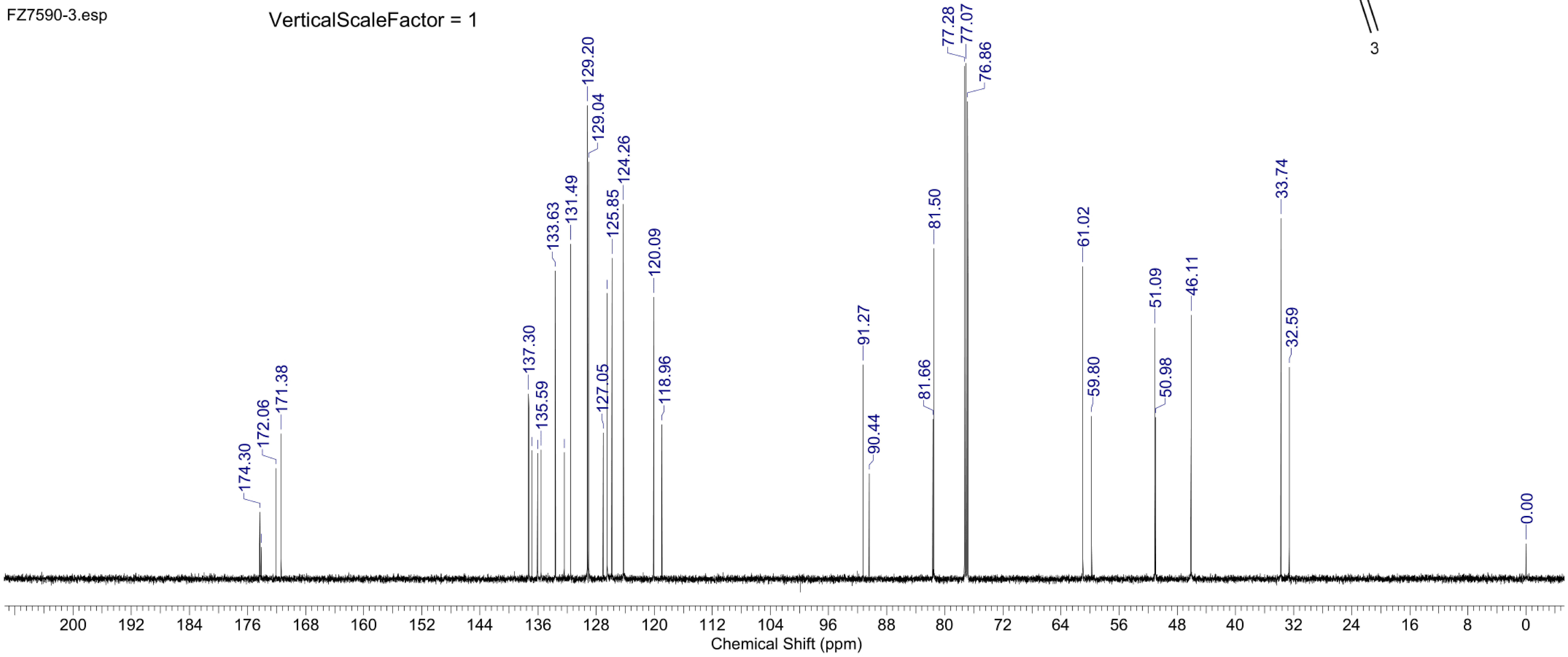

26.07.2019 11:58:43

Formula $C_{18}H_{17}NO_4$	FW 311.3319

Acquisition Time (sec)	0.6921	Comment	single pulse decoupled gated NOE			Date	13 May 1990 12:08:10
Date Stamp	23 Jul 2019 18:11:44	File Name	D:\Desktop\FZ7590-3.jdf	Frequency (MHz)	150.91		
Nucleus	13C	Number of Transients	800	Origin	ECA 600	Original Points Count	32768
Owner	delta	Points Count	32768	Pulse Sequence	single_pulse_dec	Receiver Gain	56.00
Solvent	CHLOROFORM-d	Spectrum Offset (Hz)	15077.9072	Sweep Width (Hz)	47348.49	Temperature (degree C)	23.800

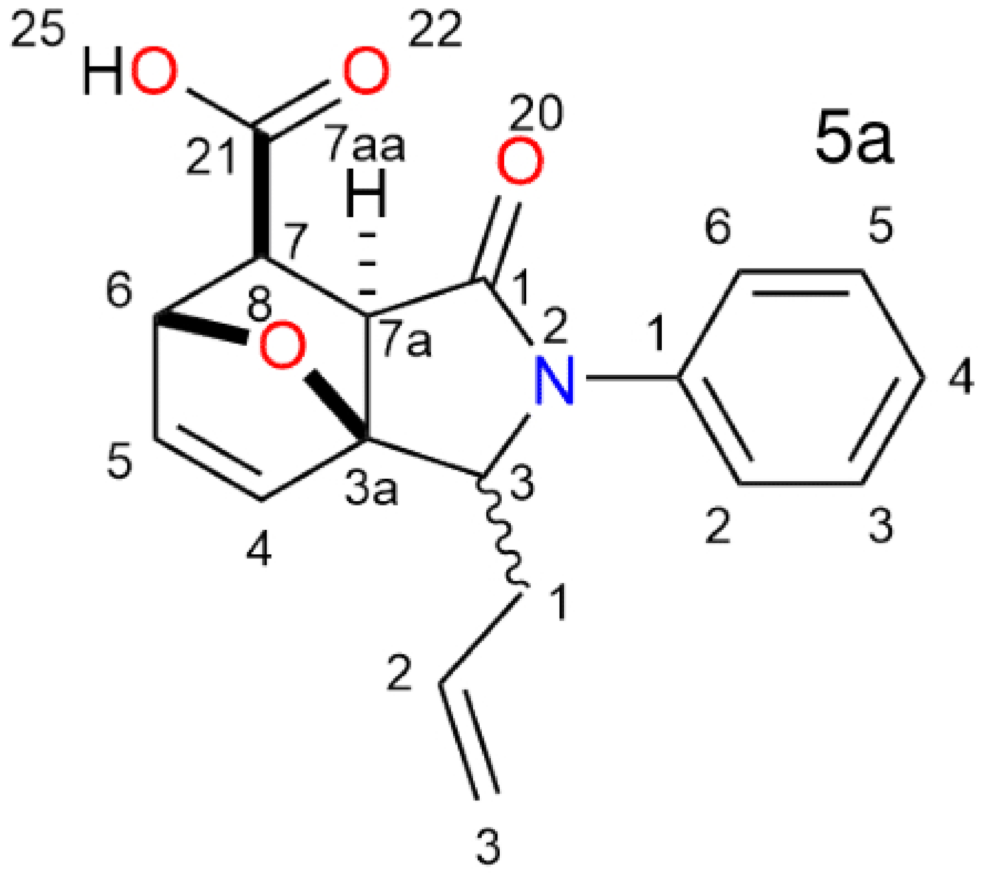

FZ7590-3.esp

VerticalScaleFactor = 1

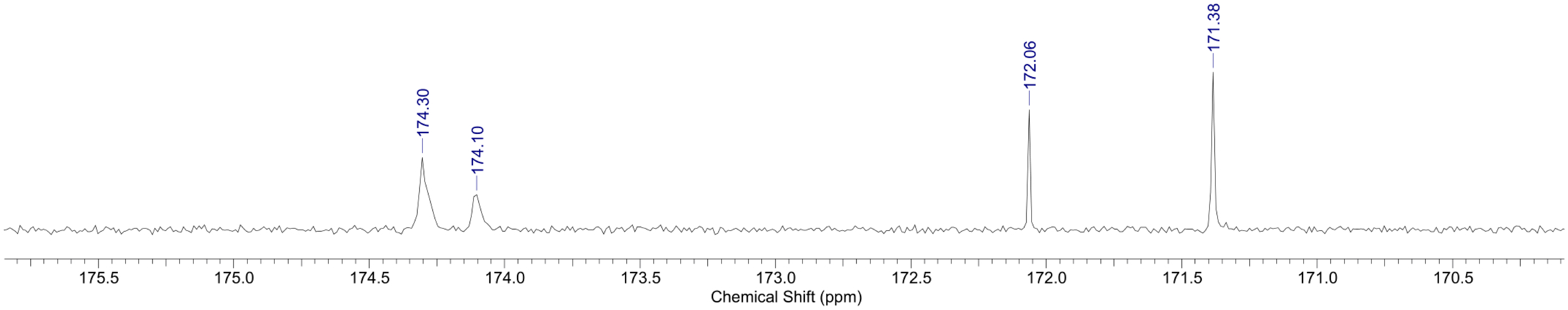

26.07.2019 11:59:03

Formula	$C_{18}H_{17}NO_4$	FW	311.3319

Acquisition Time (sec)	0.6921	Comment	single pulse decoupled gated NOE			Date	13 May 1990 12:08:10		
Date Stamp	23 Jul 2019 18:11:44			File Name	D:\Desktop\FZ7590-3.jdf			Frequency (MHz)	150.91
Nucleus	13C	Number of Transients	800	Origin	ECA 600	Original Points Count	32768	Owner	delta
Points Count	32768	Pulse Sequence	single_pulse_dec			Receiver Gain	56.00	Solvent	CHLOROFORM-d
Spectrum Offset (Hz)	15077.9072	Sweep Width (Hz)	47348.49	Temperature (degree C)	23.800				

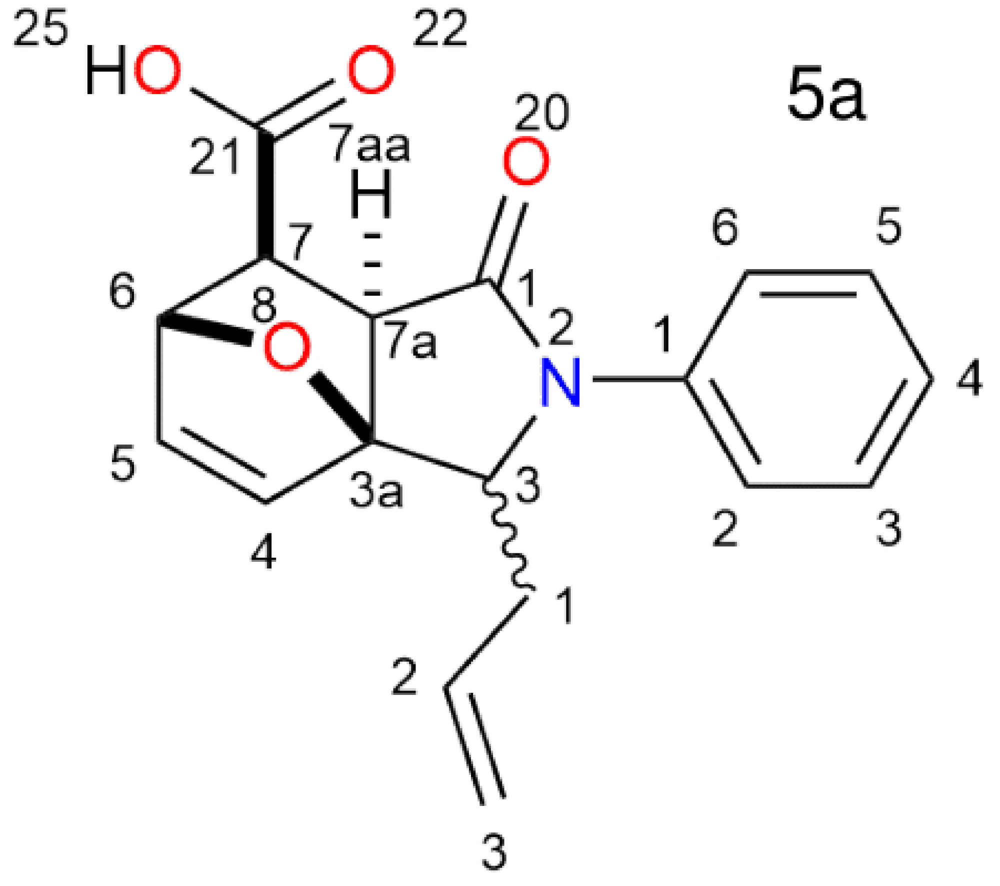

5a

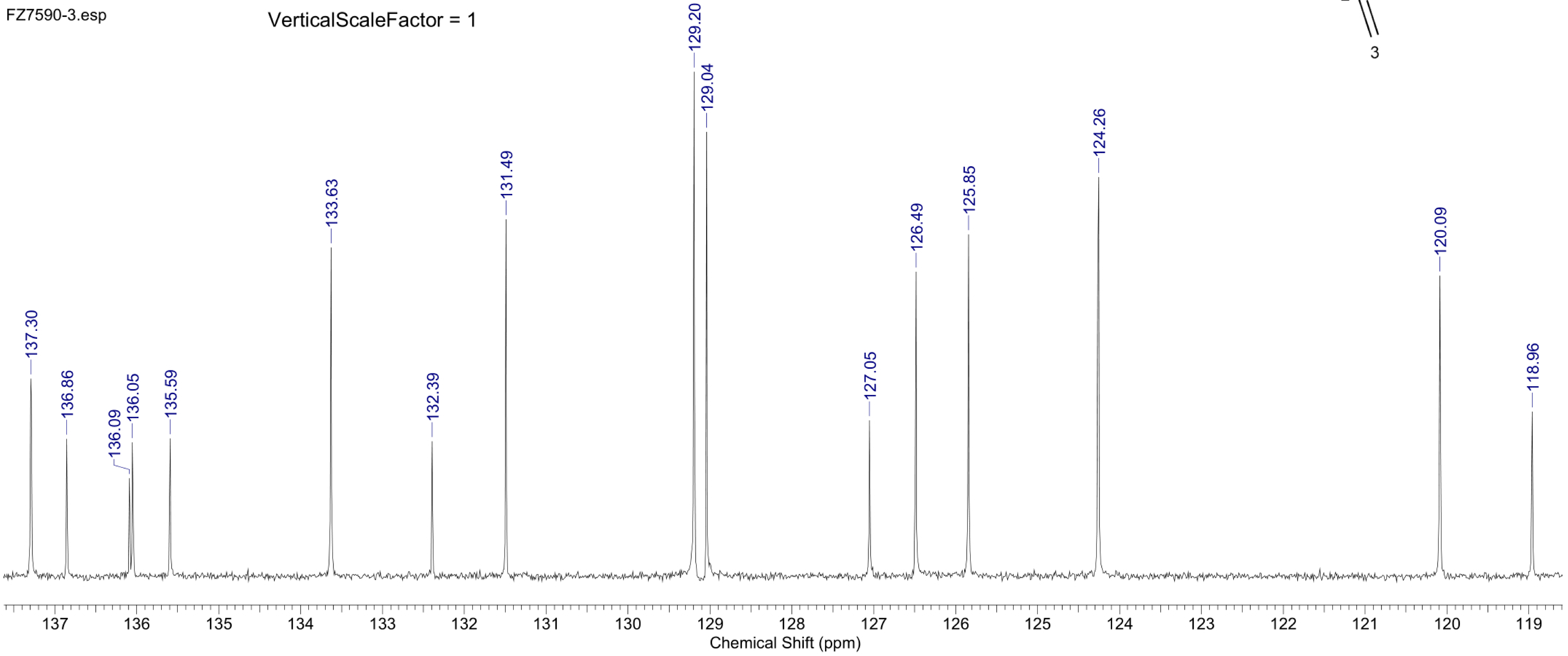

26.07.2019 11:59:29

**Formula** $C_{18}H_{17}NO_4$		**FW**	311.3319

**Acquisition Time (sec)**	0.6921	**Comment**	single pulse decoupled gated NOE			**Date**	13 May 1990 12:08:10
**Date Stamp**	23 Jul 2019 18:11:44			**File Name**	D:\Desktop\FZ7590-3.jdf	**Frequency (MHz)**	150.91
**Nucleus**	13C	**Number of Transients**	800	**Origin**	ECA 600	**Original Points Count**	32768
**Owner**	delta						
**Points Count**	32768	**Pulse Sequence**	single_pulse_dec			**Receiver Gain**	56.00
**Solvent**	CHLOROFORM-d						
**Spectrum Offset (Hz)**	15077.9072	**Sweep Width (Hz)**	47348.49	**Temperature (degree C)**	23.800		

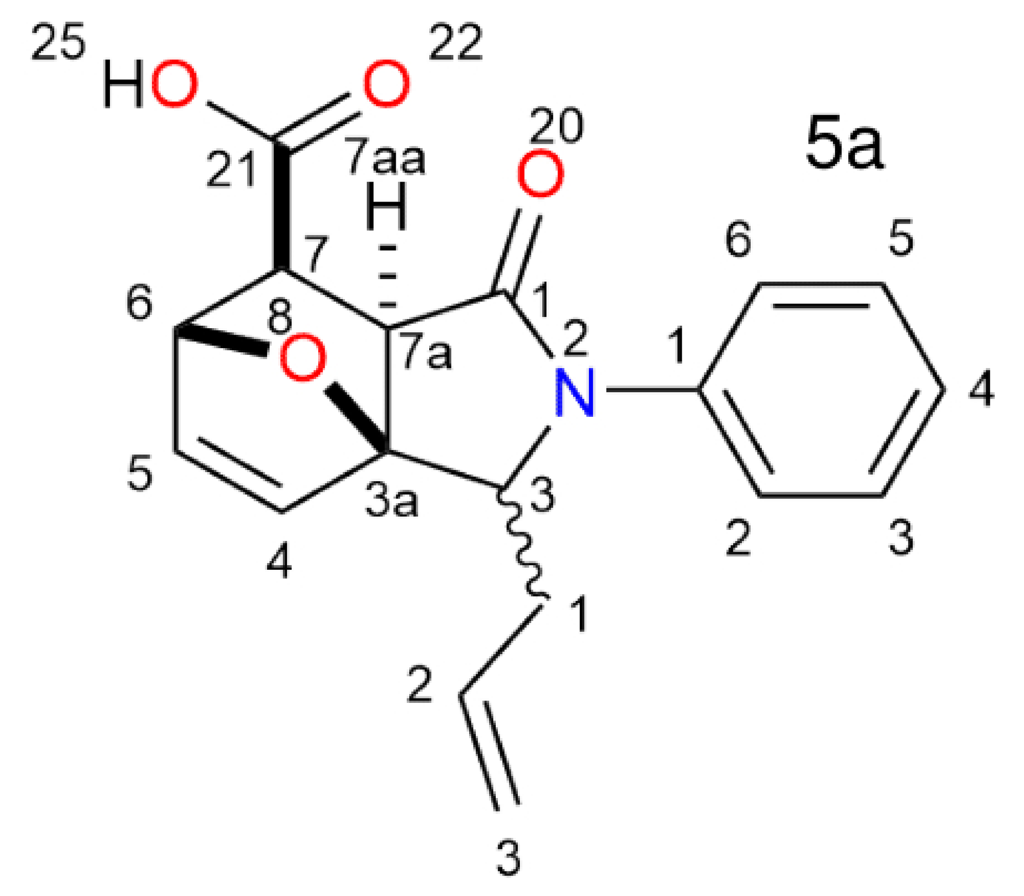

5a

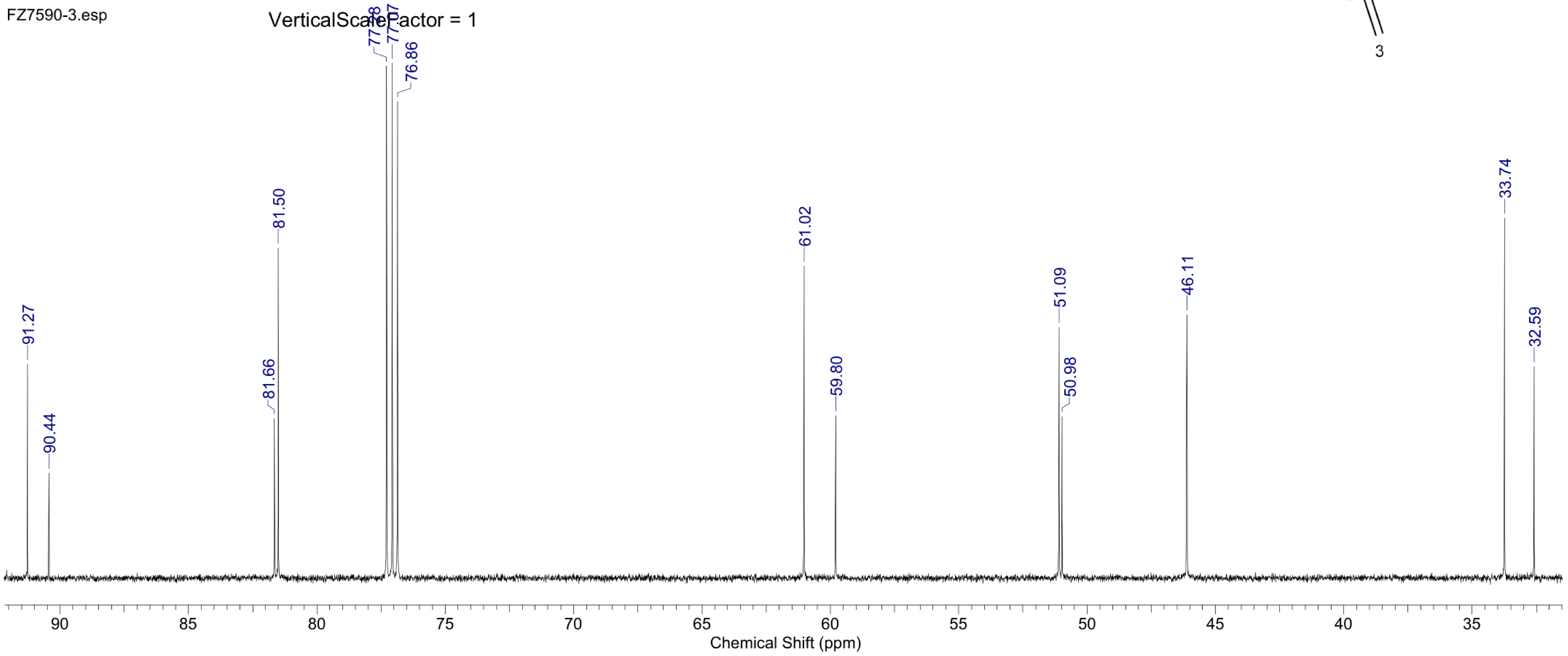

NMR spectrum data for compound 5b (DCM, −16 °C, 3 d)

**Formula**	$C_{20}H_{21}NO_4$	**FW**	339.3850

**Acquisition Time (sec)**	1.9818	**Comment**	single_pulse	**Date**	28 Jul 2020 09:28:47			**Date Stamp**	28 Jul 2020 09:30:00
**File Name**	H:\DOWNLOADS\FZ8873-1.JDF			**Frequency (MHz)**	600.17	**Nucleus**	1H	**Number of Transients**	8
**Origin**	ECA 600	**Original Points Count**	32768	**Owner**	CKP	**Points Count**	32768	**Pulse Sequence**	single_pulse.ex2
**Receiver Gain**	40.00	**Solvent**	CHLOROFORM-d			**Spectrum Offset (Hz)**	5410.6274	**Sweep Width (Hz)**	16534.39

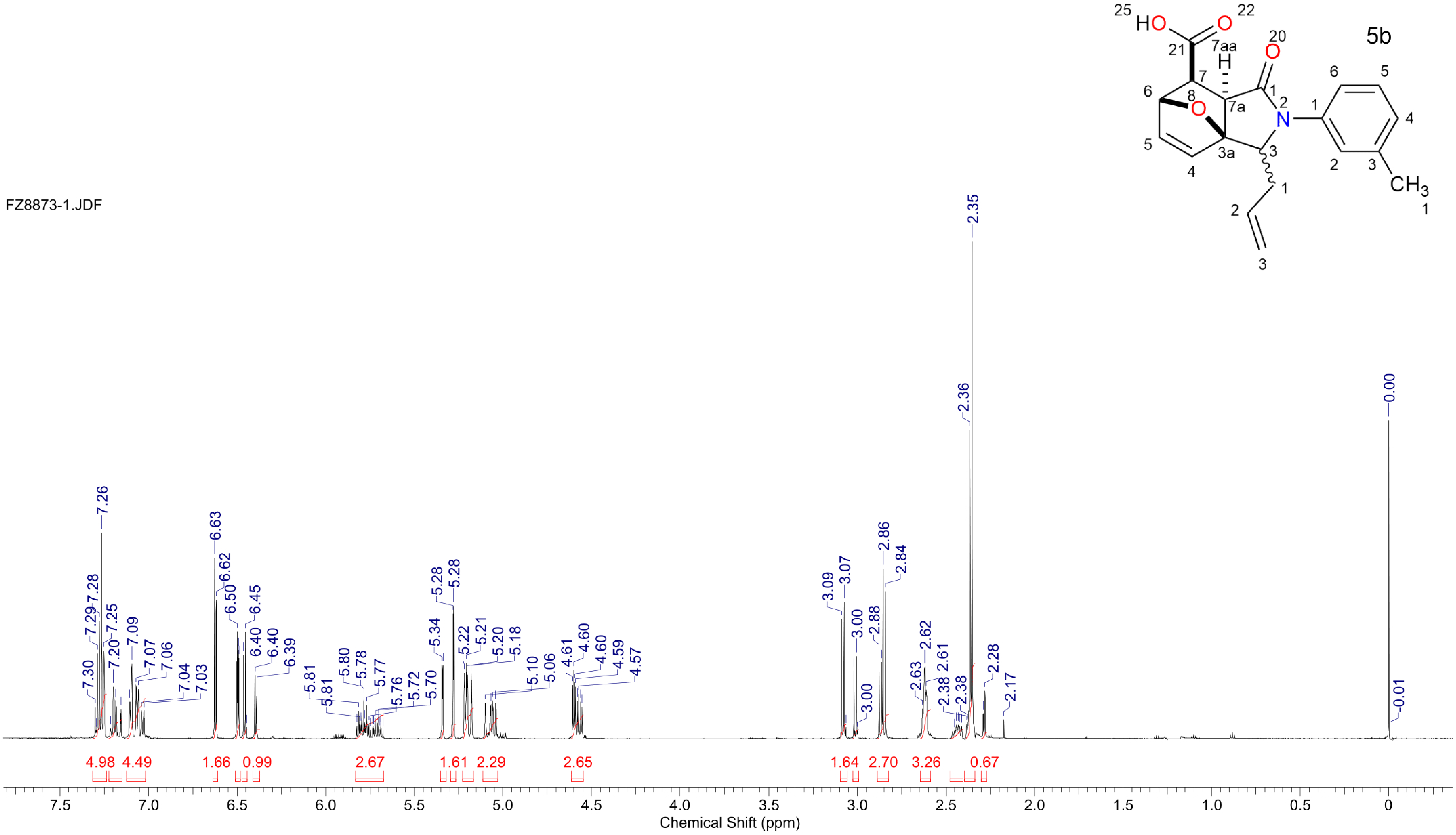

Formula $C_{20}H_{21}NO_4$	FW 339.3850

**Acquisition Time (sec)**	1.9818	**Comment**	single_pulse	**Date**	28 Jul 2020 09:28:47			**Date Stamp**	28 Jul 2020 09:30:00
**File Name**	H:\DOWNLOADS\FZ8873-1.JDF			**Frequency (MHz)**	600.17	**Nucleus**	1H	**Number of Transients**	8
**Origin**	ECA 600	**Original Points Count**	32768	**Owner**	CKP	**Points Count**	32768	**Pulse Sequence**	single_pulse.ex2
**Receiver Gain**	40.00	**Solvent**	CHLOROFORM-d			**Spectrum Offset (Hz)**	5410.6274	**Sweep Width (Hz)**	16534.39

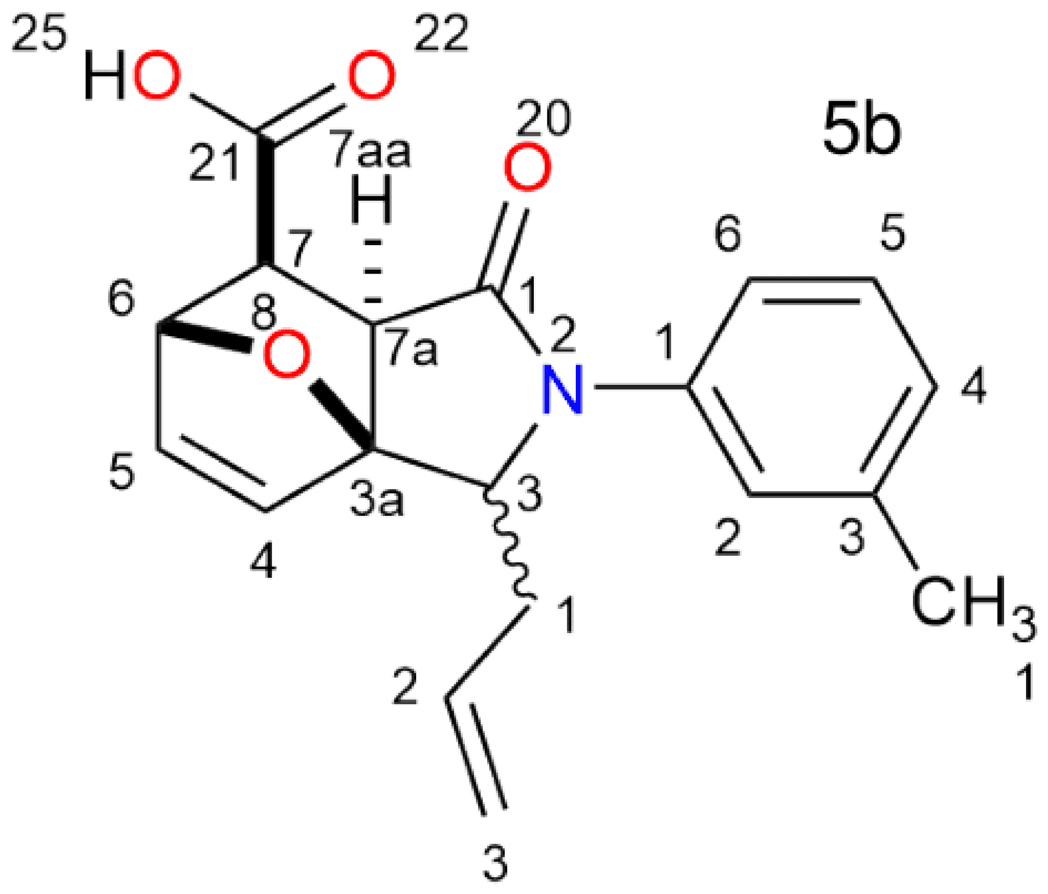

5b

FZ8873-1.JDF

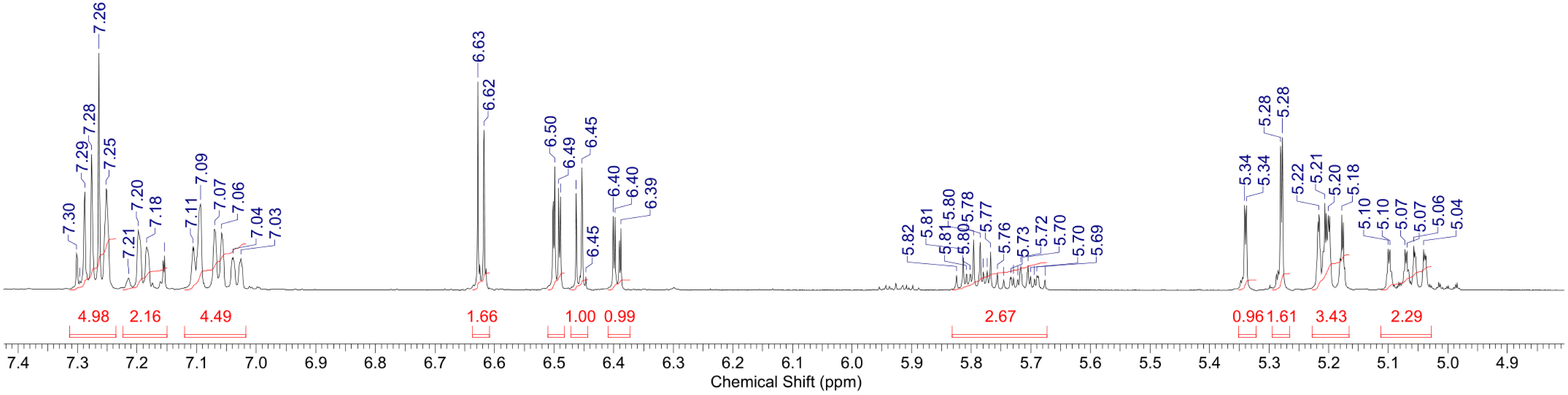

Formula $C_{20}H_{21}NO_4$	FW 339.3850

**Acquisition Time (sec)**	1.9818	**Comment**	single_pulse	**Date**	28 Jul 2020 09:28:47			**Date Stamp**	28 Jul 2020 09:30:00
**File Name**	H:\DOWNLOADS\FZ8873-1.JDF			**Frequency (MHz)**	600.17	**Nucleus**	1H	**Number of Transients**	8
**Origin**	ECA 600	**Original Points Count**	32768	**Owner**	CKP	**Points Count**	32768	**Pulse Sequence**	single_pulse.ex2
**Receiver Gain**	40.00	**Solvent**	CHLOROFORM-d			**Spectrum Offset (Hz)**	5410.6274	**Sweep Width (Hz)**	16534.39

5b

FZ8873-1.JDF

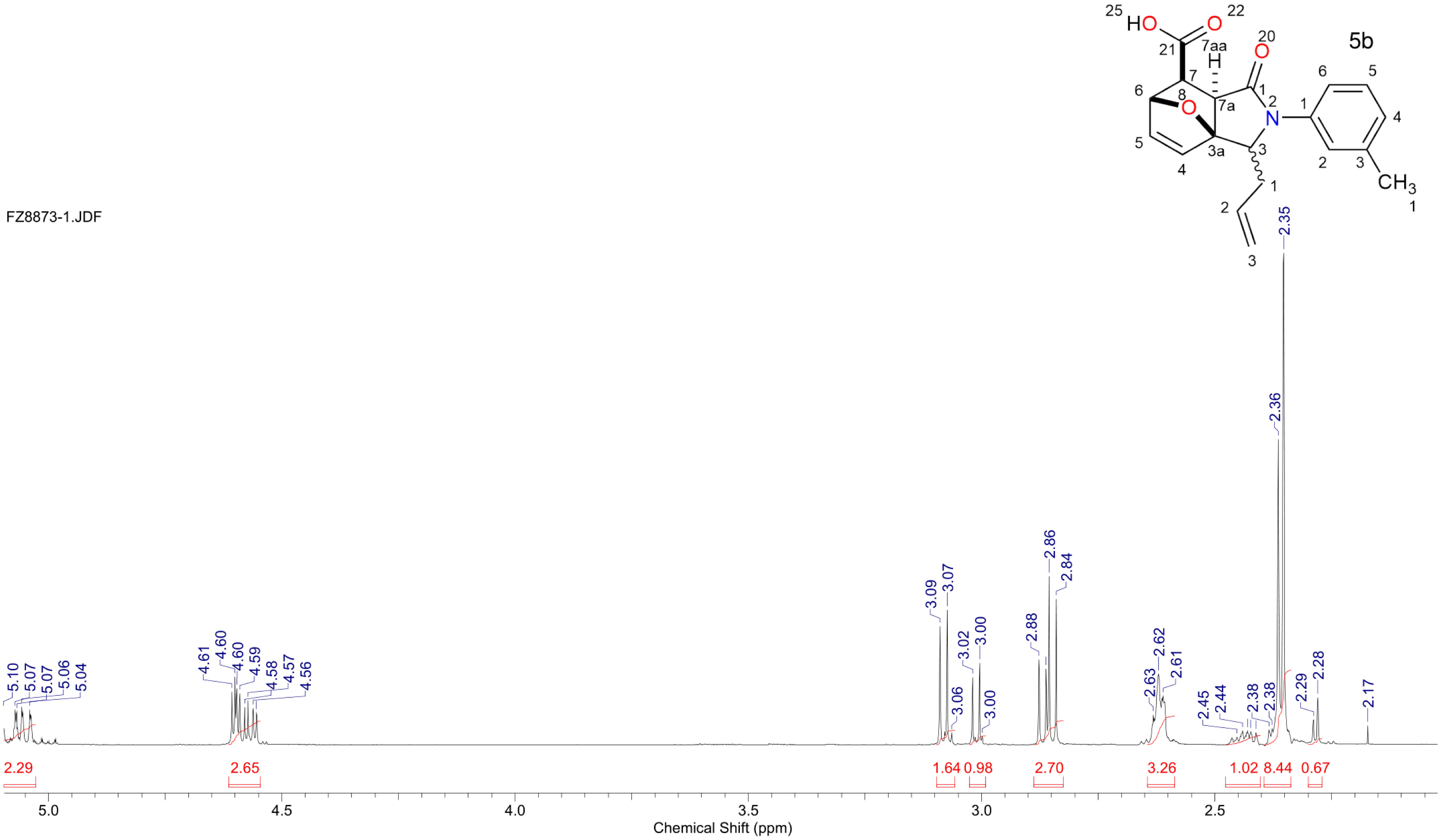

NMR spectrum data for compound 5b (DCM, −16 °C, 3 d)

**Formula** $C_{20}H_{21}NO_4$	**FW** 339.3850

**Acquisition Time (sec)**	1.9818	**Comment**	single_pulse	**Date**	28 Jul 2020 09:28:47			**Date Stamp**	28 Jul 2020 09:30:00
**File Name**	H:\DOWNLOADS\FZ8873-1.JDF			**Frequency (MHz)**	600.17	**Nucleus**	1H	**Number of Transients**	8
**Origin**	ECA 600	**Original Points Count**	32768	**Owner**	CKP	**Points Count**	32768	**Pulse Sequence**	single_pulse.ex2
**Receiver Gain**	40.00	**Solvent**	CHLOROFORM-d			**Spectrum Offset (Hz)**	5410.6274	**Sweep Width (Hz)**	16534.39

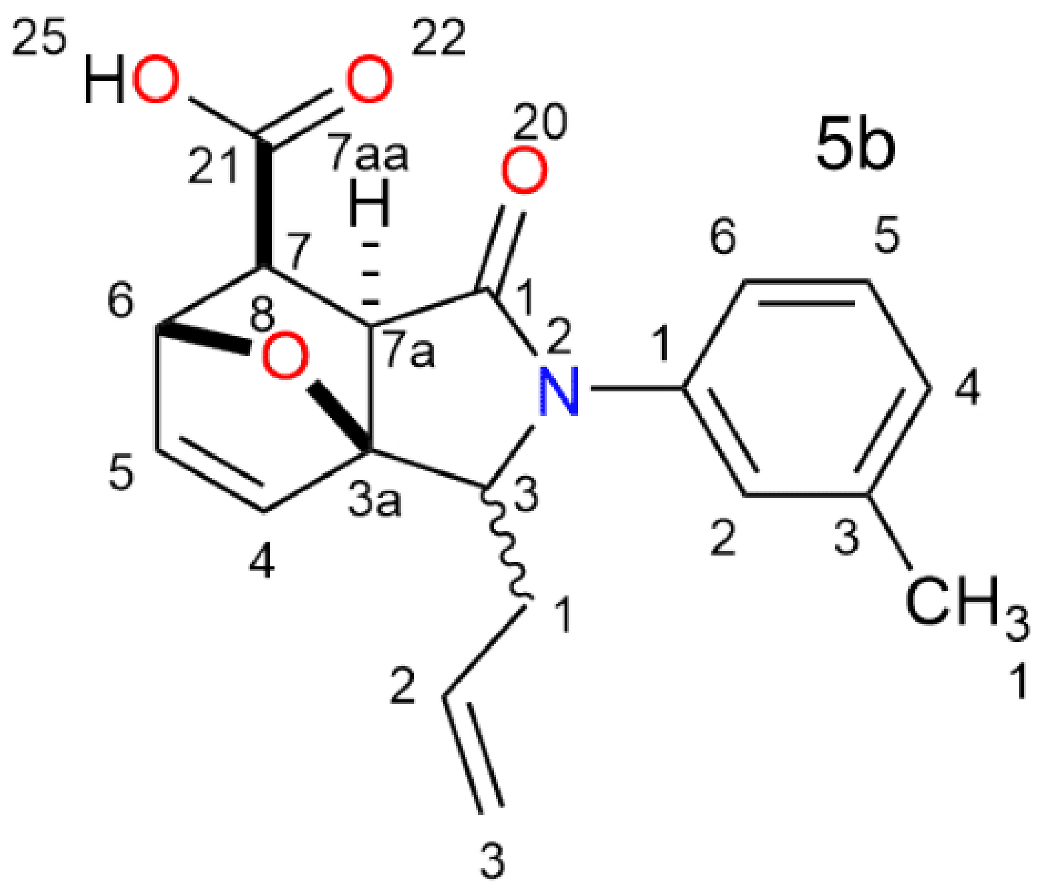

FZ8873-1.JDF

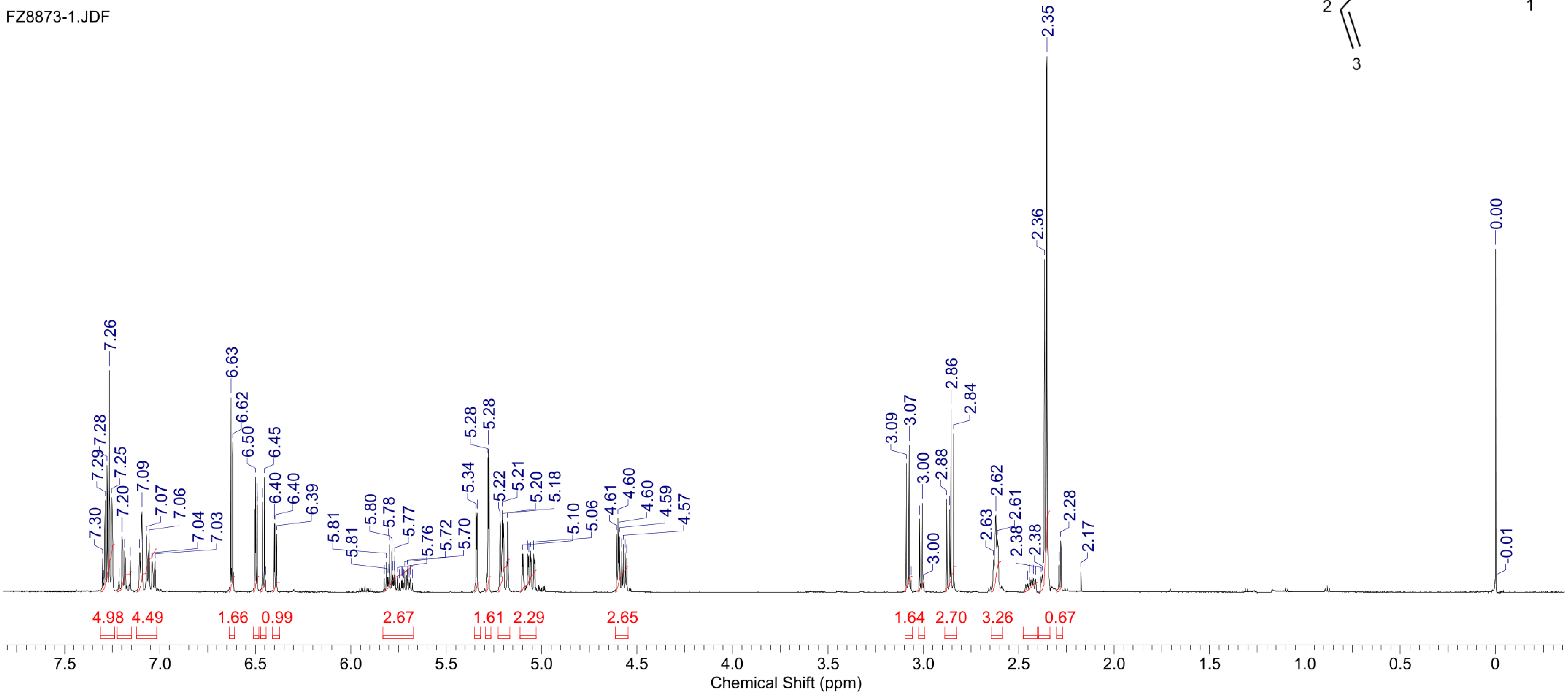

**Formula** $C_{20}H_{21}NO_4$	**FW** 339.3850

Acquisition Time (sec)	1.9818	***Comment***	single_pulse	***Date***	28 Jul 2020 09:28:47			***Date Stamp***	28 Jul 2020 09:30:00
File Name	H:\DOWNLOADS\FZ8873-1.JDF			***Frequency (MHz)***	600.17	***Nucleus***	1H	***Number of Transients***	8
Origin	ECA 600	***Original Points Count***	32768	***Owner***	CKP	***Points Count***	32768	***Pulse Sequence***	single_pulse.ex2
Receiver Gain	40.00	***Solvent***	CHLOROFORM-d			***Spectrum Offset (Hz)***	5410.6274	***Sweep Width (Hz)***	16534.39

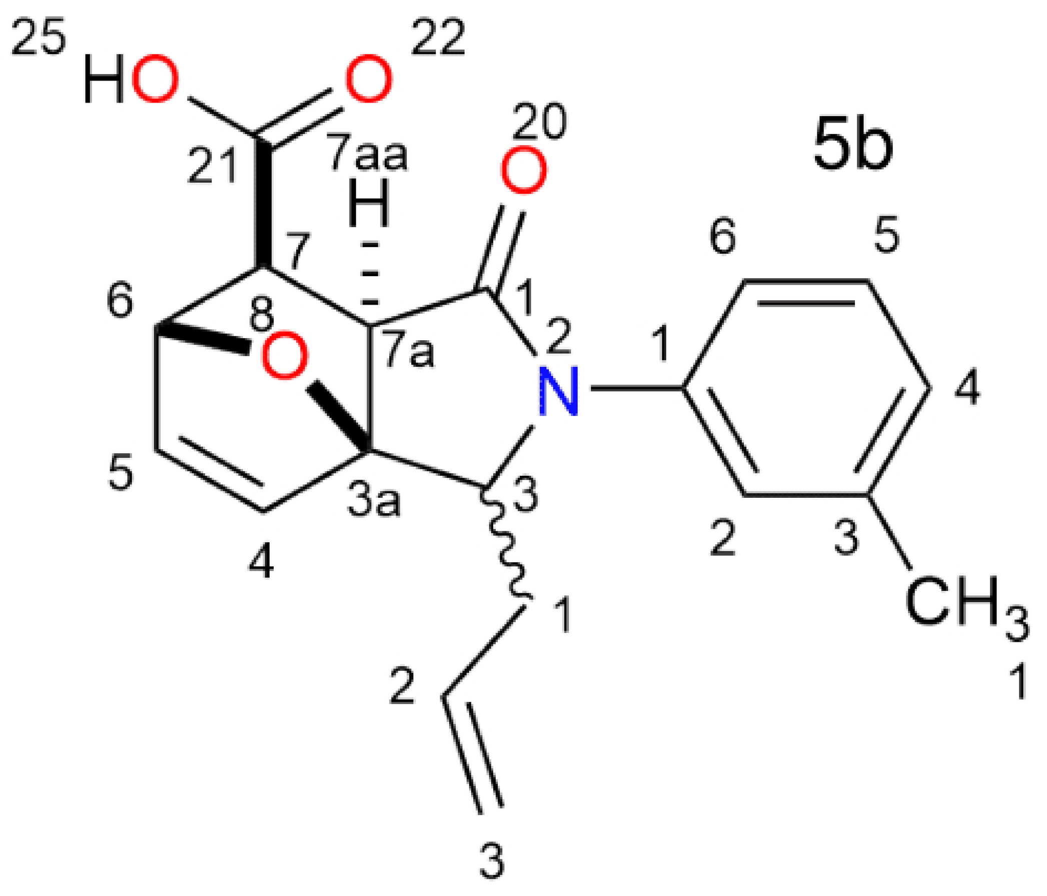

FZ8873-1.JDF

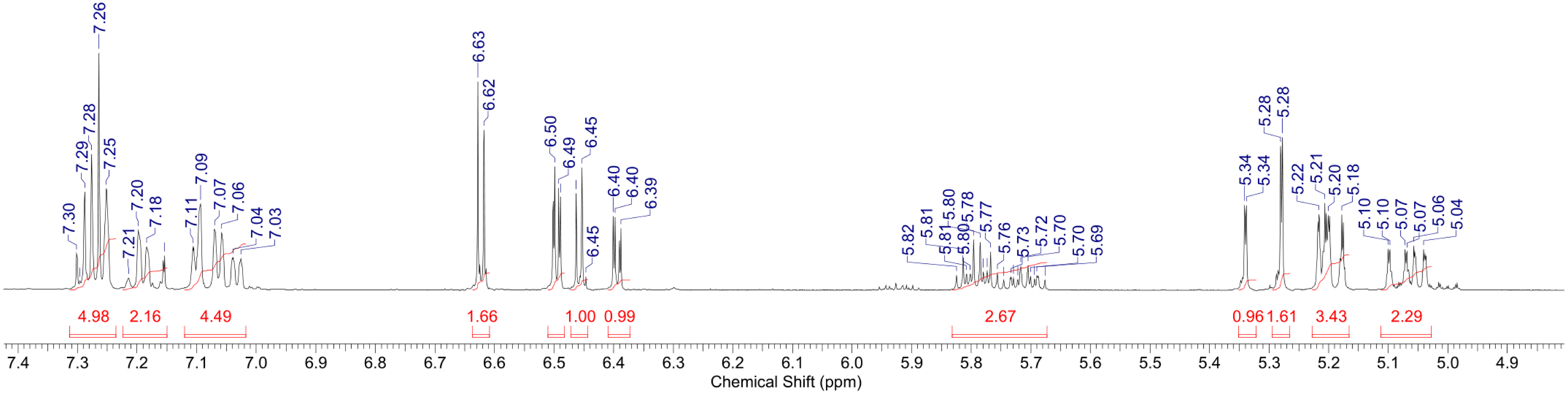

Formula $C_{20}H_{21}NO_4$	FW 339.3850

Acquisition Time (sec)	1.9818	Comment	single_pulse	Date	28 Jul 2020 09:28:47			Date Stamp	28 Jul 2020 09:30:00
File Name	H:\DOWNLOADS\FZ8873-1.JDF			Frequency (MHz)	600.17	Nucleus	1H	Number of Transients	8
Origin	ECA 600	Original Points Count	32768	Owner	CKP	Points Count	32768	Pulse Sequence	single_pulse.ex2
Receiver Gain	40.00	Solvent	CHLOROFORM-d			Spectrum Offset (Hz)	5410.6274	Sweep Width (Hz)	16534.39

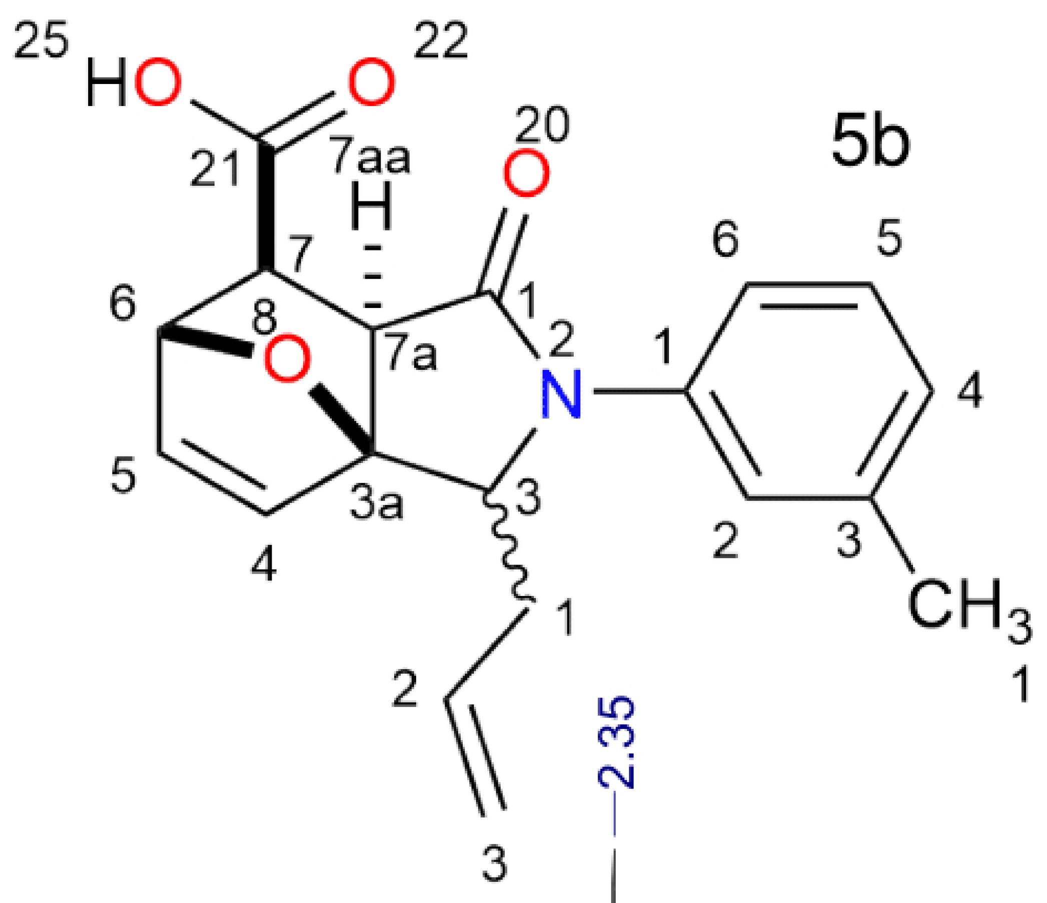

5b

FZ8873-1.JDF

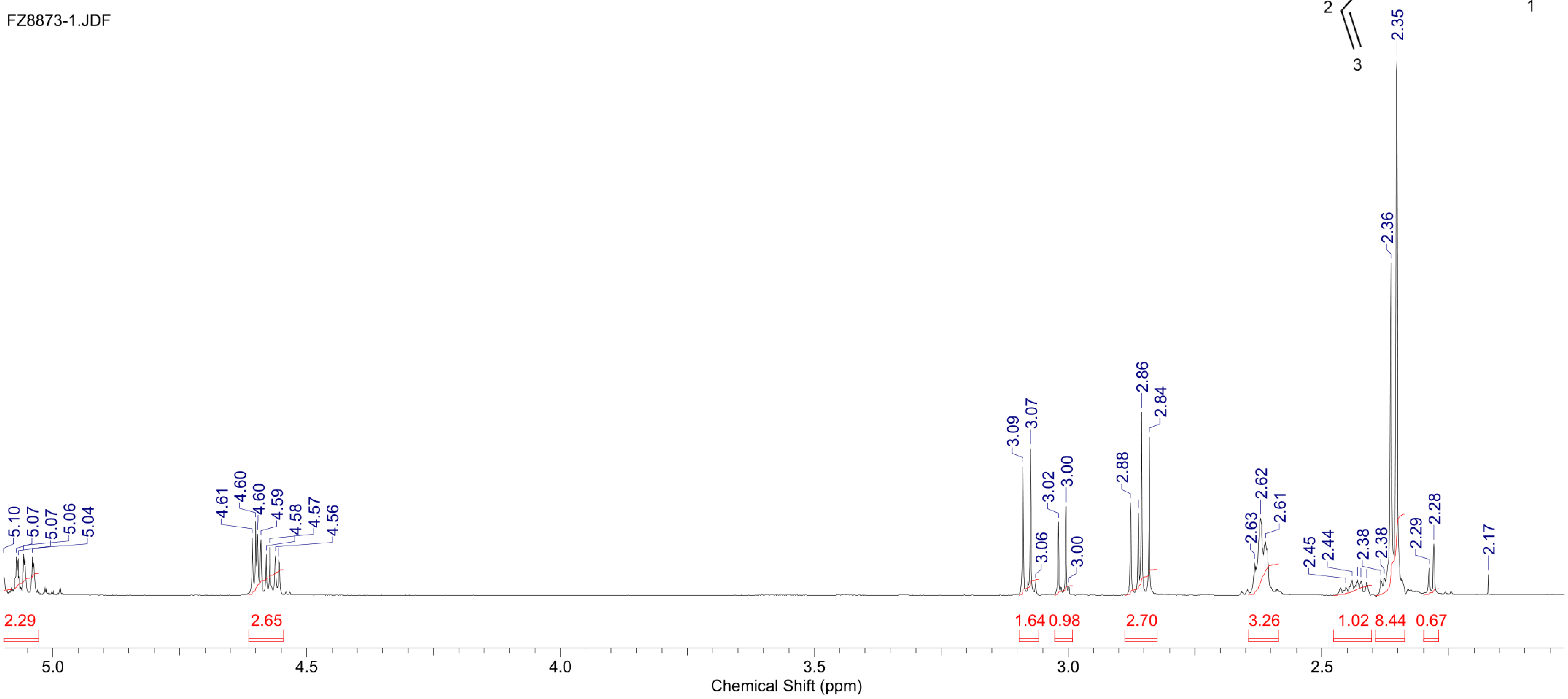

NMR spectrum data for compound 5b (MeCN, r.t, 3 d)

Acquisition Time (sec)	1.9818	***Comment***	single_pulse	***Date***	05 Aug 2020 10:24:21	***Date Stamp***	05 Aug 2020 10:25:02
File Name	C:\USERS\FEDOR\DOWNLOADS\FZ8940-1.JDF	***Frequency (MHz)***	600.17	***Nucleus***	1H	***Number of Transients***	8
Origin	ECA 600	***Original Points Count***	32768	***Owner***	CKP	***Points Count***	32768
Pulse Sequence	single_pulse.ex2	***Receiver Gain***	42.00	***Solvent***	CHLOROFORM-d	***Spectrum Offset (Hz)***	5410.6274
Sweep Width (Hz)	16534.39						

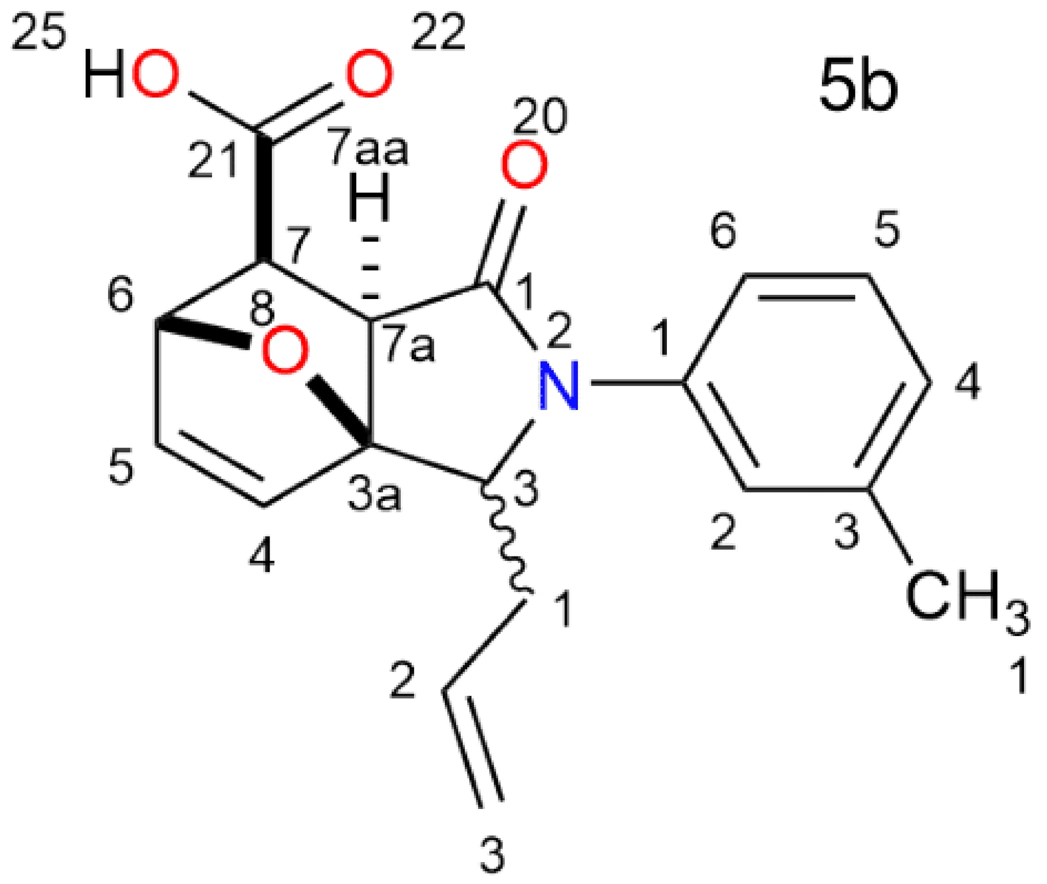

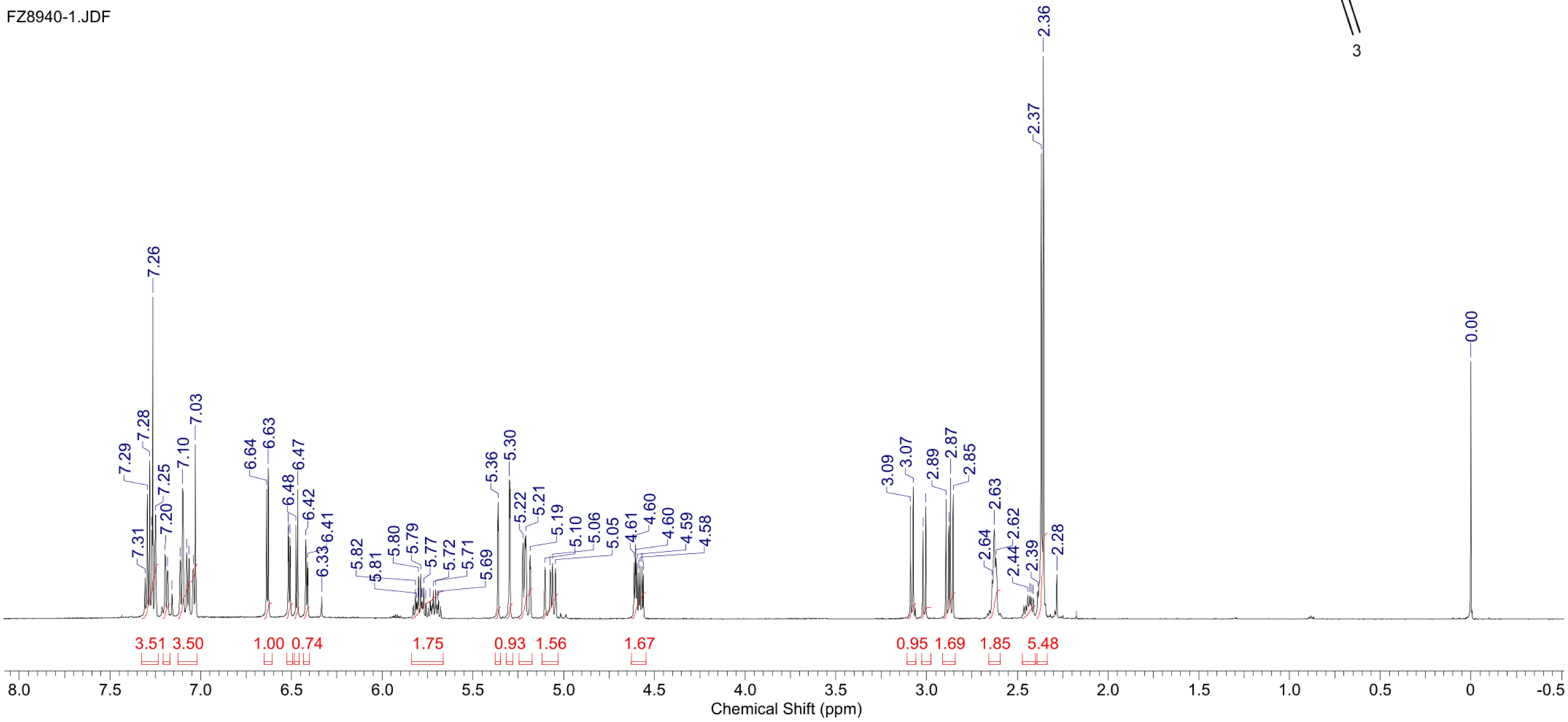

Acquisition Time (sec)	1.9818	Comment	single_pulse	Date	05 Aug 2020 10:24:21			Date Stamp	05 Aug 2020 10:25:02
File Name	C:\USERS\FEDOR\DOWNLOADS\FZ8940-1.JDF			Frequency (MHz)	600.17	Nucleus	1H	Number of Transients	8
Origin	ECA 600	Original Points Count	32768	Owner	CKP	Points Count	32768	Pulse Sequence	single_pulse.ex2
Receiver Gain	42.00	Solvent	CHLOROFORM-d			Spectrum Offset (Hz)	5410.6274	Sweep Width (Hz)	16534.39

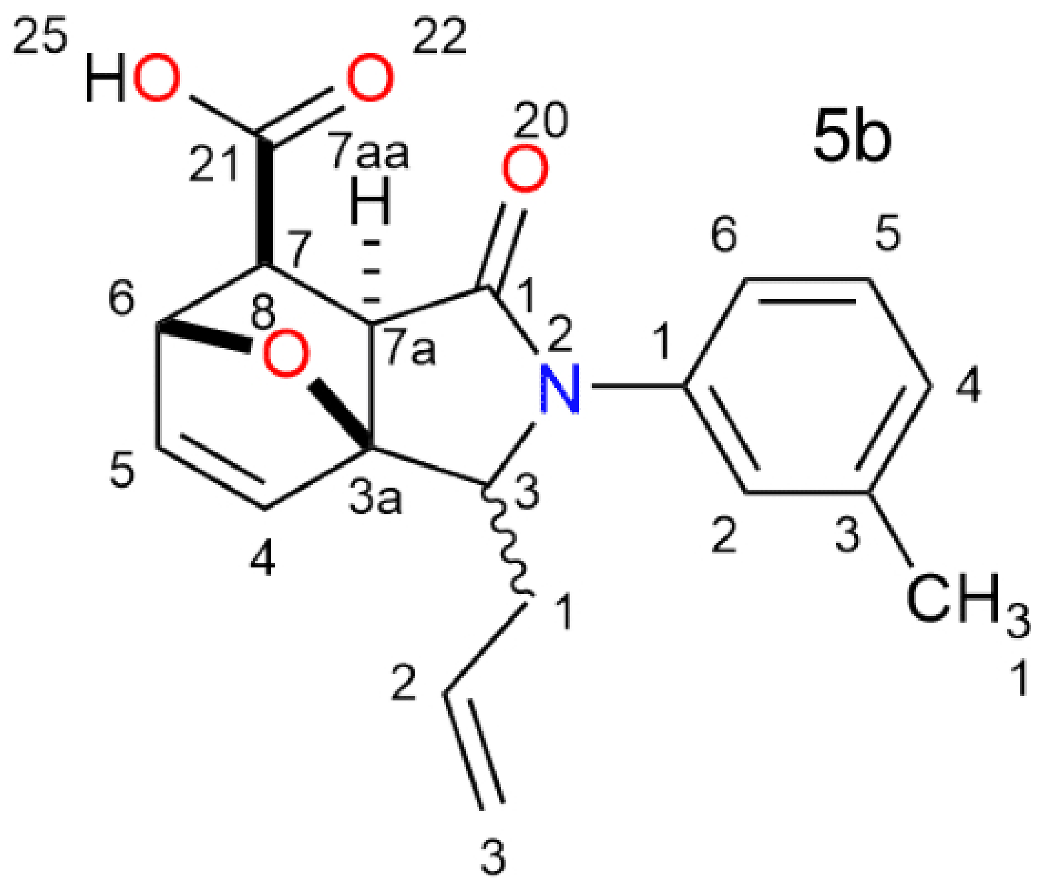

5b

FZ8940-1.JDF

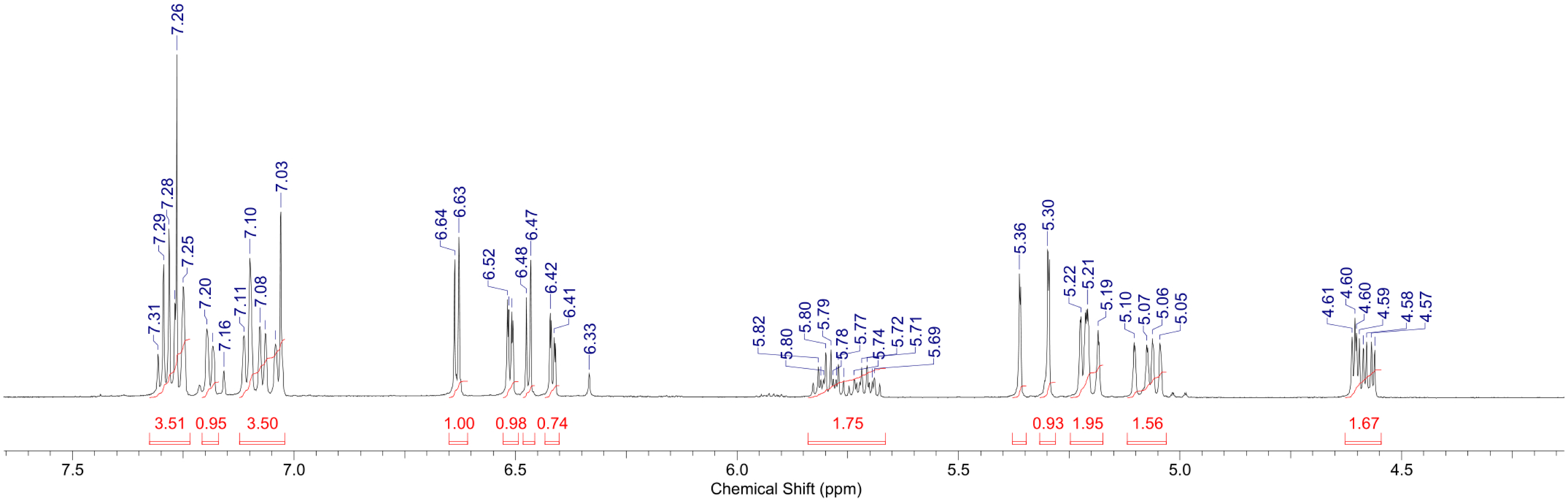

Acquisition Time (sec)	1.9818	***Comment***	single_pulse	***Date***	05 Aug 2020 10:24:21			***Date Stamp***	05 Aug 2020 10:25:02
File Name	C:\USERS\FEDOR\DOWNLOADS\FZ8940-1.JDF			***Frequency (MHz)***	600.17	***Nucleus***	1H	***Number of Transients***	8
Origin	ECA 600	***Original Points Count***	32768	***Owner***	CKP	***Points Count***	32768	***Pulse Sequence***	single_pulse.ex2
Receiver Gain	42.00	***Solvent***	CHLOROFORM-d			***Spectrum Offset (Hz)***	5410.6274	***Sweep Width (Hz)***	16534.39

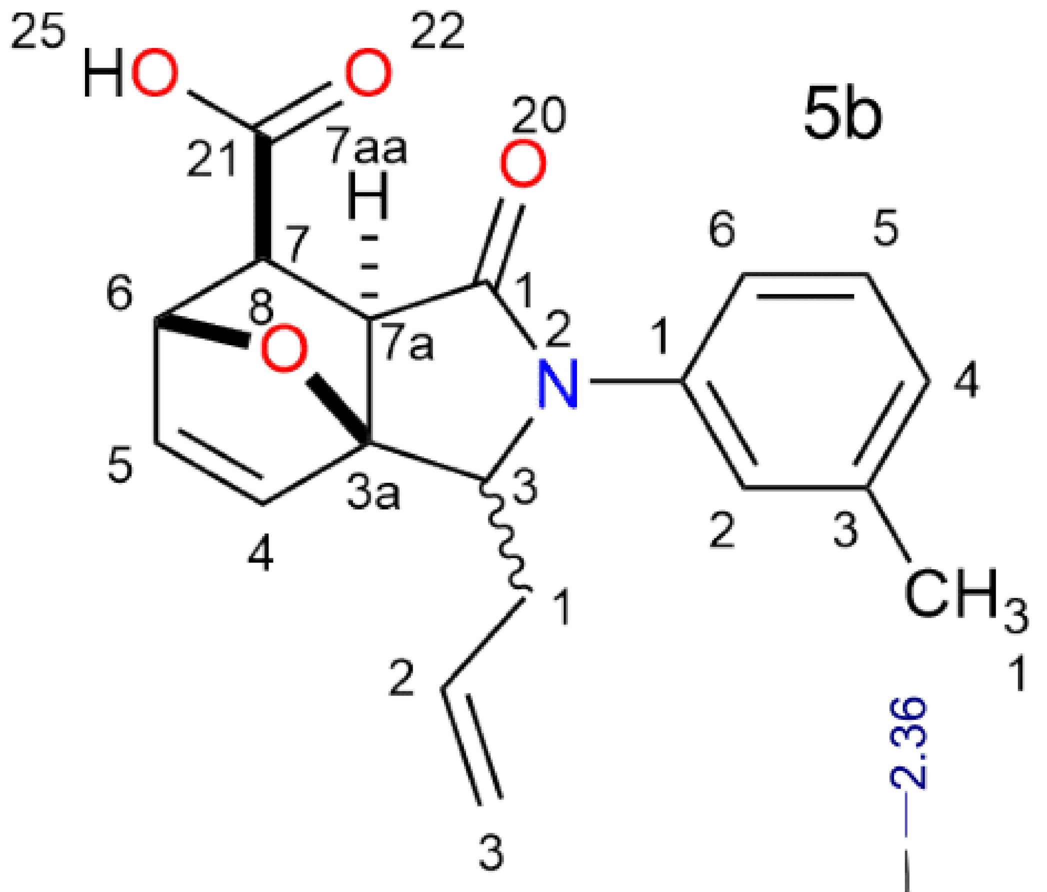

5b

FZ8940-1.JDF

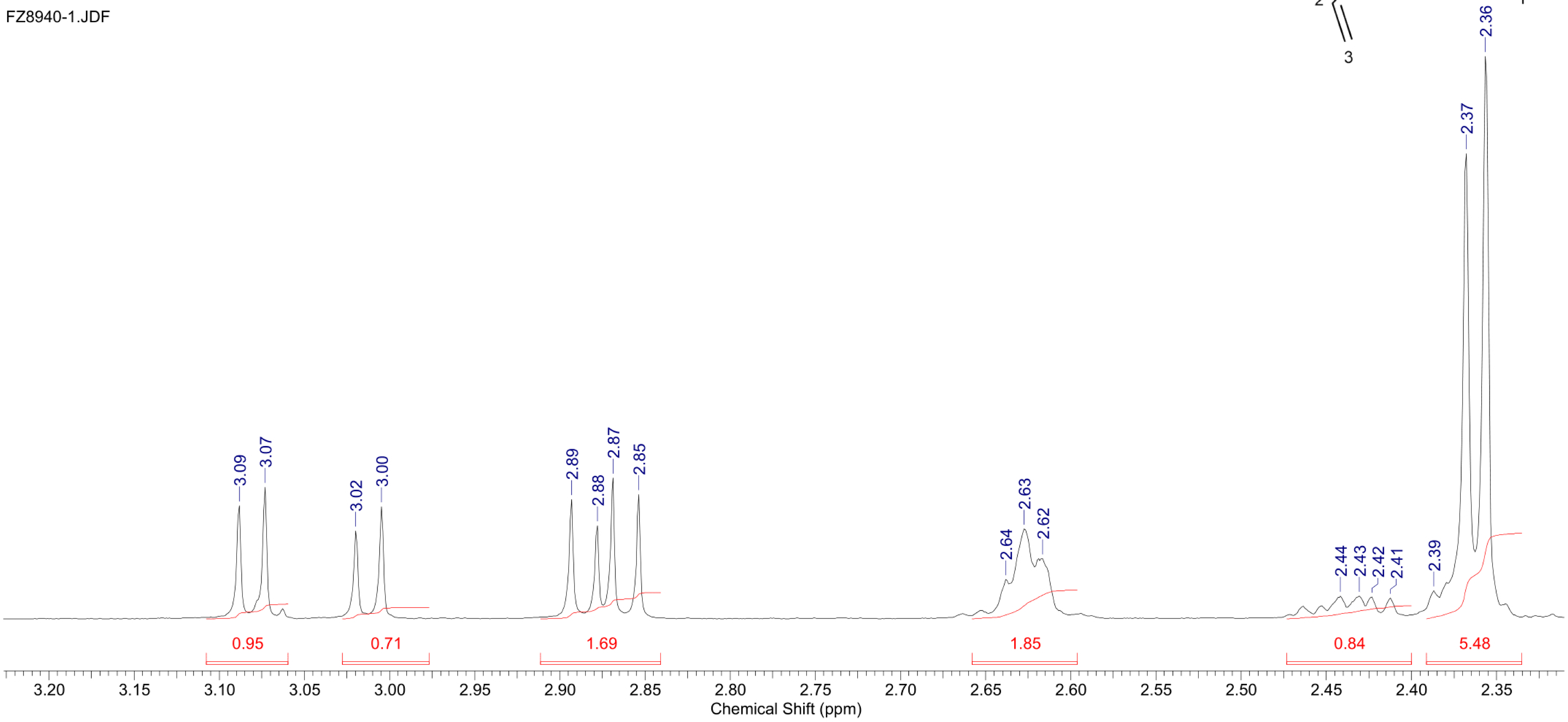

NMR spectrum data for compound 5b (MeCN, Δ, 3 h)

Acquisition Time (sec)	1.9818	Comment	single_pulse	Date	05 Aug 2020 10:39:36			Date Stamp	05 Aug 2020 10:40:17
File Name	C:\USERS\FEDOR\DOWNLOADS\FZ8941-1.JDF			Frequency (MHz)	600.17	Nucleus	1H	Number of Transients	8
Origin	ECA 600	Original Points Count	32768	Owner	CKP	Points Count	32768	Pulse Sequence	single_pulse.ex2
Receiver Gain	44.00	Solvent	CHLOROFORM-d			Spectrum Offset (Hz)	5411.1318	Sweep Width (Hz)	16534.39

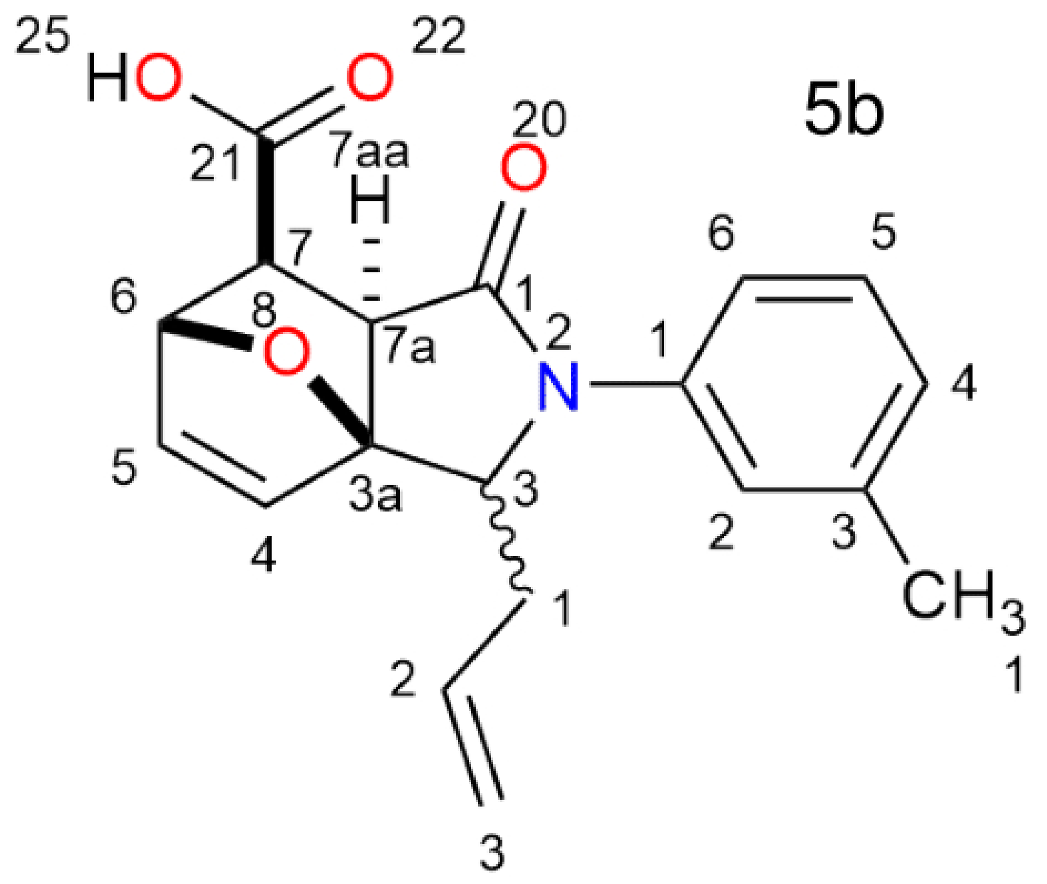

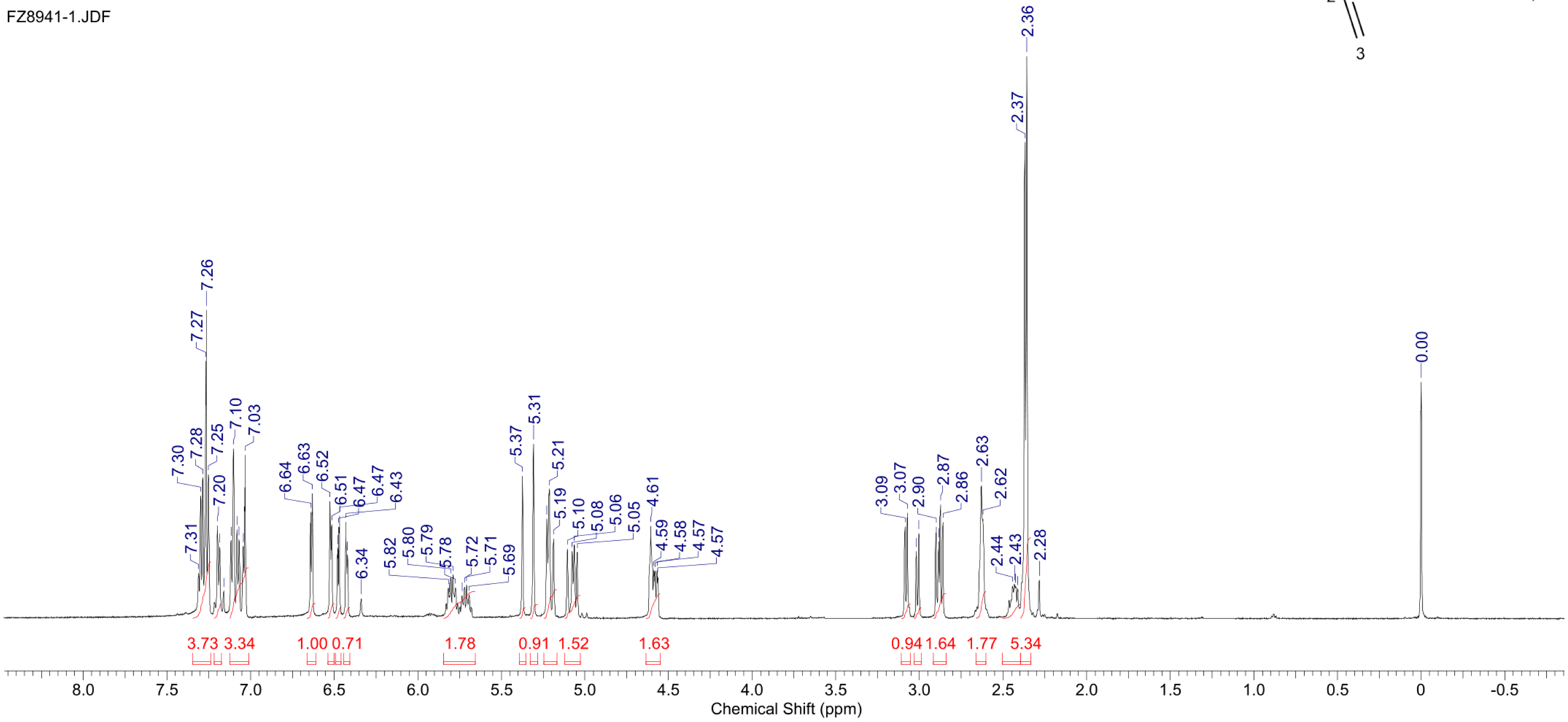

Acquisition Time (sec)	1.9818	***Comment***	single_pulse	***Date***	05 Aug 2020 10:39:36	***Date Stamp***	05 Aug 2020 10:40:17
File Name	C:\USERS\FEDOR\DOWNLOADS\FZ8941-1.JDF	***Frequency (MHz)***	600.17	***Nucleus***	1H	***Number of Transients***	8
Origin	ECA 600	***Original Points Count***	32768	***Owner***	CKP	***Points Count***	32768
Pulse Sequence	single_pulse.ex2	***Receiver Gain***	44.00	***Solvent***	CHLOROFORM-d	***Spectrum Offset (Hz)***	5411.1318
Sweep Width (Hz)	16534.39						

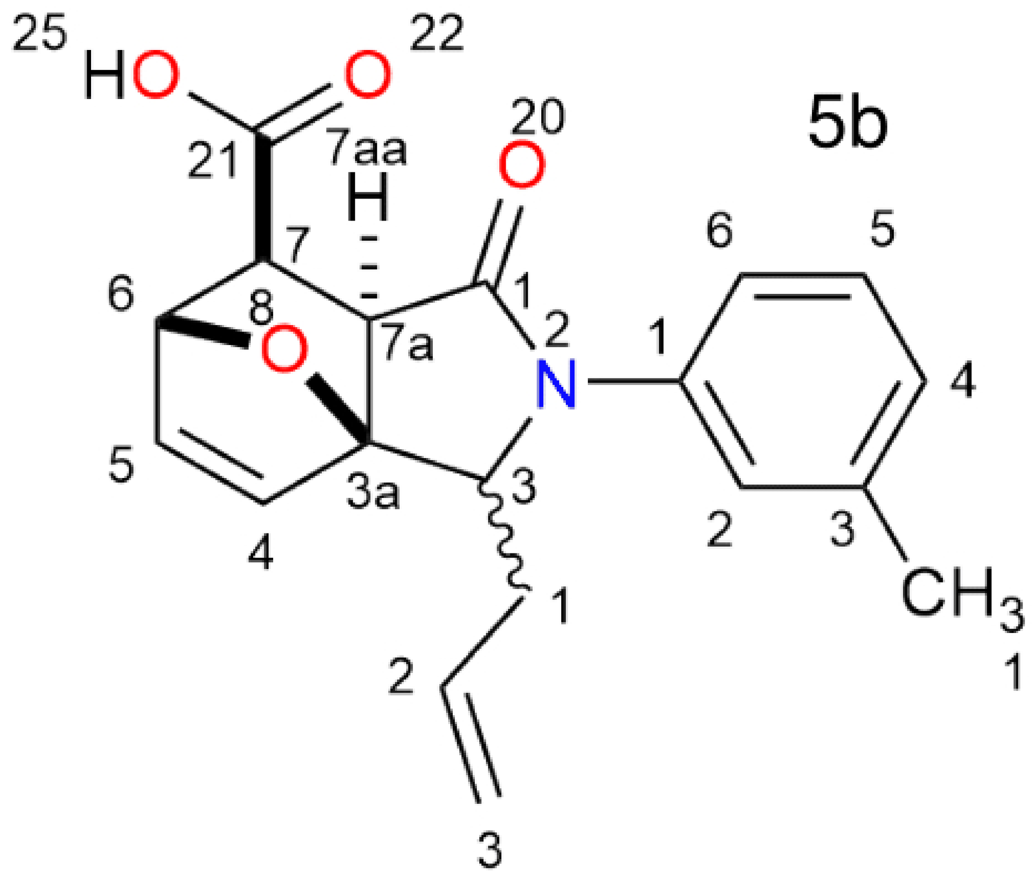

5b

FZ8941-1.JDF

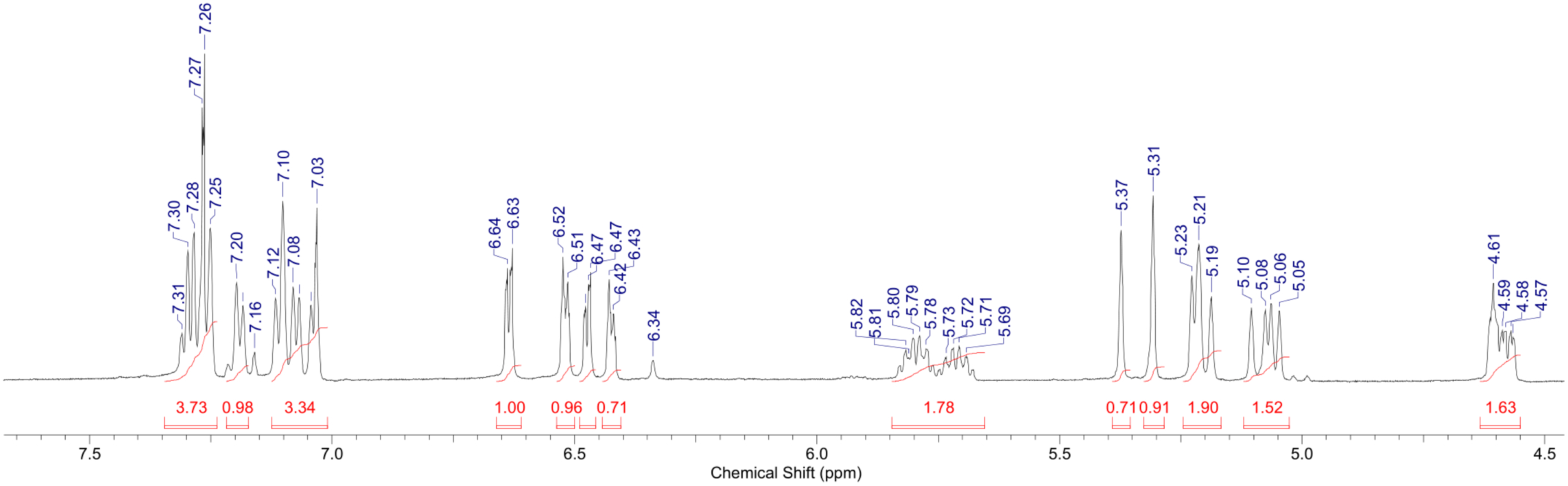

**Acquisition Time (sec)**	1.9818	**Comment**	single_pulse	**Date**	05 Aug 2020 10:39:36			**Date Stamp**	05 Aug 2020 10:40:17
**File Name**	C:\USERS\FEDOR\DOWNLOADS\FZ8941-1.JDF			**Frequency (MHz)**	600.17	**Nucleus**	1H	**Number of Transients**	8
**Origin**	ECA 600	**Original Points Count**	32768	**Owner**	CKP	**Points Count**	32768	**Pulse Sequence**	single_pulse.ex2
**Receiver Gain**	44.00	**Solvent**	CHLOROFORM-d			**Spectrum Offset (Hz)**	5411.1318	**Sweep Width (Hz)**	16534.39

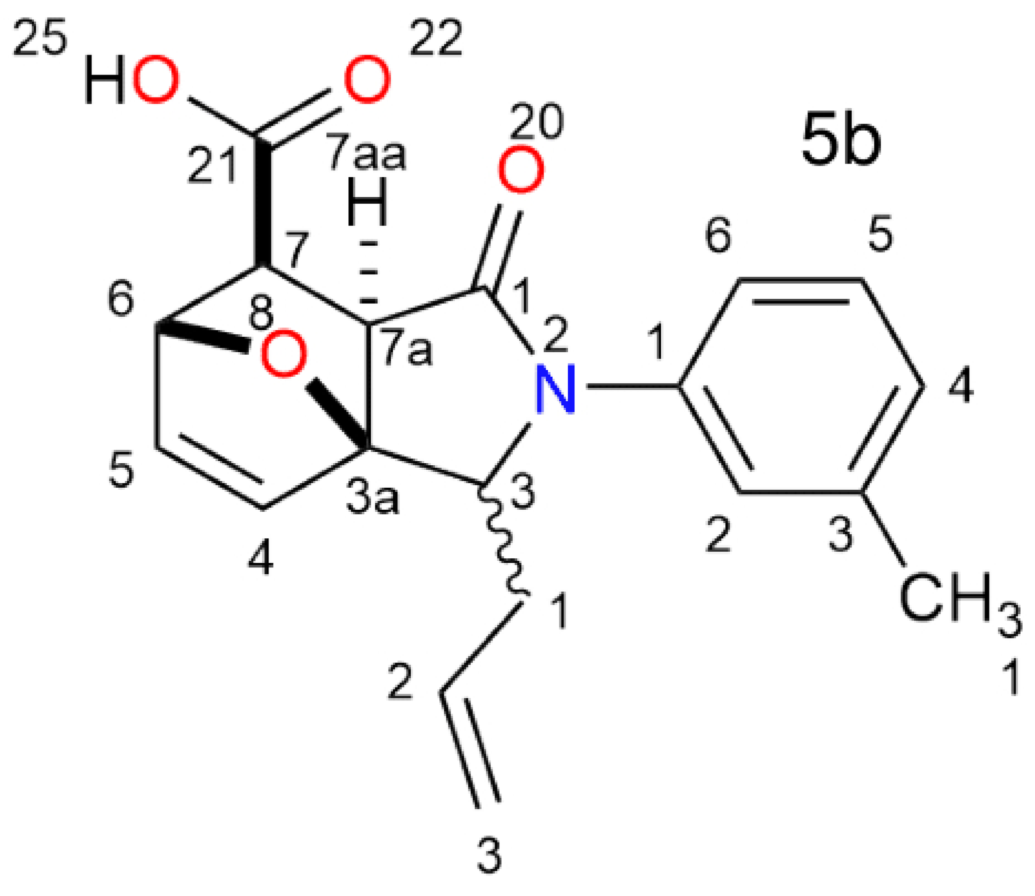

5b

FZ8941-1.JDF

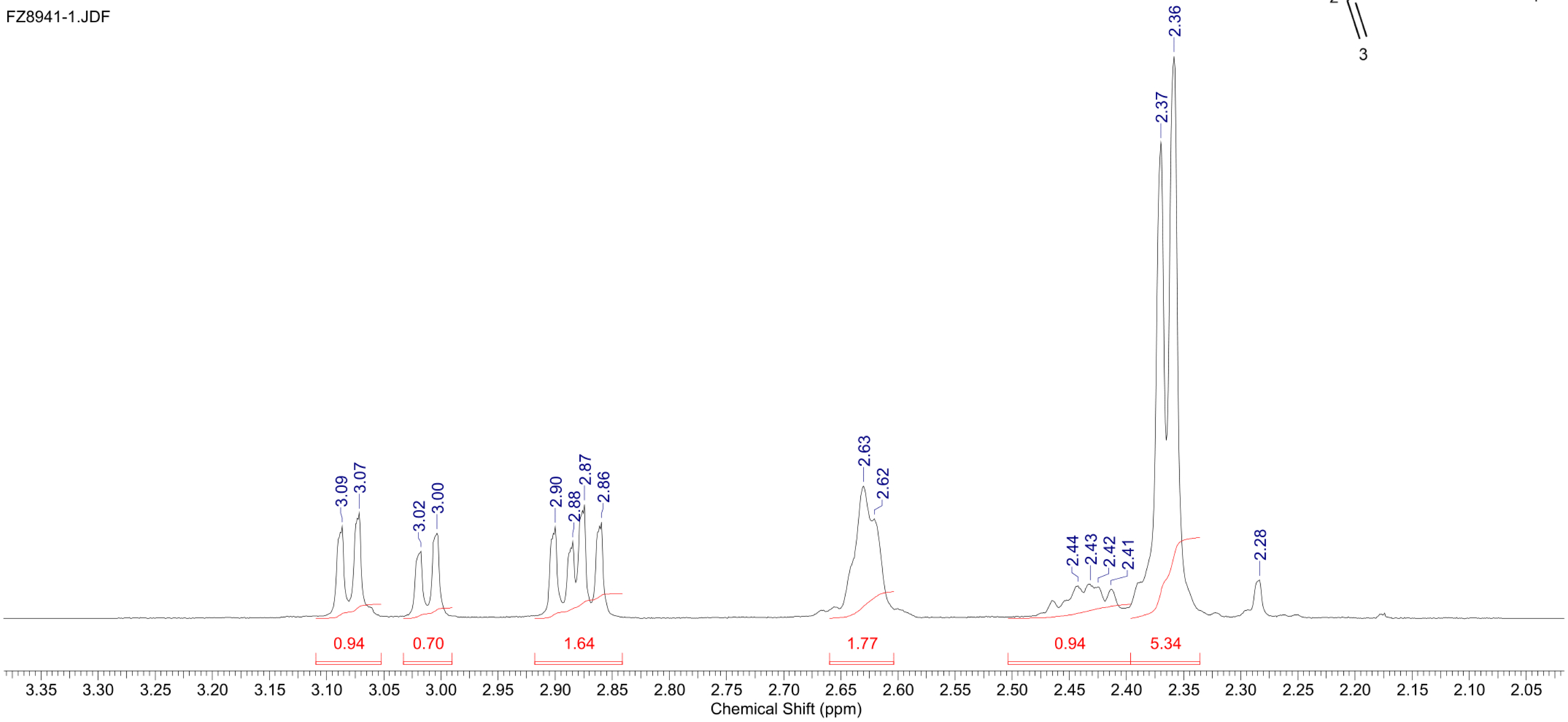

NMR spectrum data for compound 5b (PhH, Δ, 3 h)

Acquisition Time (sec)	1.9818	***Comment***	single_pulse	***Date***	05 Aug 2020 10:14:40			***Date Stamp***	05 Aug 2020 10:15:21
File Name	C:\USERS\FEDOR\DOWNLOADS\FZ8942-1.JDF			***Frequency (MHz)***	600.17	***Nucleus***	1H	***Number of Transients***	8
Origin	ECA 600	***Original Points Count***	32768	***Owner***	CKP	***Points Count***	32768	***Pulse Sequence***	single_pulse.ex2
Receiver Gain	42.00	***Solvent***	CHLOROFORM-d			***Spectrum Offset (Hz)***	5410.1226	***Sweep Width (Hz)***	16534.39

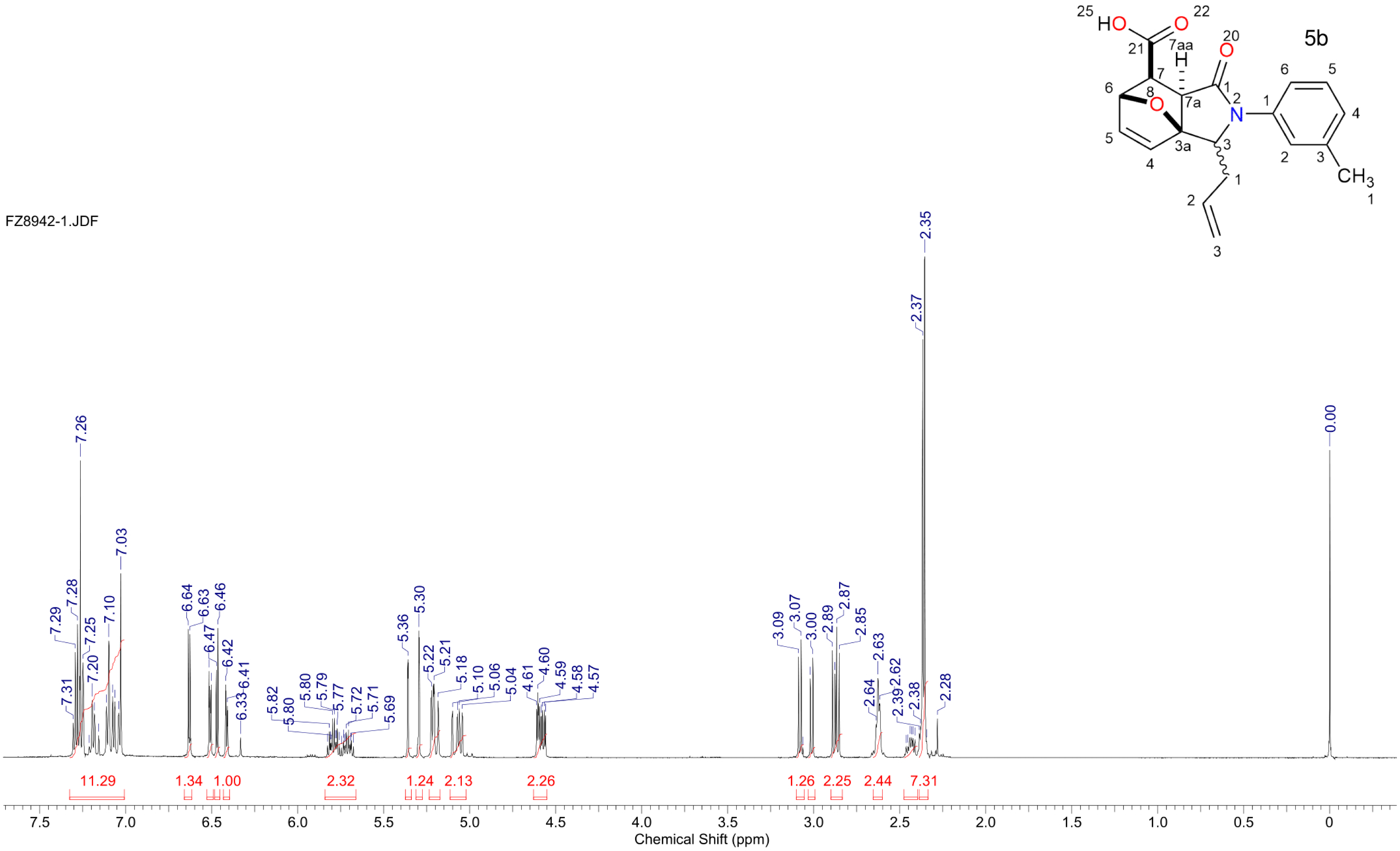

**Acquisition Time (sec)**	1.9818	**Comment**	single_pulse	**Date**	05 Aug 2020 10:14:40			**Date Stamp**	05 Aug 2020 10:15:21
**File Name**	C:\USERS\FEDOR\DOWNLOADS\FZ8942-1.JDF			**Frequency (MHz)**	600.17	**Nucleus**	1H	**Number of Transients**	8
**Origin**	ECA 600	**Original Points Count**	32768	**Owner**	CKP	**Points Count**	32768	**Pulse Sequence**	single_pulse.ex2
**Receiver Gain**	42.00	**Solvent**	CHLOROFORM-d			**Spectrum Offset (Hz)**	5410.1226	**Sweep Width (Hz)**	16534.39

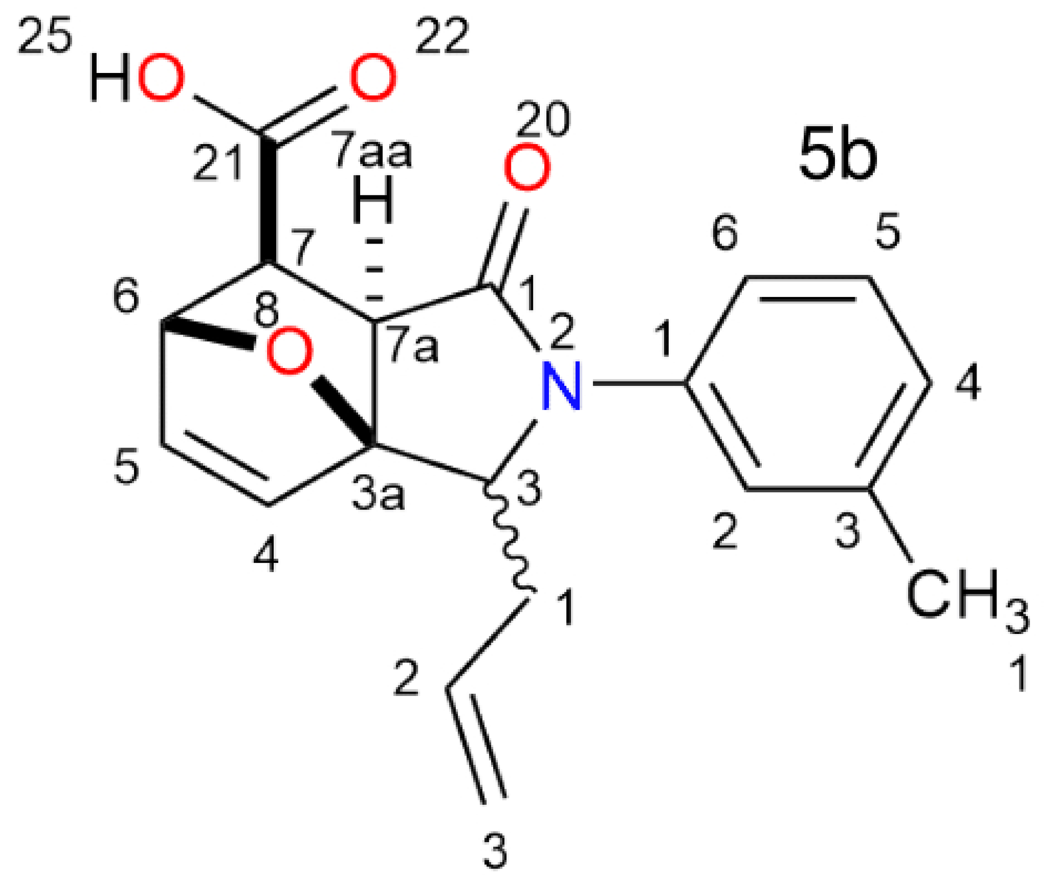

5b

FZ8942-1.JDF

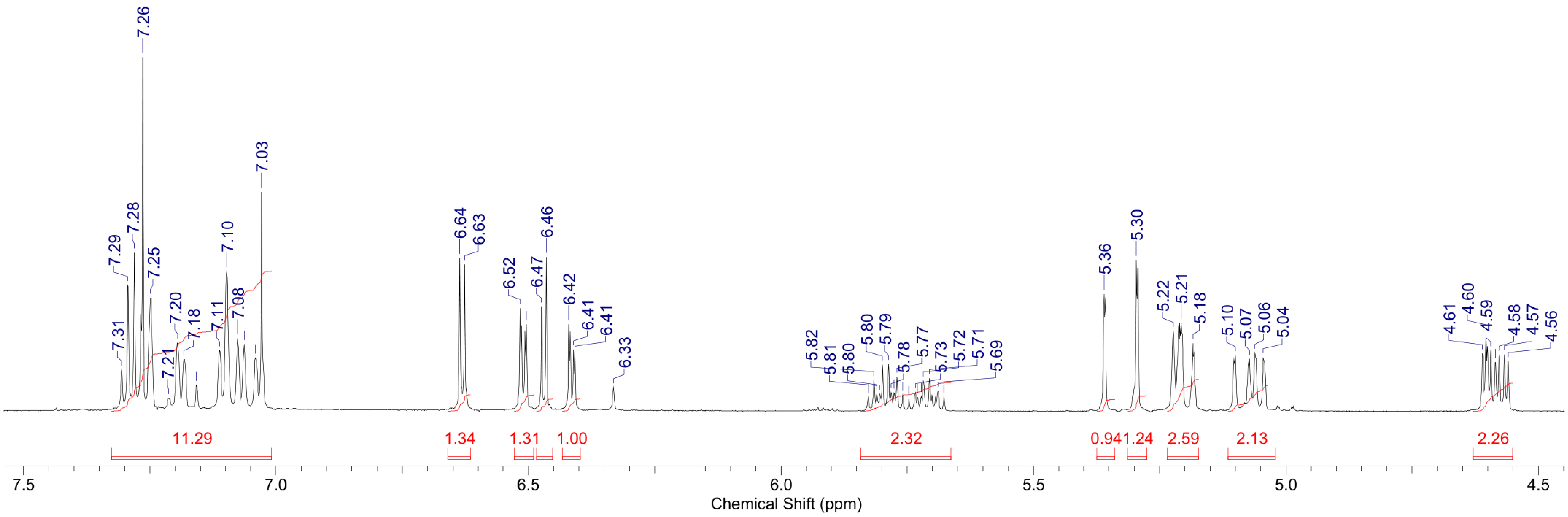

Acquisition Time (sec)	1.9818	Comment	single_pulse	Date	05 Aug 2020 10:14:40			Date Stamp	05 Aug 2020 10:15:21
File Name	C:\USERS\FEDOR\DOWNLOADS\FZ8942-1.JDF			Frequency (MHz)	600.17	Nucleus	1H	Number of Transients	8
Origin	ECA 600	Original Points Count	32768	Owner	CKP	Points Count	32768	Pulse Sequence	single_pulse.ex2
Receiver Gain	42.00	Solvent	CHLOROFORM-d			Spectrum Offset (Hz)	5410.1226	Sweep Width (Hz)	16534.39

5b

FZ8942-1.JDF

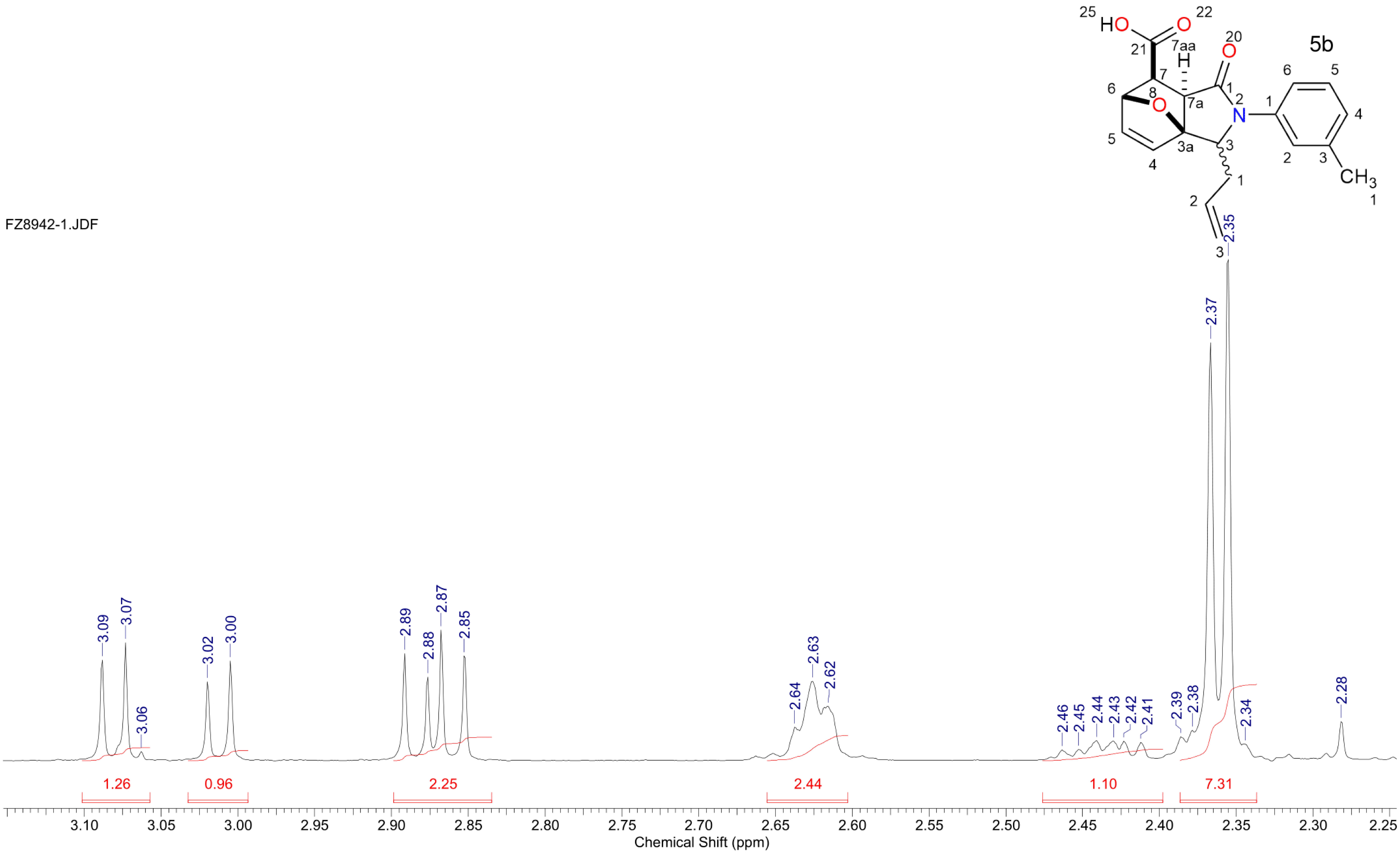

NMR spectrum data for compound 5b (PhMe, r.t, 3 d)

Formula	$C_{20}H_{21}NO_4$	FW	339.3850

**Acquisition Time (sec)**	1.9818	**Comment**	single_pulse	**Date**	28 Jul 2020 10:53:42			**Date Stamp**	28 Jul 2020 10:54:55
**File Name**	H:\DOWNLOADS\FZ8876-1.JDF			**Frequency (MHz)**	600.17	**Nucleus**	1H	**Number of Transients**	8
**Origin**	ECA 600	**Original Points Count**	32768	**Owner**	CKP	**Points Count**	32768	**Pulse Sequence**	single_pulse.ex2
**Receiver Gain**	42.00	**Solvent**	CHLOROFORM-d			**Spectrum Offset (Hz)**	5411.1318	**Sweep Width (Hz)**	16534.39

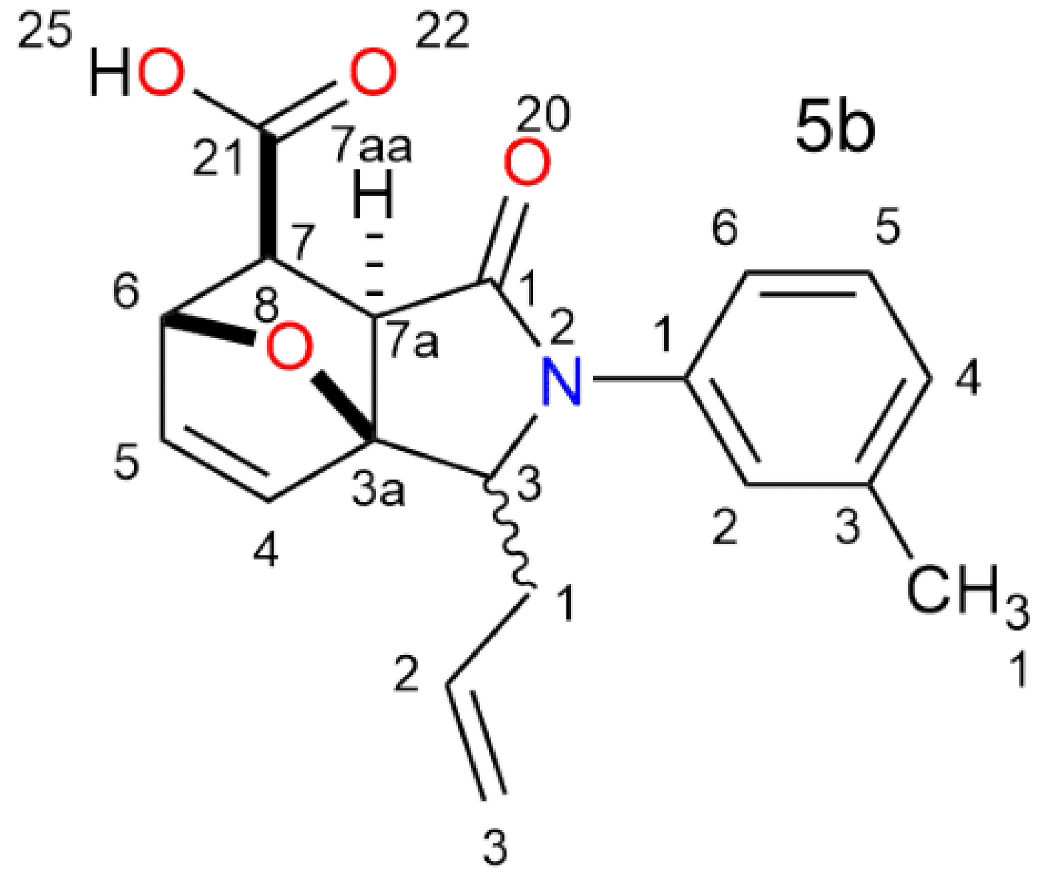

FZ8876-1.JDF

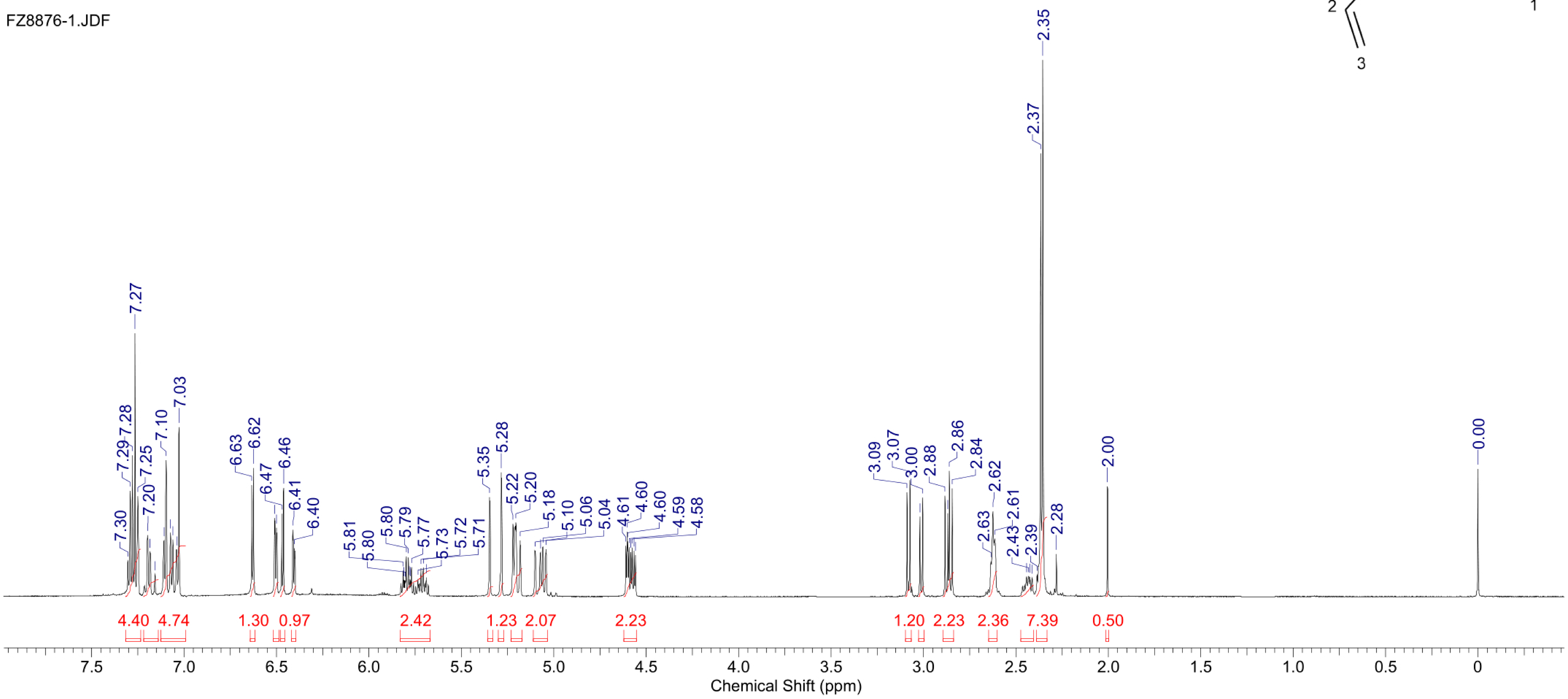

**Formula** $C_{20}H_{21}NO_4$	**FW** 339.3850

**Acquisition Time (sec)**	1.9818	**Comment**	single_pulse	**Date**	28 Jul 2020 10:53:42	**Date Stamp**	28 Jul 2020 10:54:55
**File Name**	H:\DOWNLOADS\FZ8876-1.JDF	**Frequency (MHz)**	600.17	**Nucleus**	1H	**Number of Transients**	8
**Origin**	ECA 600	**Original Points Count**	32768	**Owner**	CKP	**Points Count**	32768
**Pulse Sequence**	single_pulse.ex2	**Receiver Gain**	42.00	**Solvent**	CHLOROFORM-d	**Spectrum Offset (Hz)**	5411.1318
**Sweep Width (Hz)**	16534.39						

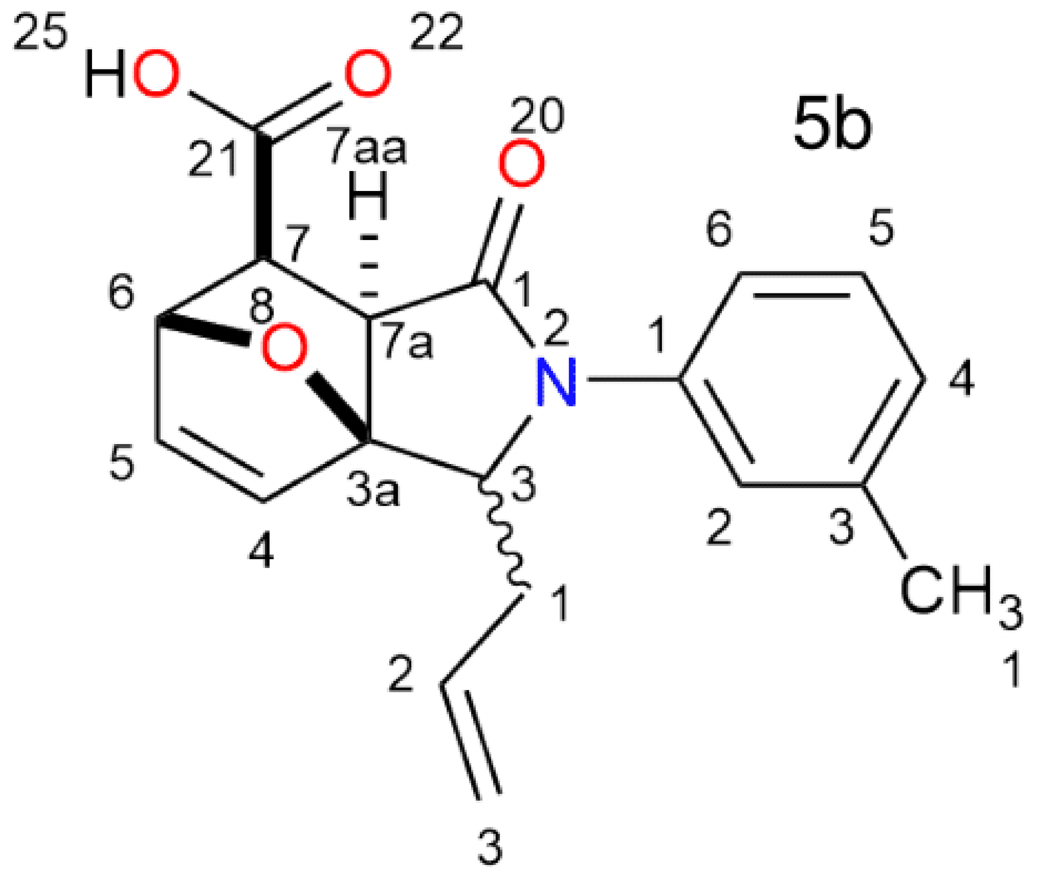

FZ8876-1.JDF

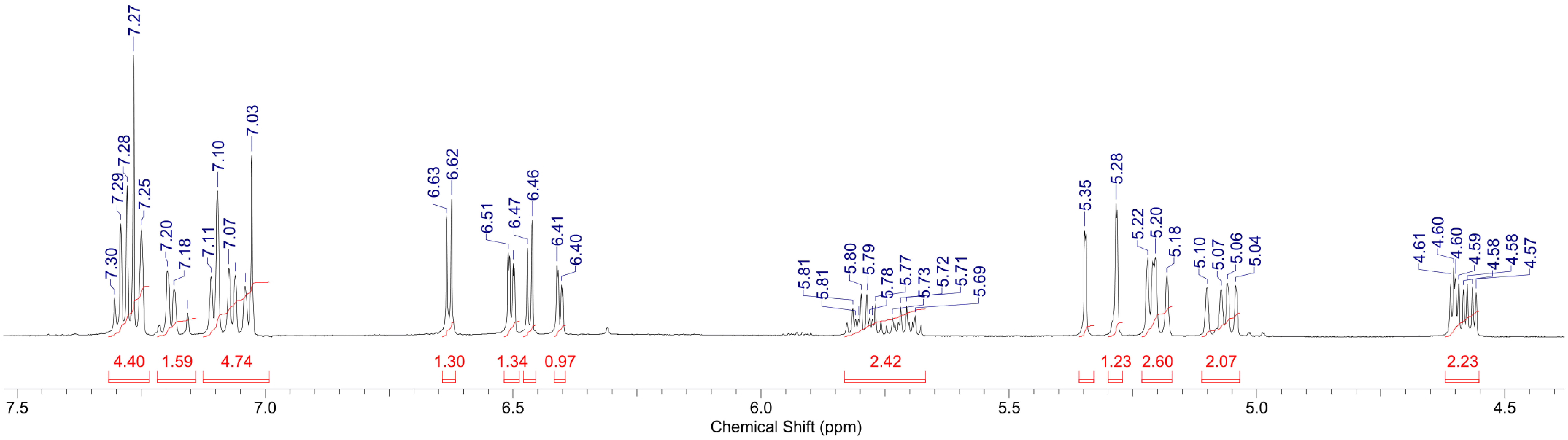

Formula	$C_{20}H_{21}NO_4$	FW	339.3850

**Acquisition Time (sec)**	1.9818	**Comment**	single_pulse	**Date**	28 Jul 2020 10:53:42			**Date Stamp**	28 Jul 2020 10:54:55
**File Name**	H:\DOWNLOADS\FZ8876-1.JDF			**Frequency (MHz)**	600.17	**Nucleus**	1H	**Number of Transients**	8
**Origin**	ECA 600	**Original Points Count**	32768	**Owner**	CKP	**Points Count**	32768	**Pulse Sequence**	single_pulse.ex2
**Receiver Gain**	42.00	**Solvent**	CHLOROFORM-d			**Spectrum Offset (Hz)**	5411.1318	**Sweep Width (Hz)**	16534.39

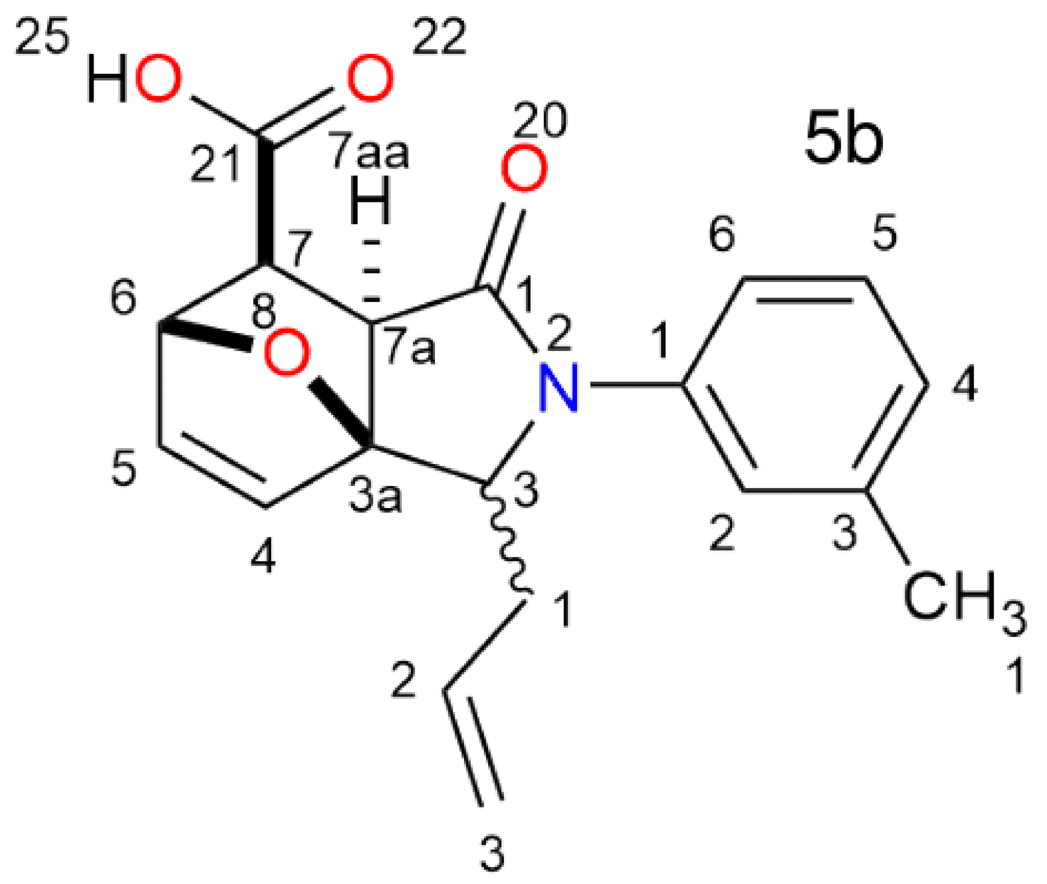

5b

FZ8876-1.JDF

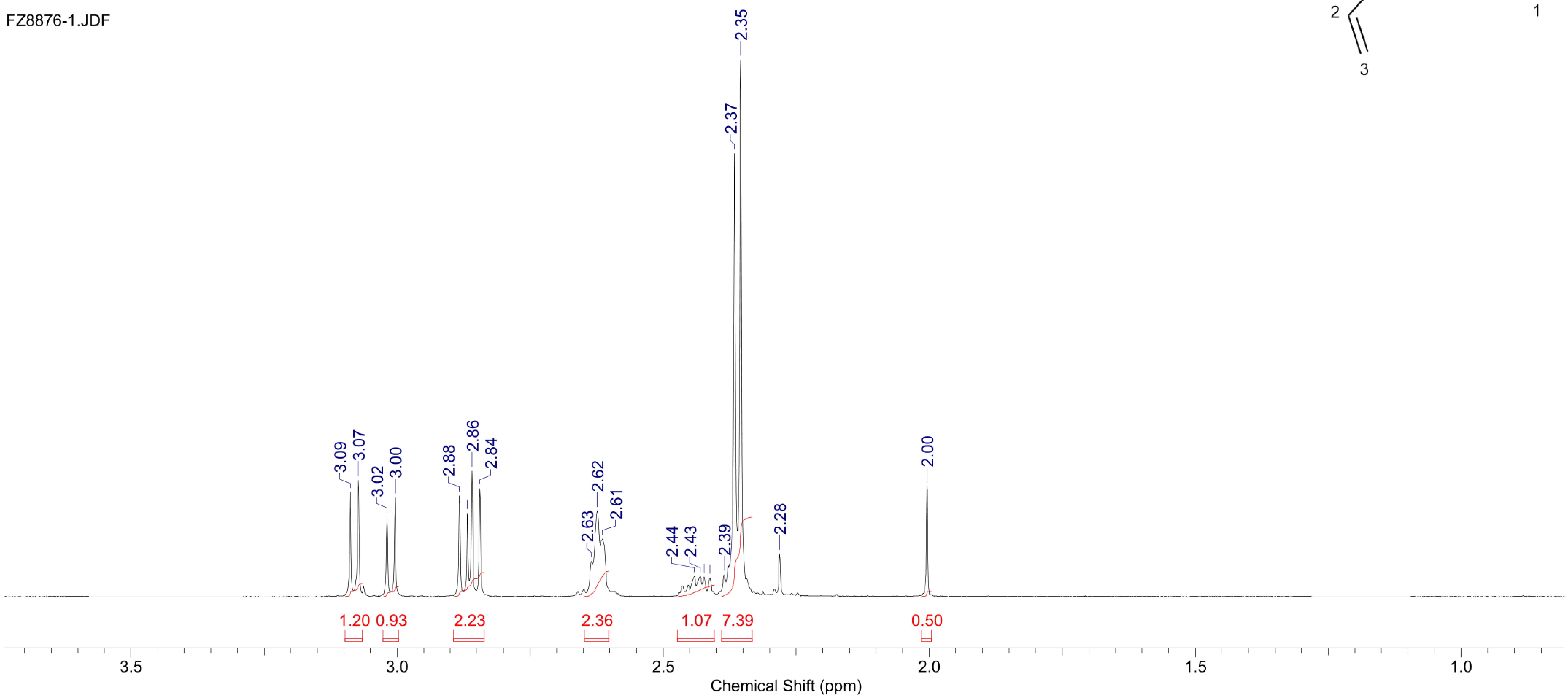

NMR spectrum data for compound 5b (PhMe, Δ, 3 h)

Acquisition Time (sec)	1.9818	***Comment***	single_pulse	***Date***	05 Aug 2020 10:49:30	***Date Stamp***	05 Aug 2020 10:50:11
File Name	C:\USERS\FEDOR\DOWNLOADS\FZ8939-1.JDF	***Frequency (MHz)***	600.17	***Nucleus***	1H	***Number of Transients***	8
Origin	ECA 600	***Original Points Count***	32768	***Owner***	CKP	***Points Count***	32768
Pulse Sequence	single_pulse.ex2	***Receiver Gain***	42.00	***Solvent***	CHLOROFORM-d	***Spectrum Offset (Hz)***	5410.6274
Sweep Width (Hz)	16534.39						

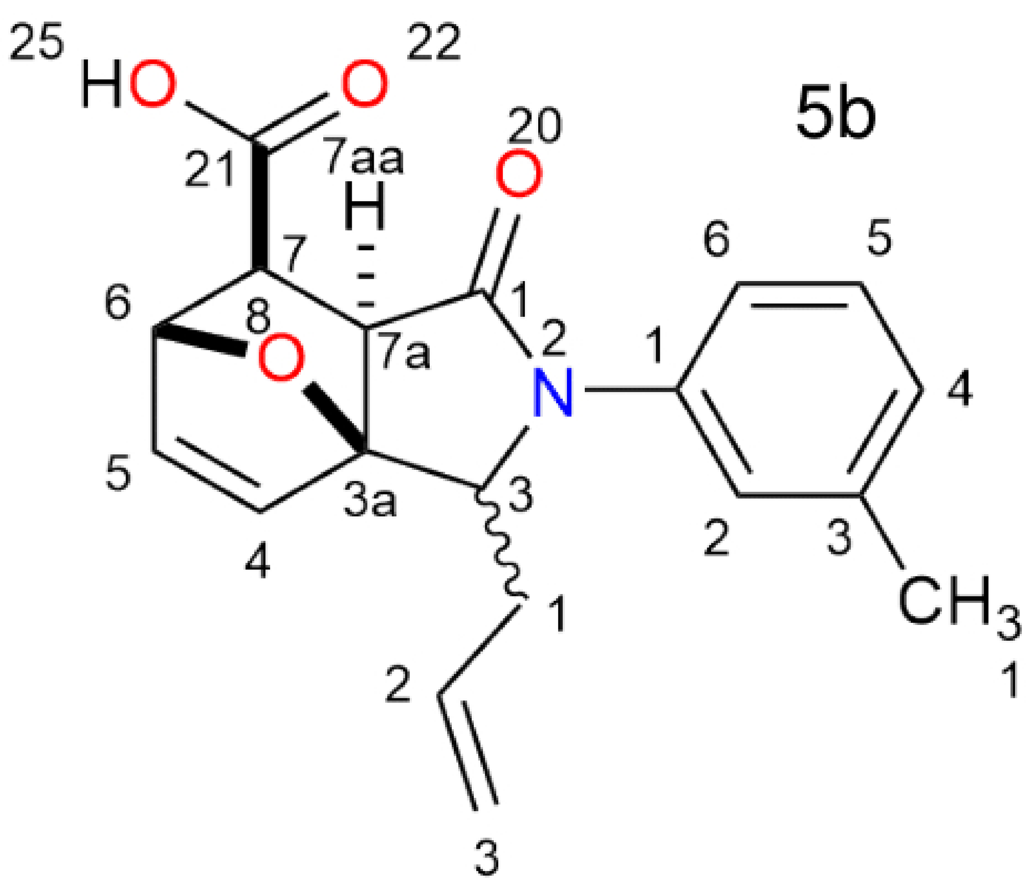

5b

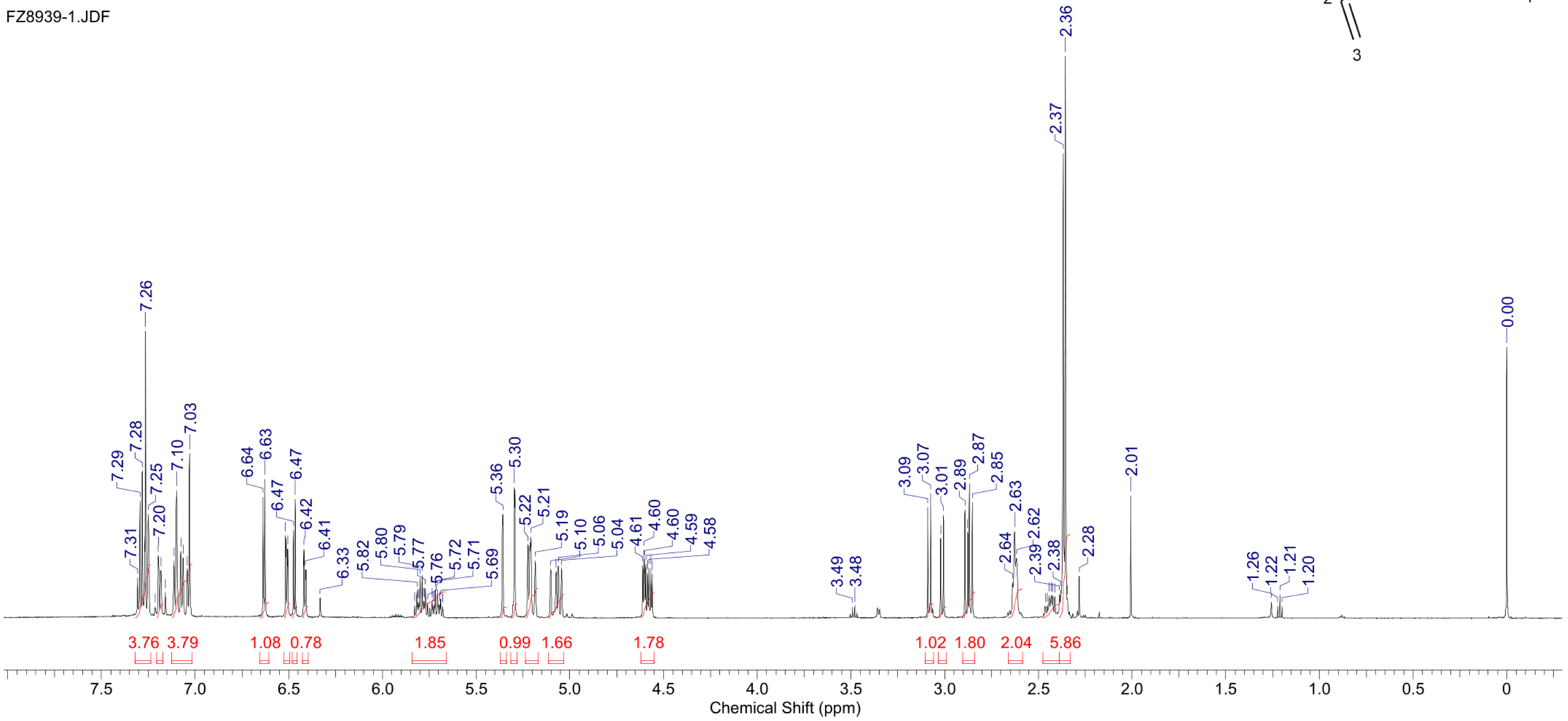

Acquisition Time (sec)	1.9818	***Comment***	single_pulse	***Date***	05 Aug 2020 10:49:30	***Date Stamp***	05 Aug 2020 10:50:11
File Name	C:\USERS\FEDOR\DOWNLOADS\FZ8939-1.JDF	***Frequency (MHz)***	600.17	***Nucleus***	1H	***Number of Transients***	8
Origin	ECA 600	***Original Points Count***	32768	***Owner***	CKP	***Points Count***	32768
Pulse Sequence	single_pulse.ex2	***Receiver Gain***	42.00	***Solvent***	CHLOROFORM-d	***Spectrum Offset (Hz)***	5410.6274
Sweep Width (Hz)	16534.39						

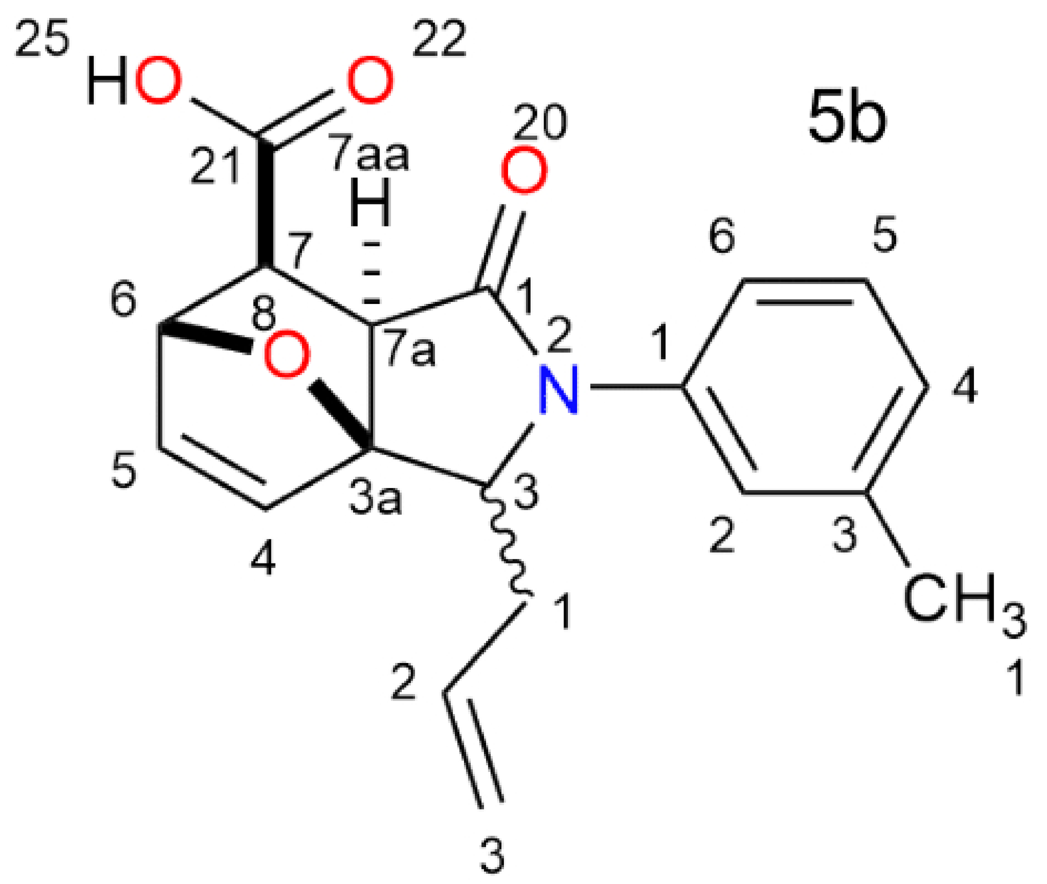

5b

FZ8939-1.JDF

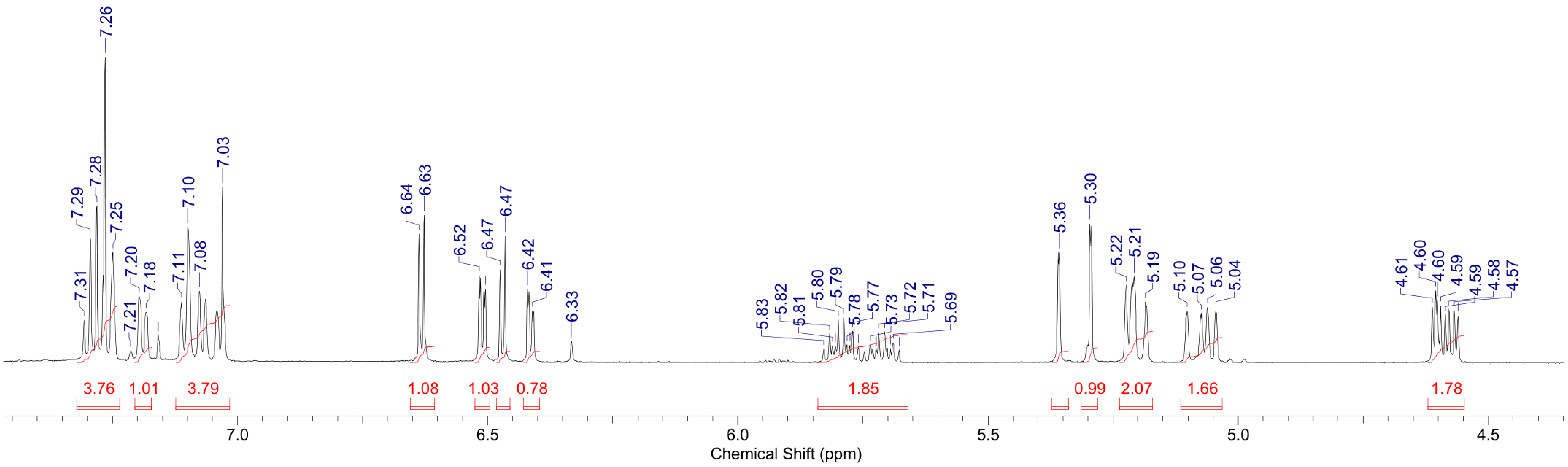

Acquisition Time (sec)	1.9818	***Comment***	single_pulse	***Date***	05 Aug 2020 10:49:30			***Date Stamp***	05 Aug 2020 10:50:11
File Name	C:\USERS\FEDOR\DOWNLOADS\FZ8939-1.JDF			***Frequency (MHz)***	600.17	***Nucleus***	1H	***Number of Transients***	8
Origin	ECA 600	***Original Points Count***	32768	***Owner***	CKP	***Points Count***	32768	***Pulse Sequence***	single_pulse.ex2
Receiver Gain	42.00	***Solvent***	CHLOROFORM-d			***Spectrum Offset (Hz)***	5410.6274	***Sweep Width (Hz)***	16534.39

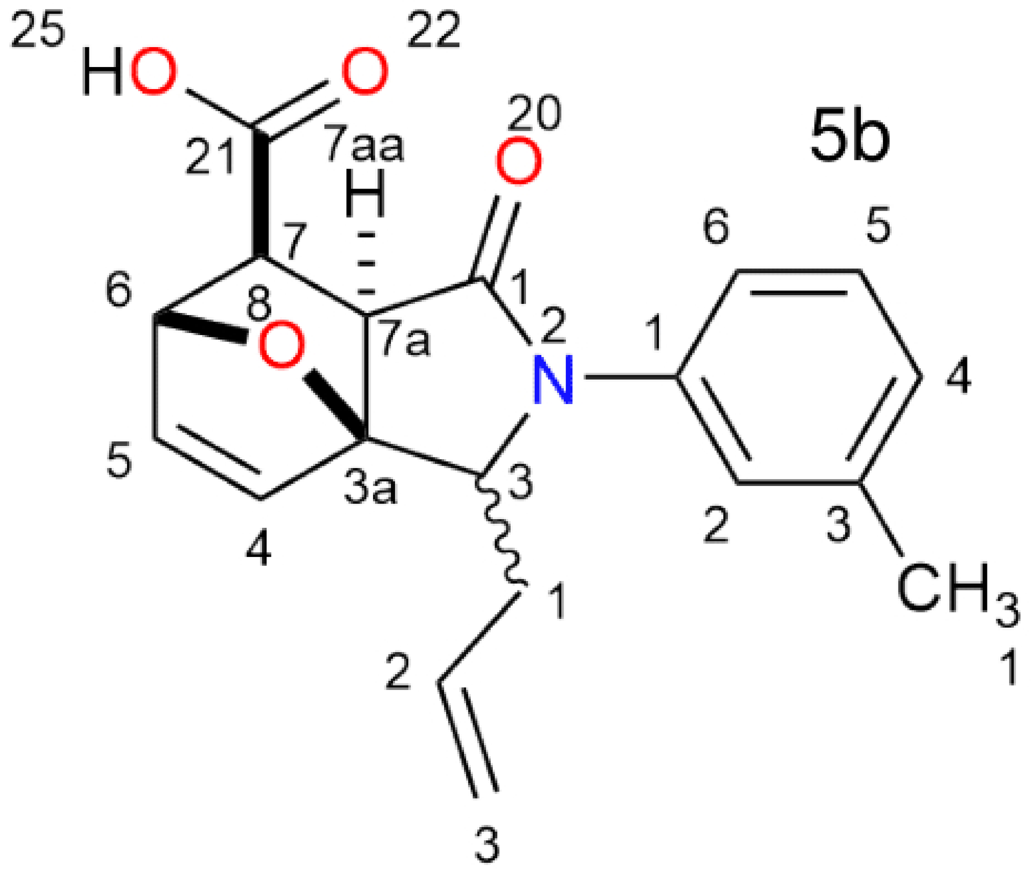

5b

FZ8939-1.JDF

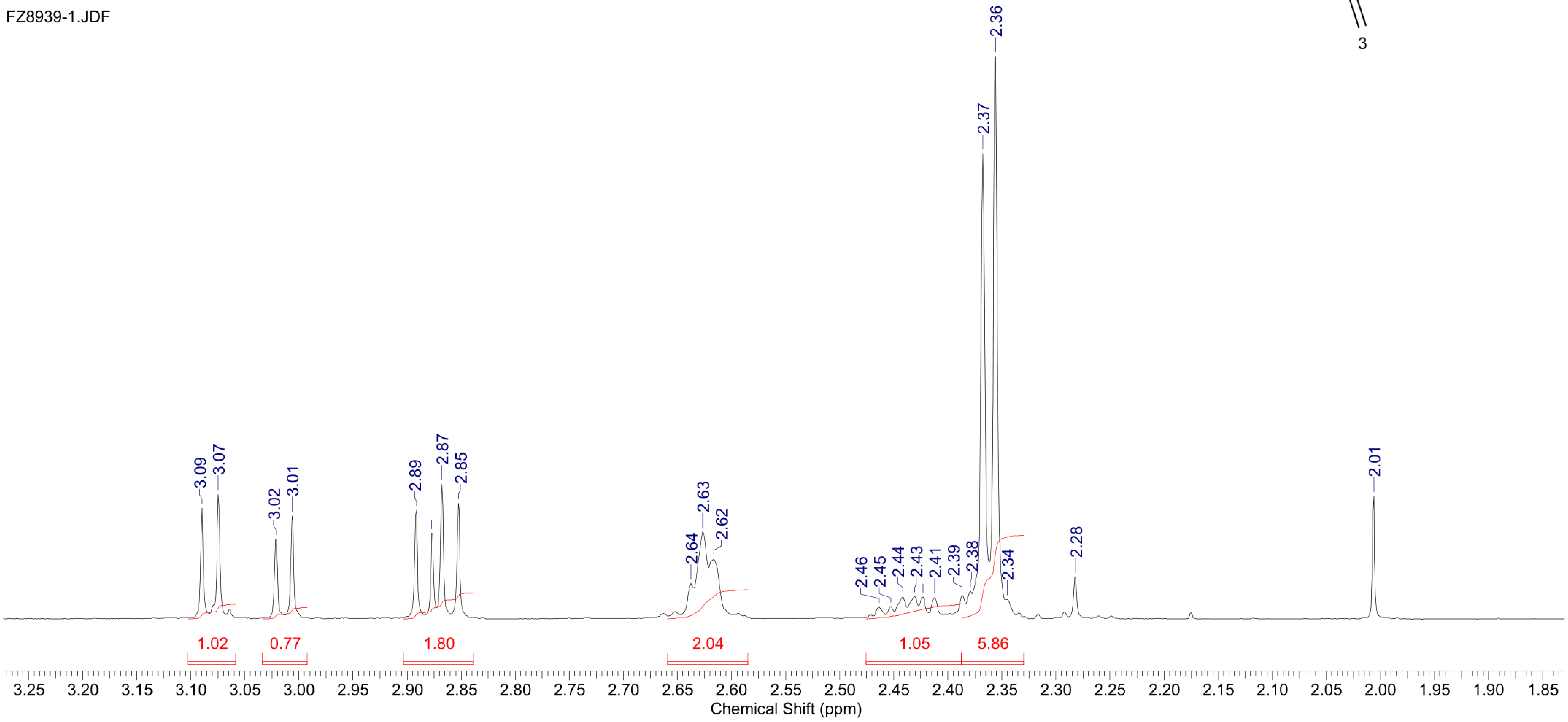

NMR spectrum data for compound 2b

Formula	$C_{19}H_{19}NO_4$	FW	325.3585

Acquisition Time (sec)	0.6921	***Comment***	single pulse decoupled gated NOE	***Date***	28 Aug 1990 07:25:11
Date Stamp	07 Nov 2019 12:42:13	***File Name***	C:\Users\User\Desktop\05.11.19\FZ8095-2.jdf		
Frequency (MHz)	150.91	***Nucleus***	13C	***Number of Transients***	1000
Origin	ECA 600	***Original Points Count***	32768	***Owner***	delta
Points Count	32768	***Pulse Sequence***	single_pulse_dec	***Receiver Gain***	56.00
Solvent	CHLOROFORM-d	***Spectrum Offset (Hz)***	15079.3525	***Sweep Width (Hz)***	47348.49
Temperature (degree C)	23.700				

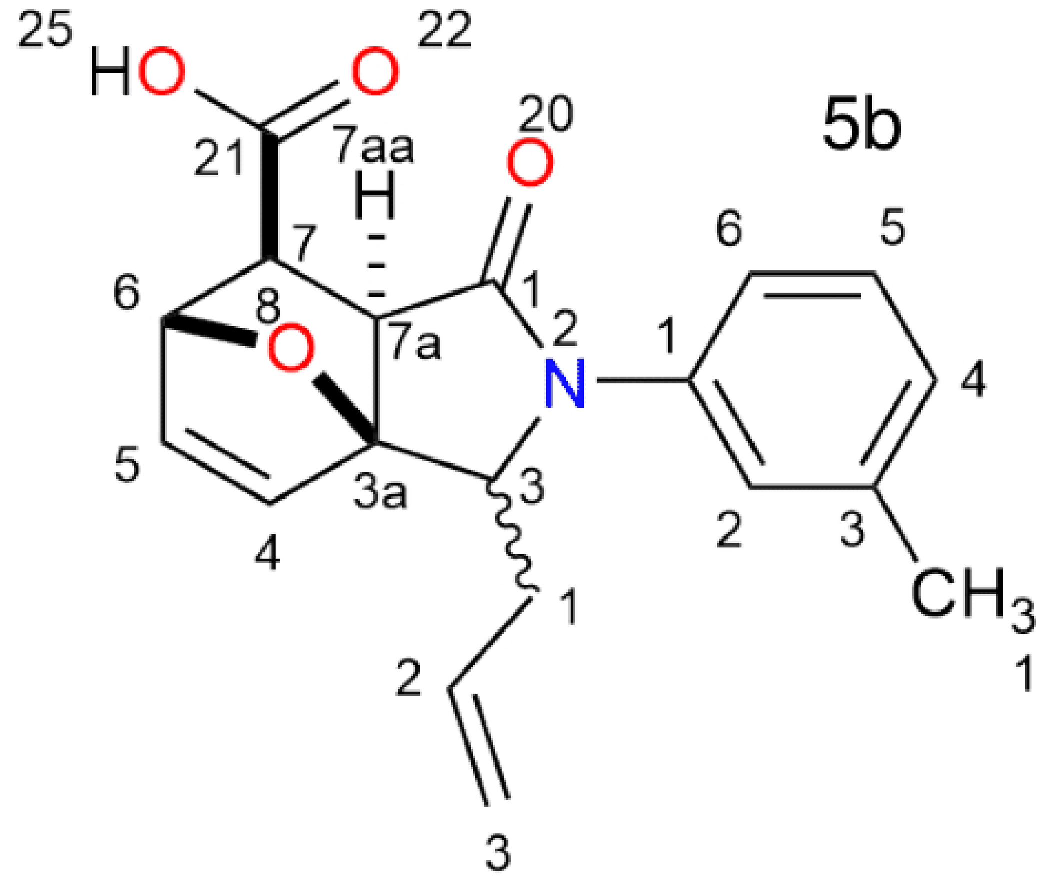

FZ8095-2.jdf

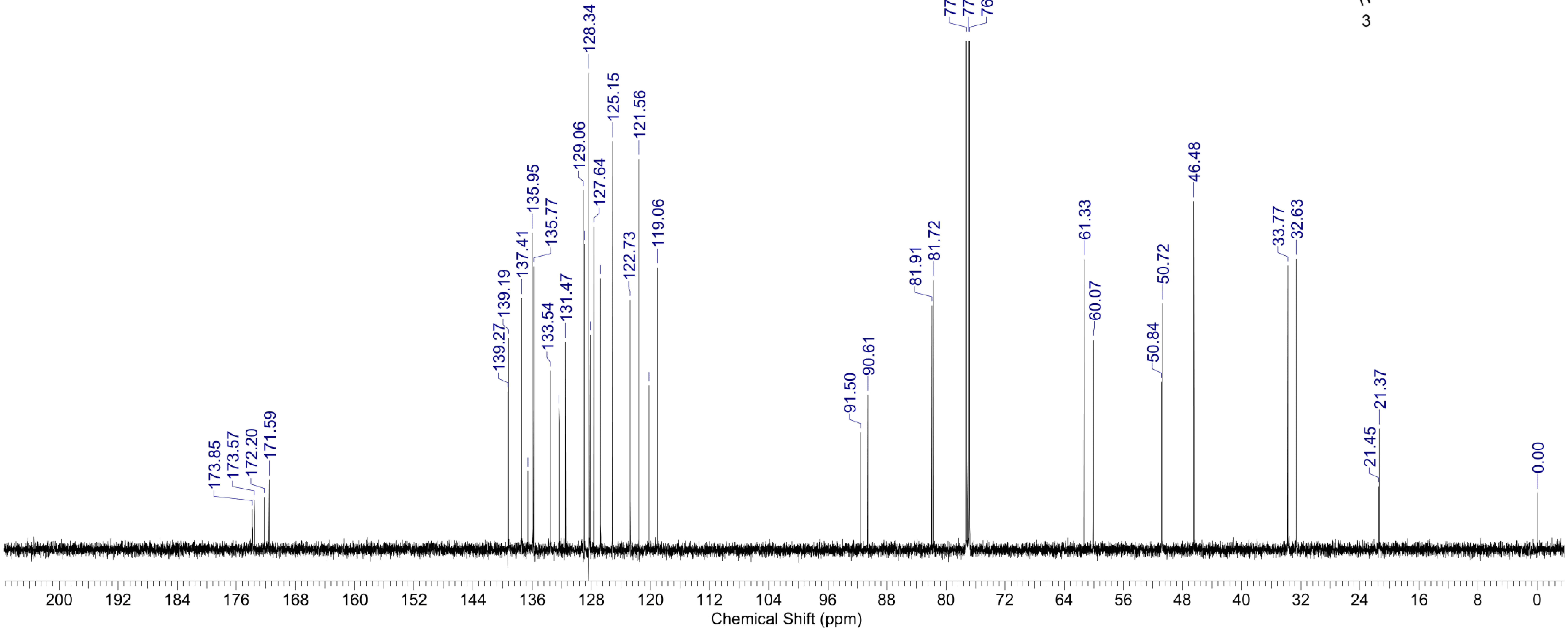

Formula $C_{19}H_{19}NO_4$	FW 325.3585

Acquisition Time (sec)	0.6921	Comment	single pulse decoupled gated NOE			Date	28 Aug 1990 07:25:11
Date Stamp	07 Nov 2019 12:42:13			File Name	C:\Users\User\Desktop\05.11.19\FZ8095-2.jdf		
Frequency (MHz)	150.91	Nucleus	13C	Number of Transients	1000	Origin	ECA 600
Original Points Count	32768	Owner	delta	Points Count	32768	Pulse Sequence	single_pulse_dec
Receiver Gain	56.00	Solvent	CHLOROFORM-d			Spectrum Offset (Hz)	15079.3525
Sweep Width (Hz)	47348.49	Temperature (degree C)	23.700				

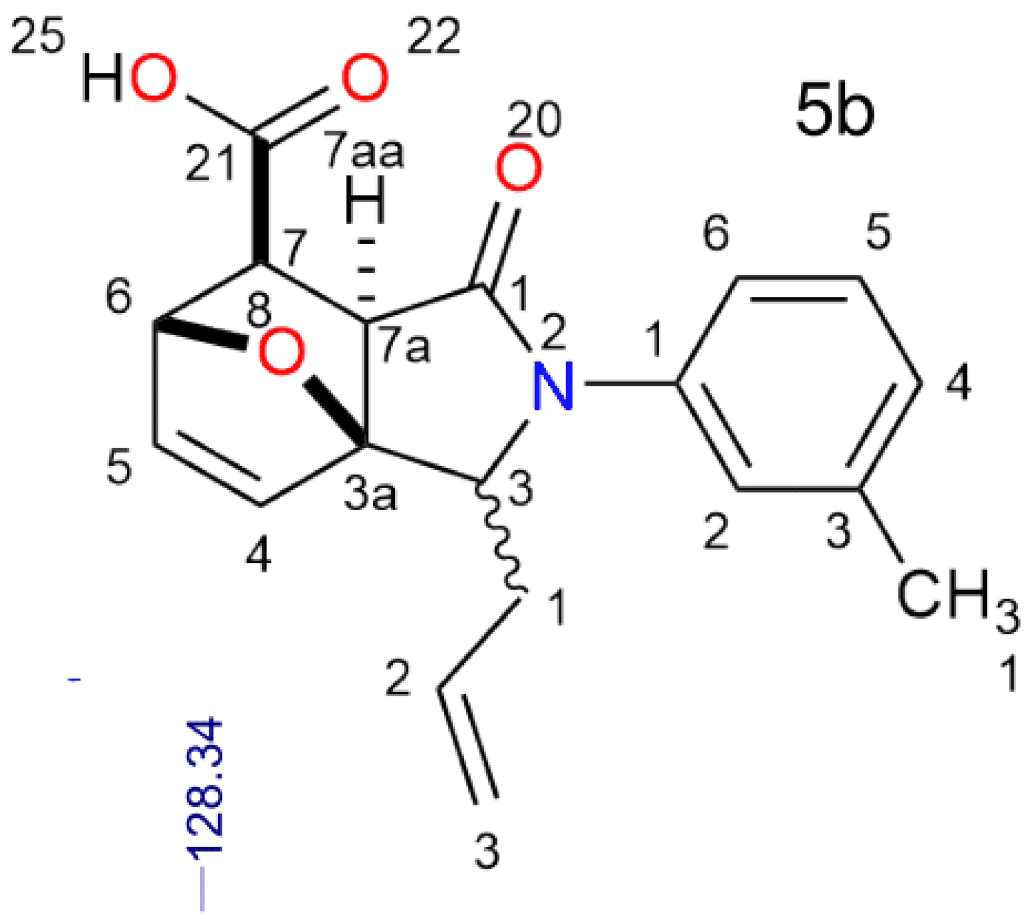

5b

FZ8095-2.jdf

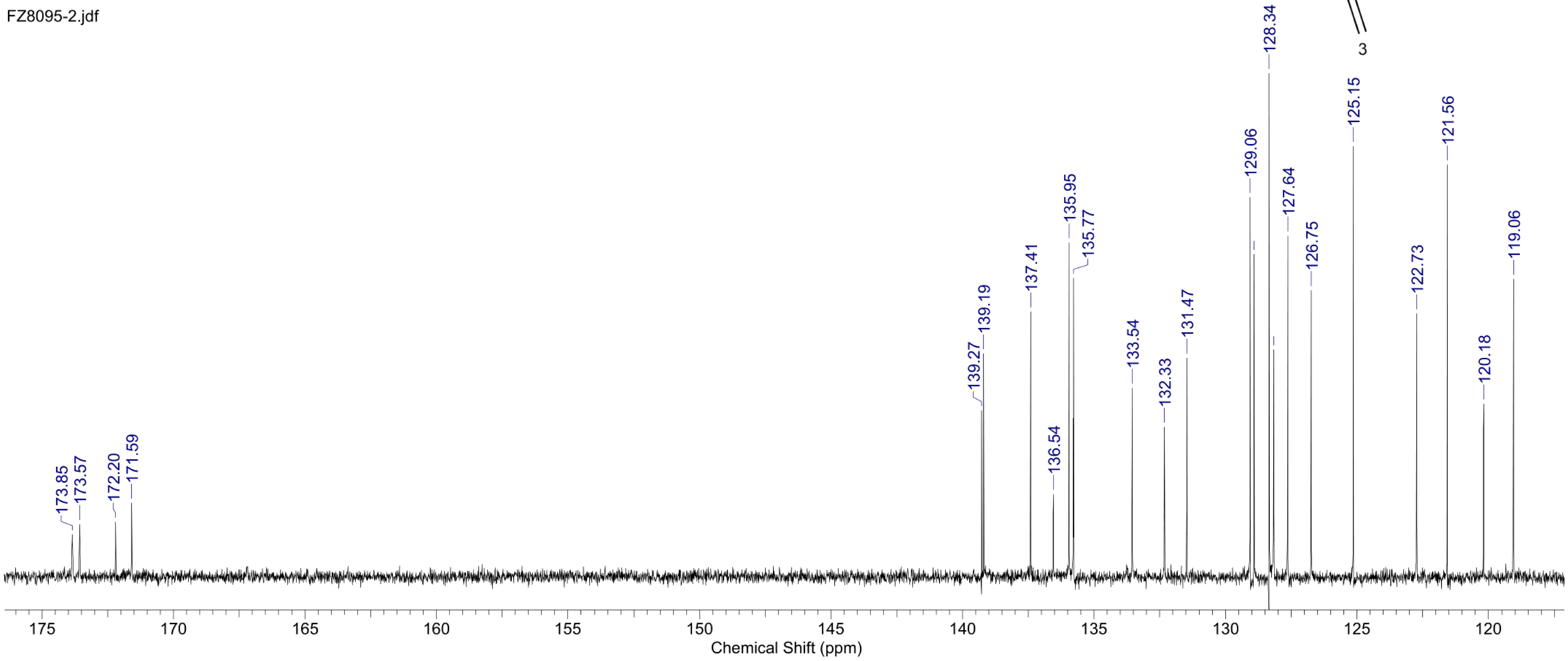

Formula	$C_{19}H_{19}NO_4$	***FW***	325.3585

Acquisition Time (sec)	0.6921	***Comment***	single pulse decoupled gated NOE	***Date***	28 Aug 1990 07:25:11
Date Stamp	07 Nov 2019 12:42:13	***File Name***	C:\Users\User\Desktop\05.11.19\FZ8095-2.jdf		
Frequency (MHz)	150.91	***Nucleus***	13C	***Number of Transients***	1000
Origin	ECA 600	***Original Points Count***	32768	***Owner***	delta
Points Count	32768	***Pulse Sequence***	single_pulse_dec	***Receiver Gain***	56.00
Solvent	CHLOROFORM-d	***Spectrum Offset (Hz)***	15079.3525	***Sweep Width (Hz)***	47348.49
Temperature (degree C)	23.700				

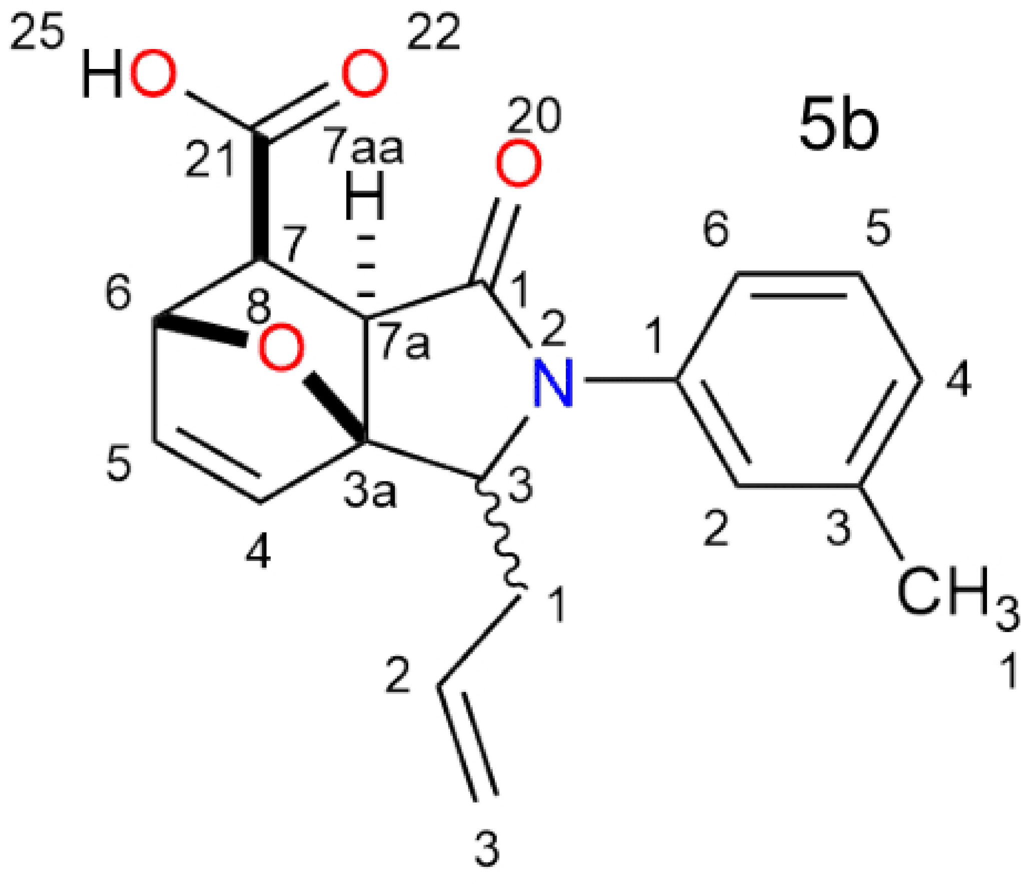

5b

FZ8095-2.jdf

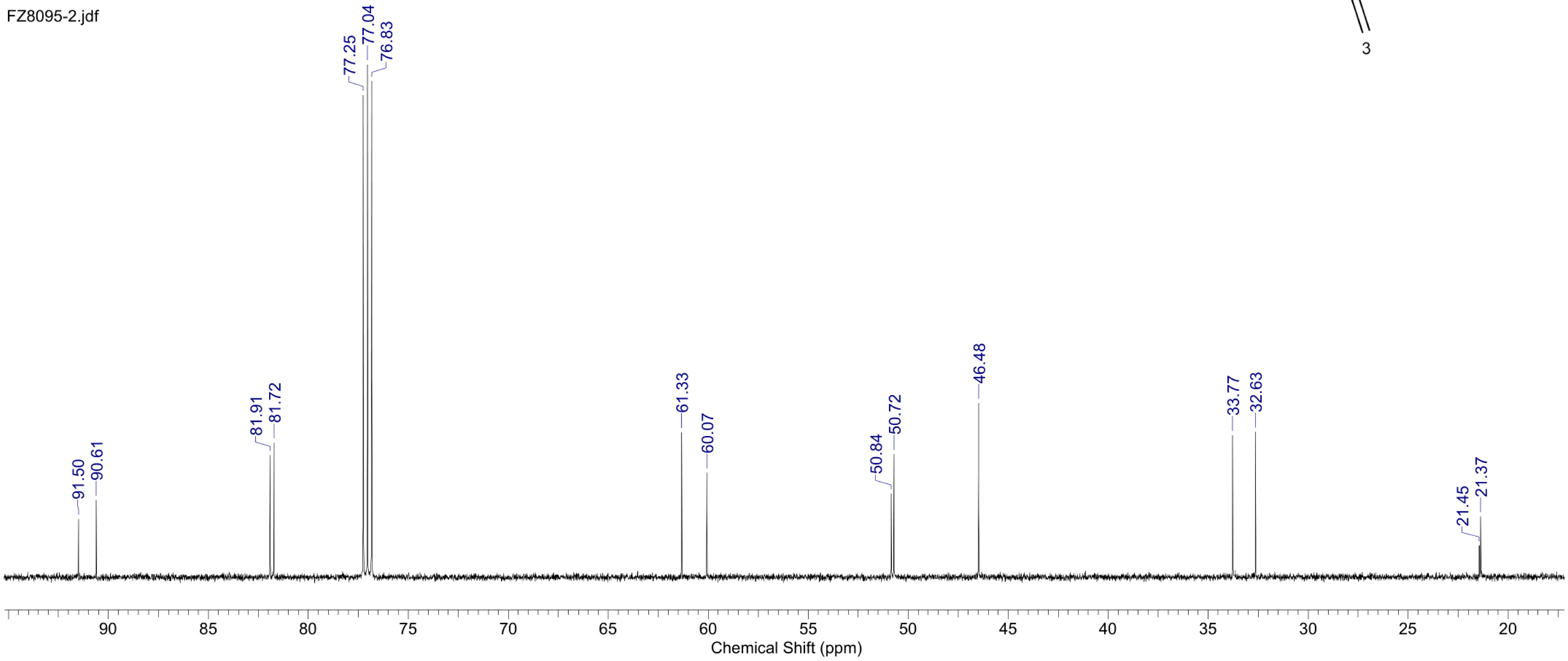

20.07.2020 18:18:07

NMR spectrum data for compound 5g (DCM, −16 °C, 3 d)

**Formula** $C_{18}H_{16}INO_4$	**FW** 437.2284

**Acquisition Time (sec)**	1.9818	**Comment**	single_pulse	**Date**	29 Jun 2020 09:18:39		
**Date Stamp**	29 Jun 2020 09:20:08			**File Name**	C:\USERS\Лаб534\DOWNLOADS\FZ8712-1.JDF	**Frequency (MHz)**	600.17
**Nucleus**	1H	**Number of Transients**	8	**Origin**	ECA 600	**Original Points Count**	32768
**Owner**	CKP						
**Points Count**	32768	**Pulse Sequence**	single_pulse.ex2	**Receiver Gain**	44.00	**Solvent**	CHLOROFORM-d
**Spectrum Offset (Hz)**	5409.1133	**Sweep Width (Hz)**	16534.39	**Temperature (degree C)**	22.100		

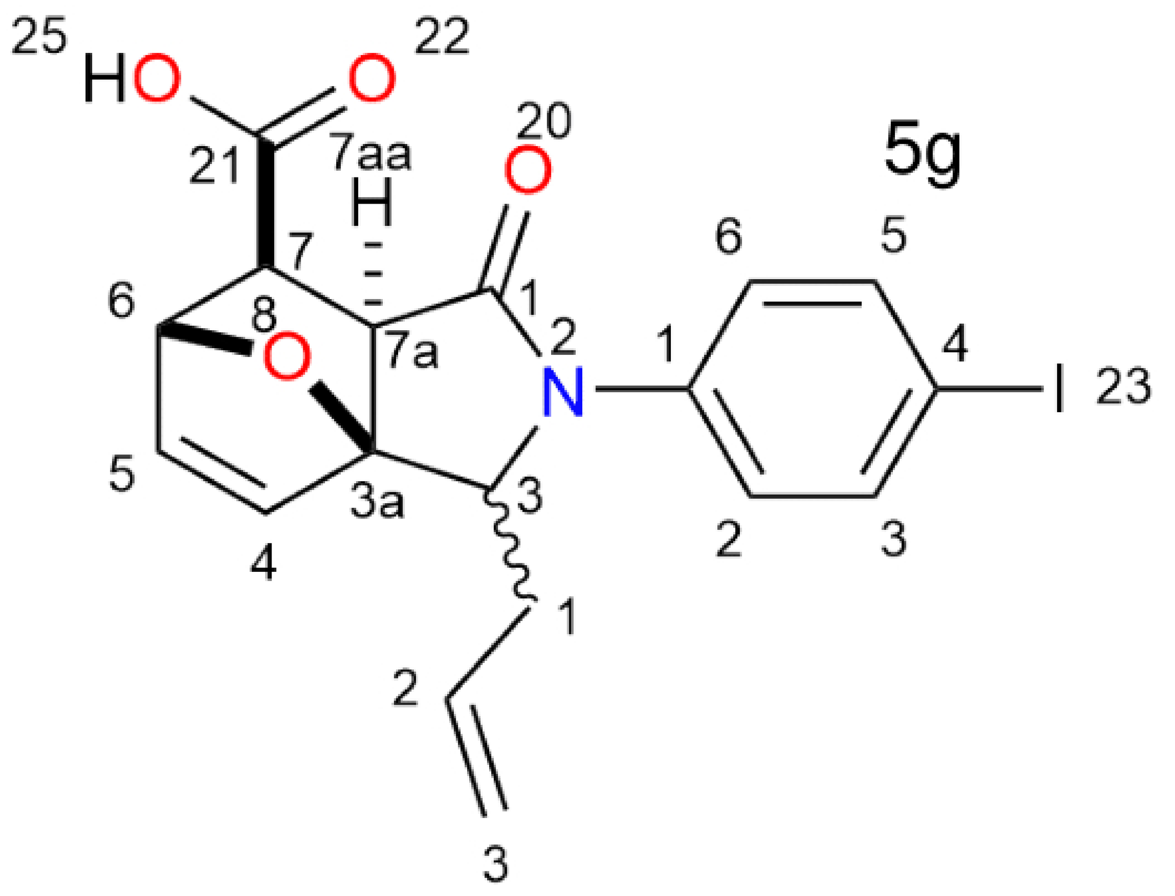

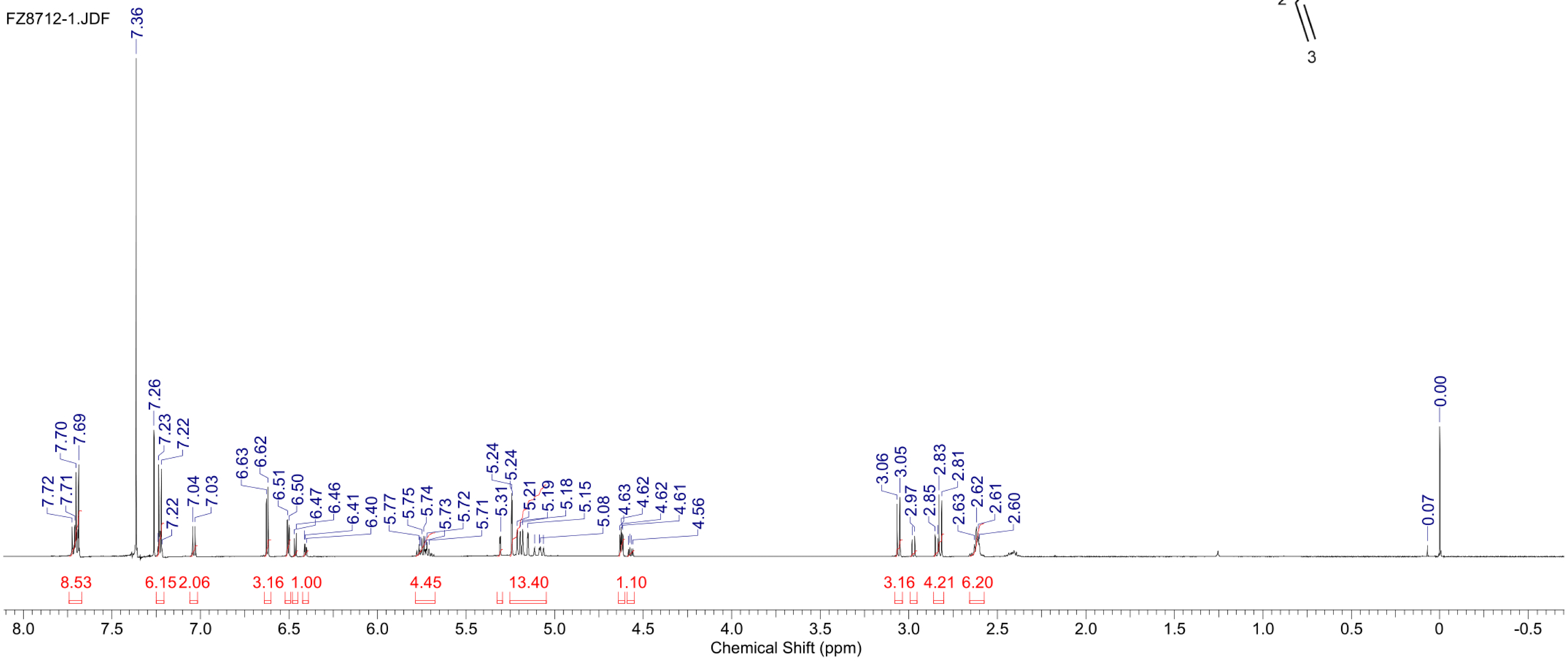

20.07.2020 18:18:19

Formula $C_{18}H_{16}INO_4$	FW 437.2284

Acquisition Time (sec)	1.9818	Comment	single_pulse	Date	29 Jun 2020 09:18:39		
Date Stamp	29 Jun 2020 09:20:08			File Name	C:\USERS\Лаба534\DOWNLOADS\FZ8712-1.JDF	Frequency (MHz)	600.17
Nucleus	1H	Number of Transients	8	Origin	ECA 600	Original Points Count 32768	Owner CKP
Points Count	32768	Pulse Sequence	single_pulse.ex2			Receiver Gain 44.00	Solvent CHLOROFORM-d
Spectrum Offset (Hz)	5409.1133	Sweep Width (Hz)	16534.39	Temperature (degree C)	22.100		

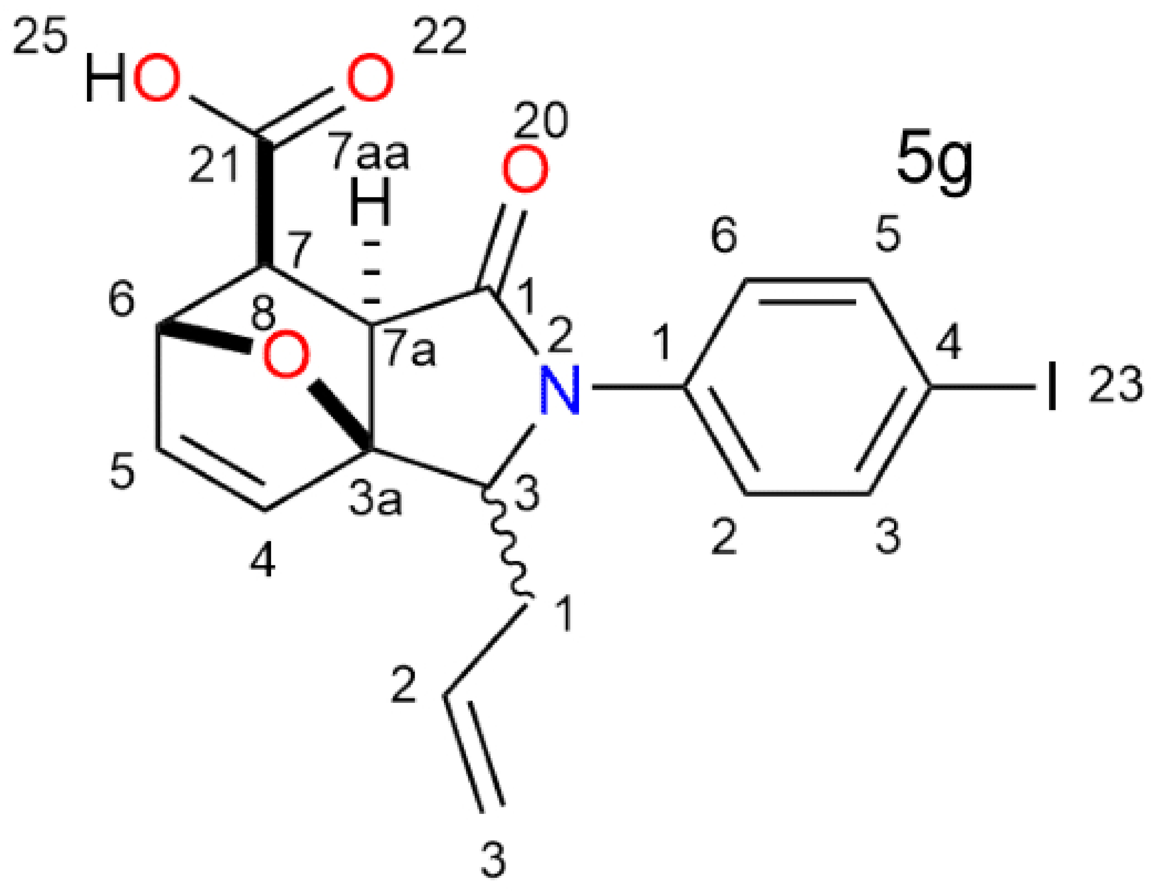

5g

FZ8712-1.JDF

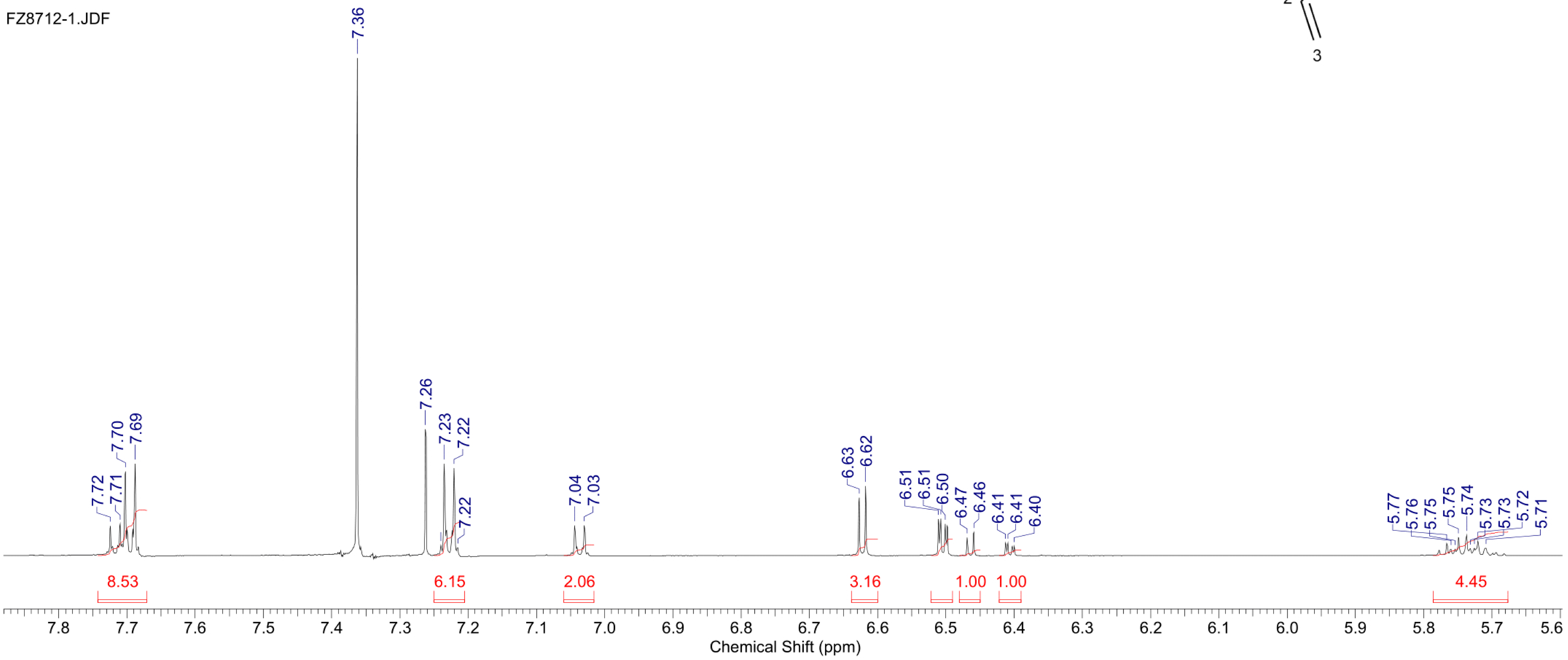

20.07.2020 18:18:29

Formula $C_{18}H_{16}INO_4$	***FW*** 437.2284

Acquisition Time (sec)	1.9818	***Comment***	single_pulse	***Date***	29 Jun 2020 09:18:39		
Date Stamp	29 Jun 2020 09:20:08			***File Name***	C:\USERS\Лаба534\DOWNLOADS\FZ8712-1.JDF	***Frequency (MHz)***	600.17
Nucleus	1H	***Number of Transients***	8	***Origin***	ECA 600	***Original Points Count*** 32768	***Owner*** CKP
Points Count	32768	***Pulse Sequence***	single_pulse.ex2			***Receiver Gain*** 44.00	***Solvent*** CHLOROFORM-d
Spectrum Offset (Hz)	5409.1133	***Sweep Width (Hz)***	16534.39	***Temperature (degree C)***	22.100		

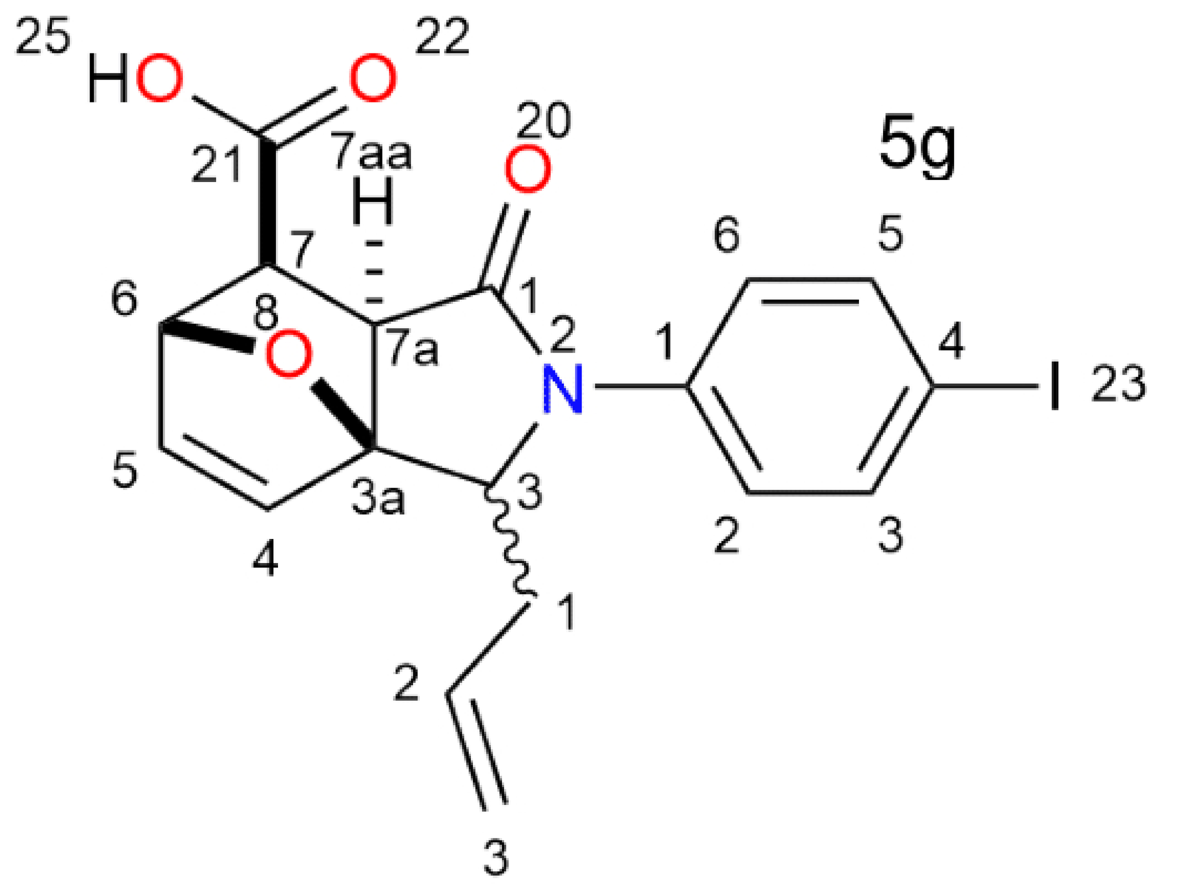

FZ8712-1.JDF

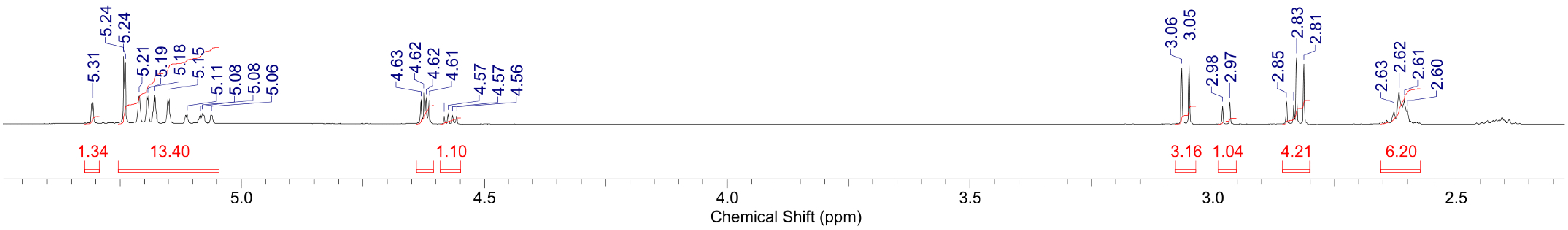

NMR spectrum data for compound 5g (DCM, r.t, 3 d)

Formula $C_{18}H_{16}INO_4$	FW 437.2284

Acquisition Time (sec)	1.9818	***Comment***	single_pulse	***Date***	29 Jun 2020 09:42:56		
Date Stamp	29 Jun 2020 09:44:25			***File Name***	F:\NMR FIDS ACIDS\4-I ALLYL\FZ8713 2.4-I.JDF		
Frequency (MHz)	600.17	***Nucleus***	1H	***Number of Transients***	8	***Origin***	ECA 600
Original Points Count	32768	***Owner***	CKP	***Points Count***	32768	***Pulse Sequence***	single_pulse.ex2
Receiver Gain	52.00	***Solvent***	CHLOROFORM-d			***Spectrum Offset (Hz)***	5409.6182
Sweep Width (Hz)	16534.39	***Temperature (degree C)***	22.200				

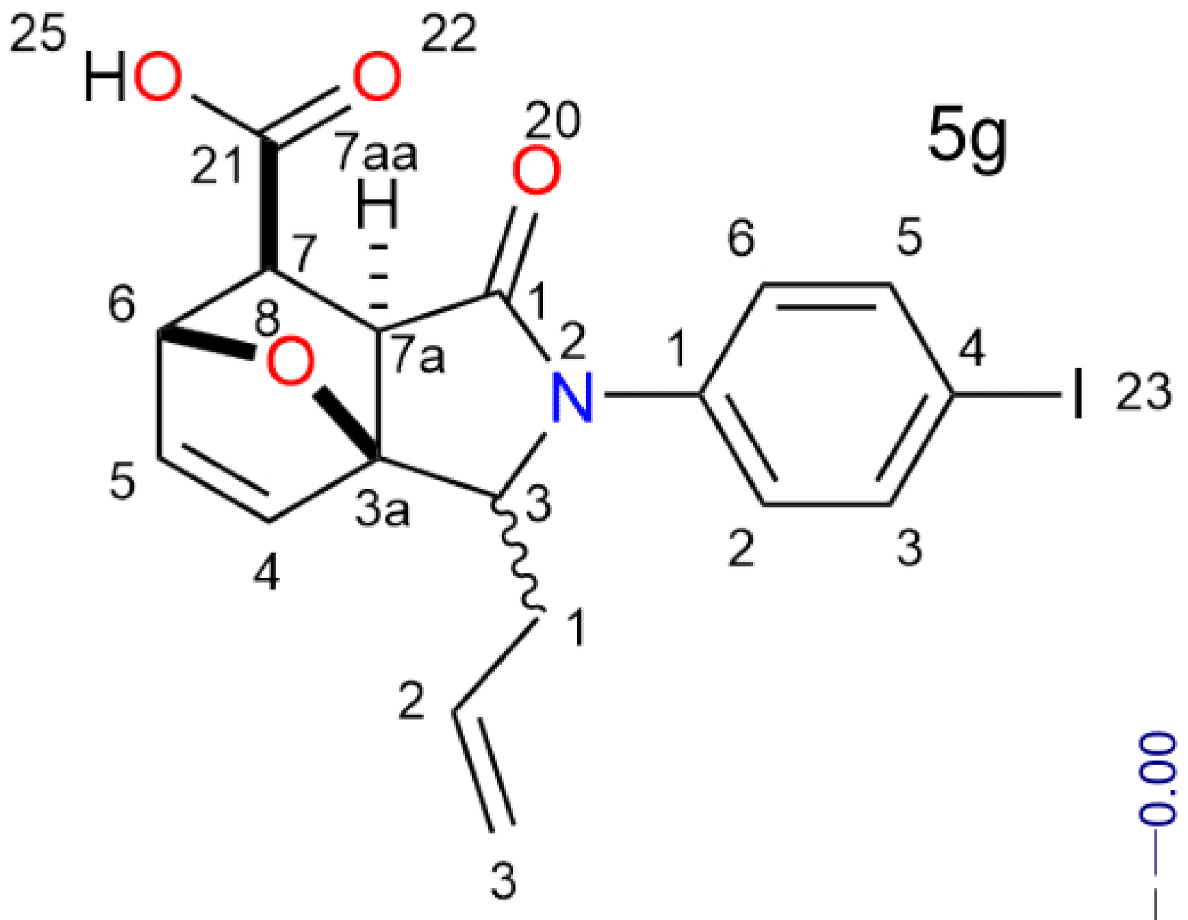

FZ8713 2.4-I.JDF

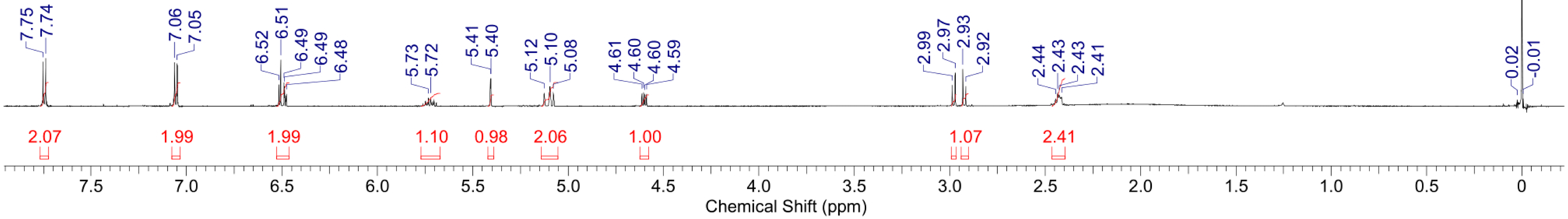

Formula $C_{18}H_{16}INO_4$	FW 437.2284

**Acquisition Time (sec)**	1.9818	**Comment**	single_pulse	**Date**	29 Jun 2020 09:42:56		
**Date Stamp**	29 Jun 2020 09:44:25			**File Name**	F:\NMR FIDS ACIDS\4-I ALLYL\FZ8713 2.4-I.JDF		
**Frequency (MHz)**	600.17	**Nucleus**	1H	**Number of Transients**	8	**Origin**	ECA 600
**Original Points Count**	32768	**Owner**	CKP	**Points Count**	32768	**Pulse Sequence**	single_pulse.ex2
**Receiver Gain**	52.00	**Solvent**	CHLOROFORM-d			**Spectrum Offset (Hz)**	5409.6182
**Sweep Width (Hz)**	16534.39	**Temperature (degree C)**	22.200				

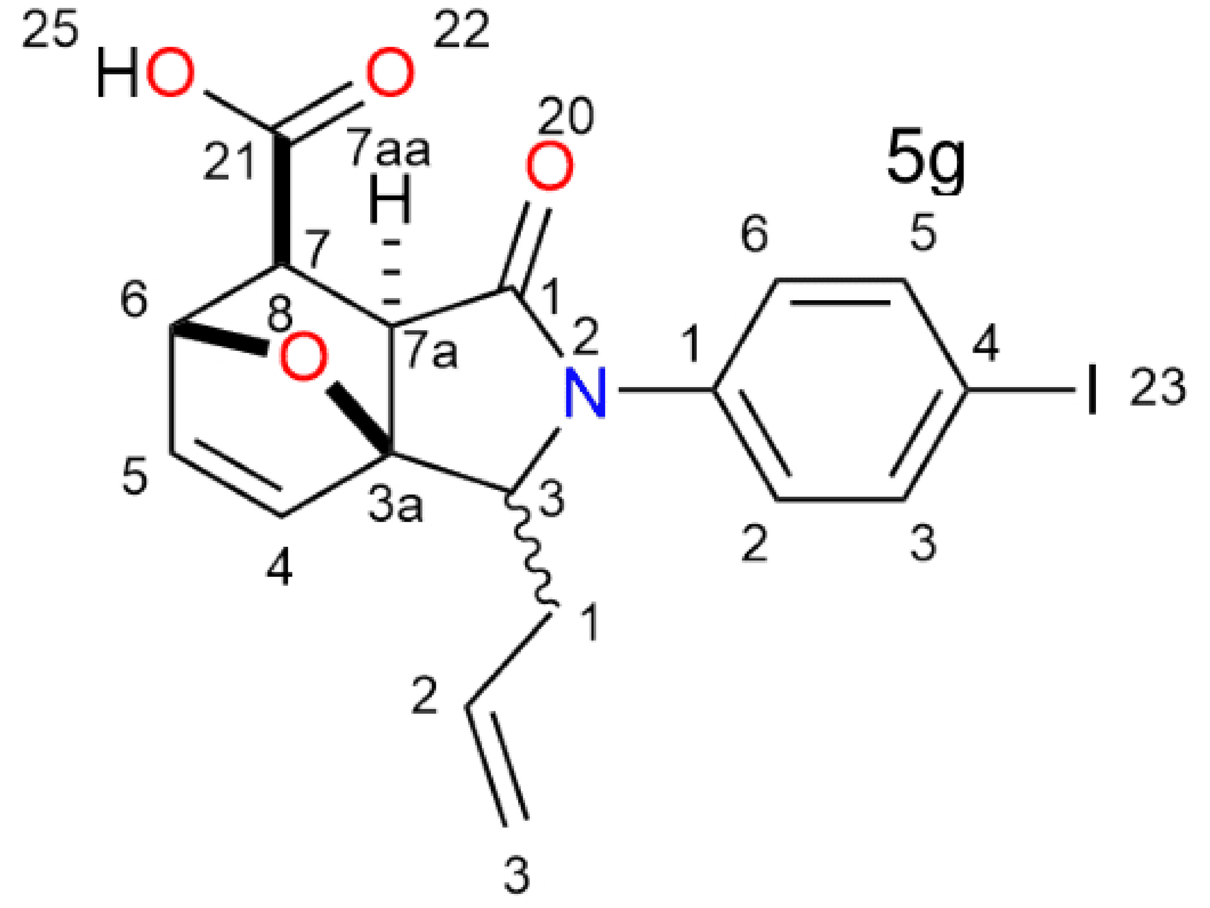

FZ8713 2.4-I.JDF

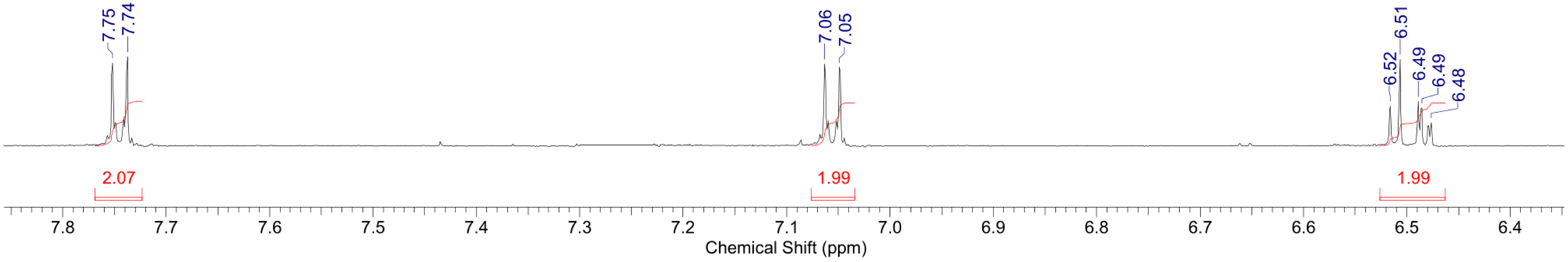

Formula $C_{18}H_{16}INO_4$	FW 437.2284

Acquisition Time (sec)	1.9818	***Comment***	single_pulse	***Date***	29 Jun 2020 09:42:56		
Date Stamp	29 Jun 2020 09:44:25			***File Name***	F:\NMR FIDS ACIDS\4-I ALLYL\FZ8713 2.4-I.JDF		
Frequency (MHz)	600.17	***Nucleus***	1H	***Number of Transients***	8	***Origin***	ECA 600
Original Points Count	32768	***Owner***	CKP	***Points Count***	32768	***Pulse Sequence***	single_pulse.ex2
Receiver Gain	52.00	***Solvent***	CHLOROFORM-d			***Spectrum Offset (Hz)***	5409.6182
Sweep Width (Hz)	16534.39	***Temperature (degree C)***	22.200				

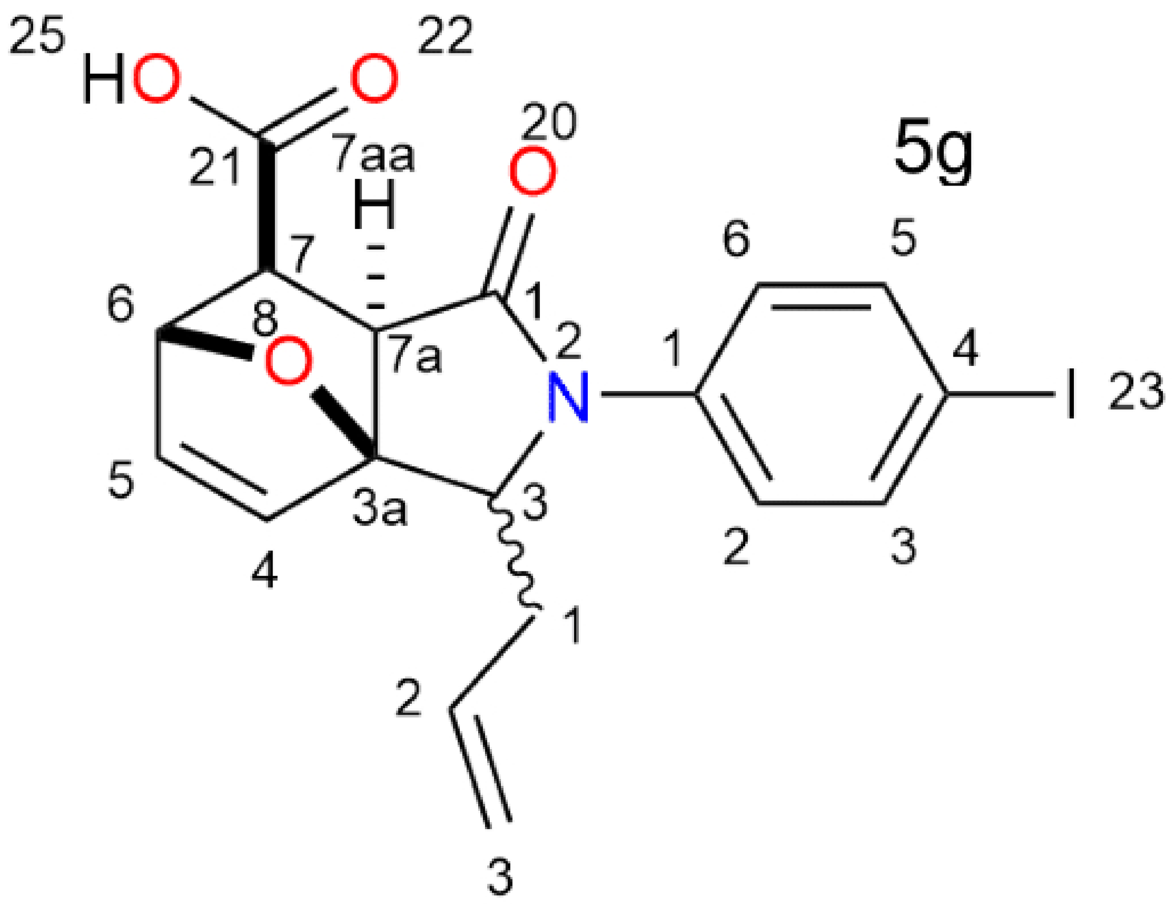

FZ8713 2.4-I.JDF

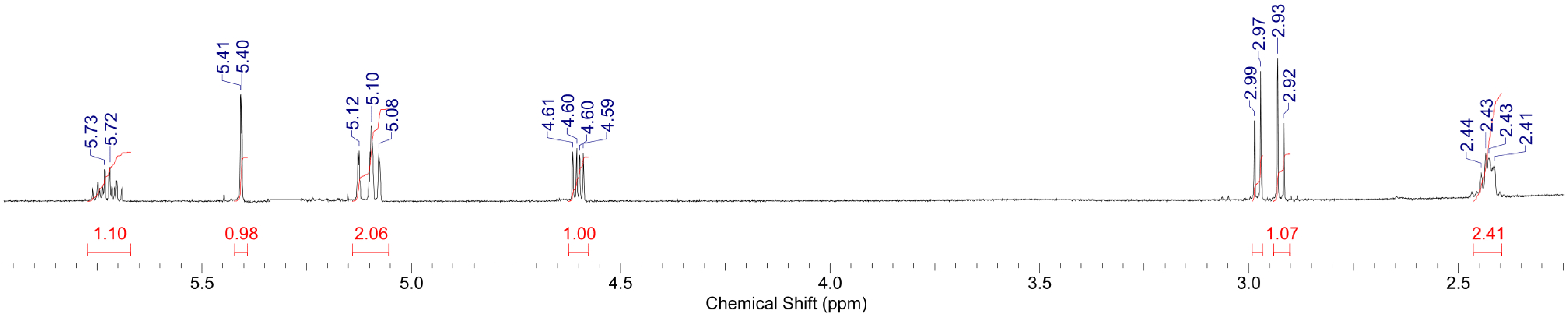

NMR spectrum data for compound 5g (MeCN, r.t., 3d)

Formula	$C_{18}H_{16}INO_4$	FW	437.2284

**Acquisition Time (sec)**	1.9818	**Comment**	single_pulse	**Date**	23 Jul 2020 13:19:24		
**Date Stamp**	23 Jul 2020 13:20:37			**File Name**	C:\USERS\na6a 534\DOWNLOADS\FZ8855-1.JDF		
**Frequency (MHz)**	600.17	**Nucleus**	1H	**Number of Transients**	8	**Origin**	ECA 600
**Original Points Count**	32768	**Owner**	CKP	**Points Count**	32768	**Pulse Sequence**	single_pulse.ex2
**Receiver Gain**	52.00	**Solvent**	CHLOROFORM-d			**Spectrum Offset (Hz)**	5409.1133
**Sweep Width (Hz)**	16534.39						

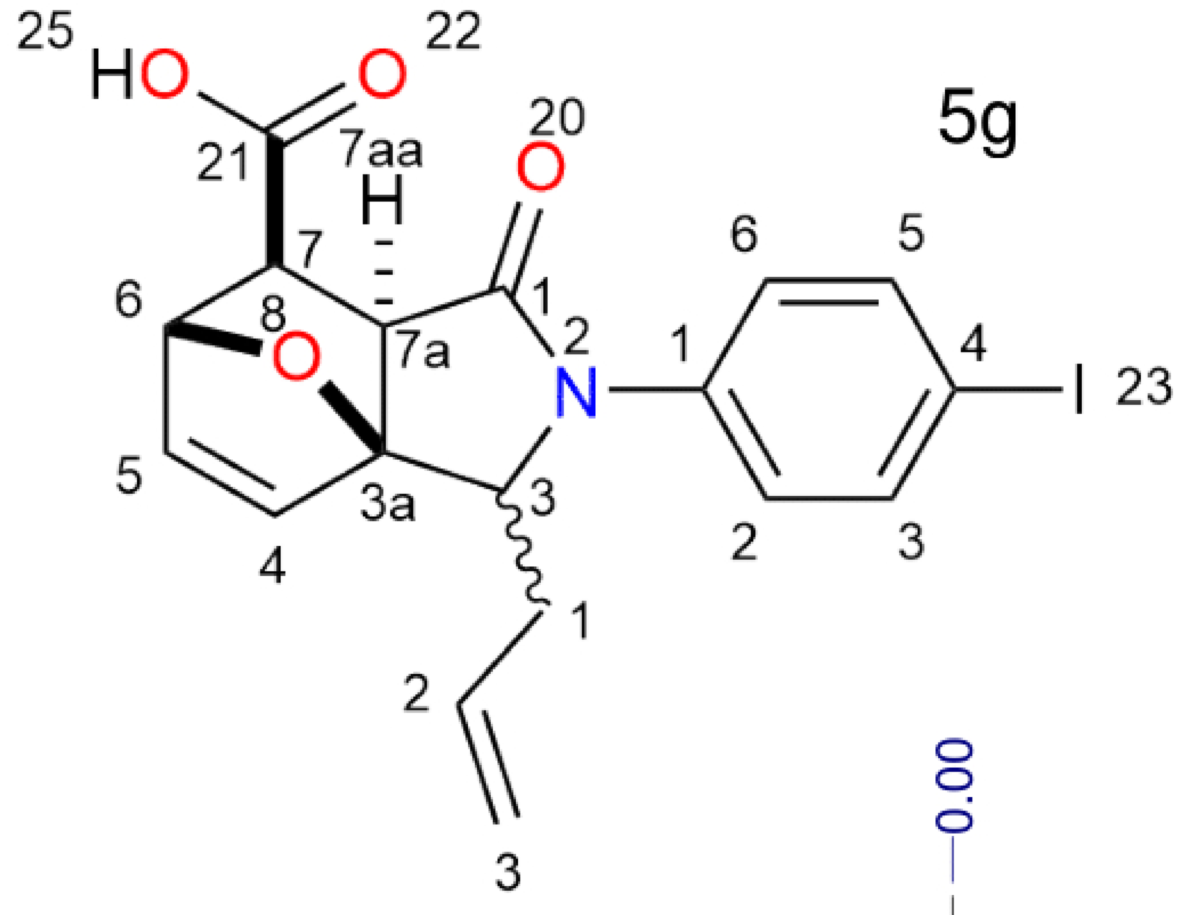

5g

FZ8855-1.JDF

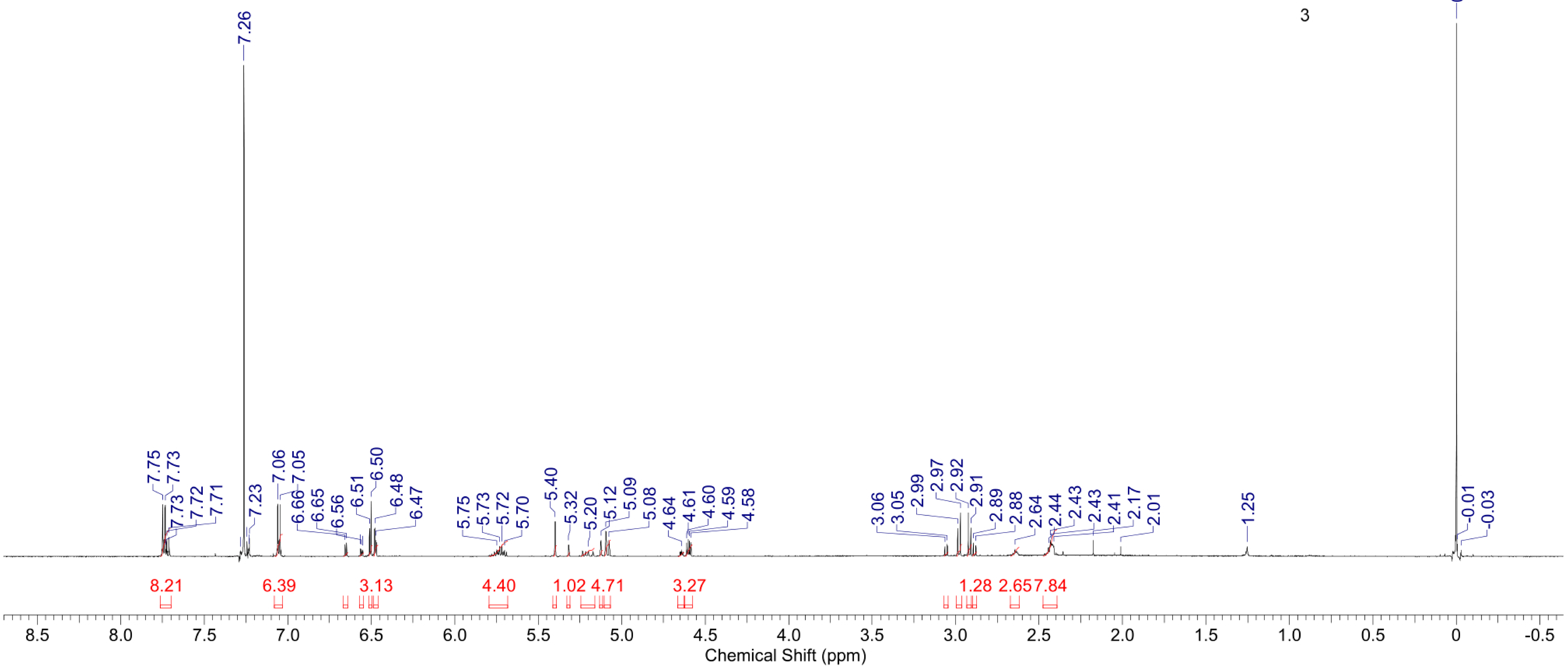

**Formula** $C_{18}H_{16}INO_4$	**FW** 437.2284

**Acquisition Time (sec)**	1.9818	**Comment**	single_pulse	**Date**	23 Jul 2020 13:19:24		
**Date Stamp**	23 Jul 2020 13:20:37			**File Name**	C:\USERS\па6а 534\DOWNLOADS\FZ8855-1.JDF		
**Frequency (MHz)**	600.17	**Nucleus**	1H	**Number of Transients**	8	**Origin**	ECA 600
**Original Points Count**	32768	**Owner**	CKP	**Points Count**	32768	**Pulse Sequence**	single_pulse.ex2
**Receiver Gain**	52.00	**Solvent**	CHLOROFORM-d			**Spectrum Offset (Hz)**	5409.1133
**Sweep Width (Hz)**	16534.39						

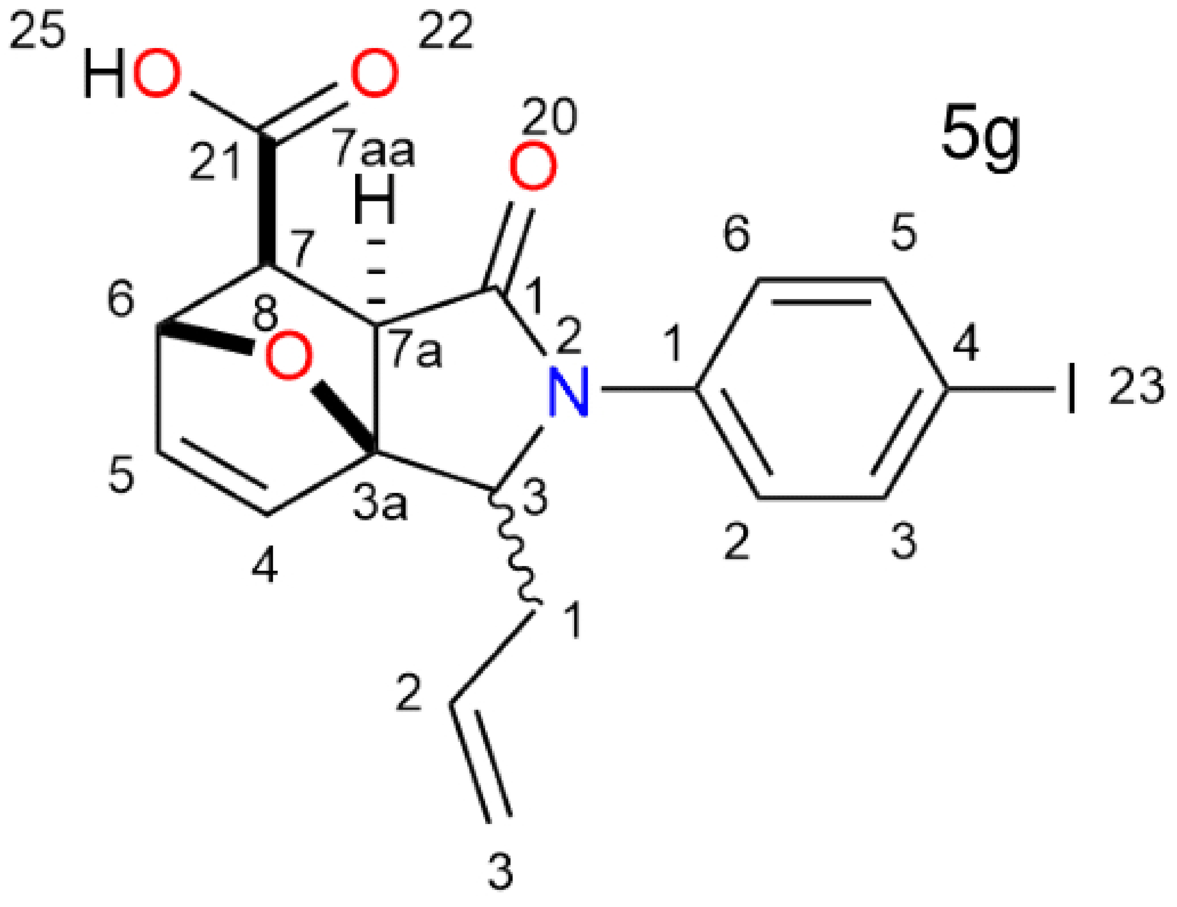

5g

FZ8855-1.JDF

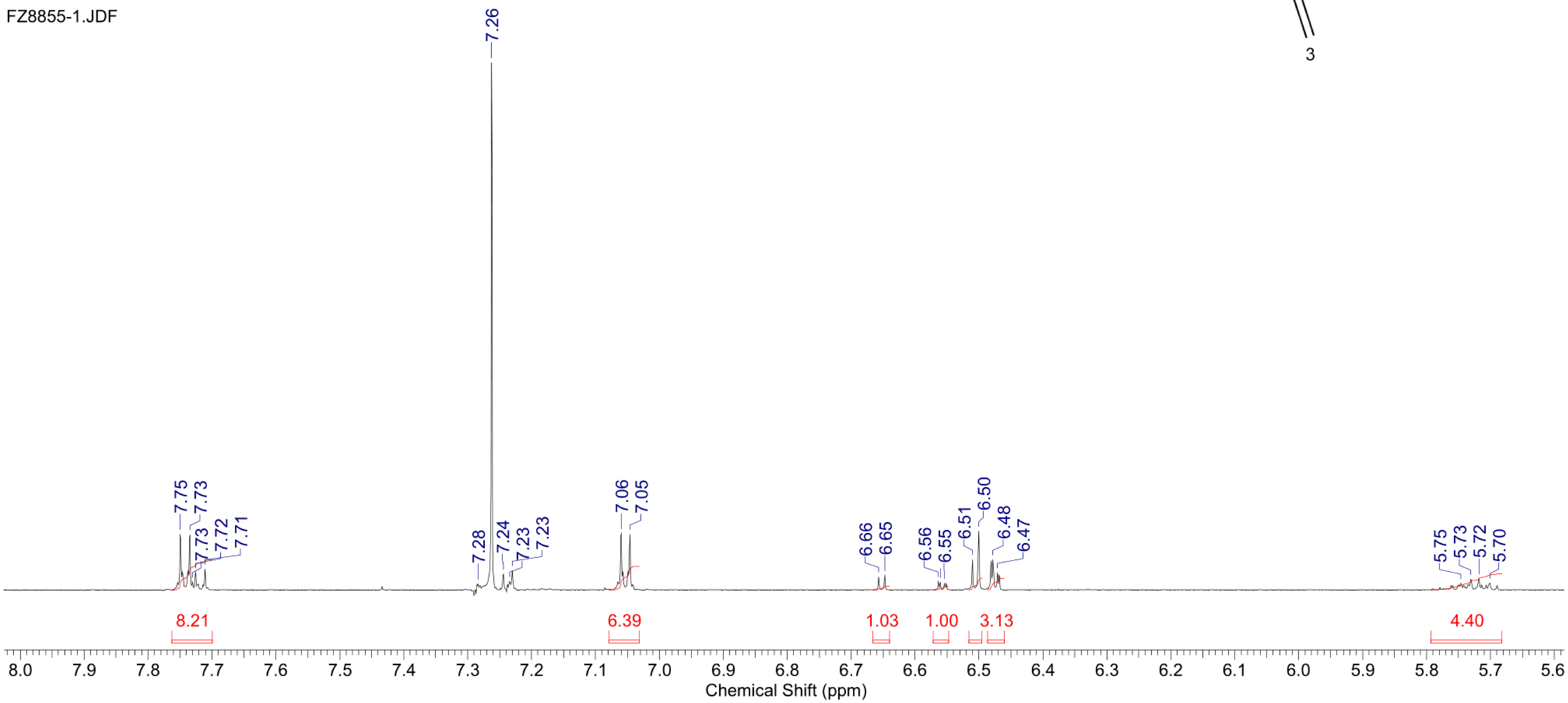

Formula $C_{18}H_{16}INO_4$	FW 437.2284

Acquisition Time (sec)	1.9818	***Comment***	single_pulse	***Date***	23 Jul 2020 13:19:24		
Date Stamp	23 Jul 2020 13:20:37			***File Name***	C:\USERS\па6а 534\DOWNLOADS\FZ8855-1.JDF		
Frequency (MHz)	600.17	***Nucleus***	1H	***Number of Transients***	8	***Origin***	ECA 600
Original Points Count	32768	***Owner***	CKP	***Points Count***	32768	***Pulse Sequence***	single_pulse.ex2
Receiver Gain	52.00	***Solvent***	CHLOROFORM-d			***Spectrum Offset (Hz)***	5409.1133
Sweep Width (Hz)	16534.39						

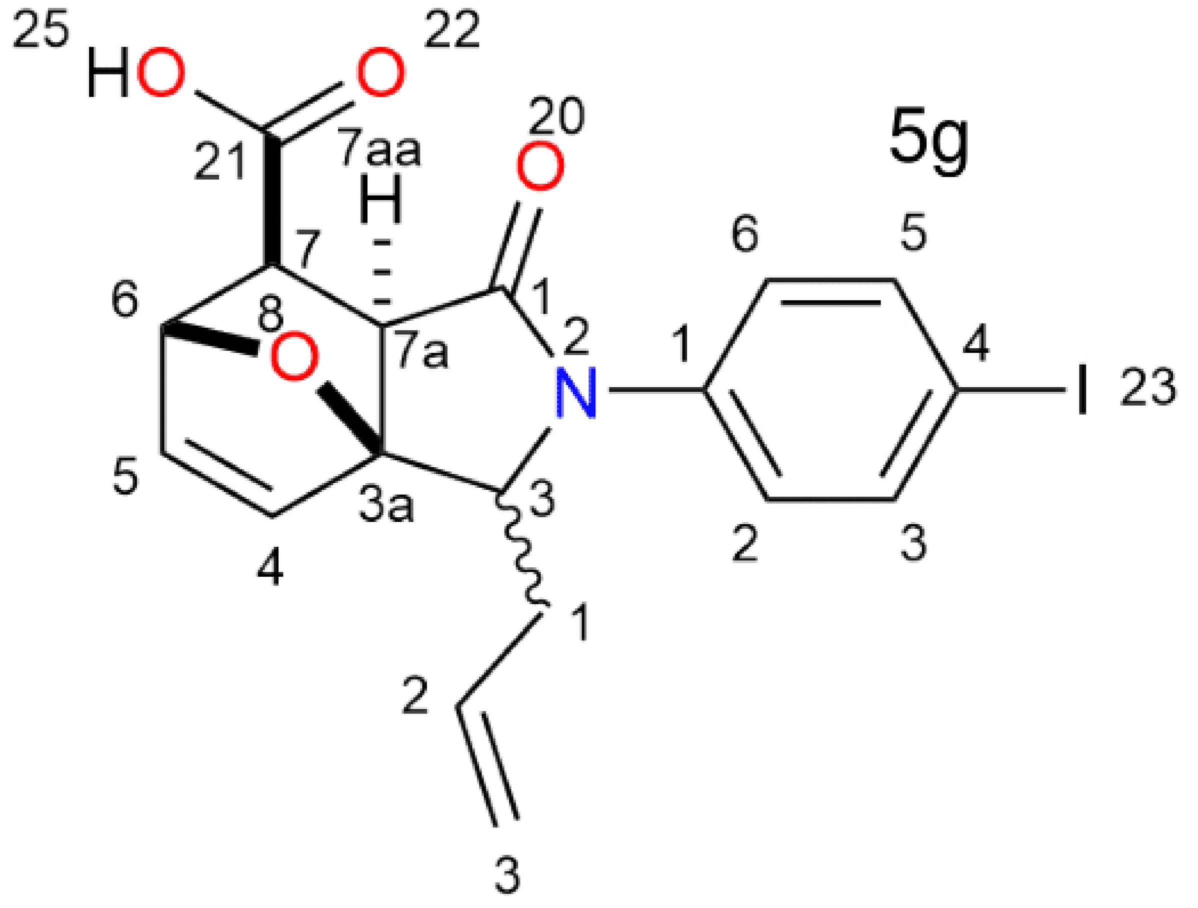

5g

FZ8855-1.JDF

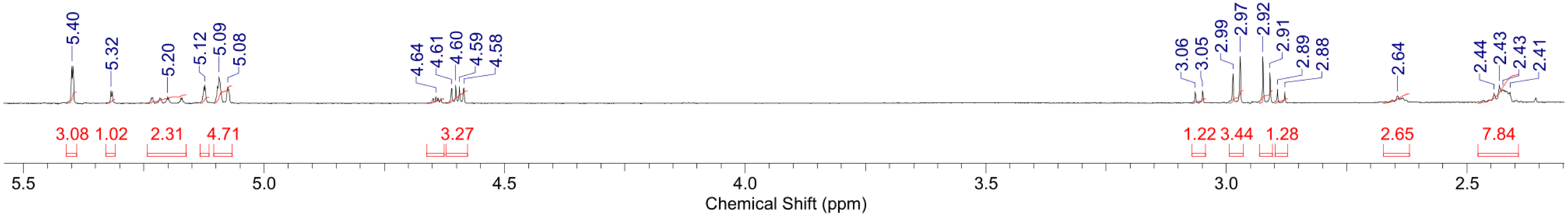

NMR spectrum data for compound 5g (MeCN, Δ, 3h)

**Formula**	$C_{18}H_{17}NO_4$	**FW**	311.3319

Acquisition Time (sec)	1.9818	***Comment***	single_pulse	***Date***	07 Jul 2020 11:36:10		
Date Stamp	07 Jul 2020 11:37:42			***File Name***	C:\USERS\лаба 534\DOWNLOADS\FZ8739-1.JDF		
Frequency (MHz)	600.17	***Nucleus***	1H	***Number of Transients***	8	***Origin***	ECA 600
Original Points Count	32768	***Owner***	CKP	***Points Count***	32768	***Pulse Sequence***	single_pulse.ex2
Receiver Gain	48.00	***Solvent***	CHLOROFORM-d			***Spectrum Offset (Hz)***	5411.1318
Sweep Width (Hz)	16534.39	***Temperature (degree C)***	23.000				

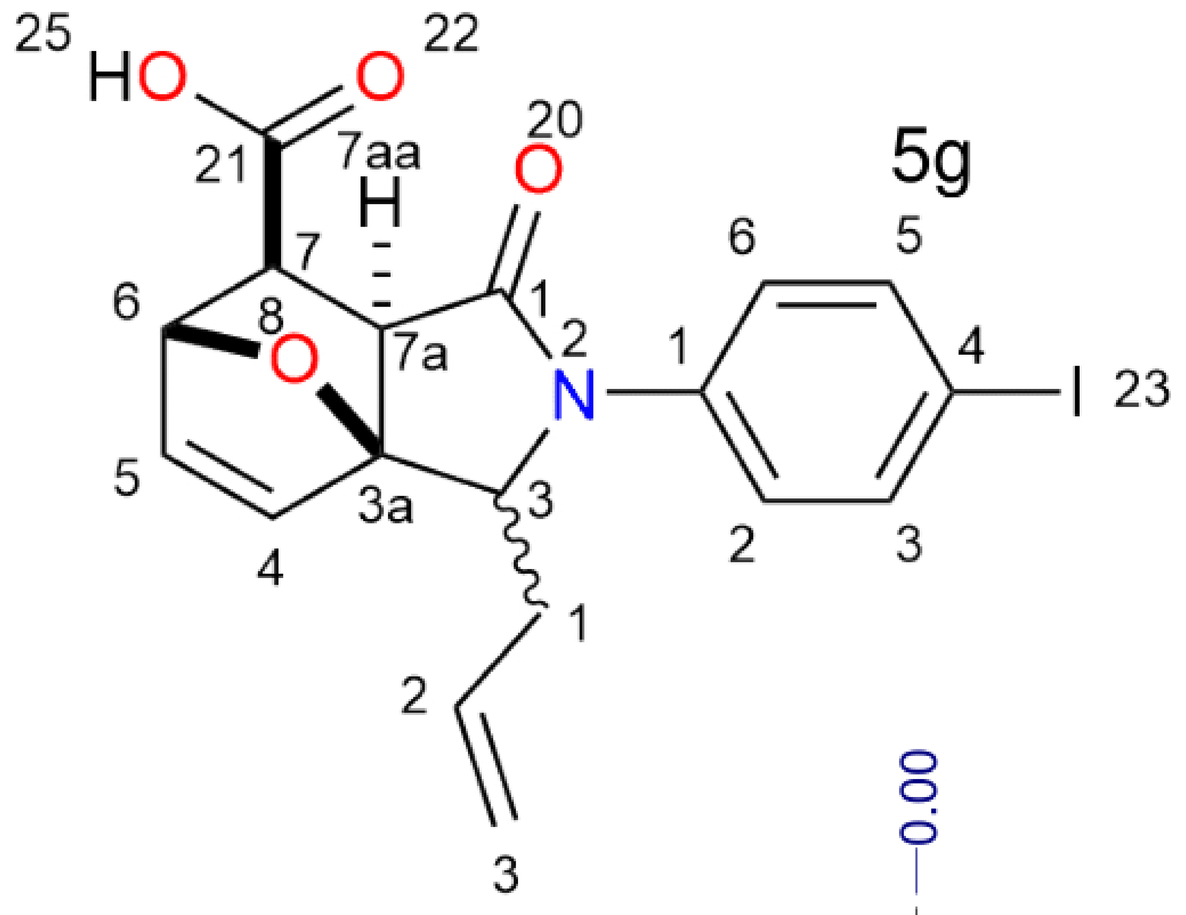

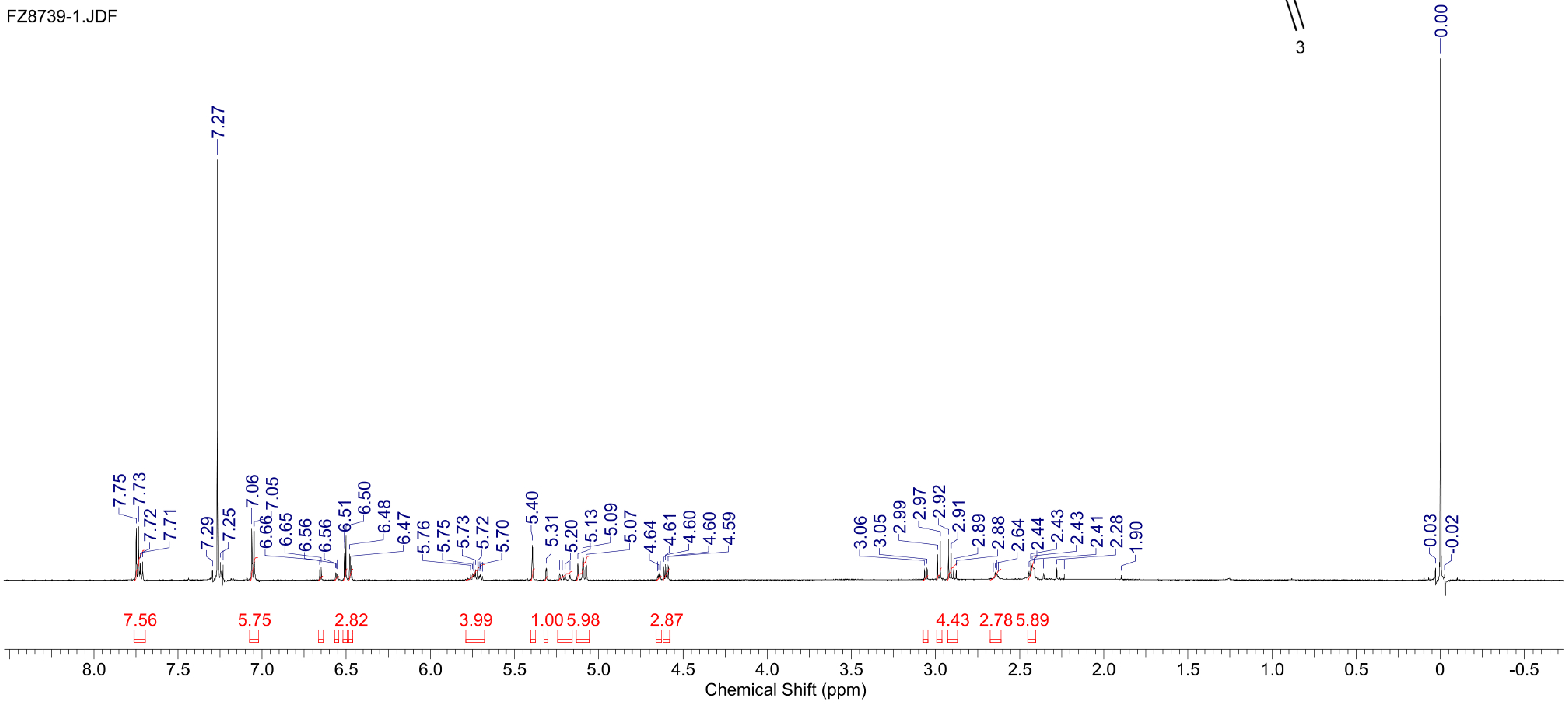

Formula $C_{18}H_{17}NO_4$	*FW* 311.3319

Acquisition Time (sec)	1.9818	***Comment***	single_pulse	***Date***	07 Jul 2020 11:36:10		
Date Stamp	07 Jul 2020 11:37:42			***File Name***	C:\USERS\па6а 534\DOWNLOADS\FZ8739-1.JDF		
Frequency (MHz)	600.17	***Nucleus***	1H	***Number of Transients***	8	***Origin***	ECA 600
Original Points Count	32768	***Owner***	CKP	***Points Count***	32768	***Pulse Sequence***	single_pulse.ex2
Receiver Gain	48.00	***Solvent***	CHLOROFORM-d			***Spectrum Offset (Hz)***	5411.1318
Sweep Width (Hz)	16534.39	***Temperature (degree C)***	23.000				

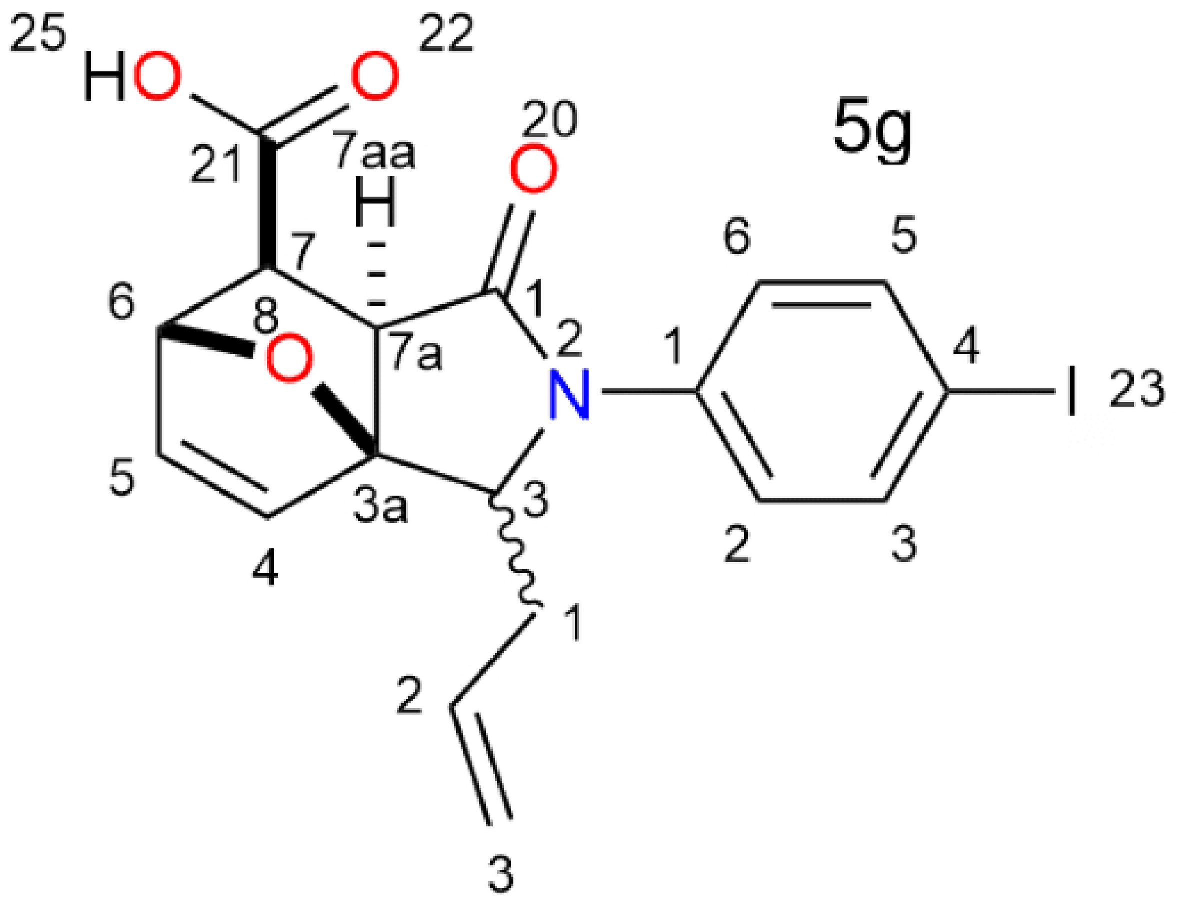

5g

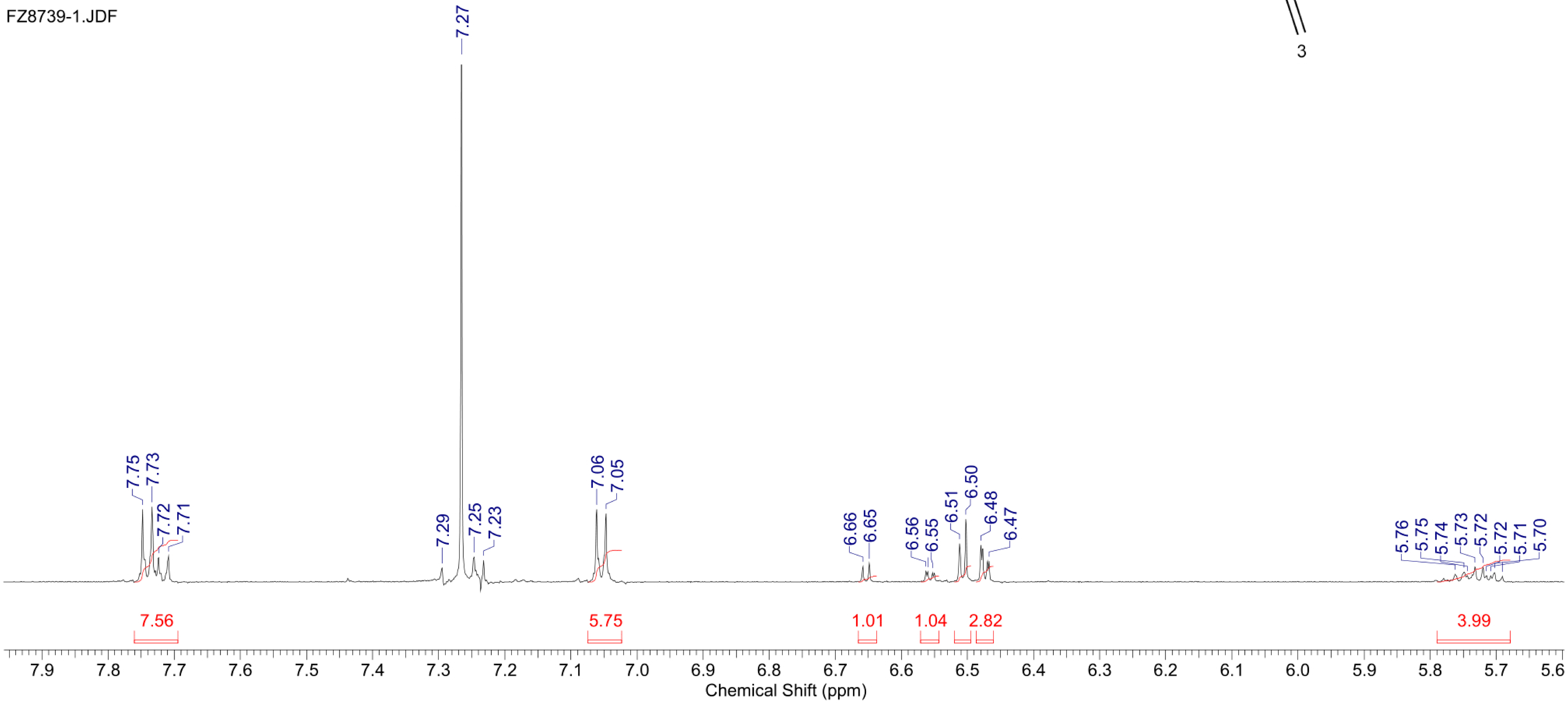

Formula $C_{18}H_{17}NO_4$	FW 311.3319

**Acquisition Time (sec)**	1.9818	**Comment**	single_pulse	**Date**	07 Jul 2020 11:36:10		
**Date Stamp**	07 Jul 2020 11:37:42			**File Name**	C:\USERS\лаба 534\DOWNLOADS\FZ8739-1.JDF		
**Frequency (MHz)**	600.17	**Nucleus**	1H	**Number of Transients**	8	**Origin**	ECA 600
**Original Points Count**	32768	**Owner**	CKP	**Points Count**	32768	**Pulse Sequence**	single_pulse.ex2
**Receiver Gain**	48.00	**Solvent**	CHLOROFORM-d			**Spectrum Offset (Hz)**	5411.1318
**Sweep Width (Hz)**	16534.39	**Temperature (degree C)**	23.000				

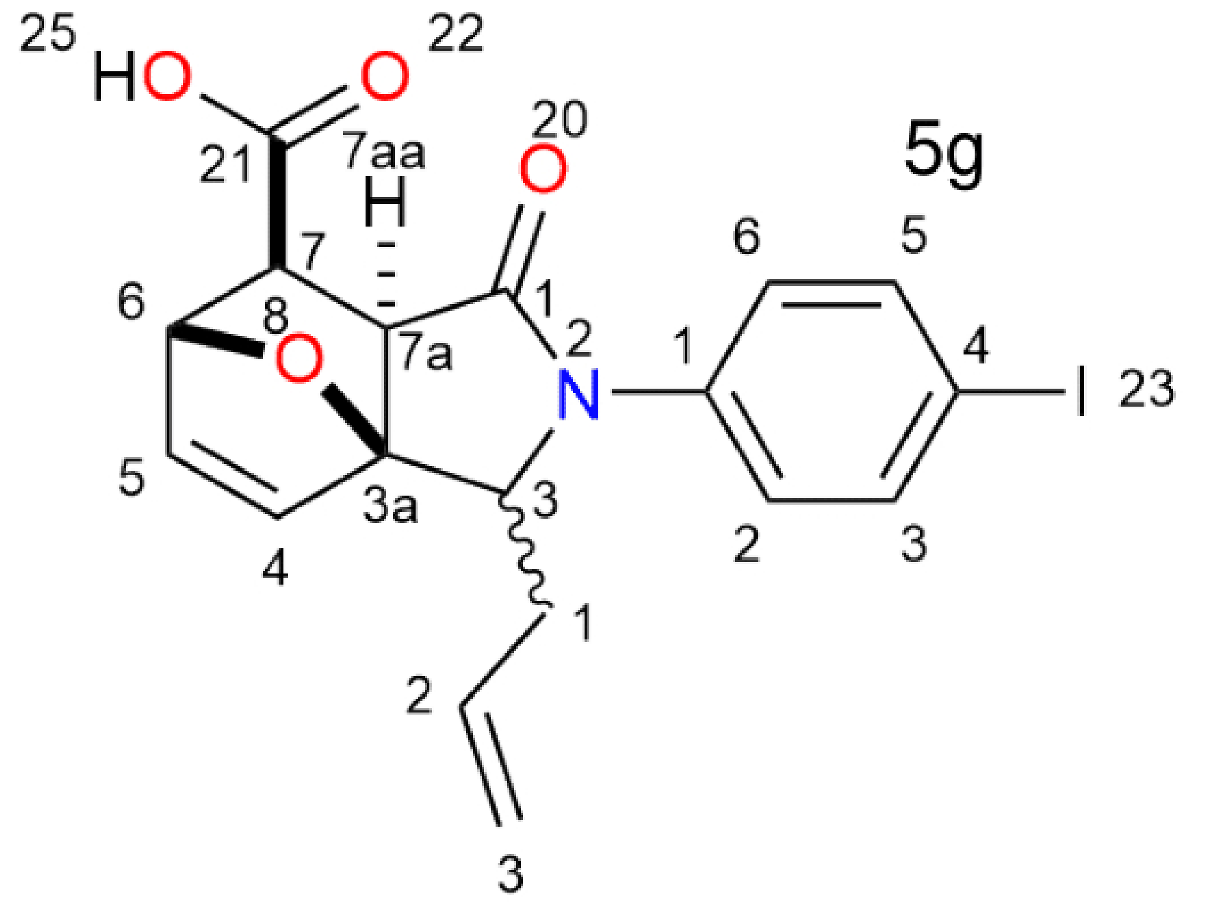

5g

FZ8739-1.JDF

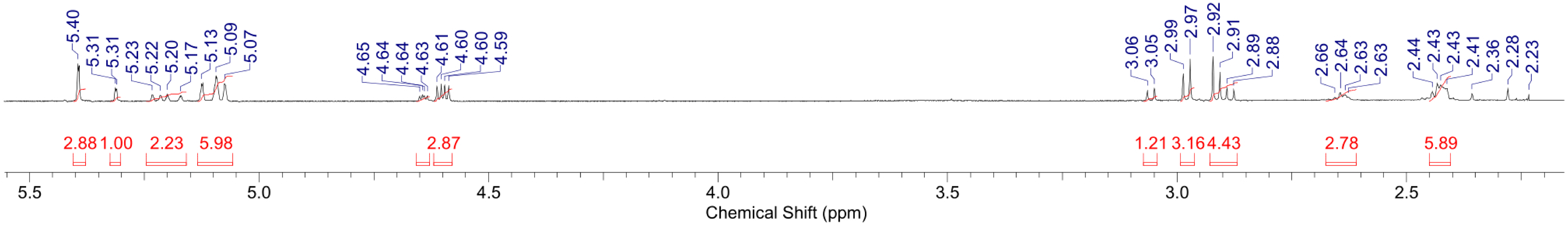

NMR spectrum data for compound 5g (PhH, Δ, 3 h)

**Formula** $C_{18}H_{16}INO_4$	**FW** 437.2284

Acquisition Time (sec)	1.9818	***Comment***	single_pulse	***Date***	24 Jul 2020 09:13:30		
Date Stamp	24 Jul 2020 09:14:44			***File Name***	C:\USERS\па6а 534\DOWNLOADS\FZ8865-1.JDF		
Frequency (MHz)	600.17	***Nucleus***	1H	***Number of Transients***	8	***Origin***	ECA 600
Original Points Count	32768	***Owner***	CKP	***Points Count***	32768	***Pulse Sequence***	single_pulse.ex2
Receiver Gain	42.00	***Solvent***	CHLOROFORM-d			***Spectrum Offset (Hz)***	5406.5903
Sweep Width (Hz)	16534.39						

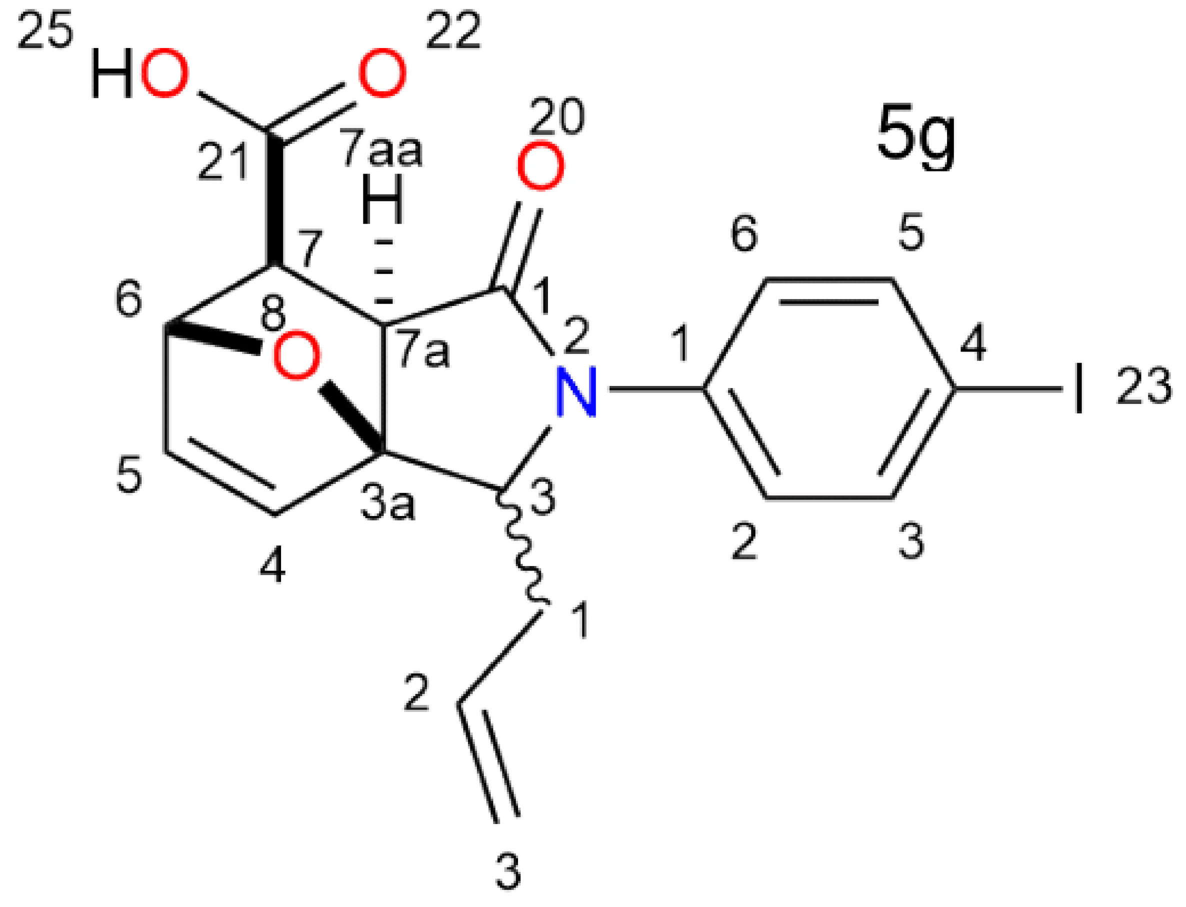

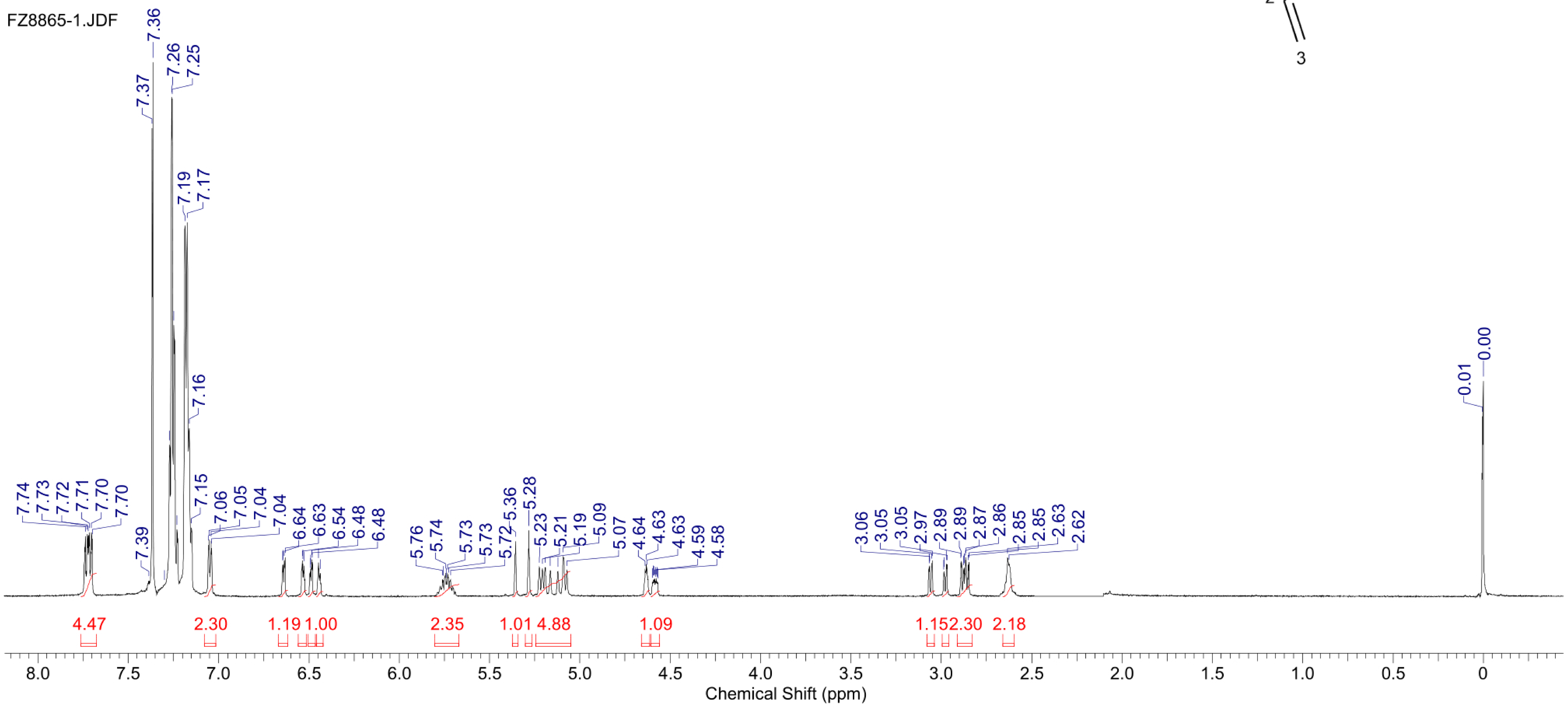

Formula $C_{18}H_{16}INO_4$	FW 437.2284

Acquisition Time (sec)	1.9818	***Comment***	single_pulse	***Date***	24 Jul 2020 09:13:30		
Date Stamp	24 Jul 2020 09:14:44			***File Name***	C:\USERS\па6а 534\DOWNLOADS\FZ8865-1.JDF		
Frequency (MHz)	600.17	***Nucleus***	1H	***Number of Transients***	8	***Origin***	ECA 600
Original Points Count	32768	***Owner***	CKP	***Points Count***	32768	***Pulse Sequence***	single_pulse.ex2
Receiver Gain	42.00	***Solvent***	CHLOROFORM-d			***Spectrum Offset (Hz)***	5406.5903
Sweep Width (Hz)	16534.39						

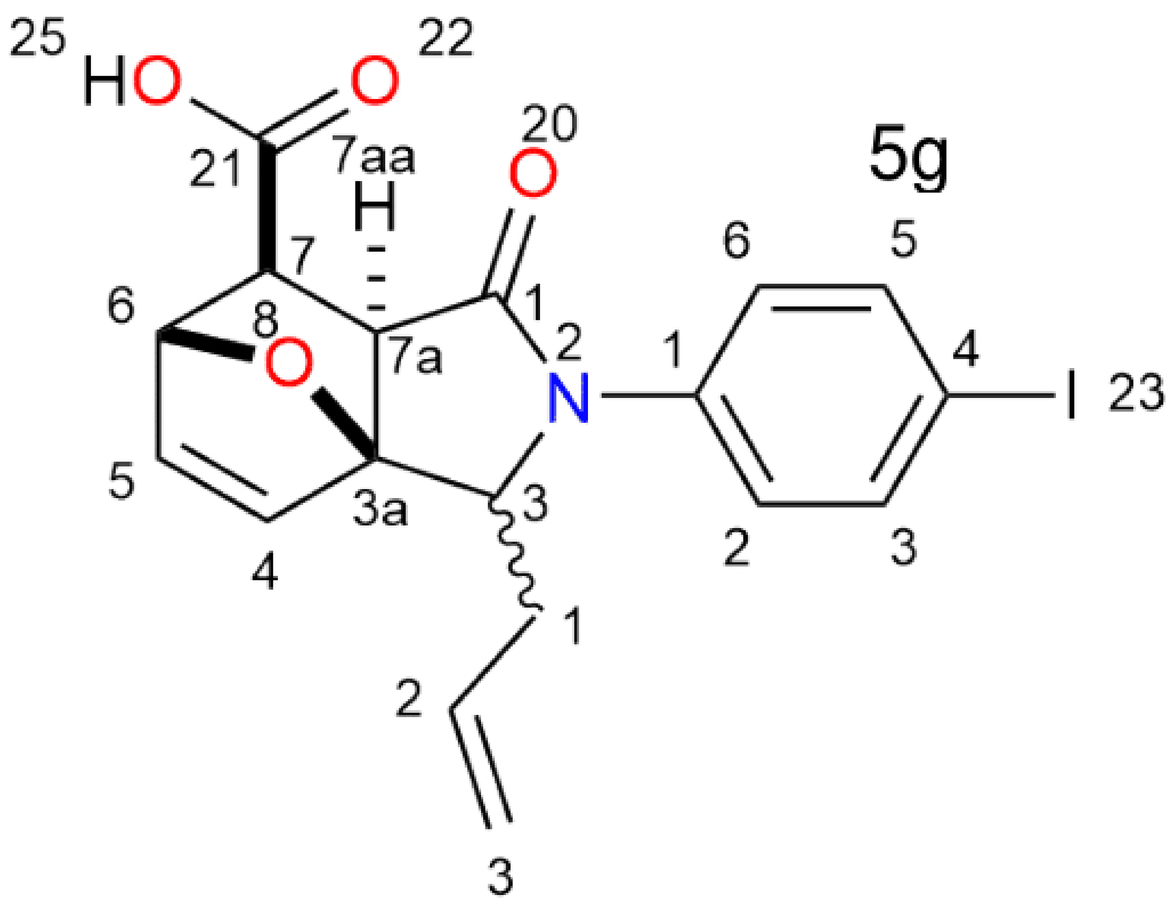

5g

FZ8865-1.JDF

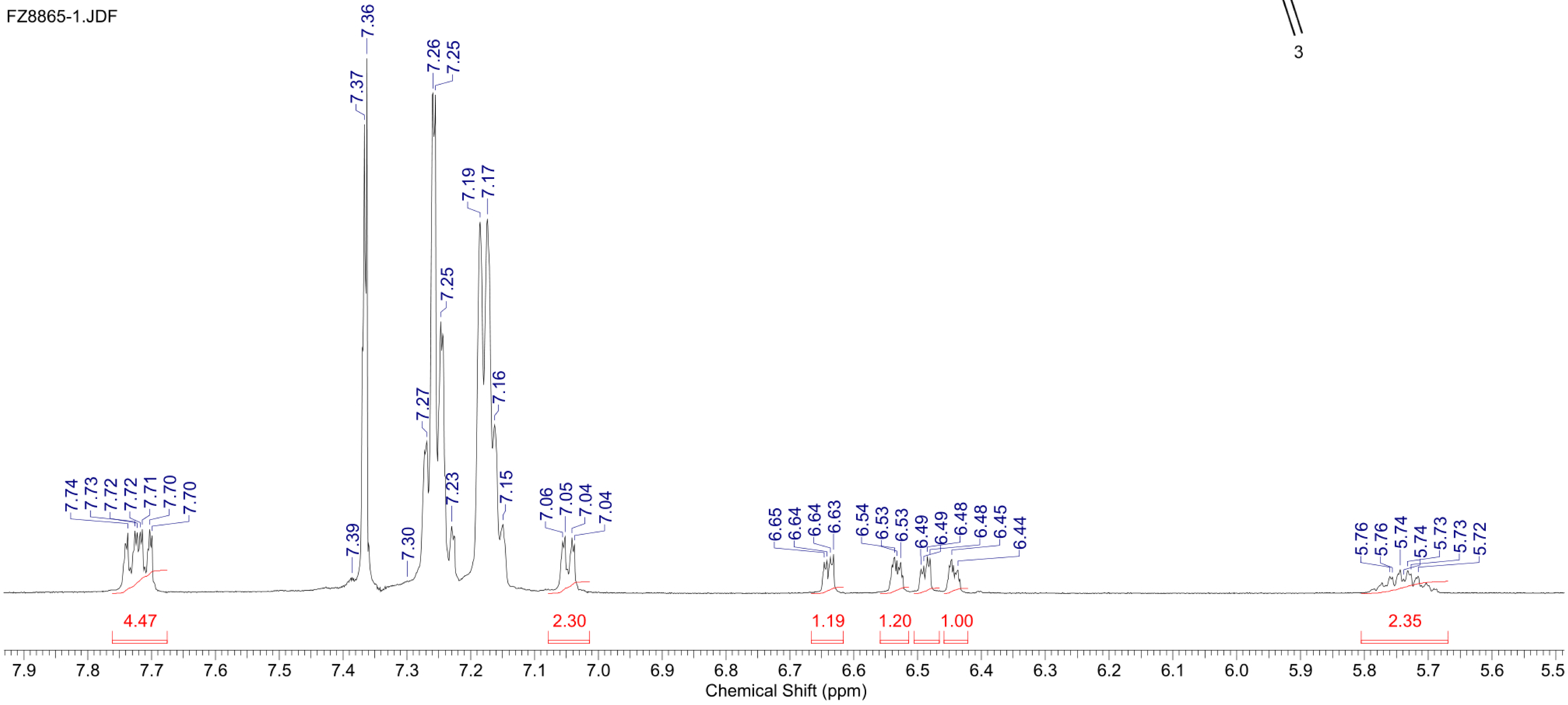

**Formula** $C_{18}H_{16}INO_4$	**FW** 437.2284

Acquisition Time (sec)	1.9818	***Comment***	single_pulse	***Date***	24 Jul 2020 09:13:30		
Date Stamp	24 Jul 2020 09:14:44			***File Name***	C:\USERS\па6а 534\DOWNLOADS\FZ8865-1.JDF		
Frequency (MHz)	600.17	***Nucleus***	1H	***Number of Transients***	8	***Origin***	ECA 600
Original Points Count	32768	***Owner***	CKP	***Points Count***	32768	***Pulse Sequence***	single_pulse.ex2
Receiver Gain	42.00	***Solvent***	CHLOROFORM-d			***Spectrum Offset (Hz)***	5406.5903
Sweep Width (Hz)	16534.39						

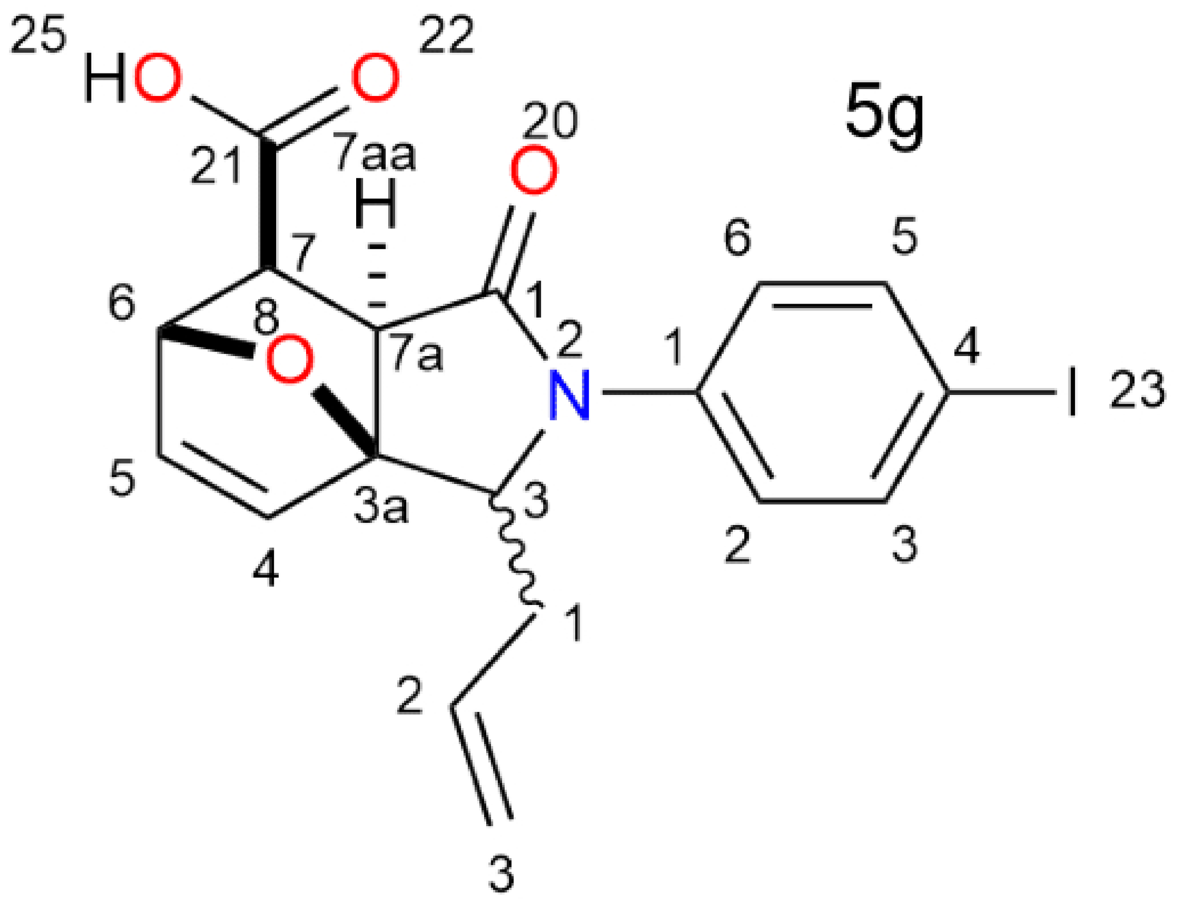

FZ8865-1.JDF

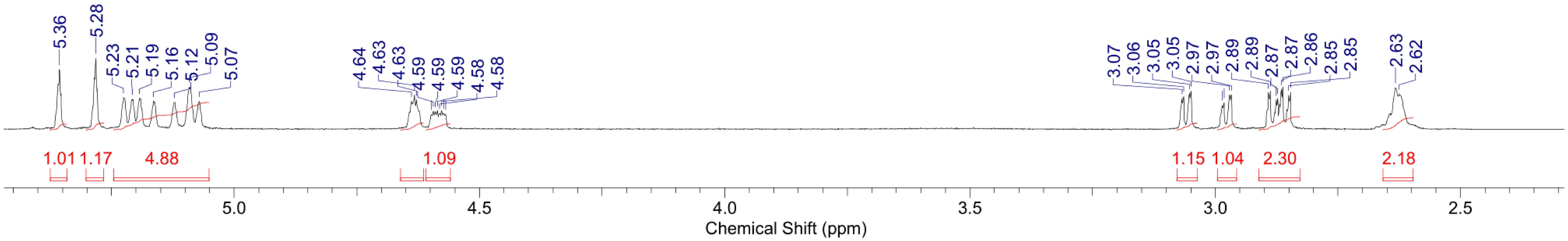

NMR spectrum data for compound 5g (PhMe, r.t, 3 d)

20.07.2020 18:27:03

Formula	$C_{18}H_{16}INO_4$	FW	437.2284

Acquisition Time (sec)	1.9818	Comment	single_pulse	Date	29 Jun 2020 09:36:33			Date Stamp	29 Jun 2020 09:38:03
File Name	E:\NMR FIDS ACIDS\FZ8714-1.JDF			Frequency (MHz)	600.17	Nucleus	1H	Number of Transients	8
Origin	ECA 600	Original Points Count	32768	Owner	CKP	Points Count	32768	Pulse Sequence	single_pulse.ex2
Receiver Gain	42.00	Solvent	CHLOROFORM-d			Spectrum Offset (Hz)	5410.1226	Sweep Width (Hz)	16534.39
Temperature (degree C)	22.200								

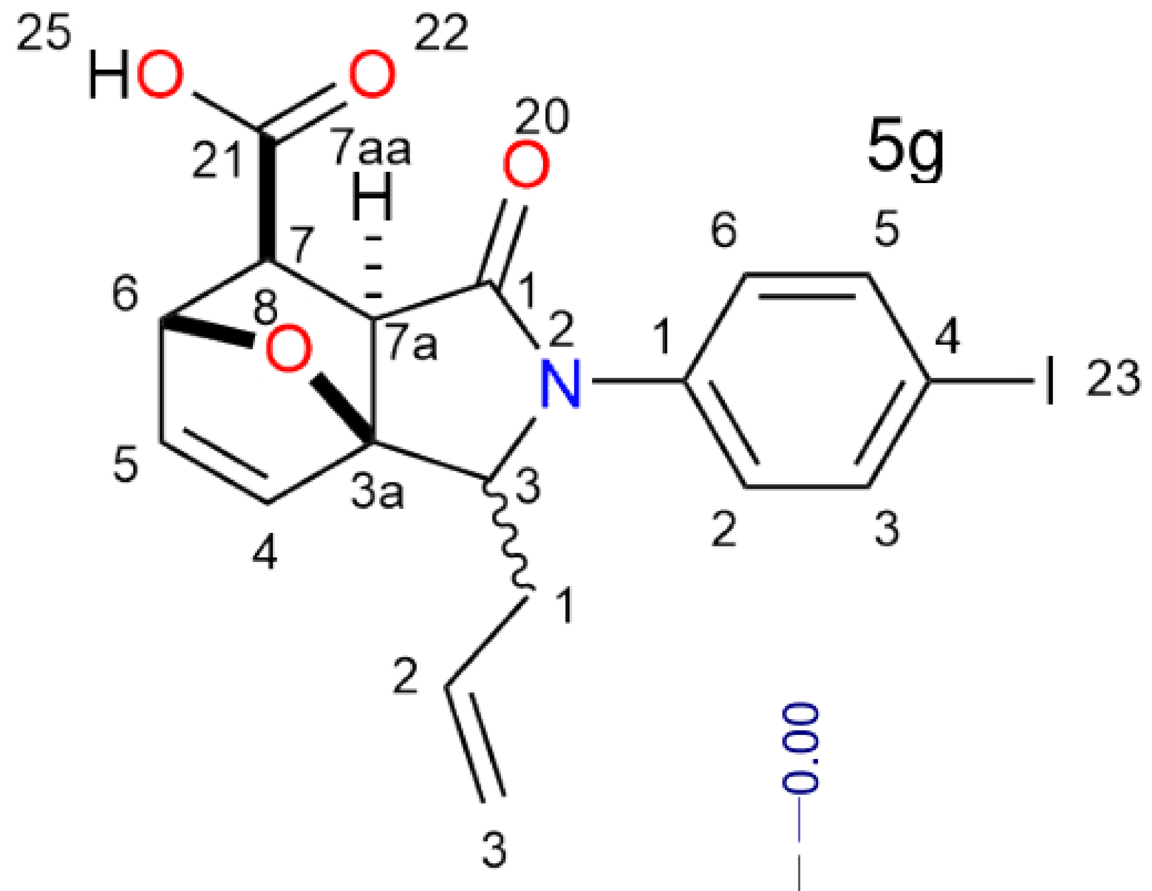

FZ8714-1.JDF

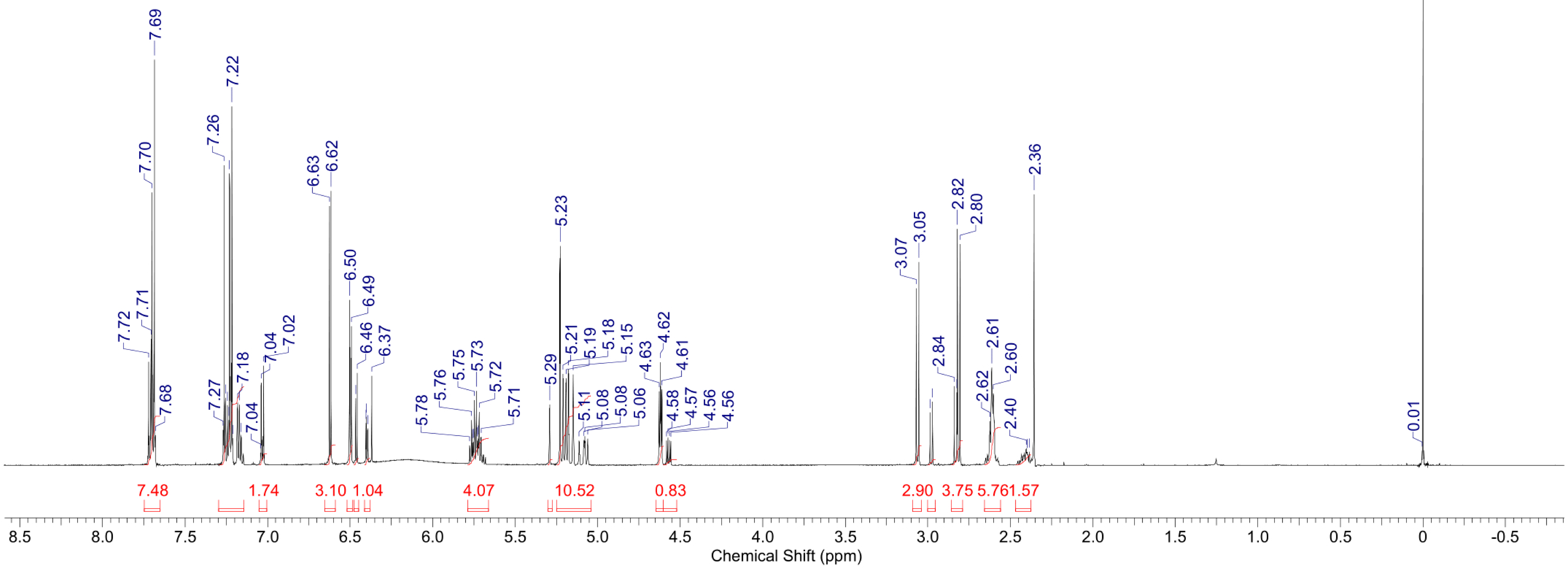

20.07.2020 18:27:11

Formula $C_{18}H_{16}INO_4$	FW 437.2284

**Acquisition Time (sec)**	1.9818	**Comment**	single_pulse	**Date**	29 Jun 2020 09:36:33			**Date Stamp**	29 Jun 2020 09:38:03
**File Name**	E:\NMR FIDS ACIDS\FZ8714-1.JDF			**Frequency (MHz)**	600.17	**Nucleus**	1H	**Number of Transients**	8
**Origin**	ECA 600	**Original Points Count**	32768	**Owner**	CKP	**Points Count**	32768	**Pulse Sequence**	single_pulse.ex2
**Receiver Gain**	42.00	**Solvent**	CHLOROFORM-d			**Spectrum Offset (Hz)**	5410.1226	**Sweep Width (Hz)**	16534.39
**Temperature (degree C)**	22.200								

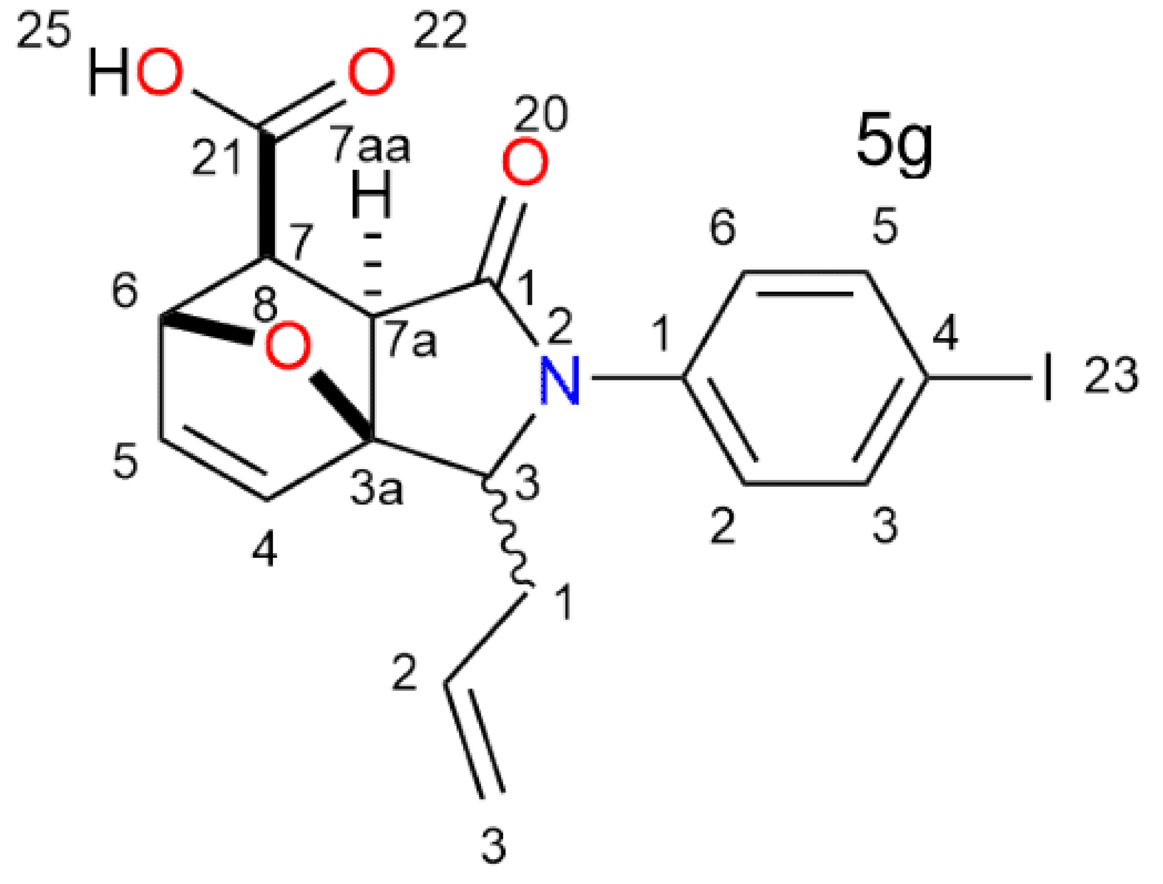

FZ8714-1.JDF

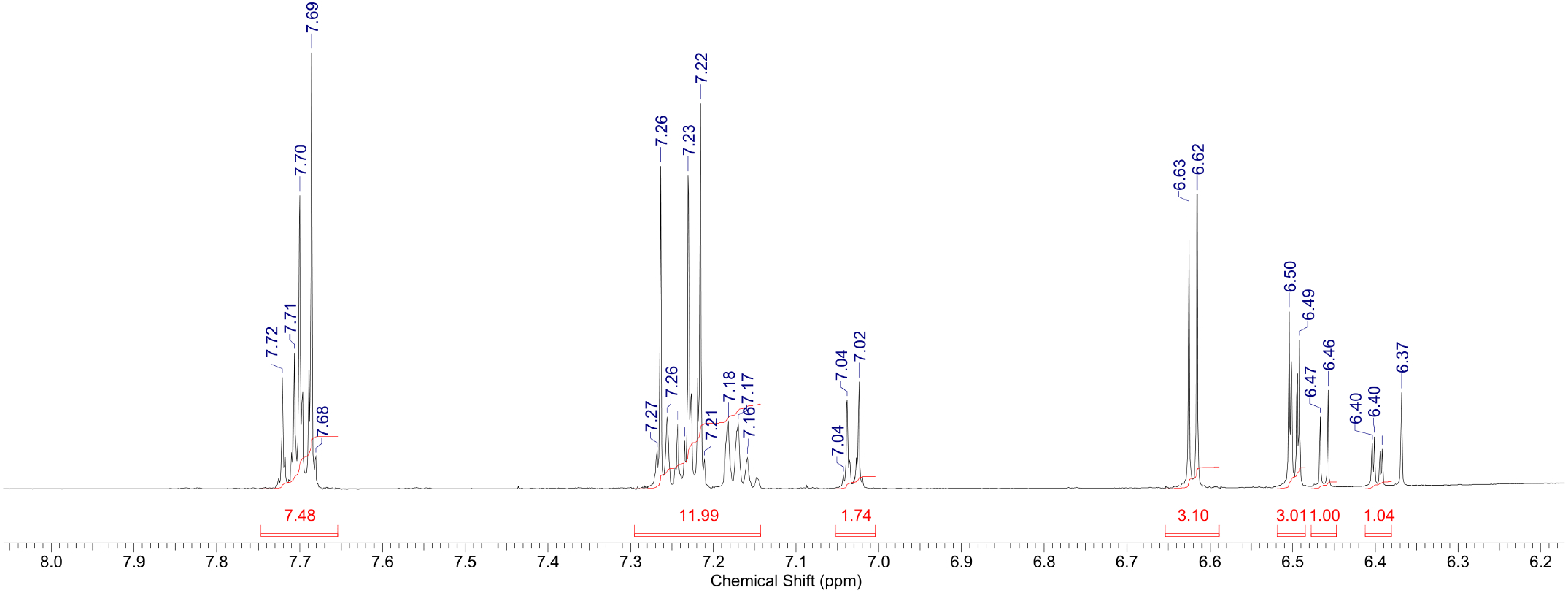

20.07.2020 18:27:21

**Formula** $C_{18}H_{16}INO_4$	**FW** 437.2284

**Acquisition Time (sec)**	1.9818	**Comment**	single_pulse	**Date**	29 Jun 2020 09:36:33			**Date Stamp**	29 Jun 2020 09:38:03
**File Name**	E:\NMR FIDS ACIDS\FZ8714-1.JDF			**Frequency (MHz)**	600.17	**Nucleus**	1H	**Number of Transients**	8
**Origin**	ECA 600	**Original Points Count**	32768	**Owner**	CKP	**Points Count**	32768	**Pulse Sequence**	single_pulse.ex2
**Receiver Gain**	42.00	**Solvent**	CHLOROFORM-d			**Spectrum Offset (Hz)**	5410.1226	**Sweep Width (Hz)**	16534.39
**Temperature (degree C)**	22.200								

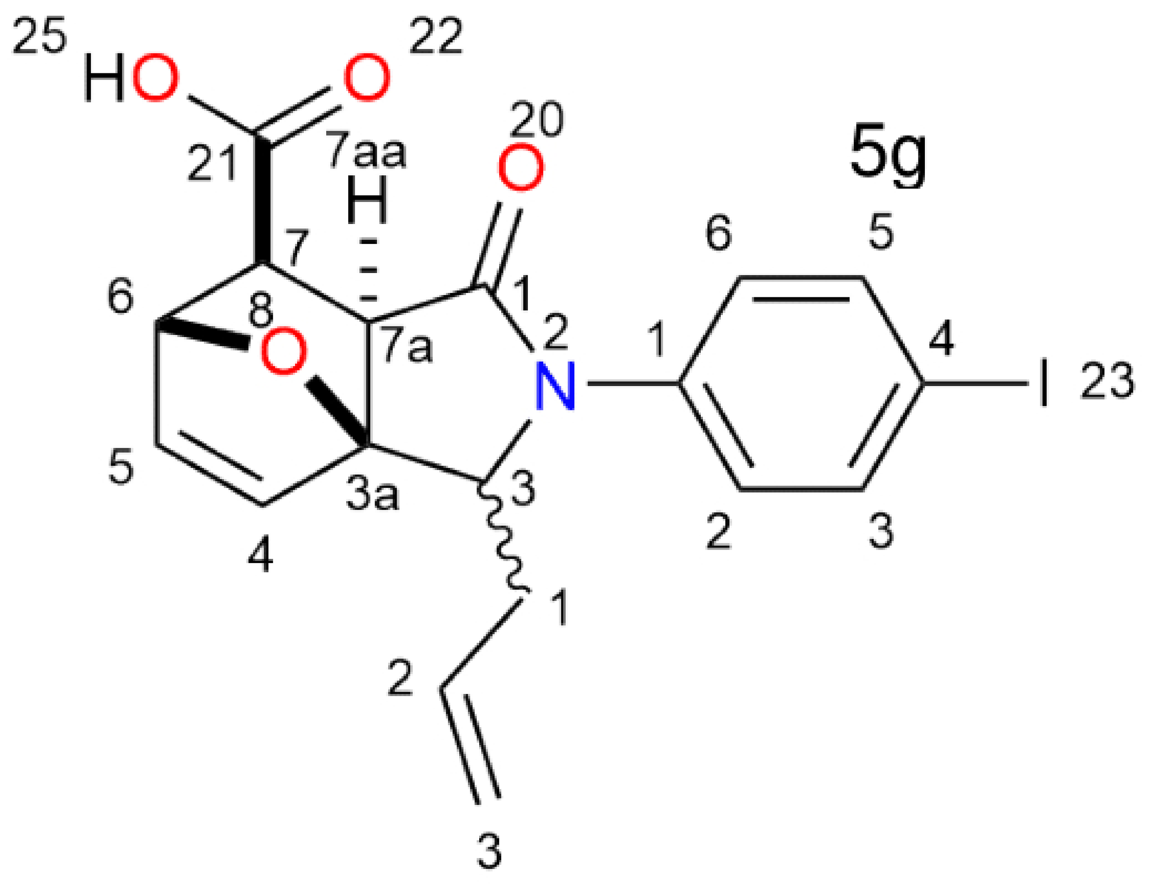

5g

FZ8714-1.JDF

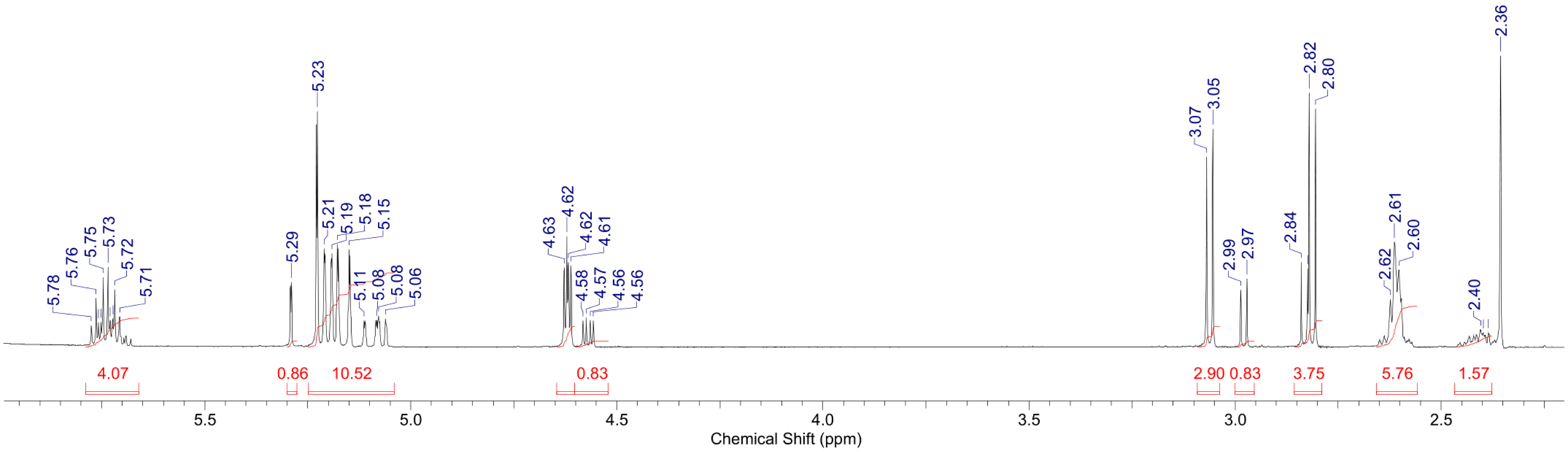

NMR spectrum data for compound 5g (PhMe, Δ, 3 h)

Formula	$C_{18}H_{16}INO_4$	FW	437.2284

**Acquisition Time (sec)**	1.9818	**Comment**	single_pulse	**Date**	07 Jul 2020 11:03:25		
**Date Stamp**	07 Jul 2020 11:04:57			**File Name**	F:\NMR FIDS ACIDS\4-I ALLYL\FZ8738 4.4-I.JDF		
**Frequency (MHz)**	600.17	**Nucleus**	1H	**Number of Transients**	8	**Origin**	ECA 600
**Original Points Count**	32768	**Owner**	CKP	**Points Count**	32768	**Pulse Sequence**	single_pulse.ex2
**Receiver Gain**	42.00	**Solvent**	CHLOROFORM-d			**Spectrum Offset (Hz)**	5408.6089
**Sweep Width (Hz)**	16534.39	**Temperature (degree C)**	23.200				

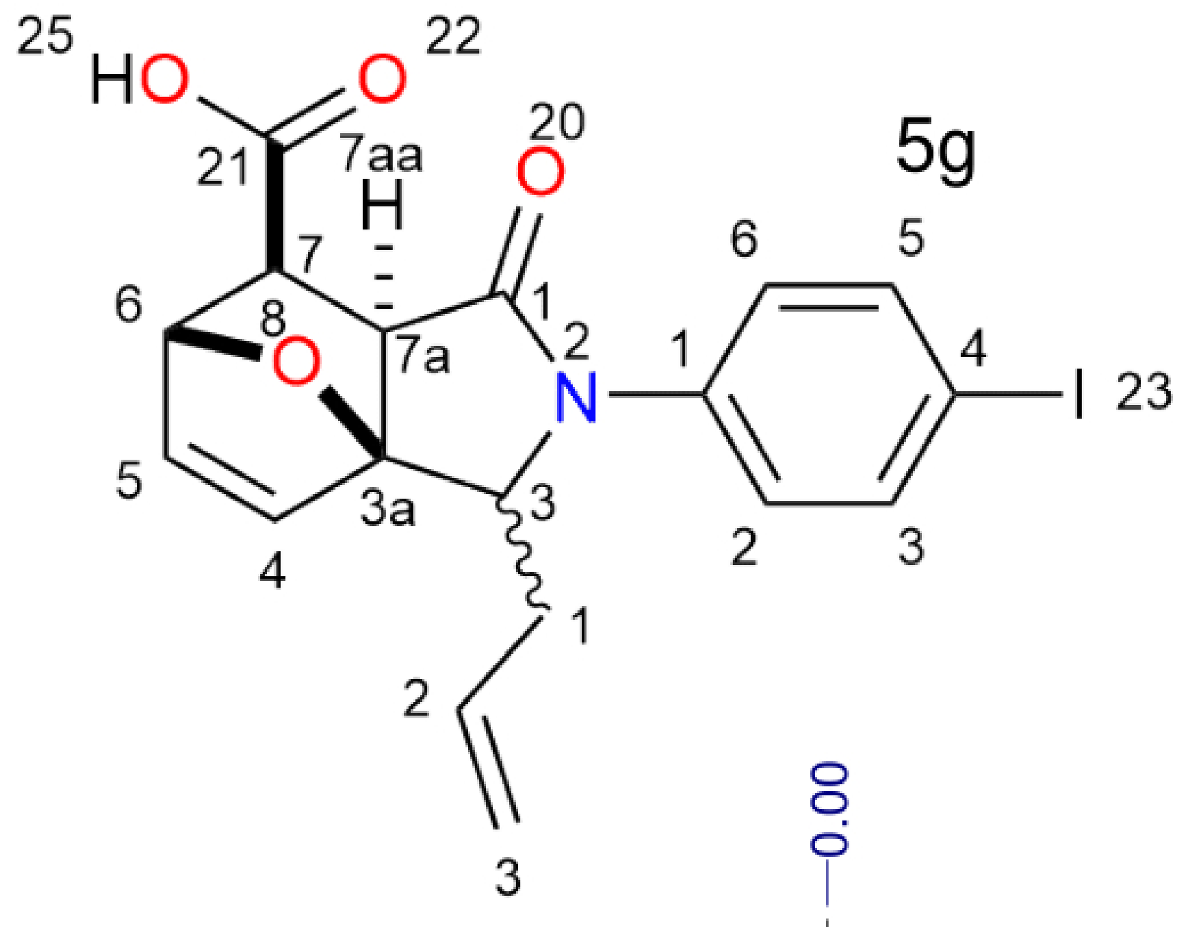

5g

FZ8738 4.4-I.JDF

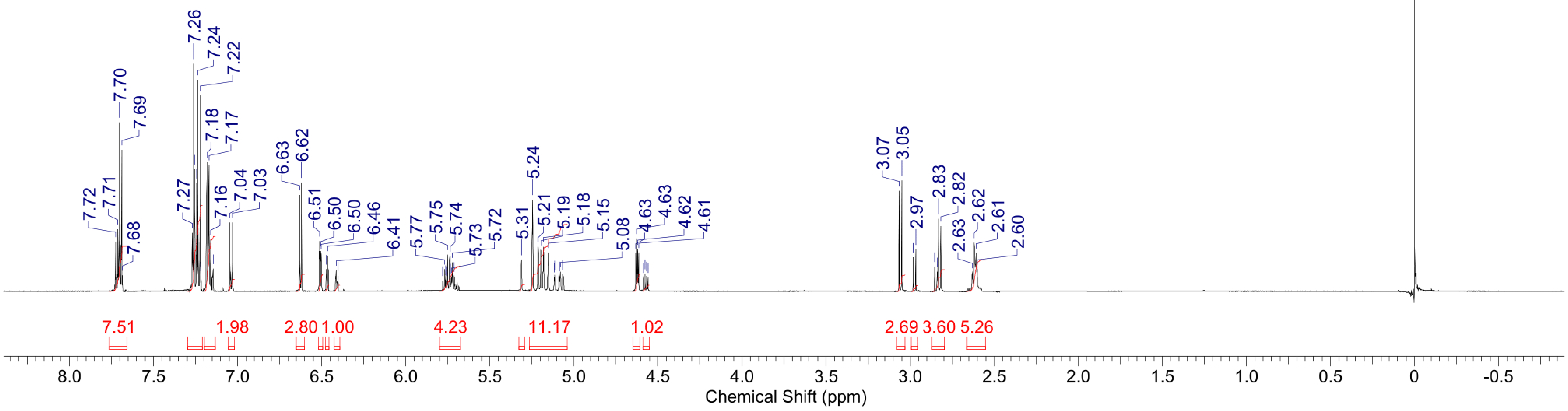

**Formula** $C_{18}H_{16}INO_4$	**FW** 437.2284

**Acquisition Time (sec)**	1.9818	**Comment**	single_pulse	**Date**	07 Jul 2020 11:03:25		
**Date Stamp**	07 Jul 2020 11:04:57			**File Name**	F:\NMR FIDS ACIDS\4-I ALLYL\FZ8738 4.4-I.JDF		
**Frequency (MHz)**	600.17	**Nucleus**	1H	**Number of Transients**	8	**Origin**	ECA 600
**Original Points Count**	32768	**Owner**	CKP	**Points Count**	32768	**Pulse Sequence**	single_pulse.ex2
**Receiver Gain**	42.00	**Solvent**	CHLOROFORM-d			**Spectrum Offset (Hz)**	5408.6089
**Sweep Width (Hz)**	16534.39	**Temperature (degree C)**	23.200				

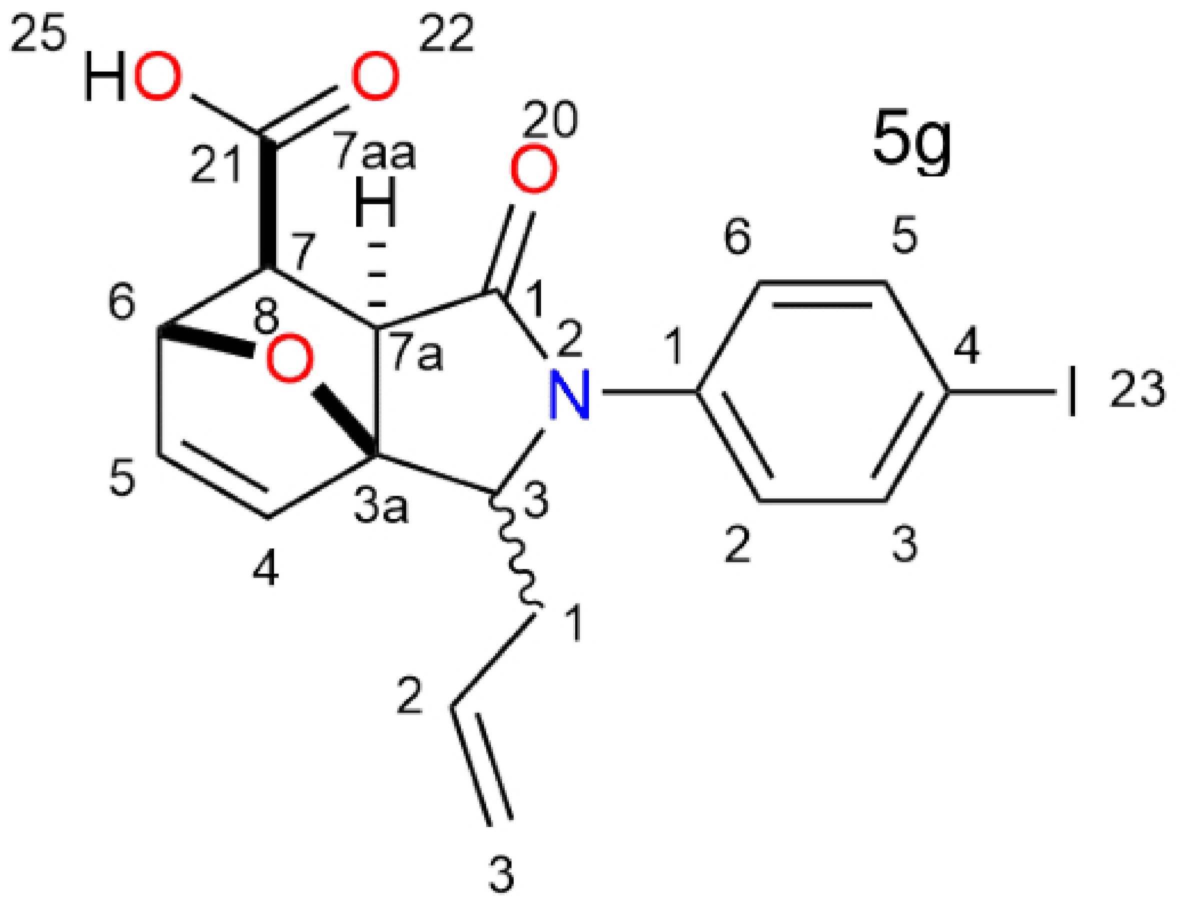

FZ8738 4.4-I.JDF

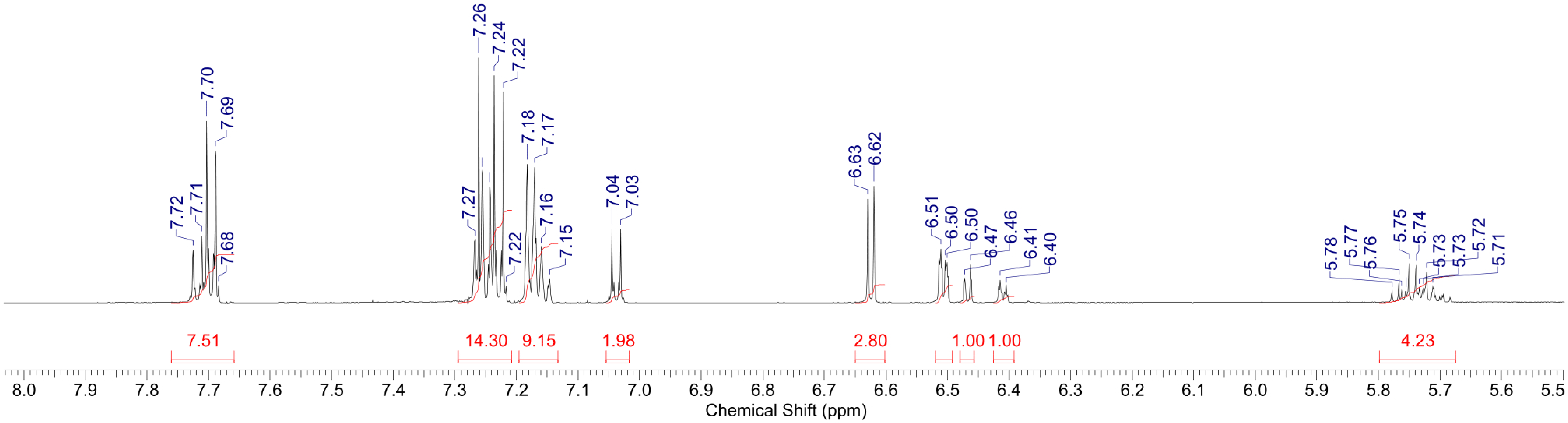

Formula $C_{18}H_{16}INO_4$	FW 437.2284

**Acquisition Time (sec)**	1.9818	**Comment**	single_pulse	**Date**	07 Jul 2020 11:03:25		
**Date Stamp**	07 Jul 2020 11:04:57			**File Name**	F:\NMR FIDS ACIDS\4-I ALLYL\FZ8738 4.4-I.JDF		
**Frequency (MHz)**	600.17	**Nucleus**	1H	**Number of Transients**	8	**Origin**	ECA 600
**Original Points Count**	32768	**Owner**	CKP	**Points Count**	32768	**Pulse Sequence**	single_pulse.ex2
**Receiver Gain**	42.00	**Solvent**	CHLOROFORM-d			**Spectrum Offset (Hz)**	5408.6089
**Sweep Width (Hz)**	16534.39	**Temperature (degree C)**	23.200				

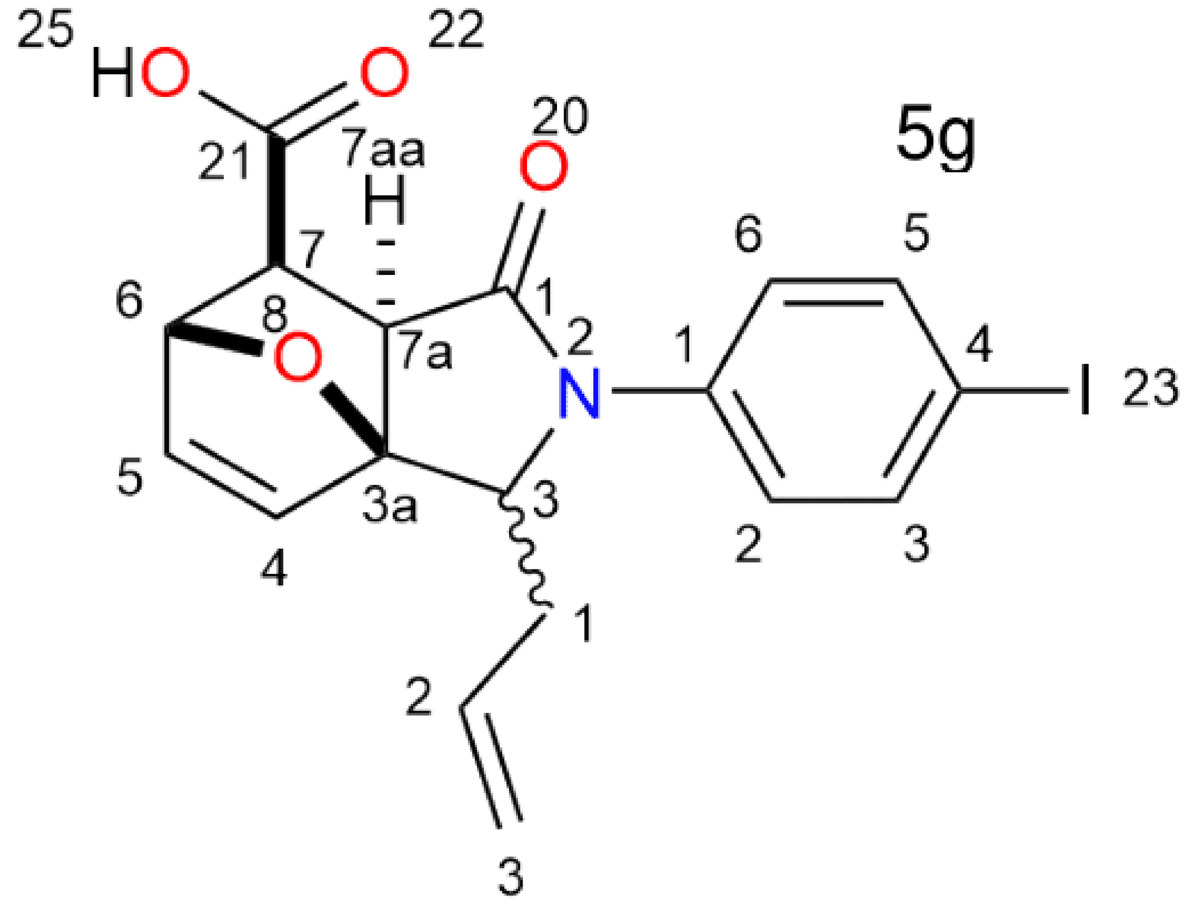

FZ8738 4.4-I.JDF

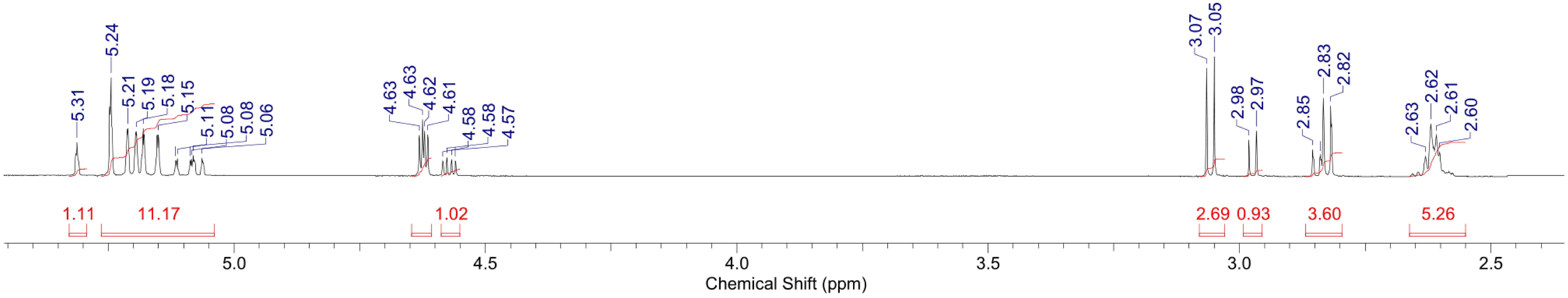

NMR spectrum data for compound 5g

**Formula**	$C_{18}H_{16}INO_4$	**FW**	437.2284

**Acquisition Time (sec)**	0.6921	**Comment**	single pulse decoupled gated NOE	**Date**	28 Aug 1990 08:44:39
**Date Stamp**	07 Nov 2019 14:01:41	**File Name**	C:\Users\User\Desktop\05.11.19\FZ8098-1.jdf		
**Frequency (MHz)**	150.91	**Nucleus**	13C	**Number of Transients**	1000
**Origin**	ECA 600	**Original Points Count**	32768	**Owner**	delta
**Points Count**	32768	**Pulse Sequence**	single_pulse_dec	**Receiver Gain**	58.00
**Solvent**	CHLOROFORM-d	**Spectrum Offset (Hz)**	15079.3525	**Sweep Width (Hz)**	47348.49
**Temperature (degree C)**	23.700				

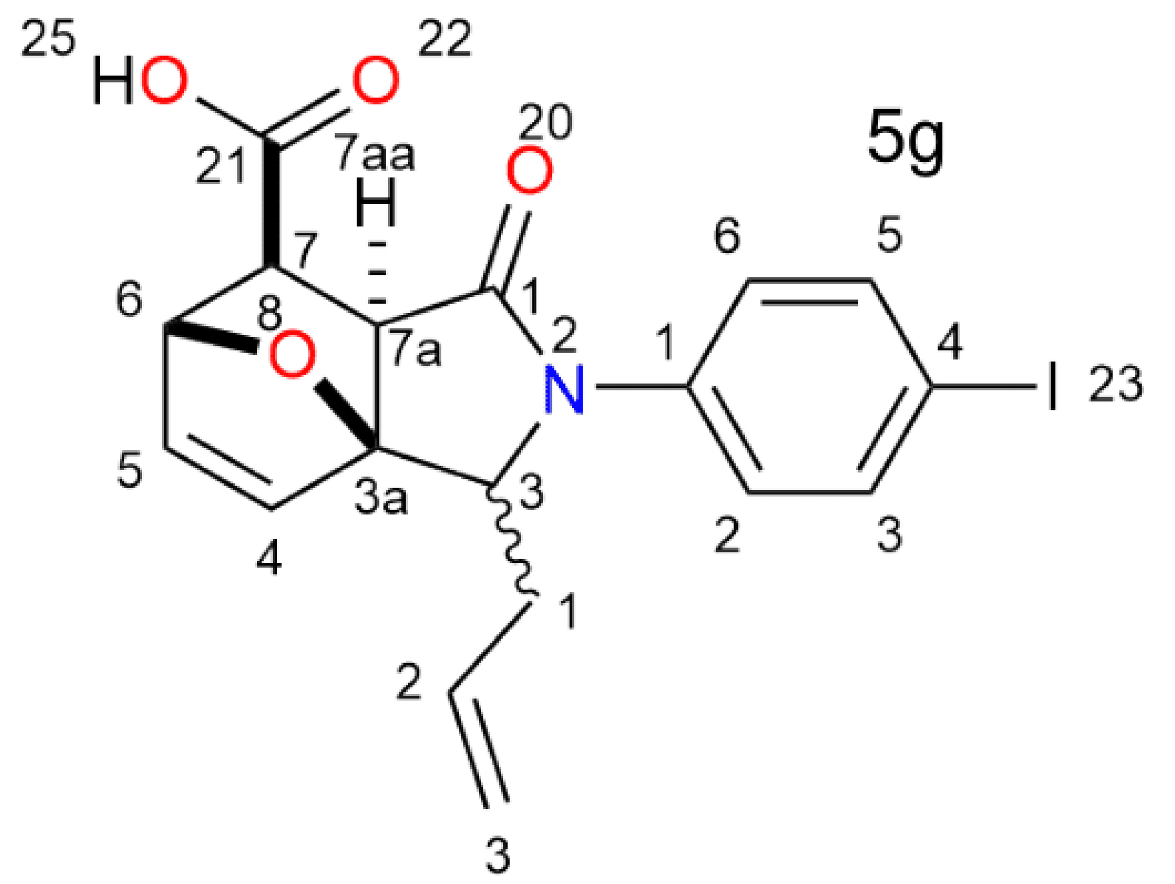

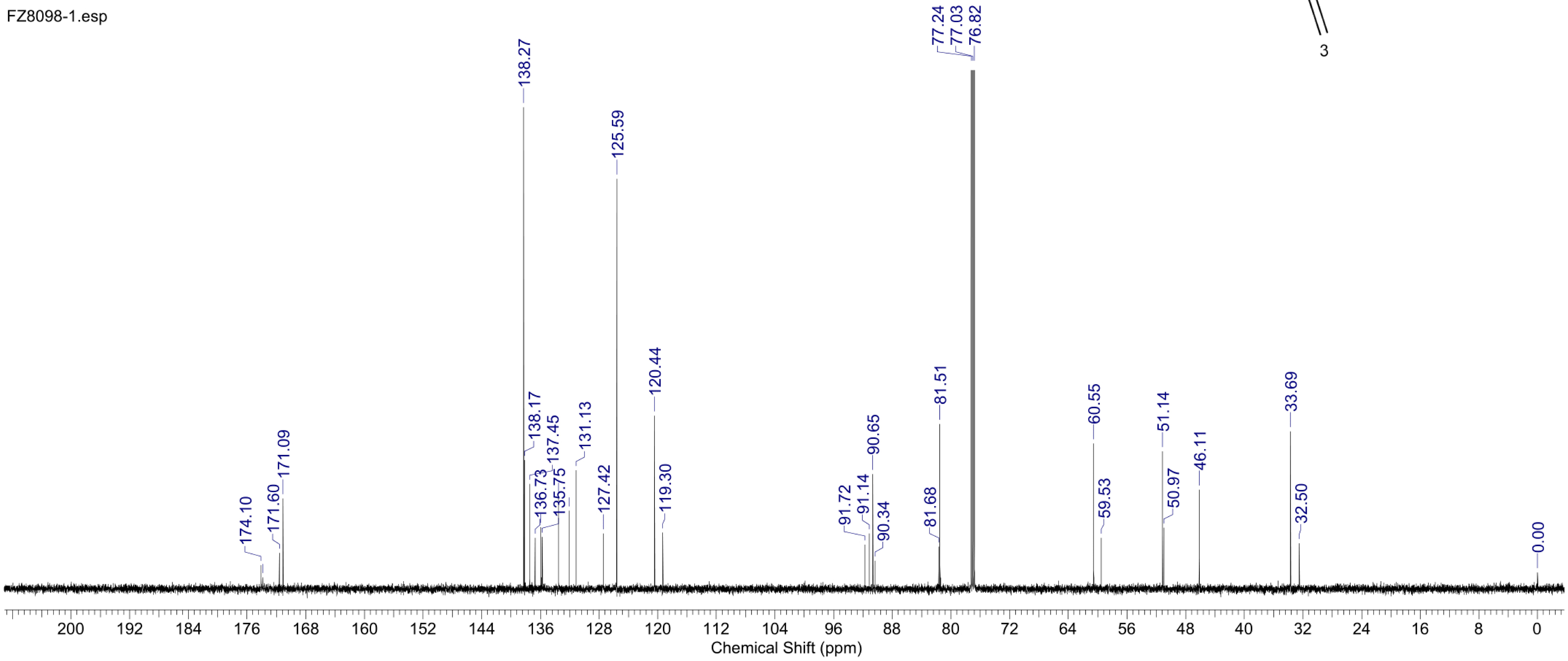

Formula	$C_{18}H_{16}INO_4$	FW	437.2284

**Acquisition Time (sec)**	0.6921	**Comment**	single pulse decoupled gated NOE			**Date**	28 Aug 1990 08:44:39
**Date Stamp**	07 Nov 2019 14:01:41			**File Name**	C:\Users\User\Desktop\05.11.19\FZ8098-1.jdf		
**Frequency (MHz)**	150.91	**Nucleus**	13C	**Number of Transients**	1000	**Origin**	ECA 600
**Original Points Count**	32768	**Owner**	delta	**Points Count**	32768	**Pulse Sequence**	single_pulse_dec
**Receiver Gain**	58.00	**Solvent**	CHLOROFORM-d			**Spectrum Offset (Hz)**	15079.3525
**Sweep Width (Hz)**	47348.49	**Temperature (degree C)**	23.700				

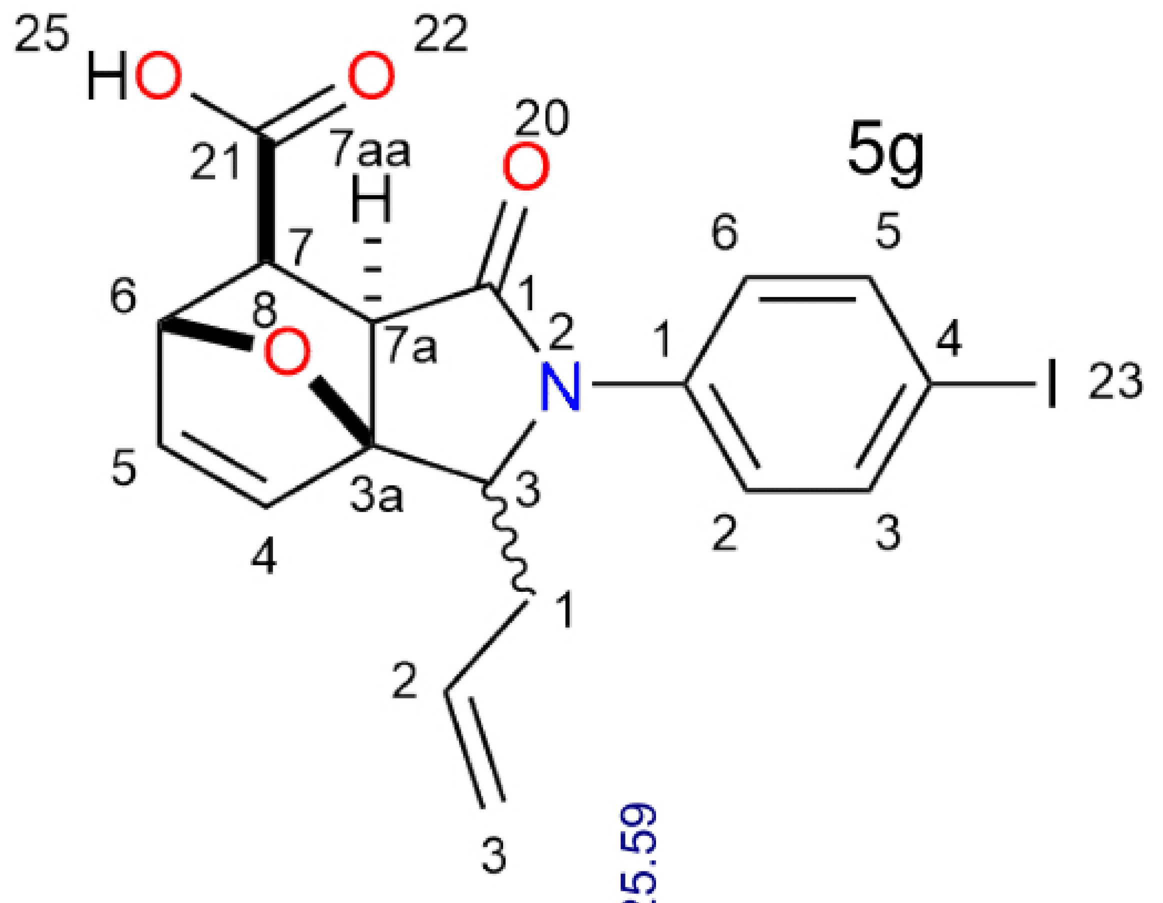

5g

FZ8098-1.esp

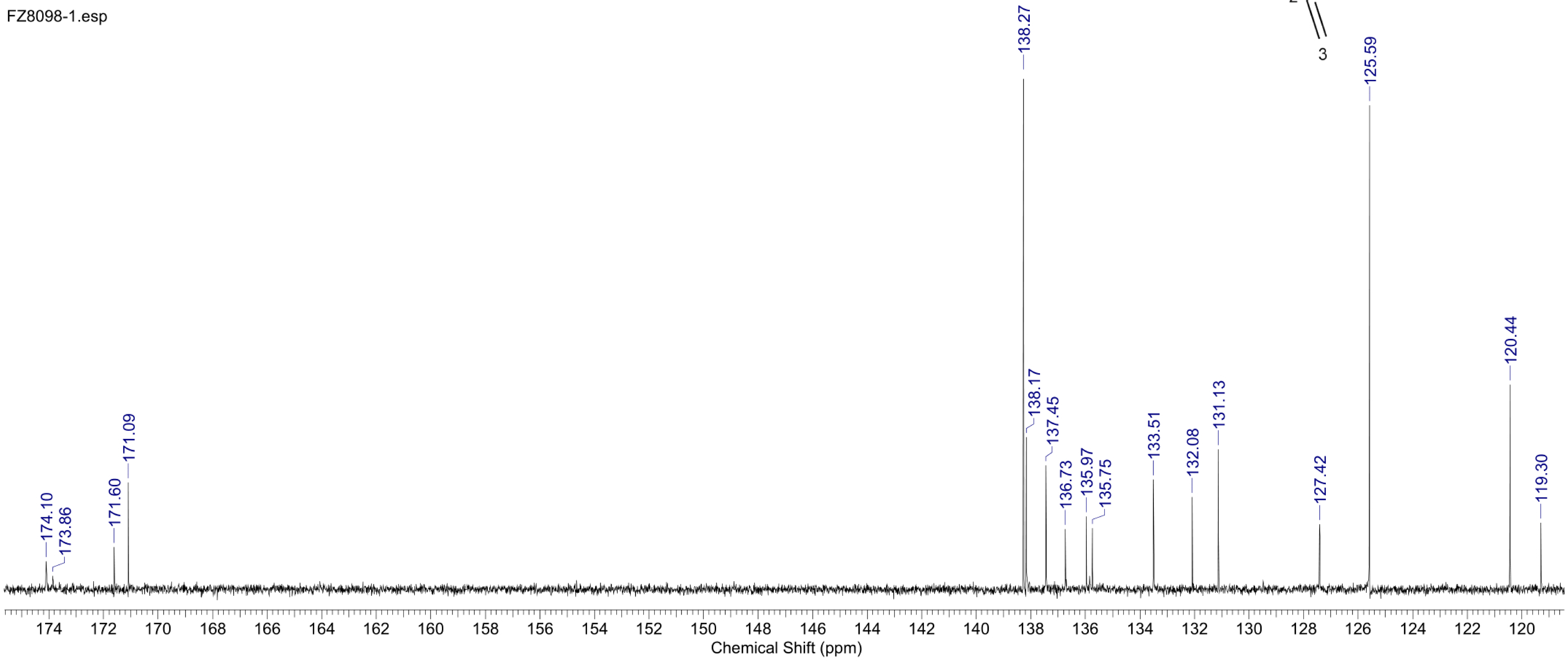

Formula	$C_{18}H_{16}INO_4$	FW	437.2284

Acquisition Time (sec)	0.6921	Comment	single pulse decoupled gated NOE	Date	28 Aug 1990 08:44:39
Date Stamp	07 Nov 2019 14:01:41	File Name	C:\Users\User\Desktop\05.11.19\FZ8098-1.jdf		
Frequency (MHz)	150.91	Nucleus	13C	Number of Transients	1000
Origin	ECA 600				
Original Points Count	32768	Owner	delta	Points Count	32768
Pulse Sequence	single_pulse_dec				
Receiver Gain	58.00	Solvent	CHLOROFORM-d	Spectrum Offset (Hz)	15079.3525
Sweep Width (Hz)	47348.49	Temperature (degree C)	23.700		

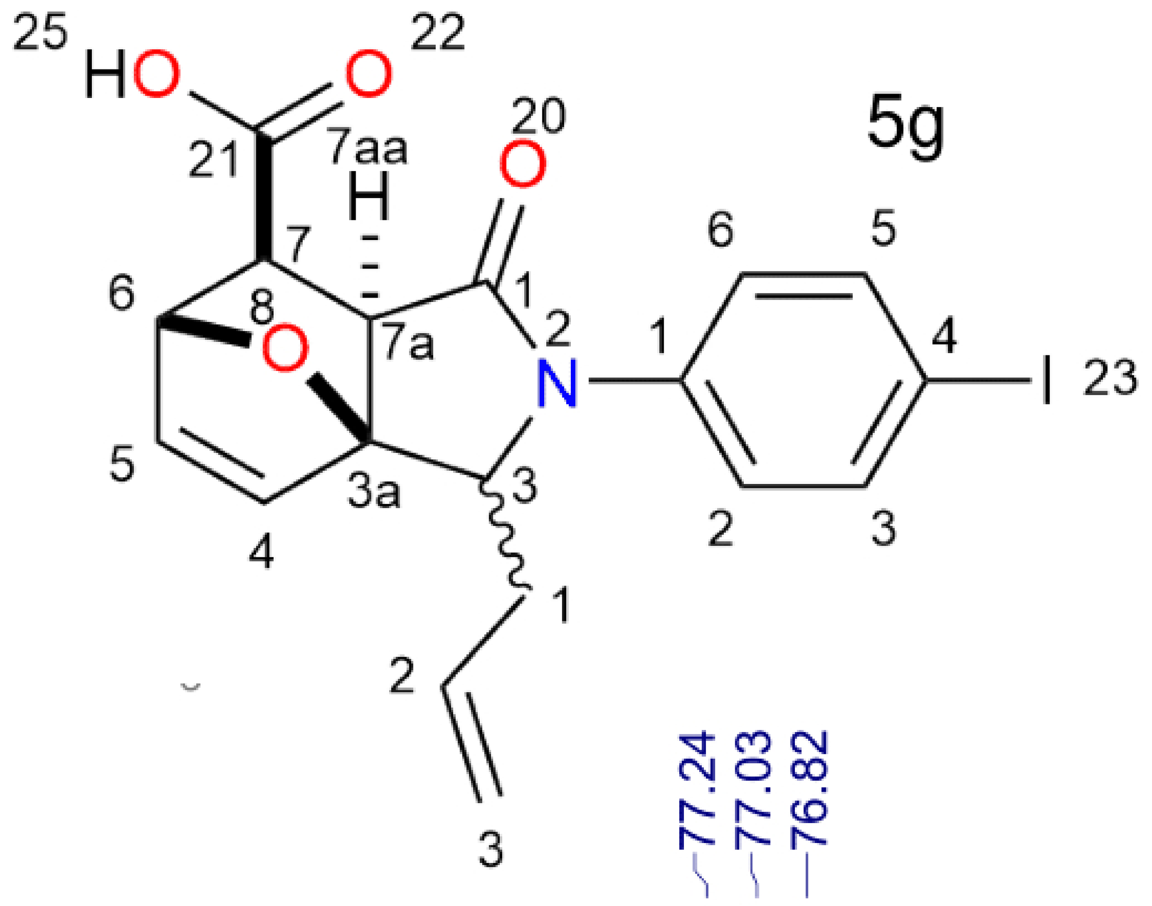

5g

FZ8098-1.esp

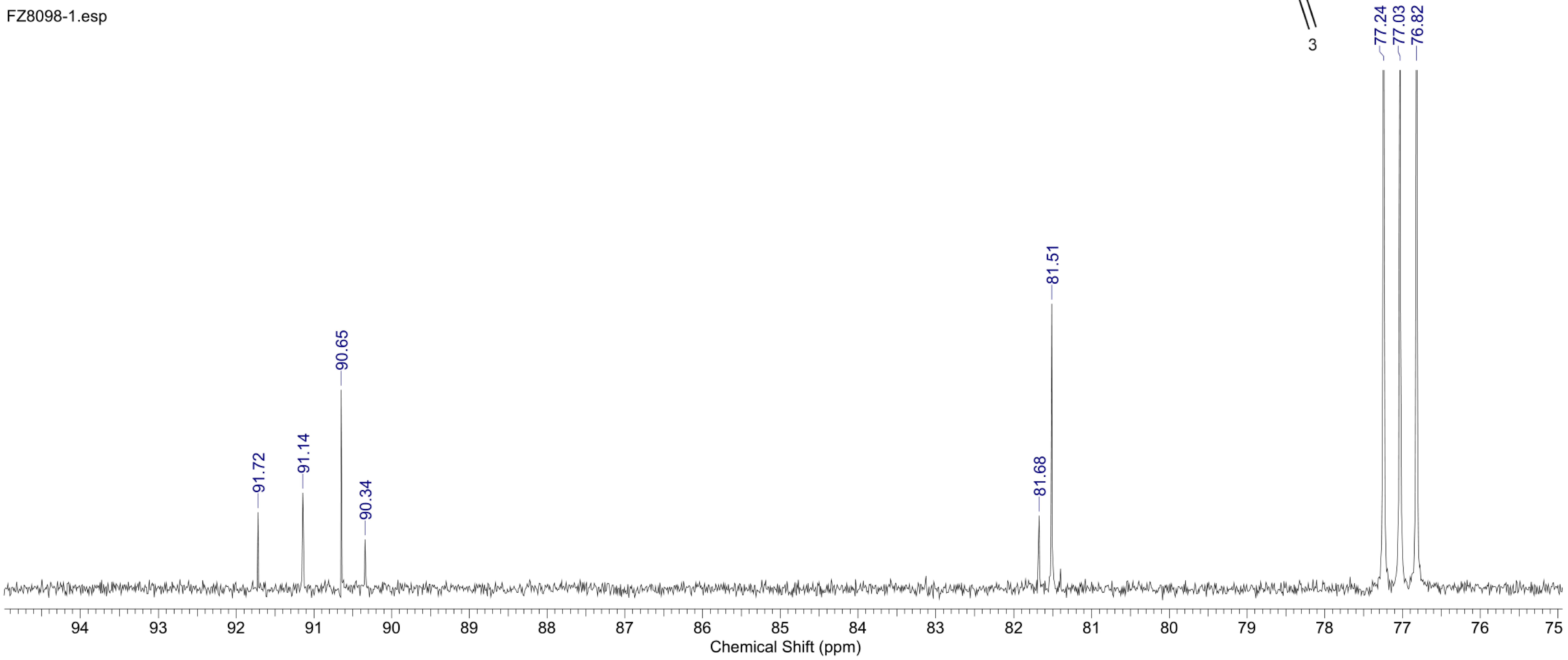

Formula	$C_{18}H_{16}INO_4$	FW	437.2284

**Acquisition Time (sec)**	0.6921	**Comment**	single pulse decoupled gated NOE			**Date**	28 Aug 1990 08:44:39
**Date Stamp**	07 Nov 2019 14:01:41			**File Name**	C:\Users\User\Desktop\05.11.19\FZ8098-1.jdf		
**Frequency (MHz)**	150.91	**Nucleus**	13C	**Number of Transients**	1000	**Origin**	ECA 600
**Original Points Count**	32768	**Owner**	delta	**Points Count**	32768	**Pulse Sequence**	single_pulse_dec
**Receiver Gain**	58.00	**Solvent**	CHLOROFORM-d			**Spectrum Offset (Hz)**	15079.3525
**Sweep Width (Hz)**	47348.49	**Temperature (degree C)**	23.700				

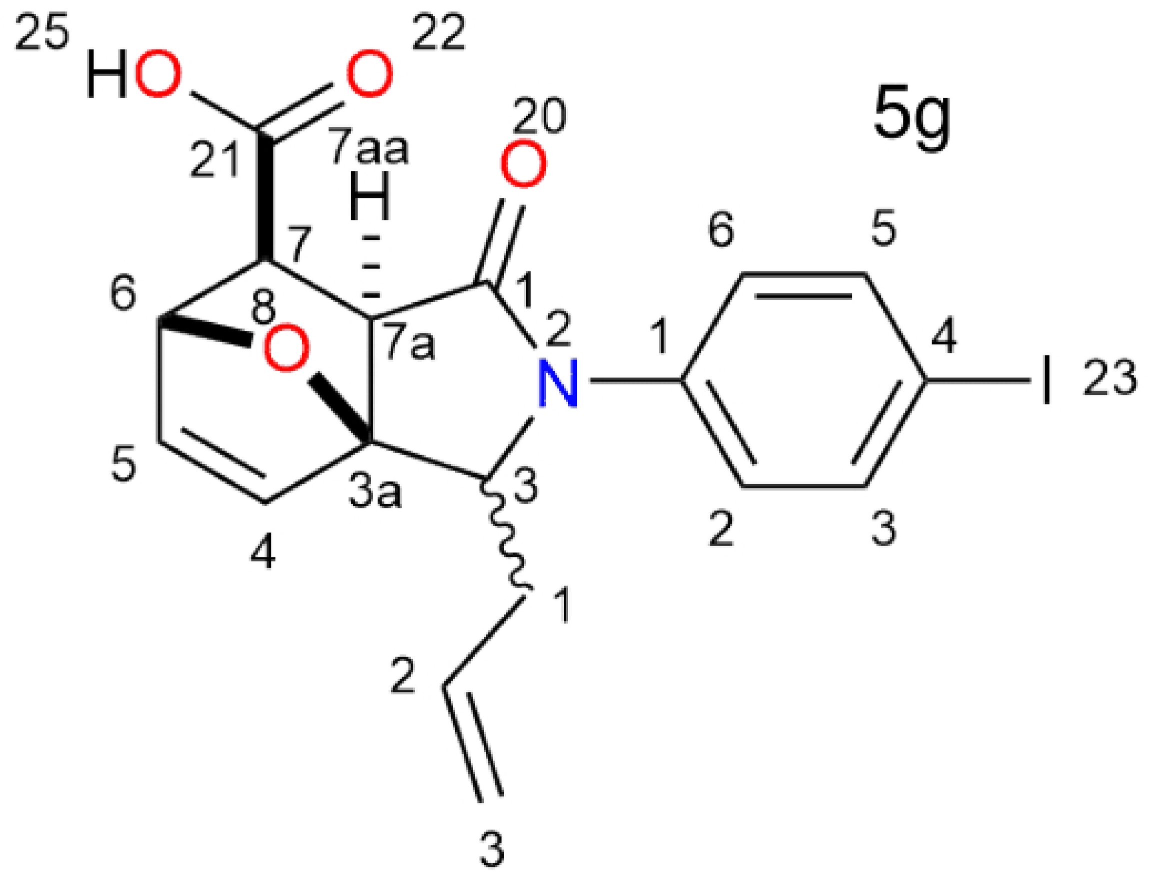

5g

FZ8098-1.esp

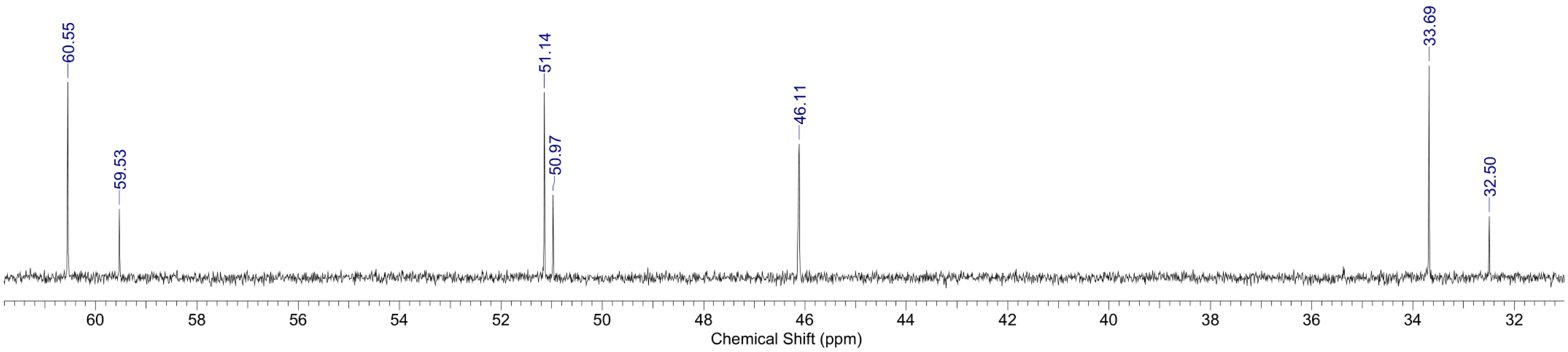

NMR spectrum data for compound 5i (DCM, −16 °C, 3 d)

Formula $C_{19}H_{19}NO_4$	FW 325.3585

Acquisition Time (sec)	1.9818	***Comment***	single_pulse	***Date***	17 Jul 2020 11:27:33		
Date Stamp	17 Jul 2020 11:29:21			***File Name***	F:\NMR FIDS ACIDS\PH METHALLYL\FZ8818-1.JDF		
Frequency (MHz)	600.17	***Nucleus***	1H	***Number of Transients***	8	***Origin***	ECA 600
Original Points Count	32768	***Owner***	CKP	***Points Count***	32768	***Pulse Sequence***	single_pulse.ex2
Receiver Gain	50.00	***Solvent***	CHLOROFORM-d			***Spectrum Offset (Hz)***	5409.6182
Sweep Width (Hz)	16534.39						

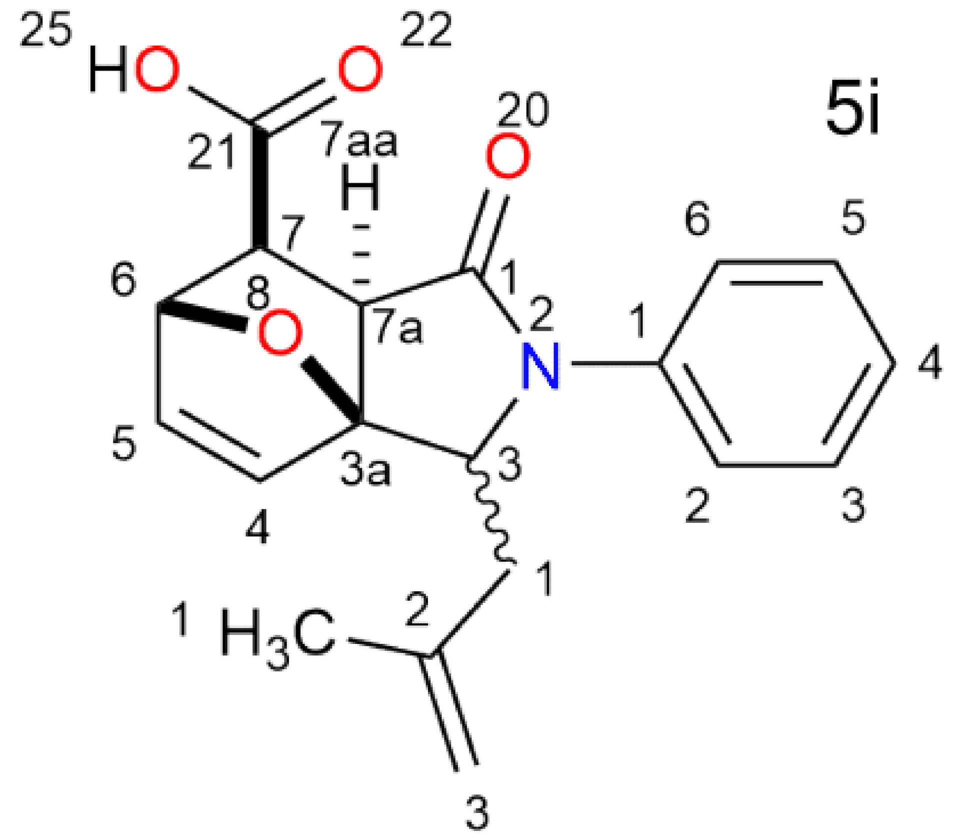

5i

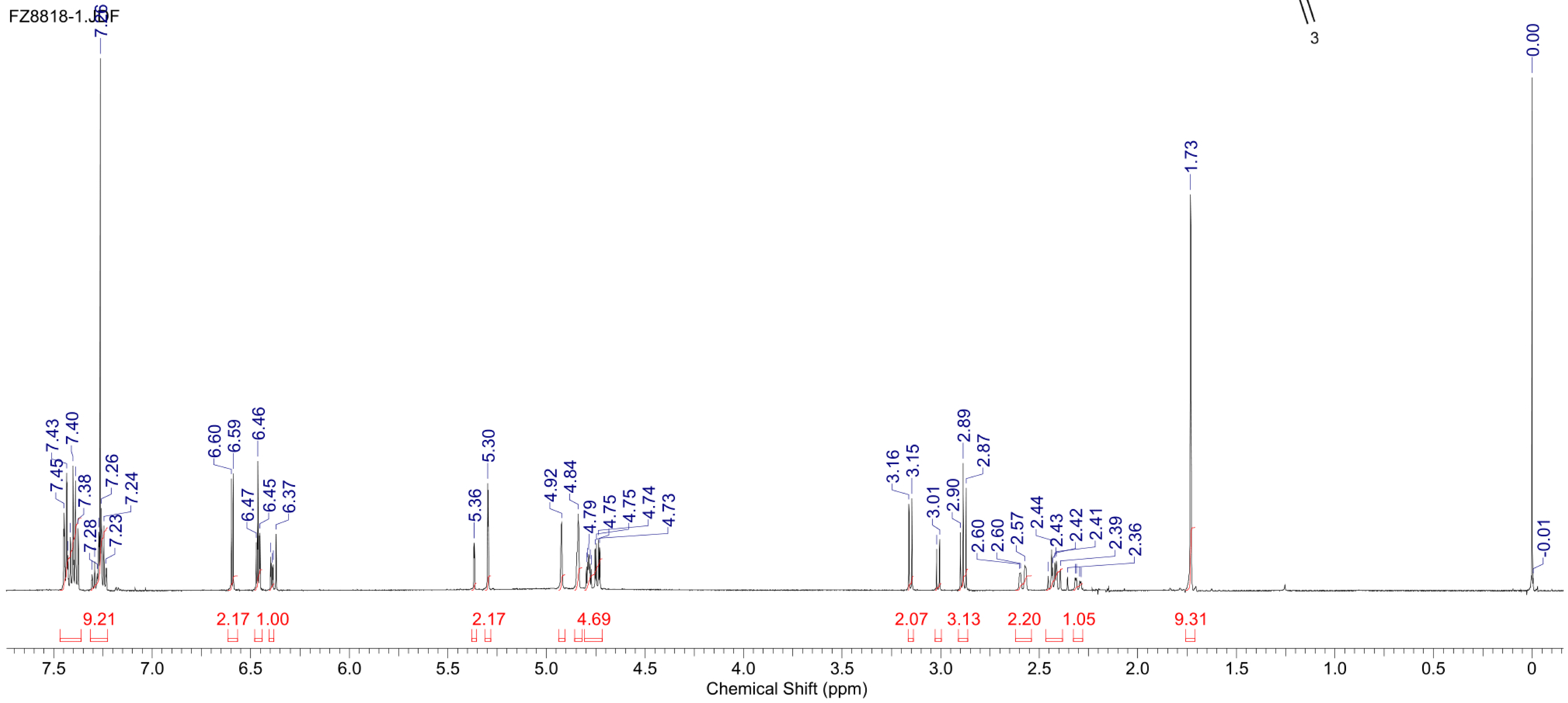

**Formula**	$C_{19}H_{19}NO_4$	**FW**	325.3585

**Acquisition Time (sec)**	1.9818	**Comment**	single_pulse	**Date**	17 Jul 2020 11:27:33		
**Date Stamp**	17 Jul 2020 11:29:21			**File Name**	F:\NMR FIDS ACIDS\PH METHALLYL\FZ8818-1.JDF		
**Frequency (MHz)**	600.17	**Nucleus**	1H	**Number of Transients**	8	**Origin**	ECA 600
**Original Points Count**	32768	**Owner**	CKP	**Points Count**	32768	**Pulse Sequence**	single_pulse.ex2
**Receiver Gain**	50.00	**Solvent**	CHLOROFORM-d			**Spectrum Offset (Hz)**	5409.6182
**Sweep Width (Hz)**	16534.39						

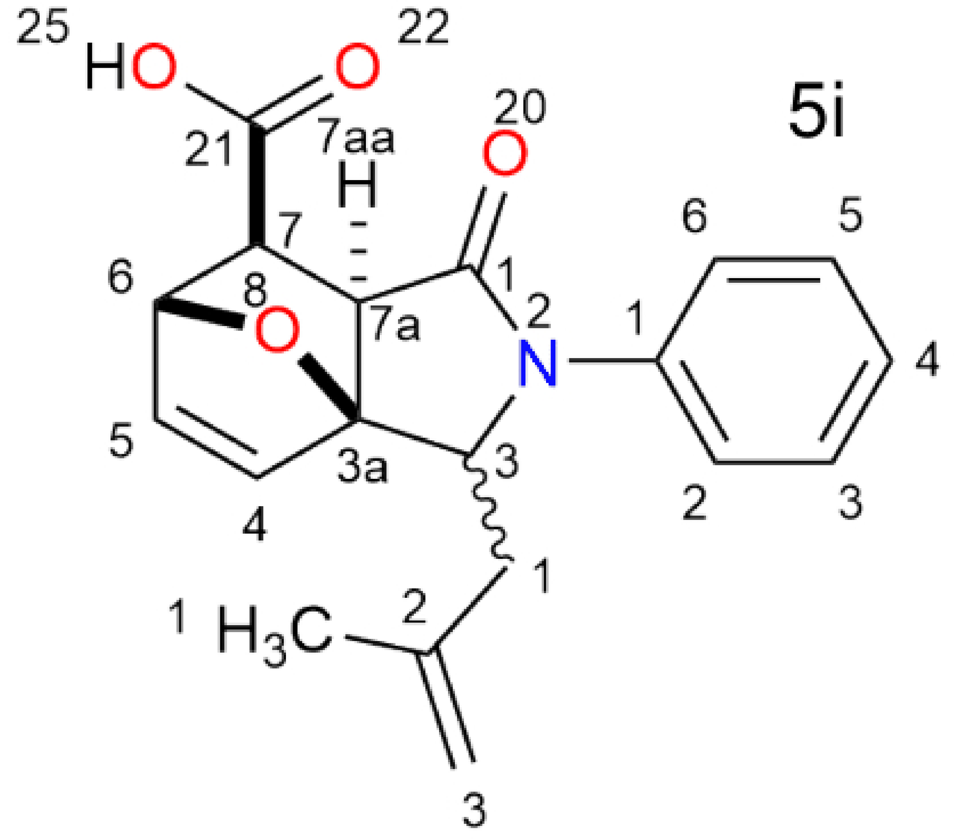

5i

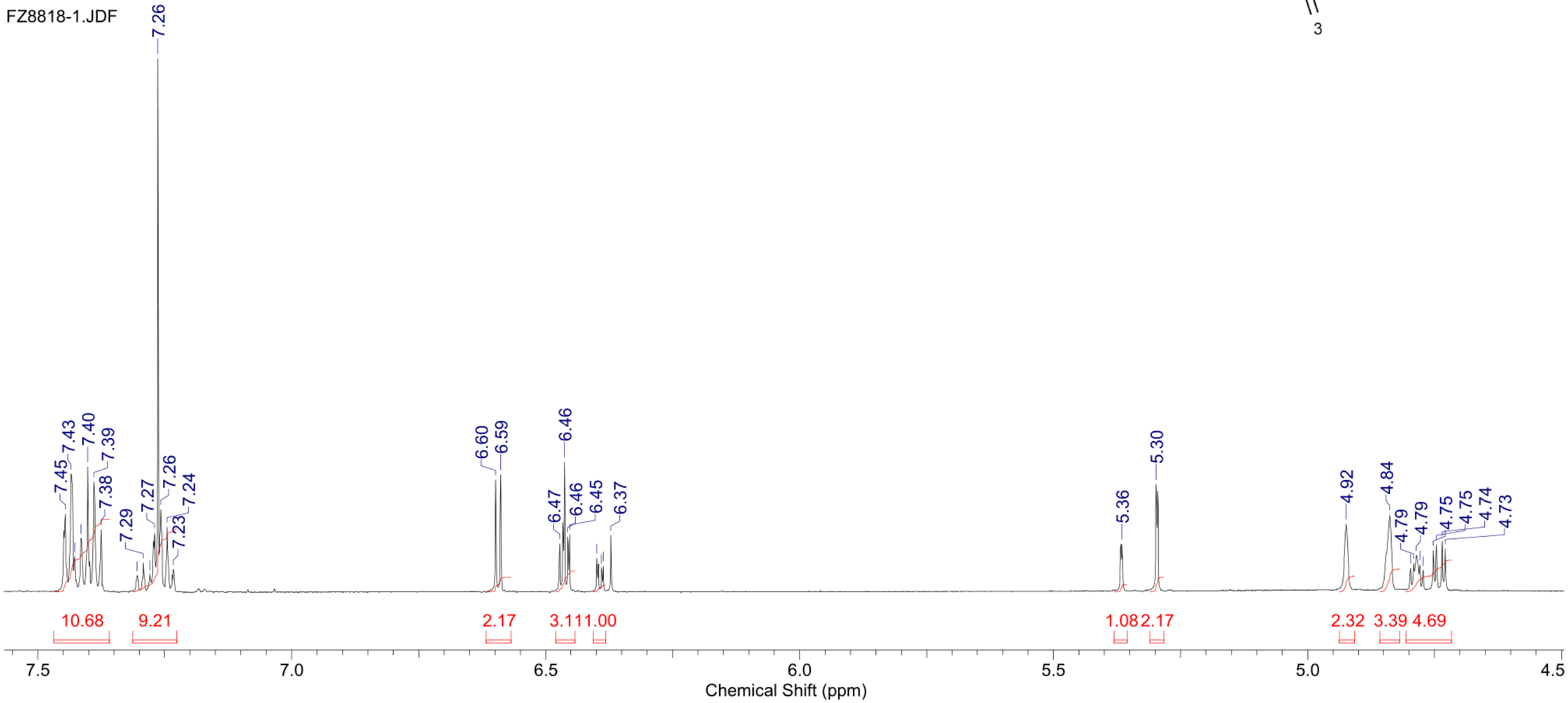

**Formula** $C_{19}H_{19}NO_4$	**FW** 325.3585

**Acquisition Time (sec)**	1.9818	**Comment**	single_pulse	**Date**	17 Jul 2020 11:27:33		
**Date Stamp**	17 Jul 2020 11:29:21			**File Name**	F:\NMR FIDS ACIDS\PH METHALLYL\FZ8818-1.JDF		
**Frequency (MHz)**	600.17	**Nucleus**	1H	**Number of Transients**	8	**Origin**	ECA 600
**Original Points Count**	32768	**Owner**	CKP	**Points Count**	32768	**Pulse Sequence**	single_pulse.ex2
**Receiver Gain**	50.00	**Solvent**	CHLOROFORM-d			**Spectrum Offset (Hz)**	5409.6182
**Sweep Width (Hz)**	16534.39						

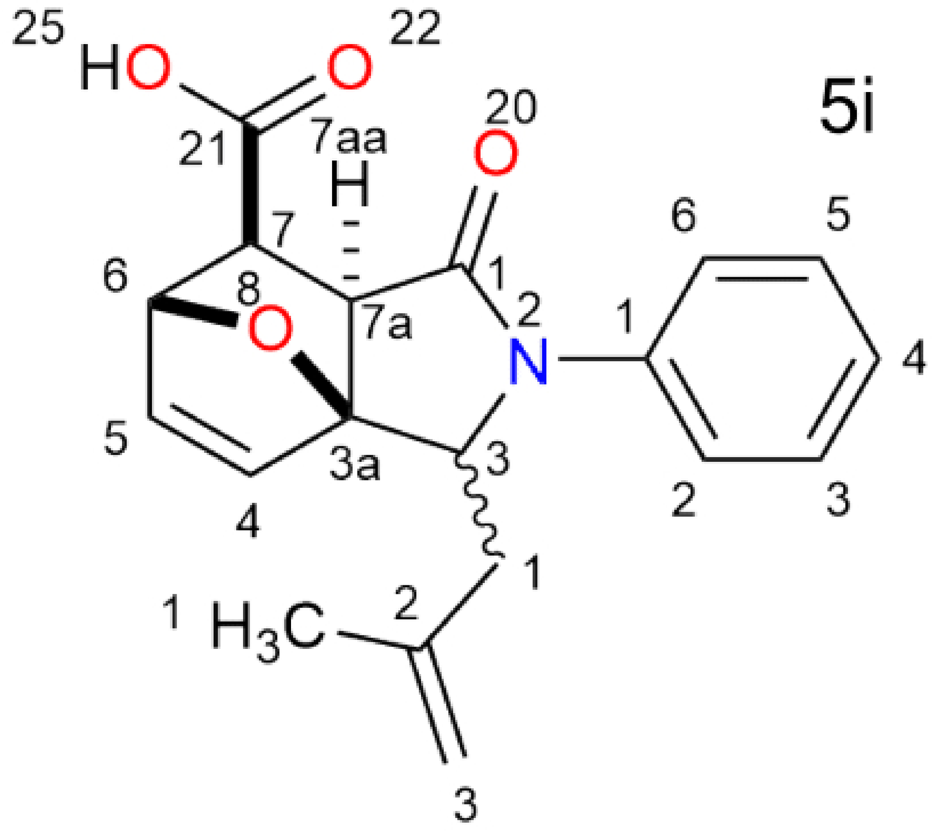

FZ8818-1.JDF

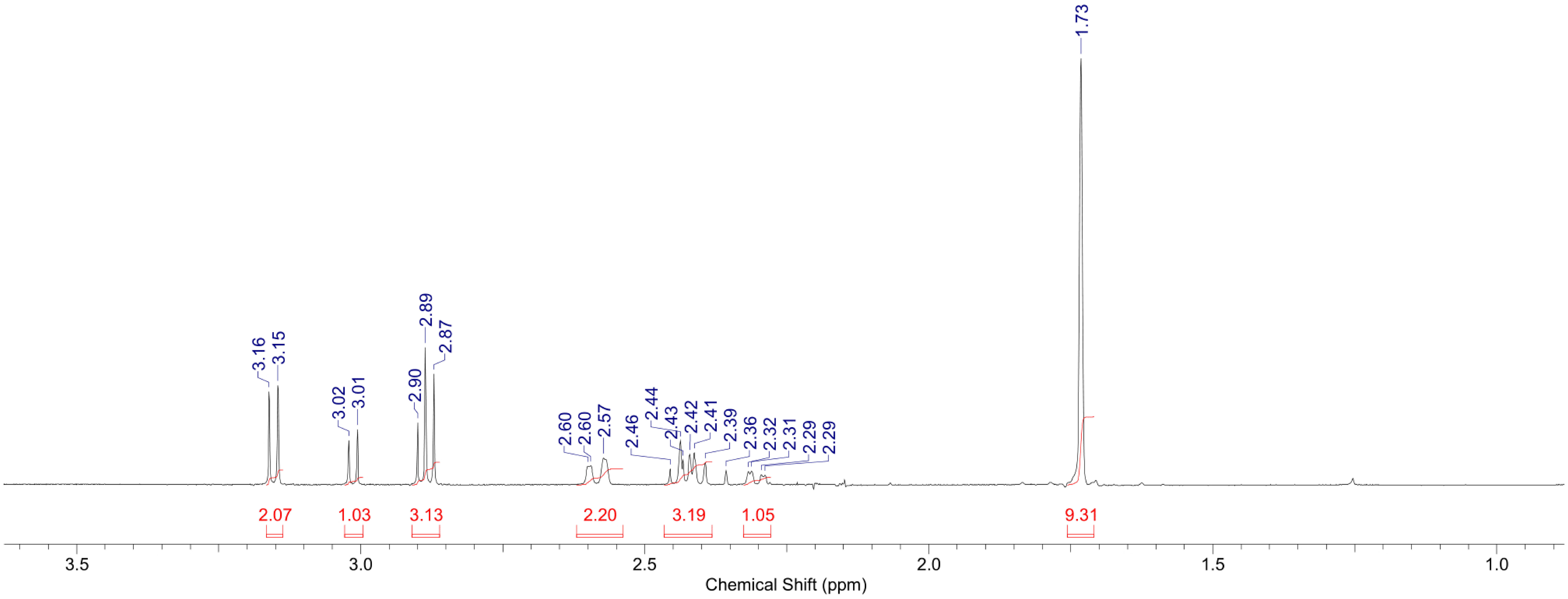

NMR spectrum data for compound 5i (DCM, r.t., 3 d)

Acquisition Time (sec)	1.9818	Comment	single_pulse	Date	28 Jul 2020 11:19:58			Date Stamp	28 Jul 2020 11:21:11
File Name	C:\USERS\FEDOR\DOWNLOADS\FZ8886-1.JDF			Frequency (MHz)	600.17	Nucleus	1H	Number of Transients	8
Origin	ECA 600	Original Points Count	32768	Owner	CKP	Points Count	32768	Pulse Sequence	single_pulse.ex2
Receiver Gain	48.00	Solvent	CHLOROFORM-d			Spectrum Offset (Hz)	5409.1133	Sweep Width (Hz)	16534.39

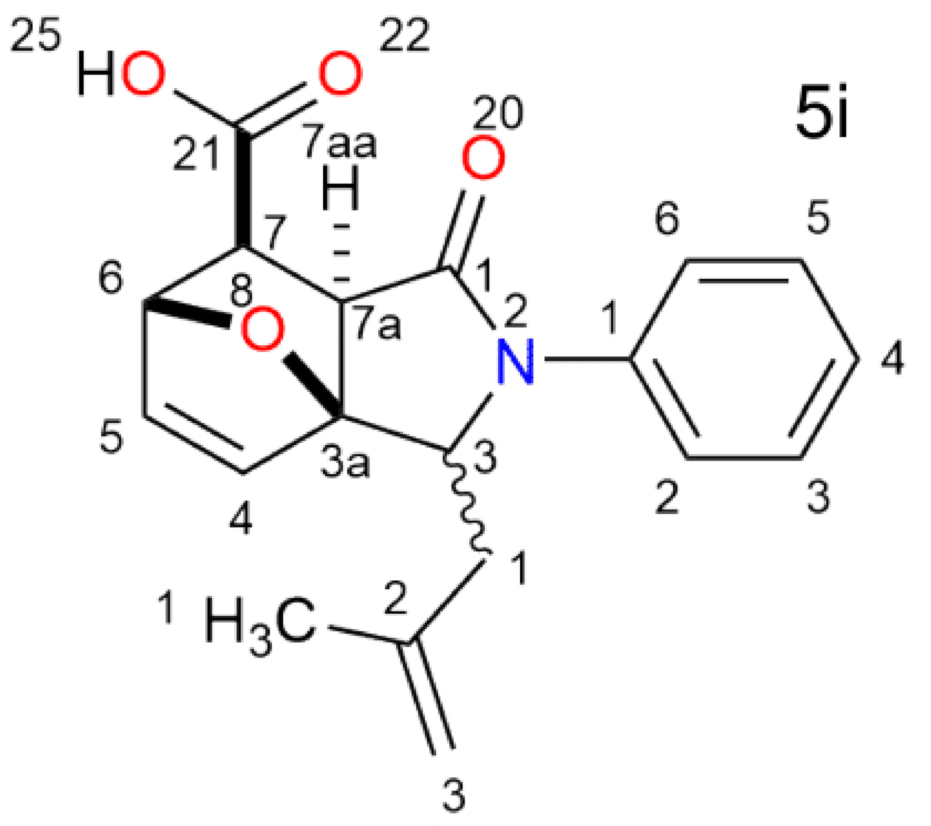

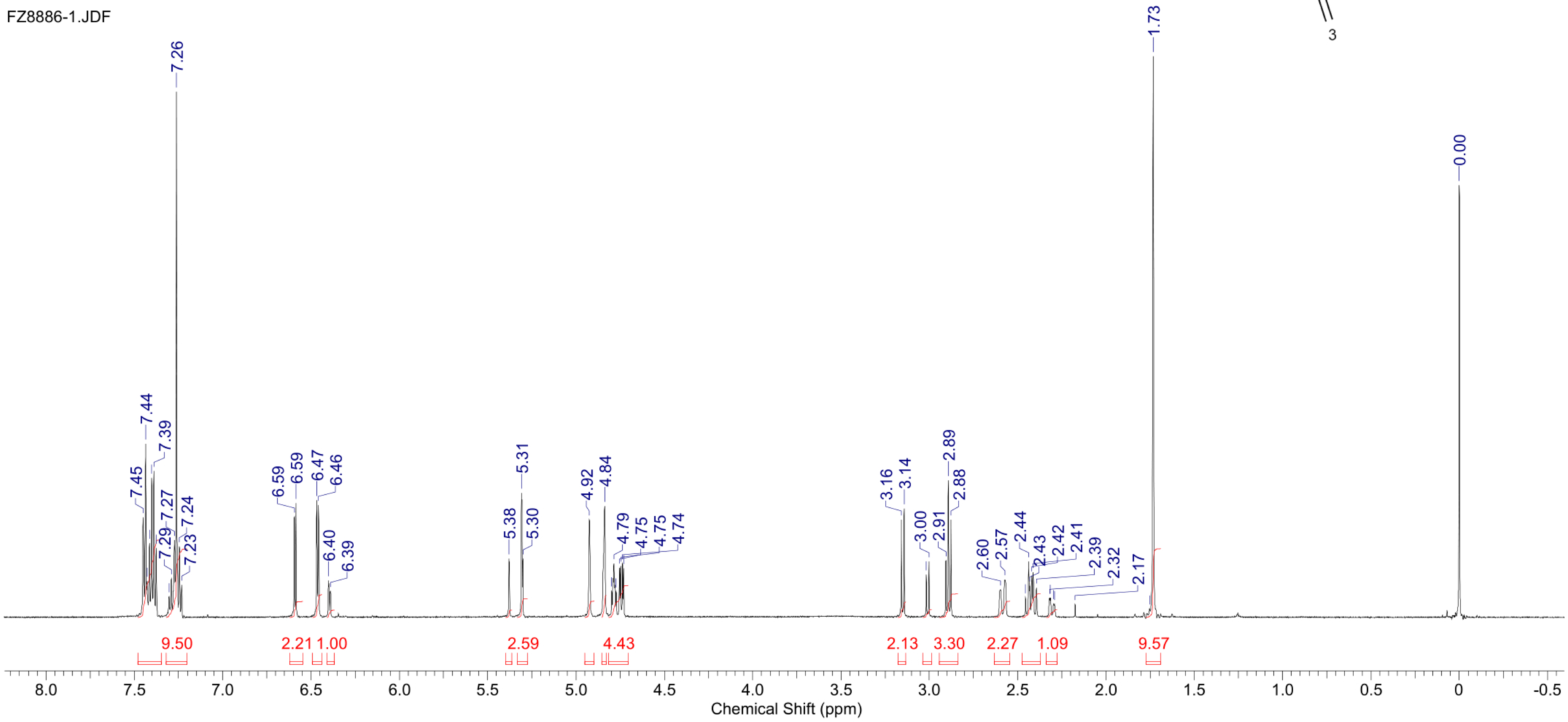

Acquisition Time (sec)	1.9818	***Comment***	single_pulse	***Date***	28 Jul 2020 11:19:58			***Date Stamp***	28 Jul 2020 11:21:11
File Name	C:\USERS\FEDOR\DOWNLOADS\FZ8886-1.JDF			***Frequency (MHz)***	600.17	***Nucleus***	1H	***Number of Transients***	8
Origin	ECA 600	***Original Points Count***	32768	***Owner***	CKP	***Points Count***	32768	***Pulse Sequence***	single_pulse.ex2
Receiver Gain	48.00	***Solvent***	CHLOROFORM-d			***Spectrum Offset (Hz)***	5409.1133	***Sweep Width (Hz)***	16534.39

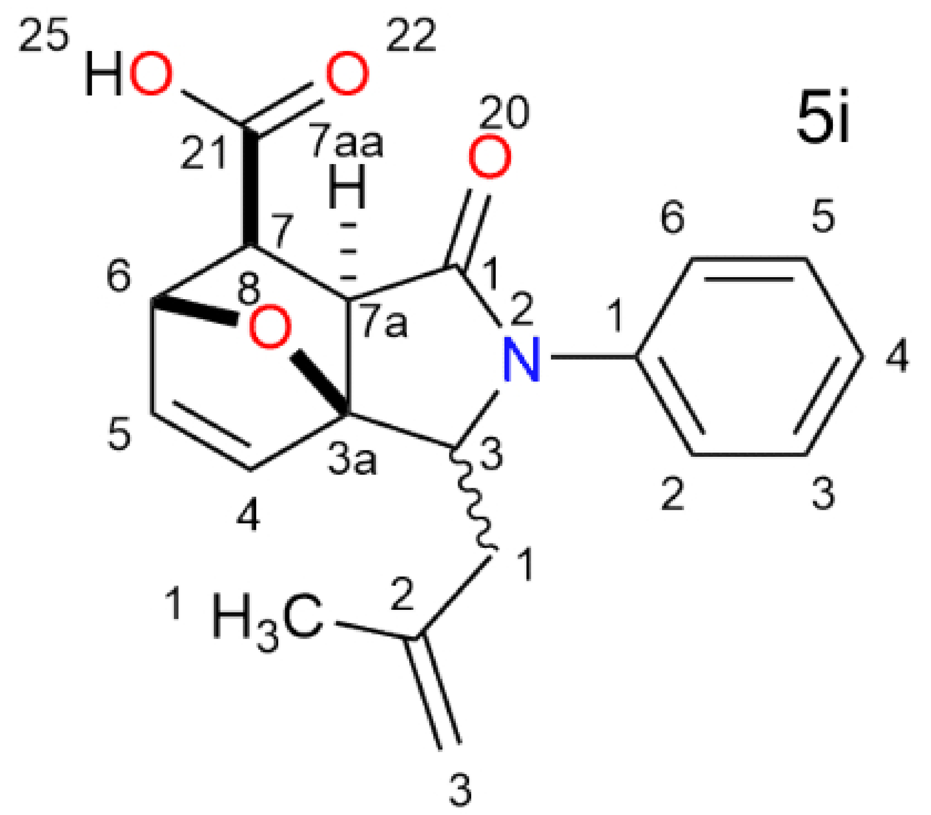

5i

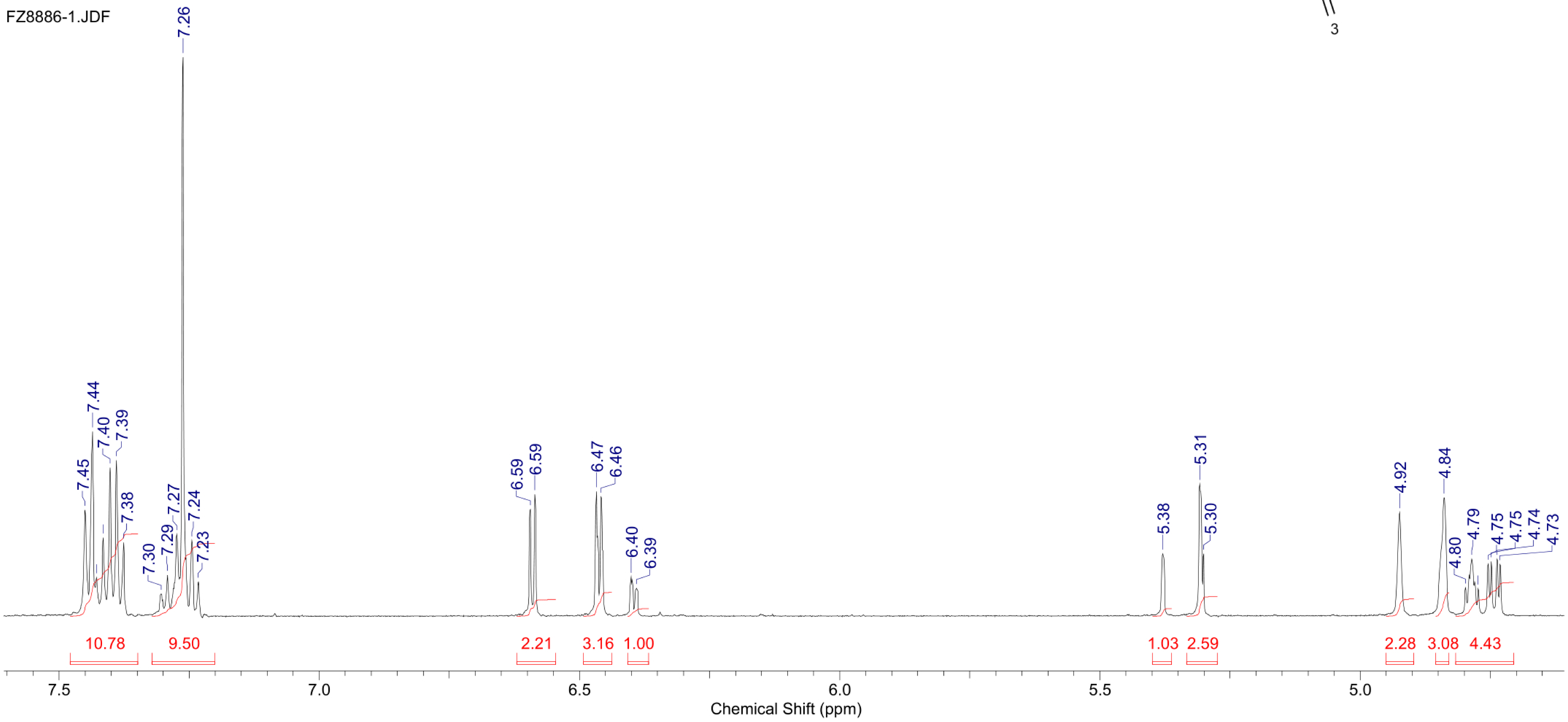

Acquisition Time (sec)	1.9818	***Comment***	single_pulse	***Date***	28 Jul 2020 11:19:58			***Date Stamp***	28 Jul 2020 11:21:11
File Name	C:\USERS\FEDOR\DOWNLOADS\FZ8886-1.JDF			***Frequency (MHz)***	600.17	***Nucleus***	1H	***Number of Transients***	8
Origin	ECA 600	***Original Points Count***	32768	***Owner***	CKP	***Points Count***	32768	***Pulse Sequence***	single_pulse.ex2
Receiver Gain	48.00	***Solvent***	CHLOROFORM-d			***Spectrum Offset (Hz)***	5409.1133	***Sweep Width (Hz)***	16534.39

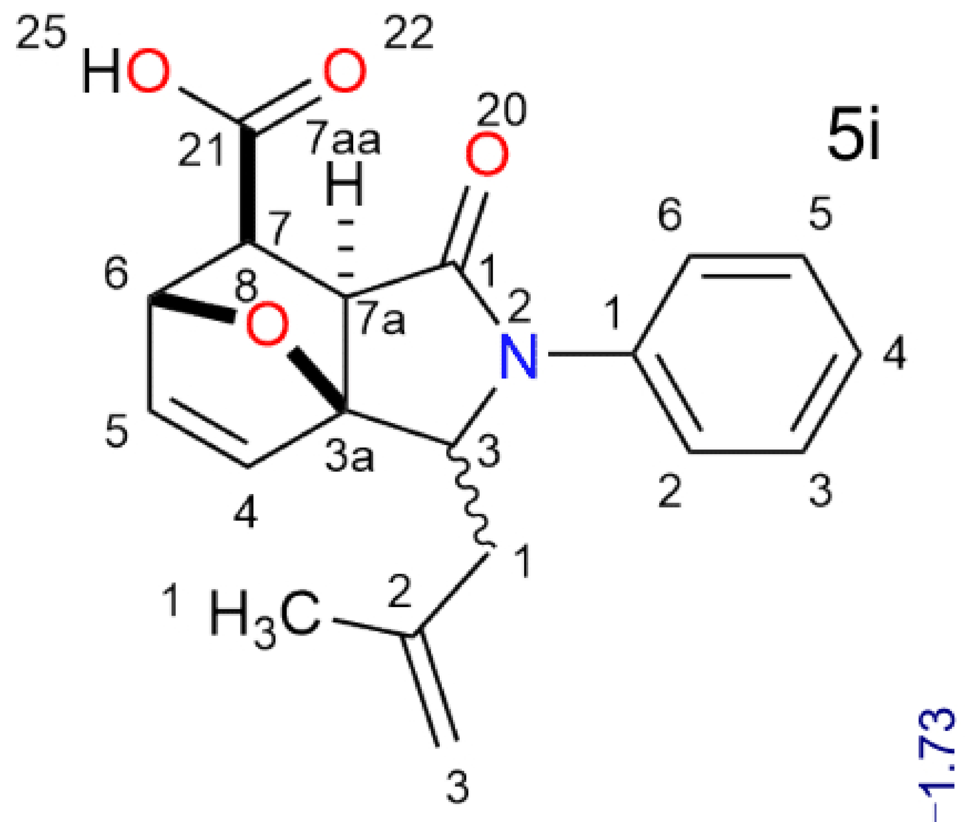

5i

FZ8886-1.JDF

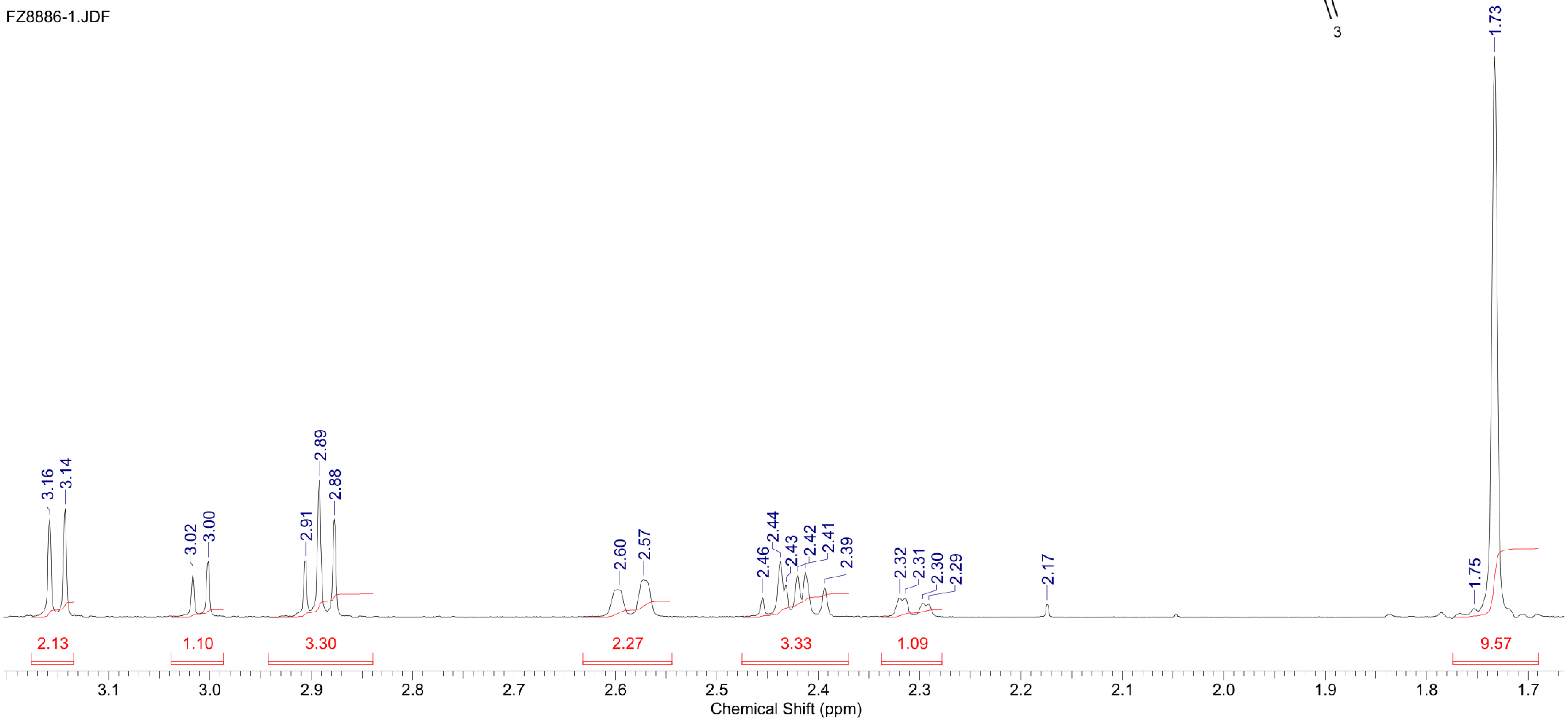

NMR spectrum data for compound 5i (MeCN, r.t., 3 d)

**Formula** $C_{19}H_{19}NO_4$	**FW** 325.3585

Acquisition Time (sec)	1.9818	***Comment***	single_pulse	***Date***	17 Jul 2020 13:10:06		
Date Stamp	17 Jul 2020 13:11:55			***File Name***	F:\NMR FIDS ACIDS\PH METHALLYL\FZ8817 2.PH M.JDF		
Frequency (MHz)	600.17	***Nucleus***	1H	***Number of Transients***	8	***Origin***	ECA 600
Original Points Count	32768	***Owner***	CKP	***Points Count***	32768	***Pulse Sequence***	single_pulse.ex2
Receiver Gain	50.00	***Solvent***	CHLOROFORM-d			***Spectrum Offset (Hz)***	5409.6182
Sweep Width (Hz)	16534.39						

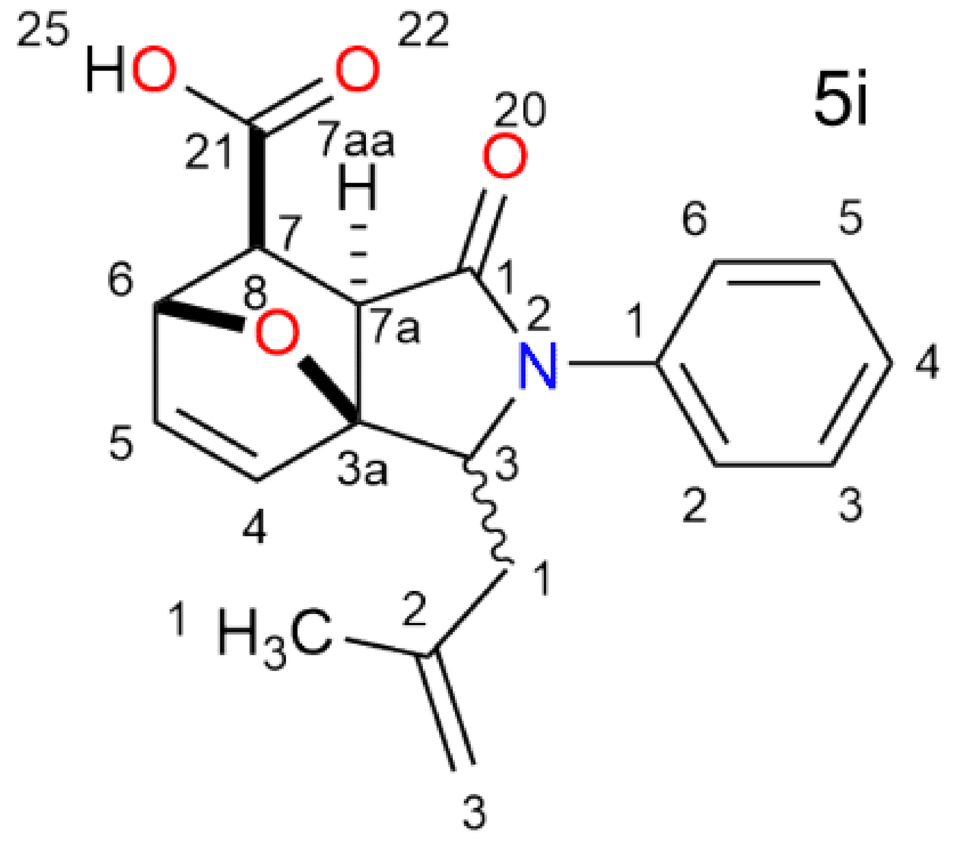

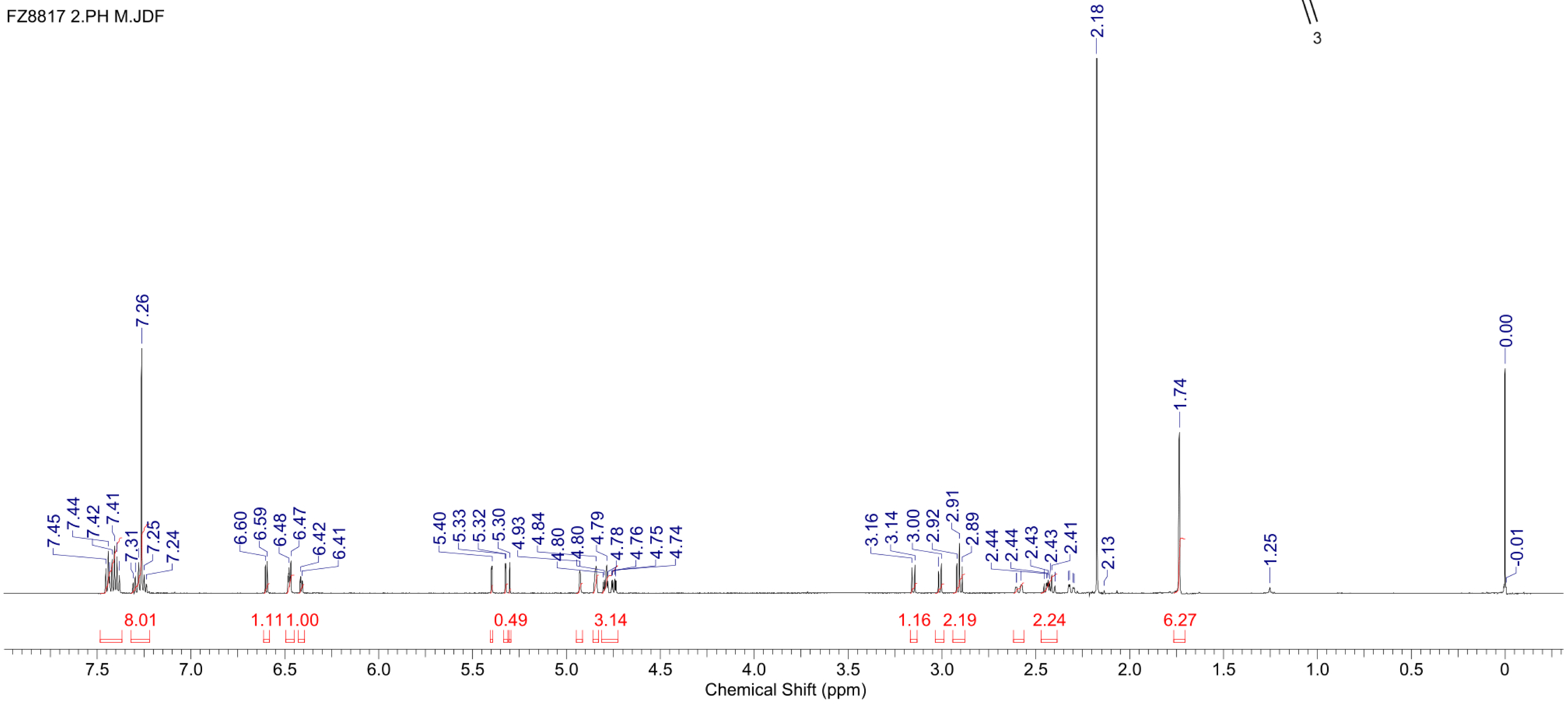

Formula $C_{19}H_{19}NO_4$	FW 325.3585

Acquisition Time (sec)	1.9818	***Comment***	single_pulse	***Date***	17 Jul 2020 13:10:06		
Date Stamp	17 Jul 2020 13:11:55			***File Name***	F:\NMR FIDS ACIDS\PH METHALLYL\FZ8817 2.PH M.JDF		
Frequency (MHz)	600.17	***Nucleus***	1H	***Number of Transients***	8	***Origin***	ECA 600
Original Points Count	32768	***Owner***	CKP	***Points Count***	32768	***Pulse Sequence***	single_pulse.ex2
Receiver Gain	50.00	***Solvent***	CHLOROFORM-d			***Spectrum Offset (Hz)***	5409.6182
Sweep Width (Hz)	16534.39						

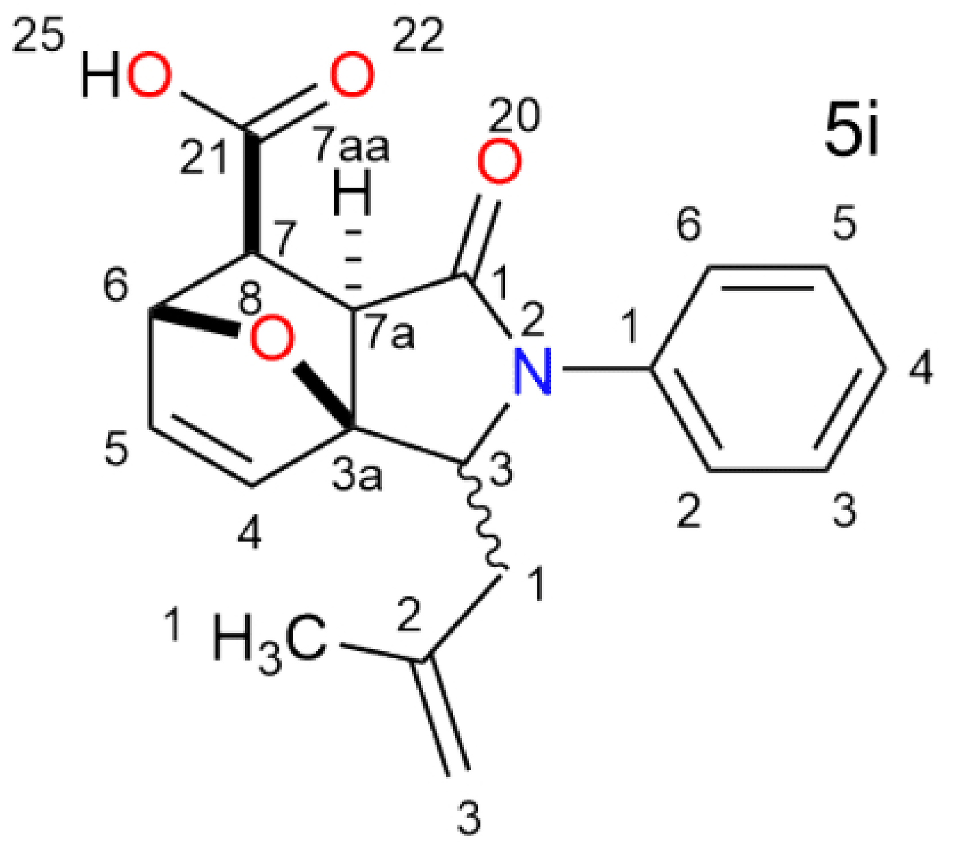

FZ8817 2.PH M.JDF

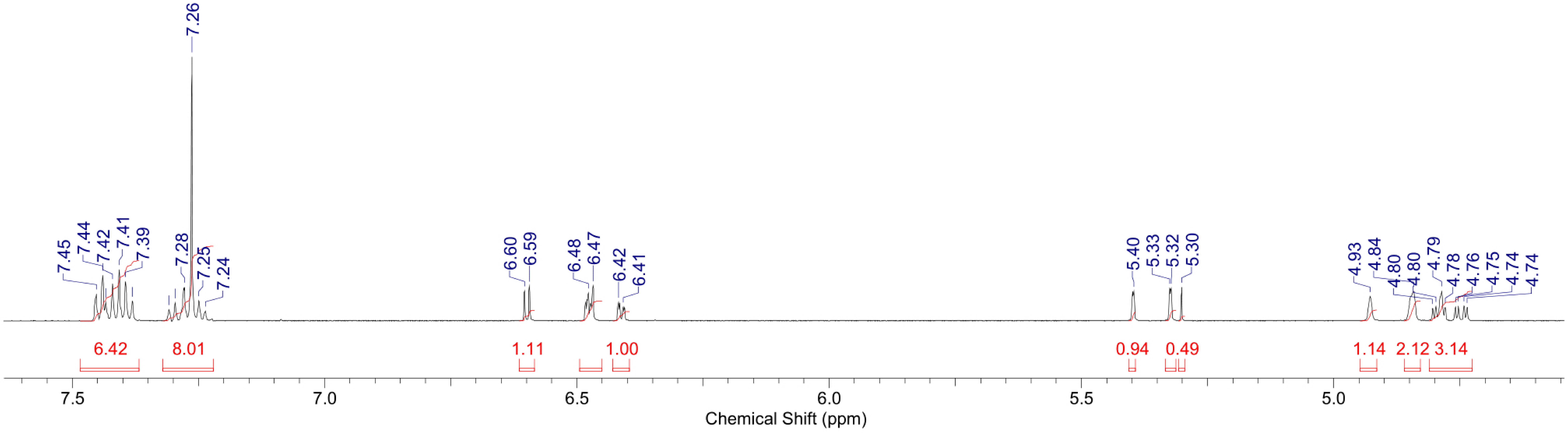

**Formula**	$C_{19}H_{19}NO_4$	**FW**	325.3585

**Acquisition Time (sec)**	1.9818	**Comment**	single_pulse	**Date**	17 Jul 2020 13:10:06		
**Date Stamp**	17 Jul 2020 13:11:55			**File Name**	F:\NMR FIDS ACIDS\PH METHALLYL\FZ8817 2.PH M.JDF		
**Frequency (MHz)**	600.17	**Nucleus**	1H	**Number of Transients**	8	**Origin**	ECA 600
**Original Points Count**	32768	**Owner**	CKP	**Points Count**	32768	**Pulse Sequence**	single_pulse.ex2
**Receiver Gain**	50.00	**Solvent**	CHLOROFORM-d			**Spectrum Offset (Hz)**	5409.6182
**Sweep Width (Hz)**	16534.39						

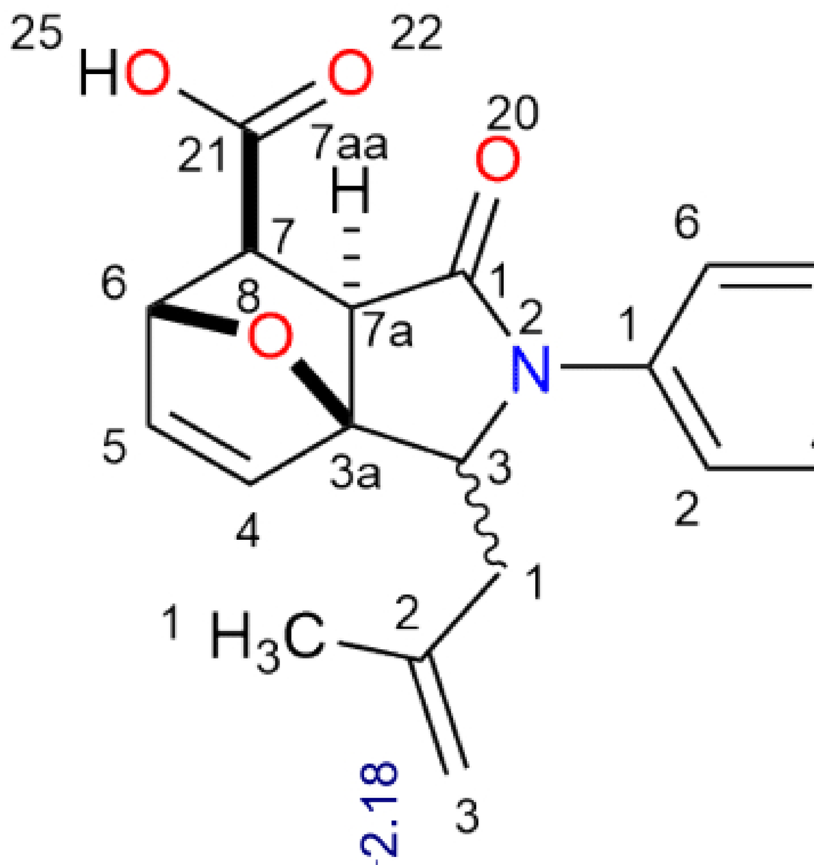

5i

FZ8817 2.PH M.JDF

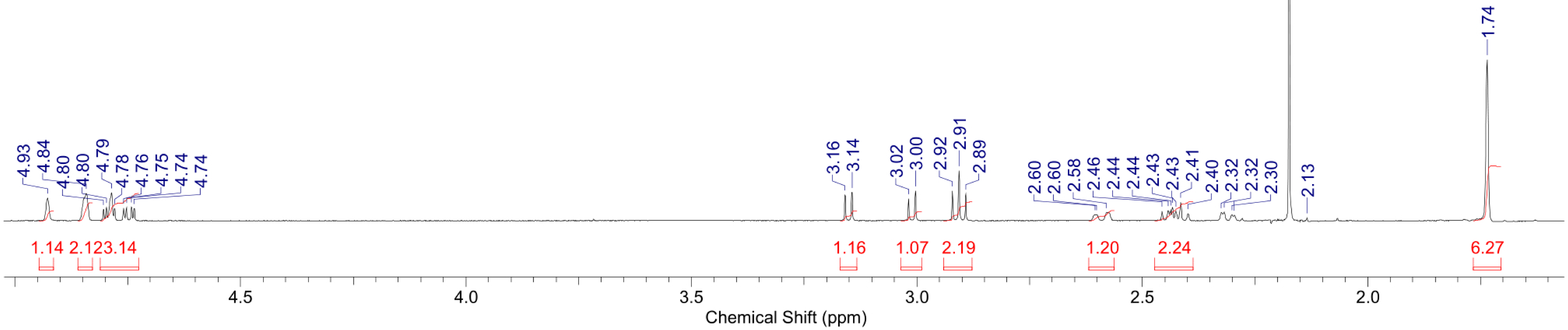

NMR spectrum data for compound 5i (MeCN, Δ, 3 h)

**Formula** $C_{19}H_{19}NO_4$	**FW** 325.3585

**Acquisition Time (sec)**	1.9818	**Comment**	single_pulse	**Date**	17 Jul 2020 11:33:49		
**Date Stamp**	17 Jul 2020 11:35:37			**File Name**	F:\NMR FIDS ACIDS\PH METHALLYL\FZ8819-1.JDF		
**Frequency (MHz)**	600.17	**Nucleus**	1H	**Number of Transients**	8	**Origin**	ECA 600
**Original Points Count**	32768	**Owner**	CKP	**Points Count**	32768	**Pulse Sequence**	single_pulse.ex2
**Receiver Gain**	52.00	**Solvent**	CHLOROFORM-d			**Spectrum Offset (Hz)**	5409.1133
**Sweep Width (Hz)**	16534.39						

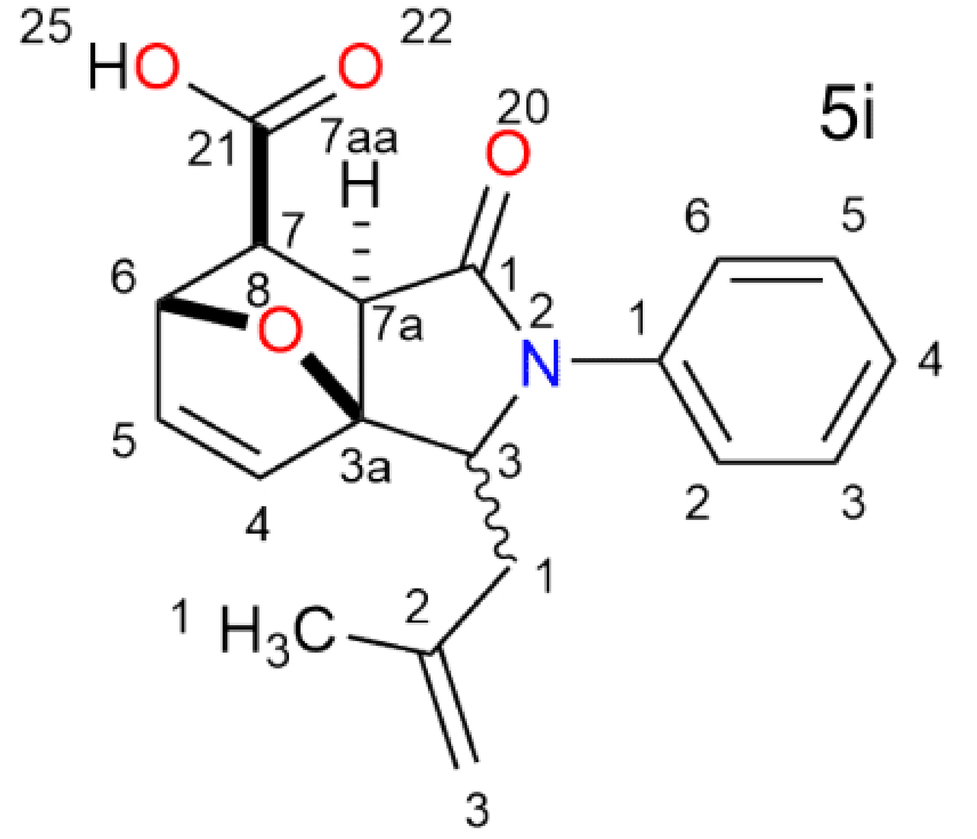

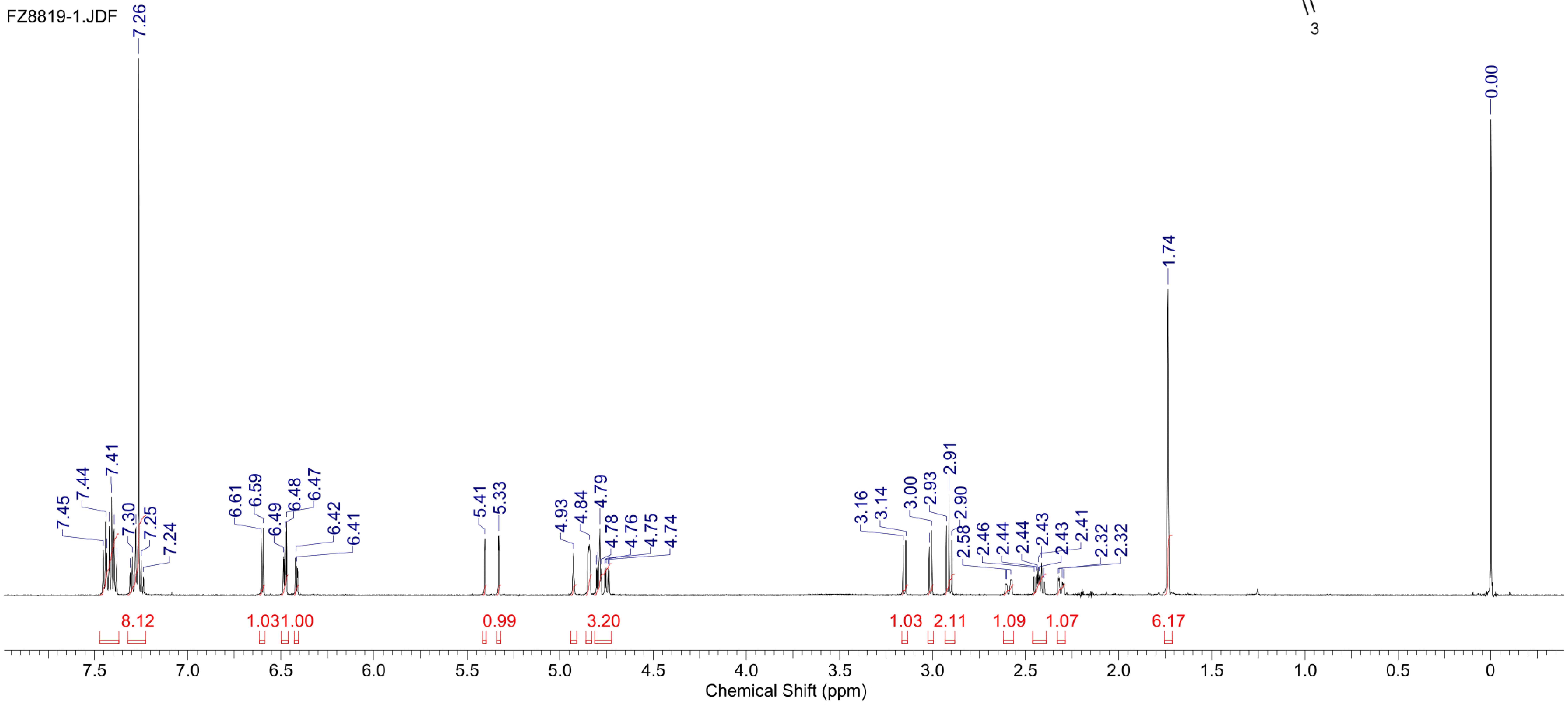

Formula	$C_{19}H_{19}NO_4$	FW	325.3585

Acquisition Time (sec)	1.9818	***Comment***	single_pulse	***Date***	17 Jul 2020 11:33:49		
Date Stamp	17 Jul 2020 11:35:37			***File Name***	F:\NMR FIDS ACIDS\PH METHALLYL\FZ8819-1.JDF		
Frequency (MHz)	600.17	***Nucleus***	1H	***Number of Transients***	8	***Origin***	ECA 600
Original Points Count	32768	***Owner***	CKP	***Points Count***	32768	***Pulse Sequence***	single_pulse.ex2
Receiver Gain	52.00	***Solvent***	CHLOROFORM-d			***Spectrum Offset (Hz)***	5409.1133
Sweep Width (Hz)	16534.39						

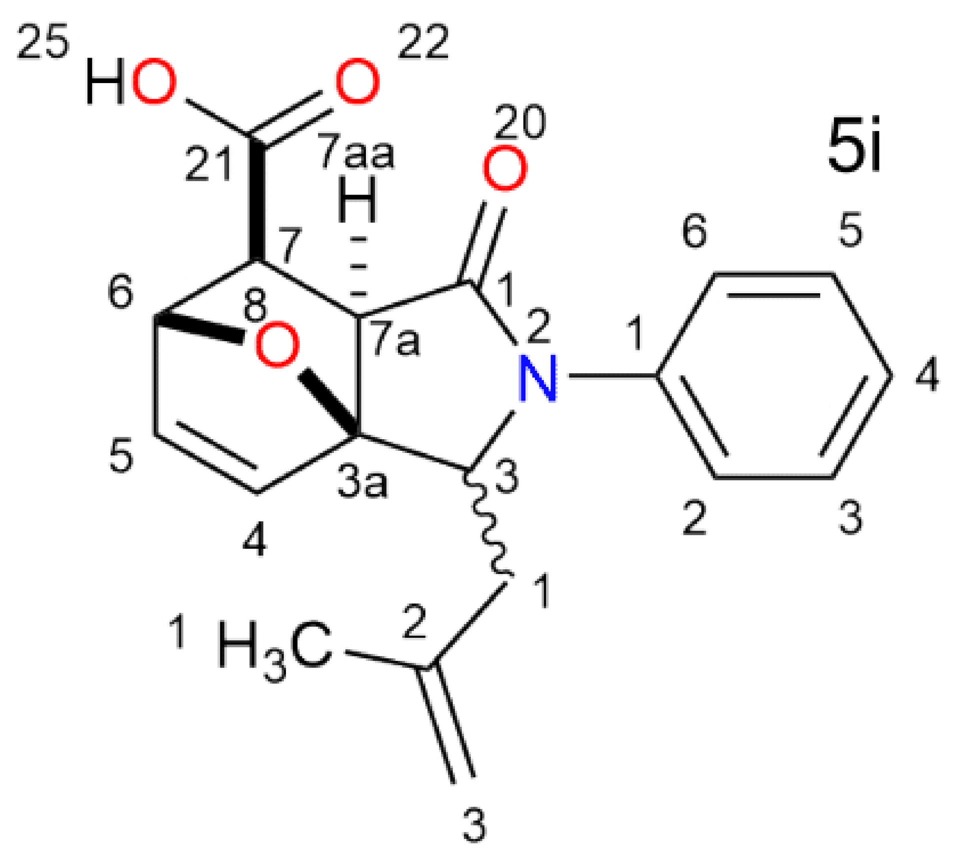

FZ8819-1.JDF

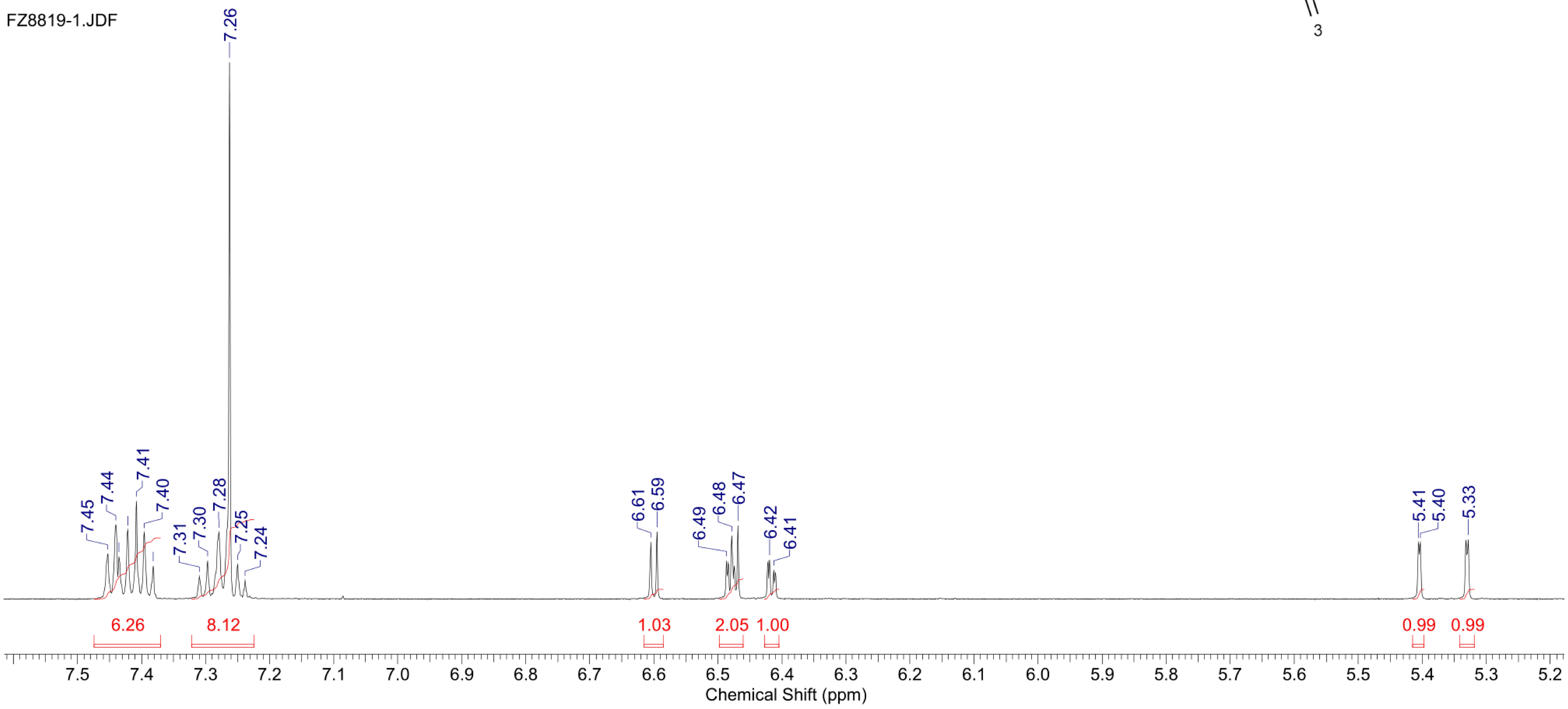

Formula $C_{19}H_{19}NO_4$	FW 325.3585

Acquisition Time (sec)	1.9818	***Comment***	single_pulse	***Date***	17 Jul 2020 11:33:49		
Date Stamp	17 Jul 2020 11:35:37			***File Name***	F:\NMR FIDS ACIDS\PH METHALLYL\FZ8819-1.JDF		
Frequency (MHz)	600.17	***Nucleus***	1H	***Number of Transients***	8	***Origin***	ECA 600
Original Points Count	32768	***Owner***	CKP	***Points Count***	32768	***Pulse Sequence***	single_pulse.ex2
Receiver Gain	52.00	***Solvent***	CHLOROFORM-d			***Spectrum Offset (Hz)***	5409.1133
Sweep Width (Hz)	16534.39						

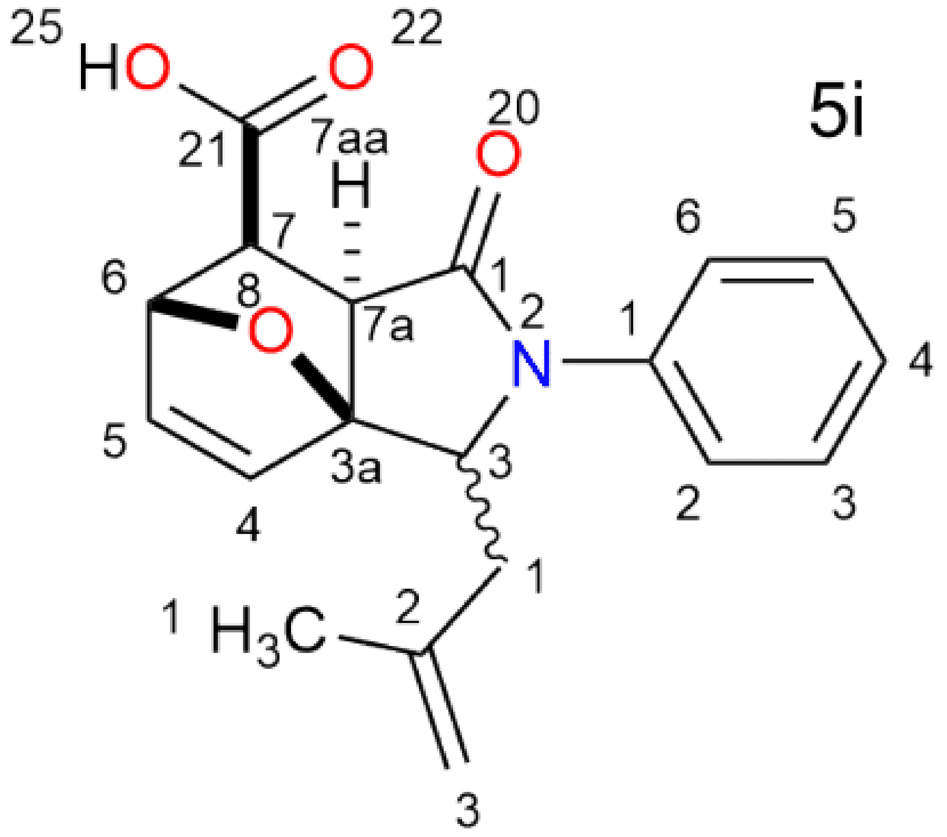

5i

FZ8819-1.JDF

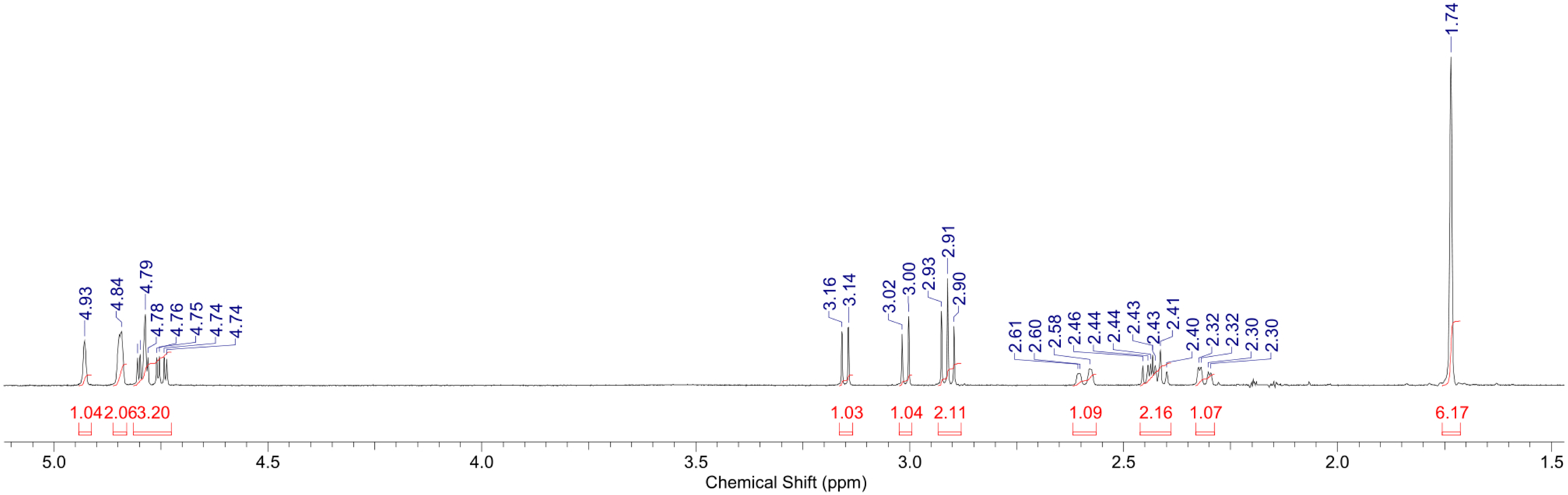

NMR spectrum data for compound 5i (PhH, Δ, 3 h)

Formula	$C_{19}H_{19}NO_4$	FW	325.3585

**Acquisition Time (sec)**	1.9818	**Comment**	single_pulse	**Date**	17 Jul 2020 11:39:53		
**Date Stamp**	17 Jul 2020 11:41:41			**File Name**	F:\NMR FIDS ACIDS\PH METHALLYL\FZ8816-1.JDF		
**Frequency (MHz)**	600.17	**Nucleus**	1H	**Number of Transients**	8	**Origin**	ECA 600
**Original Points Count**	32768	**Owner**	CKP	**Points Count**	32768	**Pulse Sequence**	single_pulse.ex2
**Receiver Gain**	48.00	**Solvent**	CHLOROFORM-d			**Spectrum Offset (Hz)**	5409.1133
**Sweep Width (Hz)**	16534.39						

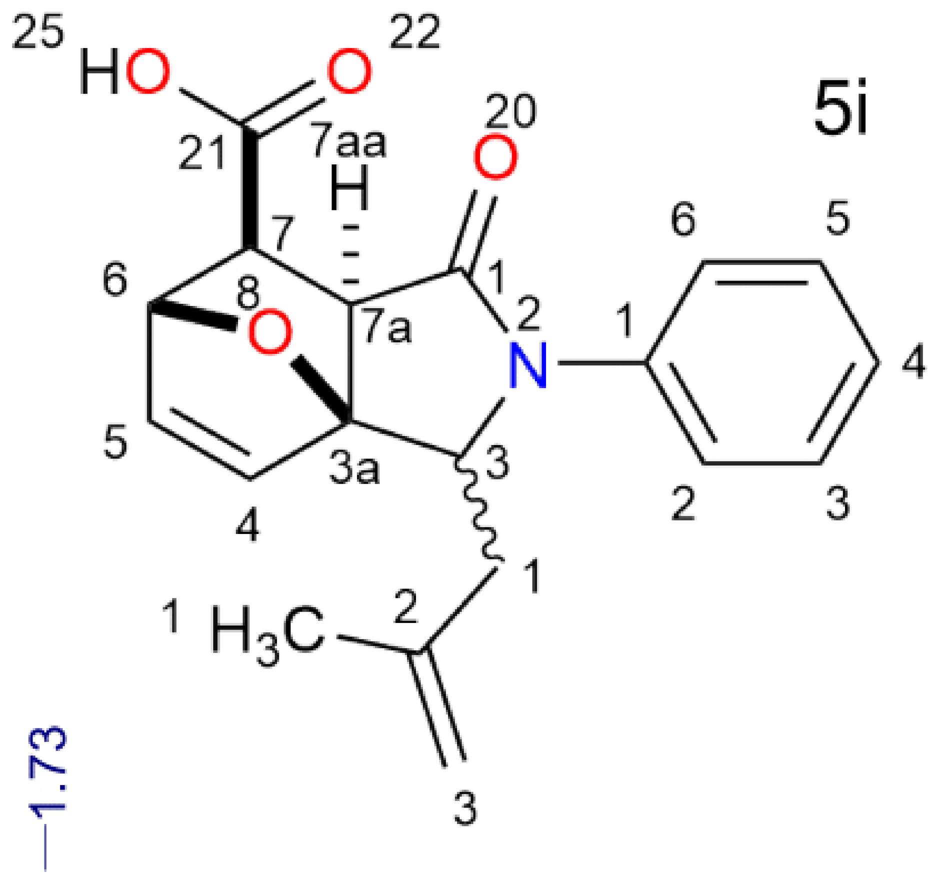

FZ8816-1.JDF

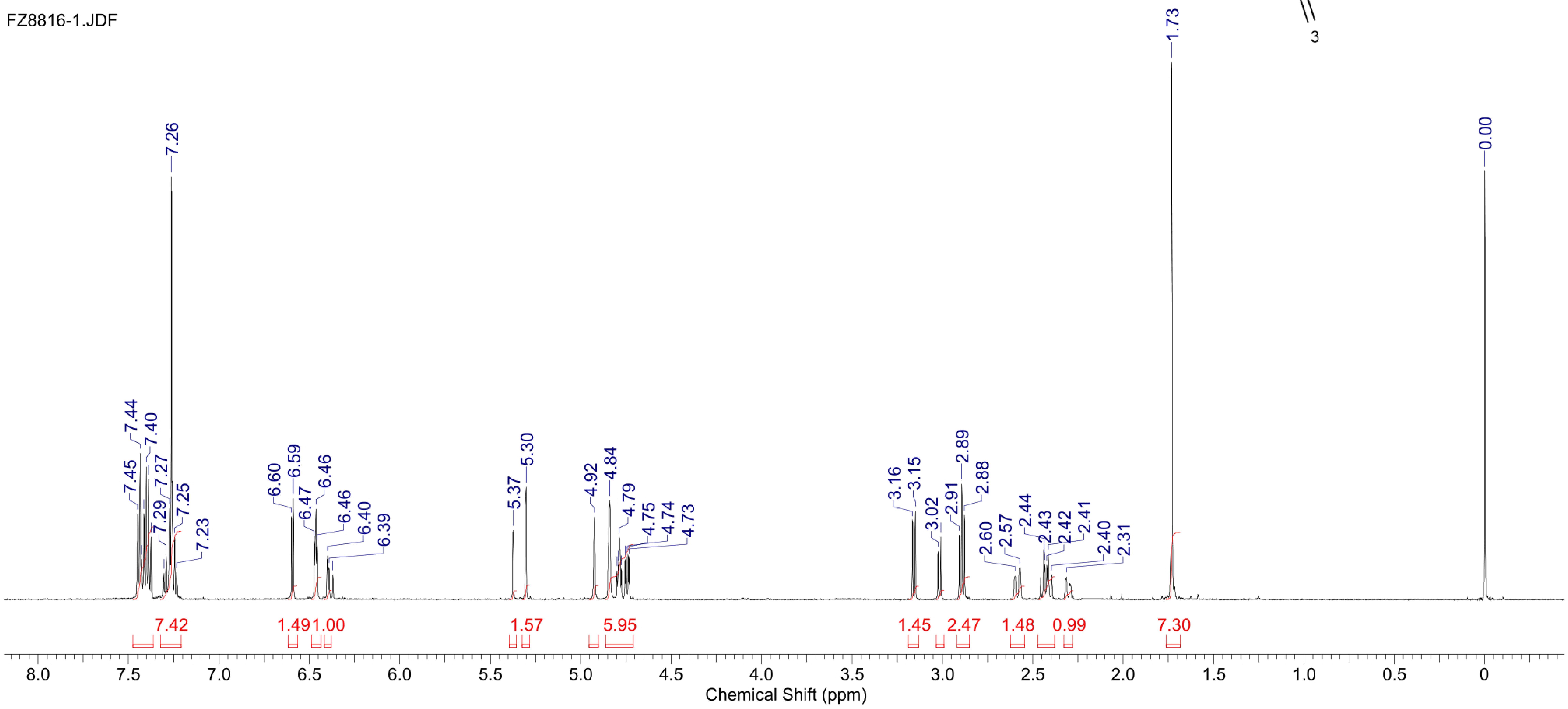

Formula $C_{19}H_{19}NO_4$	FW 325.3585

**Acquisition Time (sec)**	1.9818	**Comment**	single_pulse	**Date**	17 Jul 2020 11:39:53		
**Date Stamp**	17 Jul 2020 11:41:41			**File Name**	F:\NMR FIDS ACIDS\PH METHALLYL\FZ8816-1.JDF		
**Frequency (MHz)**	600.17	**Nucleus**	1H	**Number of Transients**	8	**Origin**	ECA 600
**Original Points Count**	32768	**Owner**	CKP	**Points Count**	32768	**Pulse Sequence**	single_pulse.ex2
**Receiver Gain**	48.00	**Solvent**	CHLOROFORM-d			**Spectrum Offset (Hz)**	5409.1133
**Sweep Width (Hz)**	16534.39						

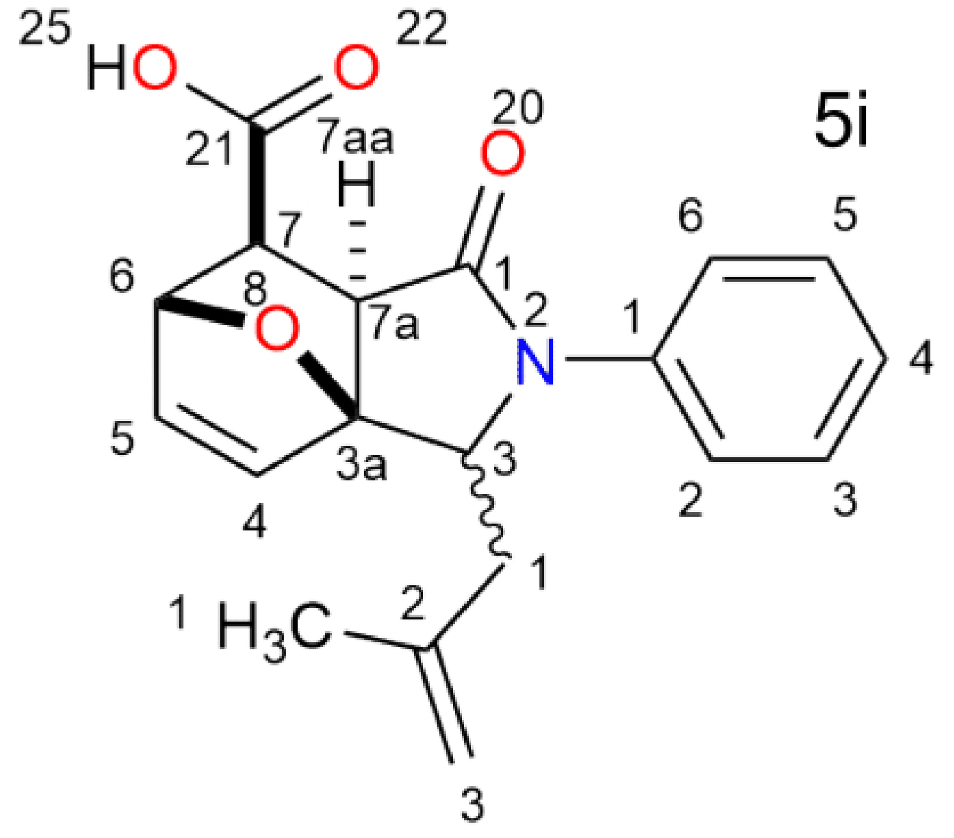

FZ8816-1.JDF

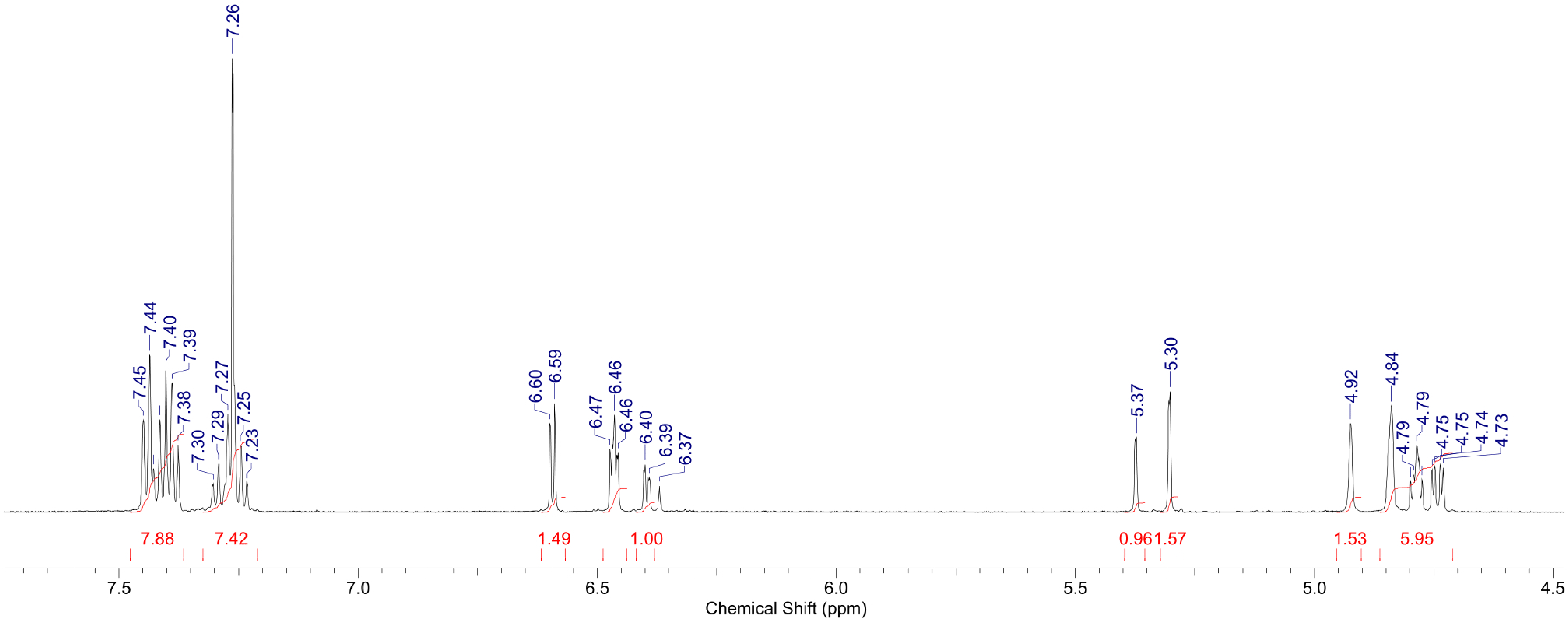

Formula $C_{19}H_{19}NO_4$	FW 325.3585

**Acquisition Time (sec)**	1.9818	**Comment**	single_pulse	**Date**	17 Jul 2020 11:39:53		
**Date Stamp**	17 Jul 2020 11:41:41			**File Name**	F:\NMR FIDS ACIDS\PH METHALLYL\FZ8816-1.JDF		
**Frequency (MHz)**	600.17	**Nucleus**	1H	**Number of Transients**	8	**Origin**	ECA 600
**Original Points Count**	32768	**Owner**	CKP	**Points Count**	32768	**Pulse Sequence**	single_pulse.ex2
**Receiver Gain**	48.00	**Solvent**	CHLOROFORM-d			**Spectrum Offset (Hz)**	5409.1133
**Sweep Width (Hz)**	16534.39						

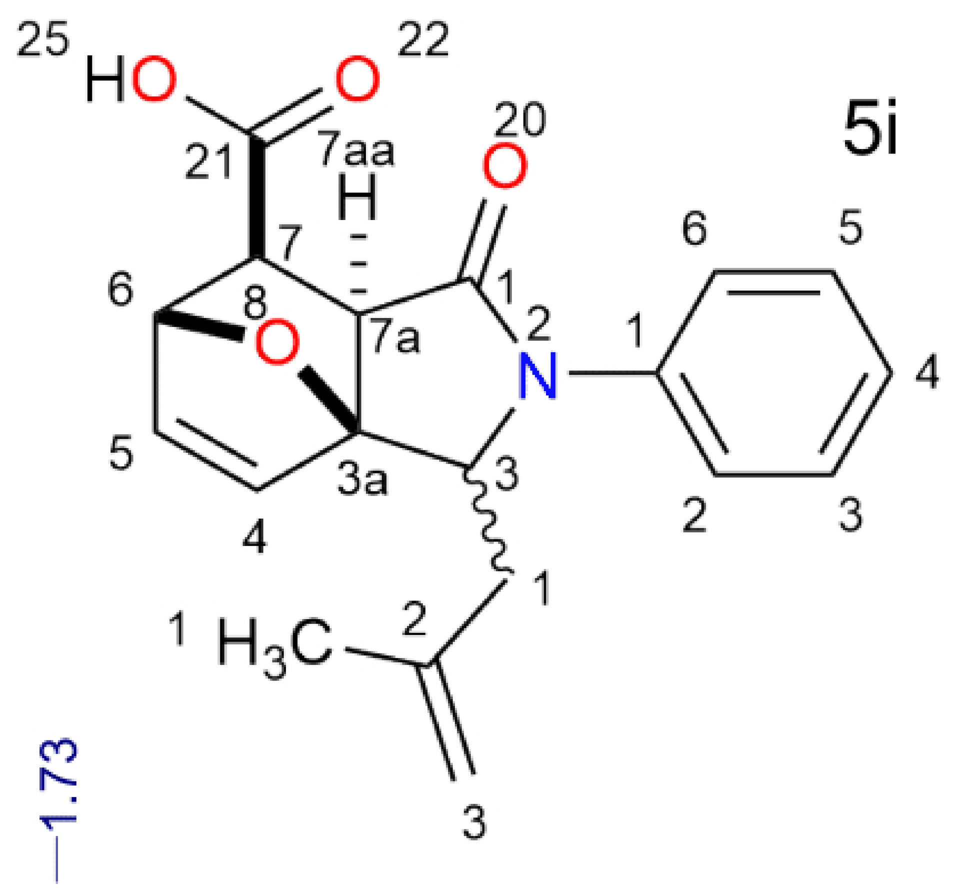

FZ8816-1.JDF

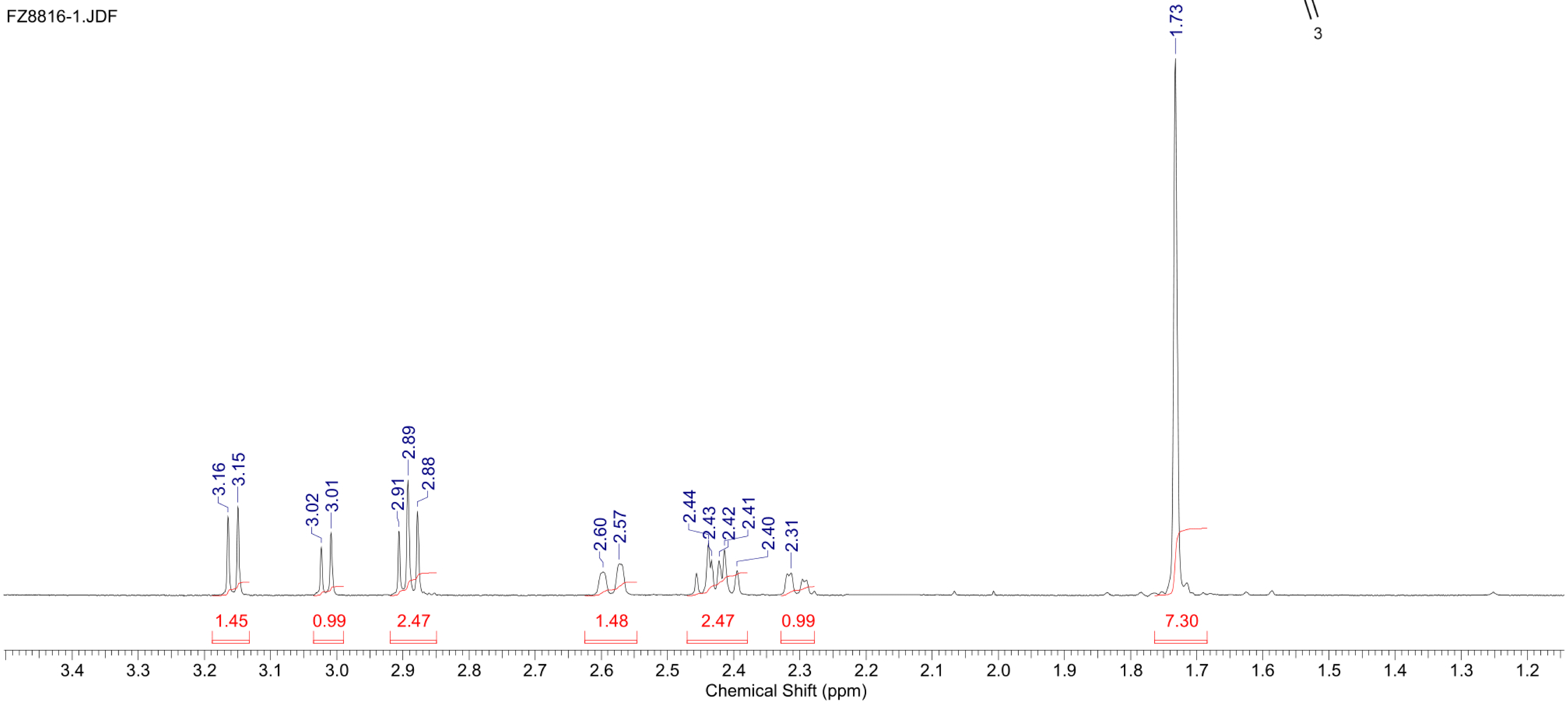

NMR spectrum data for compound 5i (PhMe, r.t., 3 h)

**Formula** $C_{19}H_{19}NO_4$	**FW** 325.3585		

**Acquisition Time (sec)**	1.9818	**Comment**	single_pulse	**Date**	14 Jul 2020 13:06:10		
**Date Stamp**	14 Jul 2020 13:07:54			**File Name**	F:\NMR FIDS ACIDS\PH METHALLYL\FZ8796-1.JDF		
**Frequency (MHz)**	600.17	**Nucleus**	1H	**Number of Transients**	8	**Origin**	ECA 600
**Original Points Count**	32768	**Owner**	CKP	**Points Count**	32768	**Pulse Sequence**	single_pulse.ex2
**Receiver Gain**	50.00	**Solvent**	CHLOROFORM-d			**Spectrum Offset (Hz)**	5408.6089
**Sweep Width (Hz)**	16534.39	**Temperature (degree C)**	37.300				

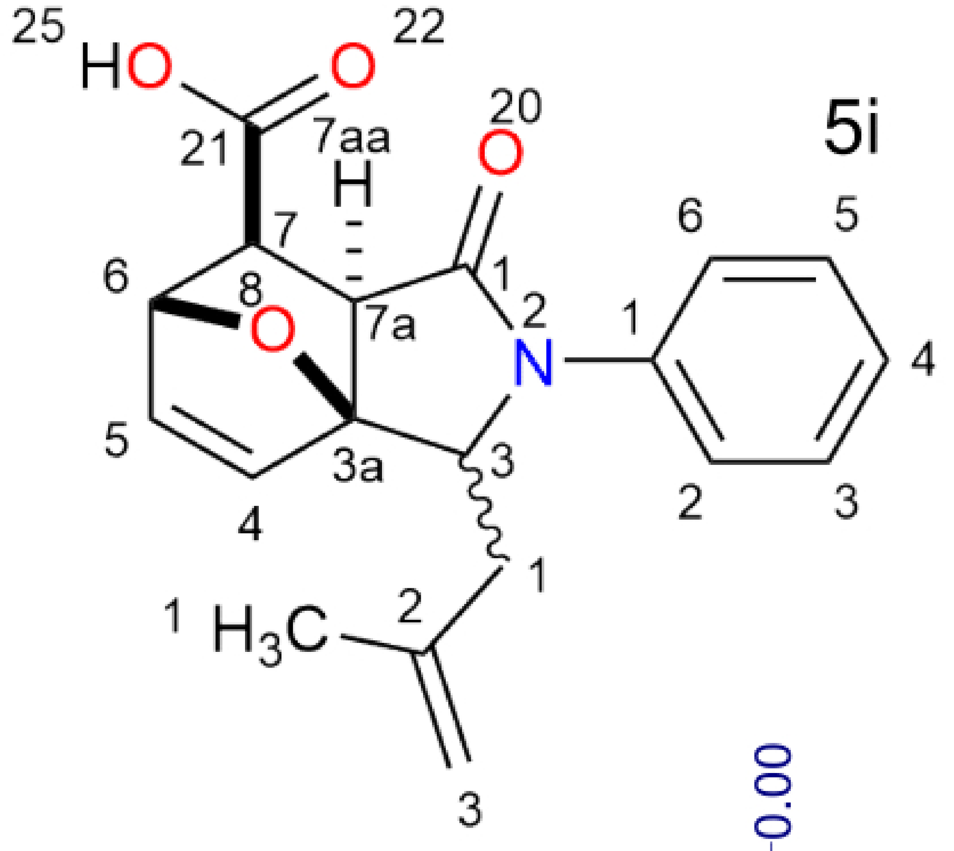

5i

FZ8796-1.JDF

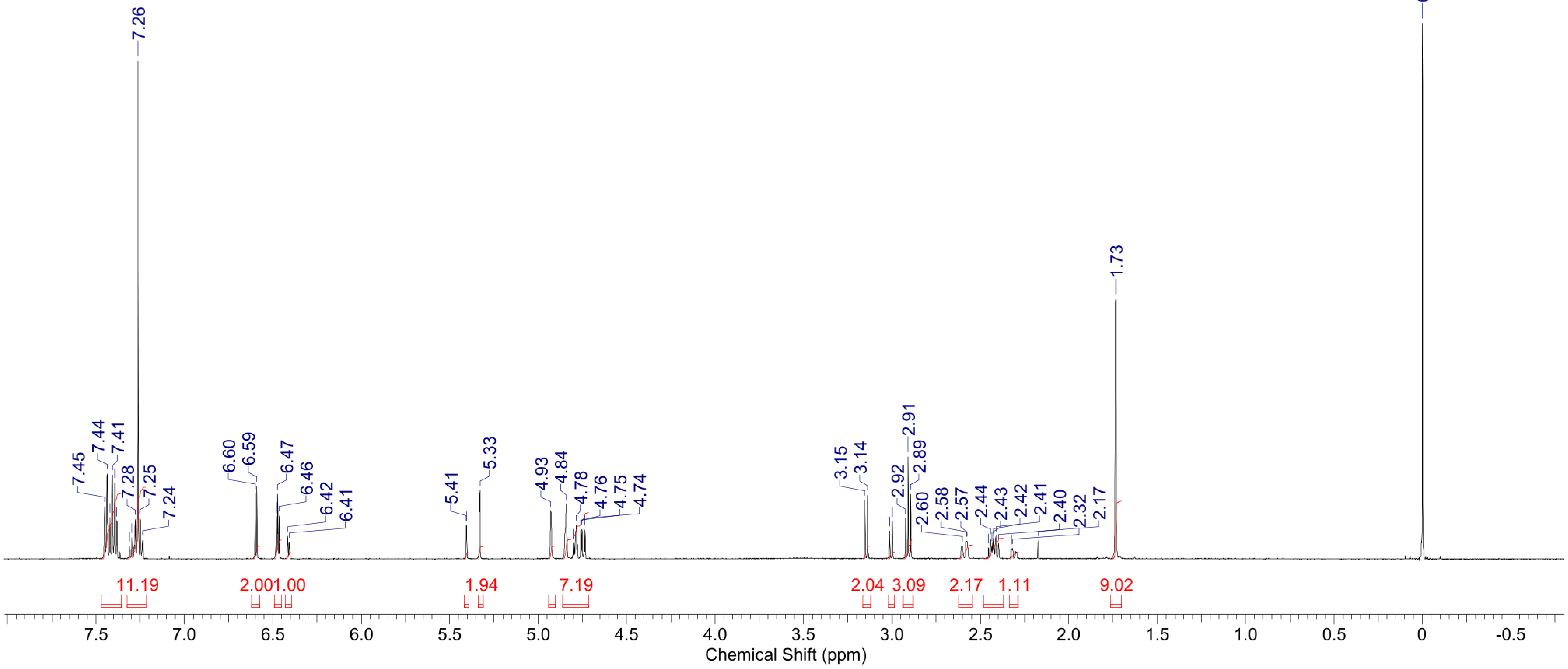

Formula	$C_{19}H_{19}NO_4$	FW	325.3585

**Acquisition Time (sec)**	1.9818	**Comment**	single_pulse	**Date**	14 Jul 2020 13:06:10		
**Date Stamp**	14 Jul 2020 13:07:54			**File Name**	F:\NMR FIDS ACIDS\PH METHALLYL\FZ8796-1.JDF		
**Frequency (MHz)**	600.17	**Nucleus**	1H	**Number of Transients**	8	**Origin**	ECA 600
**Original Points Count**	32768	**Owner**	CKP	**Points Count**	32768	**Pulse Sequence**	single_pulse.ex2
**Receiver Gain**	50.00	**Solvent**	CHLOROFORM-d			**Spectrum Offset (Hz)**	5408.6089
**Sweep Width (Hz)**	16534.39	**Temperature (degree C)**	37.300				

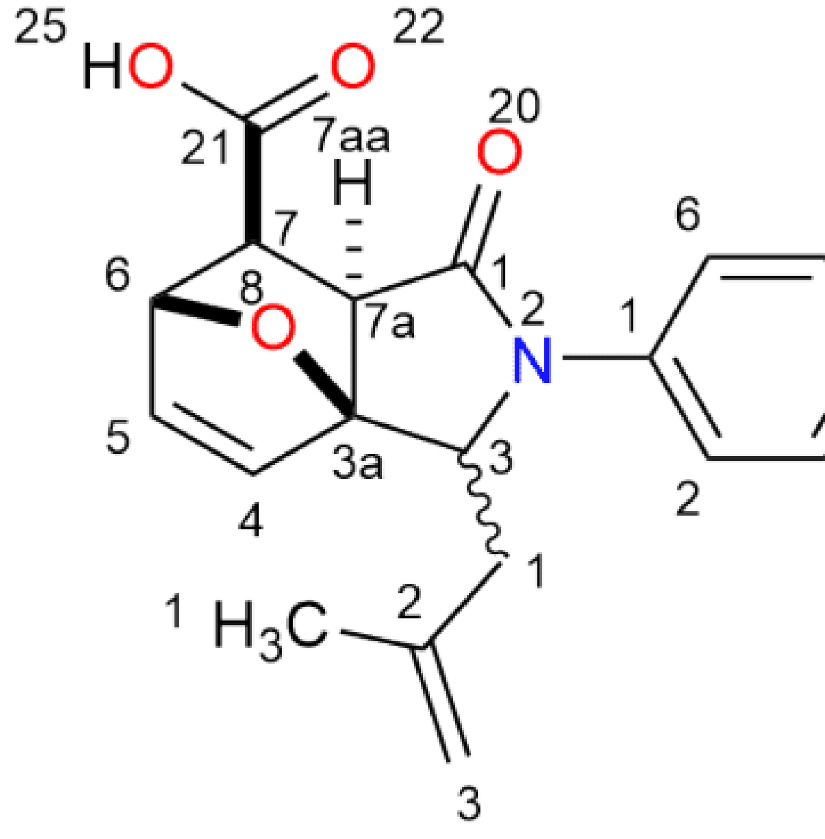

5i

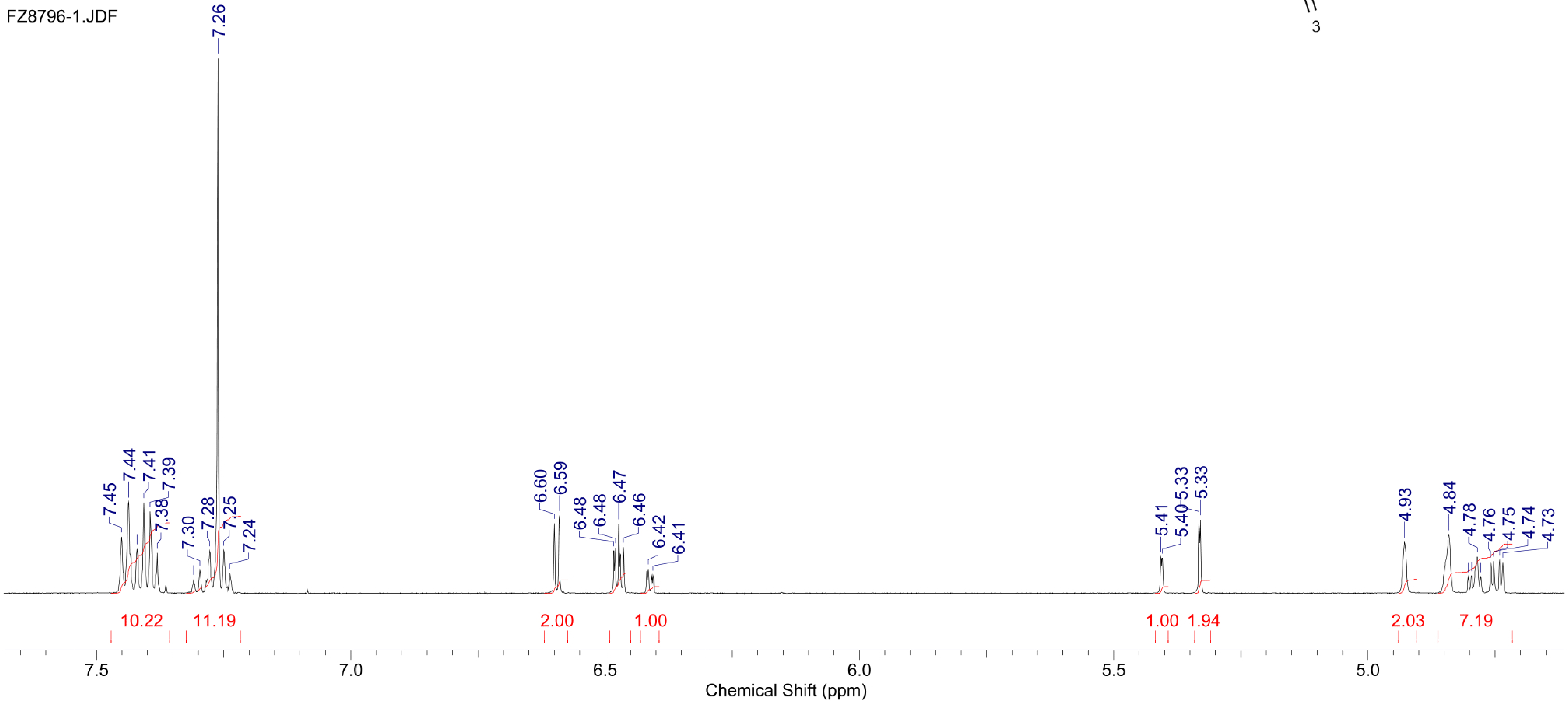

Formula $C_{19}H_{19}NO_4$	FW 325.3585

**Acquisition Time (sec)**	1.9818	**Comment**	single_pulse	**Date**	14 Jul 2020 13:06:10		
**Date Stamp**	14 Jul 2020 13:07:54			**File Name**	F:\NMR FIDS ACIDS\PH METHALLYL\FZ8796-1.JDF		
**Frequency (MHz)**	600.17	**Nucleus**	1H	**Number of Transients**	8	**Origin**	ECA 600
**Original Points Count**	32768	**Owner**	CKP	**Points Count**	32768	**Pulse Sequence**	single_pulse.ex2
**Receiver Gain**	50.00	**Solvent**	CHLOROFORM-d			**Spectrum Offset (Hz)**	5408.6089
**Sweep Width (Hz)**	16534.39	**Temperature (degree C)**	37.300				

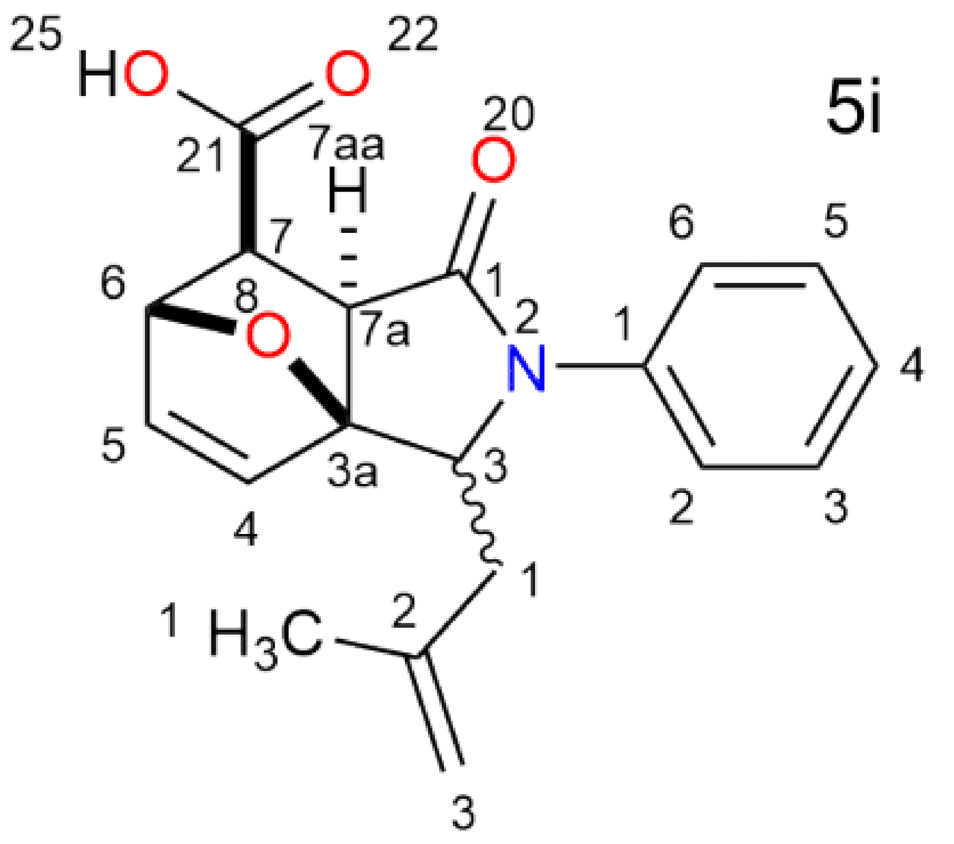

5i

FZ8796-1.JDF

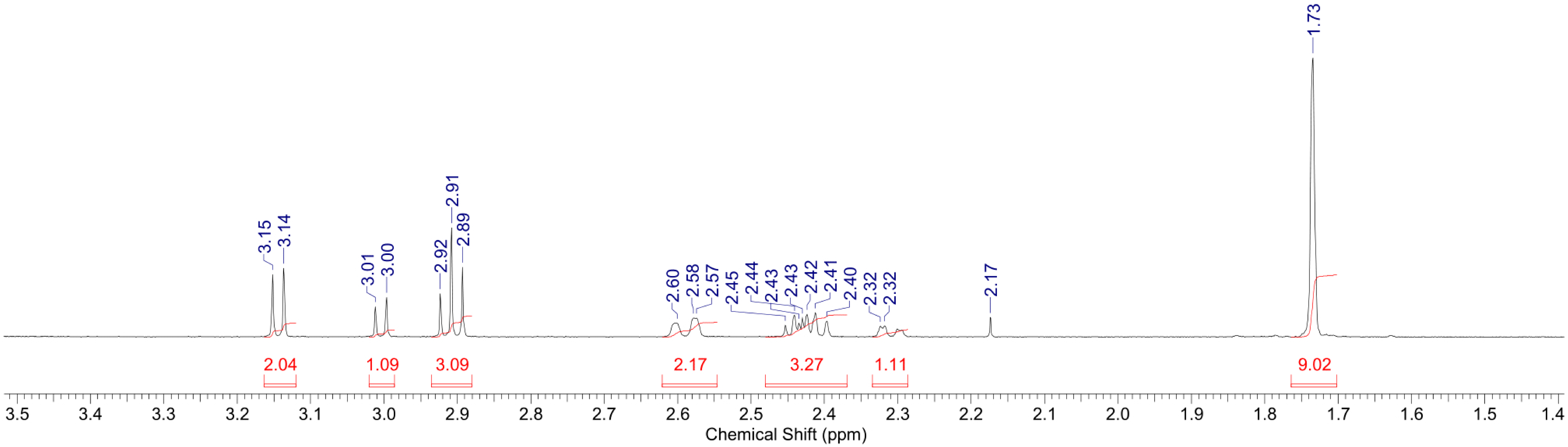

NMR spectrum data for compound 5i (PhMe, Δ, 3 h)

**Formula** $C_{19}H_{19}NO_4$	**FW** 325.3585

**Acquisition Time (sec)**	1.9818	**Comment**	single_pulse	**Date**	14 Jul 2020 13:30:09		
**Date Stamp**	14 Jul 2020 13:31:53			**File Name**	F:\NMR FIDS ACIDS\PH METHALLYL\FZ8798 7.PH M.JDF		
**Frequency (MHz)**	600.17	**Nucleus**	1H	**Number of Transients**	8	**Origin**	ECA 600
**Original Points Count**	32768	**Owner**	CKP	**Points Count**	32768	**Pulse Sequence**	single_pulse.ex2
**Receiver Gain**	50.00	**Solvent**	CHLOROFORM-d			**Spectrum Offset (Hz)**	5409.1133
**Sweep Width (Hz)**	16534.39	**Temperature (degree C)**	39.000				

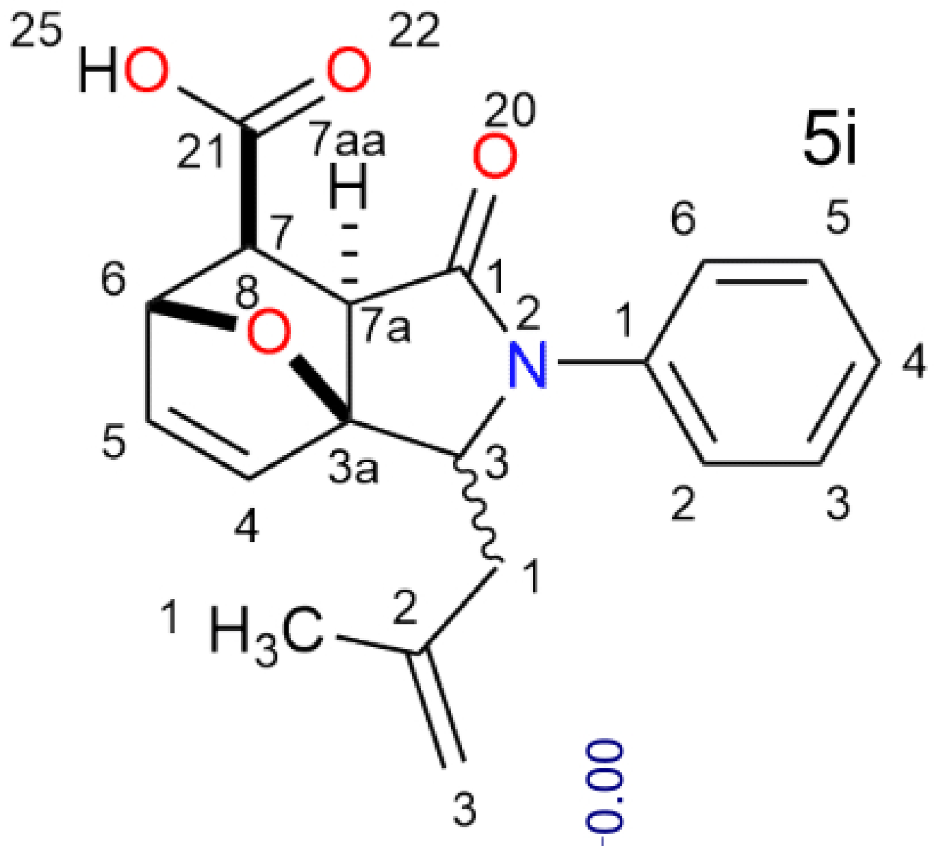

FZ8798 7.PH M.JDF

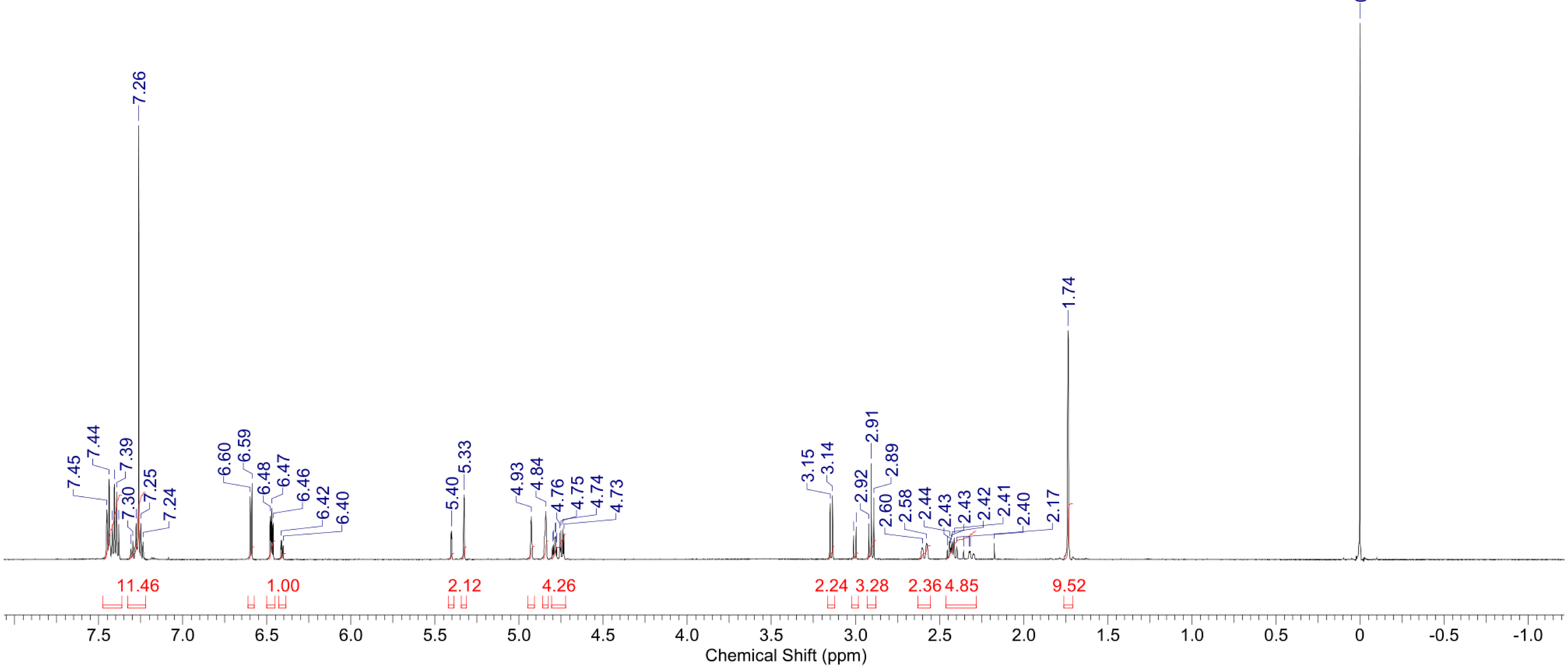

Formula $C_{19}H_{19}NO_4$	FW 325.3585

**Acquisition Time (sec)**	1.9818	**Comment**	single_pulse	**Date**	14 Jul 2020 13:30:09		
**Date Stamp**	14 Jul 2020 13:31:53			**File Name**	F:\NMR FIDS ACIDS\PH METHALLYL\FZ8798 7.PH M.JDF		
**Frequency (MHz)**	600.17	**Nucleus**	1H	**Number of Transients**	8	**Origin**	ECA 600
**Original Points Count**	32768	**Owner**	CKP	**Points Count**	32768	**Pulse Sequence**	single_pulse.ex2
**Receiver Gain**	50.00	**Solvent**	CHLOROFORM-d			**Spectrum Offset (Hz)**	5409.1133
**Sweep Width (Hz)**	16534.39	**Temperature (degree C)**	39.000				

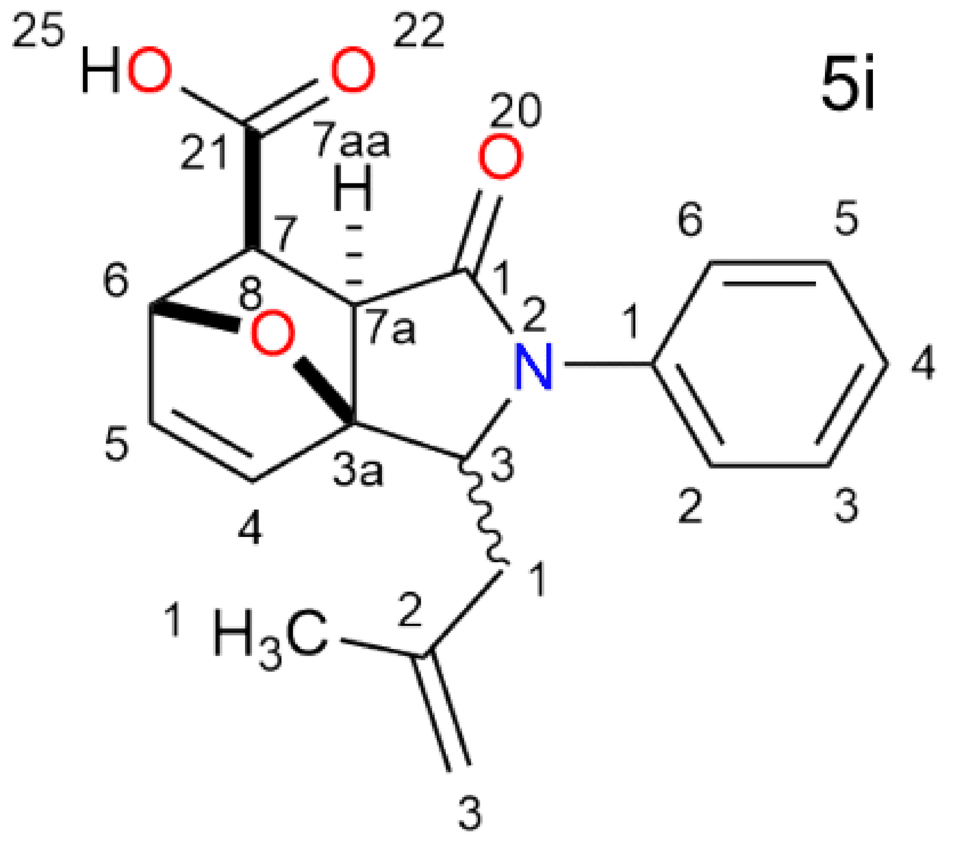

5i

FZ8798 7.PH M.JDF

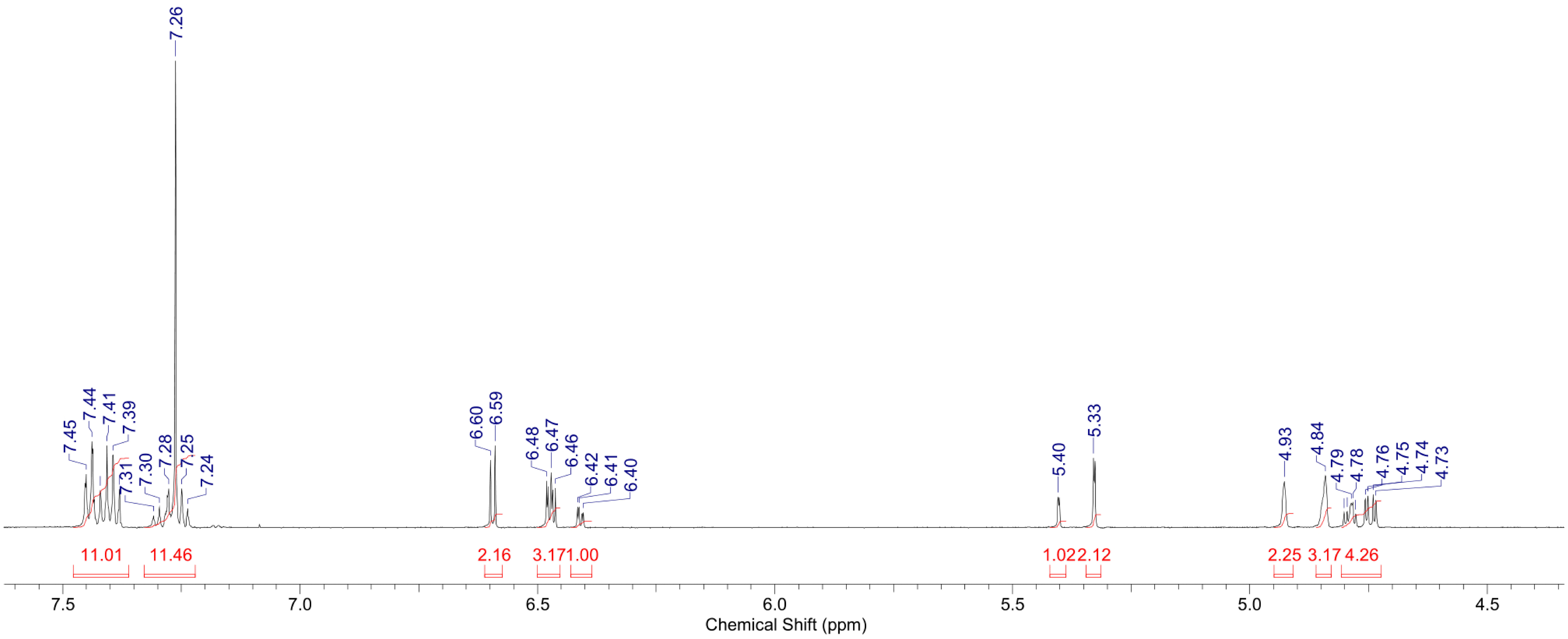

Formula $C_{19}H_{19}NO_4$	FW 325.3585

Acquisition Time (sec)	1.9818	***Comment***	single_pulse	***Date***	14 Jul 2020 13:30:09		
Date Stamp	14 Jul 2020 13:31:53			***File Name***	F:\NMR FIDS ACIDS\PH METHALLYL\FZ8798 7.PH M.JDF		
Frequency (MHz)	600.17	***Nucleus***	1H	***Number of Transients***	8	***Origin***	ECA 600
Original Points Count	32768	***Owner***	CKP	***Points Count***	32768	***Pulse Sequence***	single_pulse.ex2
Receiver Gain	50.00	***Solvent***	CHLOROFORM-d			***Spectrum Offset (Hz)***	5409.1133
Sweep Width (Hz)	16534.39	***Temperature (degree C)***	39.000				

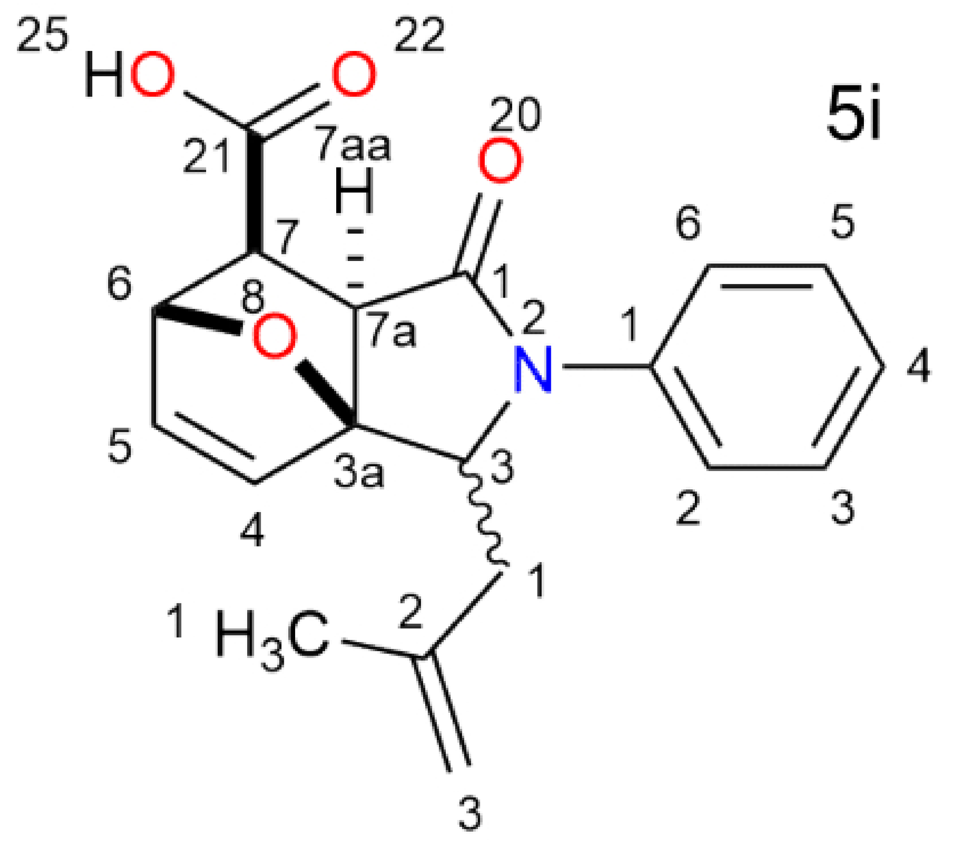

5i

FZ8798 7.PH M.JDF

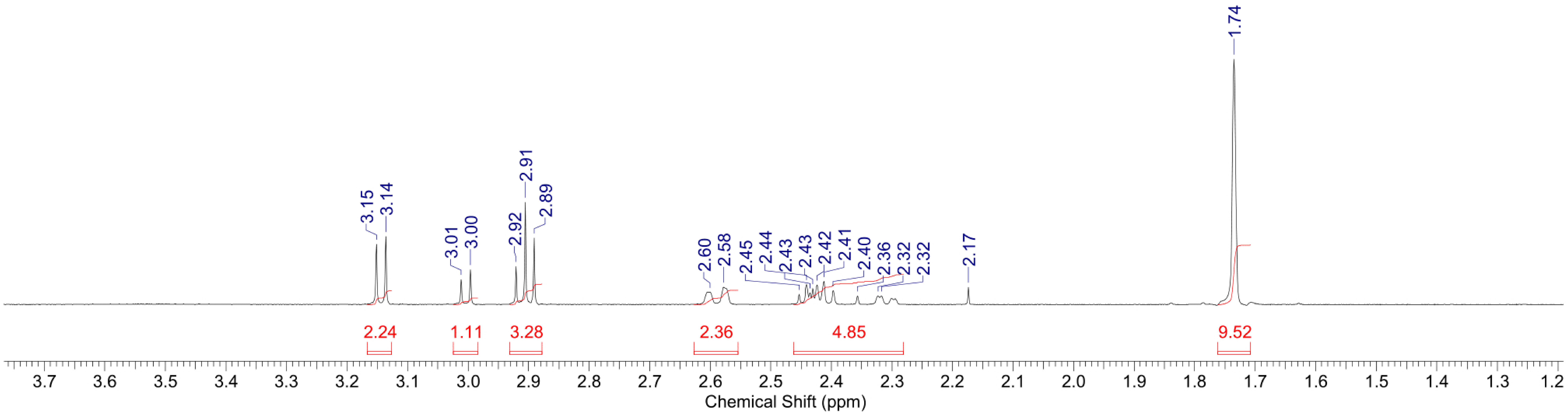

NMR spectrum data for compound 5i

Formula $C_{19}H_{19}NO_4$	***FW***	325.3585

Acquisition Time (sec)	0.6921	***Comment***	single pulse decoupled gated NOE	***Date***	08 Feb 2020 00:19:56
Date Stamp	07 Feb 2020 17:53:54	***File Name***	C:\USERS\лаба 534\DOWNLOADS\FZ8419-1.JDF		
Frequency (MHz)	150.91	***Nucleus***	13C	***Number of Transients***	1000
Origin	ECA 600				
Original Points Count	32768	***Owner***	Mass	***Points Count***	32768
Pulse Sequence	single_pulse_dec				
Receiver Gain	56.00	***Solvent***	CHLOROFORM-d	***Spectrum Offset (Hz)***	15091.3428
Sweep Width (Hz)	47348.49	***Temperature (degree C)***	25.000		

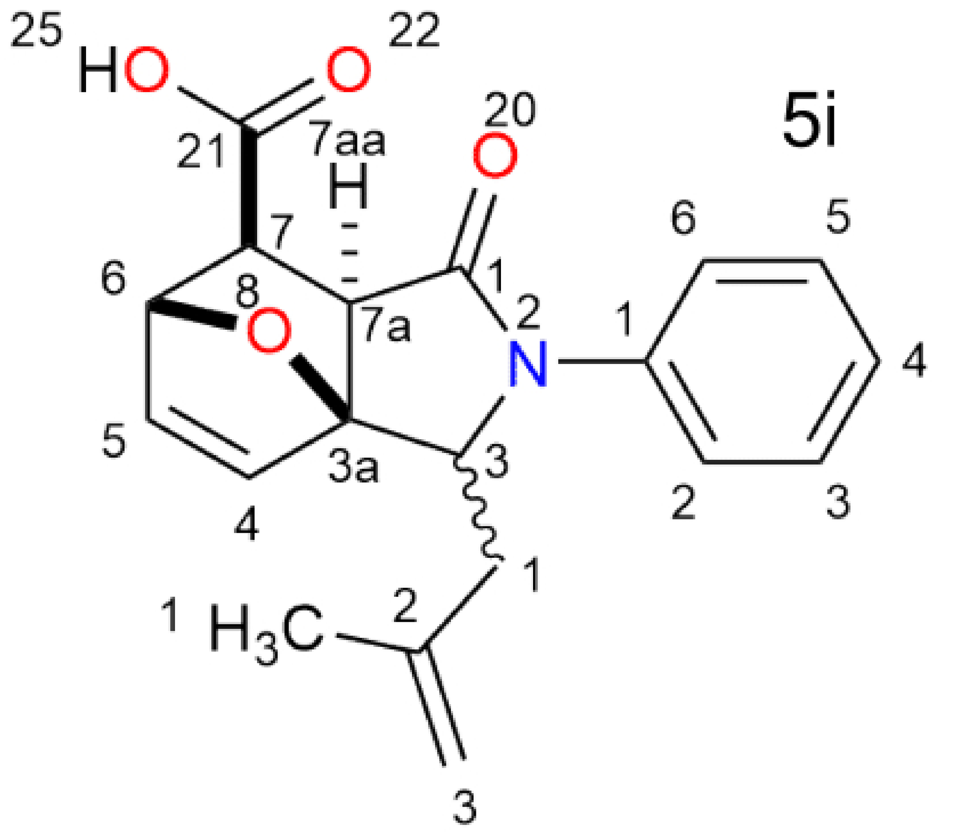

FZ8419-1.JDF

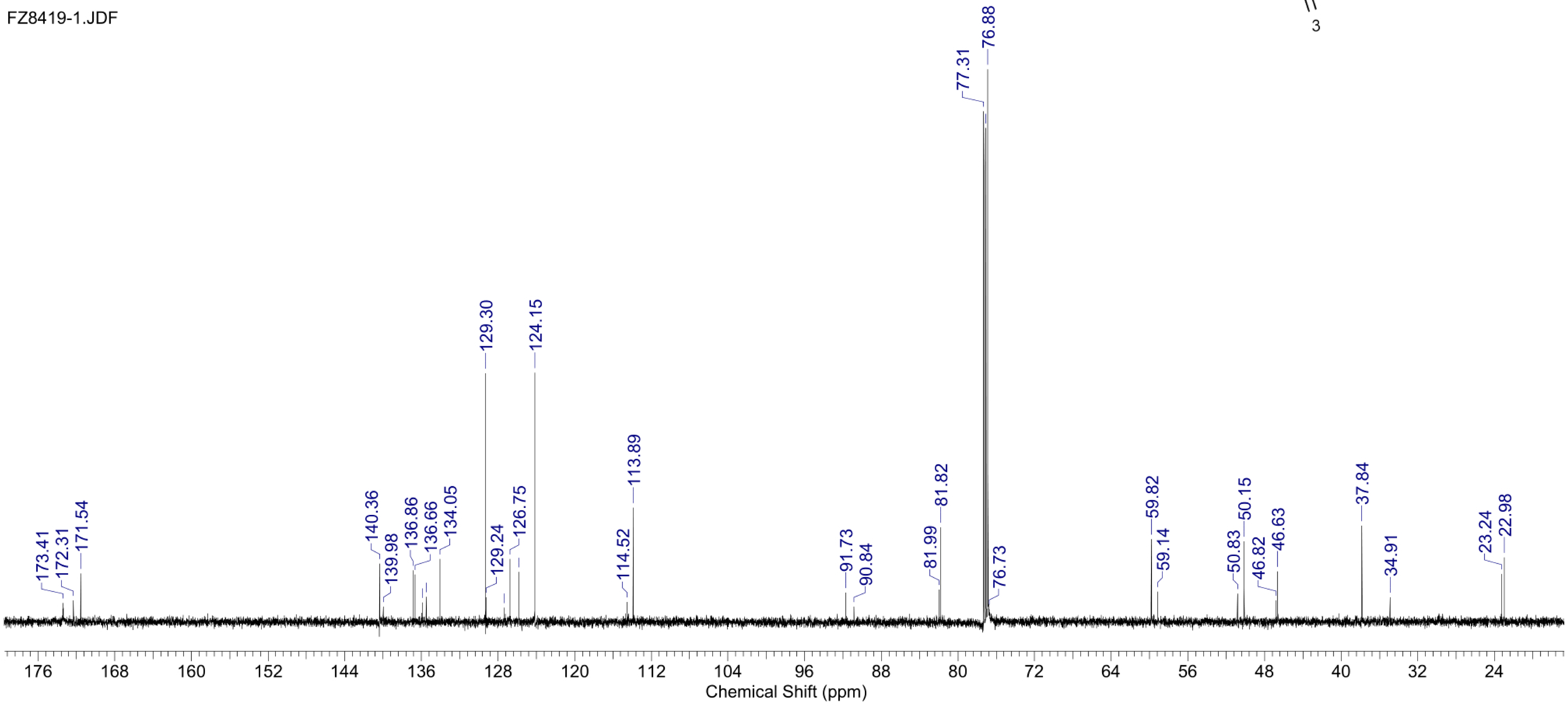

**Formula** $C_{19}H_{19}NO_4$	**FW** 325.3585

**Acquisition Time (sec)**	0.6921	**Comment**	single pulse decoupled gated NOE	**Date**	08 Feb 2020 00:19:56
**Date Stamp**	07 Feb 2020 17:53:54	**File Name**	C:\USERS\лаба 534\DOWNLOADS\FZ8419-1.JDF		
**Frequency (MHz)**	150.91	**Nucleus**	13C	**Number of Transients**	1000
**Origin**	ECA 600				
**Original Points Count**	32768	**Owner**	Mass	**Points Count**	32768
**Pulse Sequence**	single_pulse_dec				
**Receiver Gain**	56.00	**Solvent**	CHLOROFORM-d	**Spectrum Offset (Hz)**	15091.3428
**Sweep Width (Hz)**	47348.49	**Temperature (degree C)**	25.000		

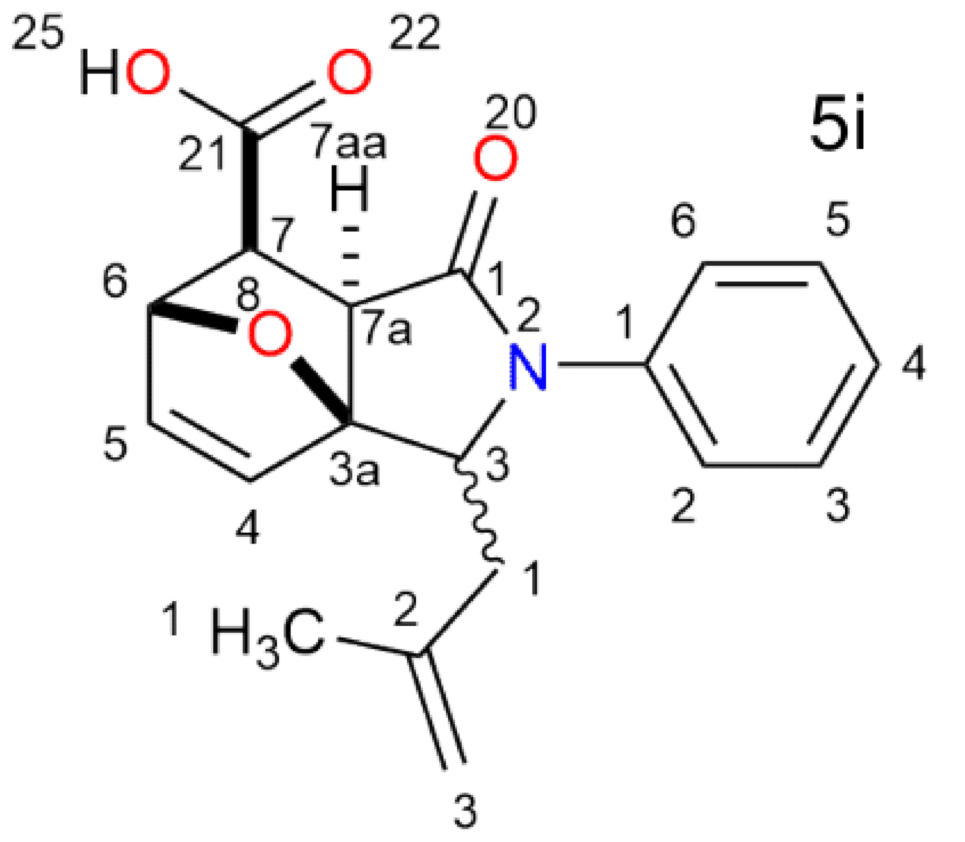

5i

FZ8419-1.JDF

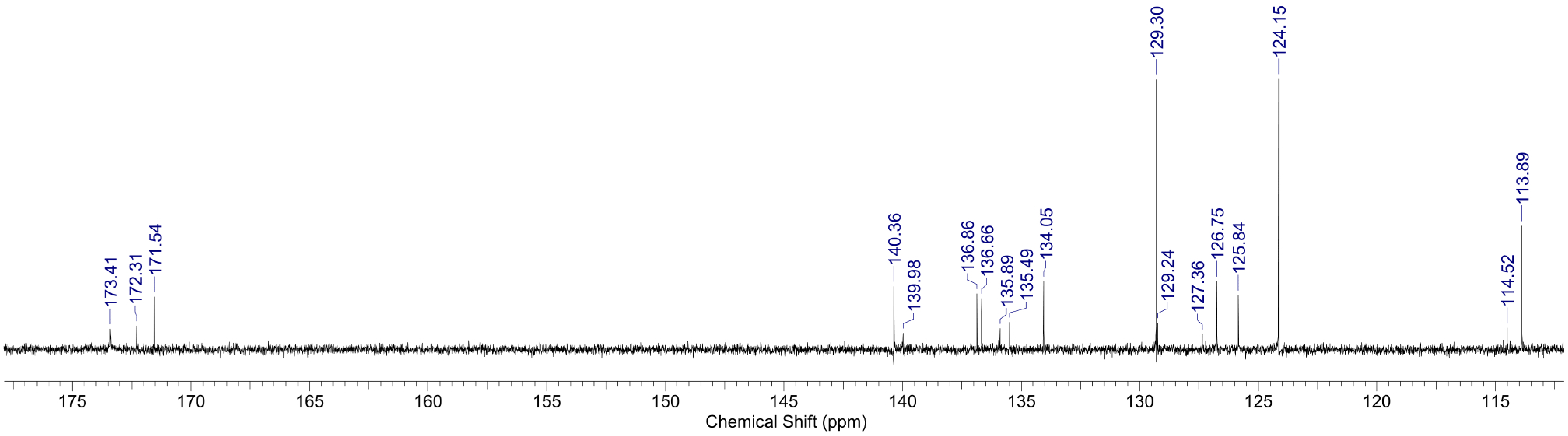

**Formula**	$C_{19}H_{19}NO_4$	**FW**	325.3585

**Acquisition Time (sec)**	0.6921	**Comment**	single pulse decoupled gated NOE	**Date**	08 Feb 2020 00:19:56
**Date Stamp**	07 Feb 2020 17:53:54	**File Name**	C:\USERS\лаба 534\DOWNLOADS\FZ8419-1.JDF		
**Frequency (MHz)**	150.91	**Nucleus**	13C	**Number of Transients**	1000
**Origin**	ECA 600				
**Original Points Count**	32768	**Owner**	Mass	**Points Count**	32768
**Pulse Sequence**	single_pulse_dec				
**Receiver Gain**	56.00	**Solvent**	CHLOROFORM-d	**Spectrum Offset (Hz)**	15091.3428
**Sweep Width (Hz)**	47348.49	**Temperature (degree C)**	25.000		

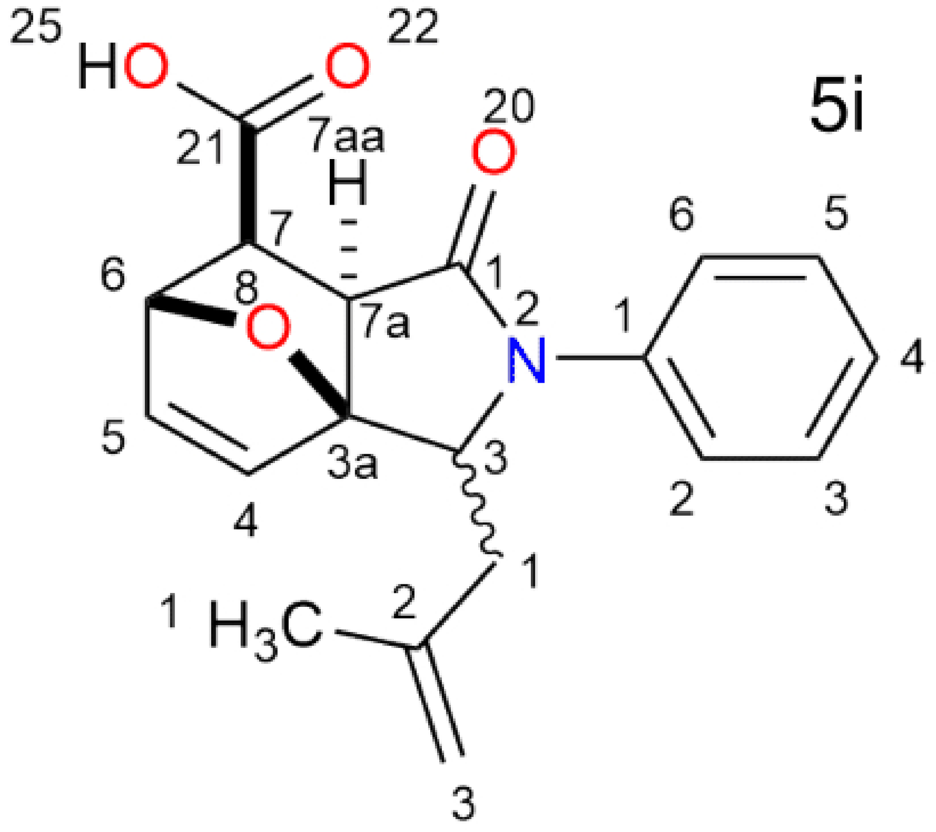

5i

FZ8419-1.JDF

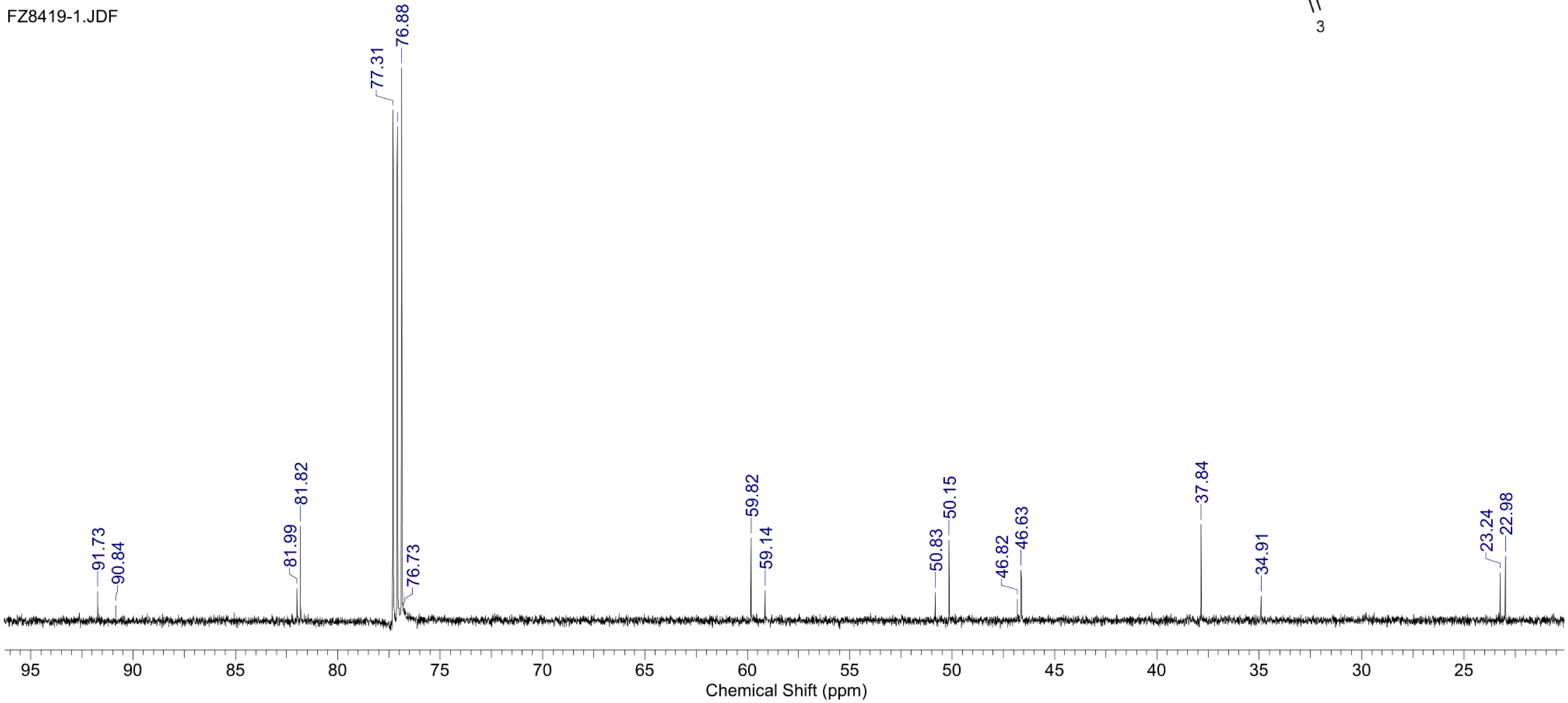

NMR spectrum data for compound 5m (DCM, −16 °C, 3 d)

**Formula** $C_{20}H_{21}NO_4$	**FW** 339.3850

**Acquisition Time (sec)**	1.9818	**Comment**	single_pulse	**Date**	28 Jul 2020 10:57:53			**Date Stamp**	28 Jul 2020 10:59:06
**File Name**	H:\DOWNLOADS\FZ8872-1.JDF			**Frequency (MHz)**	600.17	**Nucleus**	1H	**Number of Transients**	8
**Origin**	ECA 600	**Original Points Count**	32768	**Owner**	CKP	**Points Count**	32768	**Pulse Sequence**	single_pulse.ex2
**Receiver Gain**	38.00	**Solvent**	CHLOROFORM-d			**Spectrum Offset (Hz)**	5410.6274	**Sweep Width (Hz)**	16534.39

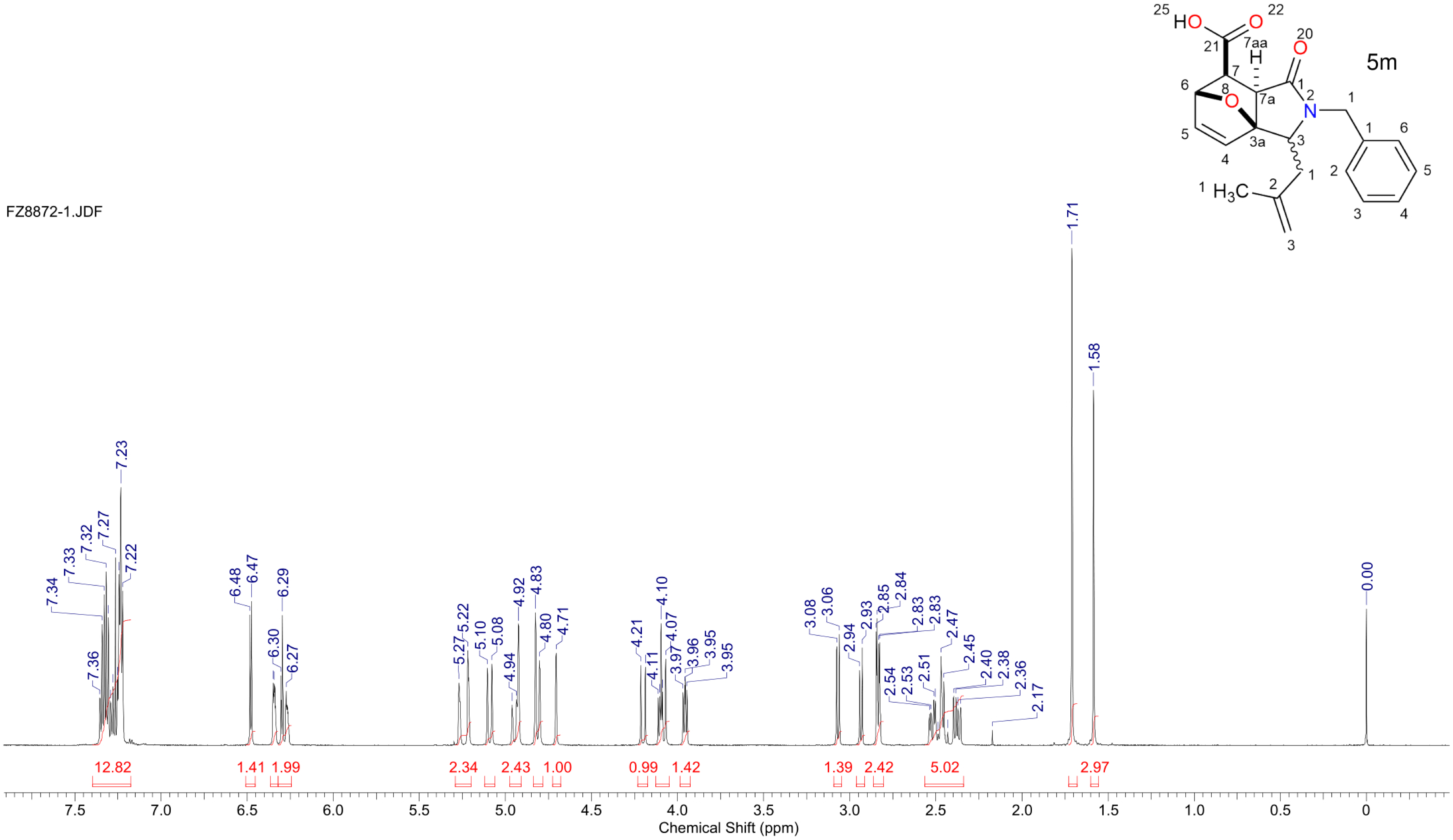

Formula $C_{20}H_{21}NO_4$	FW 339.3850

**Acquisition Time (sec)**	1.9818	**Comment**	single_pulse	**Date**	28 Jul 2020 10:57:53			**Date Stamp**	28 Jul 2020 10:59:06
**File Name**	H:\DOWNLOADS\FZ8872-1.JDF			**Frequency (MHz)**	600.17	**Nucleus**	1H	**Number of Transients**	8
**Origin**	ECA 600	**Original Points Count**	32768	**Owner**	CKP	**Points Count**	32768	**Pulse Sequence**	single_pulse.ex2
**Receiver Gain**	38.00	**Solvent**	CHLOROFORM-d			**Spectrum Offset (Hz)**	5410.6274	**Sweep Width (Hz)**	16534.39

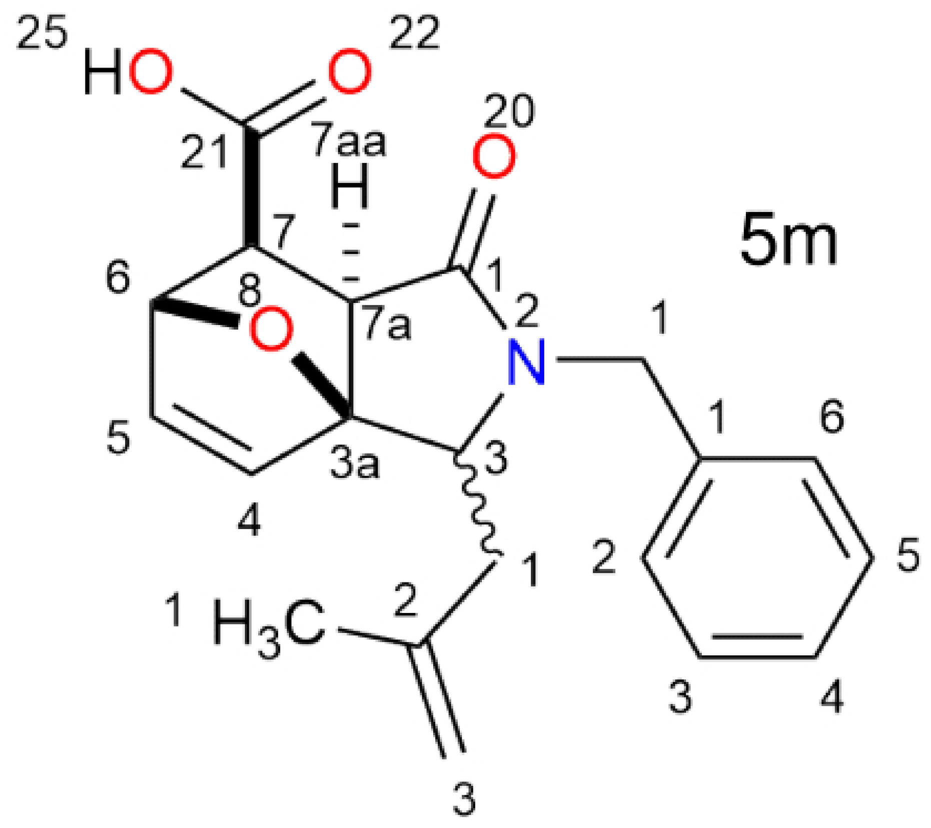

5m

FZ8872-1.JDF

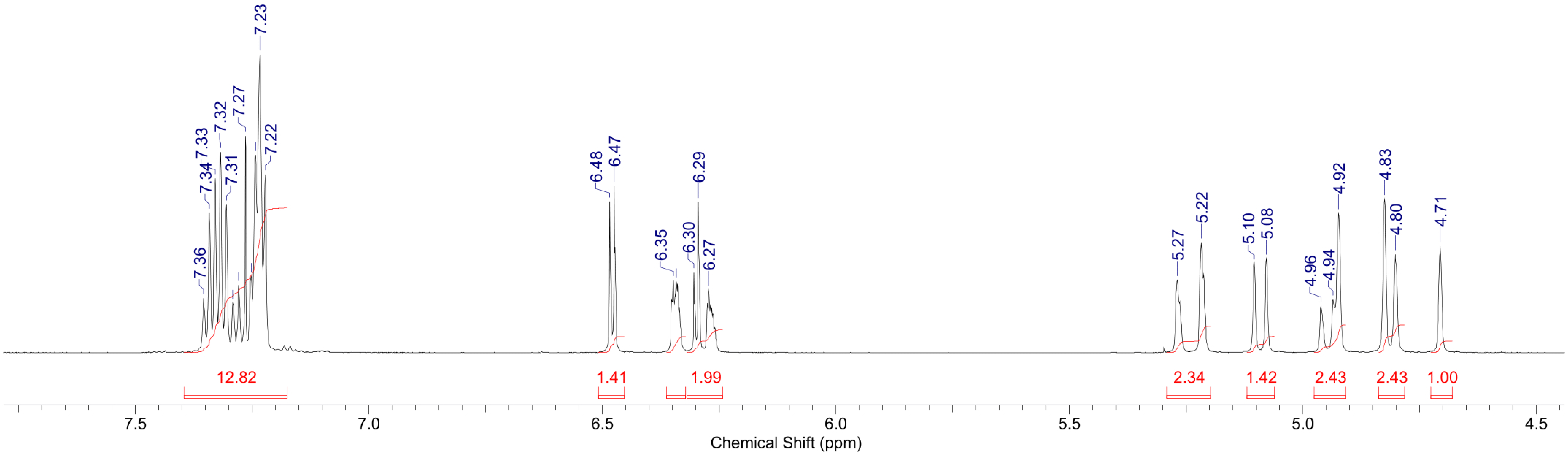

**Formula** $C_{20}H_{21}NO_4$	**FW** 339.3850

**Acquisition Time (sec)**	1.9818	**Comment**	single_pulse	**Date**	28 Jul 2020 10:57:53			**Date Stamp**	28 Jul 2020 10:59:06
**File Name**	H:\DOWNLOADS\FZ8872-1.JDF			**Frequency (MHz)**	600.17	**Nucleus**	1H	**Number of Transients**	8
**Origin**	ECA 600	**Original Points Count**	32768	**Owner**	CKP	**Points Count**	32768	**Pulse Sequence**	single_pulse.ex2
**Receiver Gain**	38.00	**Solvent**	CHLOROFORM-d			**Spectrum Offset (Hz)**	5410.6274	**Sweep Width (Hz)**	16534.39

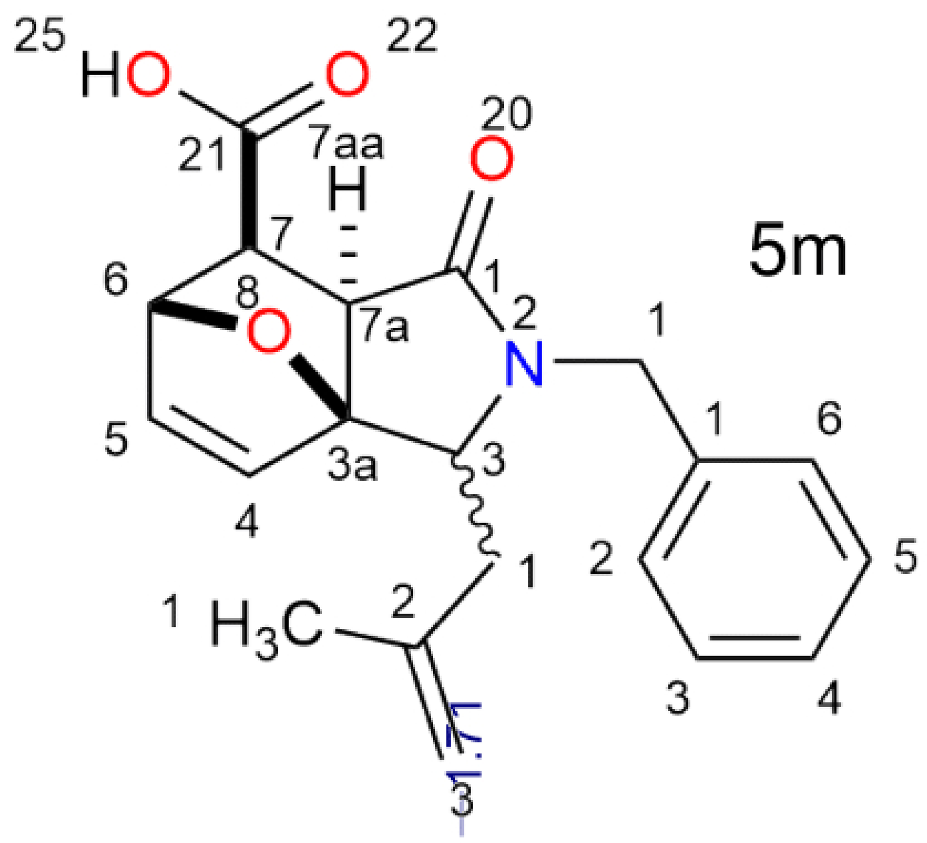

FZ8872-1.JDF

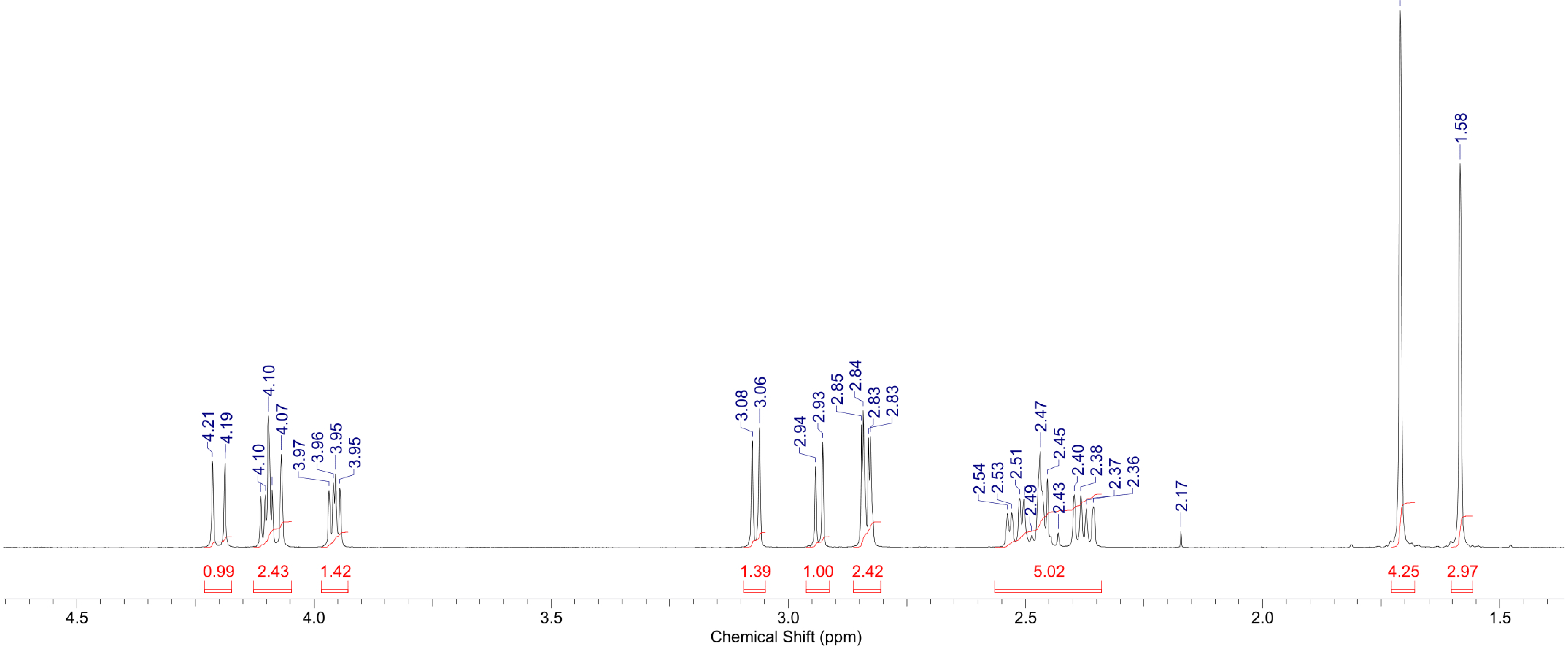

NMR spectrum data for compound 5m (DCM, r.t., 3 d)

Formula $C_{20}H_{21}NO_4$	***FW*** 339.3850		

Acquisition Time (sec)	1.9818	***Comment***	single_pulse	***Date***	17 Jul 2020 13:16:27		
Date Stamp	17 Jul 2020 13:18:16			***File Name***	F:\NMR FIDS ACIDS\BN METHALLYL\FZ8820-1.JDF		
Frequency (MHz)	600.17	***Nucleus***	1H	***Number of Transients***	8	***Origin***	ECA 600
Original Points Count	32768	***Owner***	CKP	***Points Count***	32768	***Pulse Sequence***	single_pulse.ex2
Receiver Gain	42.00	***Solvent***	CHLOROFORM-d			***Spectrum Offset (Hz)***	5410.6274
Sweep Width (Hz)	16534.39						

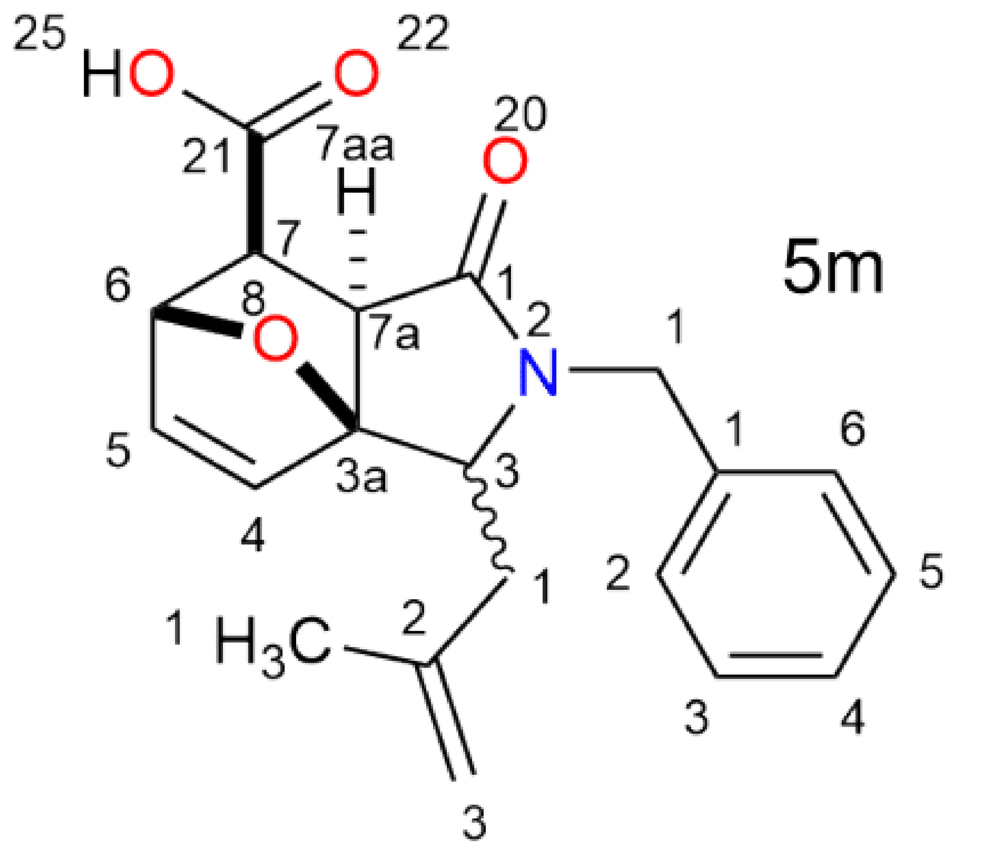

5m

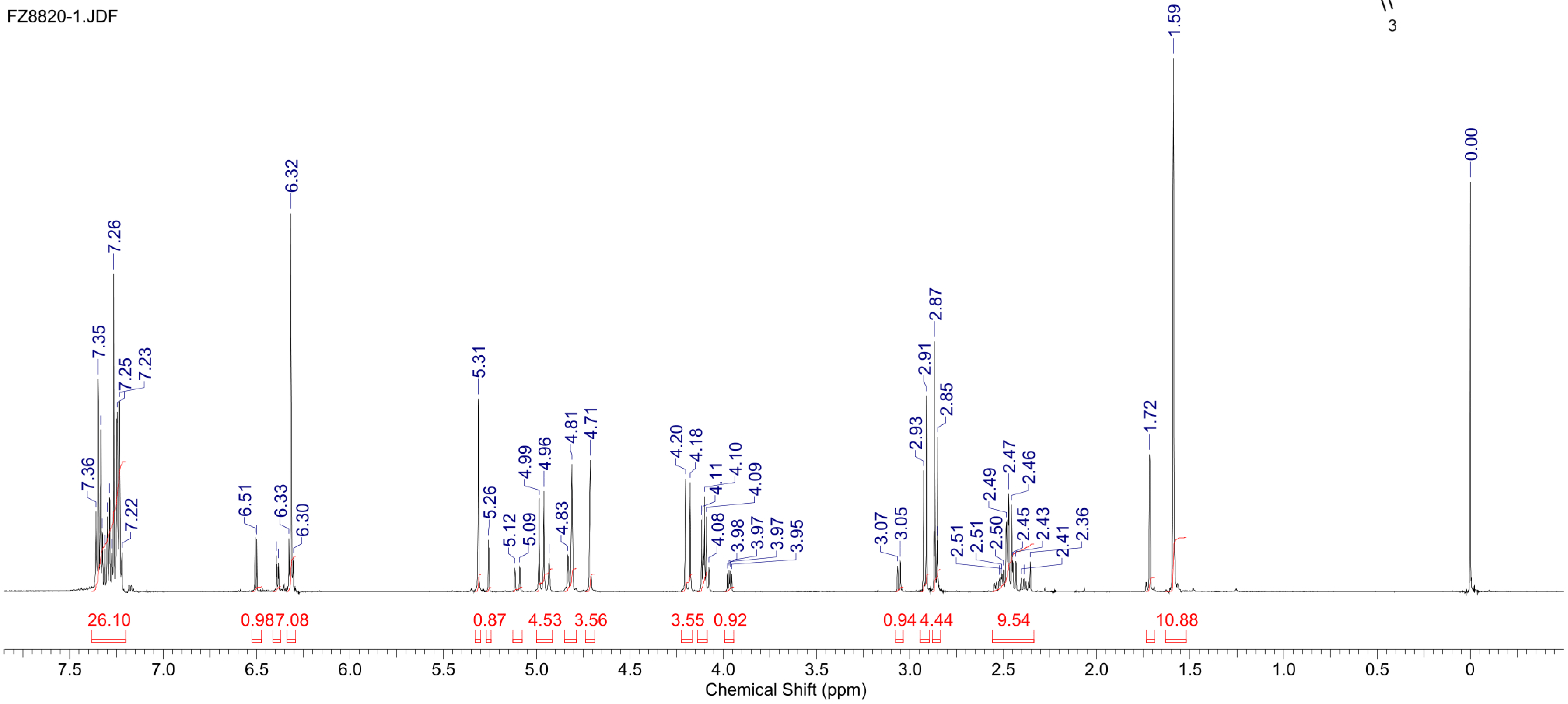

**Formula** $C_{20}H_{21}NO_4$	**FW** 339.3850

**Acquisition Time (sec)**	1.9818	**Comment**	single_pulse	**Date**	17 Jul 2020 13:16:27		
**Date Stamp**	17 Jul 2020 13:18:16			**File Name**	F:\NMR FIDS ACIDS\BN METHALLYL\FZ8820-1.JDF		
**Frequency (MHz)**	600.17	**Nucleus**	1H	**Number of Transients**	8	**Origin**	ECA 600
**Original Points Count**	32768	**Owner**	CKP	**Points Count**	32768	**Pulse Sequence**	single_pulse.ex2
**Receiver Gain**	42.00	**Solvent**	CHLOROFORM-d			**Spectrum Offset (Hz)**	5410.6274
**Sweep Width (Hz)**	16534.39						

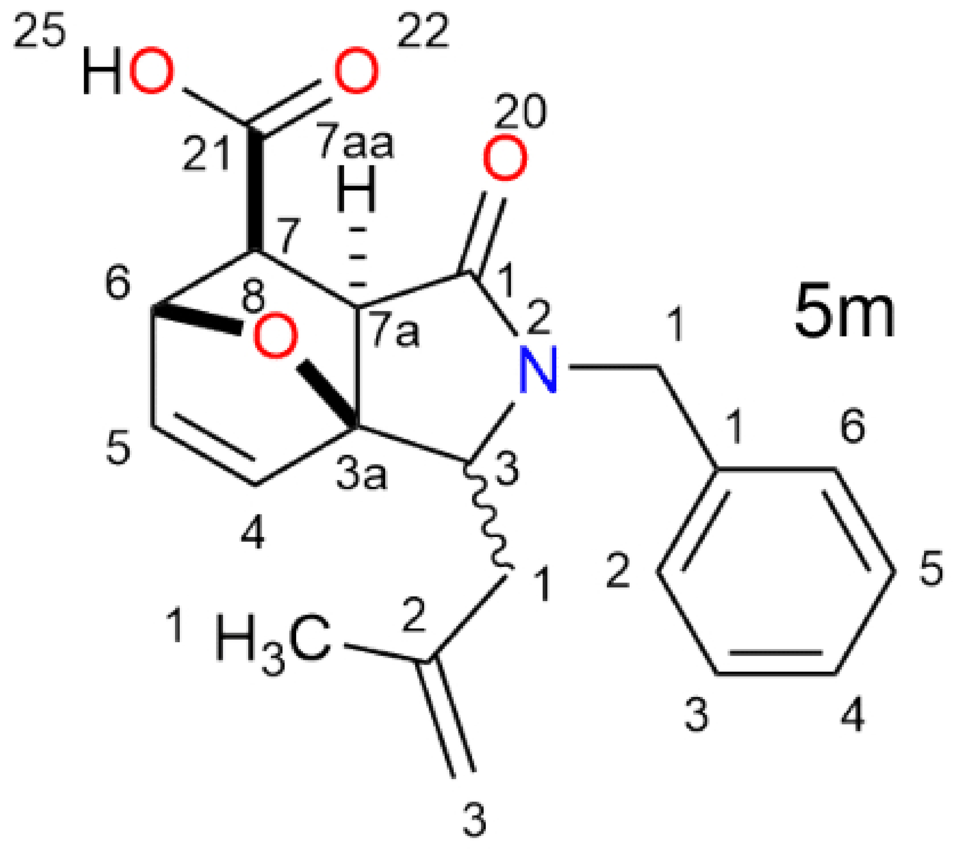

5m

FZ8820-1.JDF

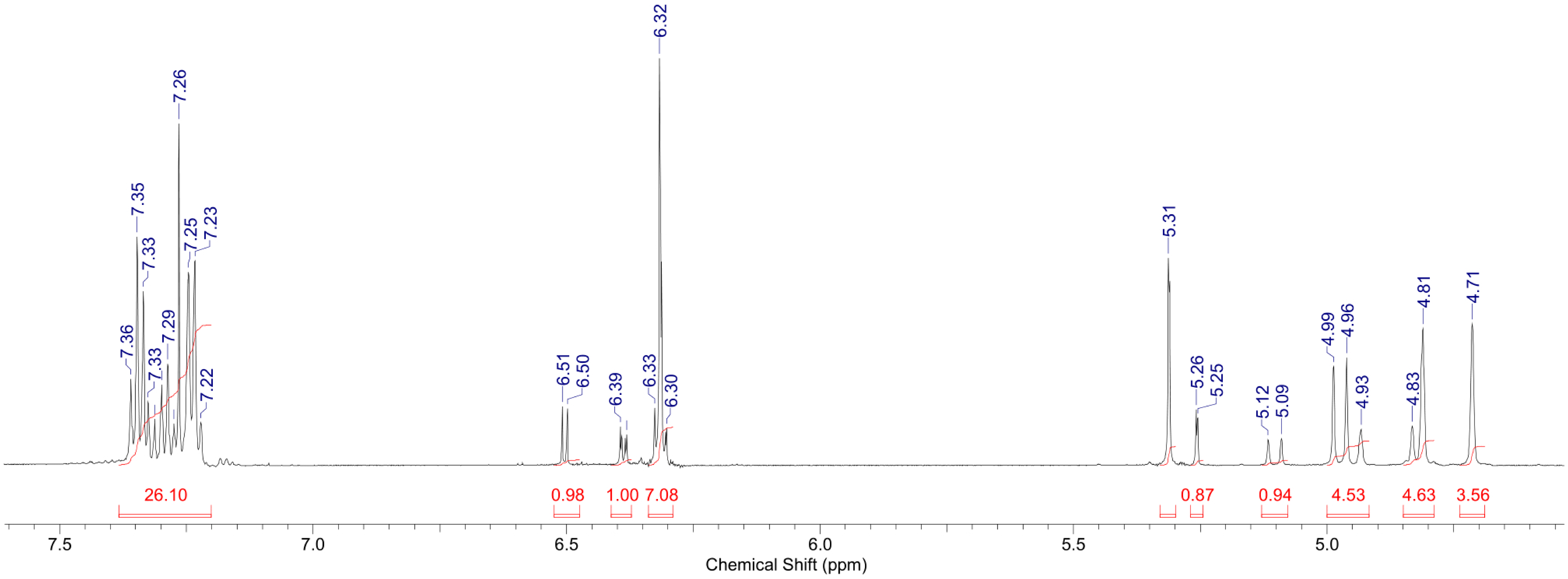

**Formula** $C_{20}H_{21}NO_4$	**FW** 339.3850

Acquisition Time (sec)	1.9818	***Comment***	single_pulse	***Date***	17 Jul 2020 13:16:27		
Date Stamp	17 Jul 2020 13:18:16			***File Name***	F:\NMR FIDS ACIDS\BN METHALLYL\FZ8820-1.JDF		
Frequency (MHz)	600.17	***Nucleus***	1H	***Number of Transients***	8	***Origin***	ECA 600
Original Points Count	32768	***Owner***	CKP	***Points Count***	32768	***Pulse Sequence***	single_pulse.ex2
Receiver Gain	42.00	***Solvent***	CHLOROFORM-d			***Spectrum Offset (Hz)***	5410.6274
Sweep Width (Hz)	16534.39						

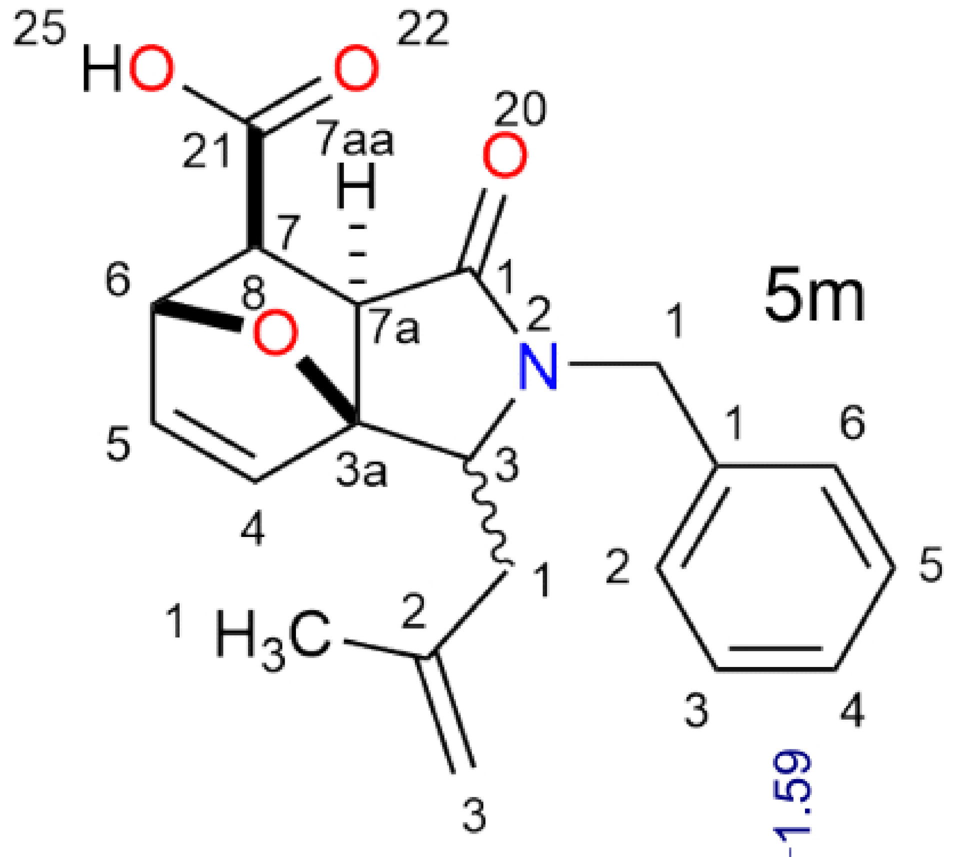

FZ8820-1.JDF

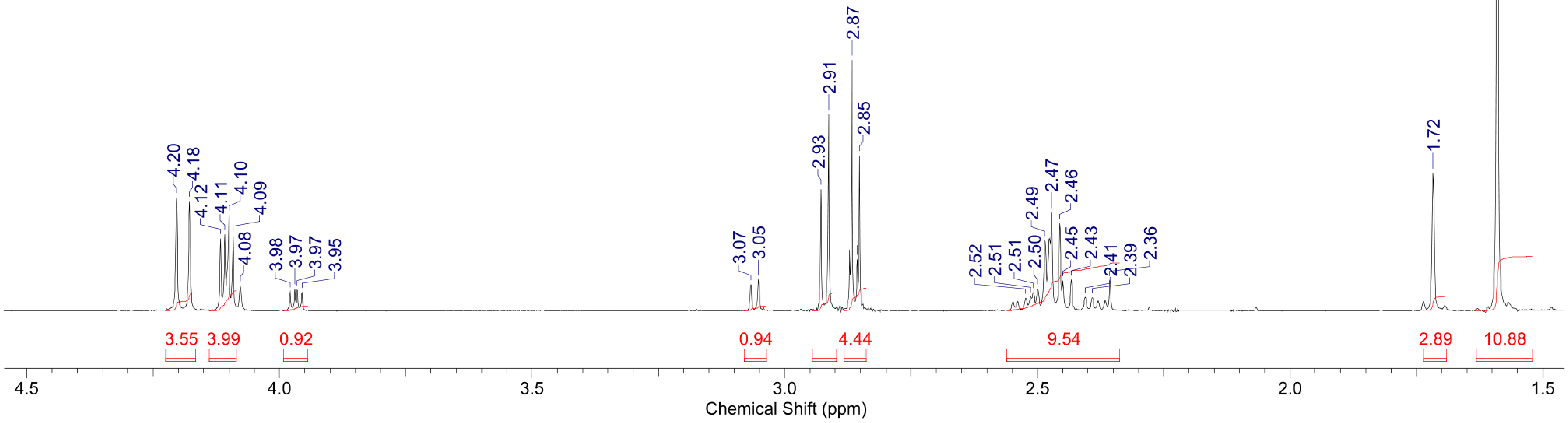

NMR spectrum data for compound 5m (MeCN, r.t., 3 d)

**Formula**	$C_{20}H_{21}NO_4$	**FW**	339.3850

**Acquisition Time (sec)**	1.9818	**Comment**	single_pulse	**Date**	17 Jul 2020 13:35:19		
**Date Stamp**	17 Jul 2020 13:37:08			**File Name**	F:\NMR FIDS ACIDS\BN METHALLYL\FZ8823-1.JDF		
**Frequency (MHz)**	600.17	**Nucleus**	1H	**Number of Transients**	8	**Origin**	ECA 600
**Original Points Count**	32768	**Owner**	CKP	**Points Count**	32768	**Pulse Sequence**	single_pulse.ex2
**Receiver Gain**	40.00	**Solvent**	CHLOROFORM-d			**Spectrum Offset (Hz)**	5410.6274
**Sweep Width (Hz)**	16534.39						

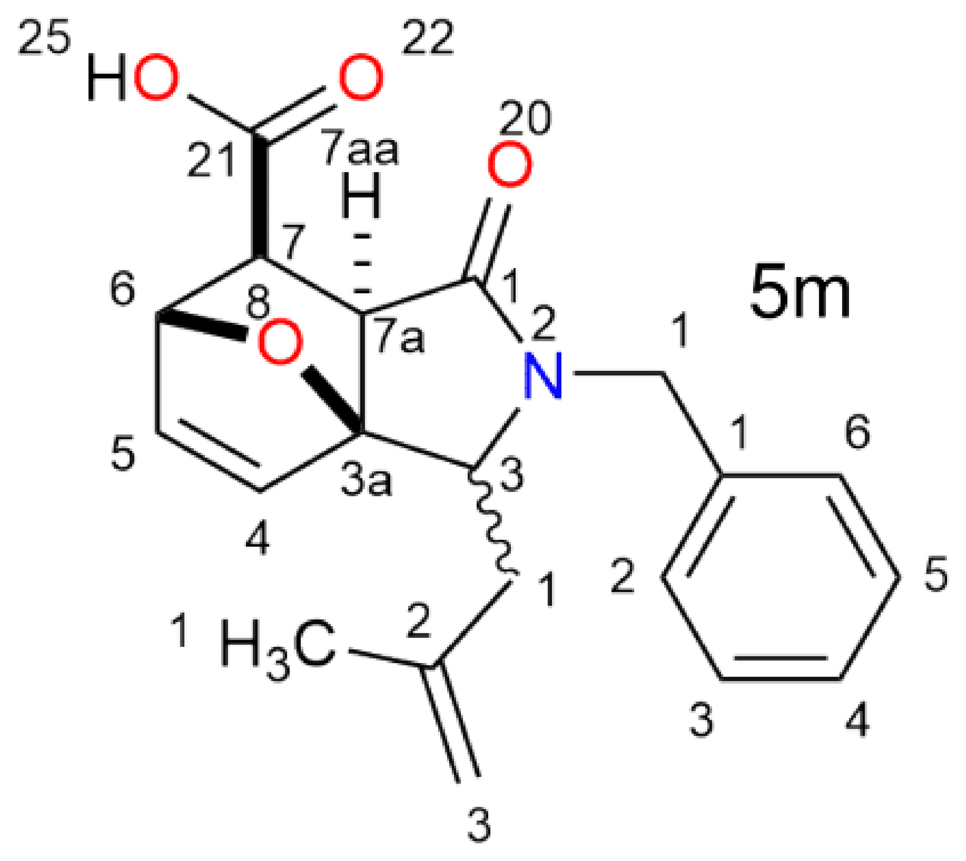

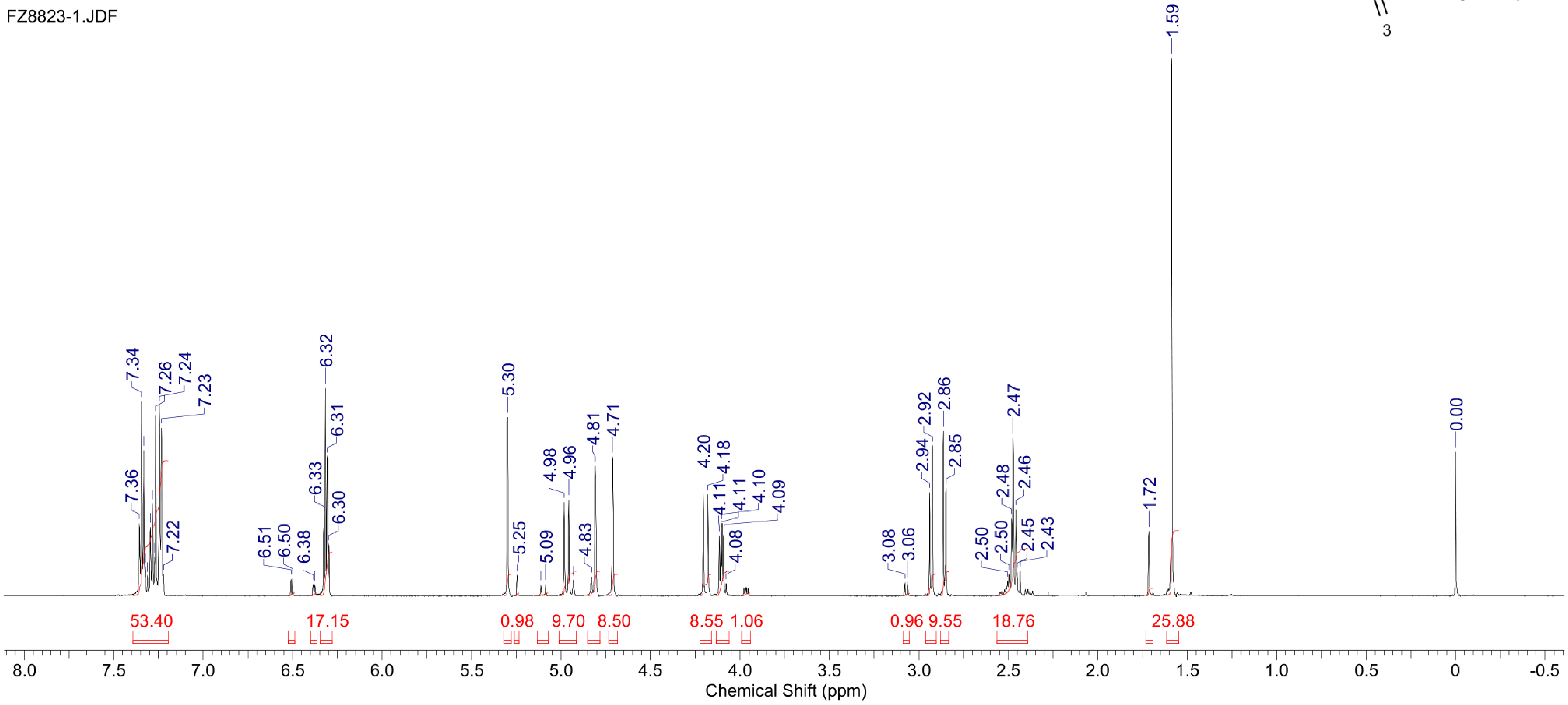

Formula	$C_{20}H_{21}NO_4$	FW	339.3850

**Acquisition Time (sec)**	1.9818	**Comment**	single_pulse	**Date**	17 Jul 2020 13:35:19		
**Date Stamp**	17 Jul 2020 13:37:08			**File Name**	F:\NMR FIDS ACIDS\BN METHALLYL\FZ8823-1.JDF		
**Frequency (MHz)**	600.17	**Nucleus**	1H	**Number of Transients**	8	**Origin**	ECA 600
**Original Points Count**	32768	**Owner**	CKP	**Points Count**	32768	**Pulse Sequence**	single_pulse.ex2
**Receiver Gain**	40.00	**Solvent**	CHLOROFORM-d			**Spectrum Offset (Hz)**	5410.6274
**Sweep Width (Hz)**	16534.39						

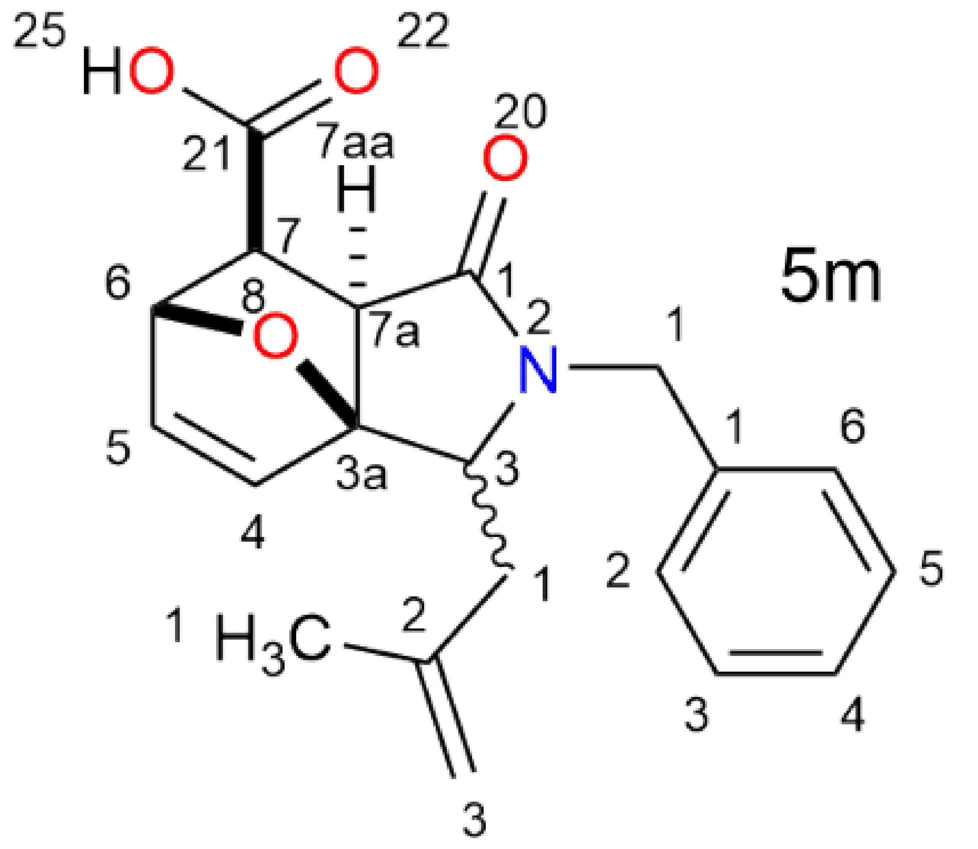

FZ8823-1.JDF

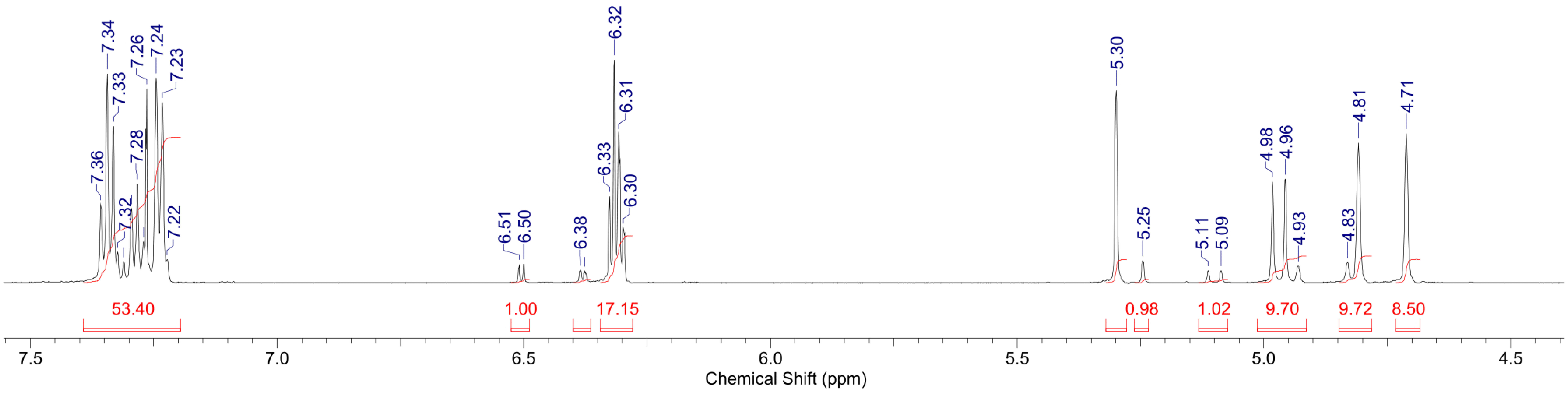

Formula $C_{20}H_{21}NO_4$	FW 339.3850

**Acquisition Time (sec)**	1.9818	**Comment**	single_pulse	**Date**	17 Jul 2020 13:35:19		
**Date Stamp**	17 Jul 2020 13:37:08			**File Name**	F:\NMR FIDS ACIDS\BN METHALLYL\FZ8823-1.JDF		
**Frequency (MHz)**	600.17	**Nucleus**	1H	**Number of Transients**	8	**Origin**	ECA 600
**Original Points Count**	32768	**Owner**	CKP	**Points Count**	32768	**Pulse Sequence**	single_pulse.ex2
**Receiver Gain**	40.00	**Solvent**	CHLOROFORM-d			**Spectrum Offset (Hz)**	5410.6274
**Sweep Width (Hz)**	16534.39						

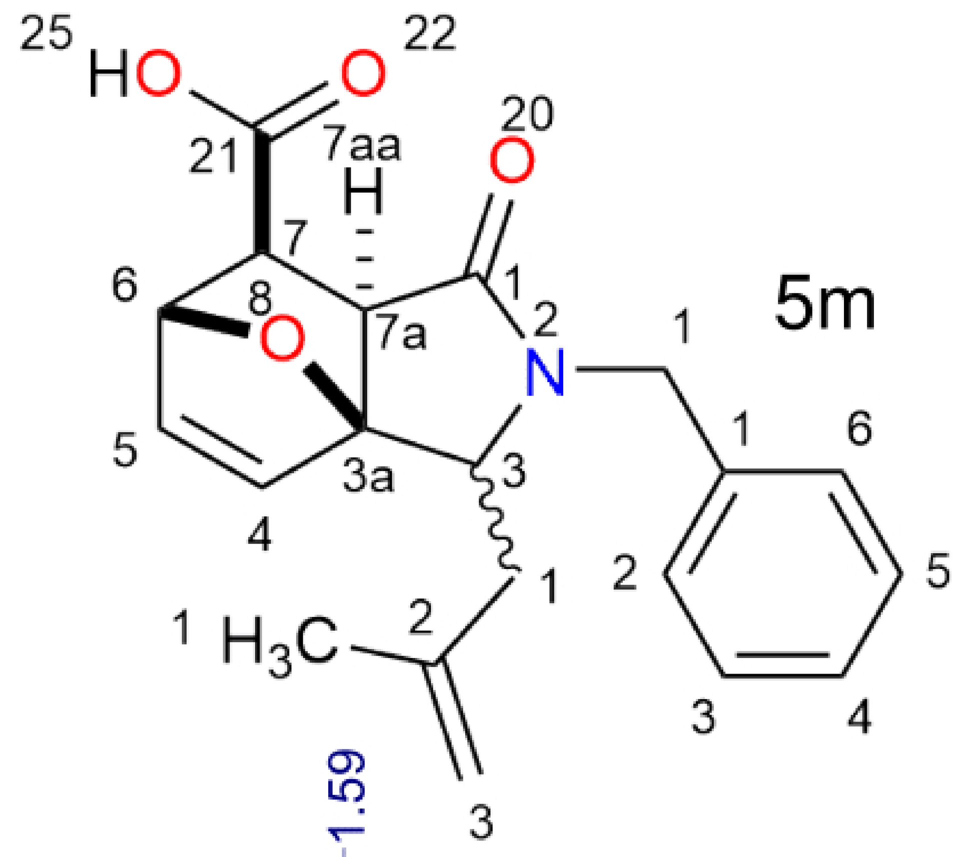

5m

FZ8823-1.JDF

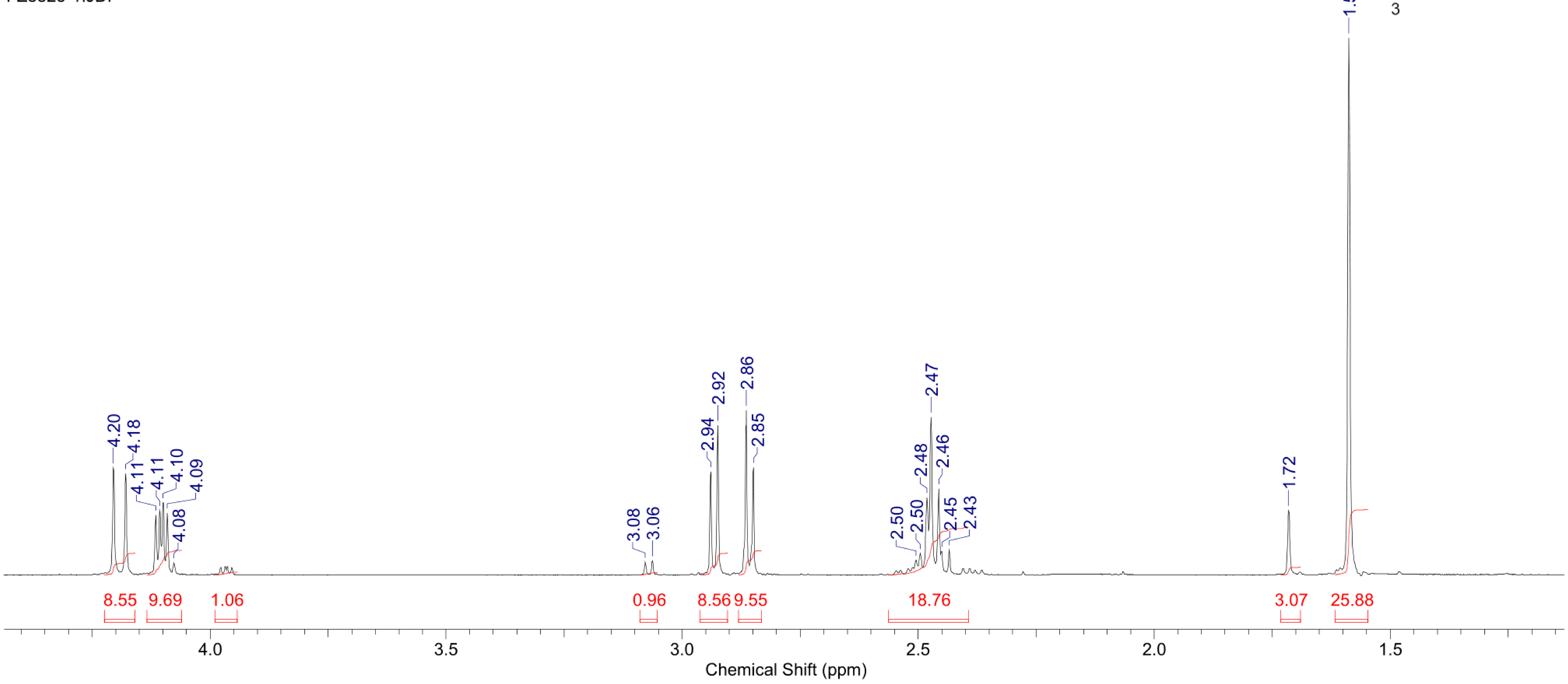

NMR spectrum data for compound 5m (MeCN, Δ, 3 h)

Formula $C_{20}H_{21}NO_4$	***FW*** 339.3850		

Acquisition Time (sec)	1.9818	***Comment***	single_pulse	***Date***	17 Jul 2020 12:46:09		
Date Stamp	17 Jul 2020 12:47:57			***File Name***	F:\NMR FIDS ACIDS\BN METHALLYL\FZ8809-1.JDF		
Frequency (MHz)	600.17	***Nucleus***	1H	***Number of Transients***	8	***Origin***	ECA 600
Original Points Count	32768	***Owner***	CKP	***Points Count***	32768	***Pulse Sequence***	single_pulse.ex2
Receiver Gain	44.00	***Solvent***	CHLOROFORM-d			***Spectrum Offset (Hz)***	5409.6182
Sweep Width (Hz)	16534.39						

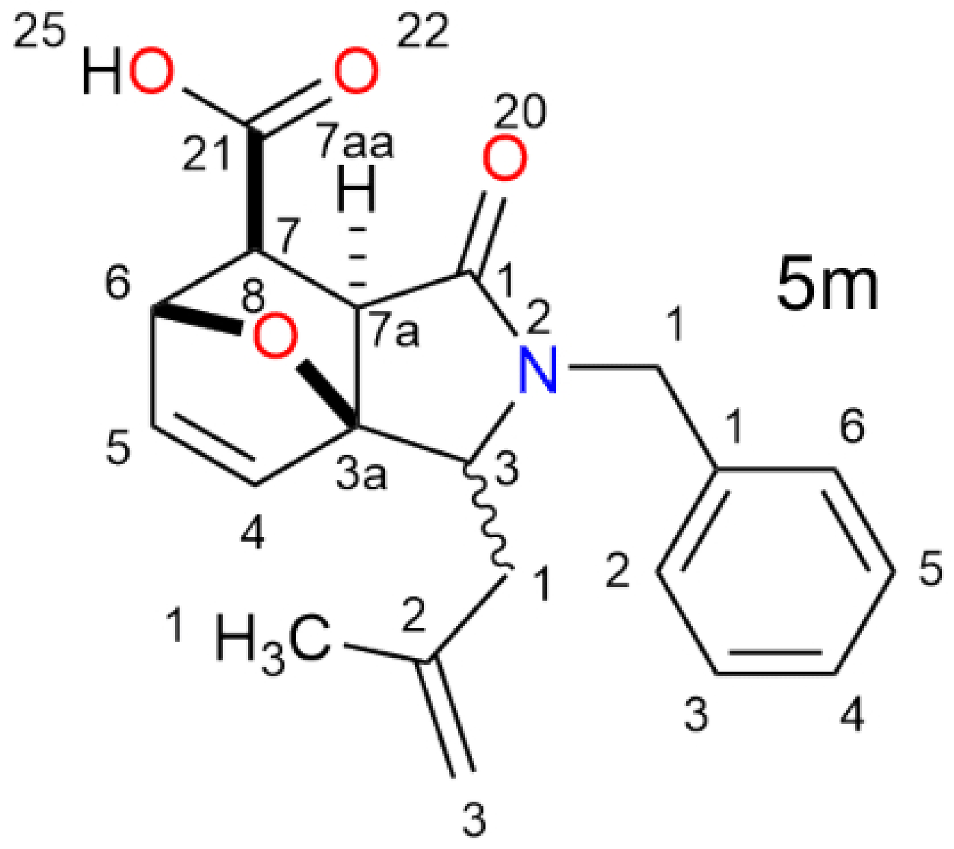

FZ8809-1.JDF

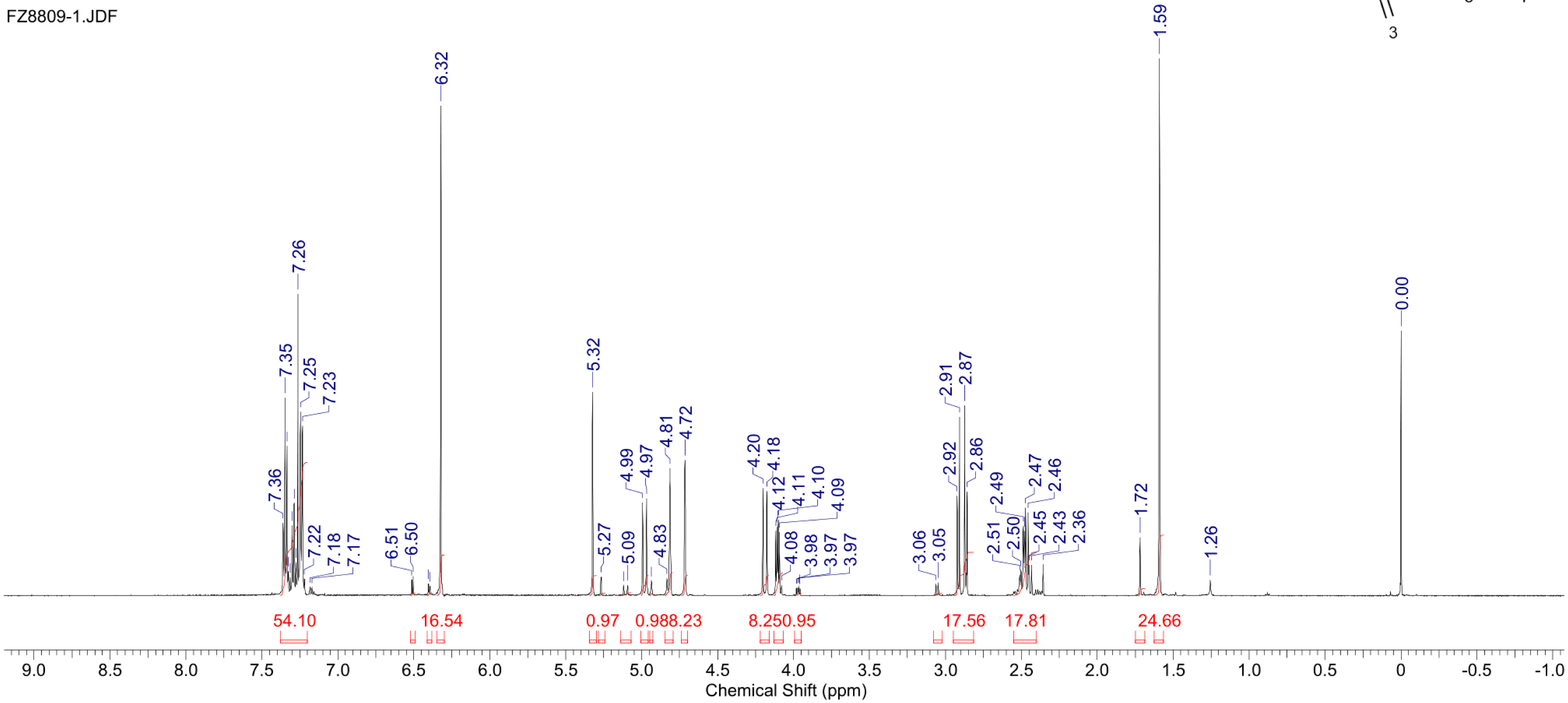

Formula	$C_{20}H_{21}NO_4$	FW	339.3850

Acquisition Time (sec)	1.9818	***Comment***	single_pulse	***Date***	17 Jul 2020 12:46:09		
Date Stamp	17 Jul 2020 12:47:57			***File Name***	F:\NMR FIDS ACIDS\BN METHALLYL\FZ8809-1.JDF		
Frequency (MHz)	600.17	***Nucleus***	1H	***Number of Transients***	8	***Origin***	ECA 600
Original Points Count	32768	***Owner***	CKP	***Points Count***	32768	***Pulse Sequence***	single_pulse.ex2
Receiver Gain	44.00	***Solvent***	CHLOROFORM-d			***Spectrum Offset (Hz)***	5409.6182
Sweep Width (Hz)	16534.39						

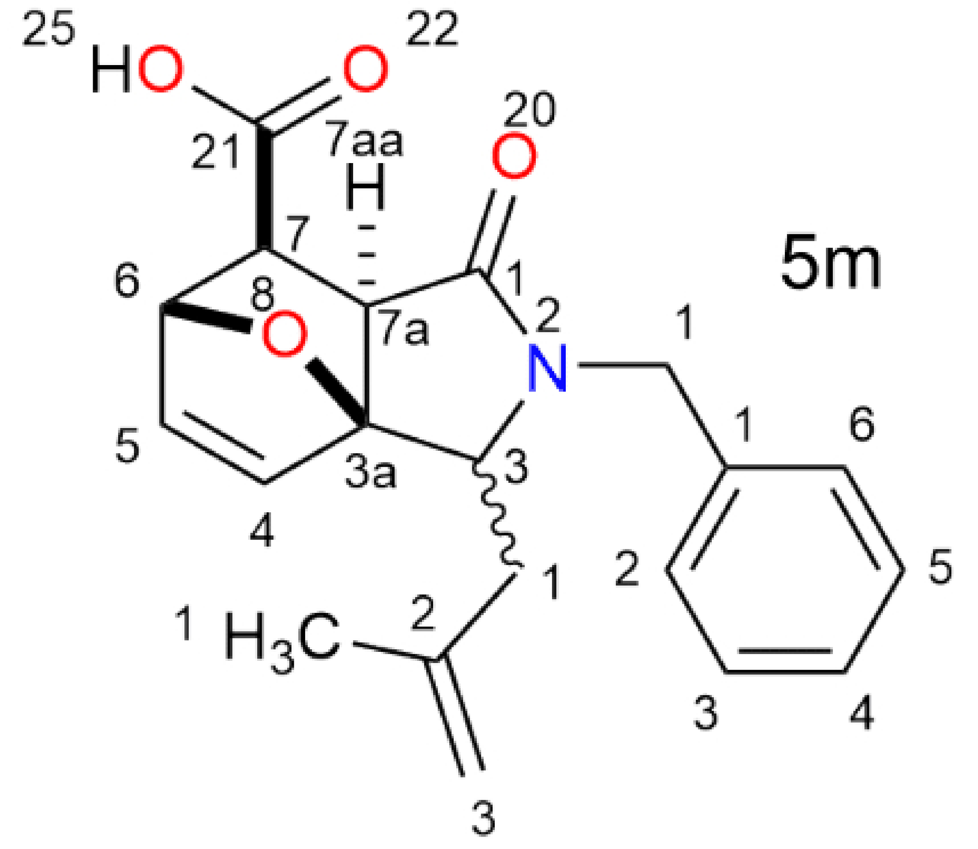

5m

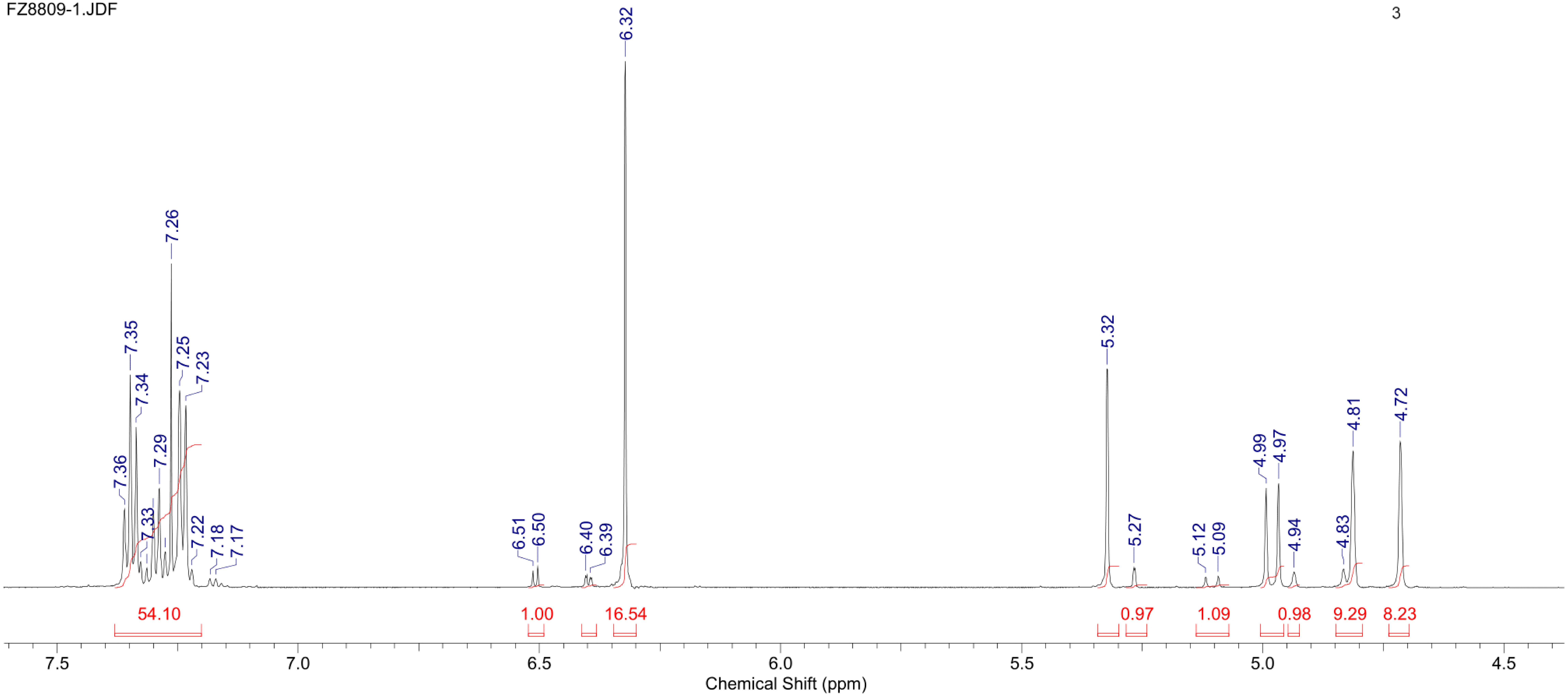

**Formula** $C_{20}H_{21}NO_4$	**FW** 339.3850

**Acquisition Time (sec)**	1.9818	**Comment**	single_pulse	**Date**	17 Jul 2020 12:46:09		
**Date Stamp**	17 Jul 2020 12:47:57			**File Name**	F:\NMR FIDS ACIDS\BN METHALLYL\FZ8809-1.JDF		
**Frequency (MHz)**	600.17	**Nucleus**	1H	**Number of Transients**	8	**Origin**	ECA 600
**Original Points Count**	32768	**Owner**	CKP	**Points Count**	32768	**Pulse Sequence**	single_pulse.ex2
**Receiver Gain**	44.00	**Solvent**	CHLOROFORM-d			**Spectrum Offset (Hz)**	5409.6182
**Sweep Width (Hz)**	16534.39						

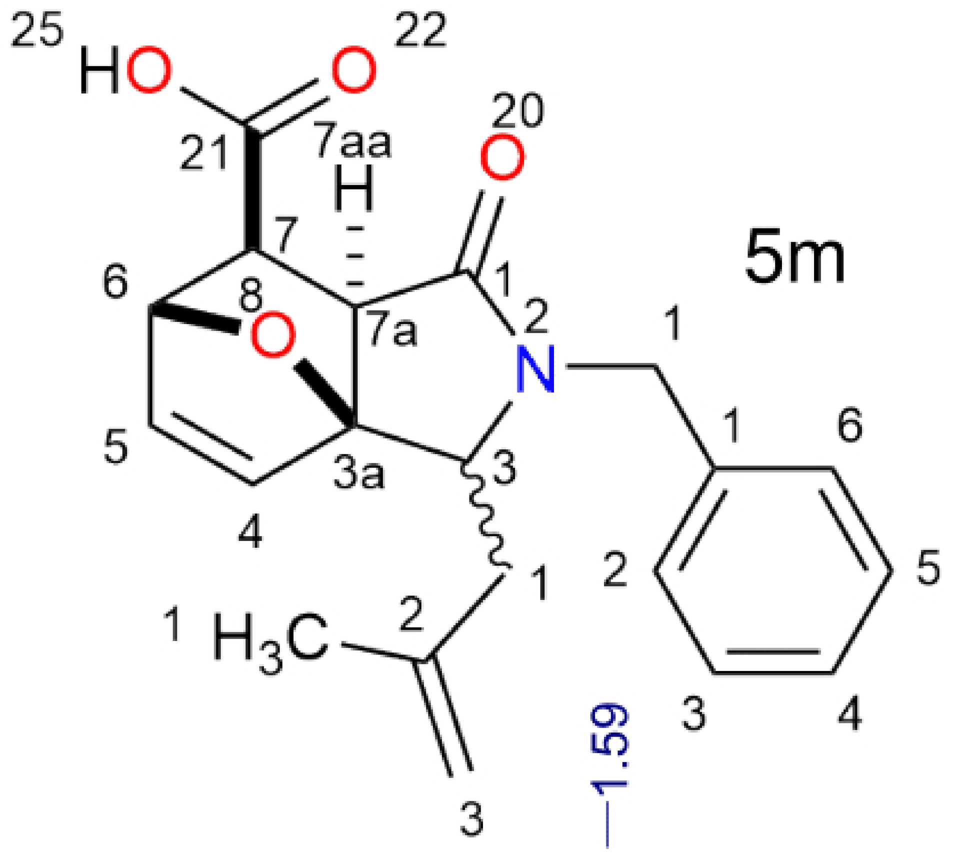

5m

FZ8809-1.JDF

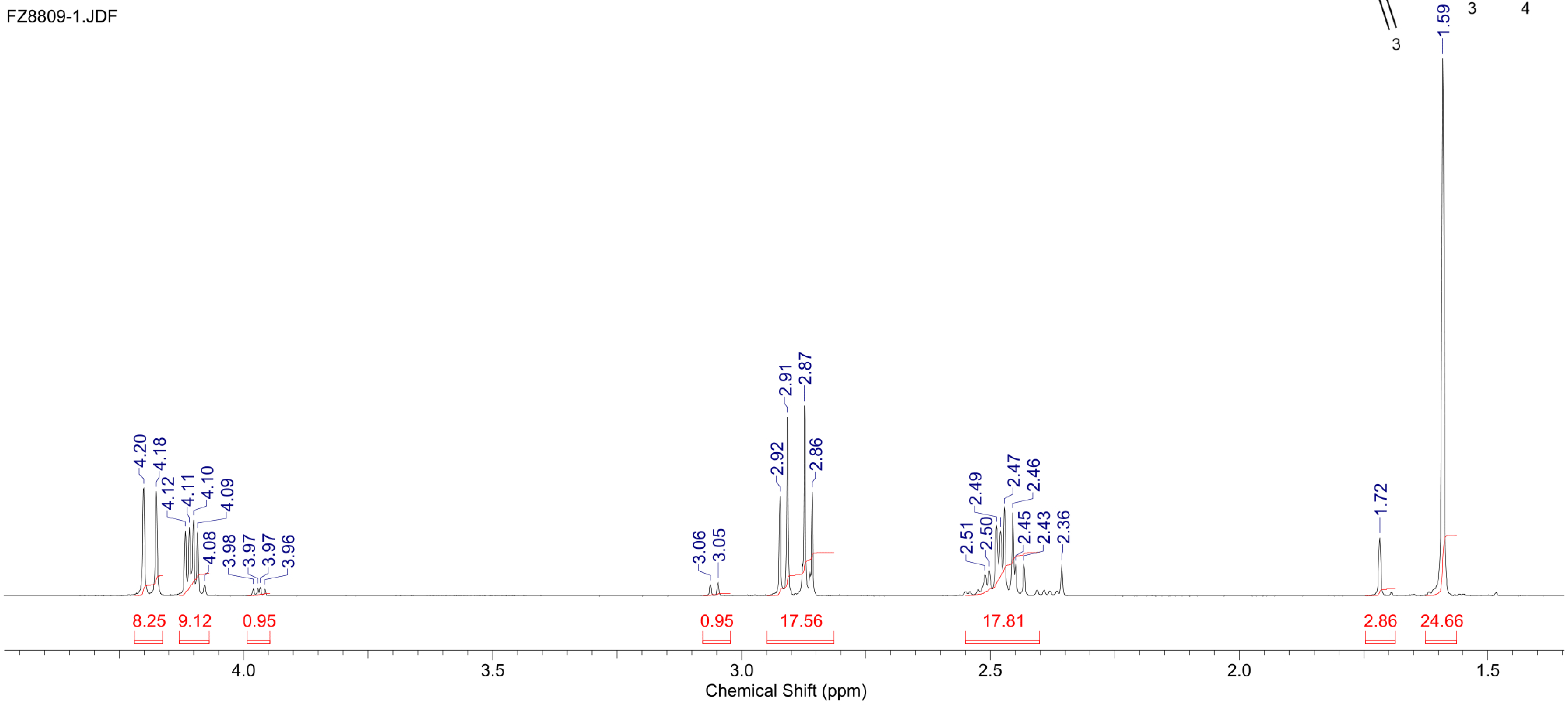

NMR spectrum data for compound 5m (PhH, Δ, 3 h)

Formula $C_{20}H_{21}NO_4$	FW 339.3850

**Acquisition Time (sec)**	1.9818	**Comment**	single_pulse	**Date**	17 Jul 2020 12:52:03		
**Date Stamp**	17 Jul 2020 12:53:51			**File Name**	F:\NMR FIDS ACIDS\BN METHALLYL\FZ8810-1.JDF		
**Frequency (MHz)**	600.17	**Nucleus**	1H	**Number of Transients**	8	**Origin**	ECA 600
**Original Points Count**	32768	**Owner**	CKP	**Points Count**	32768	**Pulse Sequence**	single_pulse.ex2
**Receiver Gain**	46.00	**Solvent**	CHLOROFORM-d			**Spectrum Offset (Hz)**	5409.6182
**Sweep Width (Hz)**	16534.39						

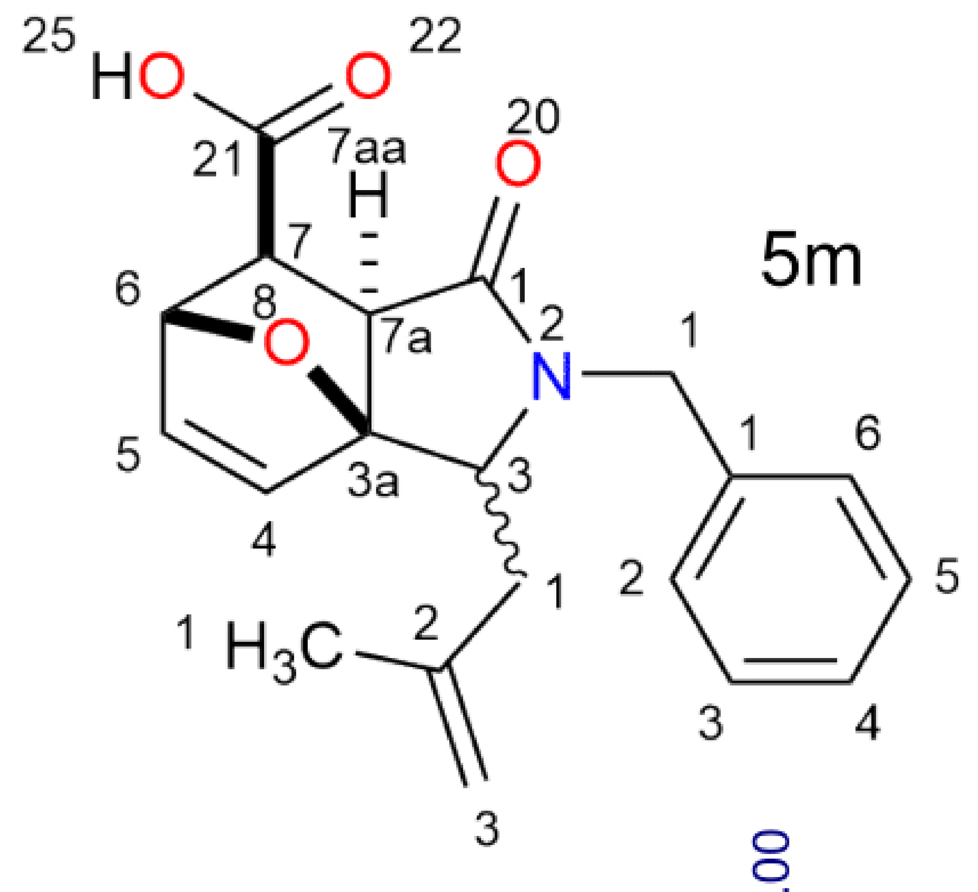

5m

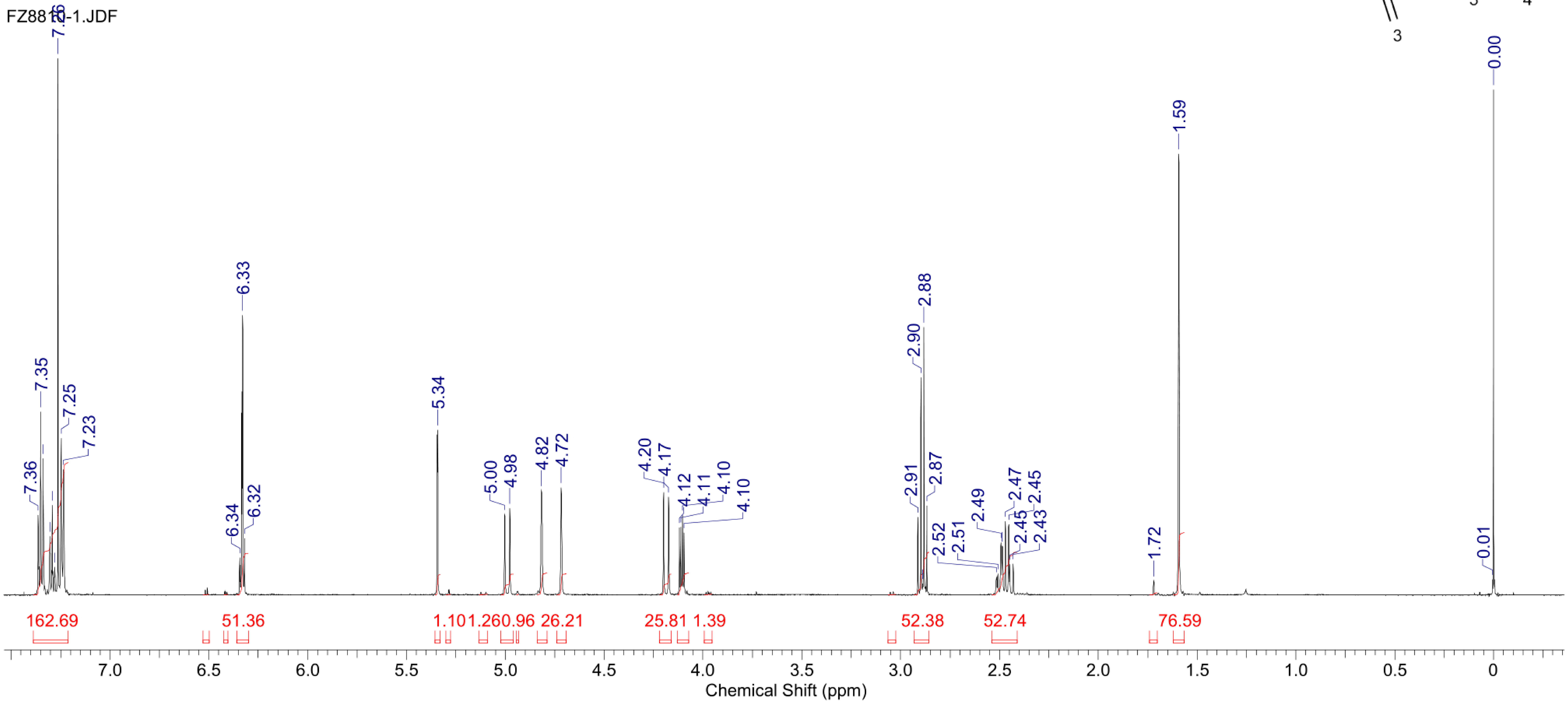

Formula $C_{20}H_{21}NO_4$	FW 339.3850

Acquisition Time (sec)	1.9818	***Comment***	single_pulse	***Date***	17 Jul 2020 12:52:03		
Date Stamp	17 Jul 2020 12:53:51			***File Name***	F:\NMR FIDS ACIDS\BN METHALLYL\FZ8810-1.JDF		
Frequency (MHz)	600.17	***Nucleus***	1H	***Number of Transients***	8	***Origin***	ECA 600
Original Points Count	32768	***Owner***	CKP	***Points Count***	32768	***Pulse Sequence***	single_pulse.ex2
Receiver Gain	46.00	***Solvent***	CHLOROFORM-d			***Spectrum Offset (Hz)***	5409.6182
Sweep Width (Hz)	16534.39						

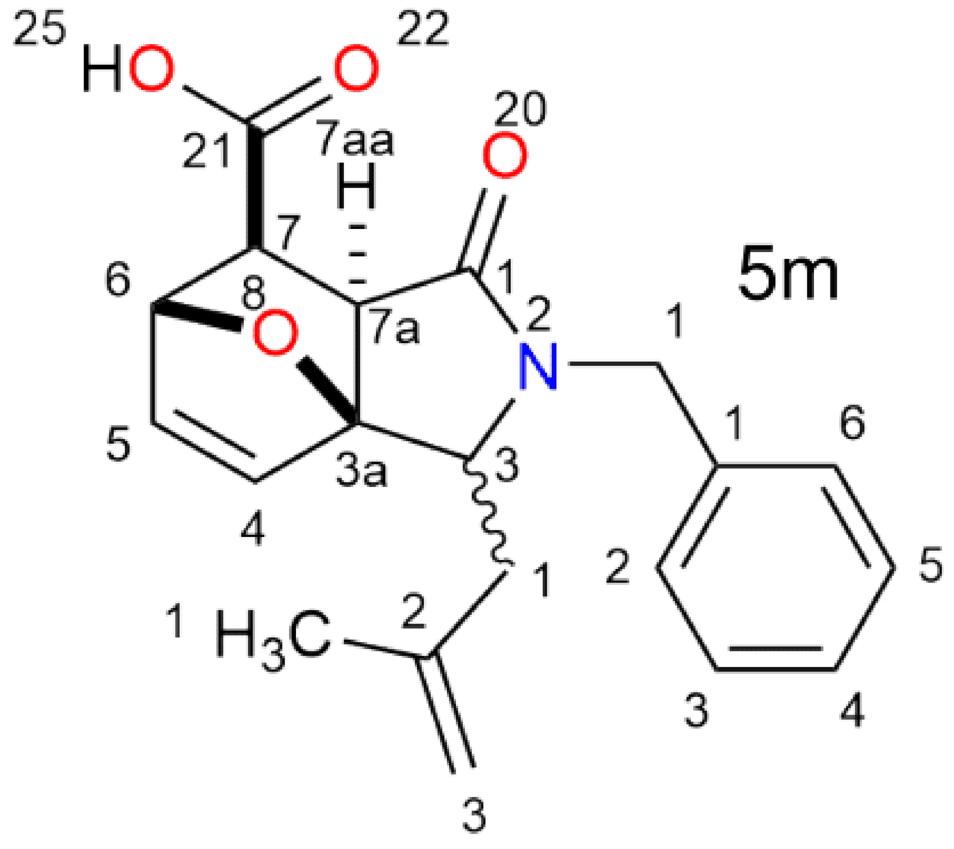

5m

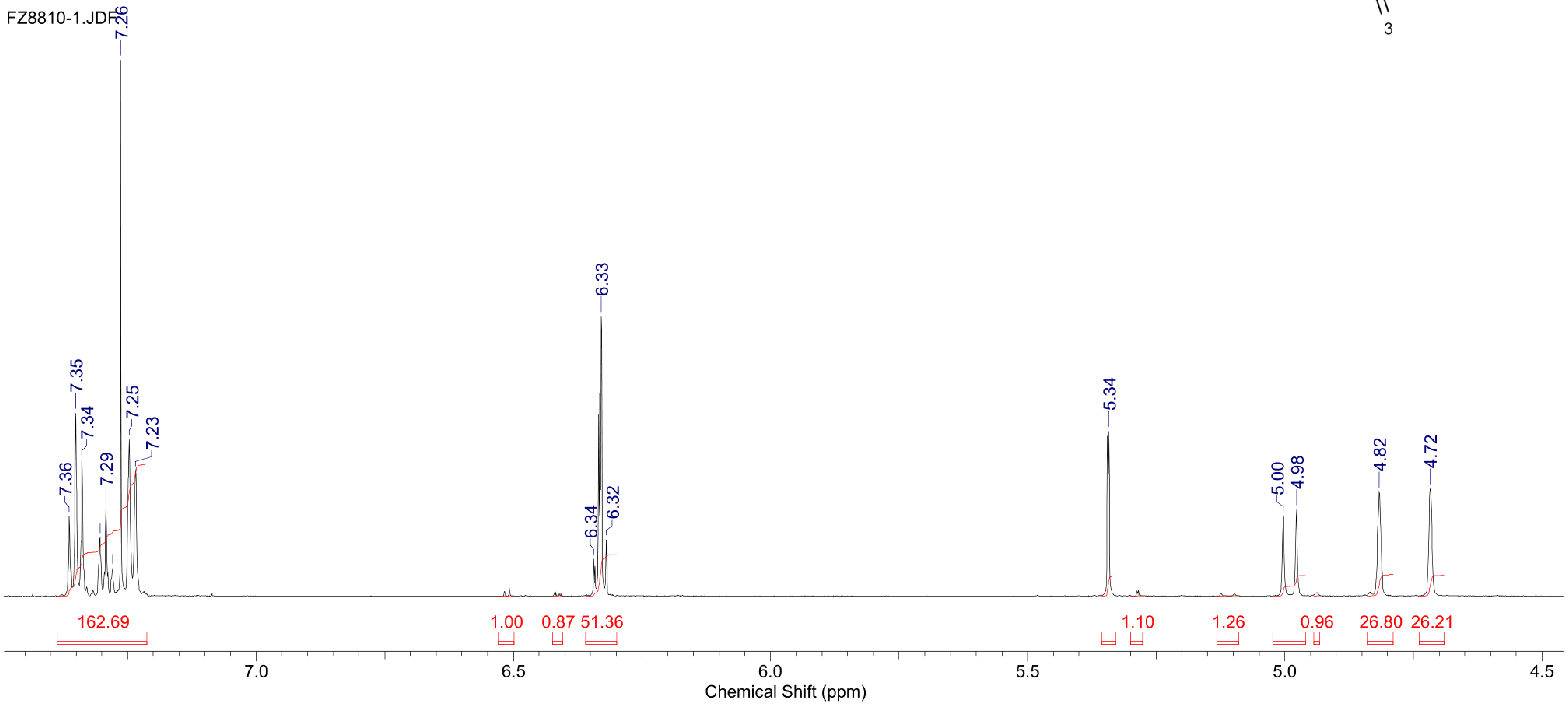

Formula $C_{20}H_{21}NO_4$	FW 339.3850

Acquisition Time (sec)	1.9818	***Comment***	single_pulse	***Date***	17 Jul 2020 12:52:03		
Date Stamp	17 Jul 2020 12:53:51			***File Name***	F:\NMR FIDS ACIDS\BN METHALLYL\FZ8810-1.JDF		
Frequency (MHz)	600.17	***Nucleus***	1H	***Number of Transients***	8	***Origin***	ECA 600
Original Points Count	32768	***Owner***	CKP	***Points Count***	32768	***Pulse Sequence***	single_pulse.ex2
Receiver Gain	46.00	***Solvent***	CHLOROFORM-d			***Spectrum Offset (Hz)***	5409.6182
Sweep Width (Hz)	16534.39						

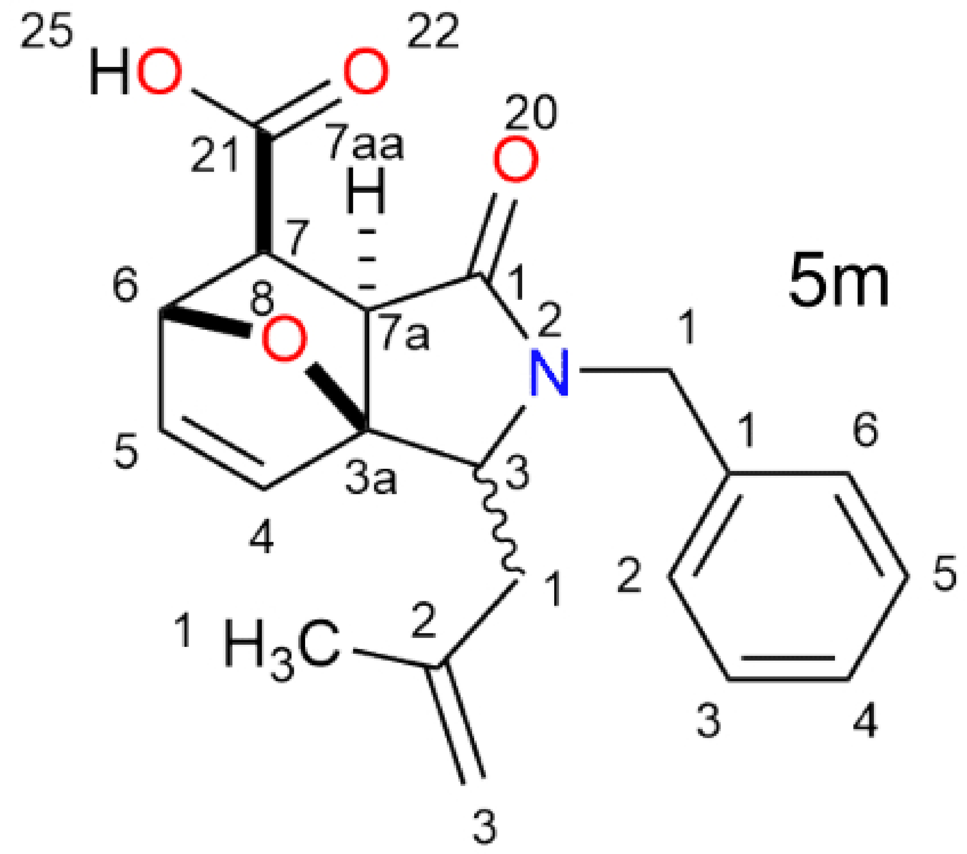

5m

FZ8810-1.JDF

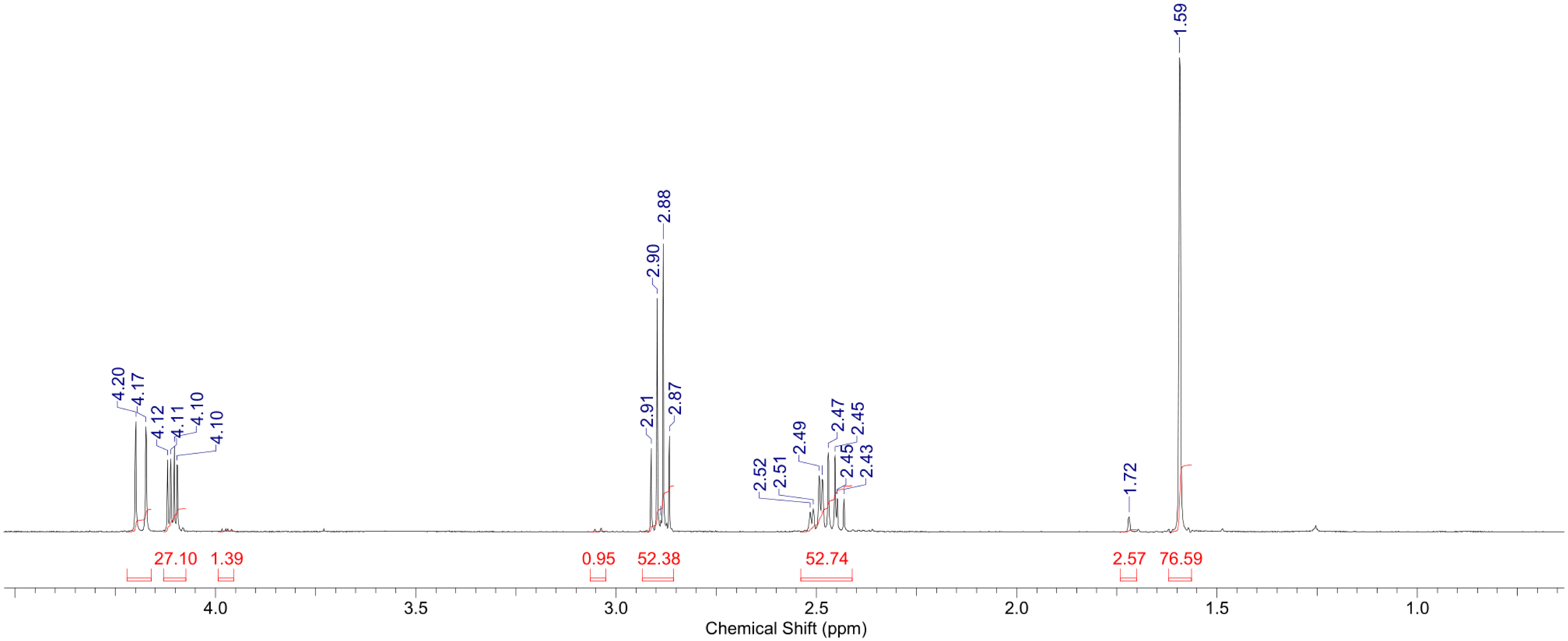

NMR spectrum data for compound 5m (PhMe, r.t, 3 d)

Formula	$C_{20}H_{21}NO_4$	FW	339.3850

**Acquisition Time (sec)**	1.9818	**Comment**	single_pulse	**Date**	17 Jul 2020 13:22:33		
**Date Stamp**	17 Jul 2020 13:24:22			**File Name**	F:\NMR FIDS ACIDS\BN METHALLYL\FZ8821-1.JDF		
**Frequency (MHz)**	600.17	**Nucleus**	1H	**Number of Transients**	8	**Origin**	ECA 600
**Original Points Count**	32768	**Owner**	CKP	**Points Count**	32768	**Pulse Sequence**	single_pulse.ex2
**Receiver Gain**	34.00	**Solvent**	CHLOROFORM-d			**Spectrum Offset (Hz)**	5411.6367
**Sweep Width (Hz)**	16534.39						

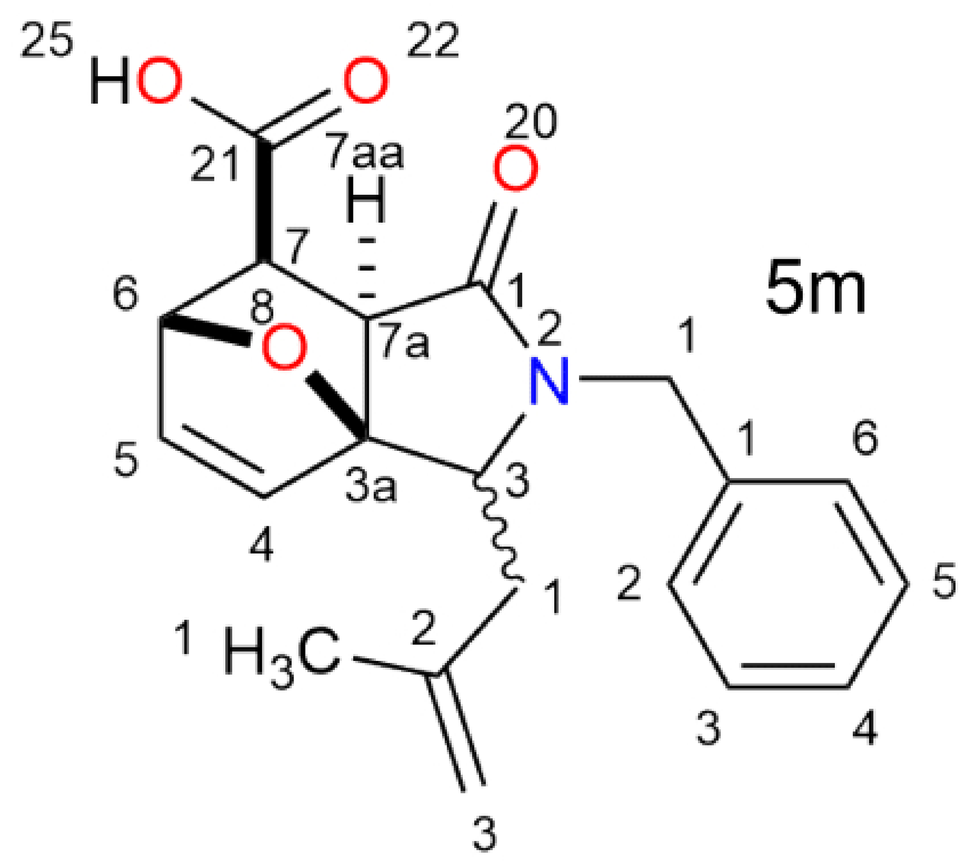

FZ8821-1.JDF

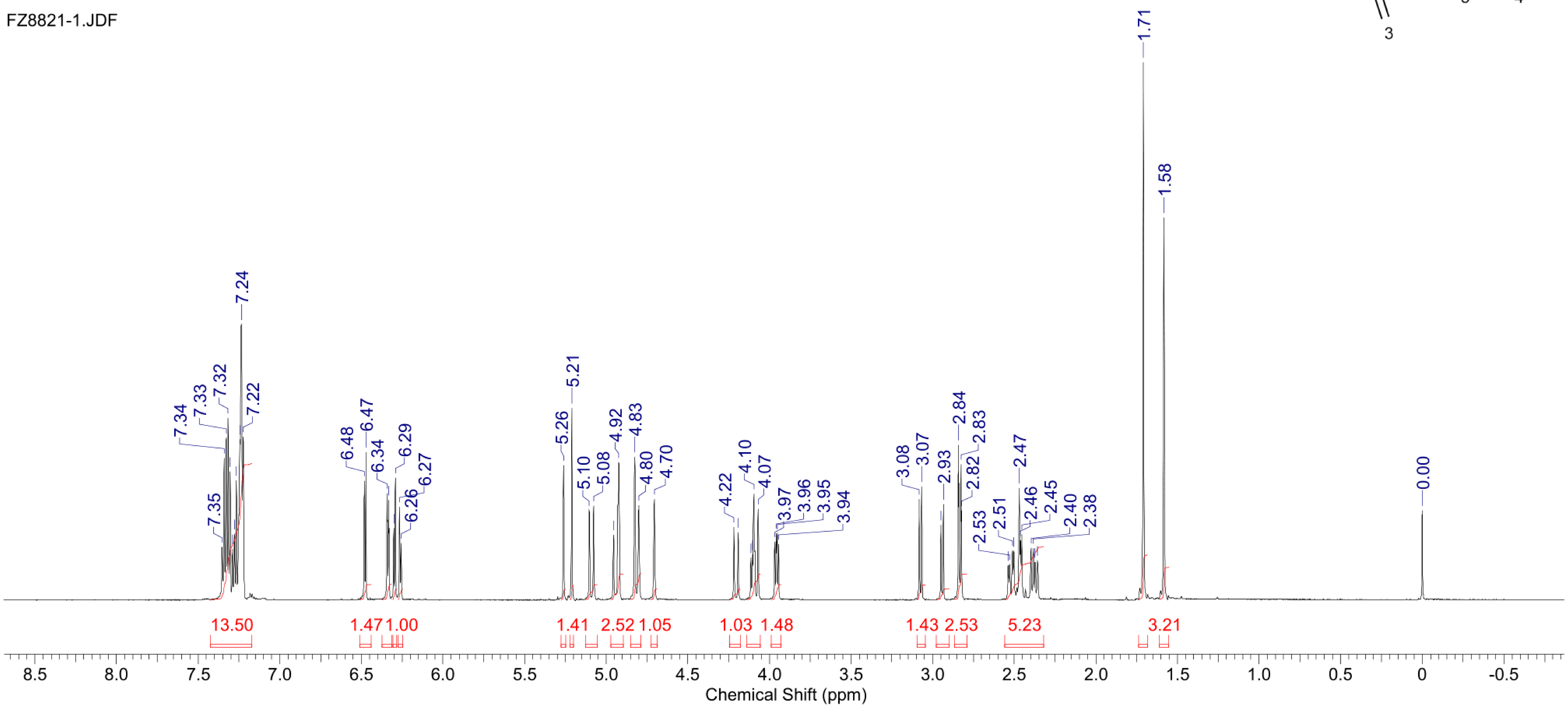

Formula $C_{20}H_{21}NO_4$	FW 339.3850

**Acquisition Time (sec)**	1.9818	**Comment**	single_pulse	**Date**	17 Jul 2020 13:22:33		
**Date Stamp**	17 Jul 2020 13:24:22			**File Name**	F:\NMR FIDS ACIDS\BN METHALLYL\FZ8821-1.JDF		
**Frequency (MHz)**	600.17	**Nucleus**	1H	**Number of Transients**	8	**Origin**	ECA 600
**Original Points Count**	32768	**Owner**	CKP	**Points Count**	32768	**Pulse Sequence**	single_pulse.ex2
**Receiver Gain**	34.00	**Solvent**	CHLOROFORM-d			**Spectrum Offset (Hz)**	5411.6367
**Sweep Width (Hz)**	16534.39						

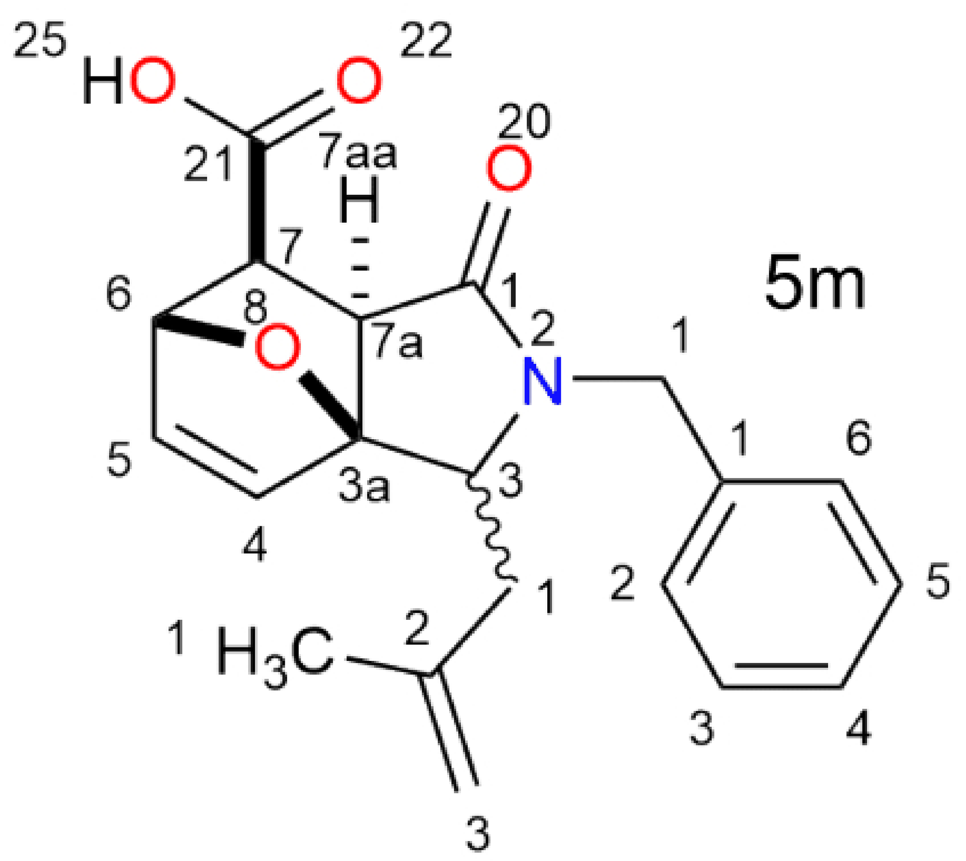

FZ8821-1.JDF

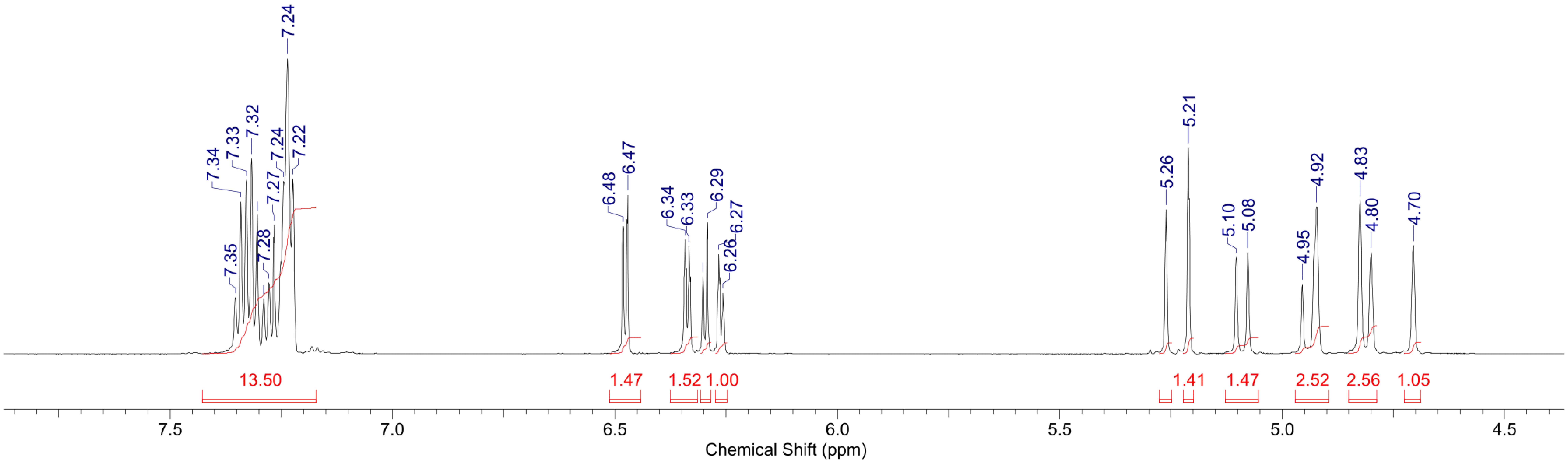

**Formula**	$C_{20}H_{21}NO_4$	**FW**	339.3850

**Acquisition Time (sec)**	1.9818	**Comment**	single_pulse	**Date**	17 Jul 2020 13:22:33		
**Date Stamp**	17 Jul 2020 13:24:22			**File Name**	F:\NMR FIDS ACIDS\BN METHALLYL\FZ8821-1.JDF		
**Frequency (MHz)**	600.17	**Nucleus**	1H	**Number of Transients**	8	**Origin**	ECA 600
**Original Points Count**	32768	**Owner**	CKP	**Points Count**	32768	**Pulse Sequence**	single_pulse.ex2
**Receiver Gain**	34.00	**Solvent**	CHLOROFORM-d			**Spectrum Offset (Hz)**	5411.6367
**Sweep Width (Hz)**	16534.39						

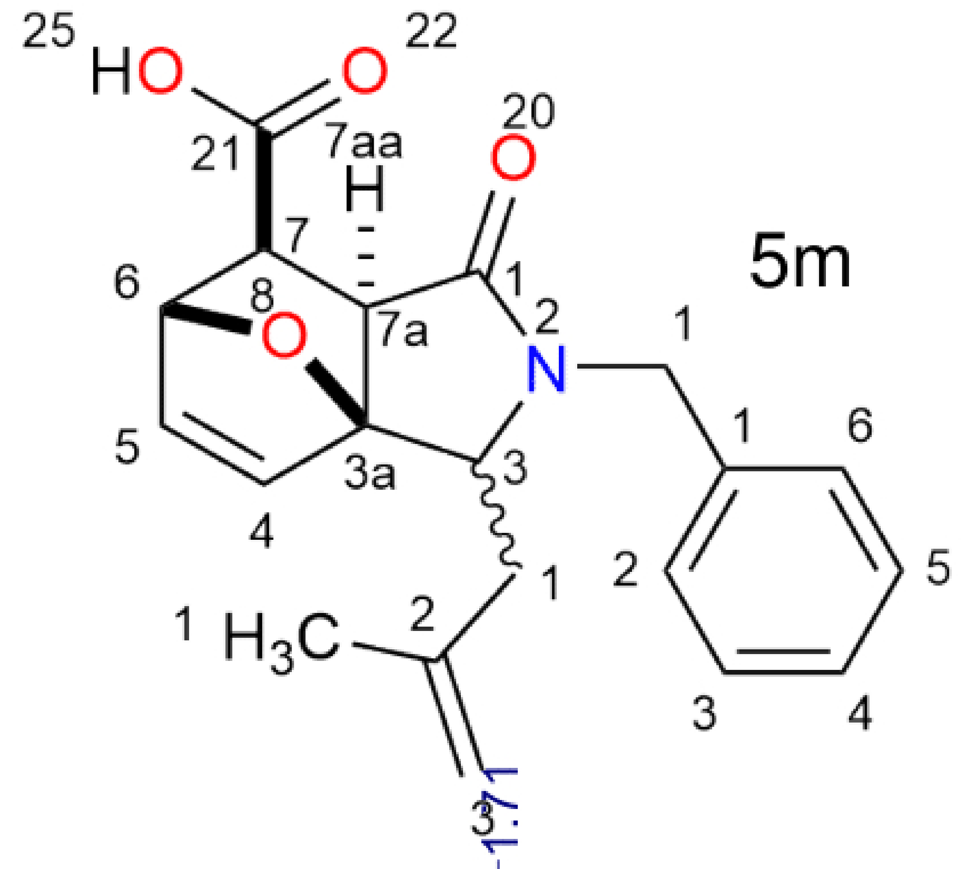

5m

FZ8821-1.JDF

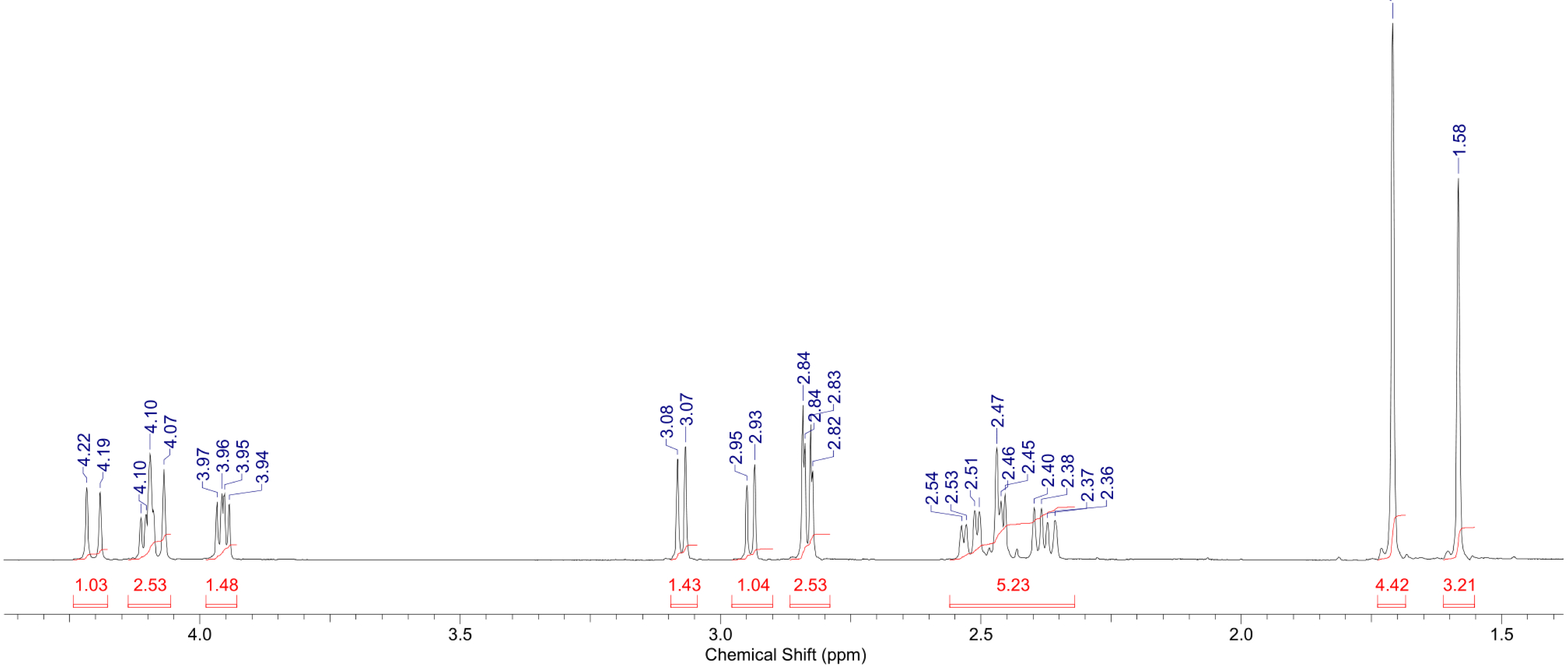

NMR spectrum data for compound 5m (PhMe, Δ, 3 h)

**Formula**	$C_{20}H_{21}NO_4$	**FW**	339.3850

**Acquisition Time (sec)**	1.9818	**Comment**	single_pulse	**Date**	17 Jul 2020 13:28:30		
**Date Stamp**	17 Jul 2020 13:30:19			**File Name**	F:\NMR FIDS ACIDS\BN METHALLYL\FZ8822-1.JDF		
**Frequency (MHz)**	600.17	**Nucleus**	1H	**Number of Transients**	8	**Origin**	ECA 600
**Original Points Count**	32768	**Owner**	CKP	**Points Count**	32768	**Pulse Sequence**	single_pulse.ex2
**Receiver Gain**	38.00	**Solvent**	CHLOROFORM-d			**Spectrum Offset (Hz)**	5411.1318
**Sweep Width (Hz)**	16534.39						

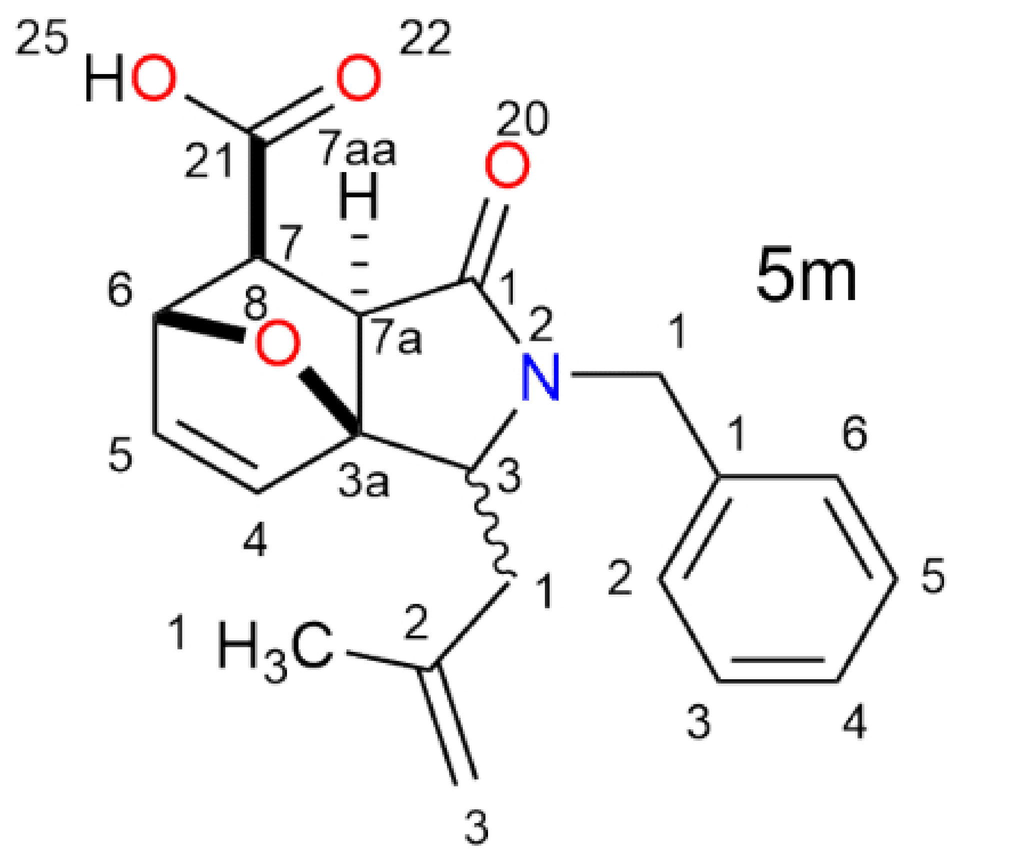

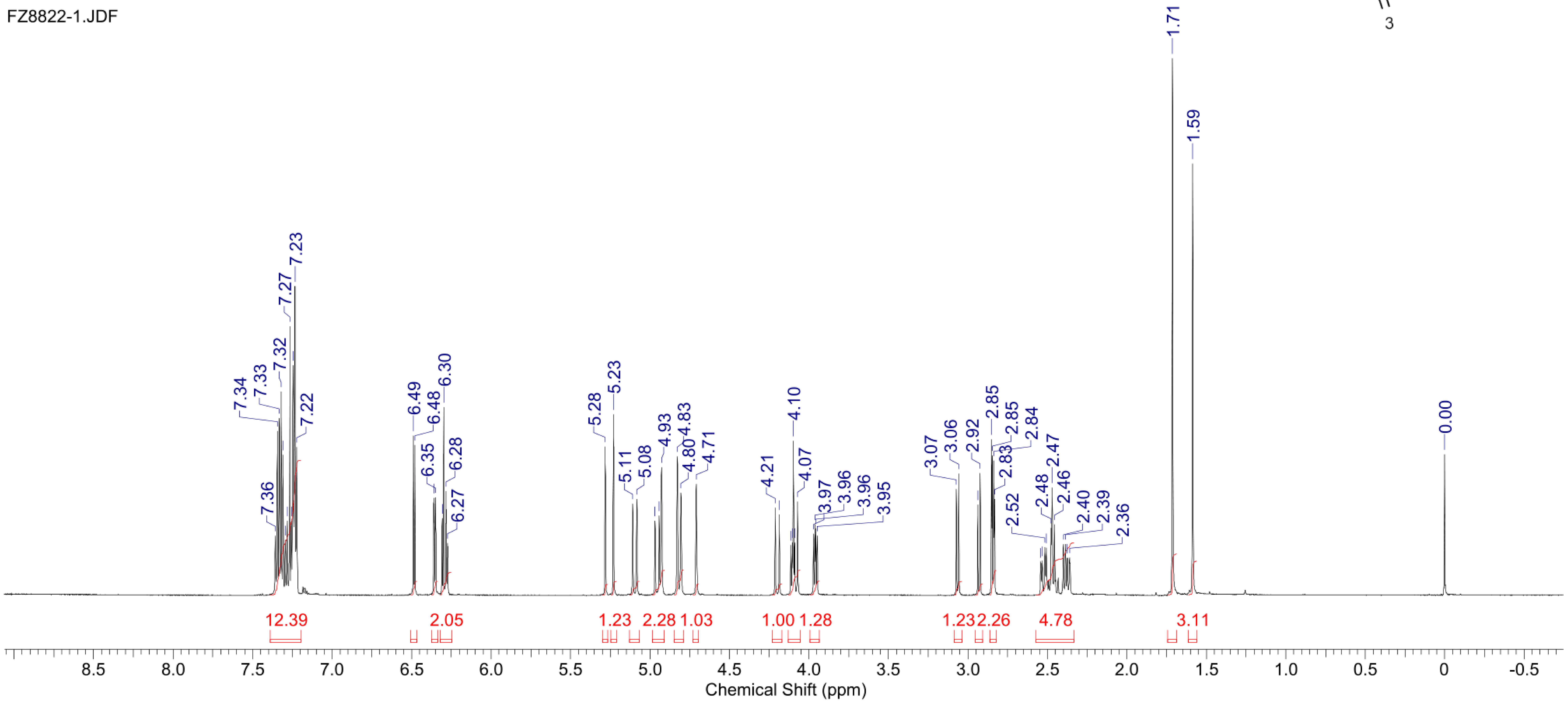

Formula $C_{20}H_{21}NO_4$	FW 339.3850

Acquisition Time (sec)	1.9818	***Comment***	single_pulse	***Date***	17 Jul 2020 13:28:30		
Date Stamp	17 Jul 2020 13:30:19			***File Name***	F:\NMR FIDS ACIDS\BN METHALLYL\FZ8822-1.JDF		
Frequency (MHz)	600.17	***Nucleus***	1H	***Number of Transients***	8	***Origin***	ECA 600
Original Points Count	32768	***Owner***	CKP	***Points Count***	32768	***Pulse Sequence***	single_pulse.ex2
Receiver Gain	38.00	***Solvent***	CHLOROFORM-d			***Spectrum Offset (Hz)***	5411.1318
Sweep Width (Hz)	16534.39						

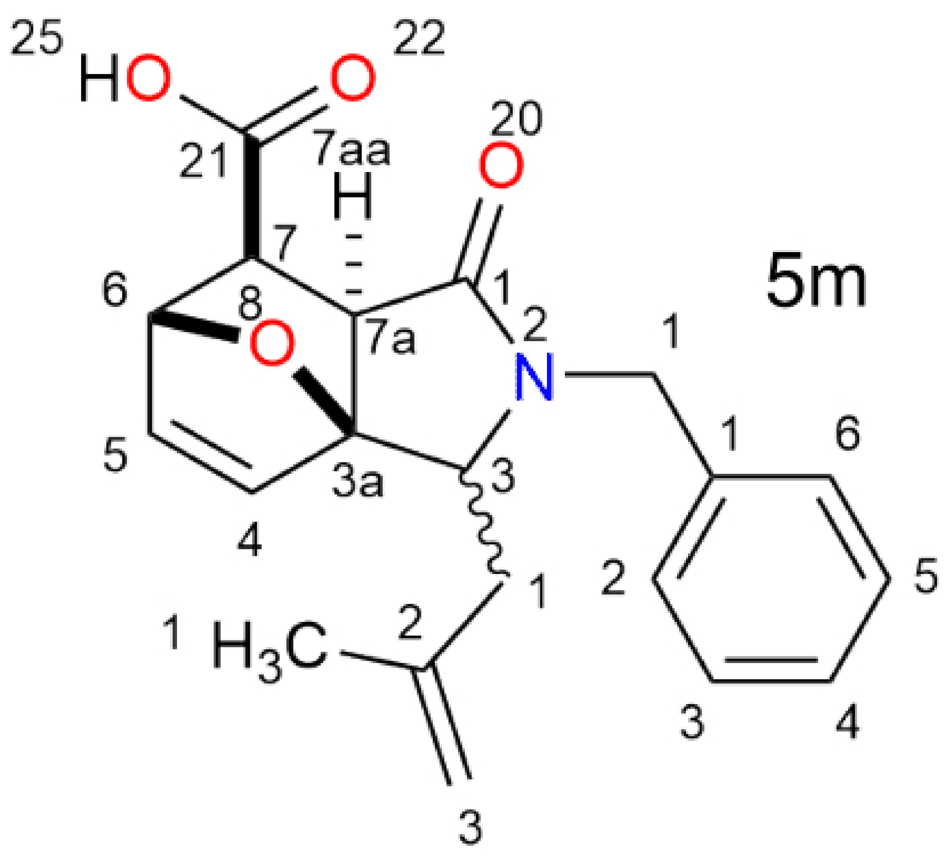

5m

FZ8822-1.JDF

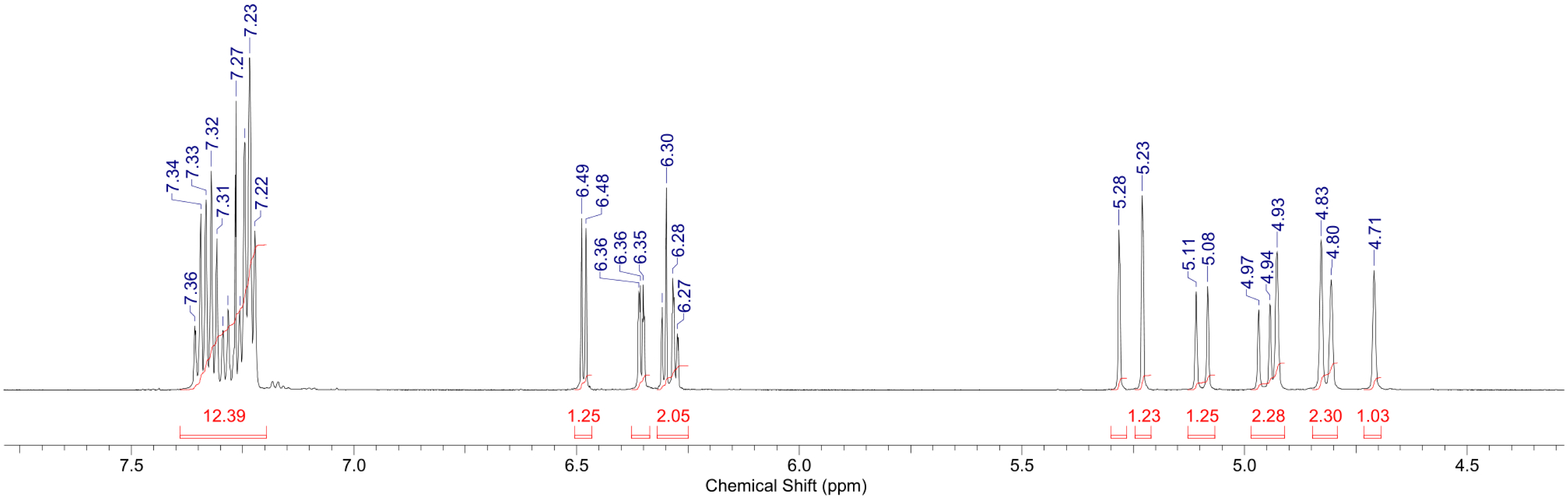

Formula	$C_{20}H_{21}NO_4$	FW	339.3850

**Acquisition Time (sec)**	1.9818	**Comment**	single_pulse	**Date**	17 Jul 2020 13:28:30		
**Date Stamp**	17 Jul 2020 13:30:19			**File Name**	F:\NMR FIDS ACIDS\BN METHALLYL\FZ8822-1.JDF		
**Frequency (MHz)**	600.17	**Nucleus**	1H	**Number of Transients**	8	**Origin**	ECA 600
**Original Points Count**	32768	**Owner**	CKP	**Points Count**	32768	**Pulse Sequence**	single_pulse.ex2
**Receiver Gain**	38.00	**Solvent**	CHLOROFORM-d			**Spectrum Offset (Hz)**	5411.1318
**Sweep Width (Hz)**	16534.39						

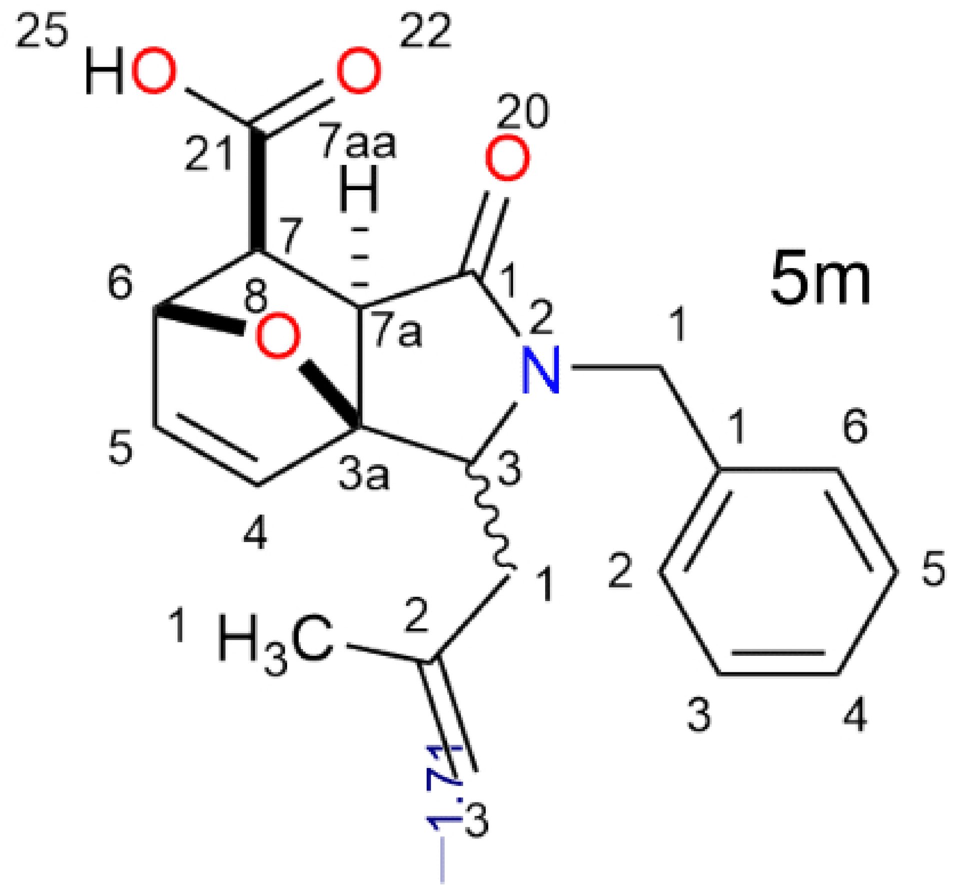

5m

FZ8822-1.JDF

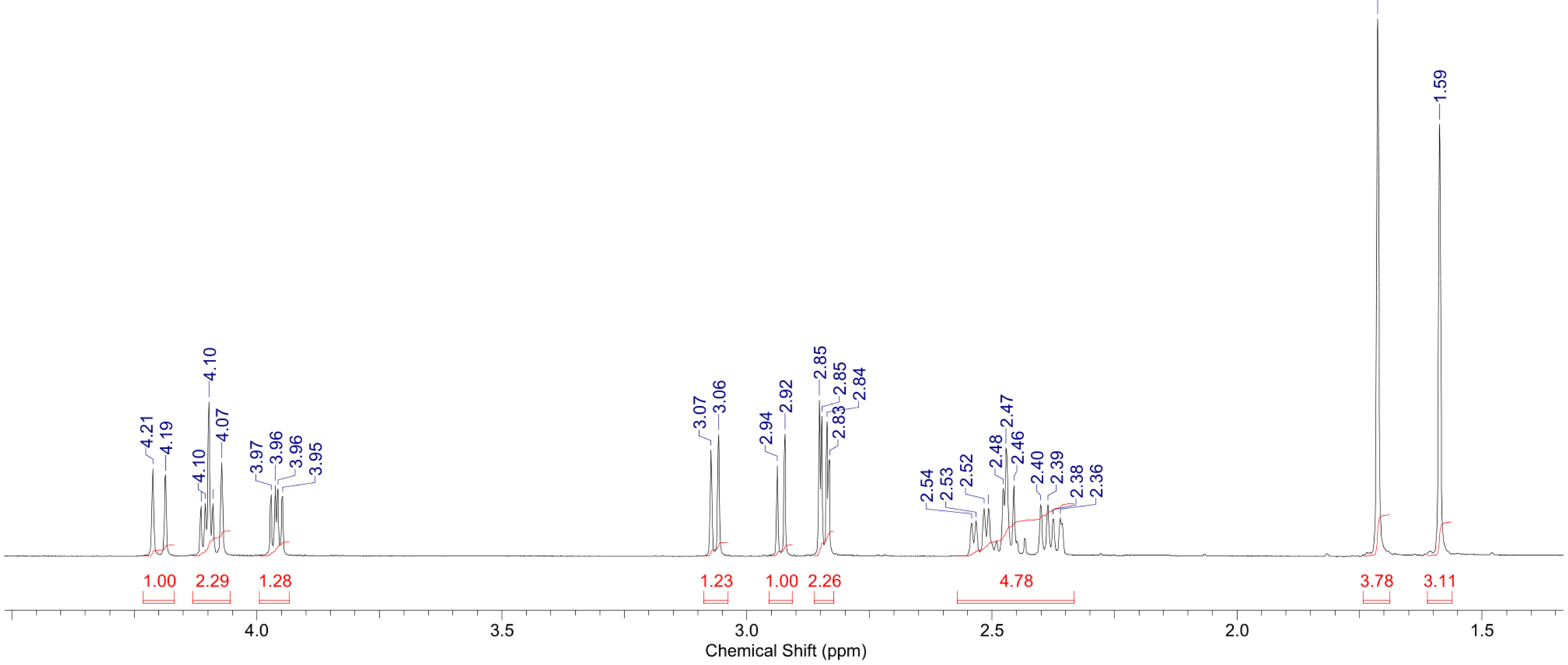

NMR spectrum data for compound 5m

Acquisition Time (sec)	0.6921	***Comment***	single pulse decoupled gated NOE			***Date***	29 May 1990 08:12:28
Date Stamp	08 Aug 2019 14:15:55			***File Name***	C:\Users\Fedor\Desktop\07.08.19\FZ7646-1.jdf		
Frequency (MHz)	150.91	***Nucleus***	13C	***Number of Transients***	800	***Origin***	ECA 600
Original Points Count	32768	***Owner***	delta	***Points Count***	32768	***Pulse Sequence***	single_pulse_dec
Receiver Gain	58.00	***Solvent***	CHLOROFORM-d			***Spectrum Offset (Hz)***	15091.3428
Sweep Width (Hz)	47348.49	***Temperature (degree C)***	23.600				

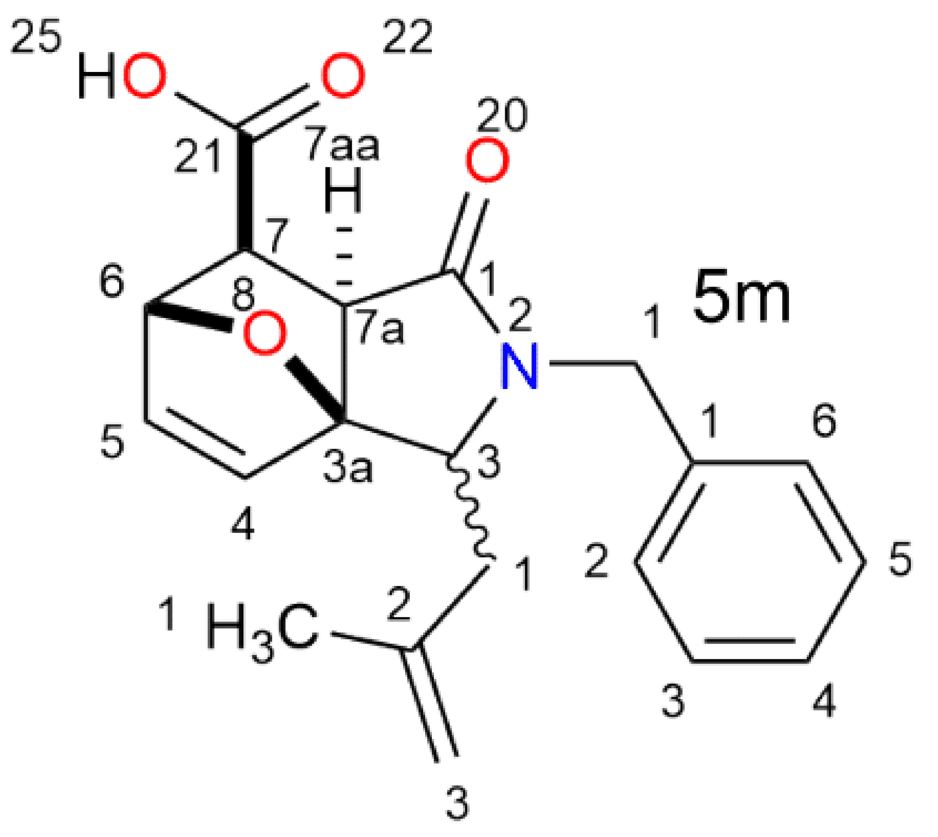

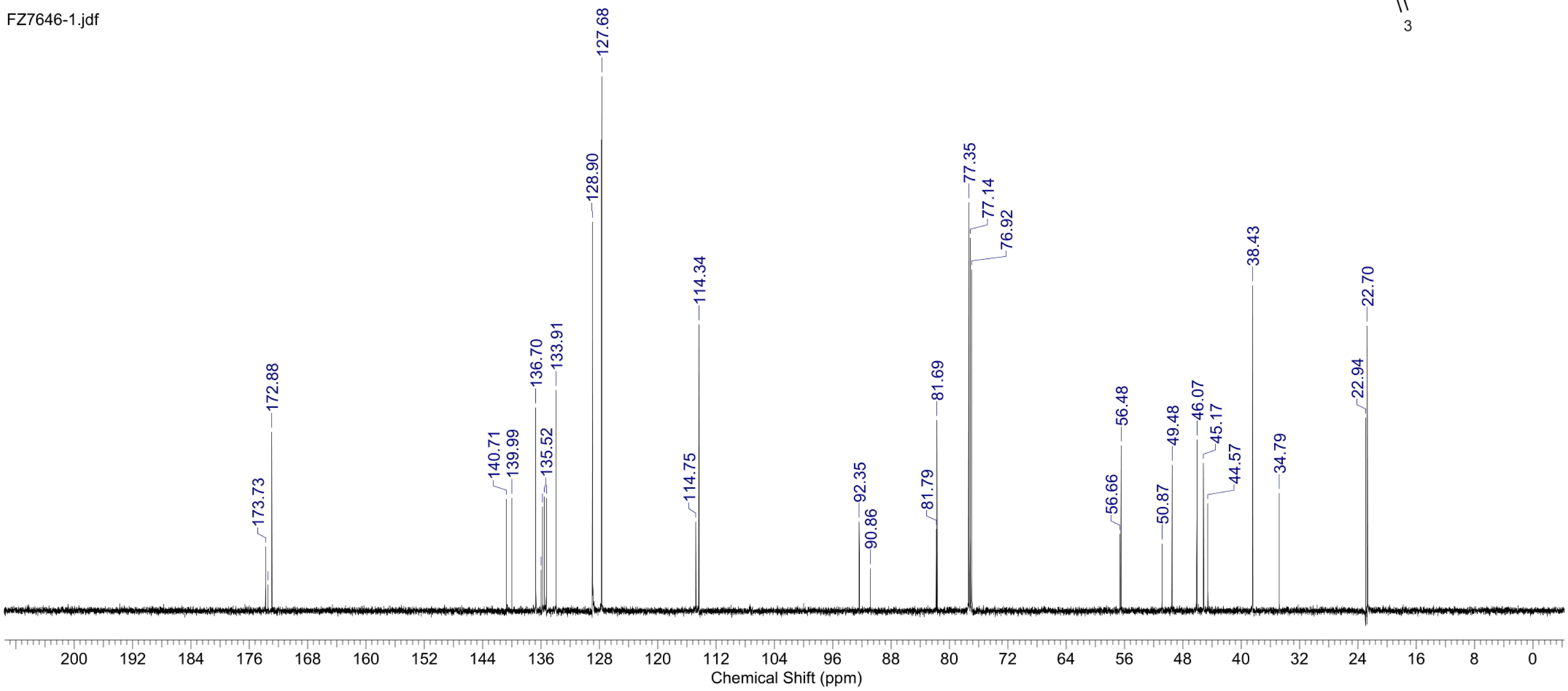

Acquisition Time (sec)	0.6921	Comment	single pulse decoupled gated NOE			Date	29 May 1990 08:12:28
Date Stamp	08 Aug 2019 14:15:55			File Name	C:\Users\Fedor\Desktop\07.08.19\FZ7646-1.jdf		
Frequency (MHz)	150.91	Nucleus	13C	Number of Transients	800	Origin	ECA 600
Original Points Count	32768	Owner	delta	Points Count	32768	Pulse Sequence	single_pulse_dec
Receiver Gain	58.00	Solvent	CHLOROFORM-d			Spectrum Offset (Hz)	15091.3428
Sweep Width (Hz)	47348.49	Temperature (degree C)	23.600				

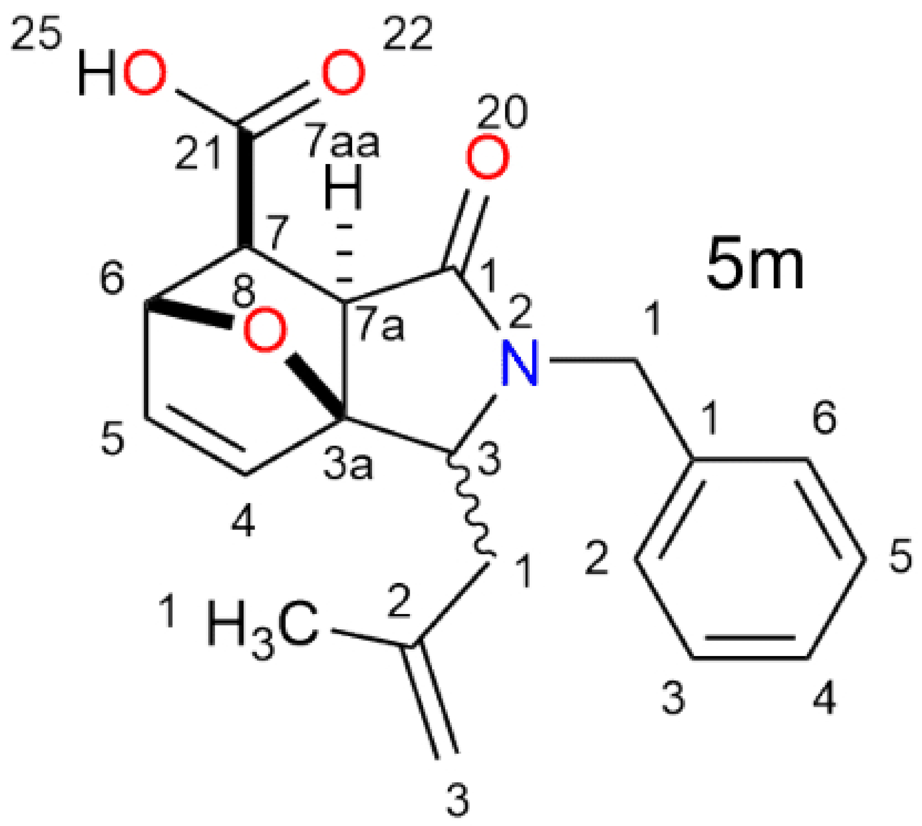

5m

FZ7646-1.jdf

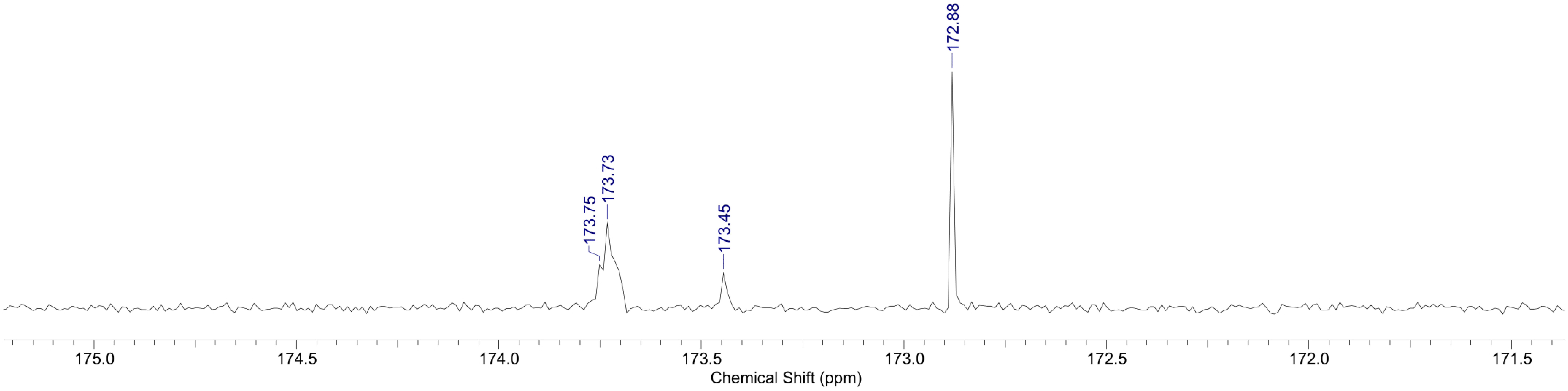

Acquisition Time (sec)	0.6921	***Comment***	single pulse decoupled gated NOE			***Date***	29 May 1990 08:12:28
Date Stamp	08 Aug 2019 14:15:55			***File Name***	C:\Users\Fedor\Desktop\07.08.19\FZ7646-1.jdf		
Frequency (MHz)	150.91	***Nucleus***	13C	***Number of Transients***	800	***Origin***	ECA 600
Original Points Count	32768	***Owner***	delta	***Points Count***	32768	***Pulse Sequence***	single_pulse_dec
Receiver Gain	58.00	***Solvent***	CHLOROFORM-d			***Spectrum Offset (Hz)***	15091.3428
Sweep Width (Hz)	47348.49	***Temperature (degree C)***	23.600				

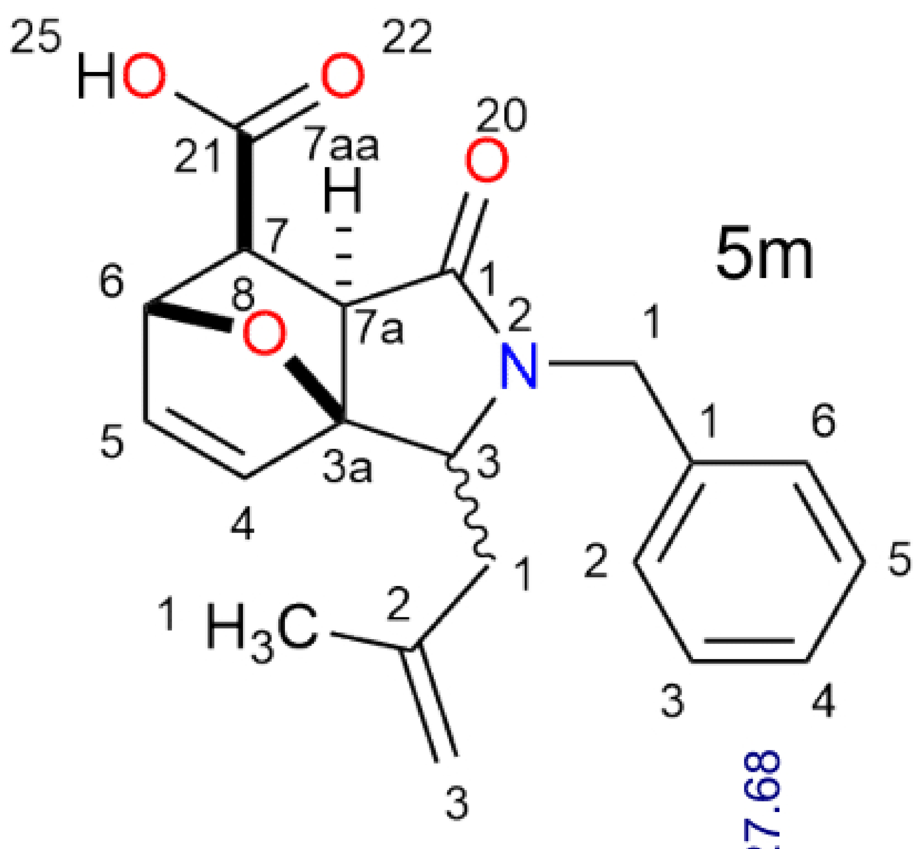

5m

FZ7646-1.jdf

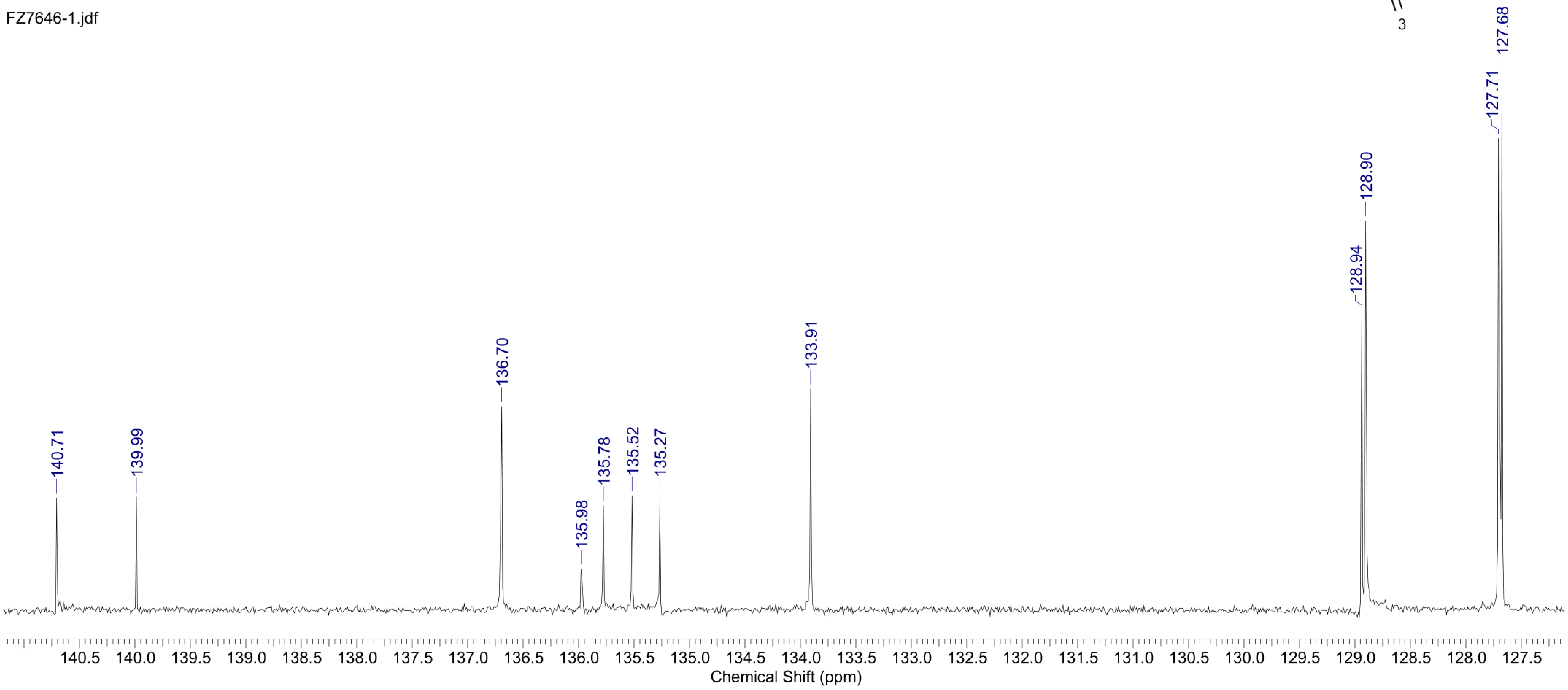

**Acquisition Time (sec)**	0.6921	**Comment**	single pulse decoupled gated NOE	**Date**	29 May 1990 08:12:28
**Date Stamp**	08 Aug 2019 14:15:55	**File Name**	C:\Users\Fedor\Desktop\07.08.19\FZ7646-1.jdf		
**Frequency (MHz)**	150.91	**Nucleus**	13C	**Number of Transients**	800
**Origin**	ECA 600				
**Original Points Count**	32768	**Owner**	delta	**Points Count**	32768
**Pulse Sequence**	single_pulse_dec				
**Receiver Gain**	58.00	**Solvent**	CHLOROFORM-d	**Spectrum Offset (Hz)**	15091.3428
**Sweep Width (Hz)**	47348.49	**Temperature (degree C)**	23.600		

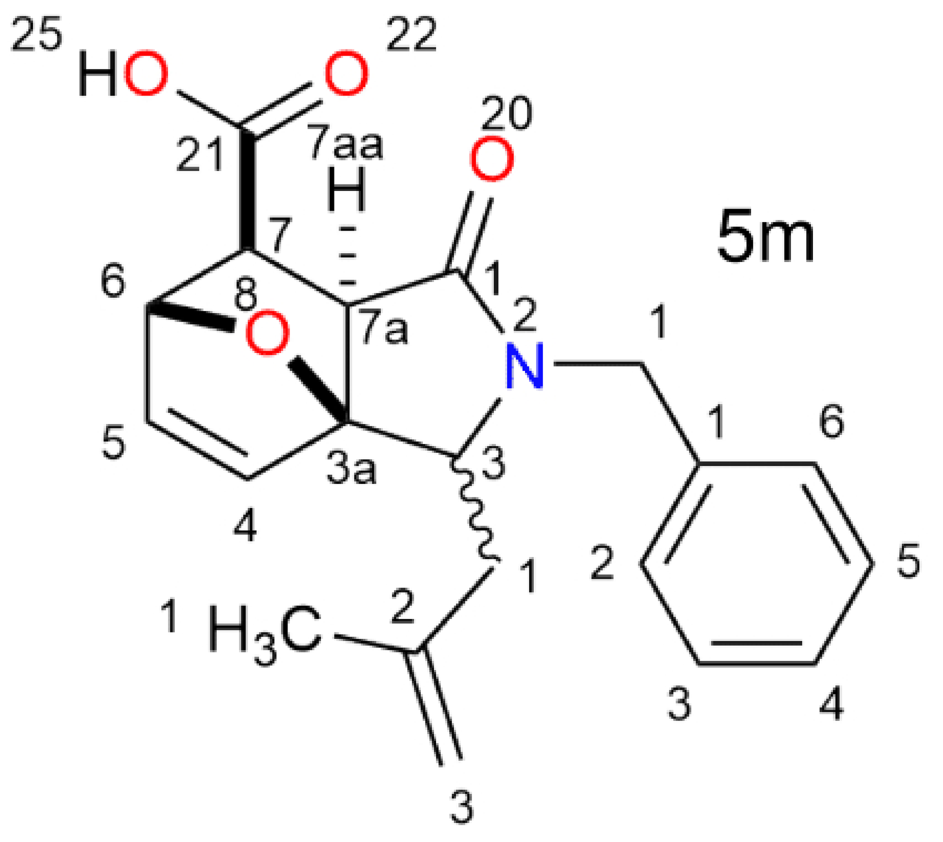

5m

FZ7646-1.jdf

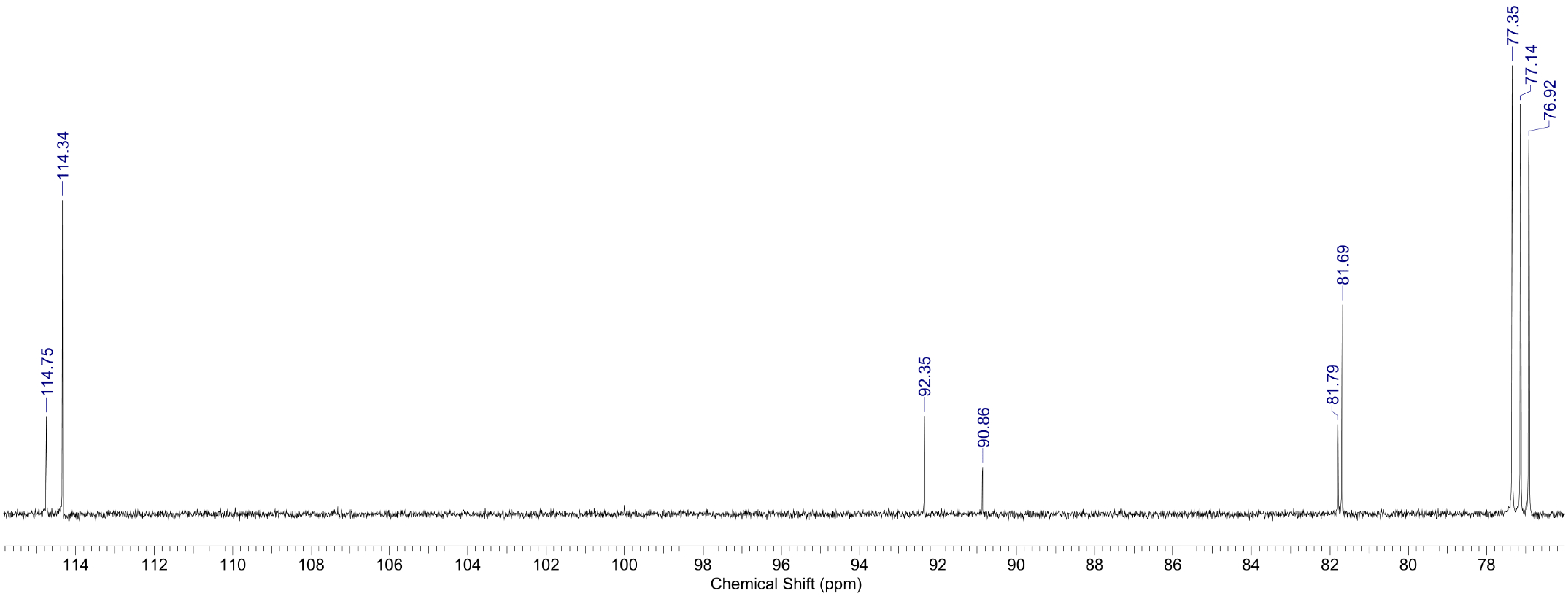

Acquisition Time (sec)	0.6921	***Comment***	single pulse decoupled gated NOE			***Date***	29 May 1990 08:12:28
Date Stamp	08 Aug 2019 14:15:55			***File Name***	C:\Users\Fedor\Desktop\07.08.19\FZ7646-1.jdf		
Frequency (MHz)	150.91	***Nucleus***	13C	***Number of Transients***	800	***Origin***	ECA 600
Original Points Count	32768	***Owner***	delta	***Points Count***	32768	***Pulse Sequence***	single_pulse_dec
Receiver Gain	58.00	***Solvent***	CHLOROFORM-d			***Spectrum Offset (Hz)***	15091.3428
Sweep Width (Hz)	47348.49	***Temperature (degree C)***	23.600				

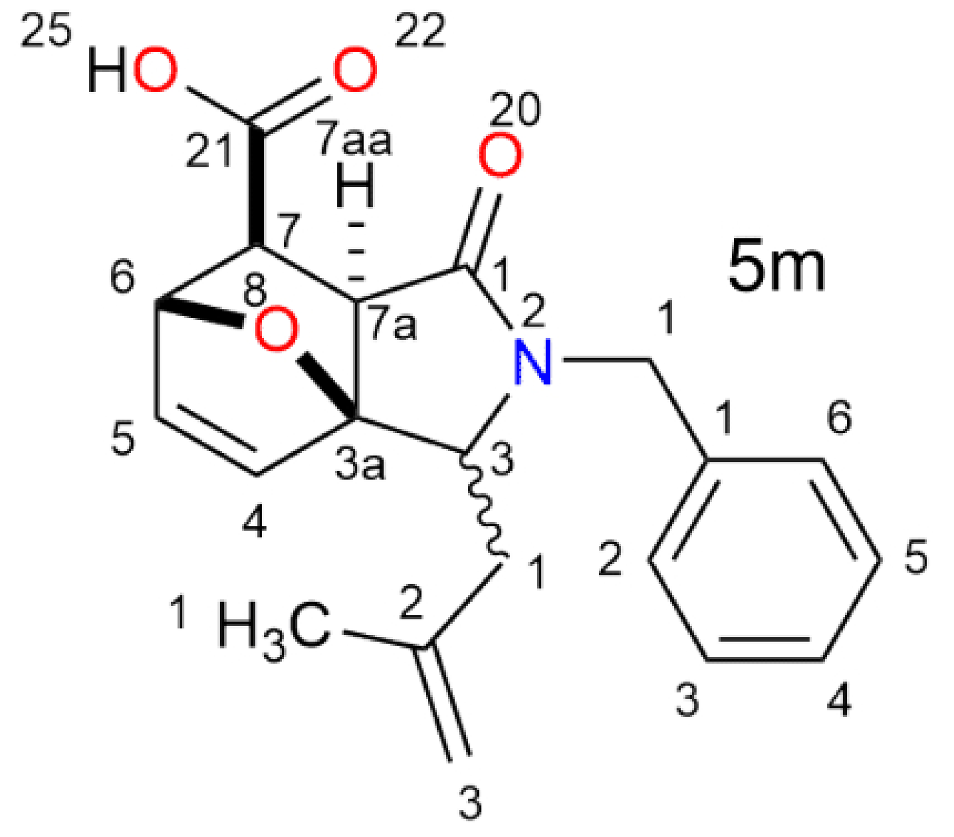

5m

FZ7646-1.jdf

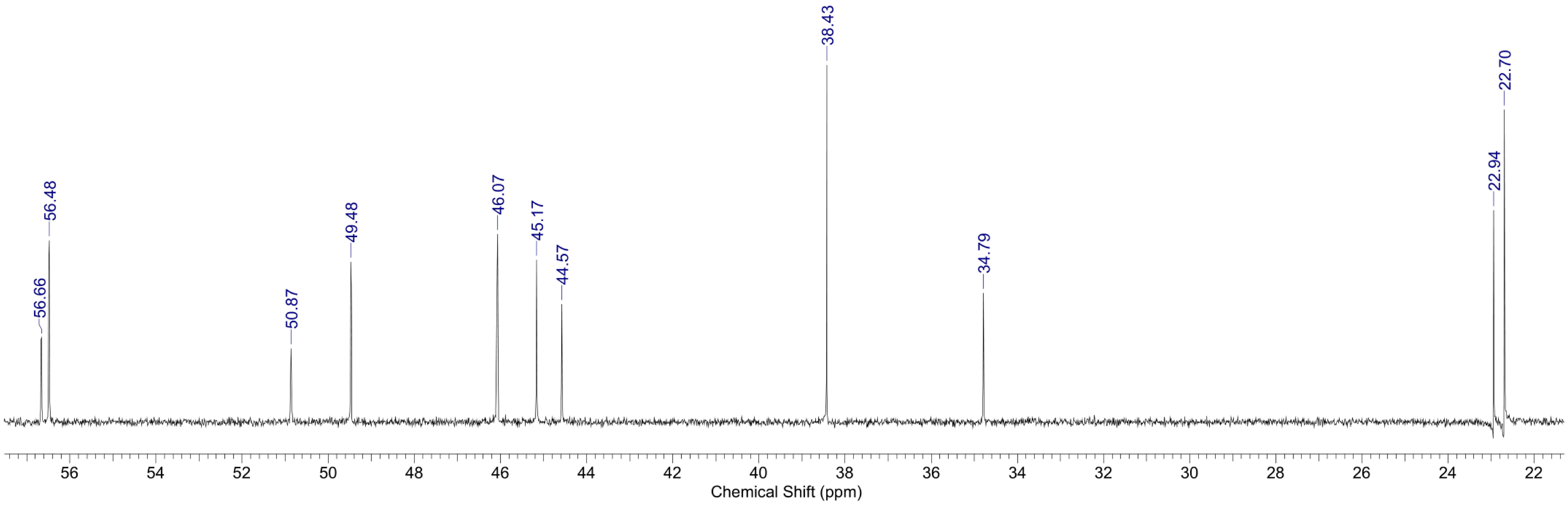

NMR spectrum data for compound 6a

Acquisition Time (sec)	1.9818	Comment	single_pulse	Date	13 May 1990 04:54:23		
Date Stamp	23 Jul 2019 10:57:57			File Name	C:\Users\Fedor\Desktop\22.07.19\FZ7587-1.jdf	Frequency (MHz)	600.17
Nucleus	1H	Number of Transients	8	Origin	ECA 600	Original Points Count	32768	Owner	delta
Points Count	32768	Pulse Sequence	single_pulse.ex2			Receiver Gain	38.00	Solvent	CHLOROFORM-d
Spectrum Offset (Hz)	5411.6367	Sweep Width (Hz)	16534.39	Temperature (degree C)	22.800		

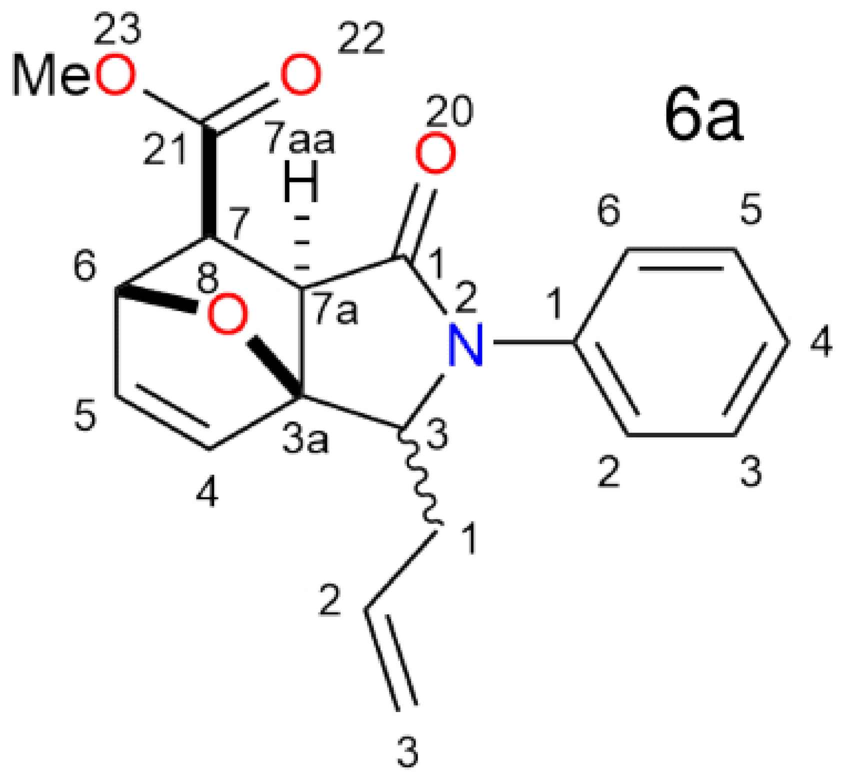

6a

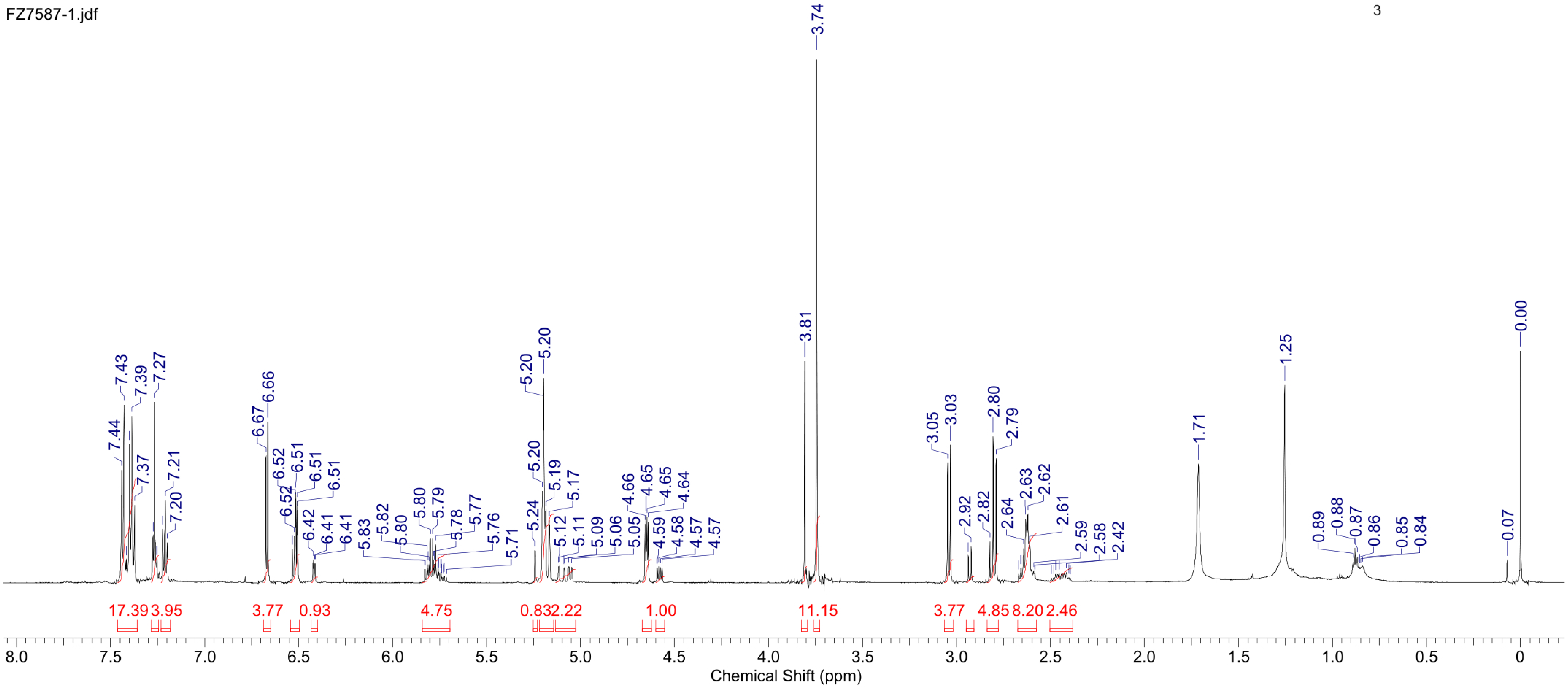

Acquisition Time (sec)	1.9818	***Comment***	single_pulse	***Date***	13 May 1990 04:54:23		
Date Stamp	23 Jul 2019 10:57:57			***File Name***	C:\Users\Fedor\Desktop\22.07.19\FZ7587-1.jdf	***Frequency (MHz)***	600.17
Nucleus	1H	***Number of Transients***	8	***Origin***	ECA 600	***Original Points Count*** 32768	***Owner*** delta
Points Count	32768	***Pulse Sequence***	single_pulse.ex2			***Receiver Gain*** 38.00	***Solvent*** CHLOROFORM-d
Spectrum Offset (Hz)	5411.6367	***Sweep Width (Hz)***	16534.39	***Temperature (degree C)***	22.800		

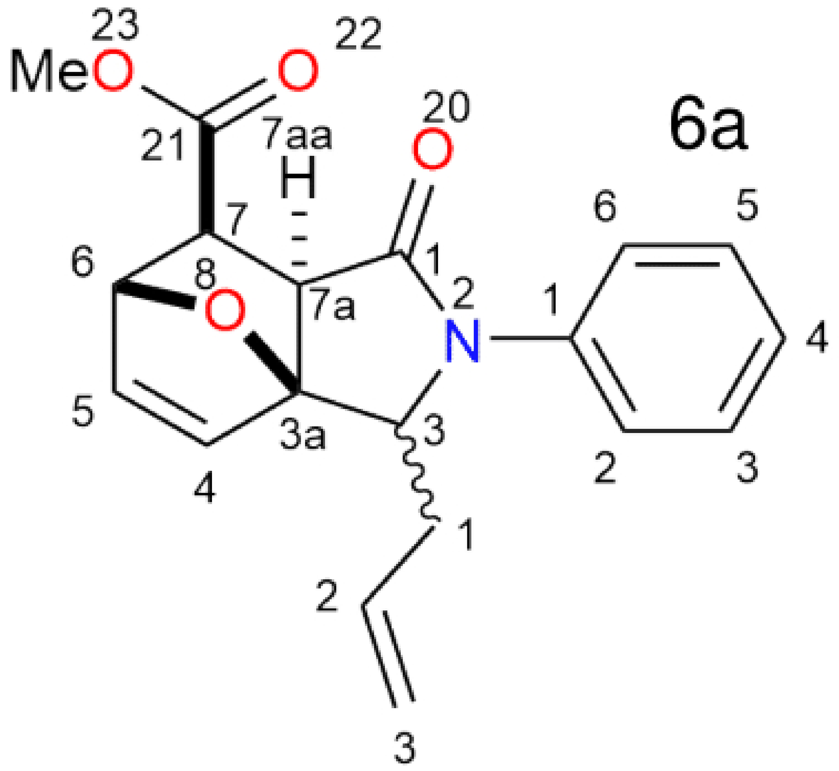

6a

FZ7587-1.jdf

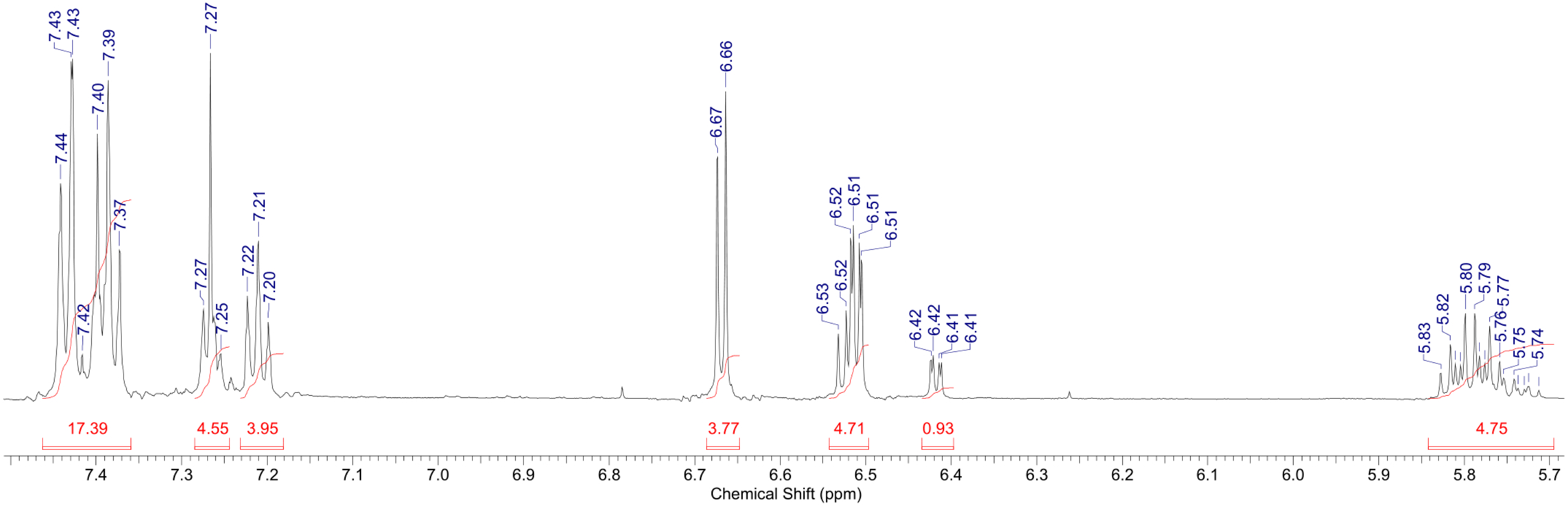

Acquisition Time (sec)	1.9818	***Comment***	single_pulse	***Date***	13 May 1990 04:54:23				
Date Stamp	23 Jul 2019 10:57:57			***File Name***	C:\Users\Fedor\Desktop\22.07.19\FZ7587-1.jdf			***Frequency (MHz)***	600.17
Nucleus	1H	***Number of Transients***	8	***Origin***	ECA 600	***Original Points Count***	32768	***Owner***	delta
Points Count	32768	***Pulse Sequence***	single_pulse.ex2			***Receiver Gain***	38.00	***Solvent***	CHLOROFORM-d
Spectrum Offset (Hz)	5411.6367	***Sweep Width (Hz)***	16534.39	***Temperature (degree C)***	22.800				

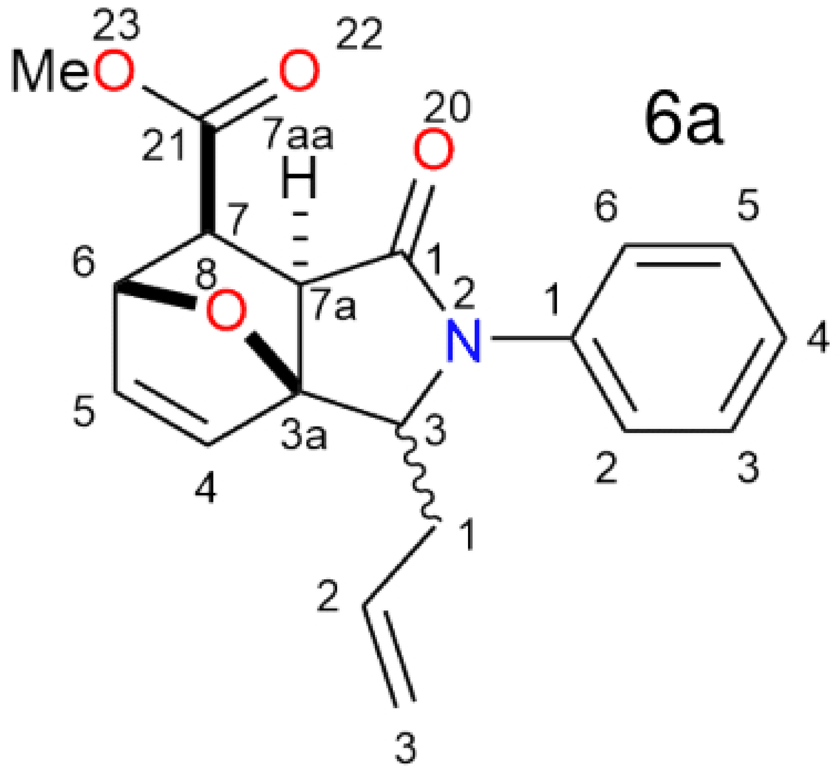

6a

FZ7587-1.jdf

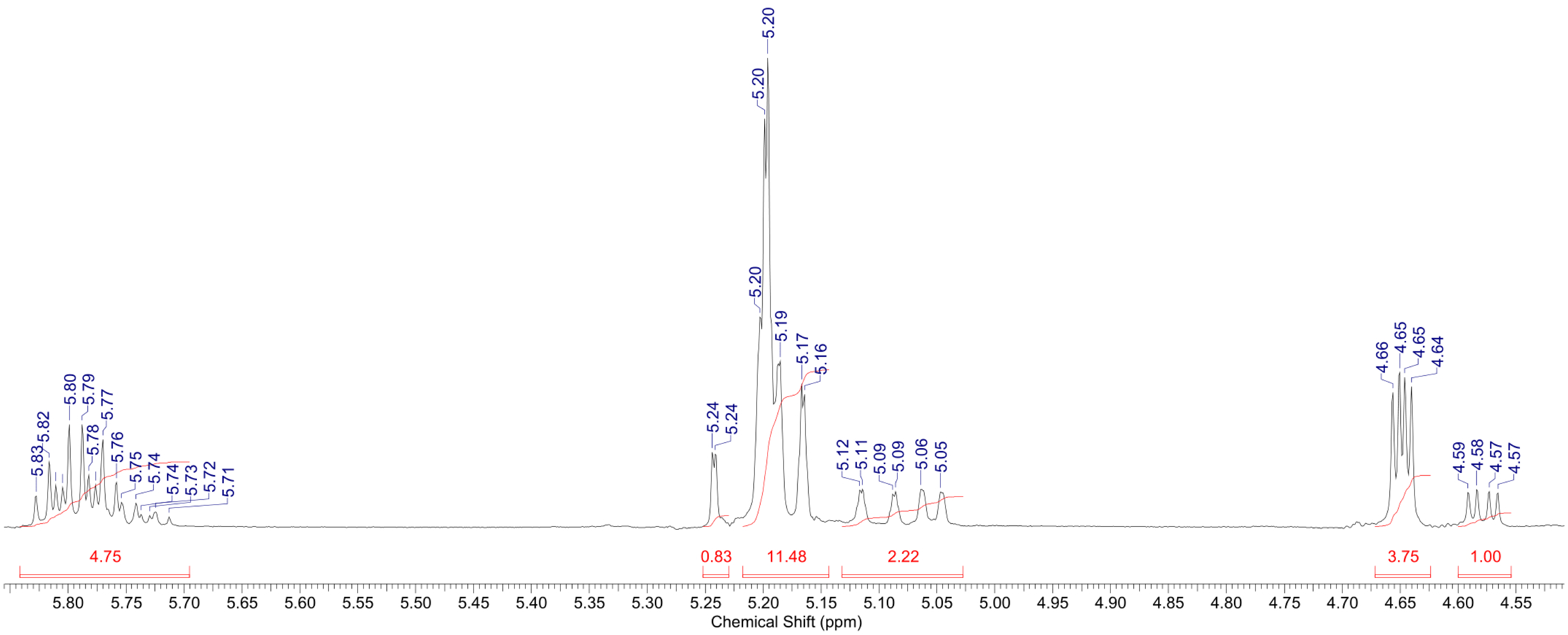

Acquisition Time (sec)	1.9818	***Comment***	single_pulse	***Date***	13 May 1990 04:54:23		
Date Stamp	23 Jul 2019 10:57:57			***File Name***	C:\Users\Fedor\Desktop\22.07.19\FZ7587-1.jdf	***Frequency (MHz)***	600.17
Nucleus	1H	***Number of Transients***	8	***Origin***	ECA 600	***Original Points Count***	32768
Owner	delta						
Points Count	32768	***Pulse Sequence***	single_pulse.ex2	***Receiver Gain***	38.00	***Solvent***	CHLOROFORM-d
Spectrum Offset (Hz)	5411.6367	***Sweep Width (Hz)***	16534.39	***Temperature (degree C)***	22.800		

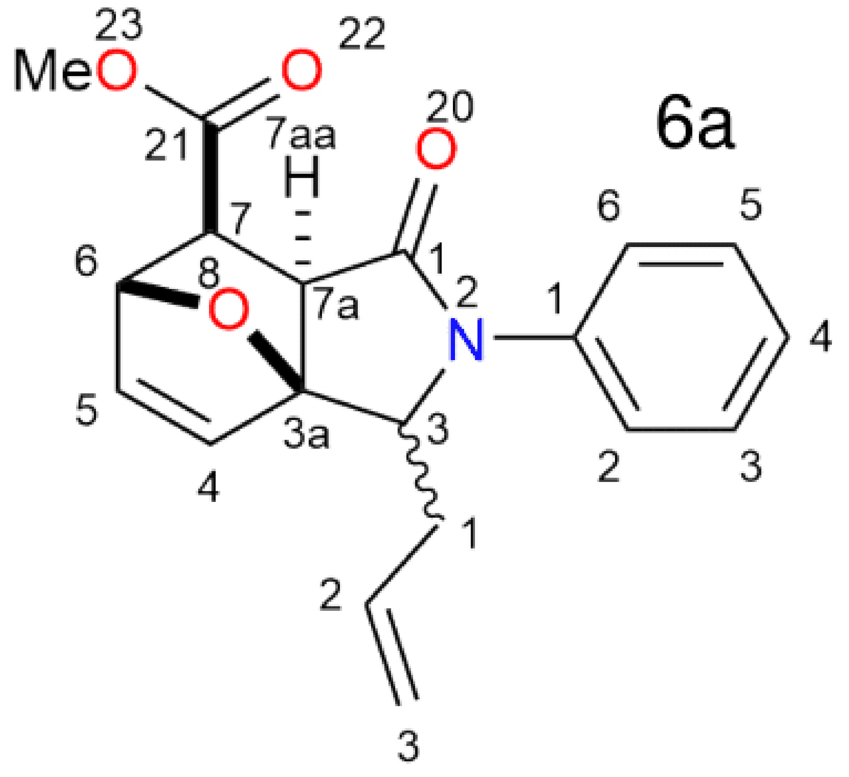

6a

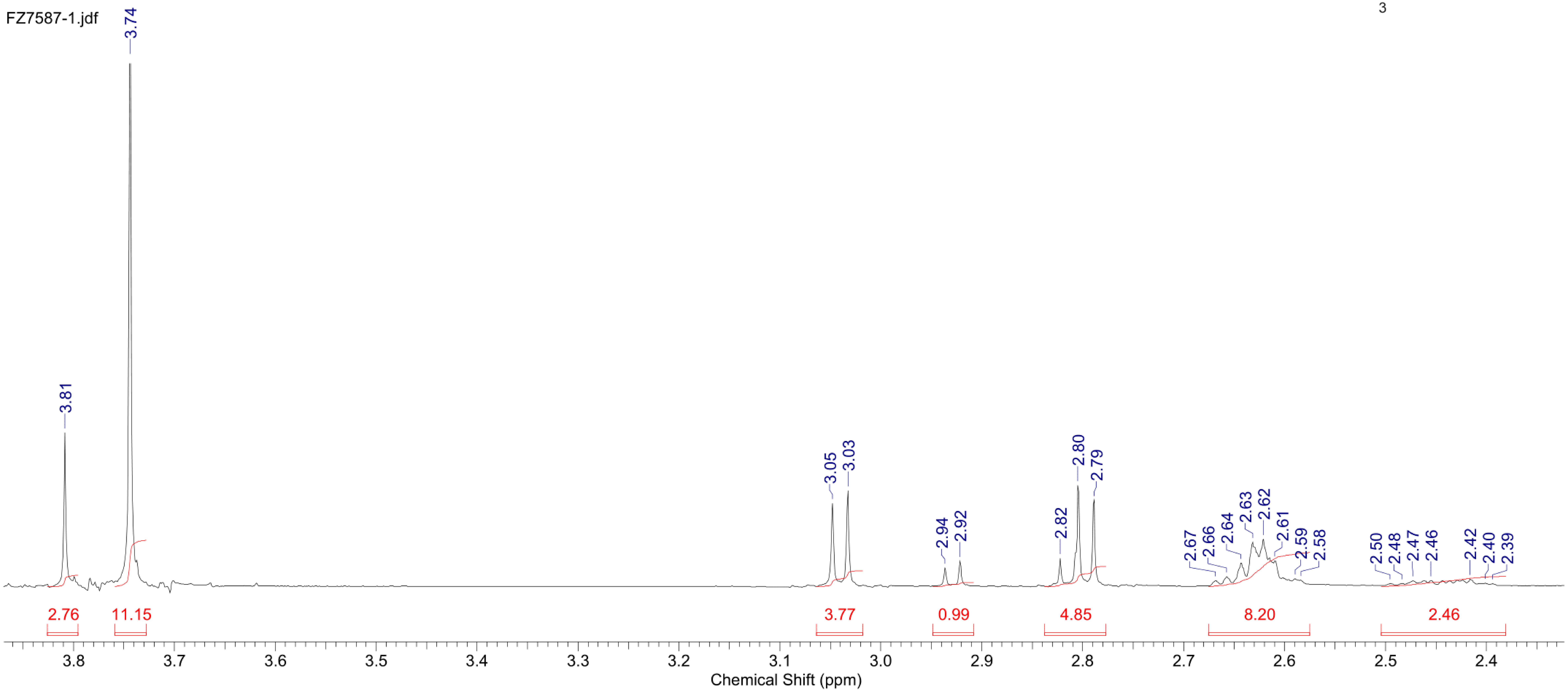

Formula	$C_{19}H_{19}NO_4$	FW	325.3585

**Acquisition Time (sec)**	0.6921	**Comment**	single pulse decoupled gated NOE	**Date**	16 Jan 2020 06:26:55
**Date Stamp**	16 Jan 2020 05:46:14	**File Name**	C:\Users\User\Desktop\14.01.20\FZ8342-1.jdf		
**Frequency (MHz)**	150.91	**Nucleus**	13C	**Number of Transients**	1000
**Origin**	ECA 600	**Original Points Count**	32768	**Owner**	delta
**Points Count**	32768	**Pulse Sequence**	single_pulse_dec	**Receiver Gain**	54.00
**Solvent**	CHLOROFORM-d	**Spectrum Offset (Hz)**	15077.9072	**Sweep Width (Hz)**	47348.49
**Temperature (degree C)**	23.200				

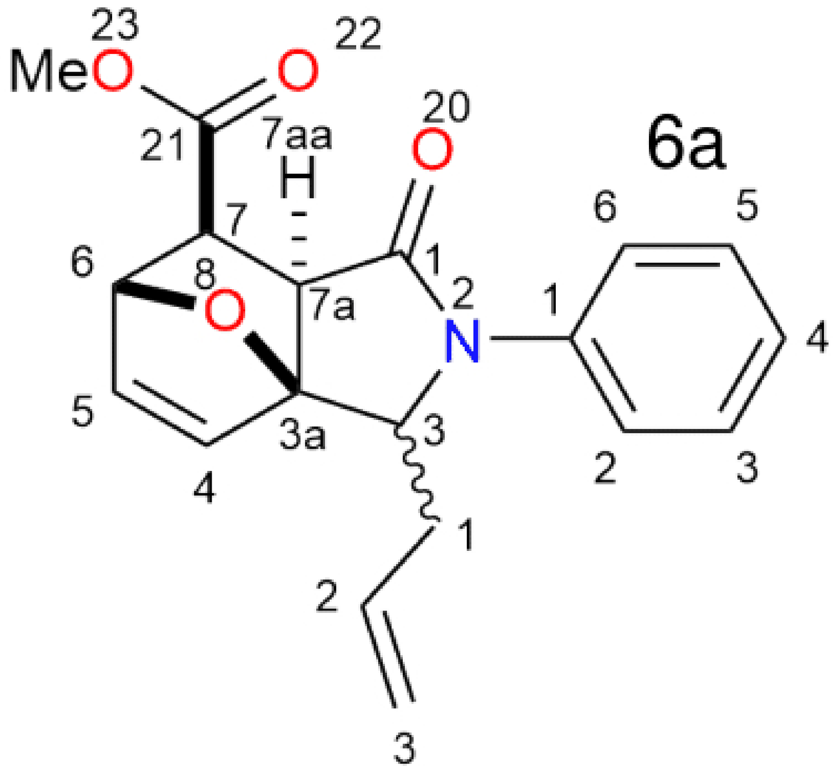

FZ8342-1.esp

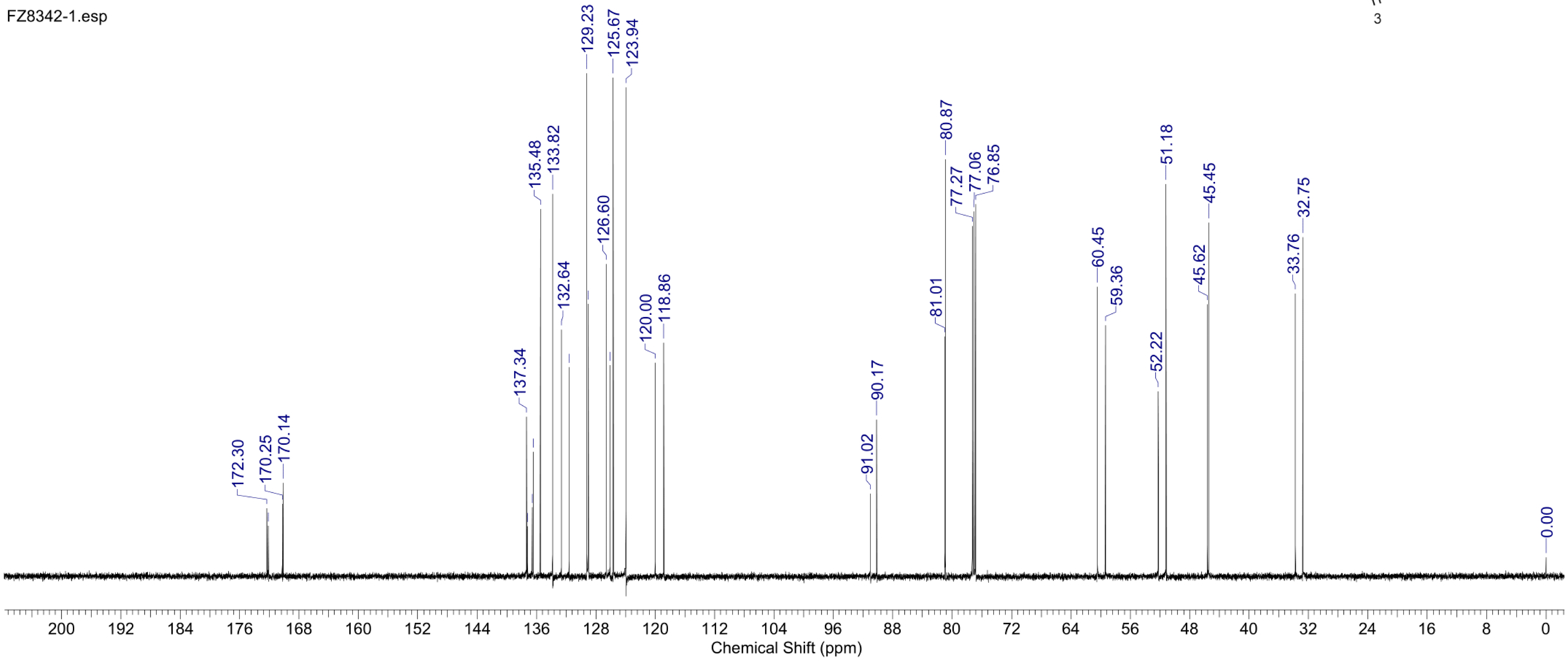

Formula	$C_{19}H_{19}NO_4$	FW	325.3585

Acquisition Time (sec)	0.6921	Comment	single pulse decoupled gated NOE			Date	16 Jan 2020 06:26:55
Date Stamp	16 Jan 2020 05:46:14			File Name	C:\Users\User\Desktop\14.01.20\FZ8342-1.jdf		
Frequency (MHz)	150.91	Nucleus	13C	Number of Transients	1000	Origin	ECA 600
Original Points Count	32768	Owner	delta	Points Count	32768	Pulse Sequence	single_pulse_dec
Receiver Gain	54.00	Solvent	CHLOROFORM-d			Spectrum Offset (Hz)	15077.9072
Sweep Width (Hz)	47348.49	Temperature (degree C)	23.200				

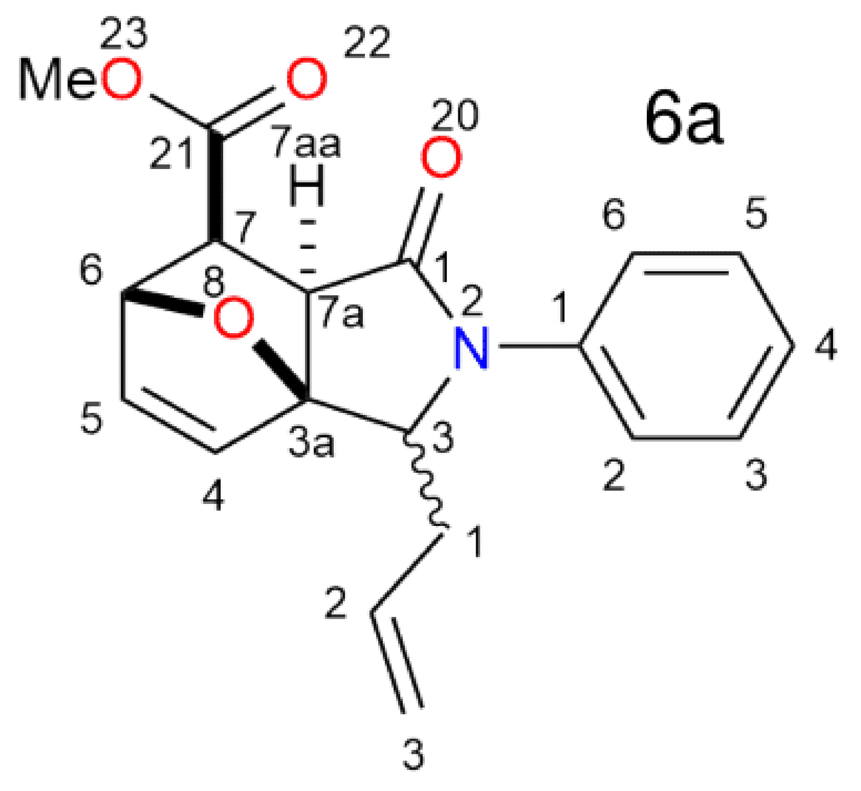

6a

FZ8342-1.esp

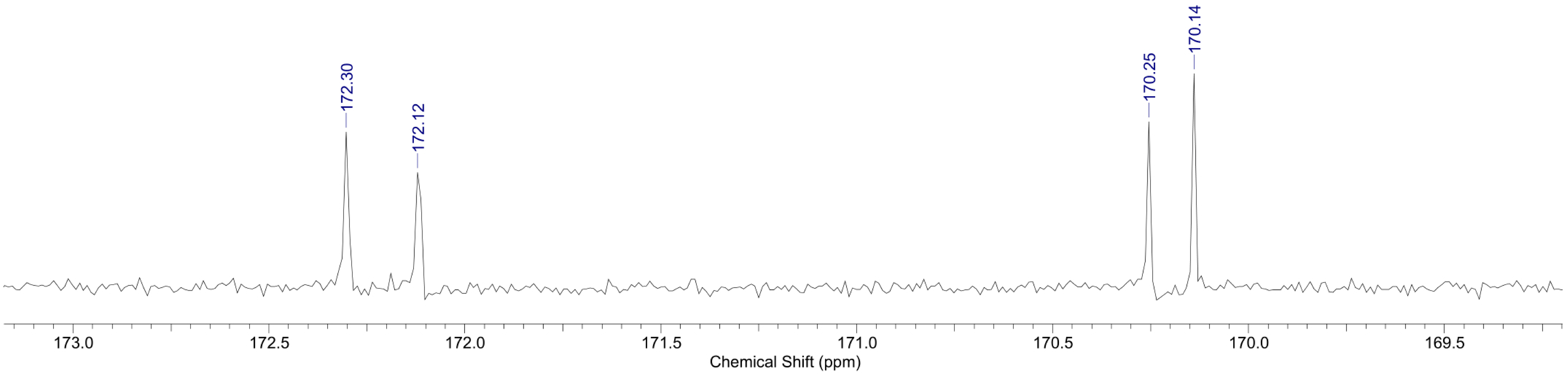

Formula $C_{19}H_{19}NO_4$	FW 325.3585

**Acquisition Time (sec)**	0.6921	**Comment**	single pulse decoupled gated NOE			**Date**	16 Jan 2020 06:26:55
**Date Stamp**	16 Jan 2020 05:46:14			**File Name**	C:\Users\User\Desktop\14.01.20\FZ8342-1.jdf		
**Frequency (MHz)**	150.91	**Nucleus**	13C	**Number of Transients**	1000	**Origin**	ECA 600
**Original Points Count**	32768	**Owner**	delta	**Points Count**	32768	**Pulse Sequence**	single_pulse_dec
**Receiver Gain**	54.00	**Solvent**	CHLOROFORM-d			**Spectrum Offset (Hz)**	15077.9072
**Sweep Width (Hz)**	47348.49	**Temperature (degree C)**	23.200				

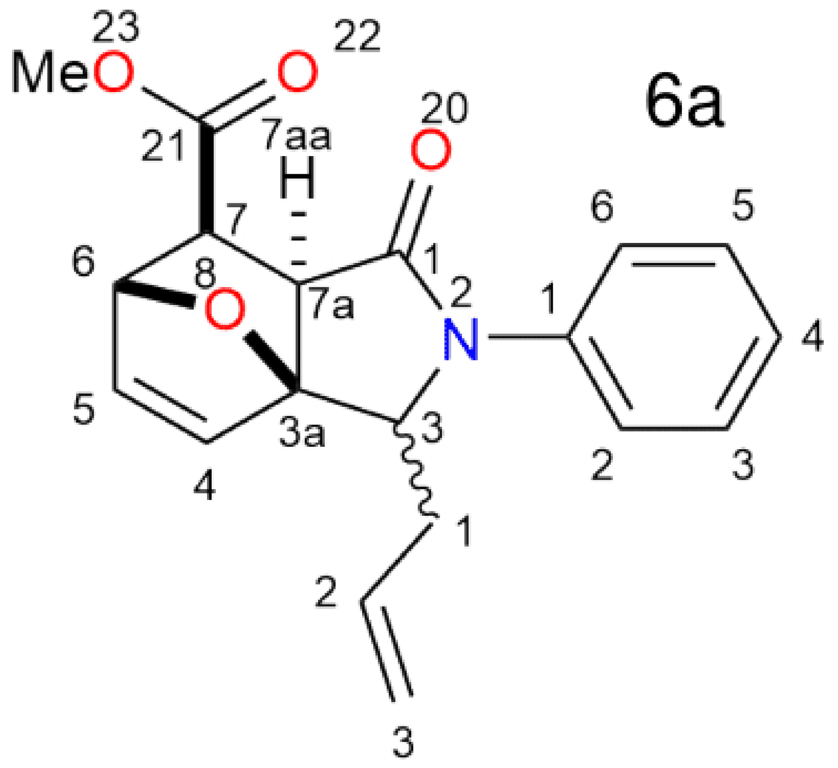

6a

FZ8342-1.esp

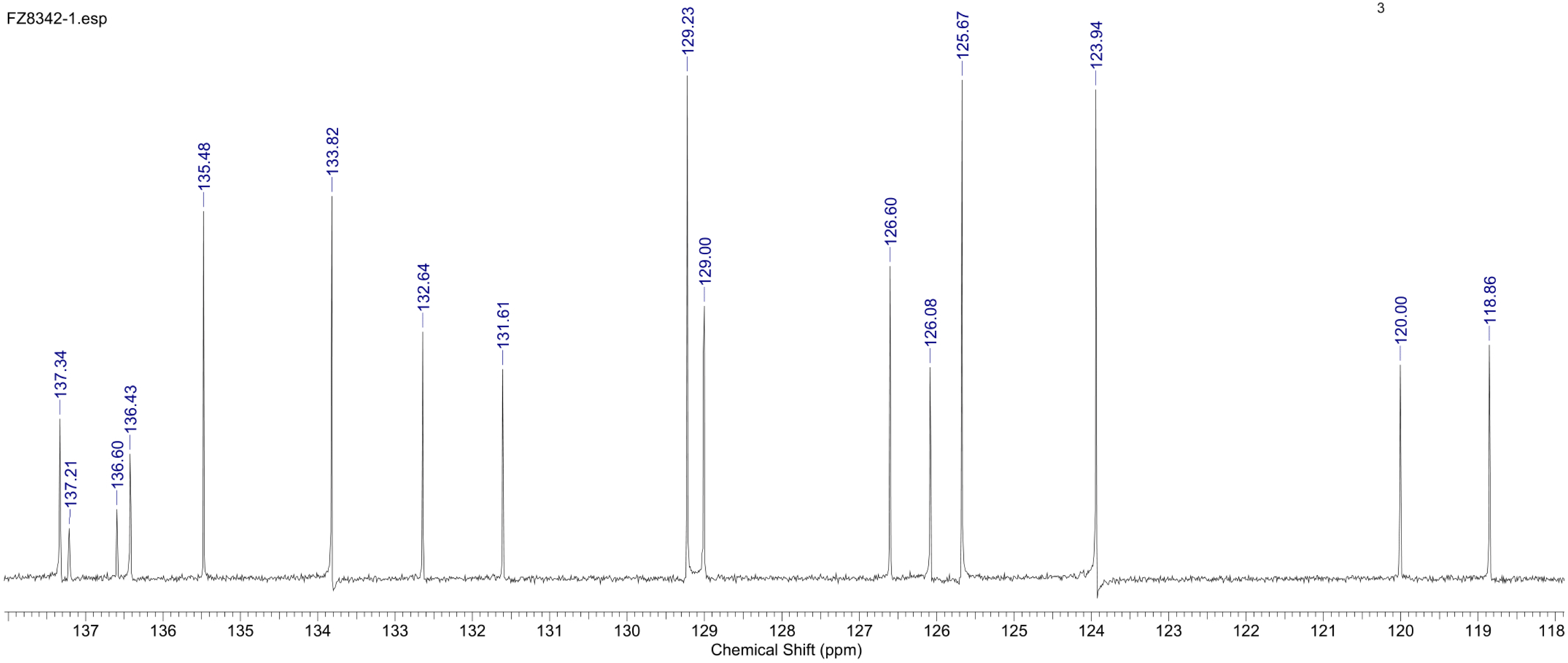

Formula $C_{19}H_{19}NO_4$	FW 325.3585

**Acquisition Time (sec)**	0.6921	**Comment**	single pulse decoupled gated NOE			**Date**	16 Jan 2020 06:26:55
**Date Stamp**	16 Jan 2020 05:46:14			**File Name**	C:\Users\User\Desktop\14.01.20\FZ8342-1.jdf		
**Frequency (MHz)**	150.91	**Nucleus**	13C	**Number of Transients**	1000	**Origin**	ECA 600
**Original Points Count**	32768	**Owner**	delta	**Points Count**	32768	**Pulse Sequence**	single_pulse_dec
**Receiver Gain**	54.00	**Solvent**	CHLOROFORM-d			**Spectrum Offset (Hz)**	15077.9072
**Sweep Width (Hz)**	47348.49	**Temperature (degree C)**	23.200				

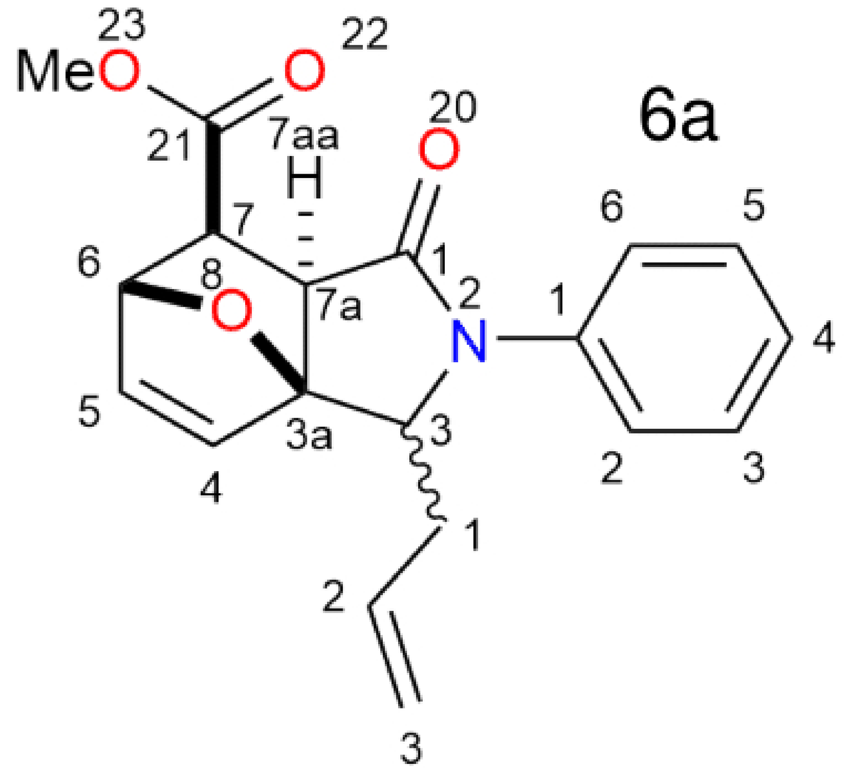

6a

FZ8342-1.esp

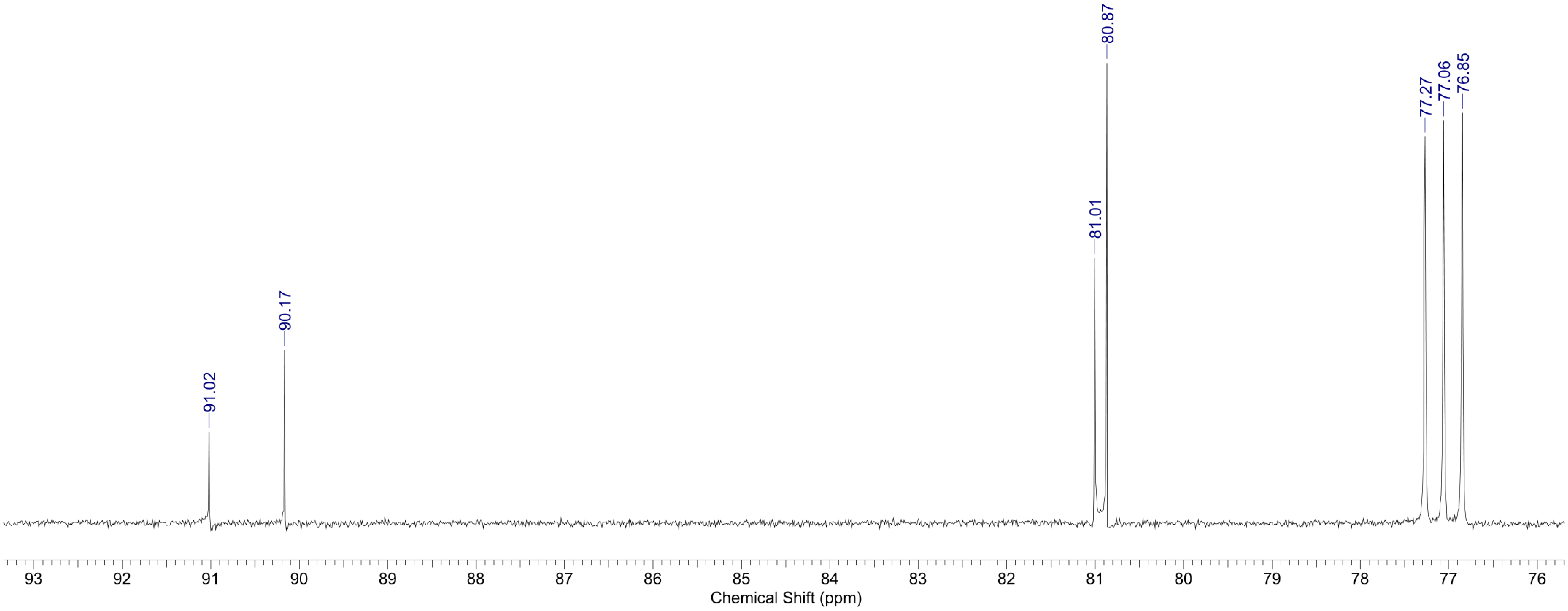

**Formula**	$C_{19}H_{19}NO_4$	**FW**	325.3585

**Acquisition Time (sec)**	0.6921	**Comment**	single pulse decoupled gated NOE	**Date**	16 Jan 2020 06:26:55
**Date Stamp**	16 Jan 2020 05:46:14	**File Name**	C:\Users\User\Desktop\14.01.20\FZ8342-1.jdf		
**Frequency (MHz)**	150.91	**Nucleus**	13C	**Number of Transients**	1000
**Origin**	ECA 600				
**Original Points Count**	32768	**Owner**	delta	**Points Count**	32768
**Pulse Sequence**	single_pulse_dec				
**Receiver Gain**	54.00	**Solvent**	CHLOROFORM-d	**Spectrum Offset (Hz)**	15077.9072
**Sweep Width (Hz)**	47348.49	**Temperature (degree C)**	23.200		

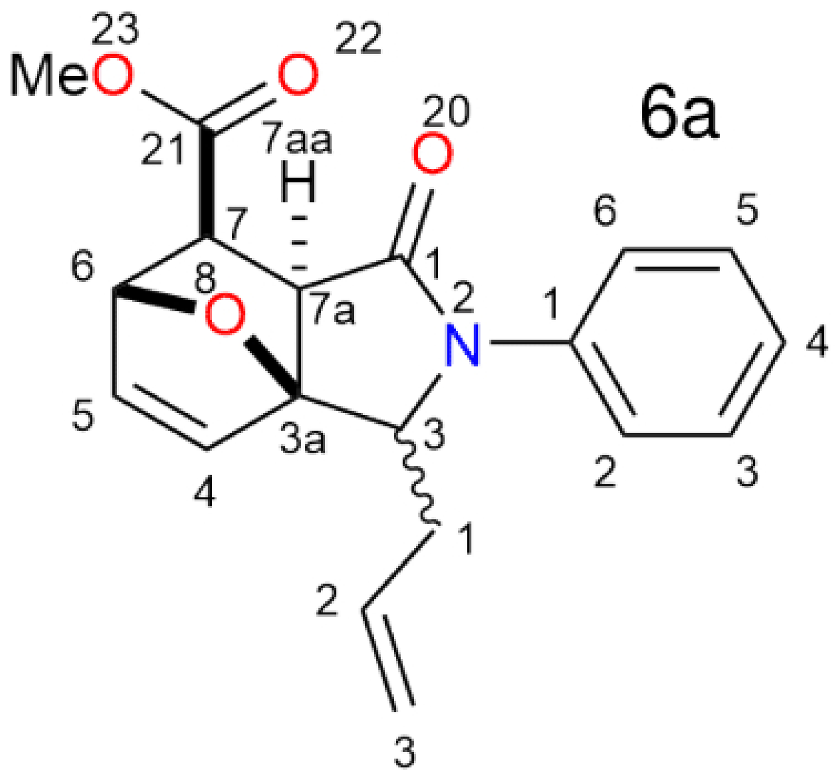

6a

FZ8342-1.esp

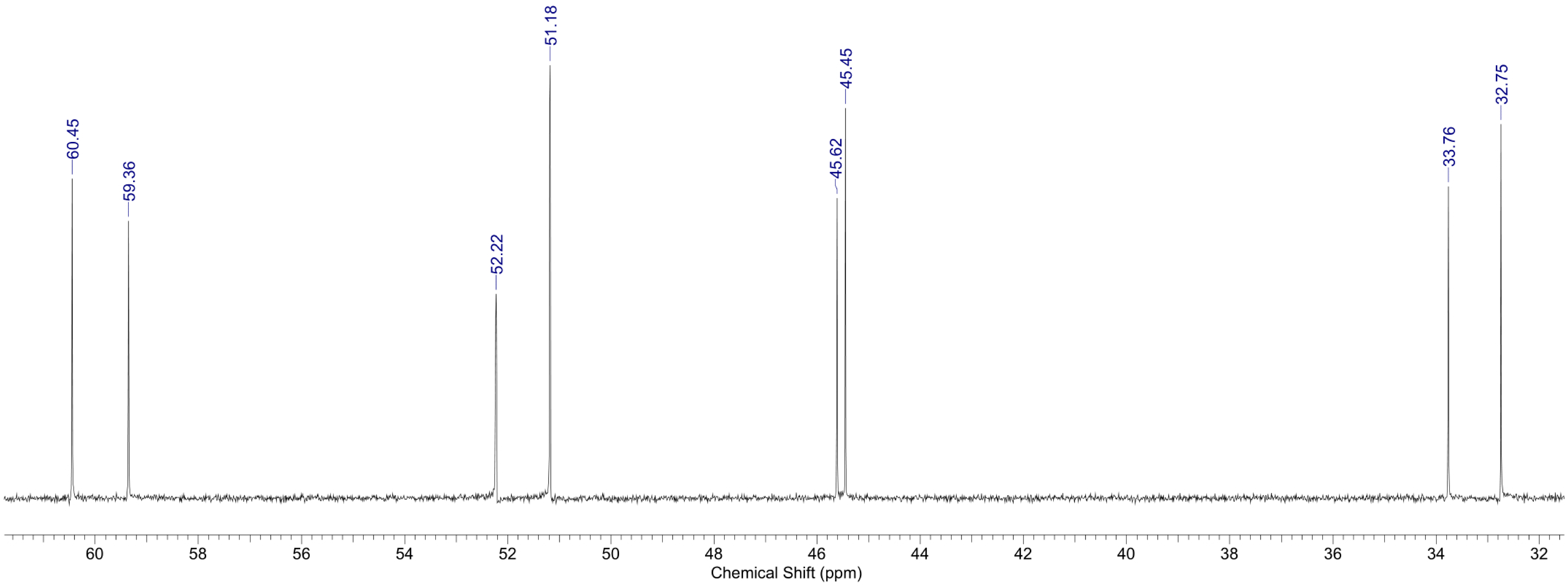

NMR spectrum data for compound 6b

Formula	$C_{20}H_{21}NO_4$	*FW*	339.3850

Acquisition Time (sec)	1.9818	***Comment***	single_pulse	***Date***	11 Oct 1990 03:21:05		
Date Stamp	21 Dec 2019 08:38:01			***File Name***	C:\Users\User\Desktop\02.12.19\FZ8315-1.jdf	***Frequency (MHz)***	600.17
Nucleus	1H	***Number of Transients***	8	***Origin***	ECA 600	***Original Points Count***	32768
Owner	delta						
Points Count	32768	***Pulse Sequence***	single_pulse.ex2	***Receiver Gain***	44.00	***Solvent***	CHLOROFORM-d
Spectrum Offset (Hz)	5412.1411	***Sweep Width (Hz)***	16534.39	***Temperature (degree C)***	23.500		

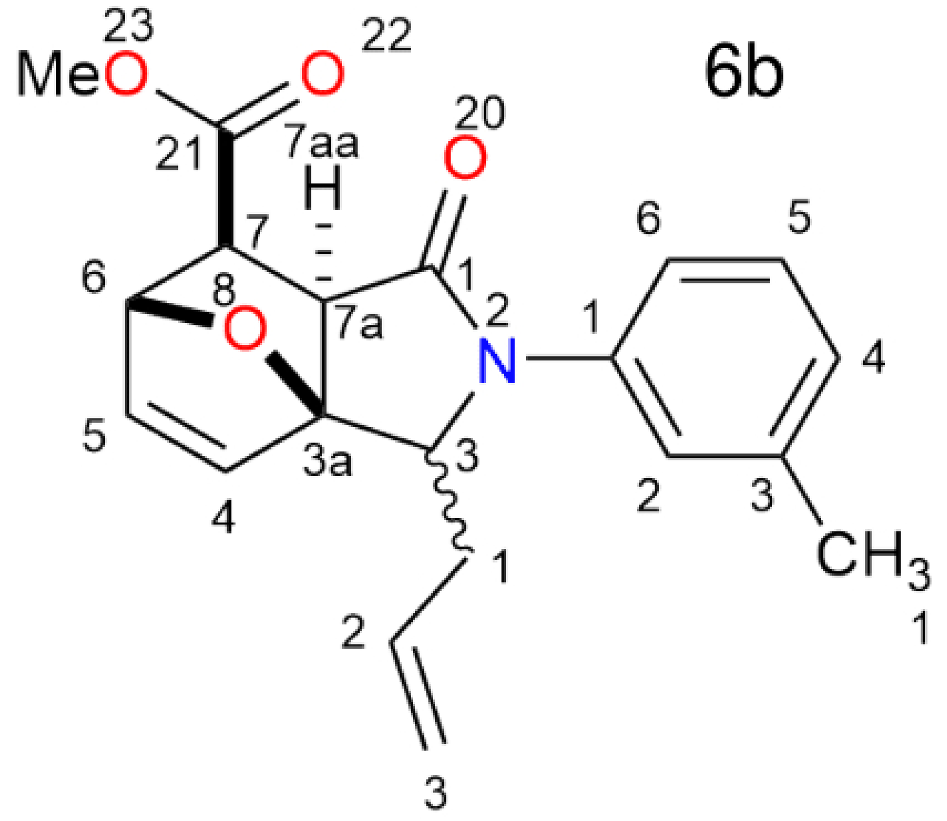

6b

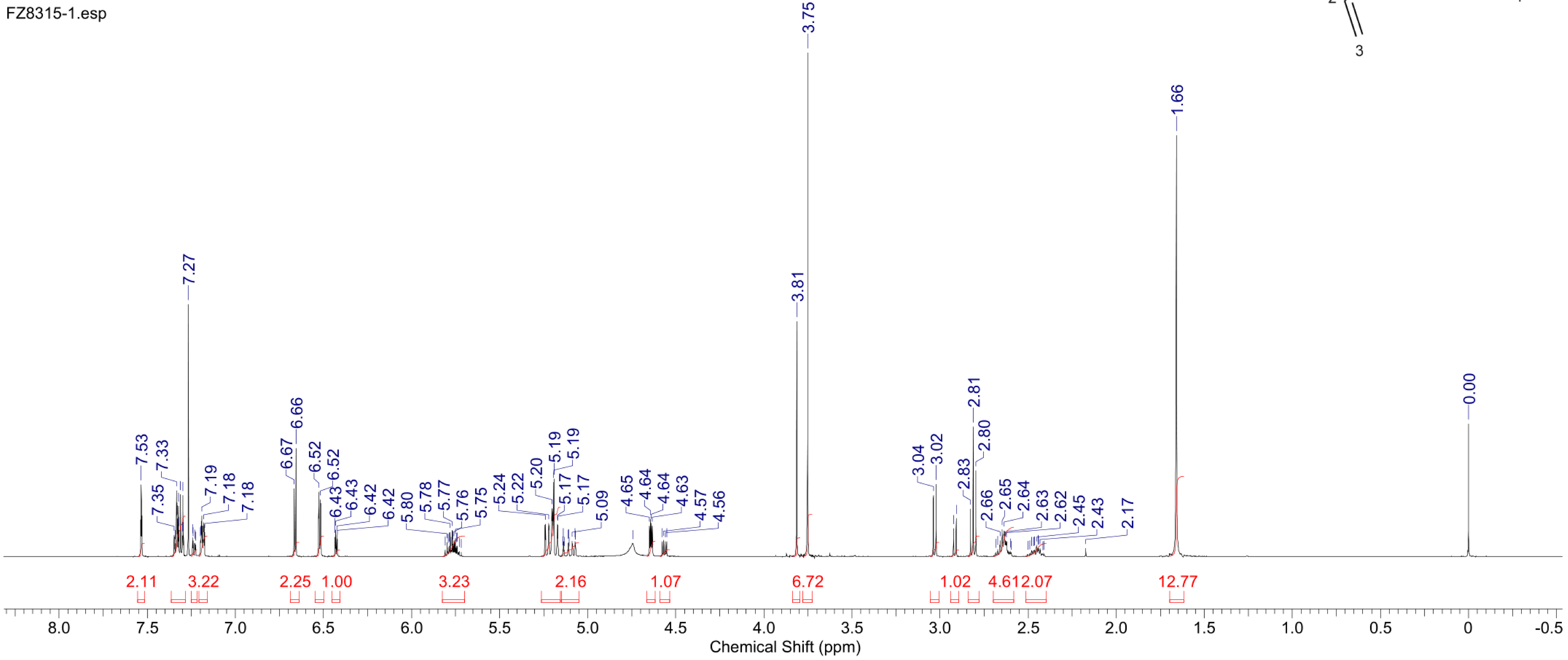

Formula $C_{20}H_{21}NO_4$	FW 339.3850

Acquisition Time (sec)	1.9818	***Comment***	single_pulse	***Date***	11 Oct 1990 03:21:05		
Date Stamp	21 Dec 2019 08:38:01			***File Name***	C:\Users\User\Desktop\02.12.19\FZ8315-1.jdf	***Frequency (MHz)***	600.17
Nucleus	1H	***Number of Transients***	8	***Origin***	ECA 600	***Original Points Count*** 32768	***Owner*** delta
Points Count	32768	***Pulse Sequence***	single_pulse.ex2			***Receiver Gain*** 44.00	***Solvent*** CHLOROFORM-d
Spectrum Offset (Hz)	5412.1411	***Sweep Width (Hz)***	16534.39	***Temperature (degree C)***	23.500		

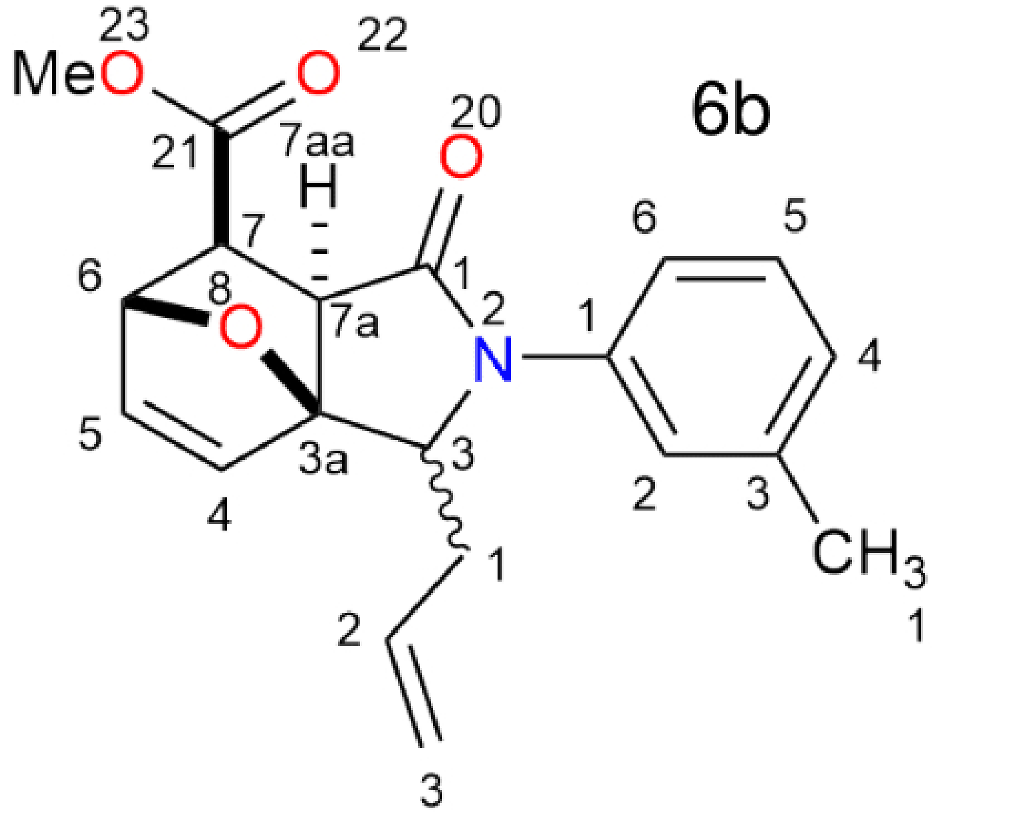

6b

FZ8315-1.esp

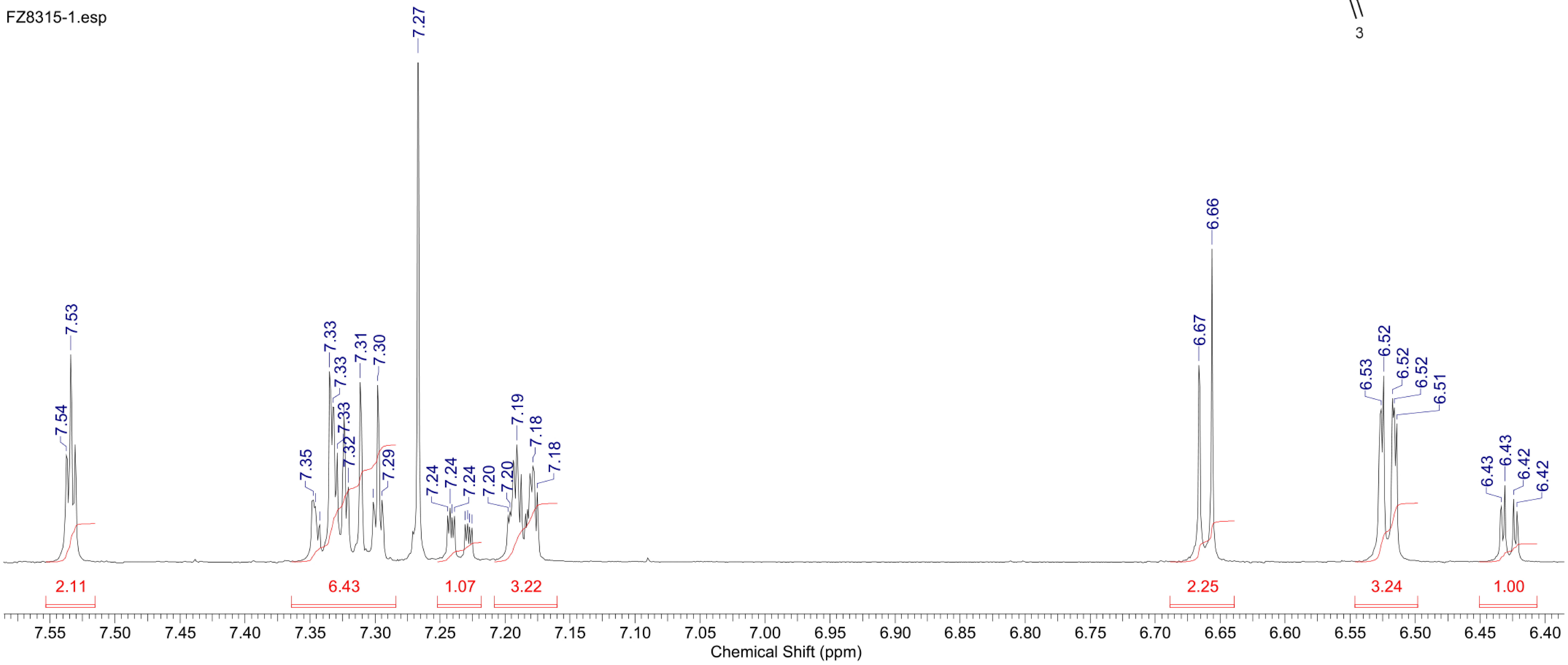

Formula	$C_{20}H_{21}NO_4$	FW	339.3850

**Acquisition Time (sec)**	1.9818	**Comment**	single_pulse	**Date**	11 Oct 1990 03:21:05		
**Date Stamp**	21 Dec 2019 08:38:01			**File Name**	C:\Users\User\Desktop\02.12.19\FZ8315-1.jdf	**Frequency (MHz)**	600.17
**Nucleus**	1H	**Number of Transients**	8	**Origin**	ECA 600	**Original Points Count**	32768
**Owner**	delta						
**Points Count**	32768	**Pulse Sequence**	single_pulse.ex2	**Receiver Gain**	44.00	**Solvent**	CHLOROFORM-d
**Spectrum Offset (Hz)**	5412.1411	**Sweep Width (Hz)**	16534.39	**Temperature (degree C)**	23.500		

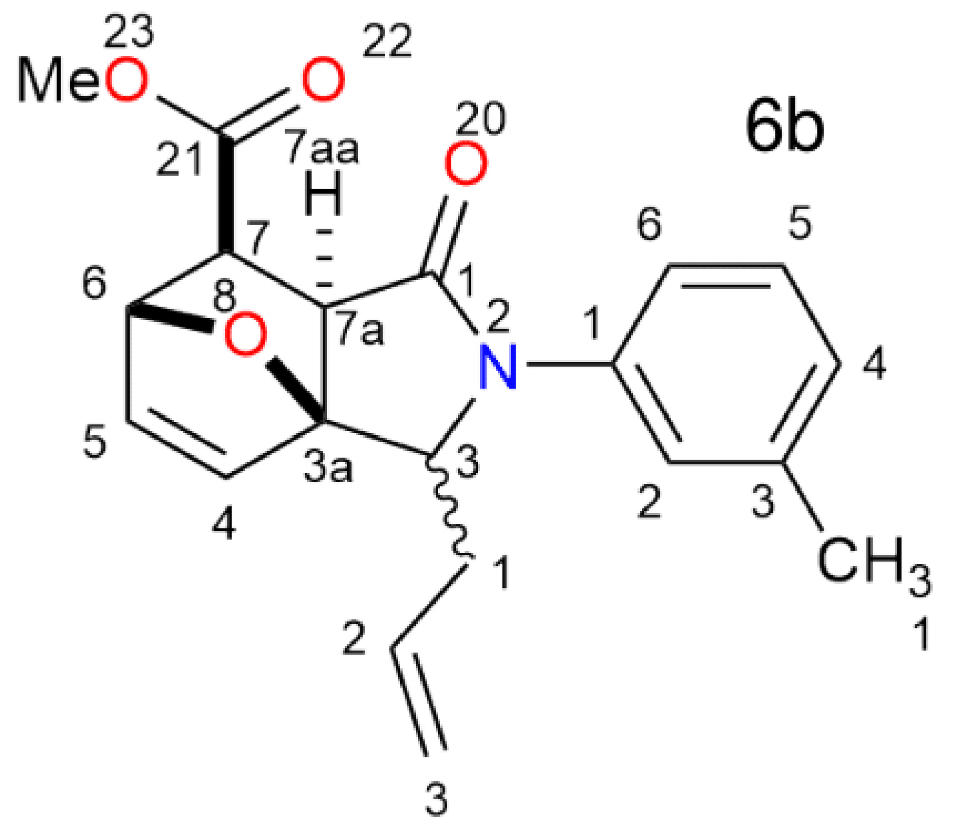

6b

FZ8315-1.esp

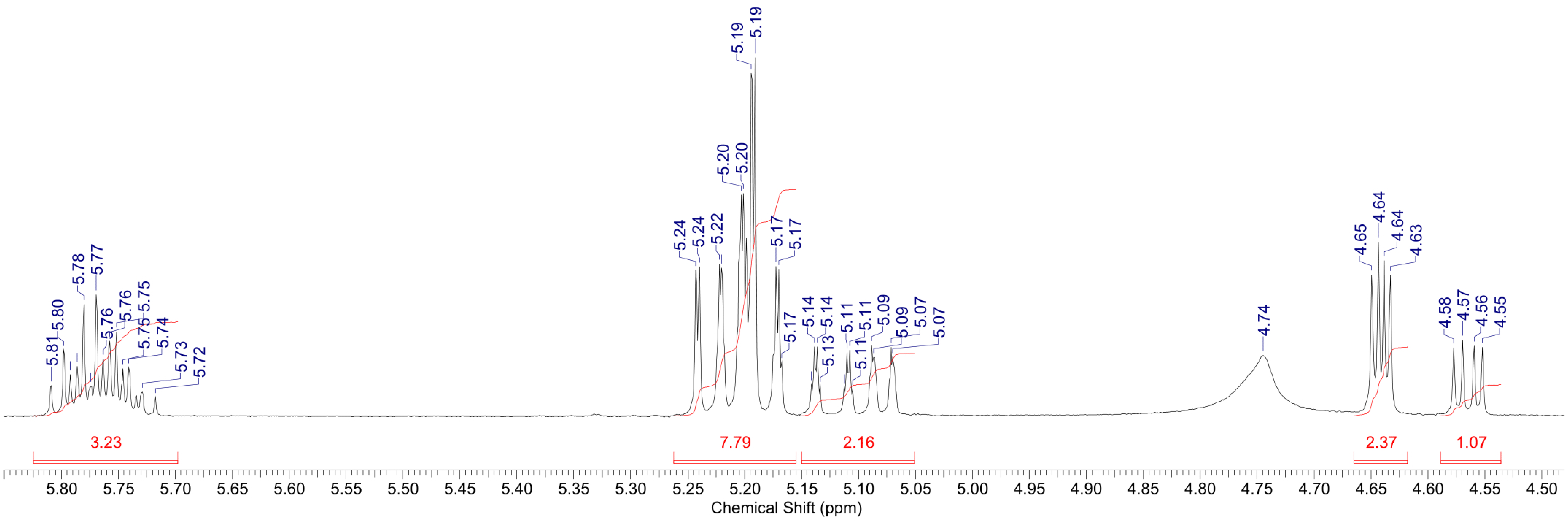

Formula	$C_{20}H_{21}NO_4$	***FW***	339.3850				

Acquisition Time (sec)	1.9818	***Comment***	single_pulse	***Date***	11 Oct 1990 03:21:05				
Date Stamp	21 Dec 2019 08:38:01			***File Name***	C:\Users\User\Desktop\02.12.19\FZ8315-1.jdf			***Frequency (MHz)***	600.17
Nucleus	1H	***Number of Transients***	8	***Origin***	ECA 600	***Original Points Count***	32768	***Owner***	delta
Points Count	32768	***Pulse Sequence***	single_pulse.ex2			***Receiver Gain***	44.00	***Solvent***	CHLOROFORM-d
Spectrum Offset (Hz)	5412.1411	***Sweep Width (Hz)***	16534.39	***Temperature (degree C)***	23.500				

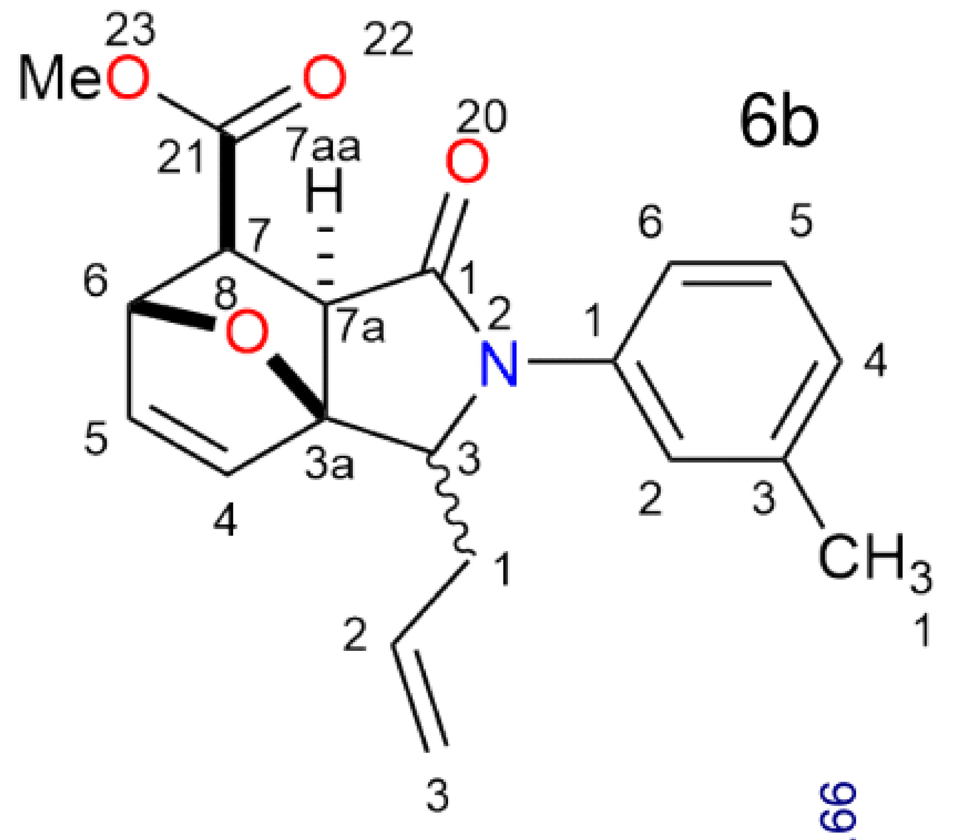

6b

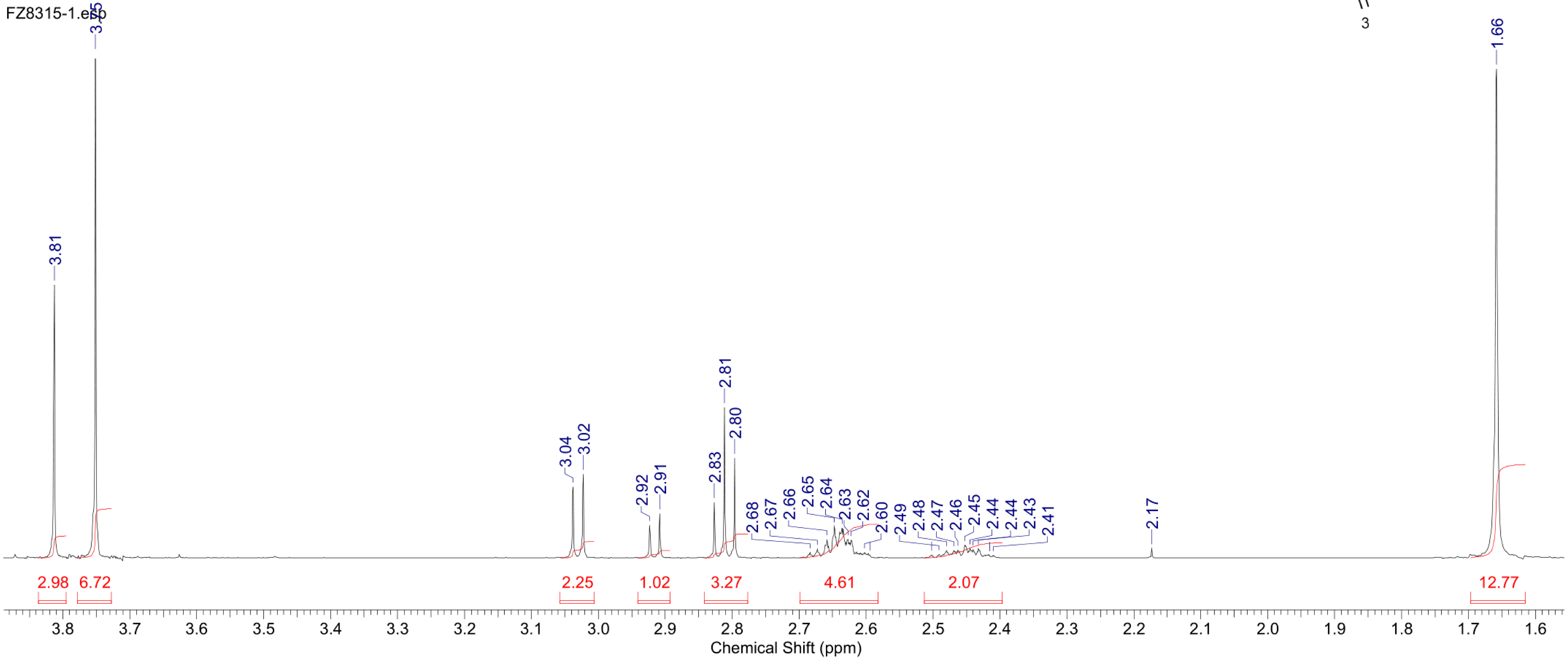

Formula $C_{20}H_{21}NO_4$	FW 339.3850

**Acquisition Time (sec)**	0.6921	**Comment**	single pulse decoupled gated NOE			**Date**	08 Oct 1990 09:09:27
**Date Stamp**	18 Dec 2019 14:26:20			**File Name**	C:\Users\User\Desktop\02.12.19\FZ8290-1.jdf		
**Frequency (MHz)**	150.91	**Nucleus**	13C	**Number of Transients**	1000	**Origin**	ECA 600
**Original Points Count**	32768	**Owner**	delta	**Points Count**	32768	**Pulse Sequence**	single_pulse_dec
**Receiver Gain**	54.00	**Solvent**	CHLOROFORM-d			**Spectrum Offset (Hz)**	15079.3525
**Sweep Width (Hz)**	47348.49	**Temperature (degree C)**	24.200				

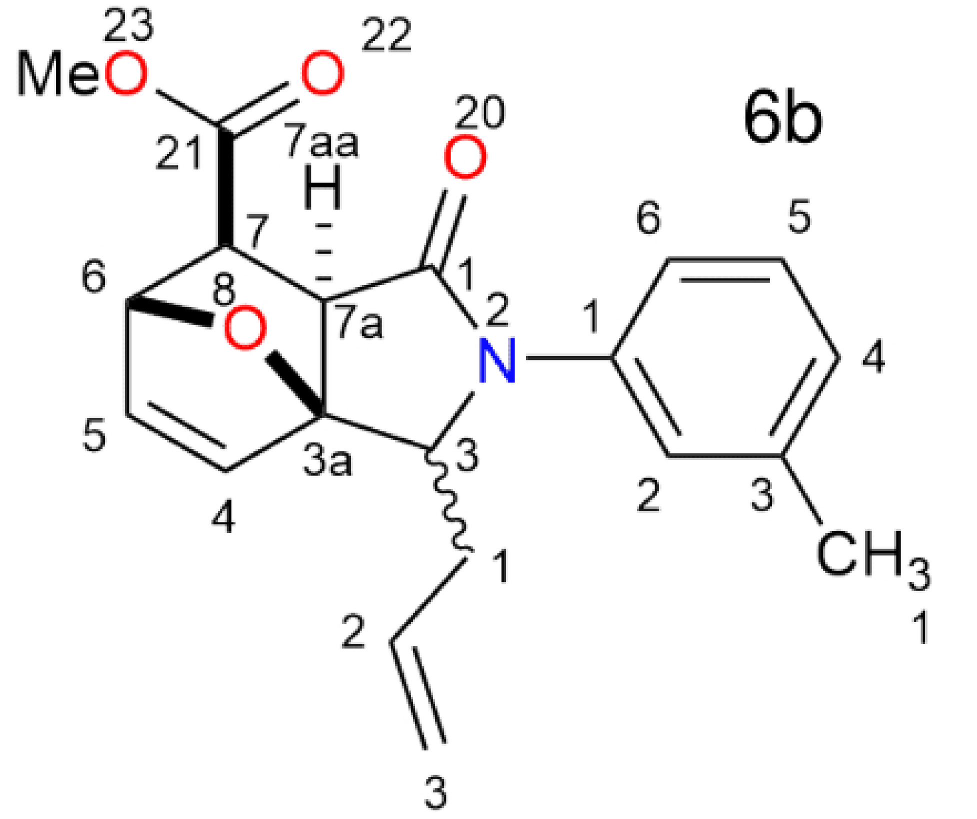

6b

FZ8290-1.jdf

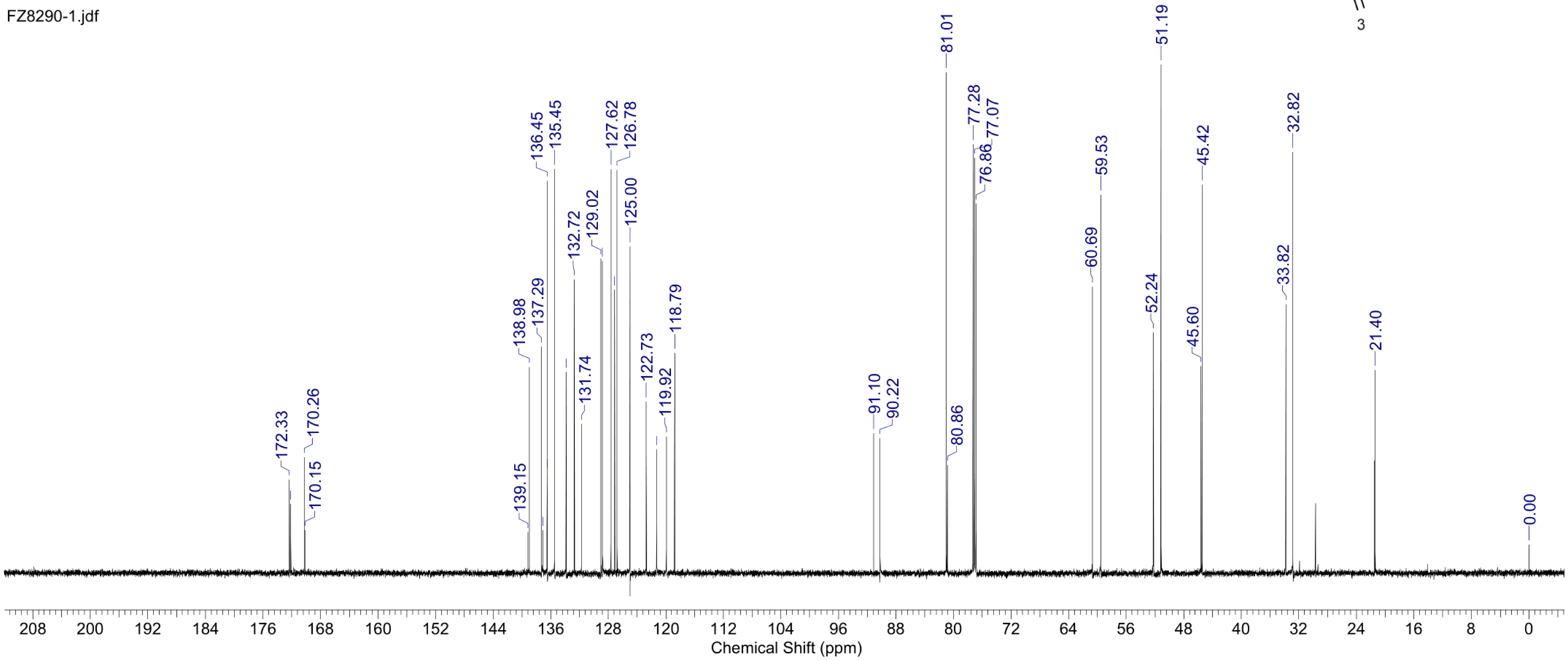

Formula	$C_{20}H_{21}NO_4$	FW	339.3850

Acquisition Time (sec)	0.6921	Comment	single pulse decoupled gated NOE			Date	08 Oct 1990 09:09:27
Date Stamp	18 Dec 2019 14:26:20			File Name	C:\Users\User\Desktop\02.12.19\FZ8290-1.jdf		
Frequency (MHz)	150.91	Nucleus	13C	Number of Transients	1000	Origin	ECA 600
Original Points Count	32768	Owner	delta	Points Count	32768	Pulse Sequence	single_pulse_dec
Receiver Gain	54.00	Solvent	CHLOROFORM-d			Spectrum Offset (Hz)	15079.3525
Sweep Width (Hz)	47348.49	Temperature (degree C)	24.200				

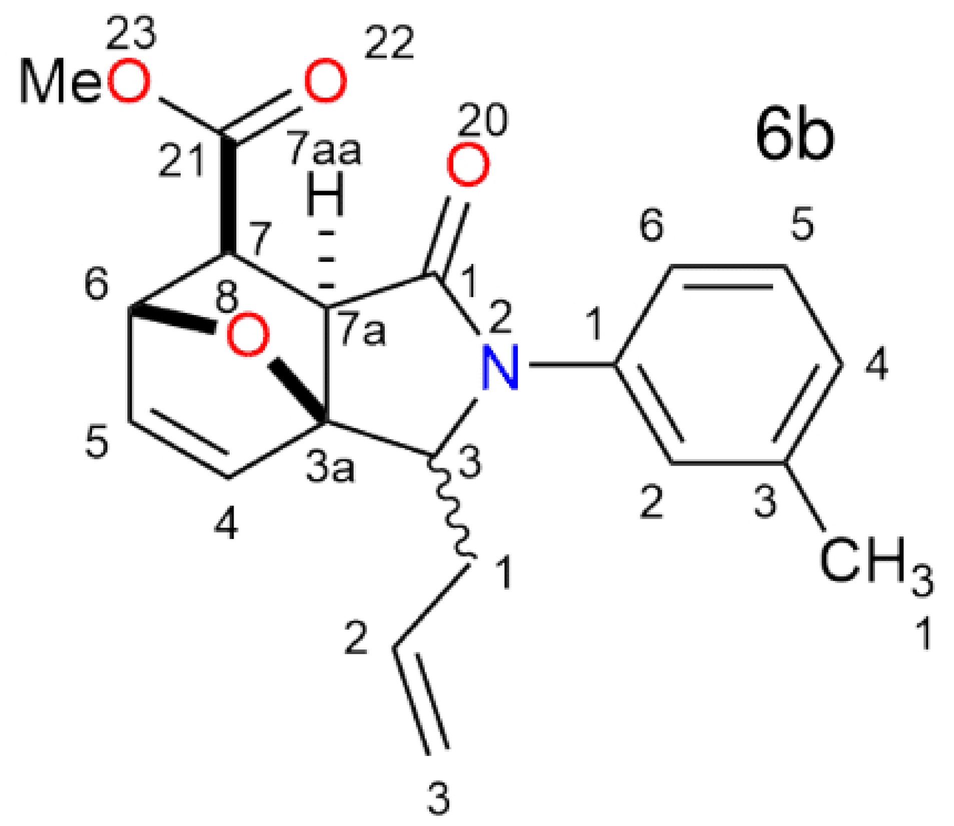

FZ8290-1.jdf

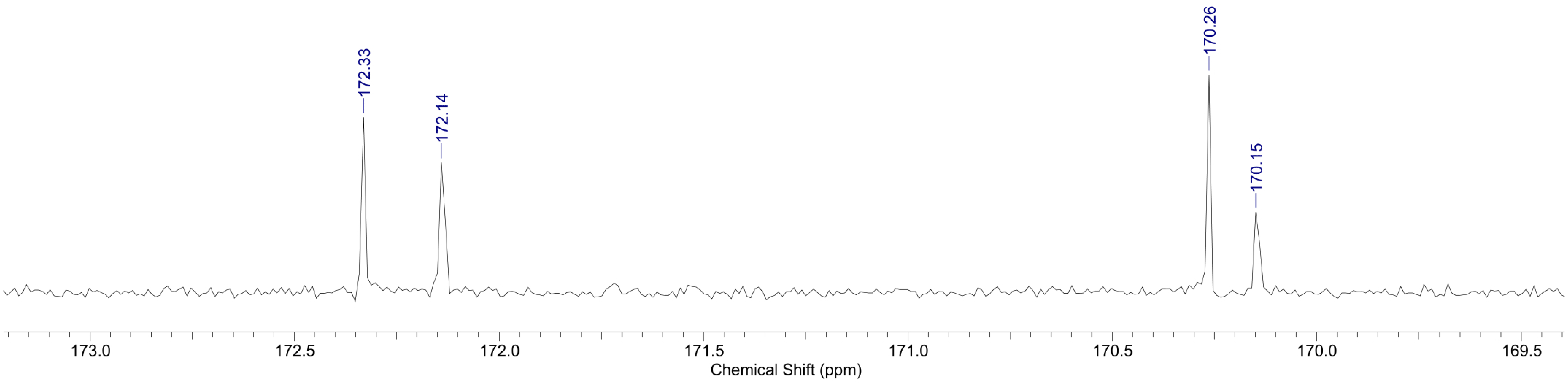

**Formula**	$C_{20}H_{21}NO_4$	**FW**	339.3850

**Acquisition Time (sec)**	0.6921	**Comment**	single pulse decoupled gated NOE	**Date**	08 Oct 1990 09:09:27
**Date Stamp**	18 Dec 2019 14:26:20	**File Name**	C:\Users\User\Desktop\02.12.19\FZ8290-1.jdf		
**Frequency (MHz)**	150.91	**Nucleus**	13C	**Number of Transients**	1000
**Origin**	ECA 600				
**Original Points Count**	32768	**Owner**	delta	**Points Count**	32768
**Pulse Sequence**	single_pulse_dec				
**Receiver Gain**	54.00	**Solvent**	CHLOROFORM-d	**Spectrum Offset (Hz)**	15079.3525
**Sweep Width (Hz)**	47348.49	**Temperature (degree C)**	24.200		

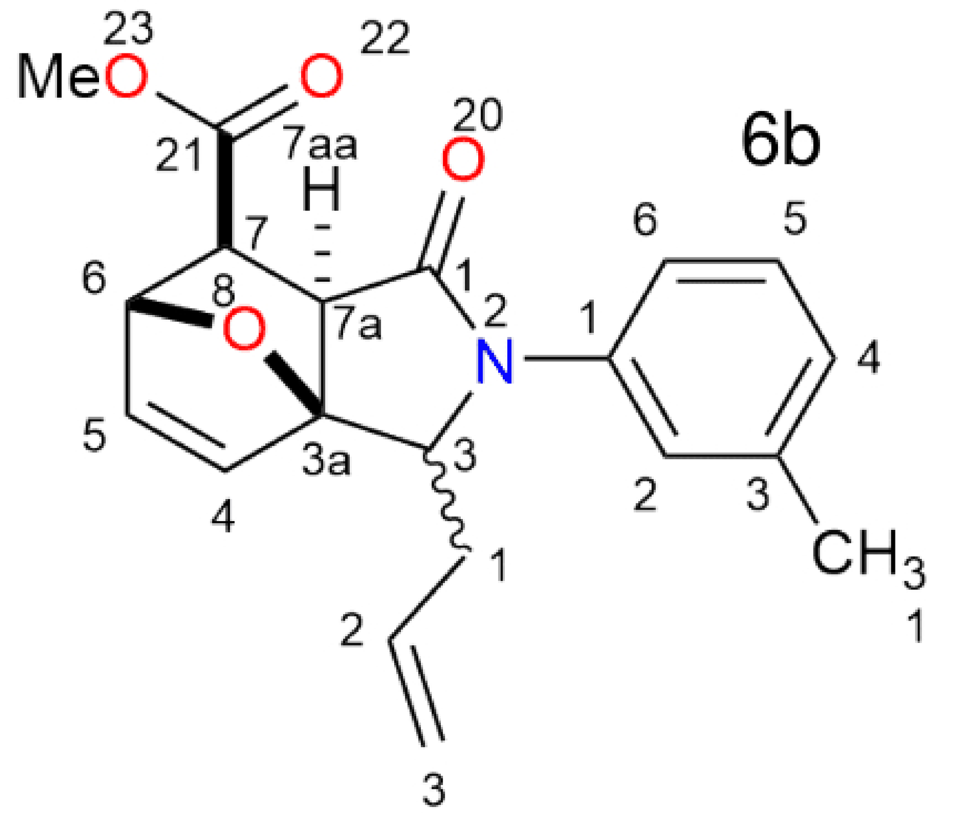

FZ8290-1.jdf

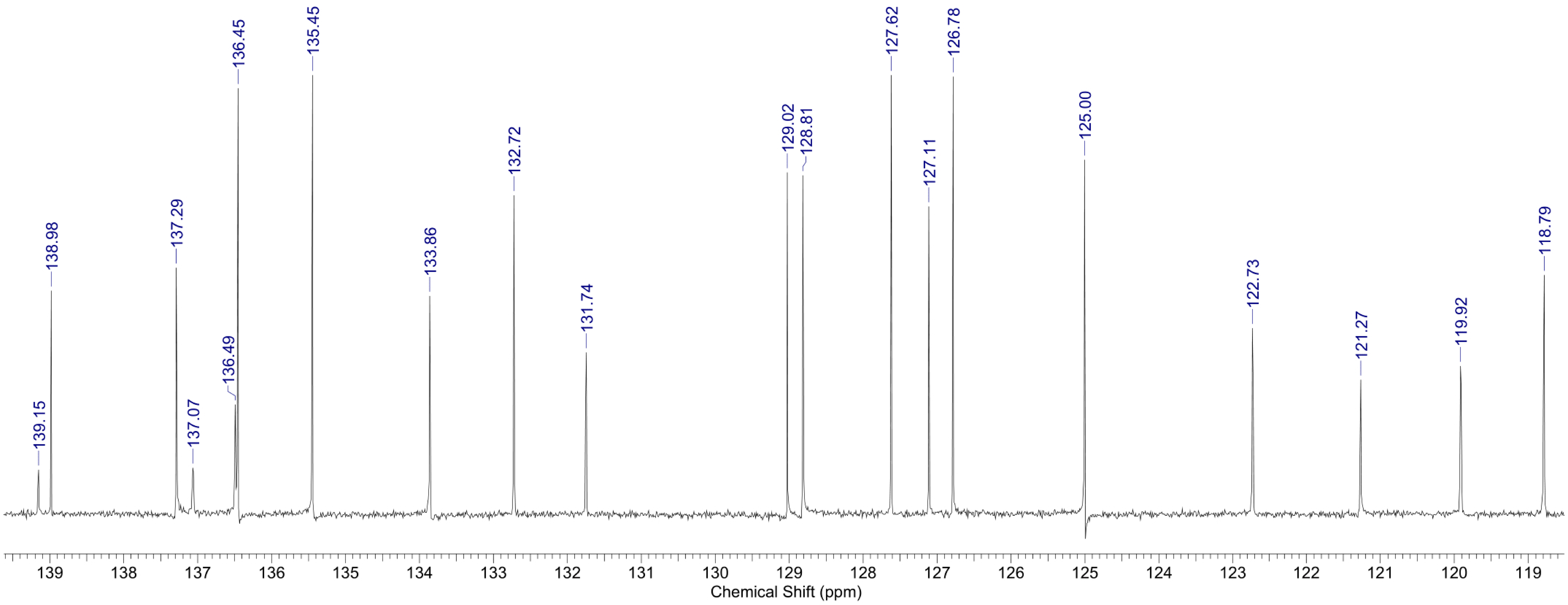

Formula $C_{20}H_{21}NO_4$	FW 339.3850

**Acquisition Time (sec)**	0.6921	**Comment**	single pulse decoupled gated NOE			**Date**	08 Oct 1990 09:09:27
**Date Stamp**	18 Dec 2019 14:26:20			**File Name**	C:\Users\User\Desktop\02.12.19\FZ8290-1.jdf		
**Frequency (MHz)**	150.91	**Nucleus**	13C	**Number of Transients**	1000	**Origin**	ECA 600
**Original Points Count**	32768	**Owner**	delta	**Points Count**	32768	**Pulse Sequence**	single_pulse_dec
**Receiver Gain**	54.00	**Solvent**	CHLOROFORM-d			**Spectrum Offset (Hz)**	15079.3525
**Sweep Width (Hz)**	47348.49	**Temperature (degree C)**	24.200				

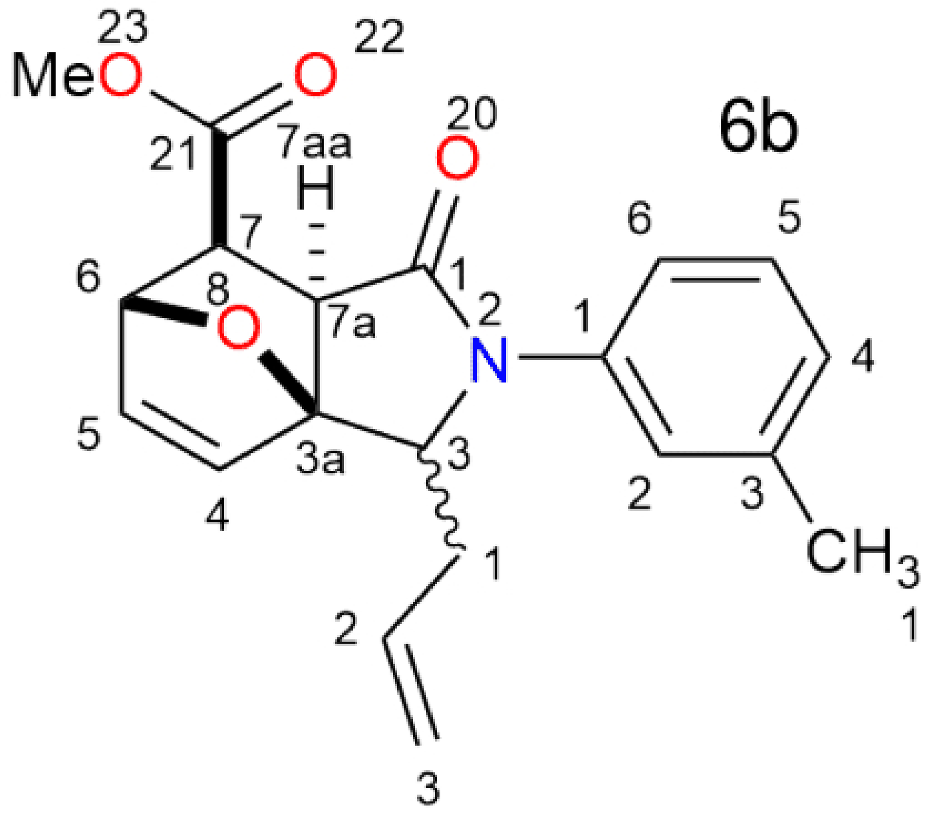

FZ8290-1.esp

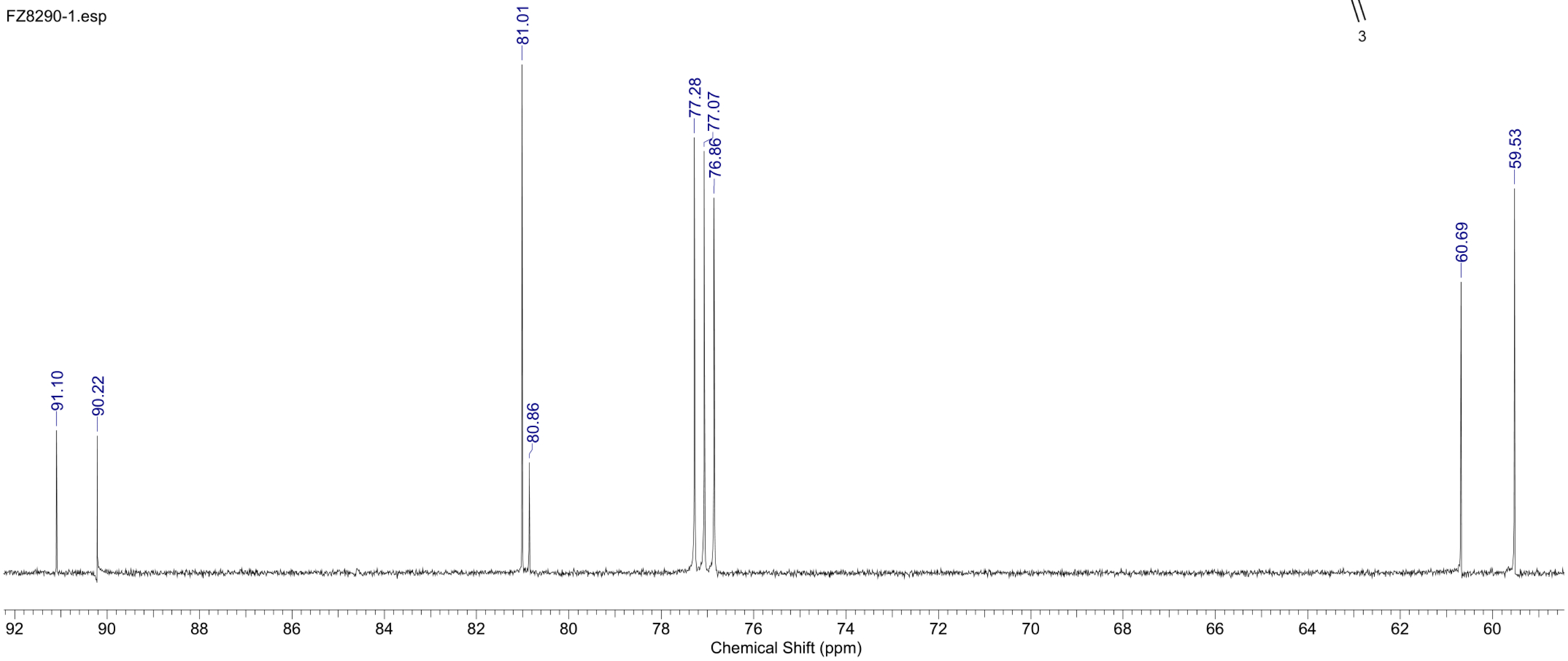

Formula	$C_{20}H_{21}NO_4$	FW	339.3850

Acquisition Time (sec)	0.6921	***Comment***	single pulse decoupled gated NOE			***Date***	08 Oct 1990 09:09:27
Date Stamp	18 Dec 2019 14:26:20			***File Name***	C:\Users\User\Desktop\02.12.19\FZ8290-1.jdf		
Frequency (MHz)	150.91	***Nucleus***	13C	***Number of Transients***	1000	***Origin***	ECA 600
Original Points Count	32768	***Owner***	delta	***Points Count***	32768	***Pulse Sequence***	single_pulse_dec
Receiver Gain	54.00	***Solvent***	CHLOROFORM-d			***Spectrum Offset (Hz)***	15079.3525
Sweep Width (Hz)	47348.49	***Temperature (degree C)***	24.200				

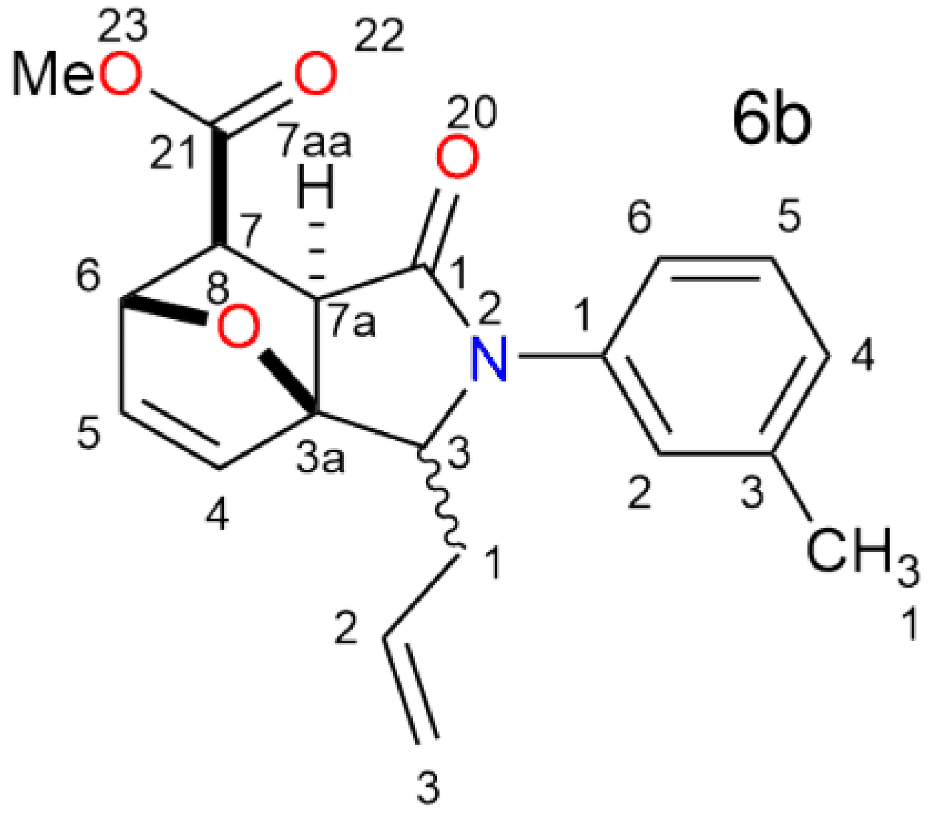

6b

FZ8290-1.esp

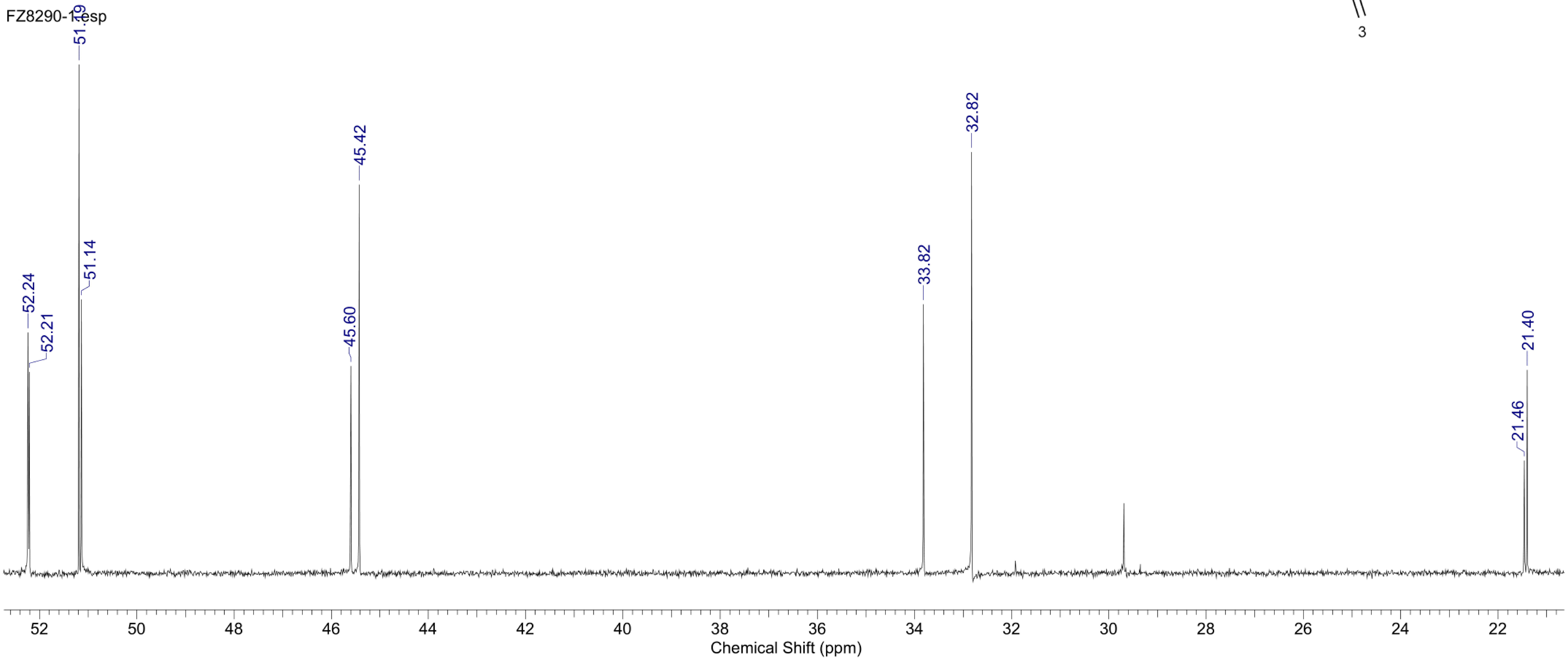

NMR spectrum data for compound 6c

Acquisition Time (sec)	1.9818	***Comment***	single_pulse	***Date***	07 May 1990 03:58:38				
Date Stamp	17 Jul 2019 10:02:17			***File Name***	C:\Users\Fedor\Desktop\15.07.19\FZ7530-1.jdf			***Frequency (MHz)***	600.17
Nucleus	1H	***Number of Transients***	8	***Origin***	ECA 600	***Original Points Count***	32768	***Owner***	delta
Points Count	32768	***Pulse Sequence***	single_pulse.ex2			***Receiver Gain***	38.00	***Solvent***	CHLOROFORM-d
Spectrum Offset (Hz)	5412.6455	***Sweep Width (Hz)***	16534.39	***Temperature (degree C)***	22.200				

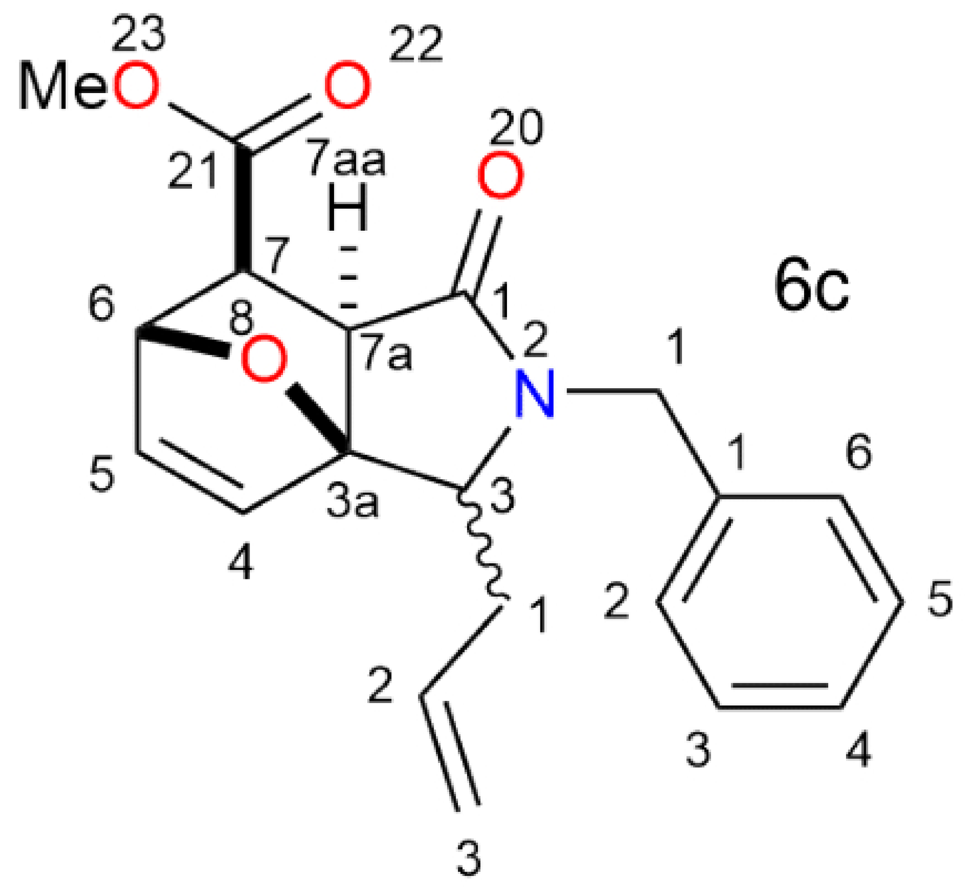

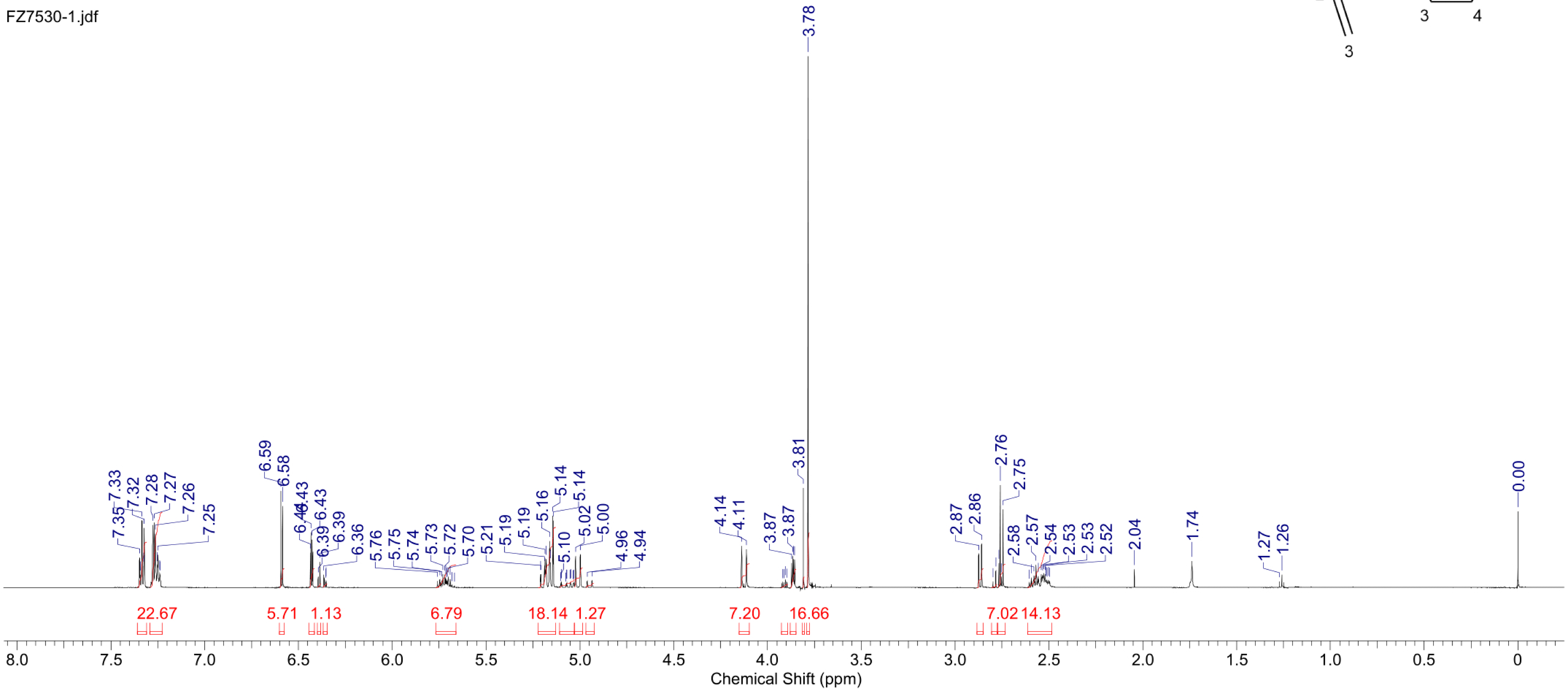

**Acquisition Time (sec)**	1.9818	**Comment**	single_pulse	**Date**	07 May 1990 03:58:38		
**Date Stamp**	17 Jul 2019 10:02:17			**File Name**	C:\Users\Fedor\Desktop\15.07.19\FZ7530-1.jdf	**Frequency (MHz)**	600.17
**Nucleus**	1H	**Number of Transients**	8	**Origin**	ECA 600	**Original Points Count** 32768	**Owner** delta
**Points Count**	32768	**Pulse Sequence**	single_pulse.ex2			**Receiver Gain** 38.00	**Solvent** CHLOROFORM-d
**Spectrum Offset (Hz)**	5412.6455	**Sweep Width (Hz)**	16534.39	**Temperature (degree C)**	22.200		

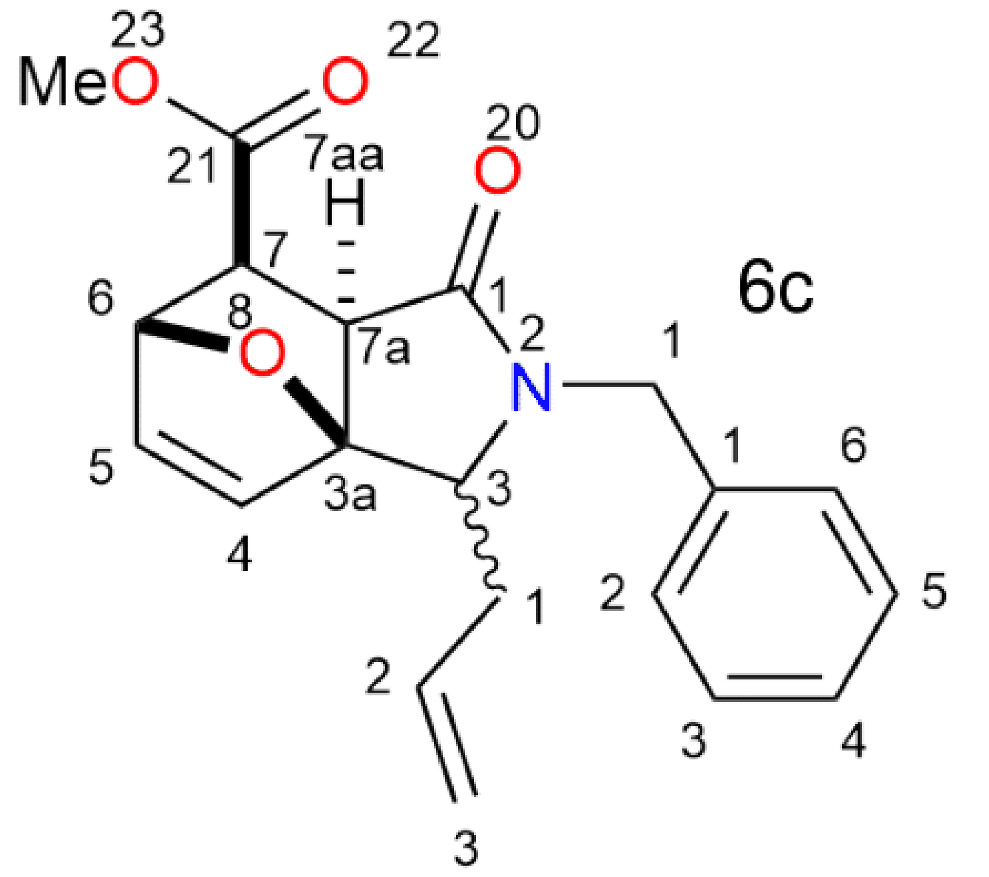

6c

FZ7530-1.jdf

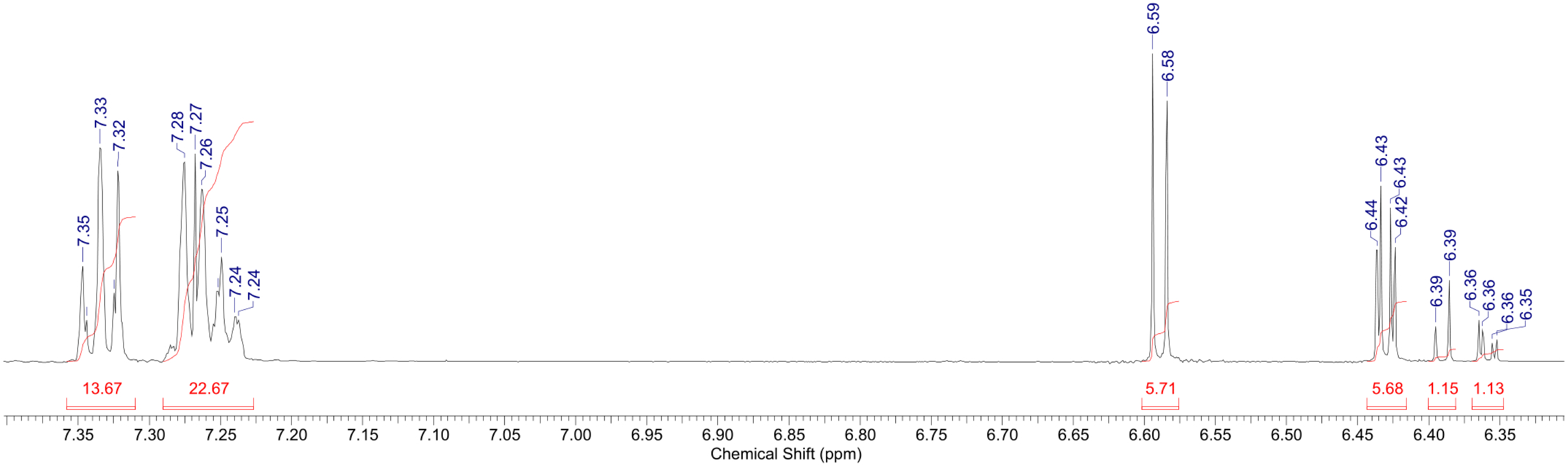

Acquisition Time (sec)	1.9818	***Comment***	single_pulse	***Date***	07 May 1990 03:58:38				
Date Stamp	17 Jul 2019 10:02:17			***File Name***	C:\Users\Fedor\Desktop\15.07.19\FZ7530-1.jdf			***Frequency (MHz)***	600.17
Nucleus	1H	***Number of Transients***	8	***Origin***	ECA 600	***Original Points Count***	32768	***Owner***	delta
Points Count	32768	***Pulse Sequence***	single_pulse.ex2			***Receiver Gain***	38.00	***Solvent***	CHLOROFORM-d
Spectrum Offset (Hz)	5412.6455	***Sweep Width (Hz)***	16534.39	***Temperature (degree C)***	22.200				

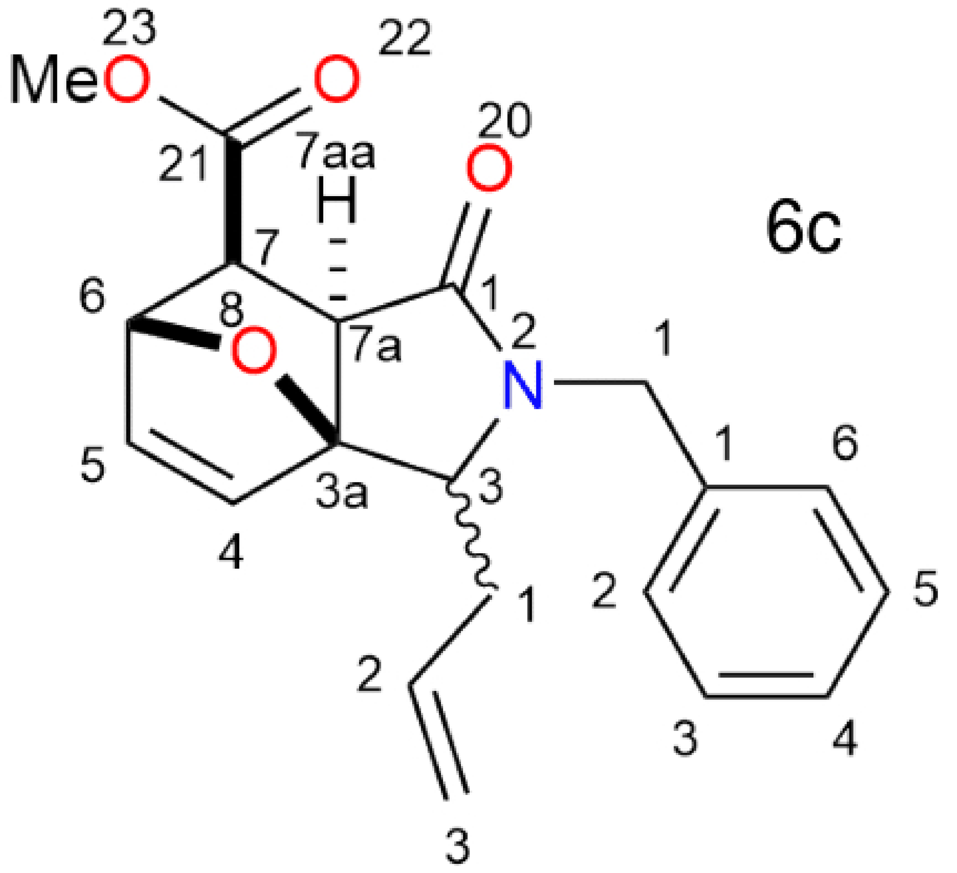

6c

FZ7530-1.jdf

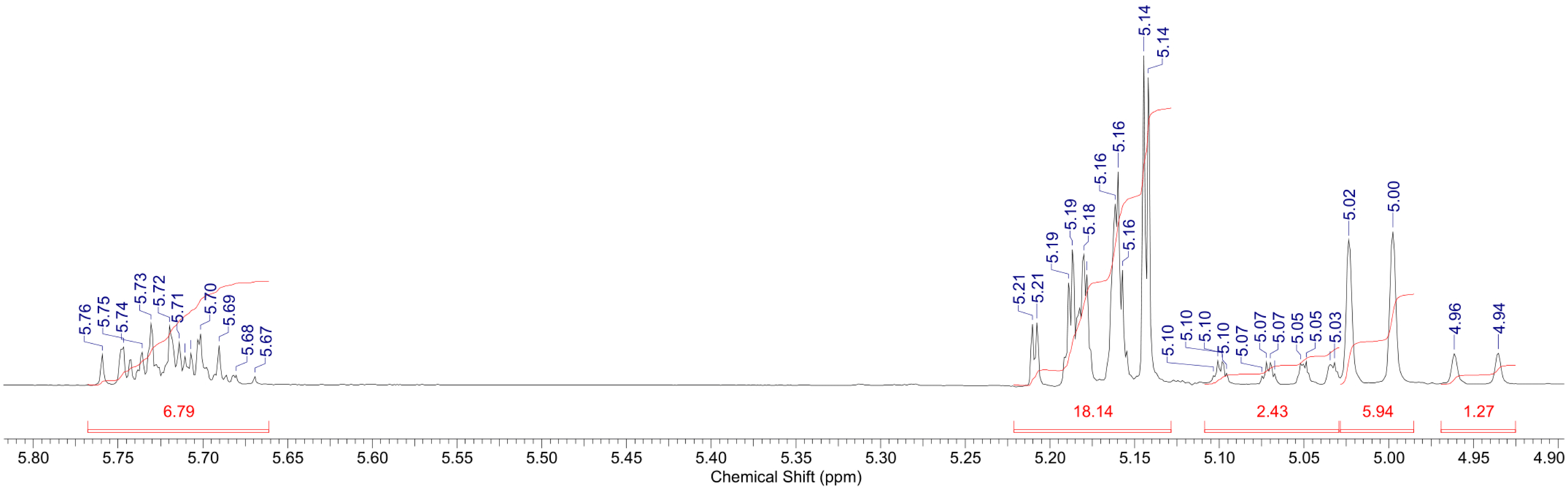

Acquisition Time (sec)	1.9818	***Comment***	single_pulse	***Date***	07 May 1990 03:58:38		
Date Stamp	17 Jul 2019 10:02:17			***File Name***	C:\Users\Fedor\Desktop\15.07.19\FZ7530-1.jdf	***Frequency (MHz)***	600.17
Nucleus	1H	***Number of Transients***	8	***Origin***	ECA 600	***Original Points Count***	32768
Owner	delta						
Points Count	32768	***Pulse Sequence***	single_pulse.ex2	***Receiver Gain***	38.00	***Solvent***	CHLOROFORM-d
Spectrum Offset (Hz)	5412.6455	***Sweep Width (Hz)***	16534.39	***Temperature (degree C)***	22.200		

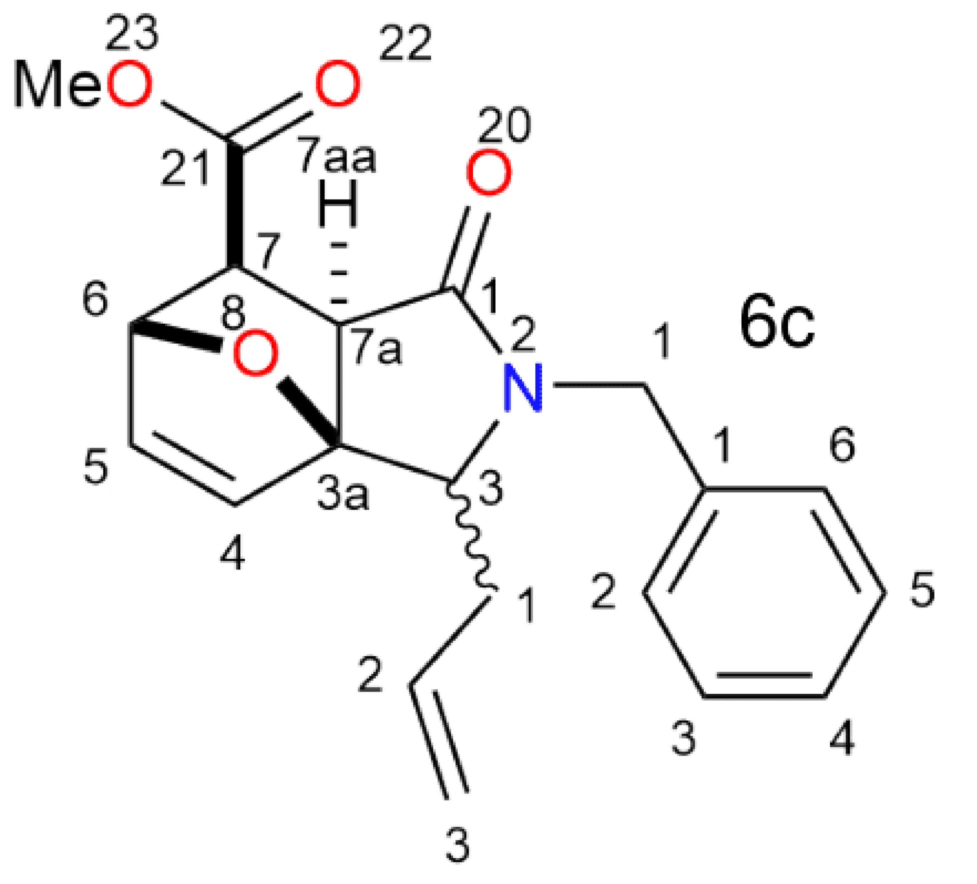

FZ7530-1.jdf

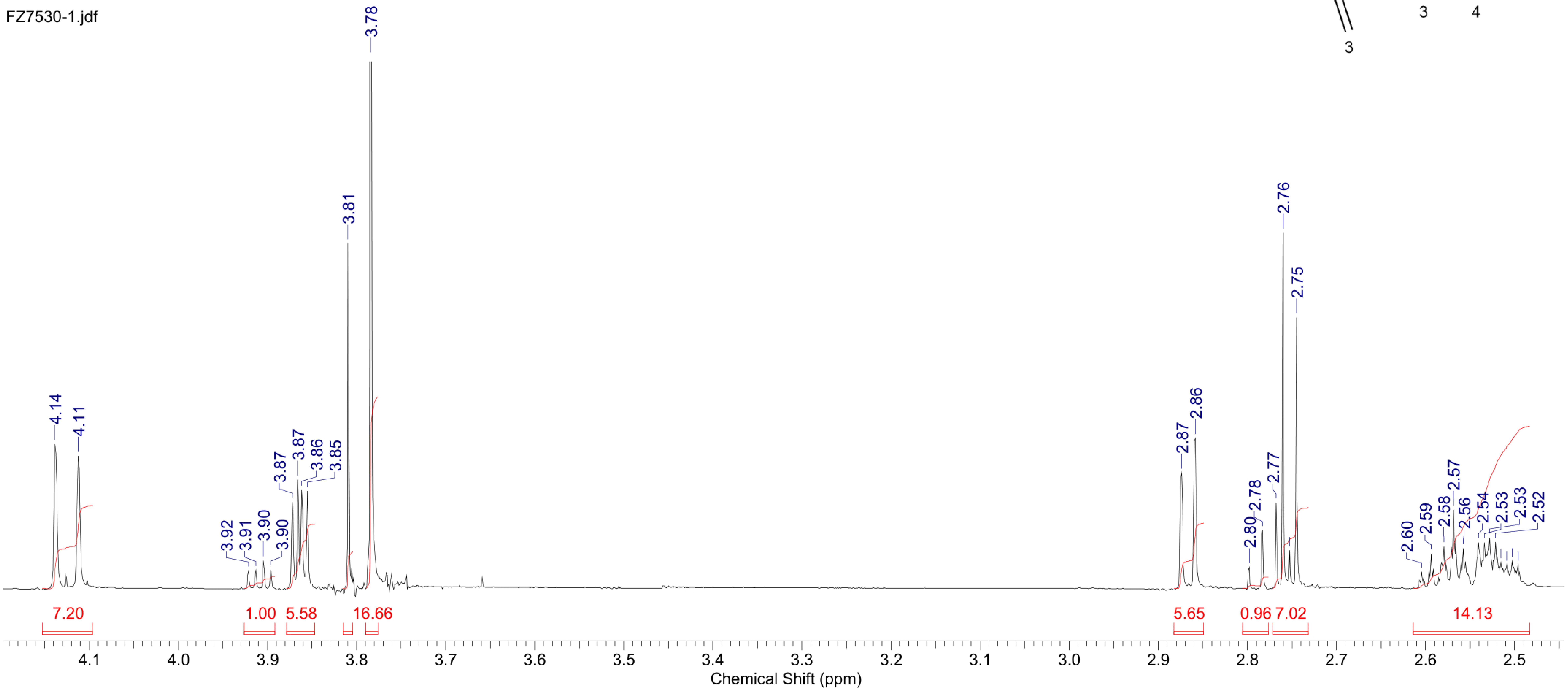

14.07.2020 18:43:15

**Formula** $C_{21}H_{23}NO_4$	**FW** 353.4116

**Acquisition Time (sec)**	0.6921	**Comment**	single pulse decoupled gated NOE			**Date**	12 Aug 1990 06:09:41
**Date Stamp**	22 Oct 2019 12:12:50			**File Name**	C:\USERS\Лаба534\DOWNLOADS\FZ7998-1.JDF		
**Frequency (MHz)**	150.91	**Nucleus**	13C	**Number of Transients**	800	**Origin**	ECA 600
**Original Points Count**	32768	**Owner**	delta	**Points Count**	32768	**Pulse Sequence**	single_pulse_dec
**Receiver Gain**	54.00	**Solvent**	CHLOROFORM-d			**Spectrum Offset (Hz)**	15079.3525
**Sweep Width (Hz)**	47348.49	**Temperature (degree C)**	24.700				

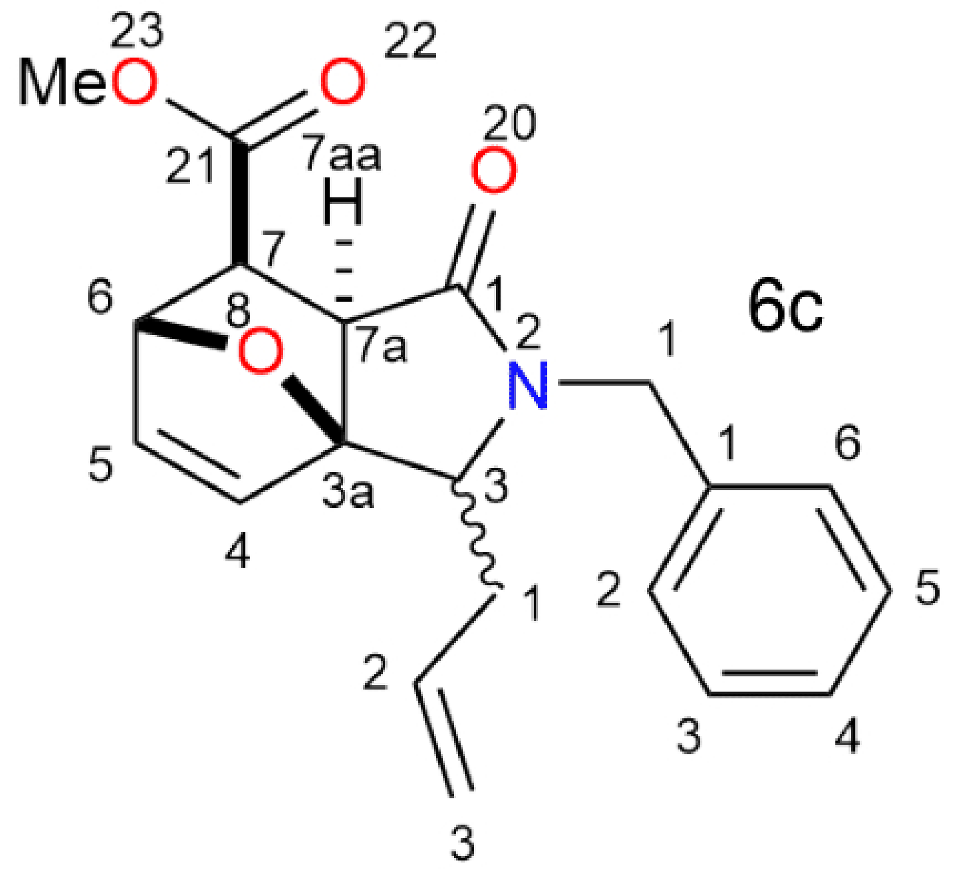

FZ7998-1.JDF

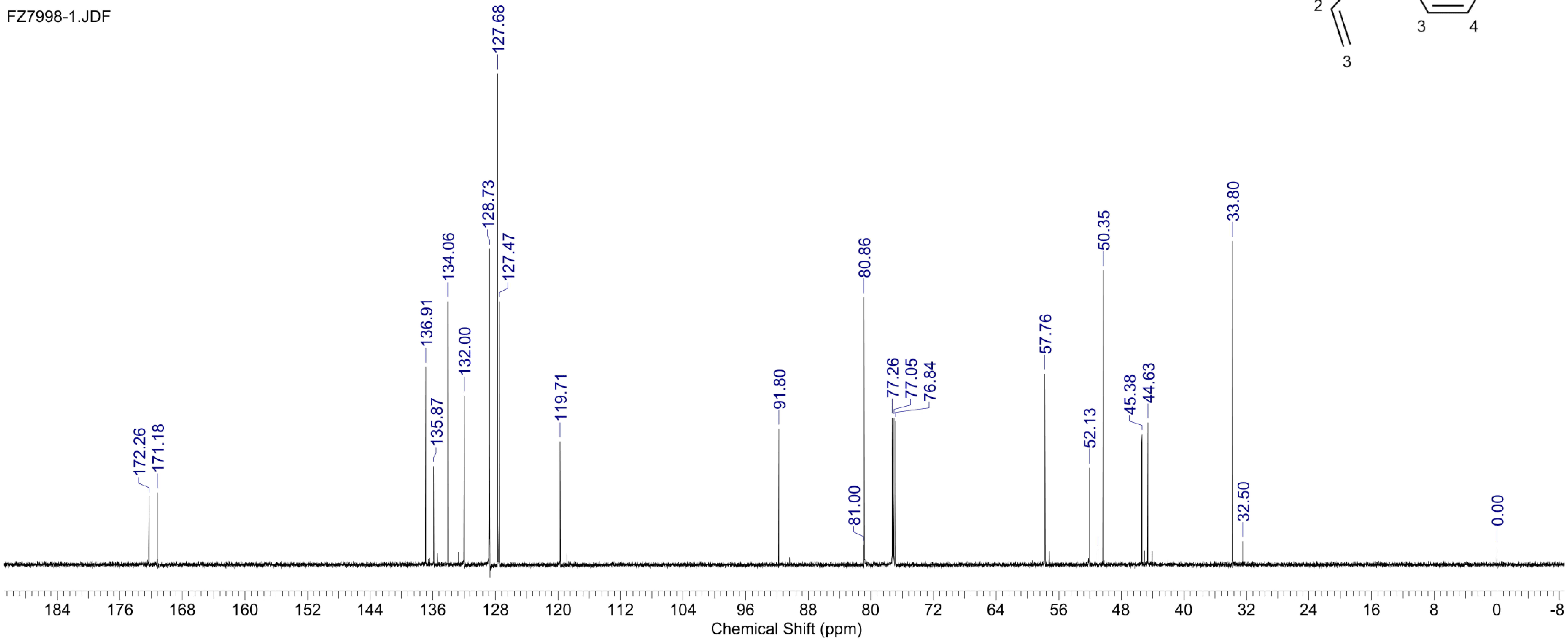

14.07.2020 18:43:22

**Formula** $C_{21}H_{23}NO_4$	**FW** 353.4116

**Acquisition Time (sec)**	0.6921	**Comment**	single pulse decoupled gated NOE	**Date**	12 Aug 1990 06:09:41
**Date Stamp**	22 Oct 2019 12:12:50	**File Name**	C:\USERS\Лаба534\DOWNLOADS\FZ7998-1.JDF		
**Frequency (MHz)**	150.91	**Nucleus**	13C	**Number of Transients**	800	**Origin**	ECA 600
**Original Points Count**	32768	**Owner**	delta	**Points Count**	32768	**Pulse Sequence**	single_pulse_dec
**Receiver Gain**	54.00	**Solvent**	CHLOROFORM-d	**Spectrum Offset (Hz)**	15079.3525		
**Sweep Width (Hz)**	47348.49	**Temperature (degree C)**	24.700				

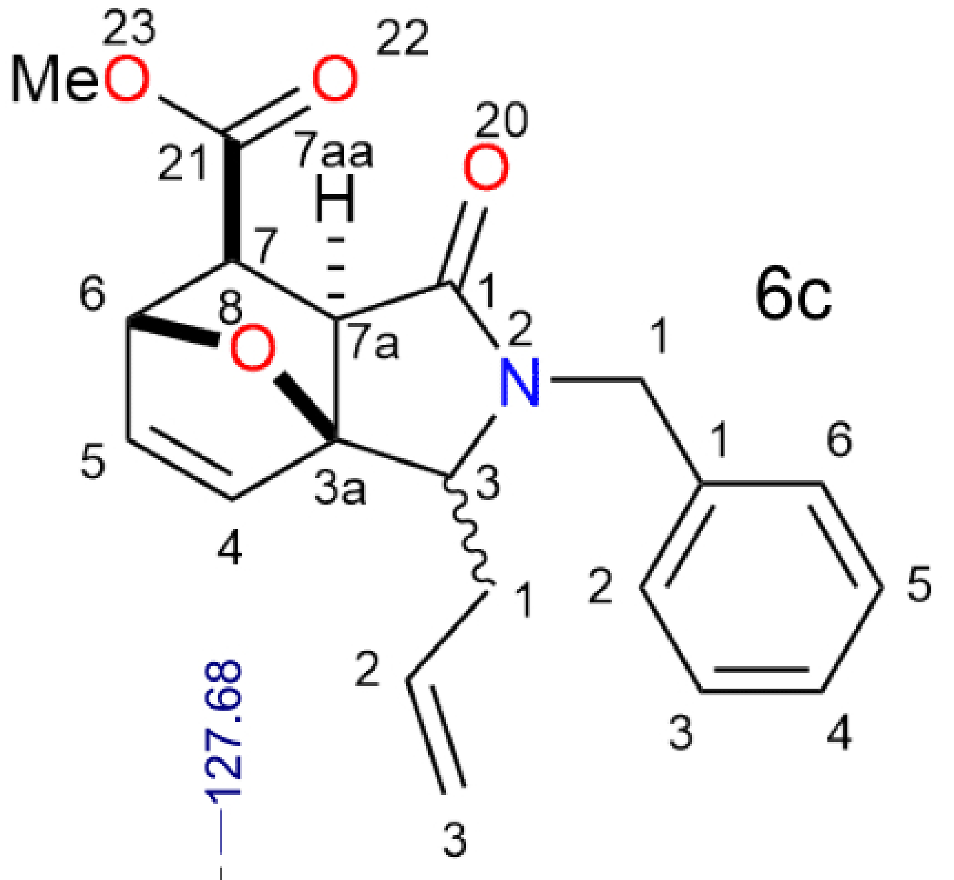

6c

FZ7998-1.JDF

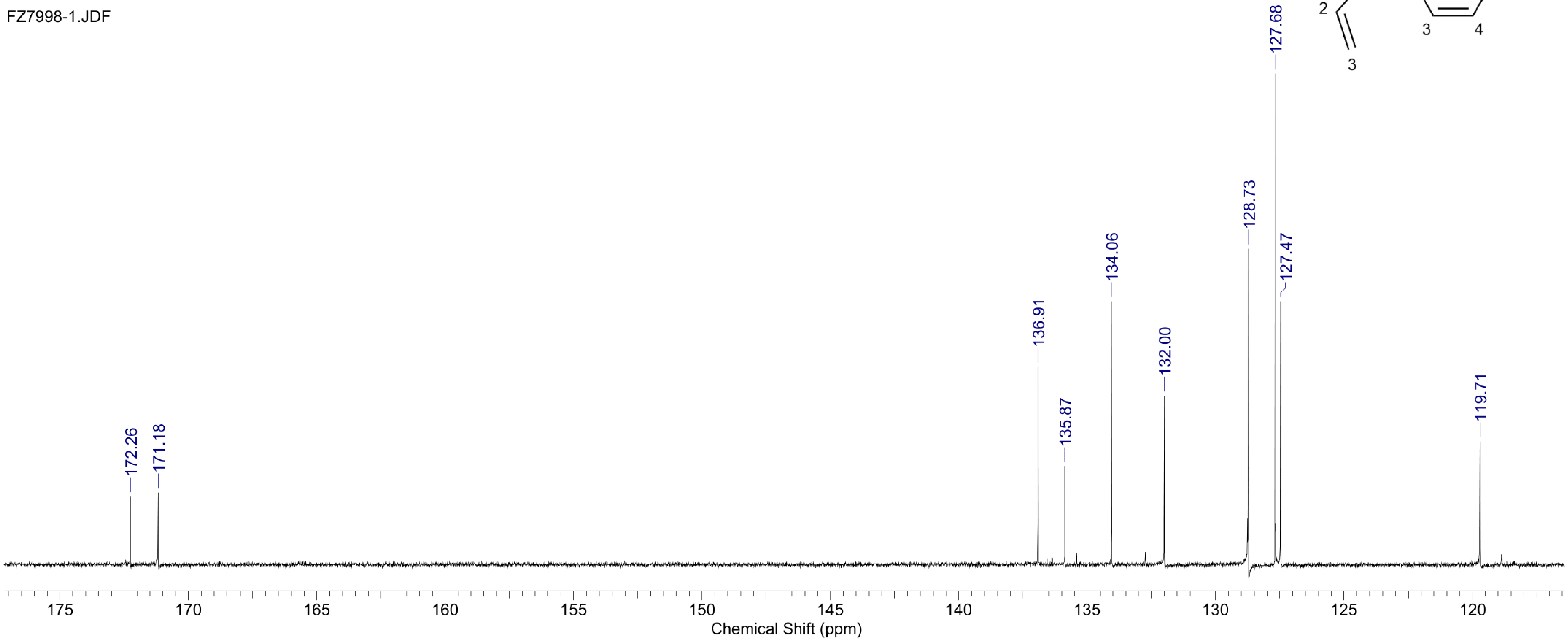

14.07.2020 18:43:30

**Formula** $C_{21}H_{23}NO_4$	**FW** 353.4116

Acquisition Time (sec)	0.6921	***Comment***	single pulse decoupled gated NOE	***Date***	12 Aug 1990 06:09:41
Date Stamp	22 Oct 2019 12:12:50	***File Name***	C:\USERS\Ла6а534\DOWNLOADS\FZ7998-1.JDF		
Frequency (MHz)	150.91	***Nucleus***	13C	***Number of Transients***	800
Origin	ECA 600				
Original Points Count	32768	***Owner***	delta	***Points Count***	32768
Pulse Sequence	single_pulse_dec				
Receiver Gain	54.00	***Solvent***	CHLOROFORM-d	***Spectrum Offset (Hz)***	15079.3525
Sweep Width (Hz)	47348.49	***Temperature (degree C)***	24.700		

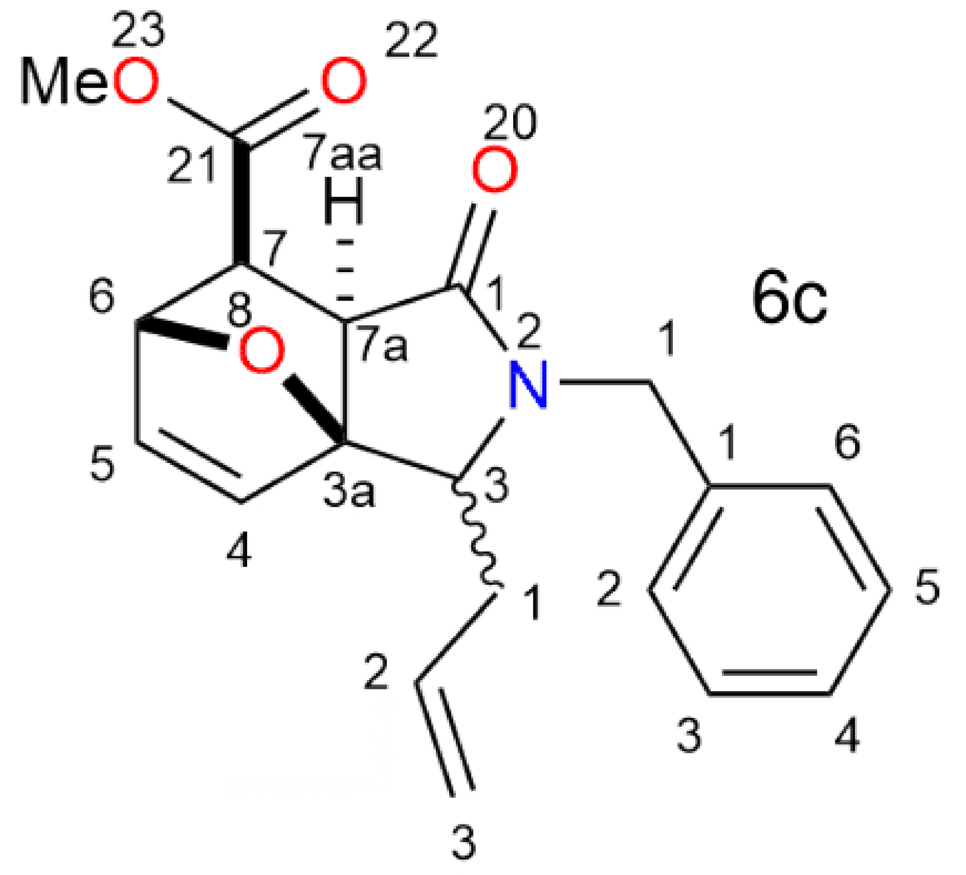

6c

FZ7998-1.JDF

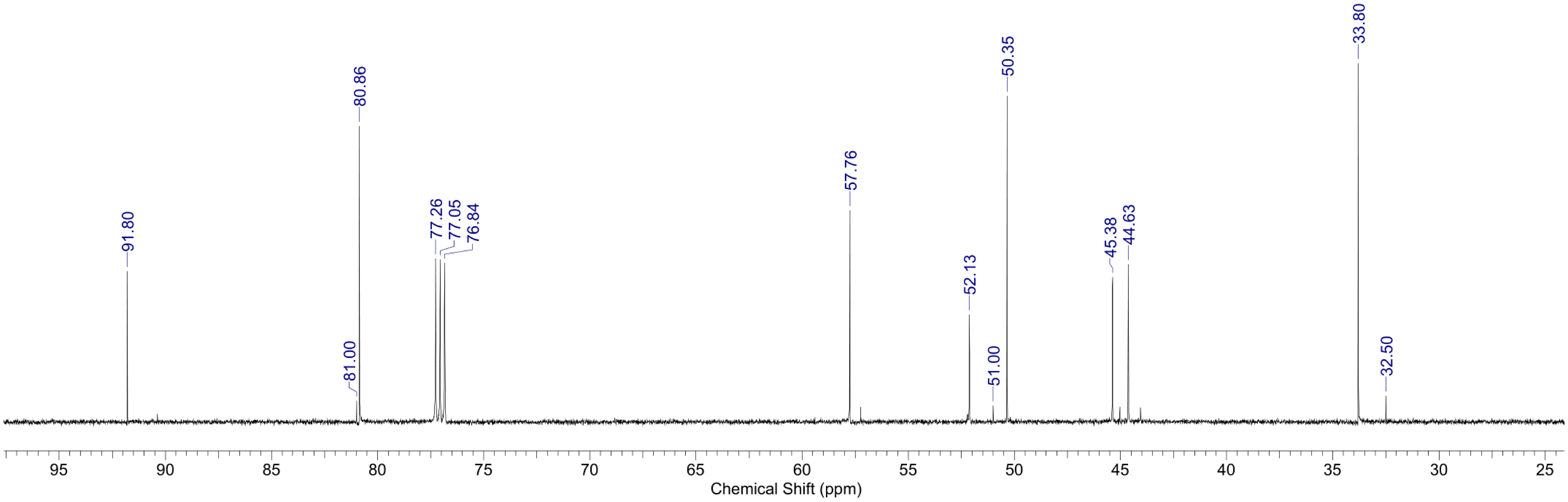

NMR spectrum data for compound 6d

Formula	$C_{19}H_{18}ClNO_4$	***FW***	359.8035

Acquisition Time (sec)	1.9818	***Comment***	single_pulse	***Date***	16 Jan 2020 05:39:20		
Date Stamp	16 Jan 2020 04:58:38			***File Name***	C:\Users\User\Desktop\14.01.20\FZ8341-1.jdf	***Frequency (MHz)***	600.17
Nucleus	1H	***Number of Transients***	8	***Origin***	ECA 600	***Original Points Count*** 32768	***Owner*** delta
Points Count	32768	***Pulse Sequence***	single_pulse.ex2			***Receiver Gain*** 40.00	***Solvent*** CHLOROFORM-d
Spectrum Offset (Hz)	5413.1504	***Sweep Width (Hz)***	16534.39	***Temperature (degree C)***	21.900		

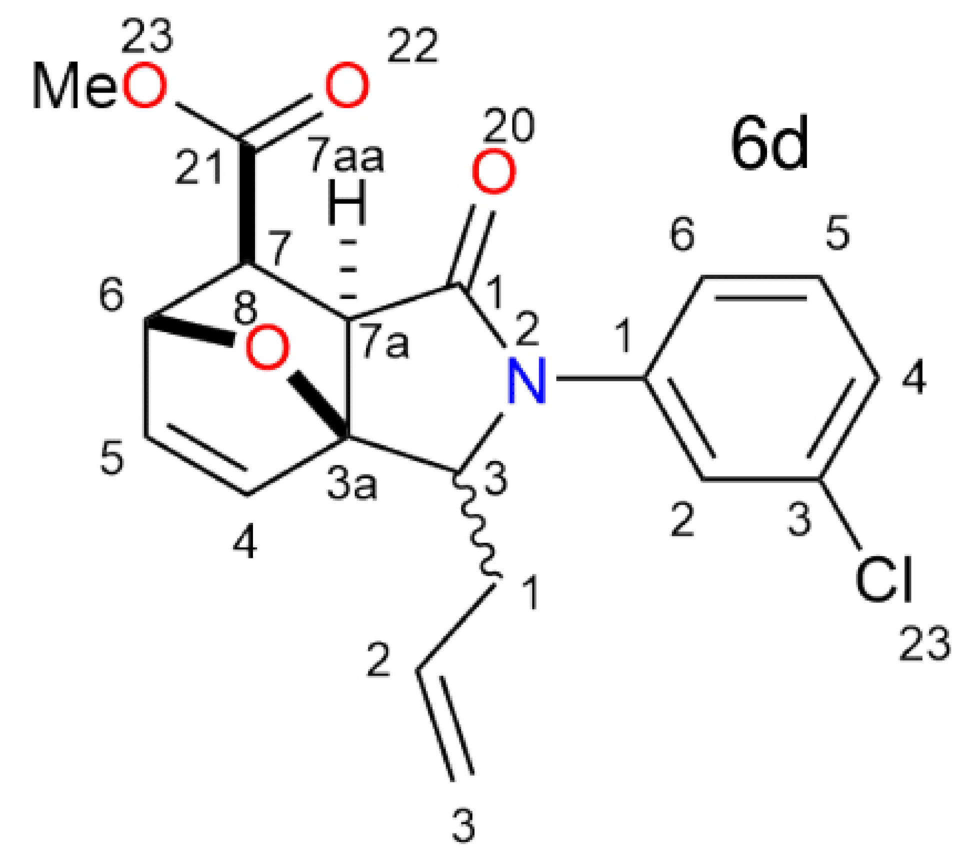

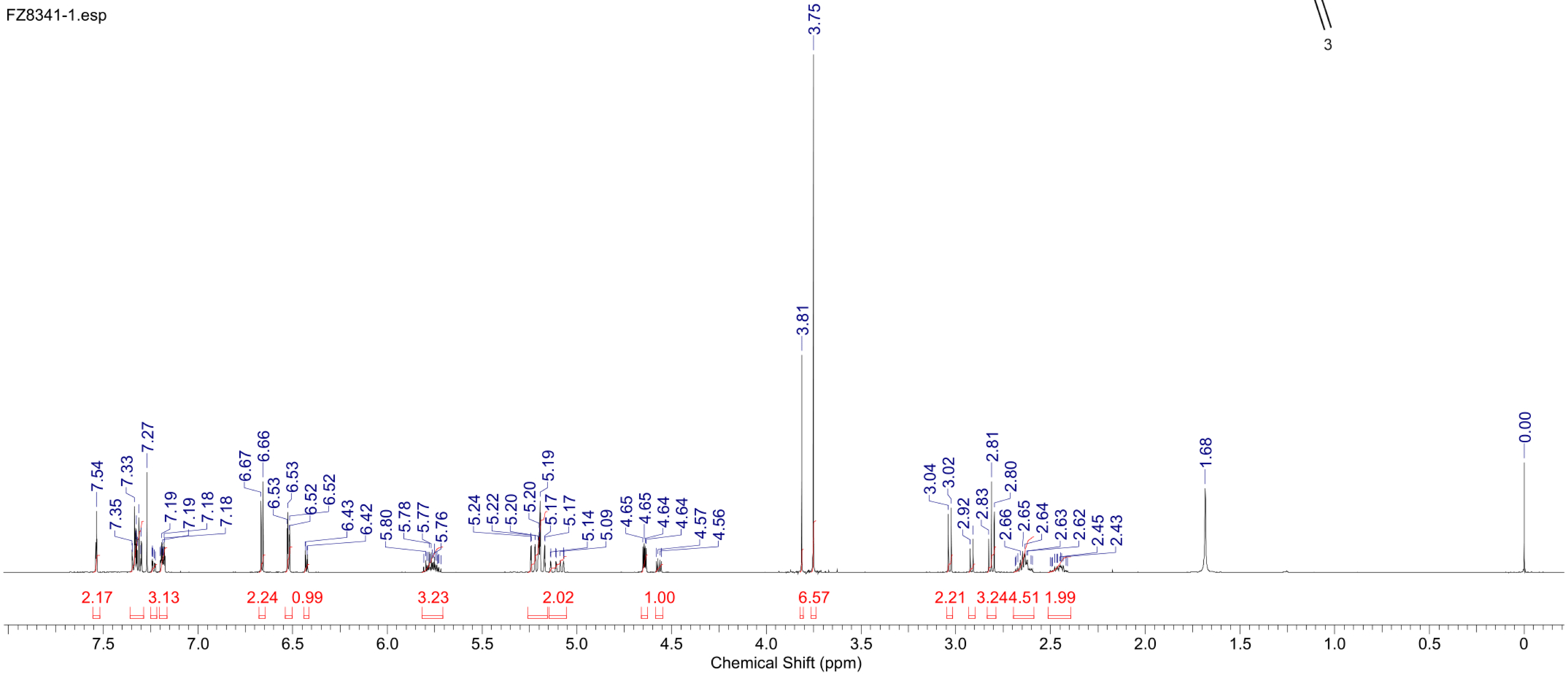

Formula	$C_{19}H_{18}ClNO_4$	FW	359.8035

Acquisition Time (sec)	1.9818	Comment	single_pulse	Date	16 Jan 2020 05:39:20				
Date Stamp	16 Jan 2020 04:58:38			File Name	C:\Users\User\Desktop\14.01.20\FZ8341-1.jdf			Frequency (MHz)	600.17
Nucleus	1H	Number of Transients	8	Origin	ECA 600	Original Points Count	32768	Owner	delta
Points Count	32768	Pulse Sequence	single_pulse.ex2			Receiver Gain	40.00	Solvent	CHLOROFORM-d
Spectrum Offset (Hz)	5413.1504	Sweep Width (Hz)	16534.39	Temperature (degree C)	21.900				

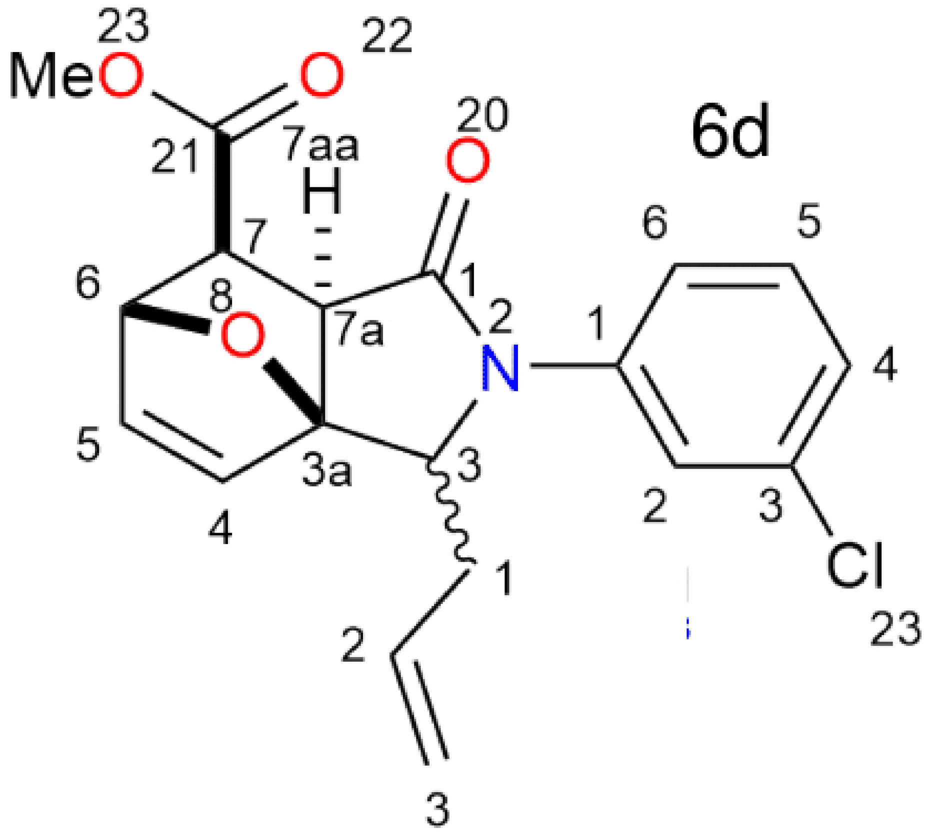

6d

FZ8341-1.esp

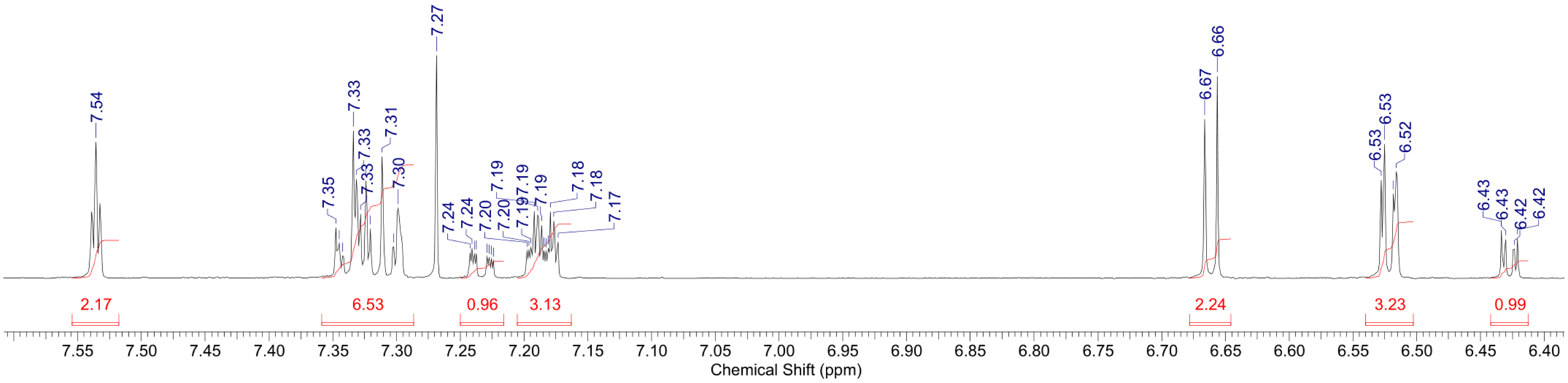

Formula $C_{19}H_{18}ClNO_4$	FW 359.8035

Acquisition Time (sec)	1.9818	Comment	single_pulse	Date	16 Jan 2020 05:39:20				
Date Stamp	16 Jan 2020 04:58:38			File Name	C:\Users\User\Desktop\14.01.20\FZ8341-1.jdf			Frequency (MHz)	600.17
Nucleus	1H	Number of Transients	8	Origin	ECA 600	Original Points Count	32768	Owner	delta
Points Count	32768	Pulse Sequence	single_pulse.ex2			Receiver Gain	40.00	Solvent	CHLOROFORM-d
Spectrum Offset (Hz)	5413.1504	Sweep Width (Hz)	16534.39	Temperature (degree C)	21.900				

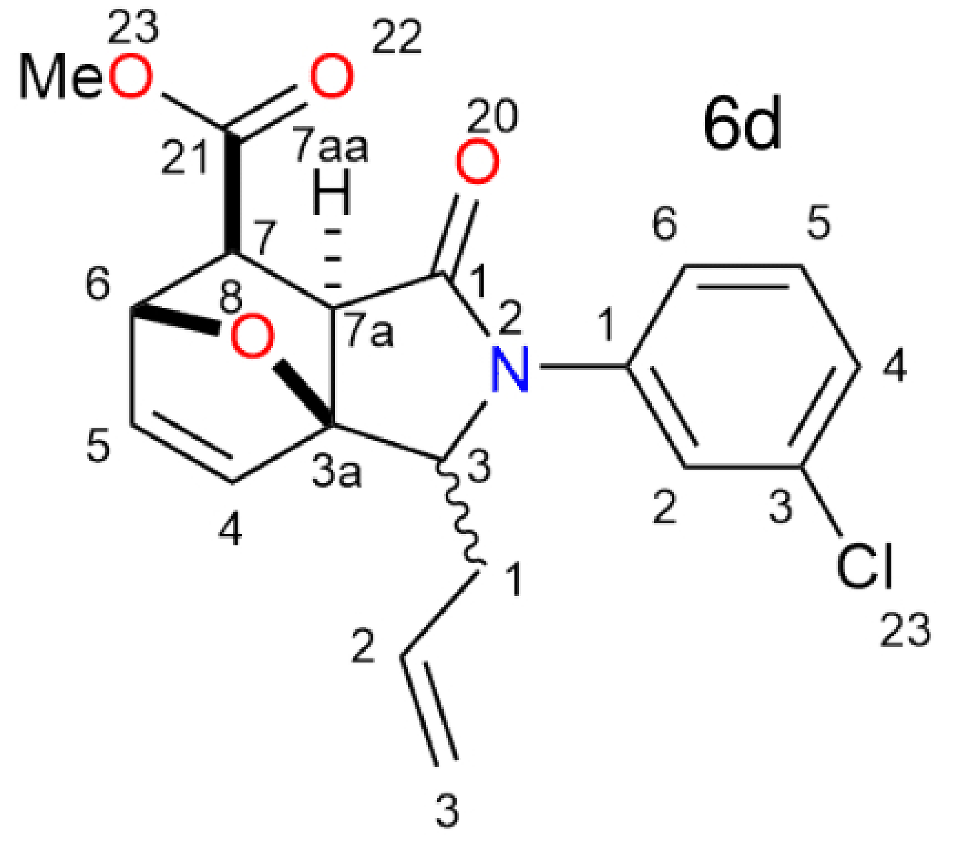

6d

FZ8341-1.esp

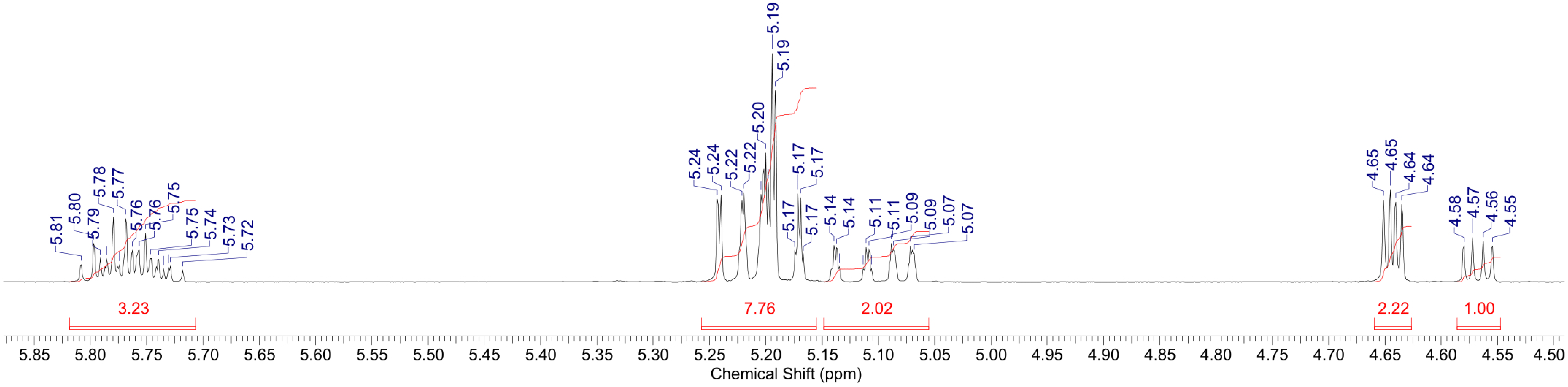

Formula	$C_{19}H_{18}ClNO_4$	FW	359.8035

Acquisition Time (sec)	1.9818	Comment	single_pulse	Date	16 Jan 2020 05:39:20				
Date Stamp	16 Jan 2020 04:58:38			File Name	C:\Users\User\Desktop\14.01.20\FZ8341-1.jdf			Frequency (MHz)	600.17
Nucleus	1H	Number of Transients	8	Origin	ECA 600	Original Points Count	32768	Owner	delta
Points Count	32768	Pulse Sequence	single_pulse.ex2			Receiver Gain	40.00	Solvent	CHLOROFORM-d
Spectrum Offset (Hz)	5413.1504	Sweep Width (Hz)	16534.39	Temperature (degree C)	21.900				

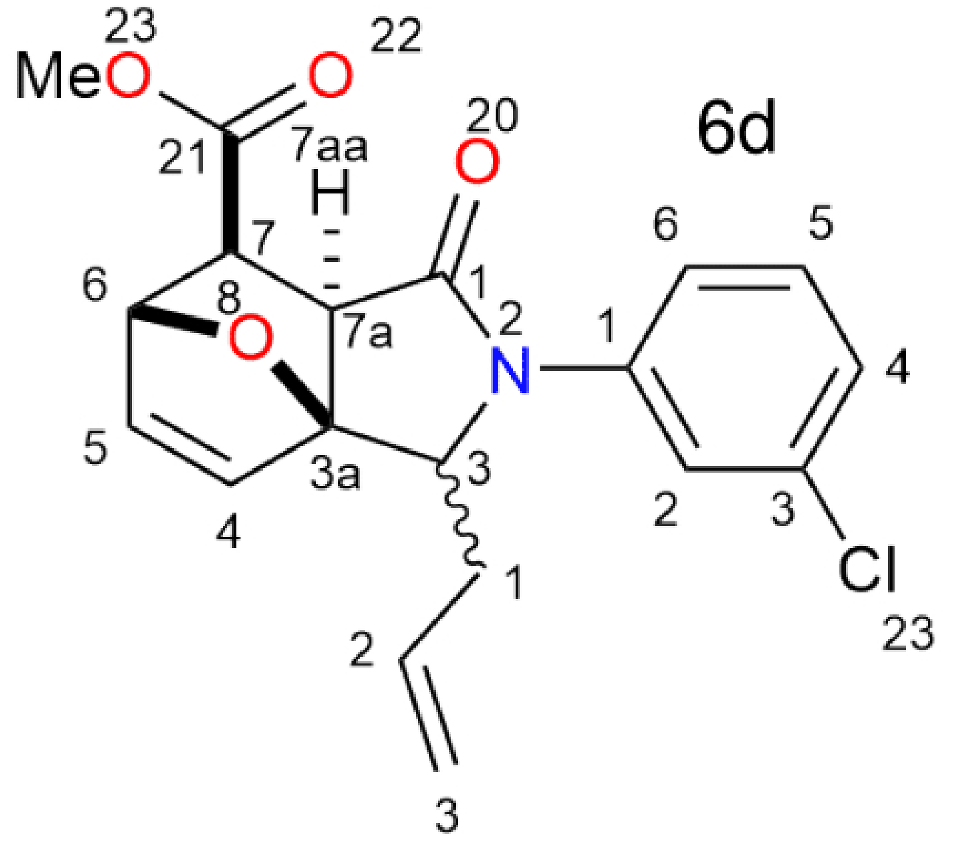

6d

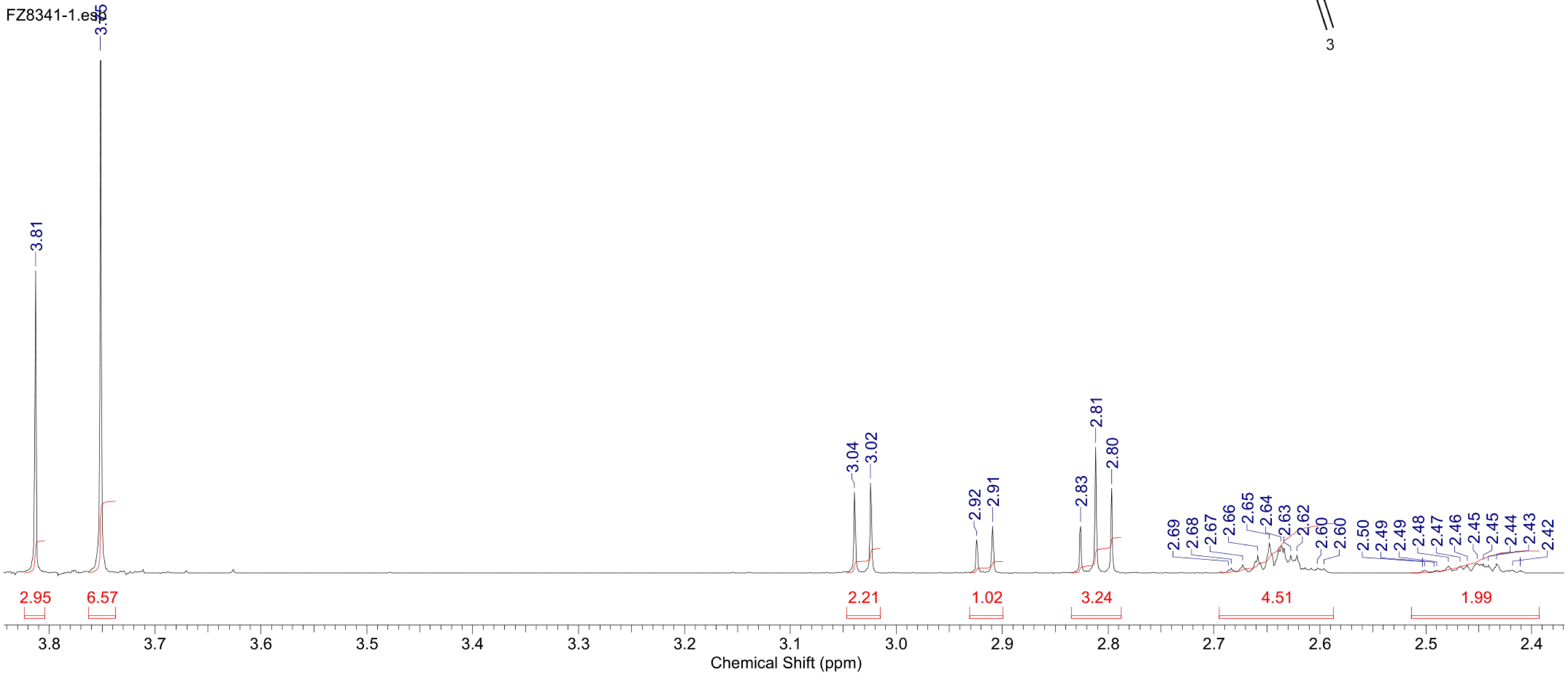

Formula $C_{19}H_{18}ClNO_4$	FW 359.8035

**Acquisition Time (sec)**	0.6921	**Comment**	single pulse decoupled gated NOE	**Date**	16 Jan 2020 06:01:08
**Date Stamp**	16 Jan 2020 05:20:26	**File Name**	C:\Users\User\Desktop\14.01.20\FZ8341-3.jdf		
**Frequency (MHz)**	150.91	**Nucleus**	13C	**Number of Transients**	1000
**Origin**	ECA 600				
**Original Points Count**	32768	**Owner**	delta	**Points Count**	32768
**Pulse Sequence**	single_pulse_dec				
**Receiver Gain**	54.00	**Solvent**	CHLOROFORM-d	**Spectrum Offset (Hz)**	15079.3525
**Sweep Width (Hz)**	47348.49	**Temperature (degree C)**	23.100		

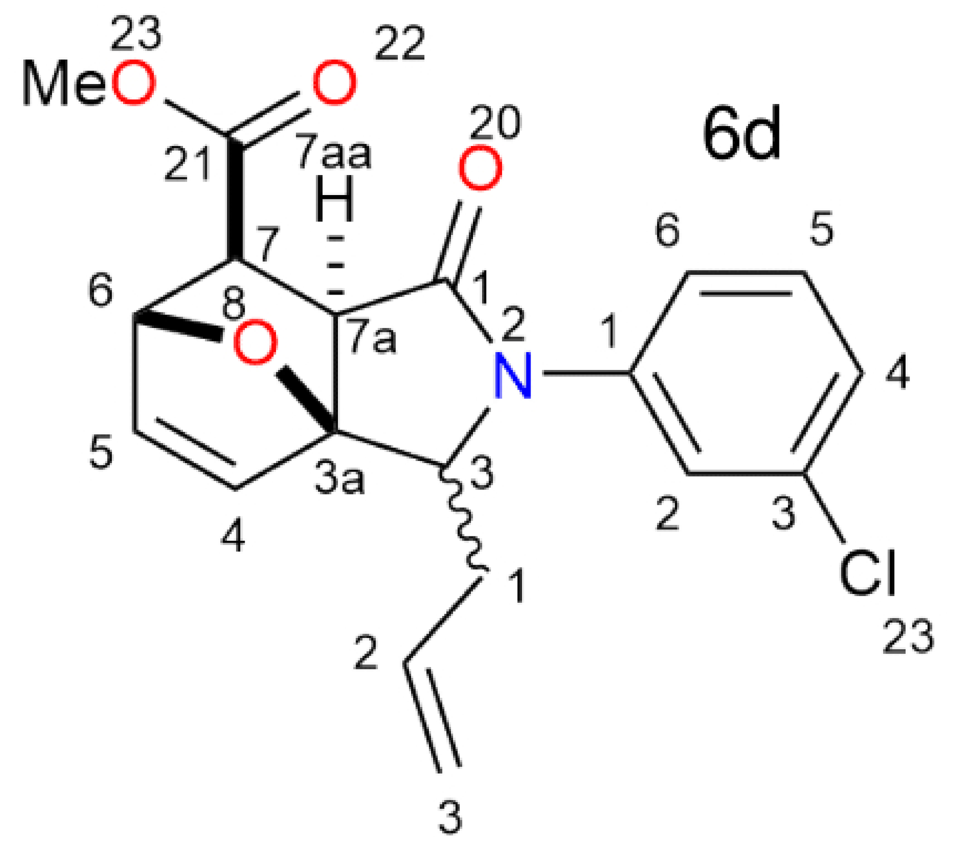

FZ8341-3.esp

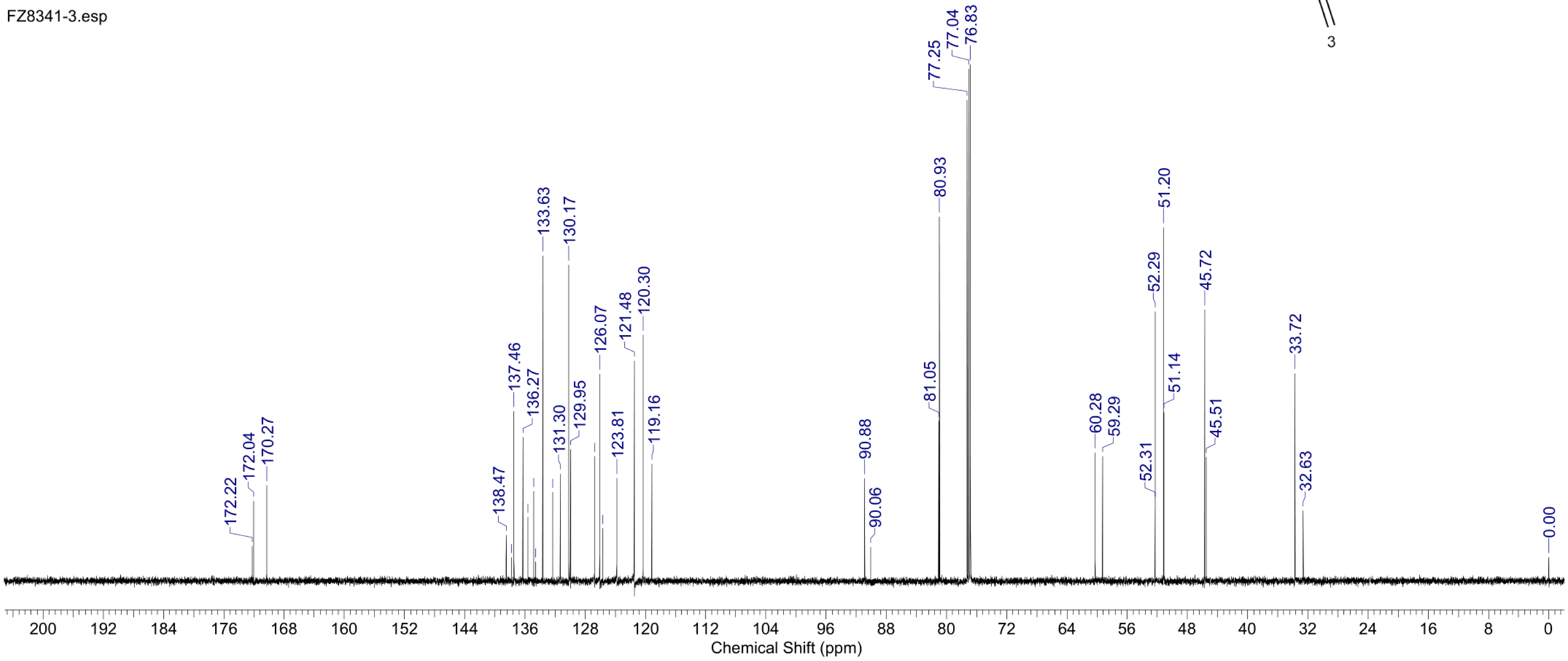

Formula $C_{19}H_{18}ClNO_4$	FW 359.8035

**Acquisition Time (sec)**	0.6921	**Comment**	single pulse decoupled gated NOE	**Date**	16 Jan 2020 06:01:08
**Date Stamp**	16 Jan 2020 05:20:26	**File Name**	C:\Users\User\Desktop\14.01.20\FZ8341-3.jdf		
**Frequency (MHz)**	150.91	**Nucleus**	13C	**Number of Transients**	1000
**Origin**	ECA 600				
**Original Points Count**	32768	**Owner**	delta	**Points Count**	32768
**Pulse Sequence**	single_pulse_dec				
**Receiver Gain**	54.00	**Solvent**	CHLOROFORM-d	**Spectrum Offset (Hz)**	15079.3525
**Sweep Width (Hz)**	47348.49	**Temperature (degree C)**	23.100		

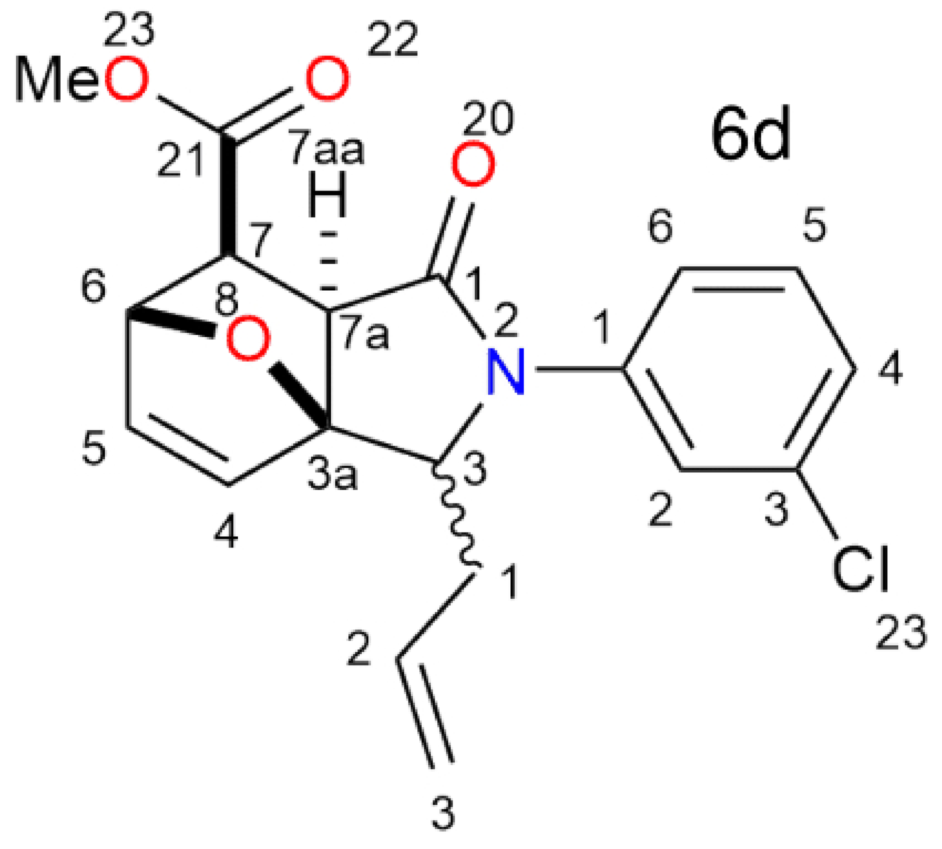

FZ8341-3.esp

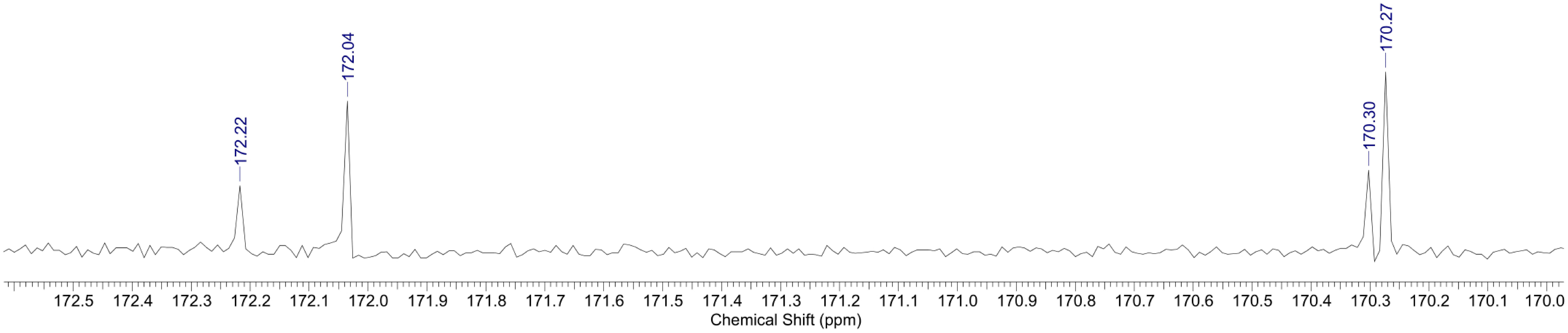

Formula	$C_{19}H_{18}ClNO_4$	FW	359.8035

Acquisition Time (sec)	0.6921	Comment	single pulse decoupled gated NOE			Date	16 Jan 2020 06:01:08
Date Stamp	16 Jan 2020 05:20:26			File Name	C:\Users\User\Desktop\14.01.20\FZ8341-3.jdf		
Frequency (MHz)	150.91	Nucleus	13C	Number of Transients	1000	Origin	ECA 600
Original Points Count	32768	Owner	delta	Points Count	32768	Pulse Sequence	single_pulse_dec
Receiver Gain	54.00	Solvent	CHLOROFORM-d			Spectrum Offset (Hz)	15079.3525
Sweep Width (Hz)	47348.49	Temperature (degree C)	23.100				

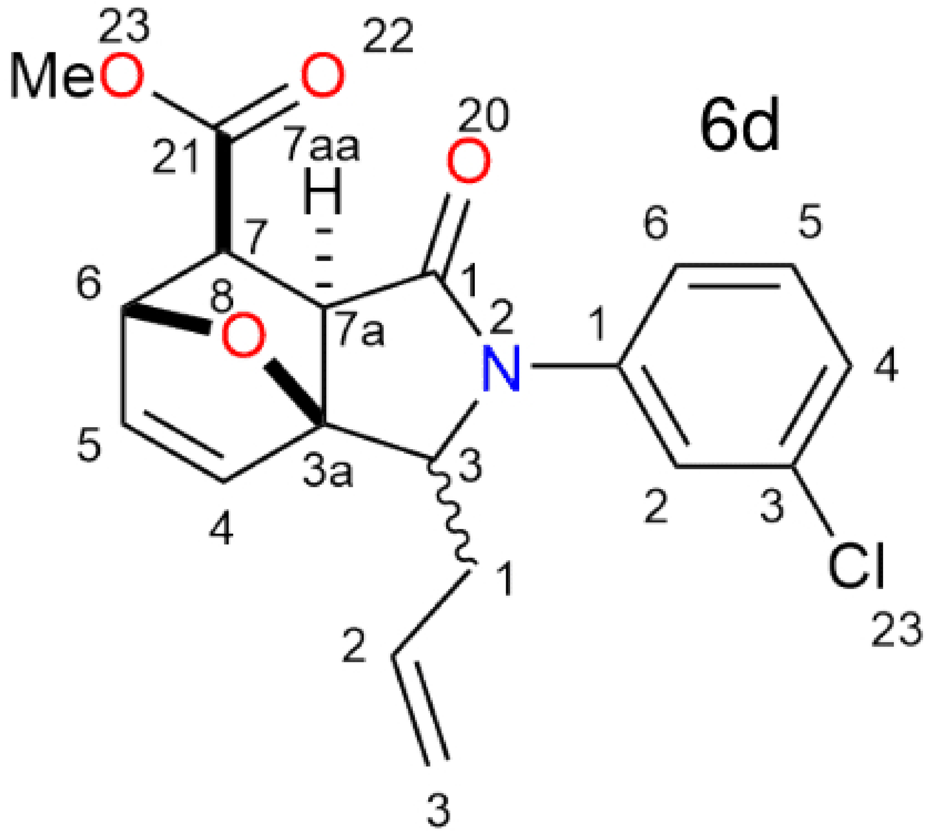

6d

FZ8341-3.esp

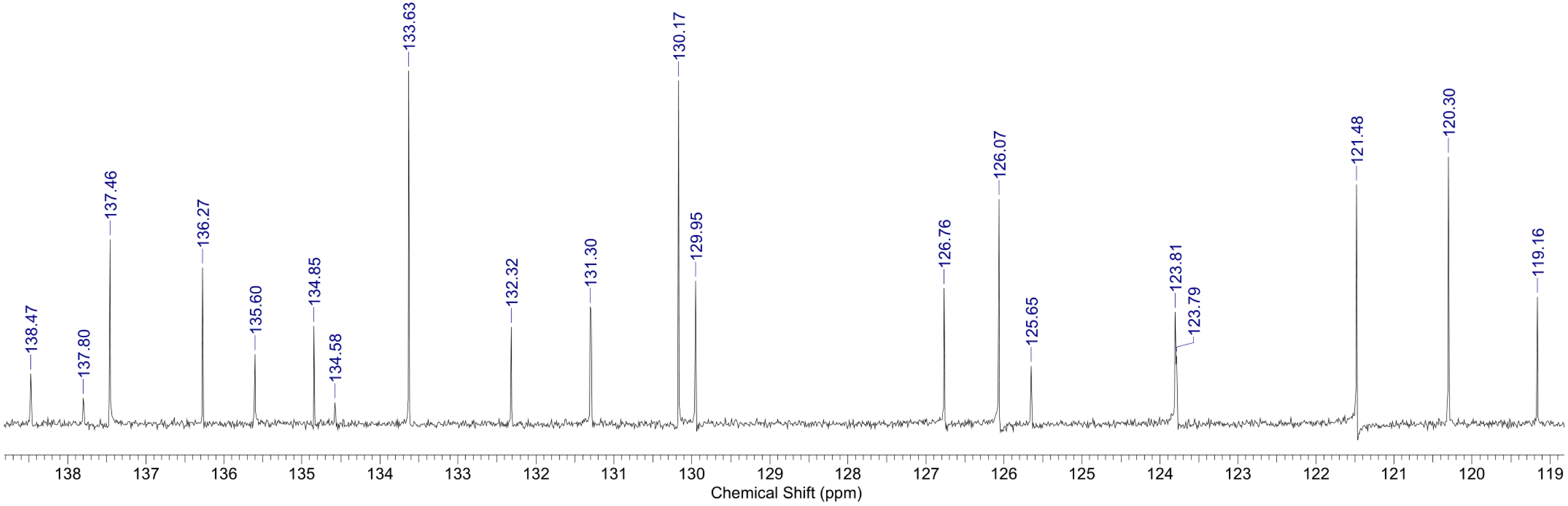

Formula $C_{19}H_{18}ClNO_4$	FW	359.8035

Acquisition Time (sec)	0.6921	Comment	single pulse decoupled gated NOE			Date	16 Jan 2020 06:01:08
Date Stamp	16 Jan 2020 05:20:26			File Name	C:\Users\User\Desktop\14.01.20\FZ8341-3.jdf		
Frequency (MHz)	150.91	Nucleus	13C	Number of Transients	1000	Origin	ECA 600
Original Points Count	32768	Owner	delta	Points Count	32768	Pulse Sequence	single_pulse_dec
Receiver Gain	54.00	Solvent	CHLOROFORM-d			Spectrum Offset (Hz)	15079.3525
Sweep Width (Hz)	47348.49	Temperature (degree C)	23.100				

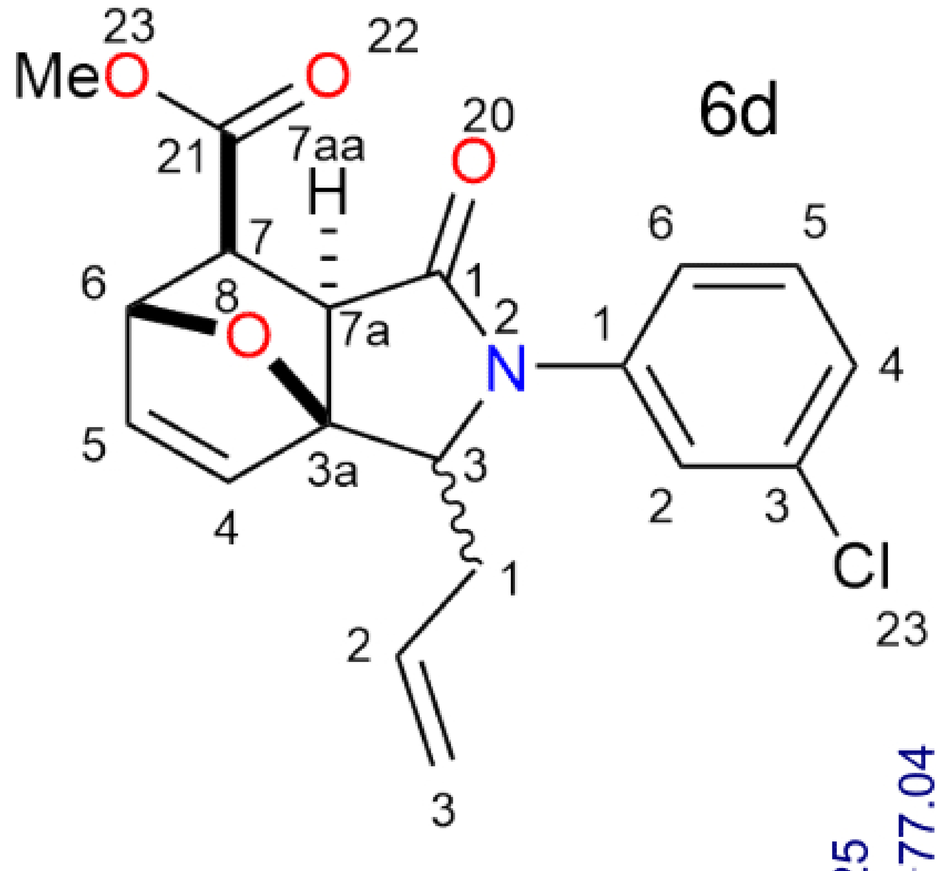

FZ8341-3.esp

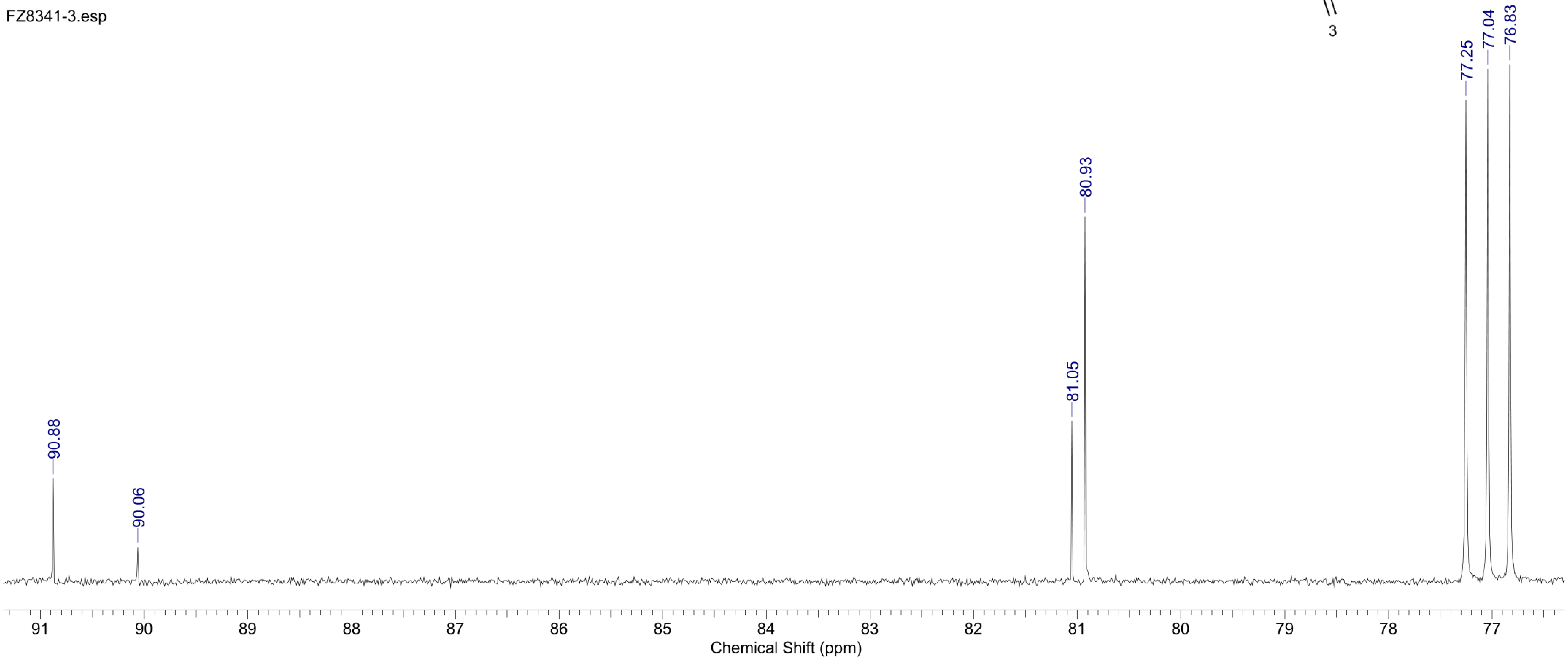

Formula	$C_{19}H_{18}ClNO_4$	FW	359.8035

**Acquisition Time (sec)**	0.6921	**Comment**	single pulse decoupled gated NOE			**Date**	16 Jan 2020 06:01:08
**Date Stamp**	16 Jan 2020 05:20:26			**File Name**	C:\Users\User\Desktop\14.01.20\FZ8341-3.jdf		
**Frequency (MHz)**	150.91	**Nucleus**	13C	**Number of Transients**	1000	**Origin**	ECA 600
**Original Points Count**	32768	**Owner**	delta	**Points Count**	32768	**Pulse Sequence**	single_pulse_dec
**Receiver Gain**	54.00	**Solvent**	CHLOROFORM-d			**Spectrum Offset (Hz)**	15079.3525
**Sweep Width (Hz)**	47348.49	**Temperature (degree C)**	23.100				

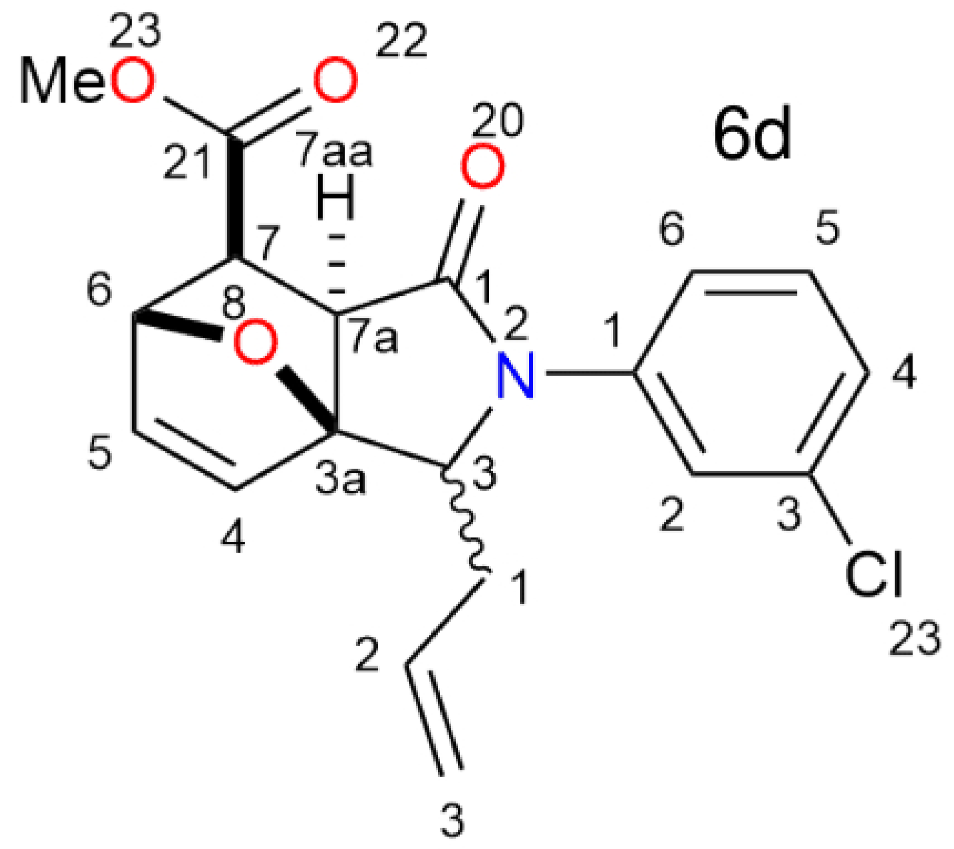

FZ8341-3.esp

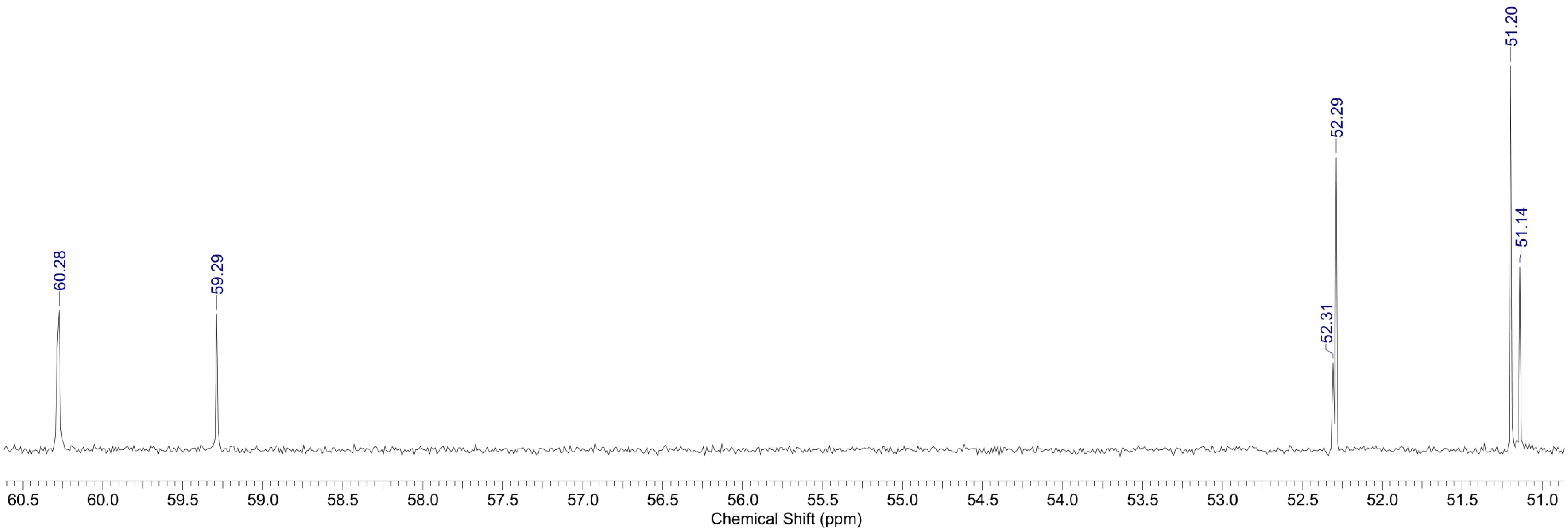

Formula	$C_{19}H_{18}ClNO_4$	FW	359.8035

**Acquisition Time (sec)**	0.6921	**Comment**	single pulse decoupled gated NOE	**Date**	16 Jan 2020 06:01:08
**Date Stamp**	16 Jan 2020 05:20:26	**File Name**	C:\Users\User\Desktop\14.01.20\FZ8341-3.jdf		
**Frequency (MHz)**	150.91	**Nucleus**	13C	**Number of Transients**	1000
**Origin**	ECA 600	**Original Points Count**	32768	**Owner**	delta
**Points Count**	32768	**Pulse Sequence**	single_pulse_dec	**Receiver Gain**	54.00
**Solvent**	CHLOROFORM-d	**Spectrum Offset (Hz)**	15079.3525	**Sweep Width (Hz)**	47348.49
**Temperature (degree C)**	23.100				

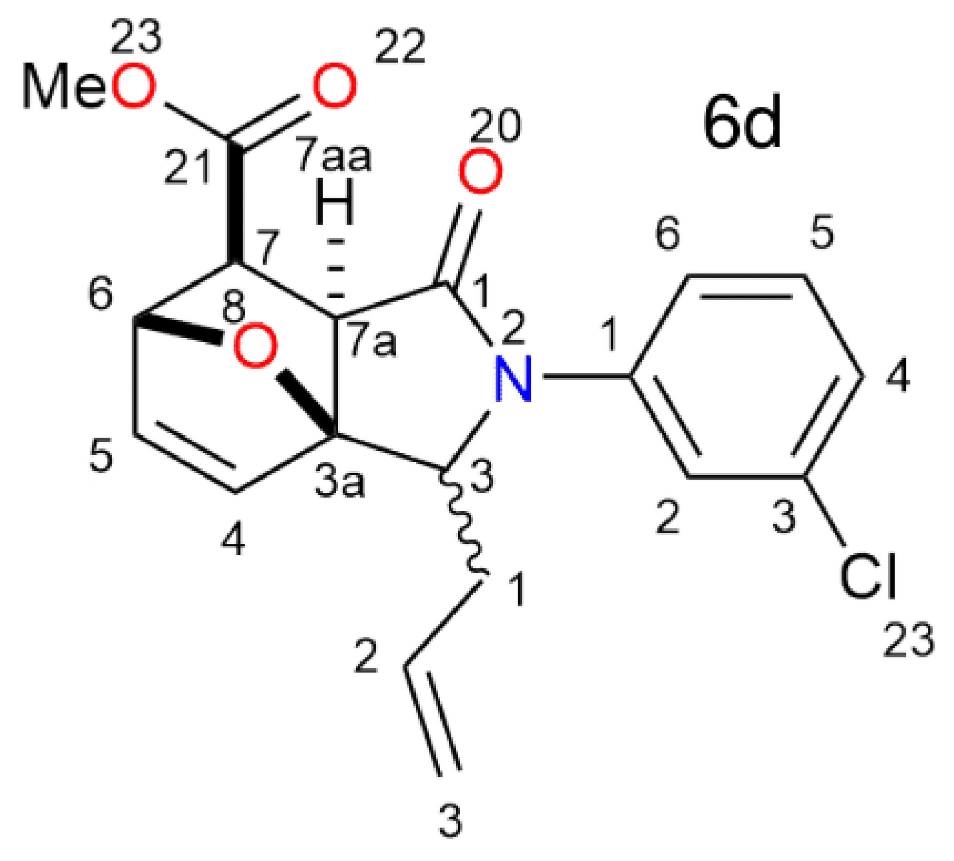

6d

FZ8341-3.esp

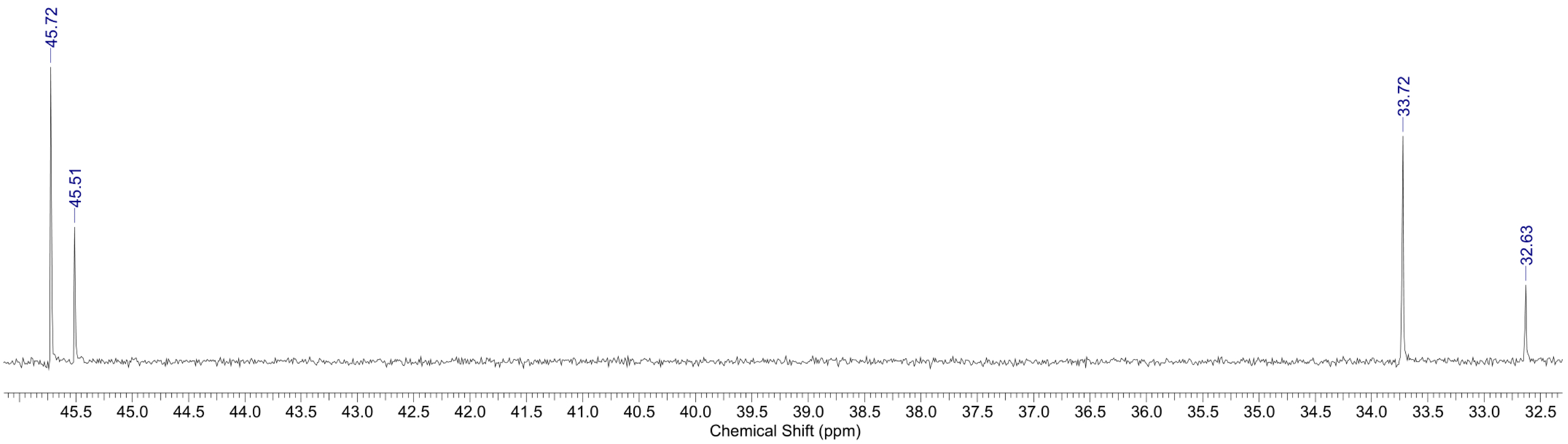

NMR spectrum data for compound 6e

Formula	$C_{18}H_{16}ClNO_4$	FW	345.7769

**Acquisition Time (sec)**	1.9818	**Comment**	single_pulse	**Date**	14 Jul 2020 13:24:21		
**Date Stamp**	14 Jul 2020 13:26:04			**File Name**	C:\USERS\па6а 534\DOWNLOADS\FZ8792-1.JDF		
**Frequency (MHz)**	600.17	**Nucleus**	1H	**Number of Transients**	8	**Origin**	ECA 600
**Original Points Count**	32768	**Owner**	CKP	**Points Count**	32768	**Pulse Sequence**	single_pulse.ex2
**Receiver Gain**	40.00	**Solvent**	CHLOROFORM-d			**Spectrum Offset (Hz)**	5409.1133
**Sweep Width (Hz)**	16534.39	**Temperature (degree C)**	39.000				

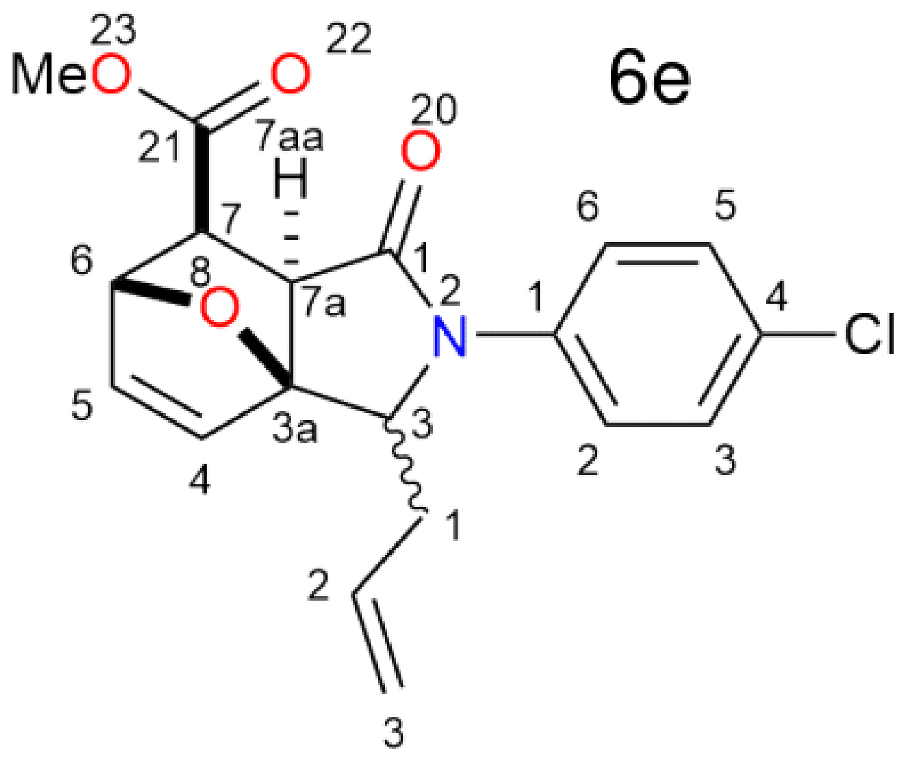

6e

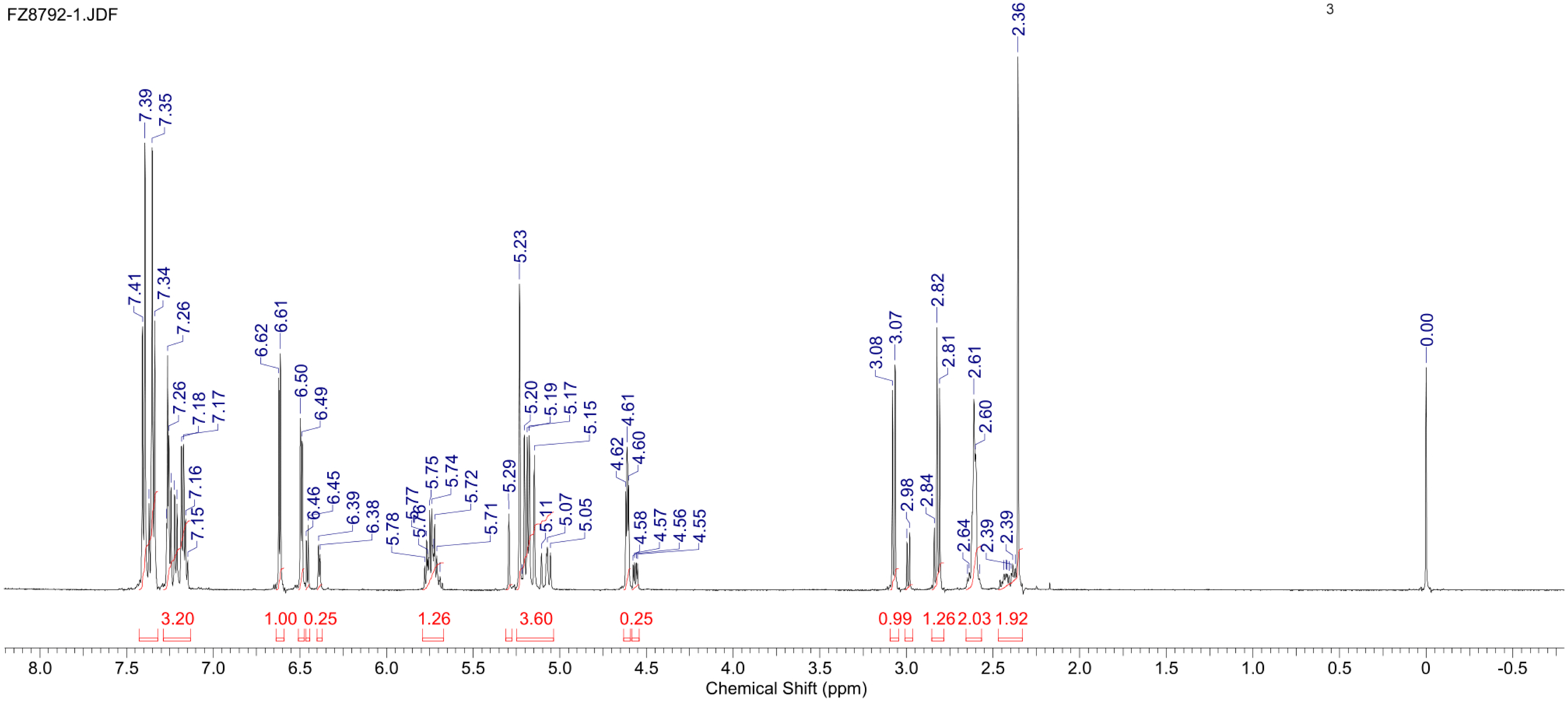

Formula	$C_{18}H_{16}ClNO_4$	FW	345.7769

**Acquisition Time (sec)**	1.9818	**Comment**	single_pulse	**Date**	14 Jul 2020 13:24:21		
**Date Stamp**	14 Jul 2020 13:26:04			**File Name**	C:\USERS\па6а 534\DOWNLOADS\FZ8792-1.JDF		
**Frequency (MHz)**	600.17	**Nucleus**	1H	**Number of Transients**	8	**Origin**	ECA 600
**Original Points Count**	32768	**Owner**	CKP	**Points Count**	32768	**Pulse Sequence**	single_pulse.ex2
**Receiver Gain**	40.00	**Solvent**	CHLOROFORM-d			**Spectrum Offset (Hz)**	5409.1133
**Sweep Width (Hz)**	16534.39	**Temperature (degree C)**	39.000				

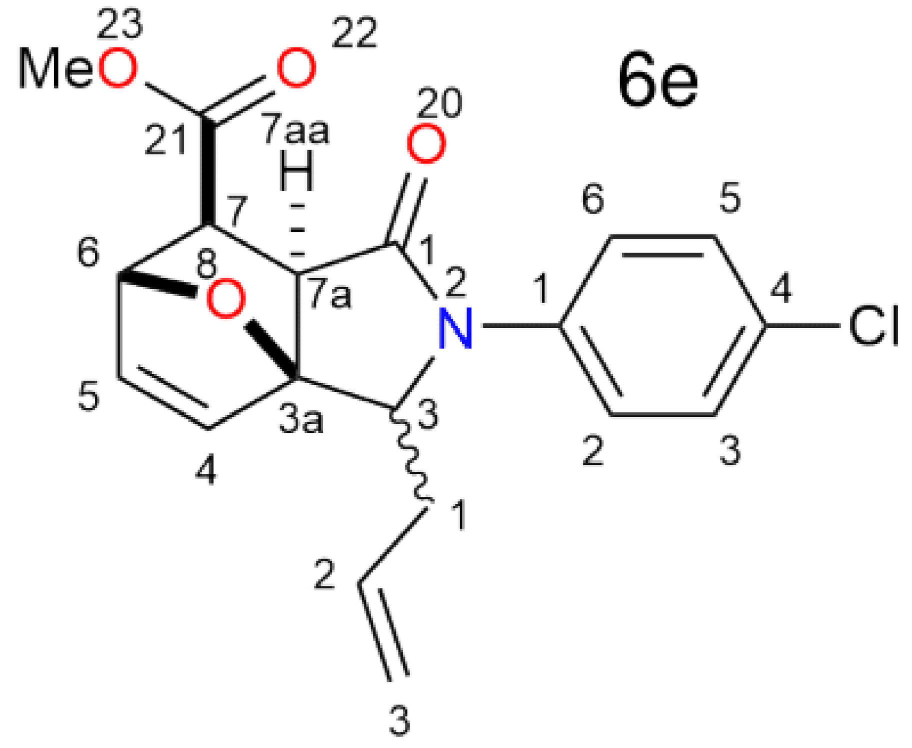

6e

FZ8792-1.JDF

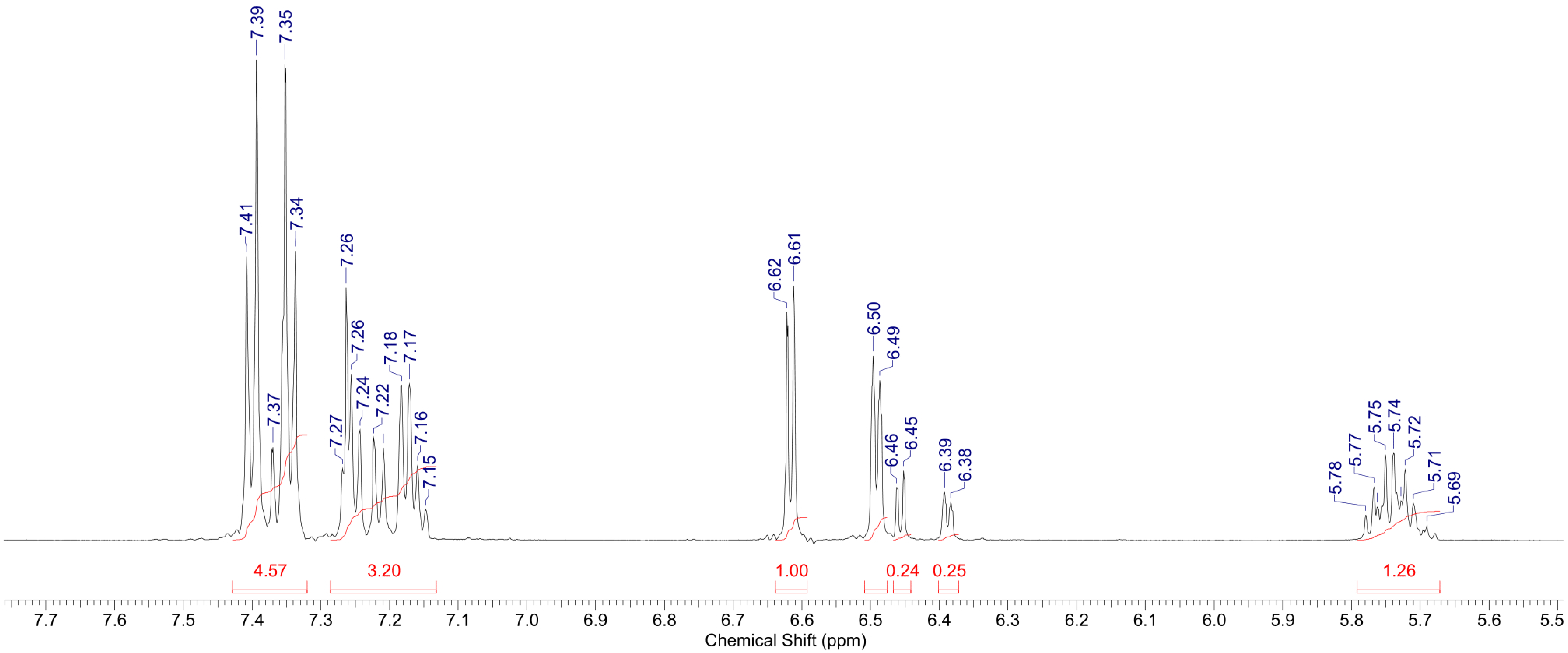

**Formula**	$C_{18}H_{16}ClNO_4$	**FW**	345.7769

**Acquisition Time (sec)**	1.9818	**Comment**	single_pulse	**Date**	14 Jul 2020 13:24:21		
**Date Stamp**	14 Jul 2020 13:26:04			**File Name**	C:\USERS\лаба 534\DOWNLOADS\FZ8792-1.JDF		
**Frequency (MHz)**	600.17	**Nucleus**	1H	**Number of Transients**	8	**Origin**	ECA 600
**Original Points Count**	32768	**Owner**	CKP	**Points Count**	32768	**Pulse Sequence**	single_pulse.ex2
**Receiver Gain**	40.00	**Solvent**	CHLOROFORM-d			**Spectrum Offset (Hz)**	5409.1133
**Sweep Width (Hz)**	16534.39	**Temperature (degree C)**	39.000				

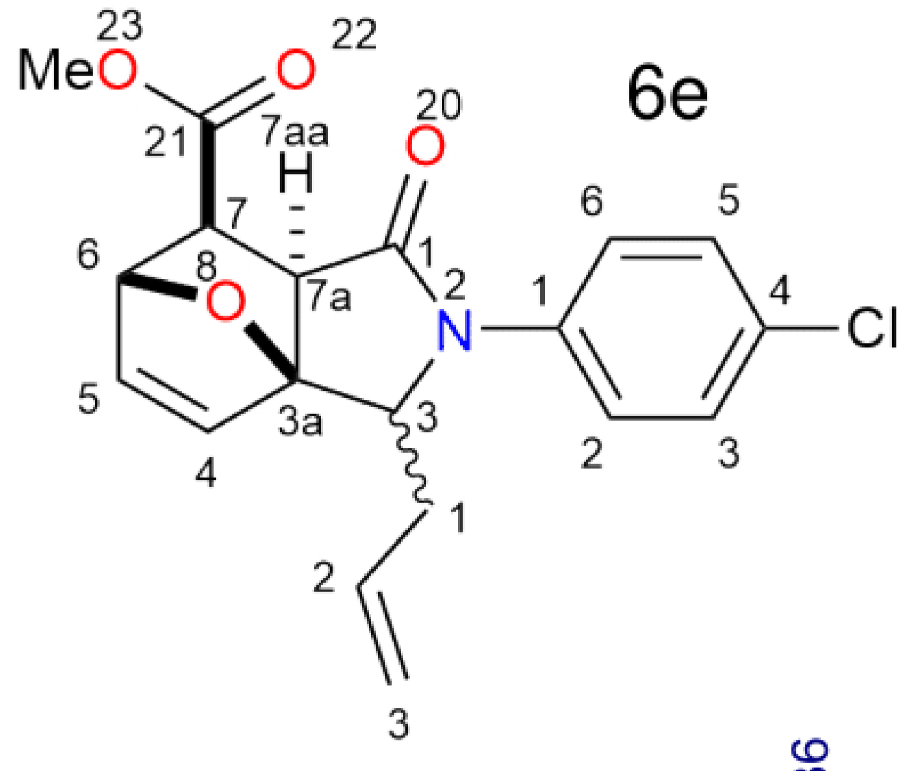

FZ8792-1.JDF

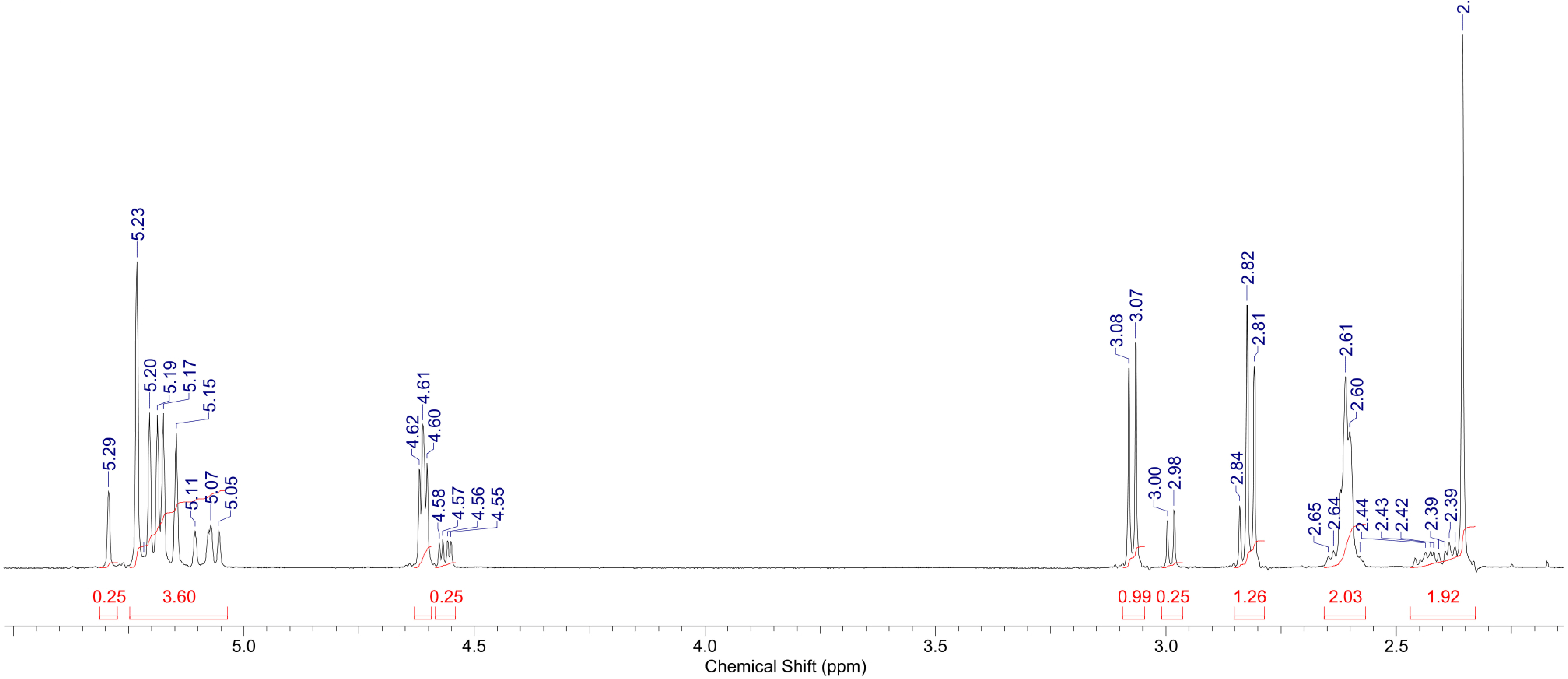

14.07.2020 18:37:45

Formula $C_{20}H_{20}ClNO_4$	FW 373.8301

**Acquisition Time (sec)**	0.6921	**Comment**	single pulse decoupled gated NOE			**Date**	12 Aug 1990 06:31:00
**Date Stamp**	22 Oct 2019 12:34:08			**File Name**	C:\USERS\Лаба534\DOWNLOADS\FZ7999-1.JDF		
**Frequency (MHz)**	150.91	**Nucleus**	13C	**Number of Transients**	800	**Origin**	ECA 600
**Original Points Count**	32768	**Owner**	delta	**Points Count**	32768	**Pulse Sequence**	single_pulse_dec
**Receiver Gain**	54.00	**Solvent**	CHLOROFORM-d			**Spectrum Offset (Hz)**	15079.3525
**Sweep Width (Hz)**	47348.49	**Temperature (degree C)**	24.500				

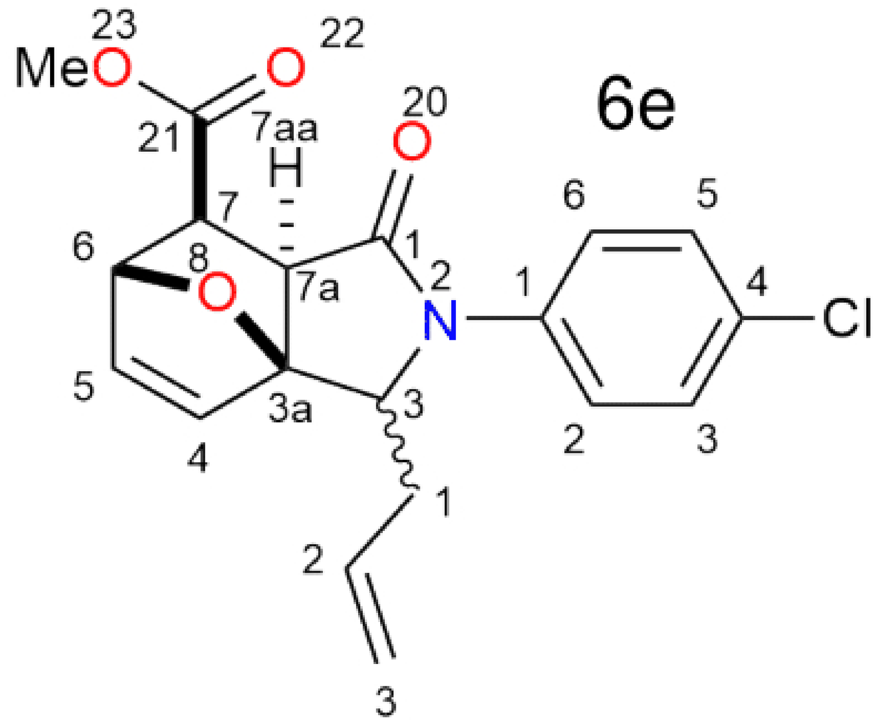

FZ7999-1.JDF

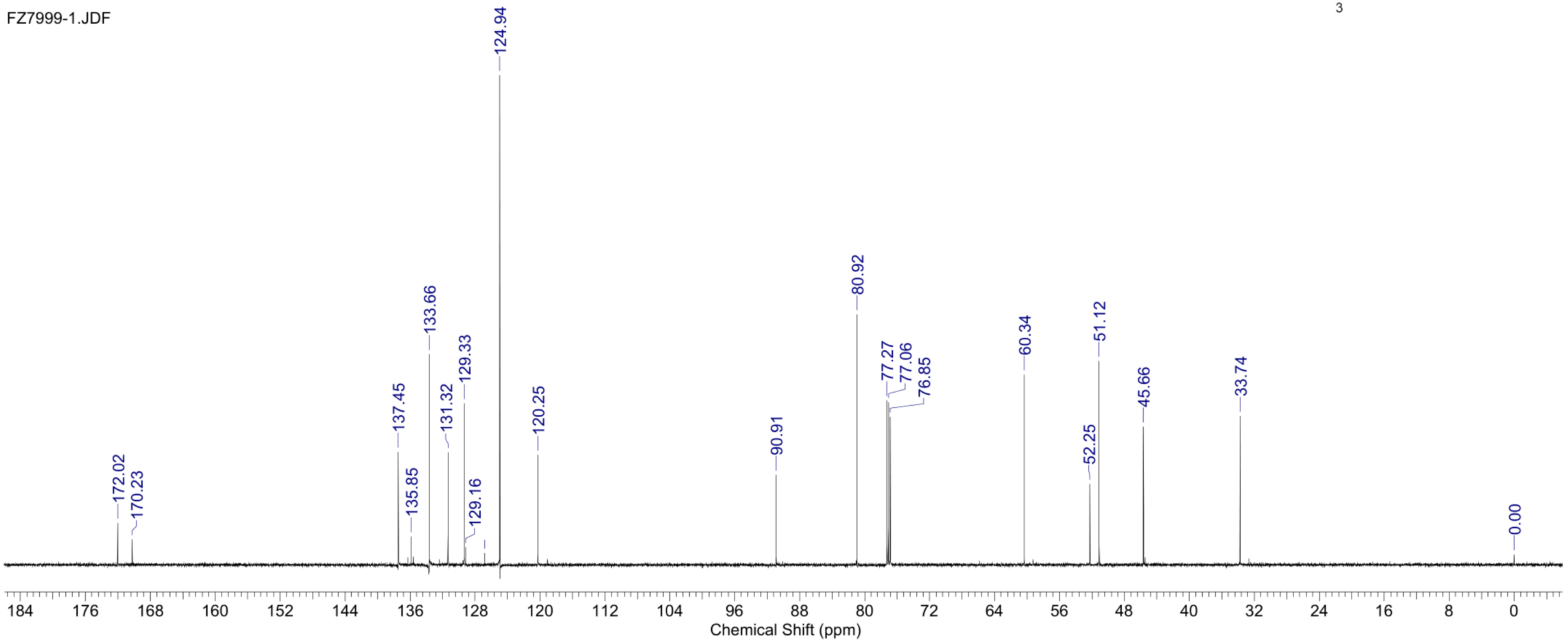

14.07.2020 18:37:56

Formula	$C_{20}H_{20}ClNO_4$	FW	373.8301

**Acquisition Time (sec)**	0.6921	**Comment**	single pulse decoupled gated NOE			**Date**	12 Aug 1990 06:31:00
**Date Stamp**	22 Oct 2019 12:34:08			**File Name**	C:\USERS\Лаба534\DOWNLOADS\FZ7999-1.JDF		
**Frequency (MHz)**	150.91	**Nucleus**	13C	**Number of Transients**	800	**Origin**	ECA 600
**Original Points Count**	32768	**Owner**	delta	**Points Count**	32768	**Pulse Sequence**	single_pulse_dec
**Receiver Gain**	54.00	**Solvent**	CHLOROFORM-d			**Spectrum Offset (Hz)**	15079.3525
**Sweep Width (Hz)**	47348.49	**Temperature (degree C)**	24.500				

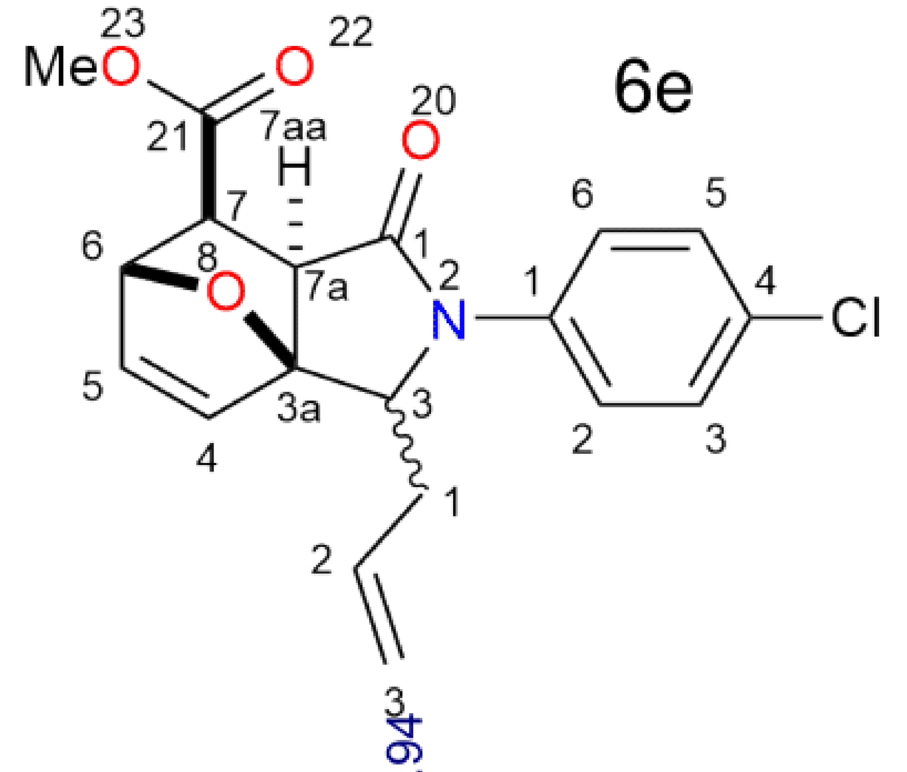

FZ7999-1.JDF

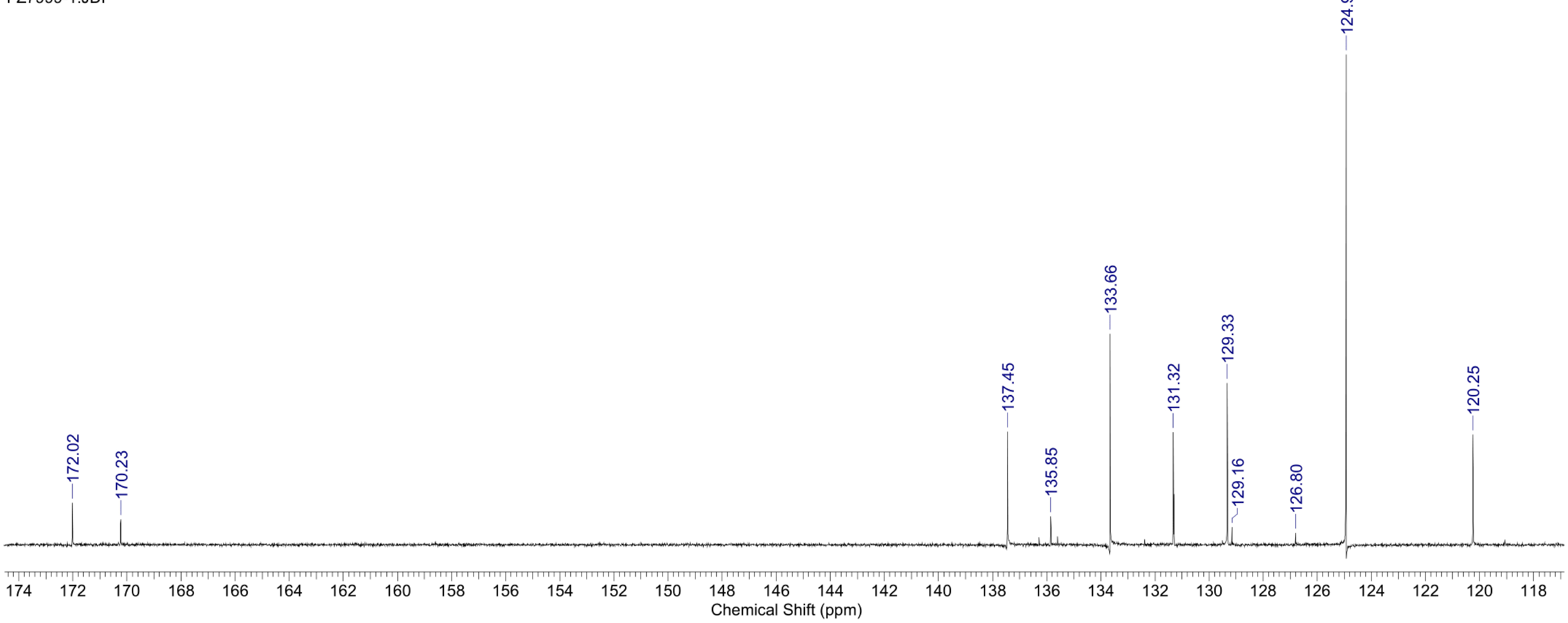

14.07.2020 18:38:05

**Formula** $C_{20}H_{20}ClNO_4$	**FW** 373.8301

**Acquisition Time (sec)**	0.6921	**Comment**	single pulse decoupled gated NOE	**Date**	12 Aug 1990 06:31:00
**Date Stamp**	22 Oct 2019 12:34:08	**File Name**	C:\USERS\Лаба534\DOWNLOADS\FZ7999-1.JDF		
**Frequency (MHz)**	150.91	**Nucleus**	13C	**Number of Transients**	800
**Origin**	ECA 600				
**Original Points Count**	32768	**Owner**	delta	**Points Count**	32768
**Pulse Sequence**	single_pulse_dec				
**Receiver Gain**	54.00	**Solvent**	CHLOROFORM-d	**Spectrum Offset (Hz)**	15079.3525
**Sweep Width (Hz)**	47348.49	**Temperature (degree C)**	24.500		

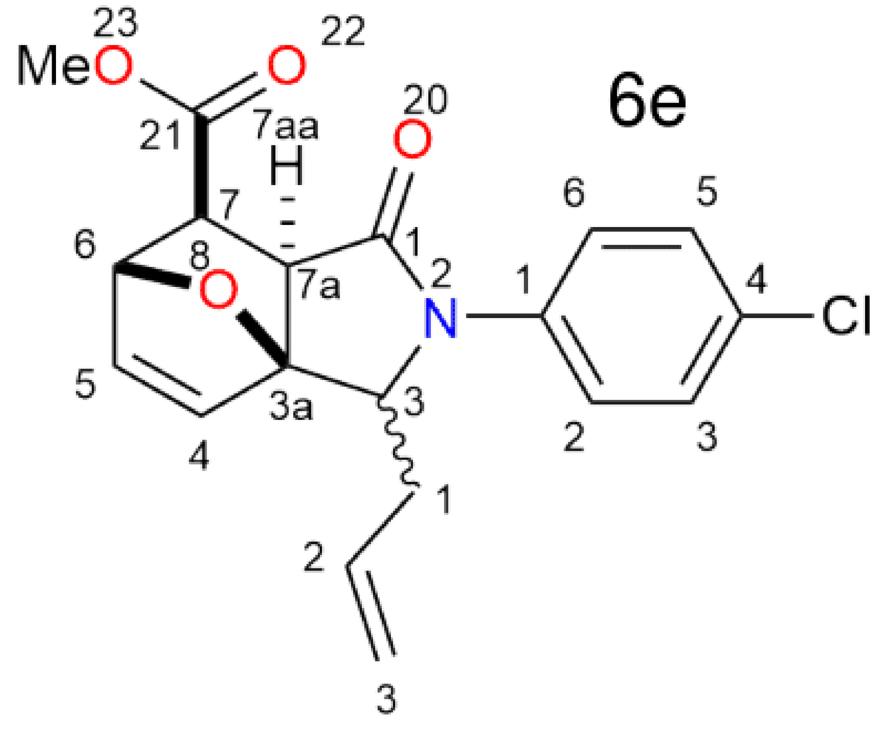

FZ7999-1.JDF

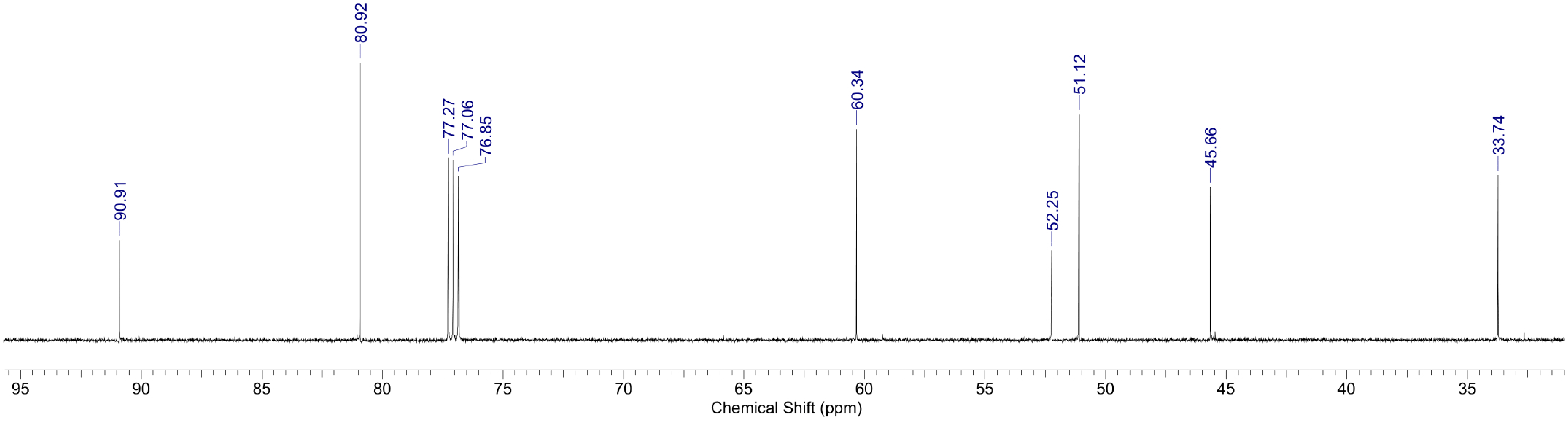

DEPT-135 NMR spectrum for compound 6e

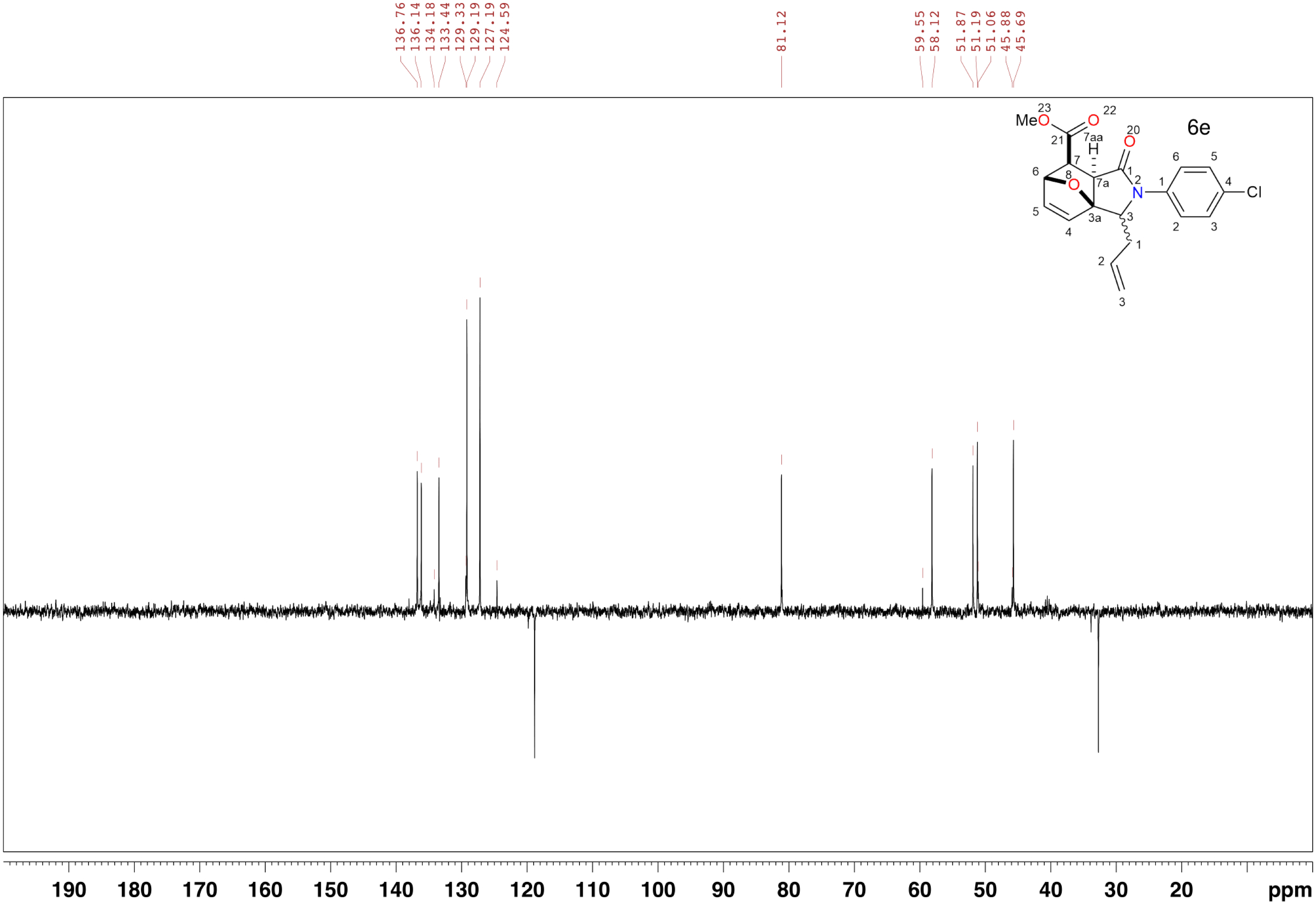

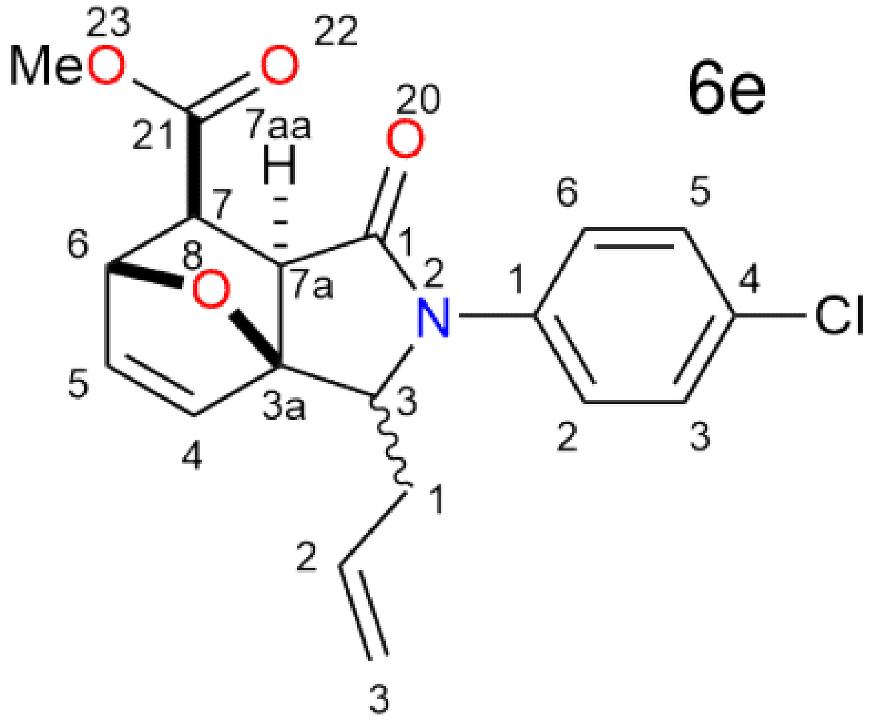

6e

NOESY NMR spectrum for compound 6e

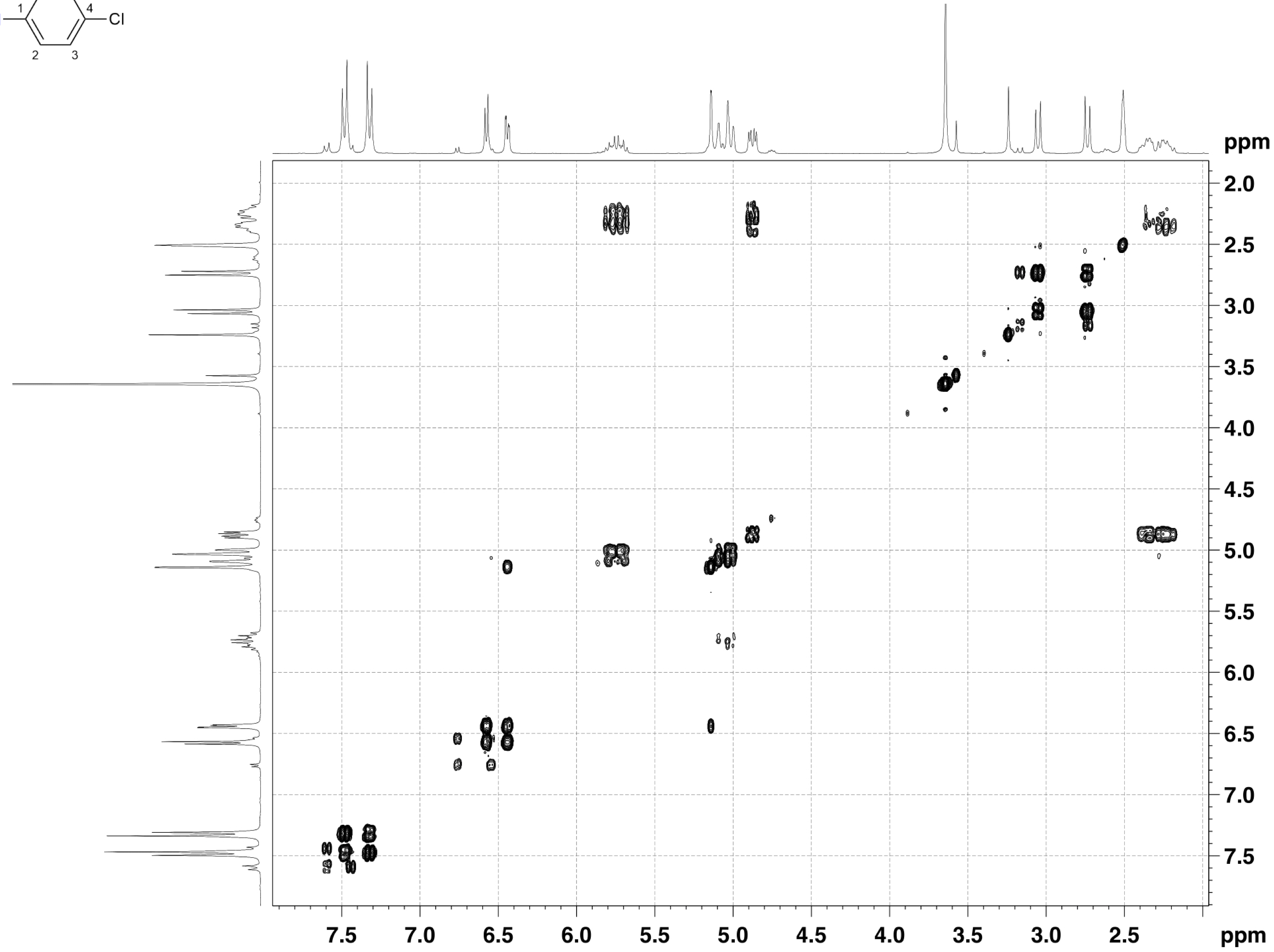

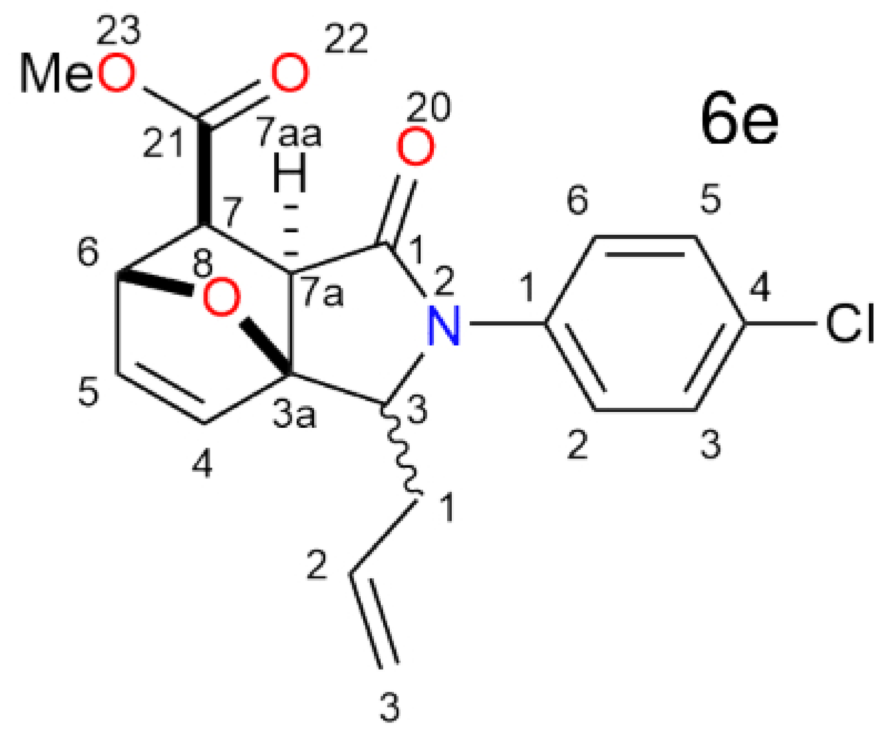

HSQC NMR spectrum for compound 5e

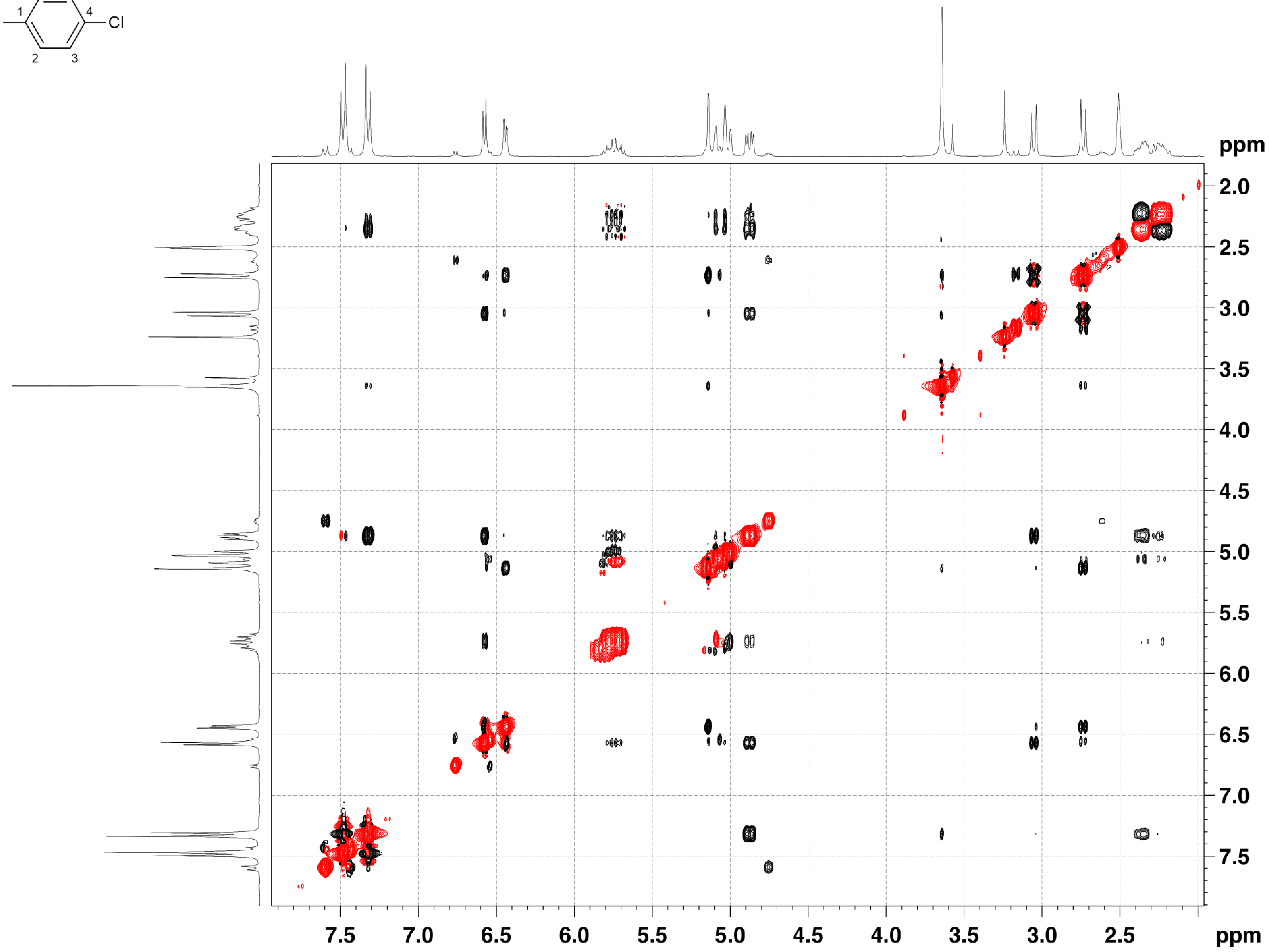

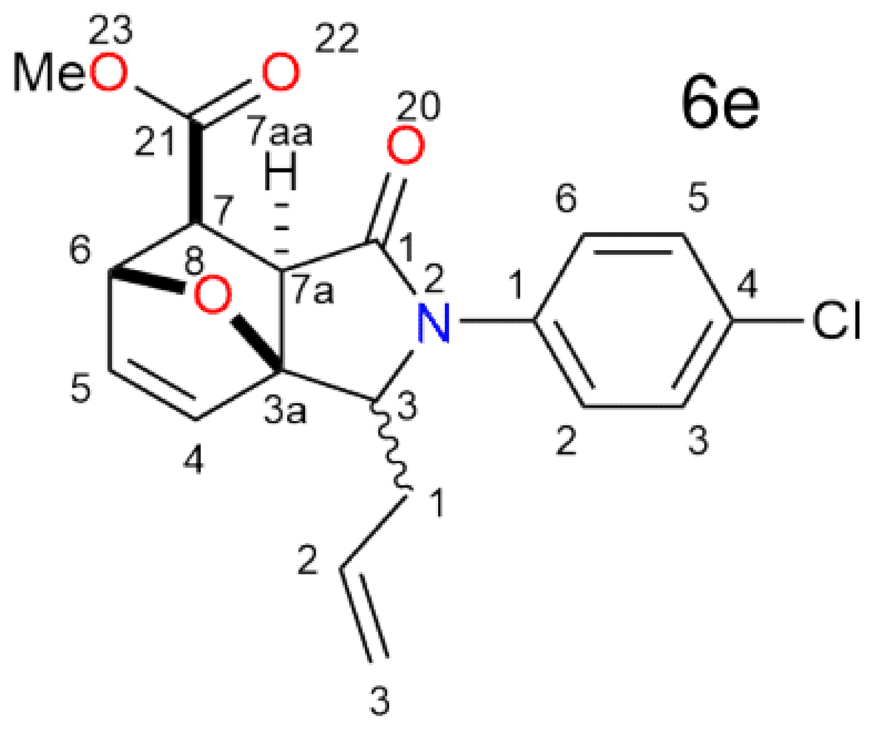

MeO
23
22
O
21
7aa
H
20
O
6e
6
5
7
8
O
7a
1
2
N
1
4
Cl
6
5
3a
3
2
3
4
1
2
3

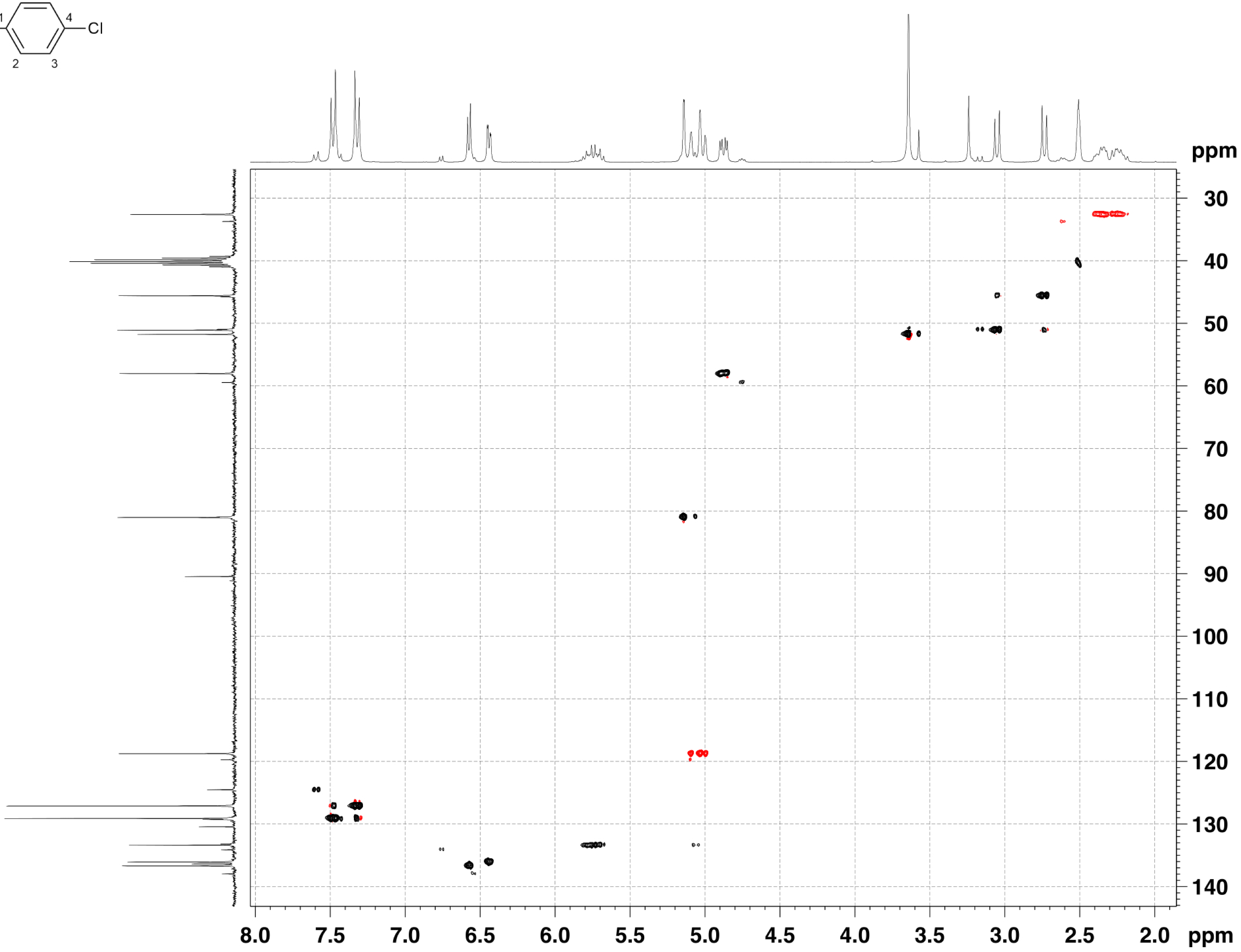

ppm
30
40
50
60
70
80
90
100
110
120
130
140
8.0
7.5
7.0
6.5
6.0
5.5
5.0
4.5
4.0
3.5
3.0
2.5
2.0
ppm

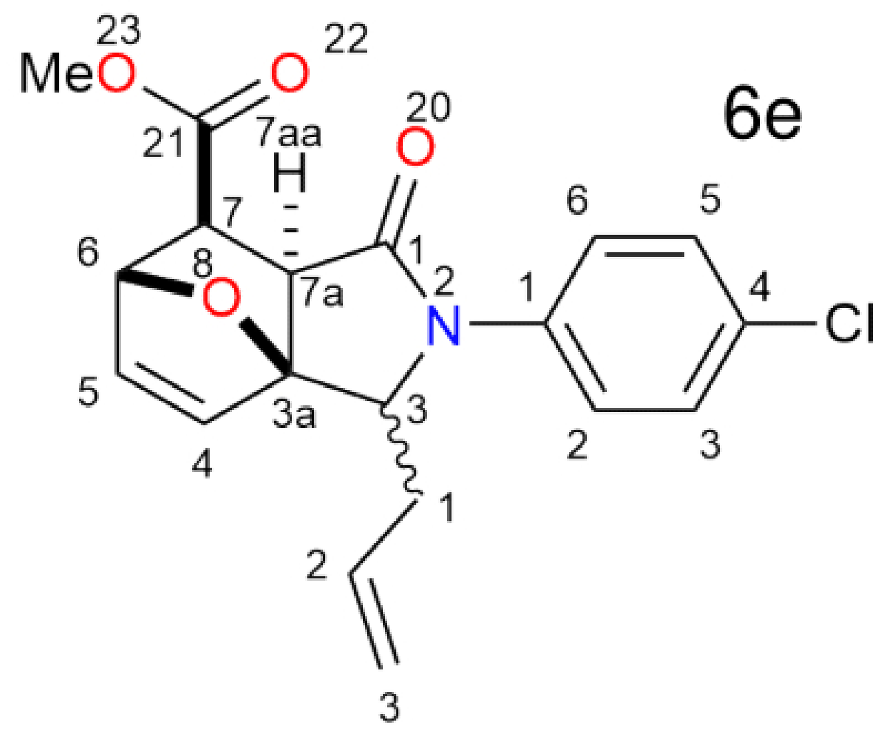
MeO
23
22
O
21
7aa
H
20
O
6e
6
7
8
O
7a
1
2
N
1
4
Cl
5
6
5
4
3a
3
2
3
1
2
3

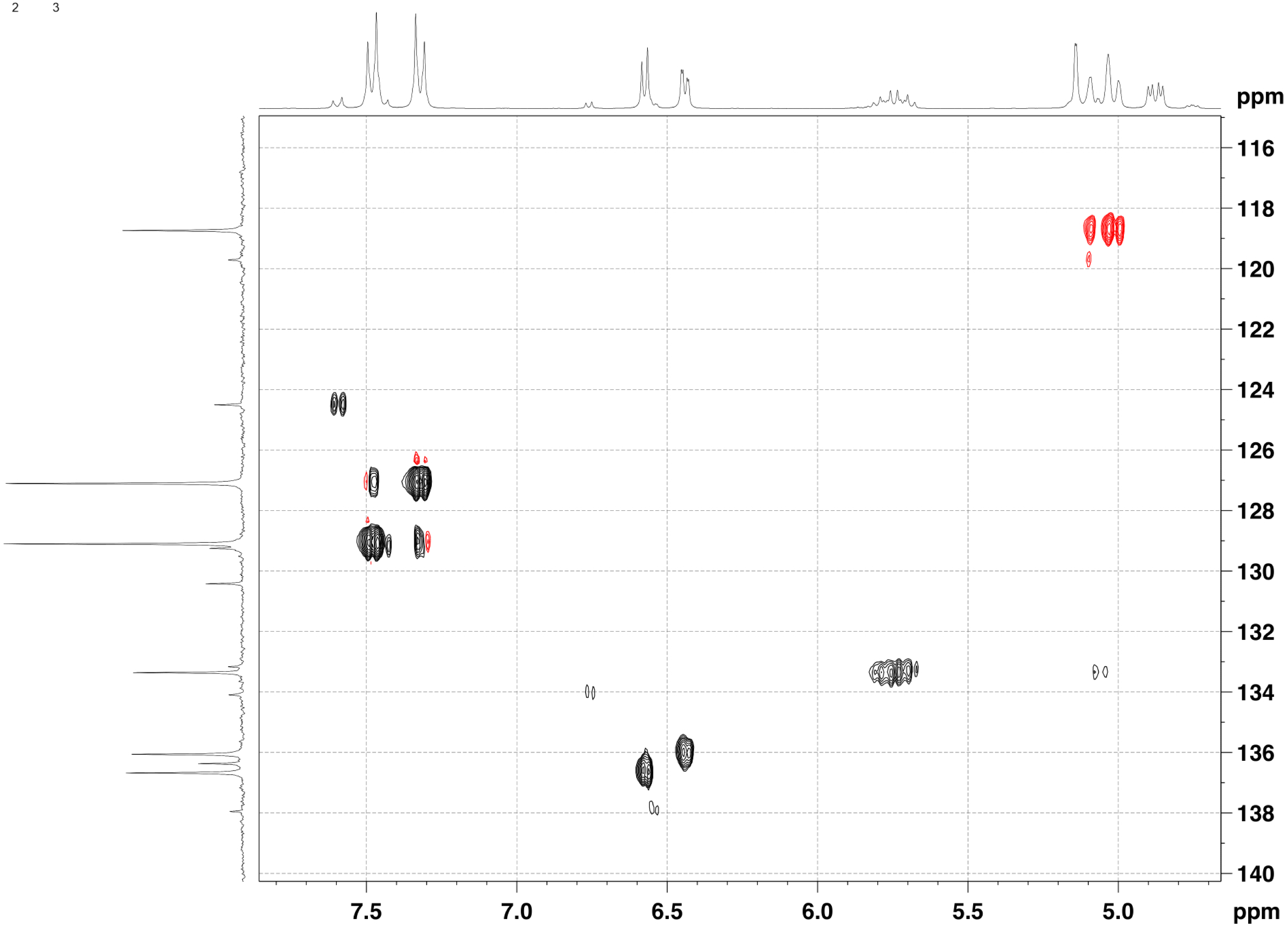
ppm
116
118
120
122
124
126
128
130
132
134
136
138
140
7.5
7.0
6.5
6.0
5.5
5.0
ppm

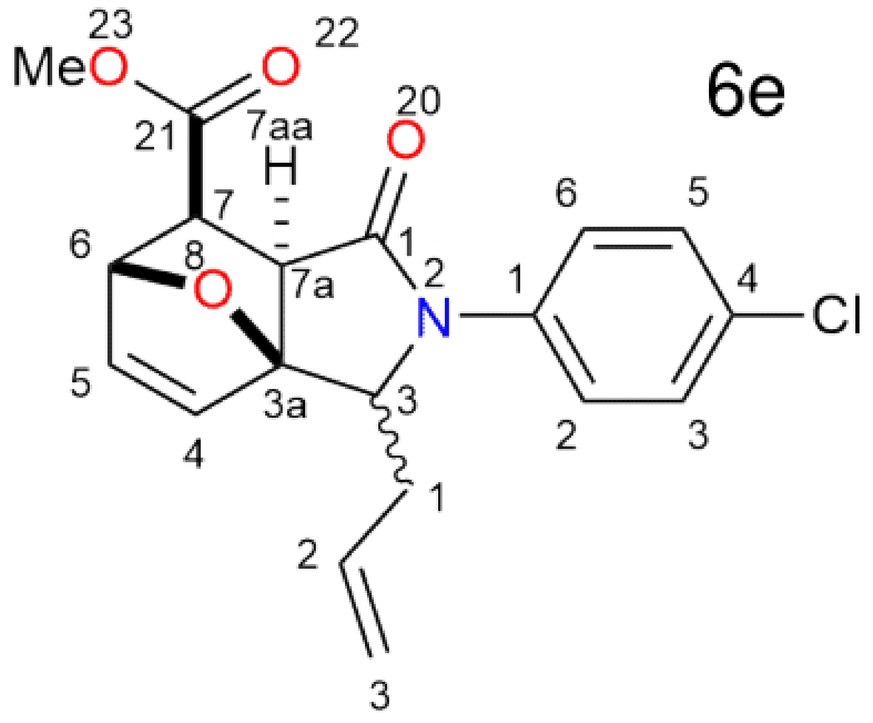

MeO
23
22
21
7aa
H
20
O
6
5
7
1
2
8
7a
N
1
4
Cl
O
5
3a
3
2
3
4
1
2
3

6e

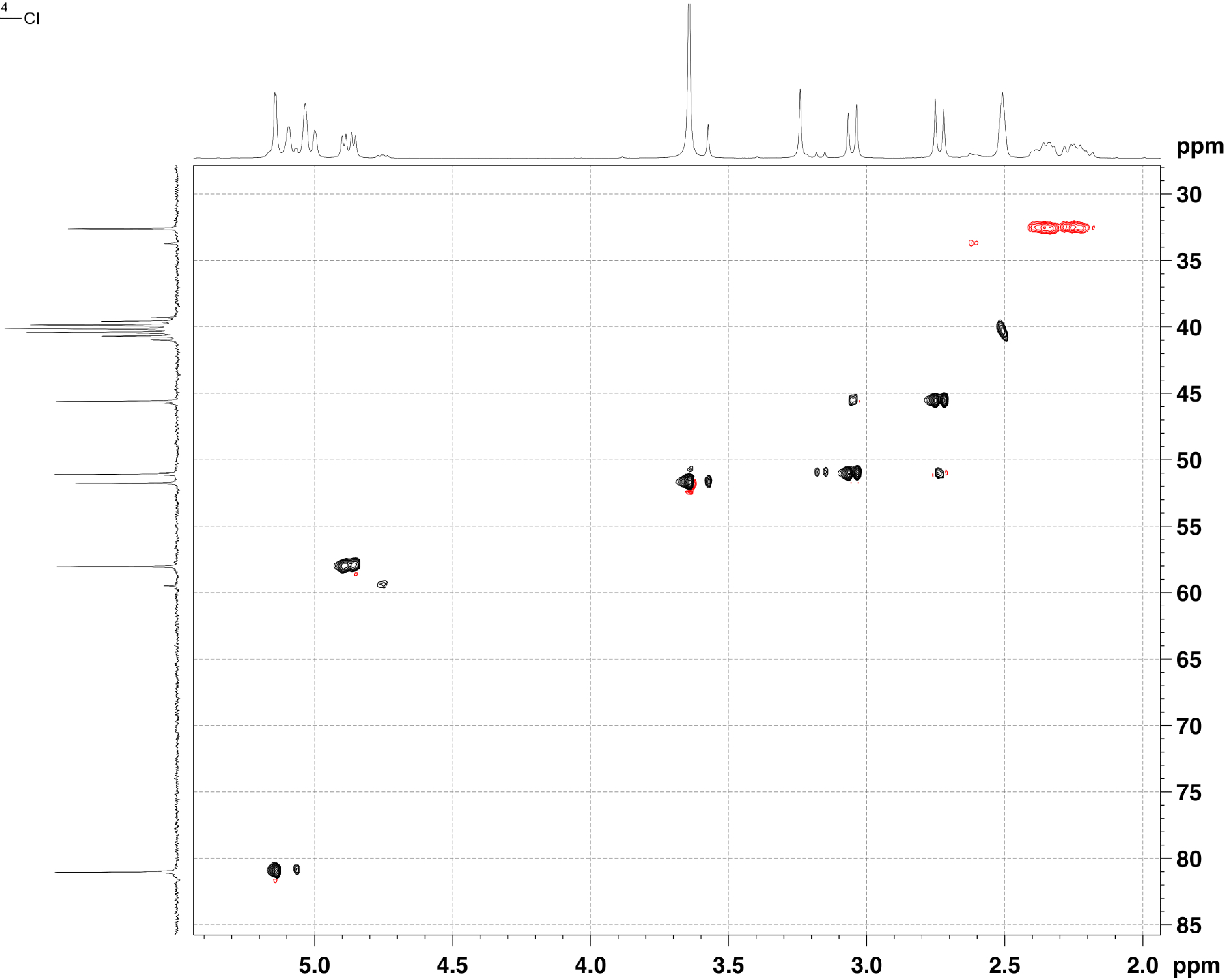

ppm
30
35
40
45
50
55
60
65
70
75
80
85
5.0
4.5
4.0
3.5
3.0
2.5
2.0
ppm

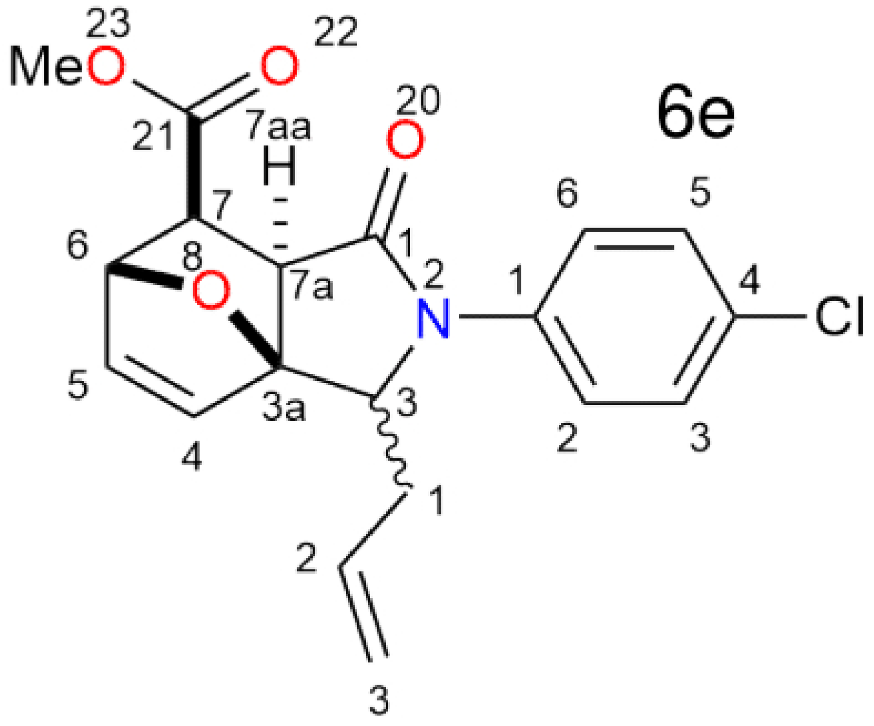

6e

HMBC NMR spectrum for compound 6e

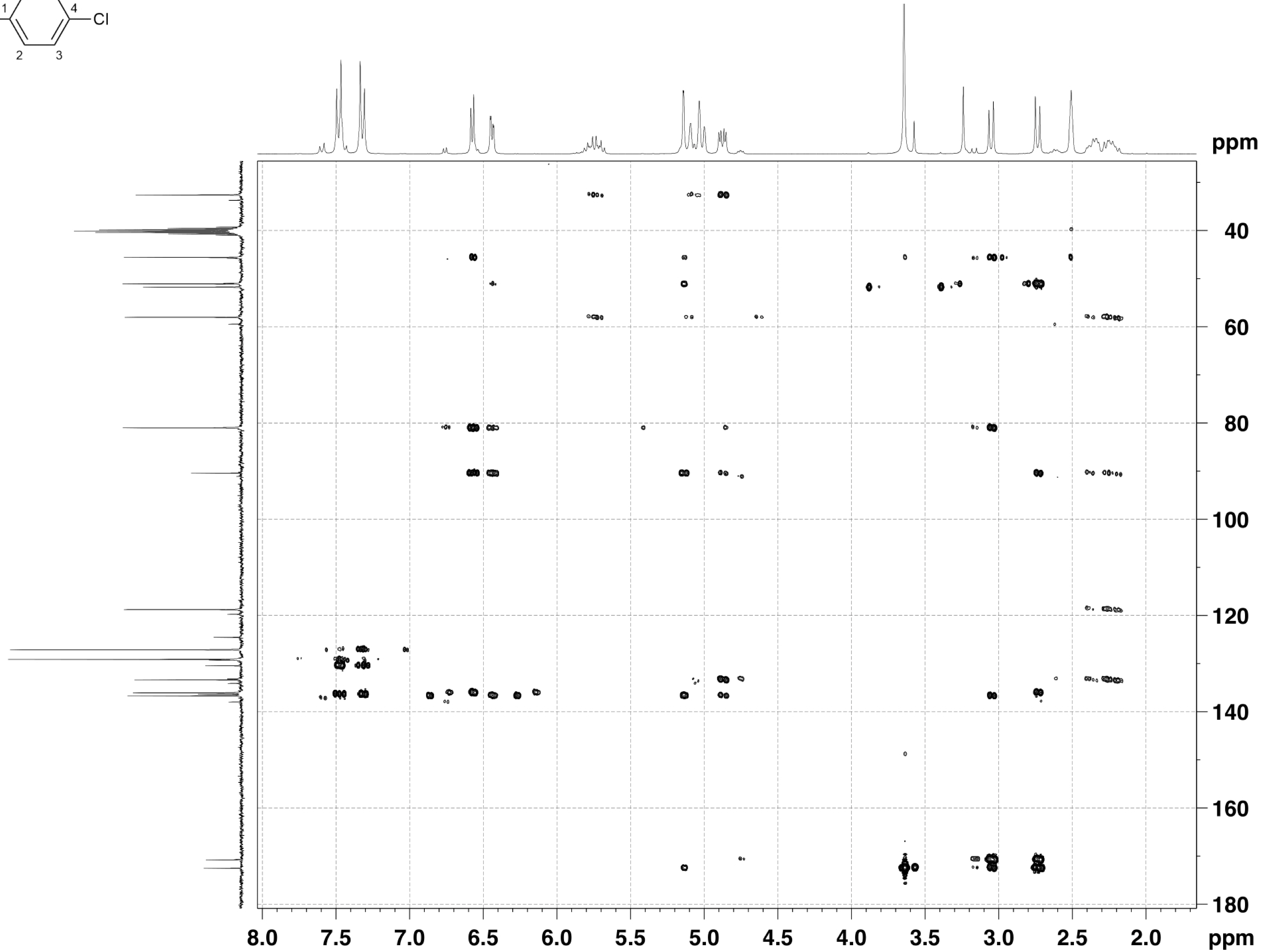

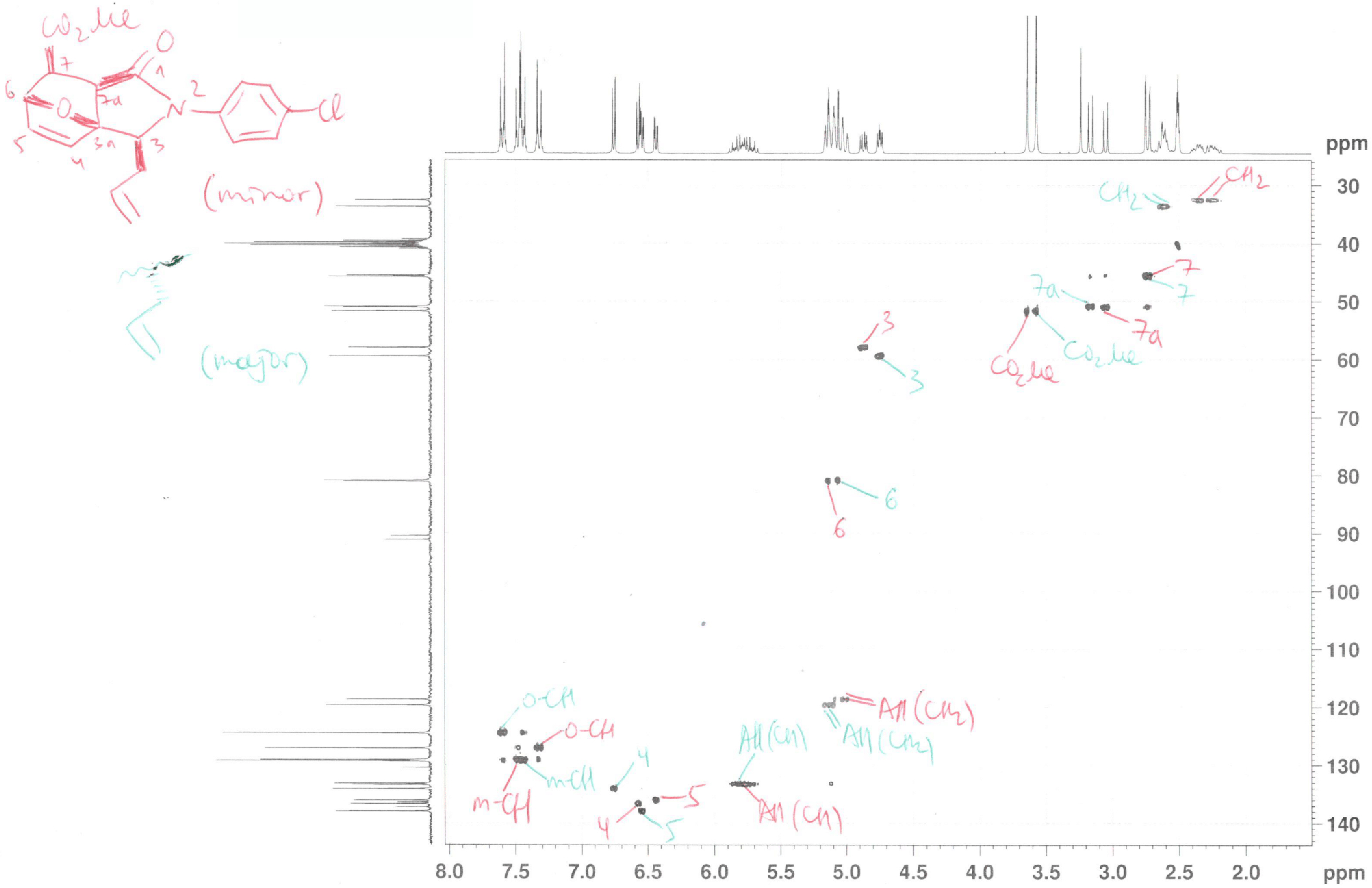

CO2Me
O
1
2
N
Cl
7
7a
6
5
4
3a
3
(minor)
(major)
ppm
30
40
50
60
70
80
90
100
110
120
130
140
8.0
7.5
7.0
6.5
6.0
5.5
5.0
4.5
4.0
3.5
3.0
2.5
2.0
CH2
7a
CO2Me
All (CH2)
All (CH)
o-CH
m-CH

NMR spectrum data for compound 6f

Acquisition Time (sec)	1.9818	***Comment***	single_pulse	***Date***	08 May 1990 05:59:05				
Date Stamp	18 Jul 2019 12:02:43			***File Name***	C:\Users\Fedor\Desktop\18.07.19\FZ7569-1.jdf			***Frequency (MHz)***	600.17
Nucleus	1H	***Number of Transients***	8	***Origin***	ECA 600	***Original Points Count***	32768	***Owner***	delta
Points Count	32768	***Pulse Sequence***	single_pulse.ex2			***Receiver Gain***	40.00	***Solvent***	CHLOROFORM-d
Spectrum Offset (Hz)	5410.6274	***Sweep Width (Hz)***	16534.39	***Temperature (degree C)***	22.700				

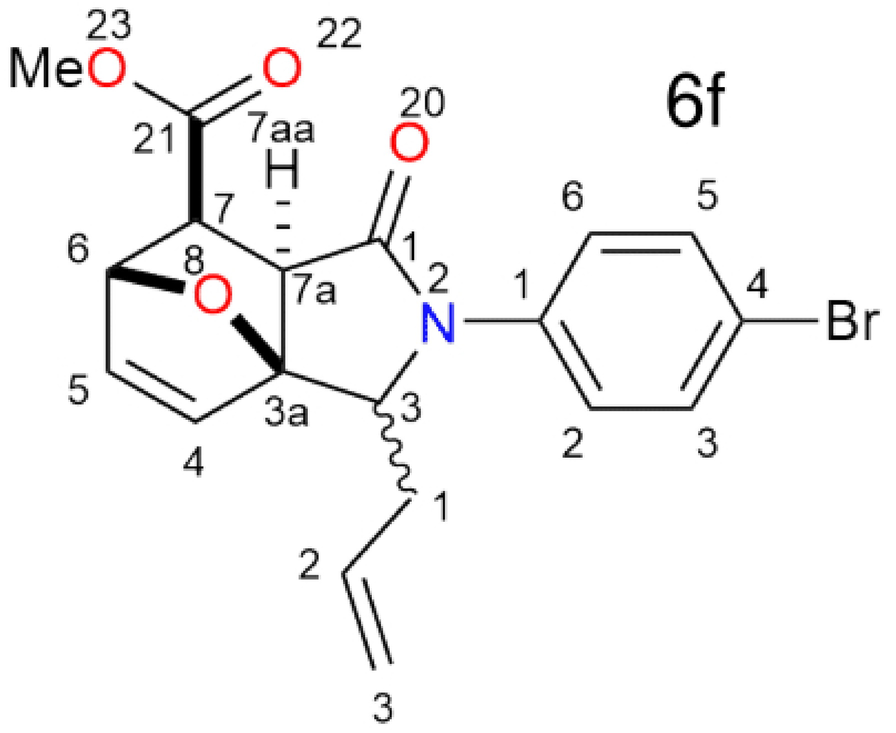

6f

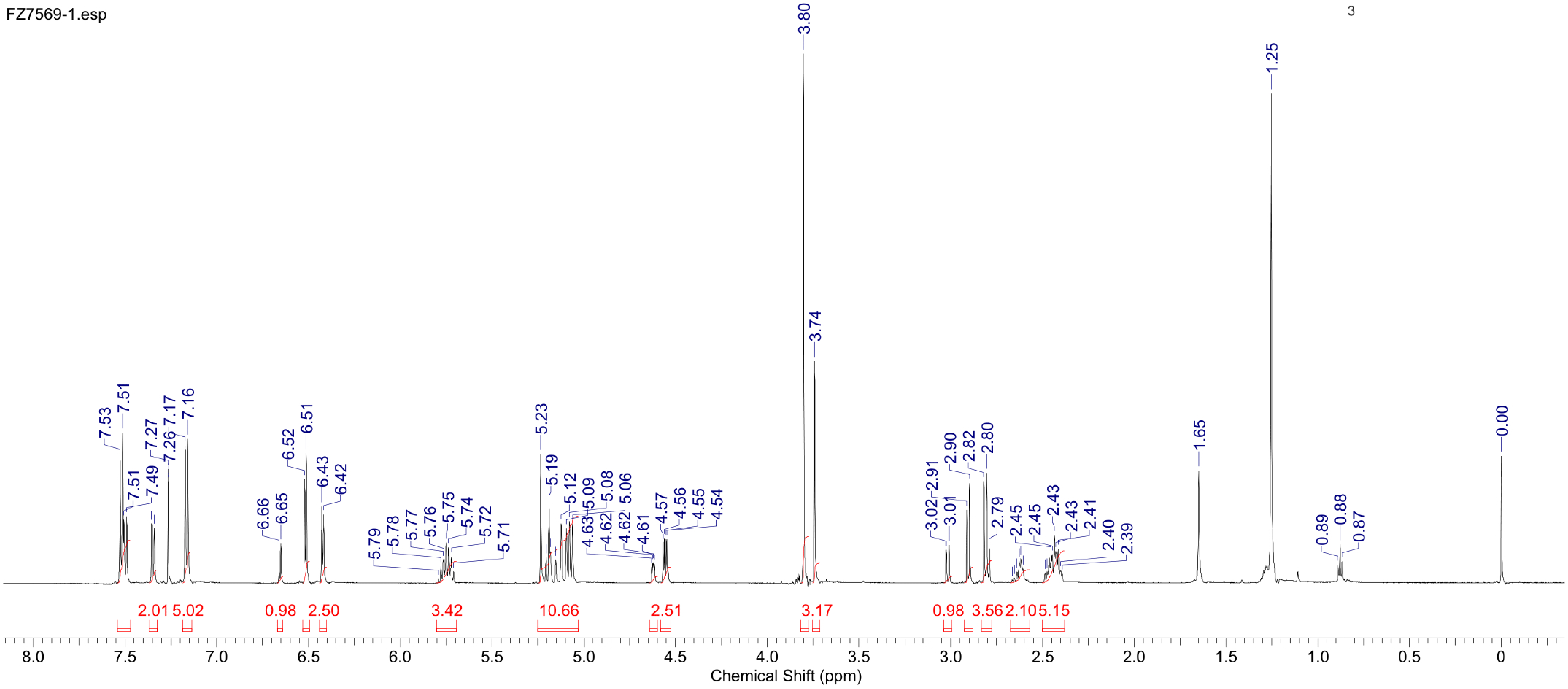

Acquisition Time (sec)	1.9818	***Comment***	single_pulse	***Date***	08 May 1990 05:59:05				
Date Stamp	18 Jul 2019 12:02:43			***File Name***	C:\Users\Fedor\Desktop\18.07.19\FZ7569-1.jdf			***Frequency (MHz)***	600.17
Nucleus	1H	***Number of Transients***	8	***Origin***	ECA 600	***Original Points Count***	32768	***Owner***	delta
Points Count	32768	***Pulse Sequence***	single_pulse.ex2			***Receiver Gain***	40.00	***Solvent***	CHLOROFORM-d
Spectrum Offset (Hz)	5410.6274	***Sweep Width (Hz)***	16534.39	***Temperature (degree C)***	22.700				

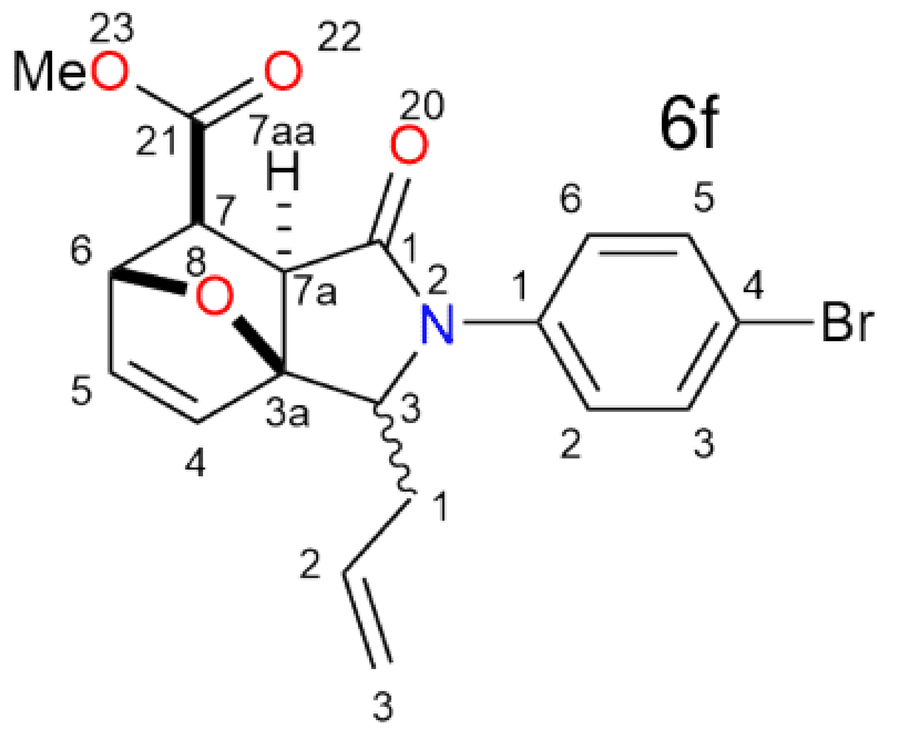

FZ7569-1.esp

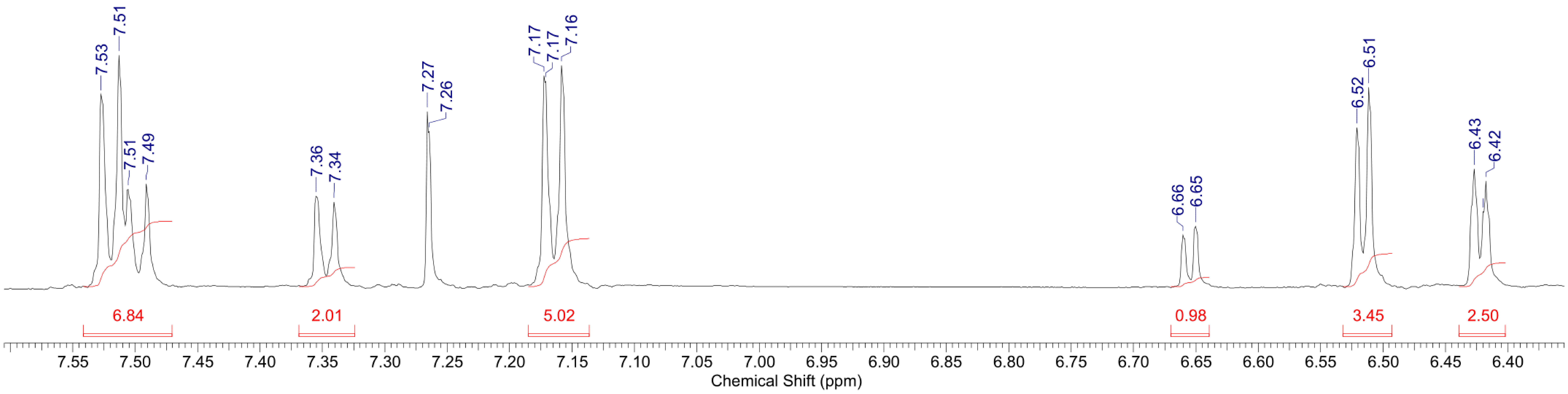

Acquisition Time (sec)	1.9818	***Comment***	single_pulse	***Date***	08 May 1990 05:59:05				
Date Stamp	18 Jul 2019 12:02:43			***File Name***	C:\Users\Fedor\Desktop\18.07.19\FZ7569-1.jdf			***Frequency (MHz)***	600.17
Nucleus	1H	***Number of Transients***	8	***Origin***	ECA 600	***Original Points Count***	32768	***Owner***	delta
Points Count	32768	***Pulse Sequence***	single_pulse.ex2			***Receiver Gain***	40.00	***Solvent***	CHLOROFORM-d
Spectrum Offset (Hz)	5410.6274	***Sweep Width (Hz)***	16534.39	***Temperature (degree C)***	22.700				

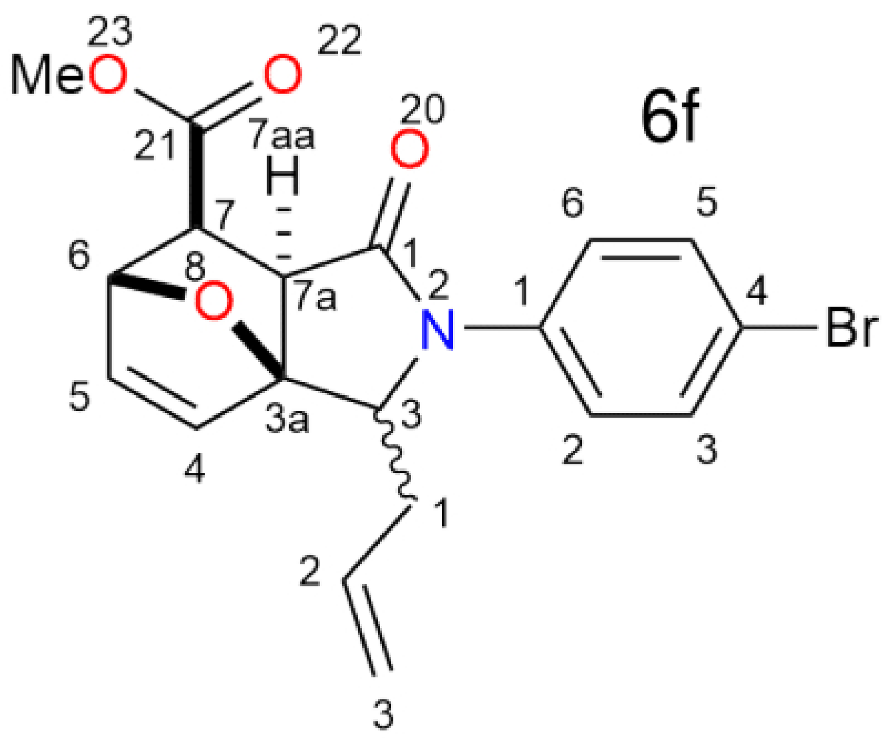

6f

FZ7569-1.esp

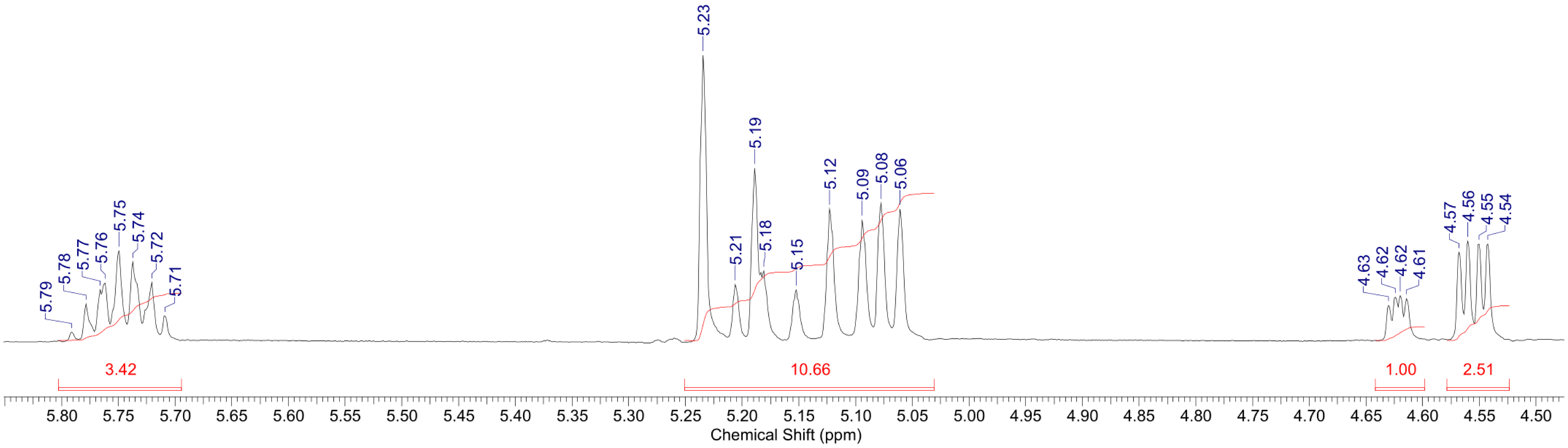

Acquisition Time (sec)	1.9818	***Comment***	single_pulse	***Date***	08 May 1990 05:59:05		
Date Stamp	18 Jul 2019 12:02:43			***File Name***	C:\Users\Fedor\Desktop\18.07.19\FZ7569-1.jdf	***Frequency (MHz)***	600.17
Nucleus	1H	***Number of Transients***	8	***Origin***	ECA 600	***Original Points Count*** 32768	***Owner*** delta
Points Count	32768	***Pulse Sequence***	single_pulse.ex2			***Receiver Gain*** 40.00	***Solvent*** CHLOROFORM-d
Spectrum Offset (Hz)	5410.6274	***Sweep Width (Hz)***	16534.39	***Temperature (degree C)***	22.700		

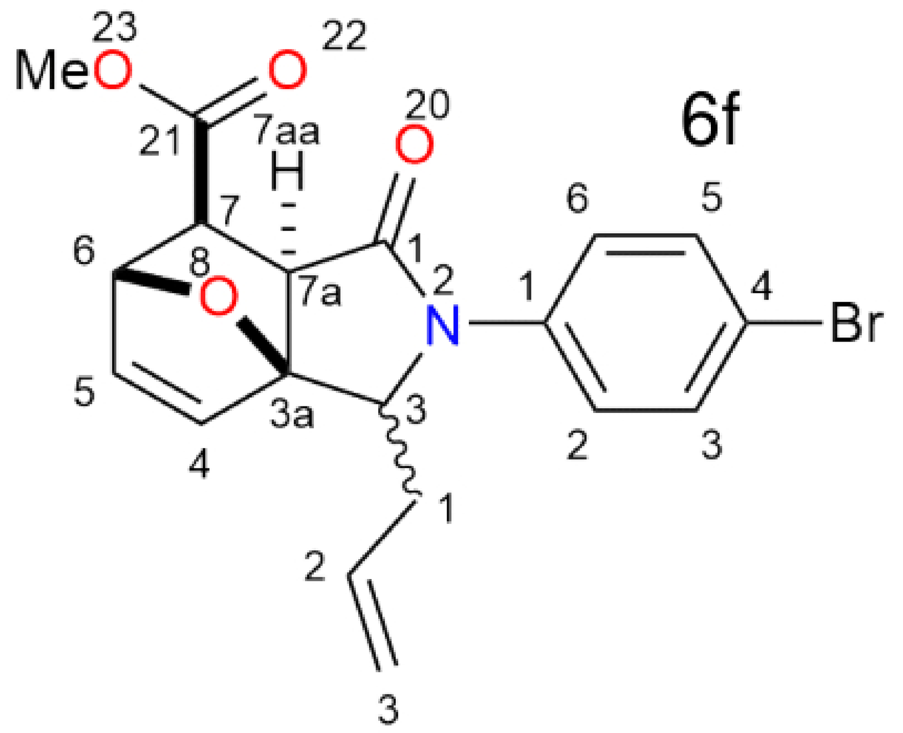

6f

FZ7569-1.esp

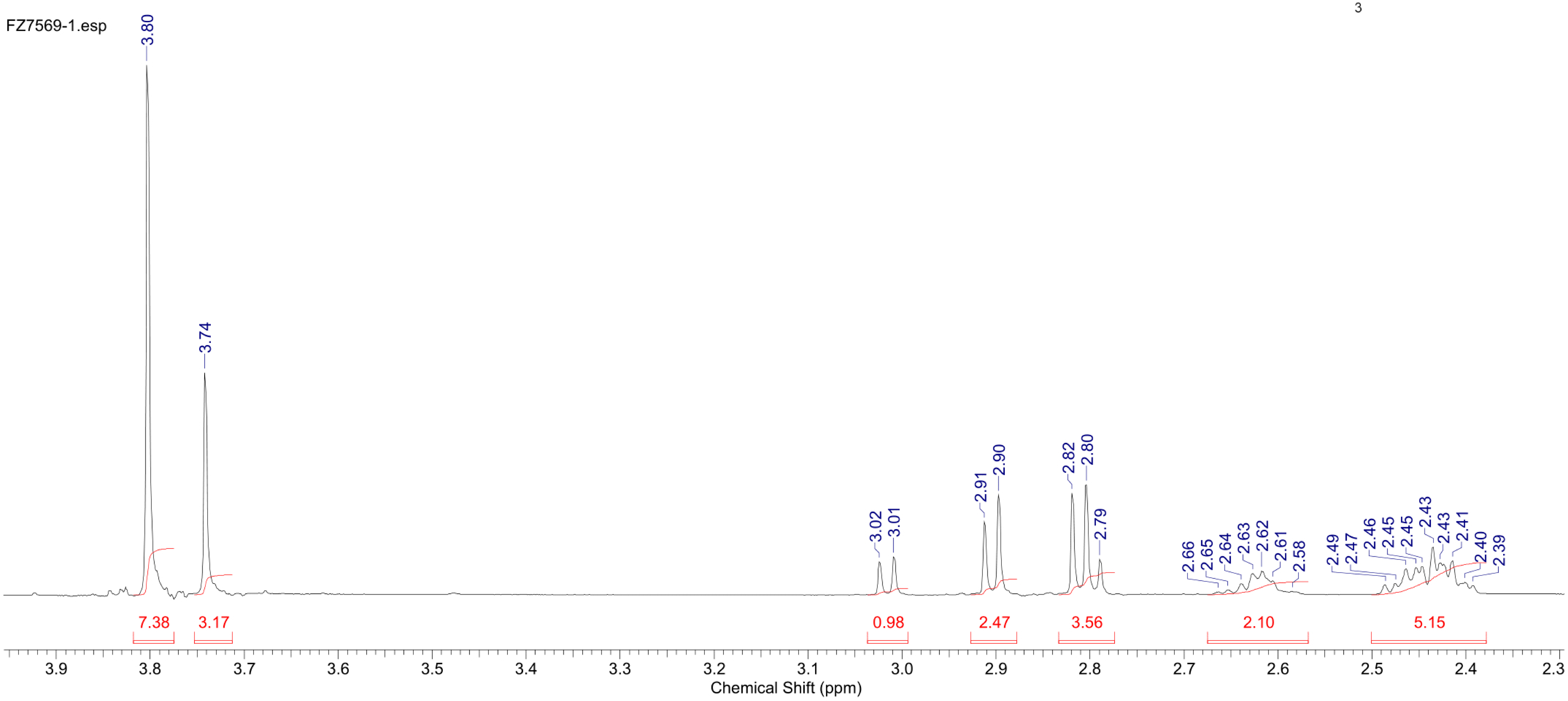

14.07.2020 18:34:57

**Formula** $C_{20}H_{20}BrNO_4$	**FW** 418.2811

**Acquisition Time (sec)**	0.6921	**Comment**	single pulse decoupled gated NOE			**Date**	12 Aug 1990 05:05:53
**Date Stamp**	22 Oct 2019 11:09:01			**File Name**	C:\USERS\Ла6а534\DOWNLOADS\FZ8000-1.JDF		
**Frequency (MHz)**	150.91	**Nucleus**	13C	**Number of Transients**	800	**Origin**	ECA 600
**Original Points Count**	32768	**Owner**	delta	**Points Count**	32768	**Pulse Sequence**	single_pulse_dec
**Receiver Gain**	54.00	**Solvent**	CHLOROFORM-d			**Spectrum Offset (Hz)**	15079.3525
**Sweep Width (Hz)**	47348.49	**Temperature (degree C)**	24.600				

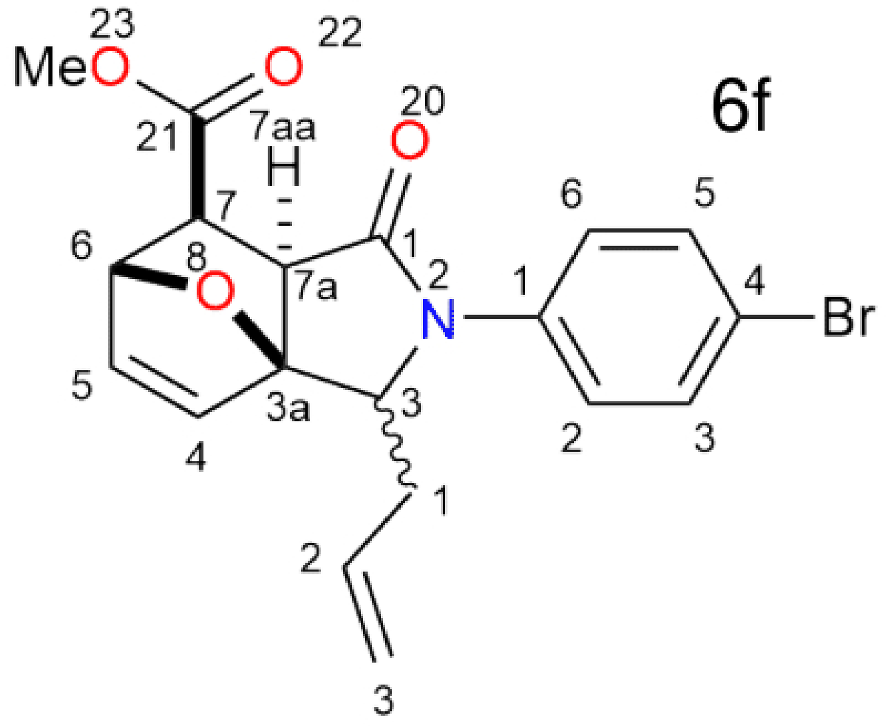

6f

FZ8000-1.JDF

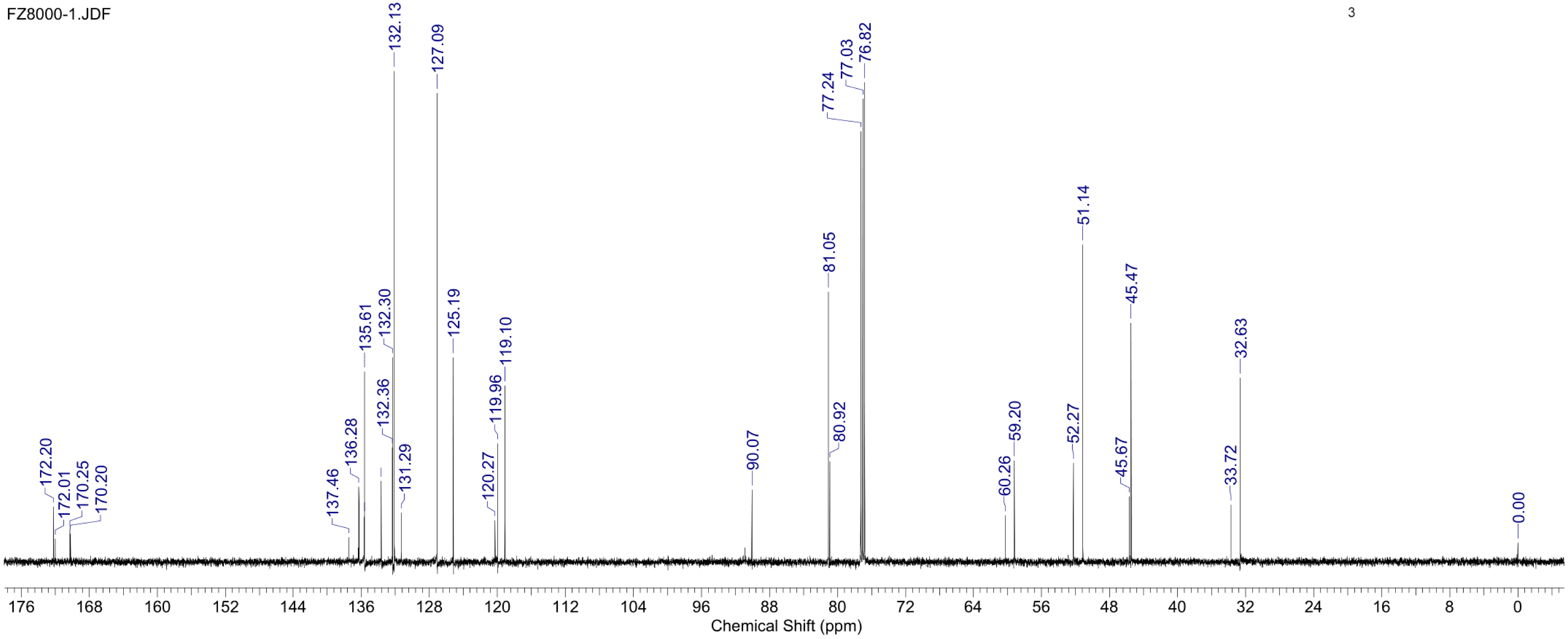

14.07.2020 18:35:08

Formula	$C_{20}H_{20}BrNO_4$	*FW*	418.2811

**Acquisition Time (sec)**	0.6921	**Comment**	single pulse decoupled gated NOE			**Date**	12 Aug 1990 05:05:53
**Date Stamp**	22 Oct 2019 11:09:01			**File Name**	C:\USERS\Ла6а534\DOWNLOADS\FZ8000-1.JDF		
**Frequency (MHz)**	150.91	**Nucleus**	13C	**Number of Transients**	800	**Origin**	ECA 600
**Original Points Count**	32768	**Owner**	delta	**Points Count**	32768	**Pulse Sequence**	single_pulse_dec
**Receiver Gain**	54.00	**Solvent**	CHLOROFORM-d			**Spectrum Offset (Hz)**	15079.3525
**Sweep Width (Hz)**	47348.49	**Temperature (degree C)**	24.600				

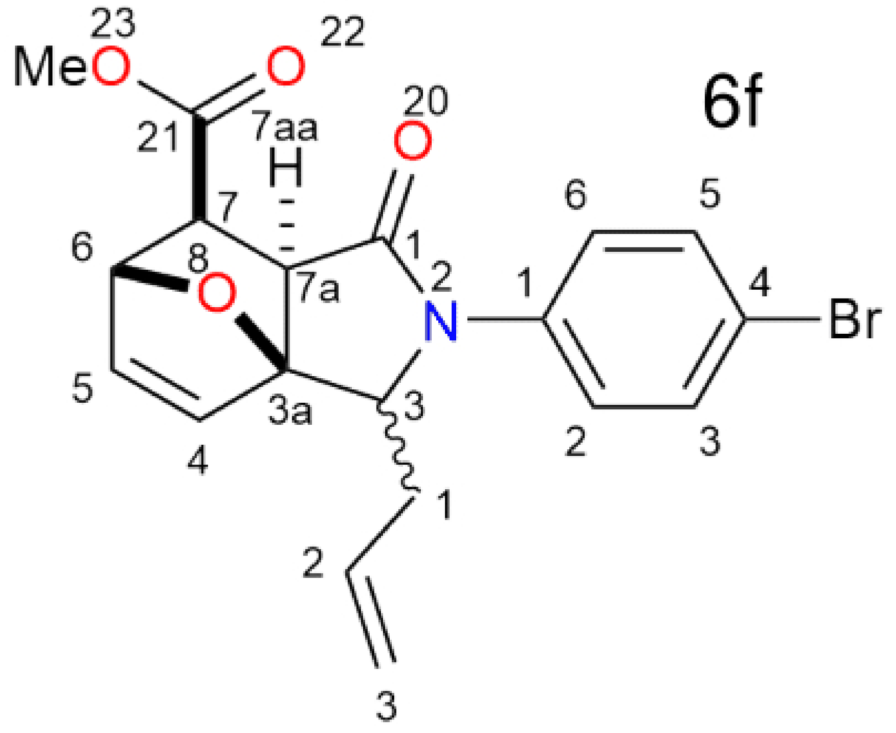

6f

FZ8000-1.JDF

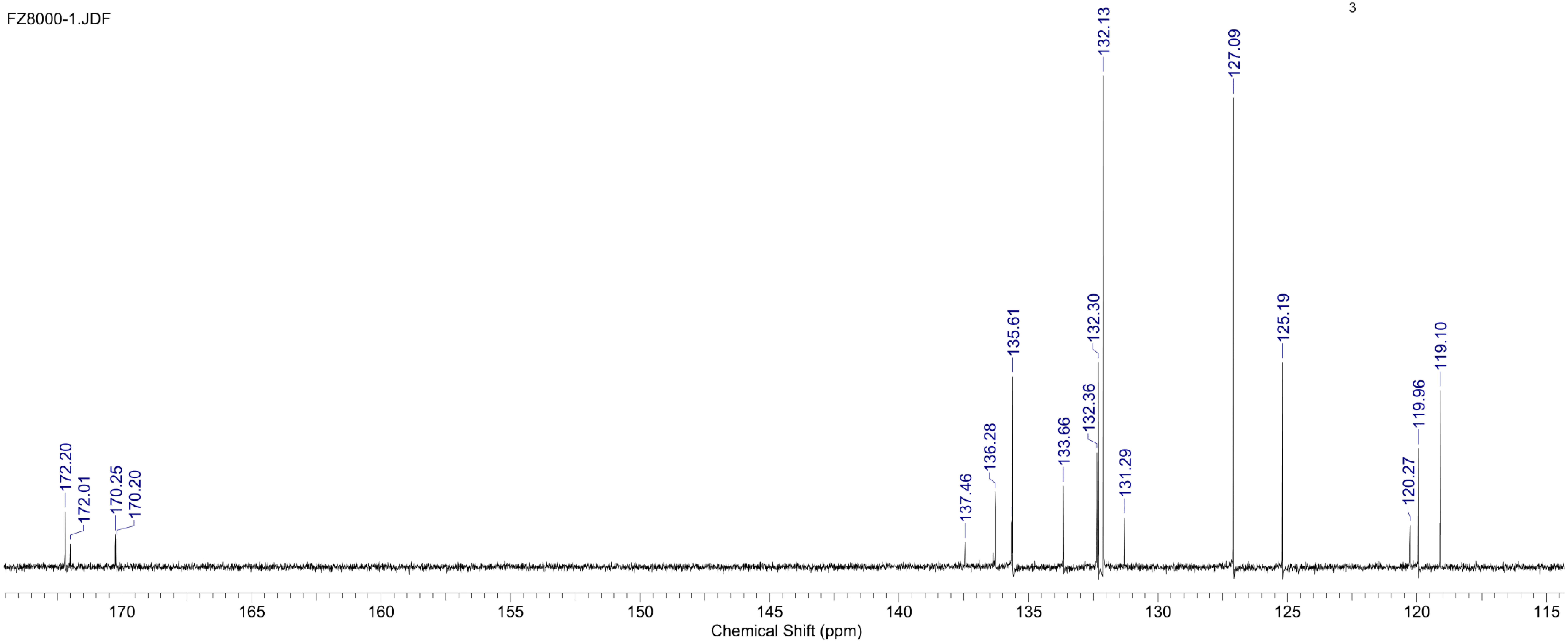

14.07.2020 18:35:19

**Formula** $C_{20}H_{20}BrNO_4$	**FW** 418.2811						
**Acquisition Time (sec)**	0.6921	**Comment**	single pulse decoupled gated NOE			**Date**	12 Aug 1990 05:05:53
**Date Stamp**	22 Oct 2019 11:09:01			**File Name**	C:\USERS\Лаба534\DOWNLOADS\FZ8000-1.JDF		
**Frequency (MHz)**	150.91	**Nucleus**	13C	**Number of Transients**	800	**Origin**	ECA 600
**Original Points Count**	32768	**Owner**	delta	**Points Count**	32768	**Pulse Sequence**	single_pulse_dec
**Receiver Gain**	54.00	**Solvent**	CHLOROFORM-d			**Spectrum Offset (Hz)**	15079.3525
**Sweep Width (Hz)**	47348.49	**Temperature (degree C)**	24.600				

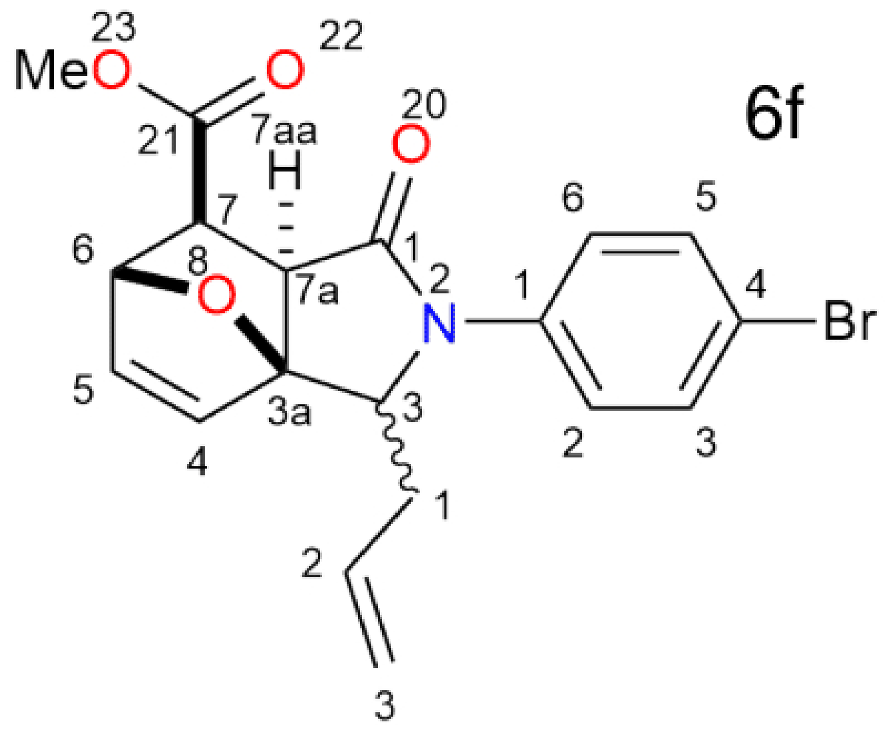

FZ8000-1.JDF

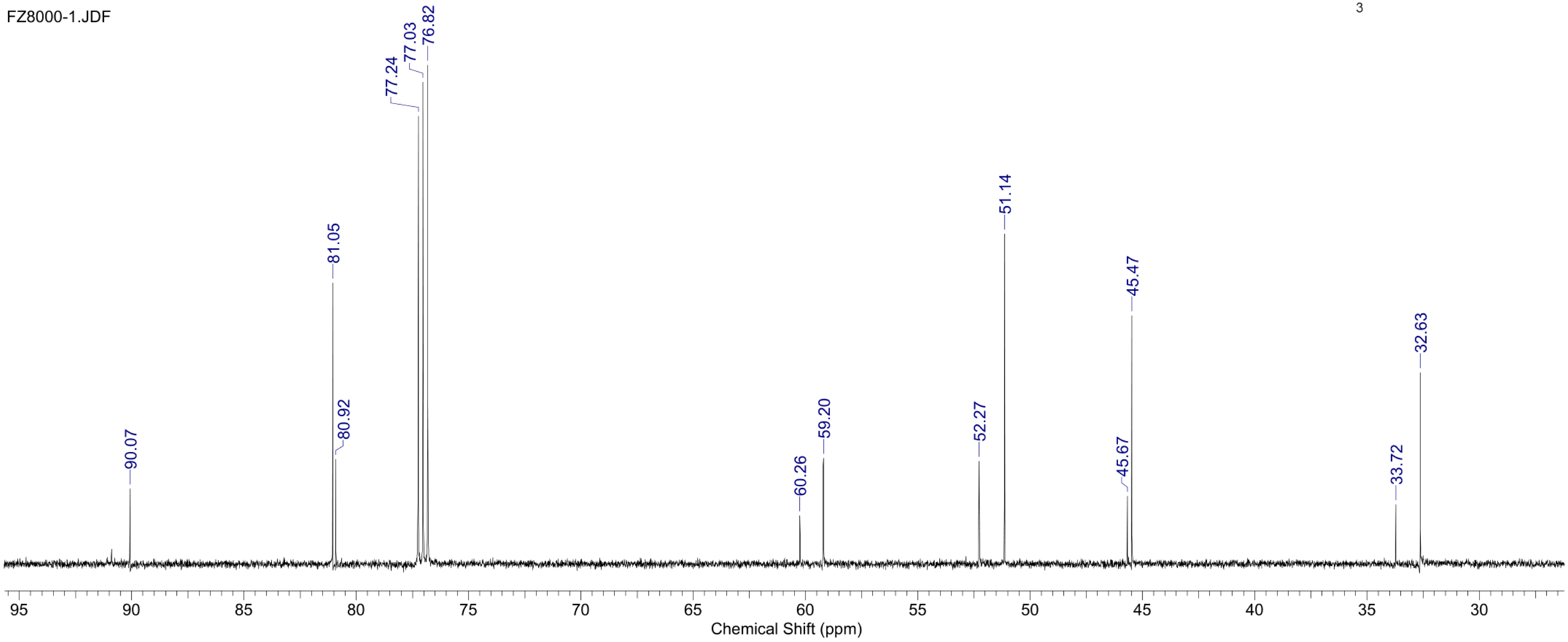

NMR spectrum data for compound 6g

26.11.2019 16:01:34

Formula	$C_{19}H_{18}INO_4$	***FW***	451.2550

Acquisition Time (sec)	1.9818	***Comment***	single_pulse	***Date***	02 Sep 1990 07:15:54		
Date Stamp	12 Nov 2019 12:33:01			***File Name***	C:\Users\User\Desktop\05.11.19\FZ8122-1.jdf	***Frequency (MHz)***	600.17
Nucleus	1H	***Number of Transients***	8	***Origin***	ECA 600	***Original Points Count*** 32768	***Owner*** delta
Points Count	32768	***Pulse Sequence***	single_pulse.ex2			***Receiver Gain*** 44.00	***Solvent*** CHLOROFORM-d
Spectrum Offset (Hz)	5401.5503	***Sweep Width (Hz)***	16534.39	***Temperature (degree C)***	23.000		

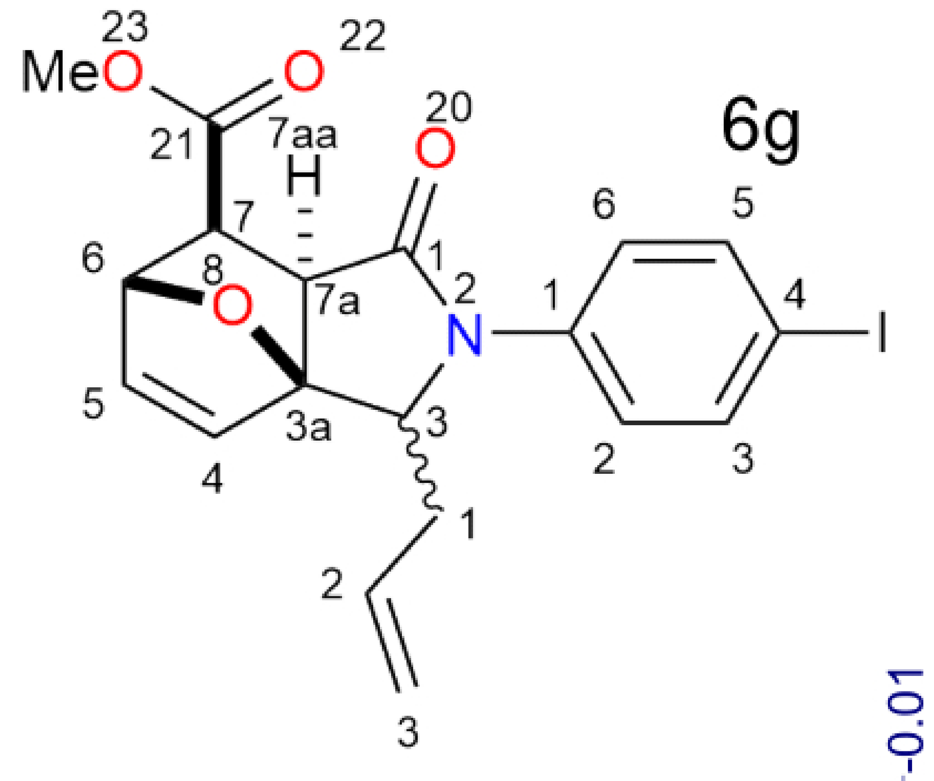

FZ8122-1.jdf

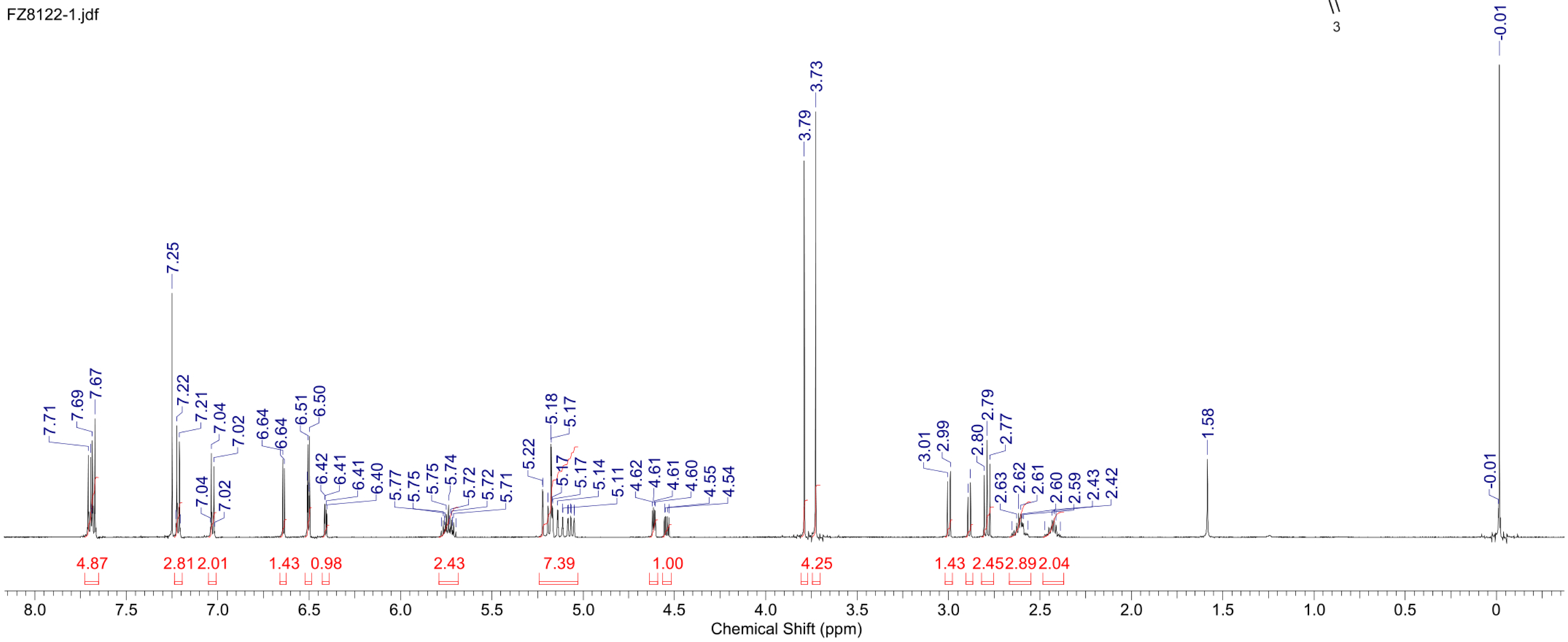

26.11.2019 16:01:54

Formula	$C_{19}H_{18}INO_4$	FW	451.2550

Acquisition Time (sec)	1.9818	Comment	single_pulse	Date	02 Sep 1990 07:15:54		
Date Stamp	12 Nov 2019 12:33:01			File Name	C:\Users\User\Desktop\05.11.19\FZ8122-1.jdf	Frequency (MHz)	600.17
Nucleus	1H	Number of Transients	8	Origin	ECA 600	Original Points Count	32768
						Owner	delta
Points Count	32768	Pulse Sequence	single_pulse.ex2			Receiver Gain	44.00
						Solvent	CHLOROFORM-d
Spectrum Offset (Hz)	5401.5503	Sweep Width (Hz)	16534.39	Temperature (degree C)	23.000		

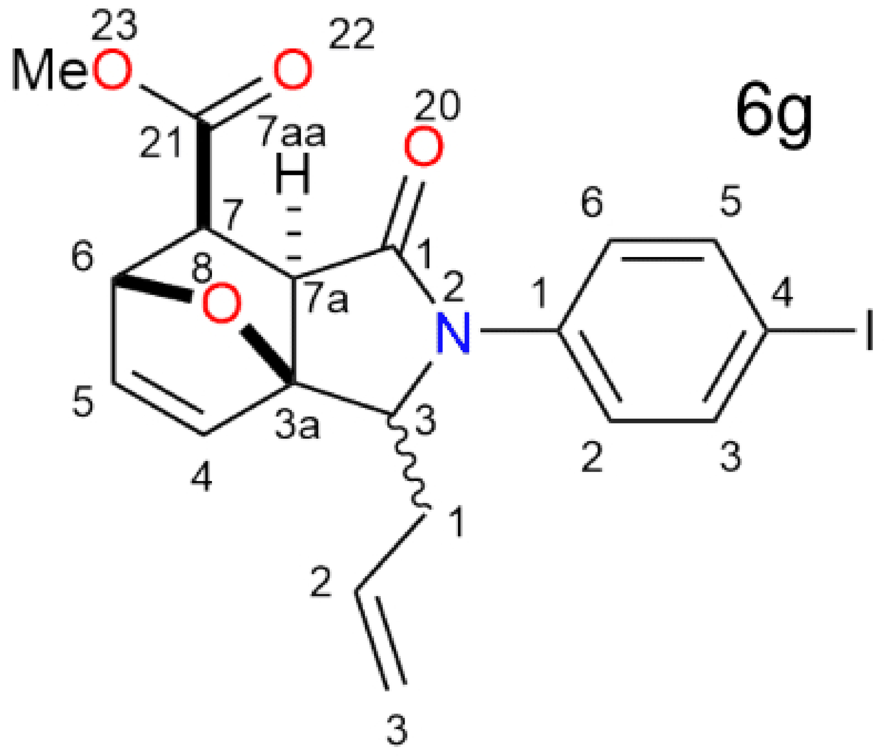

6g

FZ8122-1.jdf

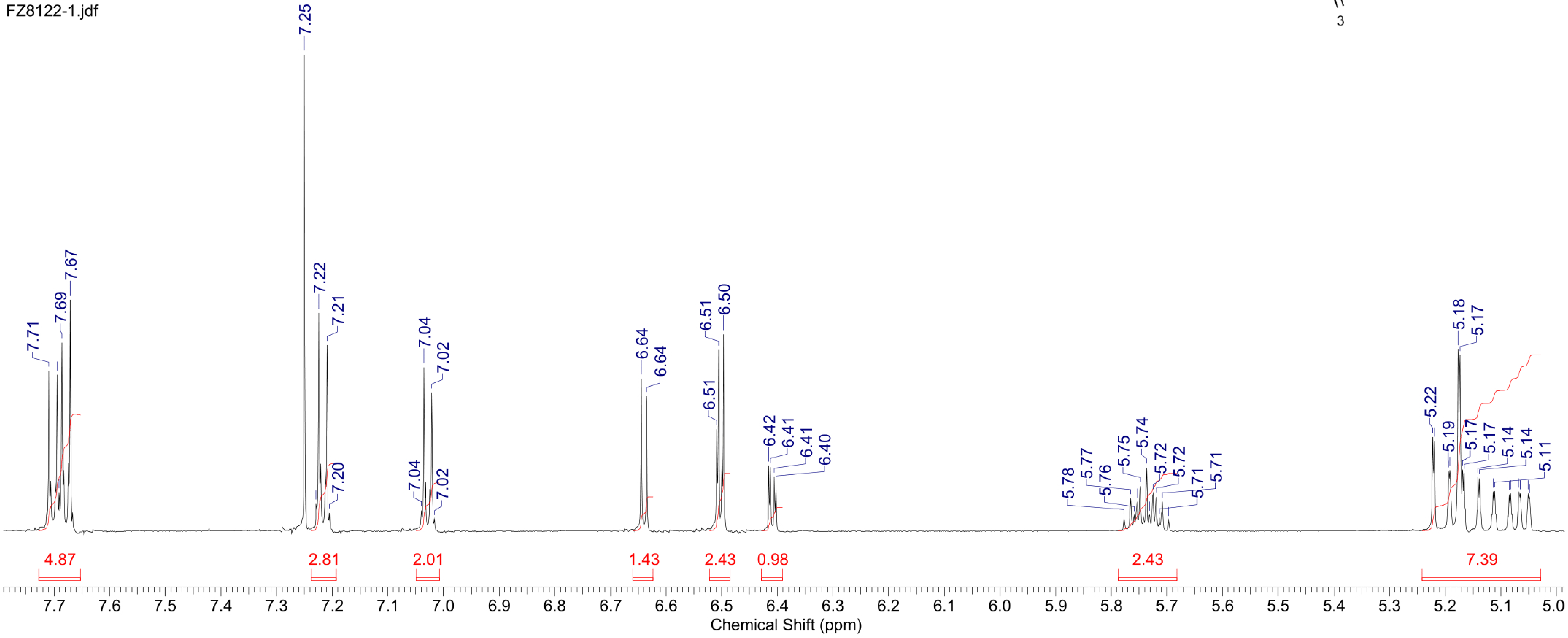

26.11.2019 16:16:59

Formula	$C_{19}H_{18}INO_4$	FW	451.2550

Acquisition Time (sec)	1.9818	Comment	single_pulse	Date	02 Sep 1990 07:15:54				
Date Stamp	12 Nov 2019 12:33:01			File Name	C:\Users\User\Desktop\05.11.19\FZ8122-1.jdf			Frequency (MHz)	600.17
Nucleus	1H	Number of Transients	8	Origin	ECA 600	Original Points Count	32768	Owner	delta
Points Count	32768	Pulse Sequence	single_pulse.ex2			Receiver Gain	44.00	Solvent	CHLOROFORM-d
Spectrum Offset (Hz)	5401.5503	Sweep Width (Hz)	16534.39	Temperature (degree C)	23.000				

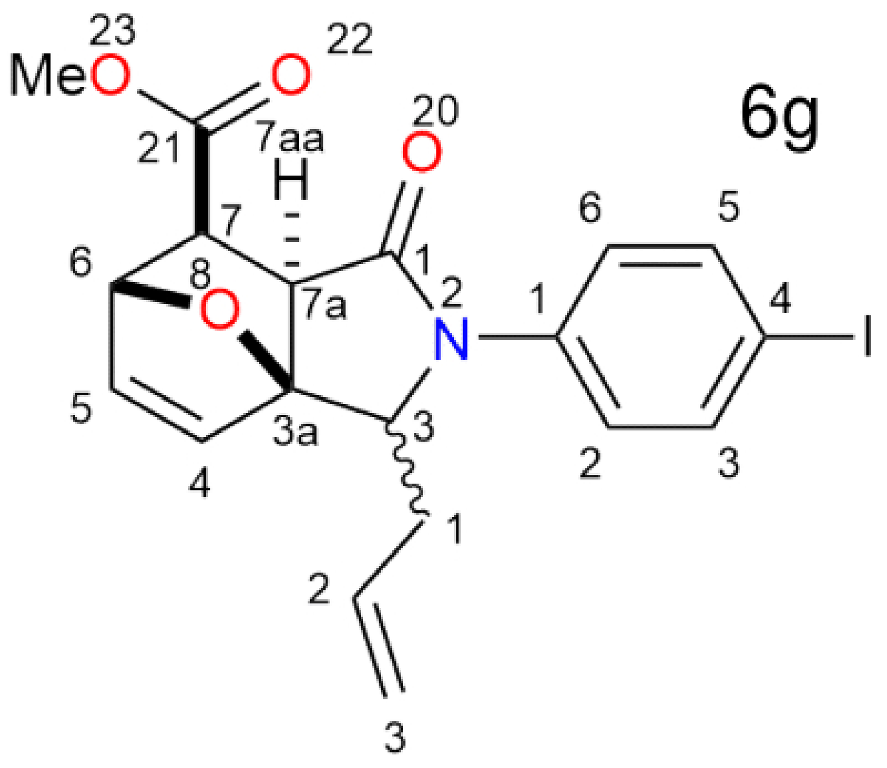

6g

FZ8122-1.jdf

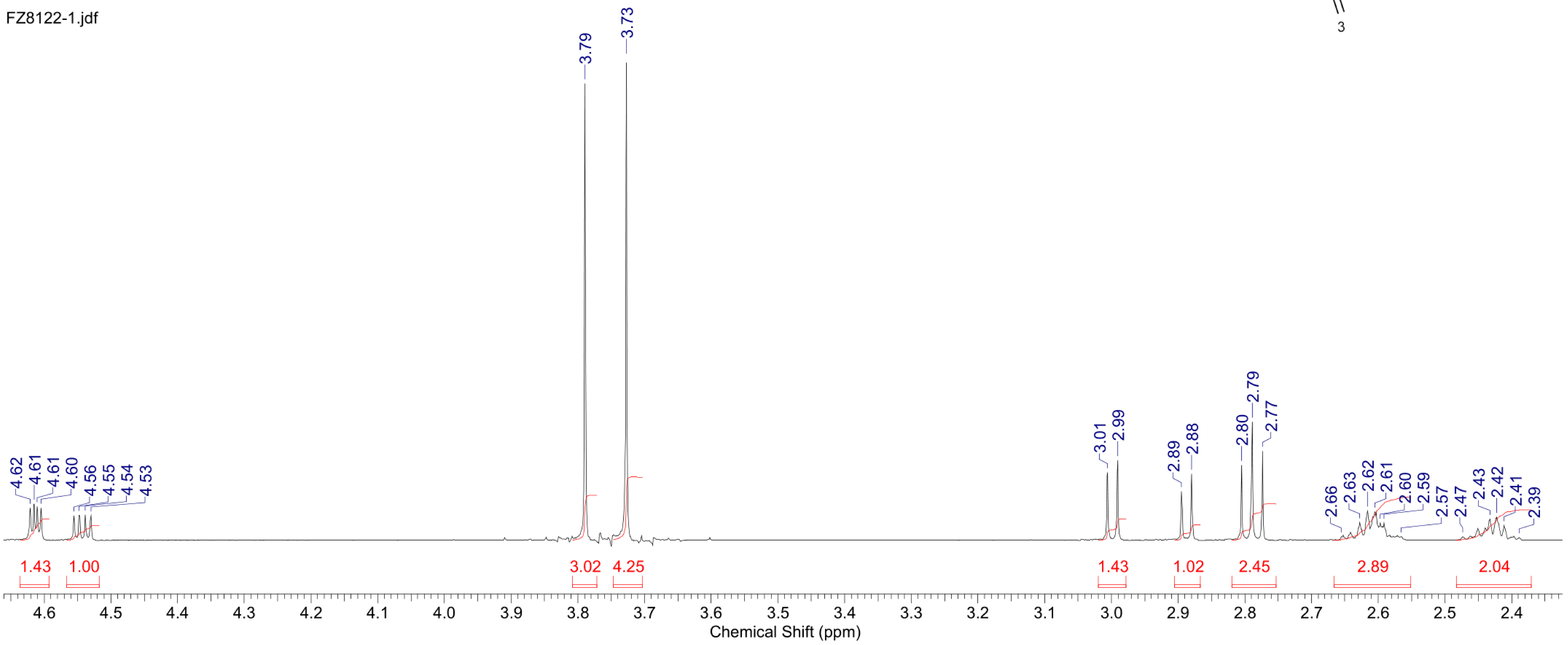

Formula	$C_{19}H_{18}INO_4$	FW	451.2550

Acquisition Time (sec)	0.6921	Comment	single pulse decoupled gated NOE			Date	19 Sep 1990 10:08:59
Date Stamp	29 Nov 2019 15:26:23			File Name	C:\Users\User\Desktop\02.12.19\FZ8205-1.jdf		
Frequency (MHz)	150.91	Nucleus	13C	Number of Transients	1000	Origin	ECA 600
Original Points Count	32768	Owner	delta	Points Count	32768	Pulse Sequence	single_pulse_dec
Receiver Gain	54.00	Solvent	CHLOROFORM-d			Spectrum Offset (Hz)	15077.9072
Sweep Width (Hz)	47348.49	Temperature (degree C)	23.800				

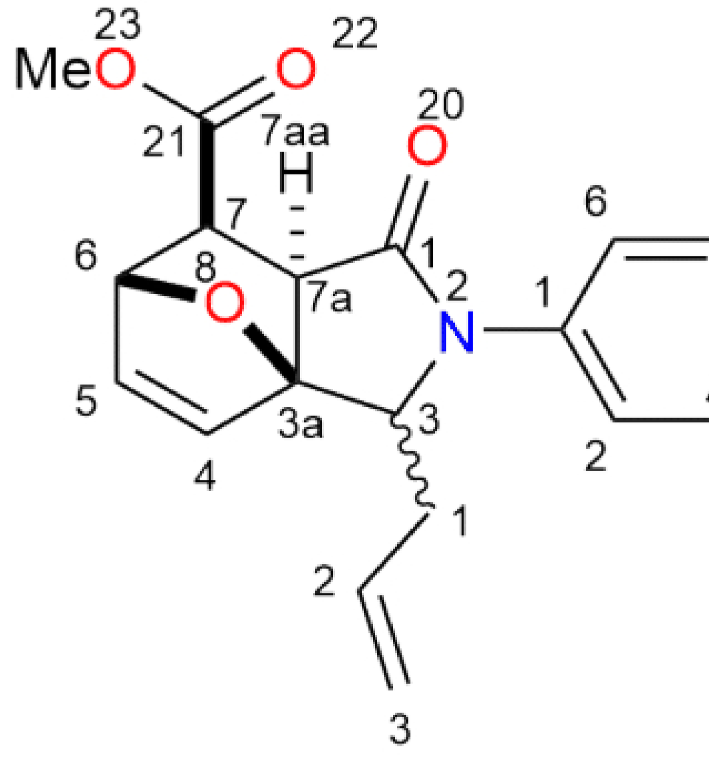

6g

FZ8205-1.esp

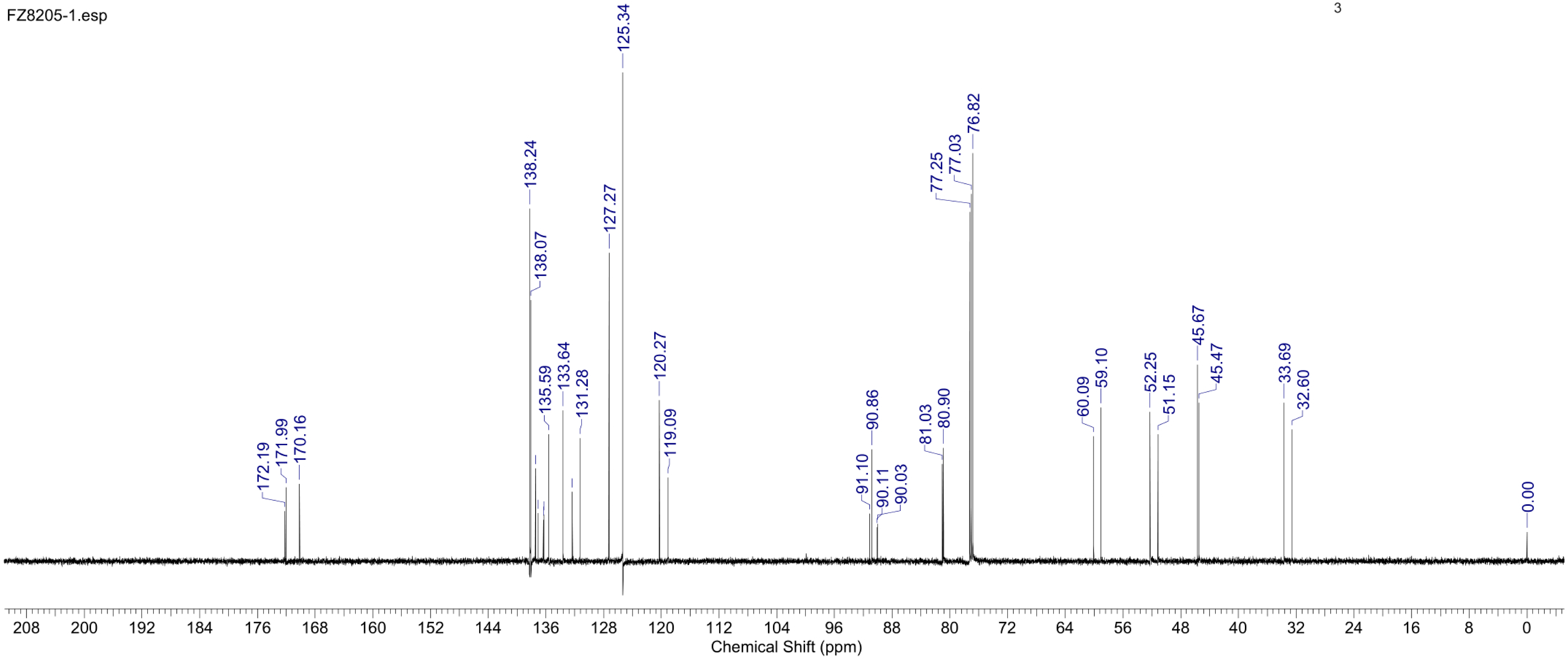

Formula $C_{19}H_{18}INO_4$	FW 451.2550

**Acquisition Time (sec)**	0.6921	**Comment**	single pulse decoupled gated NOE	**Date**	19 Sep 1990 10:08:59
**Date Stamp**	29 Nov 2019 15:26:23	**File Name**	C:\Users\User\Desktop\02.12.19\FZ8205-1.jdf		
**Frequency (MHz)**	150.91	**Nucleus**	13C	**Number of Transients**	1000
**Origin**	ECA 600				
**Original Points Count**	32768	**Owner**	delta	**Points Count**	32768
**Pulse Sequence**	single_pulse_dec				
**Receiver Gain**	54.00	**Solvent**	CHLOROFORM-d	**Spectrum Offset (Hz)**	15077.9072
**Sweep Width (Hz)**	47348.49	**Temperature (degree C)**	23.800		

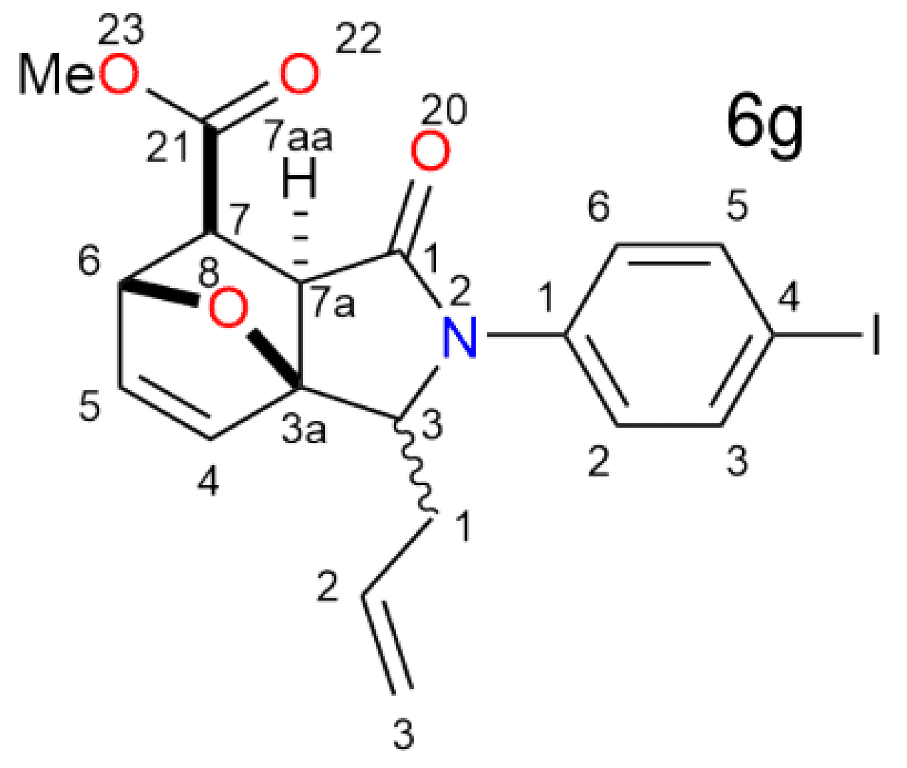

FZ8205-1.esp

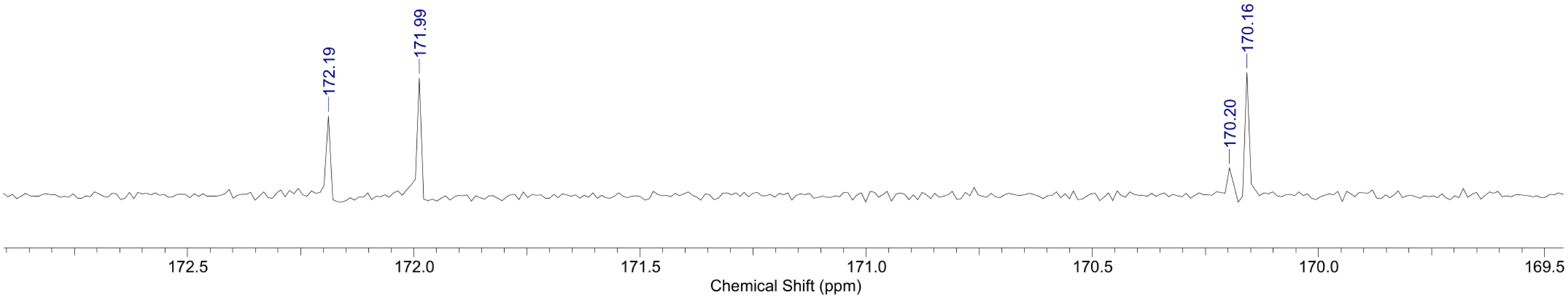

Formula	$C_{19}H_{18}INO_4$	FW	451.2550

**Acquisition Time (sec)**	0.6921	**Comment**	single pulse decoupled gated NOE	**Date**	19 Sep 1990 10:08:59
**Date Stamp**	29 Nov 2019 15:26:23	**File Name**	C:\Users\User\Desktop\02.12.19\FZ8205-1.jdf		
**Frequency (MHz)**	150.91	**Nucleus**	13C	**Number of Transients**	1000
**Origin**	ECA 600	**Original Points Count**	32768	**Owner**	delta
**Points Count**	32768	**Pulse Sequence**	single_pulse_dec	**Receiver Gain**	54.00
**Solvent**	CHLOROFORM-d	**Spectrum Offset (Hz)**	15077.9072	**Sweep Width (Hz)**	47348.49
**Temperature (degree C)**	23.800				

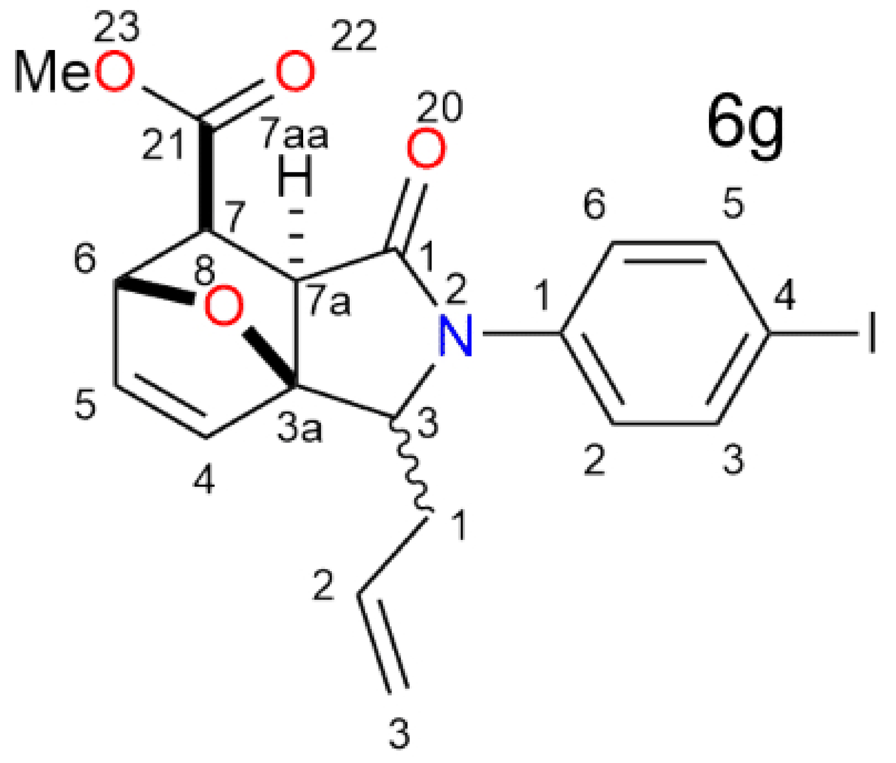

6g

FZ8205-1.esp

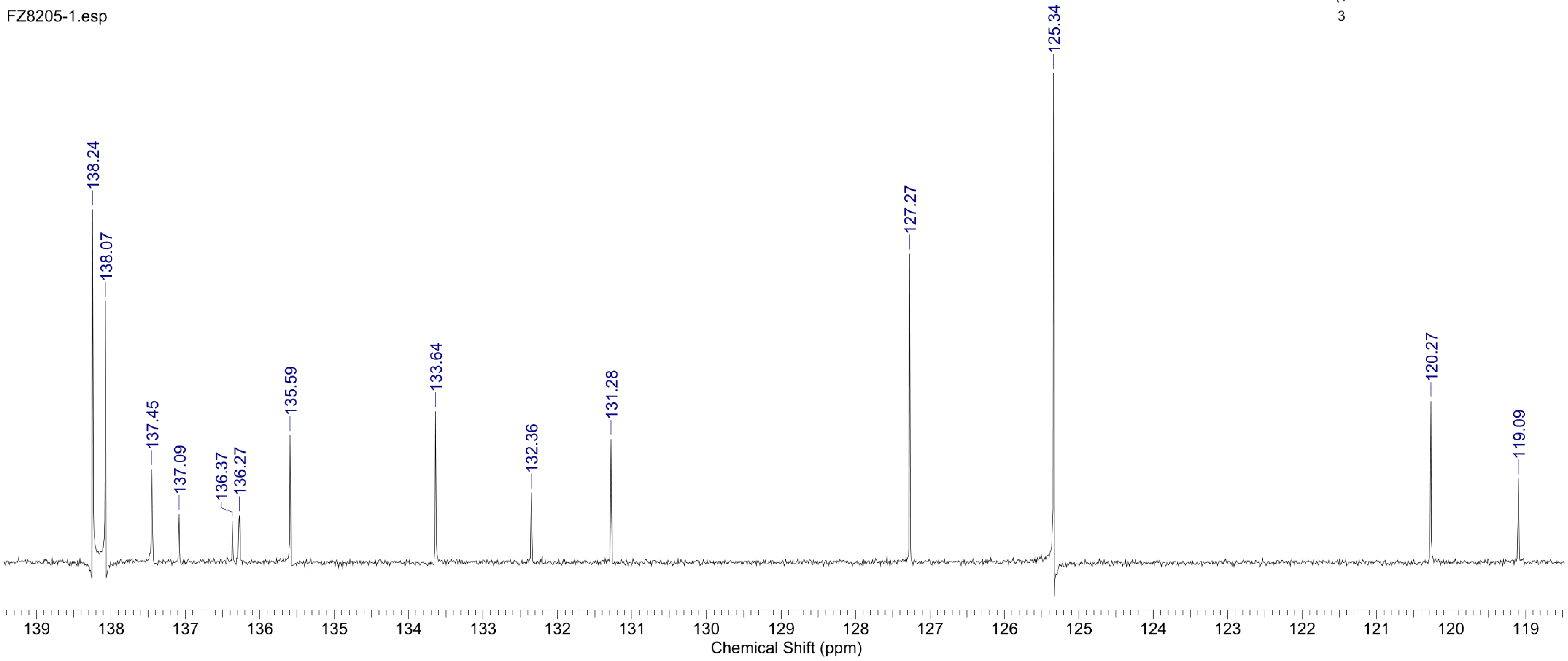

Formula $C_{19}H_{18}INO_4$	FW 451.2550

Acquisition Time (sec)	0.6921	***Comment***	single pulse decoupled gated NOE			***Date***	19 Sep 1990 10:08:59
Date Stamp	29 Nov 2019 15:26:23			***File Name***	C:\Users\User\Desktop\02.12.19\FZ8205-1.jdf		
Frequency (MHz)	150.91	***Nucleus***	13C	***Number of Transients***	1000	***Origin***	ECA 600
Original Points Count	32768	***Owner***	delta	***Points Count***	32768	***Pulse Sequence***	single_pulse_dec
Receiver Gain	54.00	***Solvent***	CHLOROFORM-d			***Spectrum Offset (Hz)***	15077.9072
Sweep Width (Hz)	47348.49	***Temperature (degree C)***	23.800				

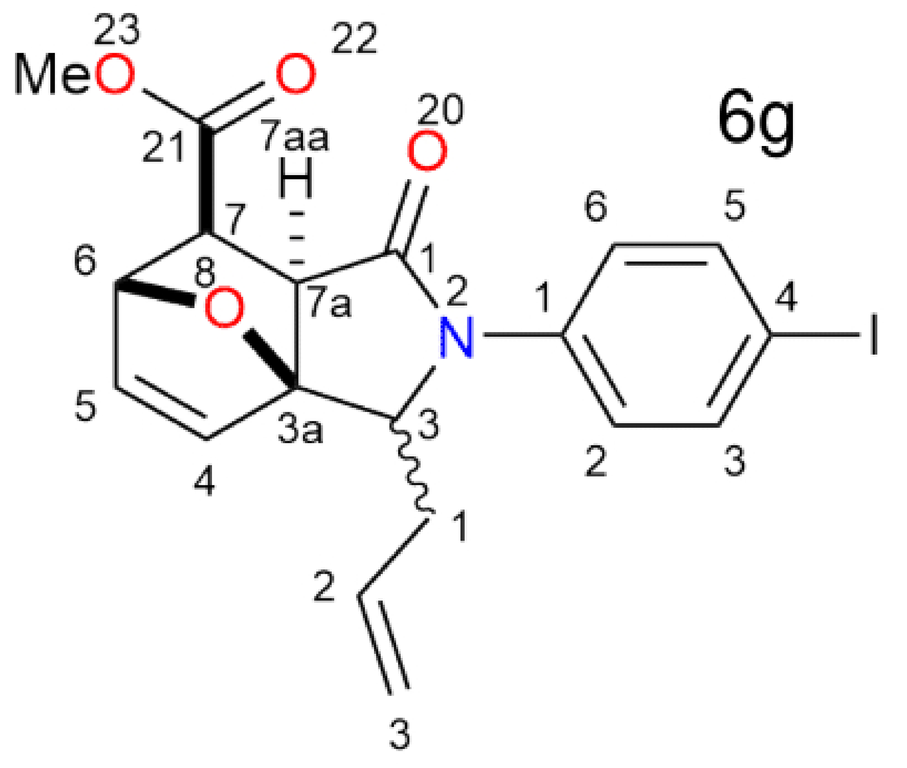

6g

FZ8205-1.esp

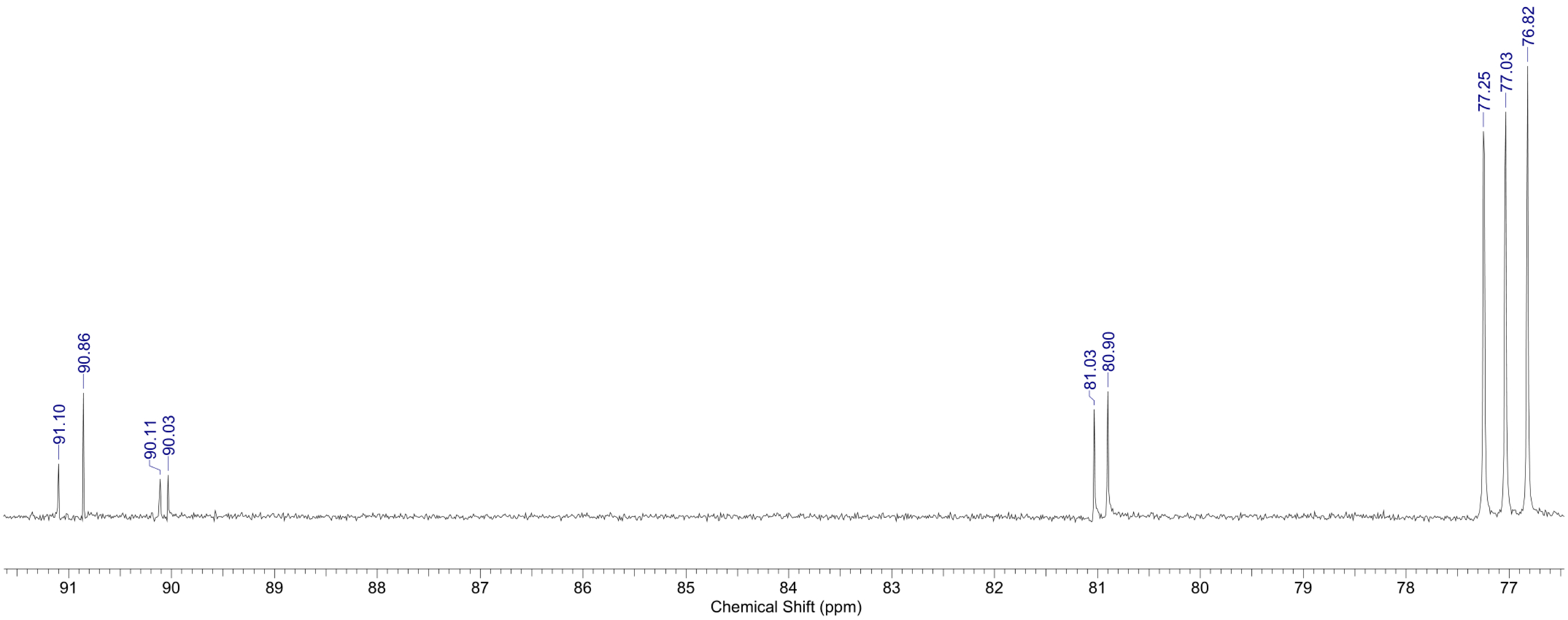

Formula	$C_{19}H_{18}INO_4$	FW	451.2550

Acquisition Time (sec)	0.6921	***Comment***	single pulse decoupled gated NOE			***Date***	19 Sep 1990 10:08:59
Date Stamp	29 Nov 2019 15:26:23			***File Name***	C:\Users\User\Desktop\02.12.19\FZ8205-1.jdf		
Frequency (MHz)	150.91	***Nucleus***	13C	***Number of Transients***	1000	***Origin***	ECA 600
Original Points Count	32768	***Owner***	delta	***Points Count***	32768	***Pulse Sequence***	single_pulse_dec
Receiver Gain	54.00	***Solvent***	CHLOROFORM-d			***Spectrum Offset (Hz)***	15077.9072
Sweep Width (Hz)	47348.49	***Temperature (degree C)***	23.800				

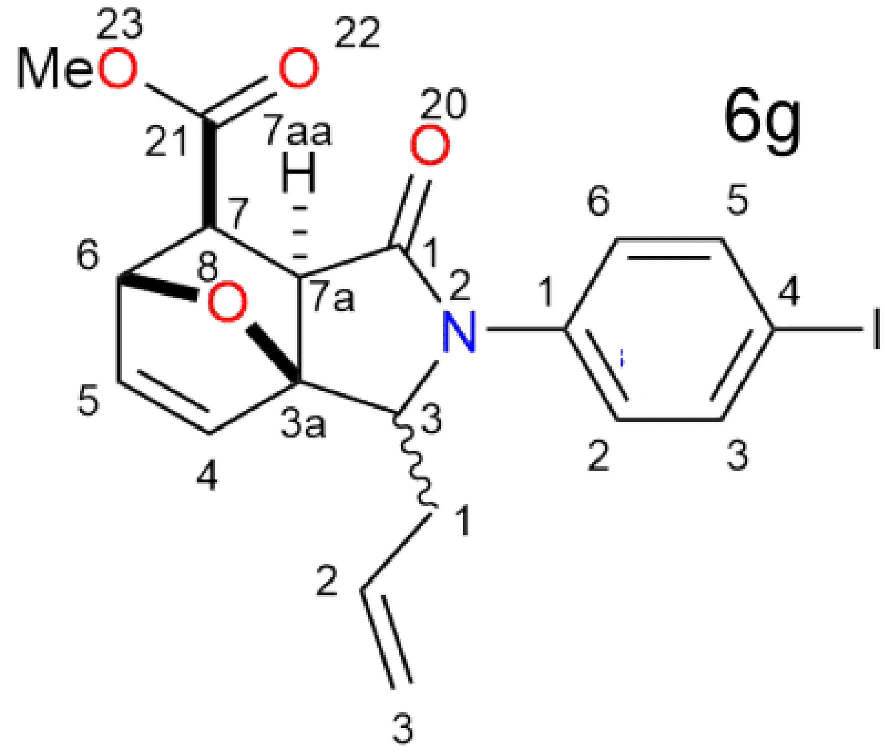

6g

FZ8205-1.esp

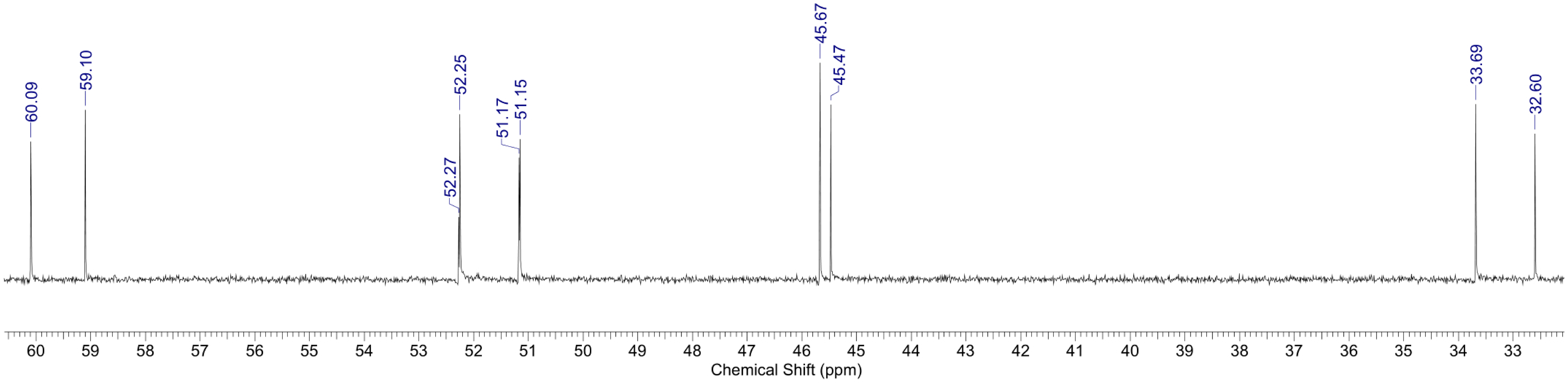

NMR spectrum data for compound 6h

Acquisition Time (sec)	1.9818	***Comment***	single_pulse	***Date***	09 May 1990 04:49:00				
Date Stamp	19 Jul 2019 10:52:40			***File Name***	C:\Users\Fedor\Desktop\18.07.19\FZ7557-1.jdf			***Frequency (MHz)***	600.17
Nucleus	1H	***Number of Transients***	8	***Origin***	ECA 600	***Original Points Count***	32768	***Owner***	delta
Points Count	32768	***Pulse Sequence***	single_pulse.ex2			***Receiver Gain***	36.00	***Solvent***	CHLOROFORM-d
Spectrum Offset (Hz)	5415.1689	***Sweep Width (Hz)***	16534.39	***Temperature (degree C)***	22.900				

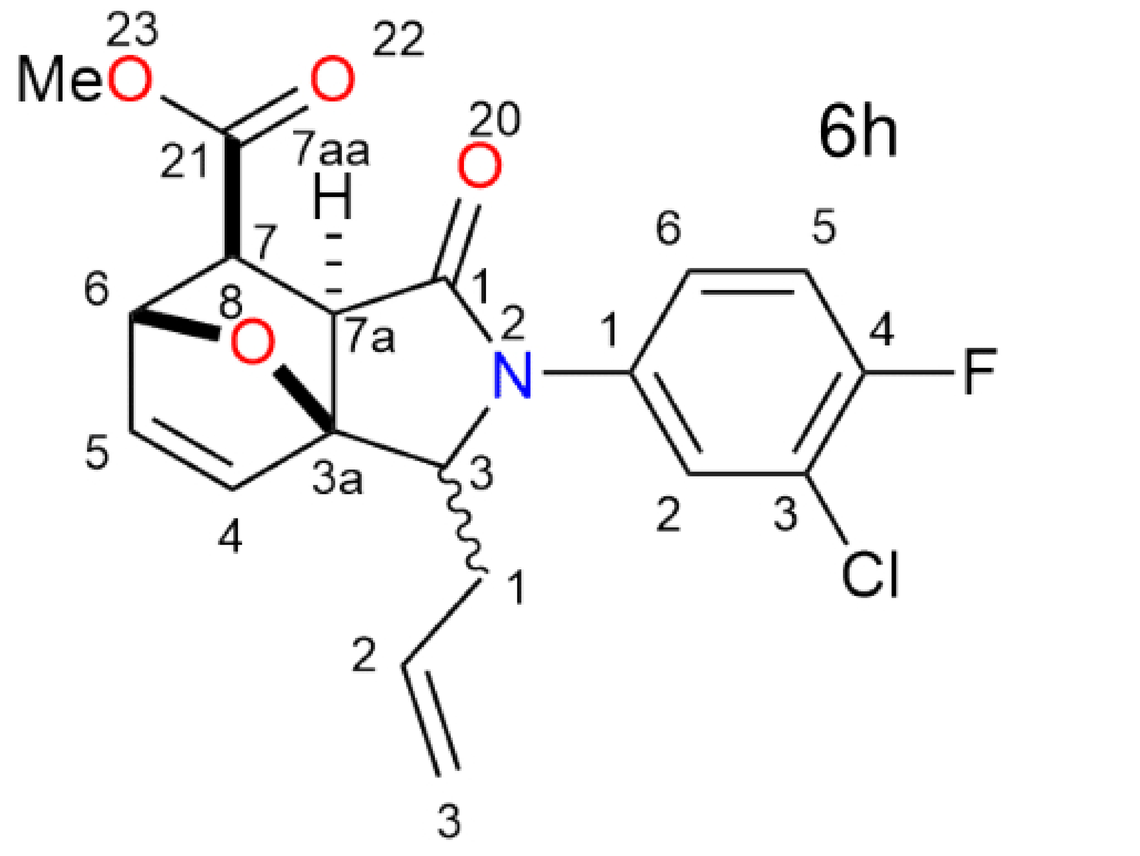

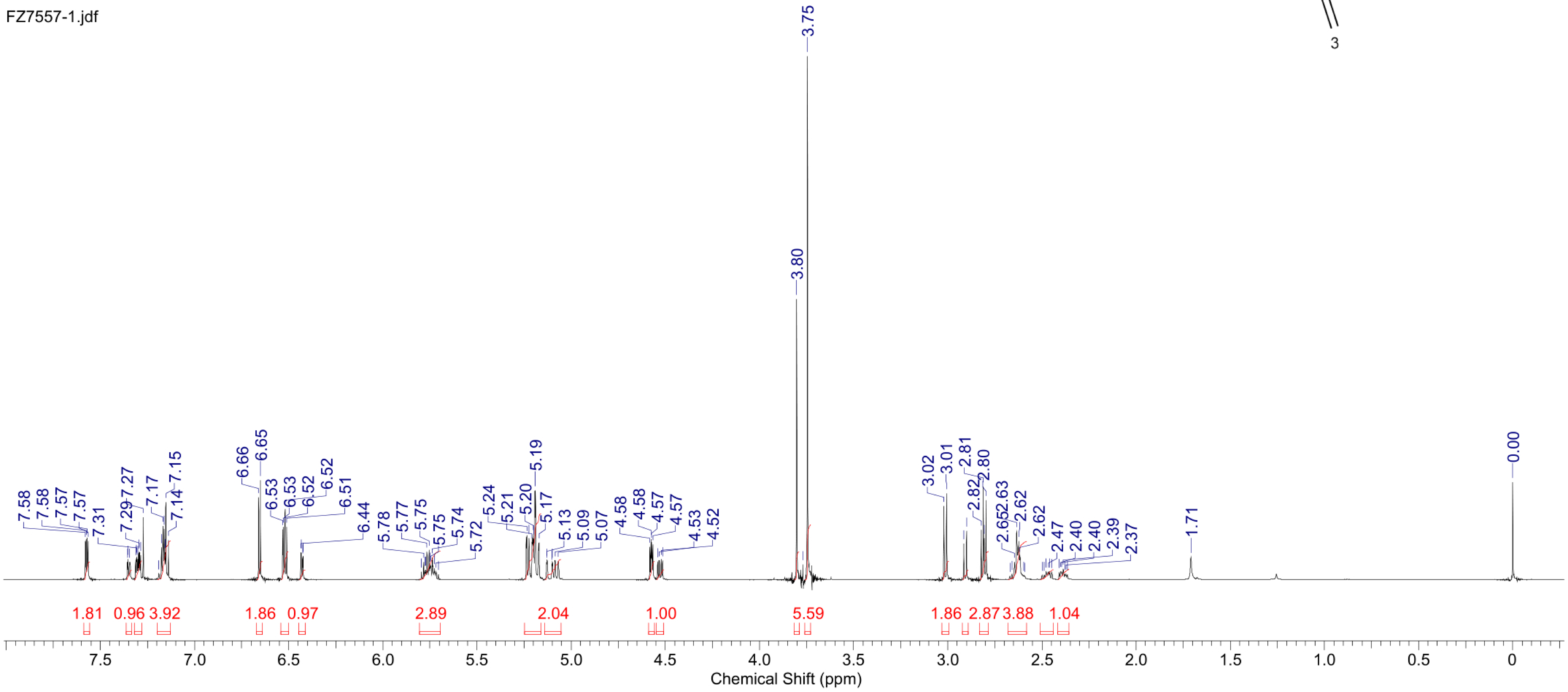

Acquisition Time (sec)	1.9818	Comment	single_pulse	Date	09 May 1990 04:49:00		
Date Stamp	19 Jul 2019 10:52:40			File Name	C:\Users\Fedor\Desktop\18.07.19\FZ7557-1.jdf	Frequency (MHz)	600.17
Nucleus	1H	Number of Transients	8	Origin	ECA 600	Original Points Count	32768	Owner	delta
Points Count	32768	Pulse Sequence	single_pulse.ex2			Receiver Gain	36.00	Solvent	CHLOROFORM-d
Spectrum Offset (Hz)	5415.1689	Sweep Width (Hz)	16534.39	Temperature (degree C)	22.900		

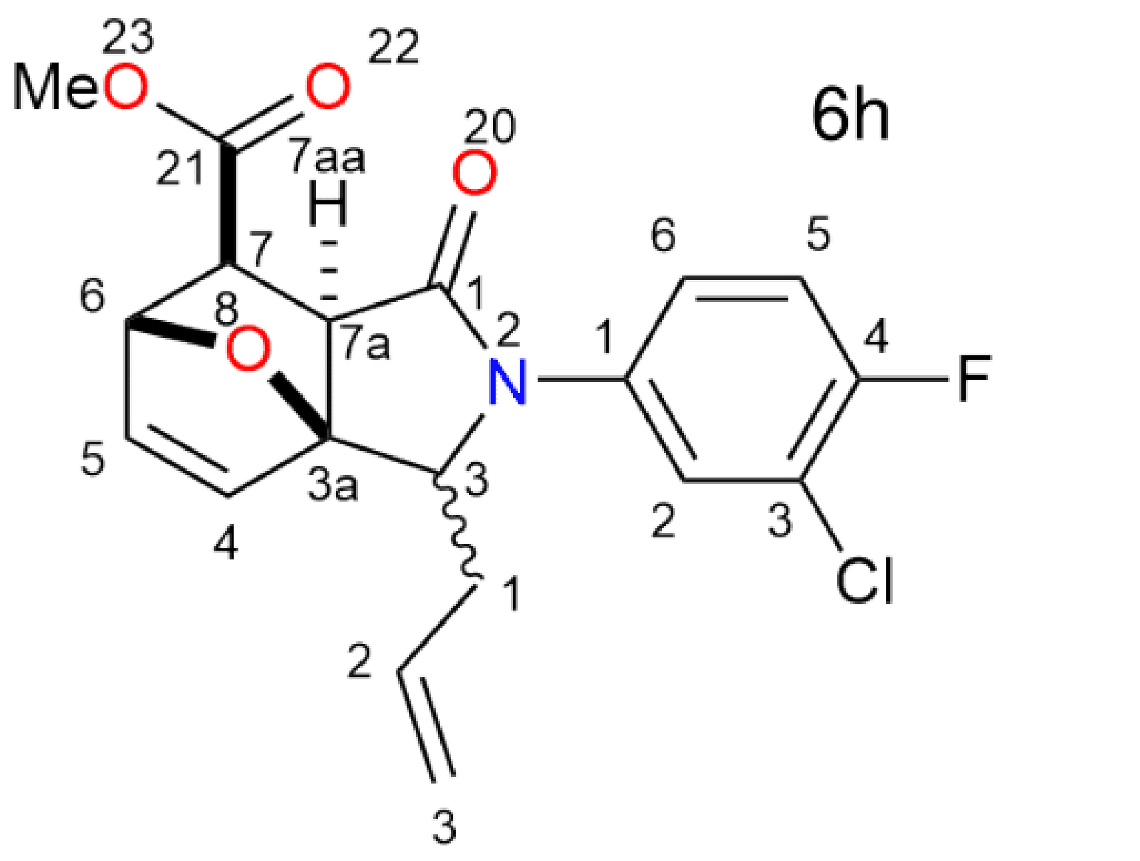

FZ7557-1.jdf

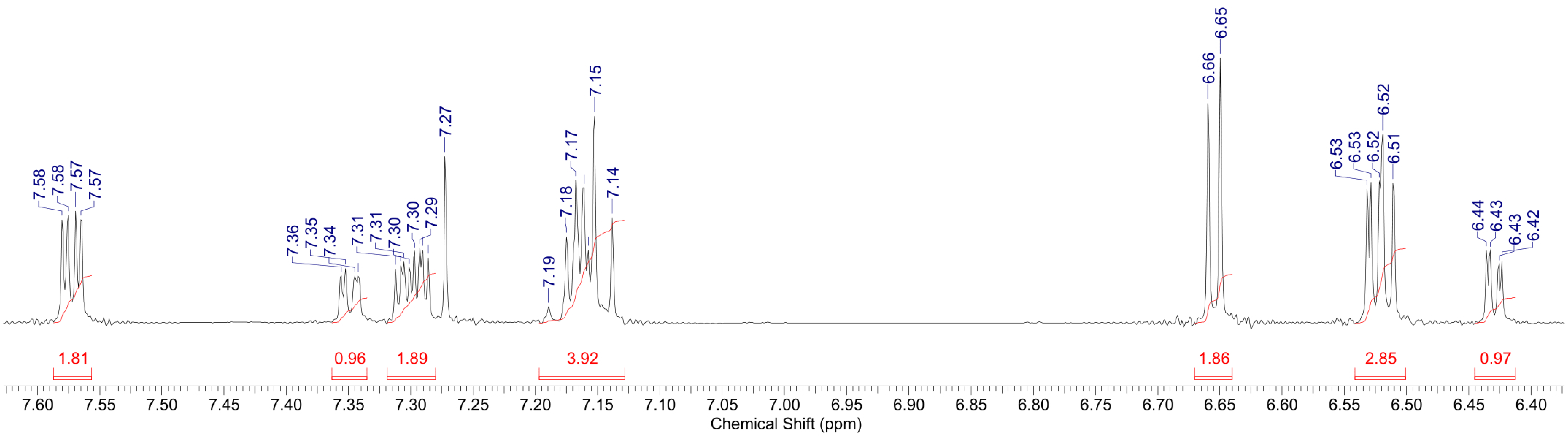

Acquisition Time (sec)	1.9818	***Comment***	single_pulse	***Date***	09 May 1990 04:49:00				
Date Stamp	19 Jul 2019 10:52:40			***File Name***	C:\Users\Fedor\Desktop\18.07.19\FZ7557-1.jdf			***Frequency (MHz)***	600.17
Nucleus	1H	***Number of Transients***	8	***Origin***	ECA 600	***Original Points Count***	32768	***Owner***	delta
Points Count	32768	***Pulse Sequence***	single_pulse.ex2			***Receiver Gain***	36.00	***Solvent***	CHLOROFORM-d
Spectrum Offset (Hz)	5415.1689	***Sweep Width (Hz)***	16534.39	***Temperature (degree C)***	22.900				

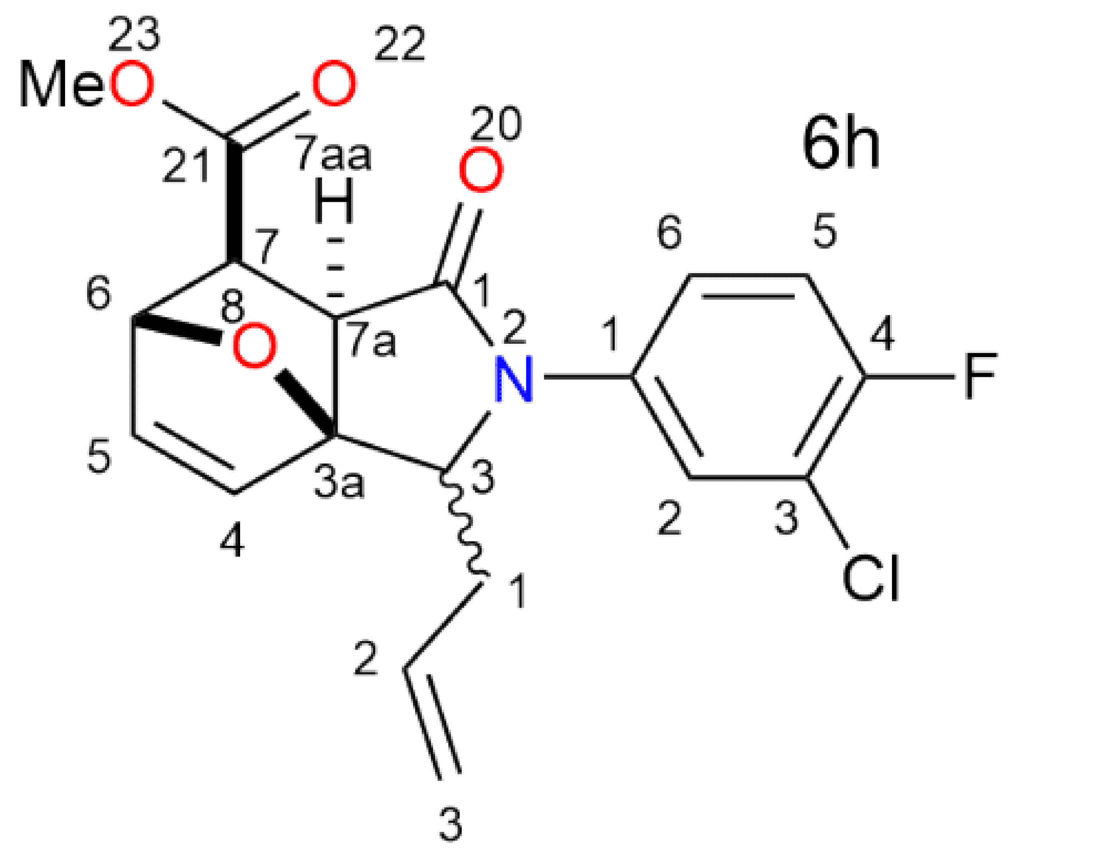

FZ7557-1.jdf

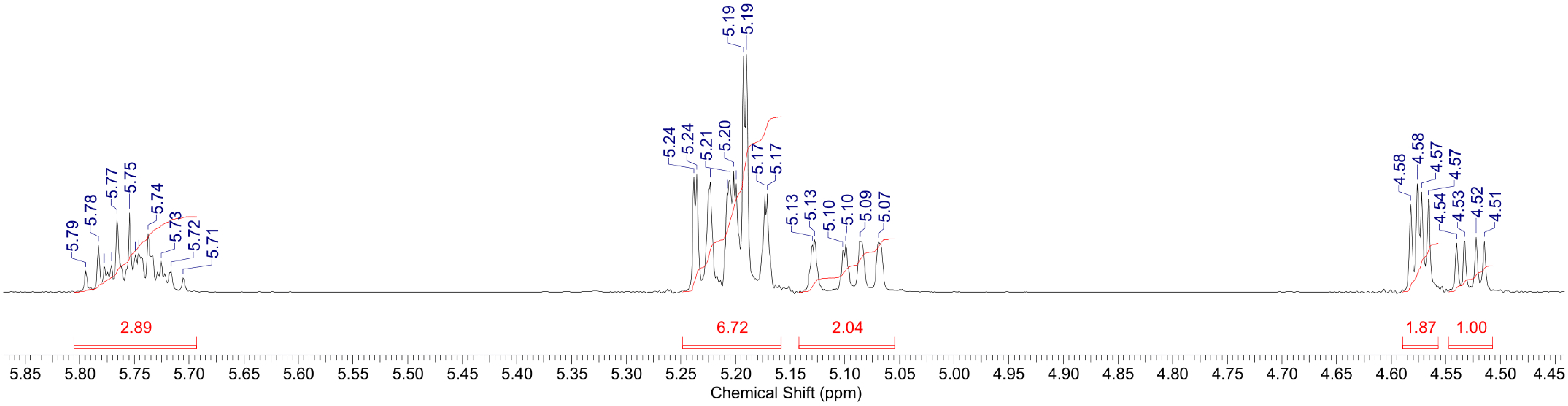

Acquisition Time (sec)	1.9818	***Comment***	single_pulse	***Date***	09 May 1990 04:49:00				
Date Stamp	19 Jul 2019 10:52:40			***File Name***	C:\Users\Fedor\Desktop\18.07.19\FZ7557-1.jdf			***Frequency (MHz)***	600.17
Nucleus	1H	***Number of Transients***	8	***Origin***	ECA 600	***Original Points Count***	32768	***Owner***	delta
Points Count	32768	***Pulse Sequence***	single_pulse.ex2			***Receiver Gain***	36.00	***Solvent***	CHLOROFORM-d
Spectrum Offset (Hz)	5415.1689	***Sweep Width (Hz)***	16534.39	***Temperature (degree C)***	22.900				

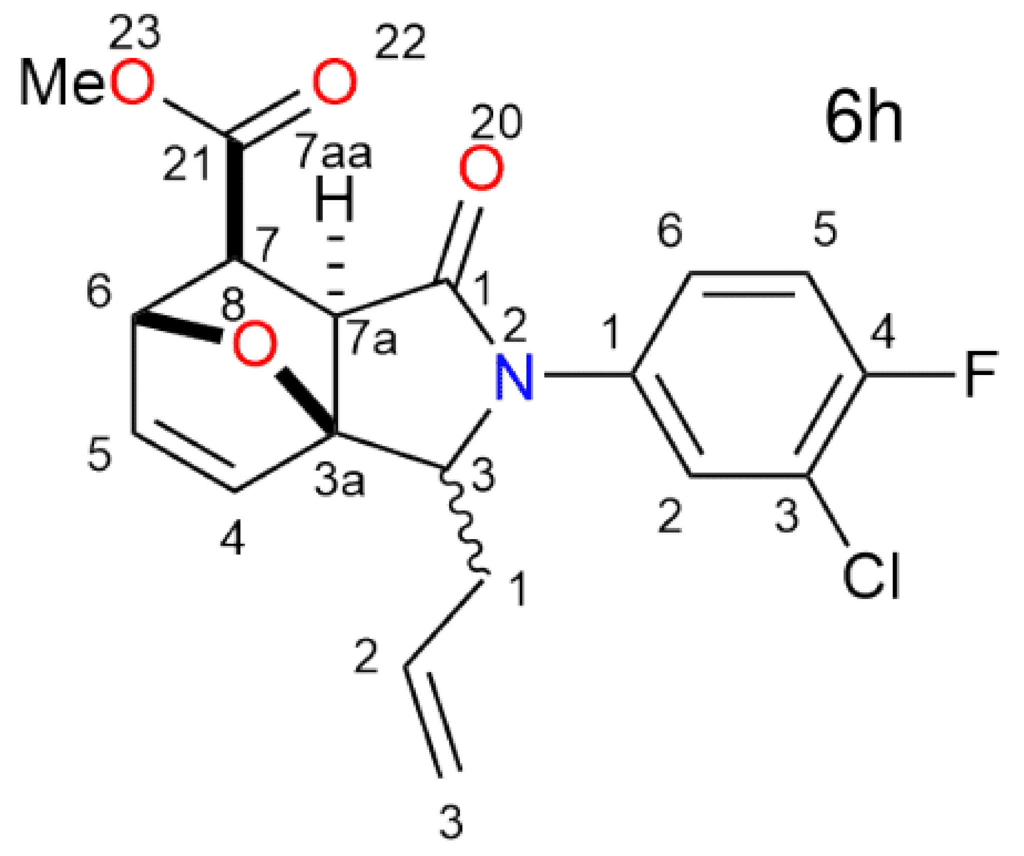

6h

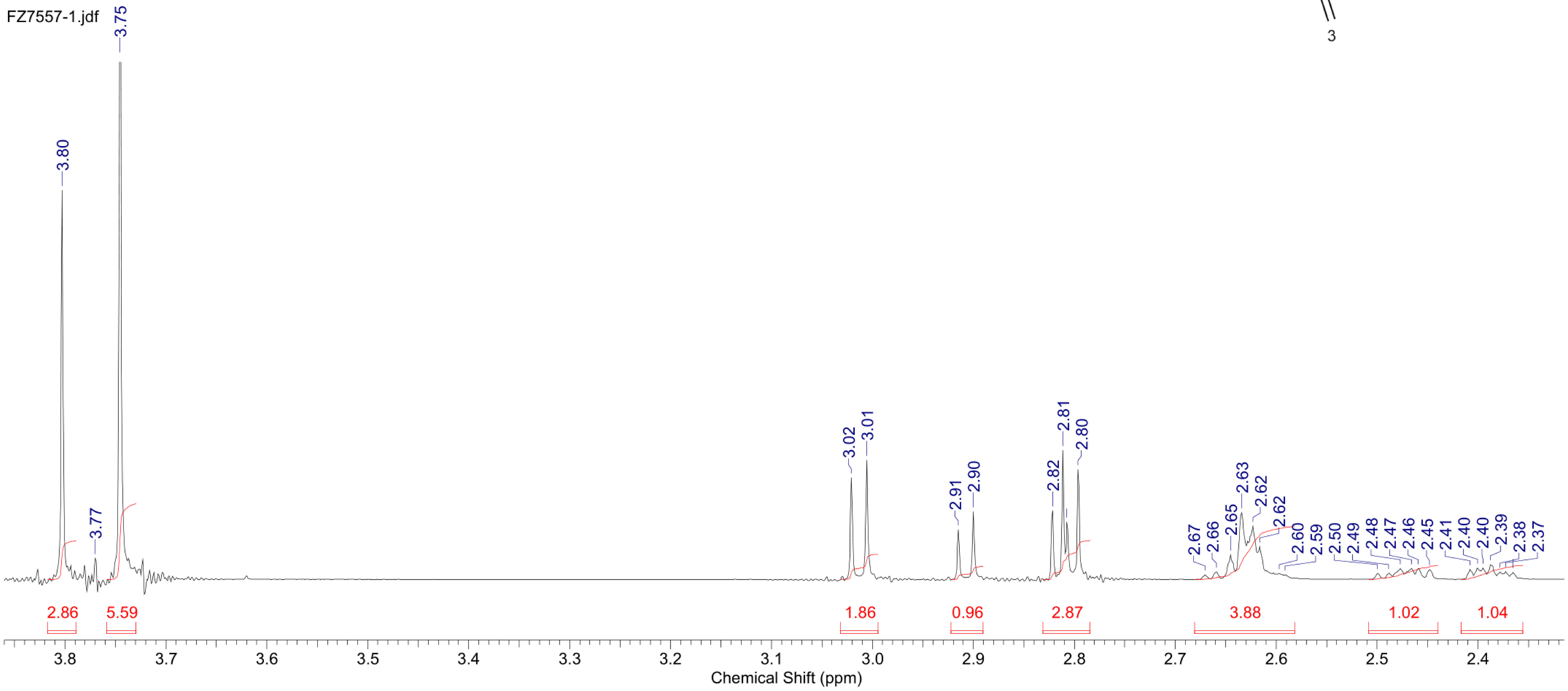

14.07.2020 18:40:05

**Formula** $C_{20}H_{19}ClFNO_4$	**FW** 391.8206

Acquisition Time (sec)	0.6921	***Comment***	single pulse decoupled gated NOE	***Date***	12 Aug 1990 04:44:21
Date Stamp	22 Oct 2019 10:47:29	***File Name***	C:\USERS\Лаба534\DOWNLOADS\FZ8001-1.JDF		
Frequency (MHz)	150.91	***Nucleus***	13C	***Number of Transients***	800
Origin	ECA 600				
Original Points Count	32768	***Owner***	delta	***Points Count***	32768
Pulse Sequence	single_pulse_dec				
Receiver Gain	54.00	***Solvent***	CHLOROFORM-d	***Spectrum Offset (Hz)***	15080.7979
Sweep Width (Hz)	47348.49	***Temperature (degree C)***	24.300		

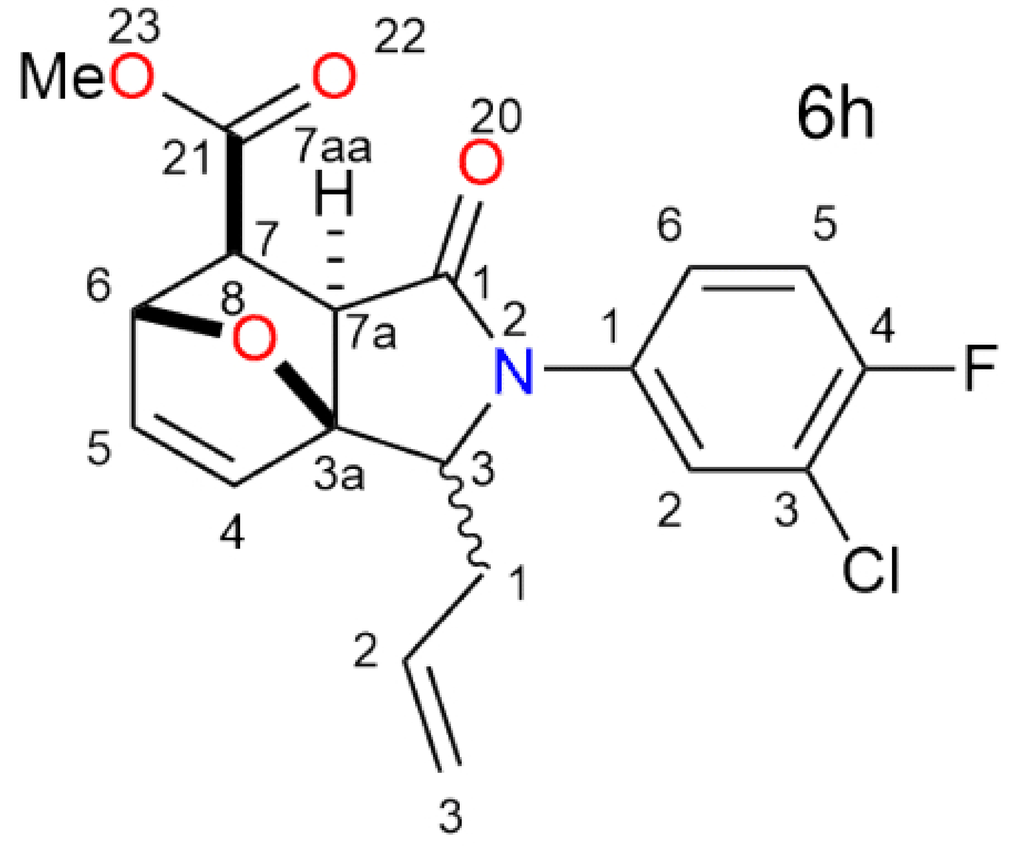

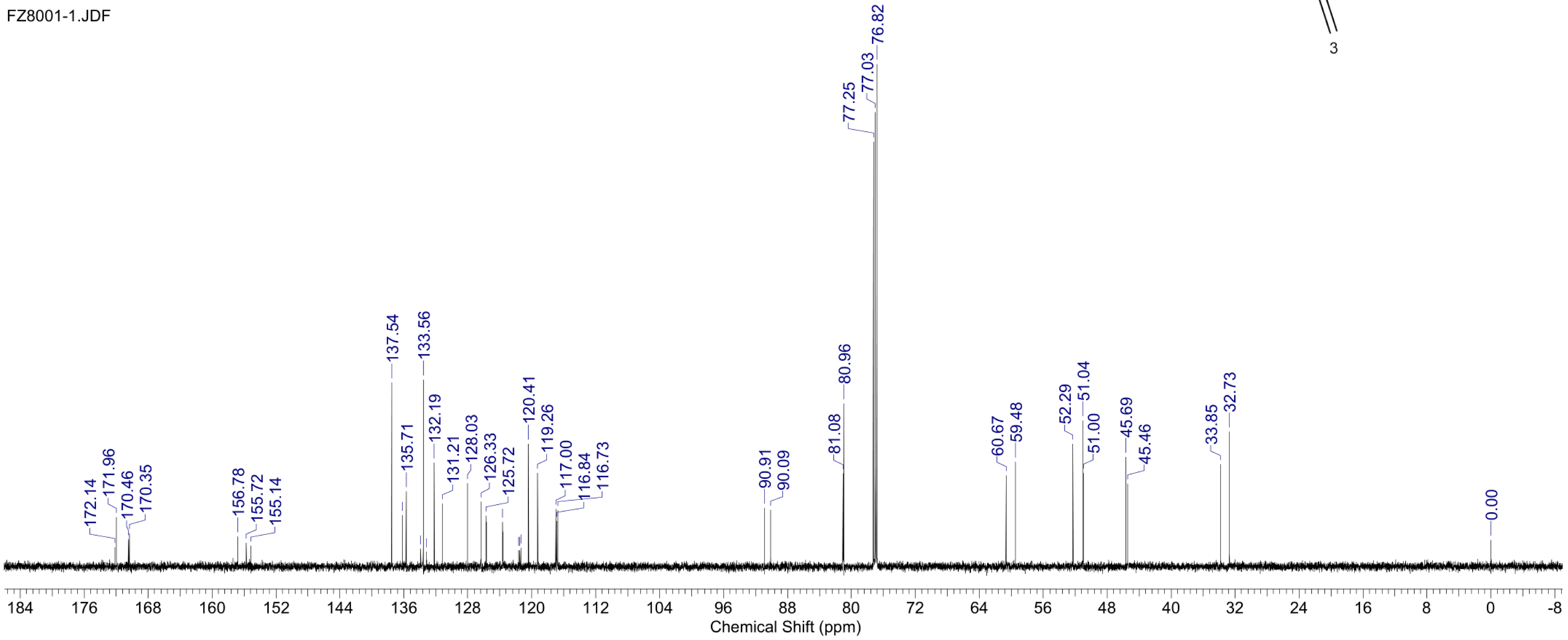

14.07.2020 18:40:13

**Formula**	$C_{20}H_{19}ClFNO_4$	**FW**	391.8206

**Acquisition Time (sec)**	0.6921	**Comment**	single pulse decoupled gated NOE			**Date**	12 Aug 1990 04:44:21
**Date Stamp**	22 Oct 2019 10:47:29			**File Name**	C:\USERS\Лаба534\DOWNLOADS\FZ8001-1.JDF		
**Frequency (MHz)**	150.91	**Nucleus**	13C	**Number of Transients**	800	**Origin**	ECA 600
**Original Points Count**	32768	**Owner**	delta	**Points Count**	32768	**Pulse Sequence**	single_pulse_dec
**Receiver Gain**	54.00	**Solvent**	CHLOROFORM-d			**Spectrum Offset (Hz)**	15080.7979
**Sweep Width (Hz)**	47348.49	**Temperature (degree C)**	24.300				

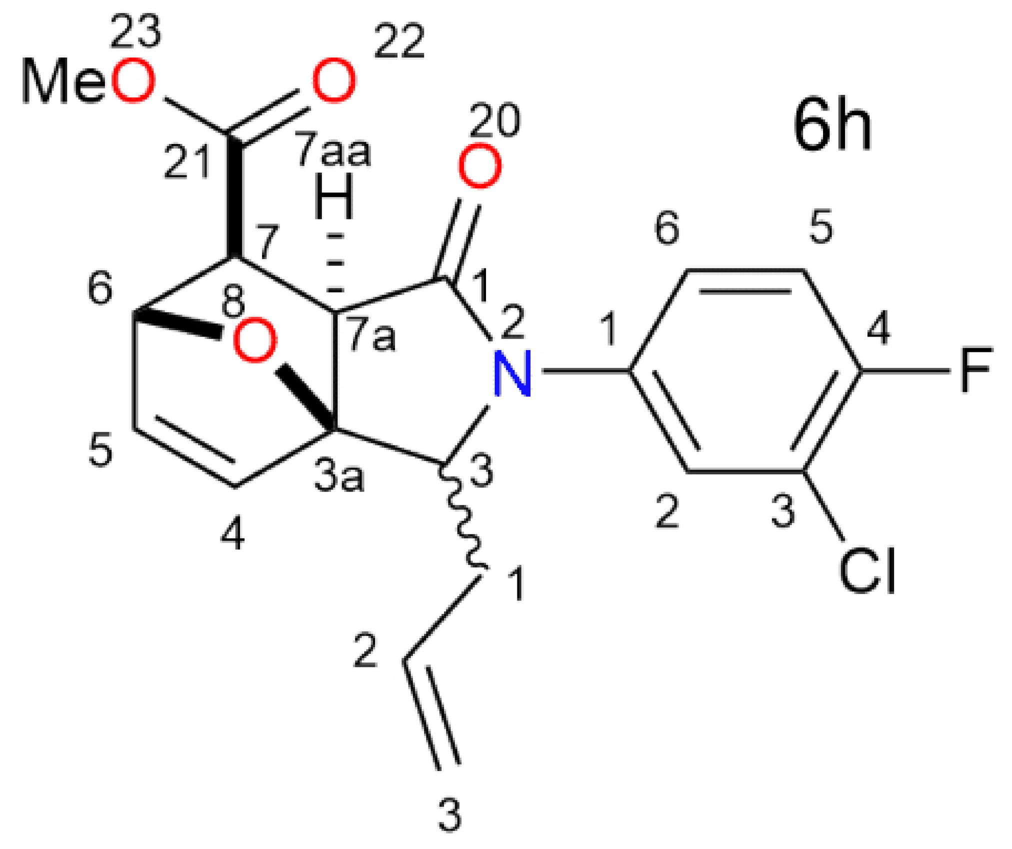

6h

FZ8001-1.JDF

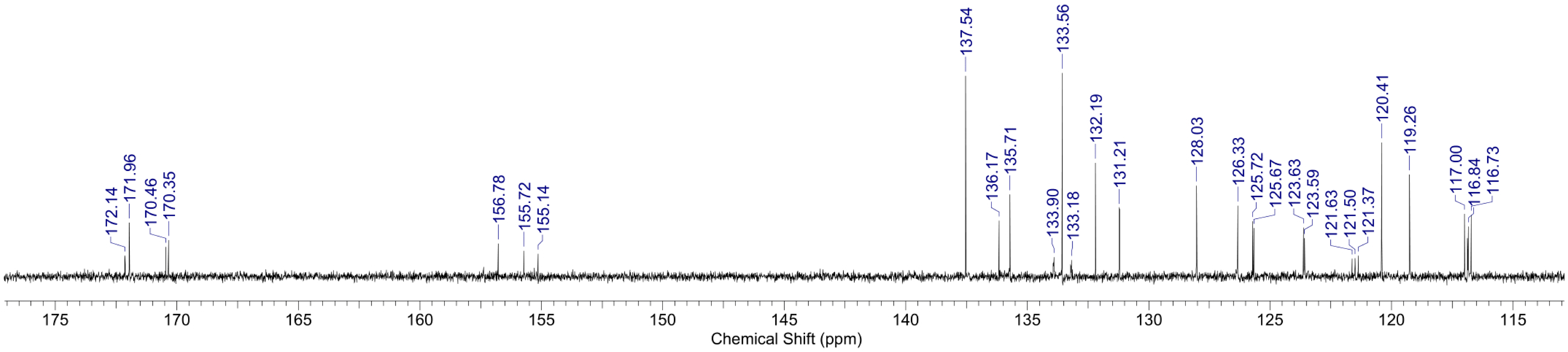

14.07.2020 18:40:22

Formula $C_{20}H_{19}ClFNO_4$	FW 391.8206

**Acquisition Time (sec)**	0.6921	**Comment**	single pulse decoupled gated NOE			**Date**	12 Aug 1990 04:44:21
**Date Stamp**	22 Oct 2019 10:47:29			**File Name**	C:\USERS\Лаба534\DOWNLOADS\FZ8001-1.JDF		
**Frequency (MHz)**	150.91	**Nucleus**	13C	**Number of Transients**	800	**Origin**	ECA 600
**Original Points Count**	32768	**Owner**	delta	**Points Count**	32768	**Pulse Sequence**	single_pulse_dec
**Receiver Gain**	54.00	**Solvent**	CHLOROFORM-d			**Spectrum Offset (Hz)**	15080.7979
**Sweep Width (Hz)**	47348.49	**Temperature (degree C)**	24.300				

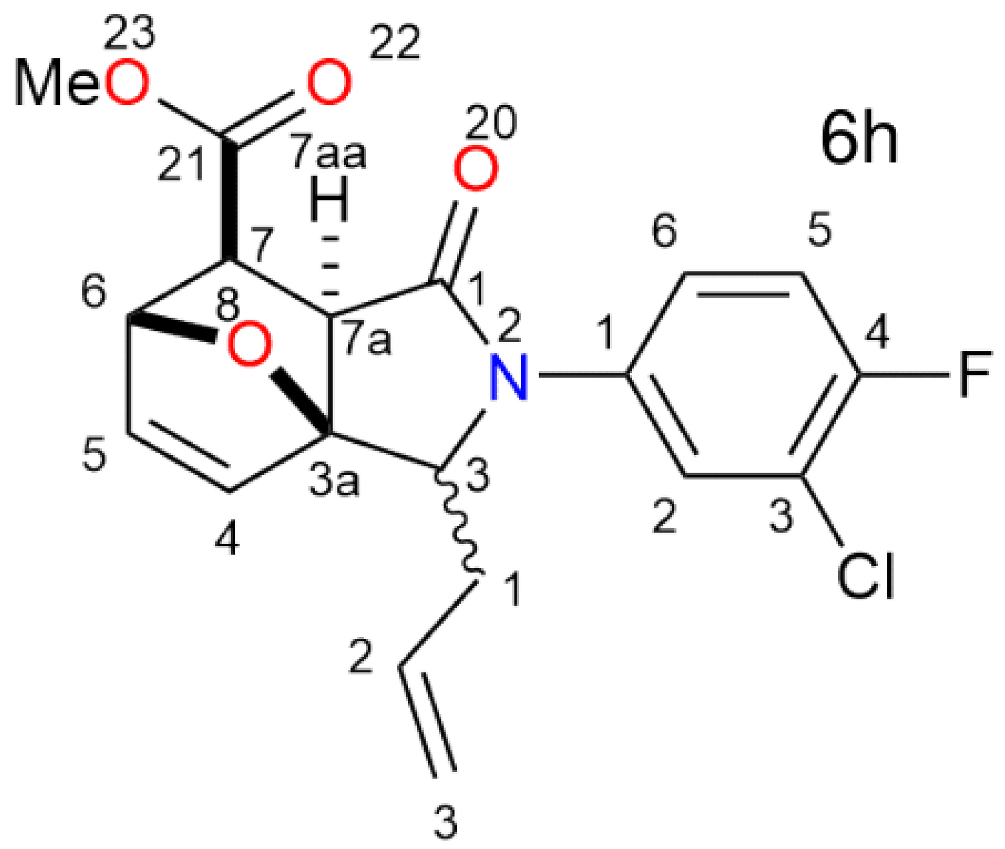

FZ8001-1.JDF

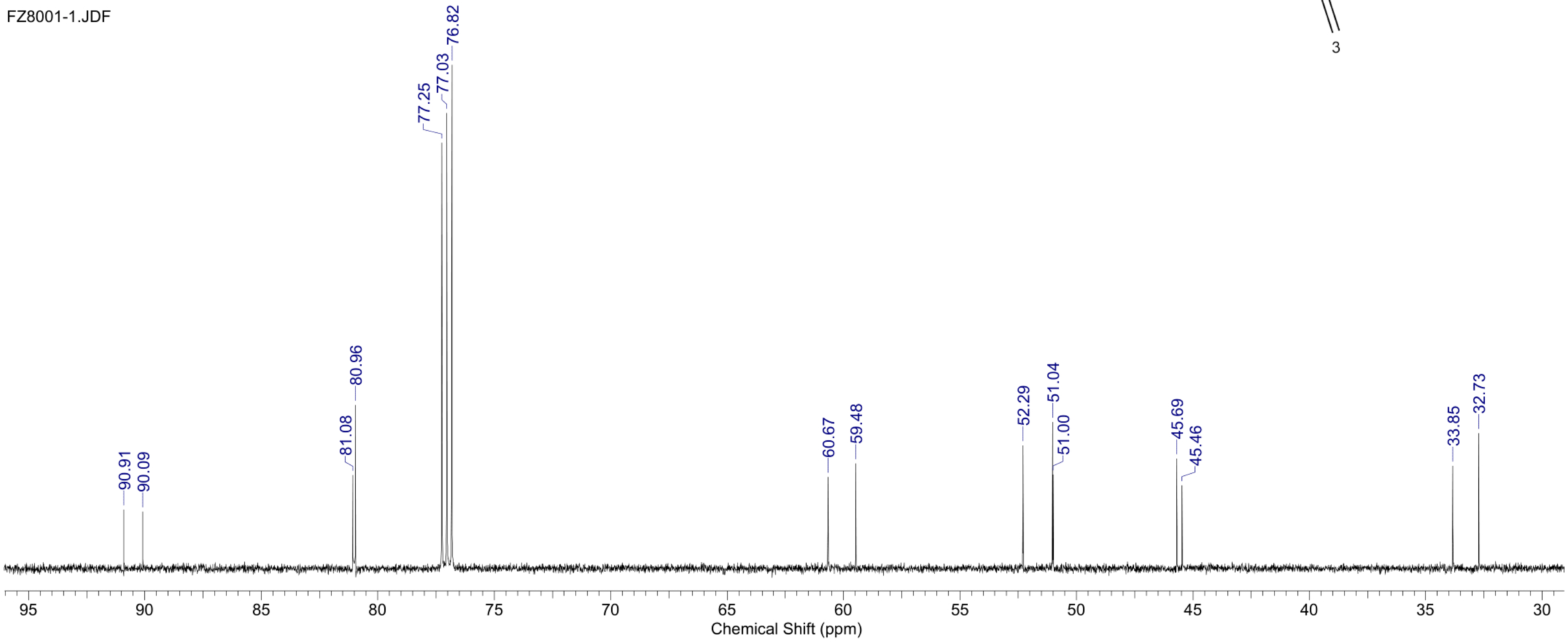

Acquisition Time (sec)	0.4614	Comment	single_pulse	Date	06 Mar 2020 11:36:45			Date Stamp	06 Mar 2020 10:56:39
File Name	E:\FZ8560-1.JDF			Frequency (MHz)	564.73	Nucleus	19F	Number of Transients	8
Origin	ECA 600	Original Points Count	65536	Owner	CKP	Points Count	65536	Pulse Sequence	single_pulse.ex2
Receiver Gain	46.00	Solvent	CHLOROFORM-d			Spectrum Offset (Hz)	-56472.6094		
Sweep Width (Hz)	142045.45	Temperature (degree C)	23.400						

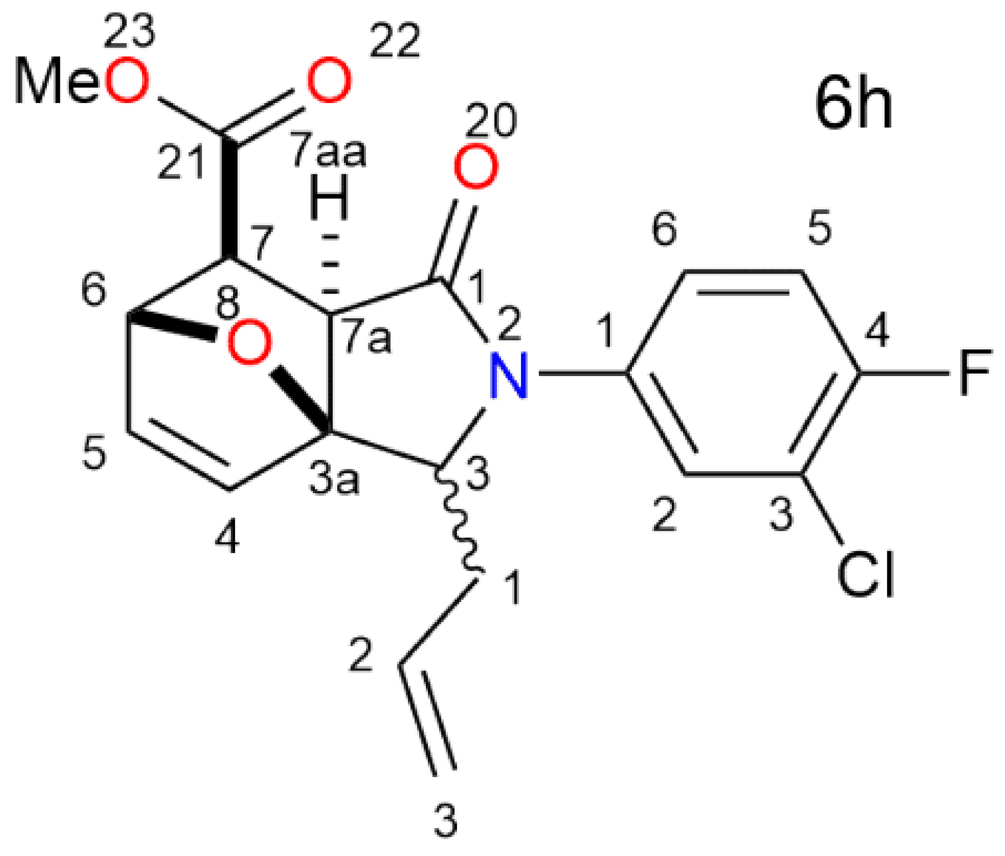

6h

FZ8560-1.JDF

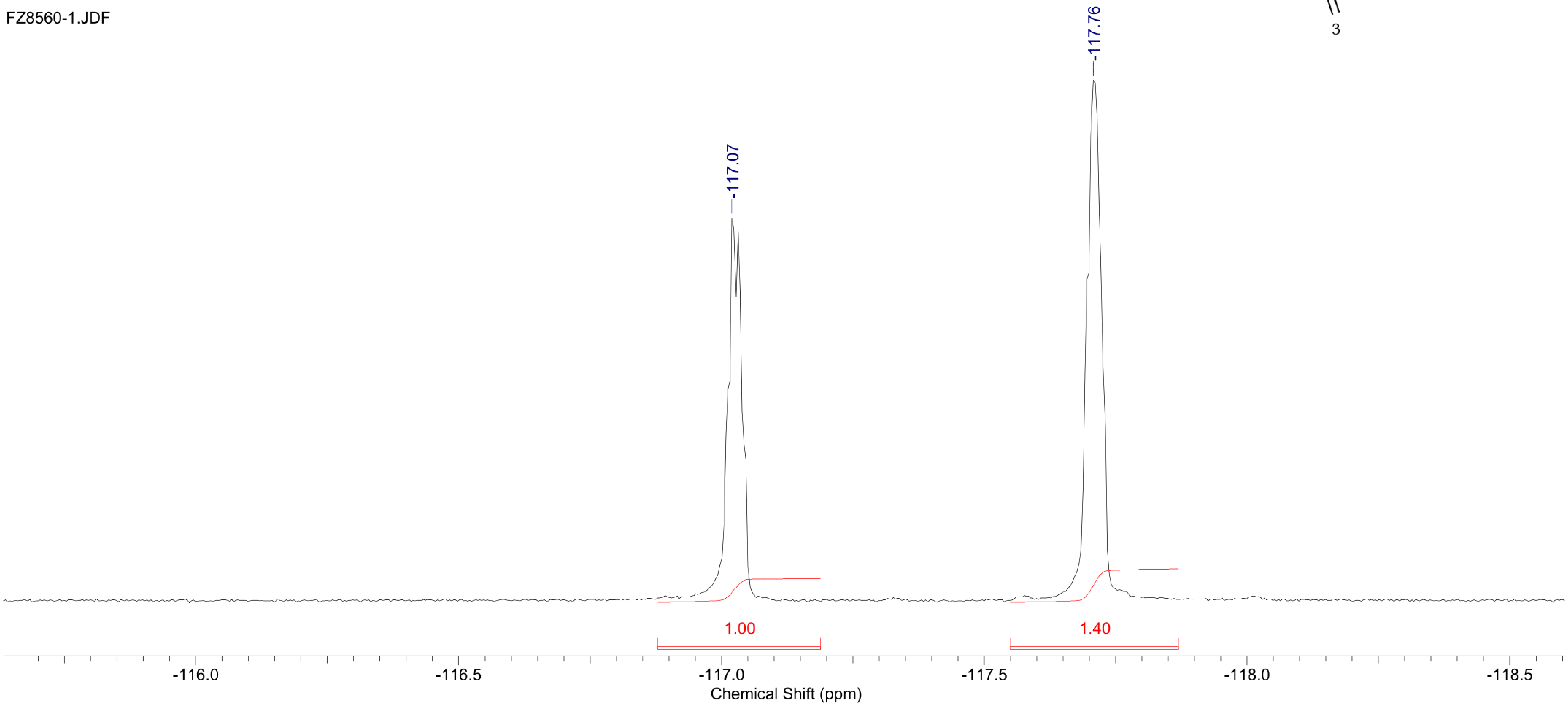

NMR spectrum data for compound 6i

Formula	$C_{21}H_{23}NO_4$	***FW***	353.4116

Acquisition Time (sec)	1.9818	***Comment***	single_pulse	***Date***	30 Jan 2020 09:25:14		
Date Stamp	30 Jan 2020 08:46:45			***File Name***	C:\USERS\лаба 534\DOWNLOADS\FZ8365-1.JDF		
Frequency (MHz)	600.17	***Nucleus***	1H	***Number of Transients***	8	***Origin***	ECA 600
Original Points Count	32768	***Owner***	Mass	***Points Count***	32768	***Pulse Sequence***	single_pulse.ex2
Receiver Gain	32.00	***Solvent***	CHLOROFORM-d			***Spectrum Offset (Hz)***	5413.1504
Sweep Width (Hz)	16534.39	***Temperature (degree C)***	24.400				

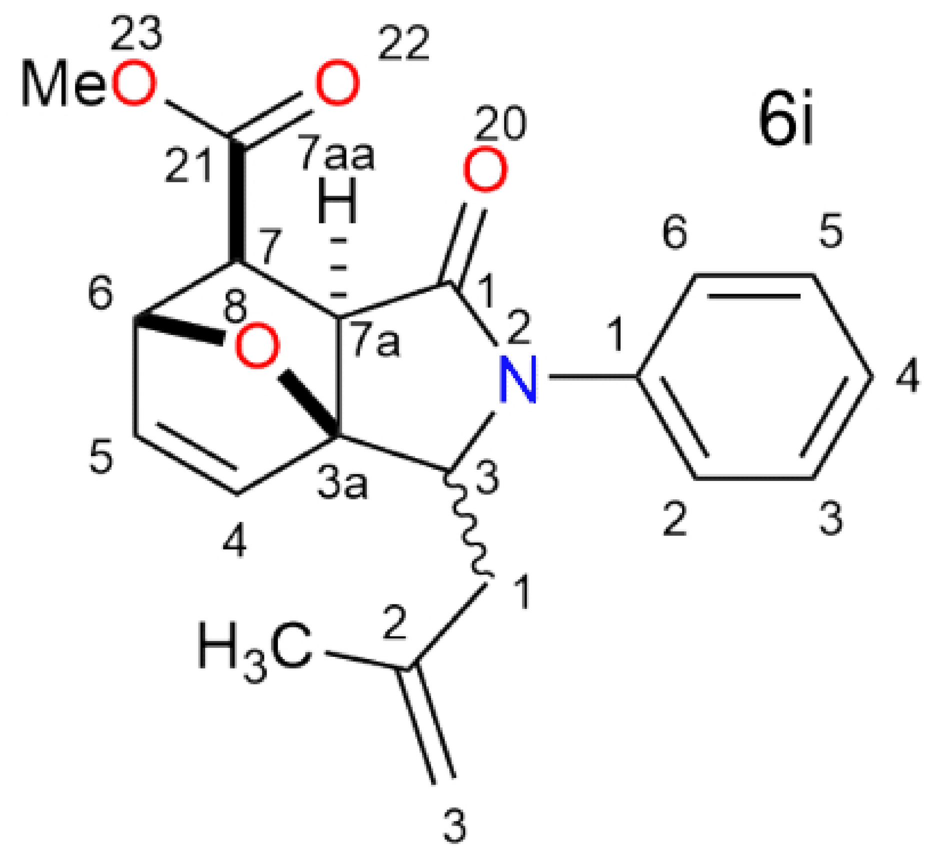

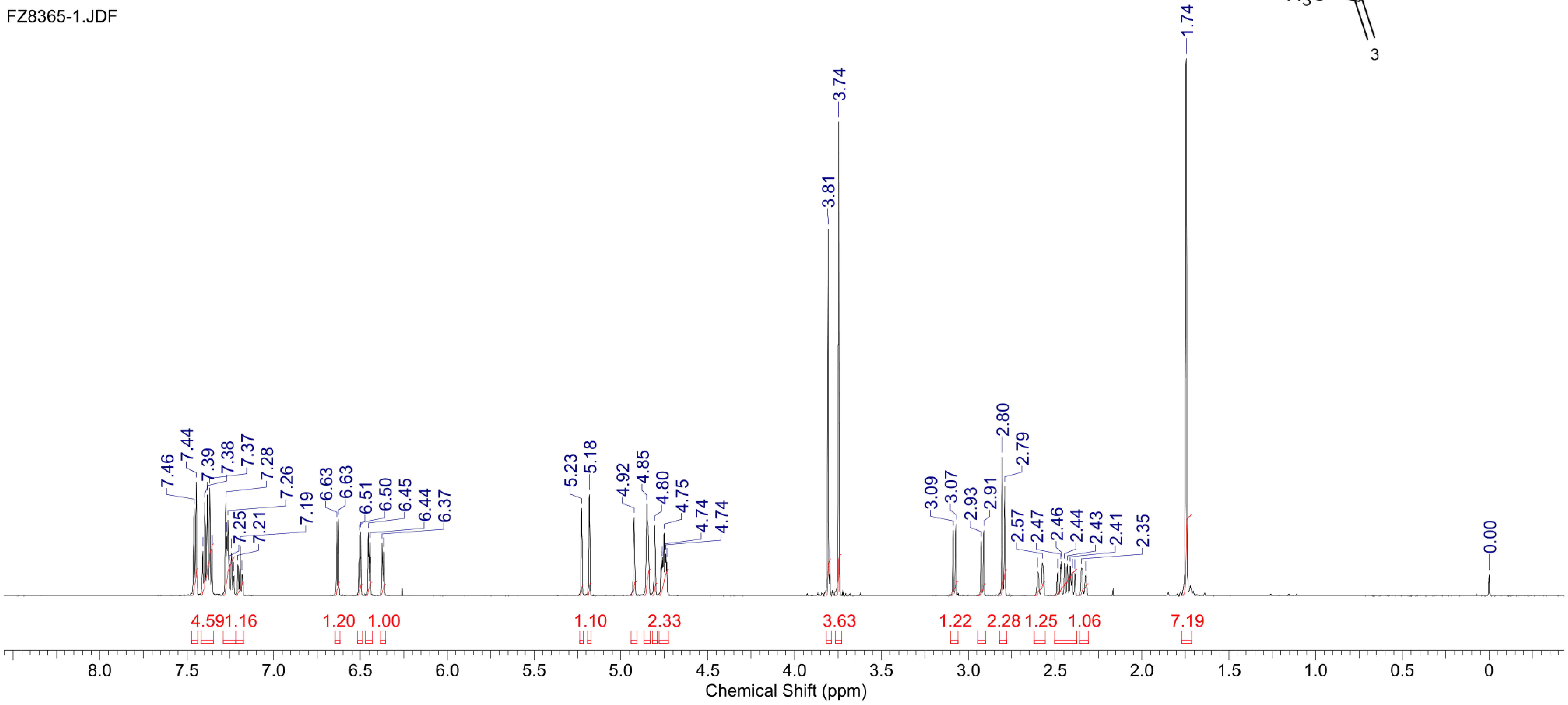

Formula $C_{21}H_{23}NO_4$	FW 353.4116

**Acquisition Time (sec)**	1.9818	**Comment**	single_pulse	**Date**	30 Jan 2020 09:25:14		
**Date Stamp**	30 Jan 2020 08:46:45			**File Name**	C:\USERS\лаба 534\DOWNLOADS\FZ8365-1.JDF		
**Frequency (MHz)**	600.17	**Nucleus**	1H	**Number of Transients**	8	**Origin**	ECA 600
**Original Points Count**	32768	**Owner**	Mass	**Points Count**	32768	**Pulse Sequence**	single_pulse.ex2
**Receiver Gain**	32.00	**Solvent**	CHLOROFORM-d			**Spectrum Offset (Hz)**	5413.1504
**Sweep Width (Hz)**	16534.39	**Temperature (degree C)**	24.400				

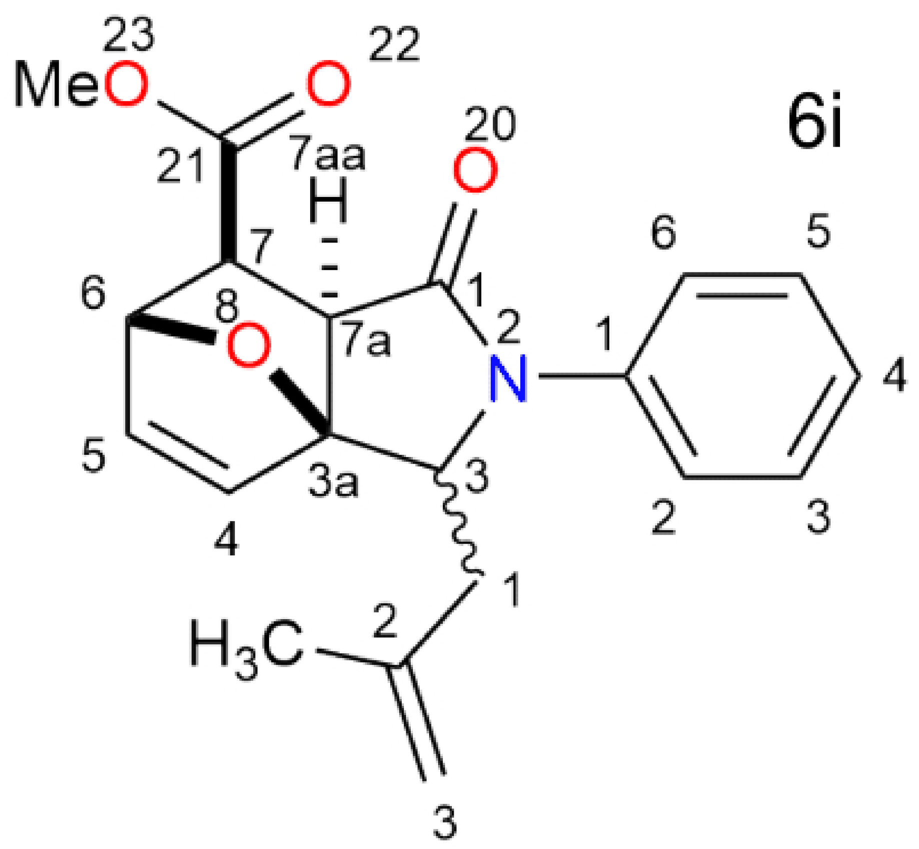

6i

FZ8365-1.JDF

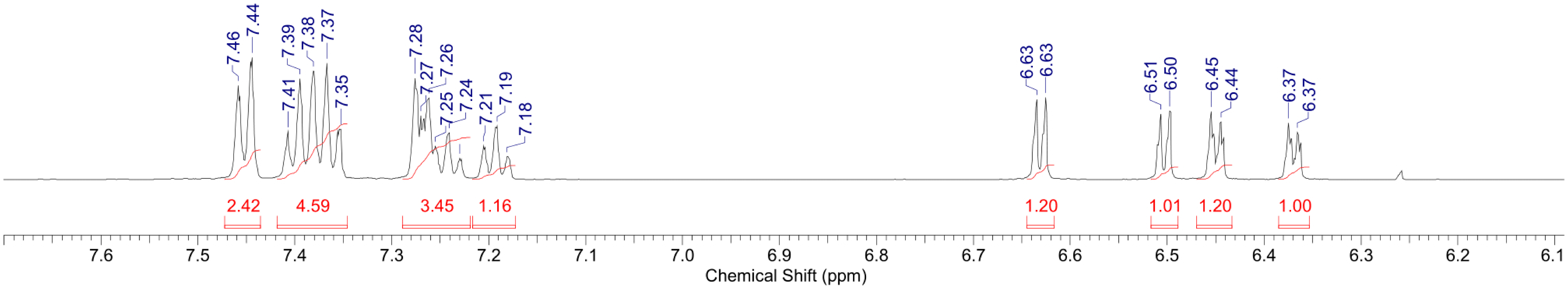

**Formula** $C_{21}H_{23}NO_4$	**FW** 353.4116

**Acquisition Time (sec)**	1.9818	**Comment**	single_pulse	**Date**	30 Jan 2020 09:25:14		
**Date Stamp**	30 Jan 2020 08:46:45			**File Name**	C:\USERS\паба 534\DOWNLOADS\FZ8365-1.JDF		
**Frequency (MHz)**	600.17	**Nucleus**	1H	**Number of Transients**	8	**Origin**	ECA 600
**Original Points Count**	32768	**Owner**	Mass	**Points Count**	32768	**Pulse Sequence**	single_pulse.ex2
**Receiver Gain**	32.00	**Solvent**	CHLOROFORM-d			**Spectrum Offset (Hz)**	5413.1504
**Sweep Width (Hz)**	16534.39	**Temperature (degree C)**	24.400				

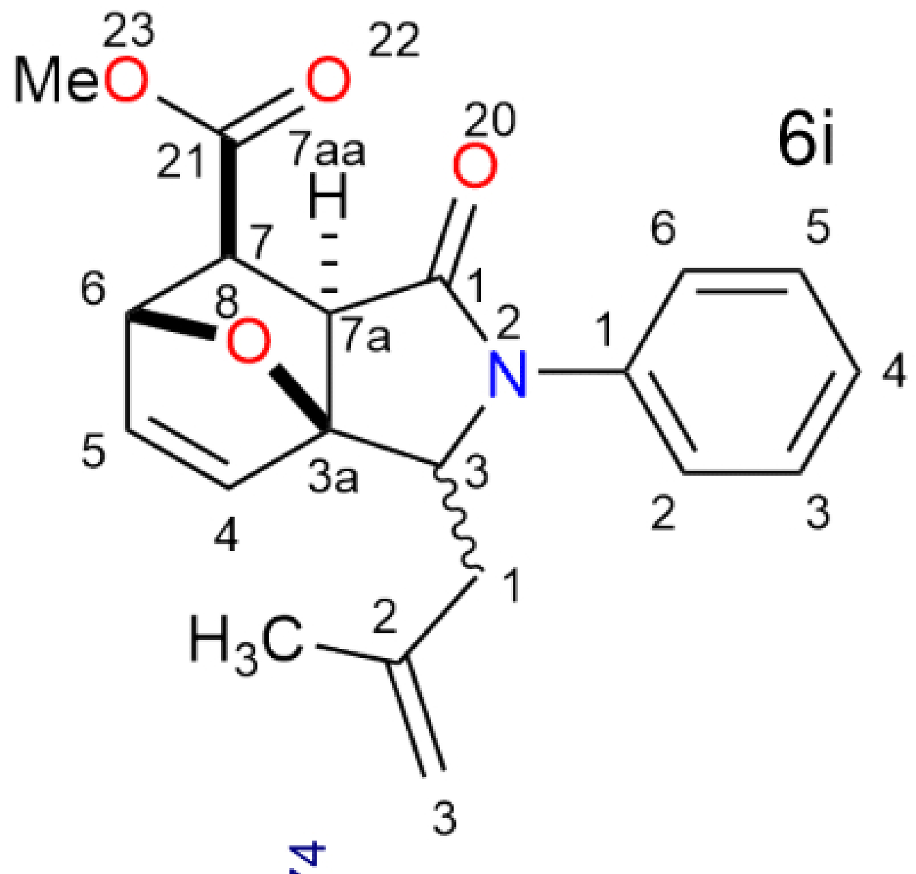

6i

FZ8365-1.JDF

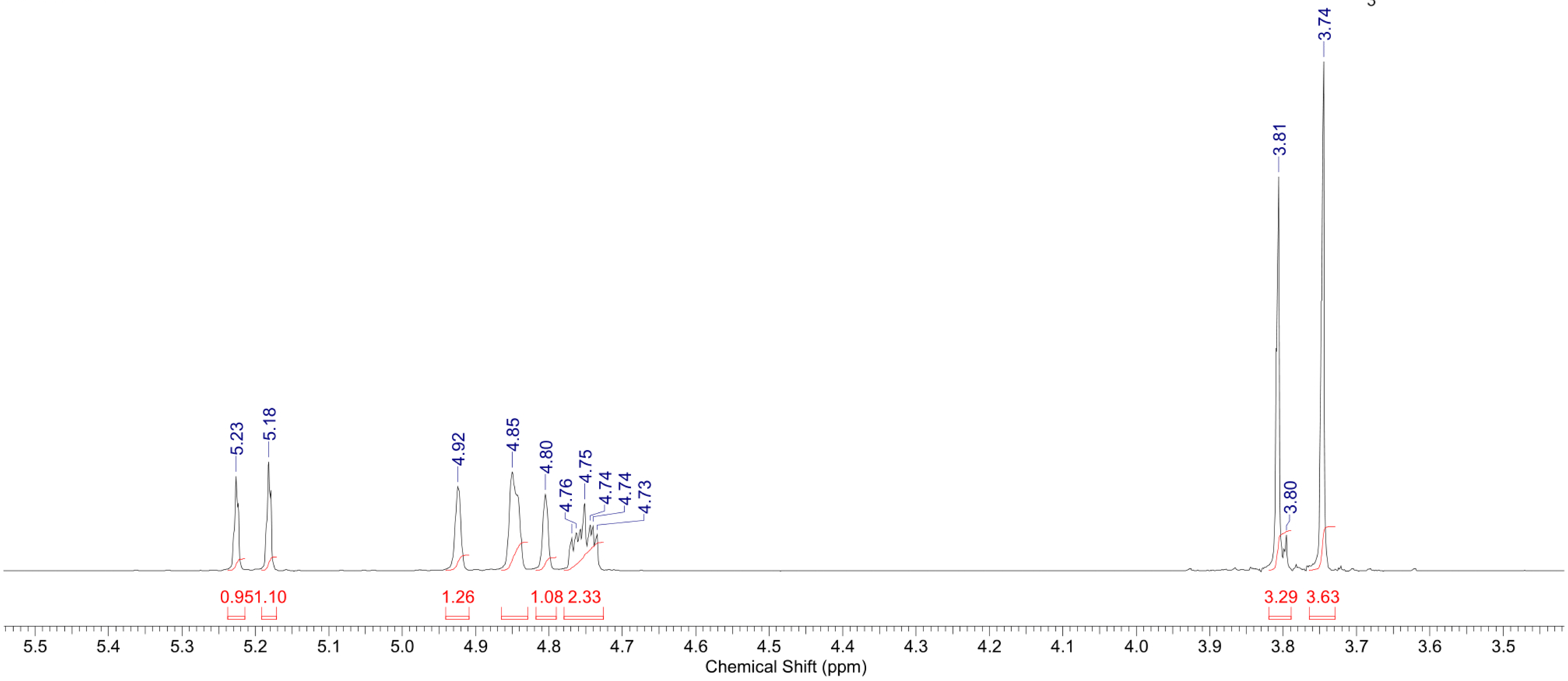

**Formula** $C_{21}H_{23}NO_4$	**FW** 353.4116

**Acquisition Time (sec)**	1.9818	**Comment**	single_pulse	**Date**	30 Jan 2020 09:25:14		
**Date Stamp**	30 Jan 2020 08:46:45			**File Name**	C:\USERS\па6а 534\DOWNLOADS\FZ8365-1.JDF		
**Frequency (MHz)**	600.17	**Nucleus**	1H	**Number of Transients**	8	**Origin**	ECA 600
**Original Points Count**	32768	**Owner**	Mass	**Points Count**	32768	**Pulse Sequence**	single_pulse.ex2
**Receiver Gain**	32.00	**Solvent**	CHLOROFORM-d			**Spectrum Offset (Hz)**	5413.1504
**Sweep Width (Hz)**	16534.39	**Temperature (degree C)**	24.400				

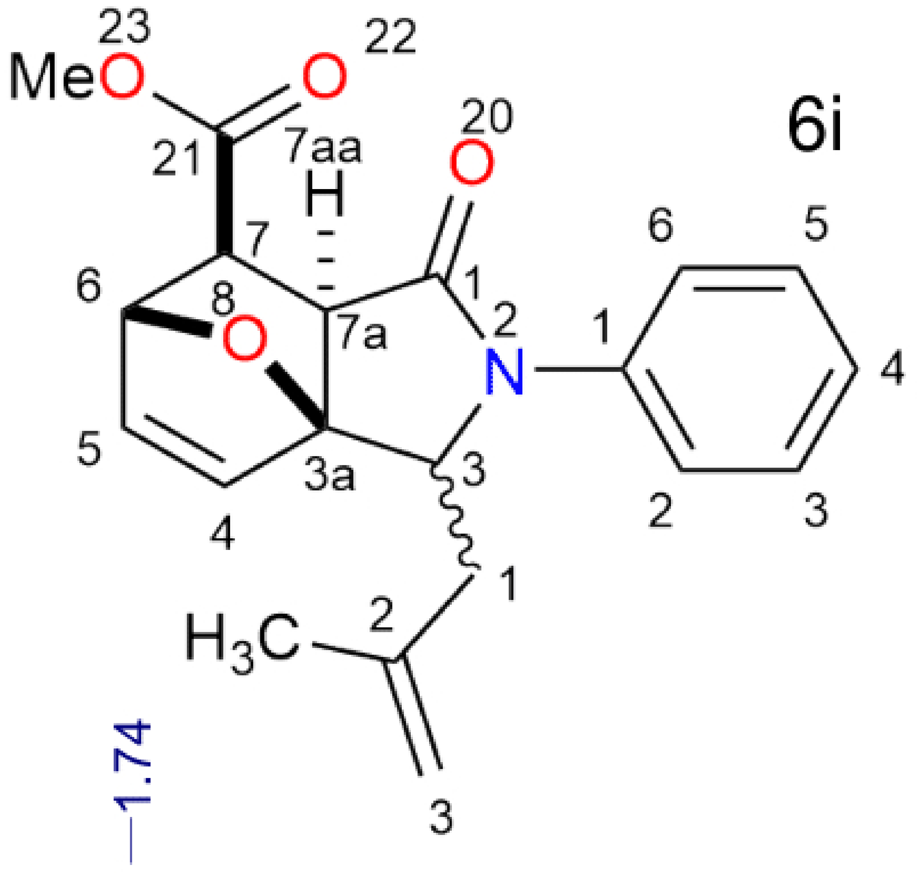

6i

FZ8365-1.JDF

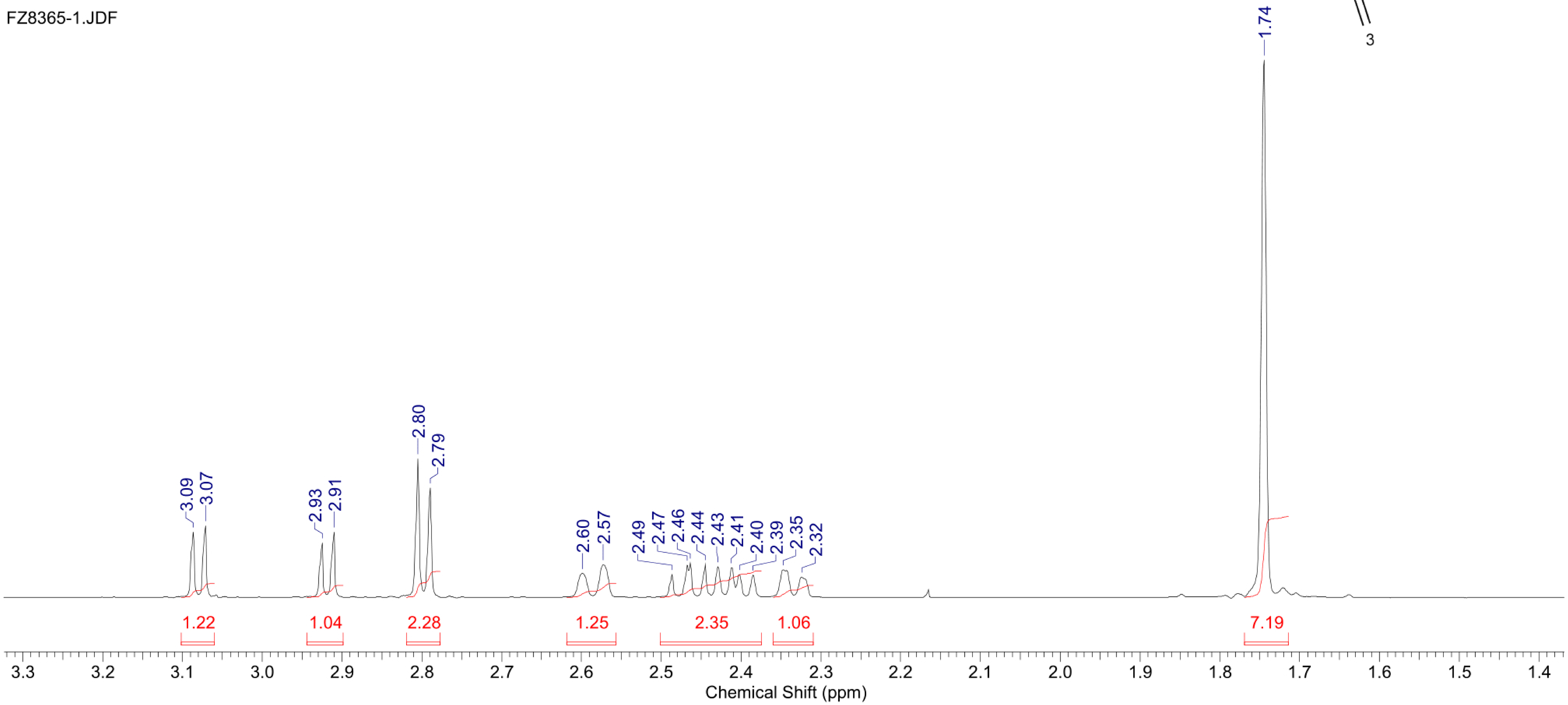

**Formula** $C_{20}H_{21}NO_4$	**FW** 339.3850

**Acquisition Time (sec)**	0.6921	**Comment**	single pulse decoupled gated NOE	**Date**	08 Feb 2020 00:46:17
**Date Stamp**	08 Feb 2020 00:07:59	**File Name**	C:\USERS\лаба 534\DOWNLOADS\FZ8420-1.JDF		
**Frequency (MHz)**	150.91	**Nucleus**	13C	**Number of Transients**	1000
**Origin**	ECA 600	**Original Points Count**	32768	**Owner**	Mass
**Points Count**	32768	**Pulse Sequence**	single_pulse_dec	**Receiver Gain**	56.00
**Solvent**	CHLOROFORM-d	**Spectrum Offset (Hz)**	15091.3428	**Sweep Width (Hz)**	47348.49
**Temperature (degree C)**	25.000				

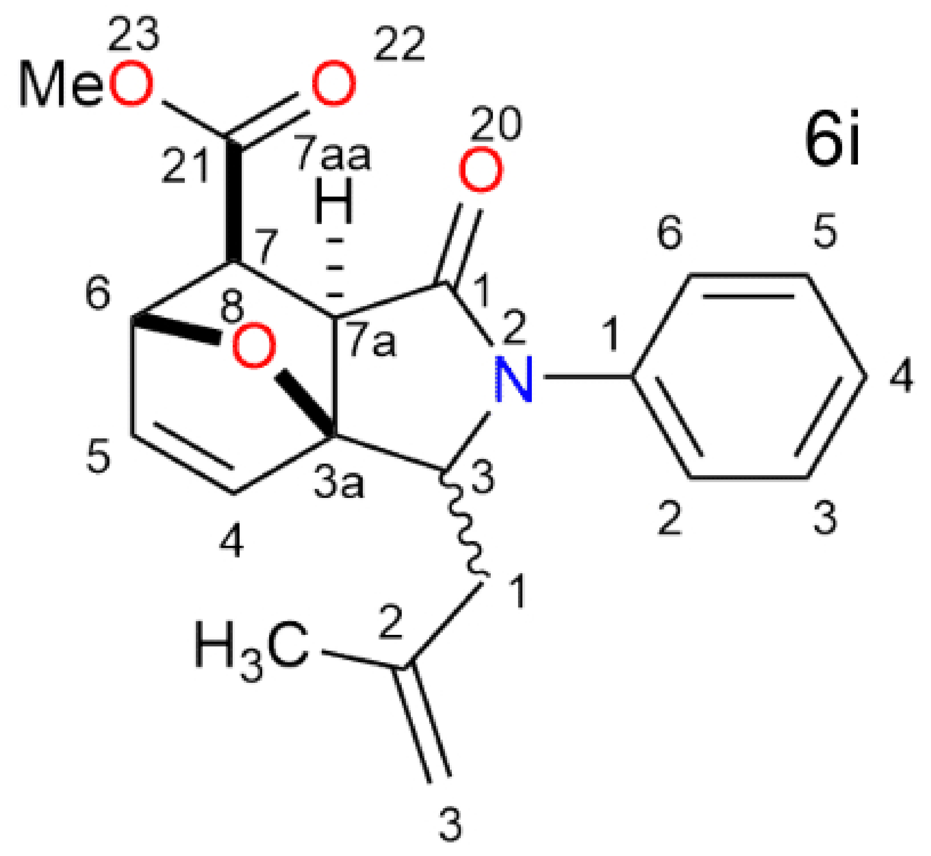

6i

FZ8420-1.JDF

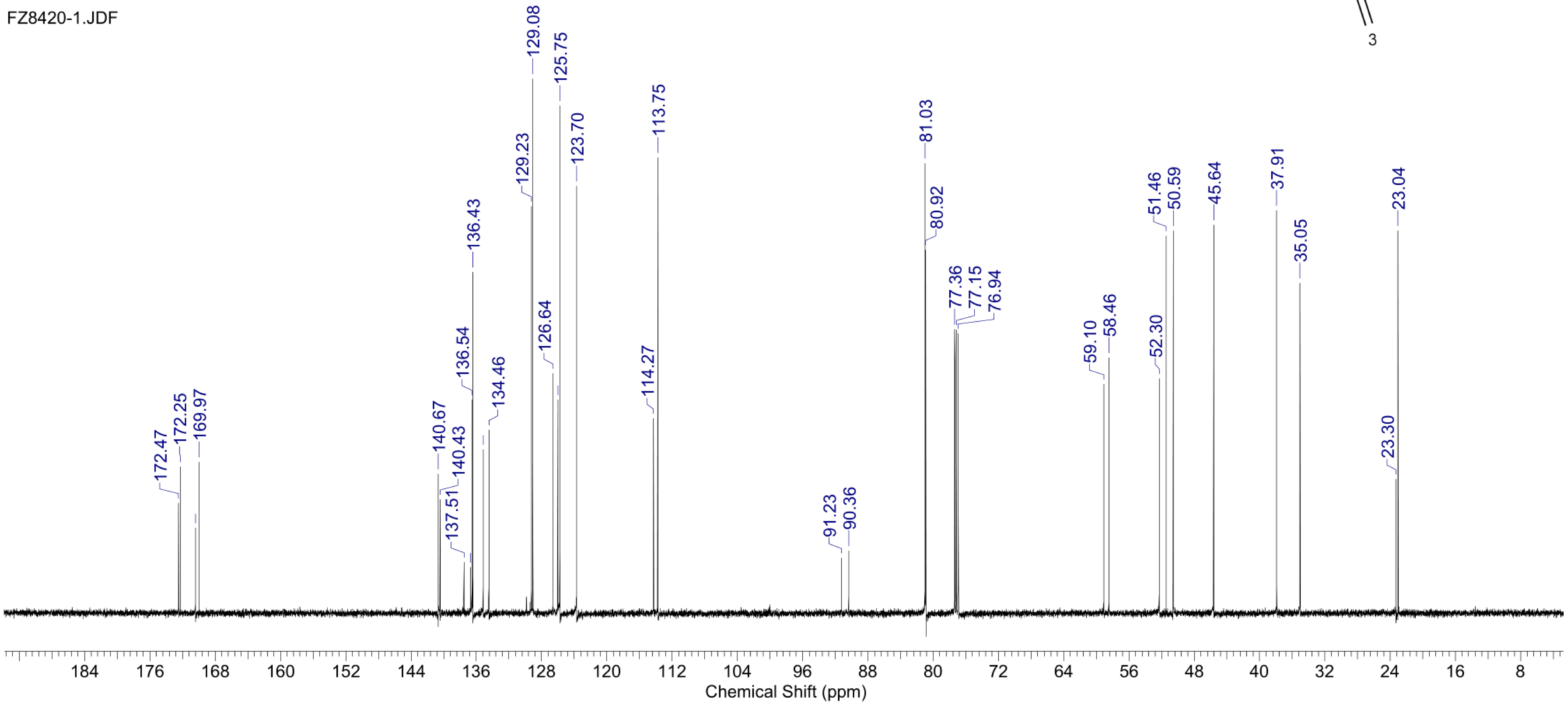

Formula $C_{20}H_{21}NO_4$	FW 339.3850

Acquisition Time (sec)	0.6921	***Comment***	single pulse decoupled gated NOE	***Date***	08 Feb 2020 00:46:17
Date Stamp	08 Feb 2020 00:07:59	***File Name***	C:\USERS\лаба 534\DOWNLOADS\FZ8420-1.JDF		
Frequency (MHz)	150.91	***Nucleus***	13C	***Number of Transients***	1000
Origin	ECA 600	***Original Points Count***	32768	***Owner***	Mass
Points Count	32768	***Pulse Sequence***	single_pulse_dec	***Receiver Gain***	56.00
Solvent	CHLOROFORM-d	***Spectrum Offset (Hz)***	15091.3428	***Sweep Width (Hz)***	47348.49
Temperature (degree C)	25.000				

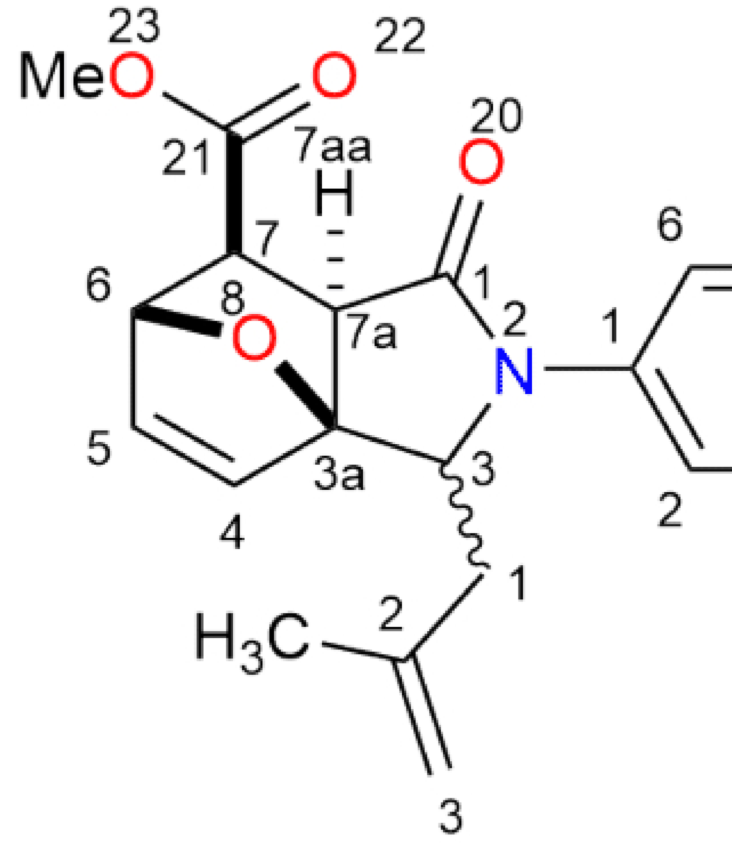

6i

FZ8420-1.JDF

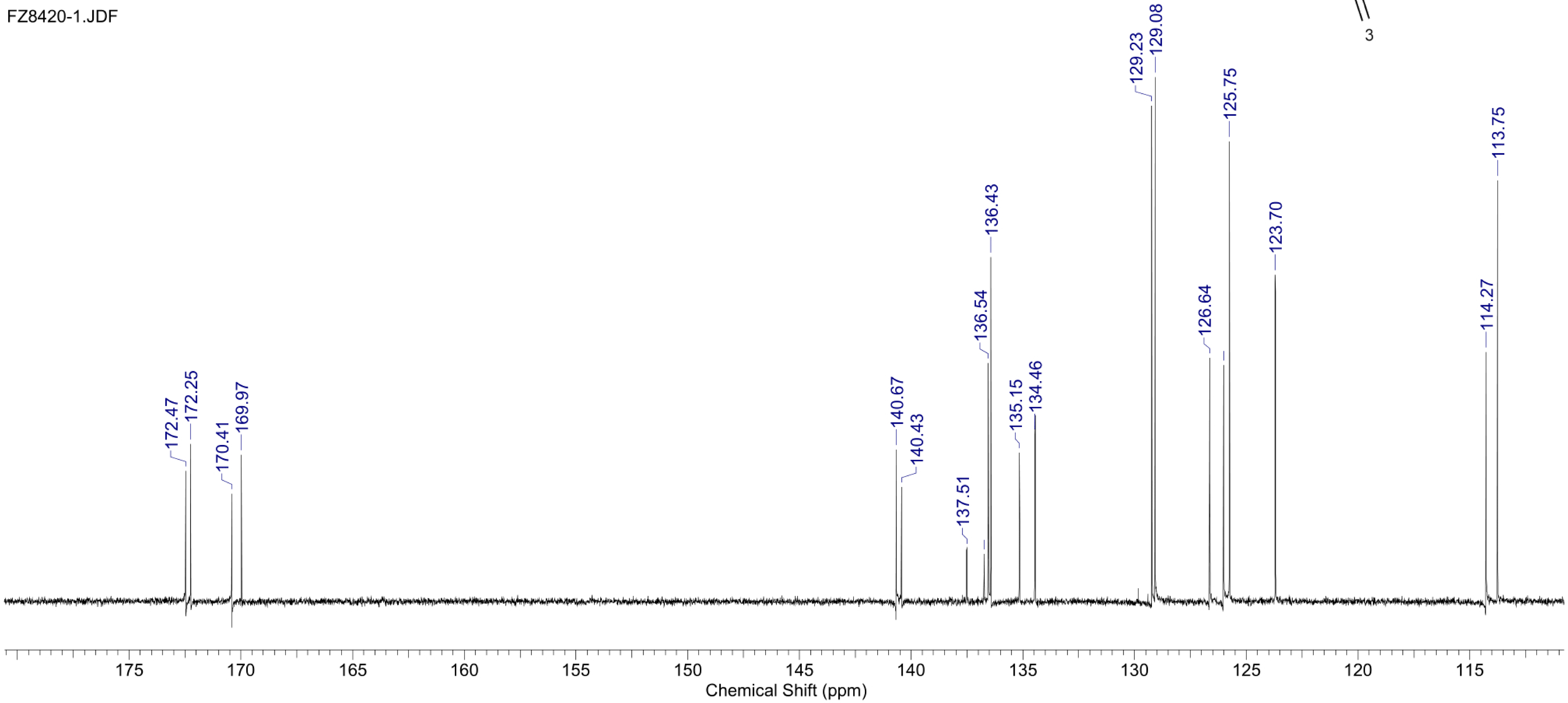

**Formula** $C_{20}H_{21}NO_4$	**FW** 339.3850

Acquisition Time (sec)	0.6921	***Comment***	single pulse decoupled gated NOE			***Date***	08 Feb 2020 00:46:17
Date Stamp	08 Feb 2020 00:07:59			***File Name***	C:\USERS\лаба 534\DOWNLOADS\FZ8420-1.JDF		
Frequency (MHz)	150.91	***Nucleus***	13C	***Number of Transients***	1000	***Origin***	ECA 600
Original Points Count	32768	***Owner***	Mass	***Points Count***	32768	***Pulse Sequence***	single_pulse_dec
Receiver Gain	56.00	***Solvent***	CHLOROFORM-d			***Spectrum Offset (Hz)***	15091.3428
Sweep Width (Hz)	47348.49	***Temperature (degree C)***	25.000				

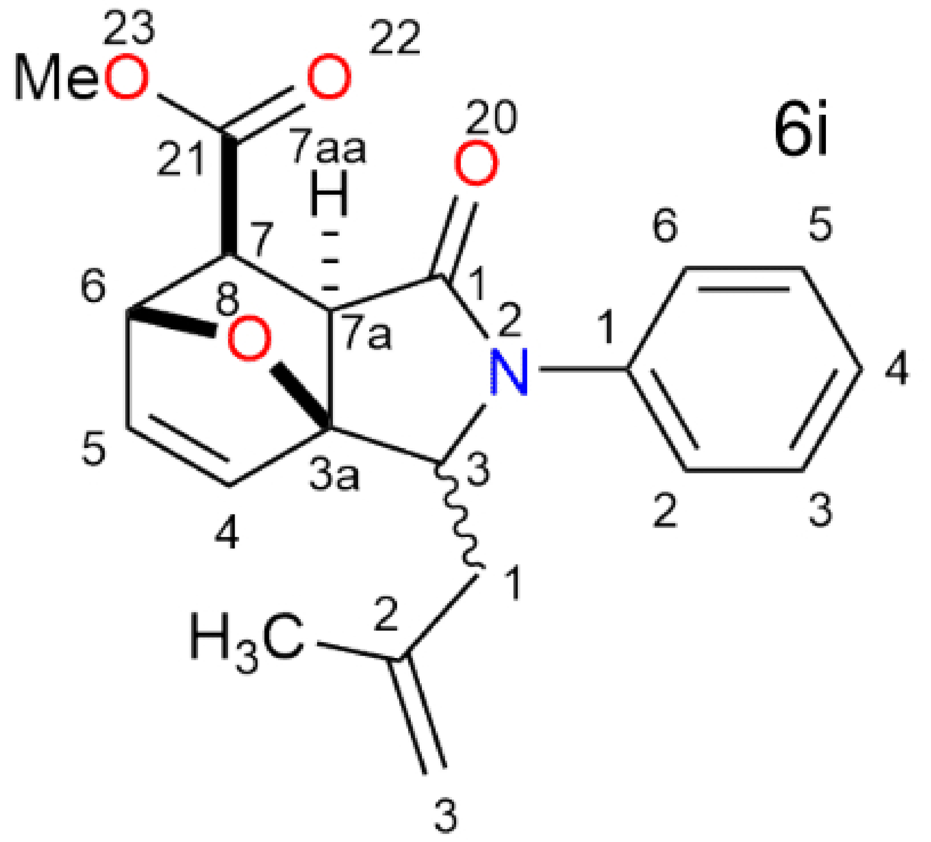

6i

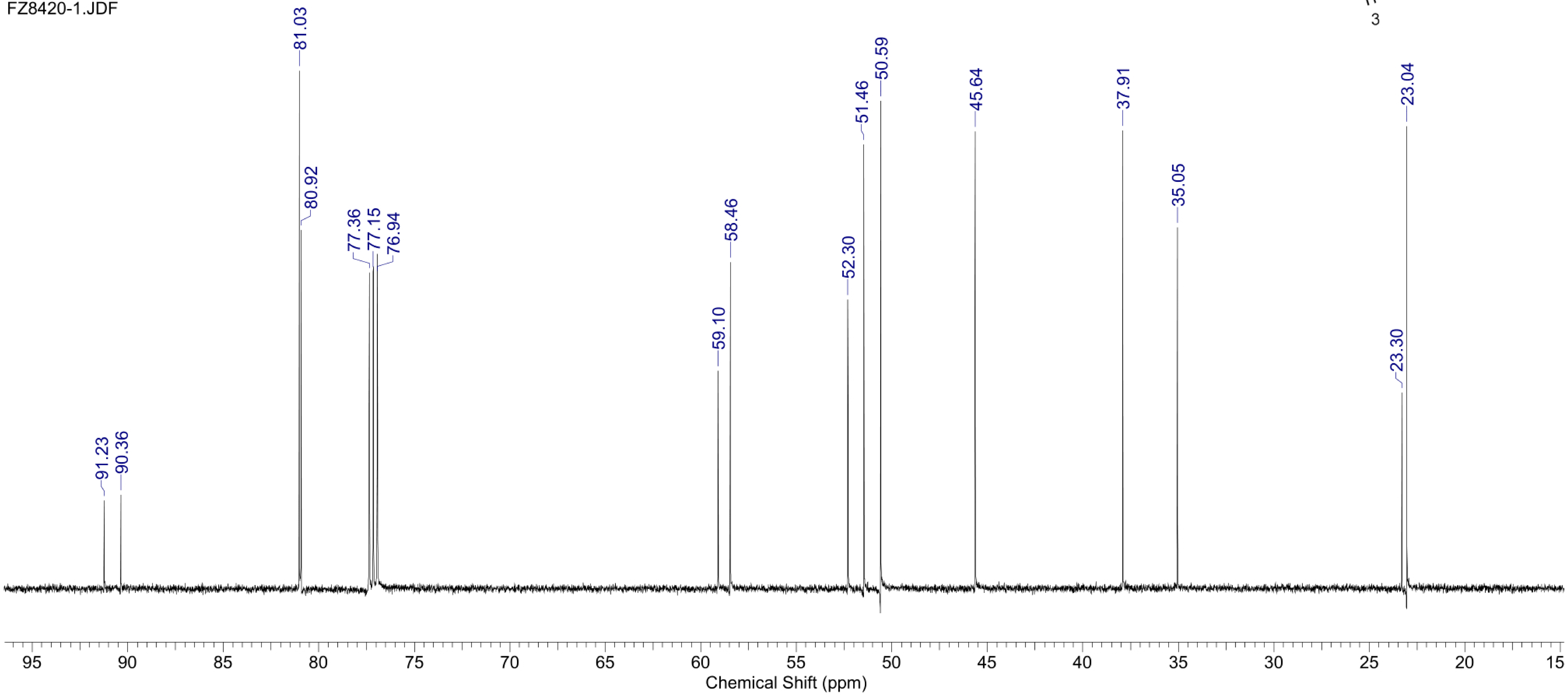

NMR spectrum data for compound 6j

**Formula**	$C_{20}H_{21}NO_4$	**FW**	339.3850

**Acquisition Time (sec)**	1.9818	**Comment**	single_pulse	**Date**	11 Feb 2020 12:45:47		
**Date Stamp**	11 Feb 2020 12:07:34			**File Name**	C:\USERS\лаба 534\DOWNLOADS\FZ8435-1 (1).JDF		
**Frequency (MHz)**	600.17	**Nucleus**	1H	**Number of Transients**	8	**Origin**	ECA 600
**Original Points Count**	32768	**Owner**	Mass	**Points Count**	32768	**Pulse Sequence**	single_pulse.ex2
**Receiver Gain**	40.00	**Solvent**	CHLOROFORM-d			**Spectrum Offset (Hz)**	5410.6274
**Sweep Width (Hz)**	16534.39	**Temperature (degree C)**	23.400				

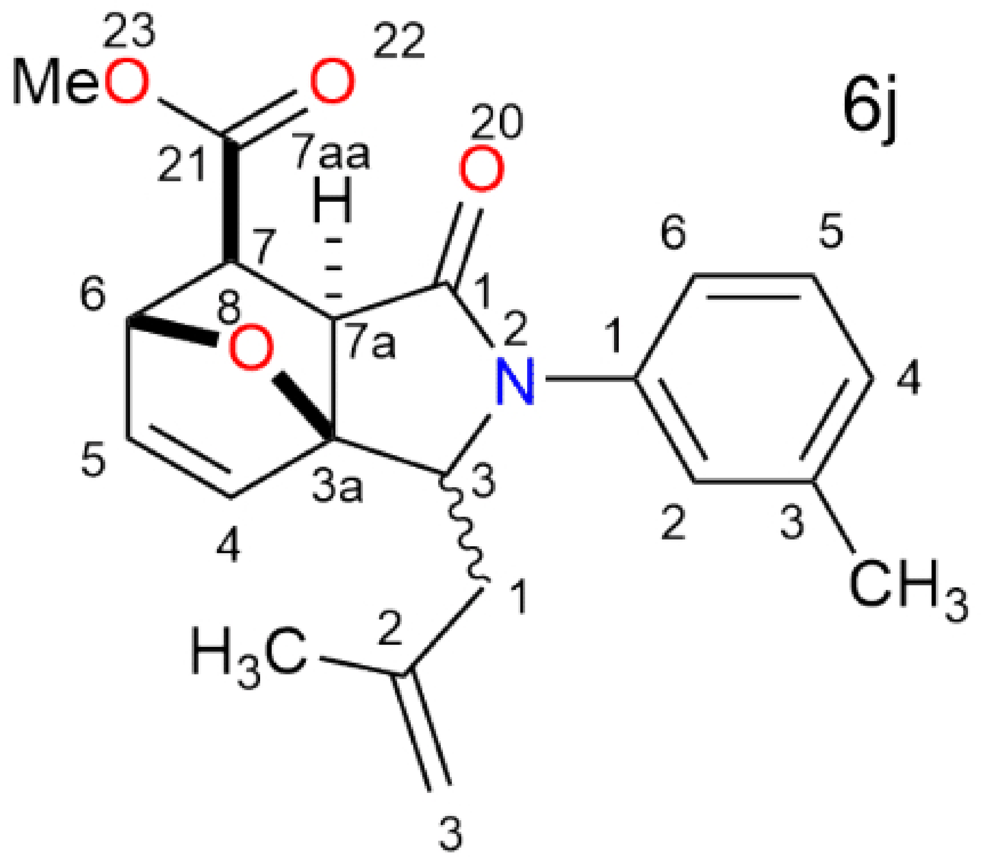

6j

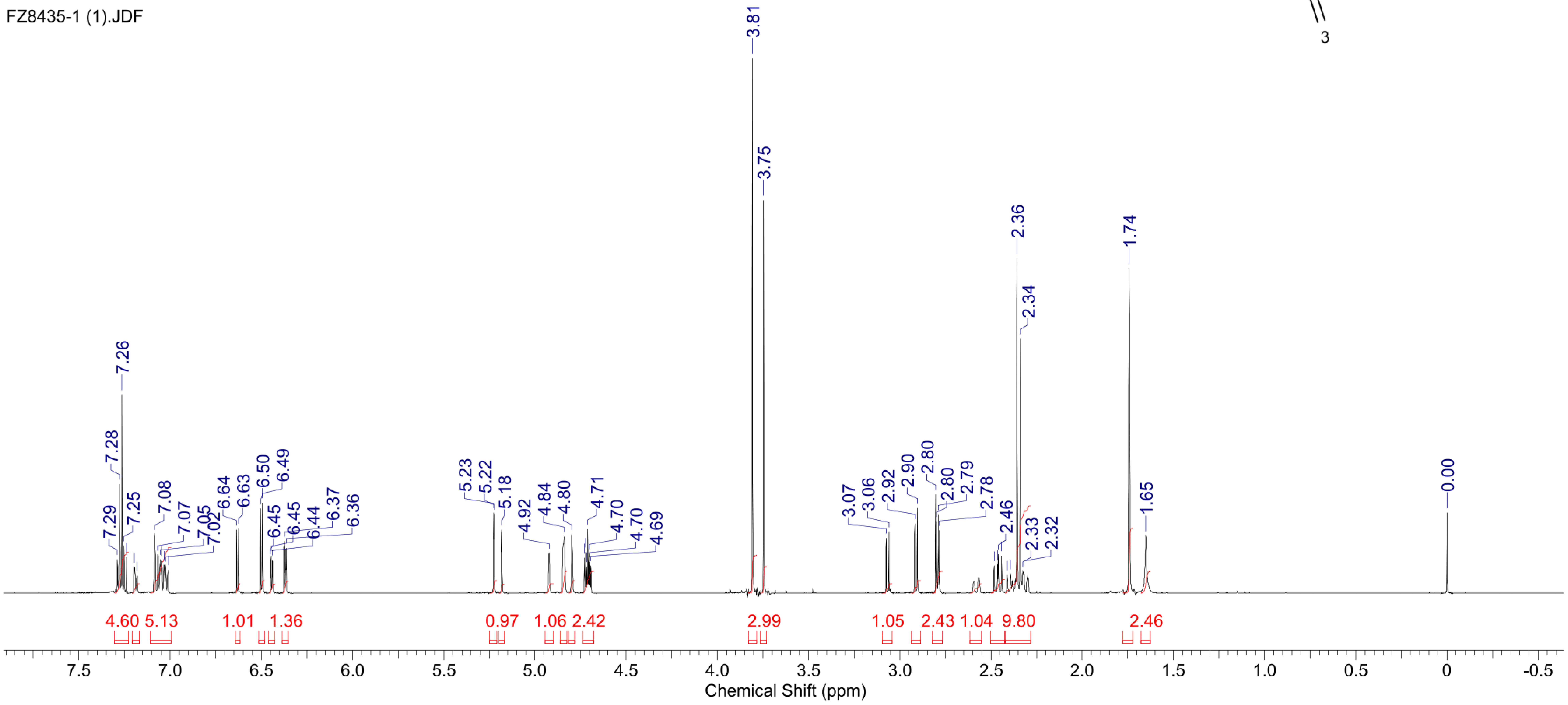

Formula $C_{20}H_{21}NO_4$	FW 339.3850

Acquisition Time (sec)	1.9818	***Comment***	single_pulse	***Date***	11 Feb 2020 12:45:47		
Date Stamp	11 Feb 2020 12:07:34			***File Name***	C:\USERS\лаба 534\DOWNLOADS\FZ8435-1 (1).JDF		
Frequency (MHz)	600.17	***Nucleus***	1H	***Number of Transients***	8	***Origin***	ECA 600
Original Points Count	32768	***Owner***	Mass	***Points Count***	32768	***Pulse Sequence***	single_pulse.ex2
Receiver Gain	40.00	***Solvent***	CHLOROFORM-d			***Spectrum Offset (Hz)***	5410.6274
Sweep Width (Hz)	16534.39	***Temperature (degree C)***	23.400				

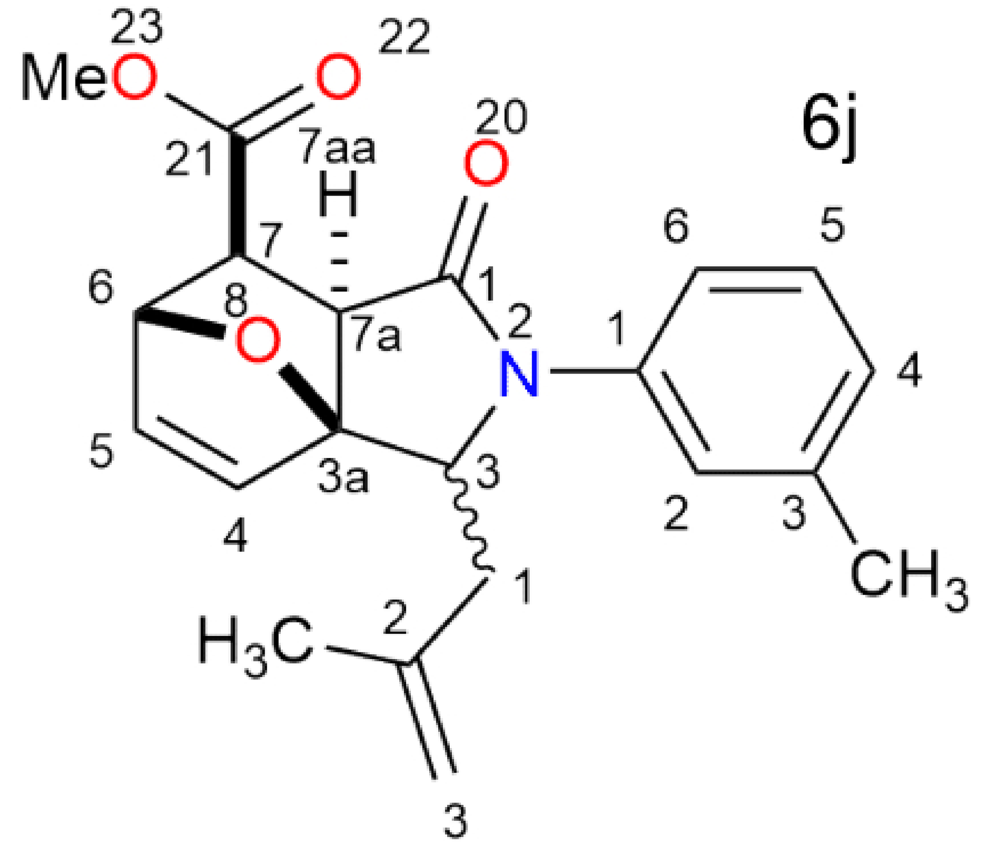

FZ8435-1 (1).JDF

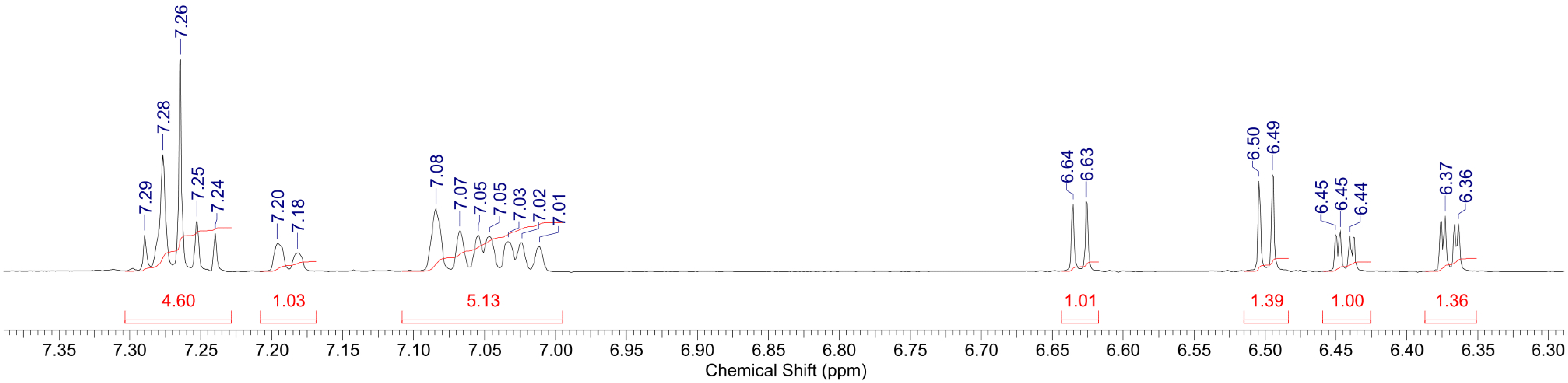

**Formula** $C_{20}H_{21}NO_4$	**FW** 339.3850

**Acquisition Time (sec)**	1.9818	**Comment**	single_pulse	**Date**	11 Feb 2020 12:45:47		
**Date Stamp**	11 Feb 2020 12:07:34			**File Name**	C:\USERS\лаба 534\DOWNLOADS\FZ8435-1 (1).JDF		
**Frequency (MHz)**	600.17	**Nucleus**	1H	**Number of Transients**	8	**Origin**	ECA 600
**Original Points Count**	32768	**Owner**	Mass	**Points Count**	32768	**Pulse Sequence**	single_pulse.ex2
**Receiver Gain**	40.00	**Solvent**	CHLOROFORM-d			**Spectrum Offset (Hz)**	5410.6274
**Sweep Width (Hz)**	16534.39	**Temperature (degree C)**	23.400				

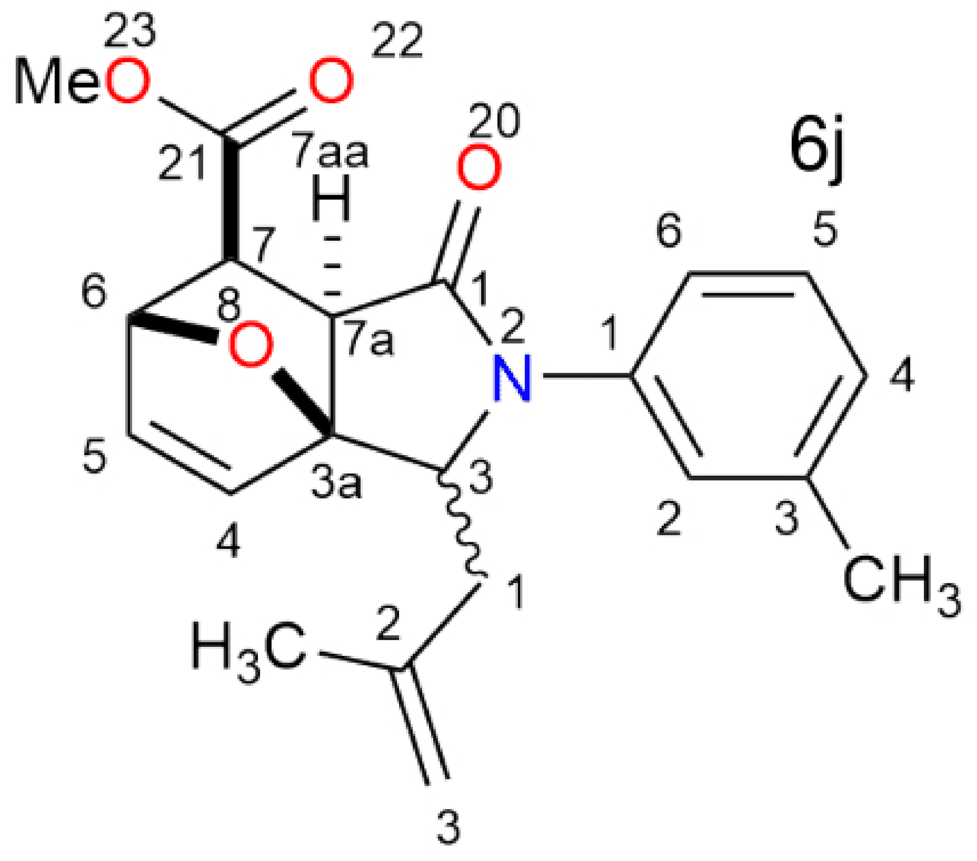

6j

FZ8435-1 (1).JDF

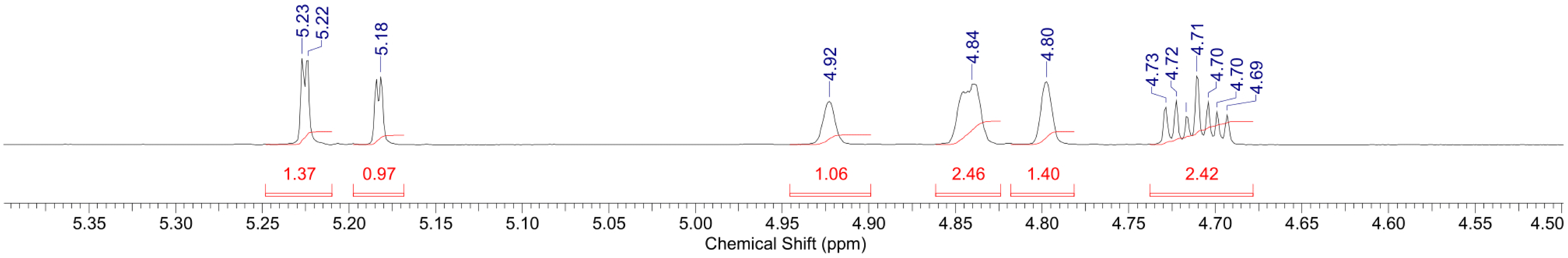

**Formula**	$C_{20}H_{21}NO_4$	**FW**	339.3850

**Acquisition Time (sec)**	1.9818	**Comment**	single_pulse	**Date**	11 Feb 2020 12:45:47		
**Date Stamp**	11 Feb 2020 12:07:34			**File Name**	C:\USERS\па6а 534\DOWNLOADS\FZ8435-1 (1).JDF		
**Frequency (MHz)**	600.17	**Nucleus**	1H	**Number of Transients**	8	**Origin**	ECA 600
**Original Points Count**	32768	**Owner**	Mass	**Points Count**	32768	**Pulse Sequence**	single_pulse.ex2
**Receiver Gain**	40.00	**Solvent**	CHLOROFORM-d			**Spectrum Offset (Hz)**	5410.6274
**Sweep Width (Hz)**	16534.39	**Temperature (degree C)**	23.400				

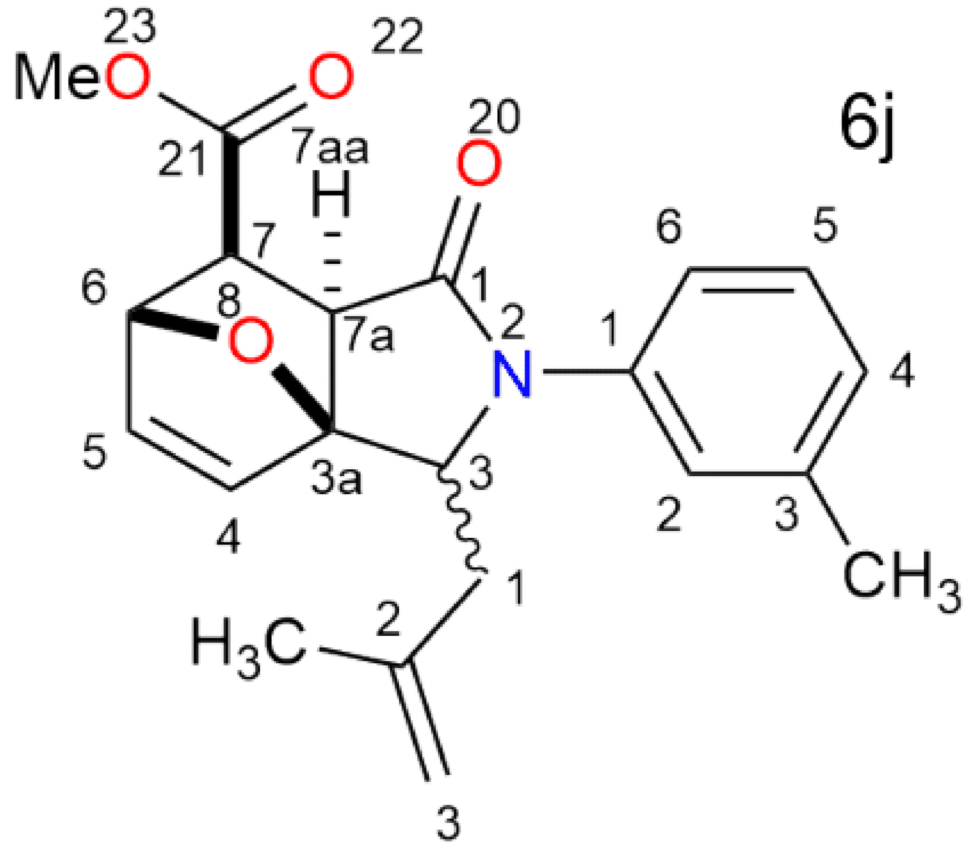

6j

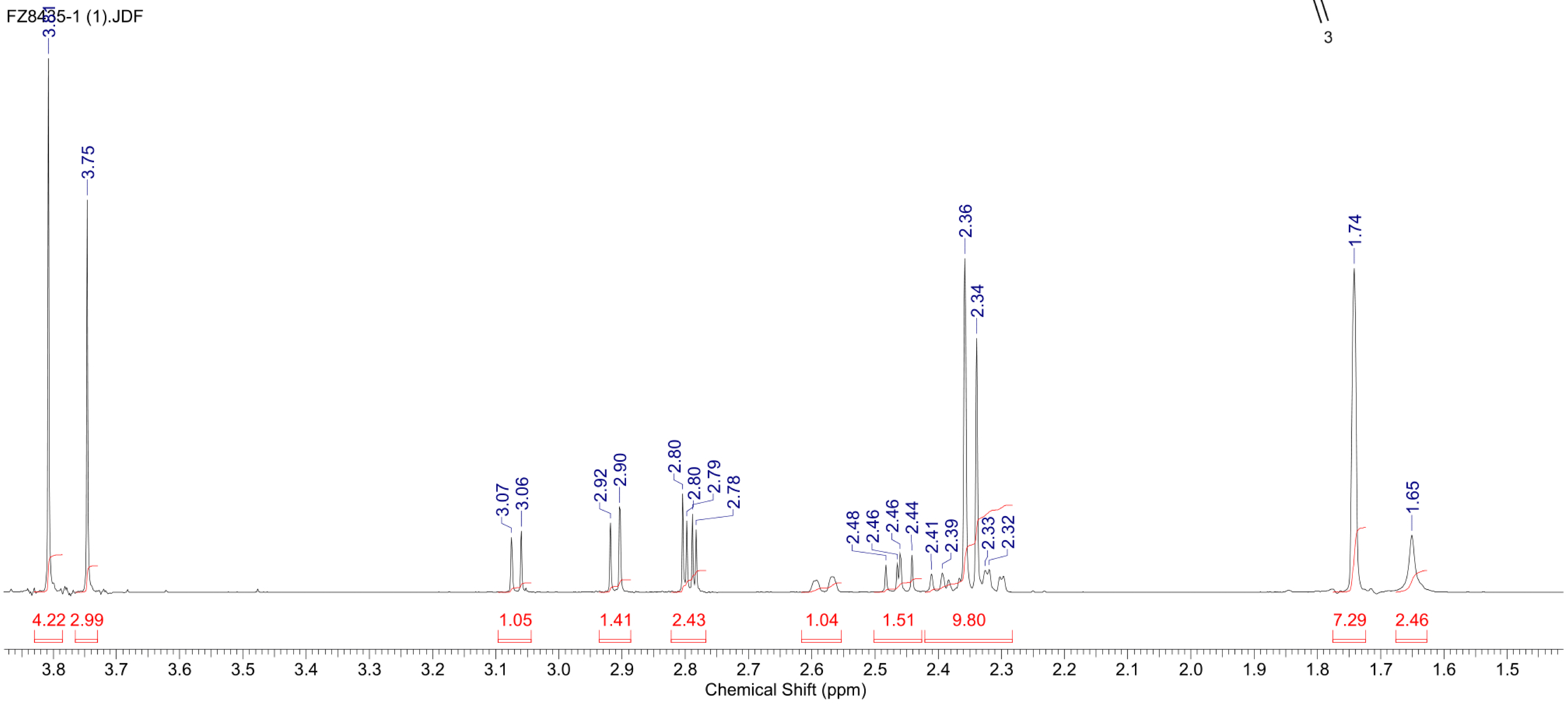

**Formula** $C_{21}H_{23}NO_4$	**FW** 353.4116

Acquisition Time (sec)	0.6921	***Comment***	single pulse decoupled gated NOE	***Date***	14 Feb 2020 14:54:34
Date Stamp	14 Feb 2020 14:16:26	***File Name***	C:\USERS\лаба 534\DESKTOP\Саша\ЯМР\NMR FIDS\FZ8466-2.JDF		
Frequency (MHz)	150.91	***Nucleus***	13C	***Number of Transients***	1000
Origin	ECA 600	***Original Points Count***	32768	***Owner***	Mass
Points Count	32768	***Pulse Sequence***	single_pulse_dec	***Receiver Gain***	54.00
Solvent	CHLOROFORM-d	***Spectrum Offset (Hz)***	15091.3428	***Sweep Width (Hz)***	47348.49
Temperature (degree C)	24.700				

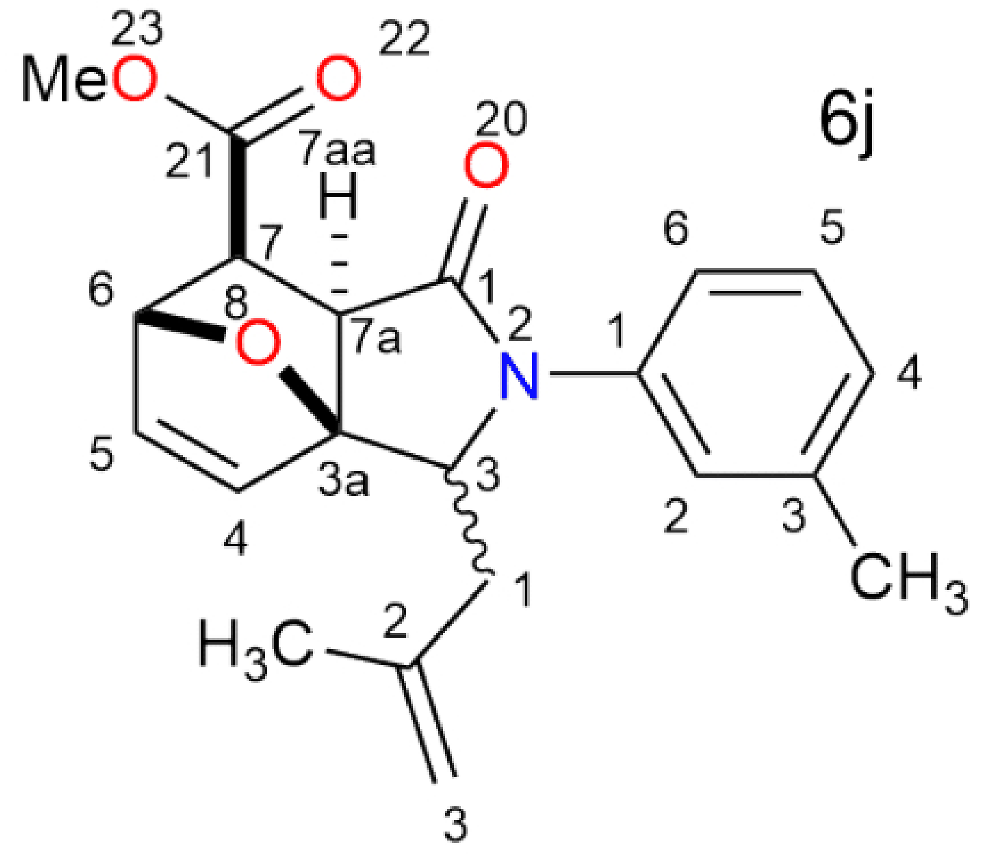

6j

FZ8466-2.JDF

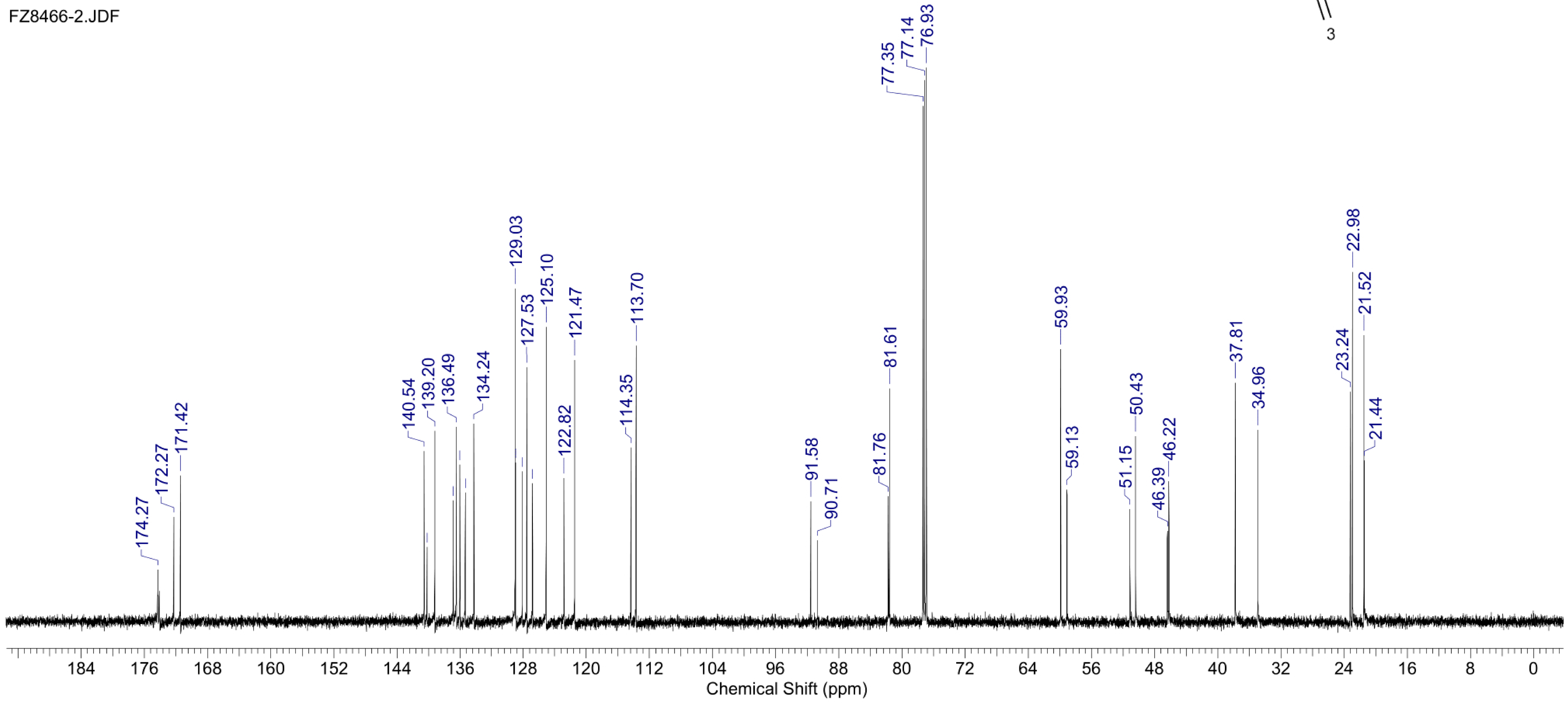

**Formula** $C_{21}H_{23}NO_4$	**FW** 353.4116

Acquisition Time (sec)	0.6921	***Comment***	single pulse decoupled gated NOE			***Date***	14 Feb 2020 14:54:34
Date Stamp	14 Feb 2020 14:16:26			***File Name***	C:\USERS\лаба 534\DESKTOP\Саша\ЯМР\NMR FIDS\FZ8466-2.JDF		
Frequency (MHz)	150.91	***Nucleus***	13C	***Number of Transients***	1000	***Origin***	ECA 600
Original Points Count	32768	***Owner***	Mass	***Points Count***	32768	***Pulse Sequence***	single_pulse_dec
Receiver Gain	54.00	***Solvent***	CHLOROFORM-d			***Spectrum Offset (Hz)***	15091.3428
Sweep Width (Hz)	47348.49	***Temperature (degree C)***	24.700				

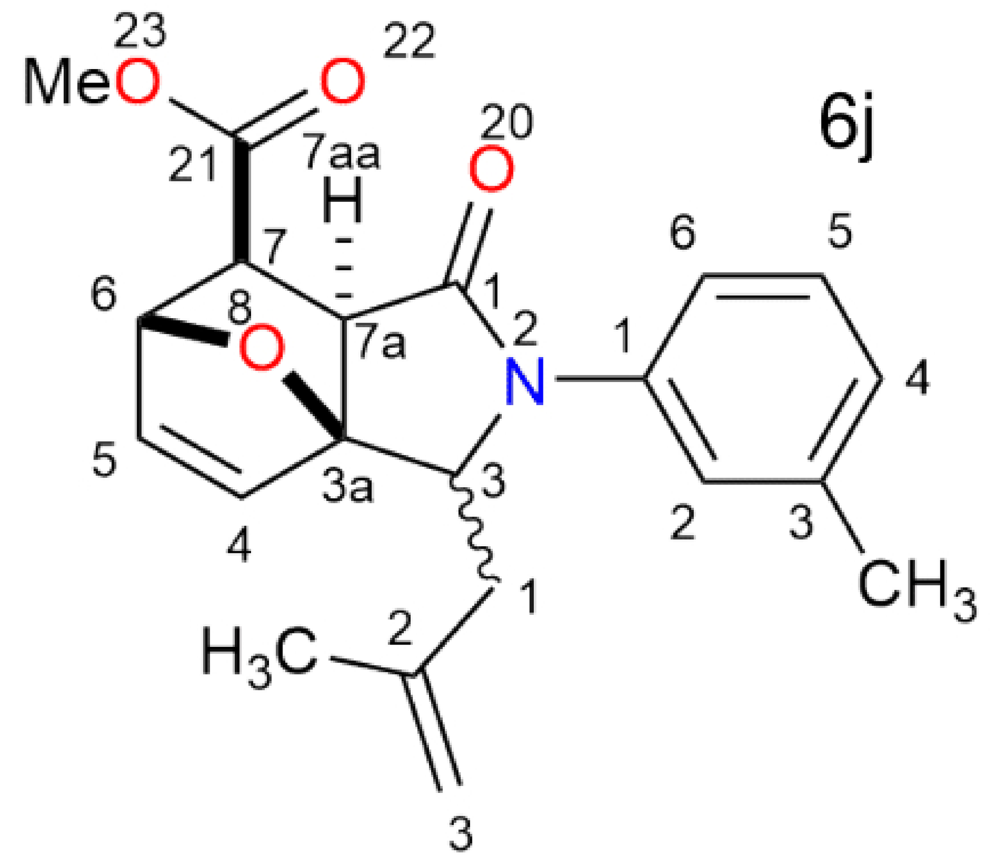

FZ8466-2.JDF

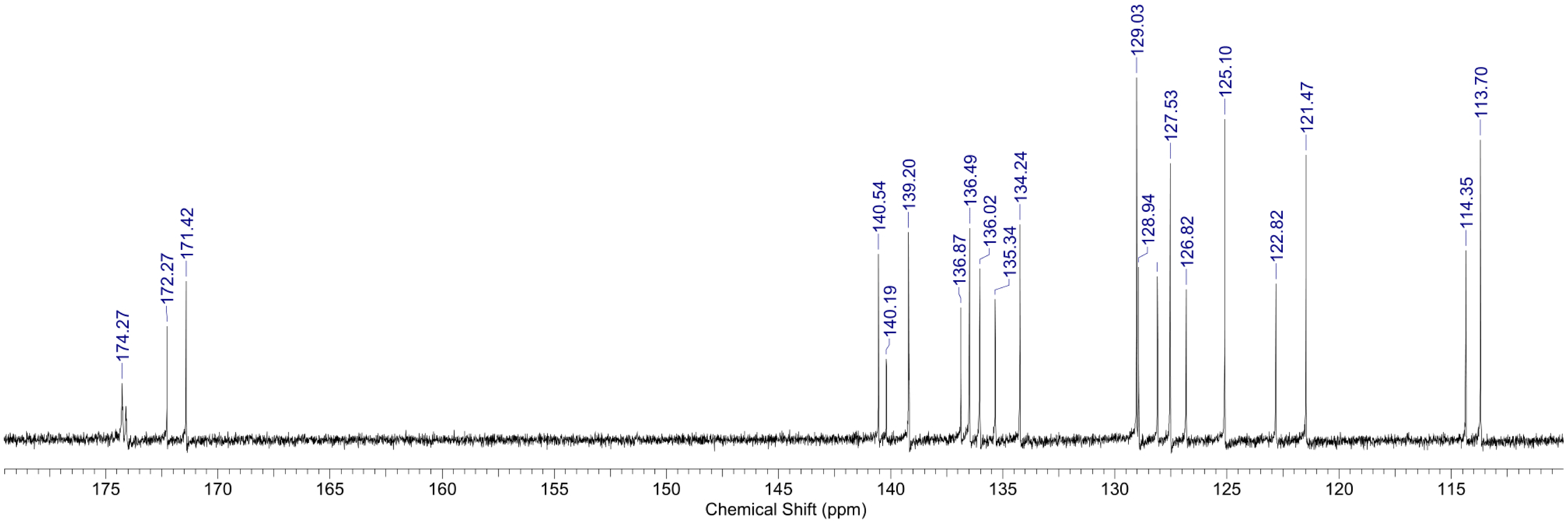

Formula $C_{21}H_{23}NO_4$	FW 353.4116

Acquisition Time (sec)	0.6921	***Comment***	single pulse decoupled gated NOE			***Date***	14 Feb 2020 14:54:34
Date Stamp	14 Feb 2020 14:16:26			***File Name***	C:\USERS\лаба 534\DESKTOP\Саша\ЯМР\NMR FIDS\FZ8466-2.JDF		
Frequency (MHz)	150.91	***Nucleus***	13C	***Number of Transients***	1000	***Origin***	ECA 600
Original Points Count	32768	***Owner***	Mass	***Points Count***	32768	***Pulse Sequence***	single_pulse_dec
Receiver Gain	54.00	***Solvent***	CHLOROFORM-d			***Spectrum Offset (Hz)***	15091.3428
Sweep Width (Hz)	47348.49	***Temperature (degree C)***	24.700				

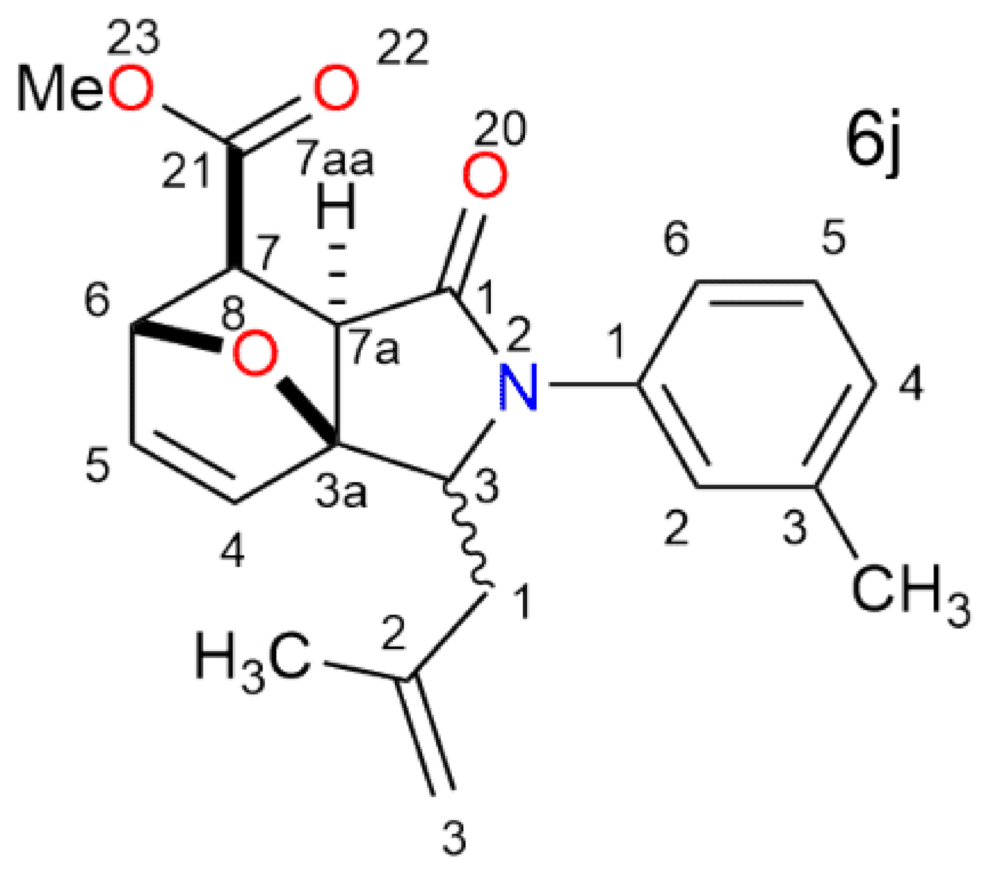

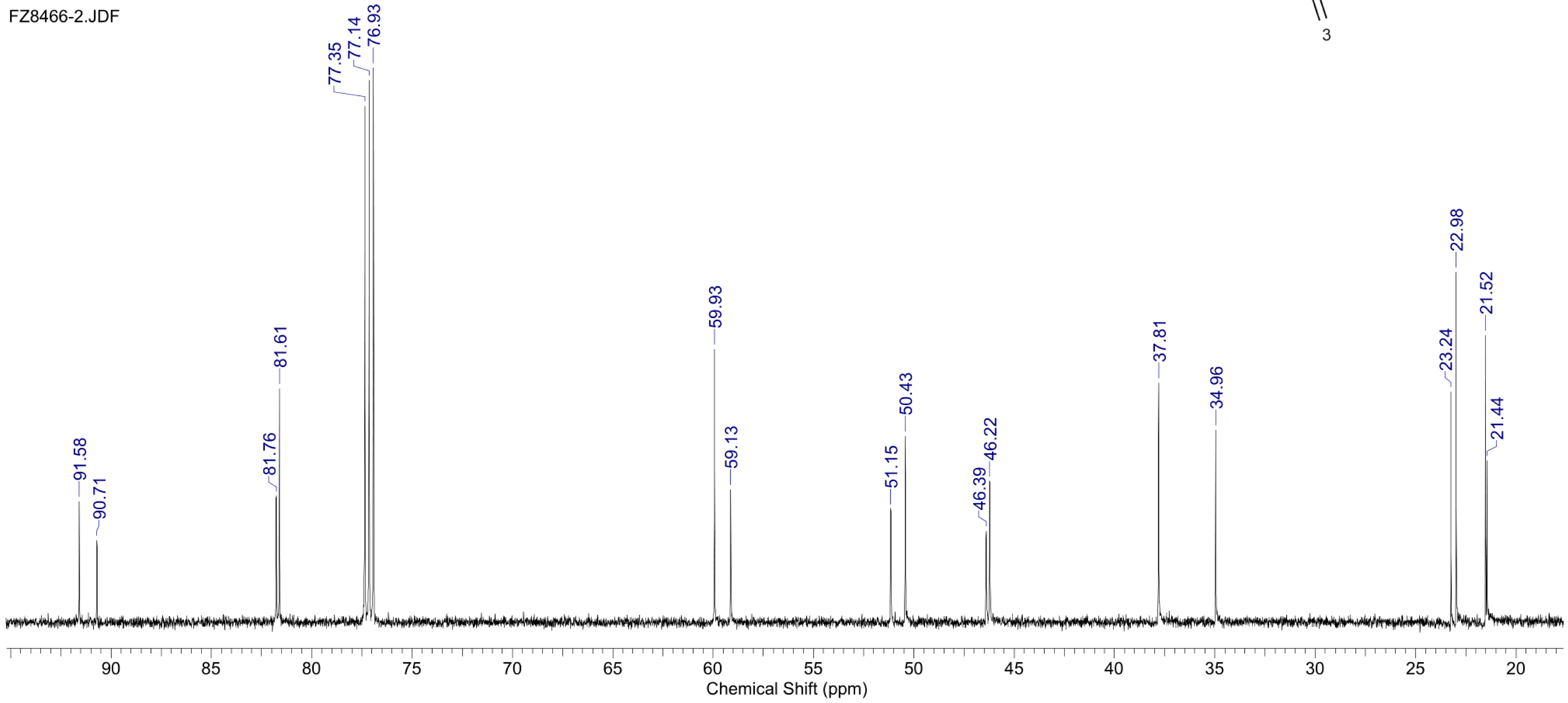

NMR spectrum data for compound 6k

Acquisition Time (sec)	1.9818	***Comment***	single_pulse	***Date***	28 May 1990 08:08:29				
Date Stamp	07 Aug 2019 14:11:55			***File Name***	C:\Users\Fedor\Desktop\07.08.19\FZ7648-1.jdf			***Frequency (MHz)***	600.17
Nucleus	1H	***Number of Transients***	8	***Origin***	ECA 600	***Original Points Count***	32768	***Owner***	delta
Points Count	32768	***Pulse Sequence***	single_pulse.ex2			***Receiver Gain***	44.00	***Solvent***	CHLOROFORM-d
Spectrum Offset (Hz)	5410.1226	***Sweep Width (Hz)***	16534.39	***Temperature (degree C)***	22.100				

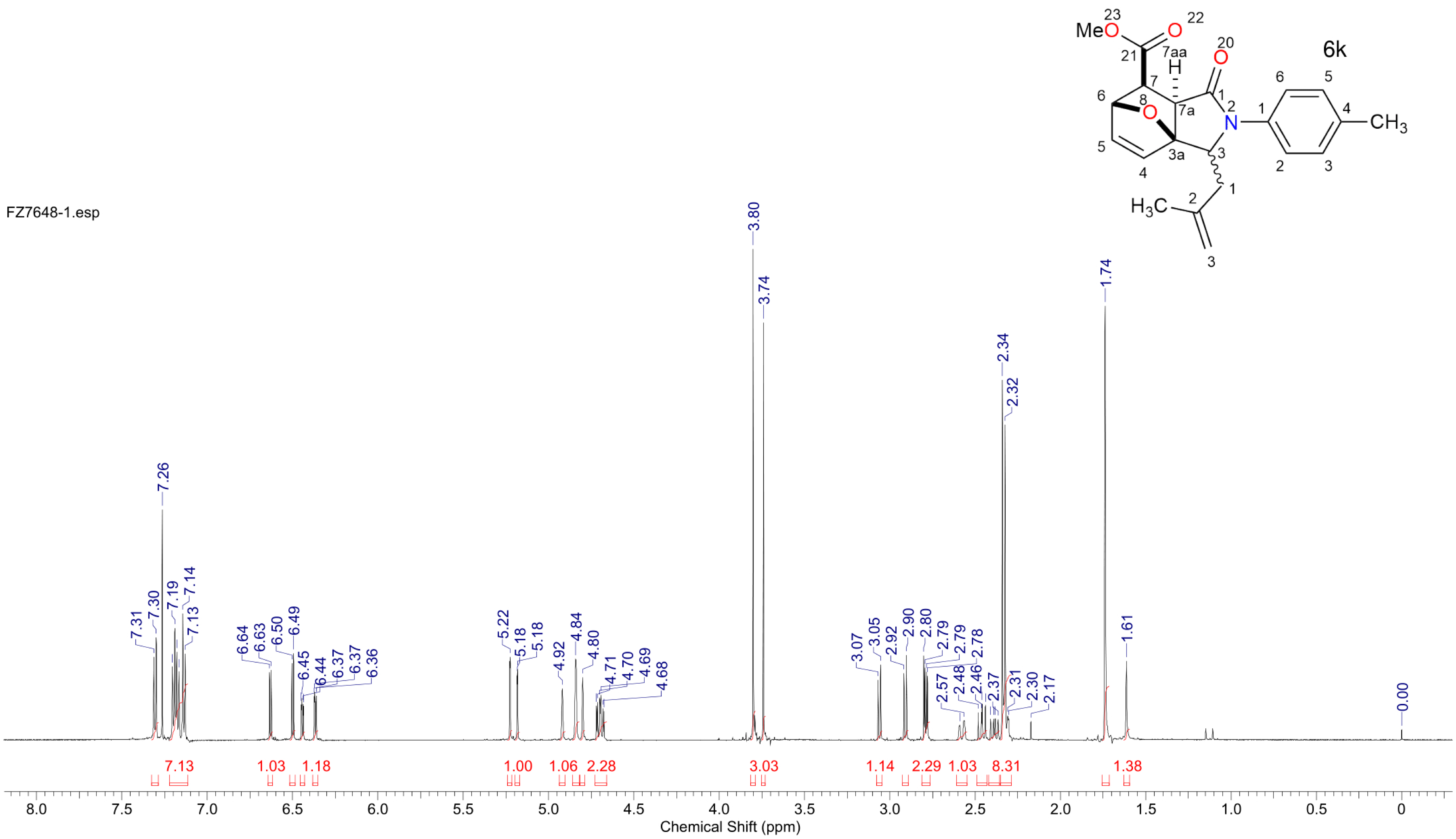

**Acquisition Time (sec)**	1.9818	**Comment**	single_pulse	**Date**	28 May 1990 08:08:29				
**Date Stamp**	07 Aug 2019 14:11:55			**File Name**	C:\Users\Fedor\Desktop\07.08.19\FZ7648-1.jdf			**Frequency (MHz)**	600.17
**Nucleus**	1H	**Number of Transients**	8	**Origin**	ECA 600	**Original Points Count**	32768	**Owner**	delta
**Points Count**	32768	**Pulse Sequence**	single_pulse.ex2			**Receiver Gain**	44.00	**Solvent**	CHLOROFORM-d
**Spectrum Offset (Hz)**	5410.1226	**Sweep Width (Hz)**	16534.39	**Temperature (degree C)**	22.100				

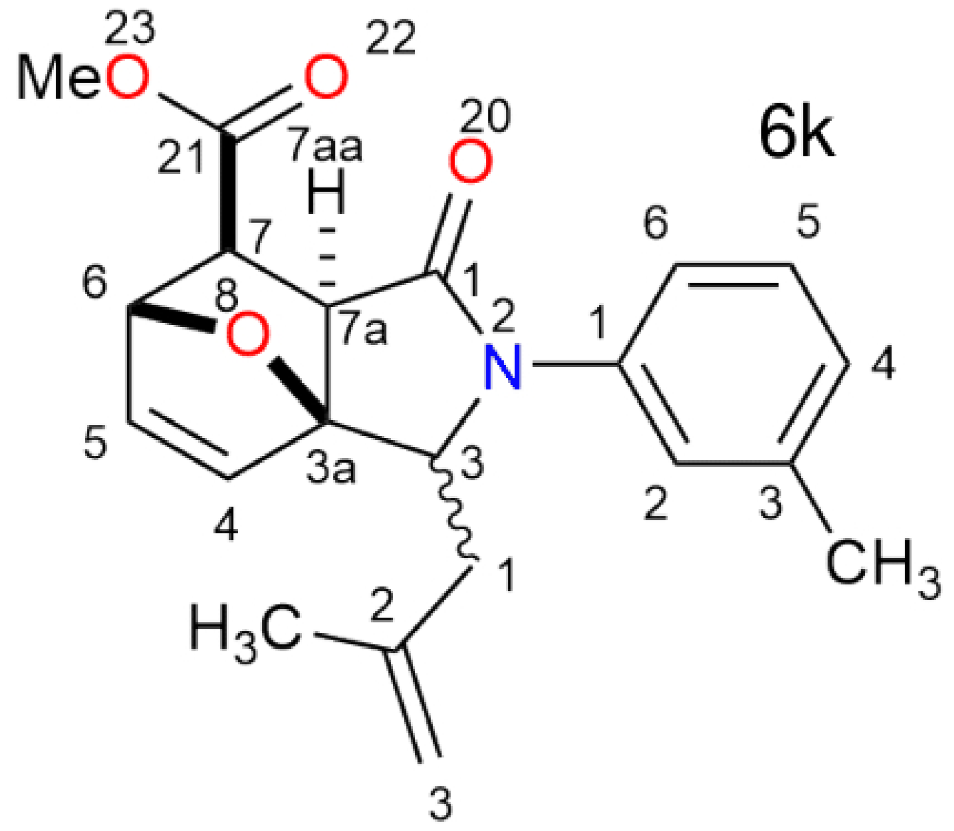

6k

FZ7648-1.esp

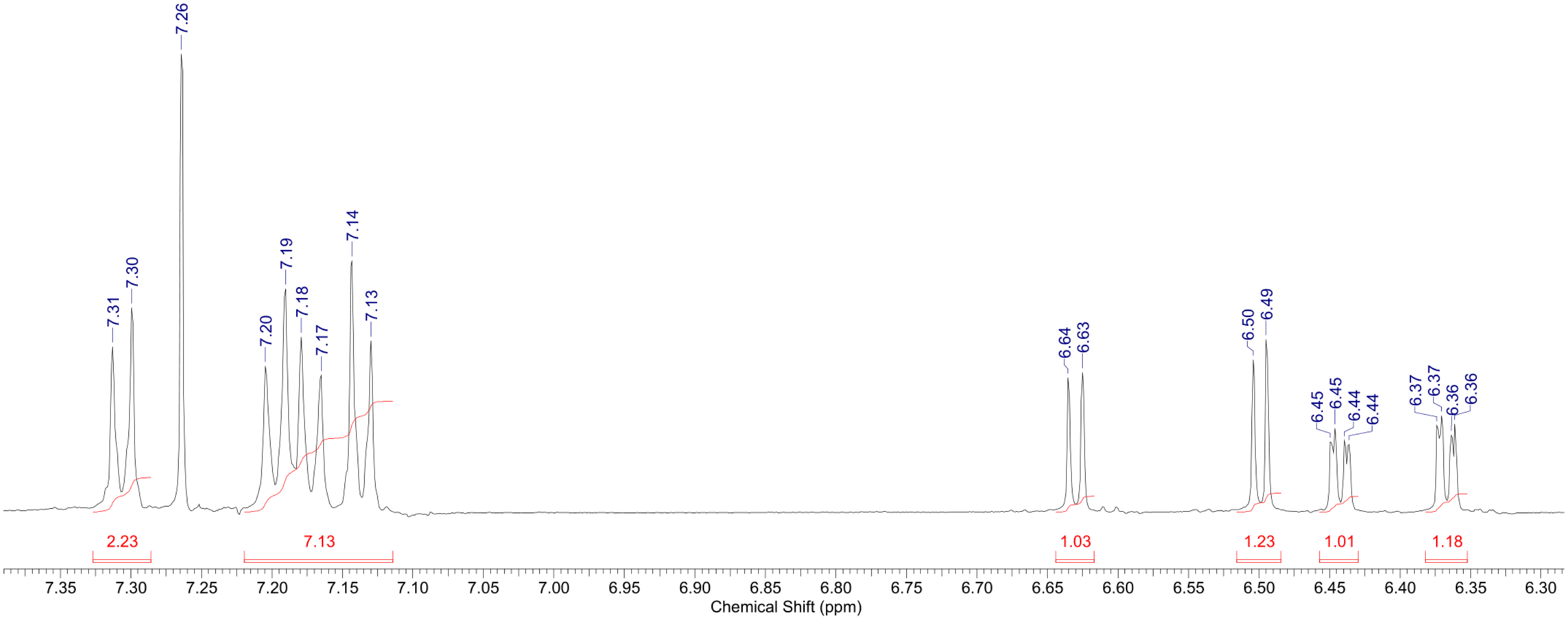

Acquisition Time (sec)	1.9818	***Comment***	single_pulse	***Date***	28 May 1990 08:08:29		
Date Stamp	07 Aug 2019 14:11:55			***File Name***	C:\Users\Fedor\Desktop\07.08.19\FZ7648-1.jdf	***Frequency (MHz)***	600.17
Nucleus	1H	***Number of Transients***	8	***Origin***	ECA 600	***Original Points Count*** 32768	***Owner*** delta
Points Count	32768	***Pulse Sequence***	single_pulse.ex2			***Receiver Gain*** 44.00	***Solvent*** CHLOROFORM-d
Spectrum Offset (Hz)	5410.1226	***Sweep Width (Hz)***	16534.39	***Temperature (degree C)***	22.100		

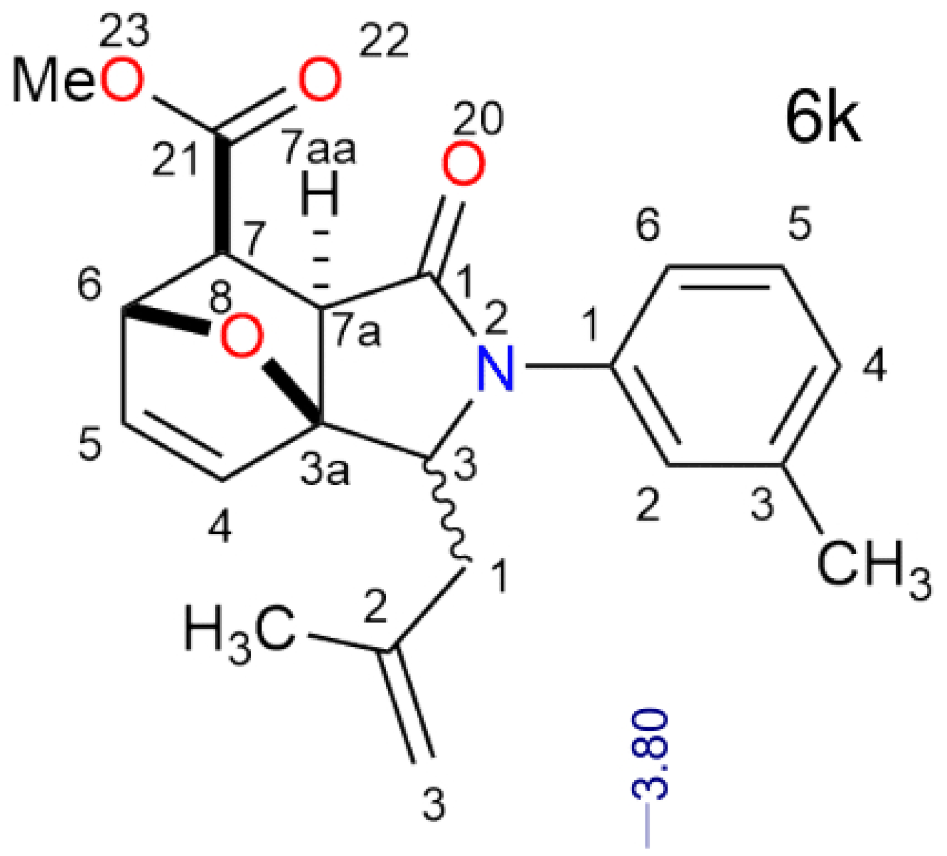

FZ7648-1.esp

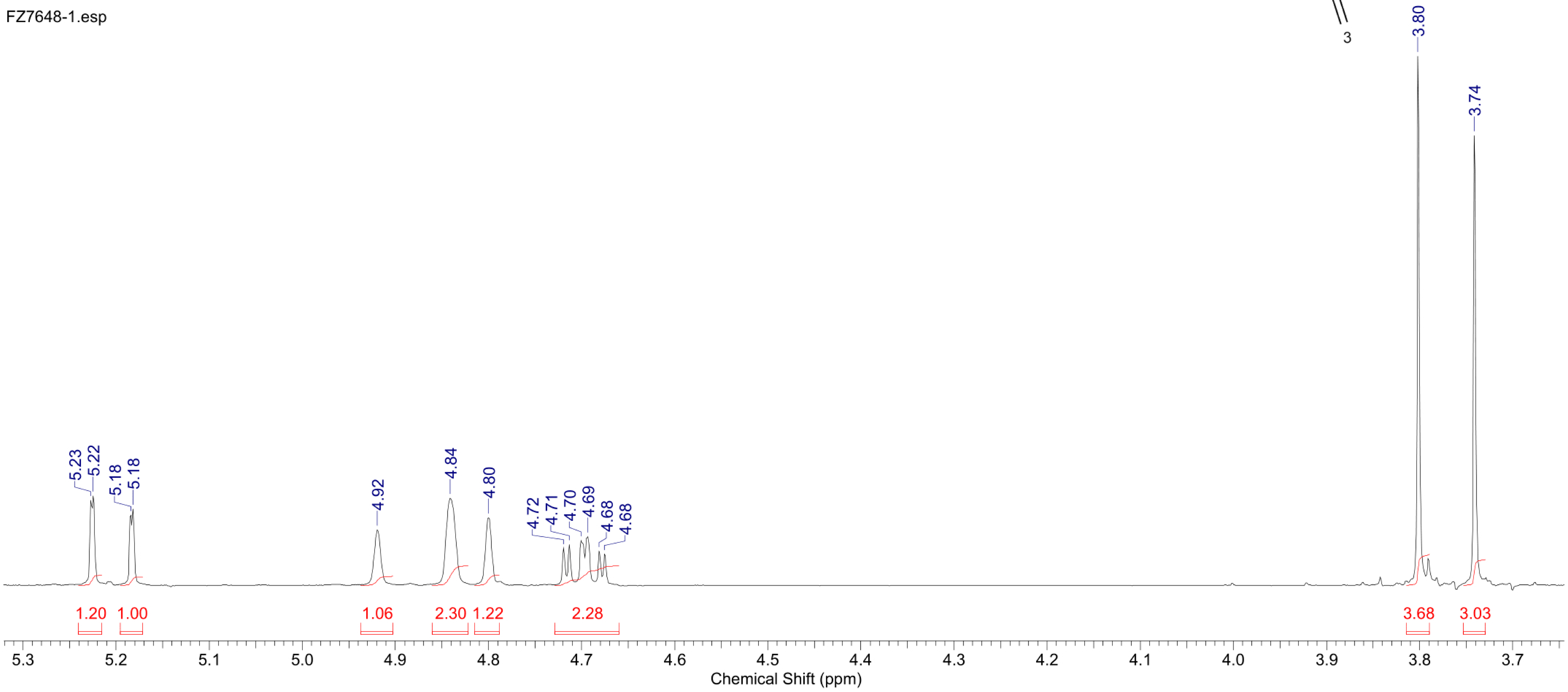

**Acquisition Time (sec)**	1.9818	**Comment**	single_pulse	**Date**	28 May 1990 08:08:29				
**Date Stamp**	07 Aug 2019 14:11:55			**File Name**	C:\Users\Fedor\Desktop\07.08.19\FZ7648-1.jdf			**Frequency (MHz)**	600.17
**Nucleus**	1H	**Number of Transients**	8	**Origin**	ECA 600	**Original Points Count**	32768	**Owner**	delta
**Points Count**	32768	**Pulse Sequence**	single_pulse.ex2			**Receiver Gain**	44.00	**Solvent**	CHLOROFORM-d
**Spectrum Offset (Hz)**	5410.1226	**Sweep Width (Hz)**	16534.39	**Temperature (degree C)**	22.100				

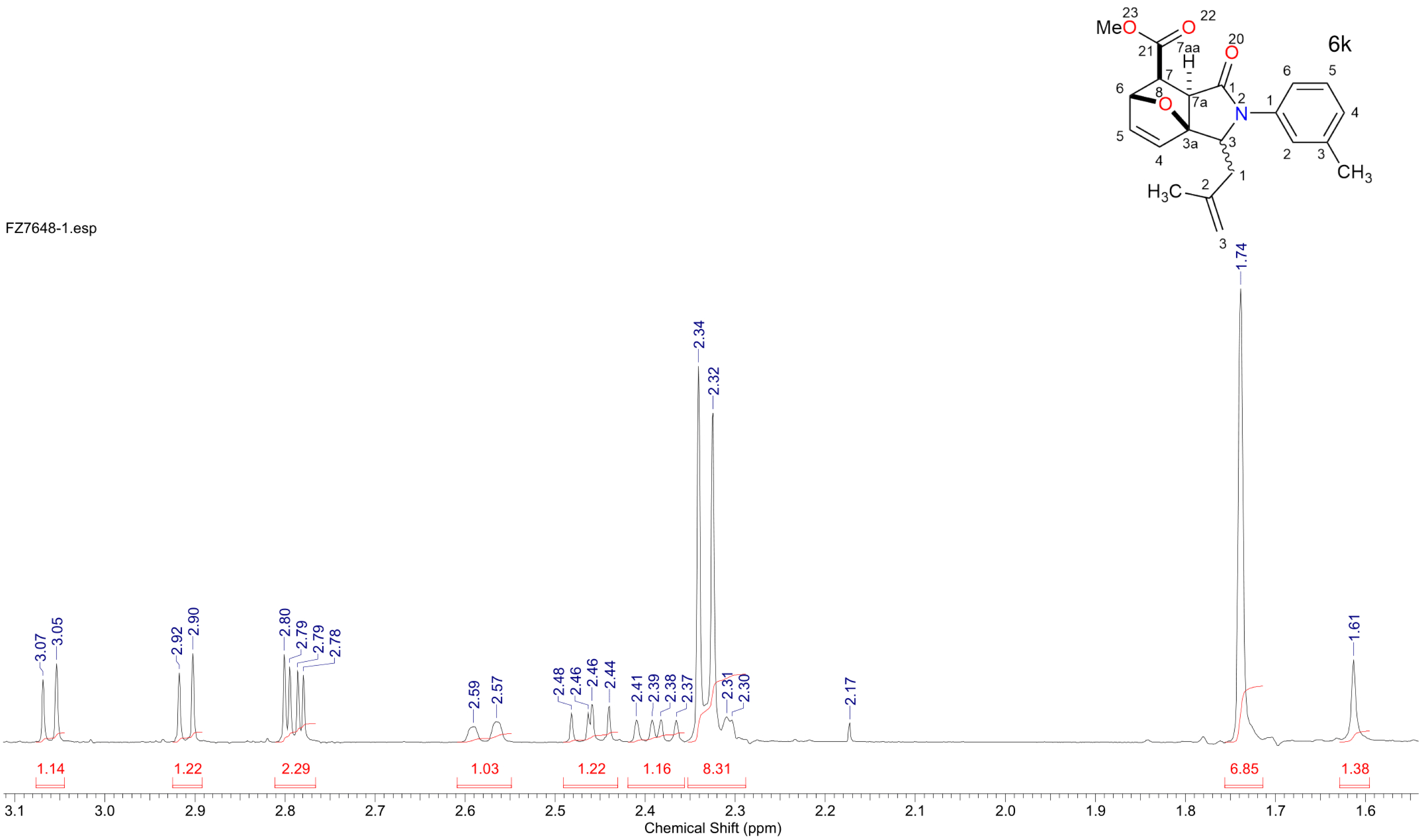

14.07.2020 18:49:17

**Formula** $C_{21}H_{23}NO_4$	**FW** 353.4116

Acquisition Time (sec)	0.6921	***Comment***	single pulse decoupled gated NOE	***Date***	12 Aug 1990 02:57:57
Date Stamp	22 Oct 2019 09:01:04	***File Name***	C:\USERS\Лаба534\DOWNLOADS\FZ8006-1.JDF		
Frequency (MHz)	150.91	***Nucleus***	13C	***Number of Transients***	800
Origin	ECA 600	***Original Points Count***	32768	***Owner***	delta
Points Count	32768	***Pulse Sequence***	single_pulse_dec	***Receiver Gain***	54.00
Solvent	CHLOROFORM-d	***Spectrum Offset (Hz)***	15079.3525	***Sweep Width (Hz)***	47348.49
Temperature (degree C)	24.300				

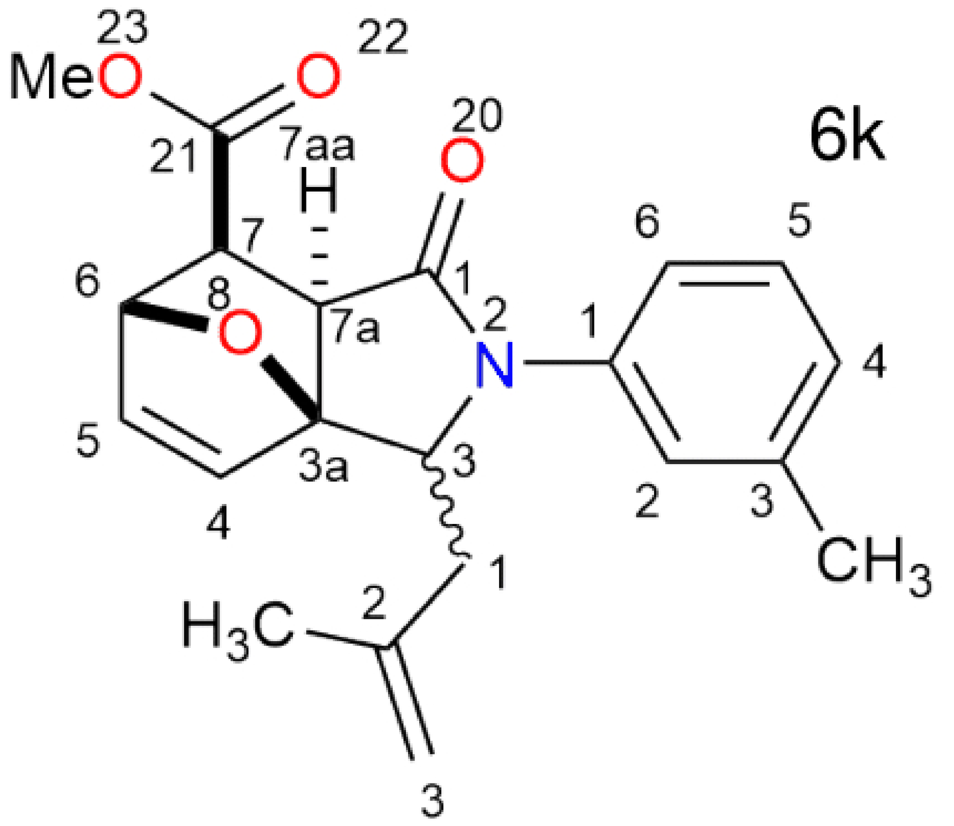

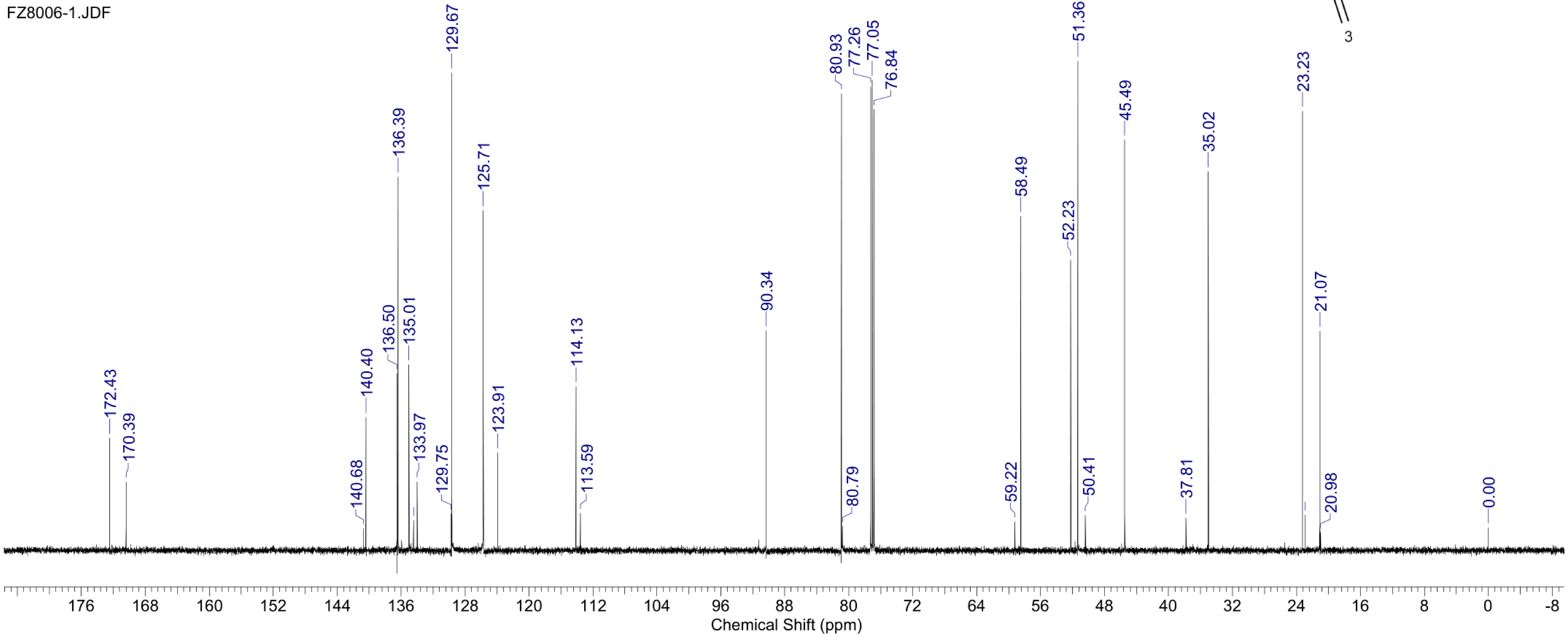

14.07.2020 18:49:24

Formula	$C_{21}H_{23}NO_4$	FW	353.4116

**Acquisition Time (sec)**	0.6921	**Comment**	single pulse decoupled gated NOE	**Date**	12 Aug 1990 02:57:57
**Date Stamp**	22 Oct 2019 09:01:04			**File Name**	C:\USERS\Лаба534\DOWNLOADS\FZ8006-1.JDF
**Frequency (MHz)**	150.91	**Nucleus**	13C	**Number of Transients**	800
**Origin**	ECA 600				
**Original Points Count**	32768	**Owner**	delta	**Points Count**	32768
**Pulse Sequence**	single_pulse_dec				
**Receiver Gain**	54.00	**Solvent**	CHLOROFORM-d	**Spectrum Offset (Hz)**	15079.3525
**Sweep Width (Hz)**	47348.49	**Temperature (degree C)**	24.300		

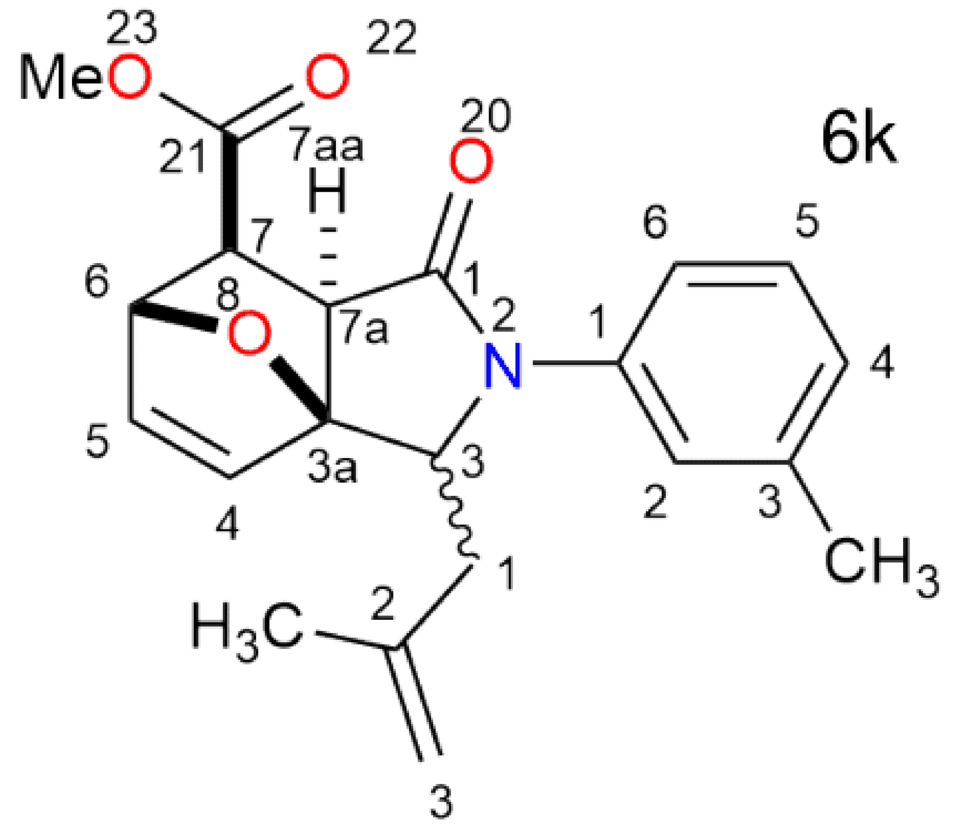

FZ8006-1.JDF

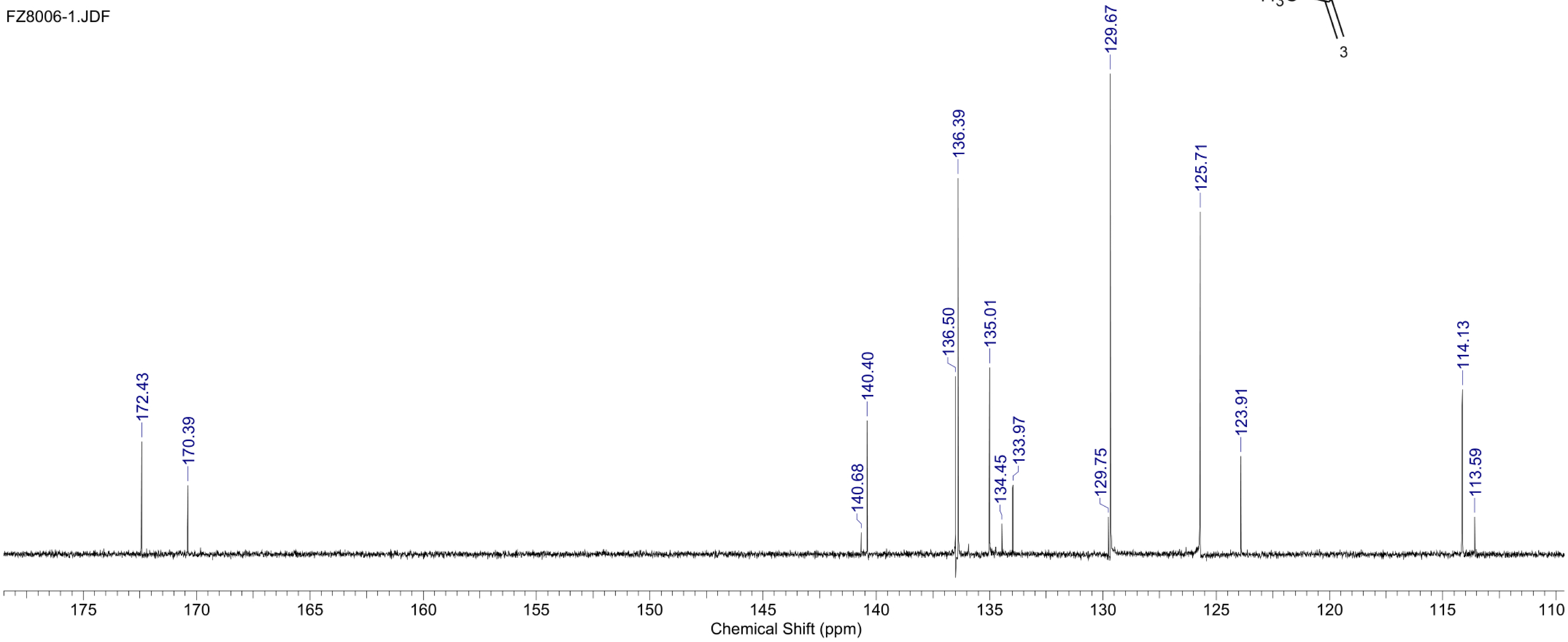

14.07.2020 18:49:32

**Formula**	$C_{21}H_{23}NO_4$	**FW**	353.4116

Acquisition Time (sec)	0.6921	***Comment***	single pulse decoupled gated NOE			***Date***	12 Aug 1990 02:57:57
Date Stamp	22 Oct 2019 09:01:04			***File Name***	C:\USERS\Лаба534\DOWNLOADS\FZ8006-1.JDF		
Frequency (MHz)	150.91	***Nucleus***	13C	***Number of Transients***	800	***Origin***	ECA 600
Original Points Count	32768	***Owner***	delta	***Points Count***	32768	***Pulse Sequence***	single_pulse_dec
Receiver Gain	54.00	***Solvent***	CHLOROFORM-d			***Spectrum Offset (Hz)***	15079.3525
Sweep Width (Hz)	47348.49	***Temperature (degree C)***	24.300				

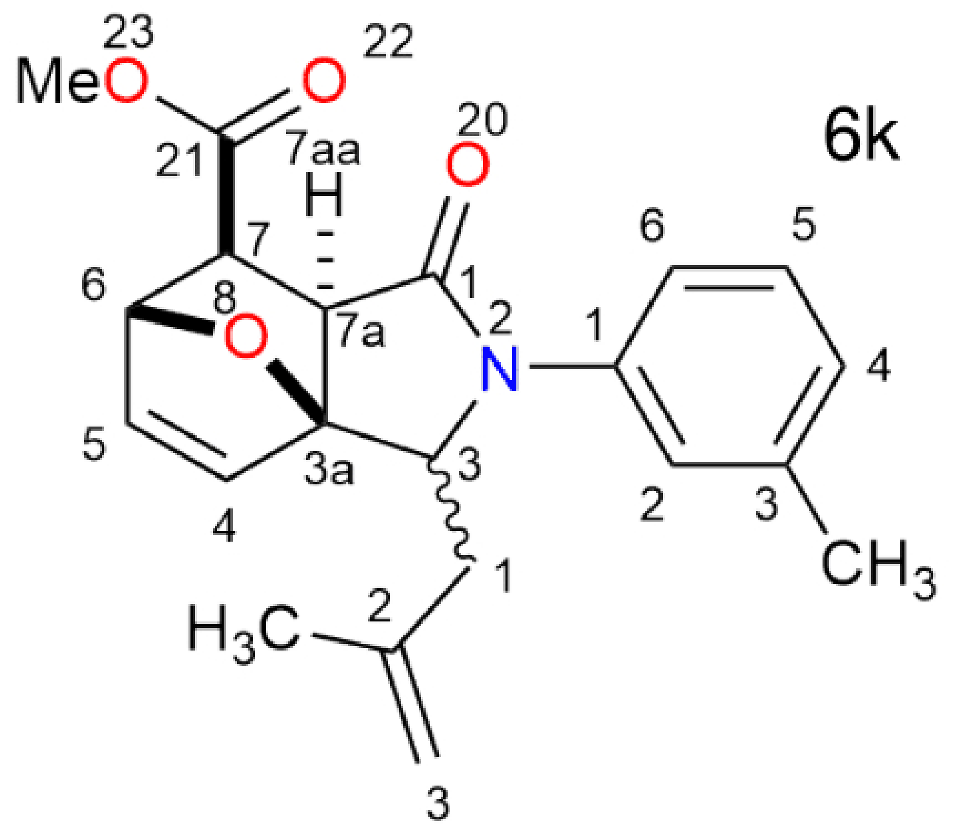

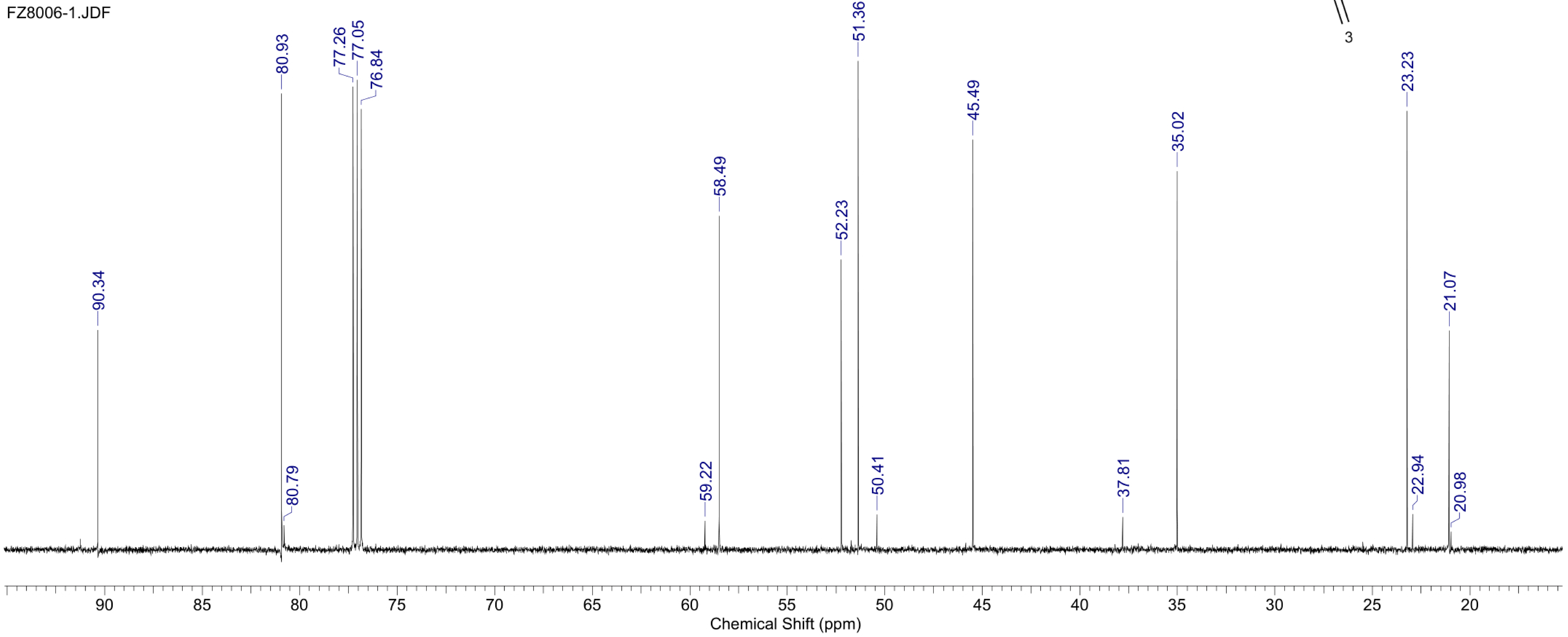

DEPT-135 NMR spectrum for compound 6k

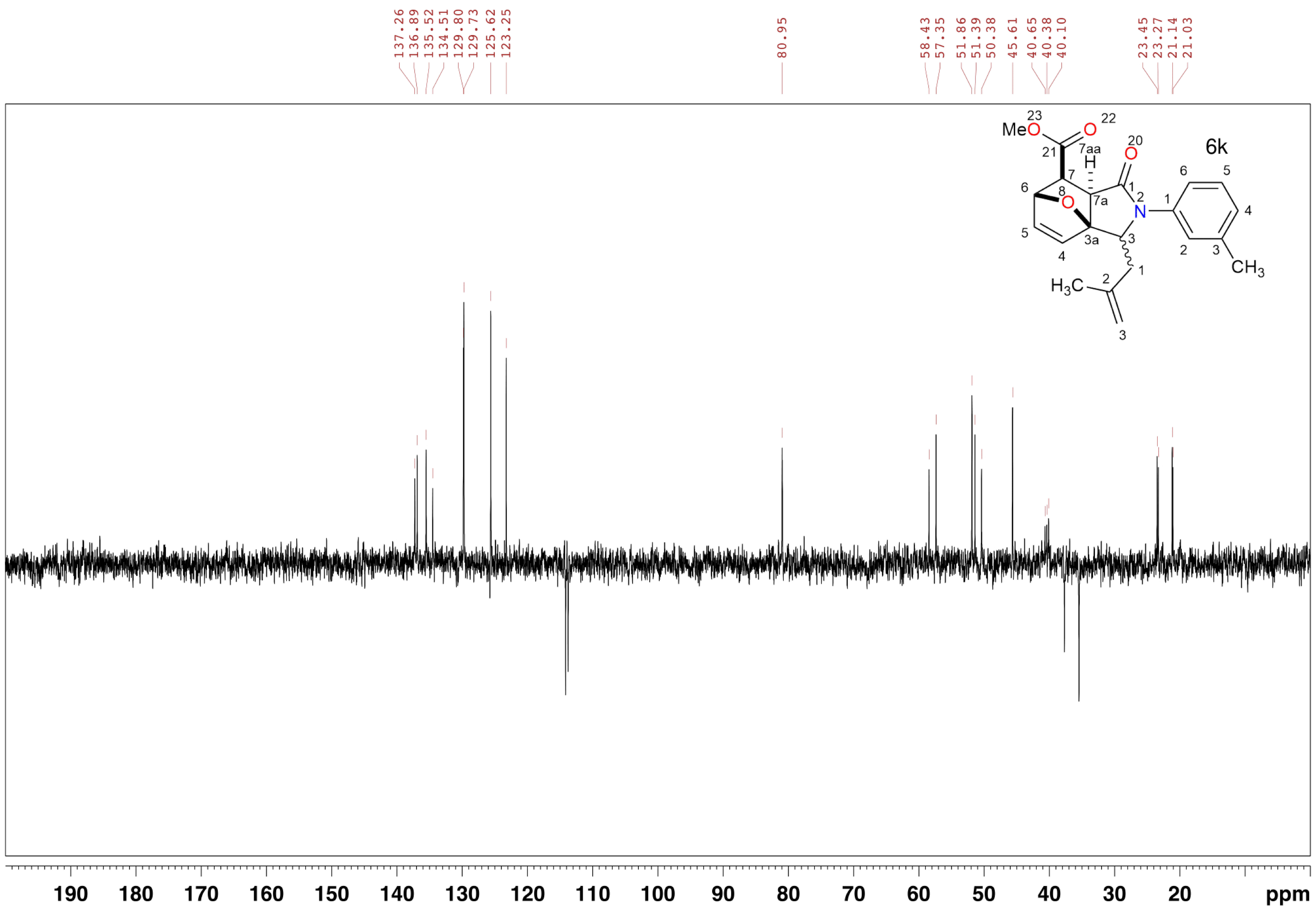

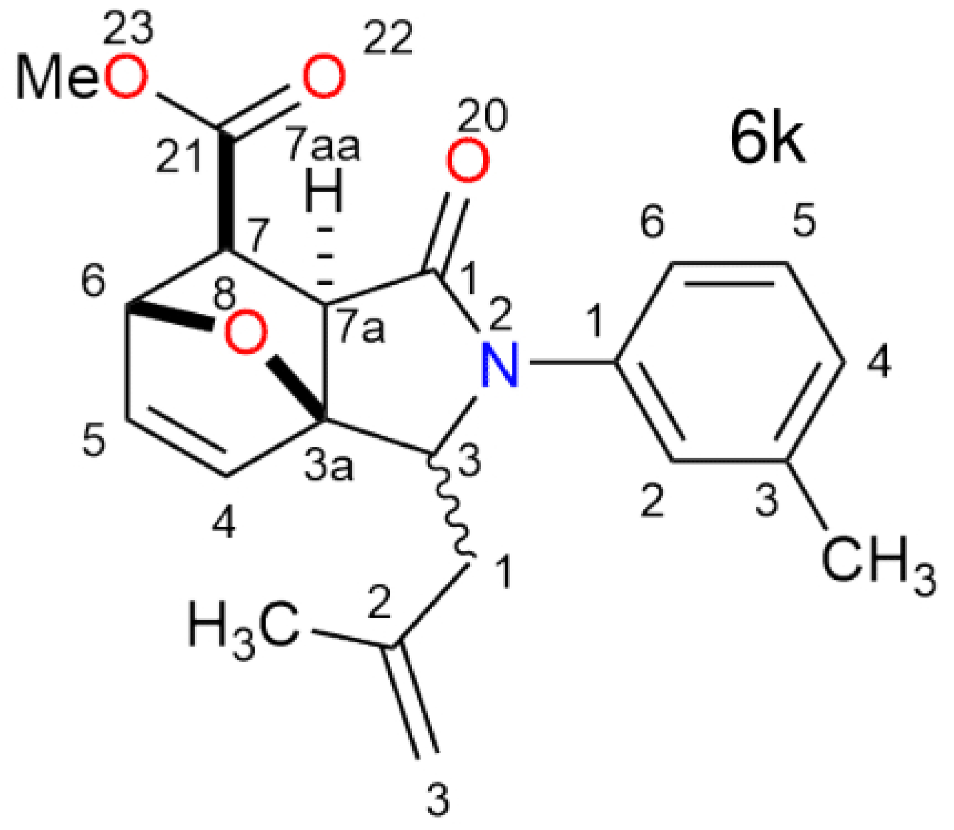

NOESY NMR spectrum for compound 6k

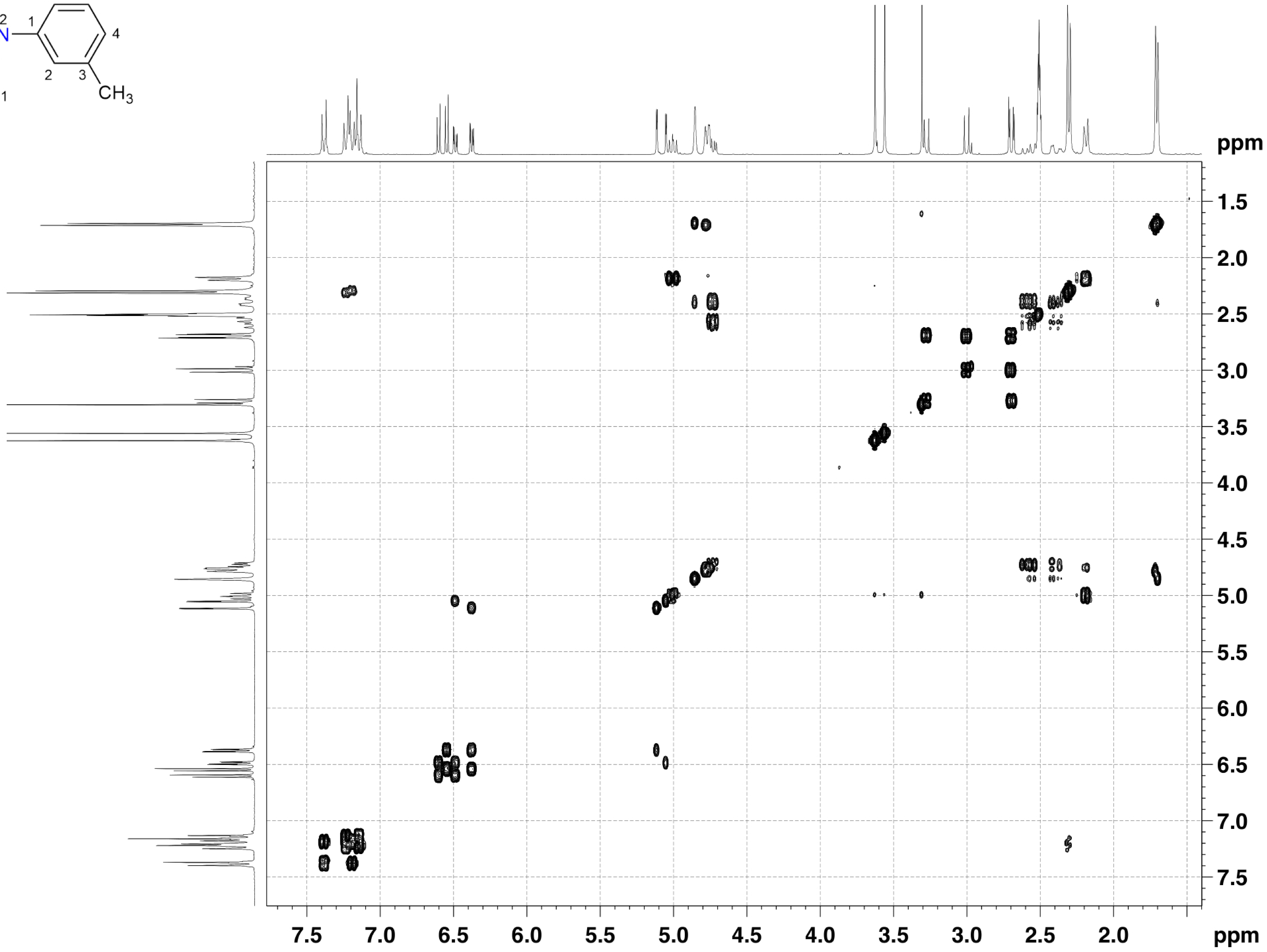

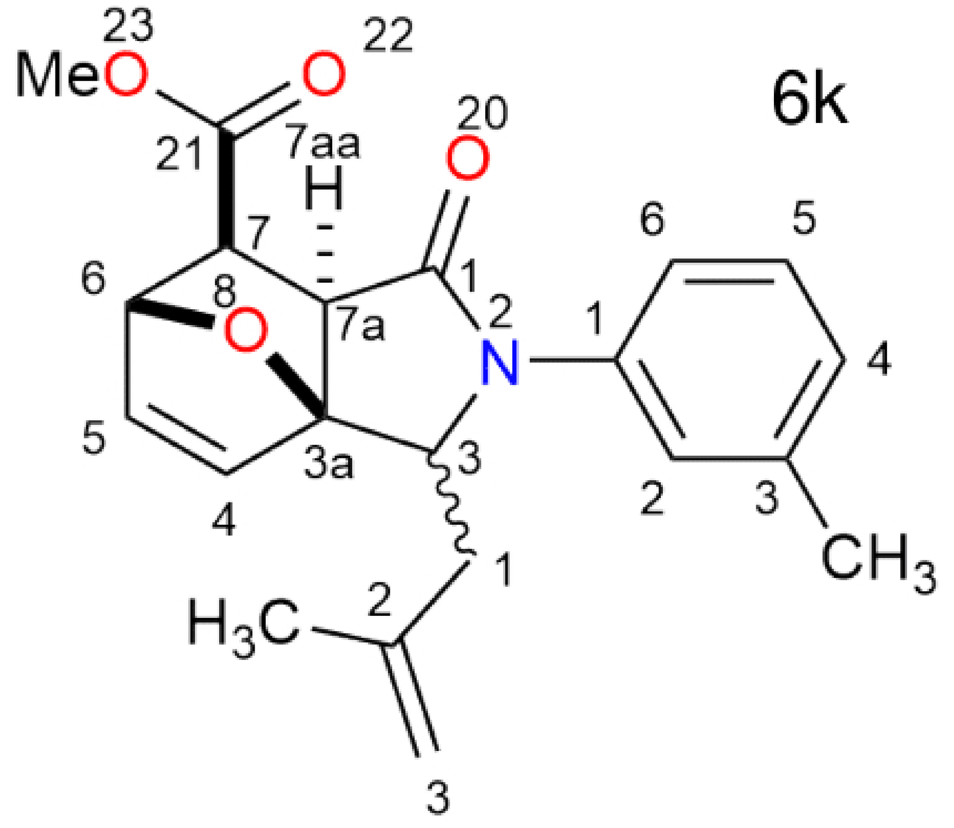

6k

HSQC NMR spectrum for compound 6k

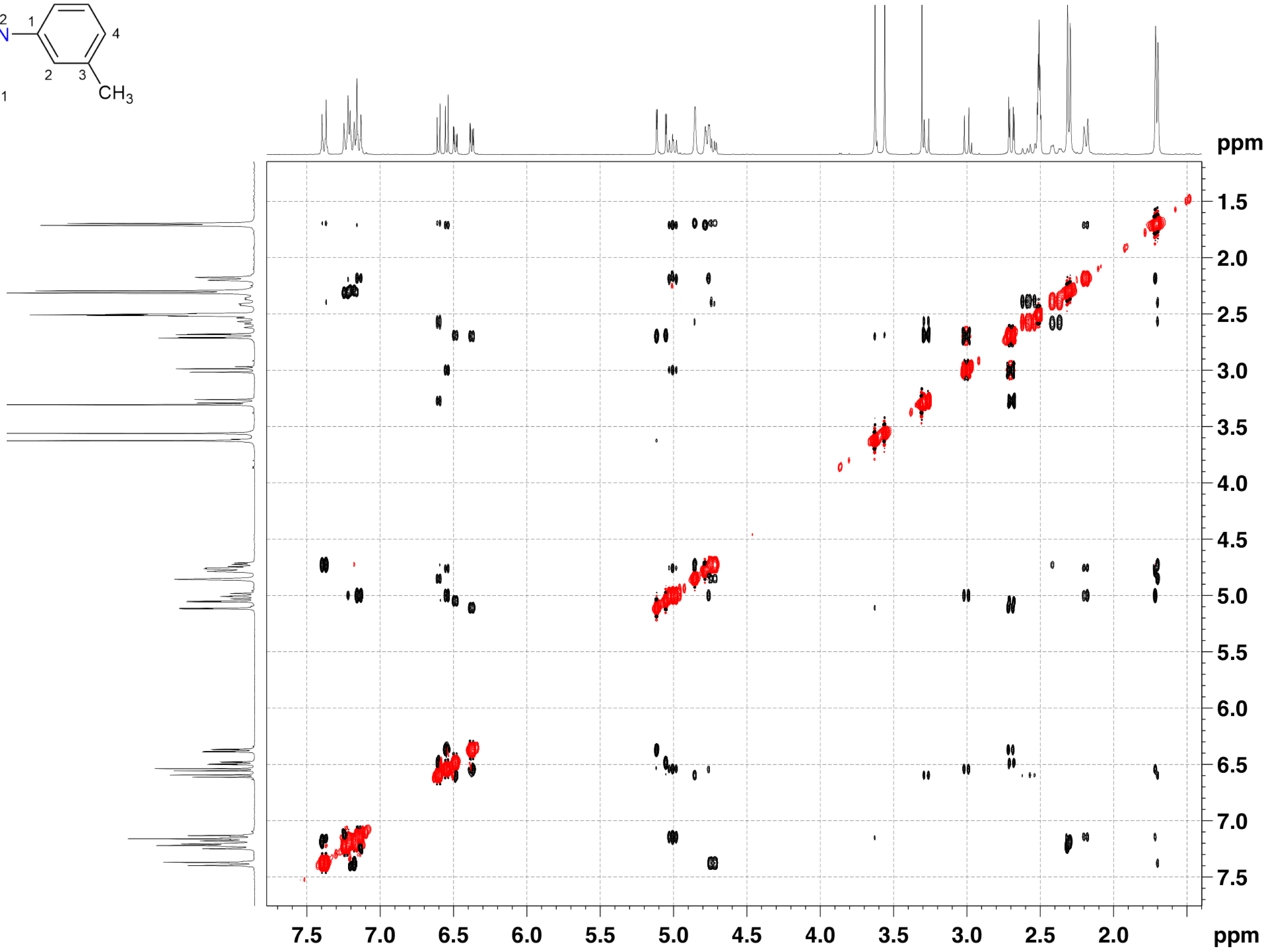

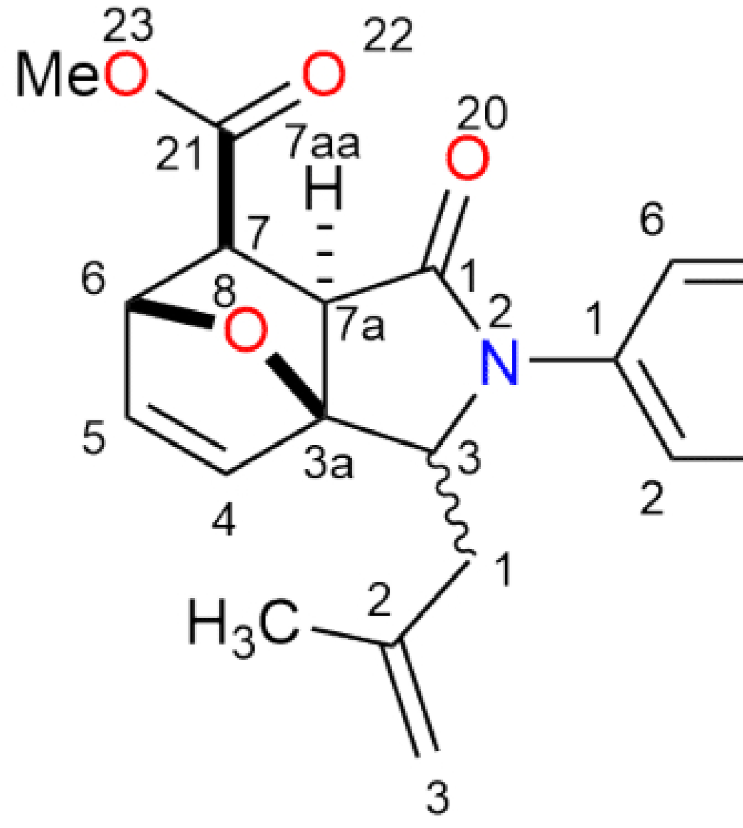
MeO
O
O
N
O
H
CH3
H3C

6k

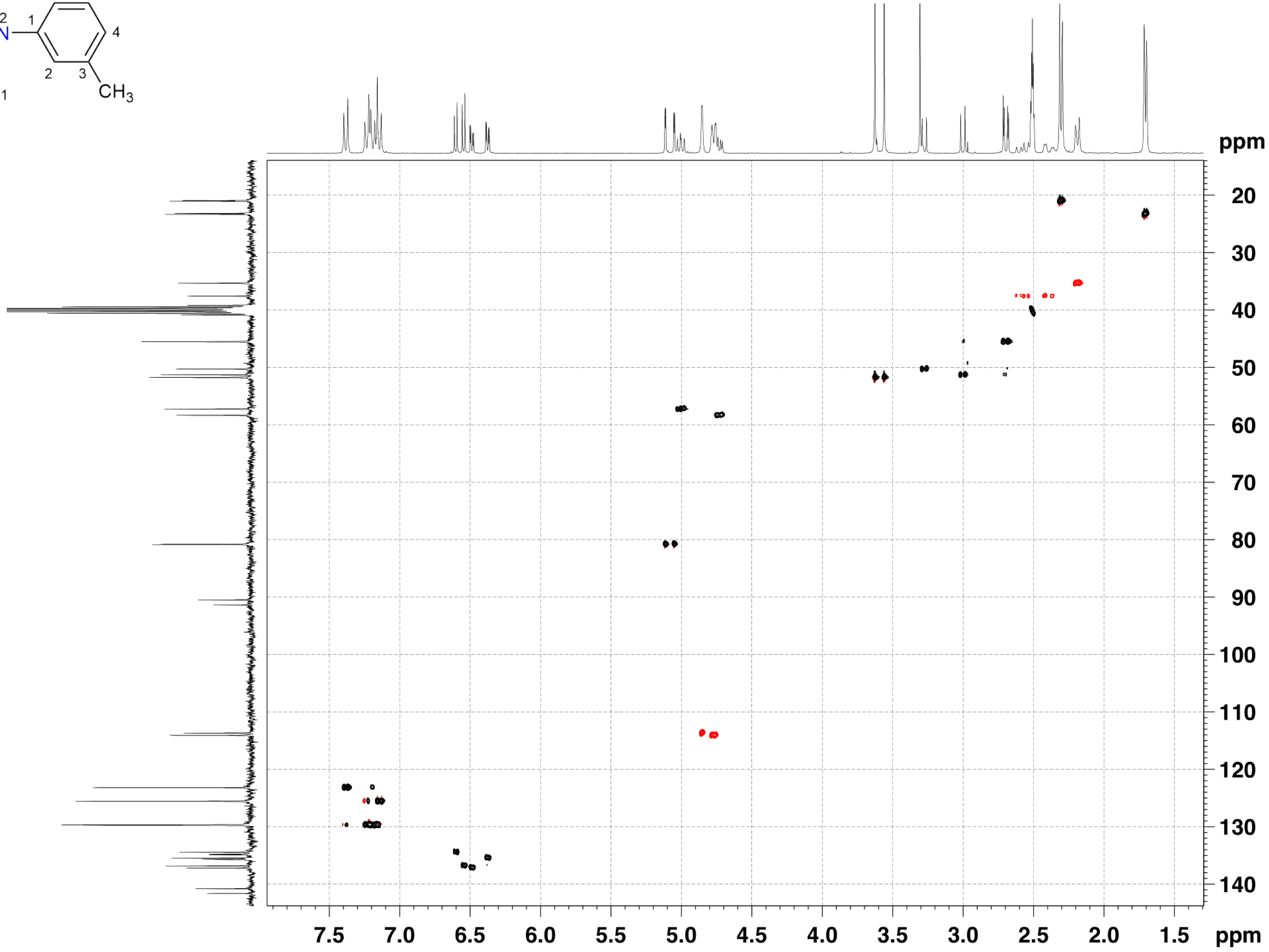
ppm
20
30
40
50
60
70
80
90
100
110
120
130
140
ppm
7.5
7.0
6.5
6.0
5.5
5.0
4.5
4.0
3.5
3.0
2.5
2.0
1.5
ppm

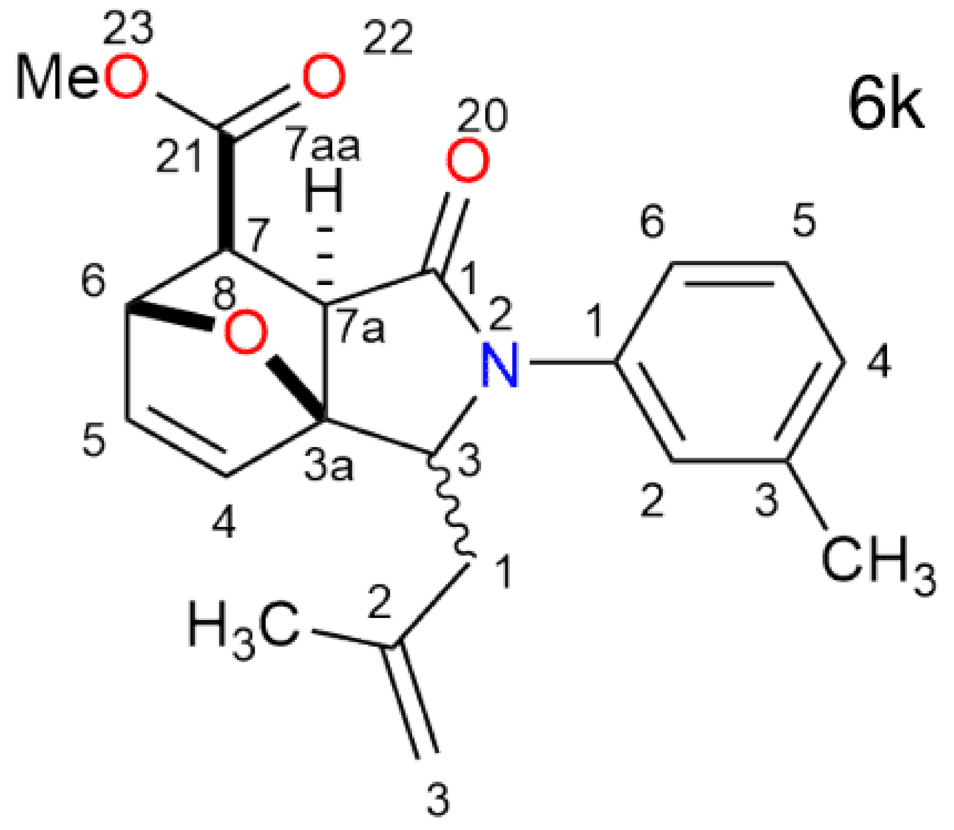
MeO
23
22
21
7aa
H
20
7
6
8
7a
1
2
N
6
5
4
5
4
3a
3
2
3
CH3
1
H3C
2
3
6k

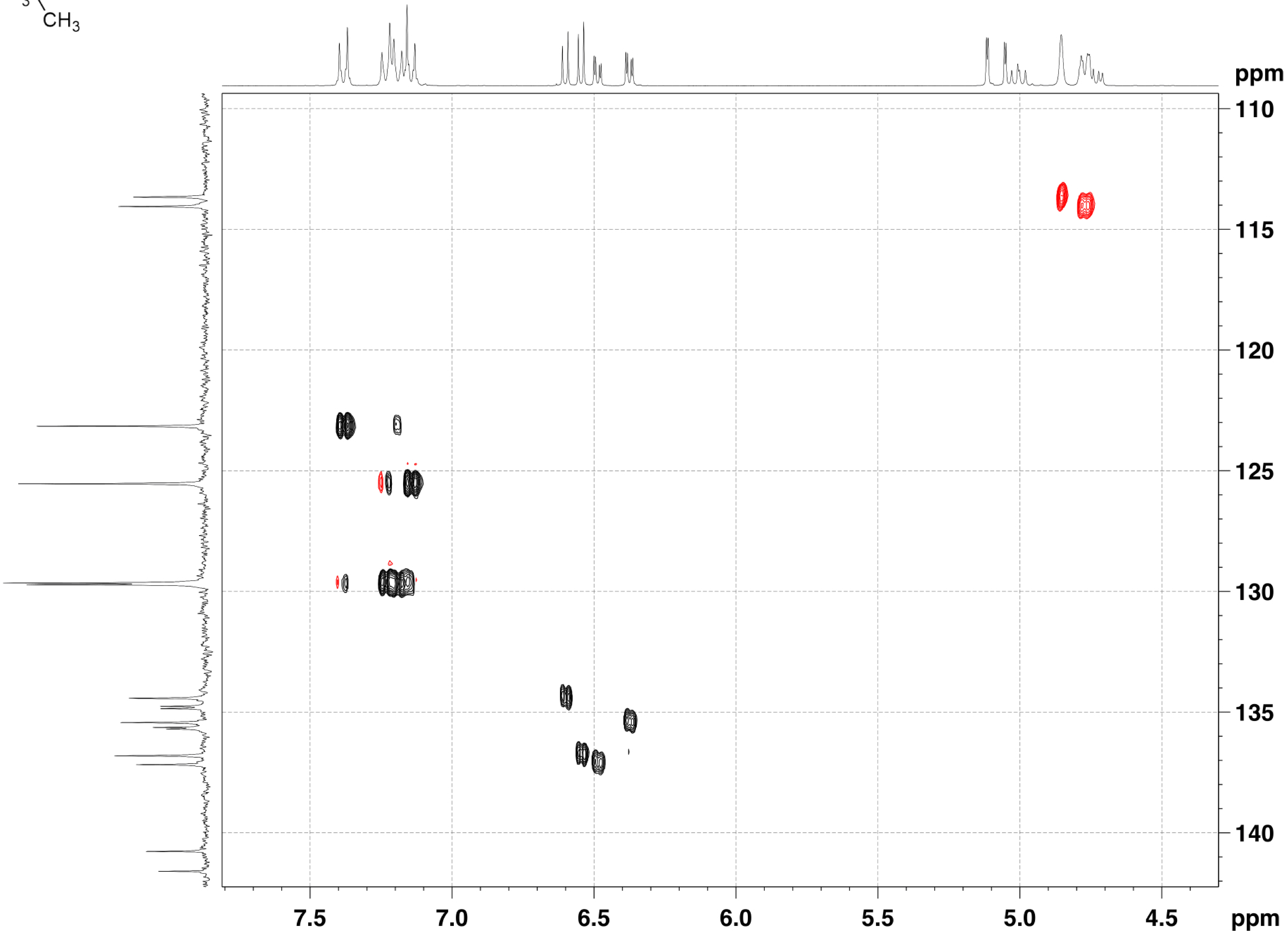
ppm
110
115
120
125
130
135
140
7.5
7.0
6.5
6.0
5.5
5.0
4.5
ppm

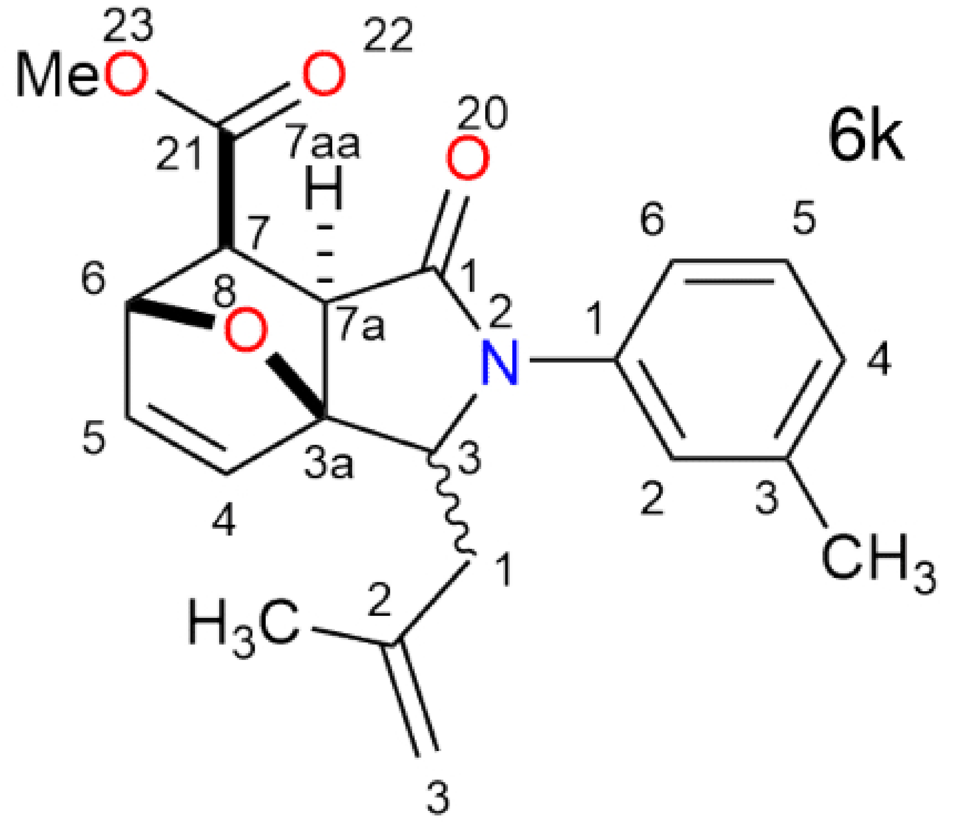
MeO
O
O
N
O
H
CH3
H3C
6k

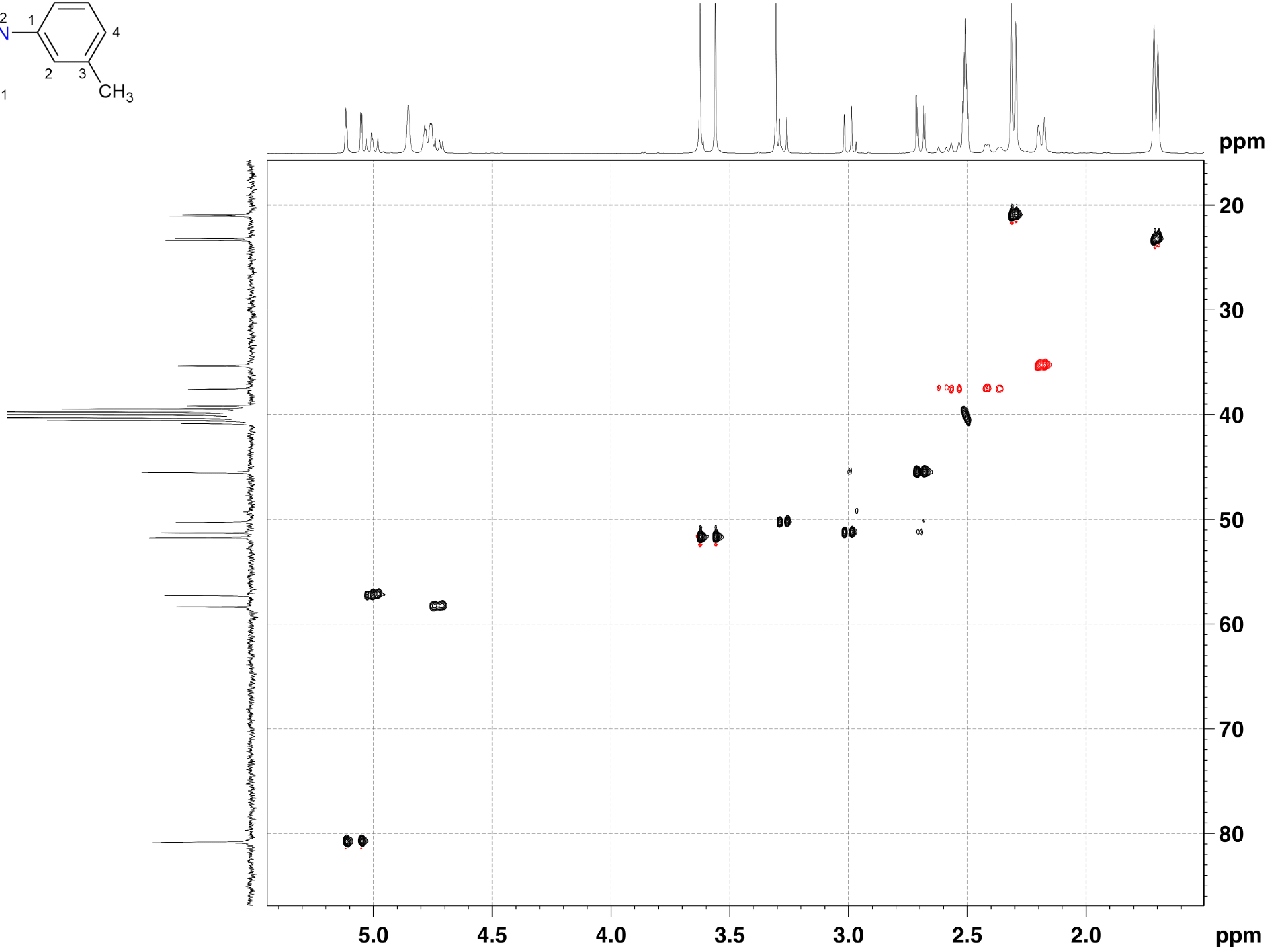
ppm
5.0
4.5
4.0
3.5
3.0
2.5
2.0
ppm
20
30
40
50
60
70
80

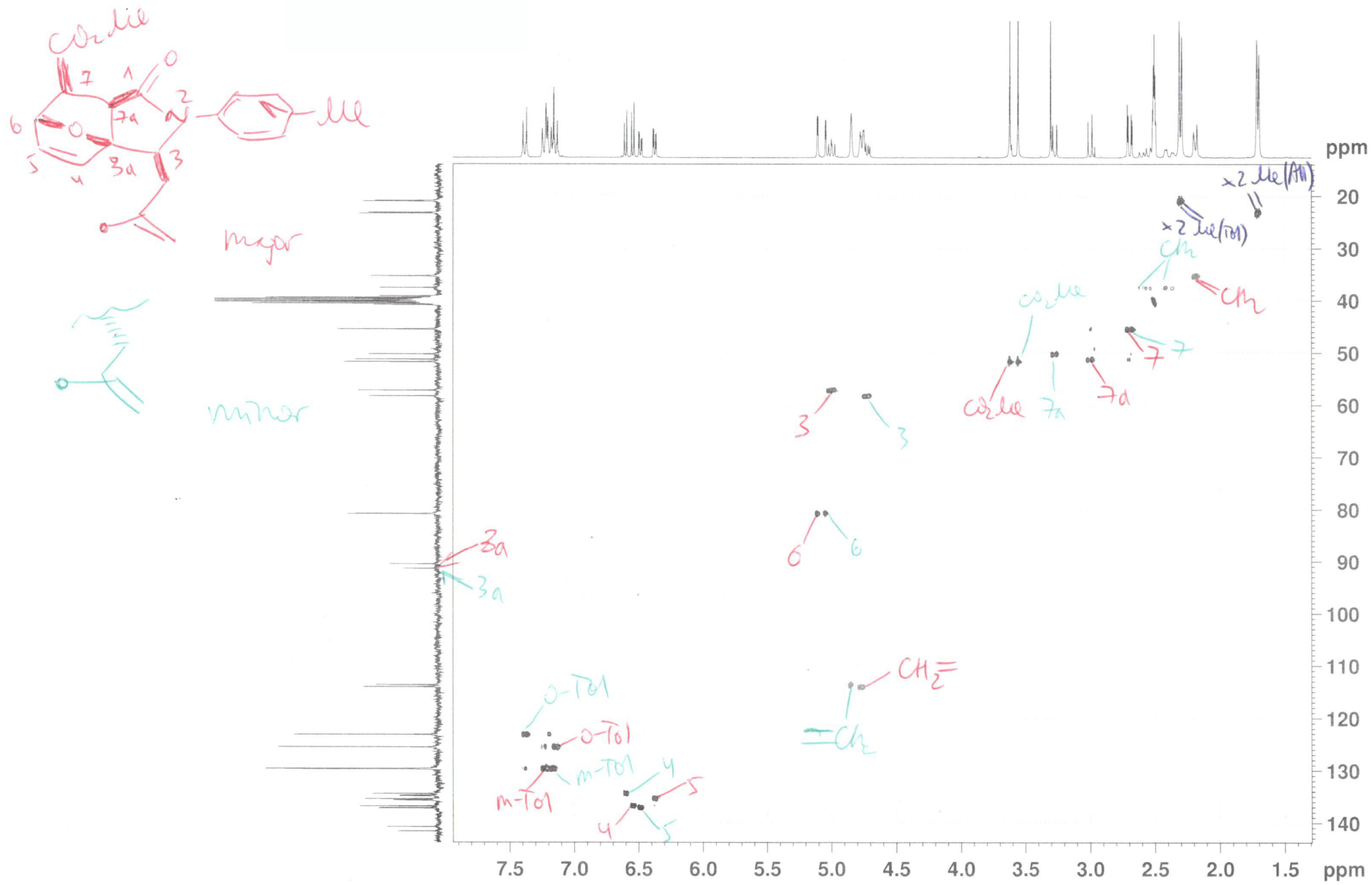

ppm
7.5
7.0
6.5
6.0
5.5
5.0
4.5
4.0
3.5
3.0
2.5
2.0
1.5
20
30
40
50
60
70
80
90
100
110
120
130
140

NMR spectrum data for compound 6l

Acquisition Time (sec)	1.9818	***Comment***	single_pulse	***Date***	13 May 1990 05:00:53		
Date Stamp	23 Jul 2019 11:04:26			***File Name***	C:\Users\Fedor\Desktop\22.07.19\FZ7588-1.jdf	***Frequency (MHz)***	600.17
Nucleus	1H	***Number of Transients***	8	***Origin***	ECA 600 ***Original Points Count*** 32768	***Owner***	delta
Points Count	32768	***Pulse Sequence***	single_pulse.ex2		***Receiver Gain*** 40.00	***Solvent***	CHLOROFORM-d
Spectrum Offset (Hz)	5409.6182	***Sweep Width (Hz)***	16534.39	***Temperature (degree C)***	22.800		

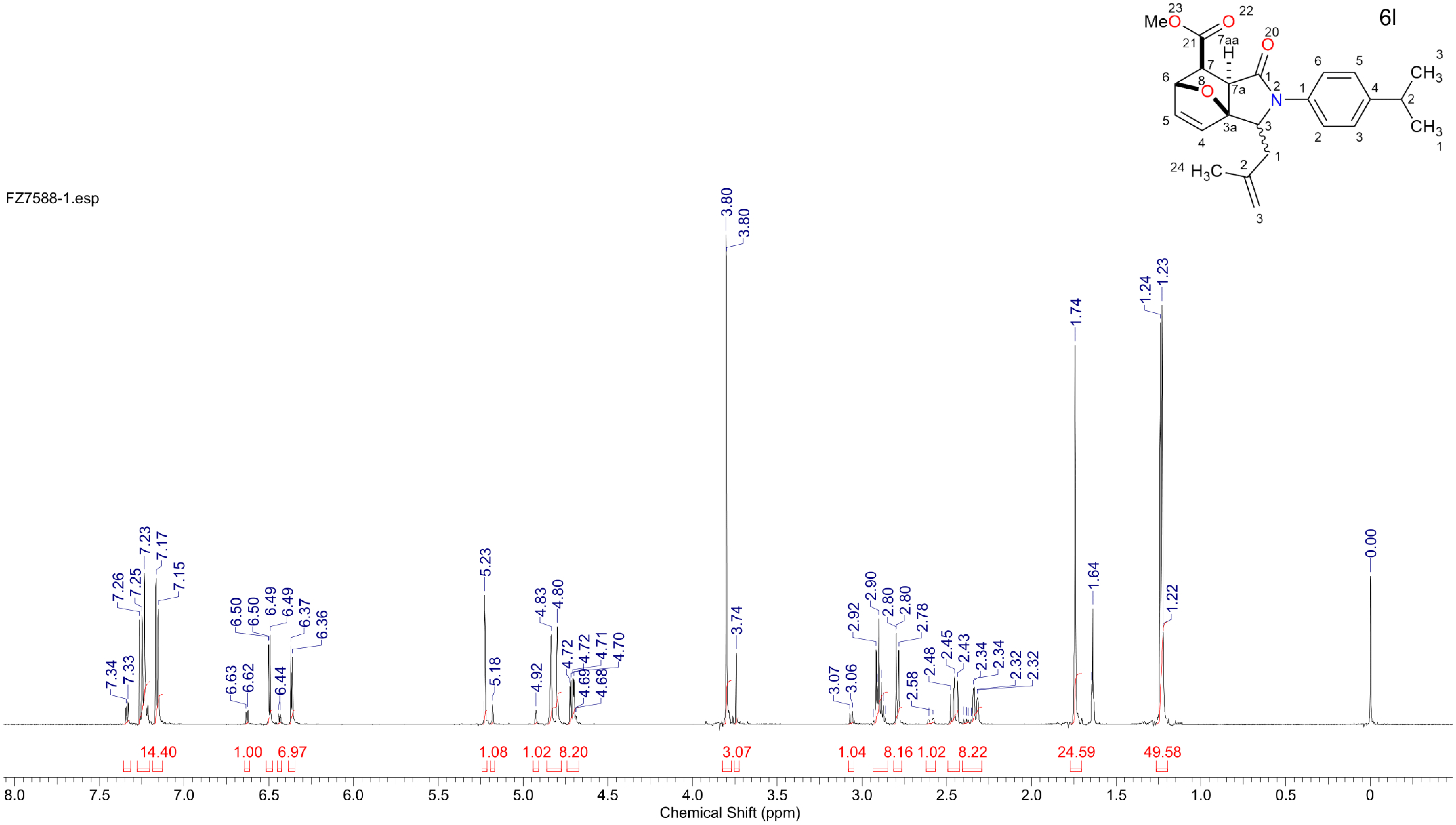

Acquisition Time (sec)	1.9818	***Comment***	single_pulse	***Date***	13 May 1990 05:00:53				
Date Stamp	23 Jul 2019 11:04:26			***File Name***	C:\Users\Fedor\Desktop\22.07.19\FZ7588-1.jdf			***Frequency (MHz)***	600.17
Nucleus	1H	***Number of Transients***	8	***Origin***	ECA 600	***Original Points Count***	32768	***Owner***	delta
Points Count	32768	***Pulse Sequence***	single_pulse.ex2			***Receiver Gain***	40.00	***Solvent***	CHLOROFORM-d
Spectrum Offset (Hz)	5409.6182	***Sweep Width (Hz)***	16534.39	***Temperature (degree C)***	22.800				

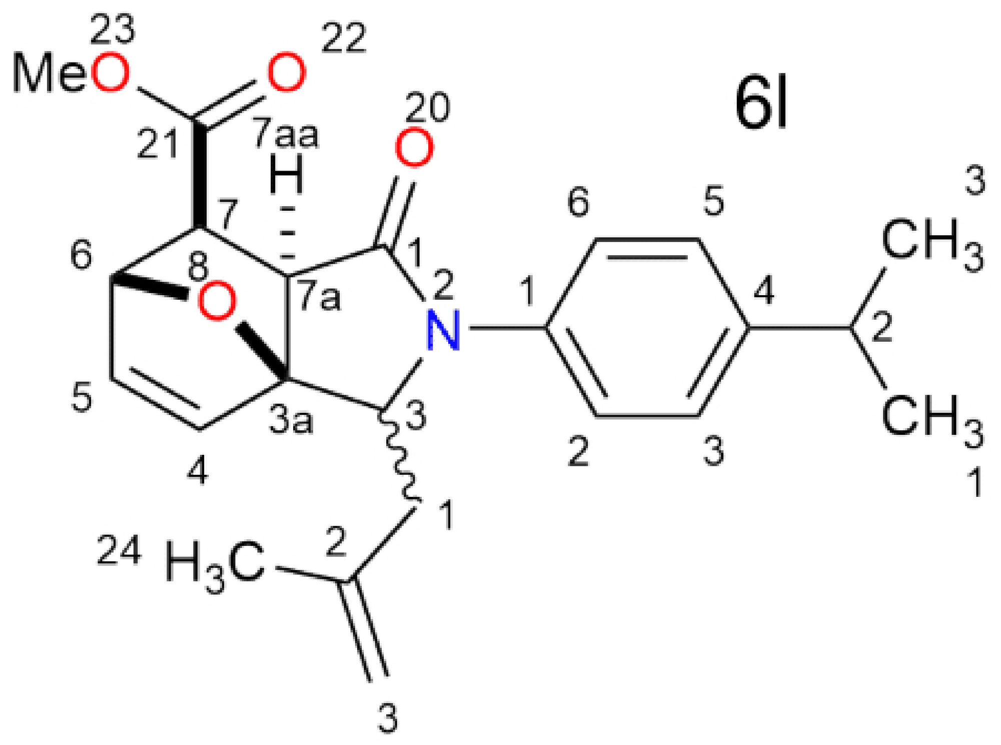

6l

FZ7588-1.esp

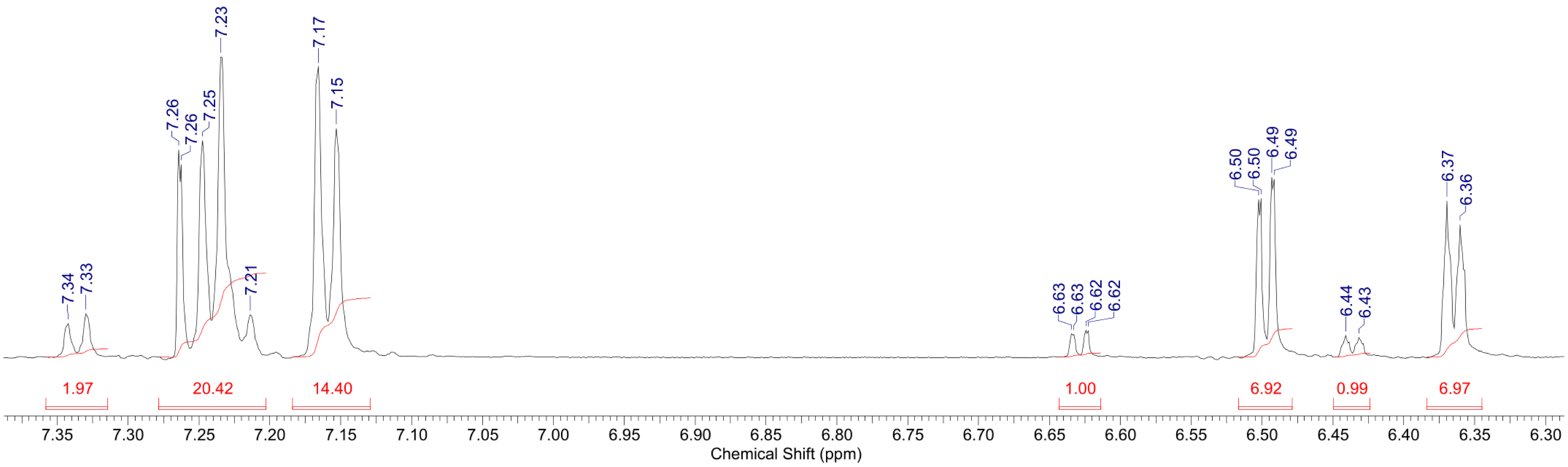

**Acquisition Time (sec)**	1.9818	**Comment**	single_pulse	**Date**	13 May 1990 05:00:53		
**Date Stamp**	23 Jul 2019 11:04:26			**File Name**	C:\Users\Fedor\Desktop\22.07.19\FZ7588-1.jdf	**Frequency (MHz)**	600.17
**Nucleus**	1H	**Number of Transients**	8	**Origin**	ECA 600	**Original Points Count** 32768	**Owner** delta
**Points Count**	32768	**Pulse Sequence**	single_pulse.ex2			**Receiver Gain** 40.00	**Solvent** CHLOROFORM-d
**Spectrum Offset (Hz)**	5409.6182	**Sweep Width (Hz)**	16534.39	**Temperature (degree C)**	22.800		

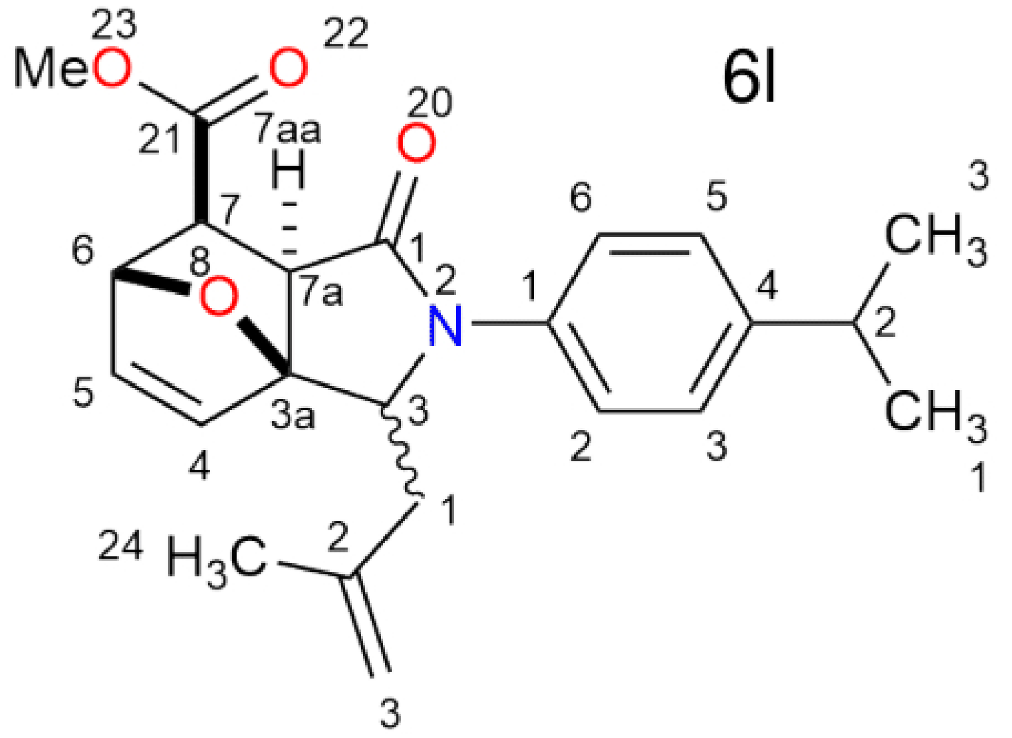

FZ7588-1.esp

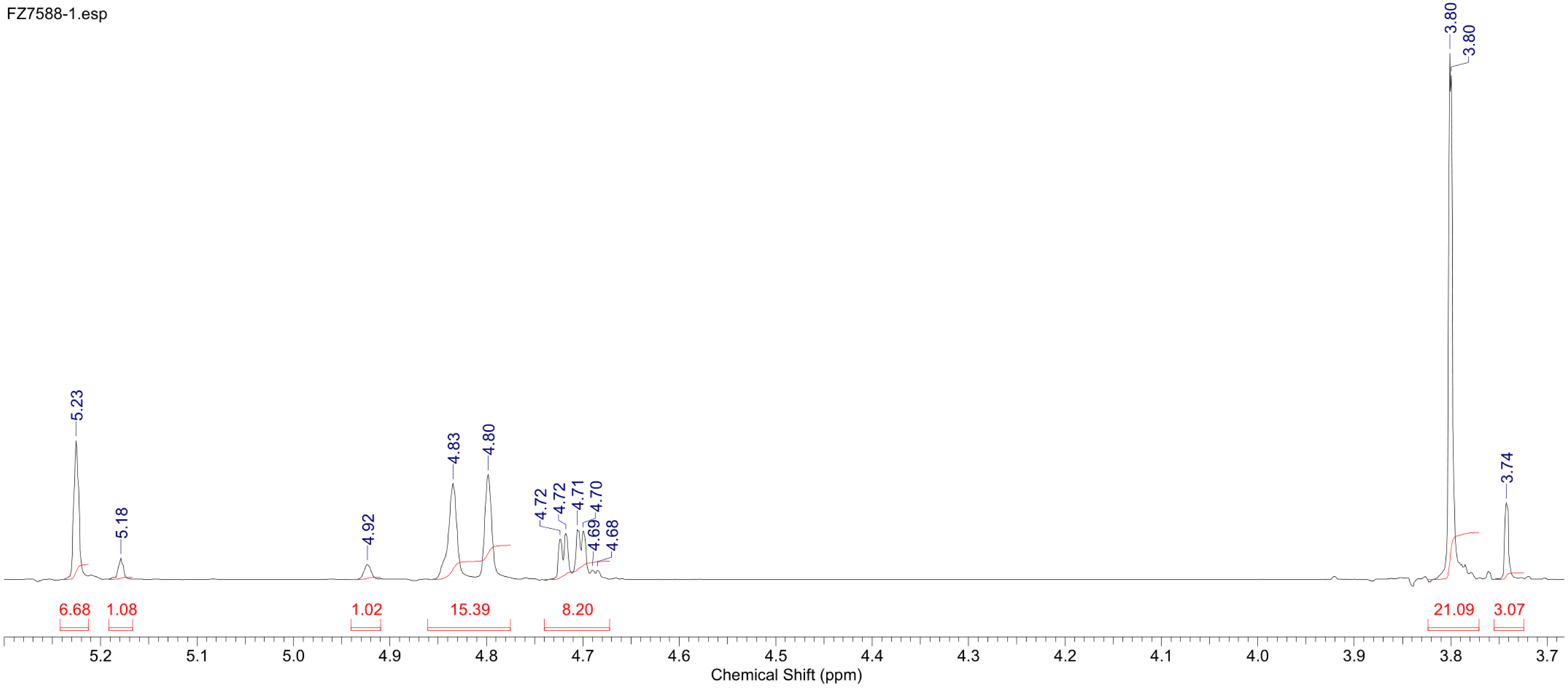

Acquisition Time (sec)	1.9818	***Comment***	single_pulse	***Date***	13 May 1990 05:00:53		
Date Stamp	23 Jul 2019 11:04:26			***File Name***	C:\Users\Fedor\Desktop\22.07.19\FZ7588-1.jdf	***Frequency (MHz)***	600.17
Nucleus	1H	***Number of Transients***	8	***Origin***	ECA 600	***Original Points Count*** 32768	***Owner*** delta
Points Count	32768	***Pulse Sequence***	single_pulse.ex2			***Receiver Gain*** 40.00	***Solvent*** CHLOROFORM-d
Spectrum Offset (Hz)	5409.6182	***Sweep Width (Hz)***	16534.39	***Temperature (degree C)***	22.800		

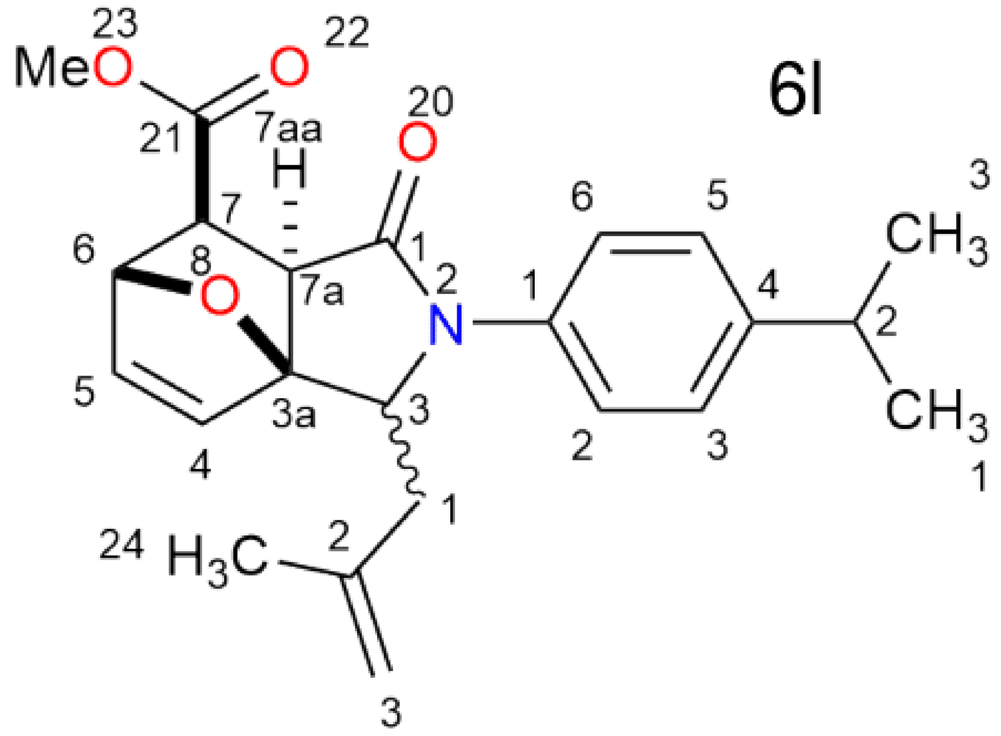

6l

FZ7588-1.esp

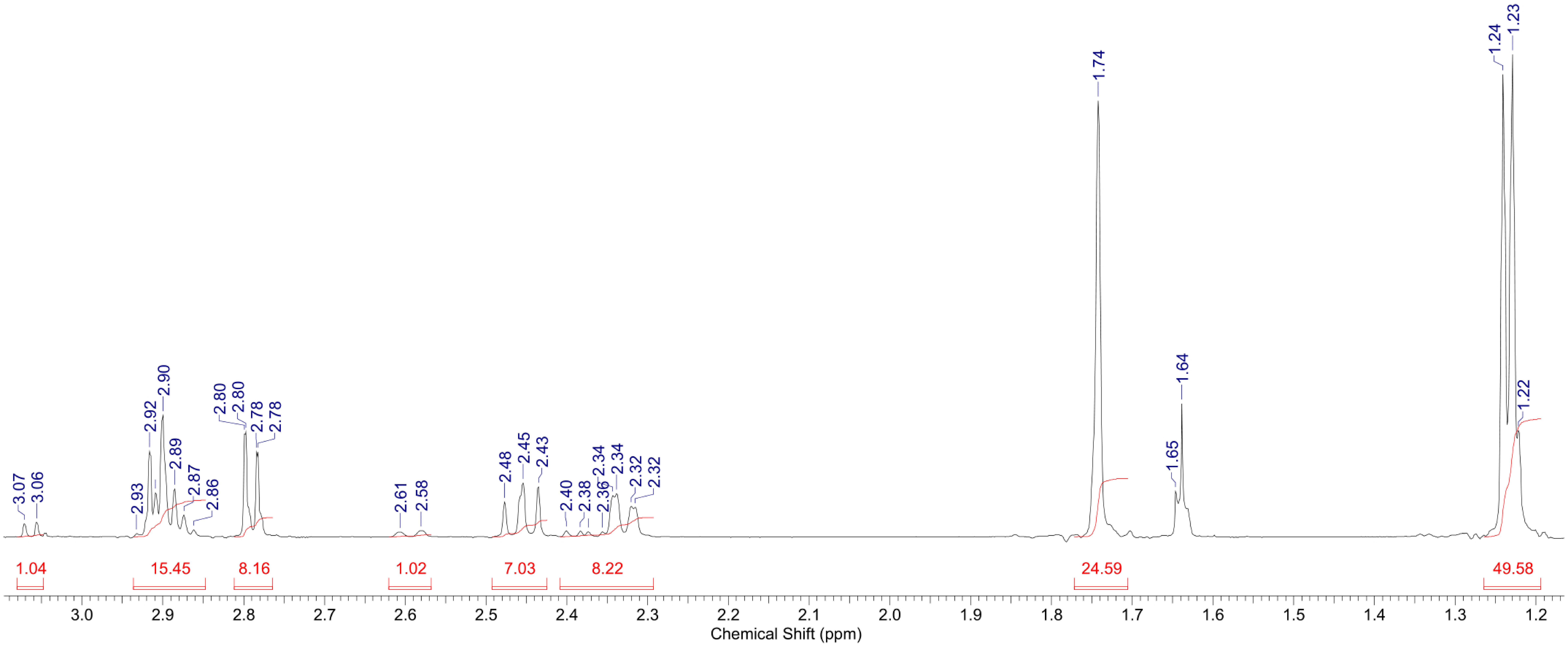

14.07.2020 18:47:07

Formula $C_{23}H_{27}NO_4$	***FW*** 381.4648		

Acquisition Time (sec)	0.6921	***Comment***	single pulse decoupled gated NOE	***Date***	12 Aug 1990 03:19:12
Date Stamp	22 Oct 2019 09:22:19	***File Name***	C:\USERS\Лаба534\DOWNLOADS\FZ8005-1.JDF		
Frequency (MHz)	150.91	***Nucleus***	13C	***Number of Transients***	800
Origin	ECA 600				
Original Points Count	32768	***Owner***	delta	***Points Count***	32768
Pulse Sequence	single_pulse_dec				
Receiver Gain	54.00	***Solvent***	CHLOROFORM-d	***Spectrum Offset (Hz)***	15091.3428
Sweep Width (Hz)	47348.49	***Temperature (degree C)***	24.500		

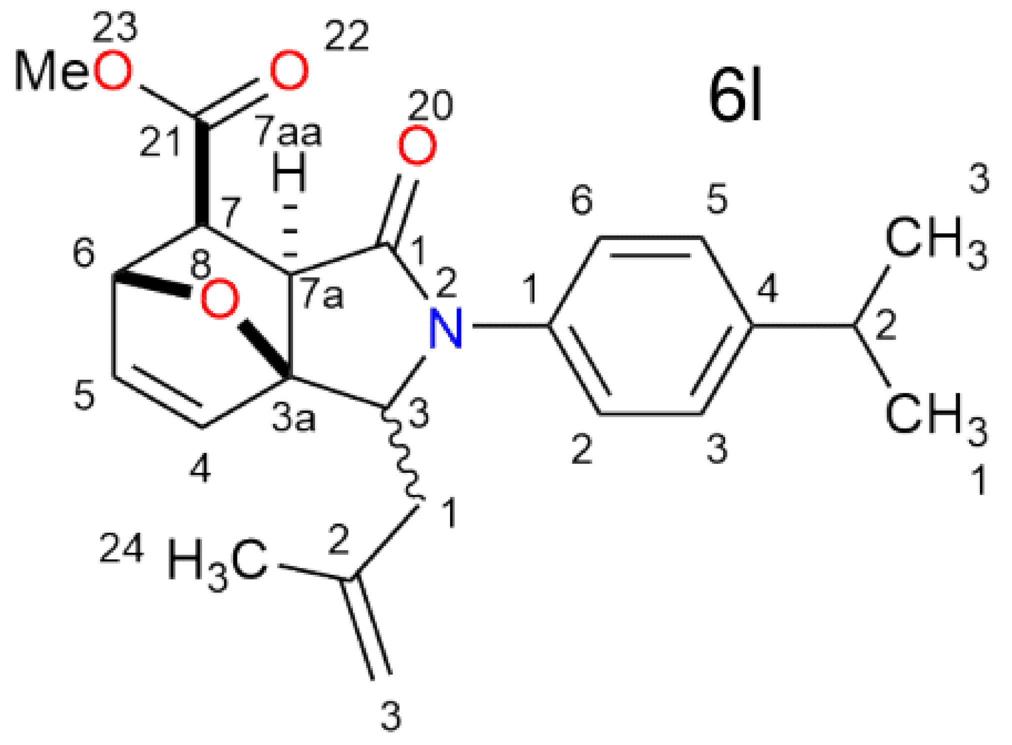

FZ8005-1.JDF

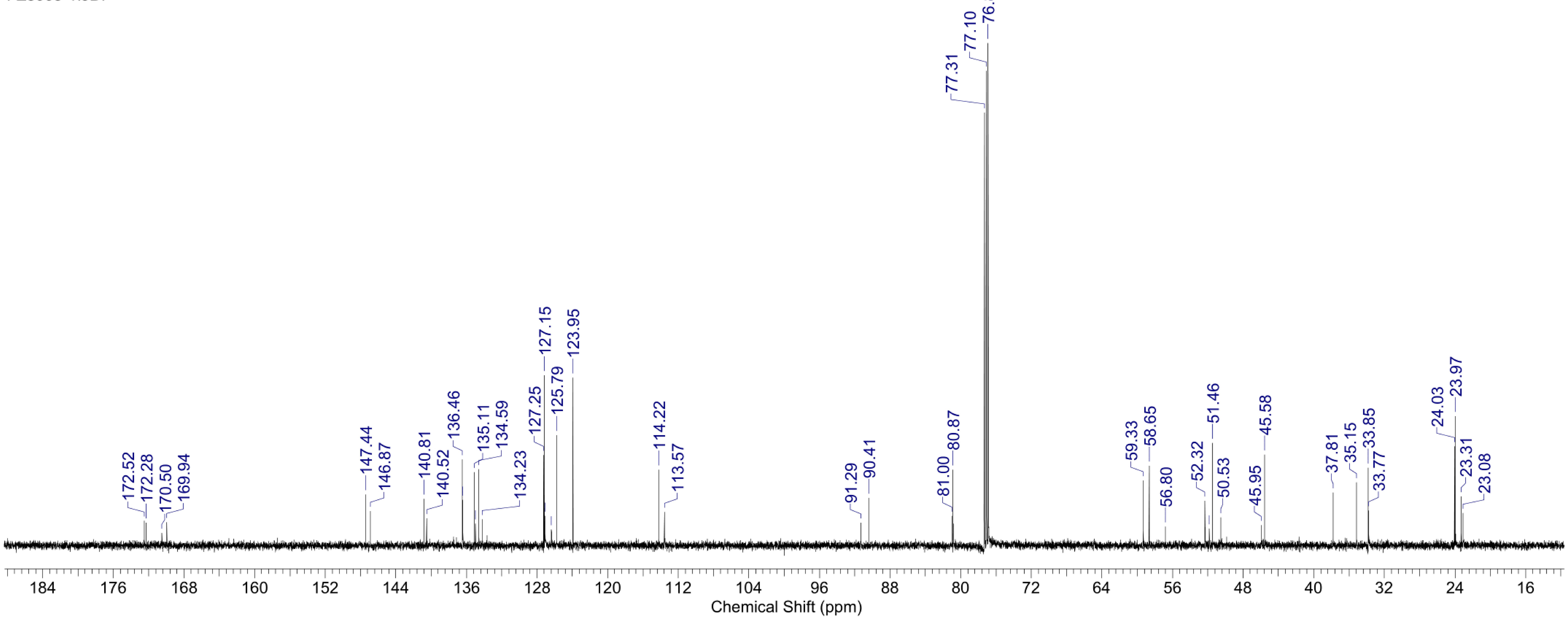

14.07.2020 18:47:16

Formula $C_{23}H_{27}NO_4$	FW 381.4648

**Acquisition Time (sec)**	0.6921	**Comment**	single pulse decoupled gated NOE			**Date**	12 Aug 1990 03:19:12
**Date Stamp**	22 Oct 2019 09:22:19			**File Name**	C:\USERS\Лаба534\DOWNLOADS\FZ8005-1.JDF		
**Frequency (MHz)**	150.91	**Nucleus**	13C	**Number of Transients**	800	**Origin**	ECA 600
**Original Points Count**	32768	**Owner**	delta	**Points Count**	32768	**Pulse Sequence**	single_pulse_dec
**Receiver Gain**	54.00	**Solvent**	CHLOROFORM-d			**Spectrum Offset (Hz)**	15091.3428
**Sweep Width (Hz)**	47348.49	**Temperature (degree C)**	24.500				

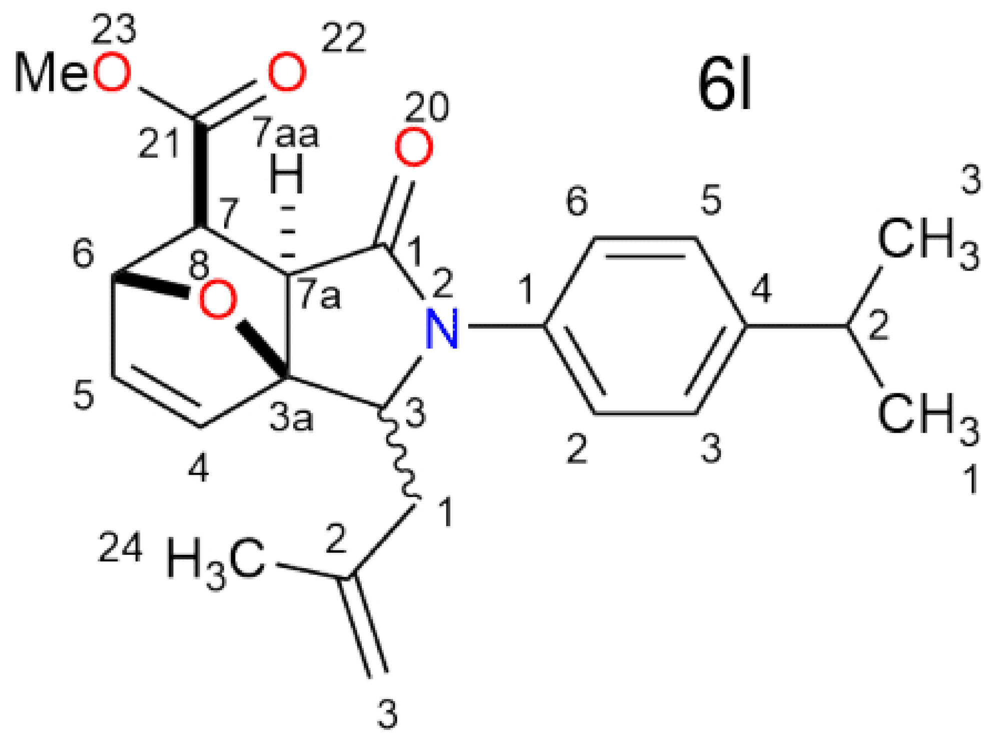

6l

FZ8005-1.JDF

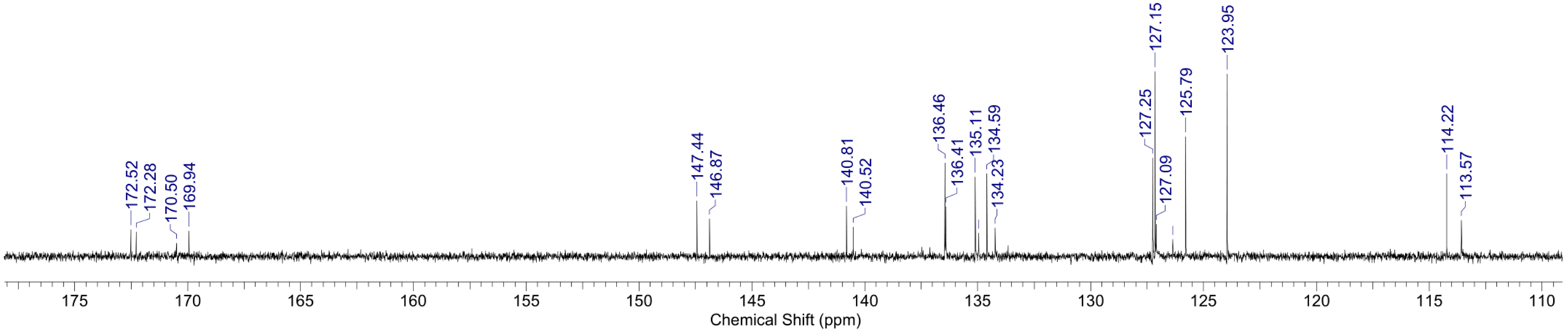

14.07.2020 18:47:25

**Formula** $C_{23}H_{27}NO_4$	**FW** 381.4648

**Acquisition Time (sec)**	0.6921	**Comment**	single pulse decoupled gated NOE	**Date**	12 Aug 1990 03:19:12
**Date Stamp**	22 Oct 2019 09:22:19	**File Name**	C:\USERS\Лаба534\DOWNLOADS\FZ8005-1.JDF		
**Frequency (MHz)**	150.91	**Nucleus**	13C	**Number of Transients**	800
**Origin**	ECA 600	**Original Points Count**	32768	**Owner**	delta
**Points Count**	32768	**Pulse Sequence**	single_pulse_dec	**Receiver Gain**	54.00
**Solvent**	CHLOROFORM-d	**Spectrum Offset (Hz)**	15091.3428	**Sweep Width (Hz)**	47348.49
**Temperature (degree C)**	24.500				

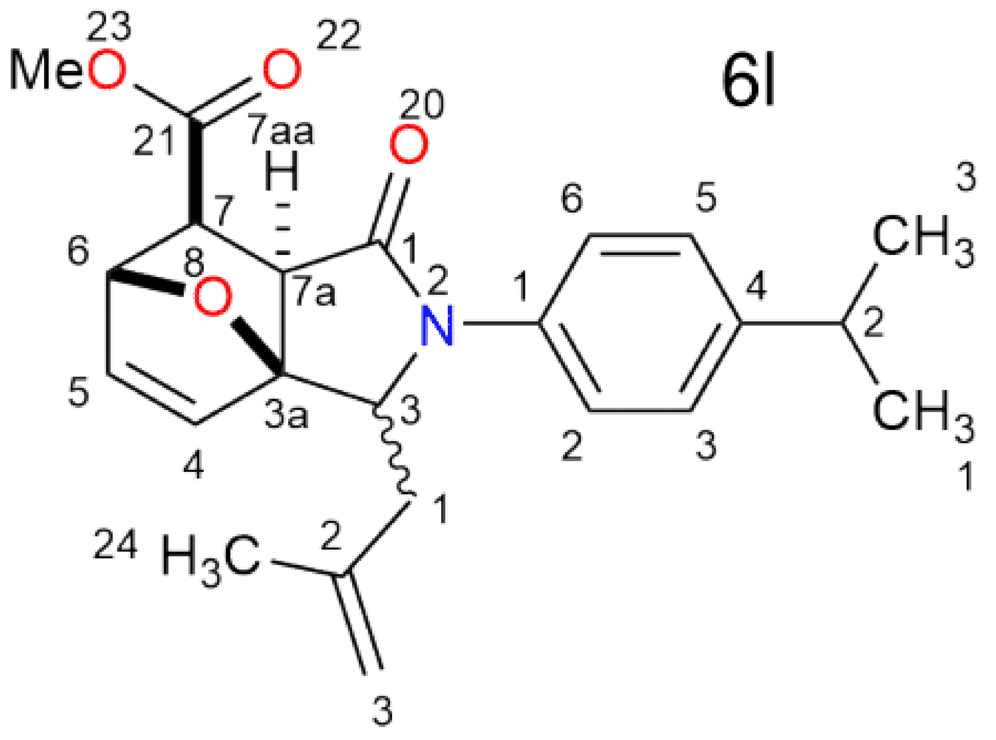

6l

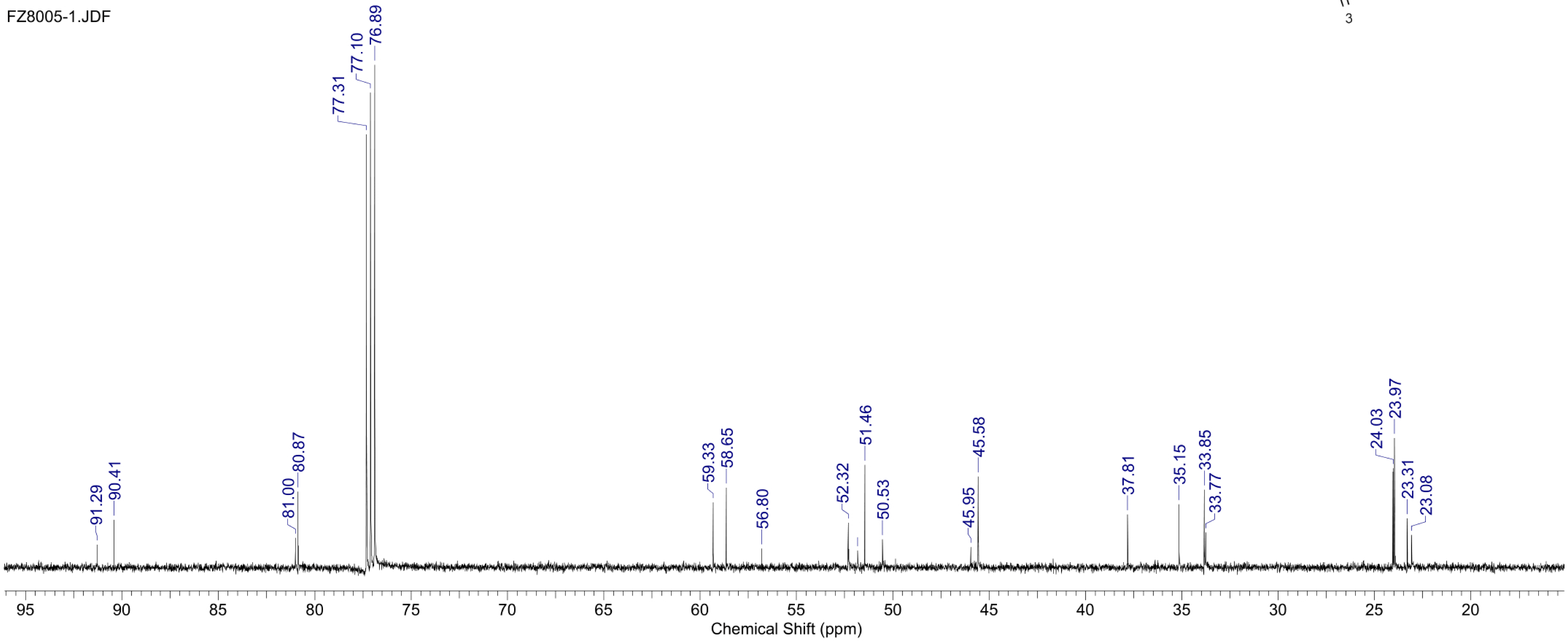

NMR spectrum data for compound 6m

Acquisition Time (sec)	1.9818	***Comment***	single_pulse	***Date***	28 May 1990 08:02:11		
Date Stamp	07 Aug 2019 14:05:37			***File Name***	C:\Users\Fedor\Desktop\07.08.19\FZ7649-1.jdf	***Frequency (MHz)***	600.17
Nucleus	1H	***Number of Transients***	8	***Origin***	ECA 600	***Original Points Count***	32768
						Owner	delta
Points Count	32768	***Pulse Sequence***	single_pulse.ex2			***Receiver Gain***	40.00
						Solvent	CHLOROFORM-d
Spectrum Offset (Hz)	5410.6274	***Sweep Width (Hz)***	16534.39	***Temperature (degree C)***	22.100		

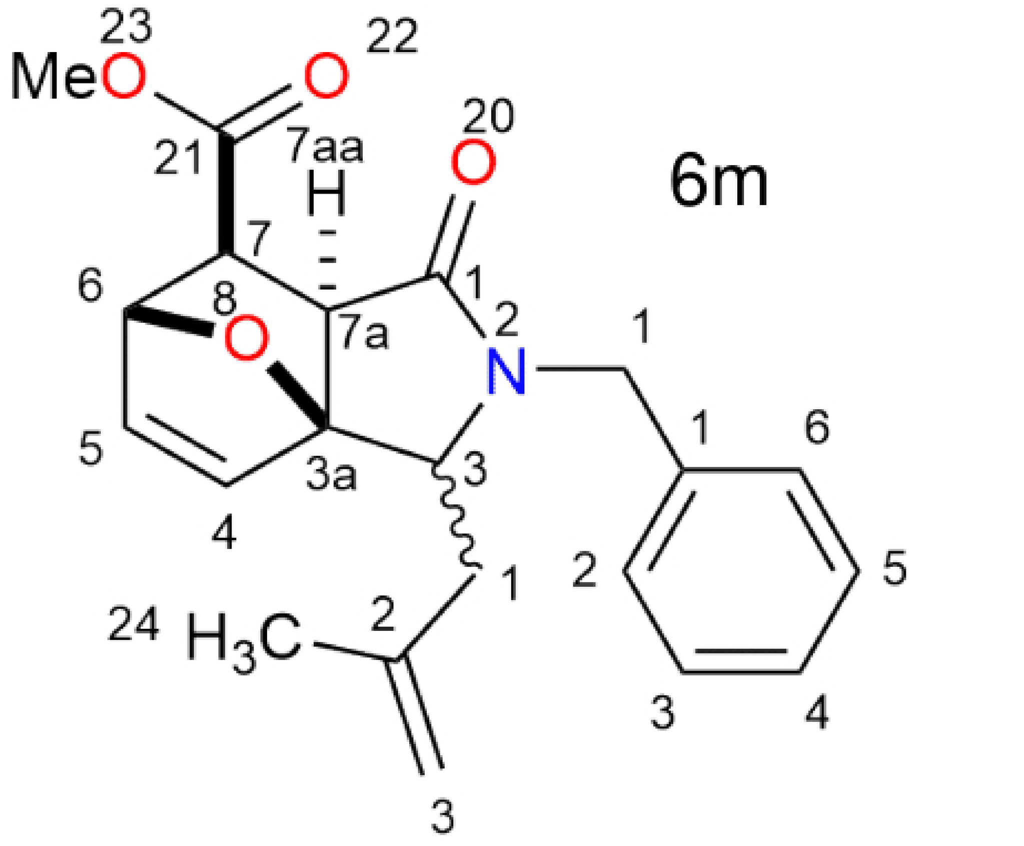

6m

FZ7649-1.esp

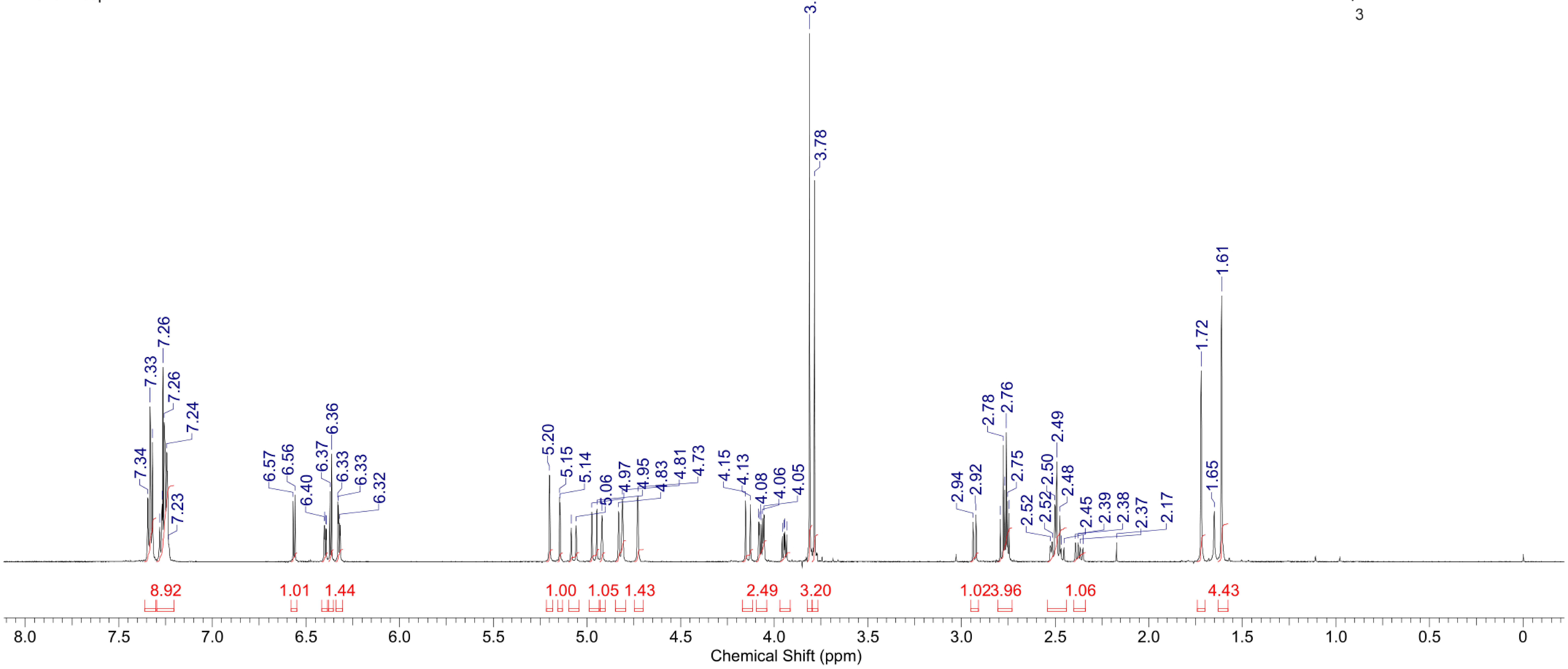

Acquisition Time (sec)	1.9818	***Comment***	single_pulse	***Date***	28 May 1990 08:02:11		
Date Stamp	07 Aug 2019 14:05:37			***File Name***	C:\Users\Fedor\Desktop\07.08.19\FZ7649-1.jdf	***Frequency (MHz)***	600.17
Nucleus	1H	***Number of Transients***	8	***Origin***	ECA 600	***Original Points Count*** 32768	***Owner*** delta
Points Count	32768	***Pulse Sequence***	single_pulse.ex2			***Receiver Gain*** 40.00	***Solvent*** CHLOROFORM-d
Spectrum Offset (Hz)	5410.6274	***Sweep Width (Hz)***	16534.39	***Temperature (degree C)***	22.100		

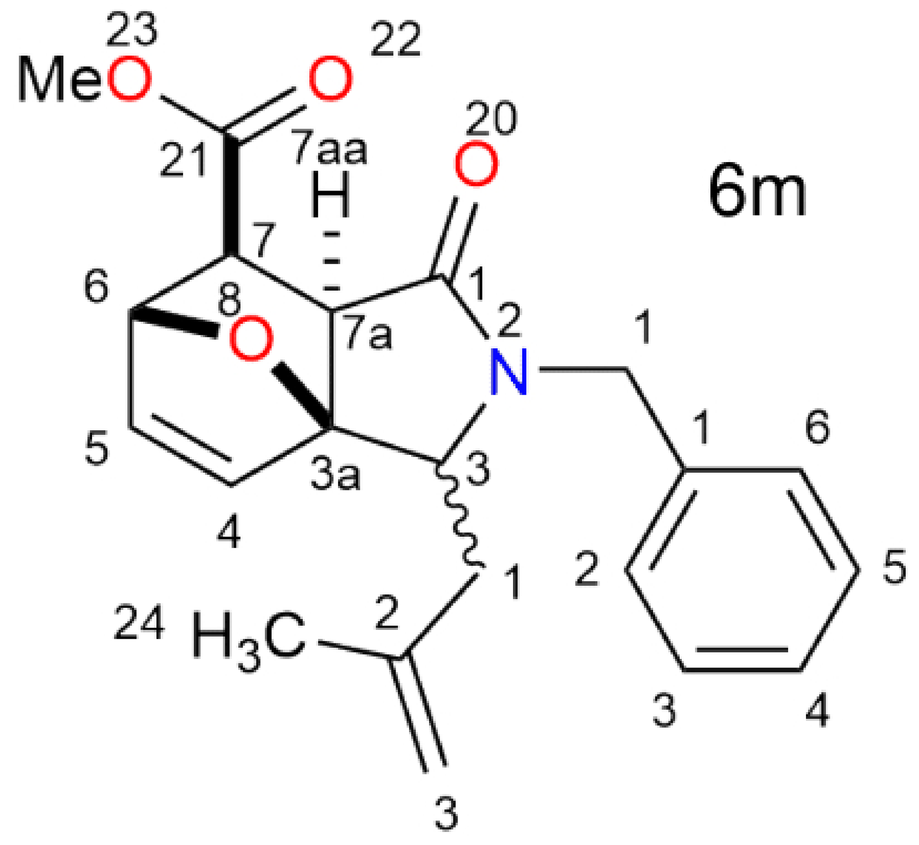

6m

FZ7649-1.esp

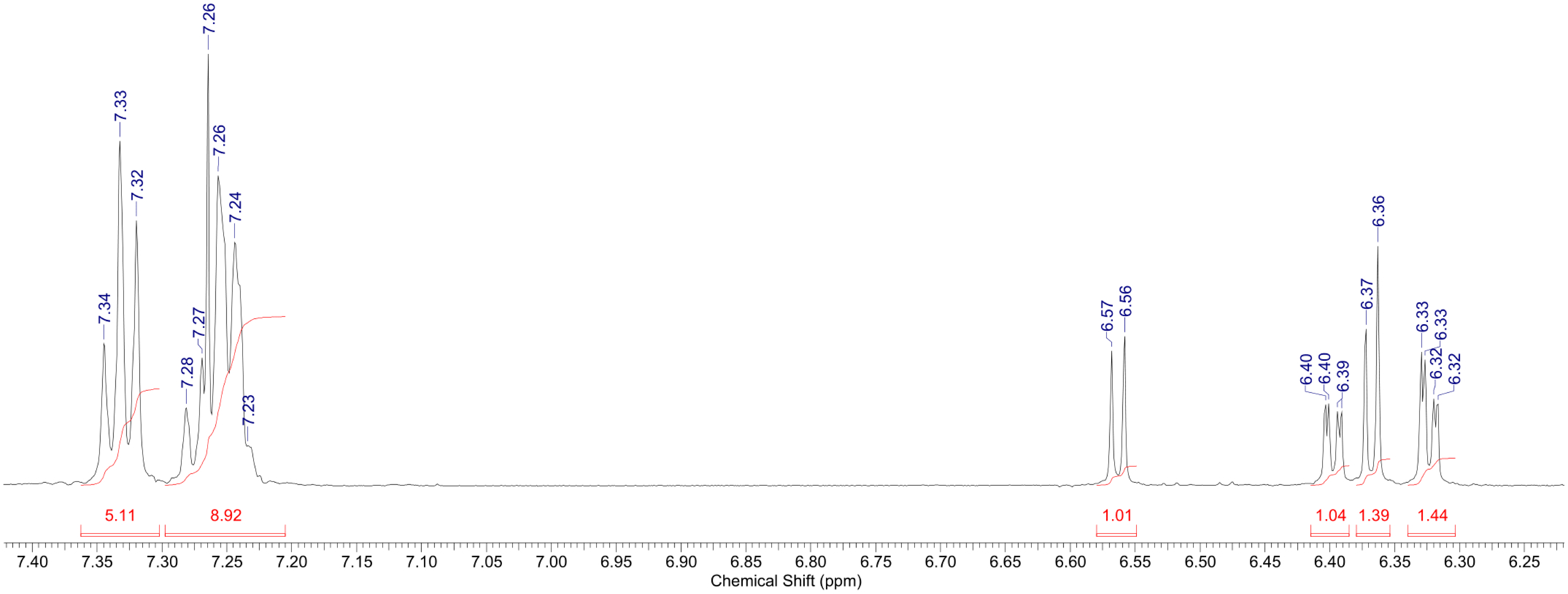

Acquisition Time (sec)	1.9818	***Comment***	single_pulse	***Date***	28 May 1990 08:02:11				
Date Stamp	07 Aug 2019 14:05:37			***File Name***	C:\Users\Fedor\Desktop\07.08.19\FZ7649-1.jdf			***Frequency (MHz)***	600.17
Nucleus	1H	***Number of Transients***	8	***Origin***	ECA 600	***Original Points Count***	32768	***Owner***	delta
Points Count	32768	***Pulse Sequence***	single_pulse.ex2			***Receiver Gain***	40.00	***Solvent***	CHLOROFORM-d
Spectrum Offset (Hz)	5410.6274	***Sweep Width (Hz)***	16534.39	***Temperature (degree C)***	22.100				

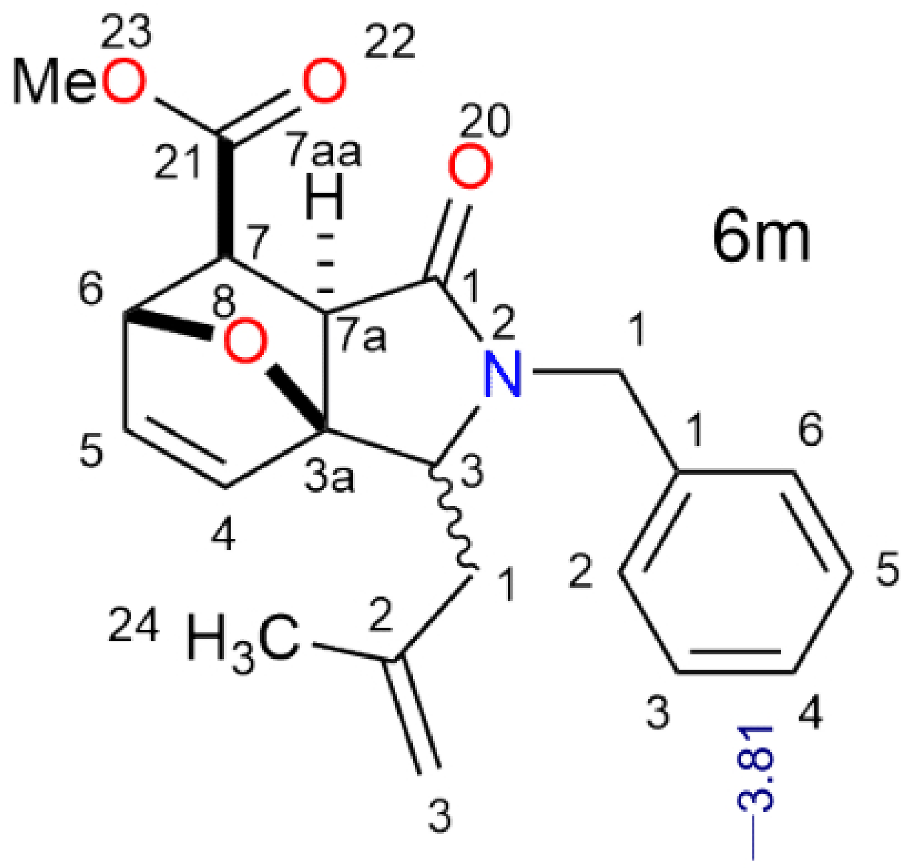

6m

FZ7649-1.esp

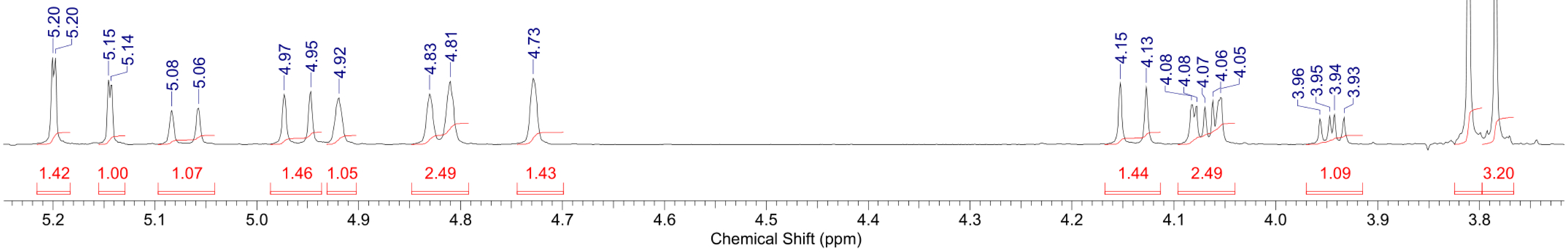

Acquisition Time (sec)	1.9818	***Comment***	single_pulse	***Date***	28 May 1990 08:02:11		
Date Stamp	07 Aug 2019 14:05:37			***File Name***	C:\Users\Fedor\Desktop\07.08.19\FZ7649-1.jdf	***Frequency (MHz)***	600.17
Nucleus	1H	***Number of Transients***	8	***Origin***	ECA 600	***Original Points Count***	32768
Owner	delta						
Points Count	32768	***Pulse Sequence***	single_pulse.ex2	***Receiver Gain***	40.00	***Solvent***	CHLOROFORM-d
Spectrum Offset (Hz)	5410.6274	***Sweep Width (Hz)***	16534.39	***Temperature (degree C)***	22.100		

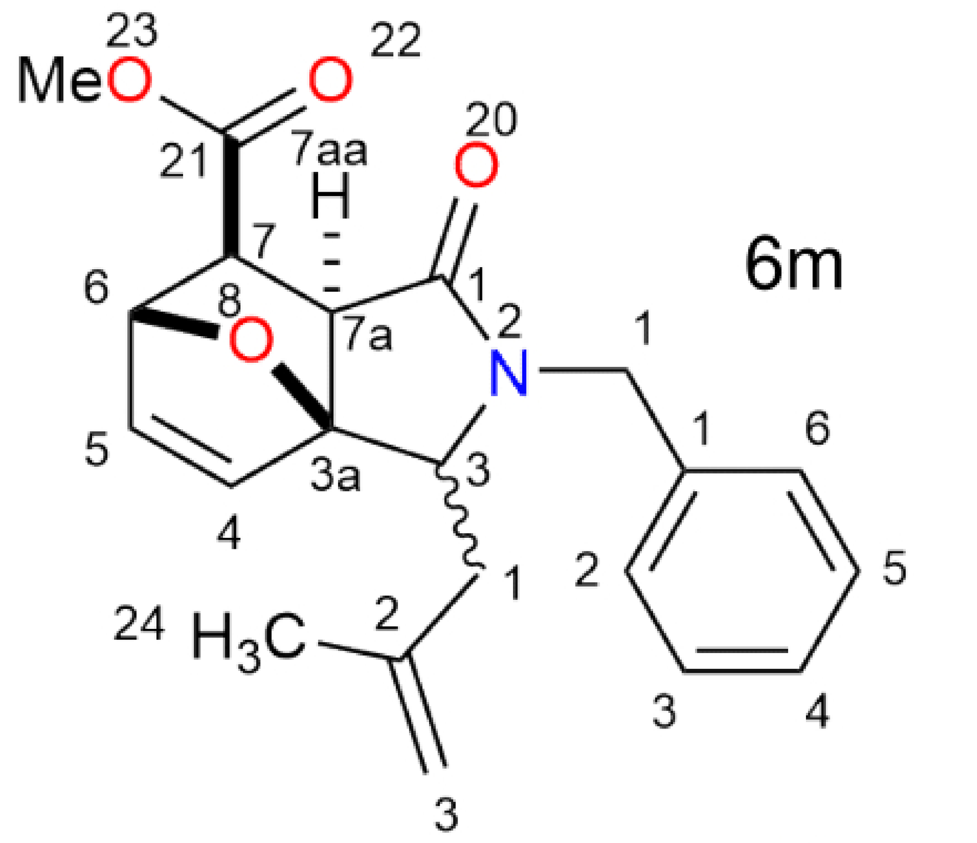

FZ7649-1.esp

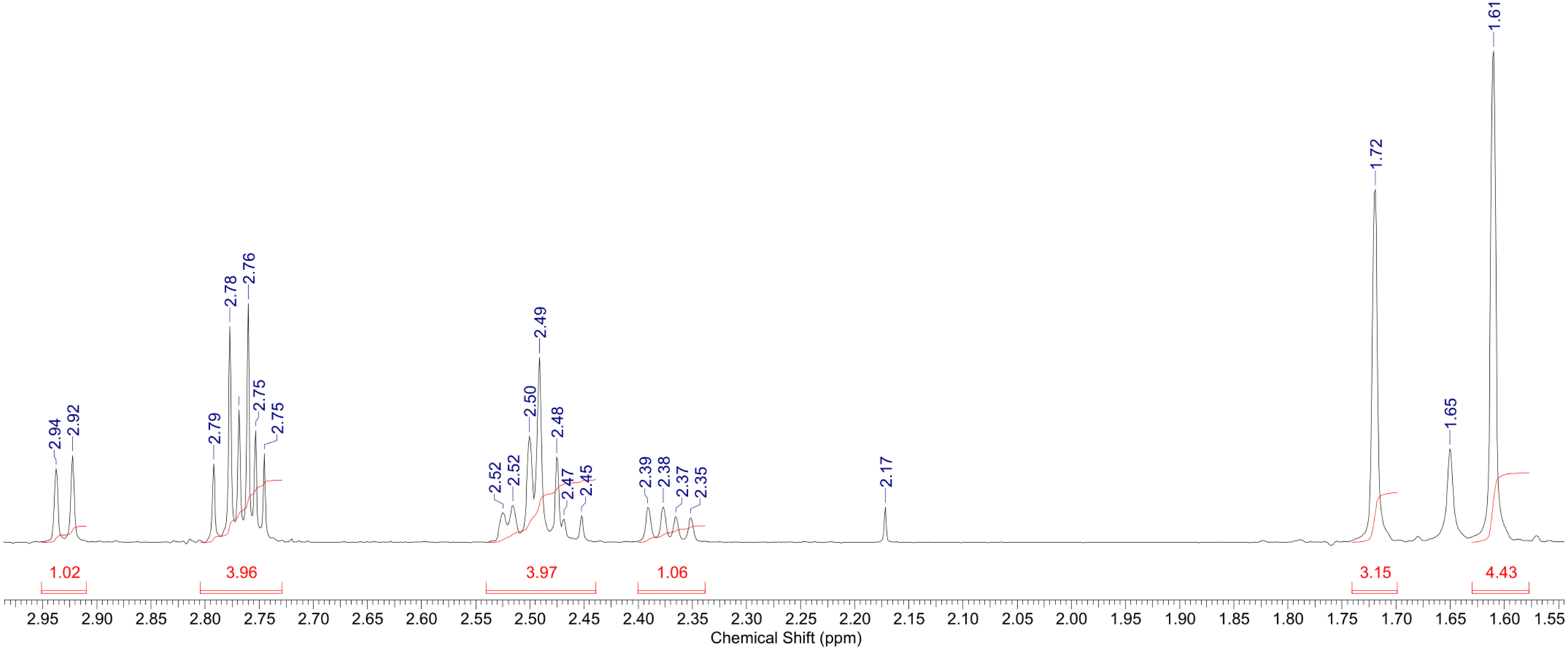

14.07.2020 18:52:23

**Formula** $C_{21}H_{23}NO_4$	**FW** 353.4116

**Acquisition Time (sec)**	0.6921	**Comment**	single pulse decoupled gated NOE	**Date**	12 Aug 1990 03:40:32
**Date Stamp**	22 Oct 2019 09:43:40	**File Name**	C:\USERS\Лаба534\DOWNLOADS\FZ8007-1.JDF		
**Frequency (MHz)**	150.91	**Nucleus**	13C	**Number of Transients**	800
**Origin**	ECA 600	**Original Points Count**	32768	**Owner**	delta
**Points Count**	32768	**Pulse Sequence**	single_pulse_dec	**Receiver Gain**	54.00
**Solvent**	CHLOROFORM-d	**Spectrum Offset (Hz)**	15080.7979	**Sweep Width (Hz)**	47348.49
**Temperature (degree C)**	24.300				

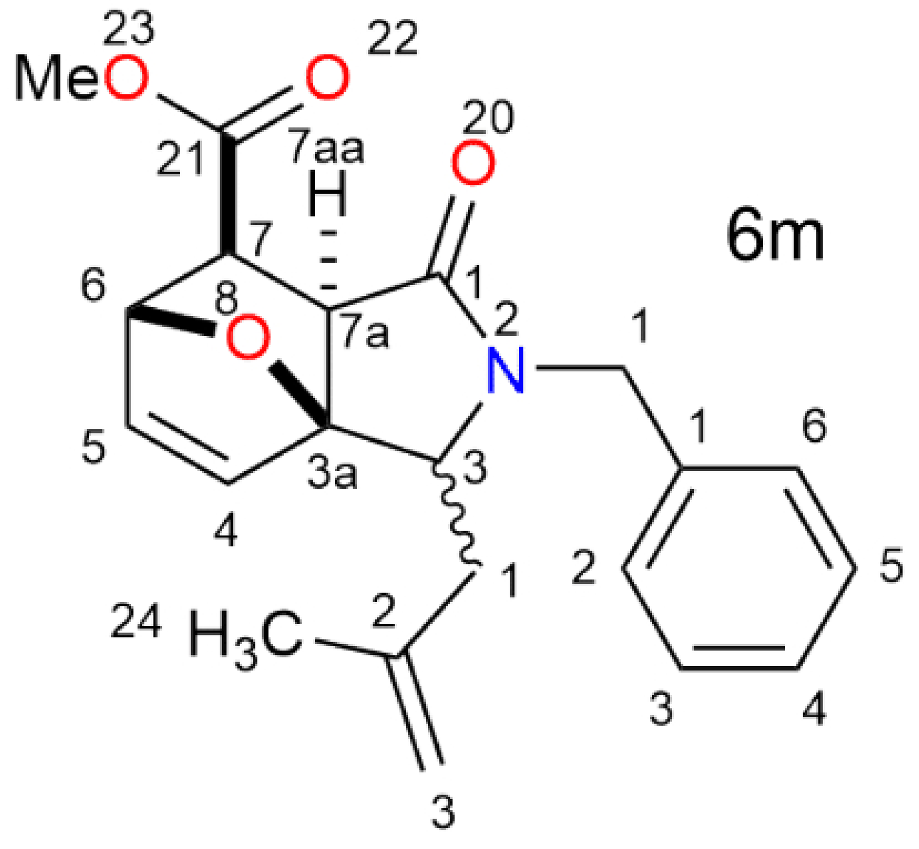

6m

FZ8007-1.JDF

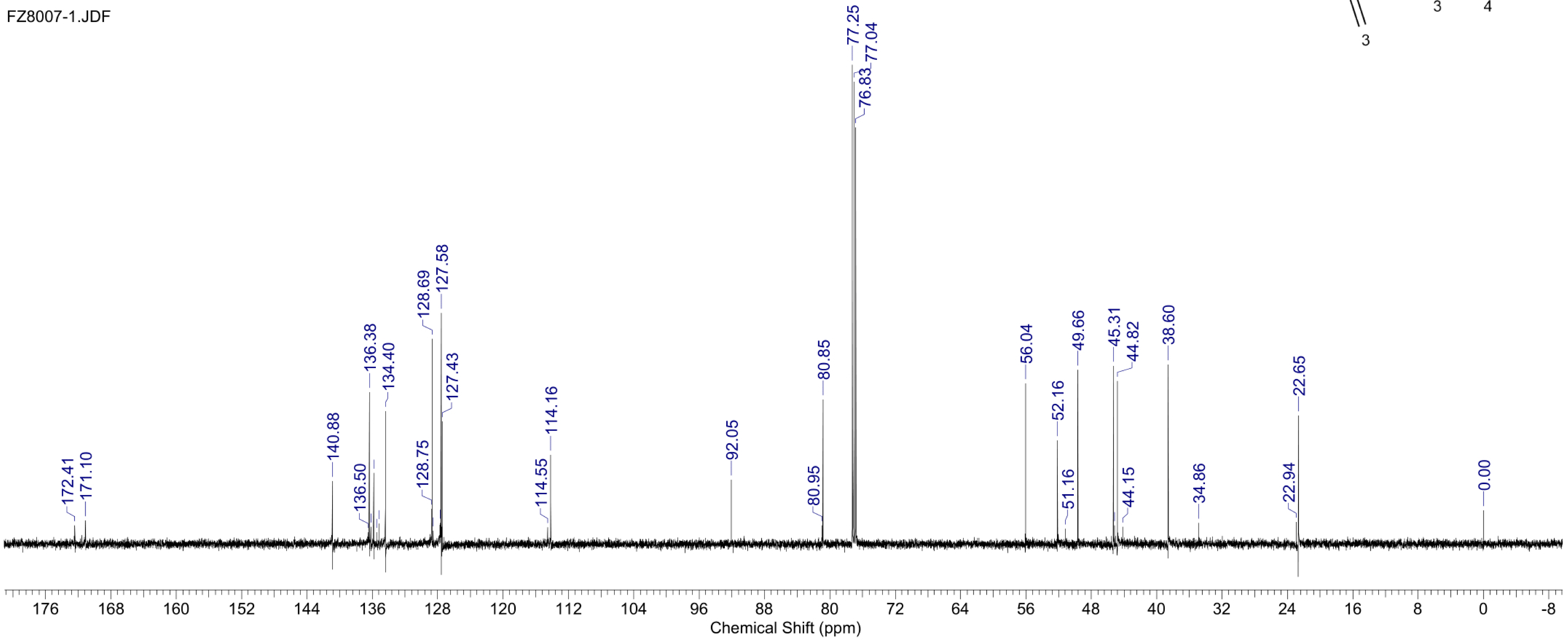

14.07.2020 18:52:30

**Formula** $C_{21}H_{23}NO_4$	**FW** 353.4116

Acquisition Time (sec)	0.6921	***Comment***	single pulse decoupled gated NOE			***Date***	12 Aug 1990 03:40:32
Date Stamp	22 Oct 2019 09:43:40			***File Name***	C:\USERS\Лаба534\DOWNLOADS\FZ8007-1.JDF		
Frequency (MHz)	150.91	***Nucleus***	13C	***Number of Transients***	800	***Origin***	ECA 600
Original Points Count	32768	***Owner***	delta	***Points Count***	32768	***Pulse Sequence***	single_pulse_dec
Receiver Gain	54.00	***Solvent***	CHLOROFORM-d			***Spectrum Offset (Hz)***	15080.7979
Sweep Width (Hz)	47348.49	***Temperature (degree C)***	24.300				

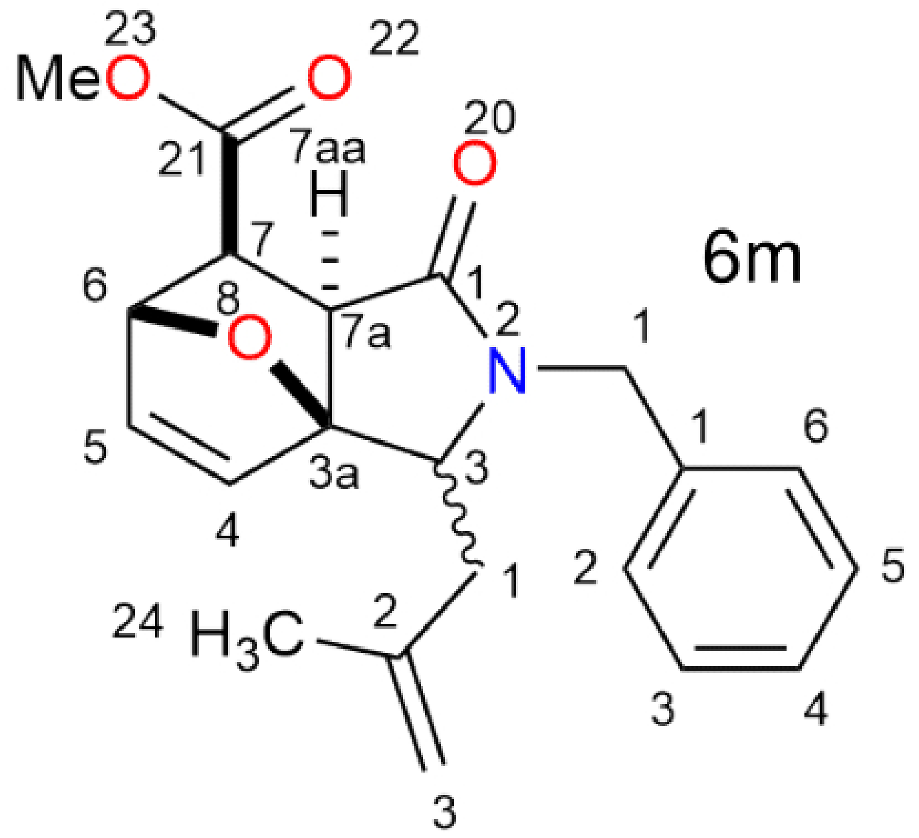

FZ8007-1.JDF

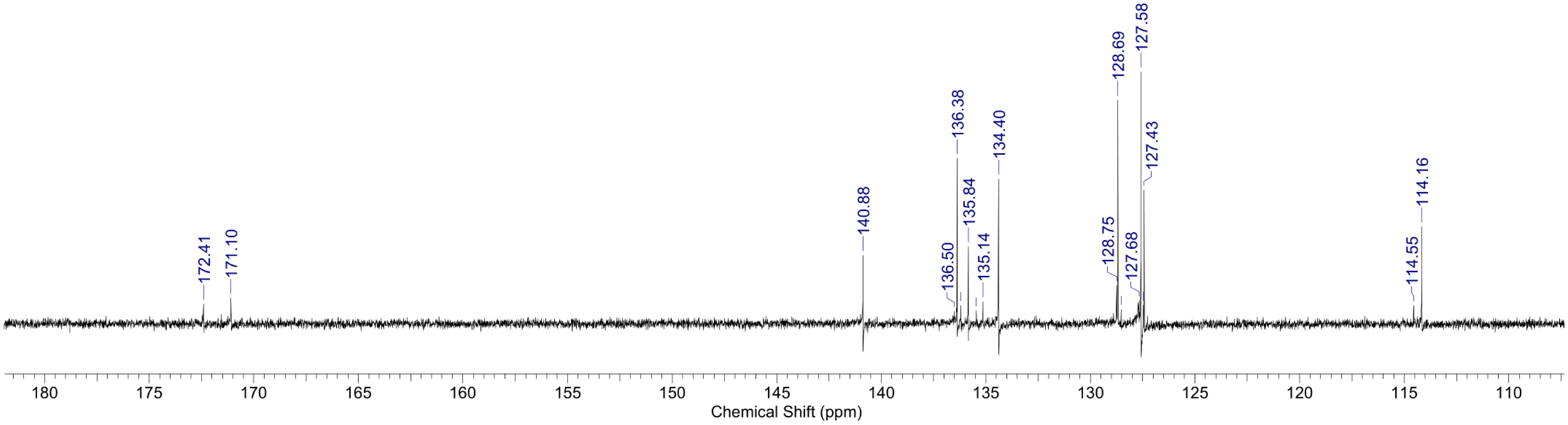

14.07.2020 18:52:38

**Formula** $C_{21}H_{23}NO_4$	**FW** 353.4116

**Acquisition Time (sec)**	0.6921	**Comment**	single pulse decoupled gated NOE			**Date**	12 Aug 1990 03:40:32
**Date Stamp**	22 Oct 2019 09:43:40			**File Name**	C:\USERS\Ла6а534\DOWNLOADS\FZ8007-1.JDF		
**Frequency (MHz)**	150.91	**Nucleus**	13C	**Number of Transients**	800	**Origin**	ECA 600
**Original Points Count**	32768	**Owner**	delta	**Points Count**	32768	**Pulse Sequence**	single_pulse_dec
**Receiver Gain**	54.00	**Solvent**	CHLOROFORM-d			**Spectrum Offset (Hz)**	15080.7979
**Sweep Width (Hz)**	47348.49	**Temperature (degree C)**	24.300				

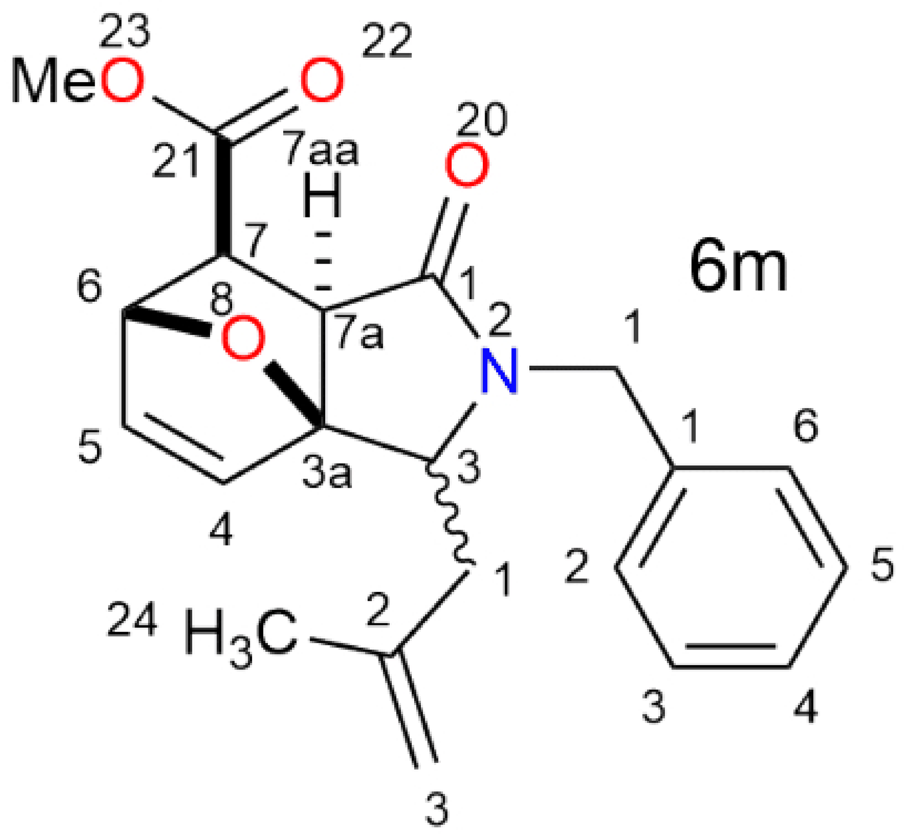

6m

FZ8007-1.JDF

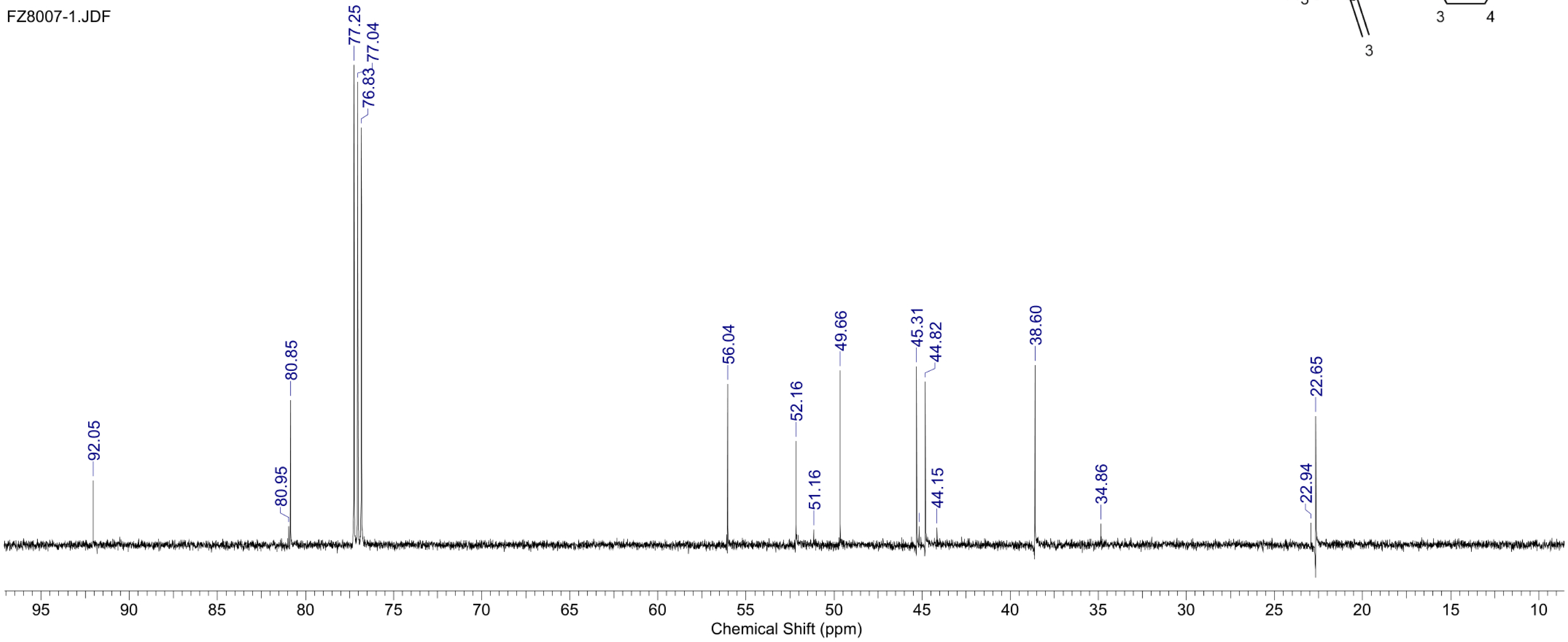

DEPT-135 NMR spectrum for compound 6m

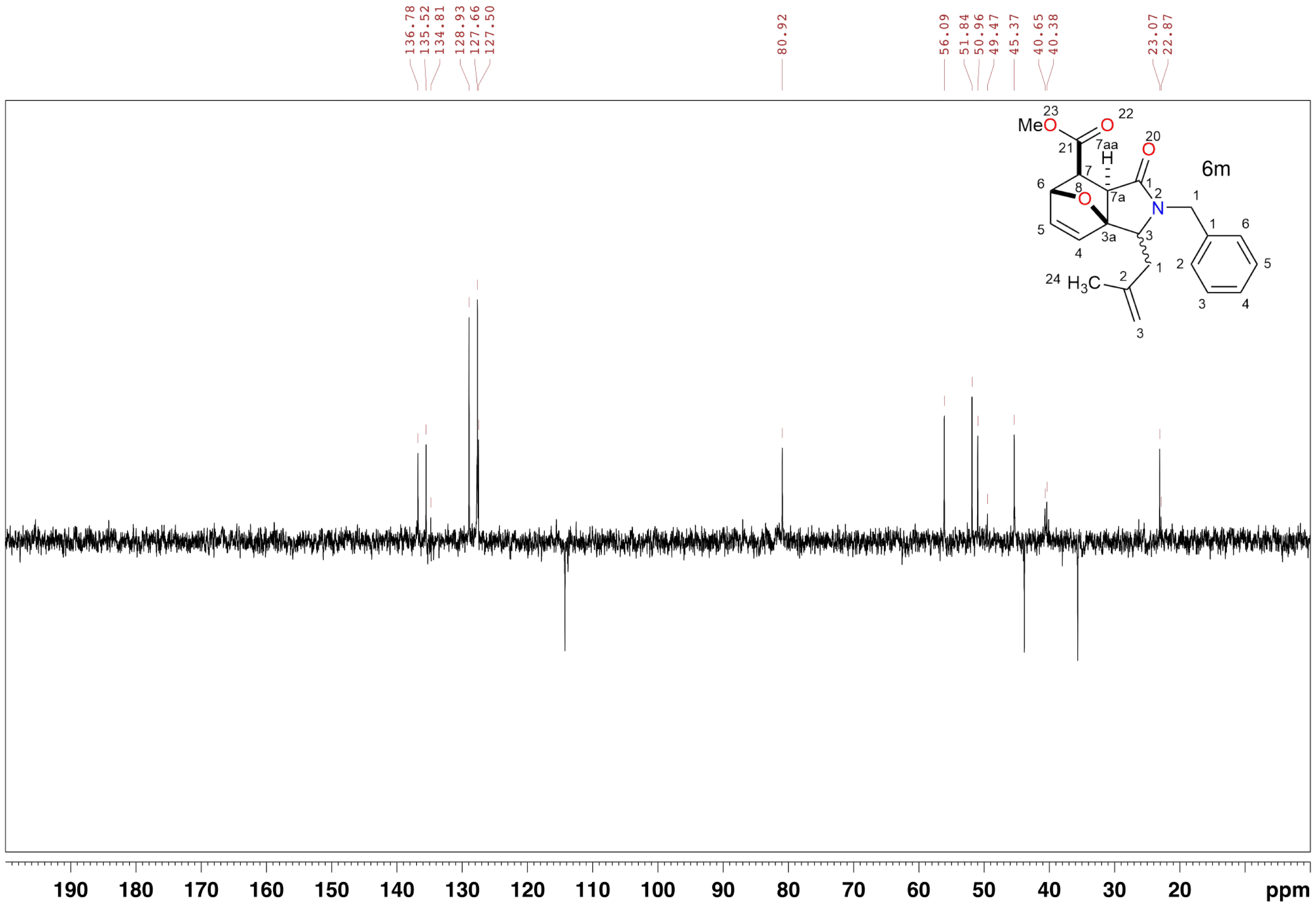

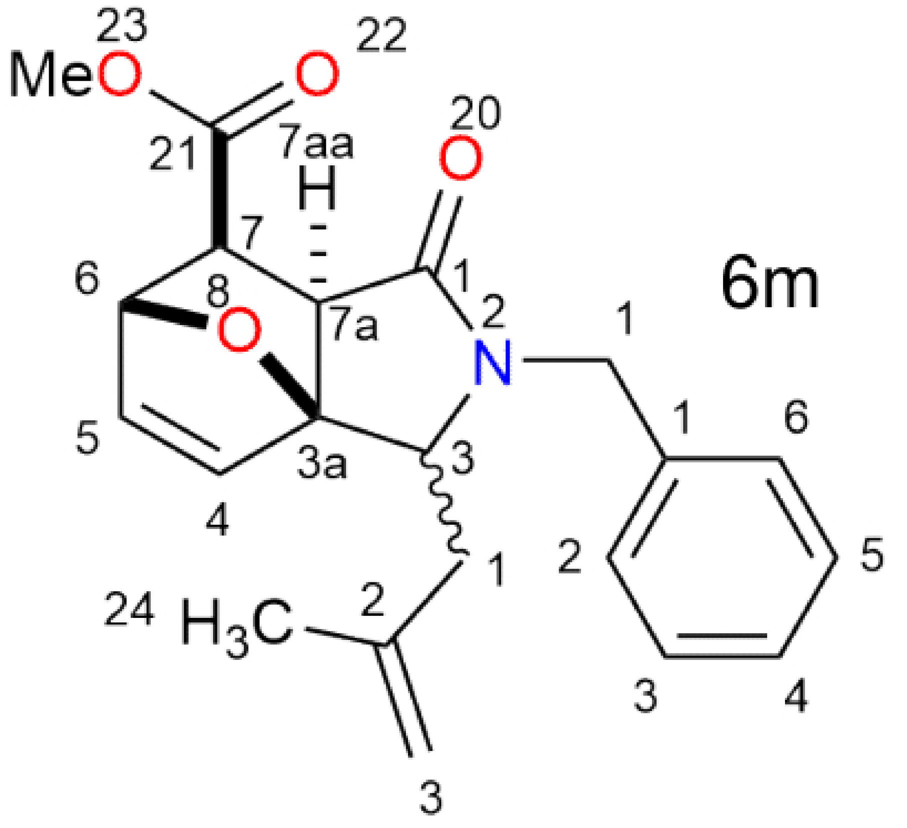

6m

COSY NMR spectrum for compound 6m

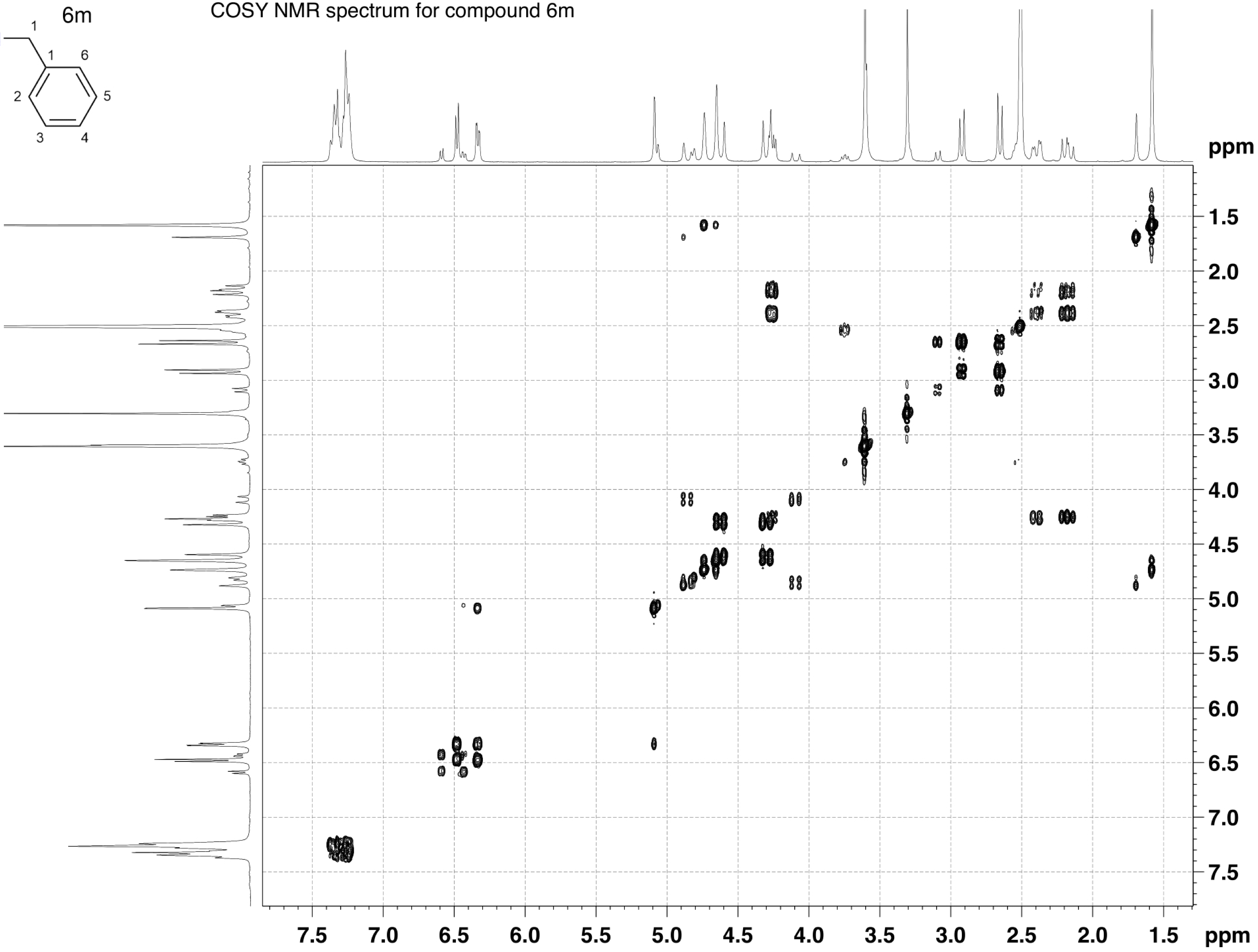

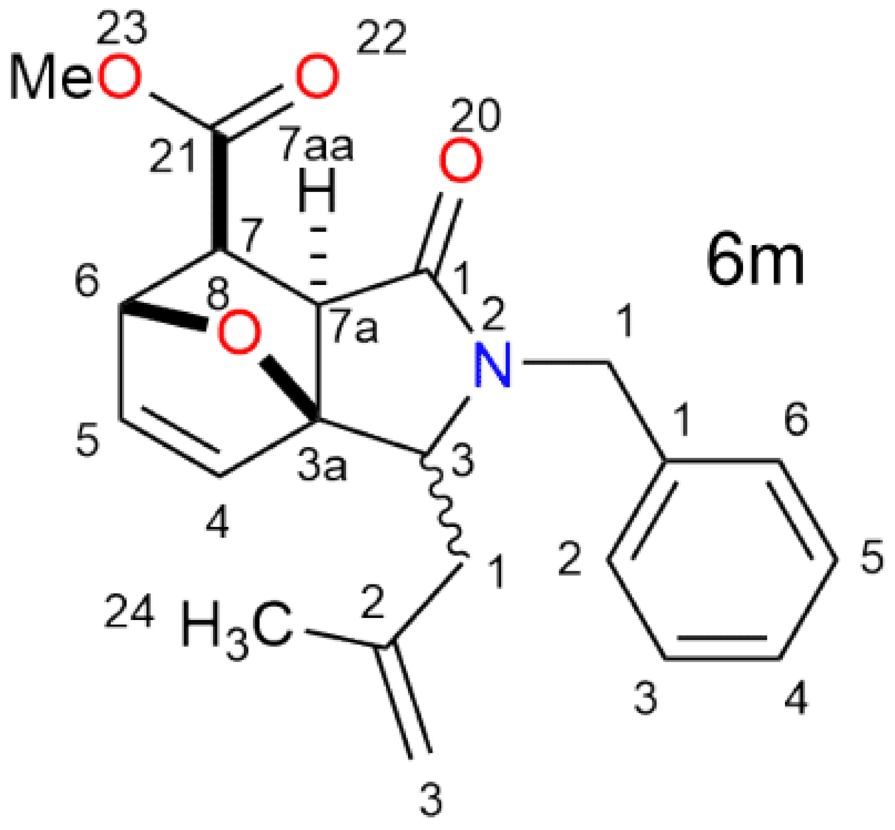

NOESY NMR spectrum for compound 6m

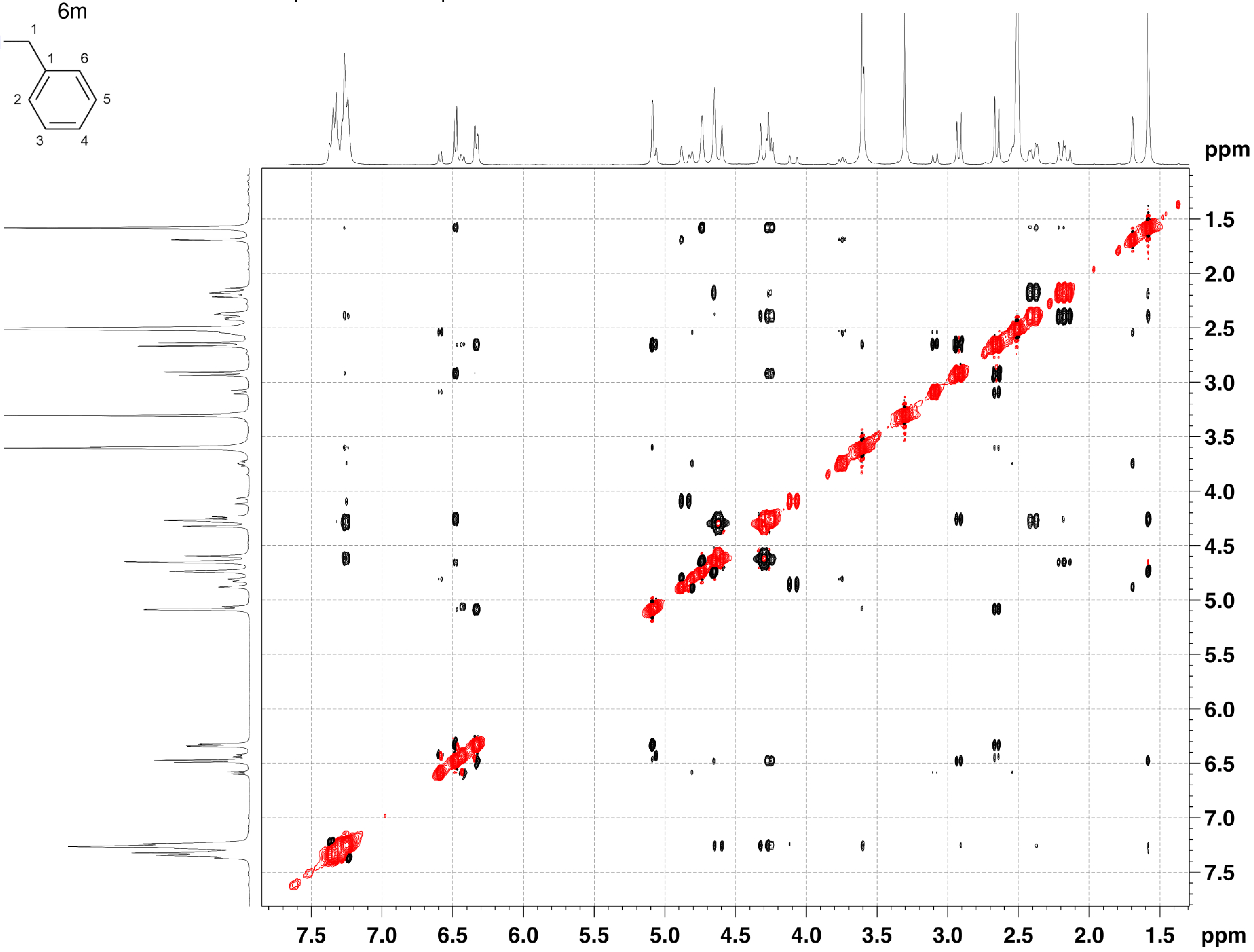

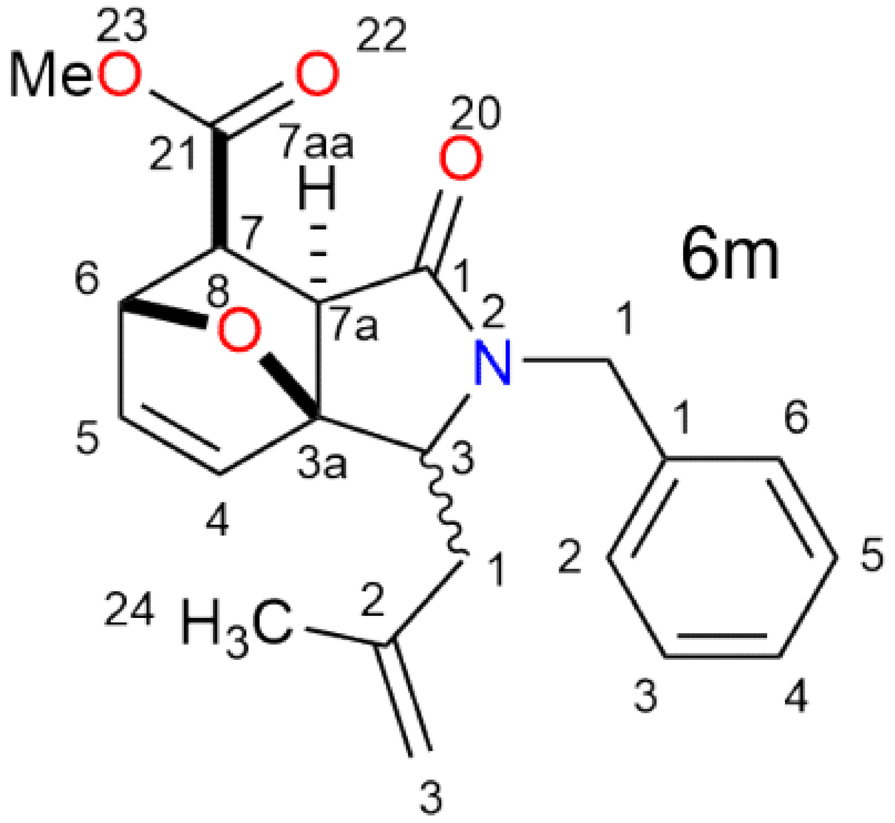

6m

HSQC NMR spectrum for compound 6m

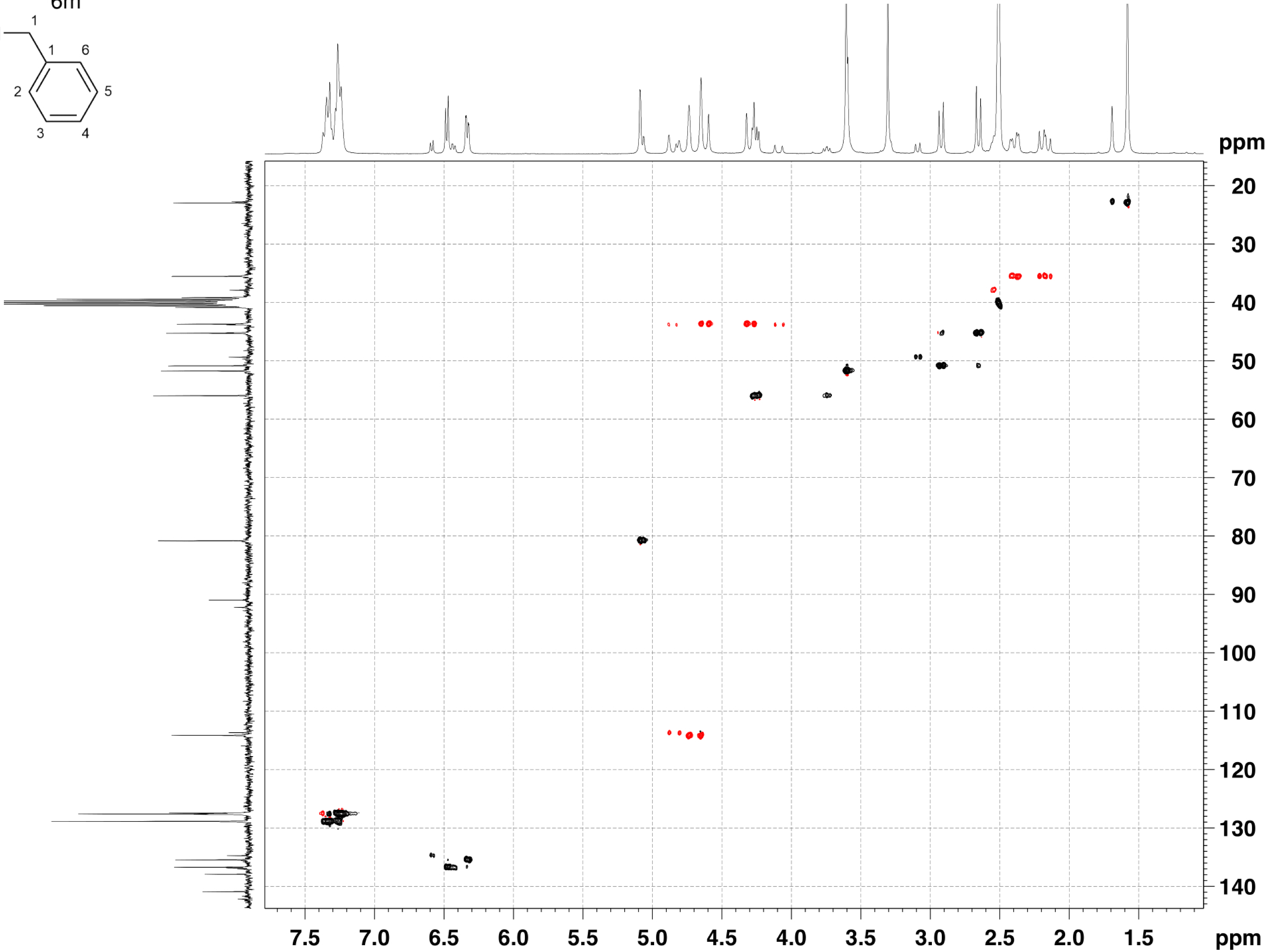

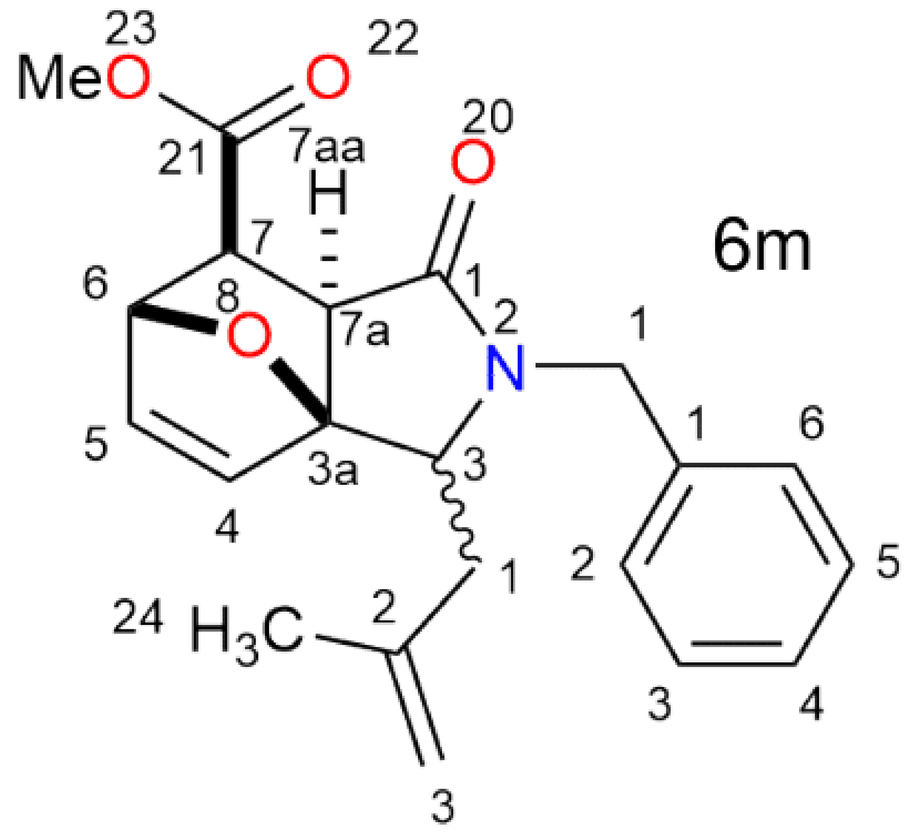

6m

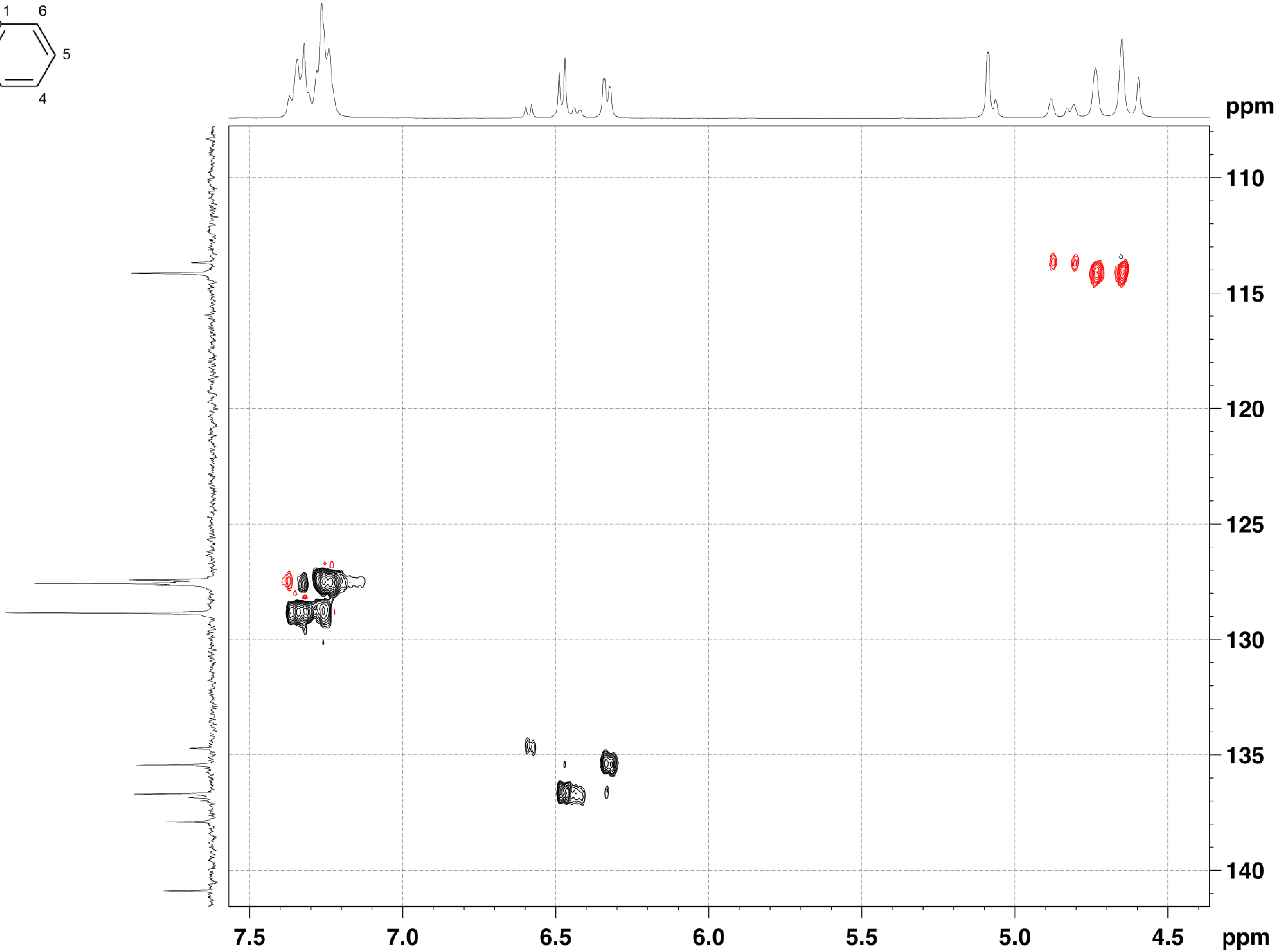

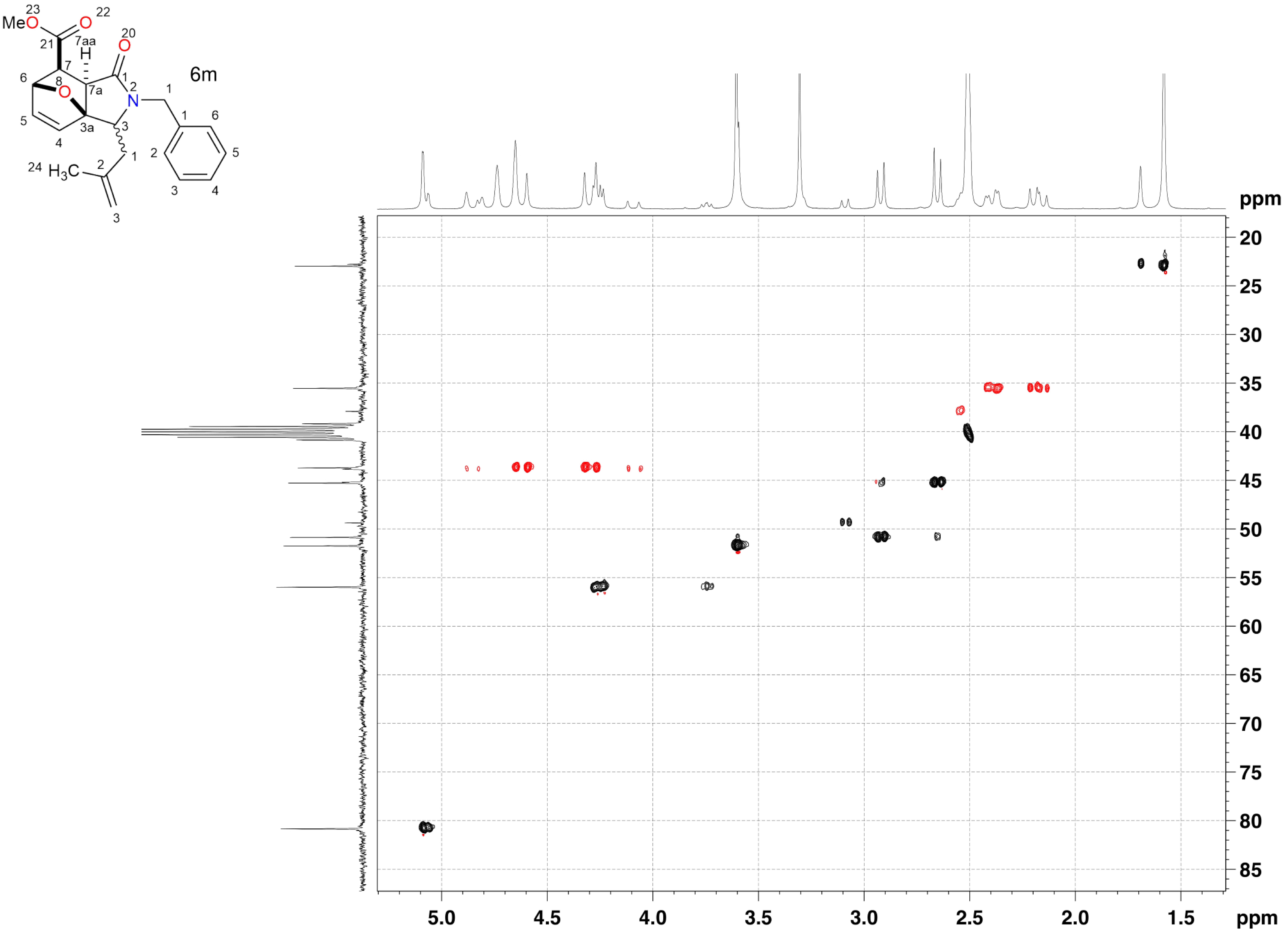

6m
ppm
20
25
30
35
40
45
50
55
60
65
70
75
80
85
5.0
4.5
4.0
3.5
3.0
2.5
2.0
1.5
ppm

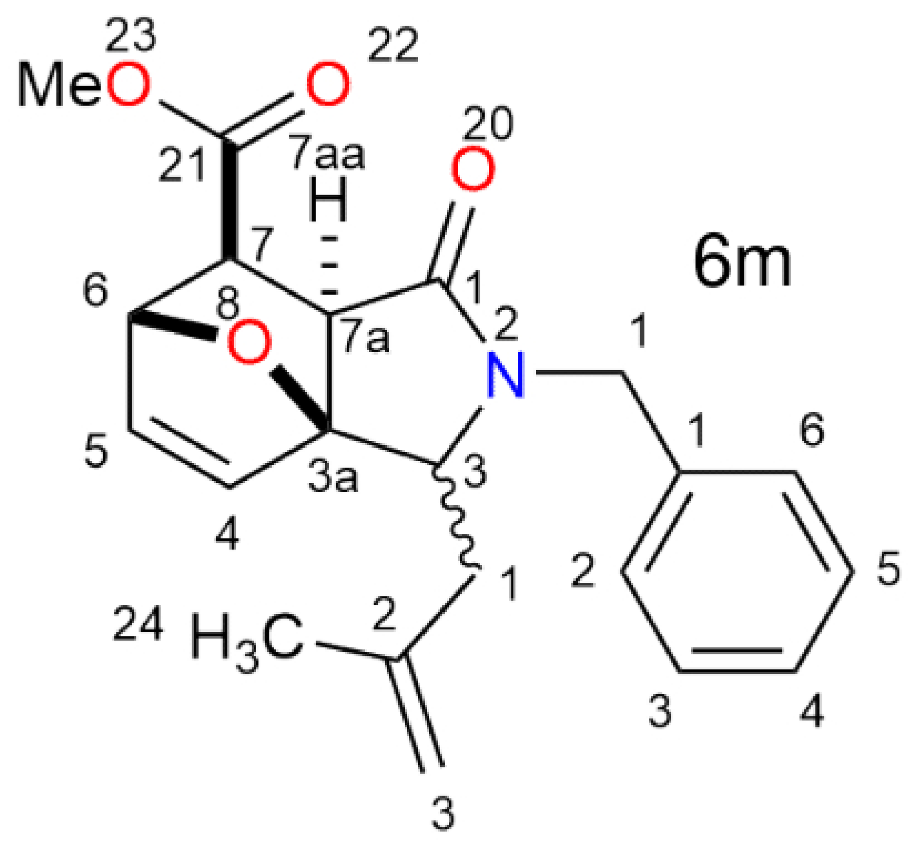

HMBC NMR spectrum for compound 6m

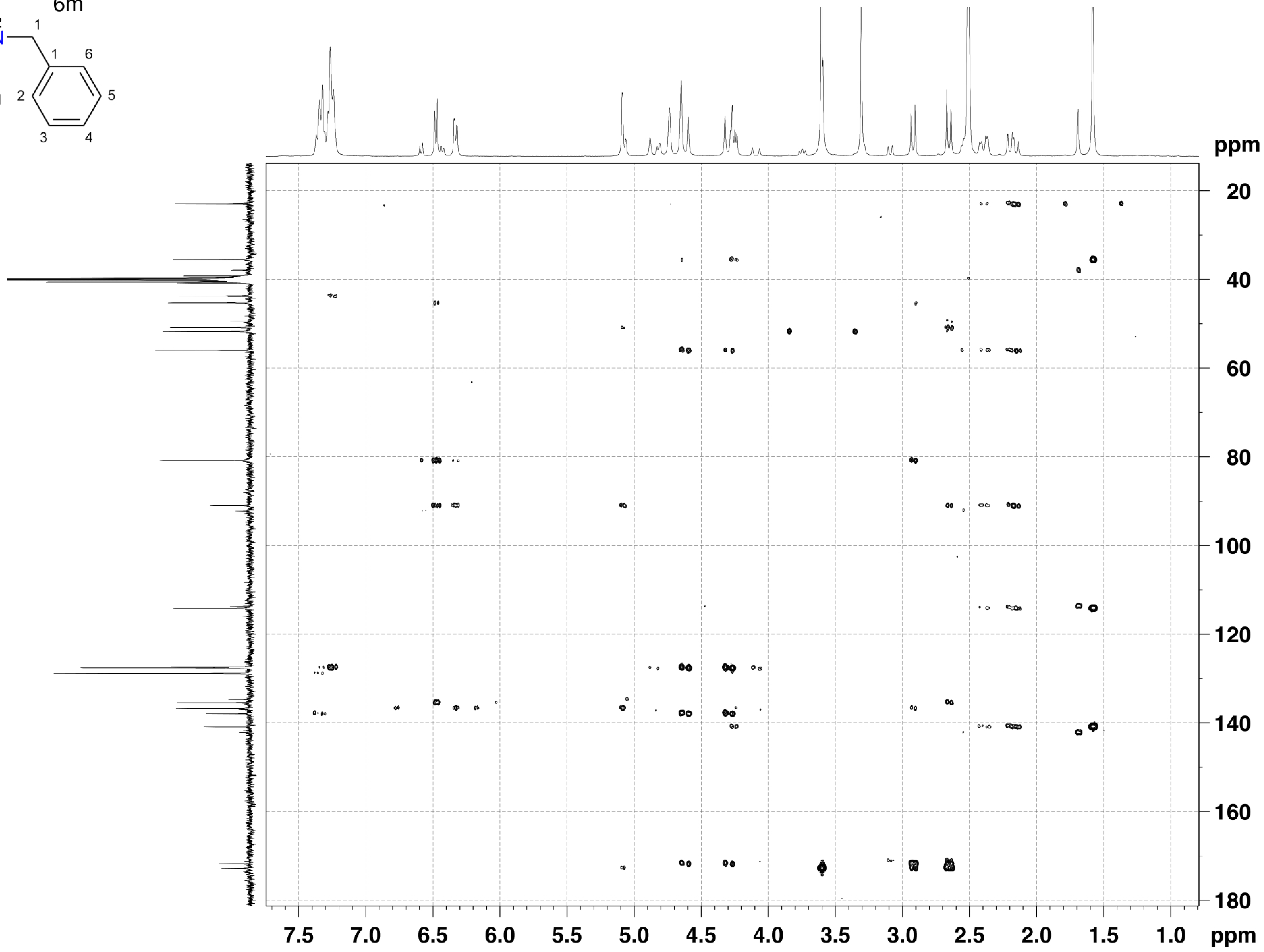

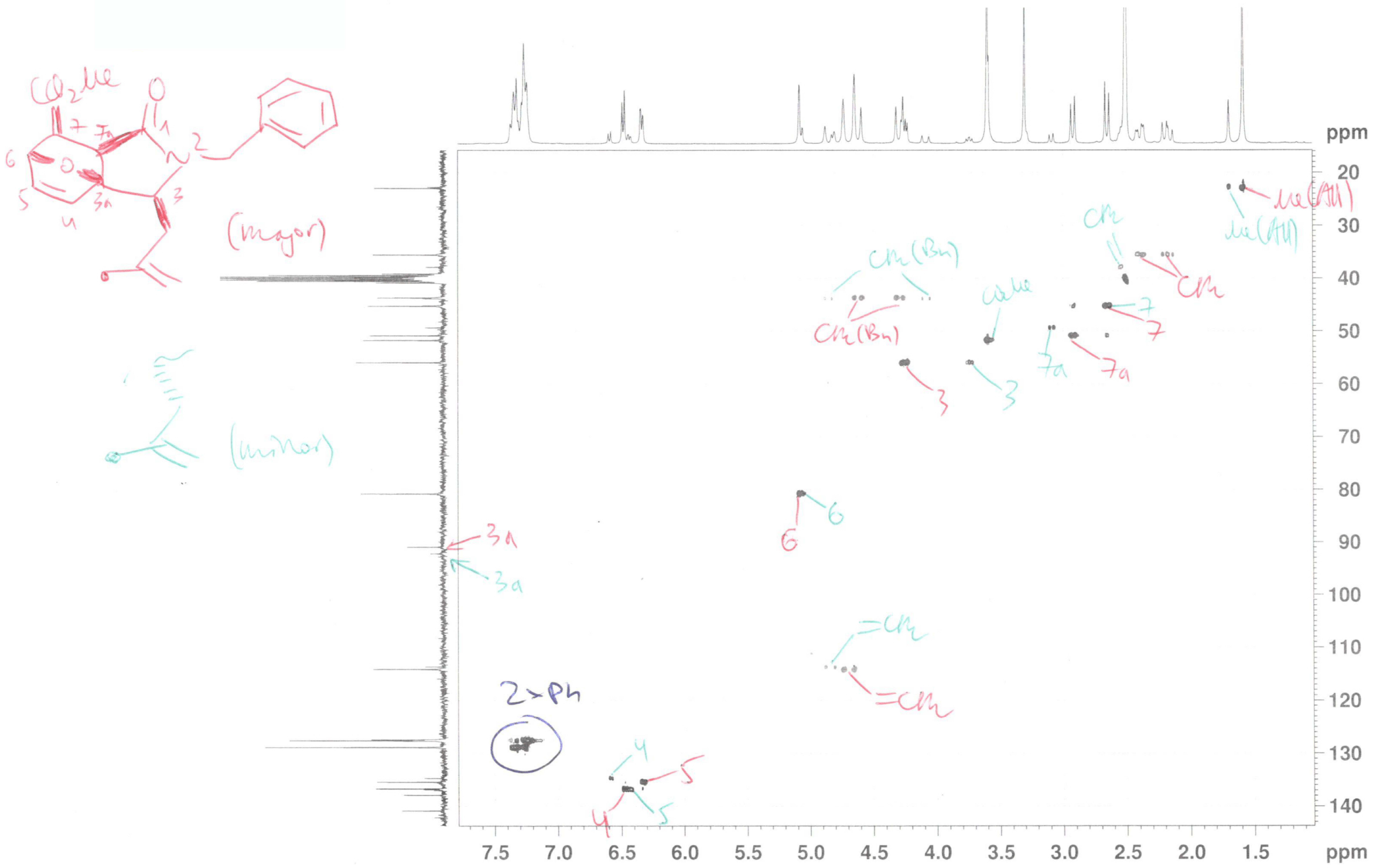

CO2Me
(major)
(minor)
2×Ph
CH2(Bn)
CO2Me
CH2
=CH2
3a
ppm
20
30
40
50
60
70
80
90
100
110
120
130
140
7.5
7.0
6.5
6.0
5.5
5.0
4.5
4.0
3.5
3.0
2.5
2.0
1.5
ppm

NMR spectrum data for compound 6n

14.07.2020 18:58:07

**Formula**	$C_{20}H_{20}ClNO_4$	**FW**	373.8301

**Acquisition Time (sec)**	0.9909	**Comment**	single_pulse	**Date**	10 Feb 2020 10:04:16				
**Date Stamp**	22 Feb 2020 11:50:00			**File Name**	C:\USERS\Лa6a534\DOWNLOADS\FZ8510-1.JDF			**Frequency (MHz)**	600.17
**Nucleus**	1H	**Number of Transients**	8	**Origin**	ECA 600	**Original Points Count**	16384	**Owner**	CKP
**Points Count**	16384	**Pulse Sequence**	single_pulse.ex2			**Receiver Gain**	38.00	**Solvent**	CHLOROFORM-d
**Spectrum Offset (Hz)**	5414.0723	**Sweep Width (Hz)**	16534.39	**Temperature (degree C)**	24.000				

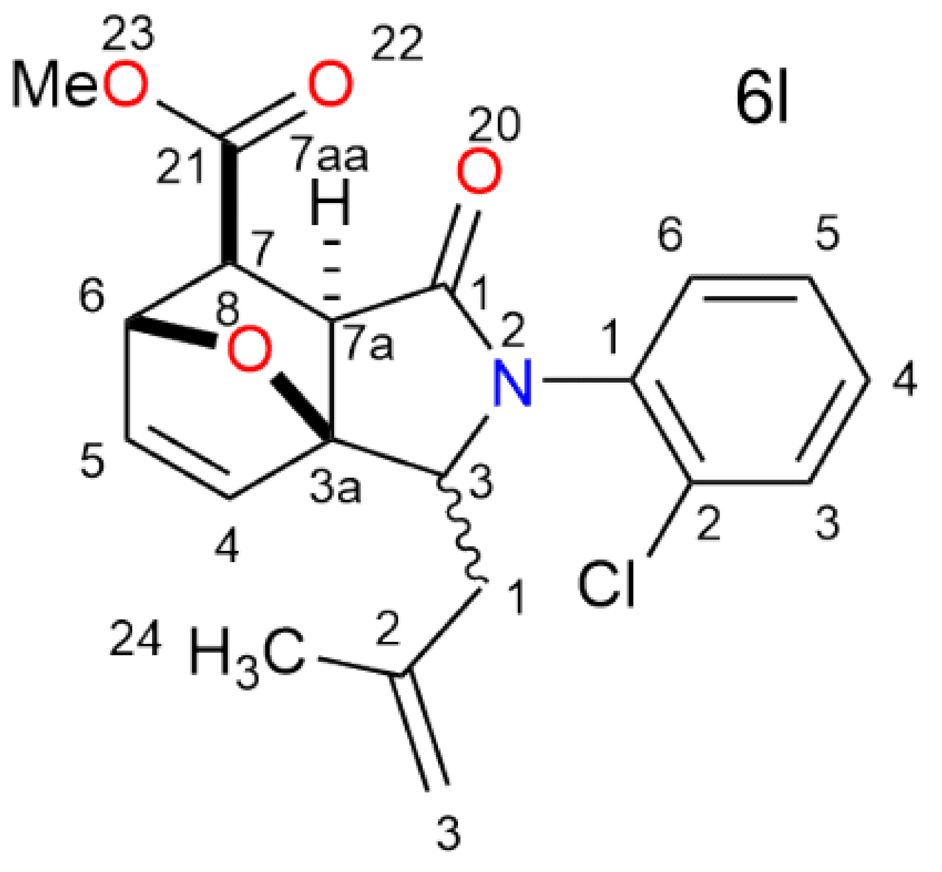

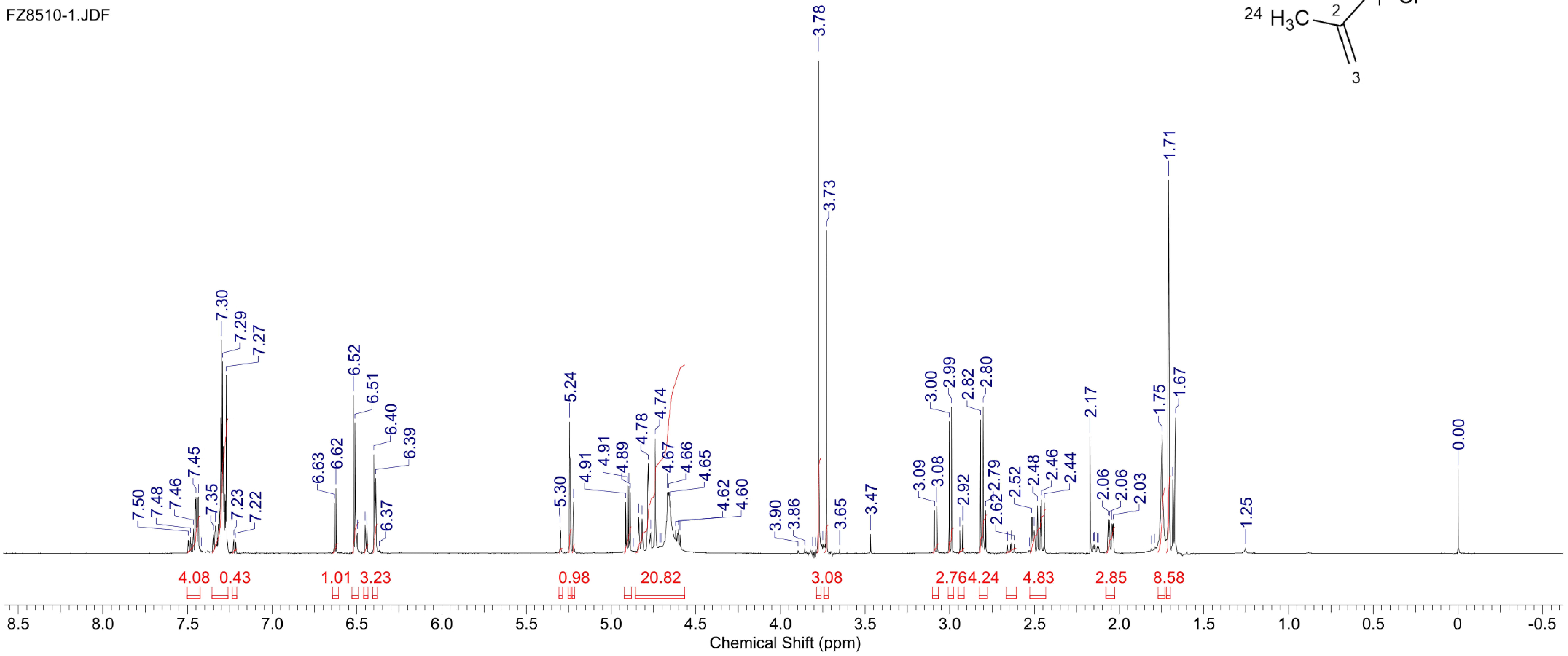

14.07.2020 18:58:22

**Formula** $C_{20}H_{20}ClNO_4$	**FW** 373.8301

**Acquisition Time (sec)**	0.9909	**Comment**	single_pulse	**Date**	10 Feb 2020 10:04:16		
**Date Stamp**	22 Feb 2020 11:50:00			**File Name**	C:\USERS\Лаба534\DOWNLOADS\FZ8510-1.JDF	**Frequency (MHz)**	600.17
**Nucleus**	1H	**Number of Transients**	8	**Origin**	ECA 600 **Original Points Count** 16384	**Owner**	CKP
**Points Count**	16384	**Pulse Sequence**	single_pulse.ex2		**Receiver Gain** 38.00	**Solvent**	CHLOROFORM-d
**Spectrum Offset (Hz)**	5414.0723	**Sweep Width (Hz)**	16534.39	**Temperature (degree C)**	24.000		

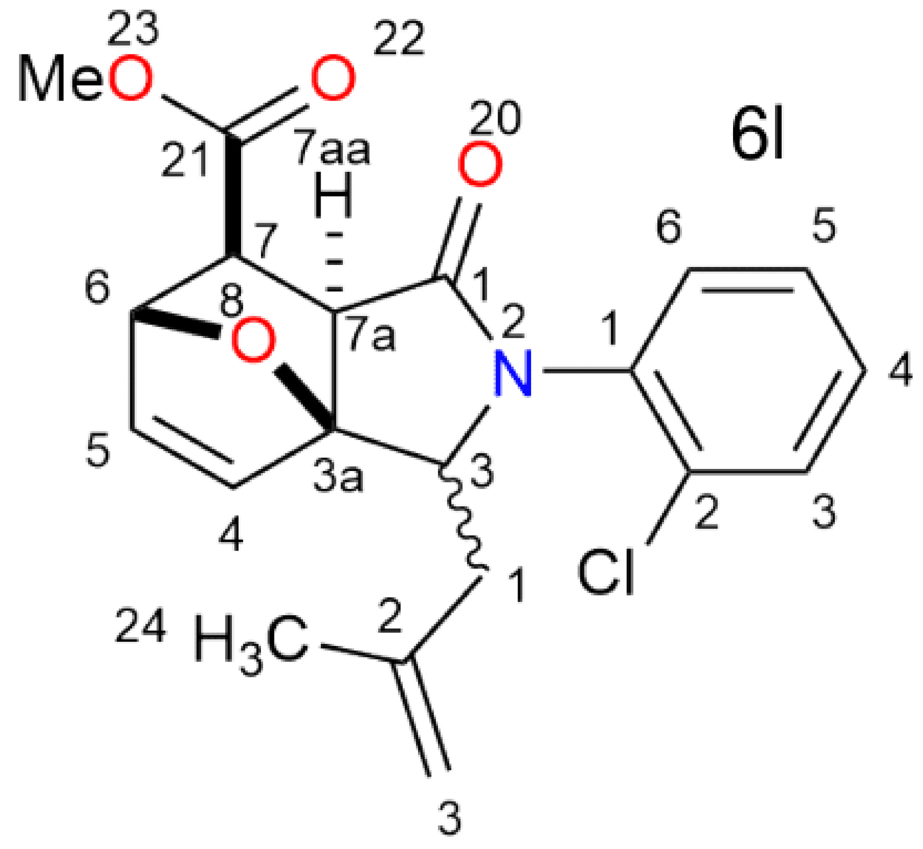

FZ8510-1.JDF

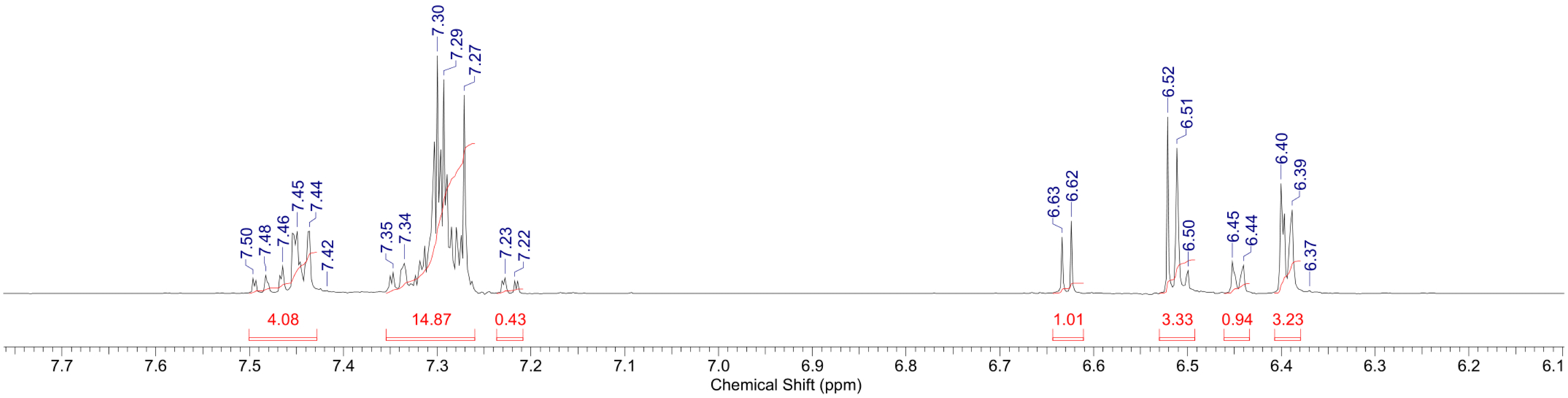

14.07.2020 18:58:34

**Formula** $C_{20}H_{20}ClNO_4$	**FW** 373.8301

**Acquisition Time (sec)**	0.9909	**Comment**	single_pulse	**Date**	10 Feb 2020 10:04:16		
**Date Stamp**	22 Feb 2020 11:50:00			**File Name**	C:\USERS\Лаб534\DOWNLOADS\FZ8510-1.JDF	**Frequency (MHz)**	600.17
**Nucleus**	1H	**Number of Transients**	8	**Origin**	ECA 600	**Original Points Count** 16384	**Owner** CKP
**Points Count**	16384	**Pulse Sequence**	single_pulse.ex2			**Receiver Gain** 38.00	**Solvent** CHLOROFORM-d
**Spectrum Offset (Hz)**	5414.0723	**Sweep Width (Hz)**	16534.39	**Temperature (degree C)**	24.000		

6l

FZ8510-1.JDF

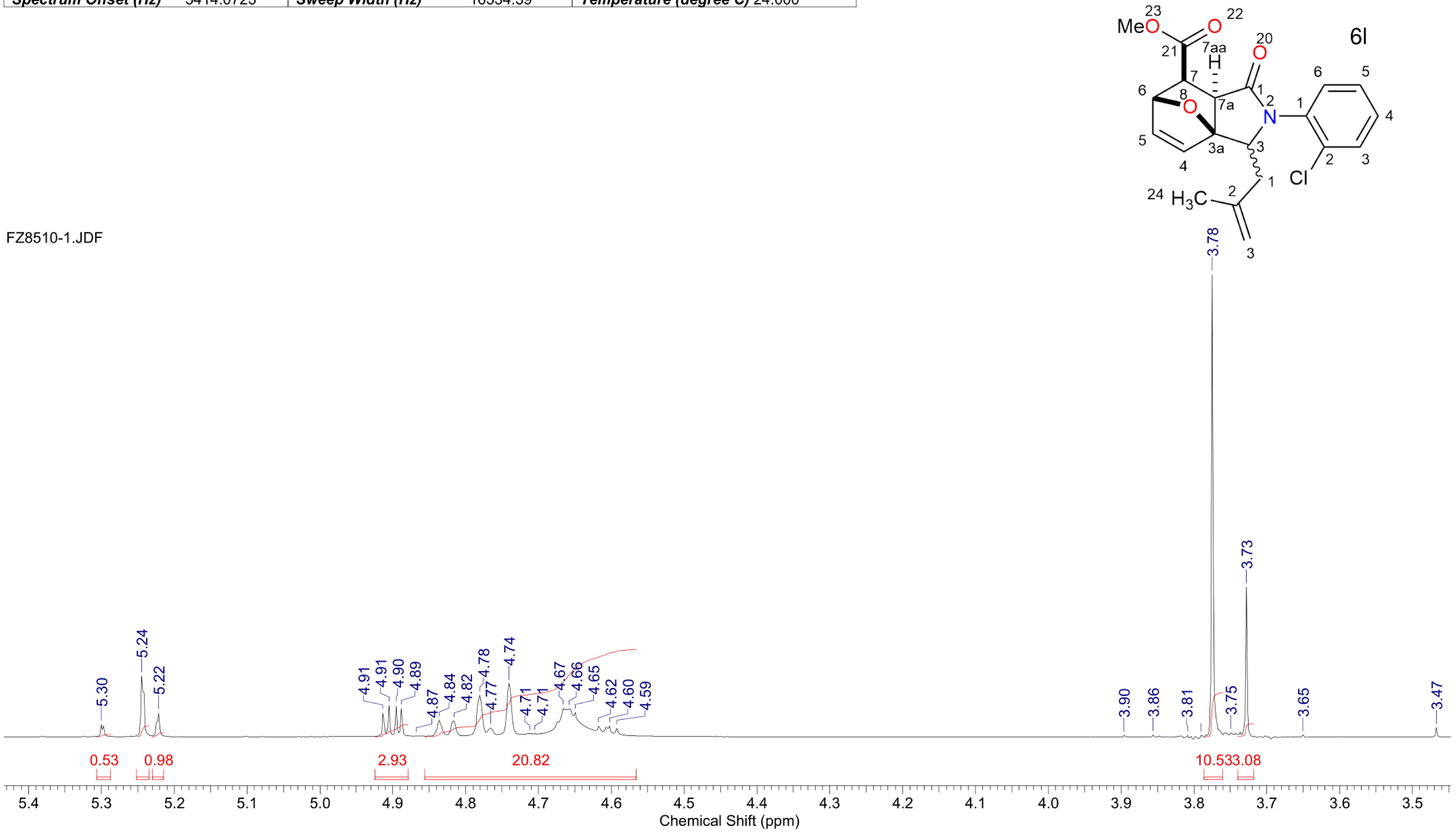

14.07.2020 18:58:44

Formula $C_{20}H_{20}ClNO_4$	FW 373.8301

**Acquisition Time (sec)**	0.9909	**Comment**	single_pulse	**Date**	10 Feb 2020 10:04:16		
**Date Stamp**	22 Feb 2020 11:50:00			**File Name**	C:\USERS\Лаба534\DOWNLOADS\FZ8510-1.JDF	**Frequency (MHz)**	600.17
**Nucleus**	1H	**Number of Transients**	8	**Origin**	ECA 600 **Original Points Count** 16384	**Owner**	CKP
**Points Count**	16384	**Pulse Sequence**	single_pulse.ex2		**Receiver Gain** 38.00	**Solvent**	CHLOROFORM-d
**Spectrum Offset (Hz)**	5414.0723	**Sweep Width (Hz)**	16534.39	**Temperature (degree C)**	24.000		

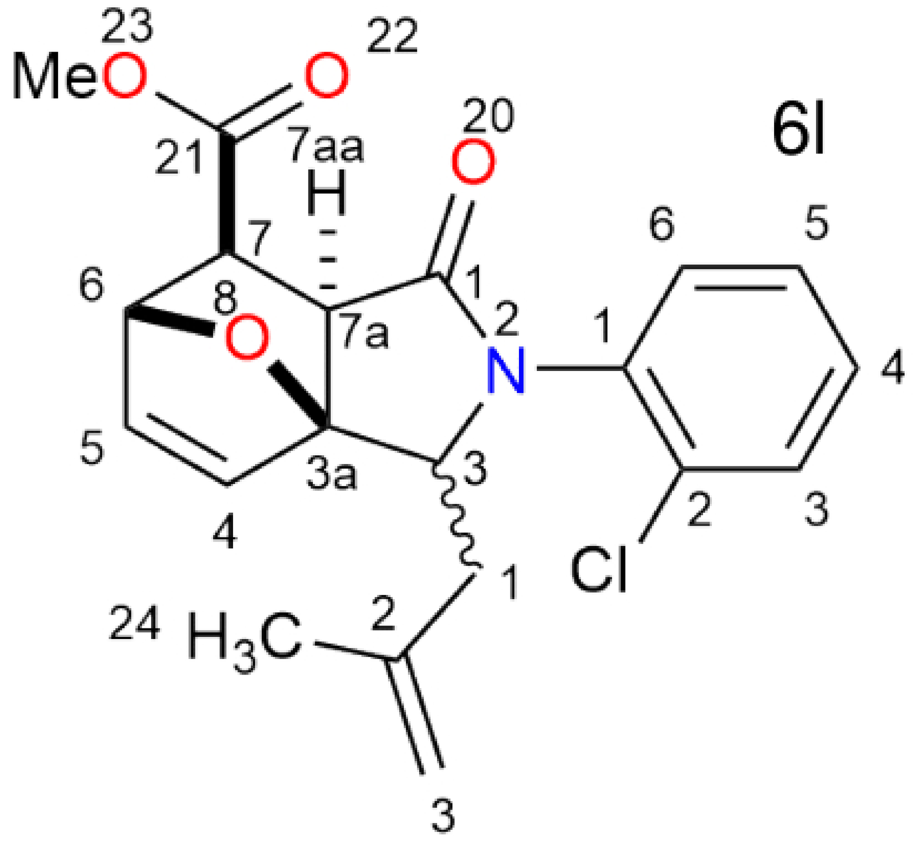

FZ8510-1.JDF

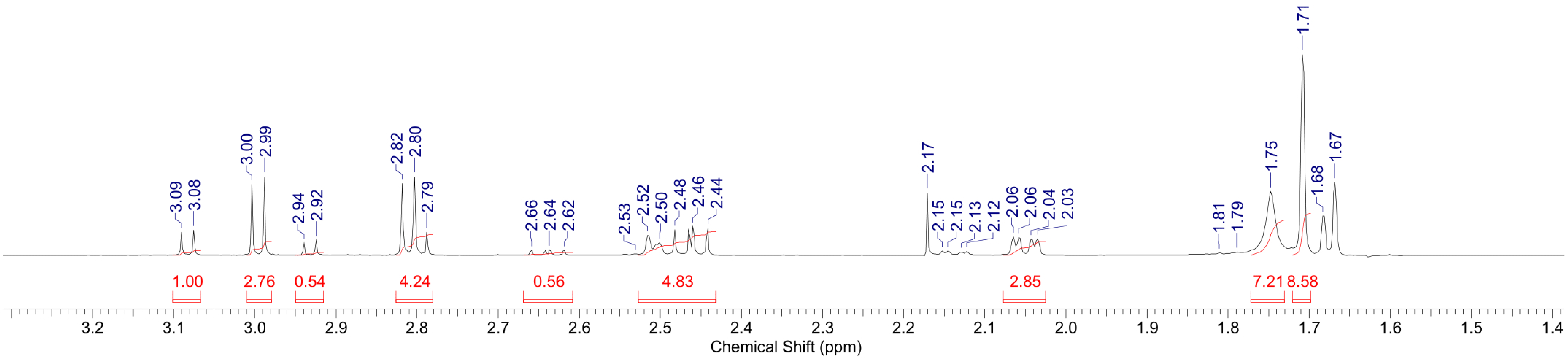

14.07.2020 19:00:31

**Formula**	$C_{20}H_{20}ClNO_4$	**FW**	373.8301

**Acquisition Time (sec)**	0.6921	**Comment**	single pulse decoupled gated NOE			**Date**	18 Mar 2020 11:27:07
**Date Stamp**	18 Mar 2020 10:45:19			**File Name**	C:\USERS\Лаба534\DOWNLOADS\FZ8609-1.JDF		
**Frequency (MHz)**	150.91	**Nucleus**	13C	**Number of Transients**	800	**Origin**	ECA 600
**Original Points Count**	32768	**Owner**	CKP	**Points Count**	32768	**Pulse Sequence**	single_pulse_dec
**Receiver Gain**	56.00	**Solvent**	CHLOROFORM-d			**Spectrum Offset (Hz)**	15091.3428
**Sweep Width (Hz)**	47348.49	**Temperature (degree C)**	23.500				

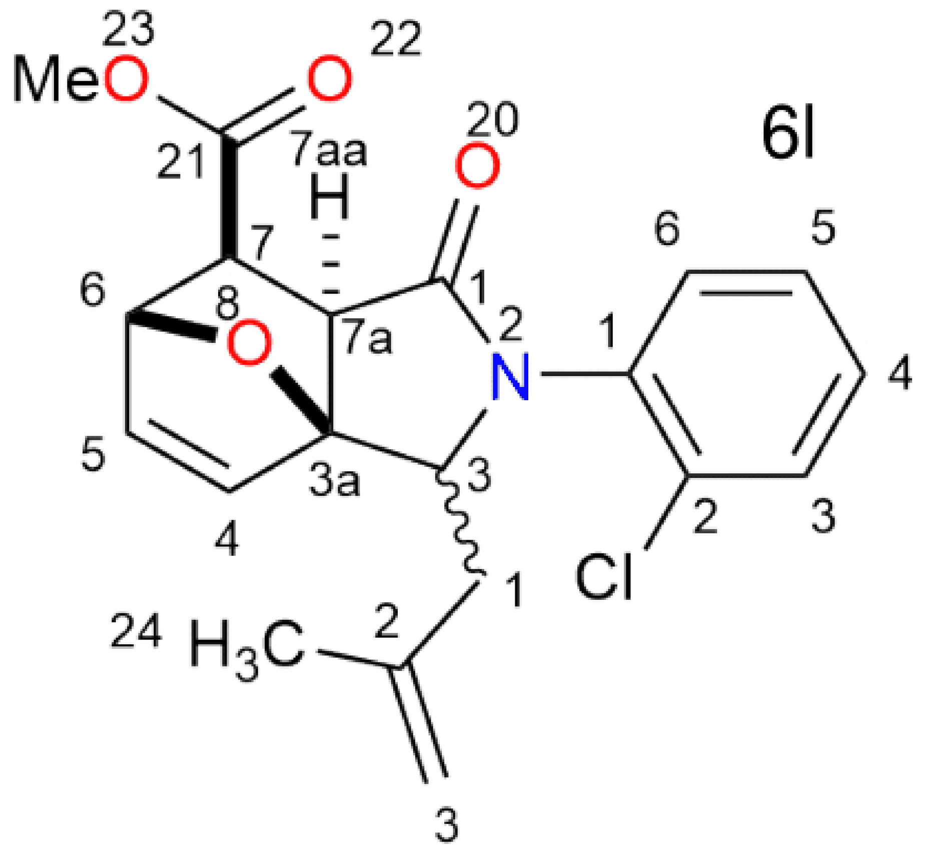

6l

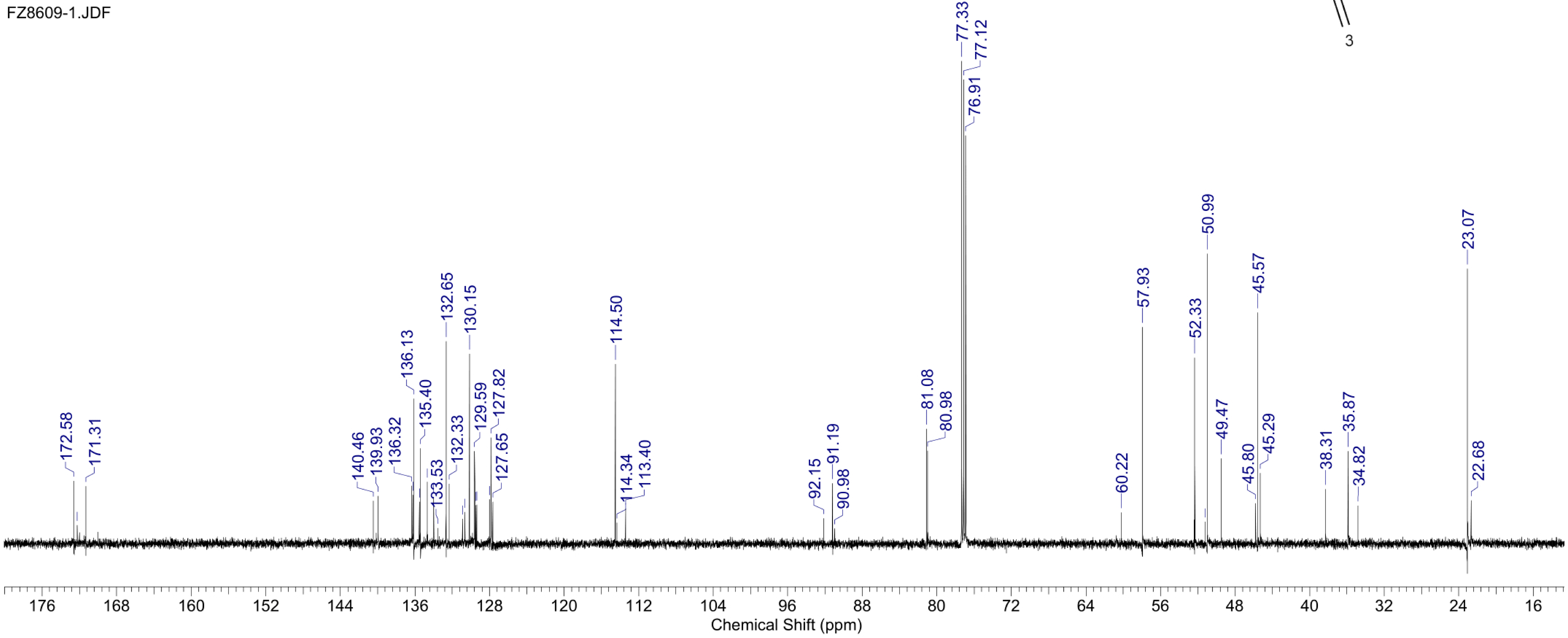

14.07.2020 19:00:43

**Formula** $C_{20}H_{20}ClNO_4$	**FW** 373.8301

**Acquisition Time (sec)**	0.6921	**Comment**	single pulse decoupled gated NOE			**Date**	18 Mar 2020 11:27:07
**Date Stamp**	18 Mar 2020 10:45:19			**File Name**	C:\USERS\Лаба534\DOWNLOADS\FZ8609-1.JDF		
**Frequency (MHz)**	150.91	**Nucleus**	13C	**Number of Transients**	800	**Origin**	ECA 600
**Original Points Count**	32768	**Owner**	CKP	**Points Count**	32768	**Pulse Sequence**	single_pulse_dec
**Receiver Gain**	56.00	**Solvent**	CHLOROFORM-d			**Spectrum Offset (Hz)**	15091.3428
**Sweep Width (Hz)**	47348.49	**Temperature (degree C)**	23.500				

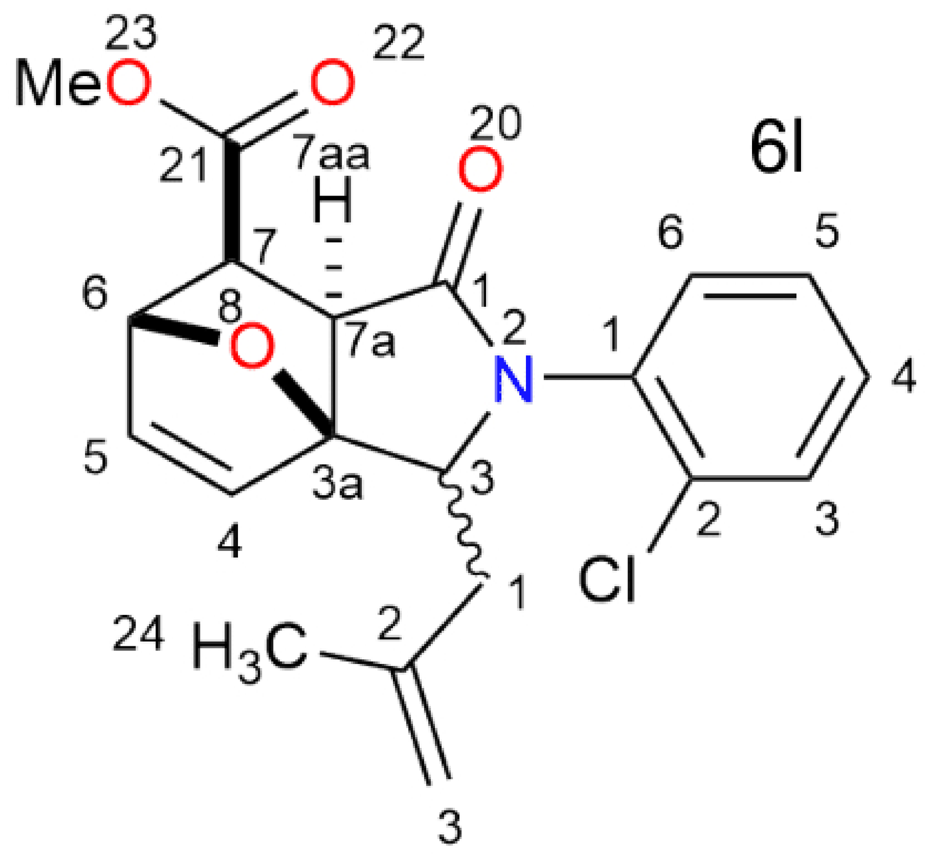

FZ8609-1.JDF

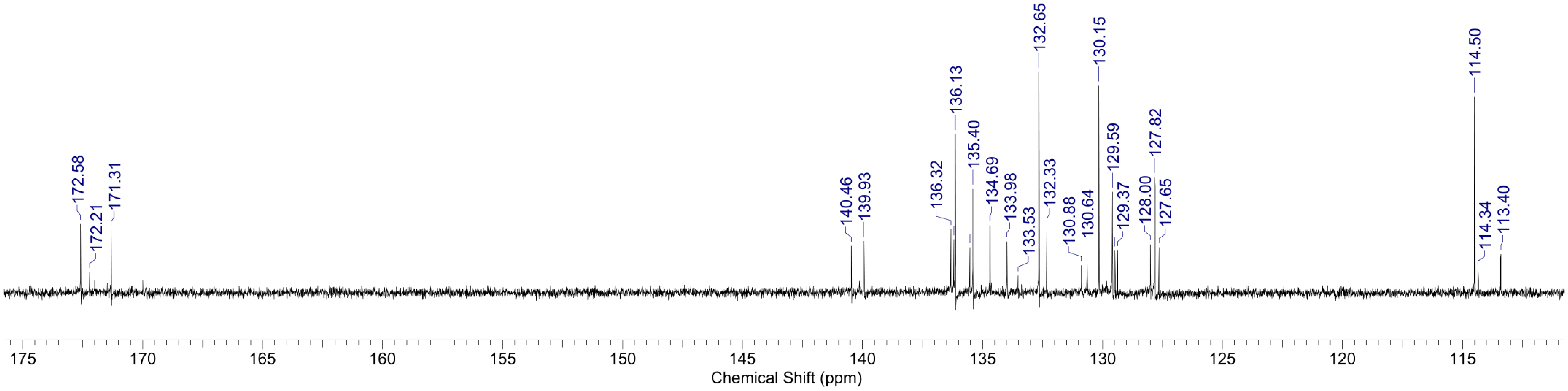

14.07.2020 19:00:59

**Formula** $C_{20}H_{20}ClNO_4$	**FW** 373.8301

**Acquisition Time (sec)**	0.6921	**Comment**	single pulse decoupled gated NOE			**Date**	18 Mar 2020 11:27:07
**Date Stamp**	18 Mar 2020 10:45:19			**File Name**	C:\USERS\Лаба534\DOWNLOADS\FZ8609-1.JDF		
**Frequency (MHz)**	150.91	**Nucleus**	13C	**Number of Transients**	800	**Origin**	ECA 600
**Original Points Count**	32768	**Owner**	CKP	**Points Count**	32768	**Pulse Sequence**	single_pulse_dec
**Receiver Gain**	56.00	**Solvent**	CHLOROFORM-d			**Spectrum Offset (Hz)**	15091.3428
**Sweep Width (Hz)**	47348.49	**Temperature (degree C)**	23.500				

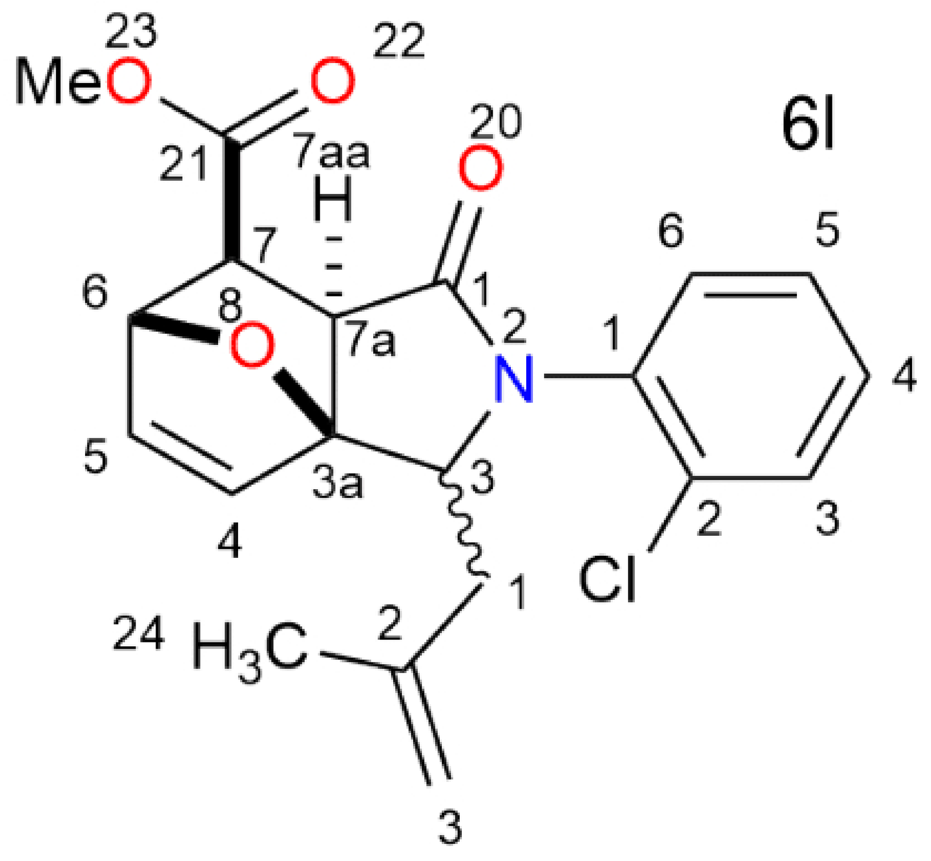

6l

FZ8609-1.JDF

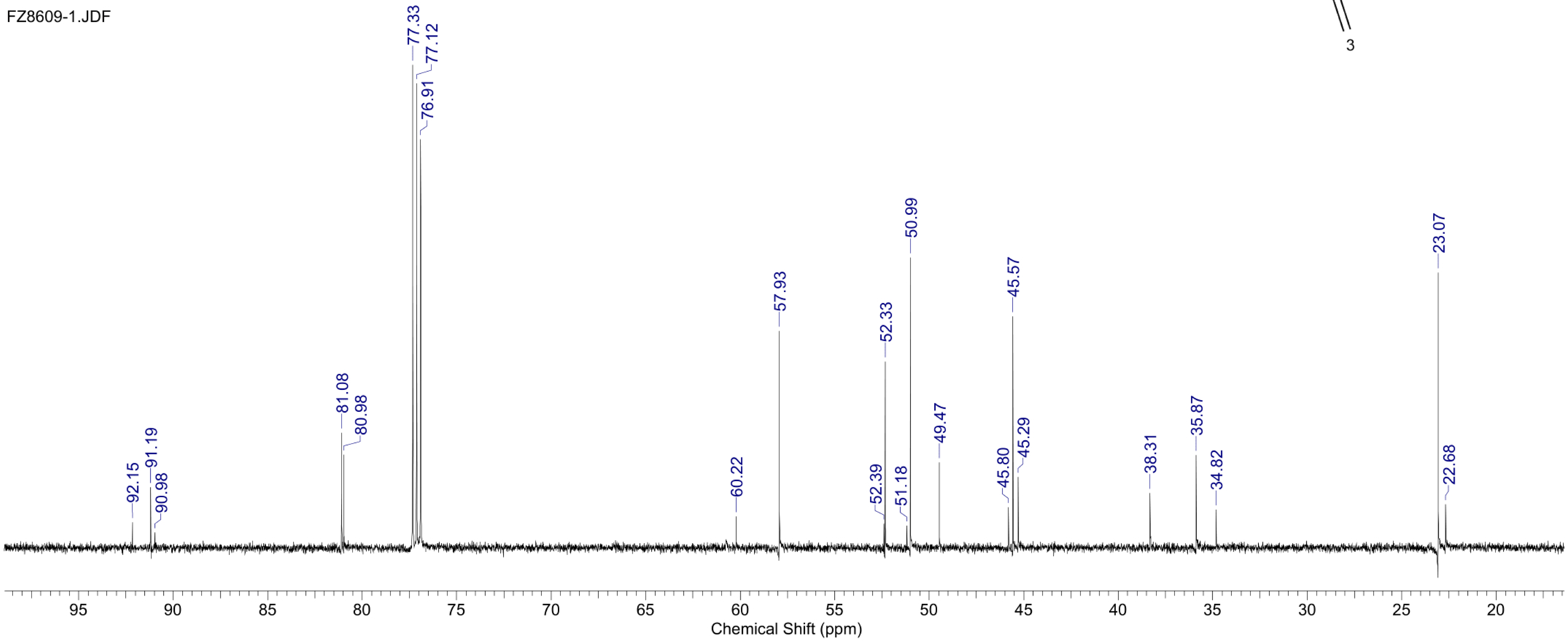

NMR spectrum data for compound 6o

**Formula** $C_{20}H_{20}ClNO_4$	**FW** 373.8301

Acquisition Time (sec)	1.9818	***Comment***	single_pulse	***Date***	07 Feb 2020 12:28:25		
Date Stamp	07 Feb 2020 11:50:07			***File Name***	C:\USERS\лаба 534\DOWNLOADS\FZ8422-1 (2).JDF		
Frequency (MHz)	600.17	***Nucleus***	1H	***Number of Transients***	8	***Origin***	ECA 600
Original Points Count	32768	***Owner***	Mass	***Points Count***	32768	***Pulse Sequence***	single_pulse.ex2
Receiver Gain	34.00	***Solvent***	CHLOROFORM-d			***Spectrum Offset (Hz)***	5415.1689
Sweep Width (Hz)	16534.39	***Temperature (degree C)***	23.900				

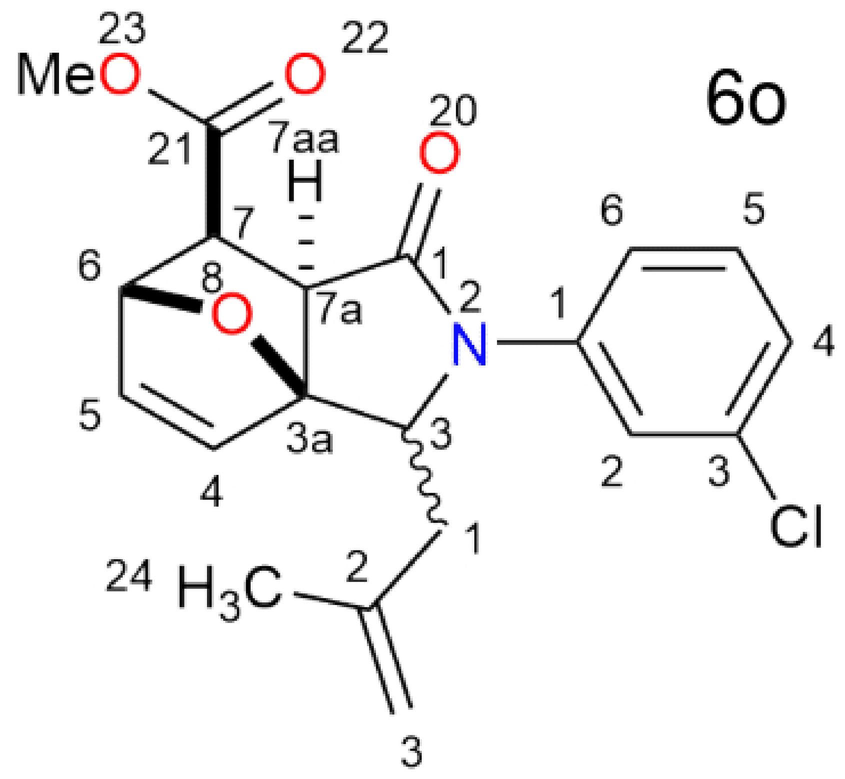

6o

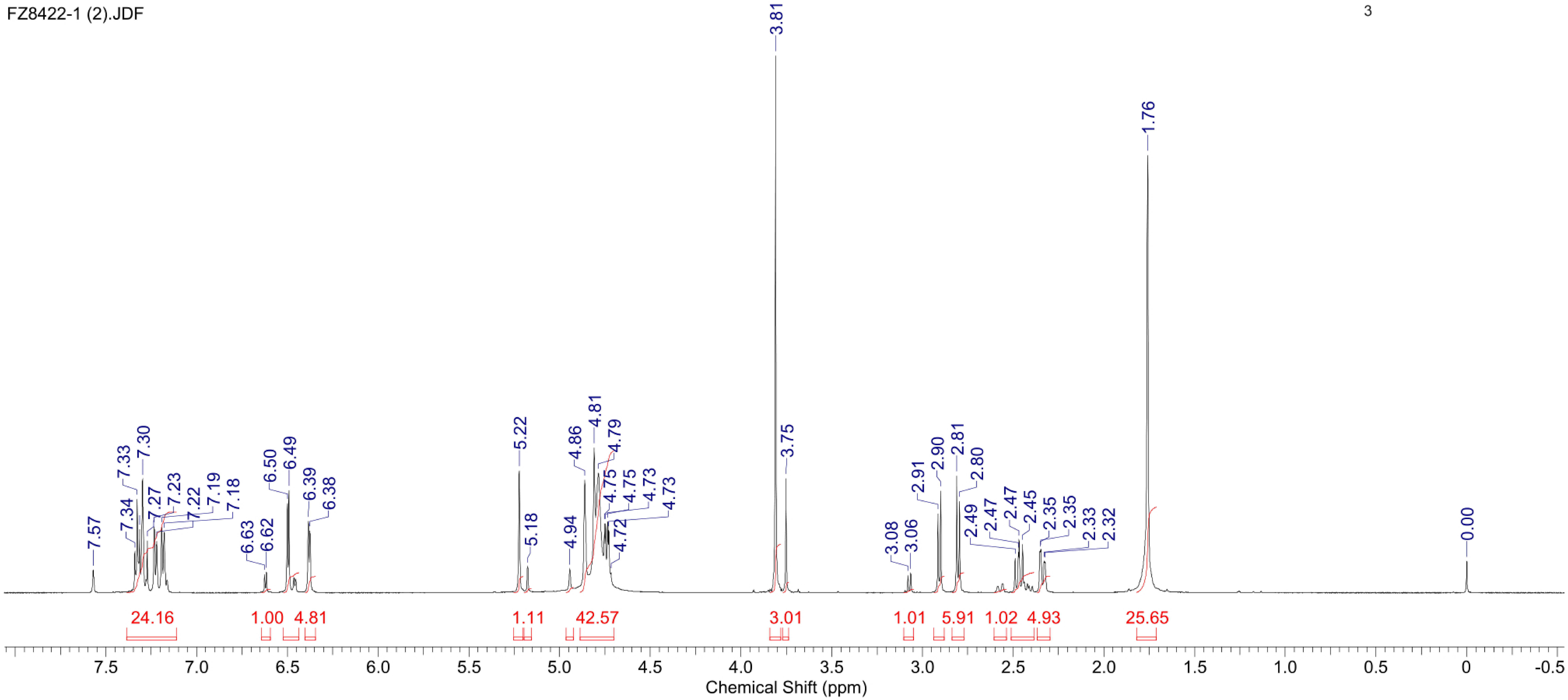

**Formula** $C_{20}H_{20}ClNO_4$	**FW** 373.8301

Acquisition Time (sec)	1.9818	***Comment***	single_pulse	***Date***	07 Feb 2020 12:28:25		
Date Stamp	07 Feb 2020 11:50:07			***File Name***	C:\USERS\па6а 534\DOWNLOADS\FZ8422-1 (2).JDF		
Frequency (MHz)	600.17	***Nucleus***	1H	***Number of Transients***	8	***Origin***	ECA 600
Original Points Count	32768	***Owner***	Mass	***Points Count***	32768	***Pulse Sequence***	single_pulse.ex2
Receiver Gain	34.00	***Solvent***	CHLOROFORM-d			***Spectrum Offset (Hz)***	5415.1689
Sweep Width (Hz)	16534.39	***Temperature (degree C)***	23.900				

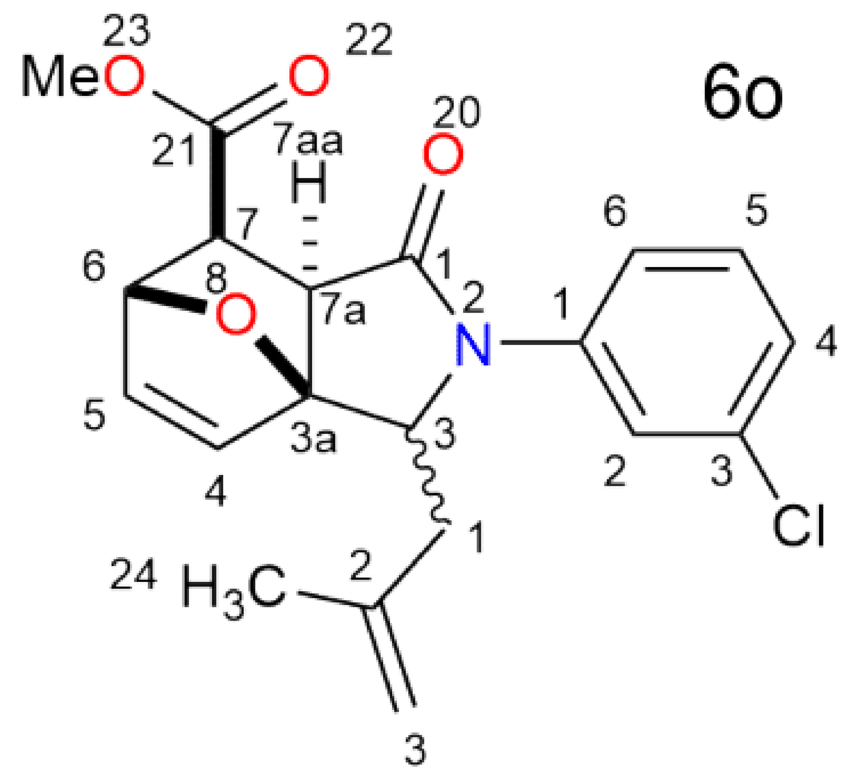

6o

FZ8422-1 (2).JDF

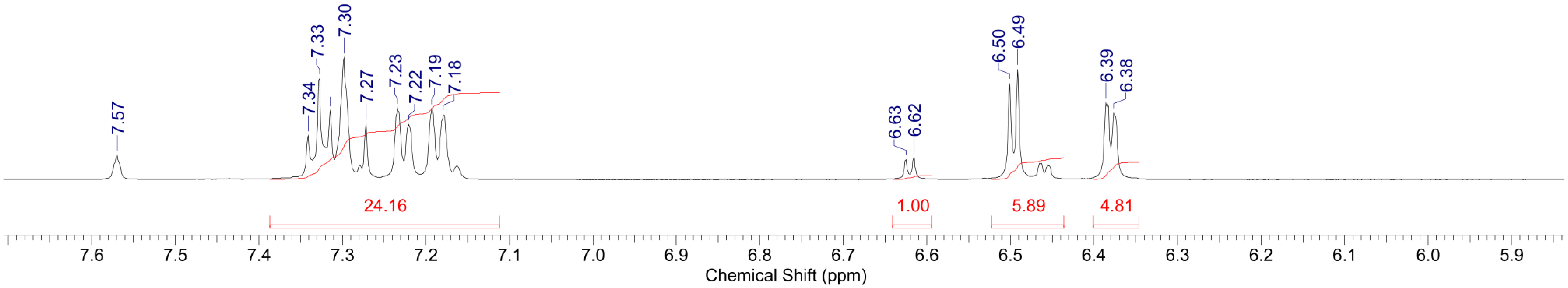

Formula $C_{20}H_{20}ClNO_4$	FW 373.8301

**Acquisition Time (sec)**	1.9818	**Comment**	single_pulse	**Date**	07 Feb 2020 12:28:25		
**Date Stamp**	07 Feb 2020 11:50:07			**File Name**	C:\USERS\па6а 534\DOWNLOADS\FZ8422-1 (2).JDF		
**Frequency (MHz)**	600.17	**Nucleus**	1H	**Number of Transients**	8	**Origin**	ECA 600
**Original Points Count**	32768	**Owner**	Mass	**Points Count**	32768	**Pulse Sequence**	single_pulse.ex2
**Receiver Gain**	34.00	**Solvent**	CHLOROFORM-d			**Spectrum Offset (Hz)**	5415.1689
**Sweep Width (Hz)**	16534.39	**Temperature (degree C)**	23.900				

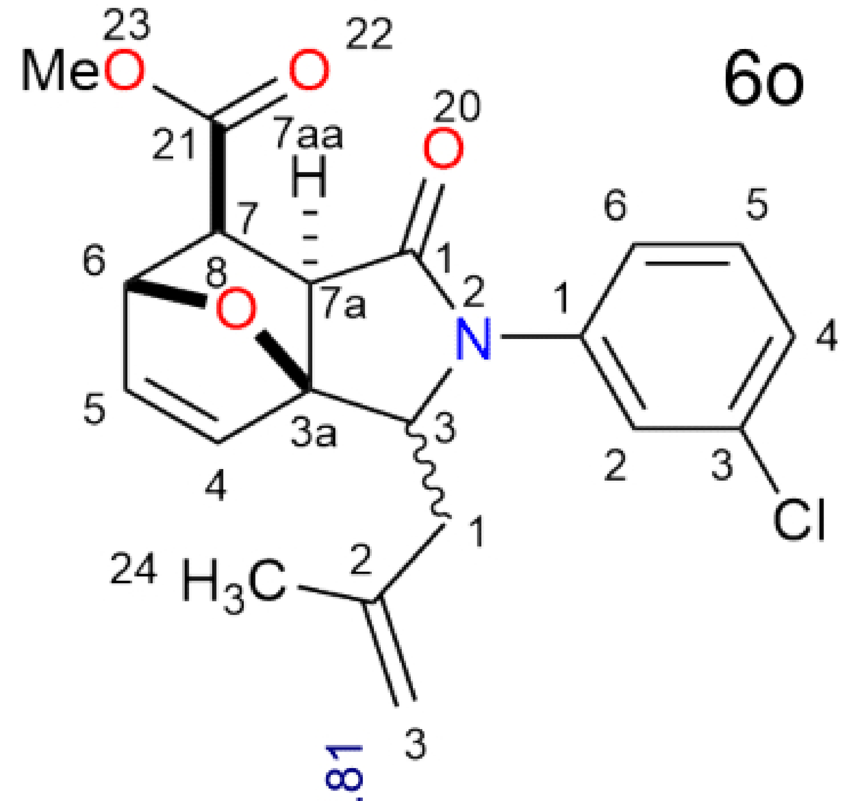

6o

FZ8422-1 (2).JDF

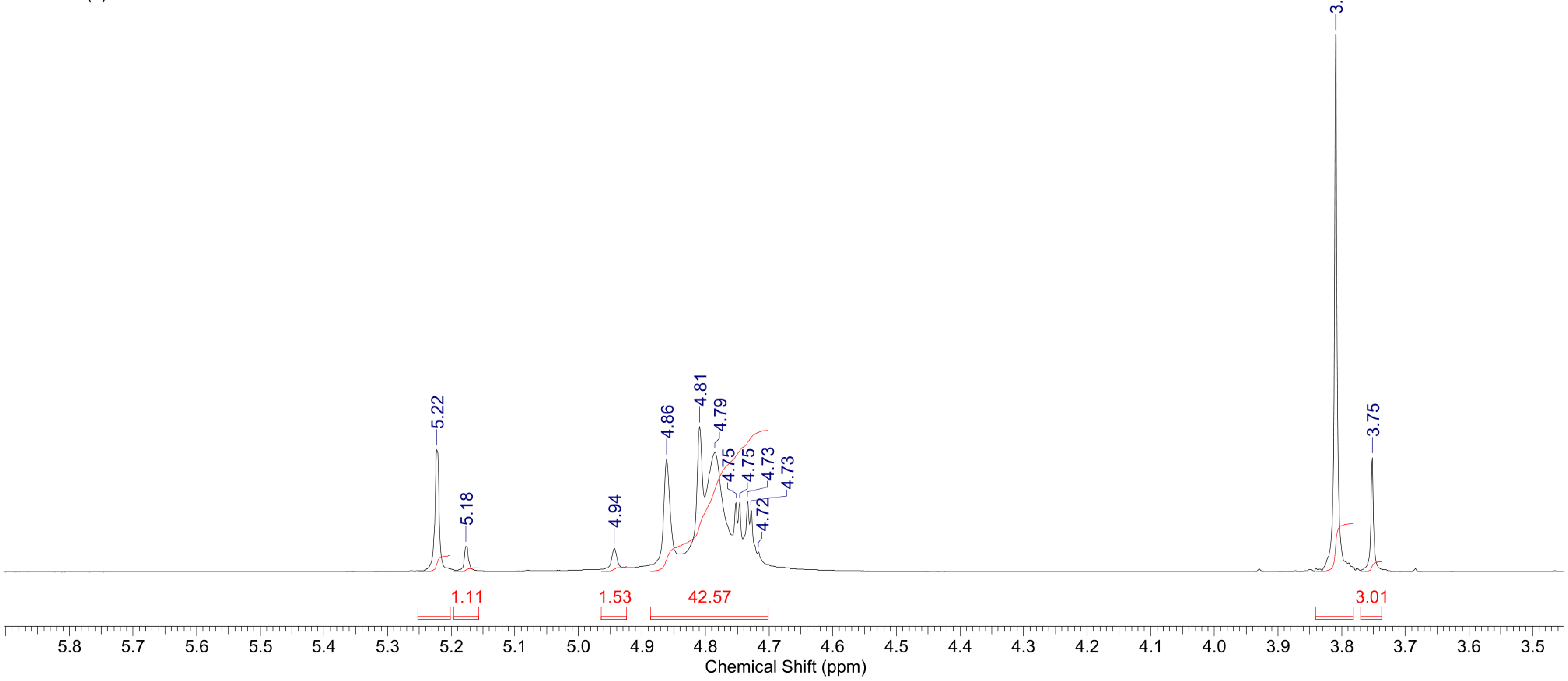

Formula $C_{20}H_{20}ClNO_4$	FW 373.8301

Acquisition Time (sec)	1.9818	***Comment***	single_pulse	***Date***	07 Feb 2020 12:28:25		
Date Stamp	07 Feb 2020 11:50:07			***File Name***	C:\USERS\па6а 534\DOWNLOADS\FZ8422-1 (2).JDF		
Frequency (MHz)	600.17	***Nucleus***	1H	***Number of Transients***	8	***Origin***	ECA 600
Original Points Count	32768	***Owner***	Mass	***Points Count***	32768	***Pulse Sequence***	single_pulse.ex2
Receiver Gain	34.00	***Solvent***	CHLOROFORM-d			***Spectrum Offset (Hz)***	5415.1689
Sweep Width (Hz)	16534.39	***Temperature (degree C)***	23.900				

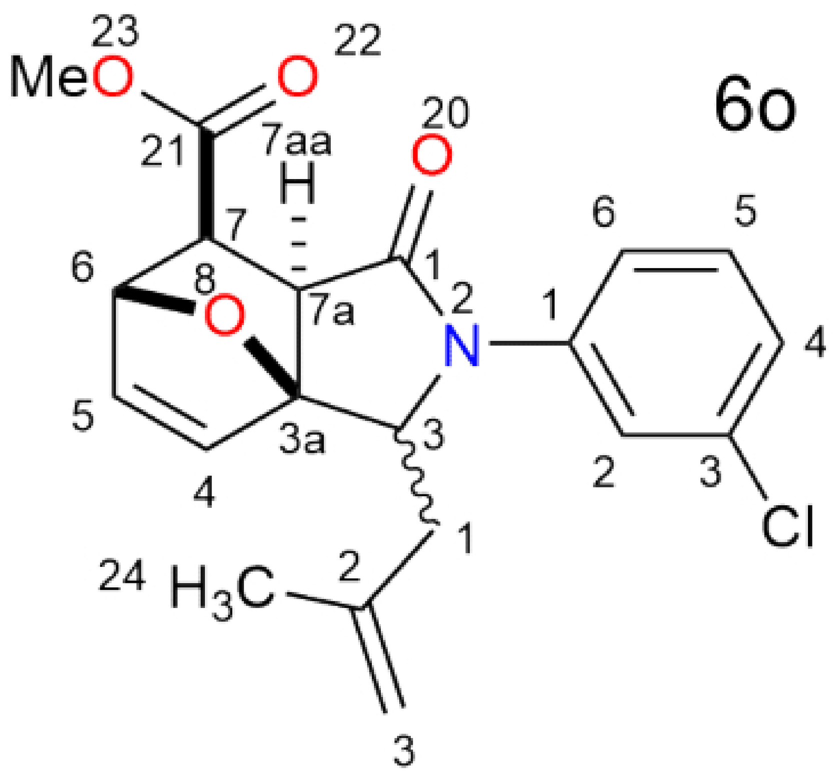

FZ8422-1 (2).JDF

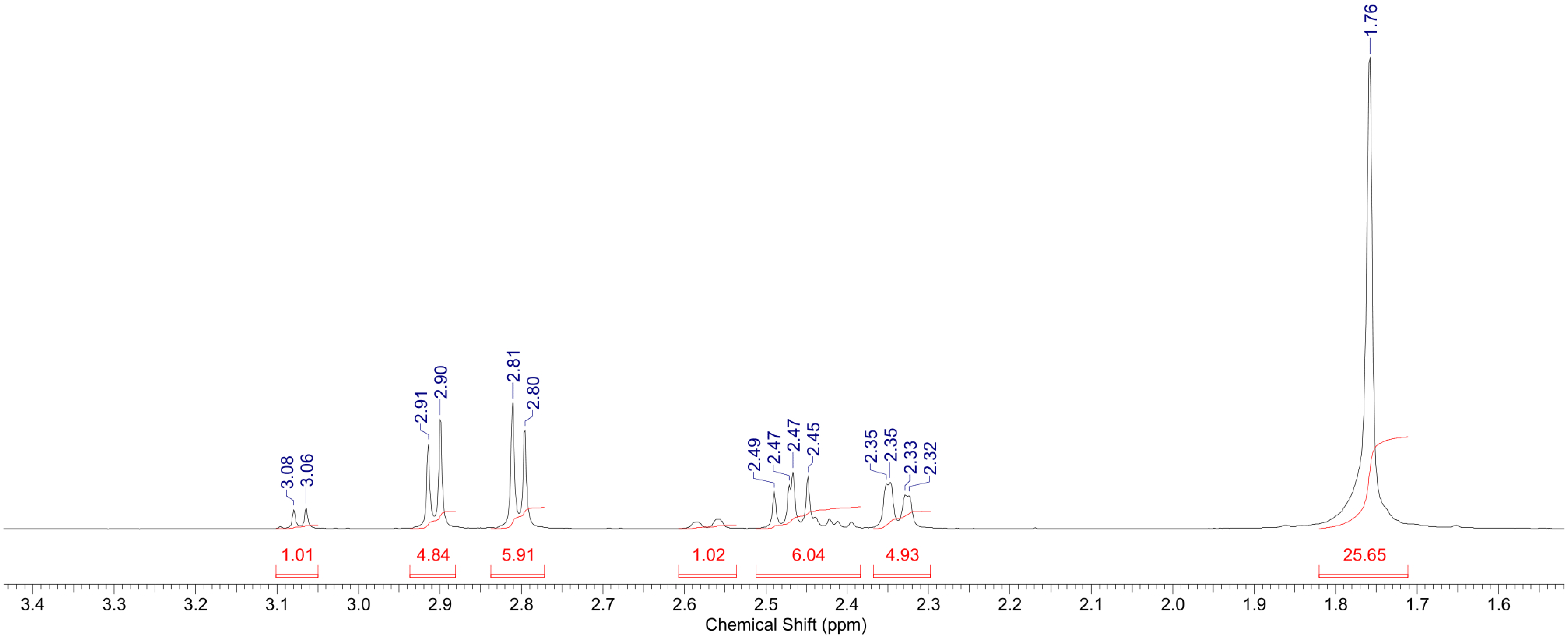

**Formula** $C_{20}H_{20}ClNO_4$	**FW** 373.8301

**Acquisition Time (sec)**	0.6921	**Comment**	single pulse decoupled gated NOE			**Date**	07 Mar 2020 11:10:22
**Date Stamp**	07 Mar 2020 10:30:17			**File Name**	C:\USERS\лаба 534\DESKTOP\Саша\ЯМР\NMR FIDS\FZ8578-1.JDF		
**Frequency (MHz)**	150.91	**Nucleus**	13C	**Number of Transients**	1000	**Origin**	ECA 600
**Original Points Count**	32768	**Owner**	CKP	**Points Count**	32768	**Pulse Sequence**	single_pulse_dec
**Receiver Gain**	52.00	**Solvent**	CHLOROFORM-d			**Spectrum Offset (Hz)**	15079.3525
**Sweep Width (Hz)**	47348.49	**Temperature (degree C)**	23.500				

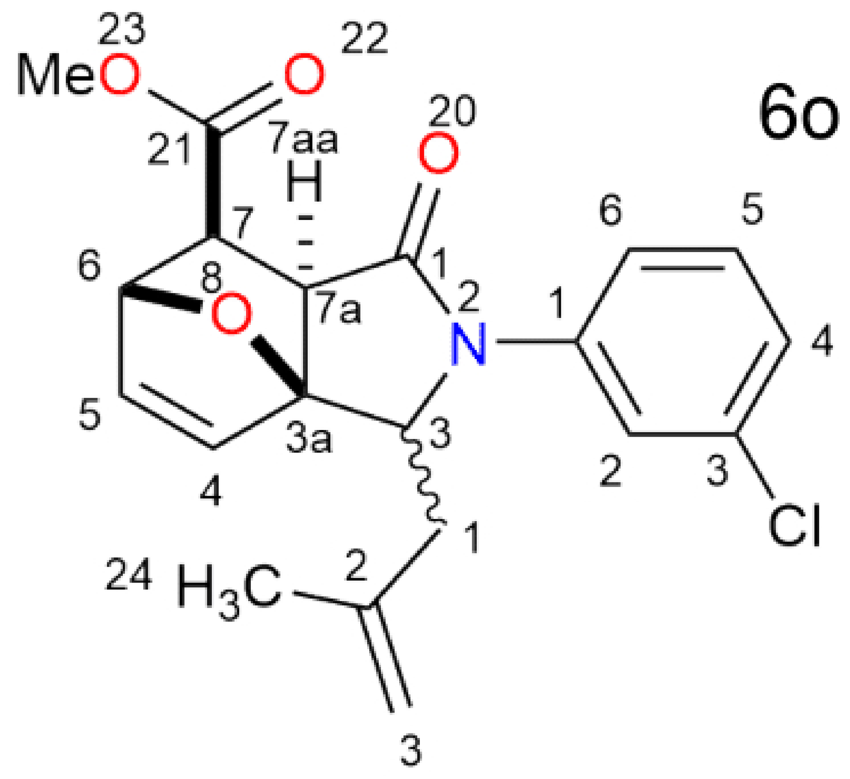

6o

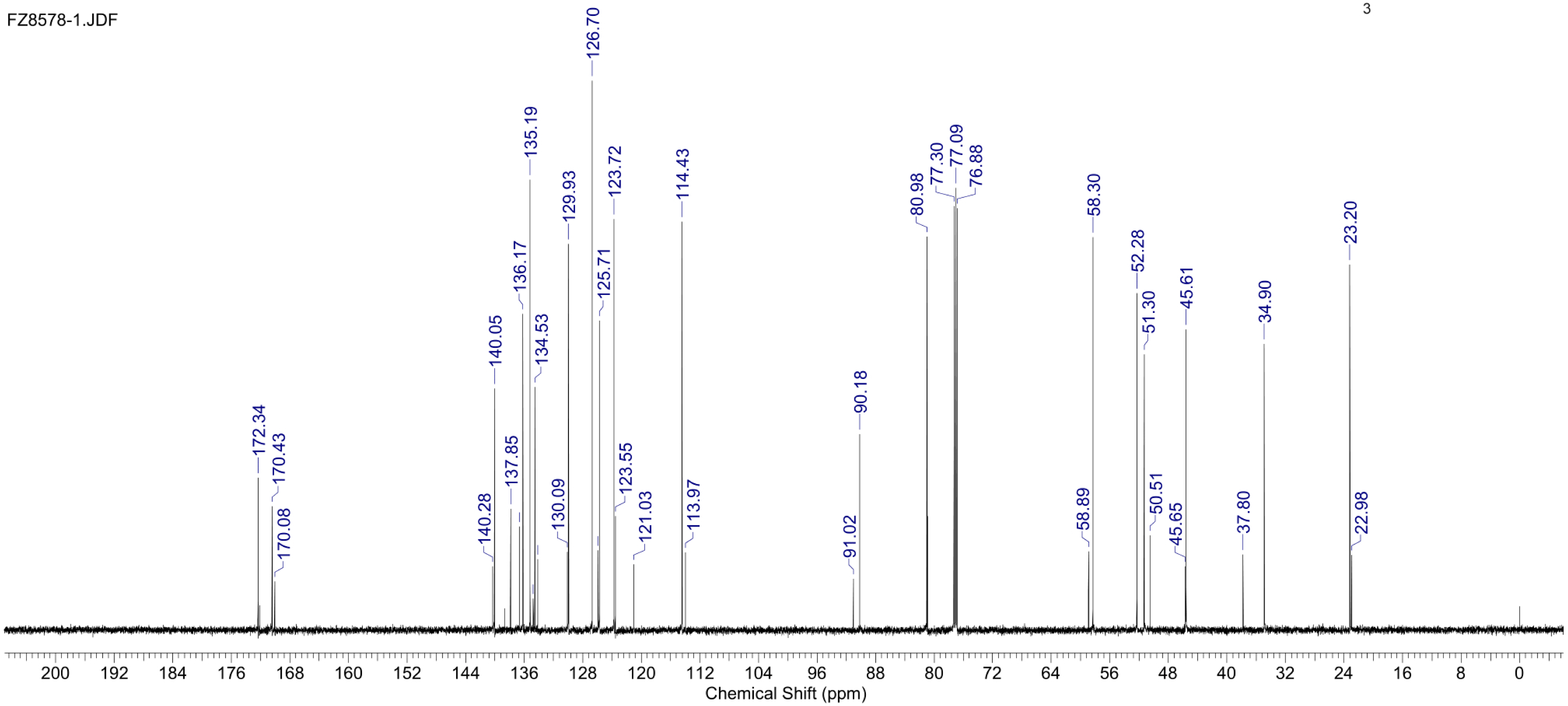

Formula $C_{20}H_{20}ClNO_4$	***FW*** 373.8301

Acquisition Time (sec)	0.6921	***Comment***	single pulse decoupled gated NOE			***Date***	07 Mar 2020 11:10:22
Date Stamp	07 Mar 2020 10:30:17			***File Name***	C:\USERS\лаба 534\DESKTOP\Саша\ЯМР\NMR FIDS\FZ8578-1.JDF		
Frequency (MHz)	150.91	***Nucleus***	13C	***Number of Transients***	1000	***Origin***	ECA 600
Original Points Count	32768	***Owner***	CKP	***Points Count***	32768	***Pulse Sequence***	single_pulse_dec
Receiver Gain	52.00	***Solvent***	CHLOROFORM-d			***Spectrum Offset (Hz)***	15079.3525
Sweep Width (Hz)	47348.49	***Temperature (degree C)***	23.500				

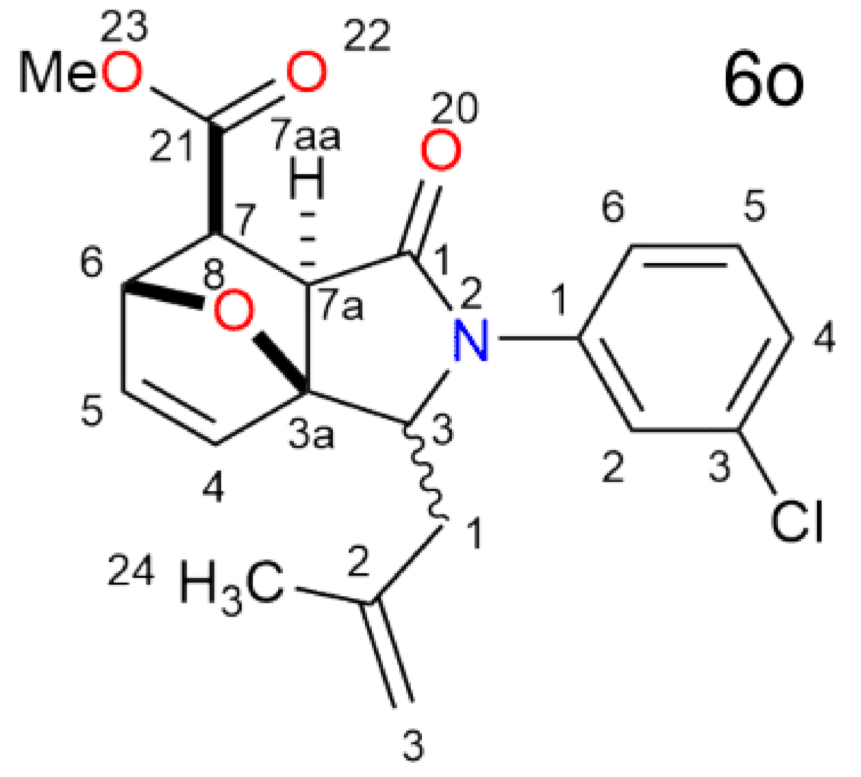

6o

FZ8578-1.JDF

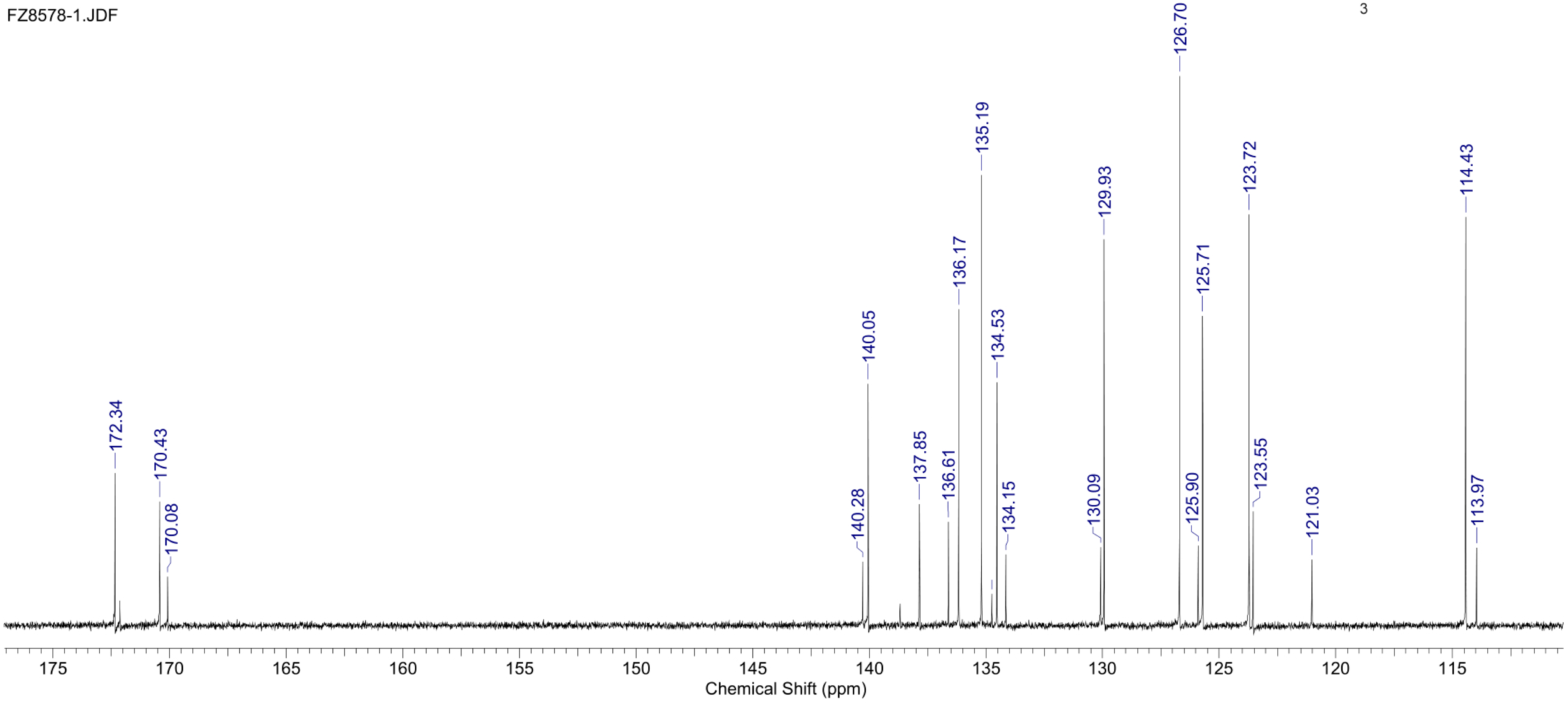

**Formula** $C_{20}H_{20}ClNO_4$	**FW** 373.8301

**Acquisition Time (sec)**	0.6921	**Comment**	single pulse decoupled gated NOE	**Date**	07 Mar 2020 11:10:22
**Date Stamp**	07 Mar 2020 10:30:17	**File Name**	C:\USERS\лаба 534\DESKTOP\Саша\ЯМР\NMR FIDS\FZ8578-1.JDF		
**Frequency (MHz)**	150.91	**Nucleus**	13C	**Number of Transients**	1000
**Origin**	ECA 600	**Original Points Count**	32768	**Owner**	CKP
**Points Count**	32768	**Pulse Sequence**	single_pulse_dec	**Receiver Gain**	52.00
**Solvent**	CHLOROFORM-d	**Spectrum Offset (Hz)**	15079.3525	**Sweep Width (Hz)**	47348.49
**Temperature (degree C)**	23.500				

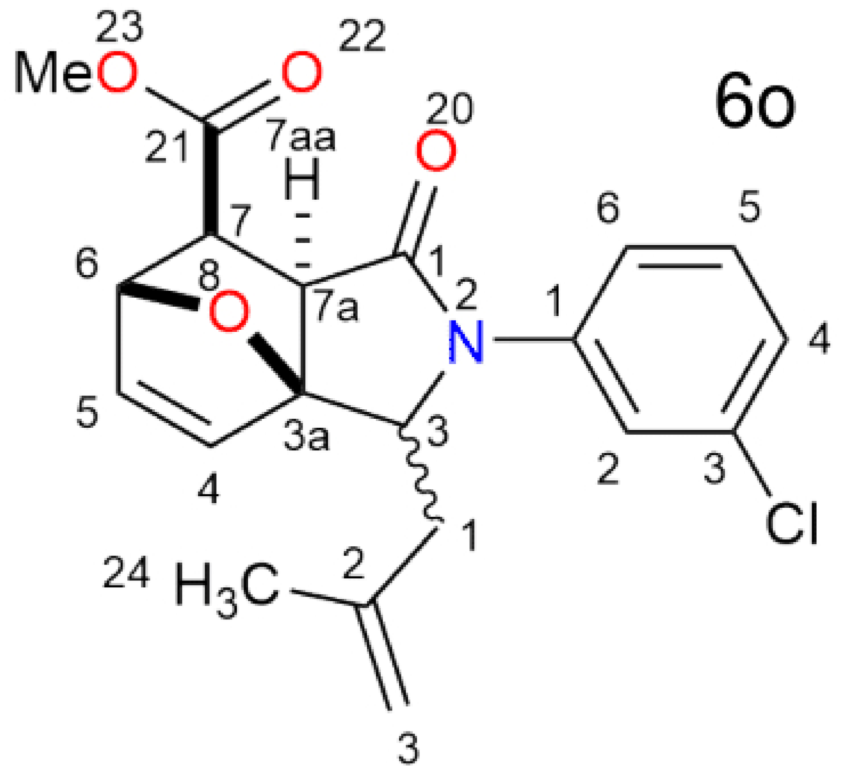

FZ8578-1.JDF

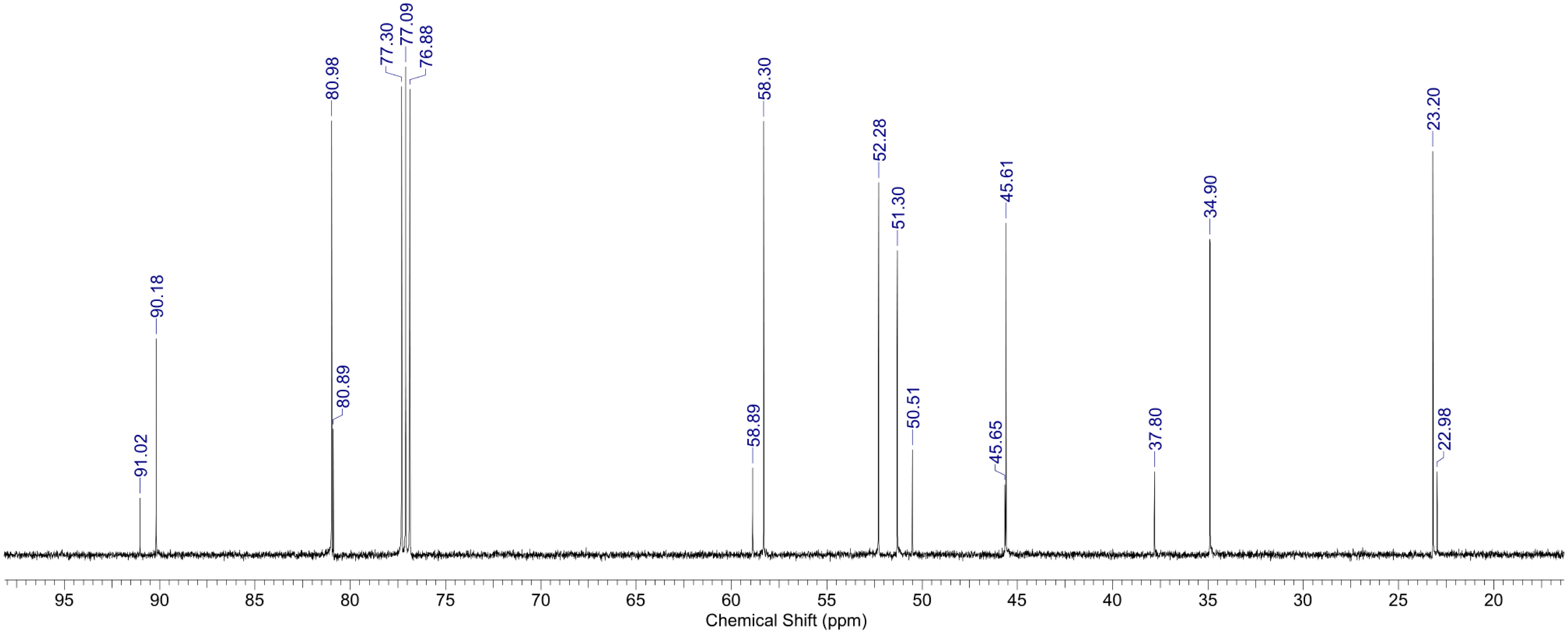

NMR spectrum data for compound 6p

**Acquisition Time (sec)**	1.9818	**Comment**	single_pulse	**Date**	28 May 1990 07:37:44		
**Date Stamp**	07 Aug 2019 13:41:10			**File Name**	C:\Users\Fedor\Desktop\07.08.19\FZ7650-1.jdf	**Frequency (MHz)**	600.17
**Nucleus**	1H	**Number of Transients**	8	**Origin**	ECA 600	**Original Points Count** 32768	**Owner** delta
**Points Count**	32768	**Pulse Sequence**	single_pulse.ex2			**Receiver Gain** 42.00	**Solvent** CHLOROFORM-d
**Spectrum Offset (Hz)**	5411.6367	**Sweep Width (Hz)**	16534.39	**Temperature (degree C)**	22.100		

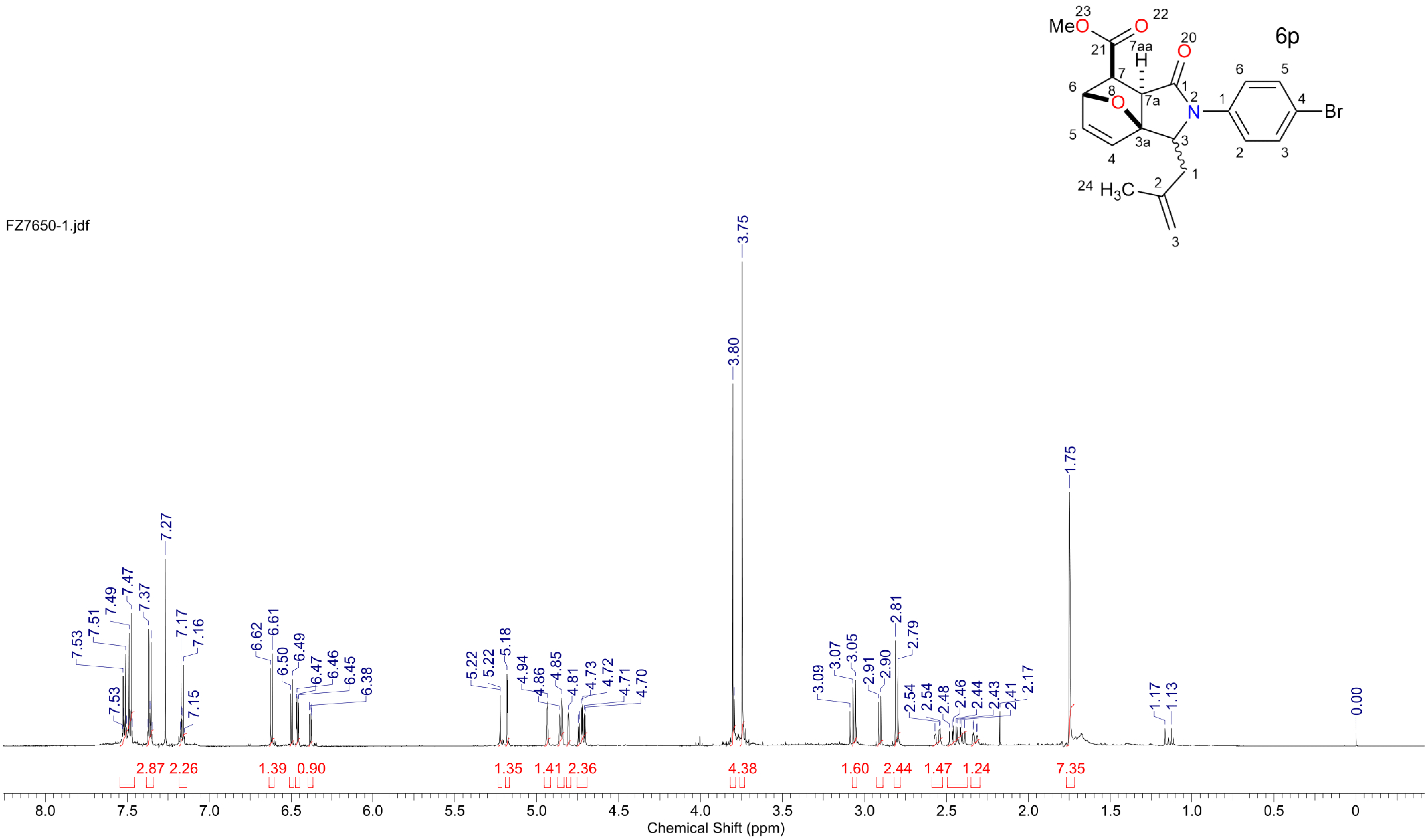

Acquisition Time (sec)	1.9818	Comment	single_pulse	Date	28 May 1990 07:37:44		
Date Stamp	07 Aug 2019 13:41:10			File Name	C:\Users\Fedor\Desktop\07.08.19\FZ7650-1.jdf	Frequency (MHz)	600.17
Nucleus	1H	Number of Transients	8	Origin	ECA 600 / Original Points Count 32768	Owner	delta
Points Count	32768	Pulse Sequence	single_pulse.ex2		Receiver Gain 42.00	Solvent	CHLOROFORM-d
Spectrum Offset (Hz)	5411.6367	Sweep Width (Hz)	16534.39	Temperature (degree C)	22.100		

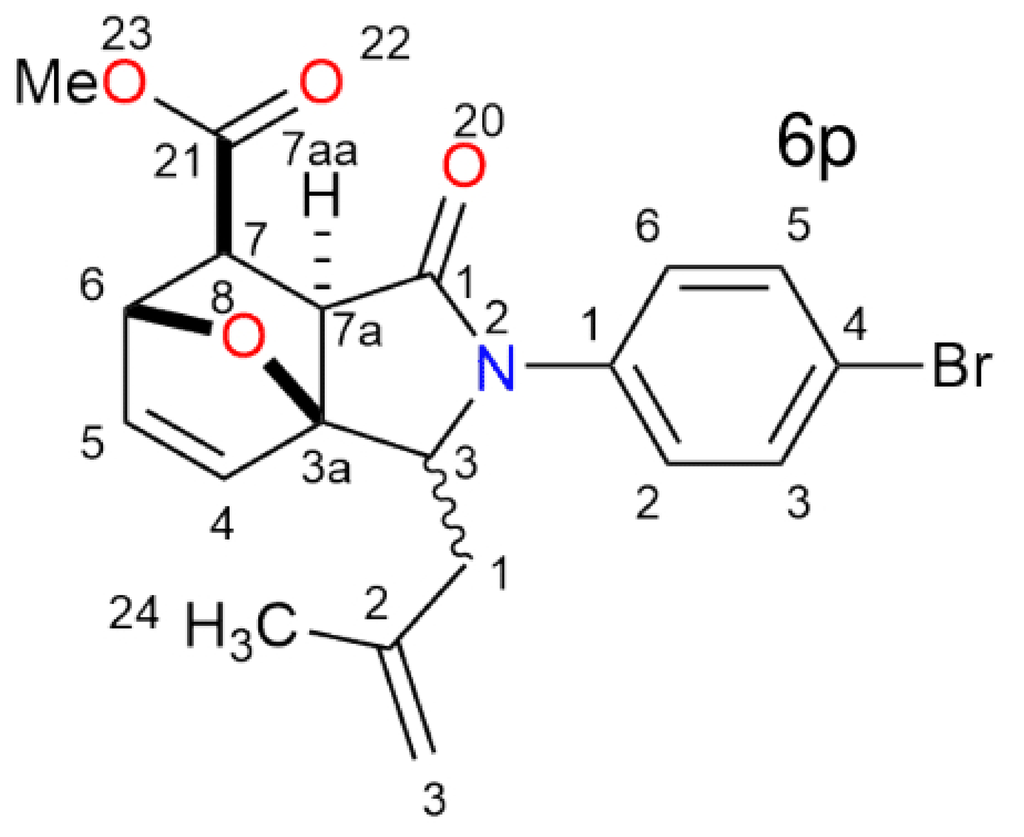

6p

FZ7650-1.jdf

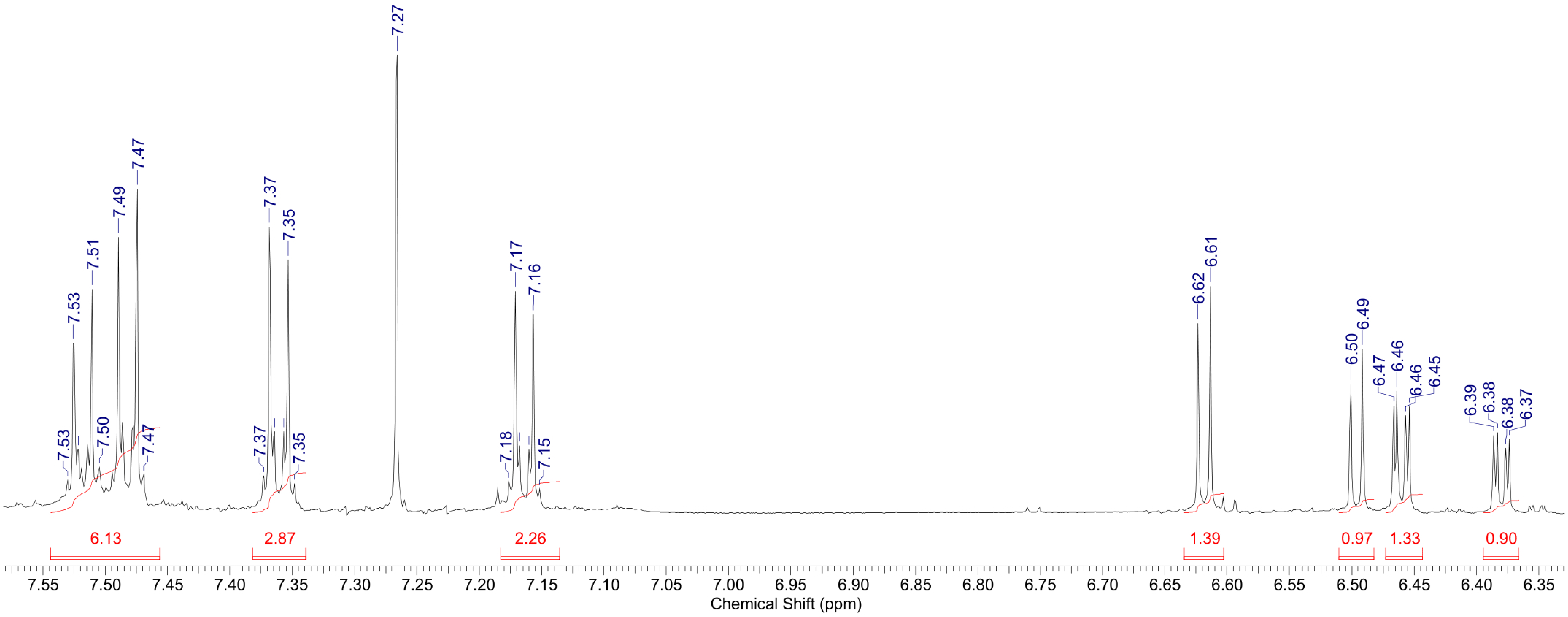

**Acquisition Time (sec)**	1.9818	**Comment**	single_pulse	**Date**	28 May 1990 07:37:44		
**Date Stamp**	07 Aug 2019 13:41:10			**File Name**	C:\Users\Fedor\Desktop\07.08.19\FZ7650-1.jdf	**Frequency (MHz)**	600.17
**Nucleus**	1H	**Number of Transients**	8	**Origin**	ECA 600	**Original Points Count** 32768	**Owner** delta
**Points Count**	32768	**Pulse Sequence**	single_pulse.ex2			**Receiver Gain** 42.00	**Solvent** CHLOROFORM-d
**Spectrum Offset (Hz)**	5411.6367	**Sweep Width (Hz)**	16534.39	**Temperature (degree C)**	22.100		

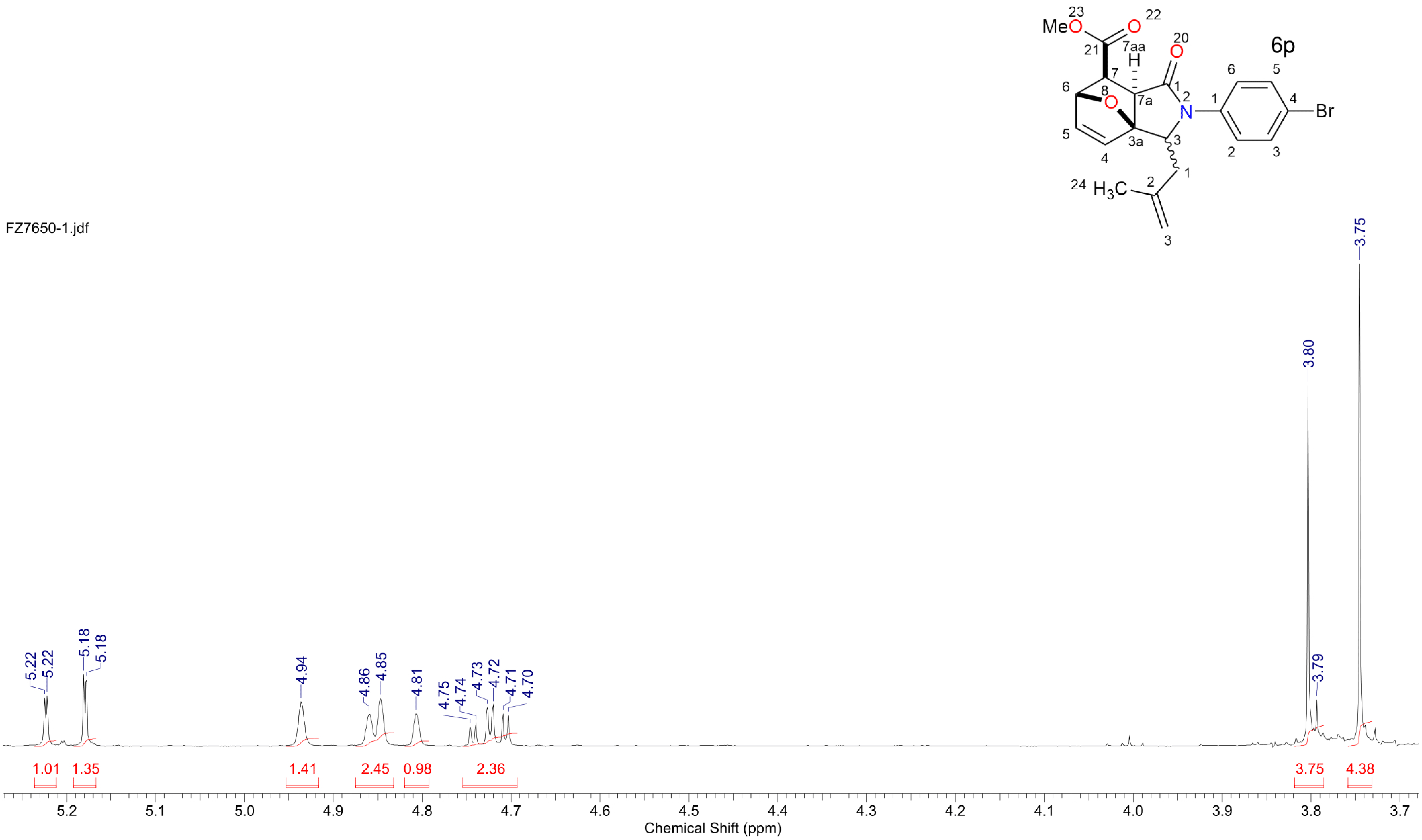

Acquisition Time (sec)	1.9818	***Comment***	single_pulse	***Date***	28 May 1990 07:37:44		
Date Stamp	07 Aug 2019 13:41:10			***File Name***	C:\Users\Fedor\Desktop\07.08.19\FZ7650-1.jdf	***Frequency (MHz)***	600.17
Nucleus	1H	***Number of Transients***	8	***Origin***	ECA 600	***Original Points Count***	32768
						Owner	delta
Points Count	32768	***Pulse Sequence***	single_pulse.ex2			***Receiver Gain***	42.00
						Solvent	CHLOROFORM-d
Spectrum Offset (Hz)	5411.6367	***Sweep Width (Hz)***	16534.39	***Temperature (degree C)***	22.100		

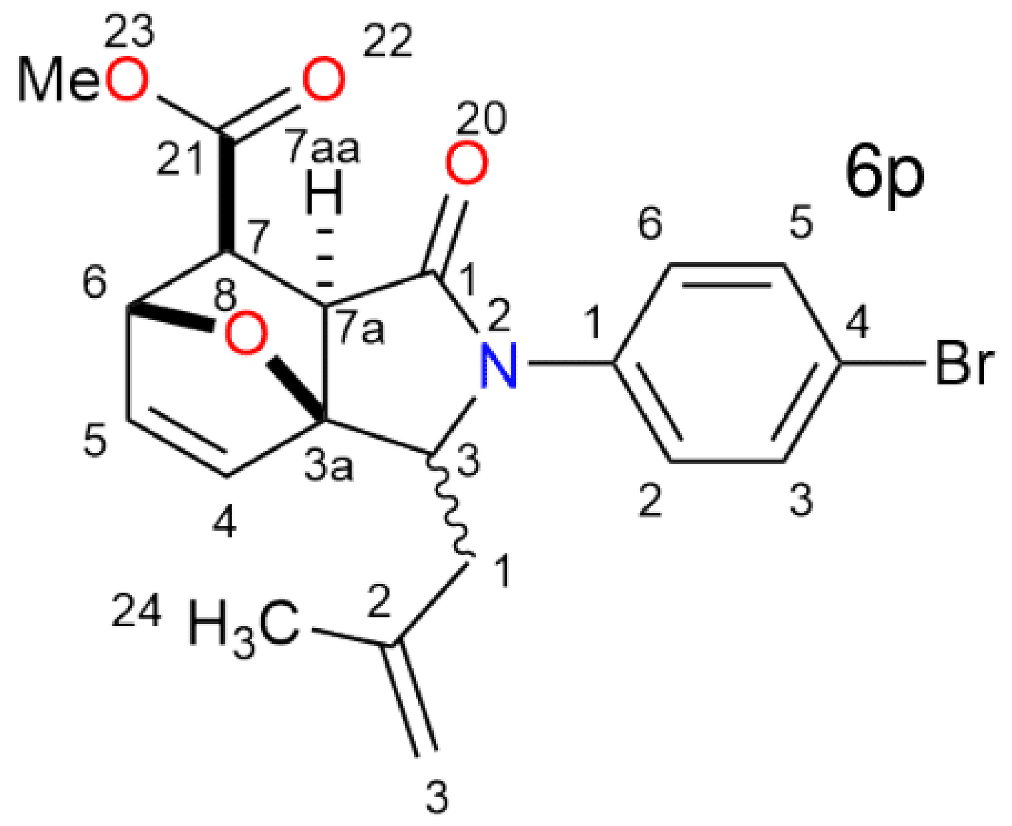

6p

FZ7650-1.jdf

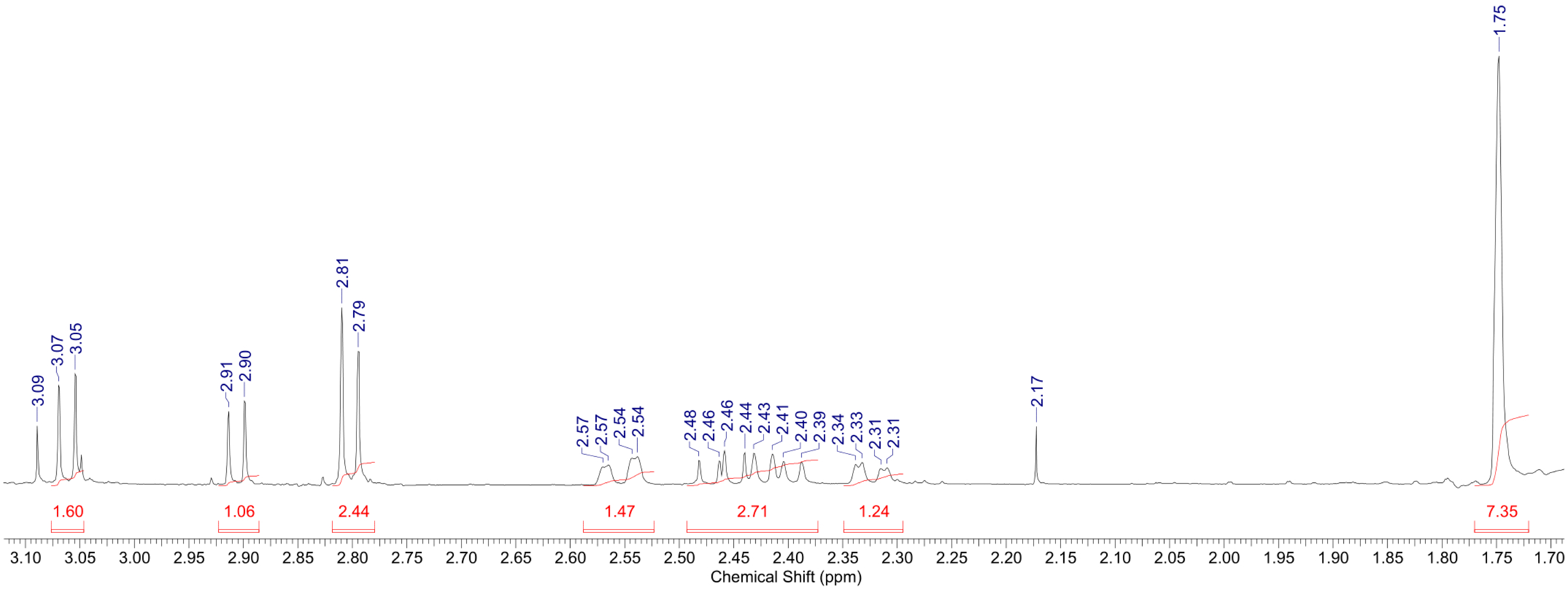

Acquisition Time (sec)	0.6921	***Comment***	single pulse decoupled gated NOE			***Date***	13 Aug 1990 07:48:00
Date Stamp	23 Oct 2019 13:51:08			***File Name***	C:\Users\Fedor\Desktop\22.10.19\FZ8026-1.jdf		
Frequency (MHz)	150.91	***Nucleus***	13C	***Number of Transients***	1000	***Origin***	ECA 600
Original Points Count	32768	***Owner***	delta	***Points Count***	32768	***Pulse Sequence***	single_pulse_dec
Receiver Gain	54.00	***Solvent***	CHLOROFORM-d			***Spectrum Offset (Hz)***	15091.3428
Sweep Width (Hz)	47348.49	***Temperature (degree C)***	24.400				

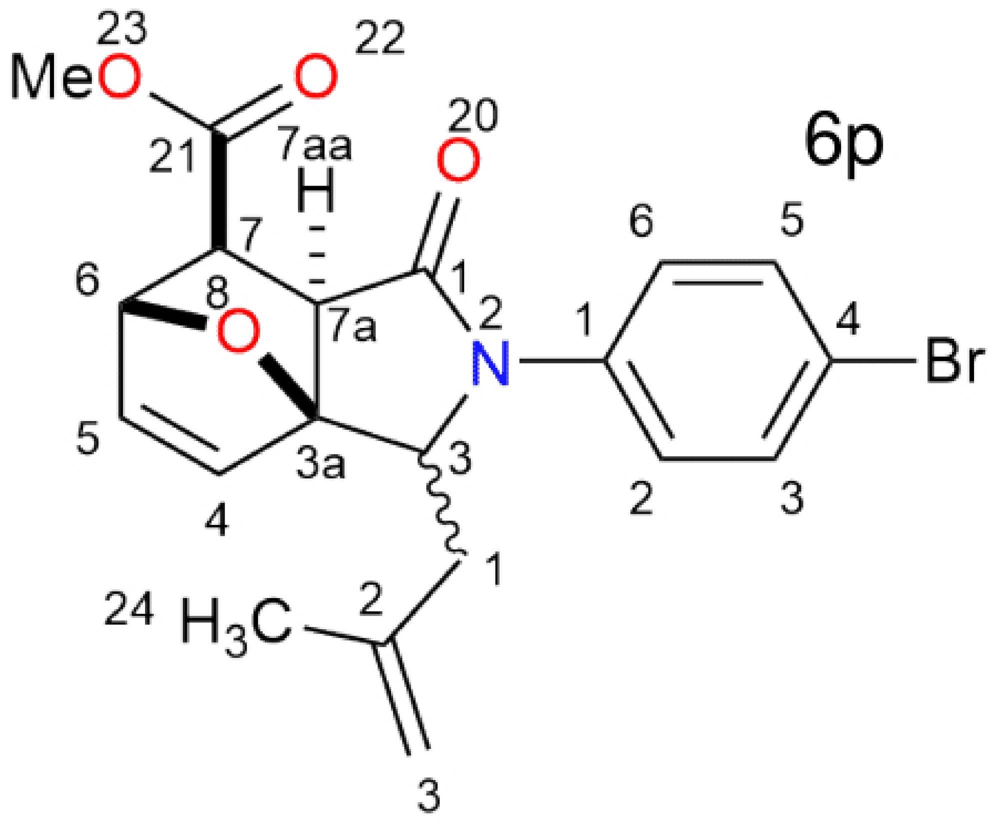

6p

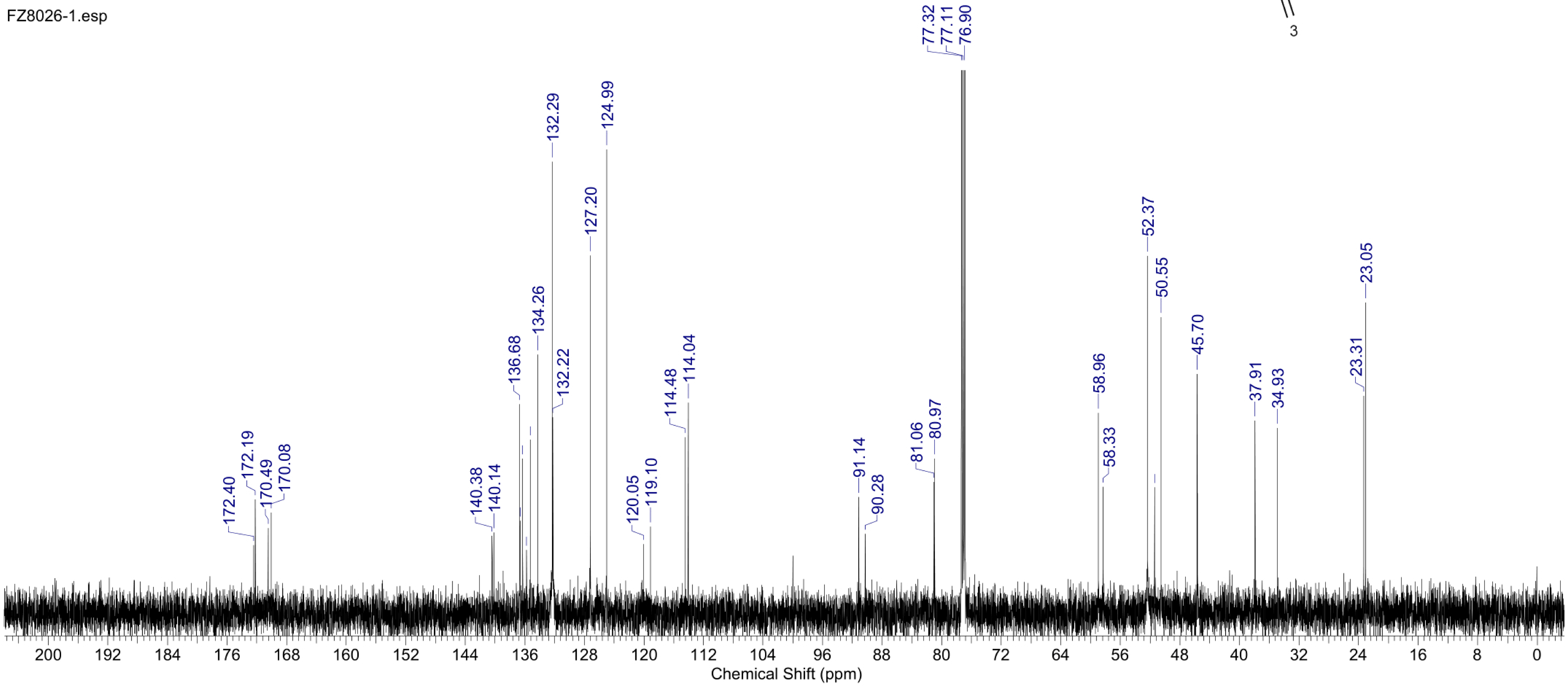

Acquisition Time (sec)	0.6921	***Comment***	single pulse decoupled gated NOE			***Date***	13 Aug 1990 07:48:00
Date Stamp	23 Oct 2019 13:51:08			***File Name***	C:\Users\Fedor\Desktop\22.10.19\FZ8026-1.jdf		
Frequency (MHz)	150.91	***Nucleus***	13C	***Number of Transients***	1000	***Origin***	ECA 600
Original Points Count	32768	***Owner***	delta	***Points Count***	32768	***Pulse Sequence***	single_pulse_dec
Receiver Gain	54.00	***Solvent***	CHLOROFORM-d			***Spectrum Offset (Hz)***	15091.3428
Sweep Width (Hz)	47348.49	***Temperature (degree C)***	24.400				

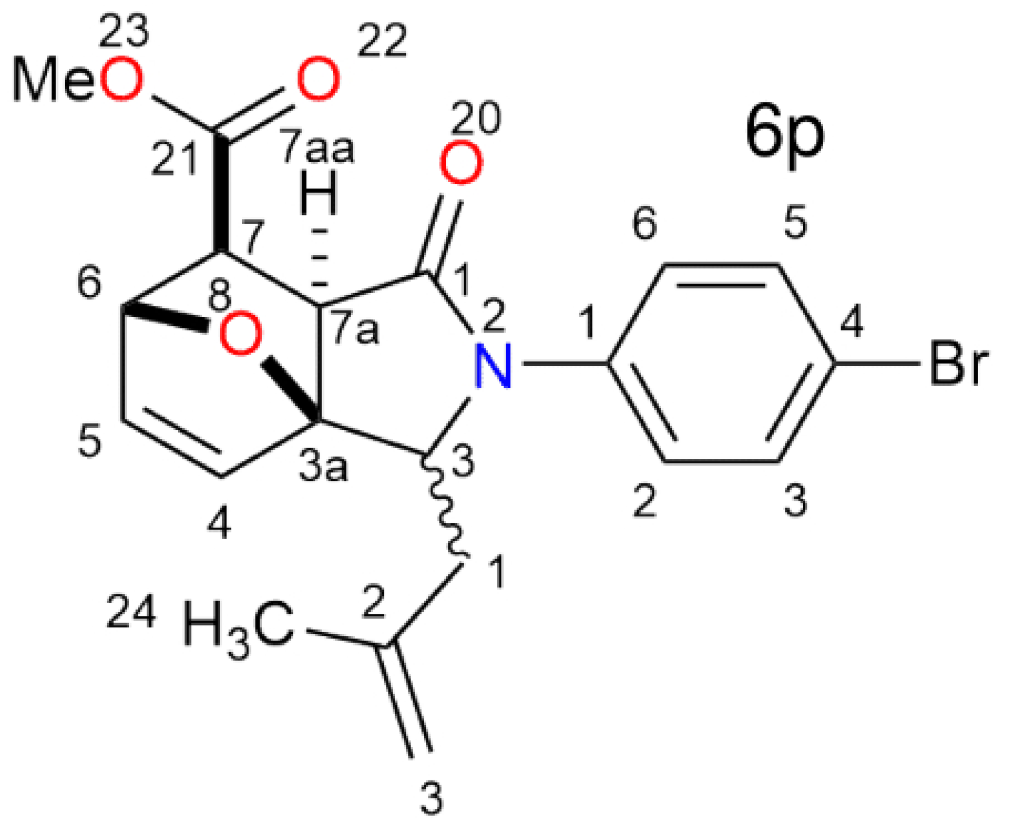

6p

FZ8026-1.esp

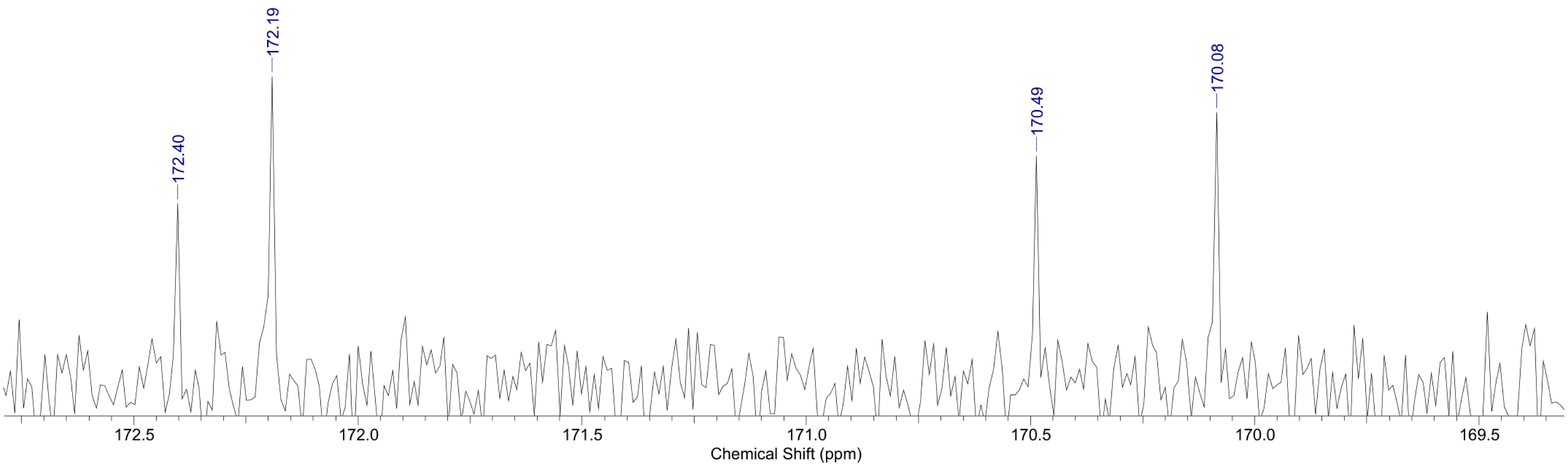

Acquisition Time (sec)	0.6921	***Comment***	single pulse decoupled gated NOE			***Date***	13 Aug 1990 07:48:00
Date Stamp	23 Oct 2019 13:51:08			***File Name***	C:\Users\Fedor\Desktop\22.10.19\FZ8026-1.jdf		
Frequency (MHz)	150.91	***Nucleus***	13C	***Number of Transients***	1000	***Origin***	ECA 600
Original Points Count	32768	***Owner***	delta	***Points Count***	32768	***Pulse Sequence***	single_pulse_dec
Receiver Gain	54.00	***Solvent***	CHLOROFORM-d			***Spectrum Offset (Hz)***	15091.3428
Sweep Width (Hz)	47348.49	***Temperature (degree C)***	24.400				

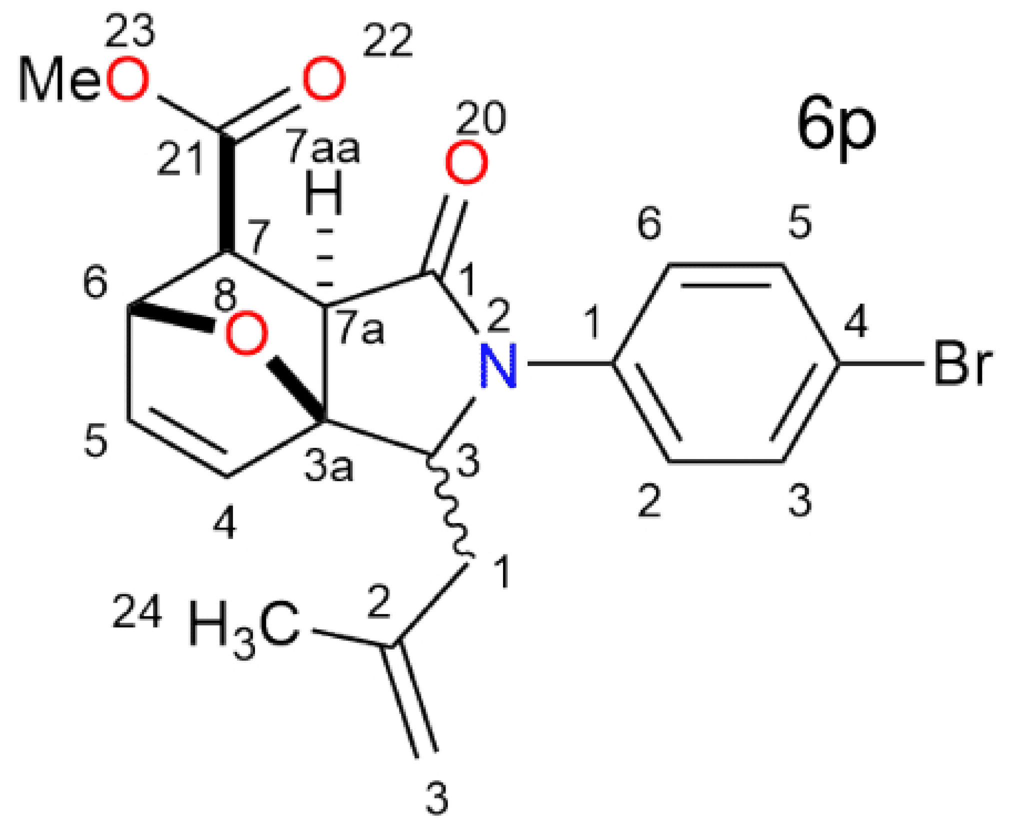

FZ8026-1.esp

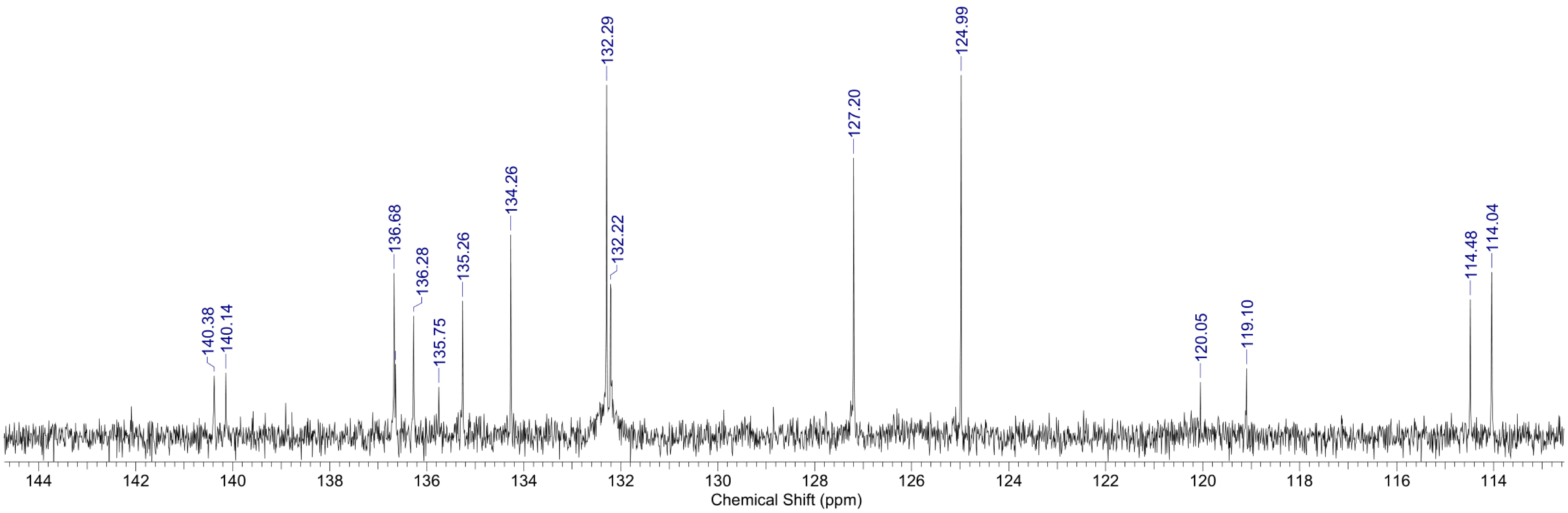

Acquisition Time (sec)	0.6921	Comment	single pulse decoupled gated NOE			Date	13 Aug 1990 07:48:00
Date Stamp	23 Oct 2019 13:51:08			File Name	C:\Users\Fedor\Desktop\22.10.19\FZ8026-1.jdf		
Frequency (MHz)	150.91	Nucleus	13C	Number of Transients	1000	Origin	ECA 600
Original Points Count	32768	Owner	delta	Points Count	32768	Pulse Sequence	single_pulse_dec
Receiver Gain	54.00	Solvent	CHLOROFORM-d			Spectrum Offset (Hz)	15091.3428
Sweep Width (Hz)	47348.49	Temperature (degree C)	24.400				

6p

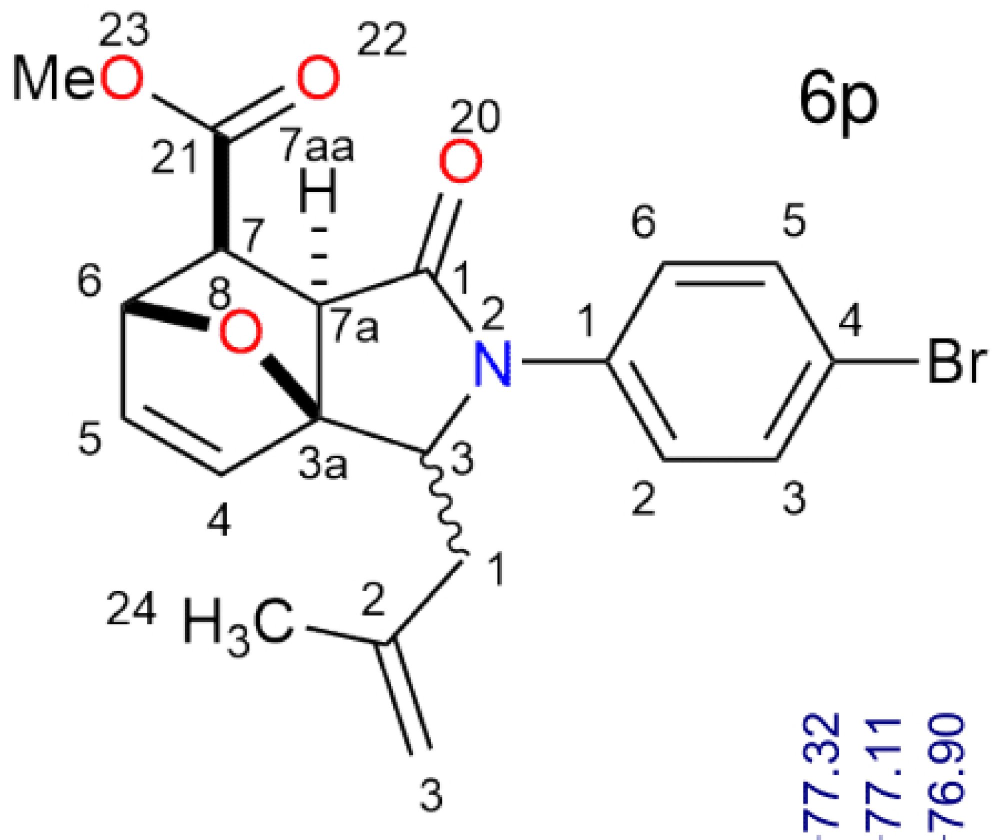

FZ8026-1.esp

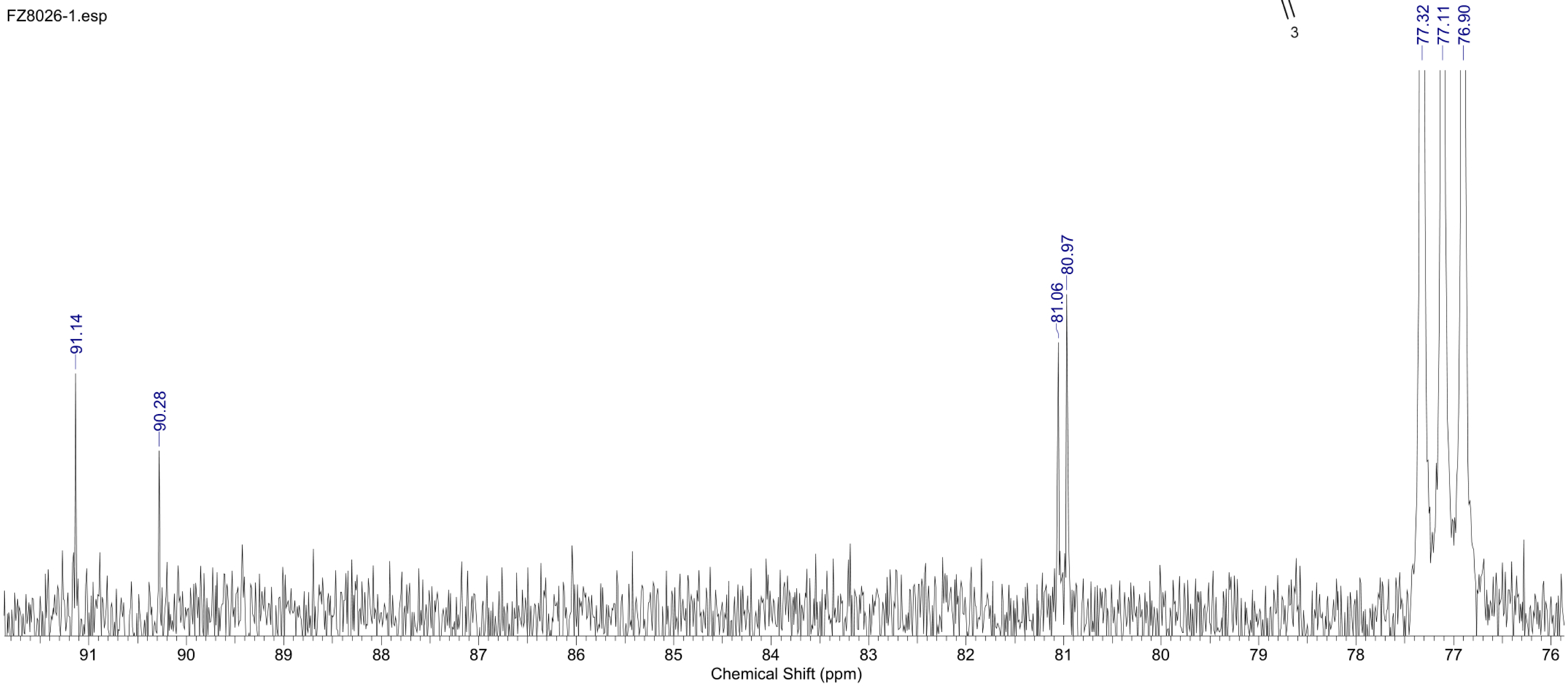

Acquisition Time (sec)	0.6921	***Comment***	single pulse decoupled gated NOE			***Date***	13 Aug 1990 07:48:00
Date Stamp	23 Oct 2019 13:51:08			***File Name***	C:\Users\Fedor\Desktop\22.10.19\FZ8026-1.jdf		
Frequency (MHz)	150.91	***Nucleus***	13C	***Number of Transients***	1000	***Origin***	ECA 600
Original Points Count	32768	***Owner***	delta	***Points Count***	32768	***Pulse Sequence***	single_pulse_dec
Receiver Gain	54.00	***Solvent***	CHLOROFORM-d			***Spectrum Offset (Hz)***	15091.3428
Sweep Width (Hz)	47348.49	***Temperature (degree C)***	24.400				

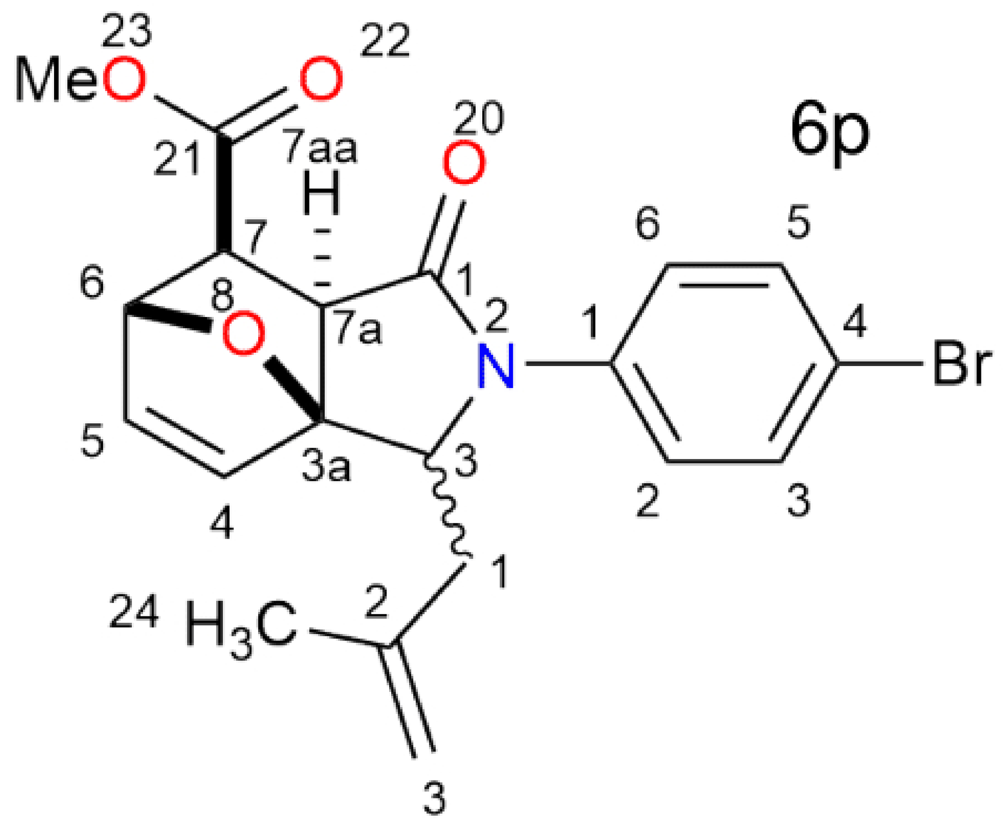

6p

FZ8026-1.esp

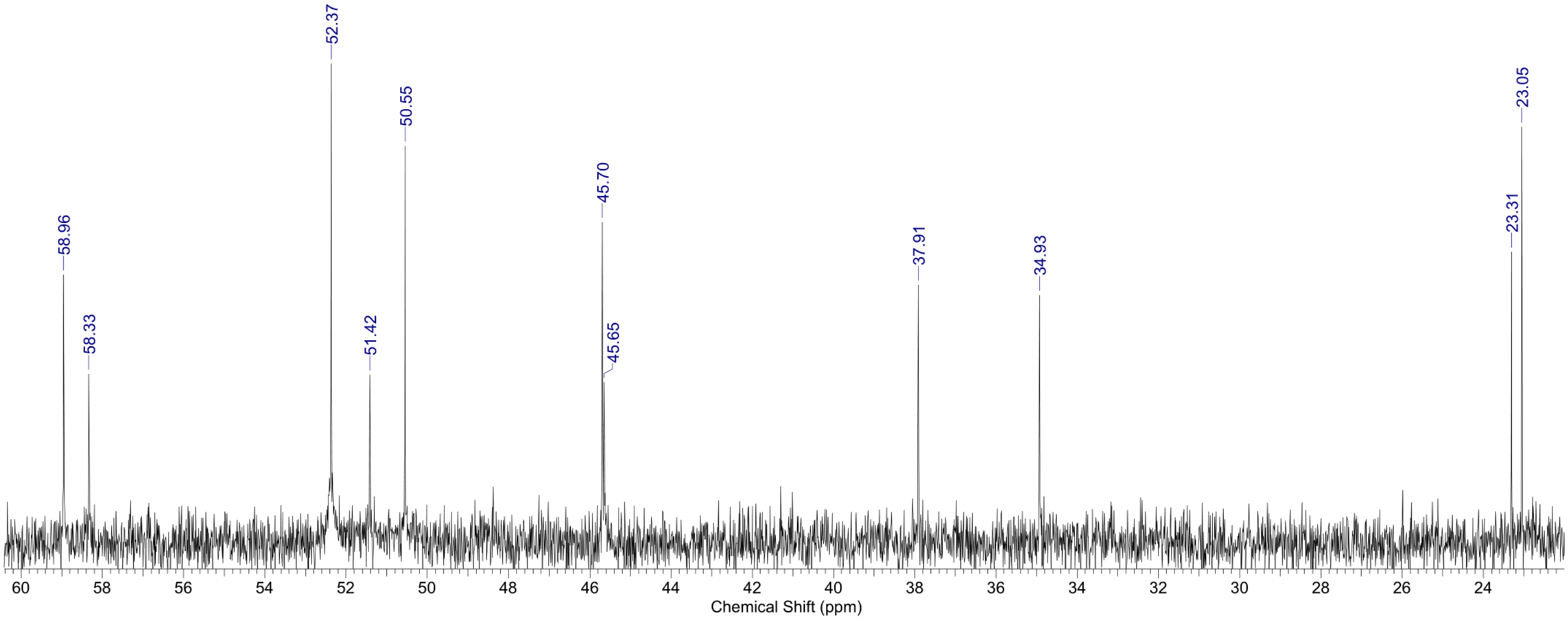

DEPT-135 NMR spectrum for compound 6p

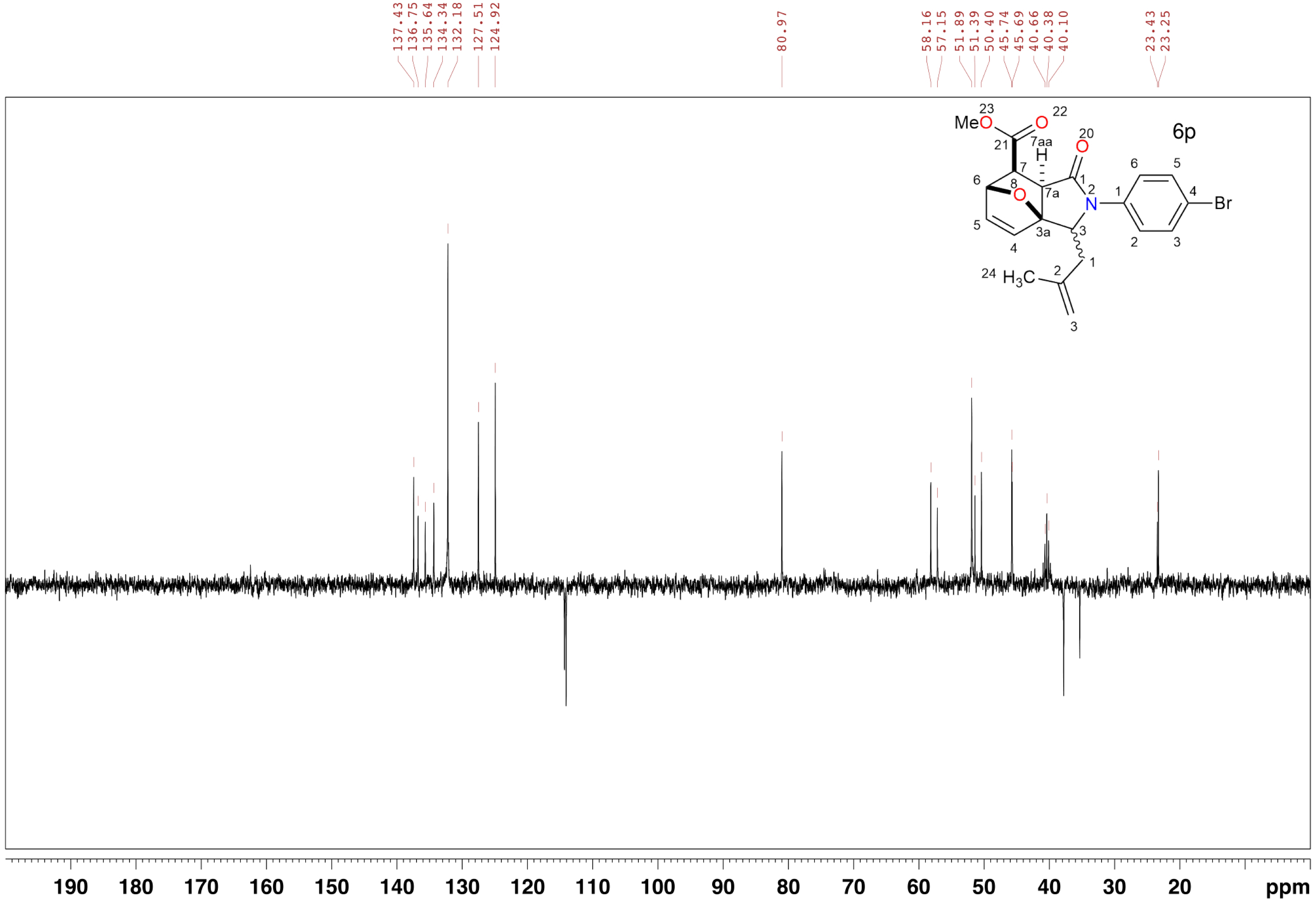

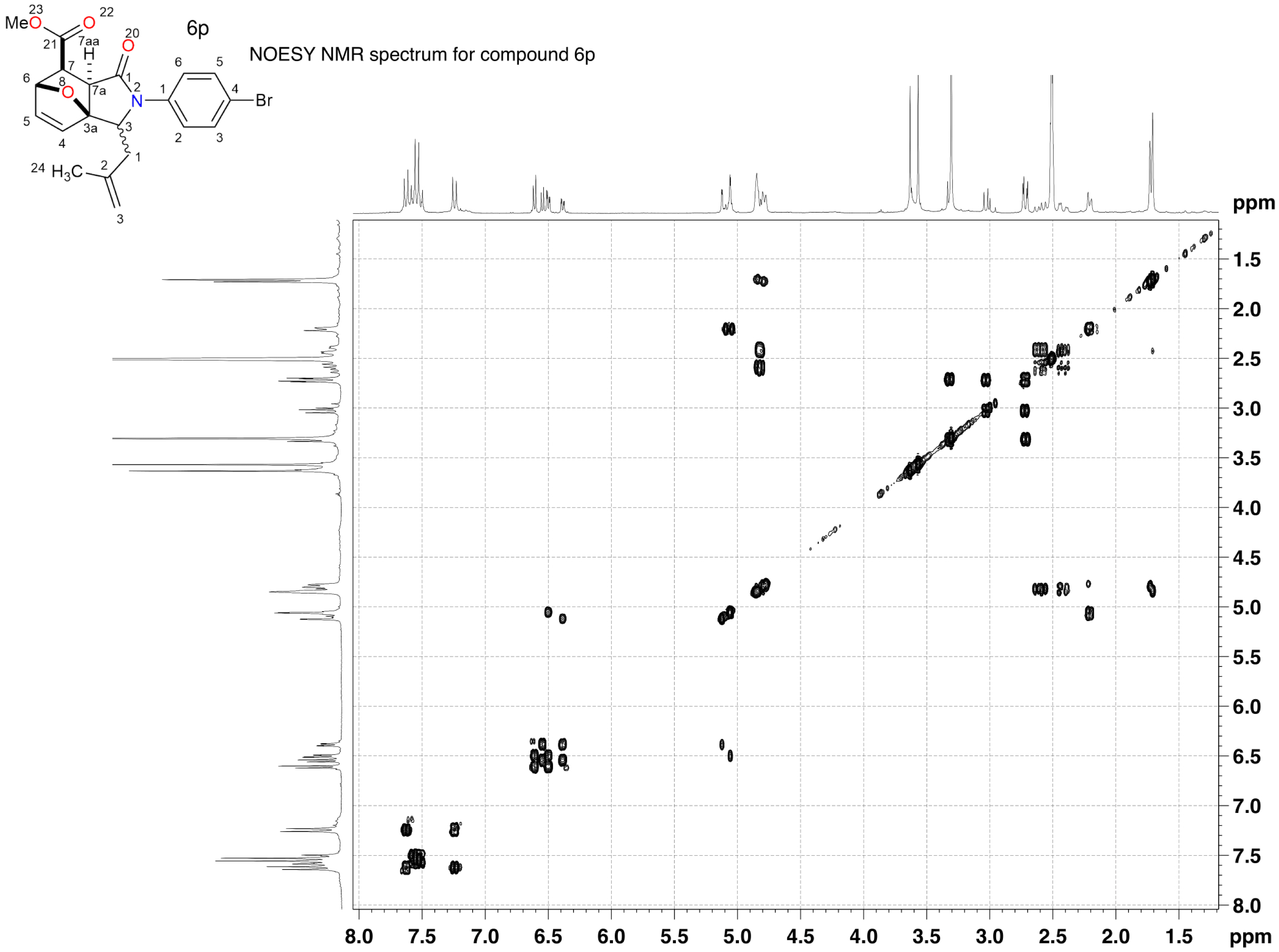

NOESY NMR spectrum for compound 6p

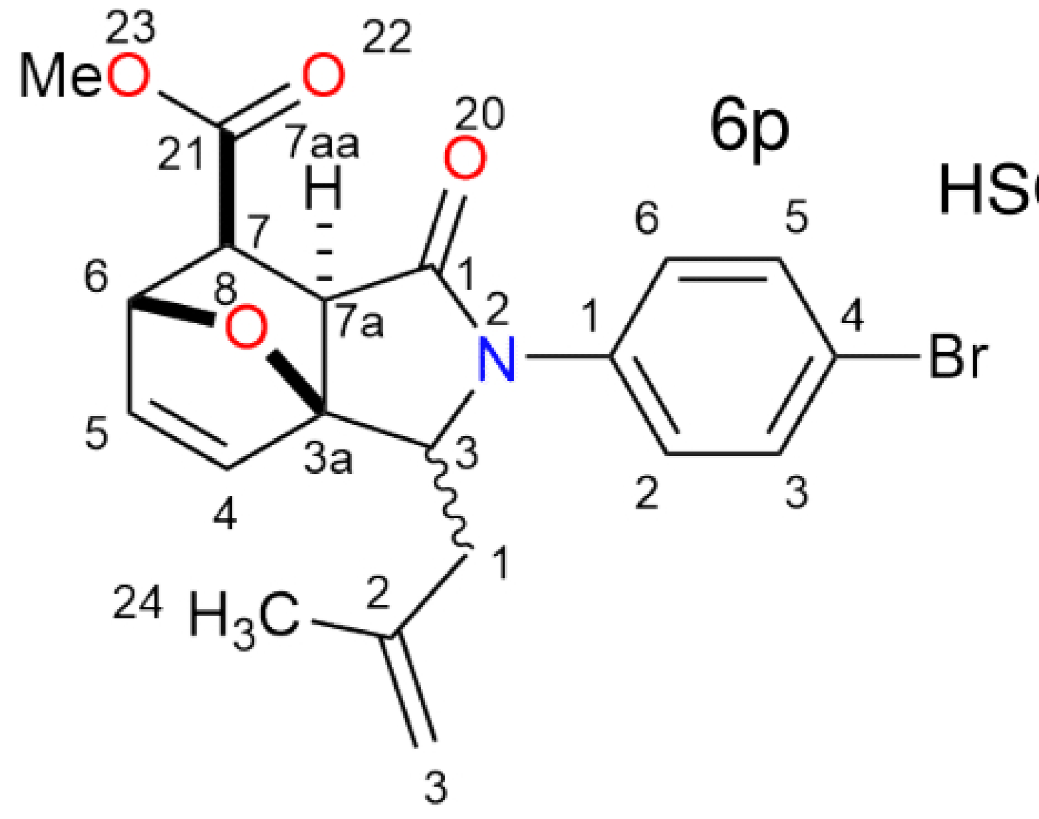

HSQC spectrum for compound 6p

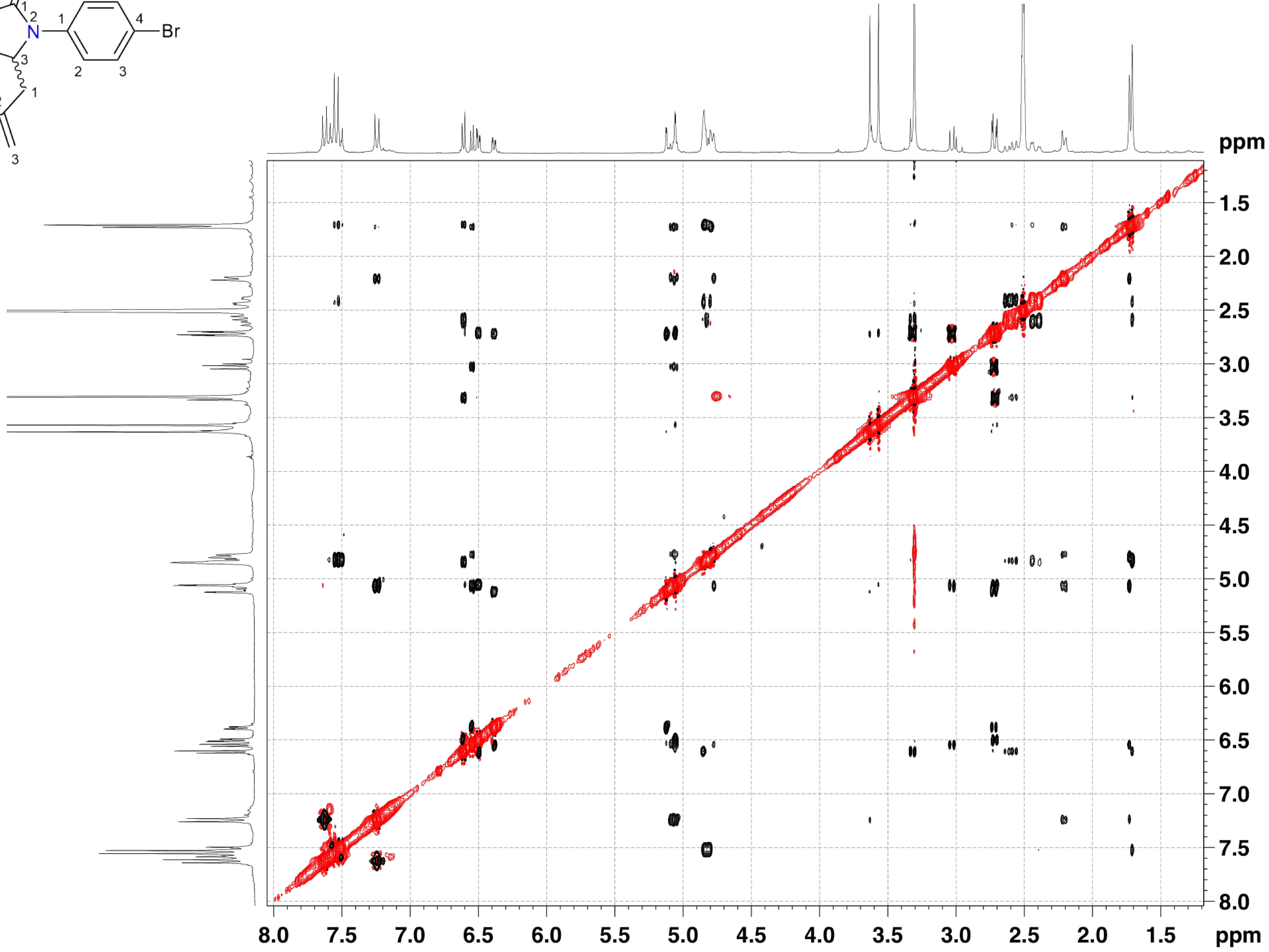

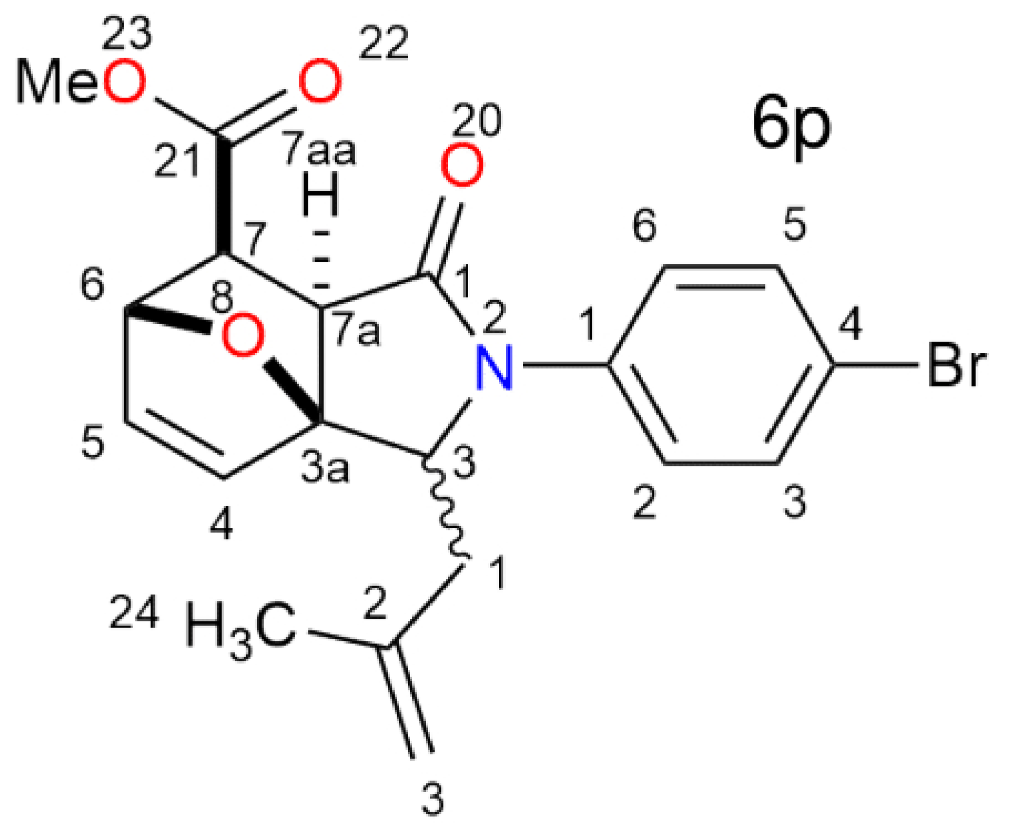

MeO
O
O
N
Br
O
H
H3C
6p

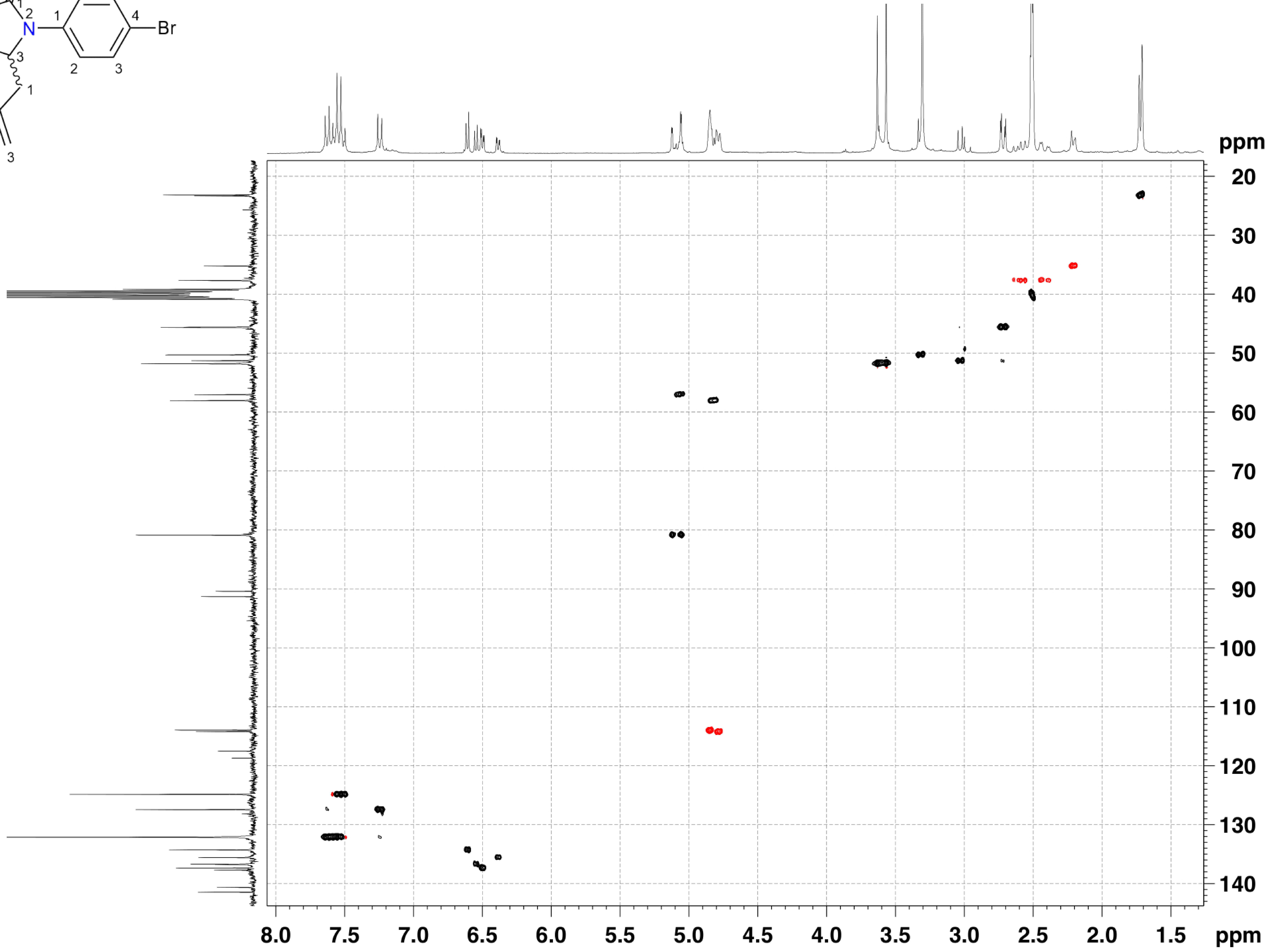

ppm
20
30
40
50
60
70
80
90
100
110
120
130
140
8.0
7.5
7.0
6.5
6.0
5.5
5.0
4.5
4.0
3.5
3.0
2.5
2.0
1.5
ppm

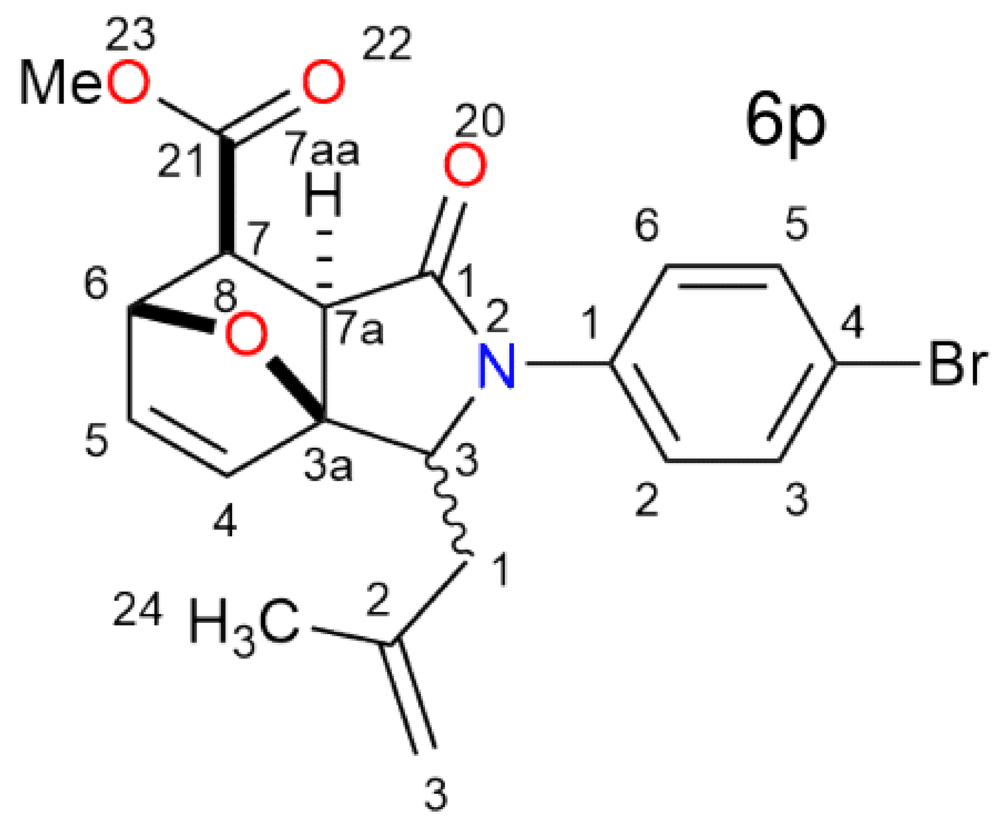
MeO
O
21
7aa
H
O
1
2
N
4
Br
6
7
8
O
7a
5
3a
3
4
24 H3C
2
1
3
23
22
20
5
6
1
2
3

6p

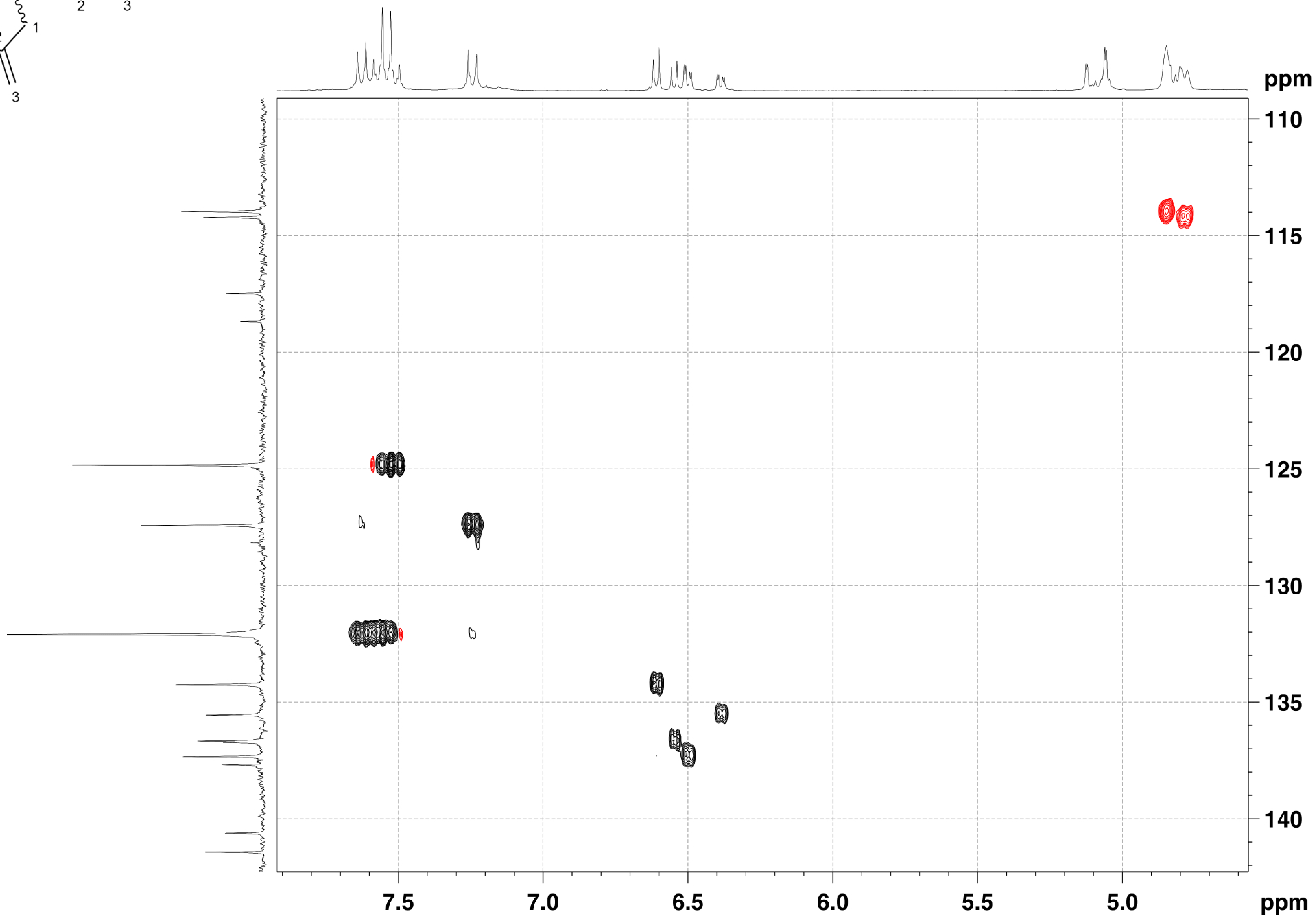
ppm
110
115
120
125
130
135
140
7.5
7.0
6.5
6.0
5.5
5.0
ppm

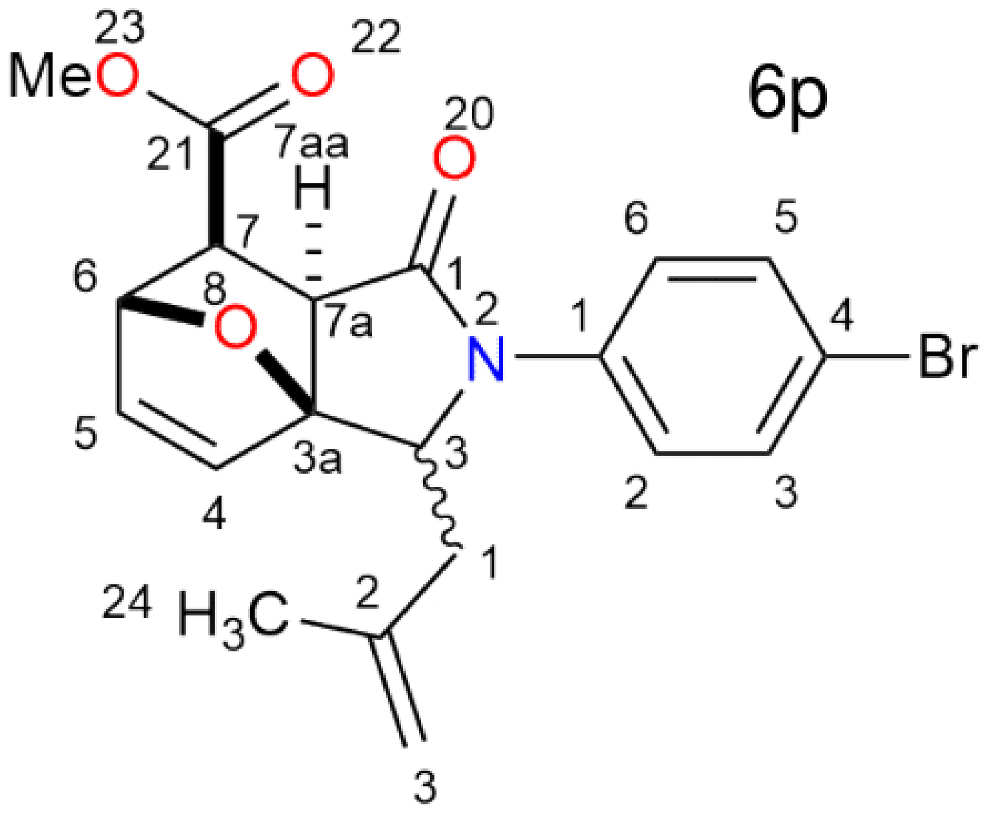

6p

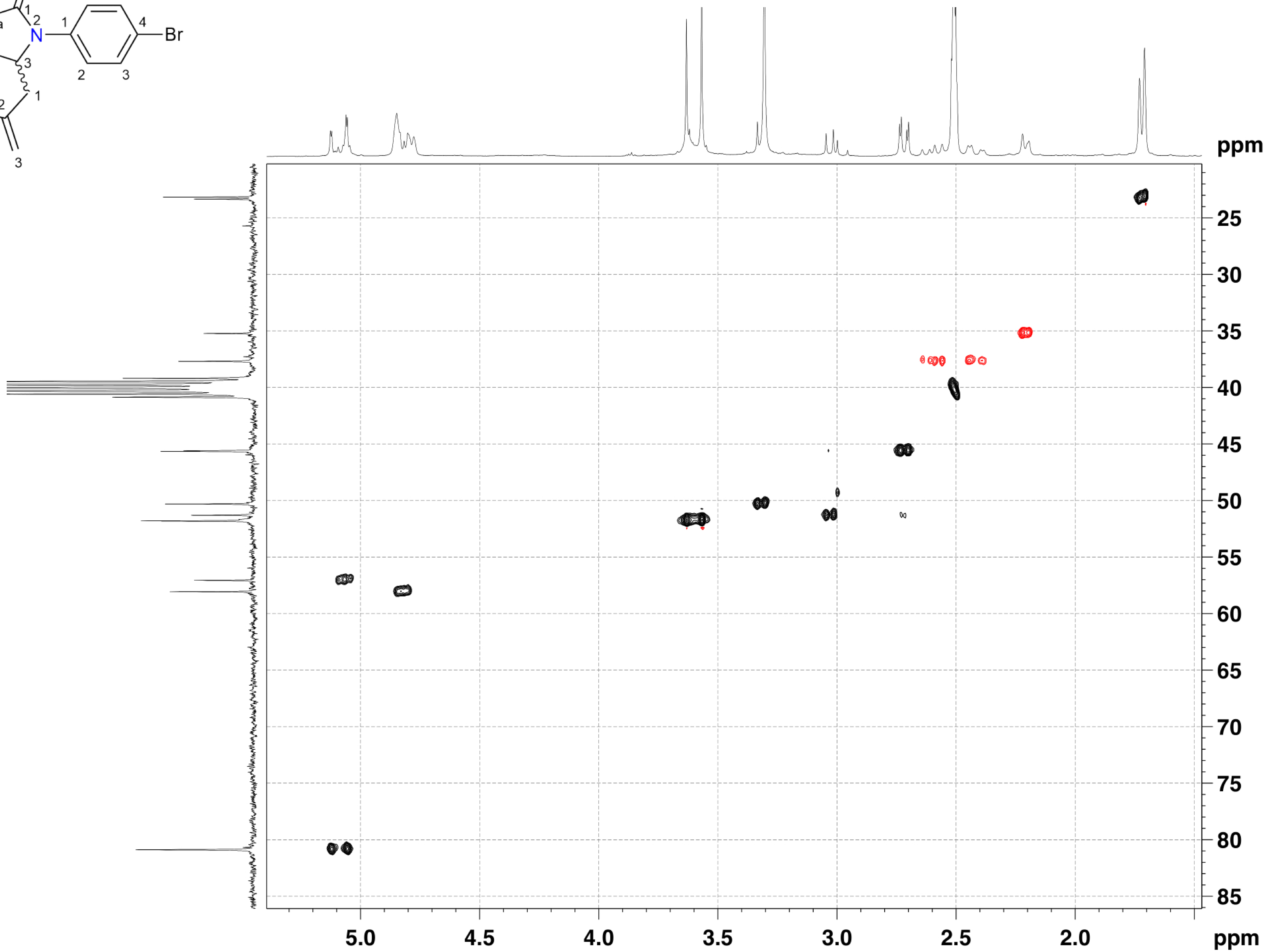

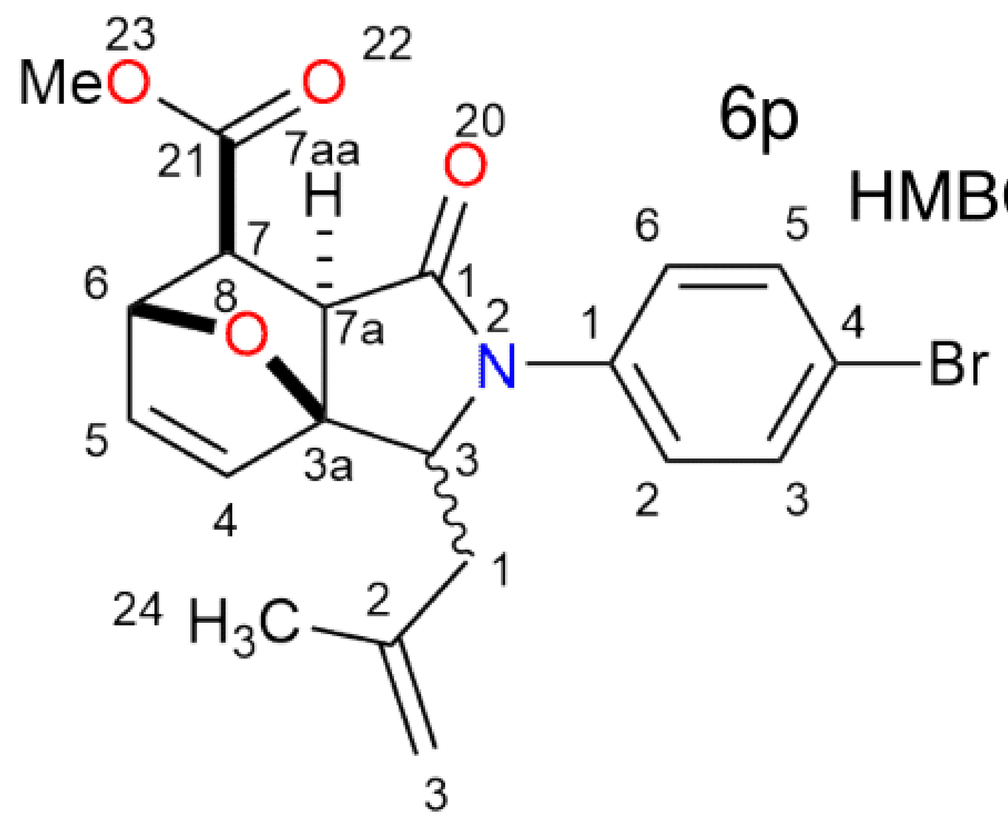

6p

HMBC NMR spectrum for compound 6p

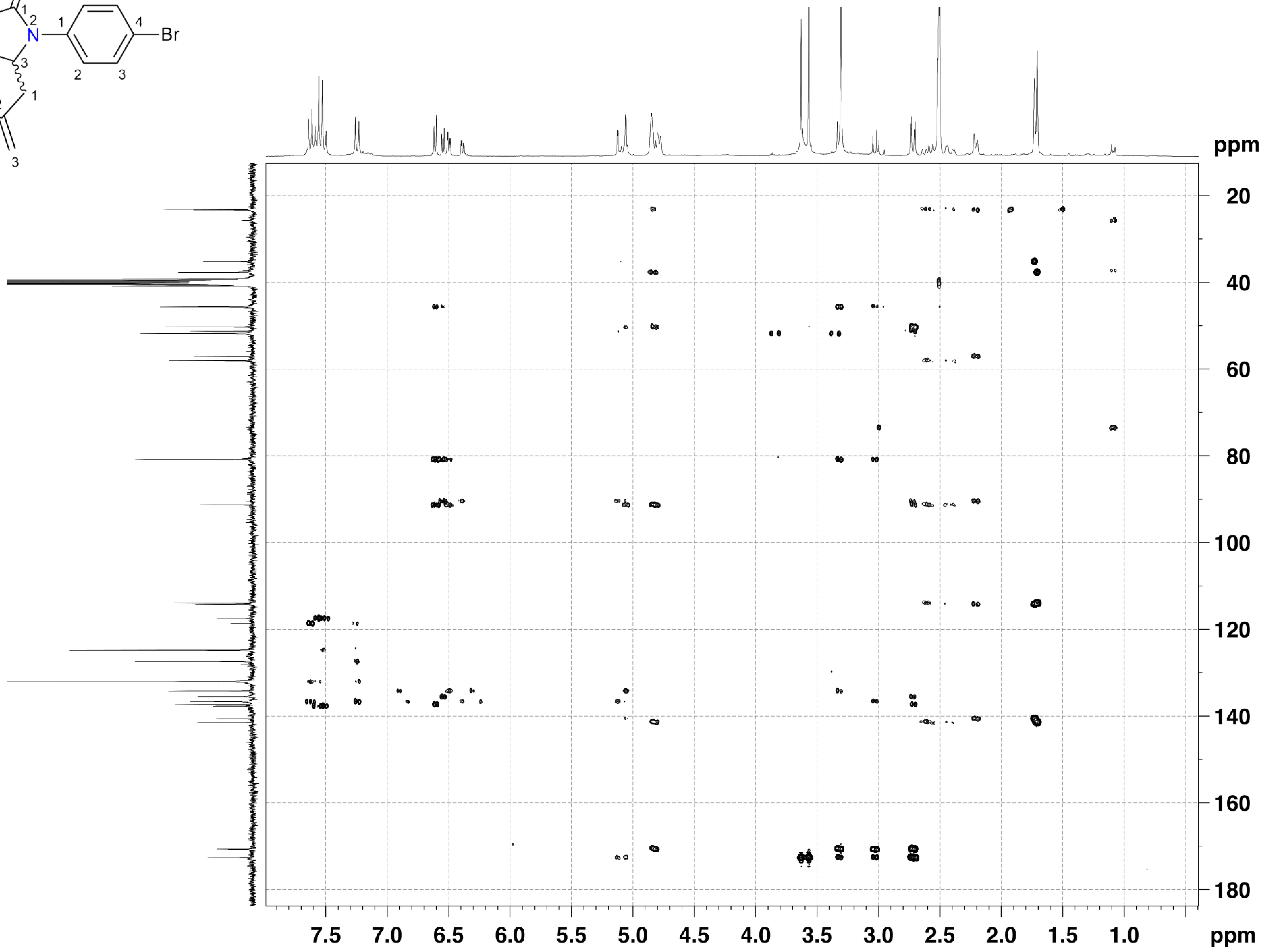

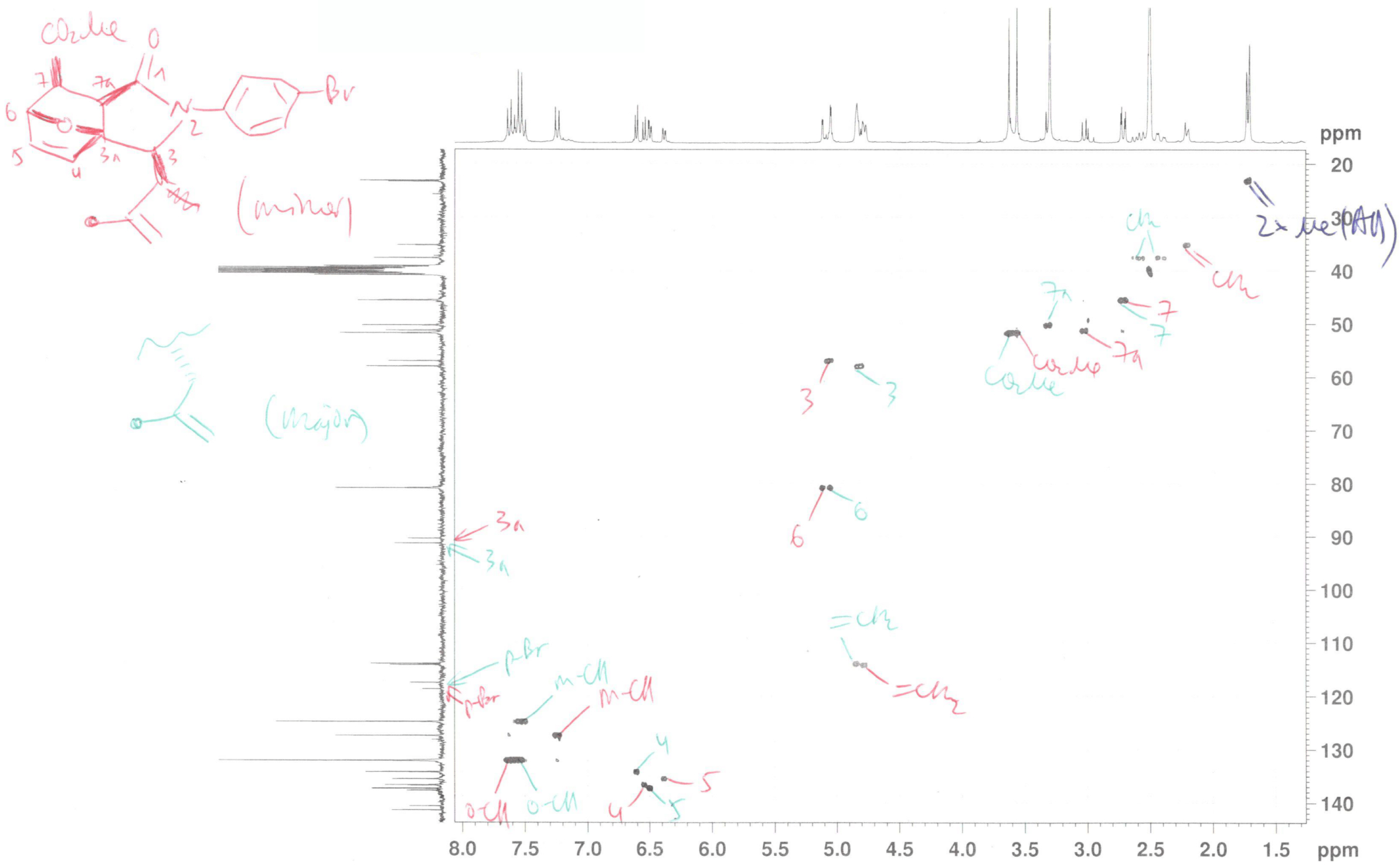
ppm
20
30
40
50
60
70
80
90
100
110
120
130
140
8.0
7.5
7.0
6.5
6.0
5.5
5.0
4.5
4.0
3.5
3.0
2.5
2.0
1.5
ppm
(minor)
(major)
Br
CO2Me
3a
p-Br
m-CH
o-CH
=CH2
7a
CH2

NMR spectrum data for compound 6Aa

**Formula**	$C_{19}H_{19}NO_4$	**FW**	325.3585

**Acquisition Time (sec)**	1.9818	**Comment**	single_pulse	**Date**	25 Aug 2020 08:54:13		
**Date Stamp**	25 Aug 2020 08:55:28			**File Name**	C:\USERS\лаба 534\DOWNLOADS\FZ9038-1.JDF		
**Frequency (MHz)**	600.17	**Nucleus**	1H	**Number of Transients**	8	**Origin**	ECA 600
**Original Points Count**	32768	**Owner**	CKP	**Points Count**	32768	**Pulse Sequence**	single_pulse.ex2
**Receiver Gain**	40.00	**Solvent**	CHLOROFORM-d			**Spectrum Offset (Hz)**	5412.6455
**Sweep Width (Hz)**	16534.39						

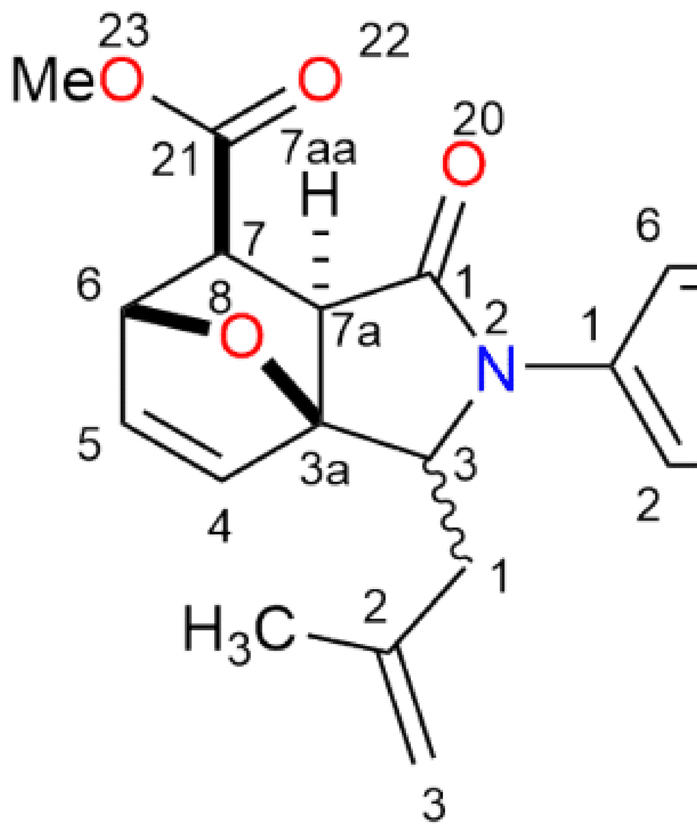

6Aa

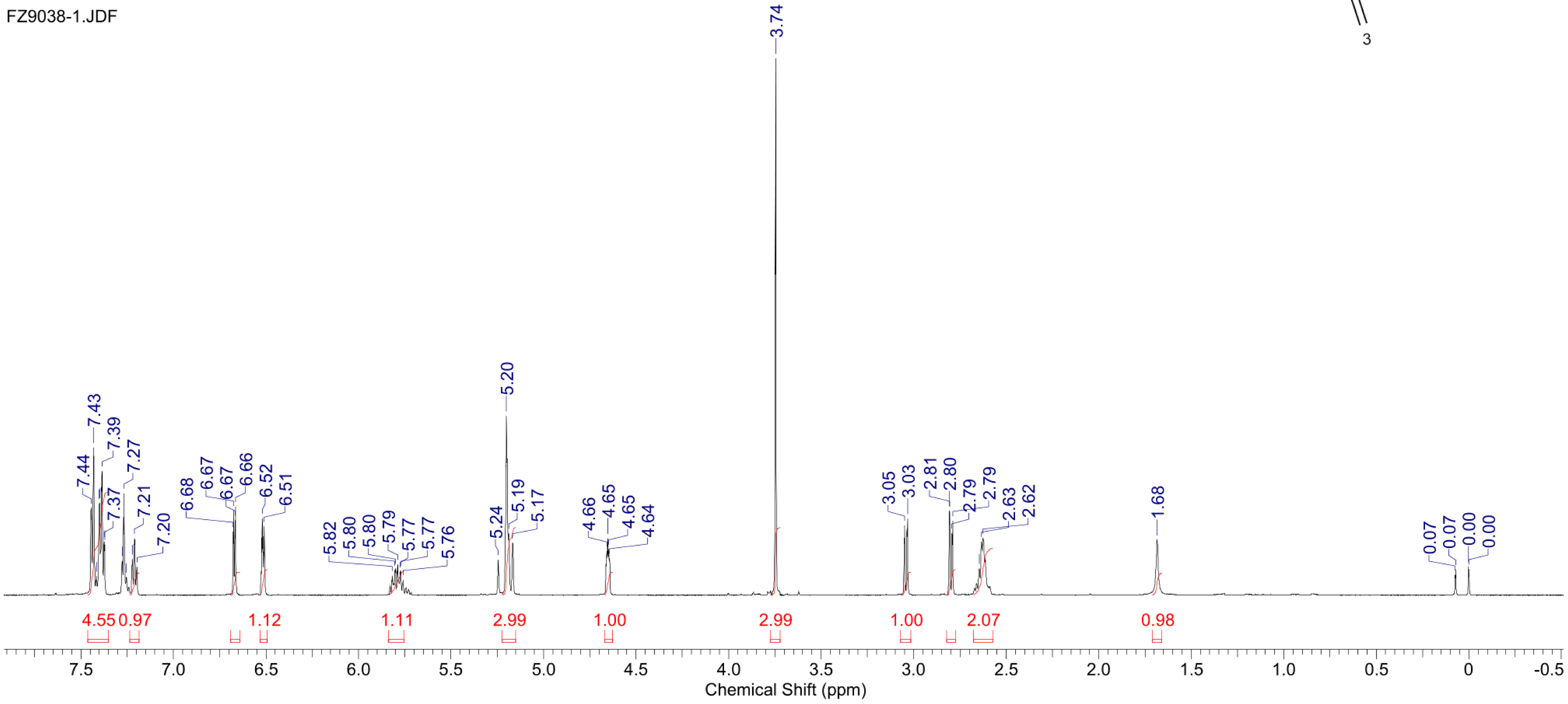

**Formula** $C_{19}H_{19}NO_4$	**FW** 325.3585

Acquisition Time (sec)	1.9818	***Comment***	single_pulse	***Date***	25 Aug 2020 08:54:13		
Date Stamp	25 Aug 2020 08:55:28			***File Name***	C:\USERS\лаба 534\DOWNLOADS\FZ9038-1.JDF		
Frequency (MHz)	600.17	***Nucleus***	1H	***Number of Transients***	8	***Origin***	ECA 600
Original Points Count	32768	***Owner***	CKP	***Points Count***	32768	***Pulse Sequence***	single_pulse.ex2
Receiver Gain	40.00	***Solvent***	CHLOROFORM-d			***Spectrum Offset (Hz)***	5412.6455
Sweep Width (Hz)	16534.39						

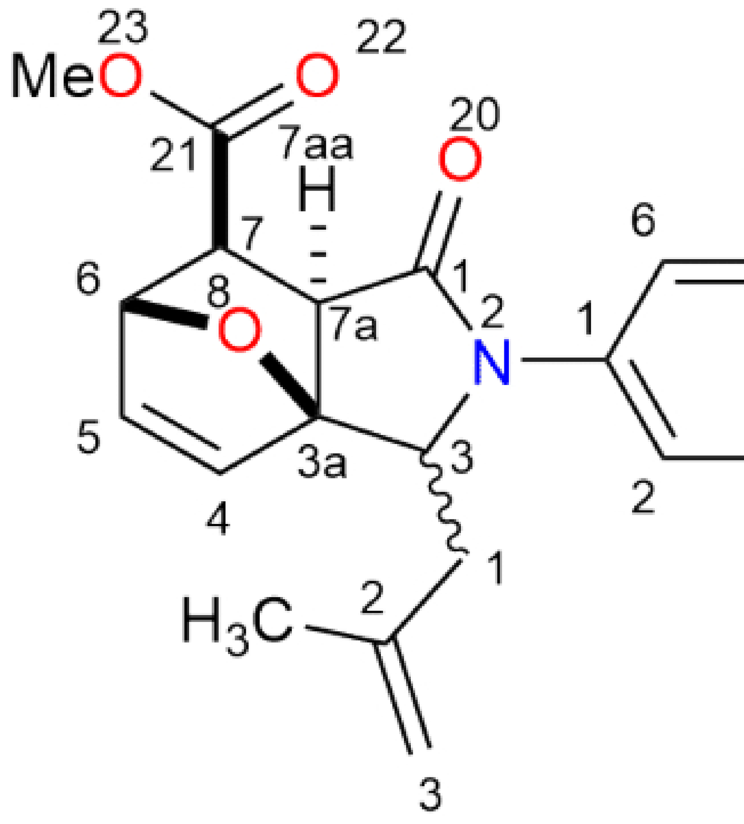

6Aa

FZ9038-1.JDF

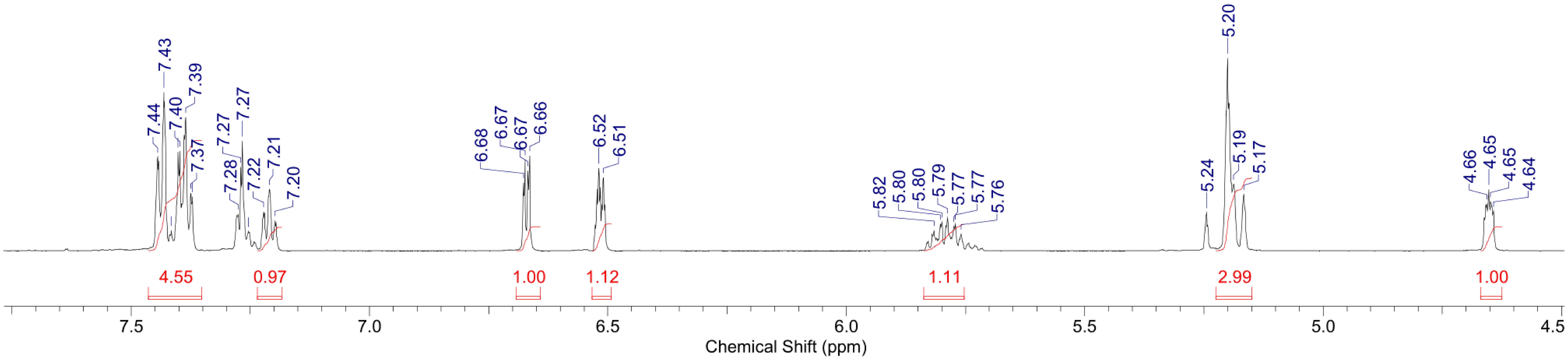

Formula $C_{19}H_{19}NO_4$	FW 325.3585

**Acquisition Time (sec)**	1.9818	**Comment**	single_pulse	**Date**	25 Aug 2020 08:54:13		
**Date Stamp**	25 Aug 2020 08:55:28			**File Name**	C:\USERS\лаба 534\DOWNLOADS\FZ9038-1.JDF		
**Frequency (MHz)**	600.17	**Nucleus**	1H	**Number of Transients**	8	**Origin**	ECA 600
**Original Points Count**	32768	**Owner**	CKP	**Points Count**	32768	**Pulse Sequence**	single_pulse.ex2
**Receiver Gain**	40.00	**Solvent**	CHLOROFORM-d			**Spectrum Offset (Hz)**	5412.6455
**Sweep Width (Hz)**	16534.39						

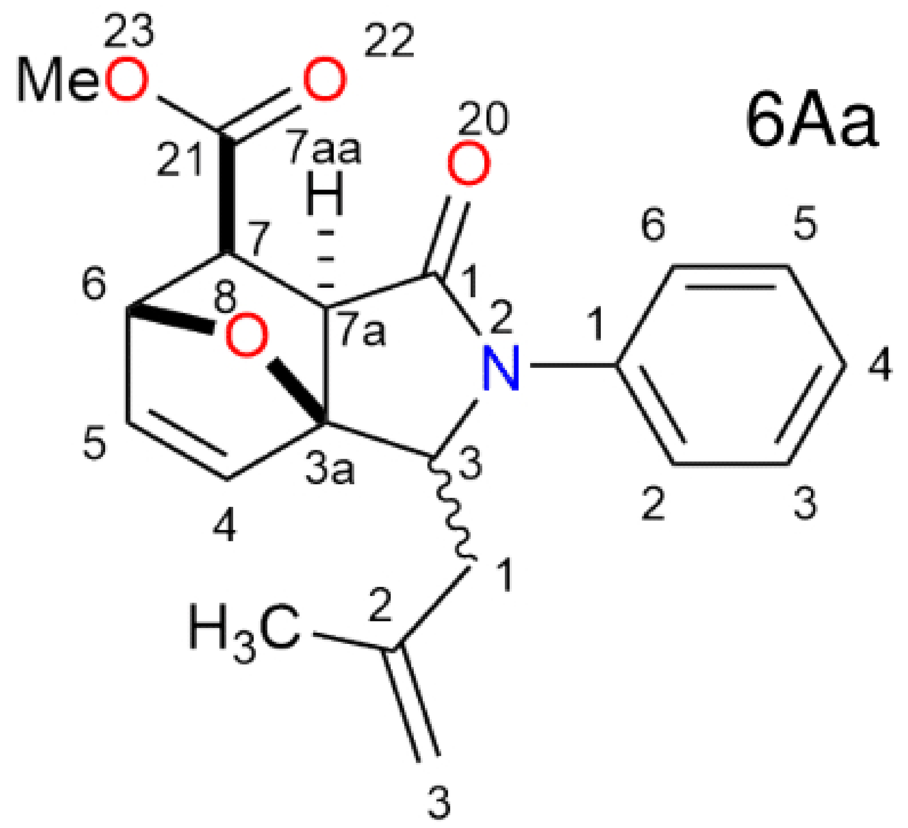

6Aa

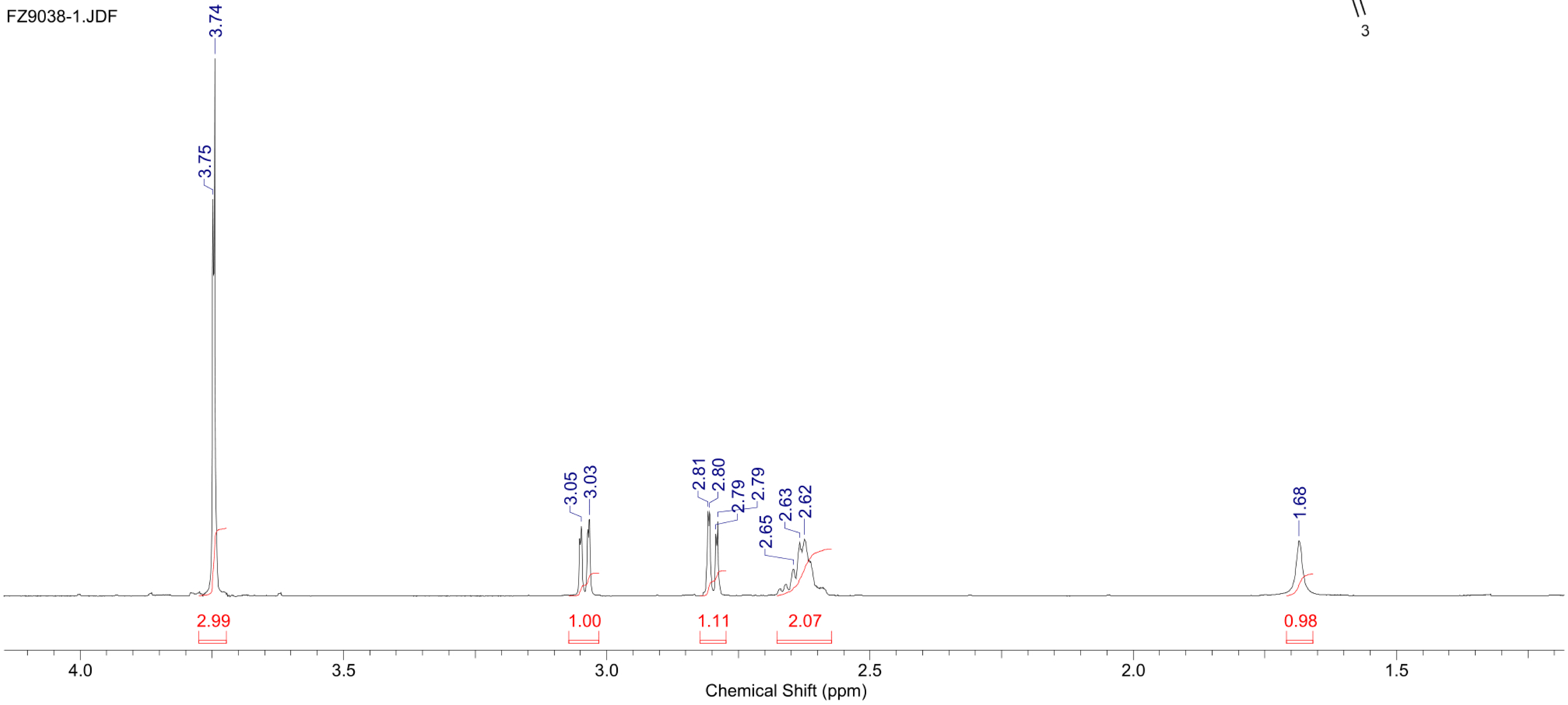

NMR spectrum data for compound 6Ba

Acquisition Time (sec)	1.9818	***Comment***	single_pulse	***Date***	13 May 1990 05:13:25		
Date Stamp	23 Jul 2019 11:16:58			***File Name***	C:\Users\Fedor\Desktop\22.07.19\FZ7586-1.jdf	***Frequency (MHz)***	600.17
Nucleus	1H	***Number of Transients***	8	***Origin***	ECA 600	***Original Points Count*** 32768	***Owner*** delta
Points Count	32768	***Pulse Sequence***	single_pulse.ex2			***Receiver Gain*** 46.00	***Solvent*** CHLOROFORM-d
Spectrum Offset (Hz)	5409.6182	***Sweep Width (Hz)***	16534.39	***Temperature (degree C)***	22.900		

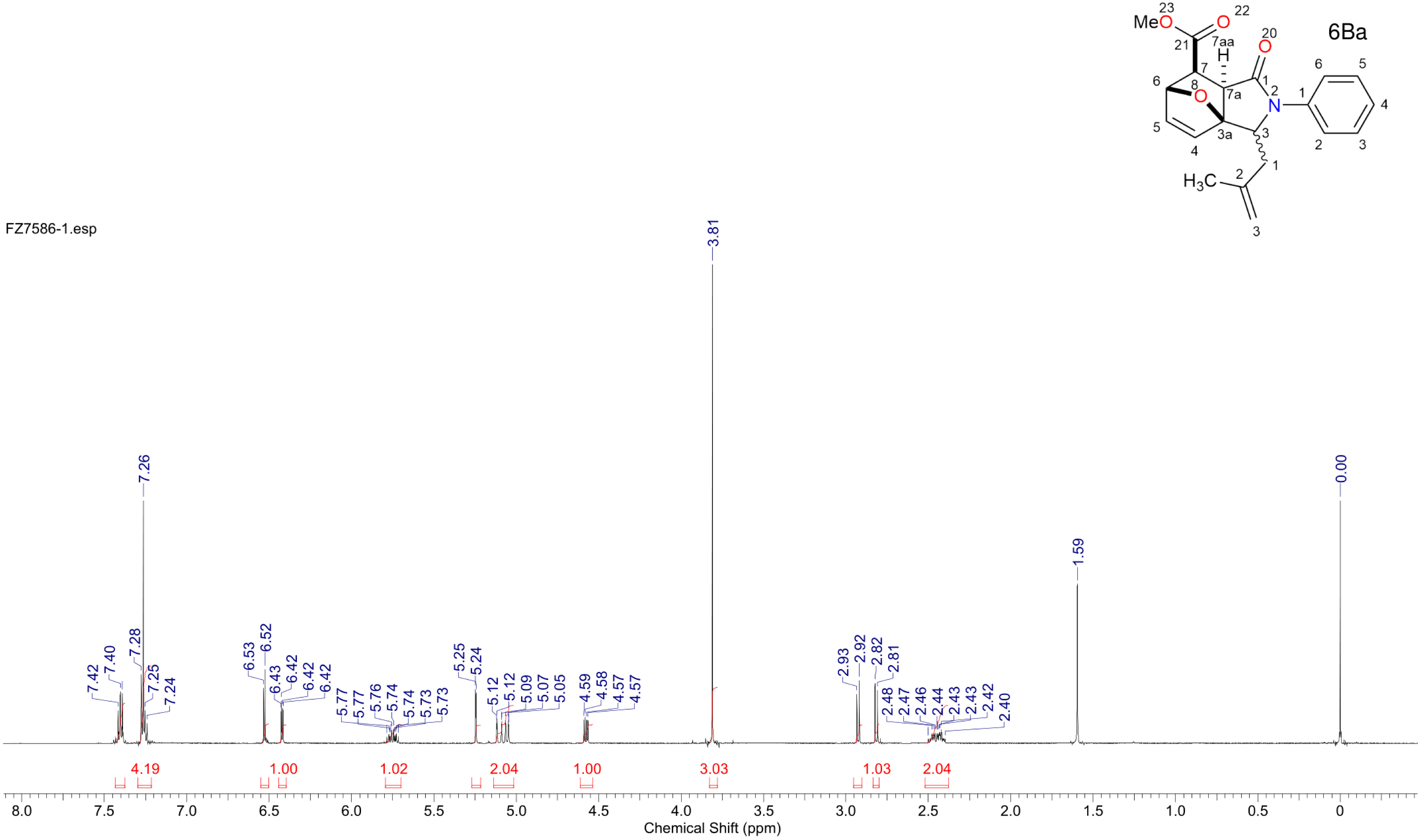

Acquisition Time (sec)	1.9818	***Comment***	single_pulse	***Date***	13 May 1990 05:13:25		
Date Stamp	23 Jul 2019 11:16:58			***File Name***	C:\Users\Fedor\Desktop\22.07.19\FZ7586-1.jdf	***Frequency (MHz)***	600.17
Nucleus	1H	***Number of Transients***	8	***Origin***	ECA 600	***Original Points Count***	32768
Owner	delta						
Points Count	32768	***Pulse Sequence***	single_pulse.ex2	***Receiver Gain***	46.00	***Solvent***	CHLOROFORM-d
Spectrum Offset (Hz)	5409.6182	***Sweep Width (Hz)***	16534.39	***Temperature (degree C)***	22.900		

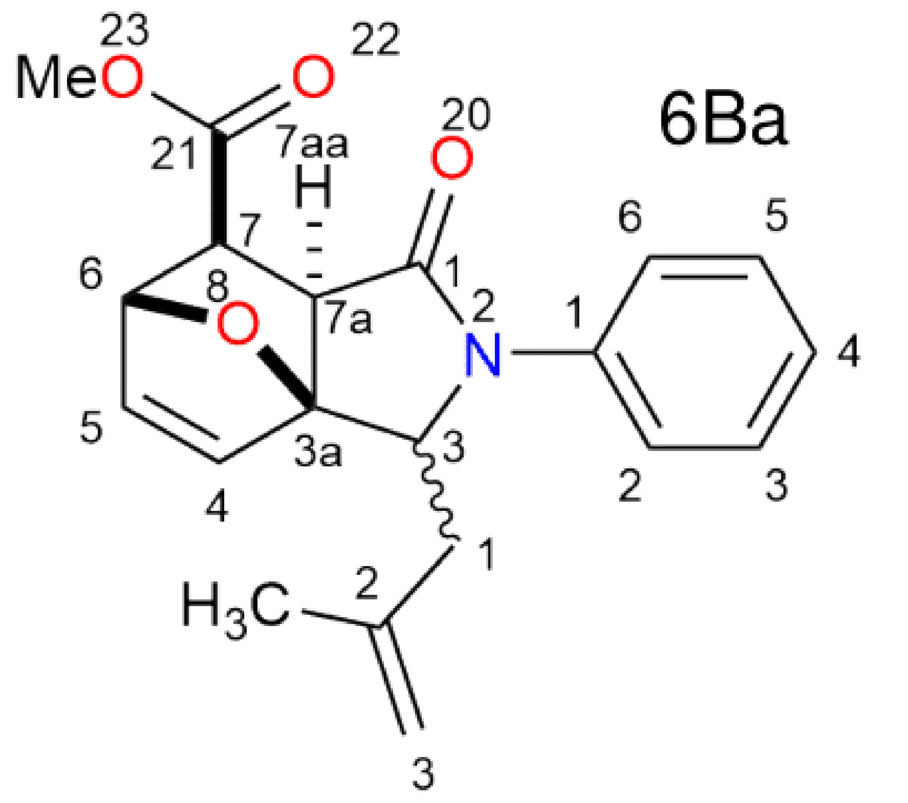

FZ7586-1.esp

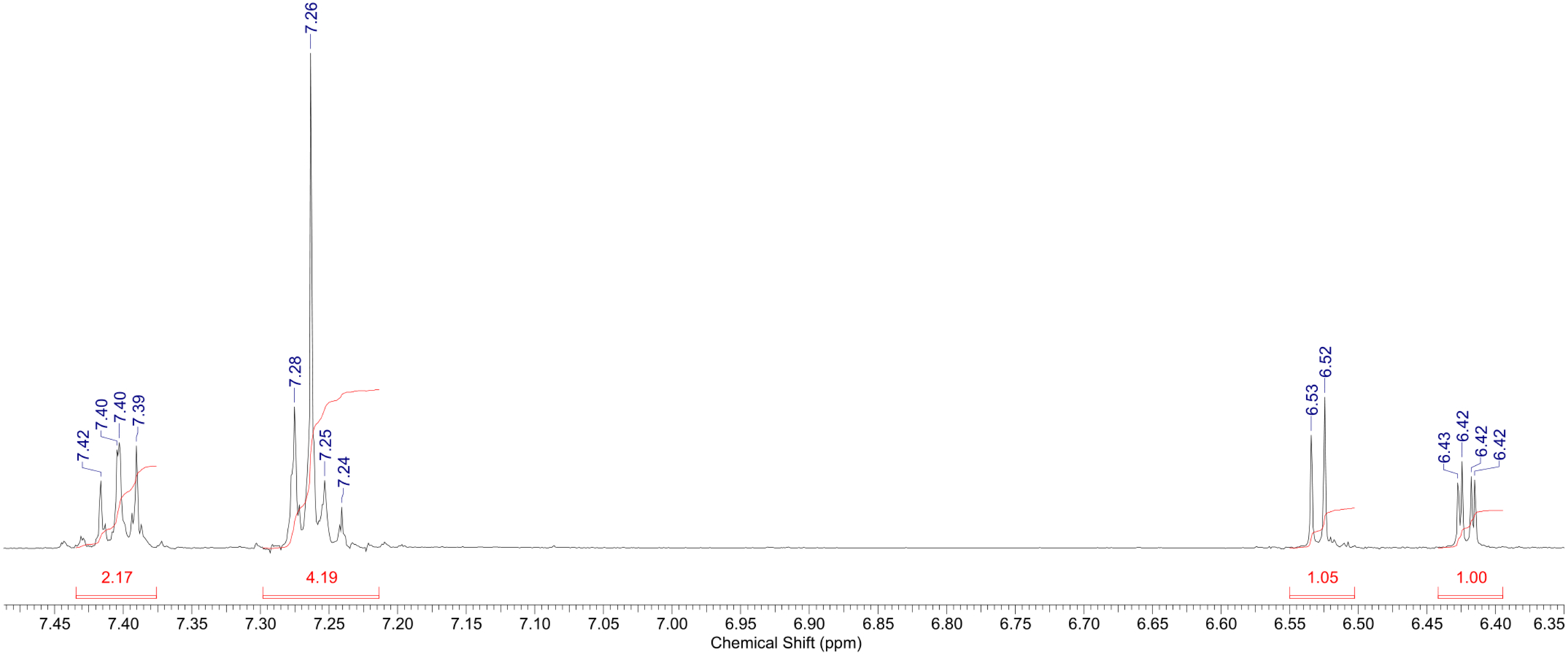

Acquisition Time (sec)	1.9818	Comment	single_pulse	Date	13 May 1990 05:13:25				
Date Stamp	23 Jul 2019 11:16:58			File Name	C:\Users\Fedor\Desktop\22.07.19\FZ7586-1.jdf			Frequency (MHz)	600.17
Nucleus	1H	Number of Transients	8	Origin	ECA 600	Original Points Count	32768	Owner	delta
Points Count	32768	Pulse Sequence	single_pulse.ex2			Receiver Gain	46.00	Solvent	CHLOROFORM-d
Spectrum Offset (Hz)	5409.6182	Sweep Width (Hz)	16534.39	Temperature (degree C)	22.900				

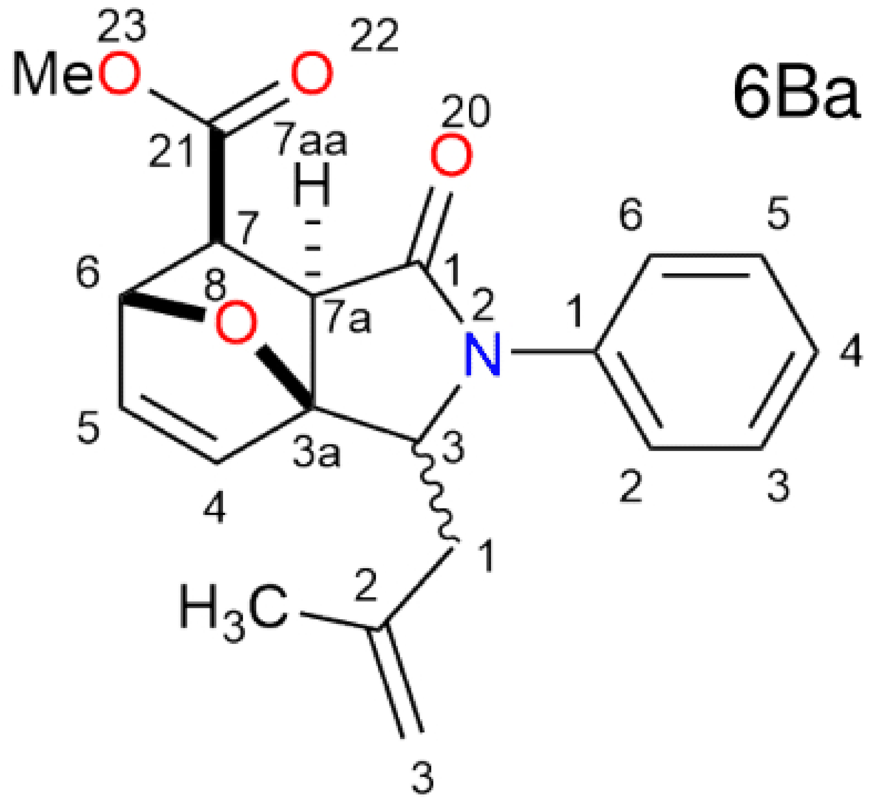

6Ba

FZ7586-1.esp

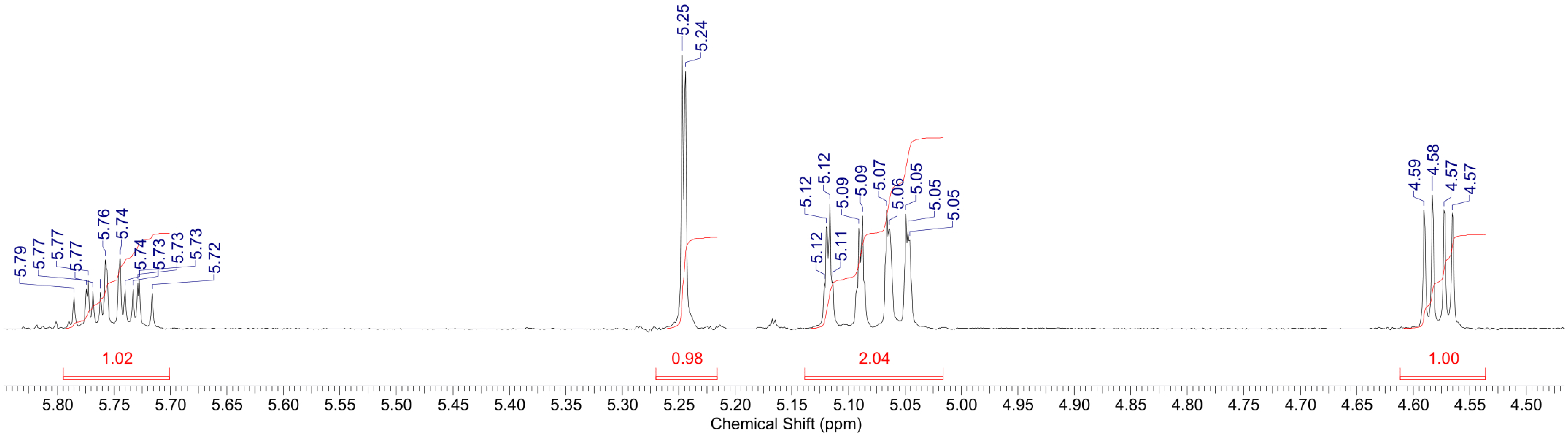

Acquisition Time (sec)	1.9818	***Comment***	single_pulse	***Date***	13 May 1990 05:13:25		
Date Stamp	23 Jul 2019 11:16:58			***File Name***	C:\Users\Fedor\Desktop\22.07.19\FZ7586-1.jdf	***Frequency (MHz)***	600.17
Nucleus	1H	***Number of Transients***	8	***Origin***	ECA 600	***Original Points Count*** 32768	***Owner*** delta
Points Count	32768	***Pulse Sequence***	single_pulse.ex2			***Receiver Gain*** 46.00	***Solvent*** CHLOROFORM-d
Spectrum Offset (Hz)	5409.6182	***Sweep Width (Hz)***	16534.39	***Temperature (degree C)***	22.900		

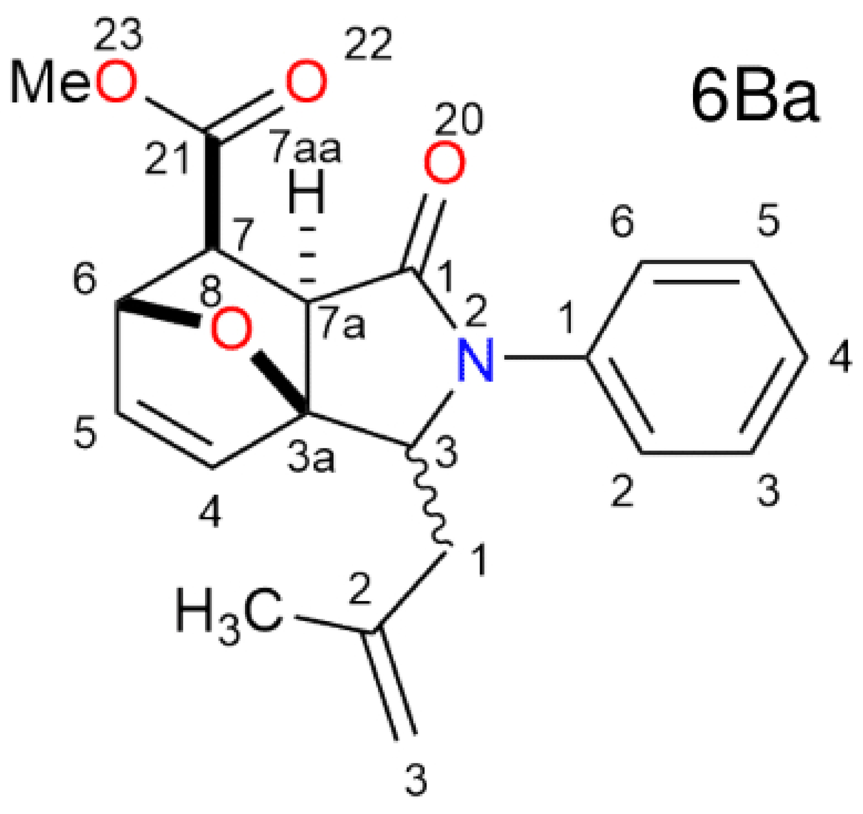

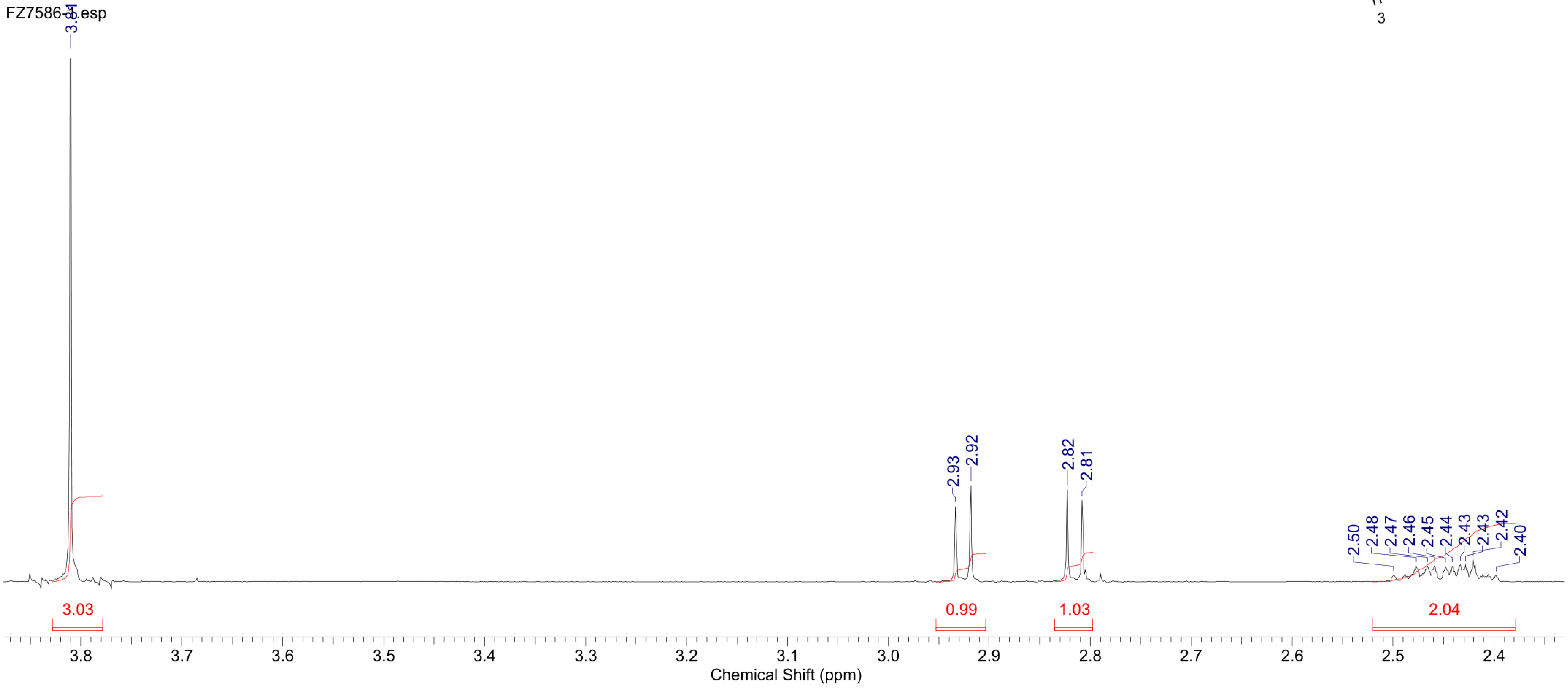

NMR spectrum data for compound 6Ae

Acquisition Time (sec)	1.9818	***Comment***	single_pulse	***Date***	19 May 1990 06:25:00		
Date Stamp	29 Jul 2019 12:28:39			***File Name***	C:\Users\Fedor\Desktop\25.07.19\FZ7600-1.jdf	***Frequency (MHz)***	600.17
Nucleus	1H	***Number of Transients***	8	***Origin***	ECA 600	***Original Points Count***	32768
Owner	delta						
Points Count	32768	***Pulse Sequence***	single_pulse.ex2	***Receiver Gain***	40.00	***Solvent***	CHLOROFORM-d
Spectrum Offset (Hz)	5413.1504	***Sweep Width (Hz)***	16534.39	***Temperature (degree C)***	23.800		

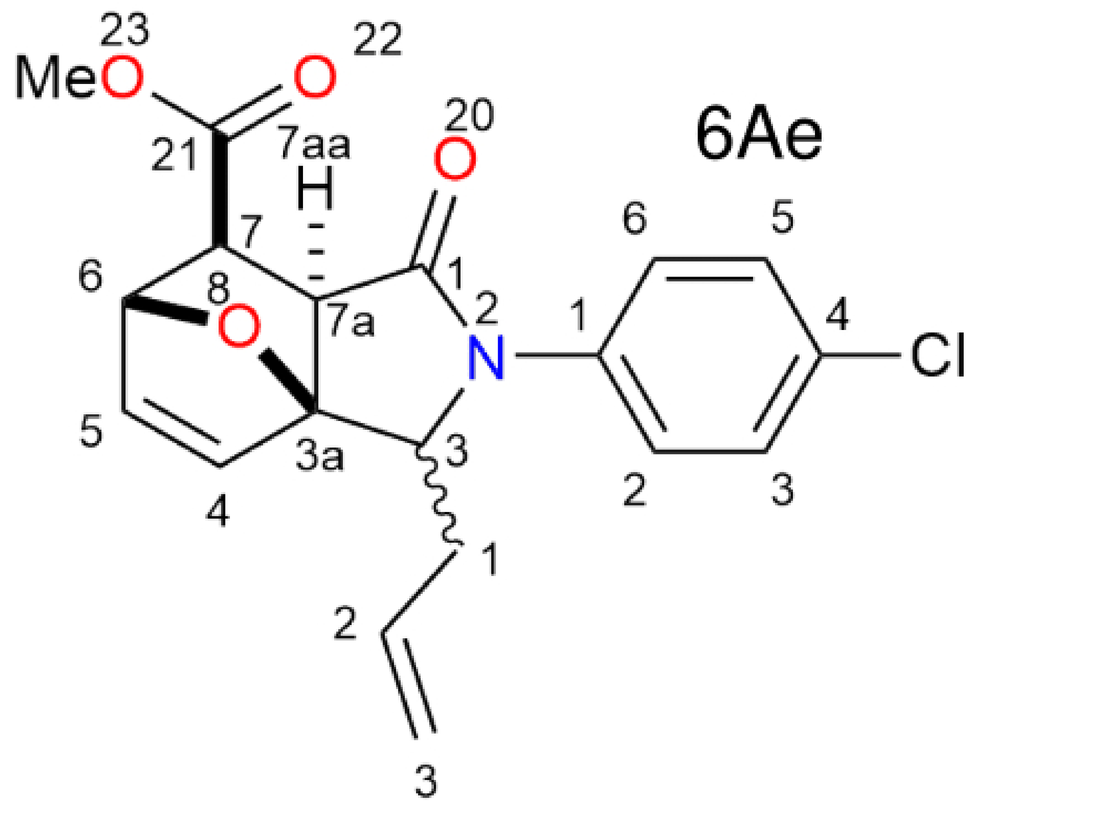

FZ7600-1.jdf

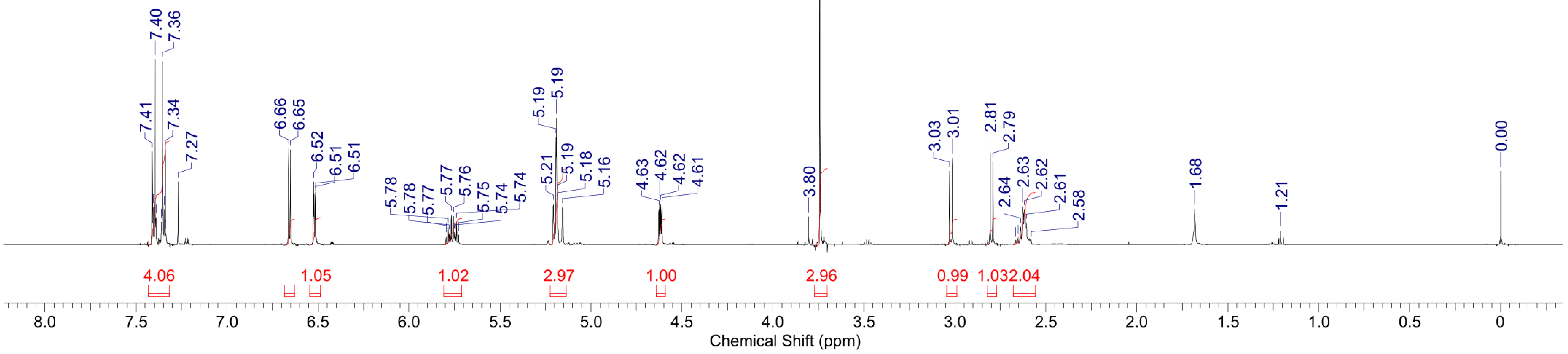

Acquisition Time (sec)	1.9818	***Comment***	single_pulse	***Date***	19 May 1990 06:25:00				
Date Stamp	29 Jul 2019 12:28:39			***File Name***	C:\Users\Fedor\Desktop\25.07.19\FZ7600-1.jdf			***Frequency (MHz)***	600.17
Nucleus	1H	***Number of Transients***	8	***Origin***	ECA 600	***Original Points Count***	32768	***Owner***	delta
Points Count	32768	***Pulse Sequence***	single_pulse.ex2			***Receiver Gain***	40.00	***Solvent***	CHLOROFORM-d
Spectrum Offset (Hz)	5413.1504	***Sweep Width (Hz)***	16534.39	***Temperature (degree C)***	23.800				

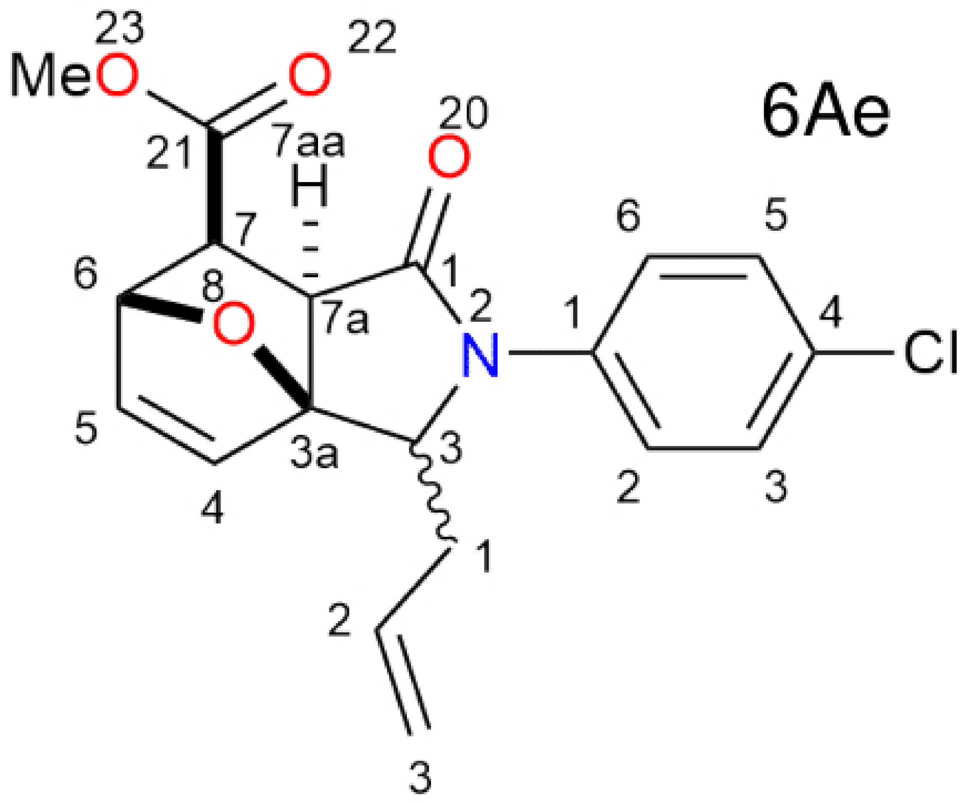

FZ7600-1.jdf

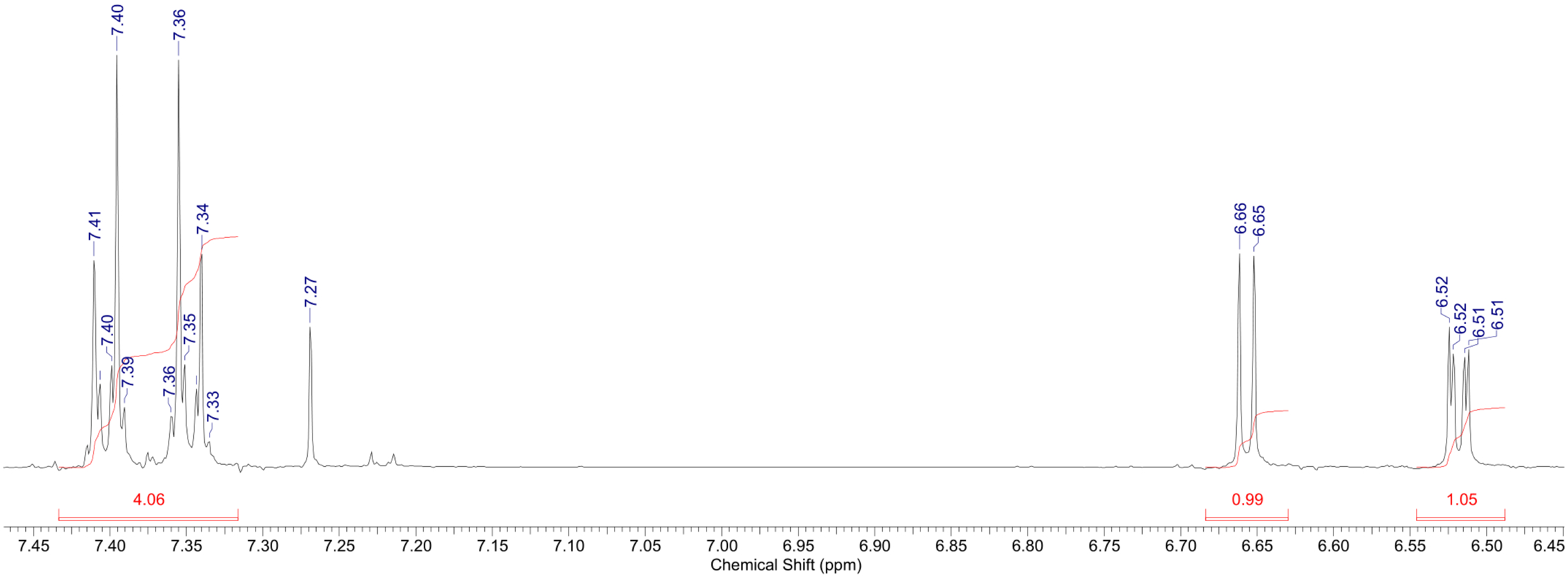

Acquisition Time (sec)	1.9818	Comment	single_pulse	Date	19 May 1990 06:25:00				
Date Stamp	29 Jul 2019 12:28:39			File Name	C:\Users\Fedor\Desktop\25.07.19\FZ7600-1.jdf			Frequency (MHz)	600.17
Nucleus	1H	Number of Transients	8	Origin	ECA 600	Original Points Count	32768	Owner	delta
Points Count	32768	Pulse Sequence	single_pulse.ex2			Receiver Gain	40.00	Solvent	CHLOROFORM-d
Spectrum Offset (Hz)	5413.1504	Sweep Width (Hz)	16534.39	Temperature (degree C)	23.800				

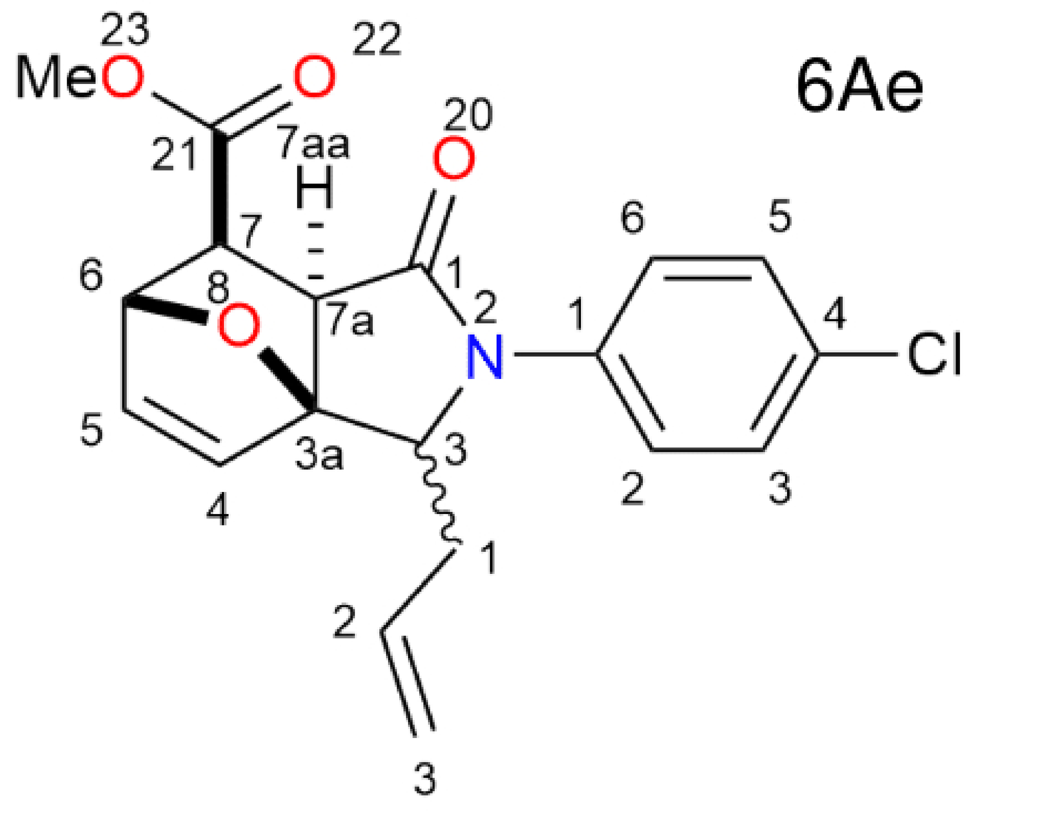

6Ae

FZ7600-1.jdf

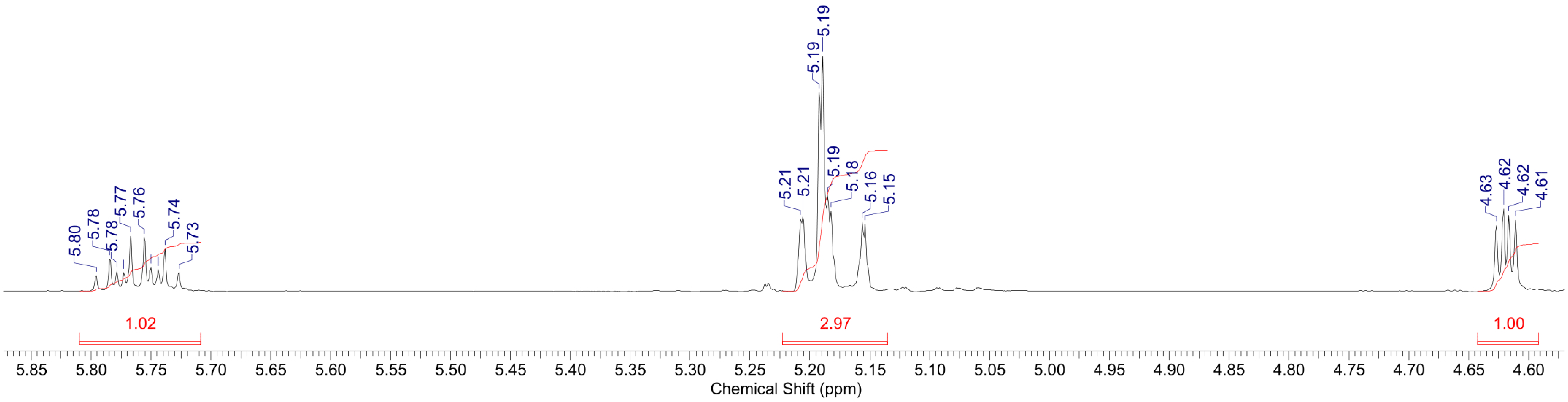

Acquisition Time (sec)	1.9818	***Comment***	single_pulse	***Date***	19 May 1990 06:25:00				
Date Stamp	29 Jul 2019 12:28:39			***File Name***	C:\Users\Fedor\Desktop\25.07.19\FZ7600-1.jdf			***Frequency (MHz)***	600.17
Nucleus	1H	***Number of Transients***	8	***Origin***	ECA 600	***Original Points Count***	32768	***Owner***	delta
Points Count	32768	***Pulse Sequence***	single_pulse.ex2			***Receiver Gain***	40.00	***Solvent***	CHLOROFORM-d
Spectrum Offset (Hz)	5413.1504	***Sweep Width (Hz)***	16534.39	***Temperature (degree C)***	23.800				

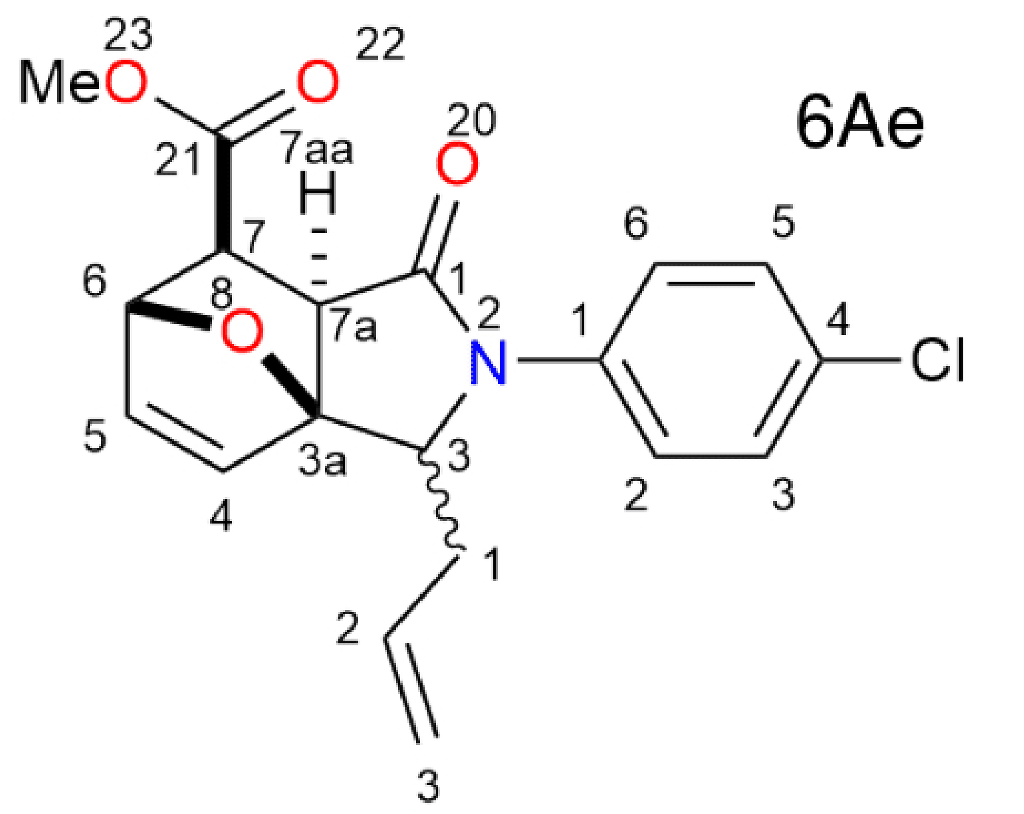

6Ae

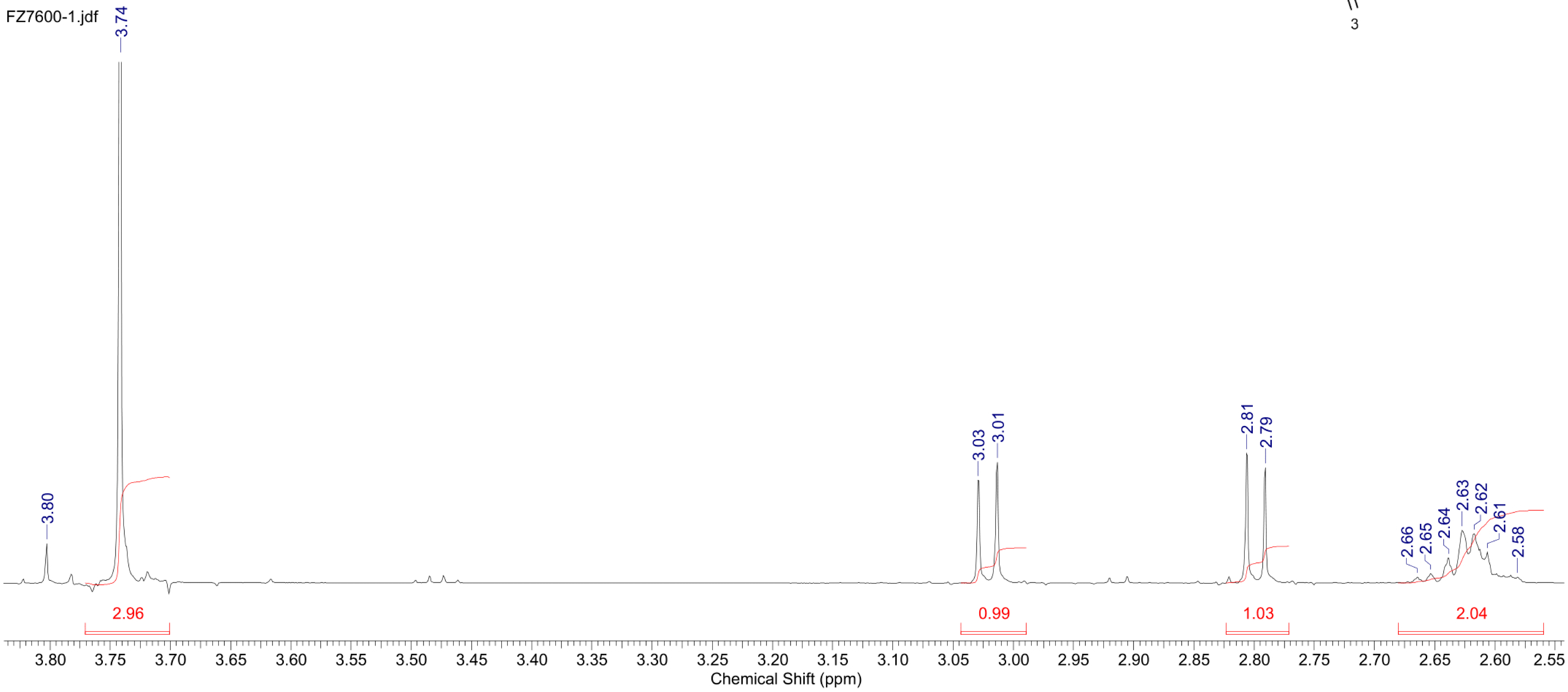

NMR spectrum data for compound 6Be

Acquisition Time (sec)	1.9818	***Comment***	single_pulse	***Date***	08 May 1990 02:57:42		
Date Stamp	18 Jul 2019 09:01:21			***File Name***	C:\Users\Fedor\Desktop\15.07.19\FZ7531-1.jdf	***Frequency (MHz)***	600.17
Nucleus	1H	***Number of Transients***	8	***Origin***	ECA 600	***Original Points Count***	32768
Owner	delta						
Points Count	32768	***Pulse Sequence***	single_pulse.ex2	***Receiver Gain***	38.00	***Solvent***	CHLOROFORM-d
Spectrum Offset (Hz)	5412.6455	***Sweep Width (Hz)***	16534.39	***Temperature (degree C)***	22.500		

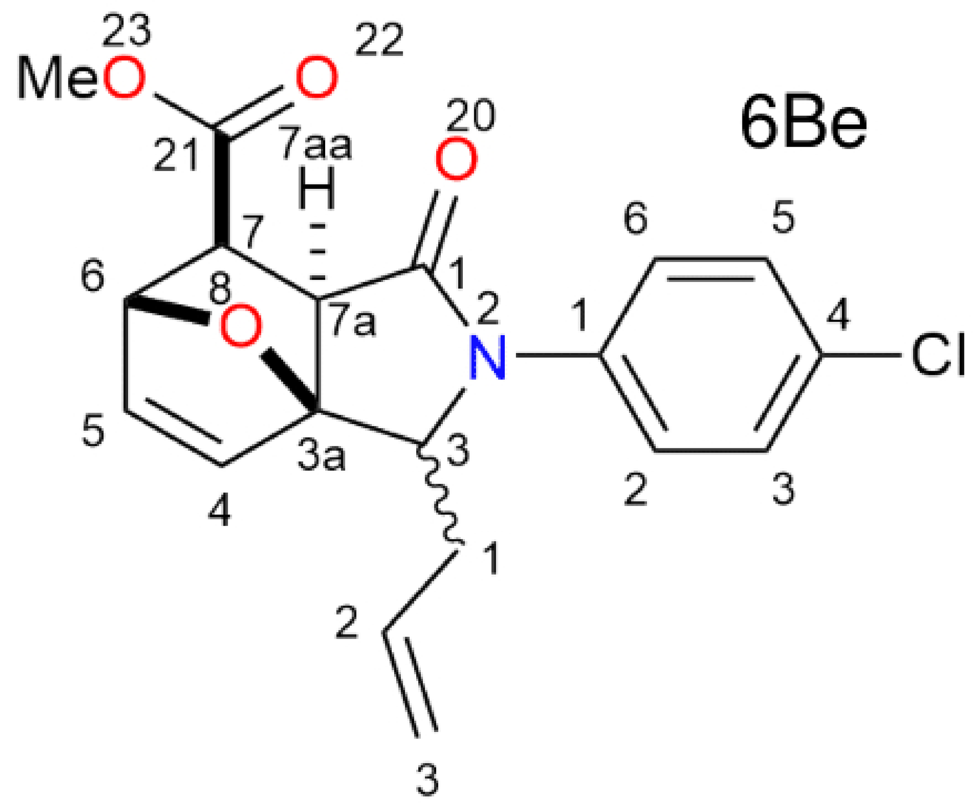

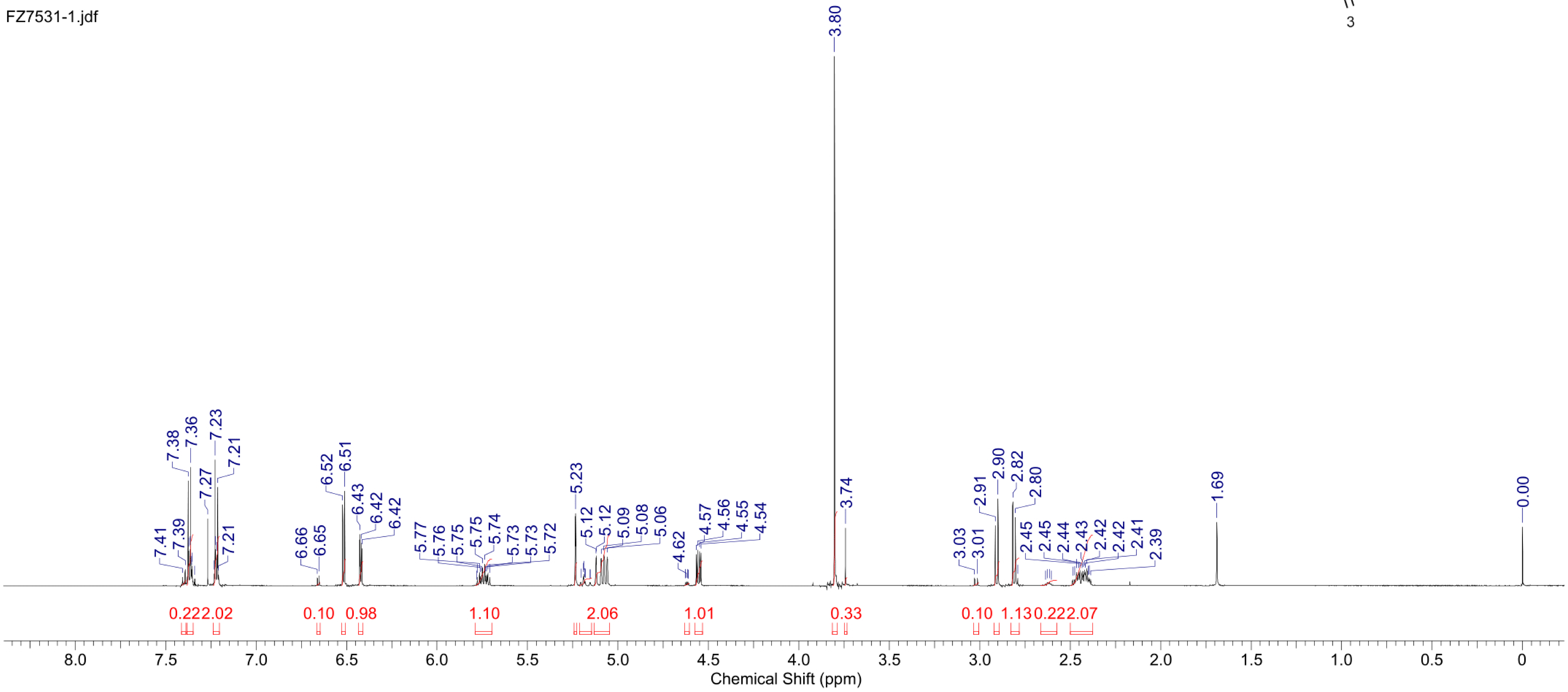

Acquisition Time (sec)	1.9818	Comment	single_pulse	Date	08 May 1990 02:57:42				
Date Stamp	18 Jul 2019 09:01:21			File Name	C:\Users\Fedor\Desktop\15.07.19\FZ7531-1.jdf			Frequency (MHz)	600.17
Nucleus	1H	Number of Transients	8	Origin	ECA 600	Original Points Count	32768	Owner	delta
Points Count	32768	Pulse Sequence	single_pulse.ex2			Receiver Gain	38.00	Solvent	CHLOROFORM-d
Spectrum Offset (Hz)	5412.6455	Sweep Width (Hz)	16534.39	Temperature (degree C)	22.500				

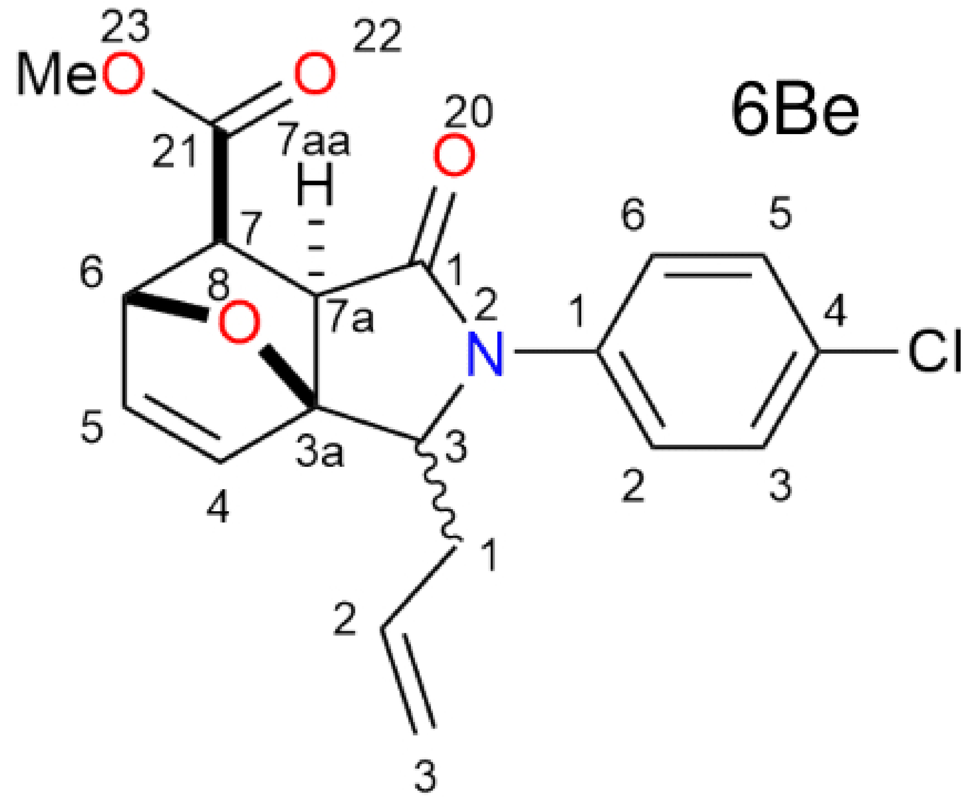

6Be

FZ7531-1.jdf

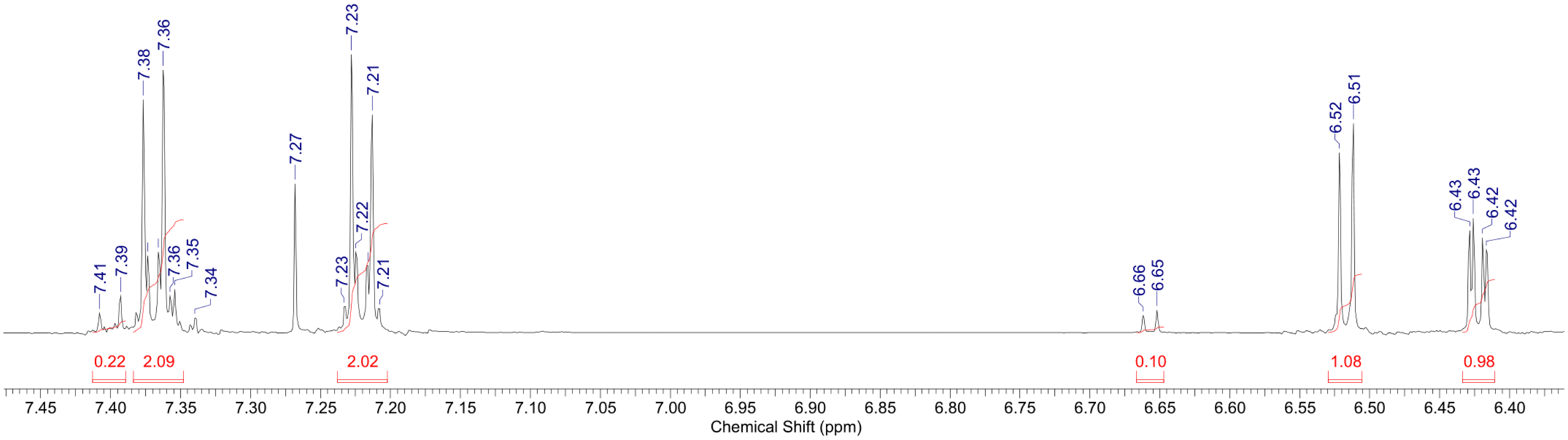

Acquisition Time (sec)	1.9818	***Comment***	single_pulse	***Date***	08 May 1990 02:57:42		
Date Stamp	18 Jul 2019 09:01:21			***File Name***	C:\Users\Fedor\Desktop\15.07.19\FZ7531-1.jdf	***Frequency (MHz)***	600.17
Nucleus	1H	***Number of Transients***	8	***Origin***	ECA 600	***Original Points Count***	32768
Owner	delta						
Points Count	32768	***Pulse Sequence***	single_pulse.ex2	***Receiver Gain***	38.00	***Solvent***	CHLOROFORM-d
Spectrum Offset (Hz)	5412.6455	***Sweep Width (Hz)***	16534.39	***Temperature (degree C)***	22.500		

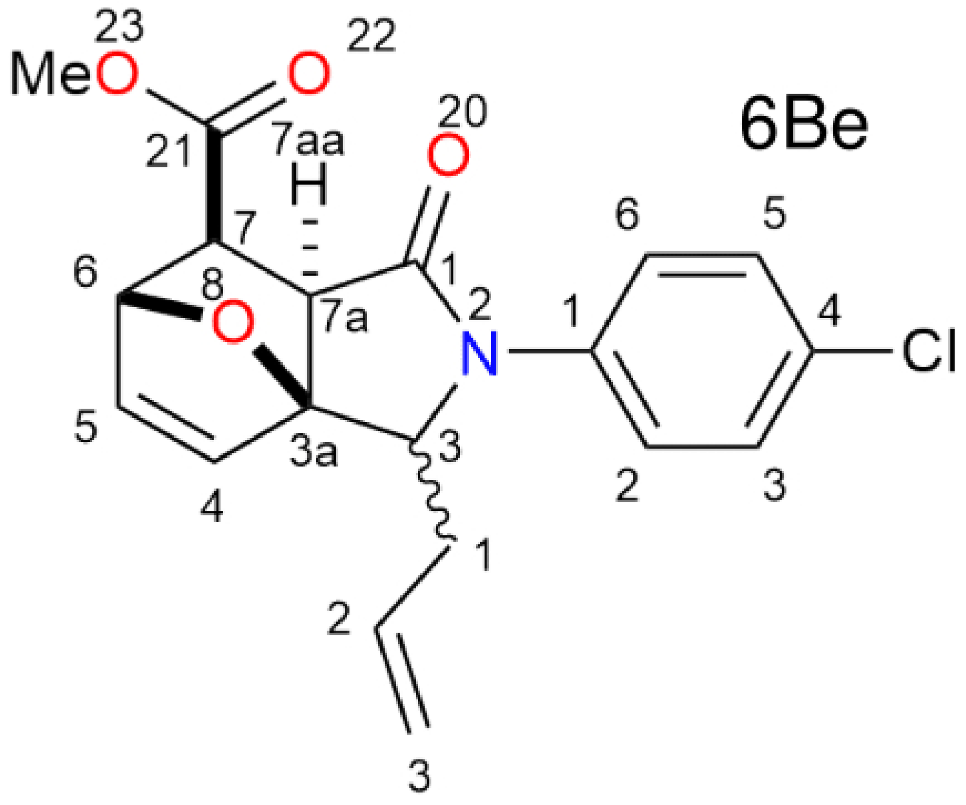

FZ7531-1.jdf

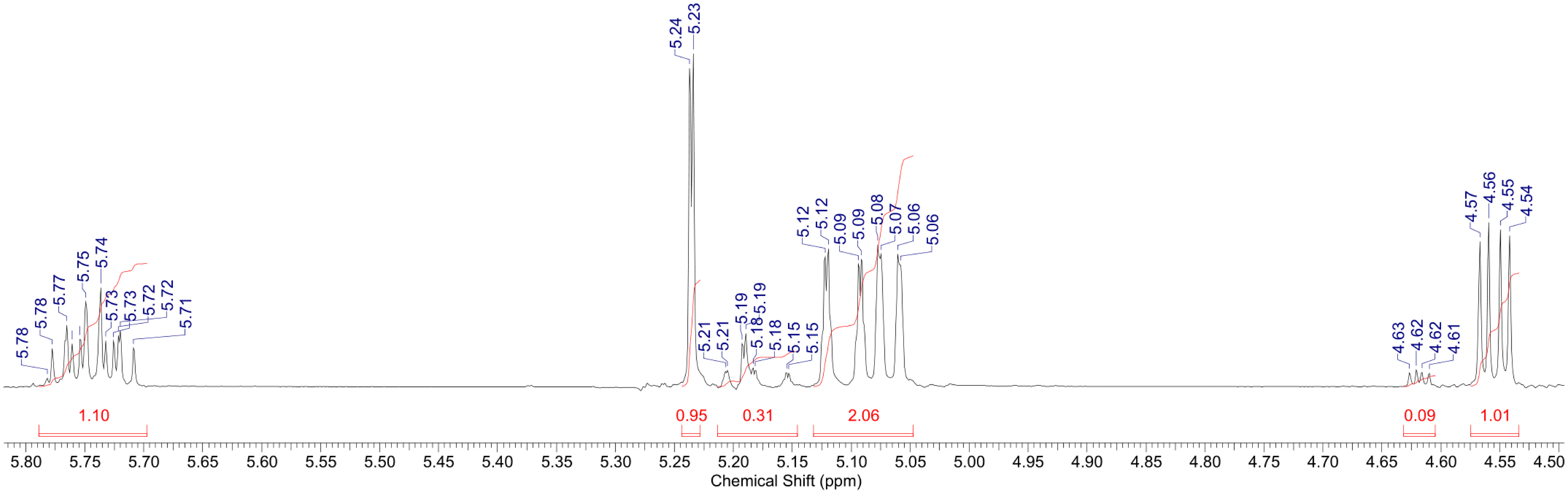

Acquisition Time (sec)	1.9818	***Comment***	single_pulse	***Date***	08 May 1990 02:57:42				
Date Stamp	18 Jul 2019 09:01:21			***File Name***	C:\Users\Fedor\Desktop\15.07.19\FZ7531-1.jdf			***Frequency (MHz)***	600.17
Nucleus	1H	***Number of Transients***	8	***Origin***	ECA 600	***Original Points Count***	32768	***Owner***	delta
Points Count	32768	***Pulse Sequence***	single_pulse.ex2			***Receiver Gain***	38.00	***Solvent***	CHLOROFORM-d
Spectrum Offset (Hz)	5412.6455	***Sweep Width (Hz)***	16534.39	***Temperature (degree C)***	22.500				

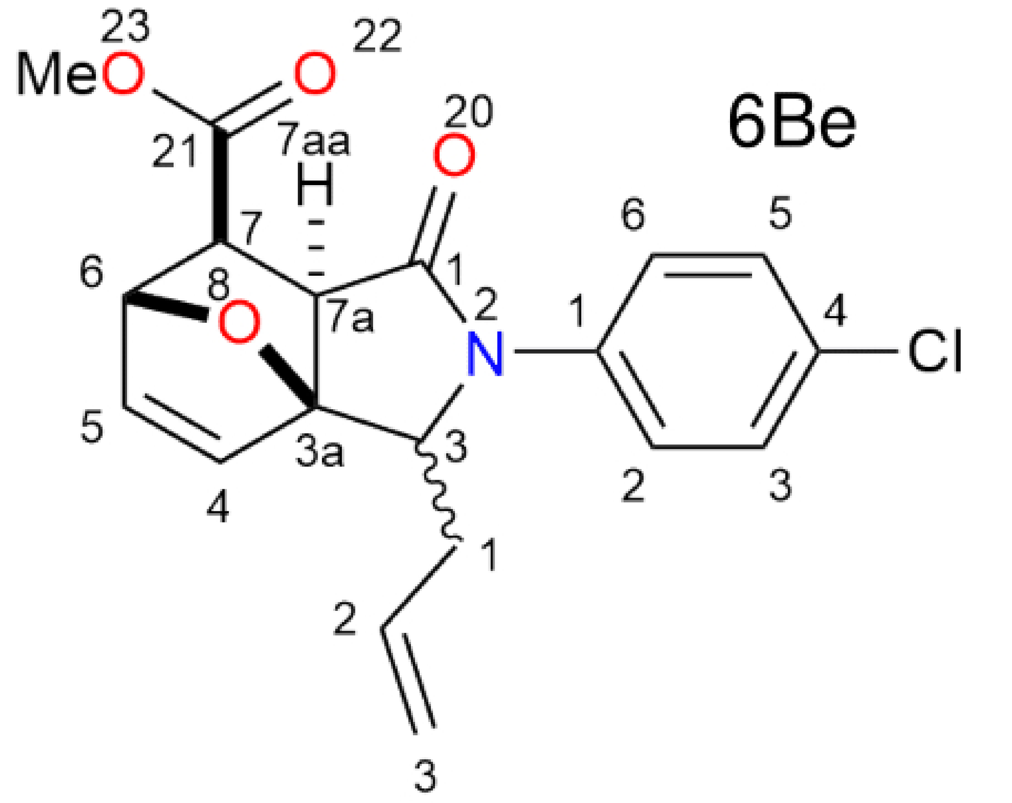

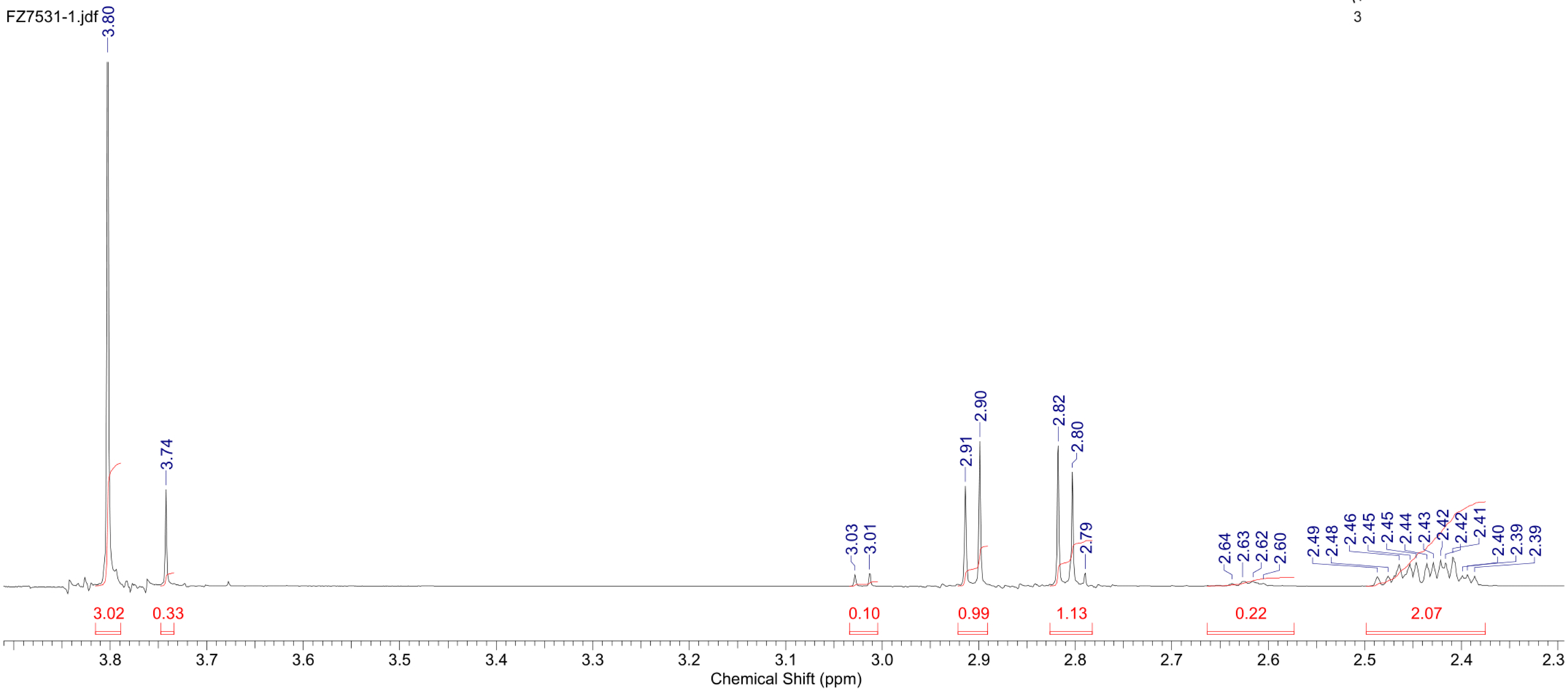

NMR spectrum data for compound 6Af

Acquisition Time (sec)	1.9818	***Comment***	single_pulse	***Date***	09 May 1990 04:12:54				
Date Stamp	19 Jul 2019 10:16:33			***File Name***	C:\Users\Fedor\Desktop\18.07.19\FZ7577-1.jdf			***Frequency (MHz)***	600.17
Nucleus	1H	***Number of Transients***	8	***Origin***	ECA 600	***Original Points Count***	32768	***Owner***	delta
Points Count	32768	***Pulse Sequence***	single_pulse.ex2			***Receiver Gain***	38.00	***Solvent***	CHLOROFORM-d
Spectrum Offset (Hz)	5413.6548	***Sweep Width (Hz)***	16534.39	***Temperature (degree C)***	22.700				

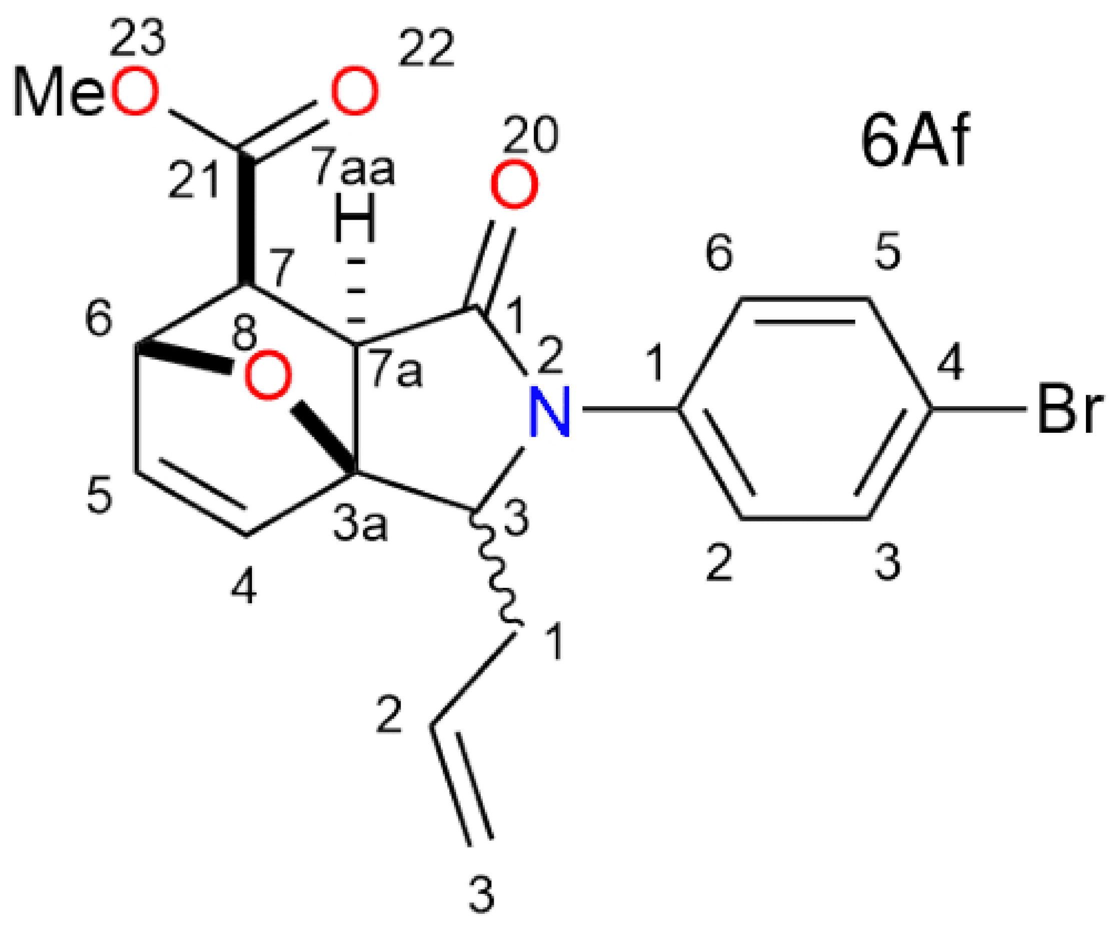

FZ7577-1.esp

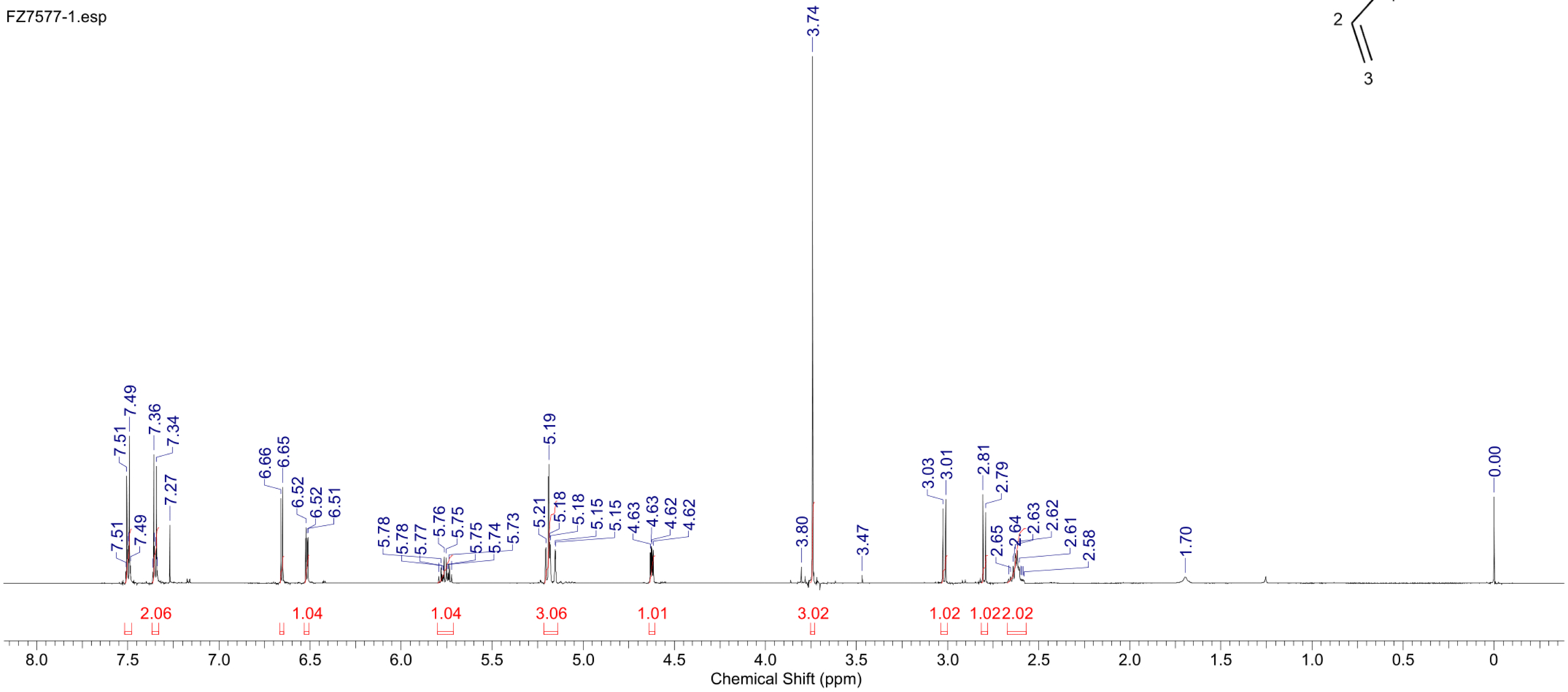

Acquisition Time (sec)	1.9818	***Comment***	single_pulse	***Date***	09 May 1990 04:12:54				
Date Stamp	19 Jul 2019 10:16:33			***File Name***	C:\Users\Fedor\Desktop\18.07.19\FZ7577-1.jdf			***Frequency (MHz)***	600.17
Nucleus	1H	***Number of Transients***	8	***Origin***	ECA 600	***Original Points Count***	32768	***Owner***	delta
Points Count	32768	***Pulse Sequence***	single_pulse.ex2			***Receiver Gain***	38.00	***Solvent***	CHLOROFORM-d
Spectrum Offset (Hz)	5413.6548	***Sweep Width (Hz)***	16534.39	***Temperature (degree C)***	22.700				

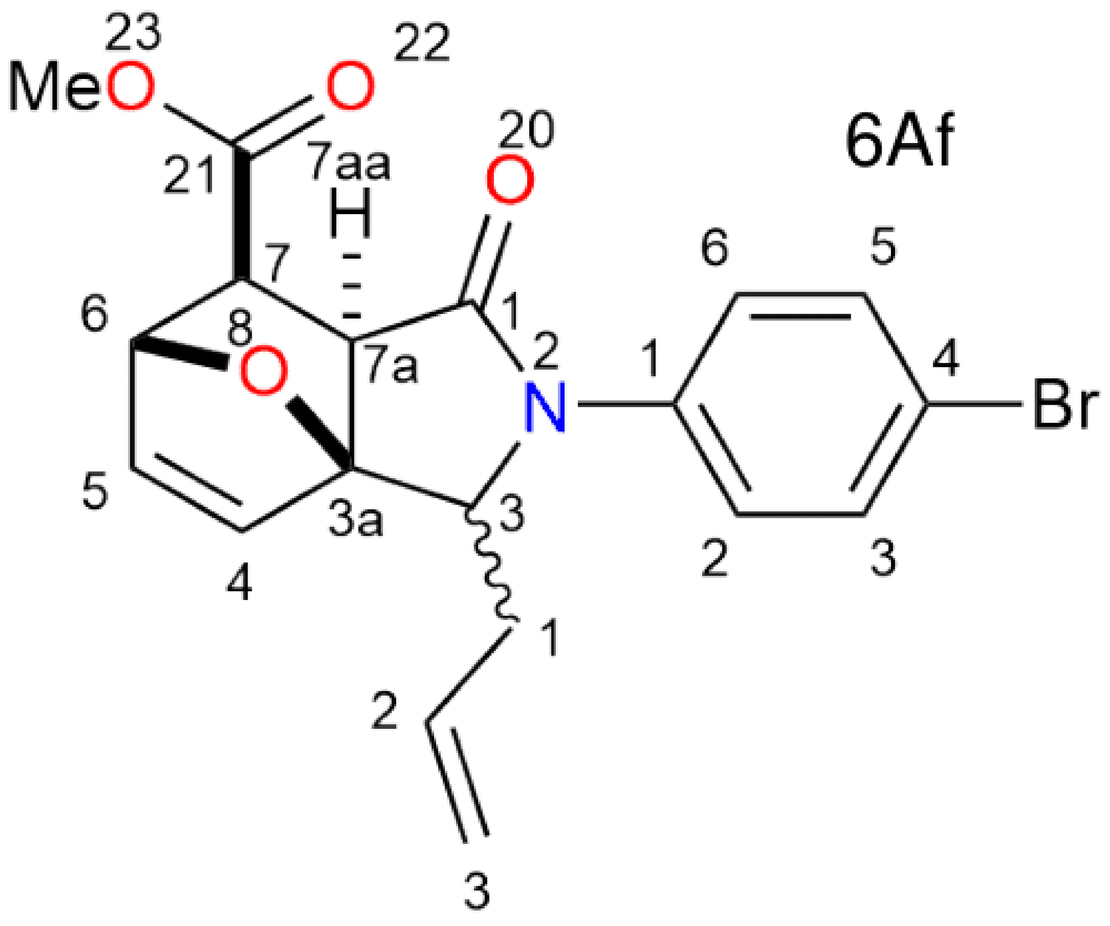

6Af

FZ7577-1.esp

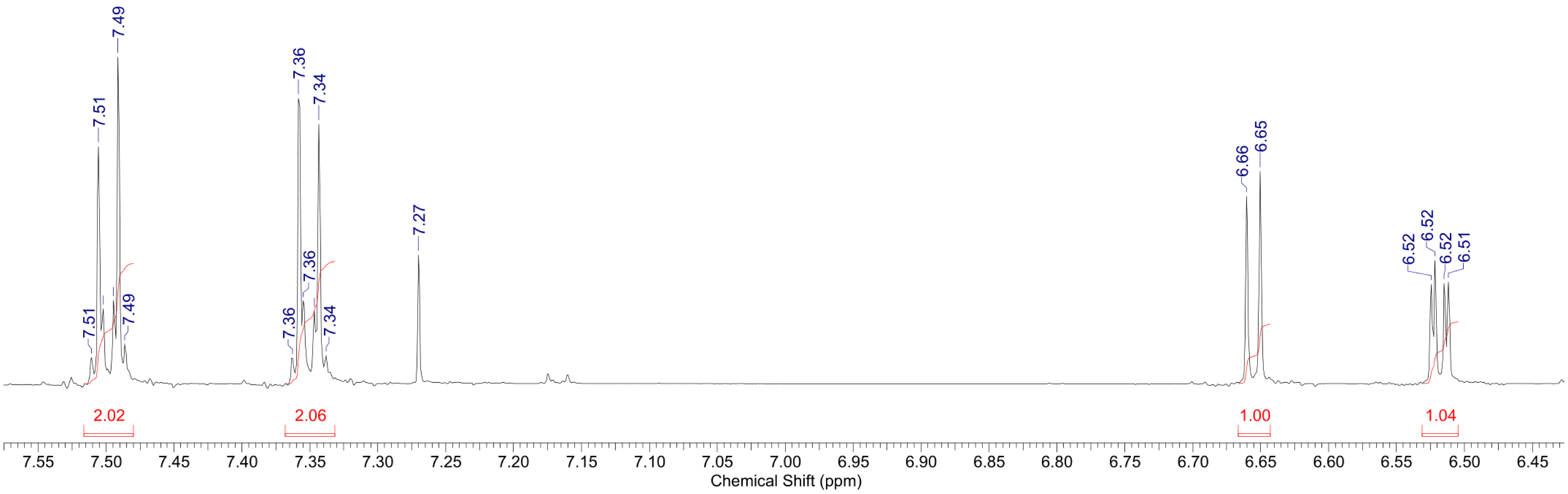

Acquisition Time (sec)	1.9818	***Comment***	single_pulse	***Date***	09 May 1990 04:12:54				
Date Stamp	19 Jul 2019 10:16:33			***File Name***	C:\Users\Fedor\Desktop\18.07.19\FZ7577-1.jdf			***Frequency (MHz)***	600.17
Nucleus	1H	***Number of Transients***	8	***Origin***	ECA 600	***Original Points Count***	32768	***Owner***	delta
Points Count	32768	***Pulse Sequence***	single_pulse.ex2			***Receiver Gain***	38.00	***Solvent***	CHLOROFORM-d
Spectrum Offset (Hz)	5413.6548	***Sweep Width (Hz)***	16534.39	***Temperature (degree C)***	22.700				

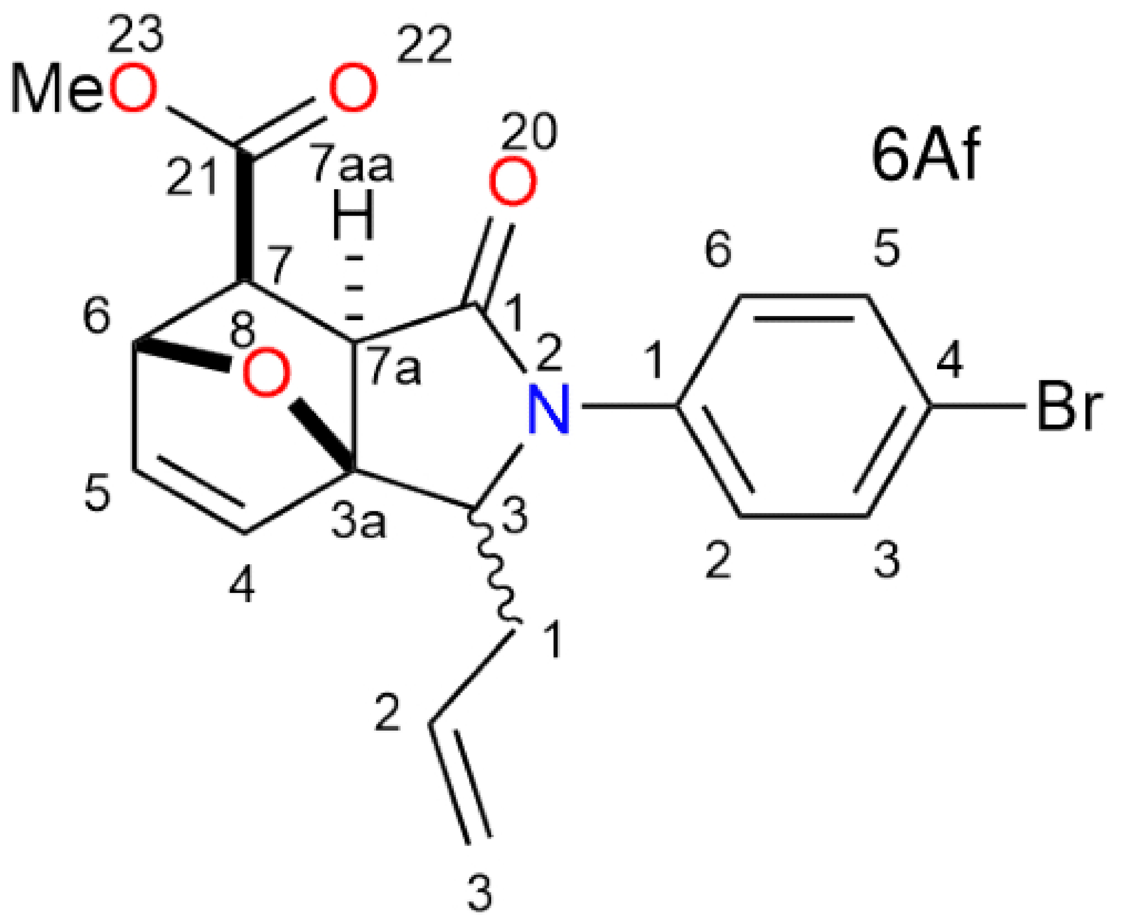

6Af

FZ7577-1.esp

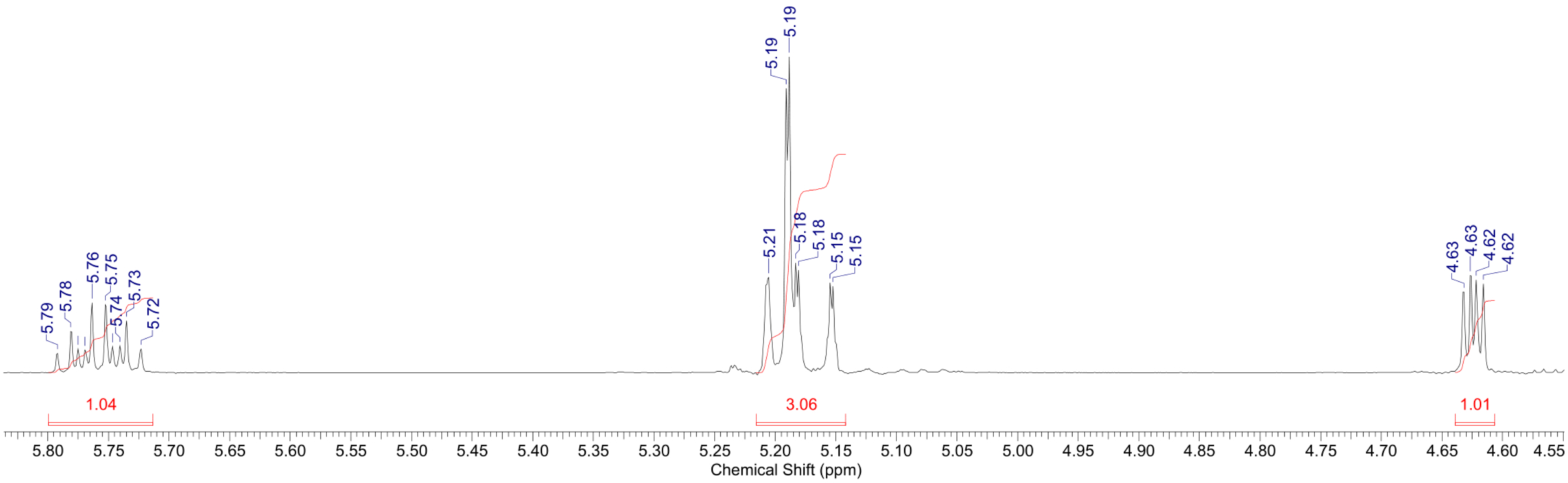

Acquisition Time (sec)	1.9818	***Comment***	single_pulse	***Date***	09 May 1990 04:12:54		
Date Stamp	19 Jul 2019 10:16:33			***File Name***	C:\Users\Fedor\Desktop\18.07.19\FZ7577-1.jdf	***Frequency (MHz)***	600.17
Nucleus	1H	***Number of Transients***	8	***Origin***	ECA 600	***Original Points Count*** 32768	***Owner*** delta
Points Count	32768	***Pulse Sequence***	single_pulse.ex2			***Receiver Gain*** 38.00	***Solvent*** CHLOROFORM-d
Spectrum Offset (Hz)	5413.6548	***Sweep Width (Hz)***	16534.39	***Temperature (degree C)***	22.700		

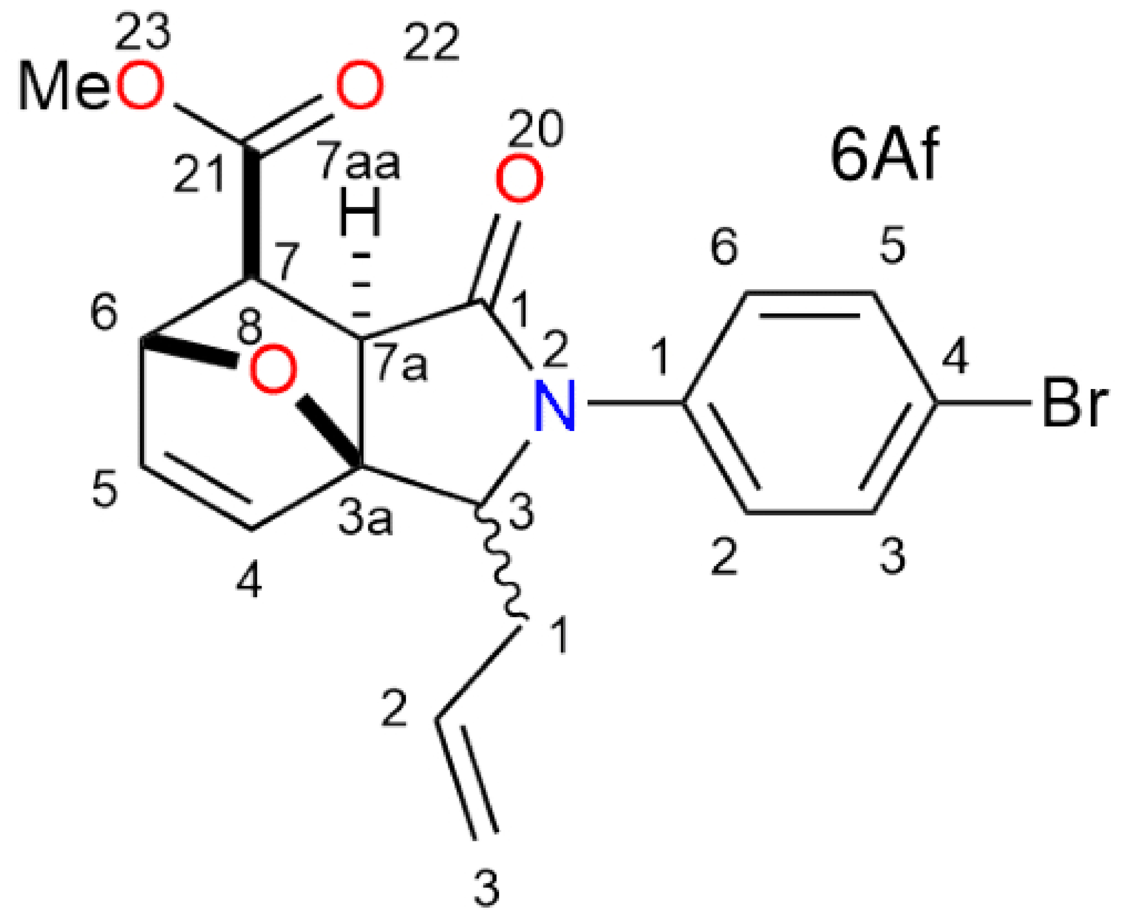

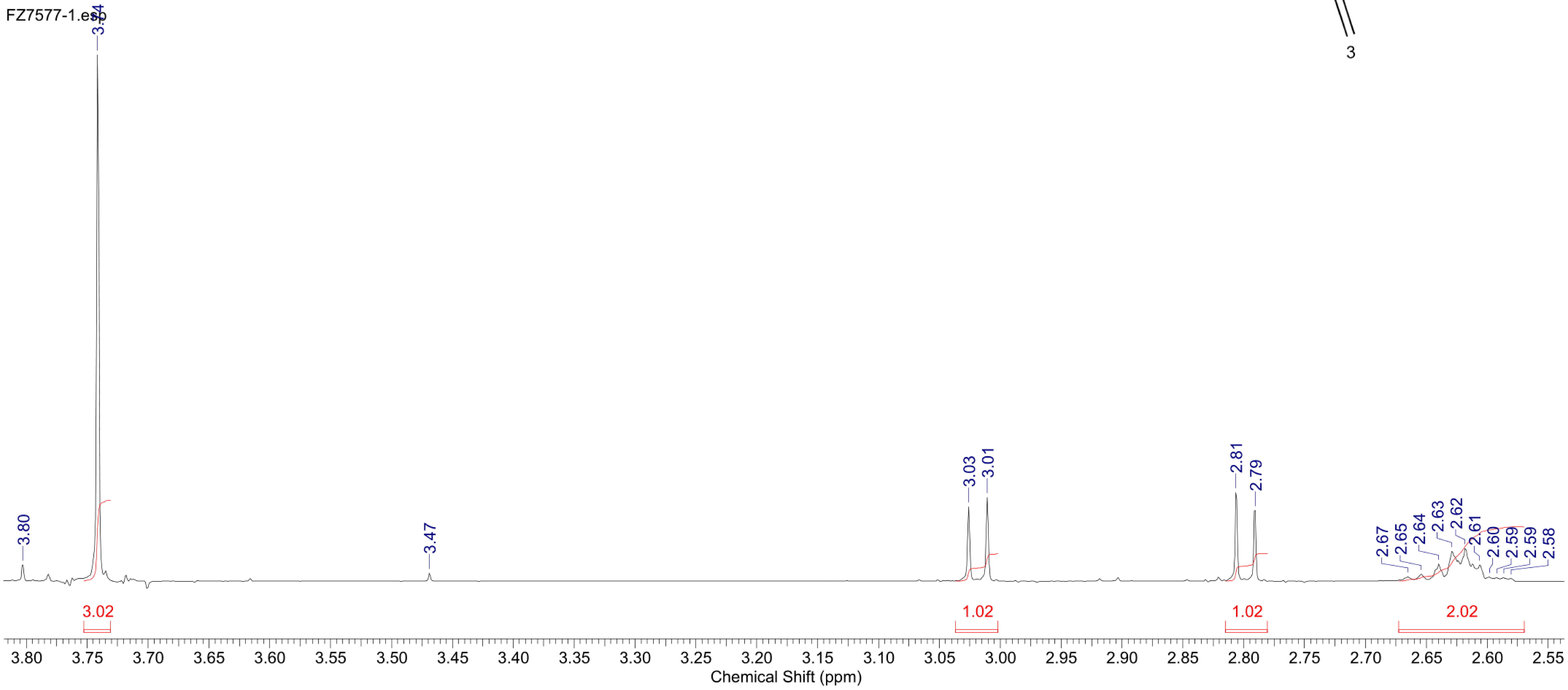

NMR spectrum data for compound 6Bf

**Acquisition Time (sec)**	1.9818	**Comment**	single_pulse	**Date**	22 May 1990 04:15:58		
**Date Stamp**	01 Aug 2019 10:19:40			**File Name**	C:\Users\Fedor\Desktop\01.08.19\FZ7623-1.jdf	**Frequency (MHz)**	600.17
**Nucleus**	1H	**Number of Transients**	8	**Origin**	ECA 600	**Original Points Count** 32768	**Owner** delta
**Points Count**	32768	**Pulse Sequence**	single_pulse.ex2			**Receiver Gain** 44.00	**Solvent** CHLOROFORM-d
**Spectrum Offset (Hz)**	5410.6274	**Sweep Width (Hz)**	16534.39	**Temperature (degree C)**	20.600		

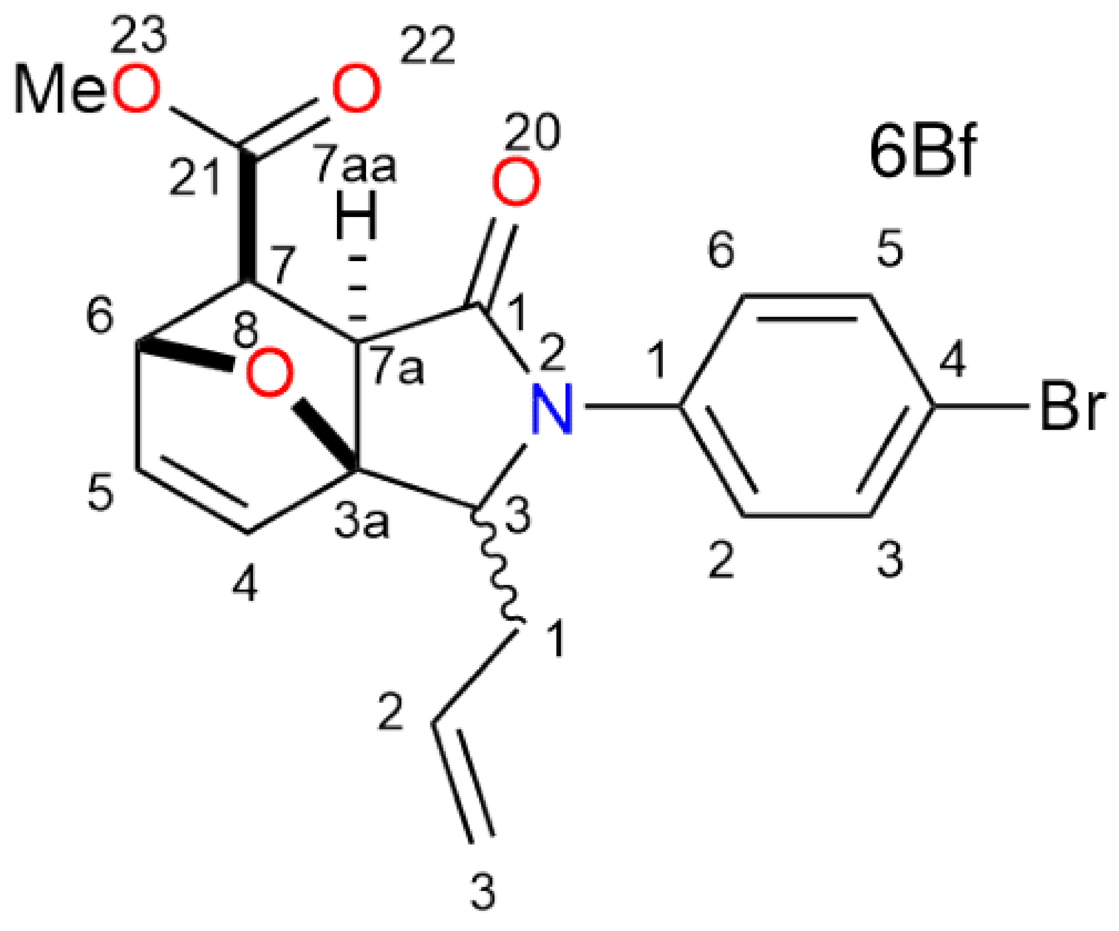

FZ7623-1.jdf

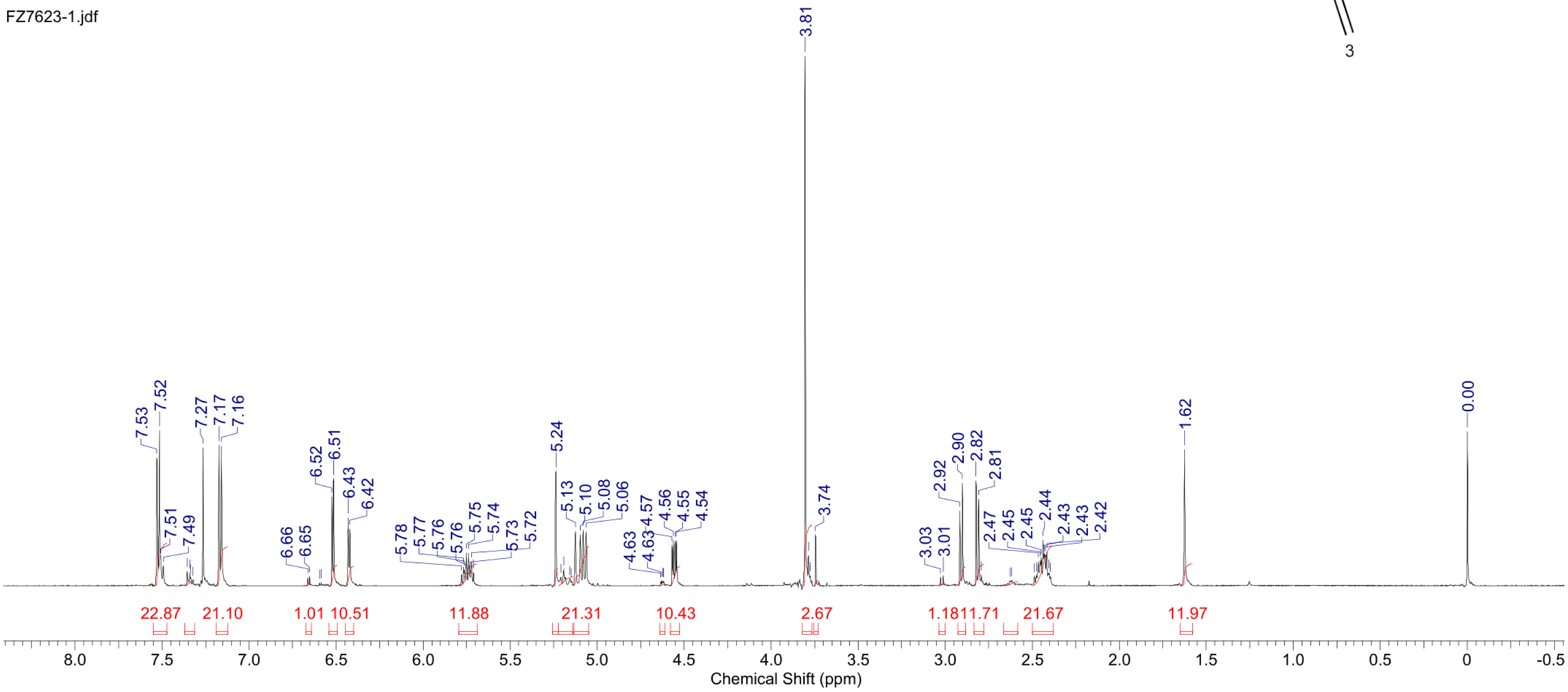

Acquisition Time (sec)	1.9818	***Comment***	single_pulse	***Date***	22 May 1990 04:15:58				
Date Stamp	01 Aug 2019 10:19:40			***File Name***	C:\Users\Fedor\Desktop\01.08.19\FZ7623-1.jdf			***Frequency (MHz)***	600.17
Nucleus	1H	***Number of Transients***	8	***Origin***	ECA 600	***Original Points Count***	32768	***Owner***	delta
Points Count	32768	***Pulse Sequence***	single_pulse.ex2			***Receiver Gain***	44.00	***Solvent***	CHLOROFORM-d
Spectrum Offset (Hz)	5410.6274	***Sweep Width (Hz)***	16534.39	***Temperature (degree C)***	20.600				

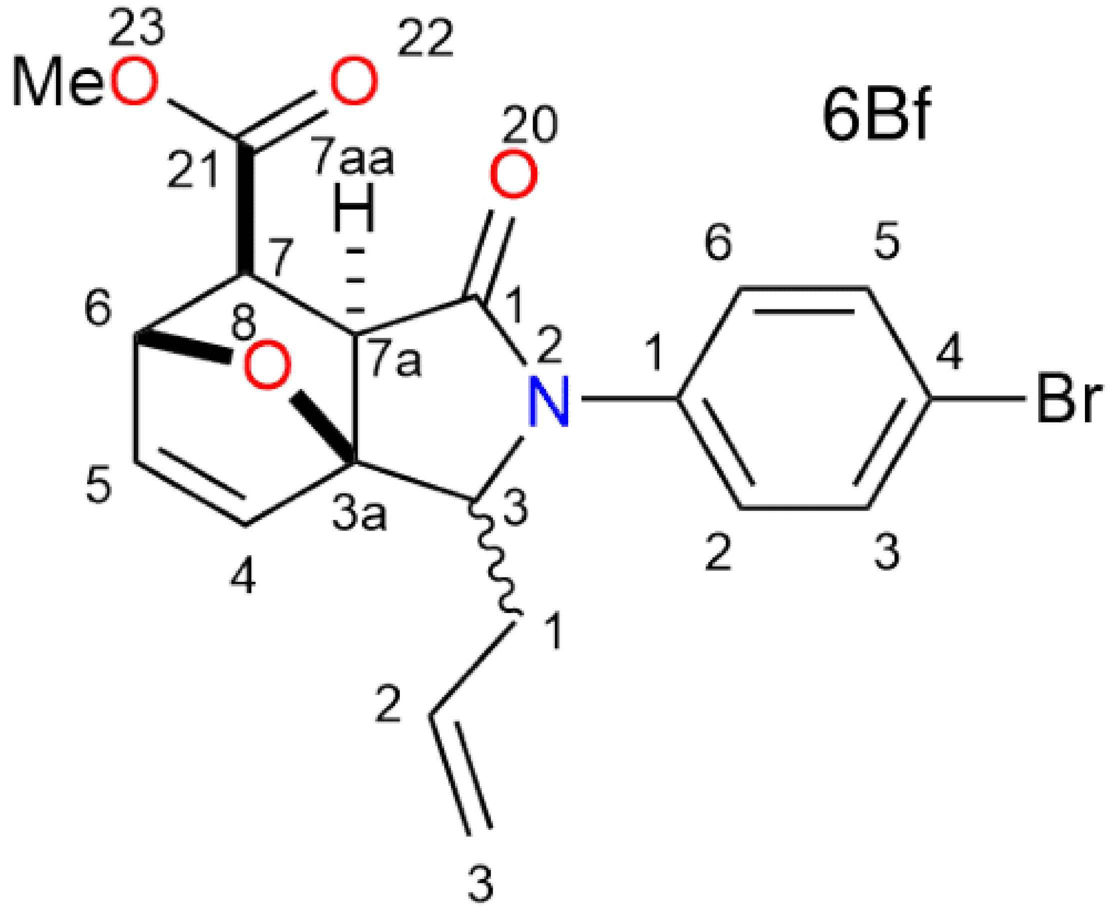

FZ7623-1.jdf

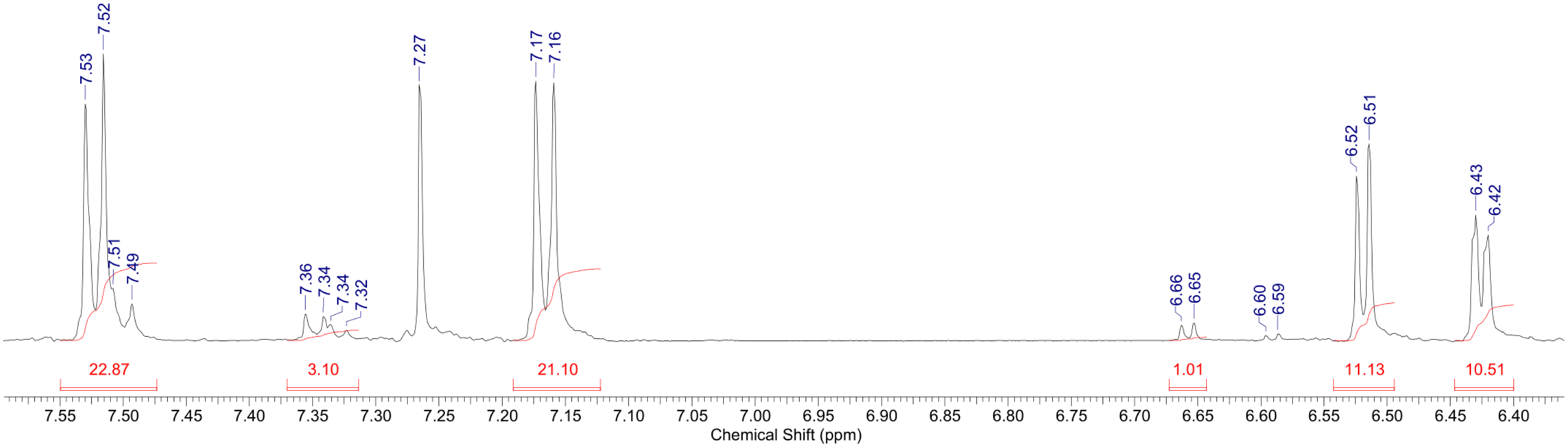

**_Acquisition Time (sec)_**	1.9818	**_Comment_**	single_pulse	**_Date_**	22 May 1990 04:15:58				
**_Date Stamp_**	01 Aug 2019 10:19:40			**_File Name_**	C:\Users\Fedor\Desktop\01.08.19\FZ7623-1.jdf			**_Frequency (MHz)_**	600.17
**_Nucleus_**	1H	**_Number of Transients_**	8	**_Origin_**	ECA 600	**_Original Points Count_**	32768	**_Owner_**	delta
**_Points Count_**	32768	**_Pulse Sequence_**	single_pulse.ex2			**_Receiver Gain_**	44.00	**_Solvent_**	CHLOROFORM-d
**_Spectrum Offset (Hz)_**	5410.6274	**_Sweep Width (Hz)_**	16534.39	**_Temperature (degree C)_**	20.600				

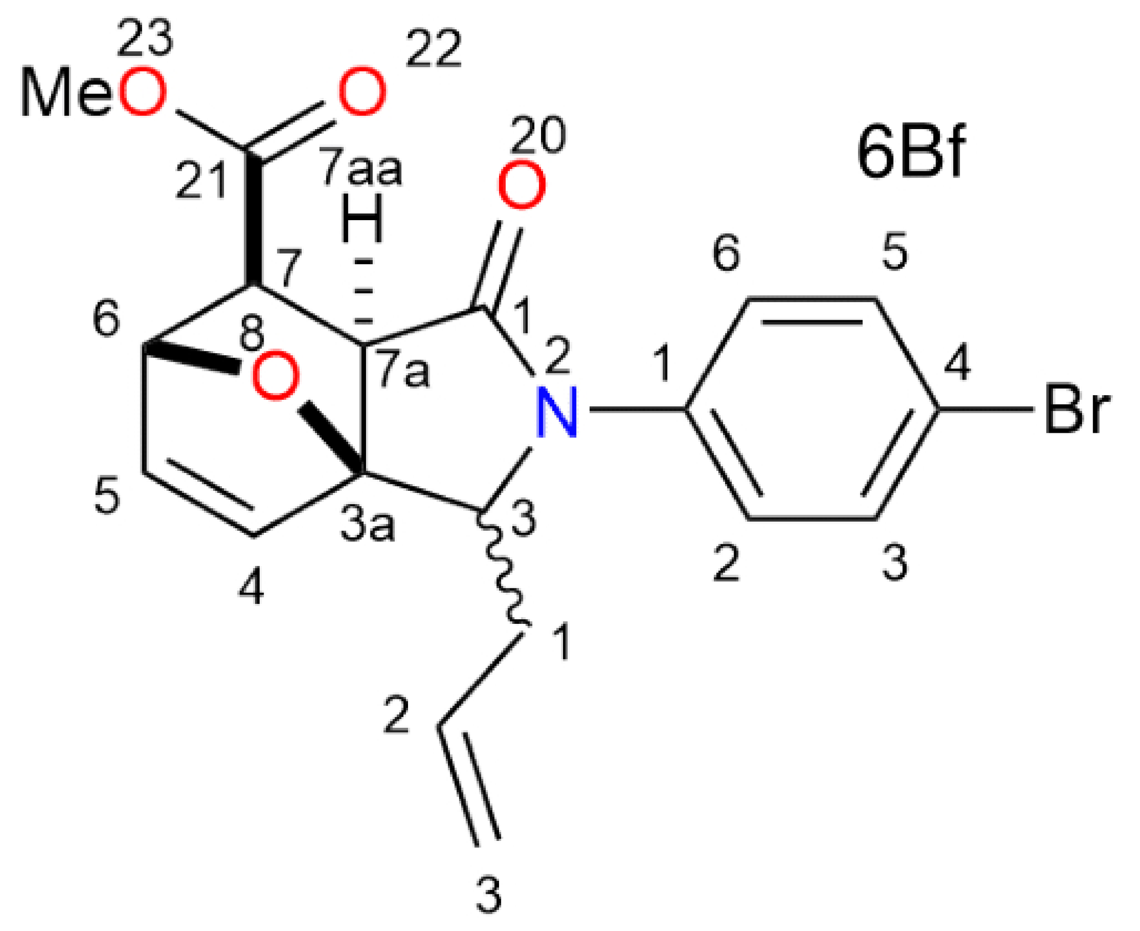

FZ7623-1.jdf

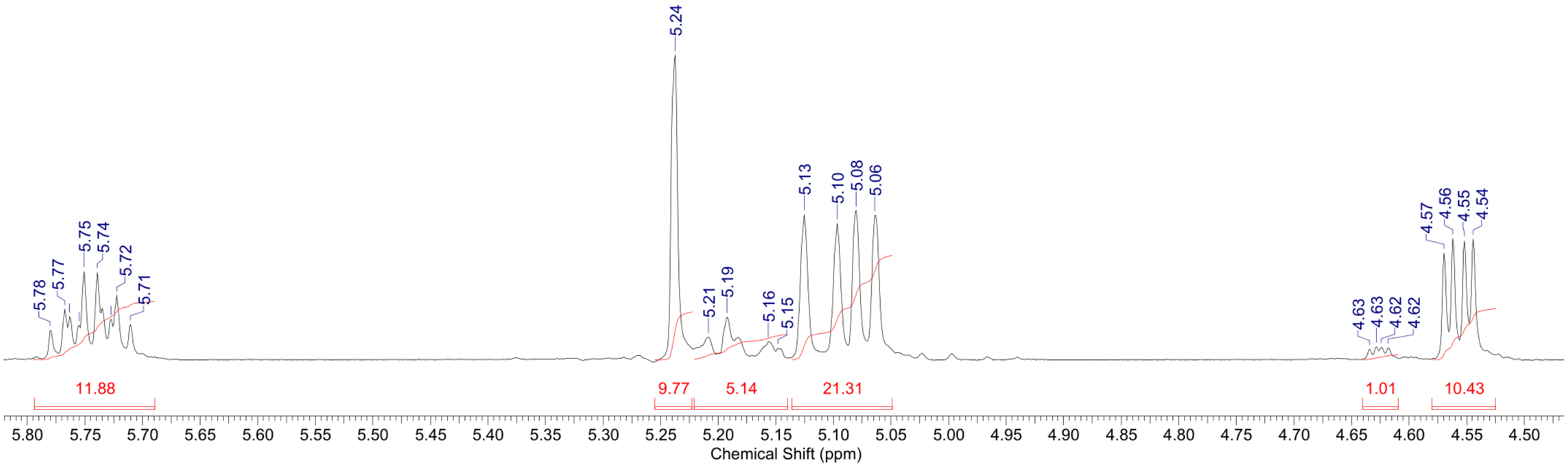

Acquisition Time (sec)	1.9818	***Comment***	single_pulse	***Date***	22 May 1990 04:15:58		
Date Stamp	01 Aug 2019 10:19:40			***File Name***	C:\Users\Fedor\Desktop\01.08.19\FZ7623-1.jdf	***Frequency (MHz)***	600.17
Nucleus	1H	***Number of Transients***	8	***Origin***	ECA 600	***Original Points Count***	32768
Owner	delta						
Points Count	32768	***Pulse Sequence***	single_pulse.ex2	***Receiver Gain***	44.00	***Solvent***	CHLOROFORM-d
Spectrum Offset (Hz)	5410.6274	***Sweep Width (Hz)***	16534.39	***Temperature (degree C)***	20.600		

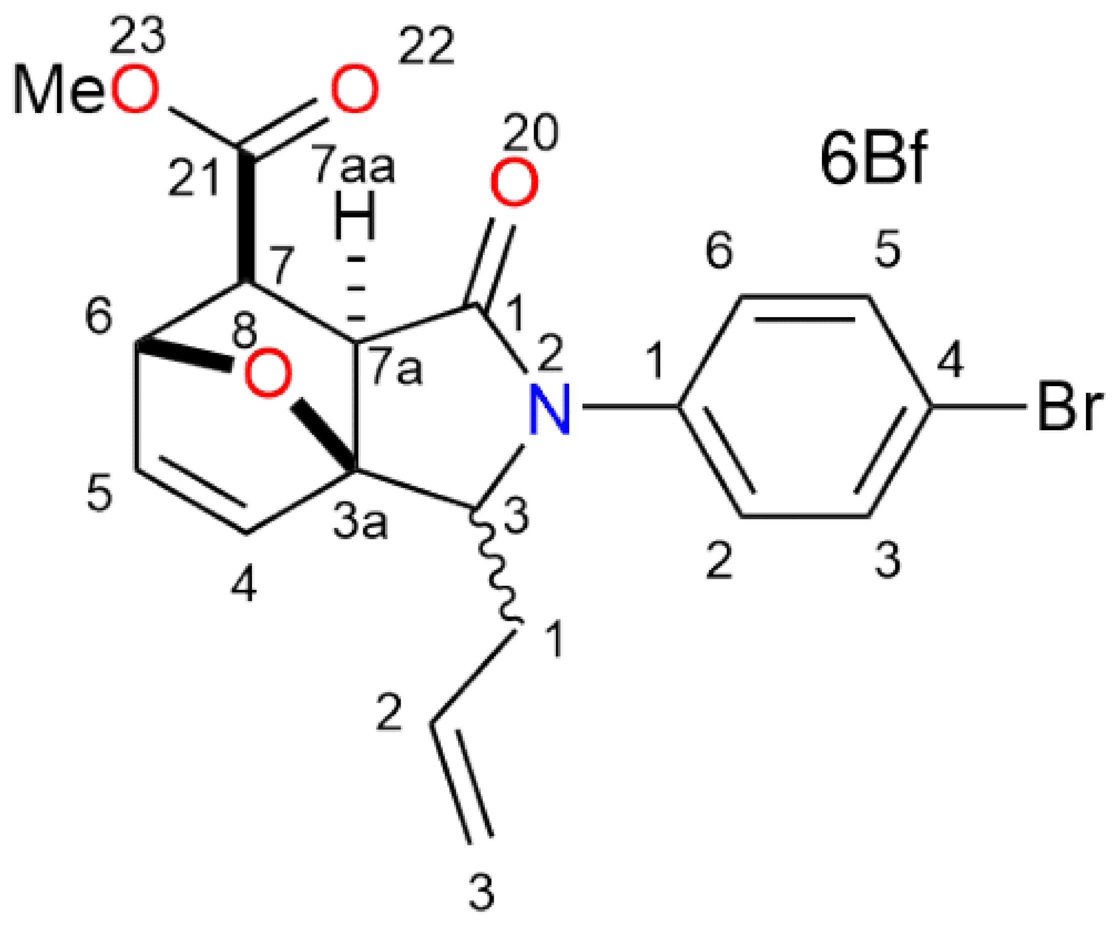

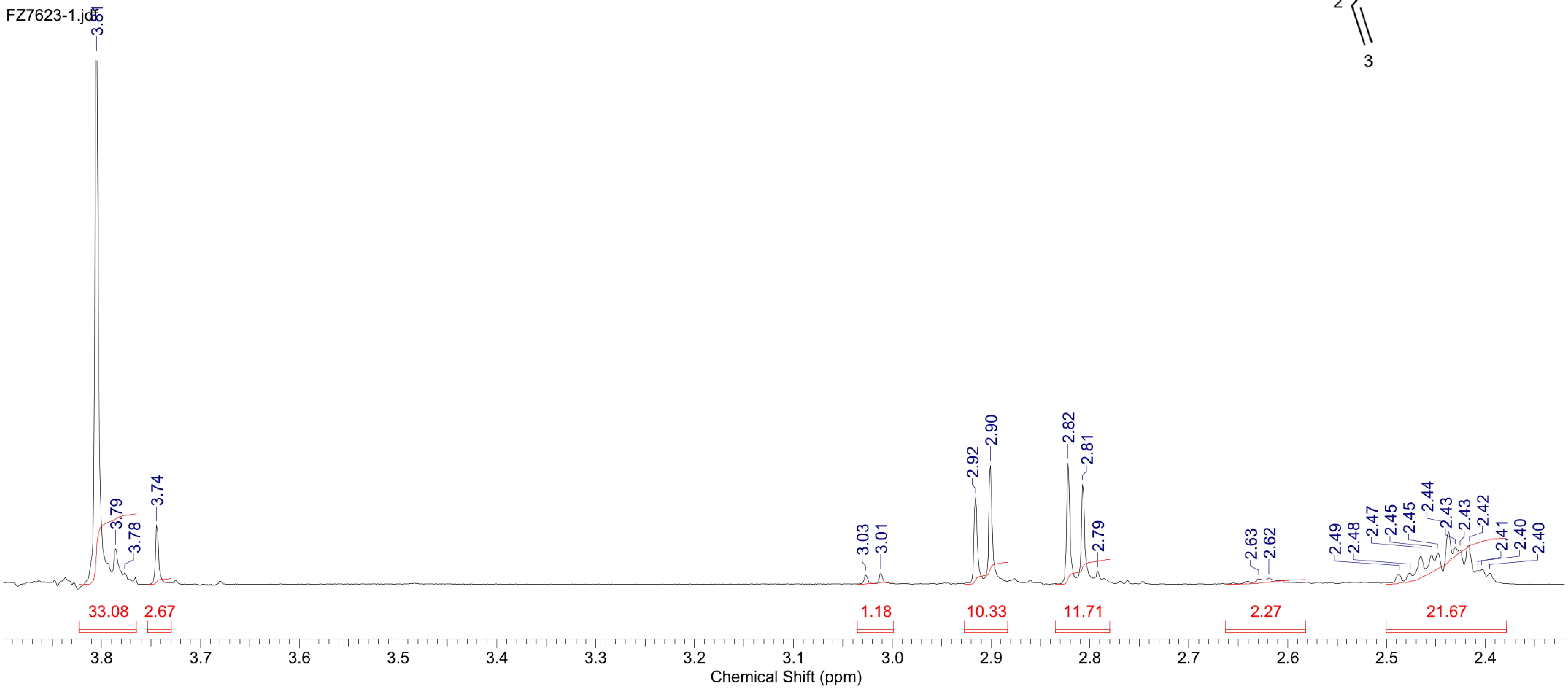

NMR spectrum data for compound 6Ap/6Bp

**Formula** $C_{20}H_{20}BrNO_4$	**FW** 418.2811

**Acquisition Time (sec)**	1.9818	**Comment**	single_pulse	**Date**	25 Aug 2020 08:23:33		
**Date Stamp**	25 Aug 2020 08:24:48			**File Name**	C:\USERS\лаба 534\DOWNLOADS\FZ9042-1.JDF		
**Frequency (MHz)**	600.17	**Nucleus**	1H	**Number of Transients**	8	**Origin**	ECA 600
**Original Points Count**	32768	**Owner**	CKP	**Points Count**	32768	**Pulse Sequence**	single_pulse.ex2
**Receiver Gain**	50.00	**Solvent**	CHLOROFORM-d			**Spectrum Offset (Hz)**	5401.5503
**Sweep Width (Hz)**	16534.39						

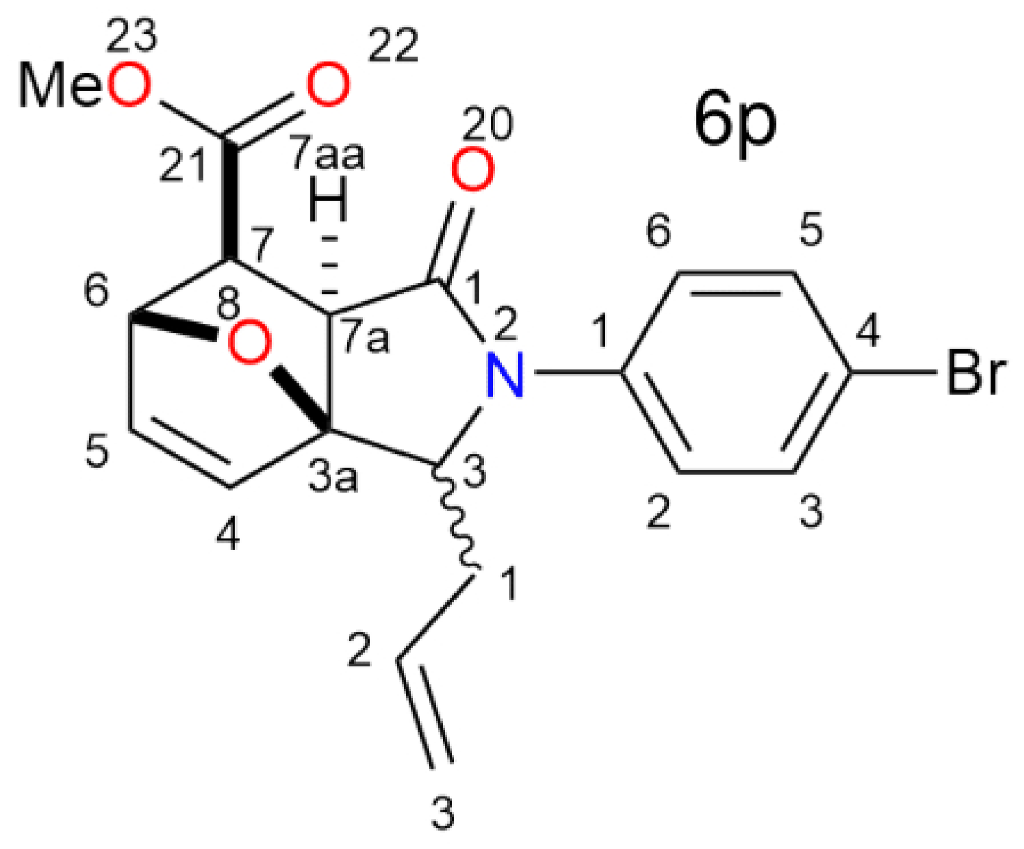

6p

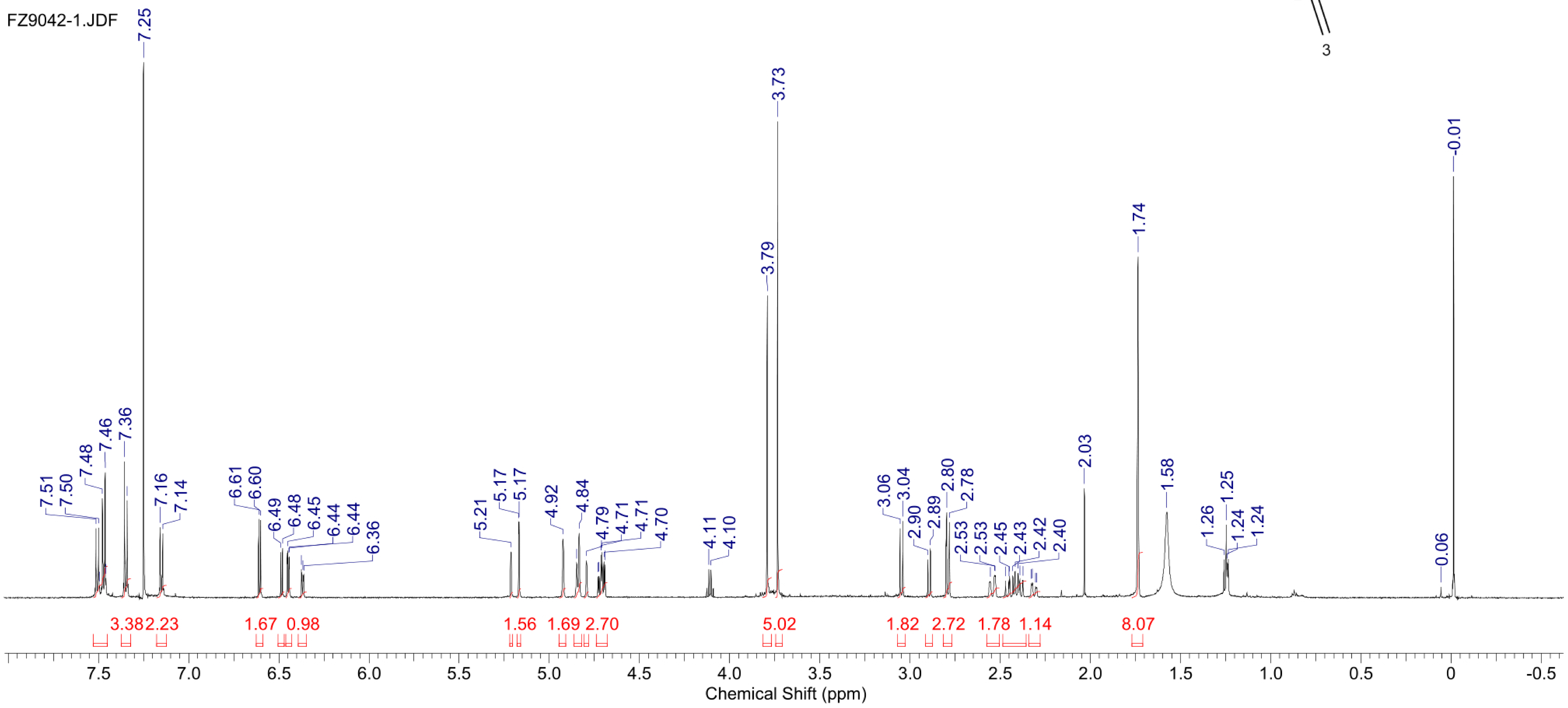

**Formula** $C_{20}H_{20}BrNO_4$	**FW**	418.2811

**Acquisition Time (sec)**	1.9818	**Comment**	single_pulse	**Date**	25 Aug 2020 08:23:33		
**Date Stamp**	25 Aug 2020 08:24:48			**File Name**	C:\USERS\лаба 534\DOWNLOADS\FZ9042-1.JDF		
**Frequency (MHz)**	600.17	**Nucleus**	1H	**Number of Transients**	8	**Origin**	ECA 600
**Original Points Count**	32768	**Owner**	CKP	**Points Count**	32768	**Pulse Sequence**	single_pulse.ex2
**Receiver Gain**	50.00	**Solvent**	CHLOROFORM-d			**Spectrum Offset (Hz)**	5401.5503
**Sweep Width (Hz)**	16534.39						

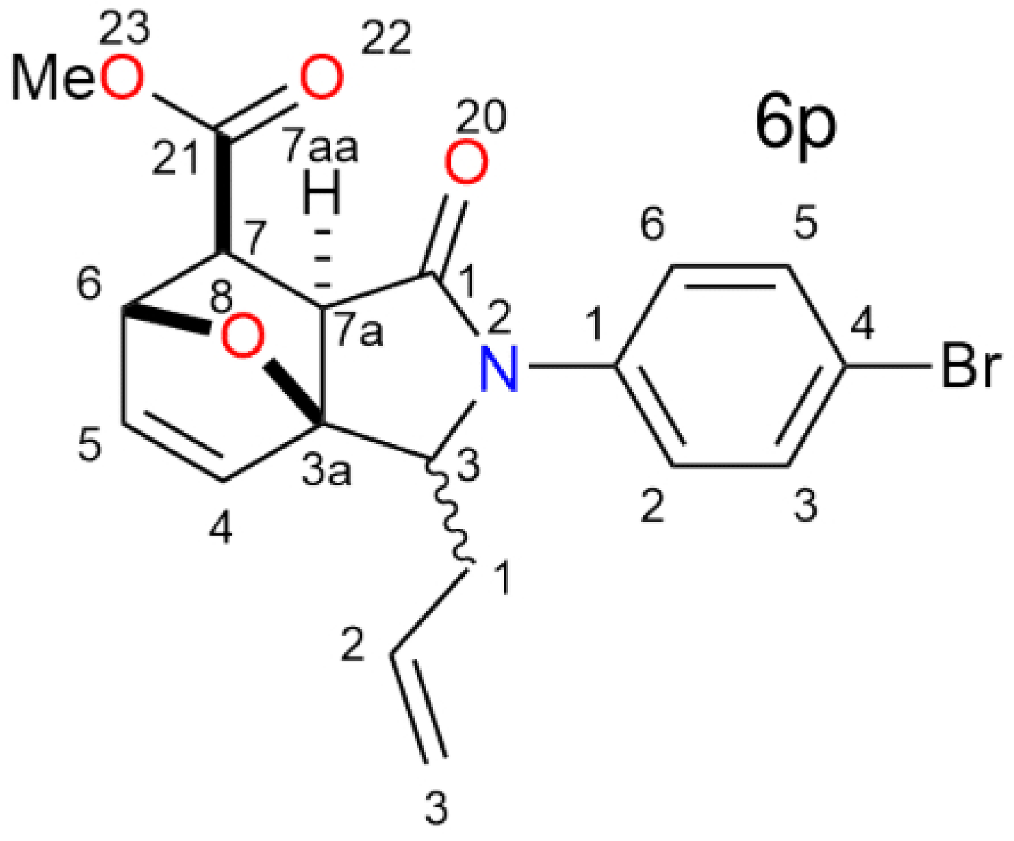

6p

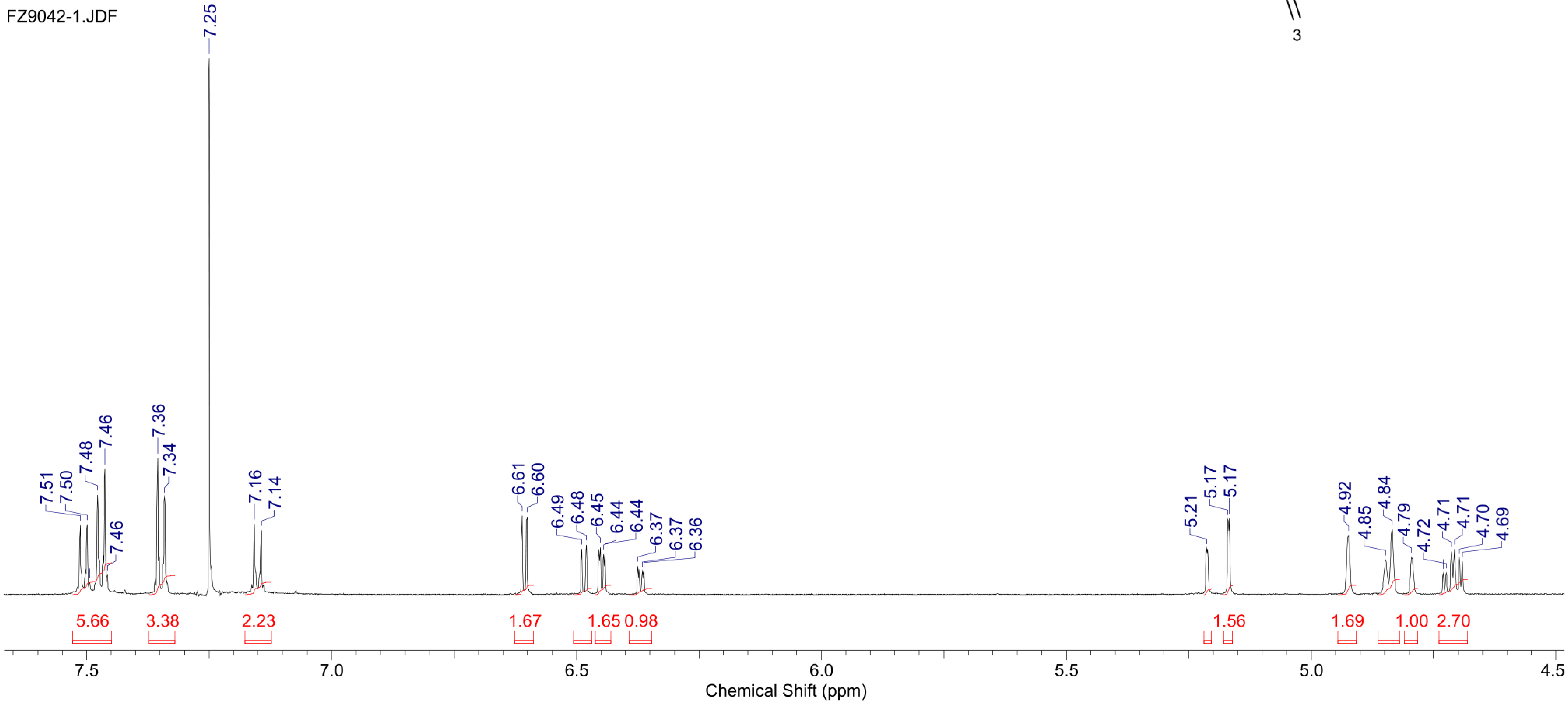

Formula	$C_{20}H_{20}BrNO_4$	FW	418.2811

Acquisition Time (sec)	1.9818	***Comment***	single_pulse	***Date***	25 Aug 2020 08:23:33		
Date Stamp	25 Aug 2020 08:24:48			***File Name***	C:\USERS\лаба 534\DOWNLOADS\FZ9042-1.JDF		
Frequency (MHz)	600.17	***Nucleus***	1H	***Number of Transients***	8	***Origin***	ECA 600
Original Points Count	32768	***Owner***	CKP	***Points Count***	32768	***Pulse Sequence***	single_pulse.ex2
Receiver Gain	50.00	***Solvent***	CHLOROFORM-d			***Spectrum Offset (Hz)***	5401.5503
Sweep Width (Hz)	16534.39						

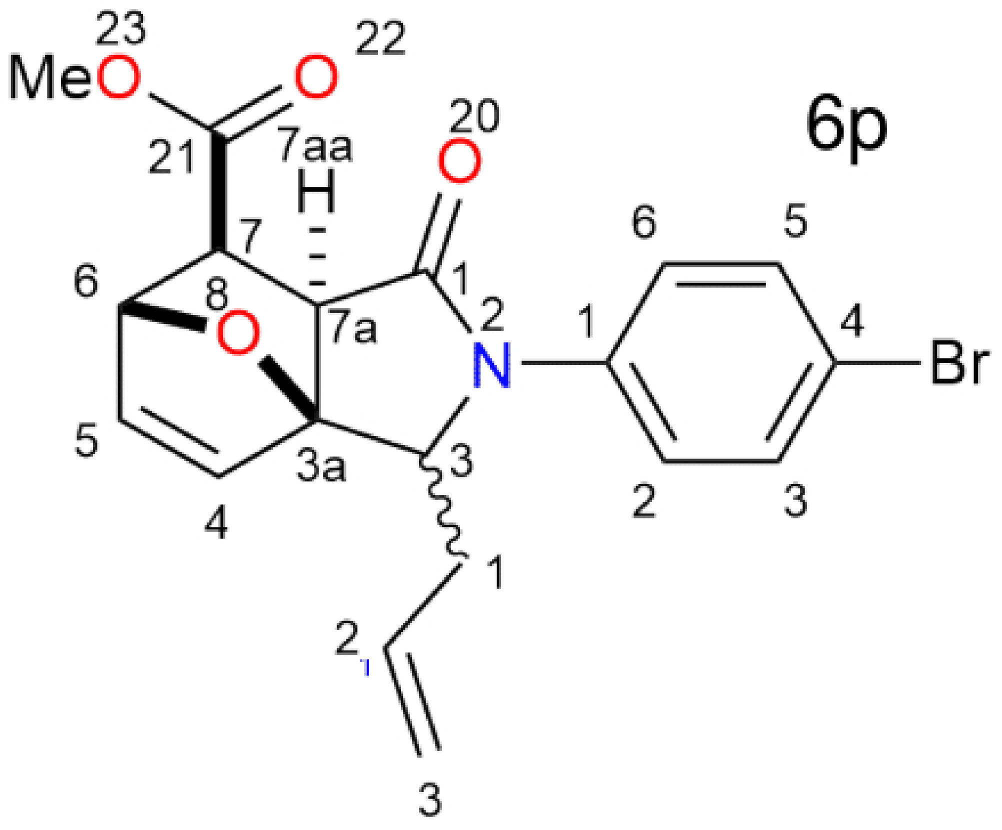

6p

FZ9042-1.JDF

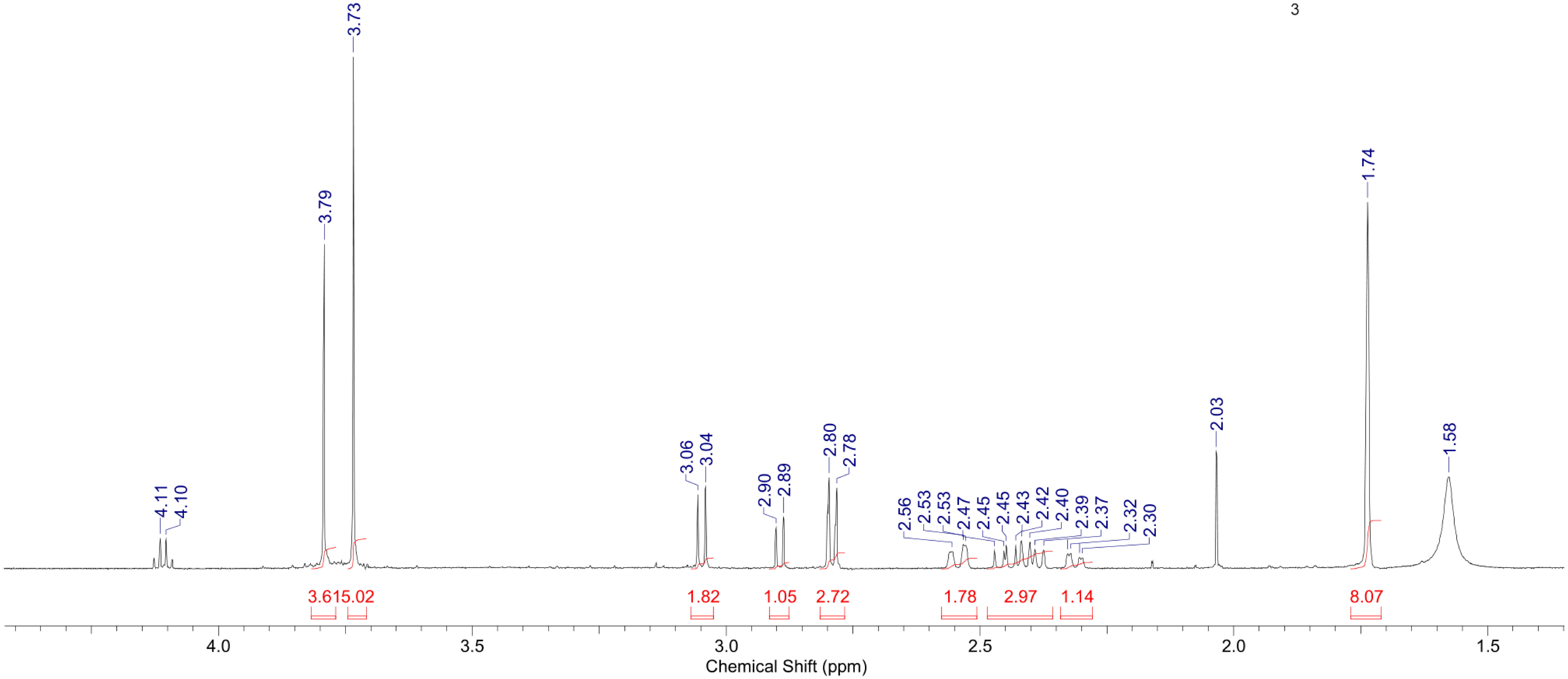

NMR spectrum data for compound 7a

26.11.2019 17:42:51

Formula	$C_{19}H_{19}NO_4$	FW	325.3585

**Acquisition Time (sec)**	1.9818	**Comment**	single_pulse	**Date**	05 Sep 1990 02:19:49		
**Date Stamp**	15 Nov 2019 07:36:58			**File Name**	C:\Users\User\Desktop\05.11.19\FZ8135-1.jdf	**Frequency (MHz)**	600.17
**Nucleus**	1H	**Number of Transients**	8	**Origin**	ECA 600	**Original Points Count** 32768	**Owner** delta
**Points Count**	32768	**Pulse Sequence**	single_pulse.ex2			**Receiver Gain** 38.00	**Solvent** CHLOROFORM-d
**Spectrum Offset (Hz)**	5410.1226	**Sweep Width (Hz)**	16534.39	**Temperature (degree C)**	22.100		

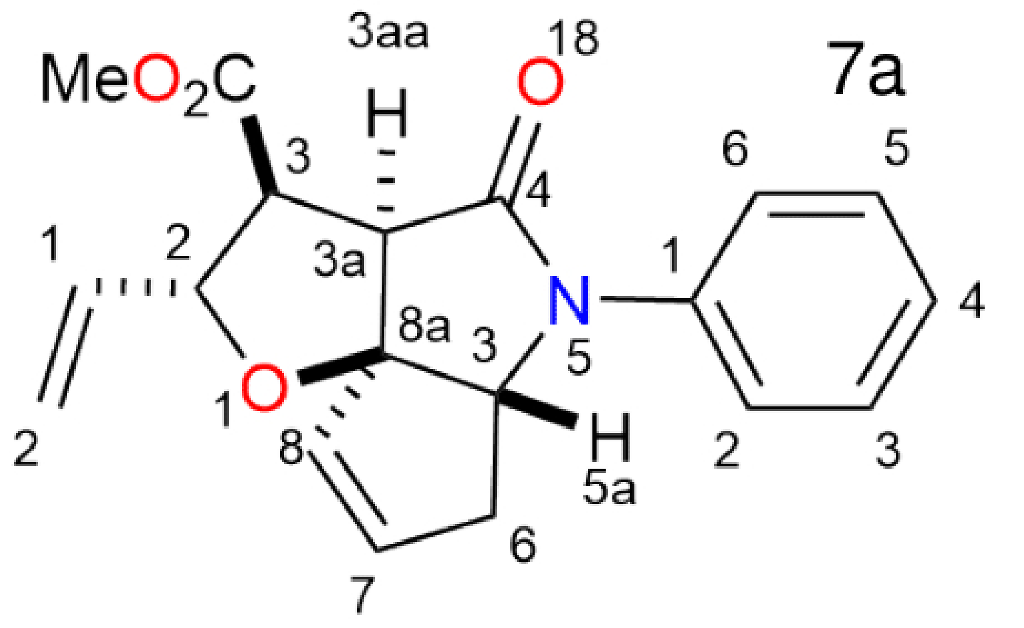

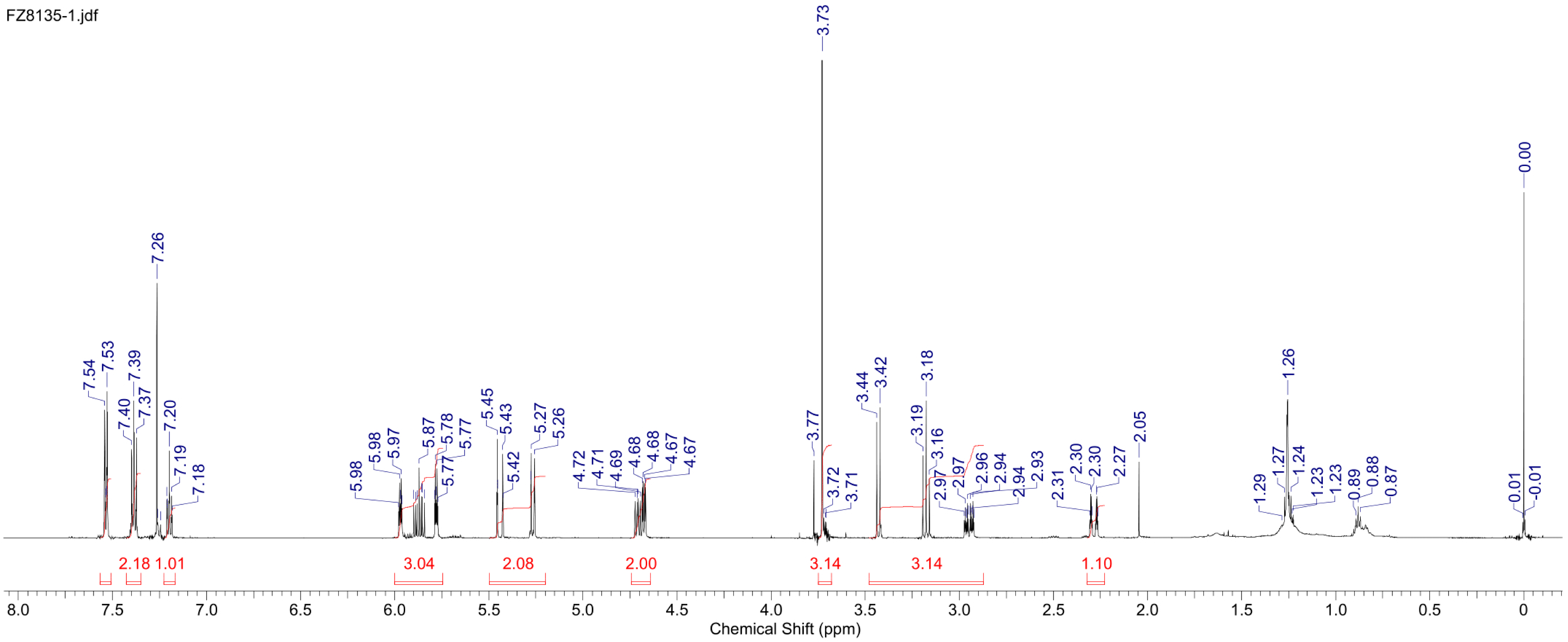

26.11.2019 17:43:09

Formula	$C_{19}H_{19}NO_4$	FW	325.3585

Acquisition Time (sec)	1.9818	Comment	single_pulse	Date	05 Sep 1990 02:19:49				
Date Stamp	15 Nov 2019 07:36:58			File Name	C:\Users\User\Desktop\05.11.19\FZ8135-1.jdf			Frequency (MHz)	600.17
Nucleus	1H	Number of Transients	8	Origin	ECA 600	Original Points Count	32768	Owner	delta
Points Count	32768	Pulse Sequence	single_pulse.ex2			Receiver Gain	38.00	Solvent	CHLOROFORM-d
Spectrum Offset (Hz)	5410.1226	Sweep Width (Hz)	16534.39	Temperature (degree C)	22.100				

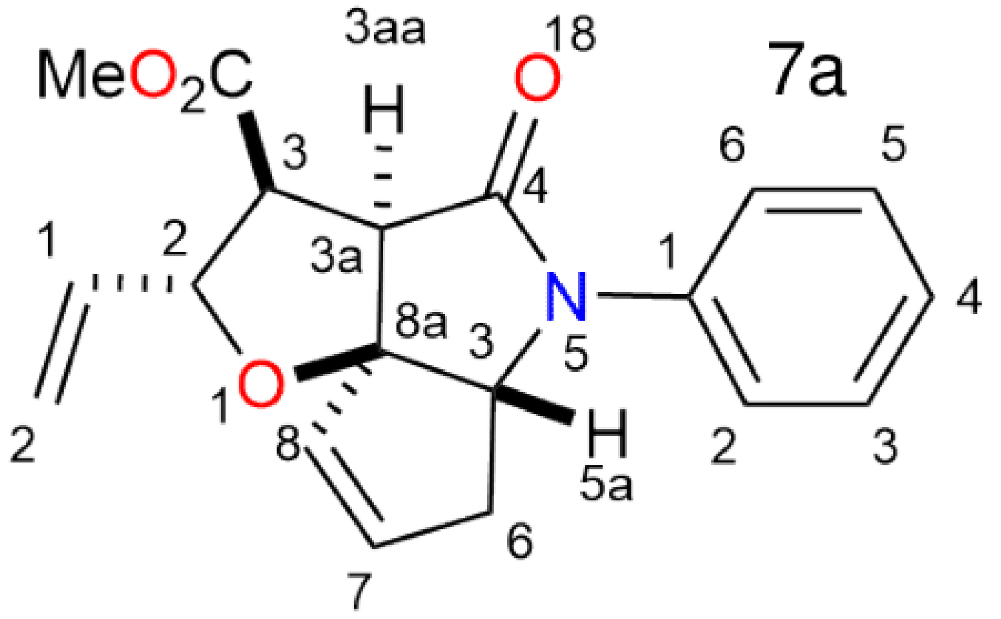

7a

FZ8135-1.jdf

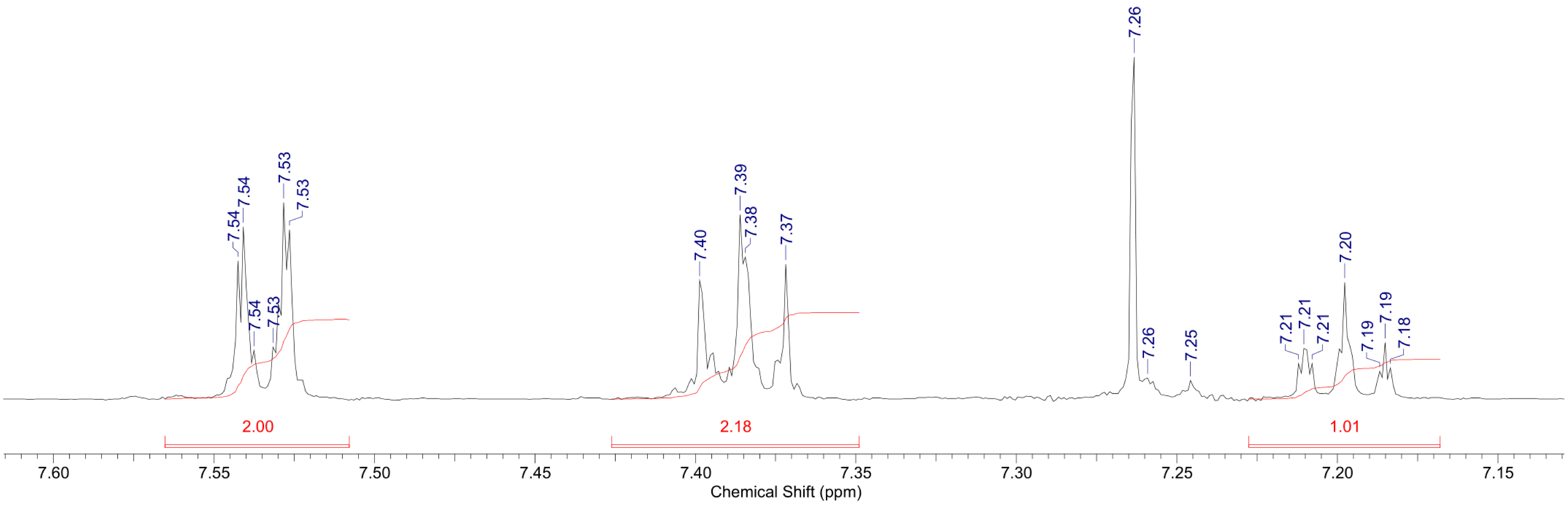

26.11.2019 17:43:34

**Formula** $C_{19}H_{19}NO_4$	**FW** 325.3585

**Acquisition Time (sec)**	1.9818	**Comment**	single_pulse	**Date**	05 Sep 1990 02:19:49		
**Date Stamp**	15 Nov 2019 07:36:58			**File Name**	C:\Users\User\Desktop\05.11.19\FZ8135-1.jdf	**Frequency (MHz)**	600.17
**Nucleus**	1H	**Number of Transients**	8	**Origin**	ECA 600 **Original Points Count** 32768	**Owner**	delta
**Points Count**	32768	**Pulse Sequence**	single_pulse.ex2		**Receiver Gain** 38.00	**Solvent**	CHLOROFORM-d
**Spectrum Offset (Hz)**	5410.1226	**Sweep Width (Hz)**	16534.39	**Temperature (degree C)**	22.100		

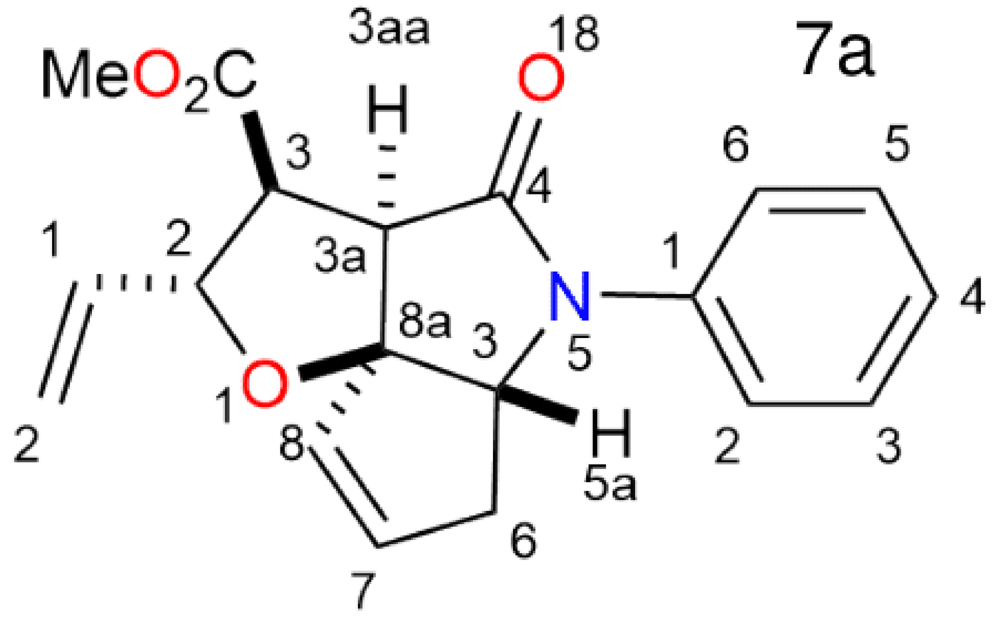

FZ8135-1.jdf

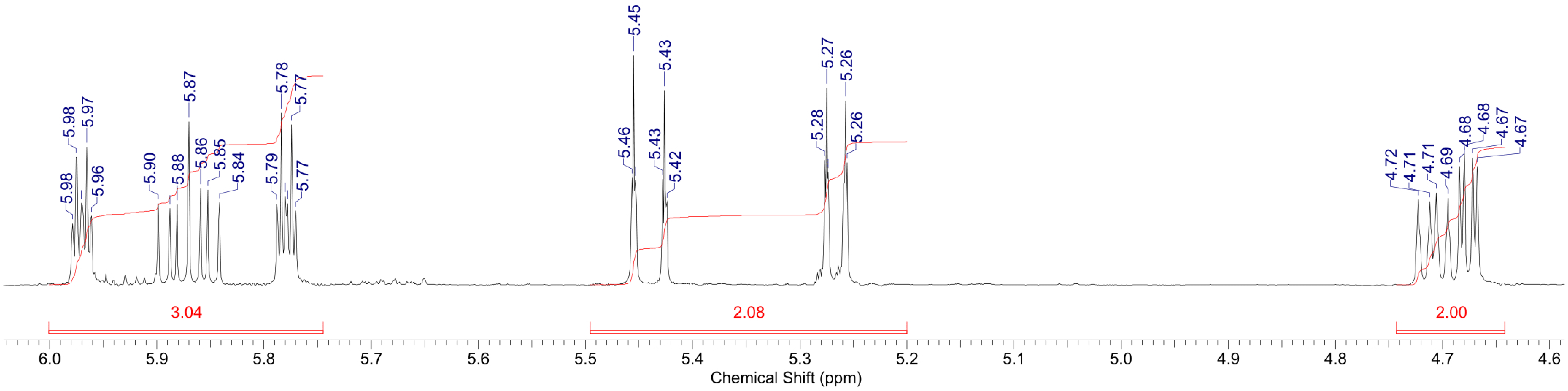

26.11.2019 17:44:00

**Formula**	$C_{19}H_{19}NO_4$	**FW**	325.3585				
**Acquisition Time (sec)**	1.9818	**Comment**	single_pulse	**Date**	05 Sep 1990 02:19:49		
**Date Stamp**	15 Nov 2019 07:36:58			**File Name**	C:\Users\User\Desktop\05.11.19\FZ8135-1.jdf	**Frequency (MHz)**	600.17
**Nucleus**	1H	**Number of Transients**	8	**Origin**	ECA 600	**Original Points Count**	32768
**Owner**	delta						
**Points Count**	32768	**Pulse Sequence**	single_pulse.ex2	**Receiver Gain**	38.00	**Solvent**	CHLOROFORM-d
**Spectrum Offset (Hz)**	5410.1226	**Sweep Width (Hz)**	16534.39	**Temperature (degree C)**	22.100		

7a

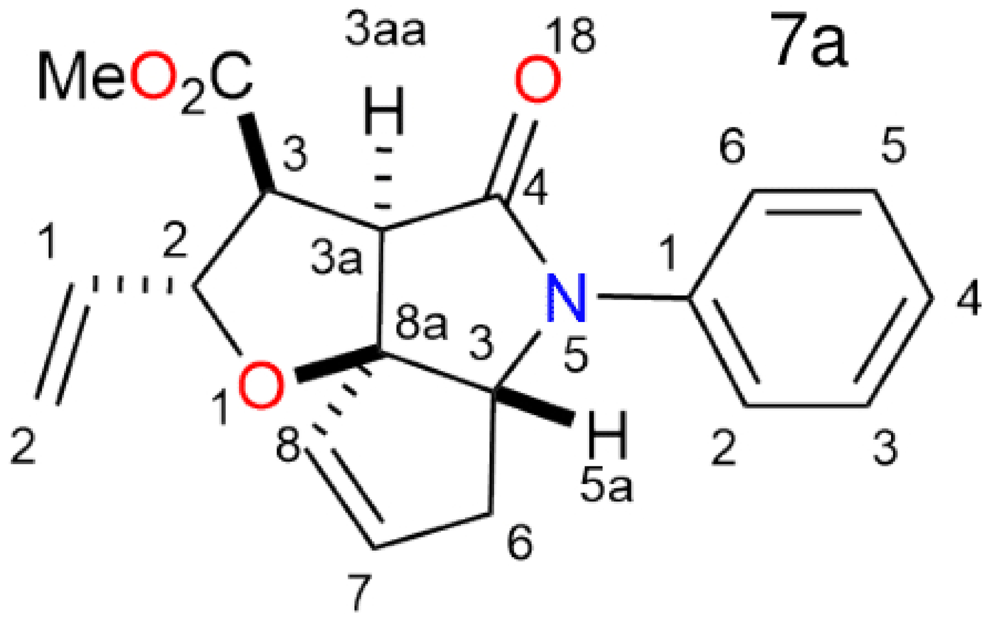

FZ8135-1.jdf

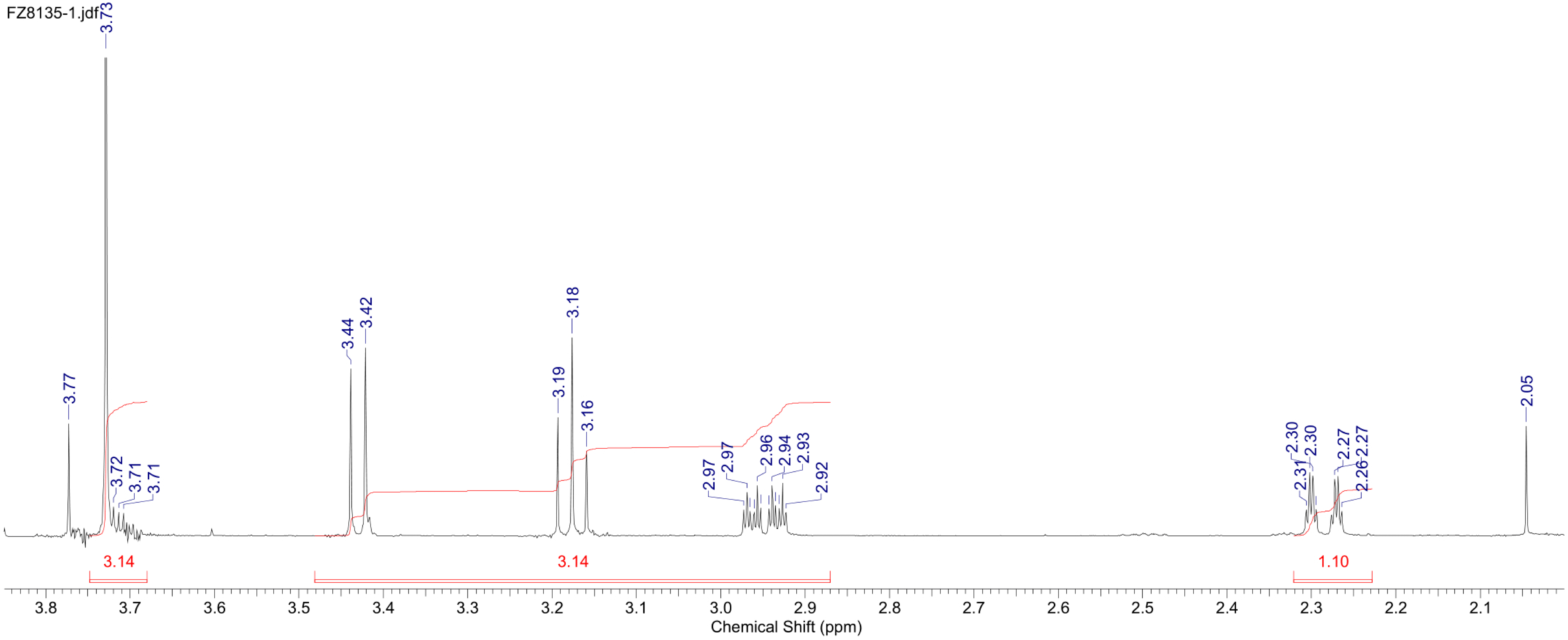

Formula $C_{19}H_{19}NO_4$ | **FW** 325.3585

Acquisition Time (sec)	0.6921	***Comment***	single pulse decoupled gated NOE			***Date***	09 Feb 2020 18:11:16
Date Stamp	22 Feb 2020 01:44:44			***File Name***	C:\USERS\лаба 534\DESKTOP\Саша\ЯМР\NMR FIDS\FZ8501-1.JDF		
Frequency (MHz)	150.91	***Nucleus***	13C	***Number of Transients***	4000	***Origin***	ECA 600
Original Points Count	32768	***Owner***	CKP	***Points Count***	32768	***Pulse Sequence***	single_pulse_dec
Receiver Gain	56.00	***Solvent***	CHLOROFORM-d			***Spectrum Offset (Hz)***	15079.3525
Sweep Width (Hz)	47348.49	***Temperature (degree C)***	23.900				

7a

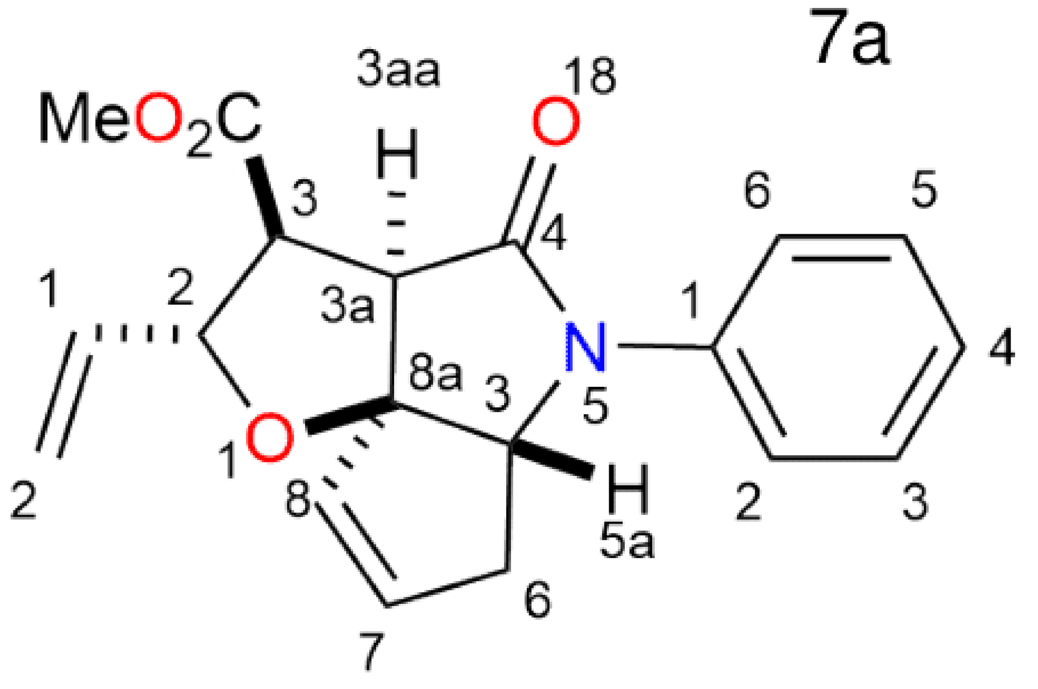

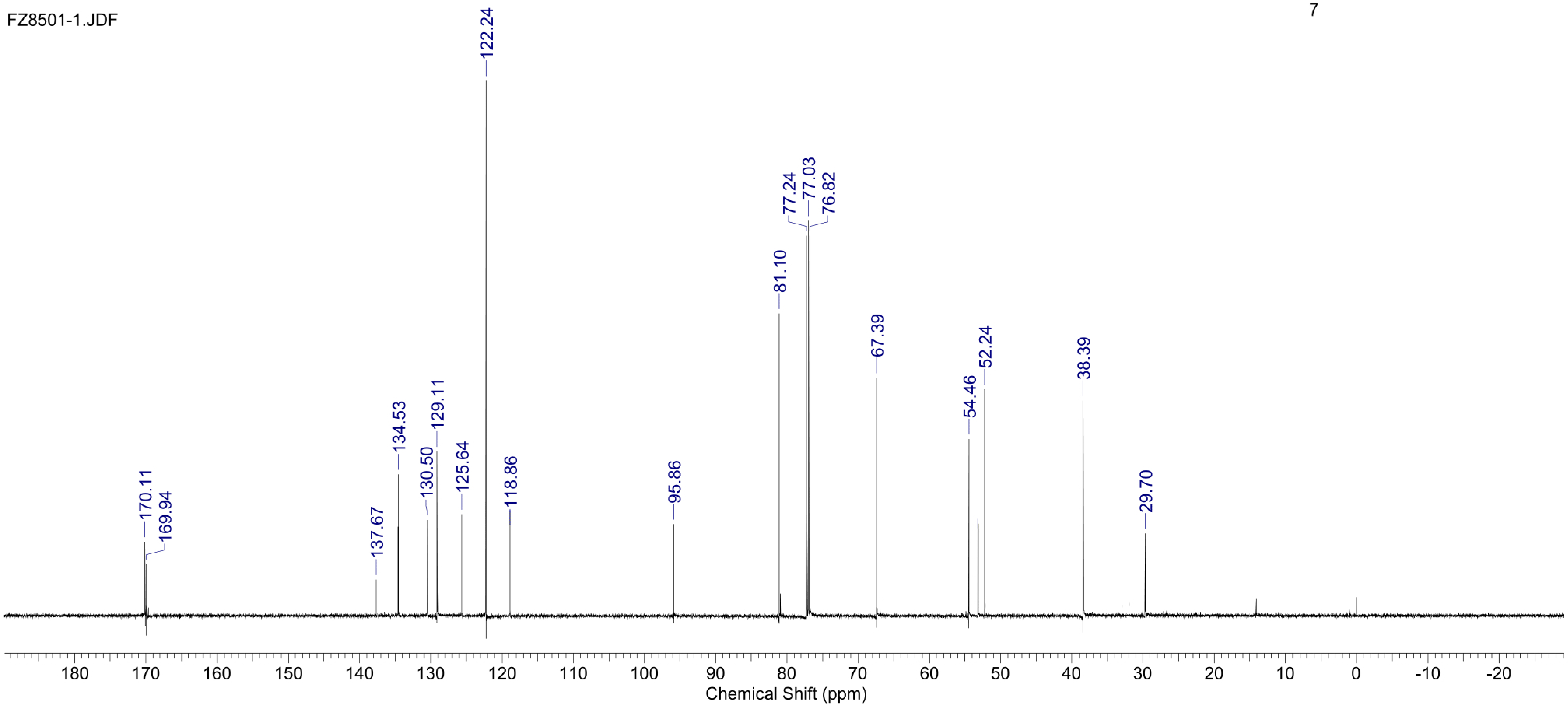

Formula $C_{19}H_{19}NO_4$	FW 325.3585

**Acquisition Time (sec)**	0.6921	**Comment**	single pulse decoupled gated NOE	**Date**	09 Feb 2020 18:11:16
**Date Stamp**	22 Feb 2020 01:44:44	**File Name**	C:\USERS\лаба 534\DESKTOP\Саша\ЯМР\NMR FIDS\FZ8501-1.JDF		
**Frequency (MHz)**	150.91	**Nucleus**	13C	**Number of Transients**	4000
**Origin**	ECA 600	**Original Points Count**	32768	**Owner**	CKP
**Points Count**	32768	**Pulse Sequence**	single_pulse_dec	**Receiver Gain**	56.00
**Solvent**	CHLOROFORM-d	**Spectrum Offset (Hz)**	15079.3525	**Sweep Width (Hz)**	47348.49
**Temperature (degree C)**	23.900				

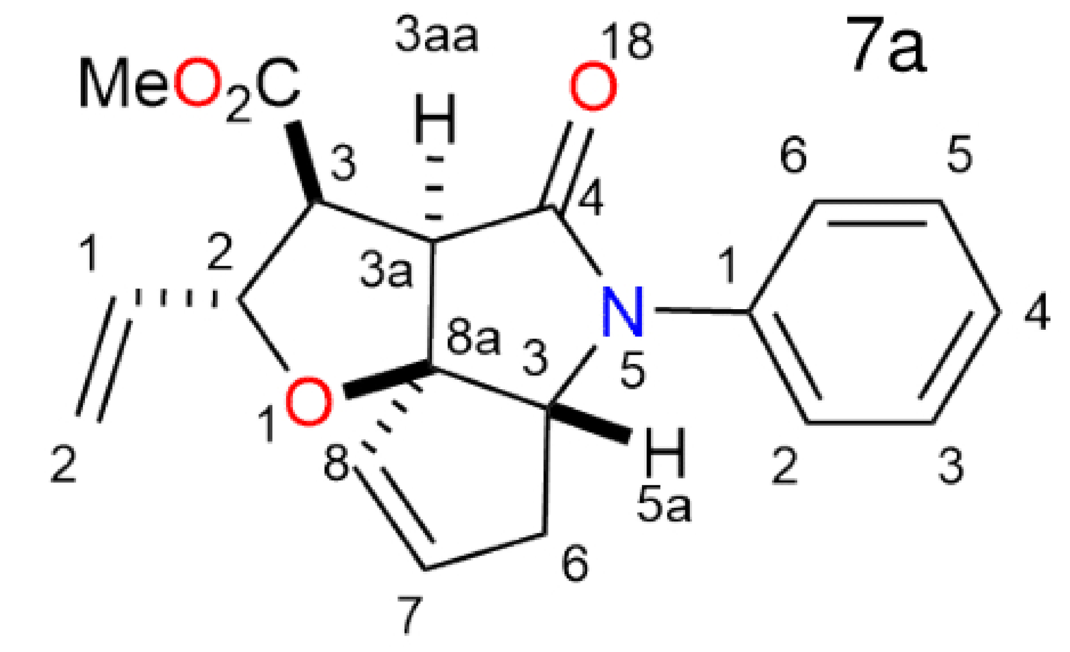

7a

FZ8501-1.JDF

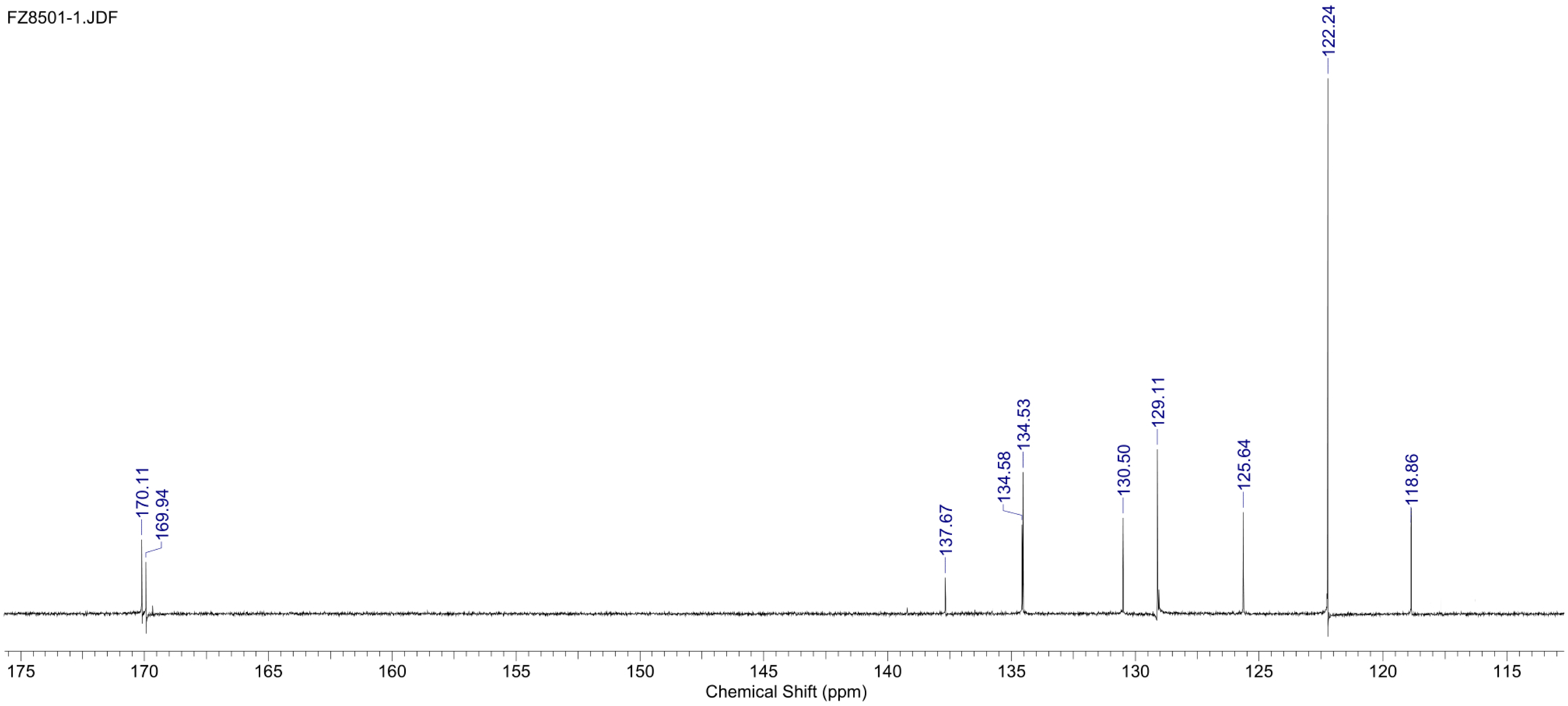

Formula $C_{19}H_{19}NO_4$	FW 325.3585

Acquisition Time (sec)	0.6921	***Comment***	single pulse decoupled gated NOE			***Date***	09 Feb 2020 18:11:16
Date Stamp	22 Feb 2020 01:44:44			***File Name***	C:\USERS\лаба 534\DESKTOP\Саша\ЯМР\NMR FIDS\FZ8501-1.JDF		
Frequency (MHz)	150.91	***Nucleus***	13C	***Number of Transients***	4000	***Origin***	ECA 600
Original Points Count	32768	***Owner***	CKP	***Points Count***	32768	***Pulse Sequence***	single_pulse_dec
Receiver Gain	56.00	***Solvent***	CHLOROFORM-d			***Spectrum Offset (Hz)***	15079.3525
Sweep Width (Hz)	47348.49	***Temperature (degree C)***	23.900				

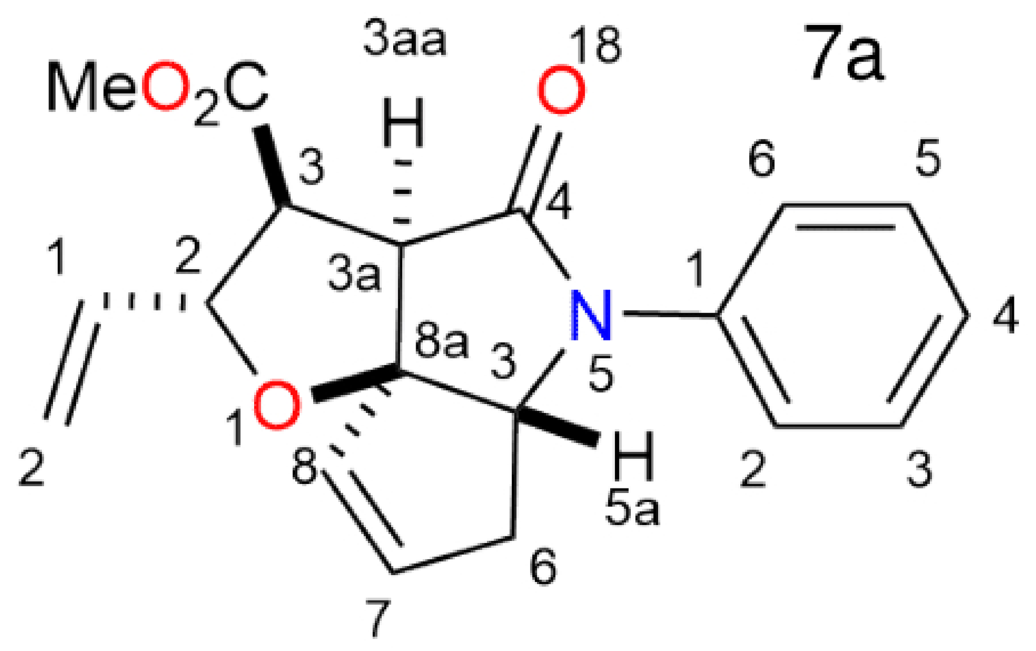

7a

FZ8501-1.JDF

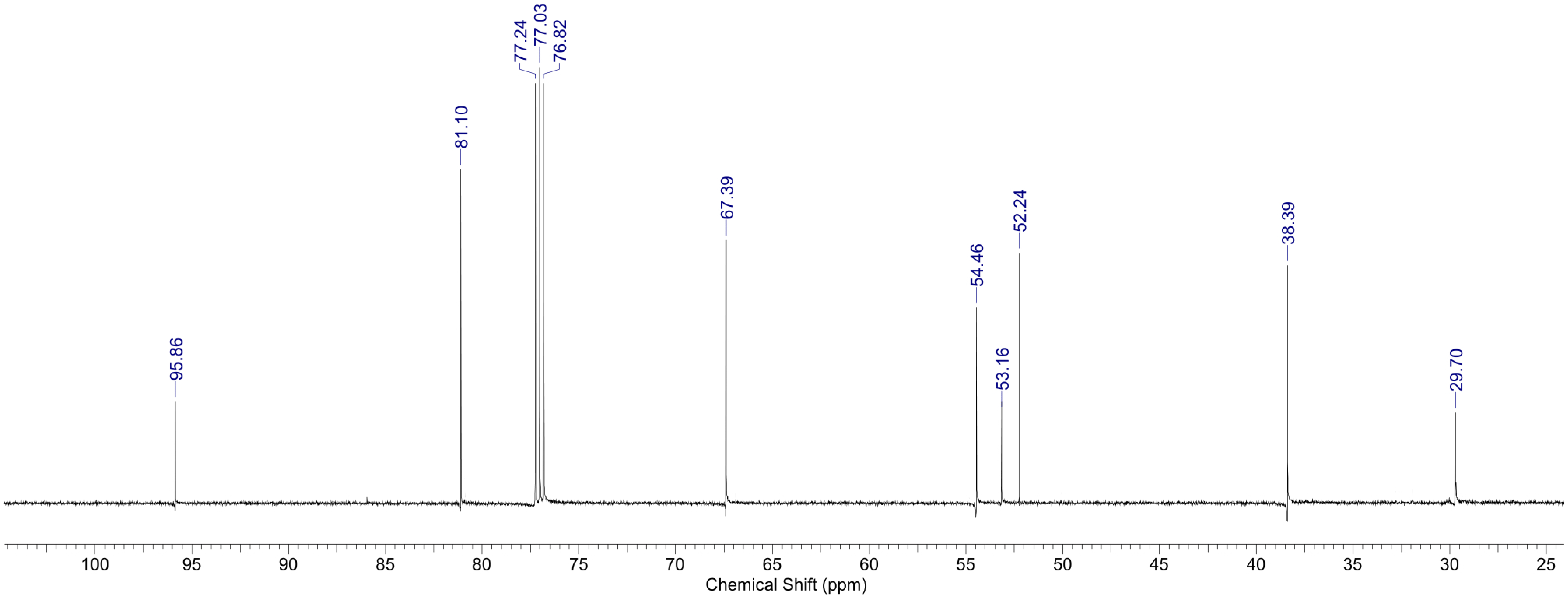

NMR spectrum data for compound 7b

Formula $C_{20}H_{21}NO_4$	FW 339.3850

**Acquisition Time (sec)**	1.9818	**Comment**	single_pulse	**Date**	30 Sep 1990 05:18:40		
**Date Stamp**	10 Dec 2019 10:35:23			**File Name**	C:\Users\User\Desktop\02.12.19\FZ8261-1.jdf	**Frequency (MHz)**	600.17
**Nucleus**	1H	**Number of Transients**	8	**Origin**	ECA 600	**Original Points Count** 32768	**Owner** delta
**Points Count**	32768	**Pulse Sequence**	single_pulse.ex2			**Receiver Gain** 38.00	**Solvent** CHLOROFORM-d
**Spectrum Offset (Hz)**	5409.1133	**Sweep Width (Hz)**	16534.39	**Temperature (degree C)**	23.600		

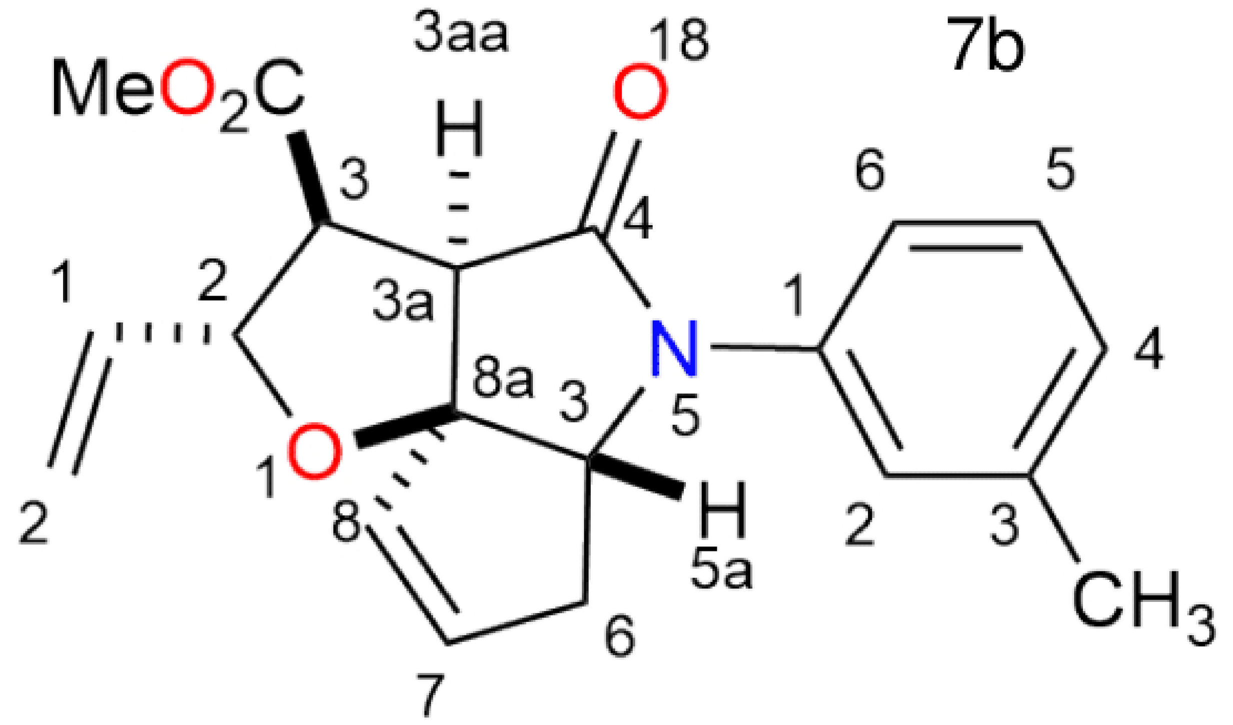

FZ8261-1.esp

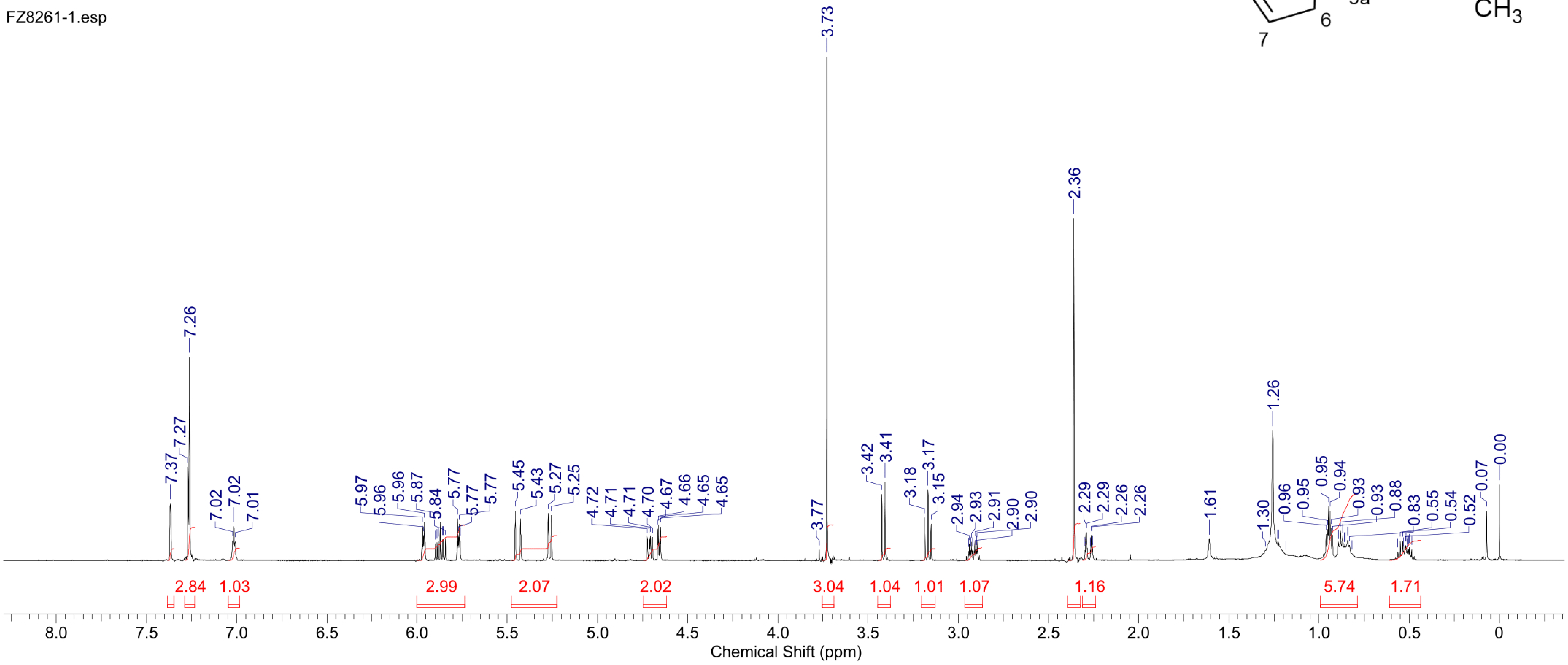

Formula	$C_{20}H_{21}NO_4$	FW	339.3850

**Acquisition Time (sec)**	1.9818	**Comment**	single_pulse	**Date**	30 Sep 1990 05:18:40				
**Date Stamp**	10 Dec 2019 10:35:23			**File Name**	C:\Users\User\Desktop\02.12.19\FZ8261-1.jdf			**Frequency (MHz)**	600.17
**Nucleus**	1H	**Number of Transients**	8	**Origin**	ECA 600	**Original Points Count**	32768	**Owner**	delta
**Points Count**	32768	**Pulse Sequence**	single_pulse.ex2			**Receiver Gain**	38.00	**Solvent**	CHLOROFORM-d
**Spectrum Offset (Hz)**	5409.1133	**Sweep Width (Hz)**	16534.39	**Temperature (degree C)**	23.600				

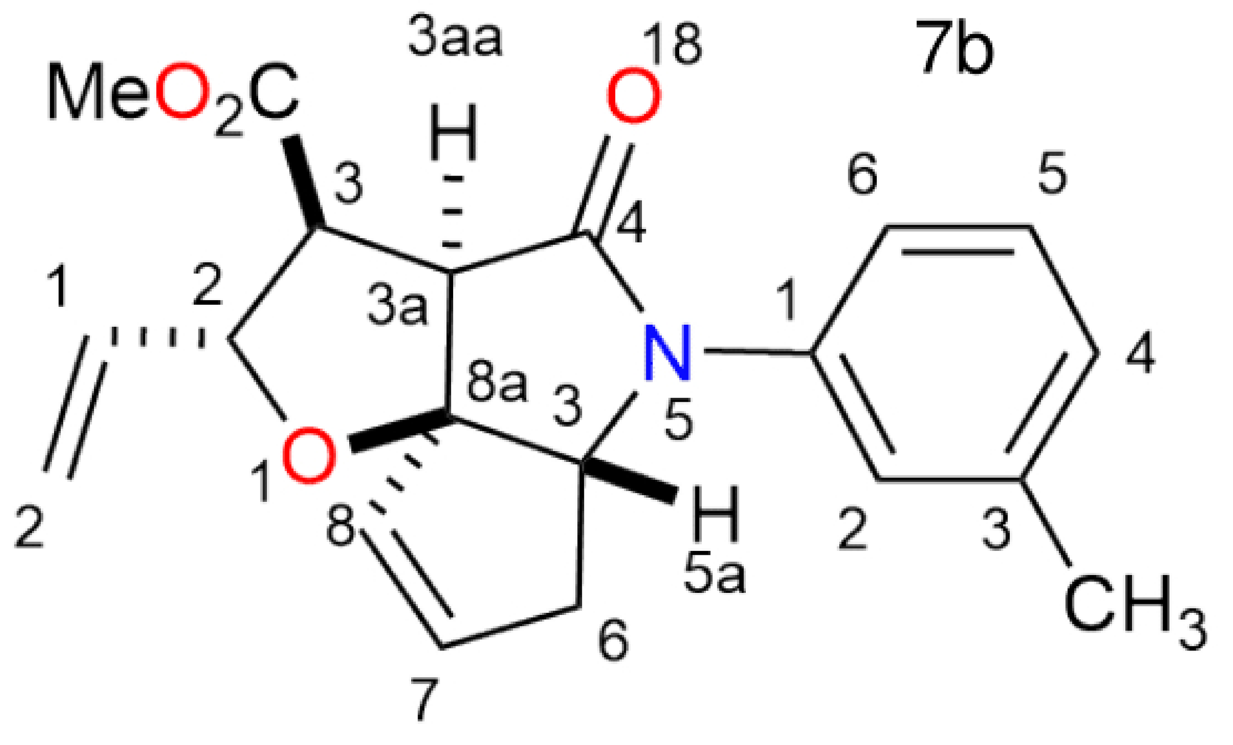

FZ8261-1.esp

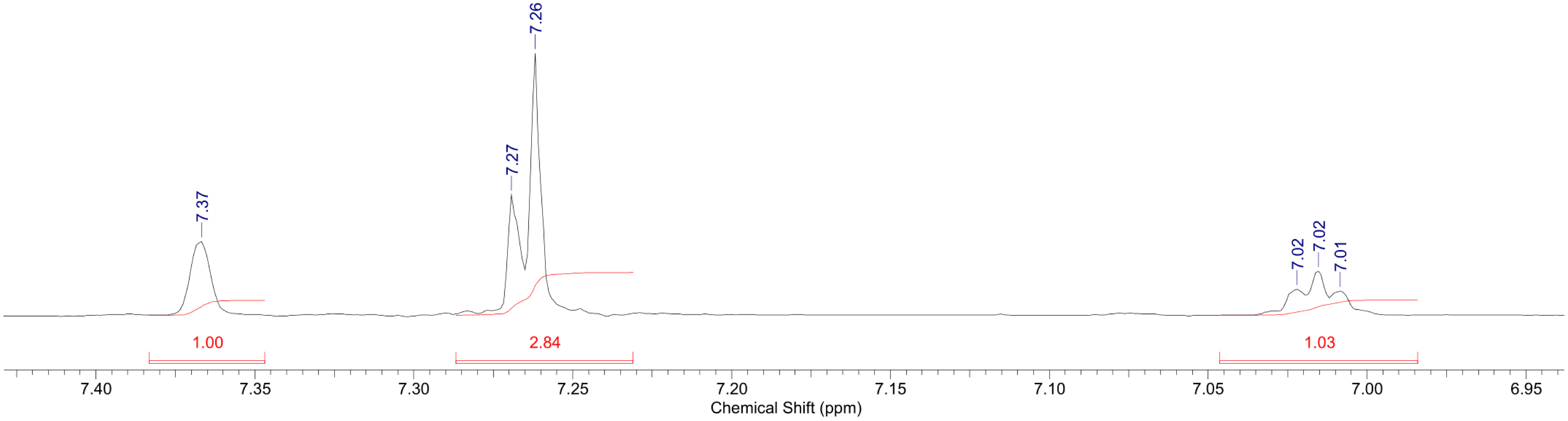

Formula $C_{20}H_{21}NO_4$	FW 339.3850

Acquisition Time (sec)	1.9818	Comment	single_pulse	Date	30 Sep 1990 05:18:40				
Date Stamp	10 Dec 2019 10:35:23			File Name	C:\Users\User\Desktop\02.12.19\FZ8261-1.jdf			Frequency (MHz)	600.17
Nucleus	1H	Number of Transients	8	Origin	ECA 600	Original Points Count	32768	Owner	delta
Points Count	32768	Pulse Sequence	single_pulse.ex2			Receiver Gain	38.00	Solvent	CHLOROFORM-d
Spectrum Offset (Hz)	5409.1133	Sweep Width (Hz)	16534.39	Temperature (degree C)	23.600				

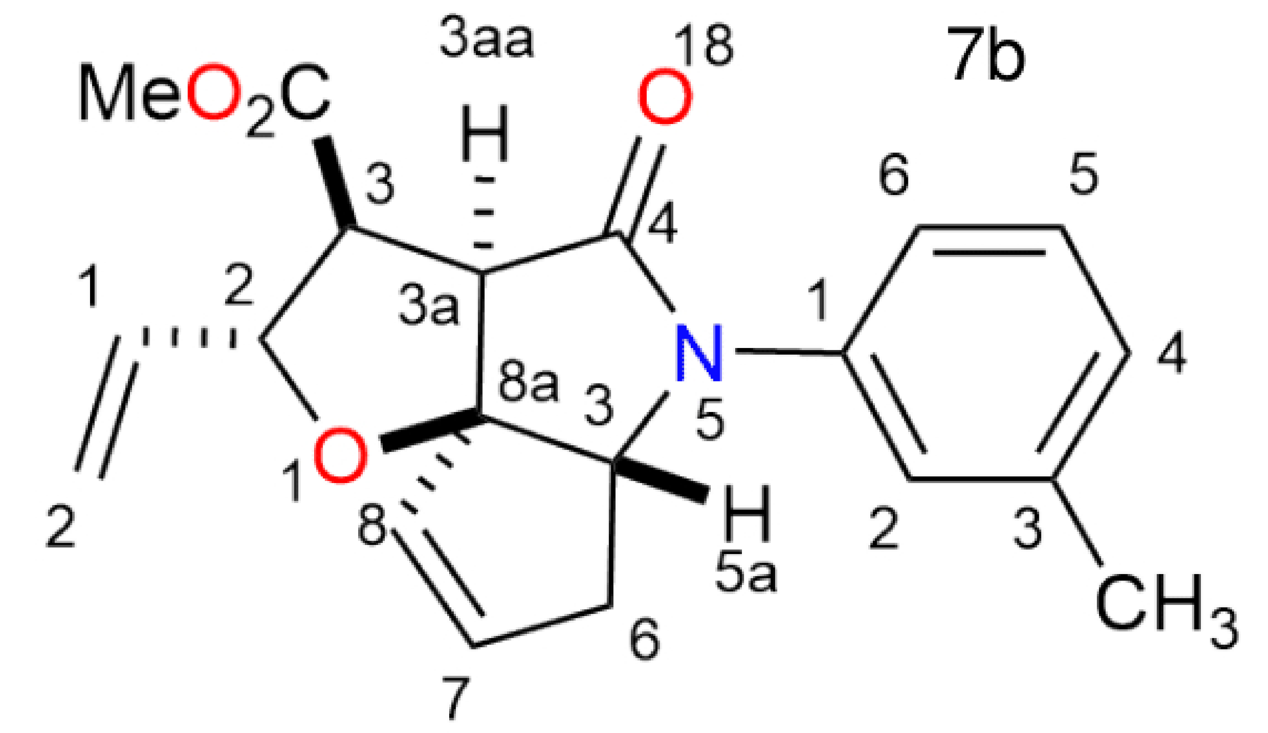

FZ8261-1.esp

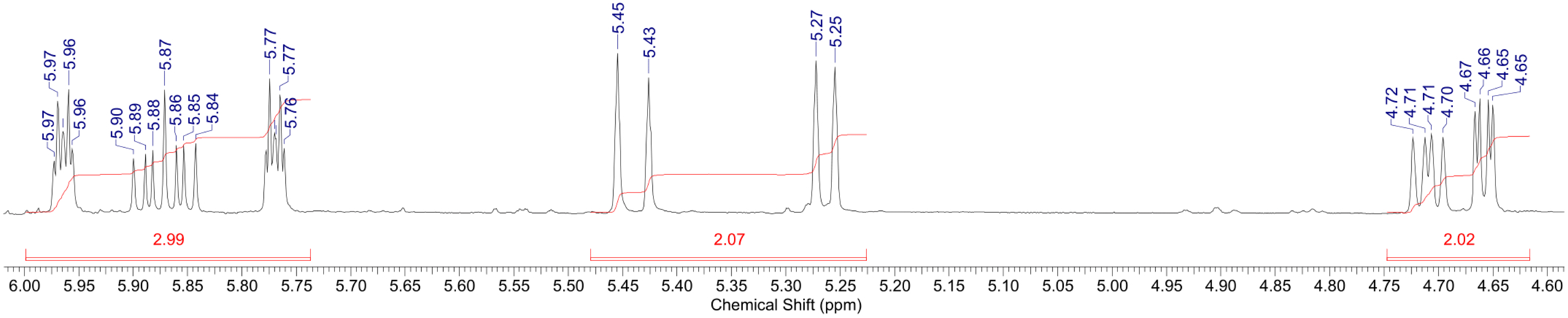

Formula $C_{20}H_{21}NO_4$	FW 339.3850

**Acquisition Time (sec)**	1.9818	**Comment**	single_pulse	**Date**	30 Sep 1990 05:18:40		
**Date Stamp**	10 Dec 2019 10:35:23			**File Name**	C:\Users\User\Desktop\02.12.19\FZ8261-1.jdf	**Frequency (MHz)**	600.17
**Nucleus**	1H	**Number of Transients**	8	**Origin**	ECA 600	**Original Points Count**	32768
**Owner**	delta						
**Points Count**	32768	**Pulse Sequence**	single_pulse.ex2	**Receiver Gain**	38.00	**Solvent**	CHLOROFORM-d
**Spectrum Offset (Hz)**	5409.1133	**Sweep Width (Hz)**	16534.39	**Temperature (degree C)**	23.600		

7b

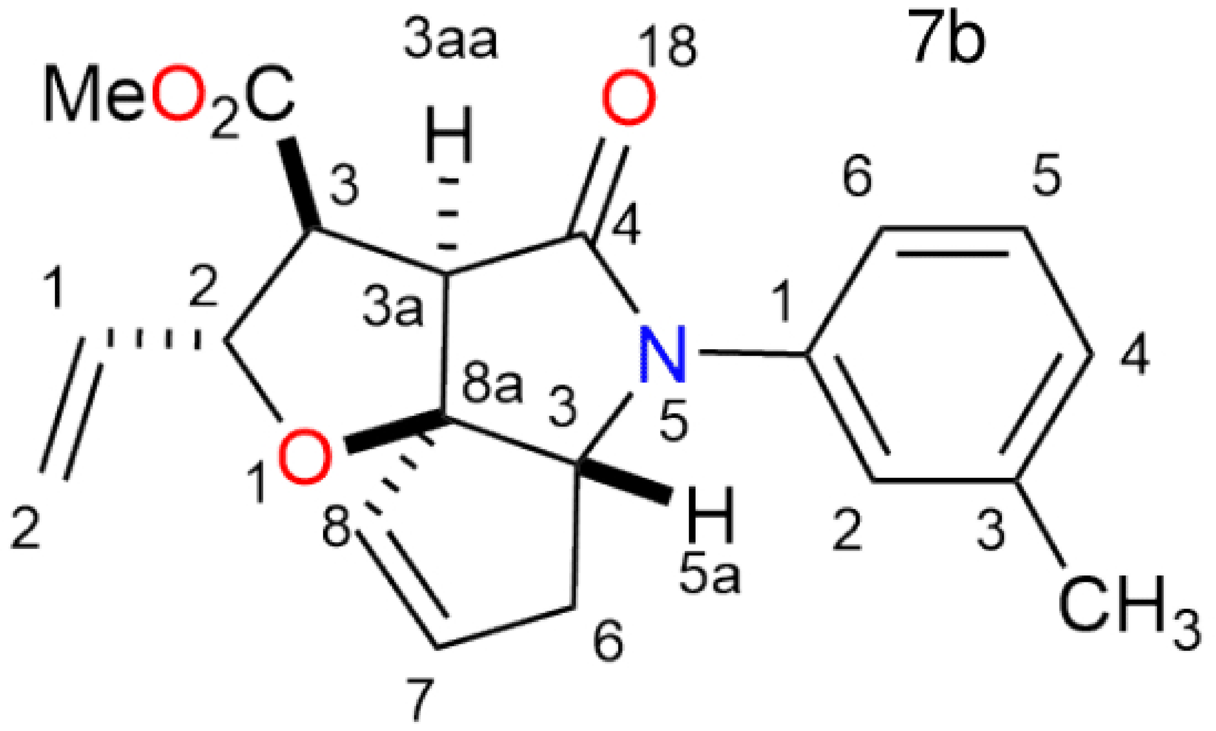

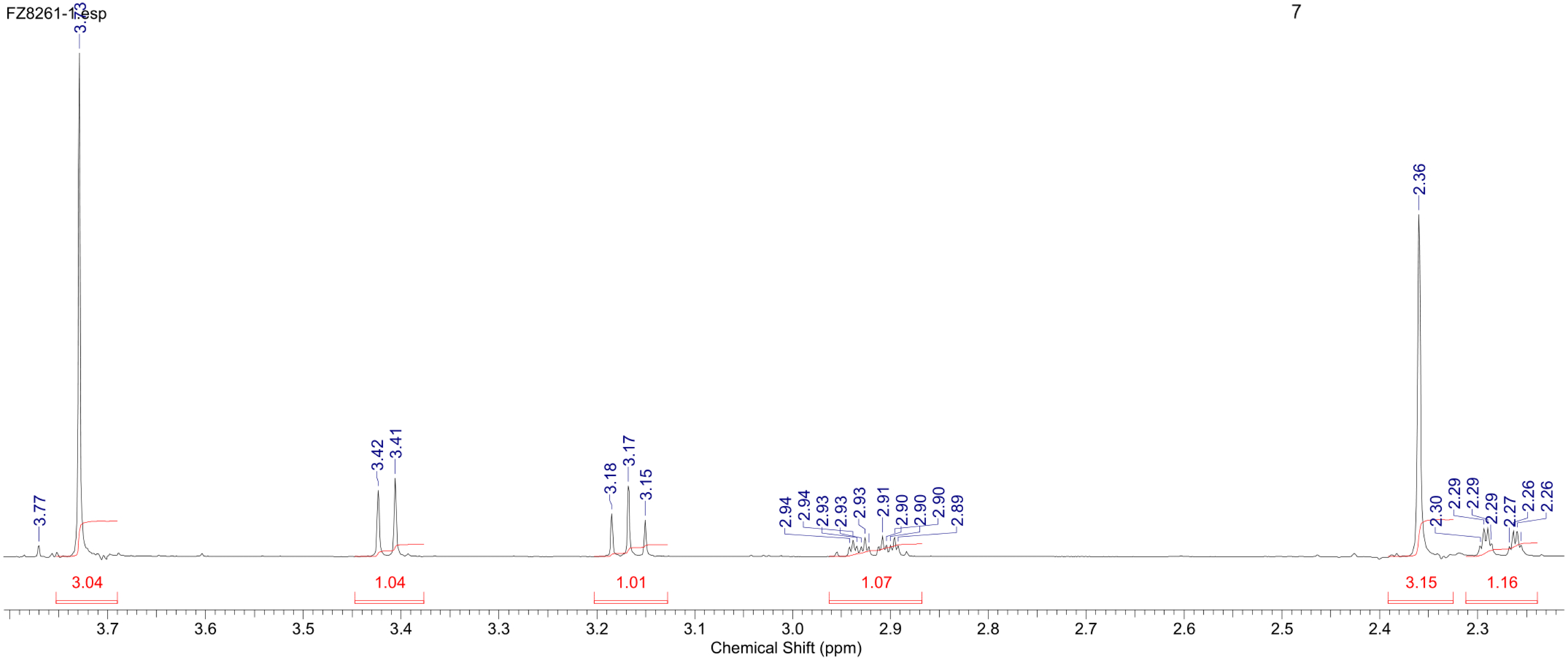

Formula $C_{20}H_{21}NO_4$	FW 339.3850

Acquisition Time (sec)	1.9818	Comment	single_pulse	Date	30 Sep 1990 05:18:40				
Date Stamp	10 Dec 2019 10:35:23			File Name	C:\Users\User\Desktop\02.12.19\FZ8261-1.jdf			Frequency (MHz)	600.17
Nucleus	1H	Number of Transients	8	Origin	ECA 600	Original Points Count	32768	Owner	delta
Points Count	32768	Pulse Sequence	single_pulse.ex2			Receiver Gain	38.00	Solvent	CHLOROFORM-d
Spectrum Offset (Hz)	5409.1133	Sweep Width (Hz)	16534.39	Temperature (degree C)	23.600				

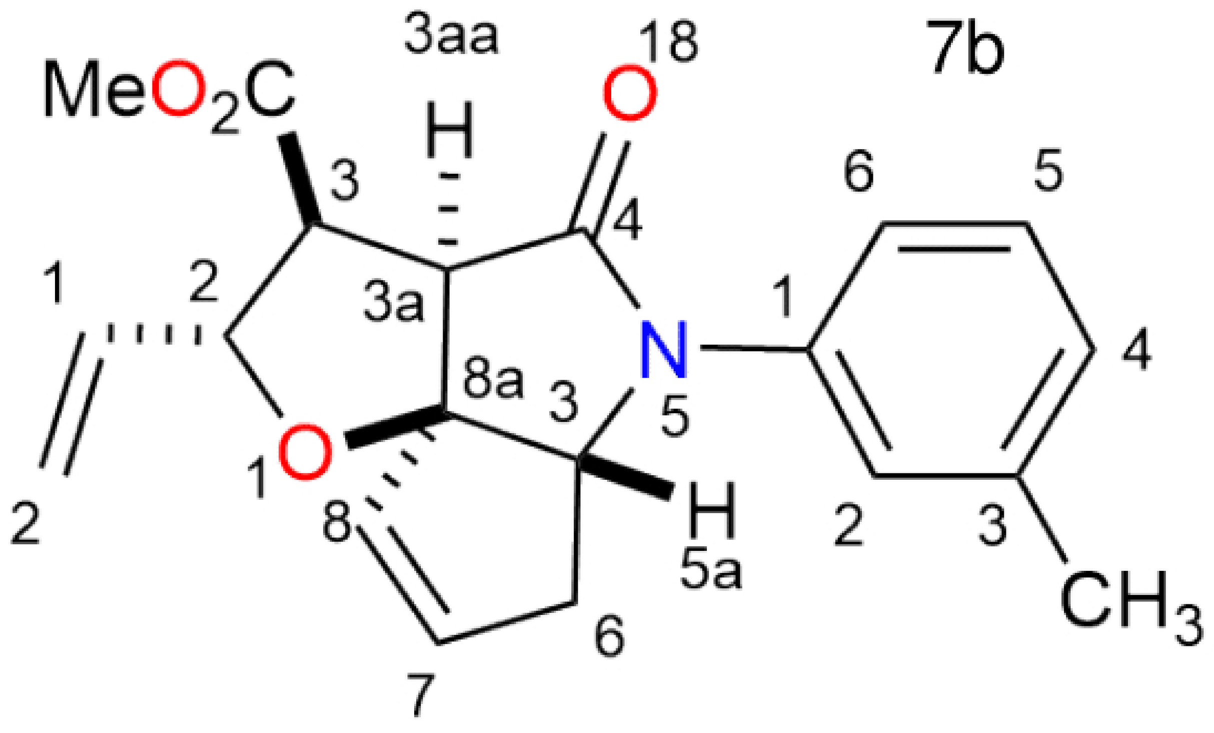

FZ8261-1.esp

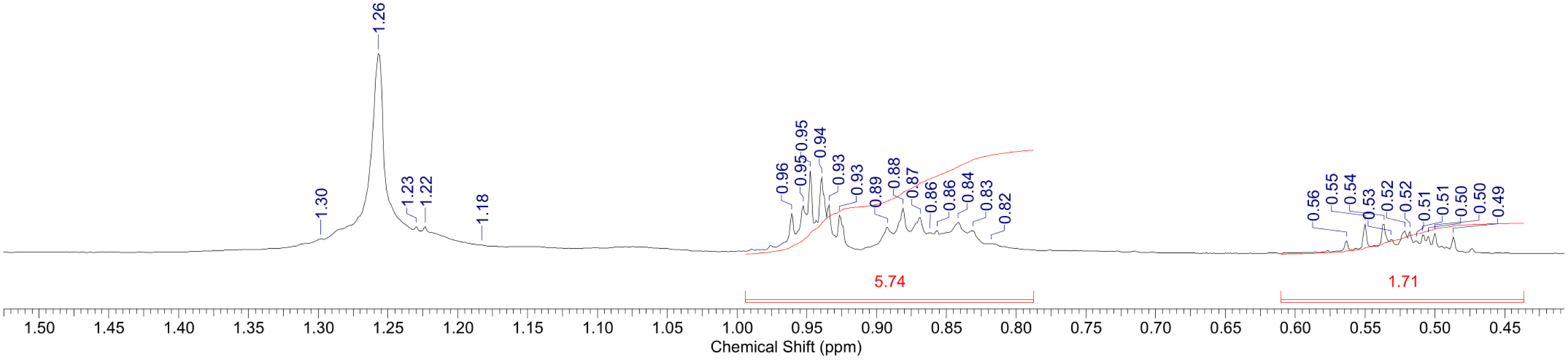

Formula	$C_{20}H_{21}NO_4$	FW	339.3850

**Acquisition Time (sec)**	0.6921	**Comment**	single pulse decoupled gated NOE			**Date**	10 Feb 2020 01:29:38
**Date Stamp**	22 Feb 2020 03:15:21			**File Name**	C:\USERS\лаба 534\DESKTOP\Саша\ЯМР\NMR FIDS\FZ8502-1.JDF		
**Frequency (MHz)**	150.91	**Nucleus**	13C	**Number of Transients**	4000	**Origin**	ECA 600
**Original Points Count**	32768	**Owner**	CKP	**Points Count**	32768	**Pulse Sequence**	single_pulse_dec
**Receiver Gain**	54.00	**Solvent**	CHLOROFORM-d			**Spectrum Offset (Hz)**	15091.3428
**Sweep Width (Hz)**	47348.49	**Temperature (degree C)**	24.000				

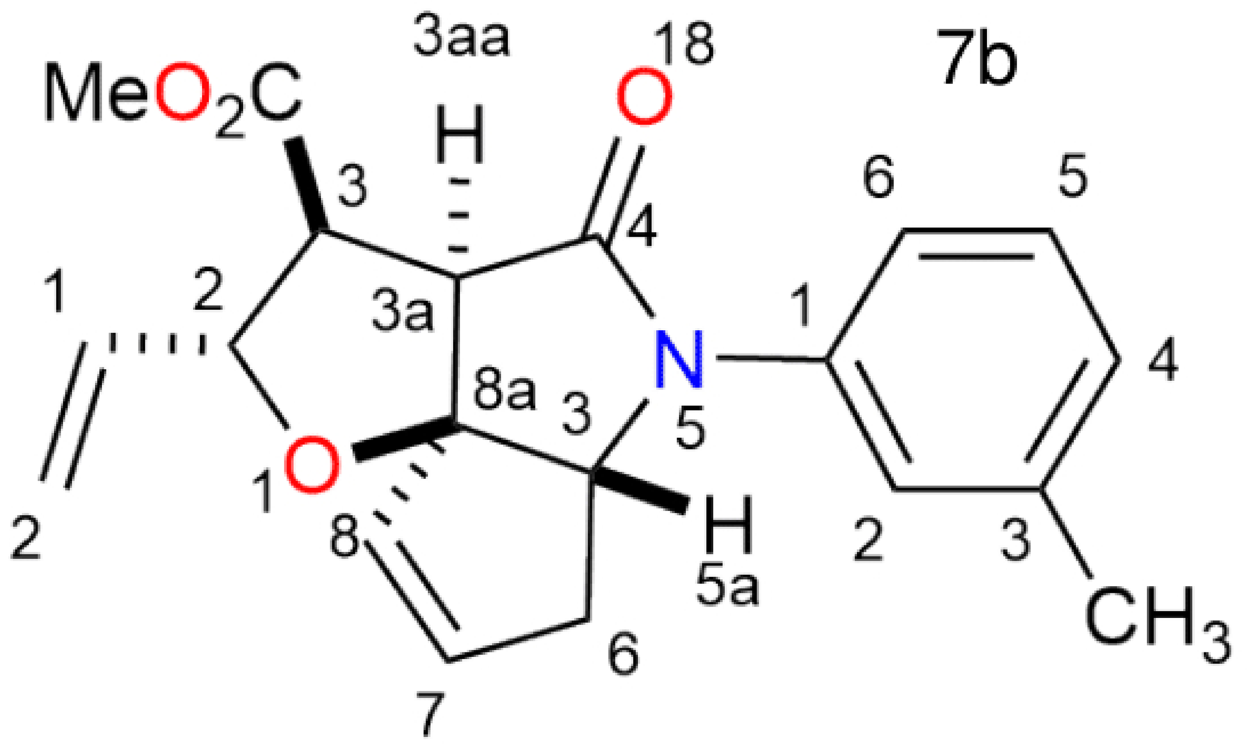

FZ8502-1.JDF

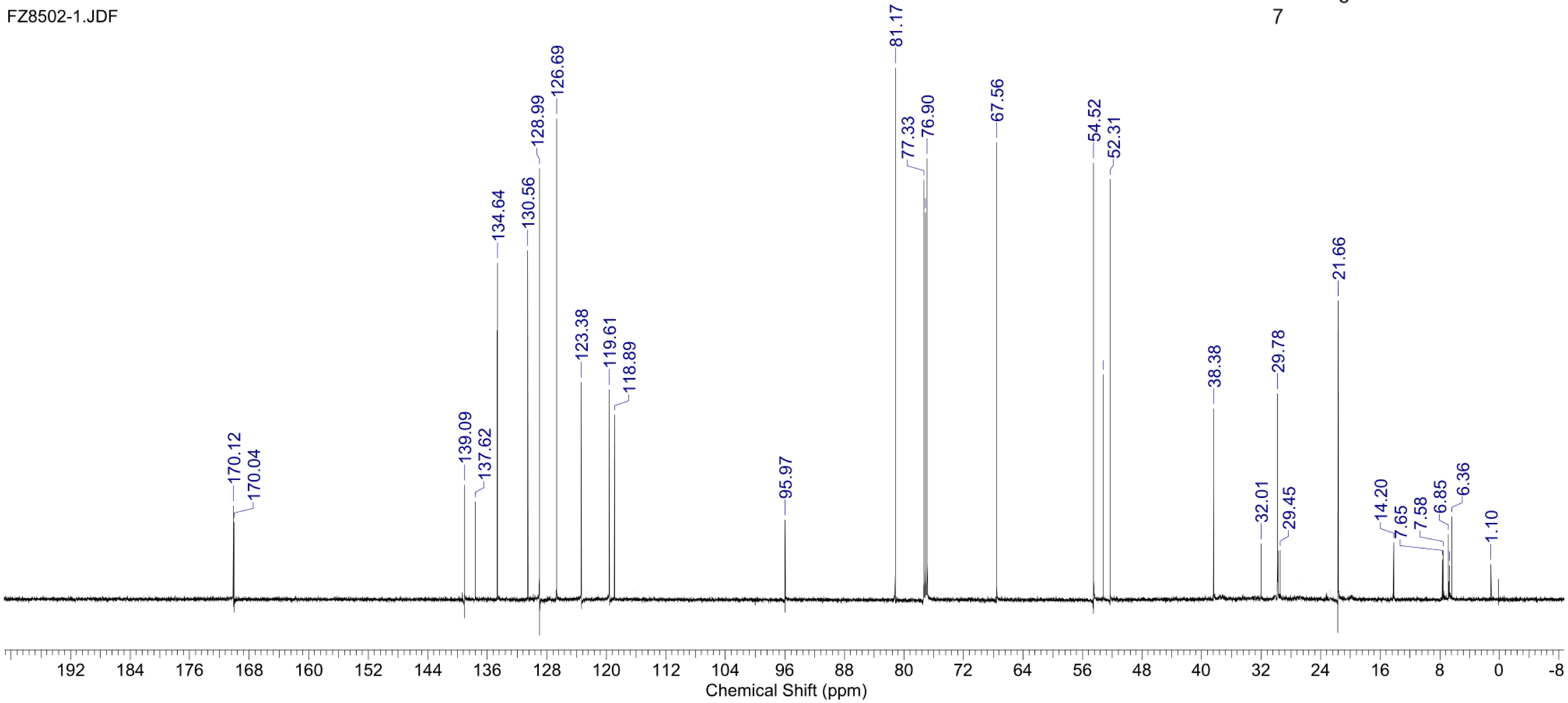

**Formula** $C_{20}H_{21}NO_4$	**FW** 339.3850

Acquisition Time (sec)	0.6921	***Comment***	single pulse decoupled gated NOE			***Date***	10 Feb 2020 01:29:38
Date Stamp	22 Feb 2020 03:15:21			***File Name***	C:\USERS\лаба 534\DESKTOP\Саша\ЯМР\NMR FIDS\FZ8502-1.JDF		
Frequency (MHz)	150.91	***Nucleus***	13C	***Number of Transients***	4000	***Origin***	ECA 600
Original Points Count	32768	***Owner***	CKP	***Points Count***	32768	***Pulse Sequence***	single_pulse_dec
Receiver Gain	54.00	***Solvent***	CHLOROFORM-d			***Spectrum Offset (Hz)***	15091.3428
Sweep Width (Hz)	47348.49	***Temperature (degree C)***	24.000				

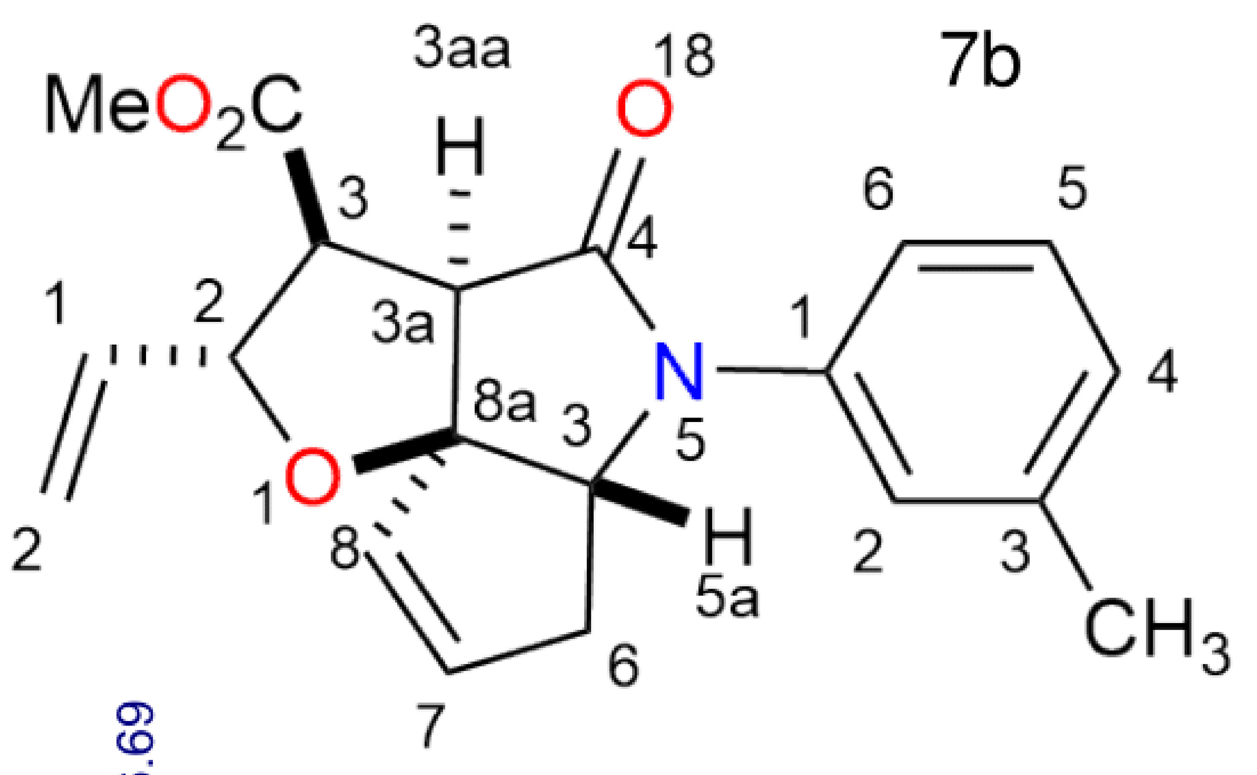

FZ8502-1.JDF

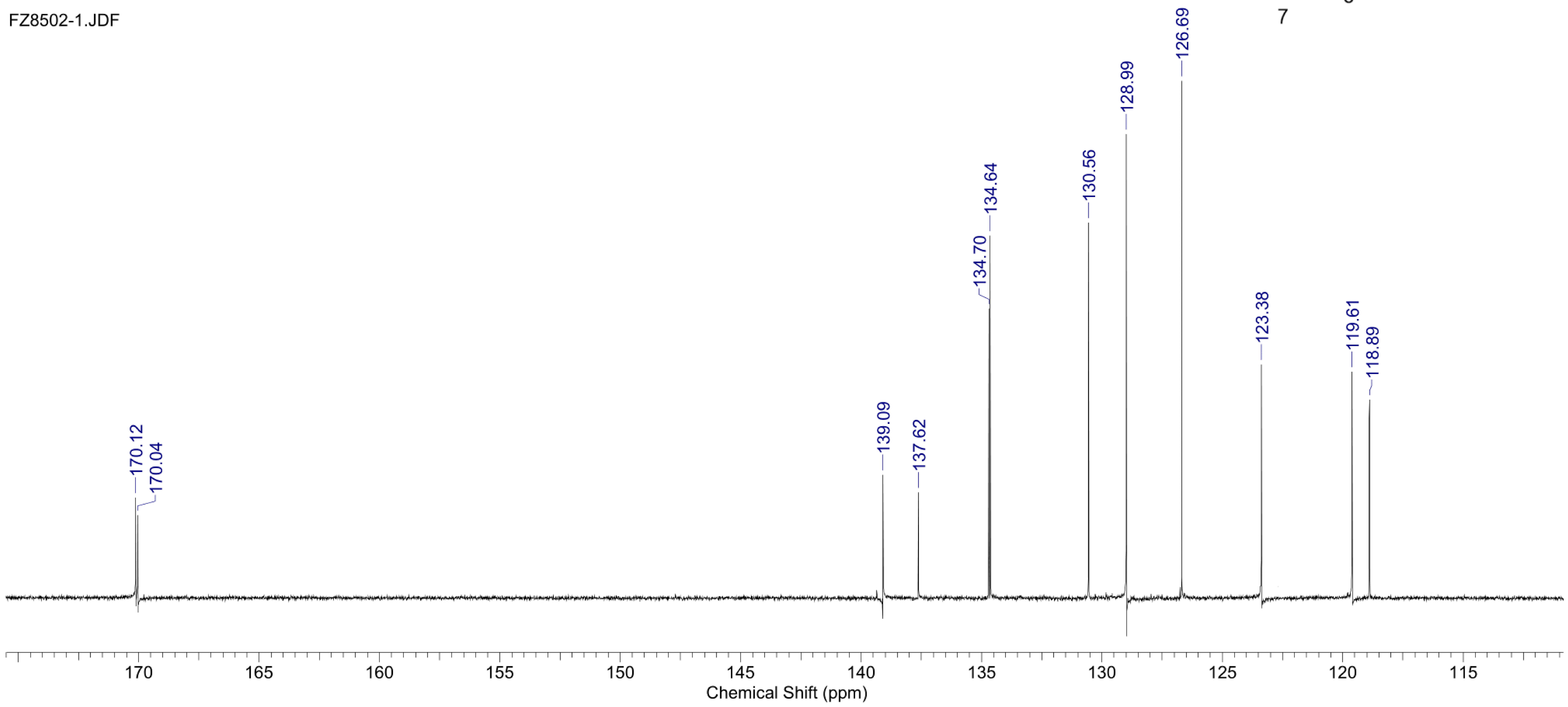

Formula $C_{20}H_{21}NO_4$	FW 339.3850

**Acquisition Time (sec)**	0.6921	**Comment**	single pulse decoupled gated NOE	**Date**	10 Feb 2020 01:29:38
**Date Stamp**	22 Feb 2020 03:15:21	**File Name**	C:\USERS\лаба 534\DESKTOP\Саша\ЯМР\NMR FIDS\FZ8502-1.JDF		
**Frequency (MHz)**	150.91	**Nucleus**	13C	**Number of Transients**	4000
**Origin**	ECA 600				
**Original Points Count**	32768	**Owner**	CKP	**Points Count**	32768
**Pulse Sequence**	single_pulse_dec				
**Receiver Gain**	54.00	**Solvent**	CHLOROFORM-d	**Spectrum Offset (Hz)**	15091.3428
**Sweep Width (Hz)**	47348.49	**Temperature (degree C)**	24.000		

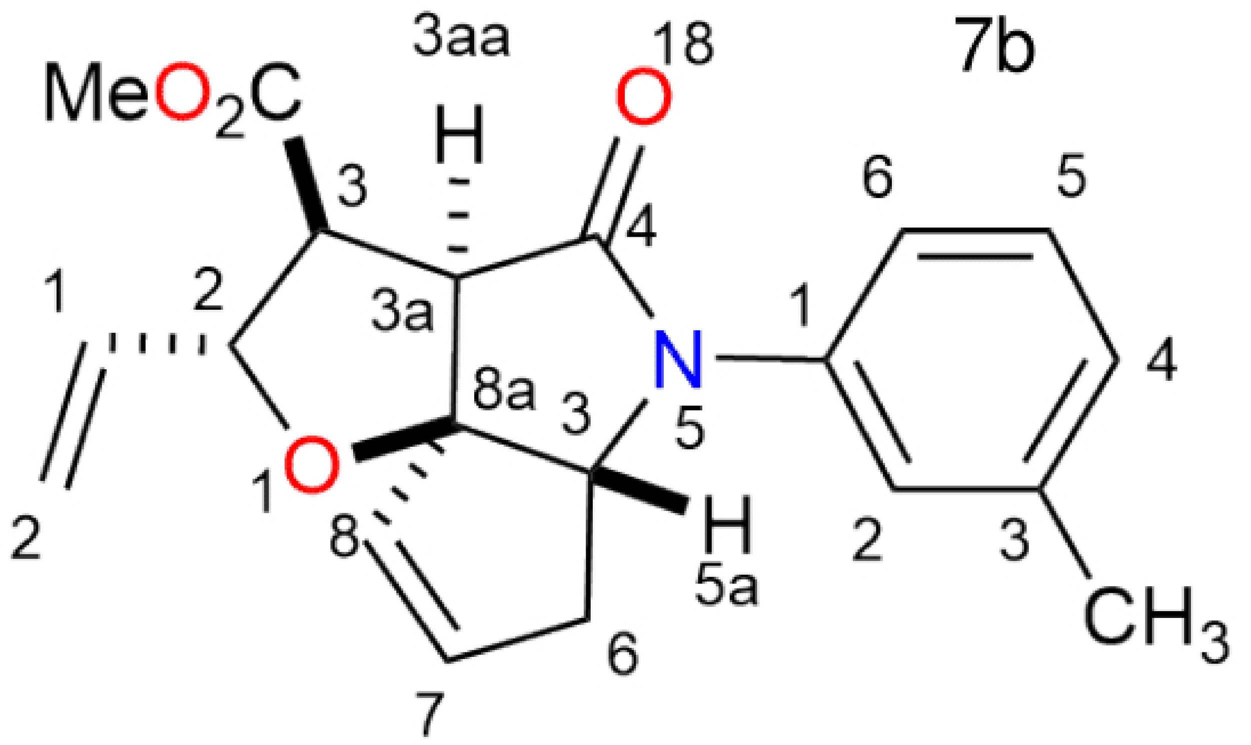

7b

FZ8502-1.JDF

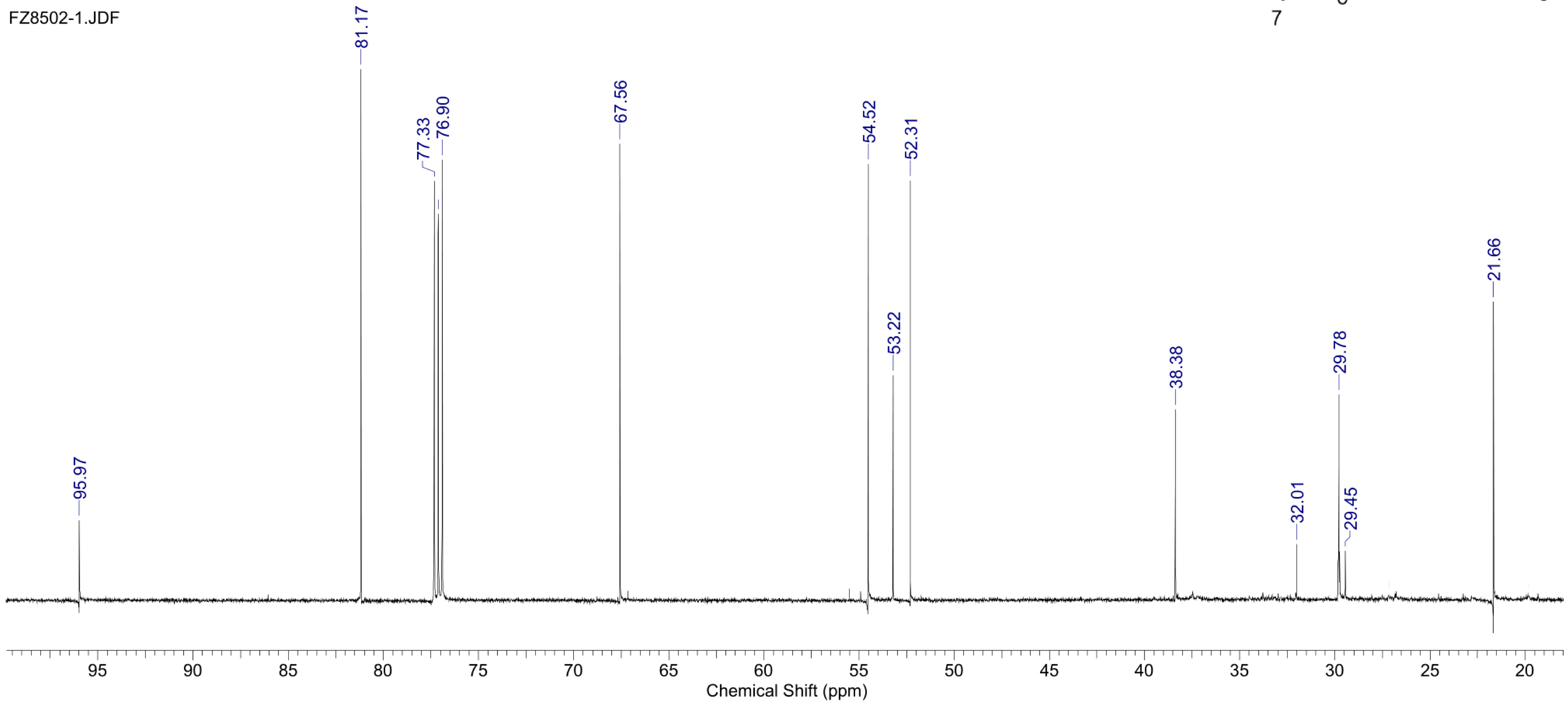

NMR spectrum data for compound 7c

Acquisition Time (sec)	1.9818	***Comment***	single_pulse	***Date***	03 Jul 1990 03:07:55		
Date Stamp	12 Sep 2019 09:10:58			***File Name***	C:\Users\Fedor\Desktop\11.09.19\FZ7800-1.jdf	***Frequency (MHz)***	600.17
Nucleus	1H	***Number of Transients***	8	***Origin***	ECA 600	***Original Points Count*** 32768	***Owner*** delta
Points Count	32768	***Pulse Sequence***	single_pulse.ex2			***Receiver Gain*** 42.00	***Solvent*** CHLOROFORM-d
Spectrum Offset (Hz)	5411.6367	***Sweep Width (Hz)***	16534.39	***Temperature (degree C)***	20.600		

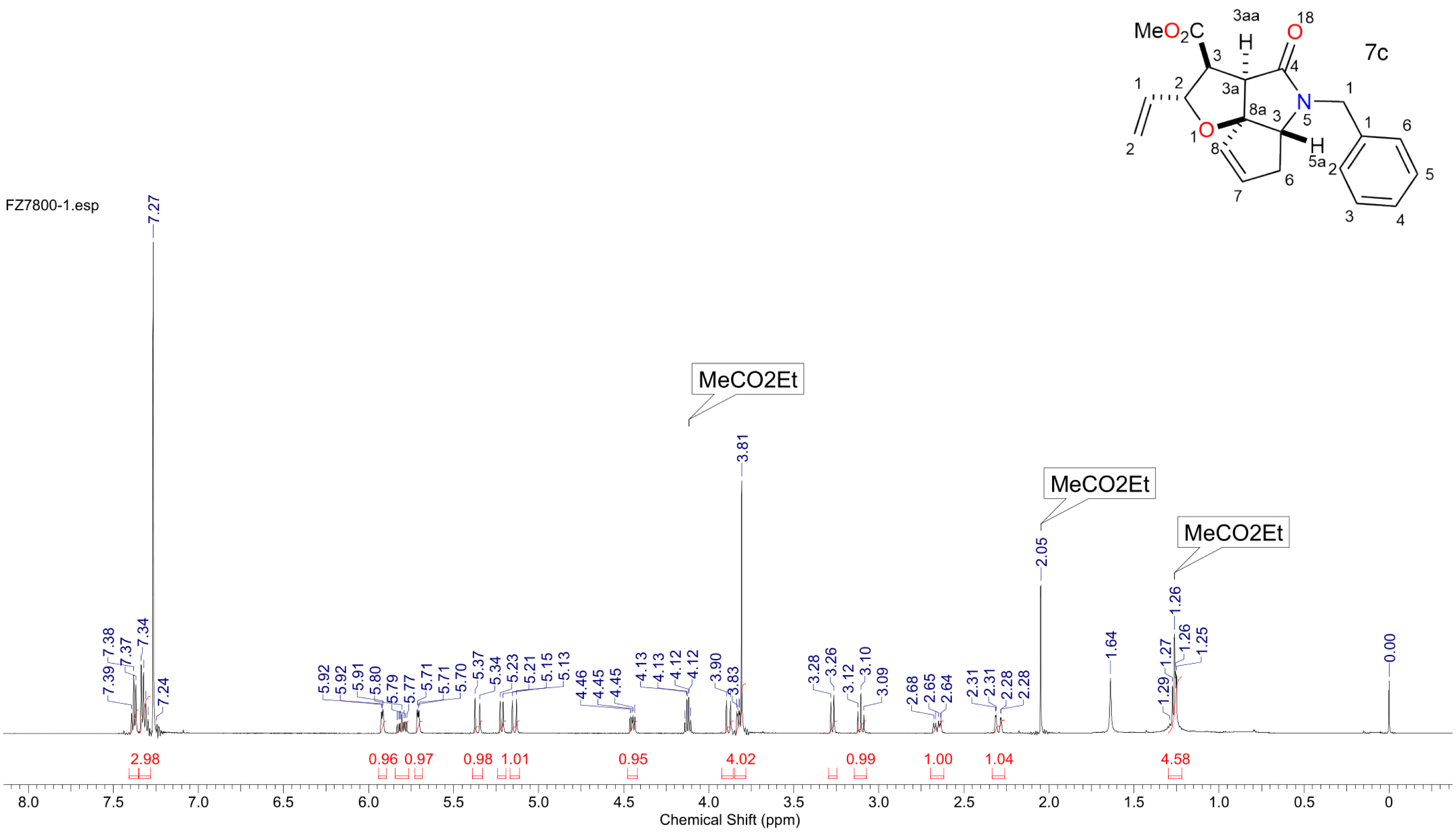

Acquisition Time (sec)	1.9818	***Comment***	single_pulse	***Date***	03 Jul 1990 03:07:55				
Date Stamp	12 Sep 2019 09:10:58			***File Name***	C:\Users\Fedor\Desktop\11.09.19\FZ7800-1.jdf			***Frequency (MHz)***	600.17
Nucleus	1H	***Number of Transients***	8	***Origin***	ECA 600	***Original Points Count***	32768	***Owner***	delta
Points Count	32768	***Pulse Sequence***	single_pulse.ex2			***Receiver Gain***	42.00	***Solvent***	CHLOROFORM-d
Spectrum Offset (Hz)	5411.6367	***Sweep Width (Hz)***	16534.39	***Temperature (degree C)***	20.600				

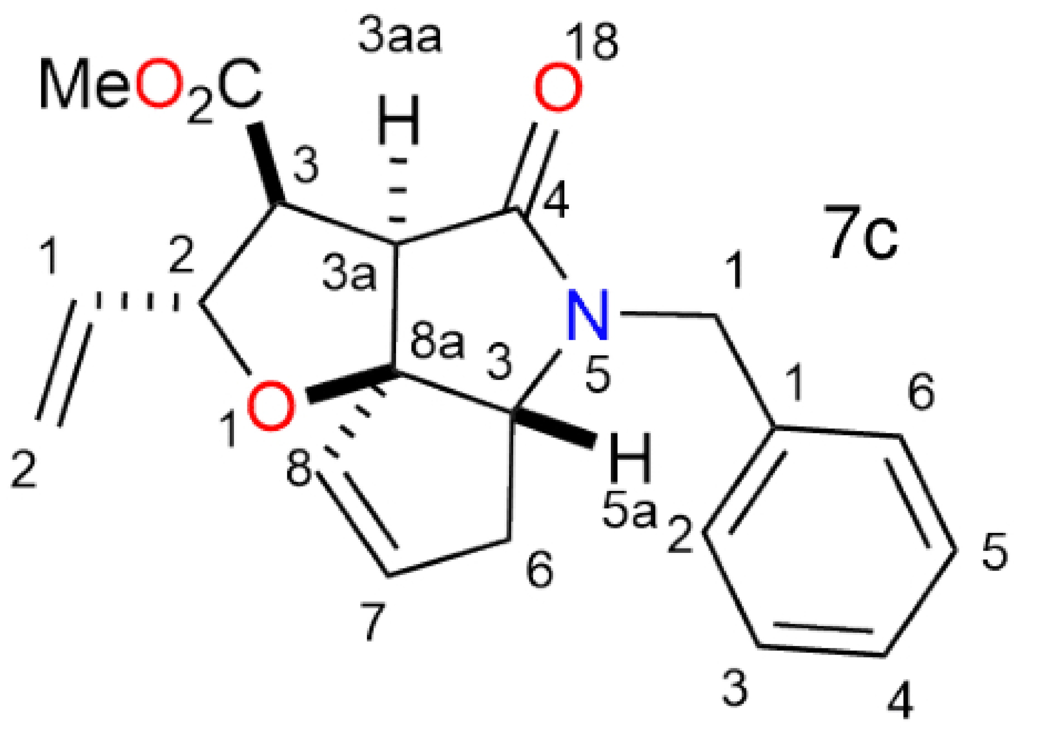

FZ7800-1.esp

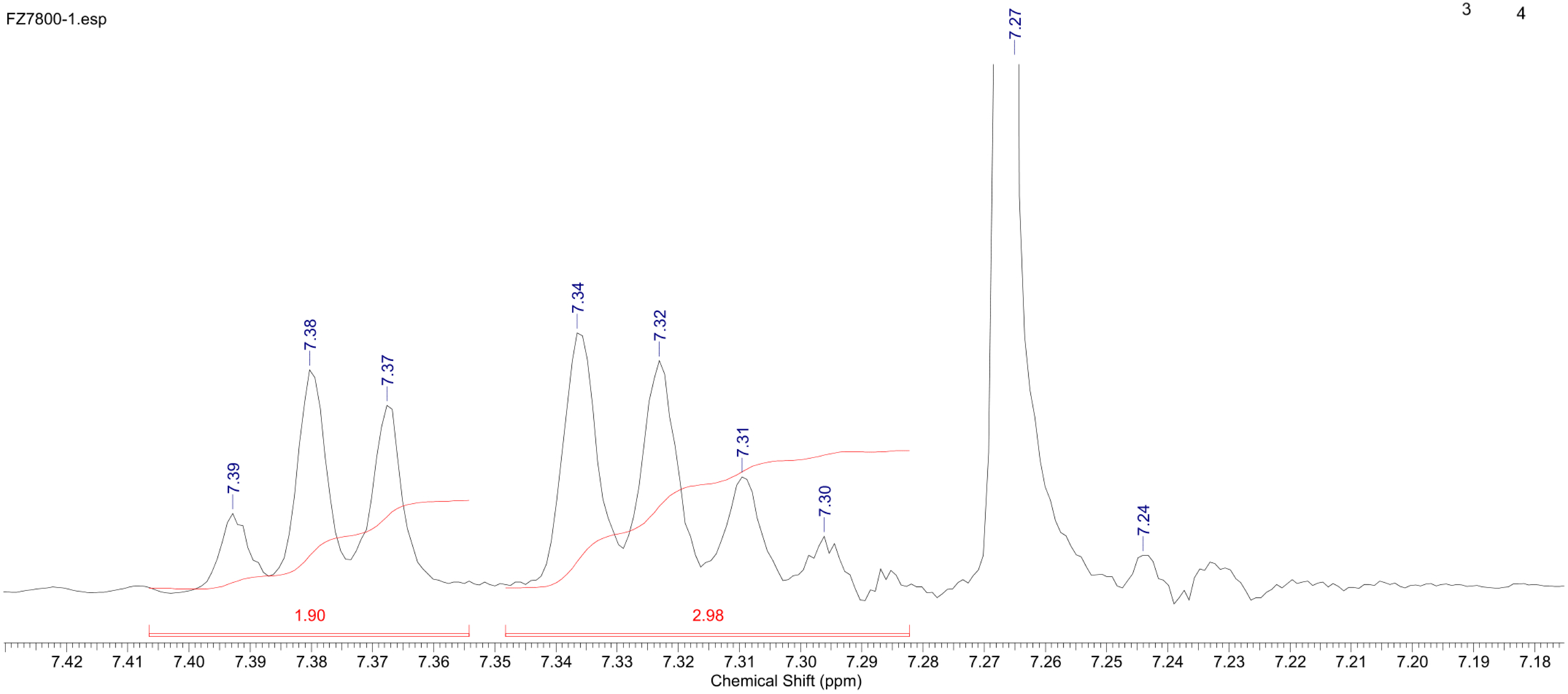

Acquisition Time (sec)	1.9818	***Comment***	single_pulse	***Date***	03 Jul 1990 03:07:55		
Date Stamp	12 Sep 2019 09:10:58			***File Name***	C:\Users\Fedor\Desktop\11.09.19\FZ7800-1.jdf	***Frequency (MHz)***	600.17
Nucleus	1H	***Number of Transients***	8	***Origin***	ECA 600	***Original Points Count*** 32768	***Owner*** delta
Points Count	32768	***Pulse Sequence***	single_pulse.ex2			***Receiver Gain*** 42.00	***Solvent*** CHLOROFORM-d
Spectrum Offset (Hz)	5411.6367	***Sweep Width (Hz)***	16534.39	***Temperature (degree C)***	20.600		

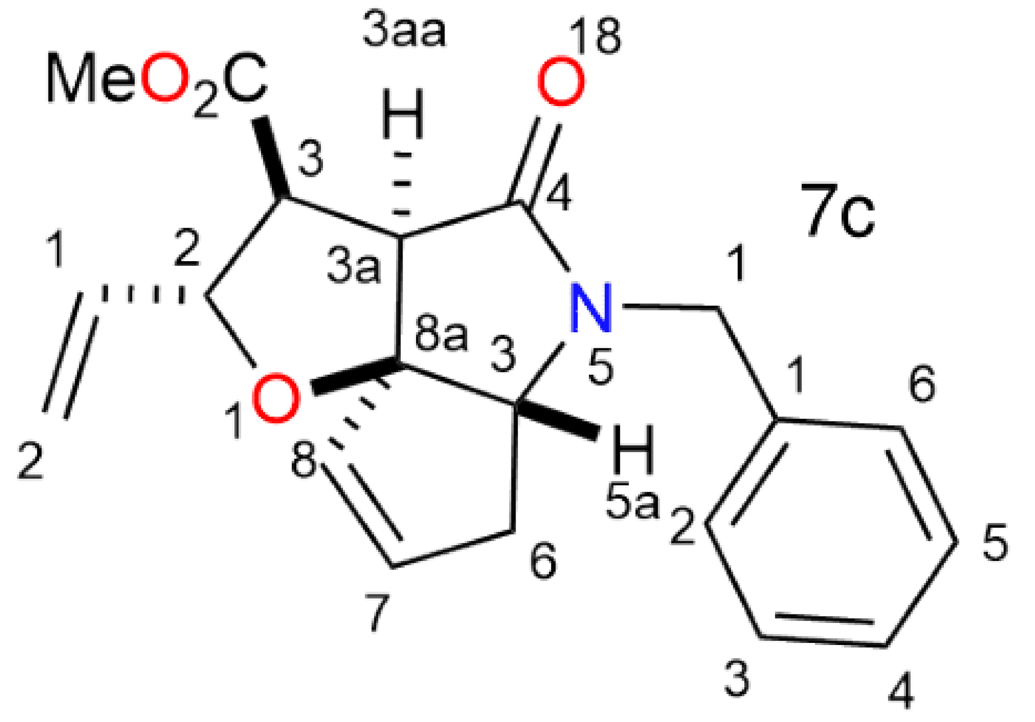

7c

FZ7800-1.esp

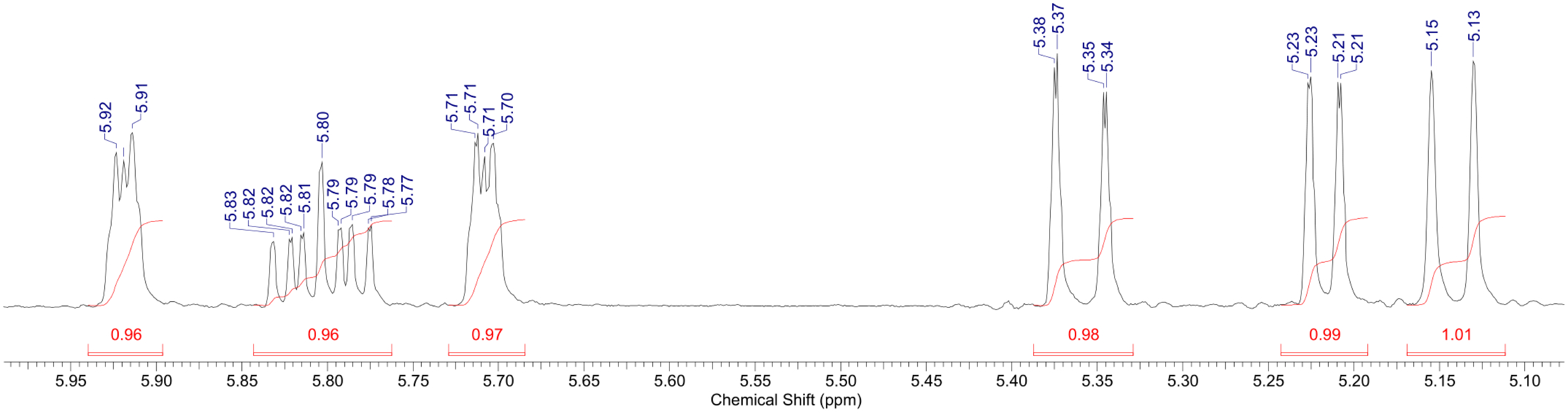

Acquisition Time (sec)	1.9818	Comment	single_pulse	Date	03 Jul 1990 03:07:55				
Date Stamp	12 Sep 2019 09:10:58			File Name	C:\Users\Fedor\Desktop\11.09.19\FZ7800-1.jdf			Frequency (MHz)	600.17
Nucleus	1H	Number of Transients	8	Origin	ECA 600	Original Points Count	32768	Owner	delta
Points Count	32768	Pulse Sequence	single_pulse.ex2			Receiver Gain	42.00	Solvent	CHLOROFORM-d
Spectrum Offset (Hz)	5411.6367	Sweep Width (Hz)	16534.39	Temperature (degree C)	20.600				

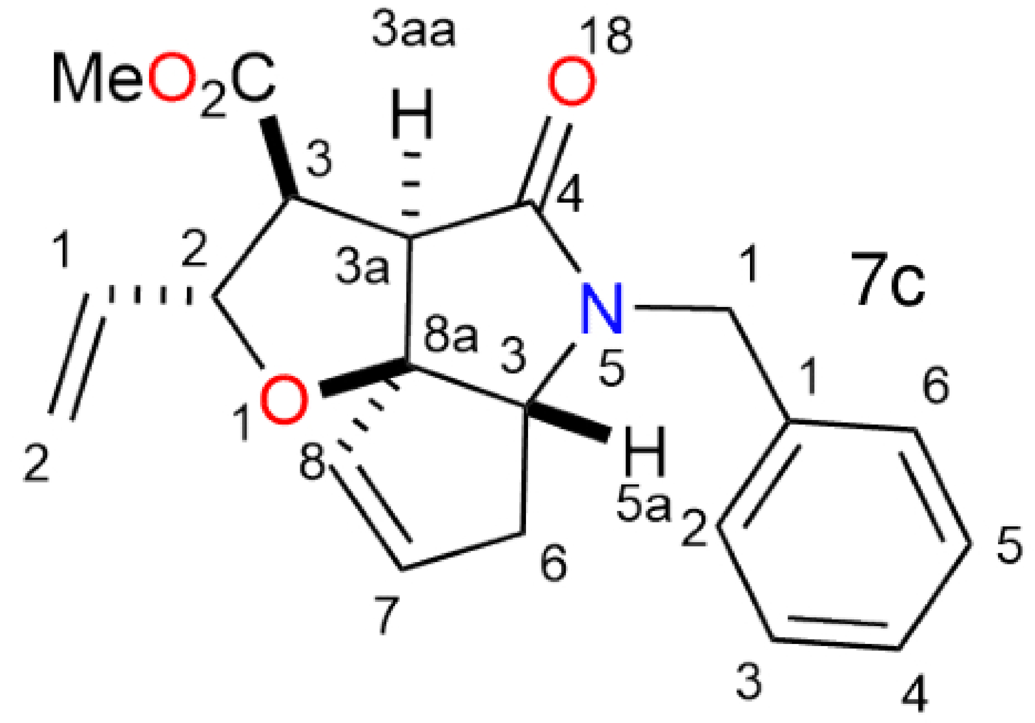

7c

FZ7800-1.esp

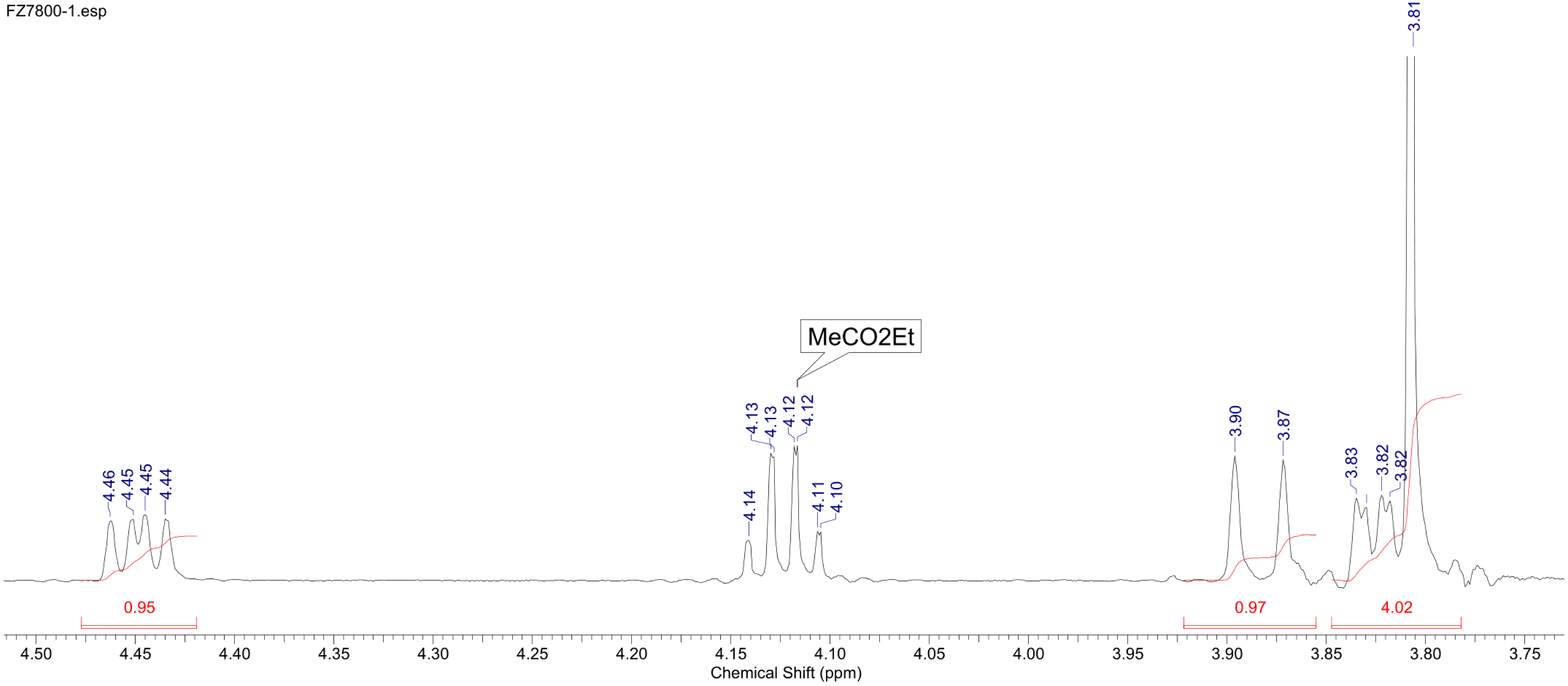

Acquisition Time (sec)	1.9818	***Comment***	single_pulse	***Date***	03 Jul 1990 03:07:55				
Date Stamp	12 Sep 2019 09:10:58			***File Name***	C:\Users\Fedor\Desktop\11.09.19\FZ7800-1.jdf			***Frequency (MHz)***	600.17
Nucleus	1H	***Number of Transients***	8	***Origin***	ECA 600	***Original Points Count***	32768	***Owner***	delta
Points Count	32768	***Pulse Sequence***	single_pulse.ex2			***Receiver Gain***	42.00	***Solvent***	CHLOROFORM-d
Spectrum Offset (Hz)	5411.6367	***Sweep Width (Hz)***	16534.39	***Temperature (degree C)***	20.600				

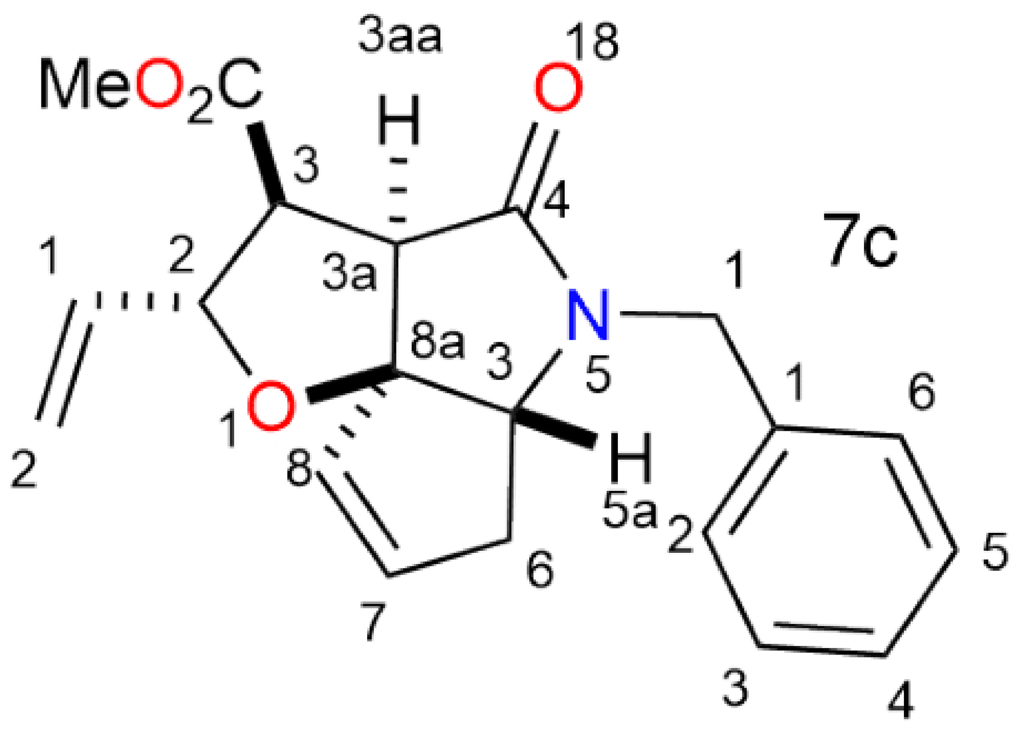

FZ7800-1.esp

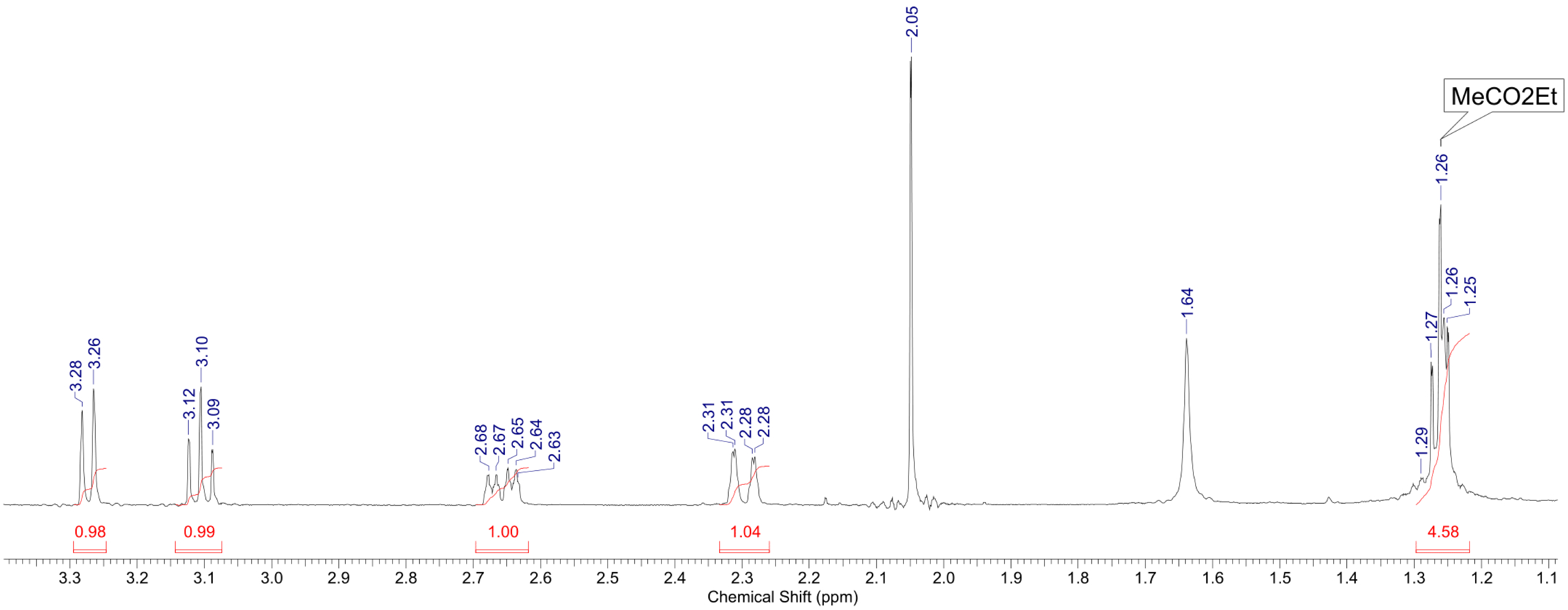

Acquisition Time (sec)	0.6921	***Comment***	single pulse decoupled gated NOE	***Date***	07 Jul 1990 07:07:32
Date Stamp	16 Sep 2019 13:10:37	***File Name***	C:\Users\Fedor\Desktop\16.09.19\FZ7820-1.jdf		
Frequency (MHz)	150.91	***Nucleus***	13C	***Number of Transients***	1000
Origin	ECA 600	***Original Points Count***	32768	***Owner***	delta
Points Count	32768	***Pulse Sequence***	single_pulse_dec	***Receiver Gain***	58.00
Solvent	CHLOROFORM-d	***Spectrum Offset (Hz)***	15079.3525	***Sweep Width (Hz)***	47348.49
Temperature (degree C)	23.300				

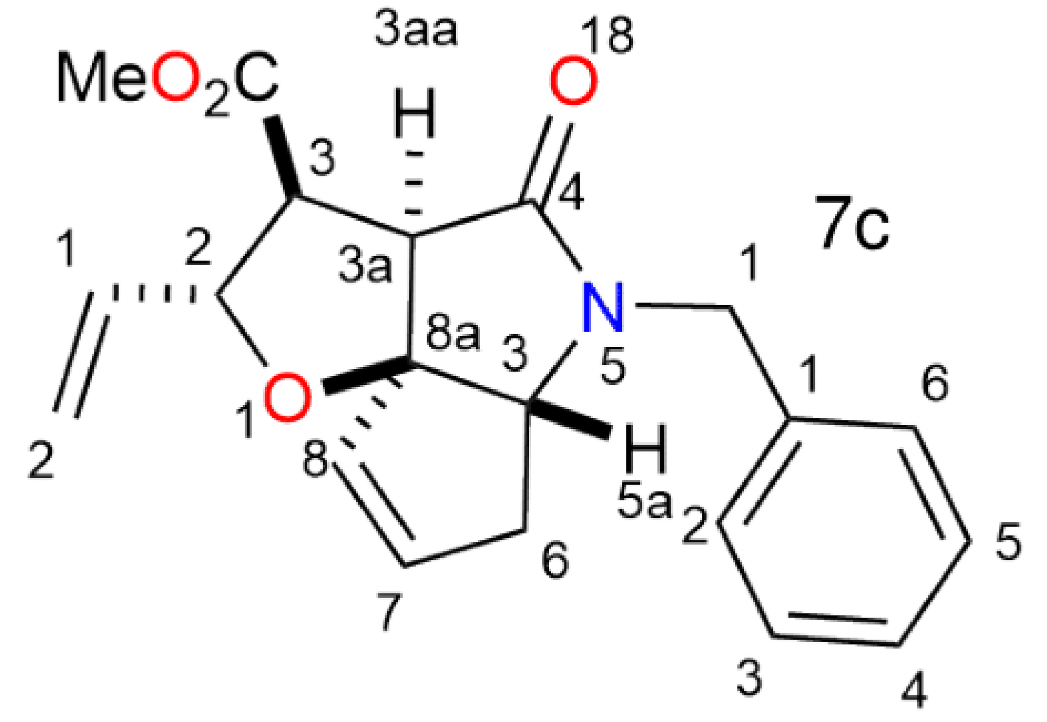

7c

FZ7820-1.jdf

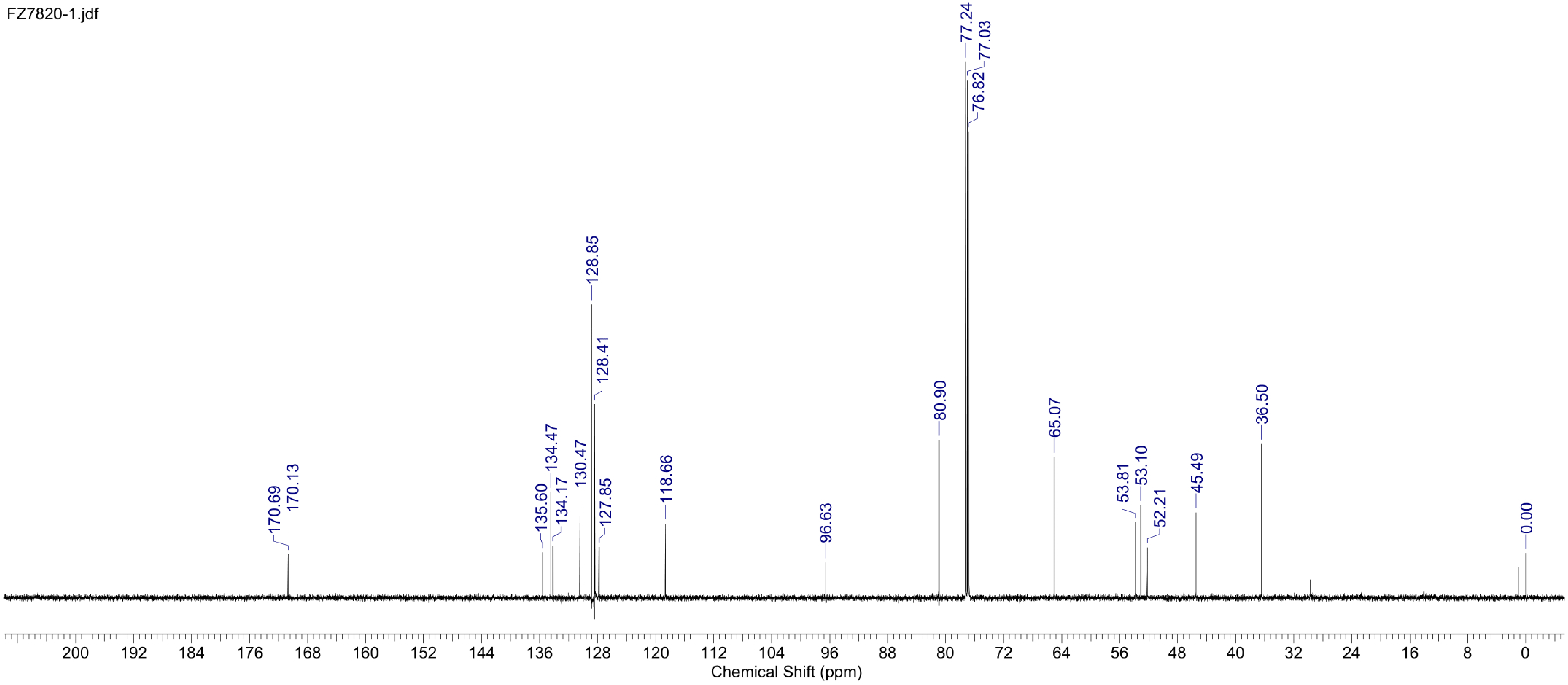

**Acquisition Time (sec)**	0.6921	**Comment**	single pulse decoupled gated NOE			**Date**	07 Jul 1990 07:07:32
**Date Stamp**	16 Sep 2019 13:10:37			**File Name**	C:\Users\Fedor\Desktop\16.09.19\FZ7820-1.jdf		
**Frequency (MHz)**	150.91	**Nucleus**	13C	**Number of Transients**	1000	**Origin**	ECA 600
**Original Points Count**	32768	**Owner**	delta	**Points Count**	32768	**Pulse Sequence**	single_pulse_dec
**Receiver Gain**	58.00	**Solvent**	CHLOROFORM-d			**Spectrum Offset (Hz)**	15079.3525
**Sweep Width (Hz)**	47348.49	**Temperature (degree C)**	23.300				

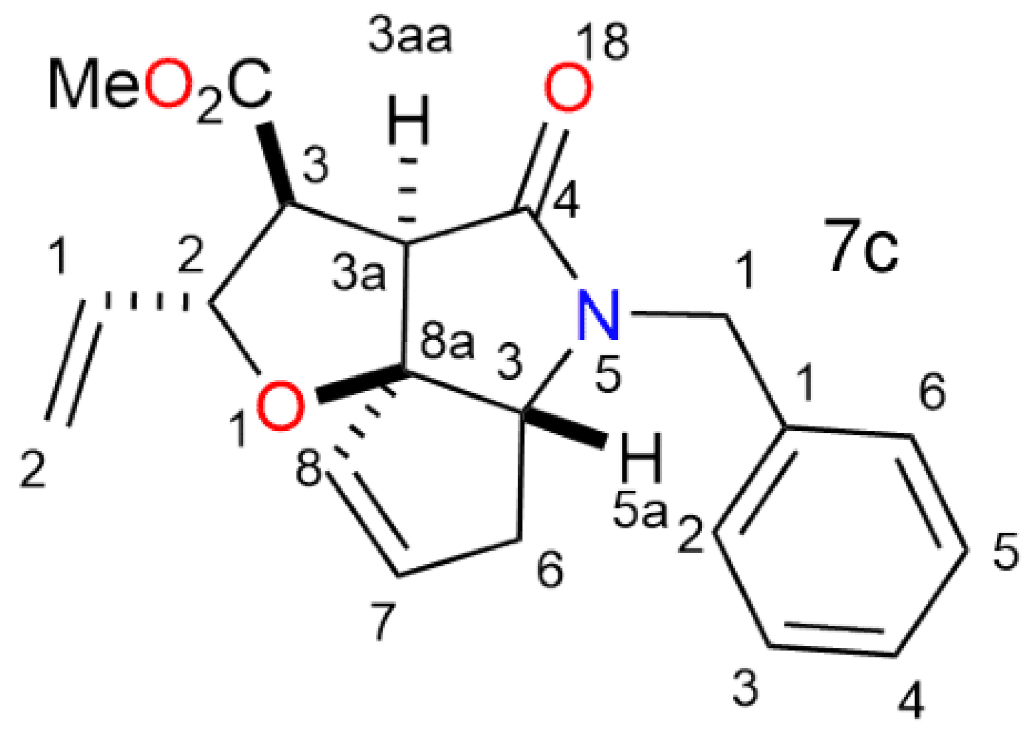

7c

FZ7820-1.jdf

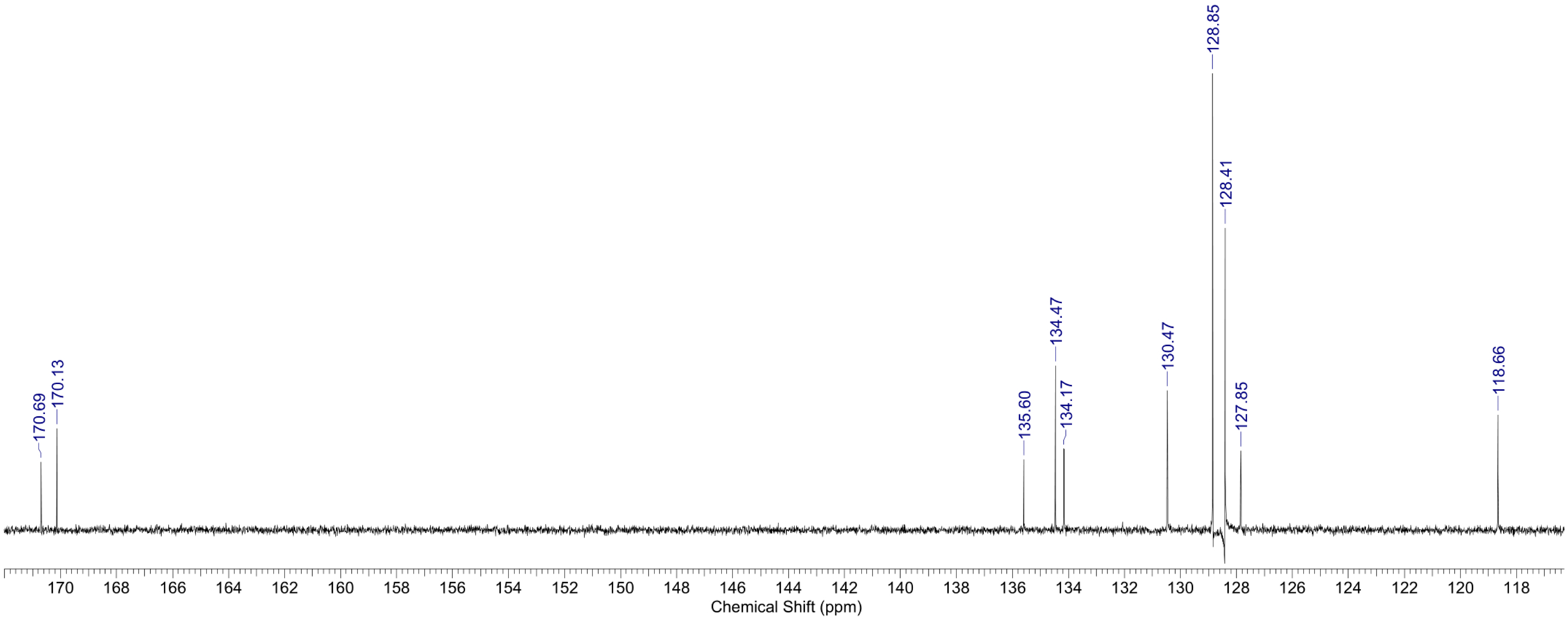

Acquisition Time (sec)	0.6921	***Comment***	single pulse decoupled gated NOE			***Date***	07 Jul 1990 07:07:32
Date Stamp	16 Sep 2019 13:10:37			***File Name***	C:\Users\Fedor\Desktop\16.09.19\FZ7820-1.jdf		
Frequency (MHz)	150.91	***Nucleus***	13C	***Number of Transients***	1000	***Origin***	ECA 600
Original Points Count	32768	***Owner***	delta	***Points Count***	32768	***Pulse Sequence***	single_pulse_dec
Receiver Gain	58.00	***Solvent***	CHLOROFORM-d			***Spectrum Offset (Hz)***	15079.3525
Sweep Width (Hz)	47348.49	***Temperature (degree C)***	23.300				

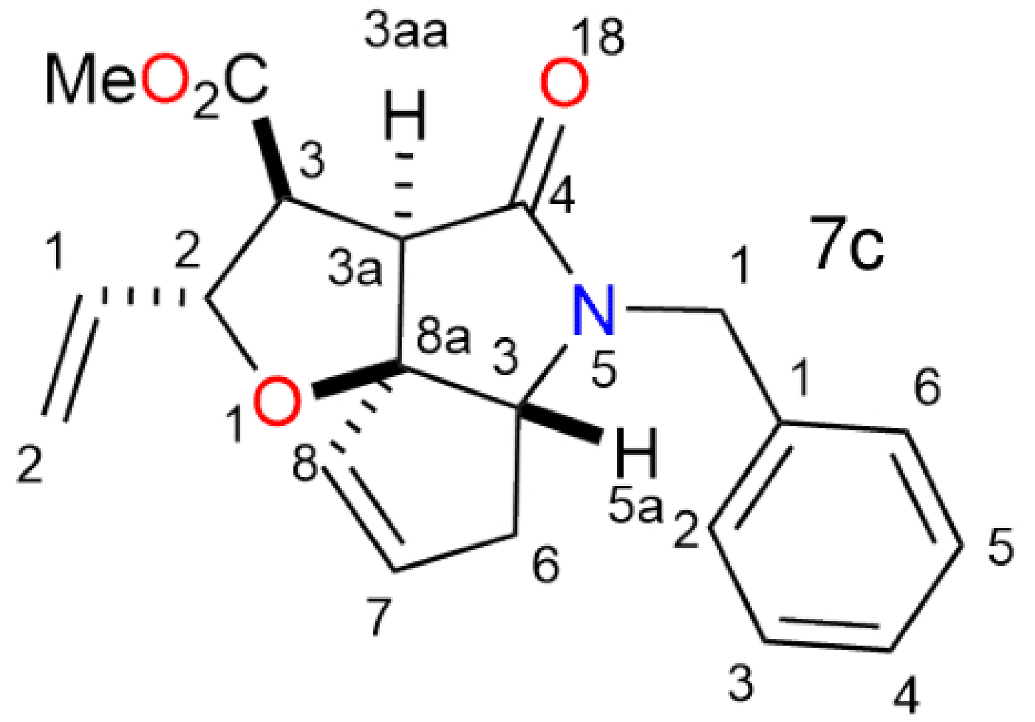

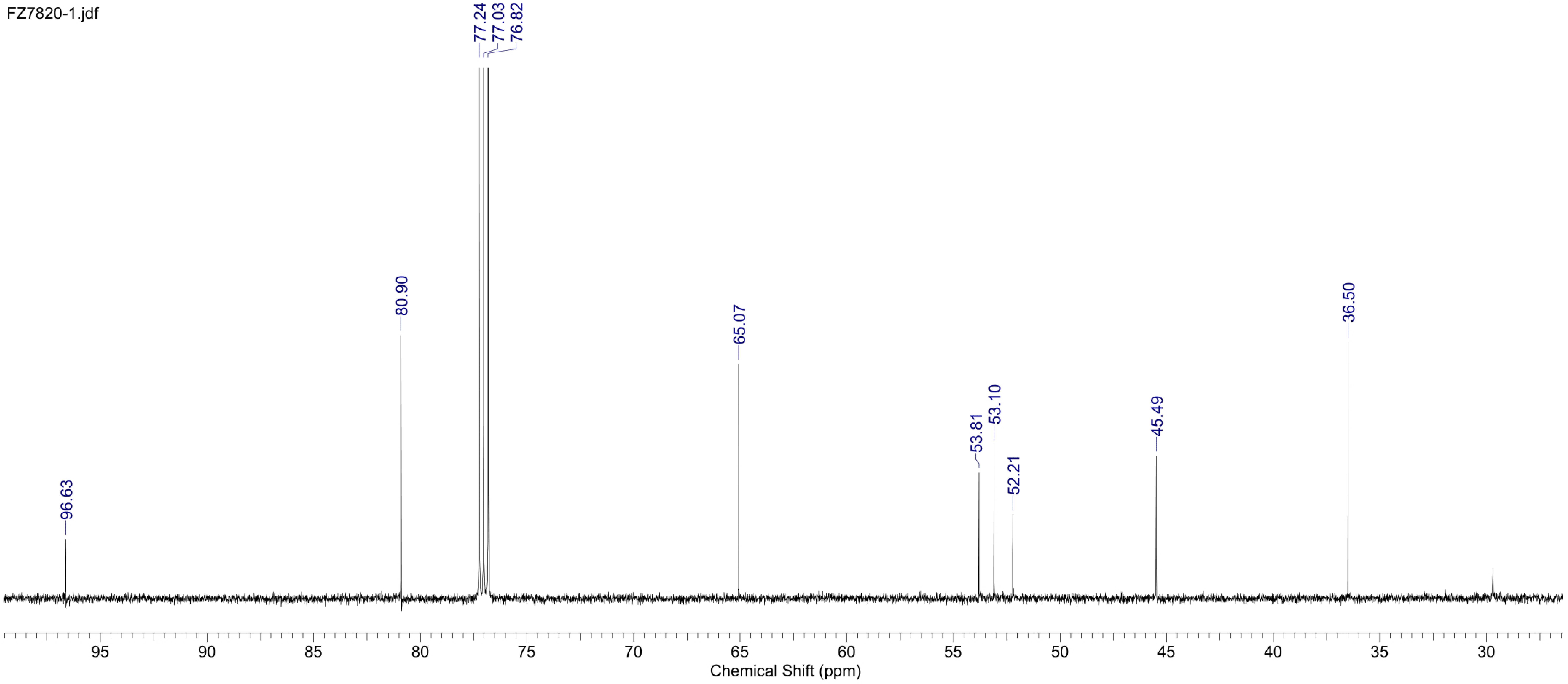

NMR spectrum data for compound 7d

14.07.2020 19:08:54

**Formula** $C_{19}H_{18}ClNO_4$	**FW** 359.8035

**Acquisition Time (sec)**	1.9818	**Comment**	single_pulse	**Date**	03 Mar 2020 13:23:30		
**Date Stamp**	03 Mar 2020 12:43:19			**File Name**	C:\USERS\Лаб534\DOWNLOADS\FZ8553-1.JDF	**Frequency (MHz)**	600.17
**Nucleus**	1H	**Number of Transients**	8	**Origin**	ECA 600 **Original Points Count** 32768	**Owner**	CKP
**Points Count**	32768	**Pulse Sequence**	single_pulse.ex2		**Receiver Gain** 36.00	**Solvent**	CHLOROFORM-d
**Spectrum Offset (Hz)**	5412.6455	**Sweep Width (Hz)**	16534.39	**Temperature (degree C)**	22.800		

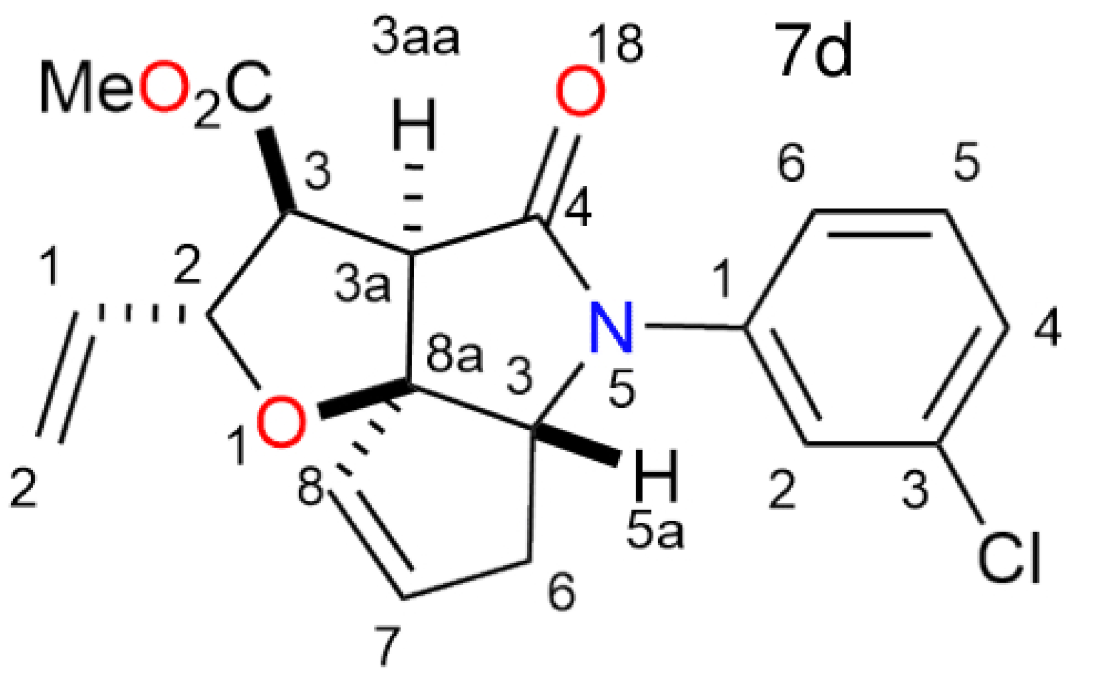

FZ8553-1.JDF

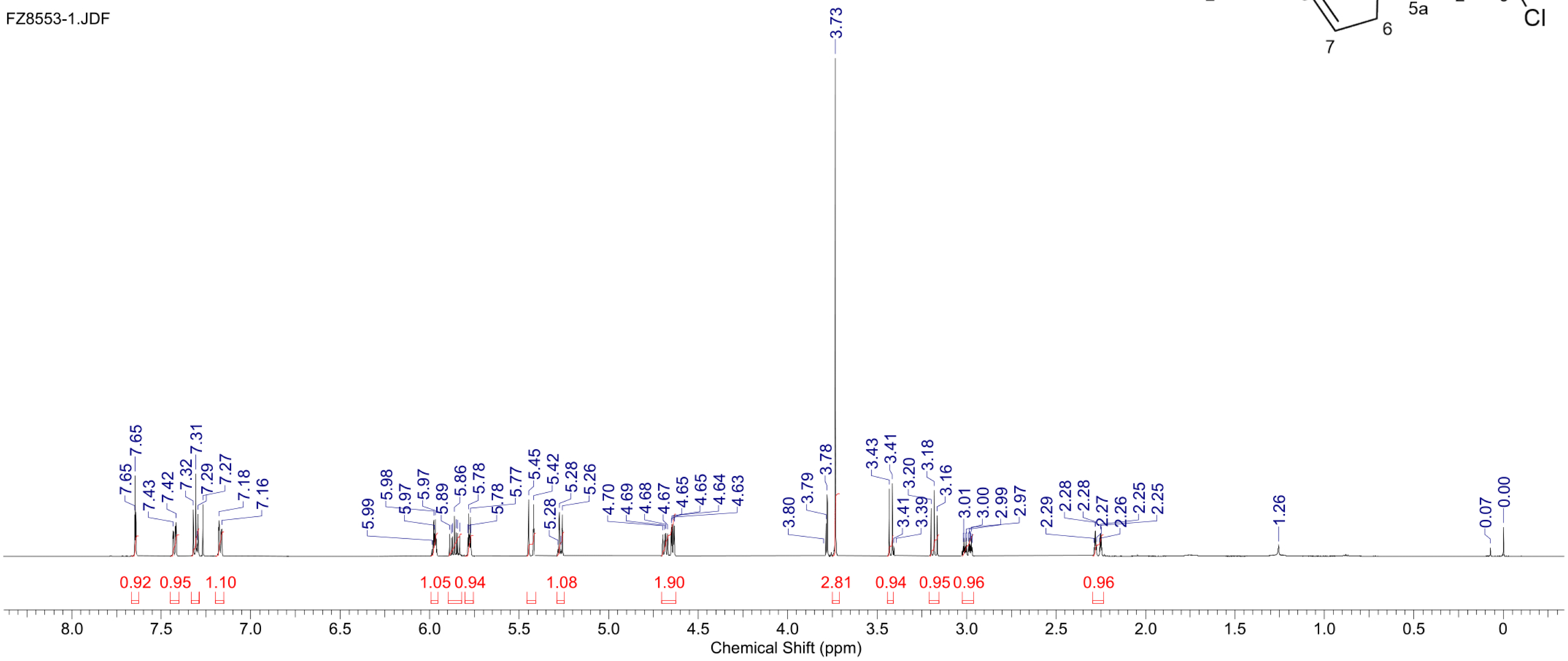

14.07.2020 19:09:22

**Formula** $C_{19}H_{18}ClNO_4$	**FW** 359.8035

**Acquisition Time (sec)**	1.9818	**Comment**	single_pulse	**Date**	03 Mar 2020 13:23:30				
**Date Stamp**	03 Mar 2020 12:43:19			**File Name**	C:\USERS\Лаба534\DOWNLOADS\FZ8553-1.JDF			**Frequency (MHz)**	600.17
**Nucleus**	1H	**Number of Transients**	8	**Origin**	ECA 600	**Original Points Count**	32768	**Owner**	CKP
**Points Count**	32768	**Pulse Sequence**	single_pulse.ex2			**Receiver Gain**	36.00	**Solvent**	CHLOROFORM-d
**Spectrum Offset (Hz)**	5412.6455	**Sweep Width (Hz)**	16534.39	**Temperature (degree C)**	22.800				

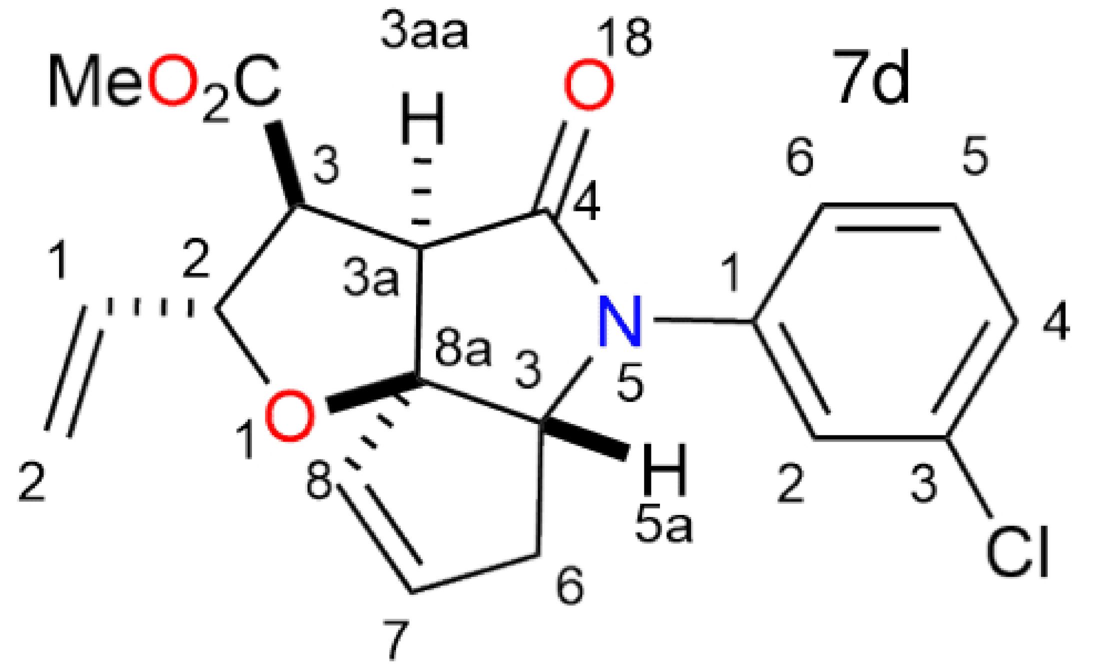

FZ8553-1.JDF

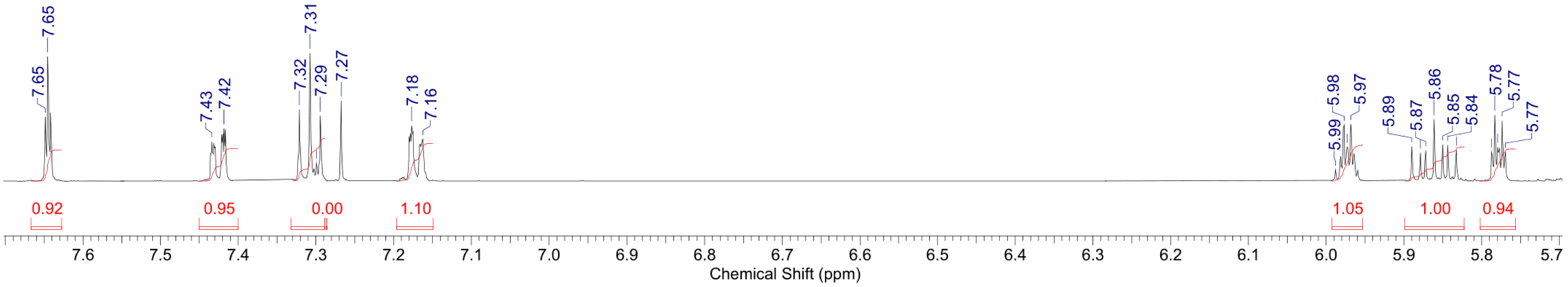

14.07.2020 19:09:42

Formula	$C_{19}H_{18}ClNO_4$	FW	359.8035

**Acquisition Time (sec)**	1.9818	**Comment**	single_pulse	**Date**	03 Mar 2020 13:23:30		
**Date Stamp**	03 Mar 2020 12:43:19			**File Name**	C:\USERS\Лаба534\DOWNLOADS\FZ8553-1.JDF	**Frequency (MHz)**	600.17
**Nucleus**	1H	**Number of Transients**	8	**Origin**	ECA 600	**Original Points Count** 32768	**Owner** CKP
**Points Count**	32768	**Pulse Sequence**	single_pulse.ex2			**Receiver Gain** 36.00	**Solvent** CHLOROFORM-d
**Spectrum Offset (Hz)**	5412.6455	**Sweep Width (Hz)**	16534.39	**Temperature (degree C)**	22.800		

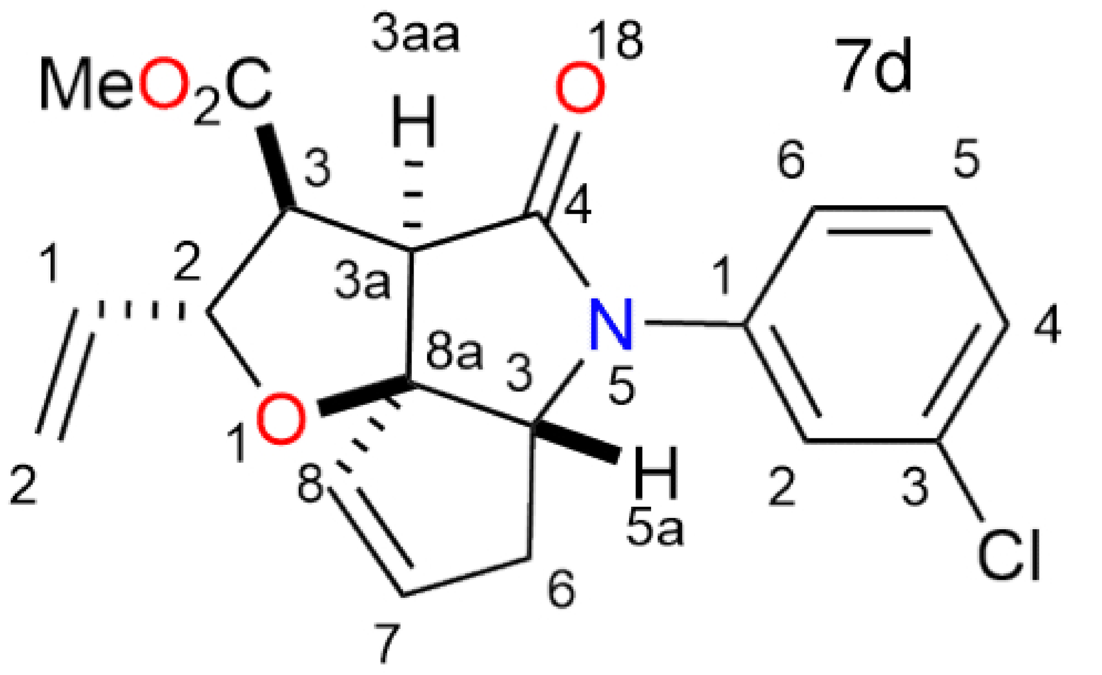

FZ8553-1.JDF

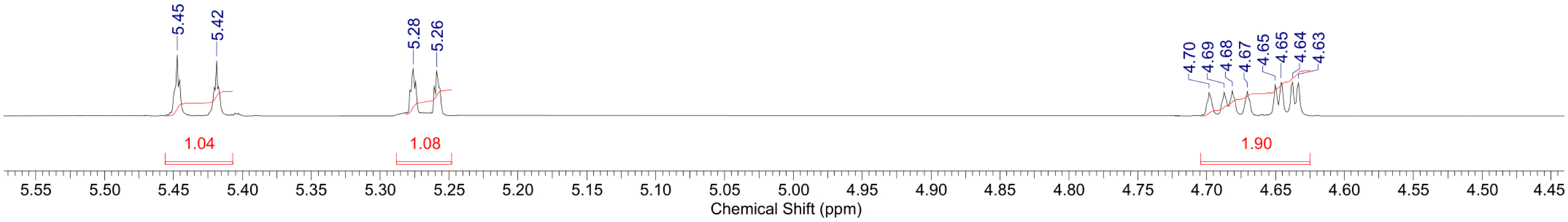

14.07.2020 19:10:01

**Formula** $C_{19}H_{18}ClNO_4$	**FW** 359.8035

**Acquisition Time (sec)**	1.9818	**Comment**	single_pulse	**Date**	03 Mar 2020 13:23:30				
**Date Stamp**	03 Mar 2020 12:43:19			**File Name**	C:\USERS\Лаба534\DOWNLOADS\FZ8553-1.JDF			**Frequency (MHz)**	600.17
**Nucleus**	1H	**Number of Transients**	8	**Origin**	ECA 600	**Original Points Count**	32768	**Owner**	CKP
**Points Count**	32768	**Pulse Sequence**	single_pulse.ex2			**Receiver Gain**	36.00	**Solvent**	CHLOROFORM-d
**Spectrum Offset (Hz)**	5412.6455	**Sweep Width (Hz)**	16534.39	**Temperature (degree C)**	22.800				

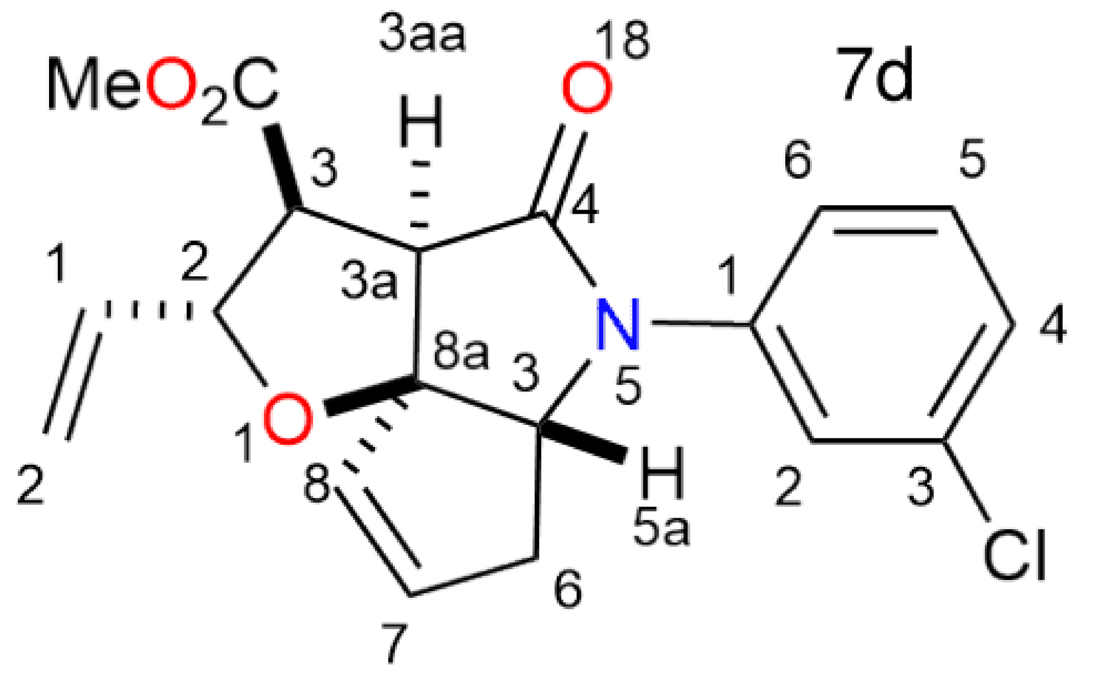

FZ8553-1.JDF

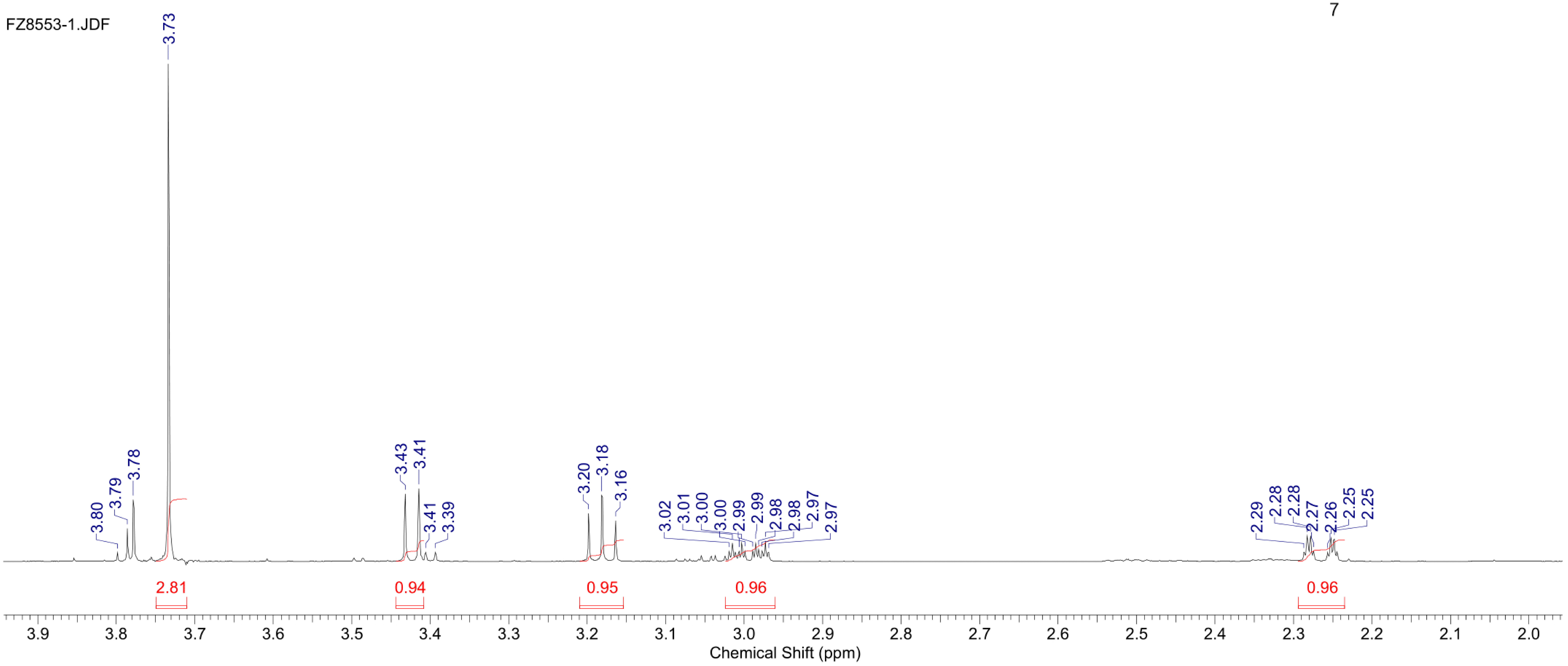

14.07.2020 19:13:02

Formula $C_{19}H_{18}ClNO_4$	*FW* 359.8035

Acquisition Time (sec)	0.6921	***Comment***	single pulse decoupled gated NOE			***Date***	18 Mar 2020 09:58:24
Date Stamp	18 Mar 2020 09:16:35			***File Name***	C:\USERS\Лаба534\DOWNLOADS\FZ8618-1.JDF		
Frequency (MHz)	150.91	***Nucleus***	13C	***Number of Transients***	800	***Origin***	ECA 600
Original Points Count	32768	***Owner***	CKP	***Points Count***	32768	***Pulse Sequence***	single_pulse_dec
Receiver Gain	58.00	***Solvent***	CHLOROFORM-d			***Spectrum Offset (Hz)***	15091.3428
Sweep Width (Hz)	47348.49	***Temperature (degree C)***	23.100				

7d

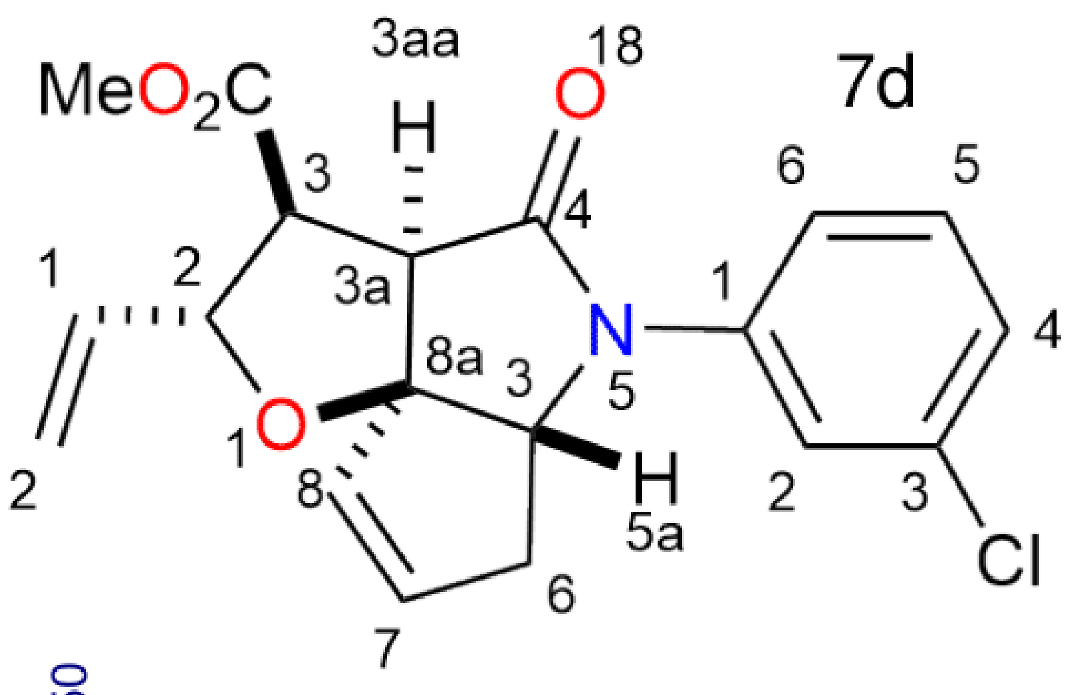

FZ8618-1.JDF

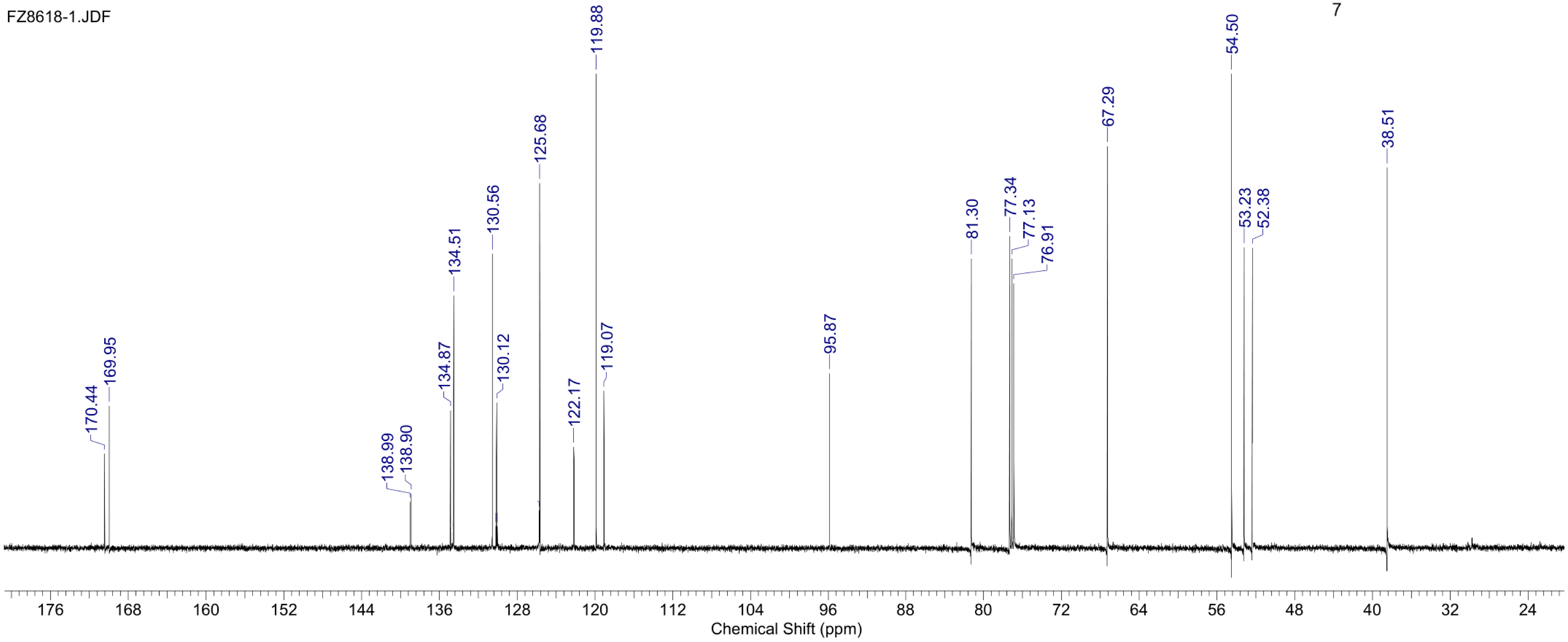

14.07.2020 19:13:11

Formula $C_{19}H_{18}ClNO_4$	FW 359.8035

**Acquisition Time (sec)**	0.6921	**Comment**	single pulse decoupled gated NOE			**Date**	18 Mar 2020 09:58:24
**Date Stamp**	18 Mar 2020 09:16:35			**File Name**	C:\USERS\Лаба534\DOWNLOADS\FZ8618-1.JDF		
**Frequency (MHz)**	150.91	**Nucleus**	13C	**Number of Transients**	800	**Origin**	ECA 600
**Original Points Count**	32768	**Owner**	CKP	**Points Count**	32768	**Pulse Sequence**	single_pulse_dec
**Receiver Gain**	58.00	**Solvent**	CHLOROFORM-d			**Spectrum Offset (Hz)**	15091.3428
**Sweep Width (Hz)**	47348.49	**Temperature (degree C)**	23.100				

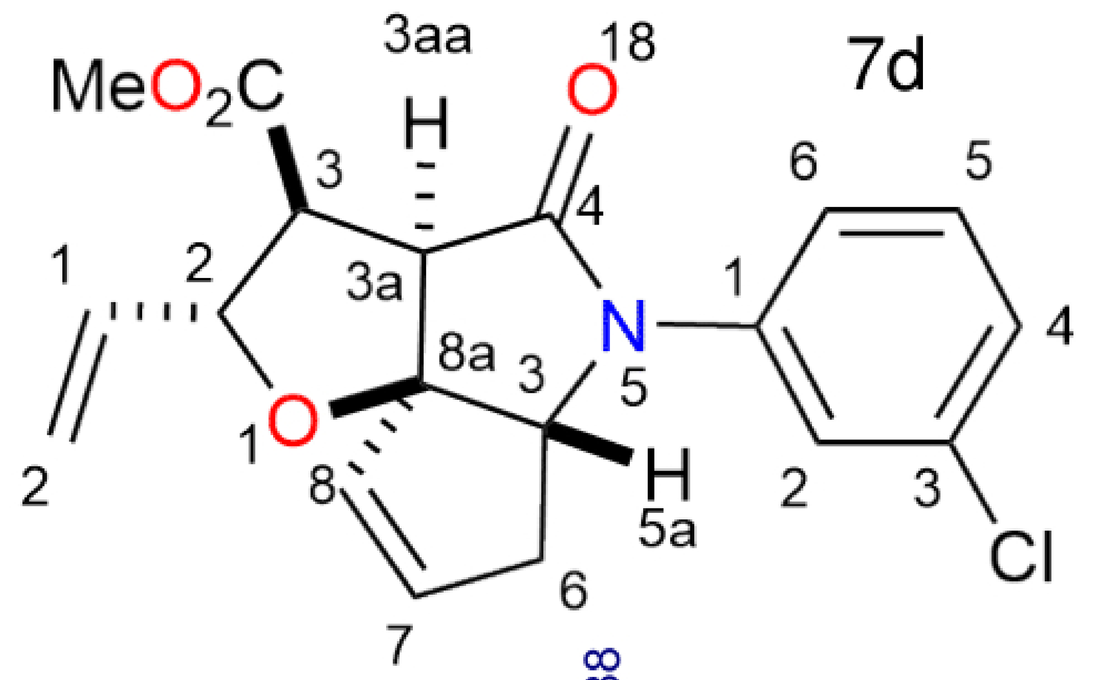

FZ8618-1.JDF

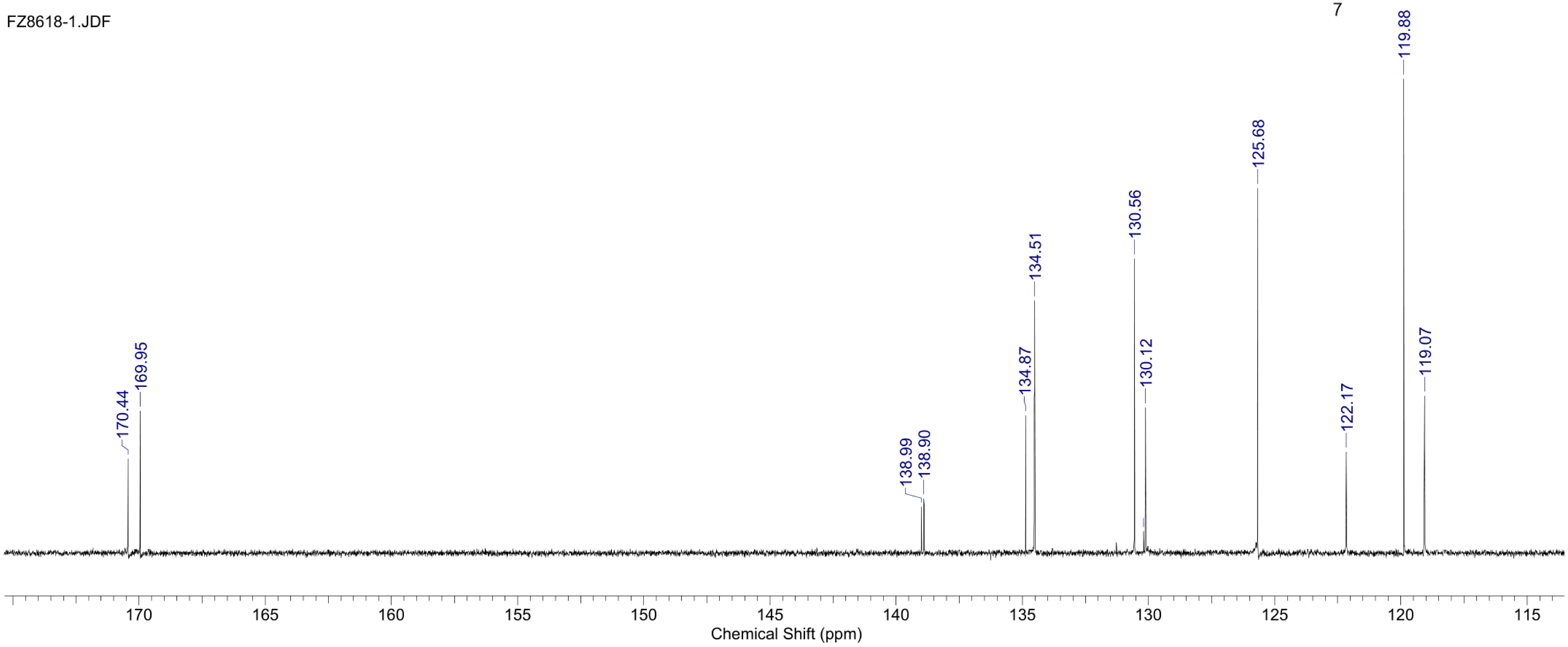

14.07.2020 19:13:21

Formula $C_{19}H_{18}ClNO_4$	FW 359.8035

**Acquisition Time (sec)**	0.6921	**Comment**	single pulse decoupled gated NOE	**Date**	18 Mar 2020 09:58:24
**Date Stamp**	18 Mar 2020 09:16:35	**File Name**	C:\USERS\Лаба534\DOWNLOADS\FZ8618-1.JDF		
**Frequency (MHz)**	150.91	**Nucleus**	13C	**Number of Transients**	800
**Origin**	ECA 600				
**Original Points Count**	32768	**Owner**	CKP	**Points Count**	32768
**Pulse Sequence**	single_pulse_dec				
**Receiver Gain**	58.00	**Solvent**	CHLOROFORM-d	**Spectrum Offset (Hz)**	15091.3428
**Sweep Width (Hz)**	47348.49	**Temperature (degree C)**	23.100		

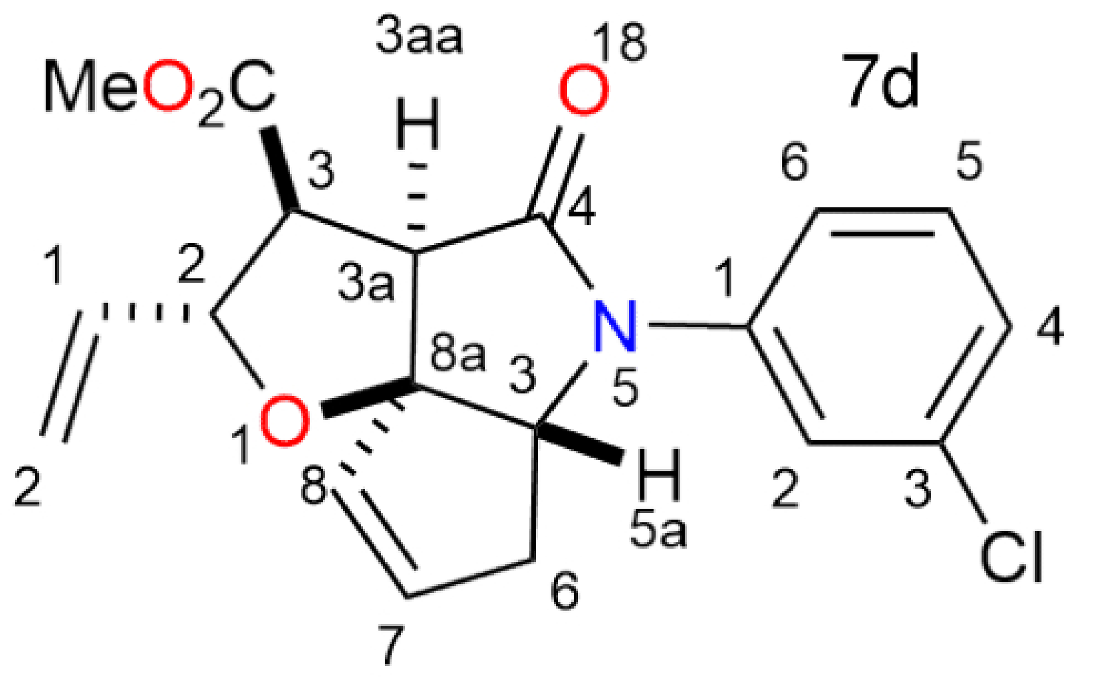

FZ8618-1.JDF

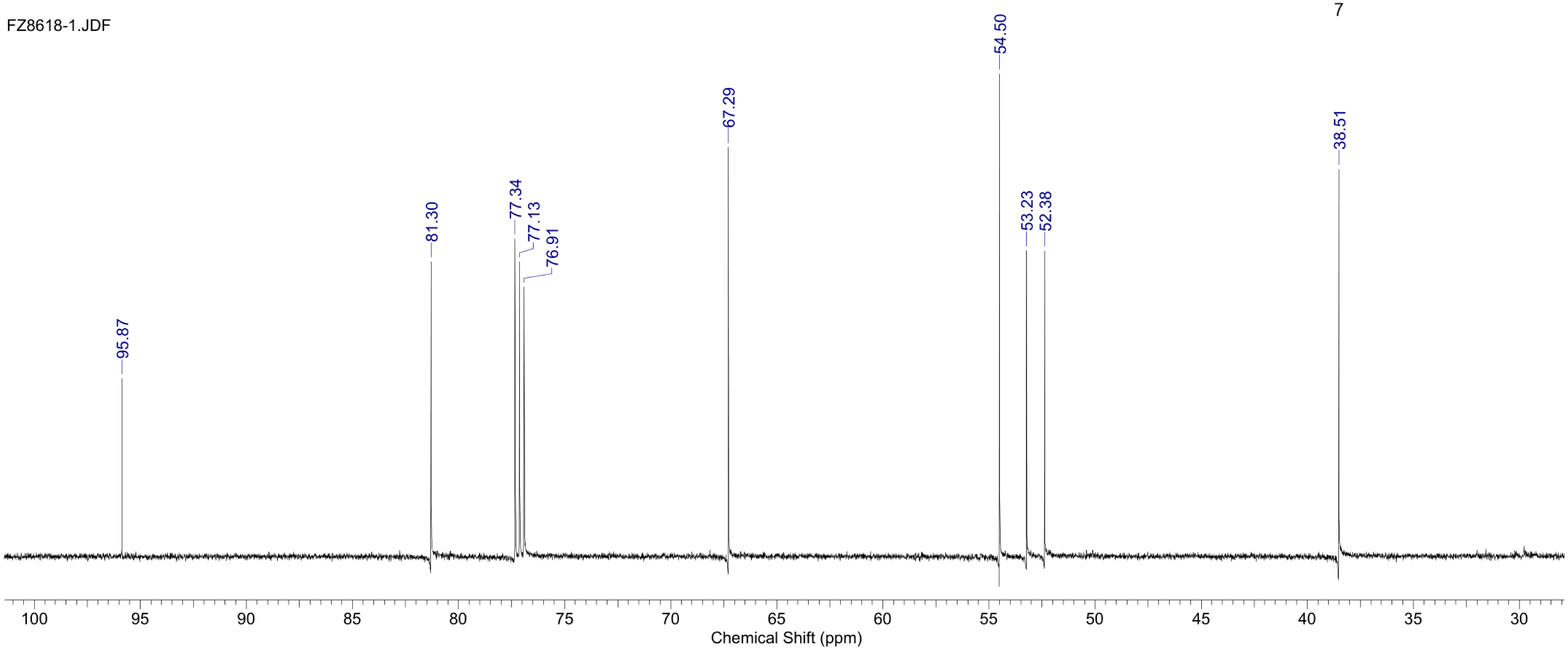

NMR spectrum data for compound 7e

Acquisition Time (sec)	1.9818	***Comment***	single_pulse	***Date***	08 Aug 1990 06:29:56		
Date Stamp	18 Oct 2019 12:33:00			***File Name***	C:\Users\Fedor\Desktop\18.10.19\FZ7994-1.jdf	***Frequency (MHz)***	600.17
Nucleus	1H	***Number of Transients***	8	***Origin***	ECA 600 ***Original Points Count*** 32768	***Owner***	delta
Points Count	32768	***Pulse Sequence***	single_pulse.ex2		***Receiver Gain*** 40.00	***Solvent***	CHLOROFORM-d
Spectrum Offset (Hz)	5410.6274	***Sweep Width (Hz)***	16534.39	***Temperature (degree C)***	23.300		

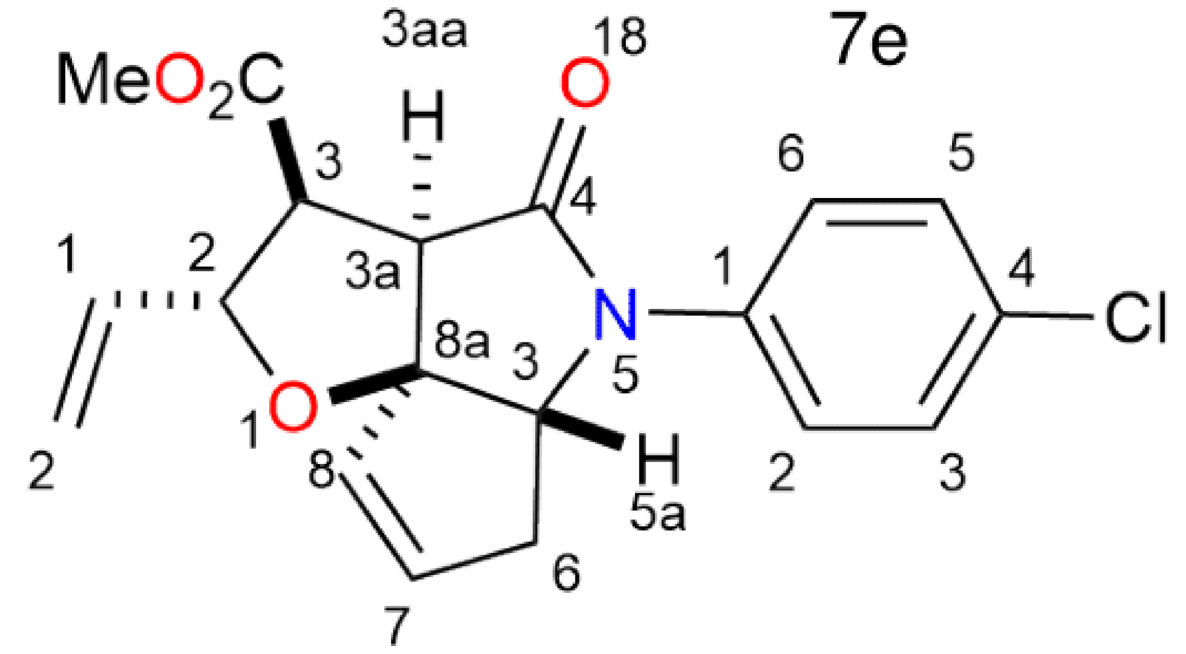

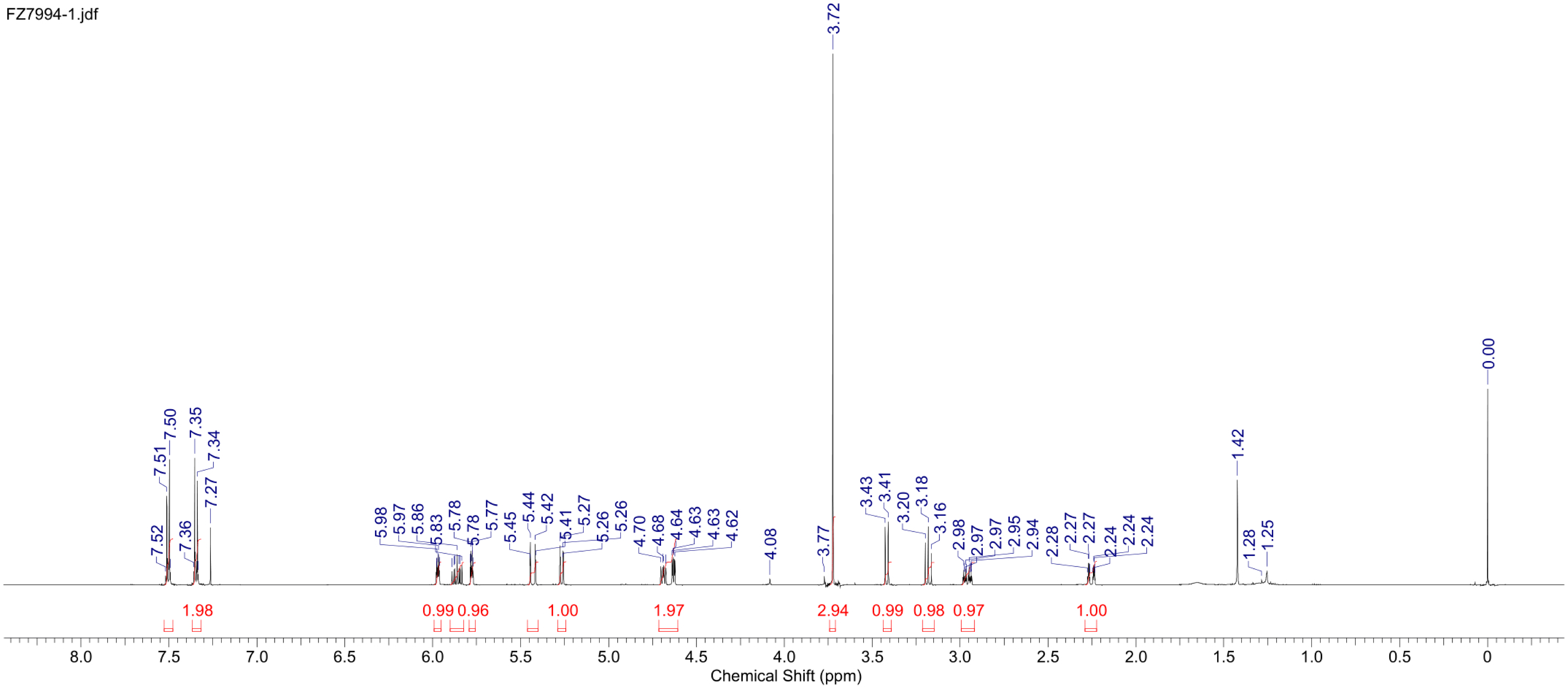

Acquisition Time (sec)	1.9818	Comment	single_pulse	Date	08 Aug 1990 06:29:56				
Date Stamp	18 Oct 2019 12:33:00			File Name	C:\Users\Fedor\Desktop\18.10.19\FZ7994-1.jdf			Frequency (MHz)	600.17
Nucleus	1H	Number of Transients	8	Origin	ECA 600	Original Points Count	32768	Owner	delta
Points Count	32768	Pulse Sequence	single_pulse.ex2			Receiver Gain	40.00	Solvent	CHLOROFORM-d
Spectrum Offset (Hz)	5410.6274	Sweep Width (Hz)	16534.39	Temperature (degree C)	23.300				

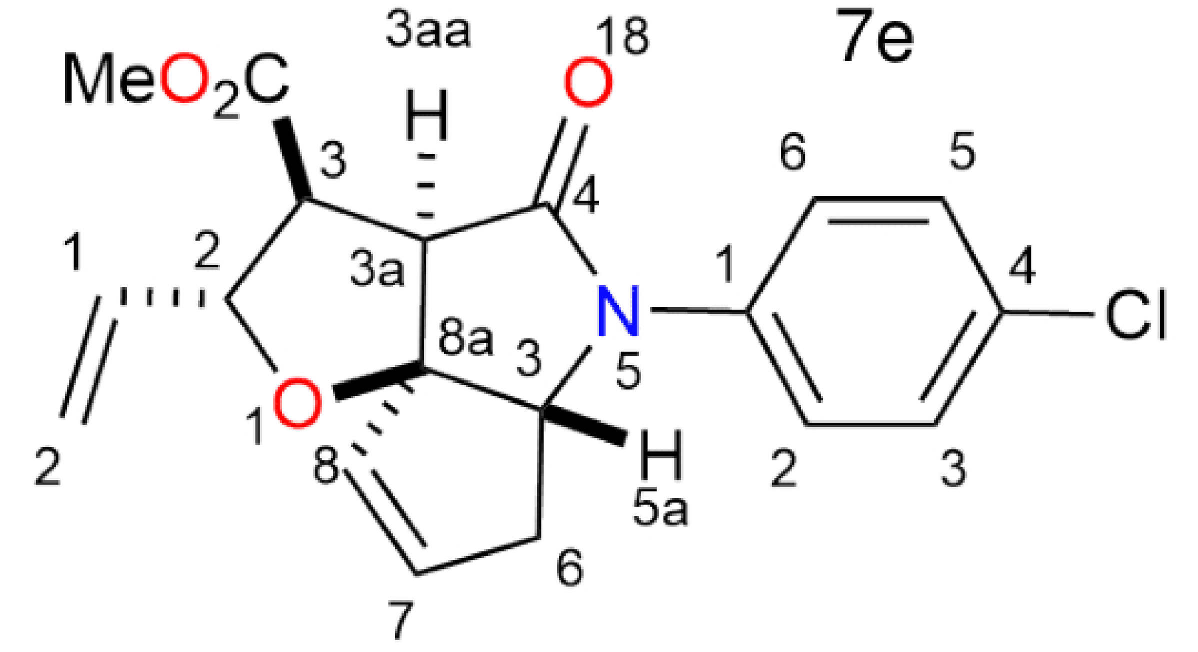

7e

FZ7994-1.jdf

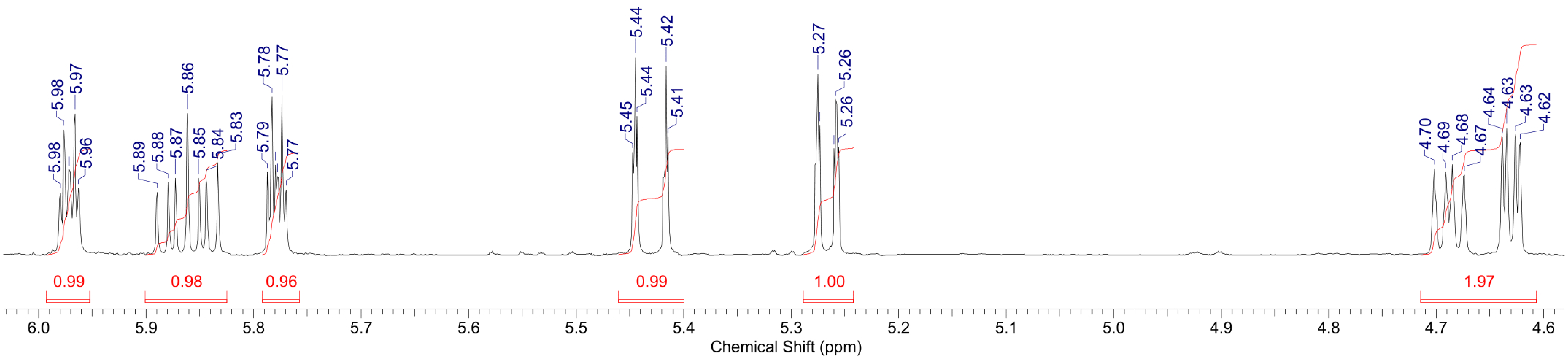

Acquisition Time (sec)	1.9818	***Comment***	single_pulse	***Date***	08 Aug 1990 06:29:56				
Date Stamp	18 Oct 2019 12:33:00			***File Name***	C:\Users\Fedor\Desktop\18.10.19\FZ7994-1.jdf			***Frequency (MHz)***	600.17
Nucleus	1H	***Number of Transients***	8	***Origin***	ECA 600	***Original Points Count***	32768	***Owner***	delta
Points Count	32768	***Pulse Sequence***	single_pulse.ex2			***Receiver Gain***	40.00	***Solvent***	CHLOROFORM-d
Spectrum Offset (Hz)	5410.6274	***Sweep Width (Hz)***	16534.39	***Temperature (degree C)***	23.300				

7e

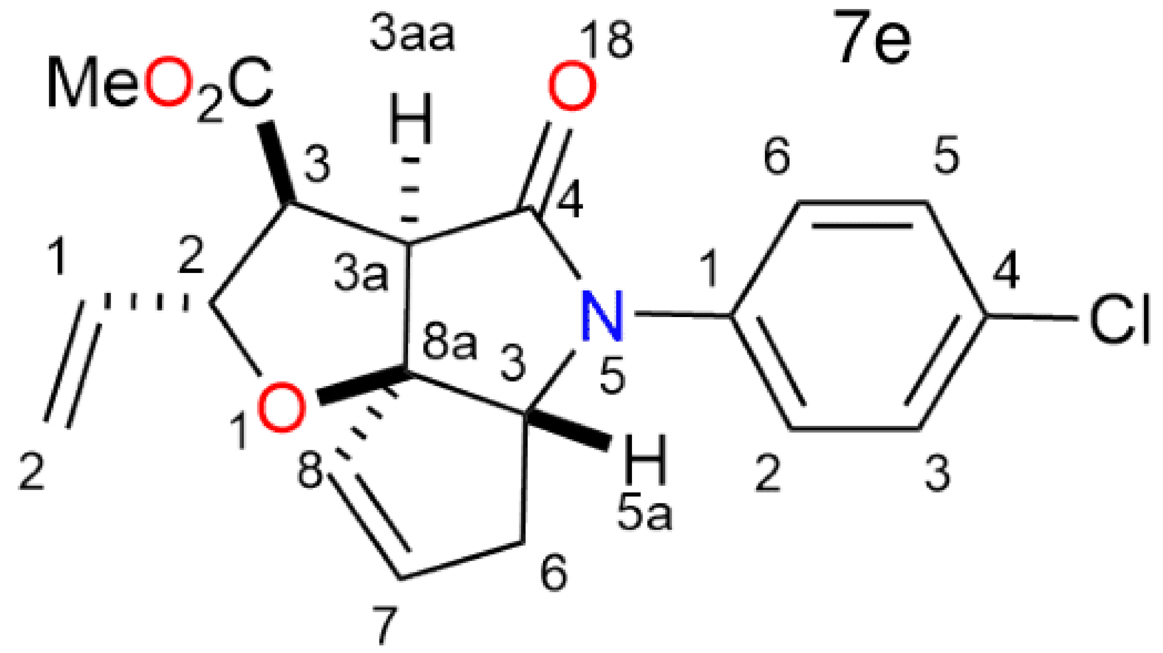

FZ7994-1.jdf

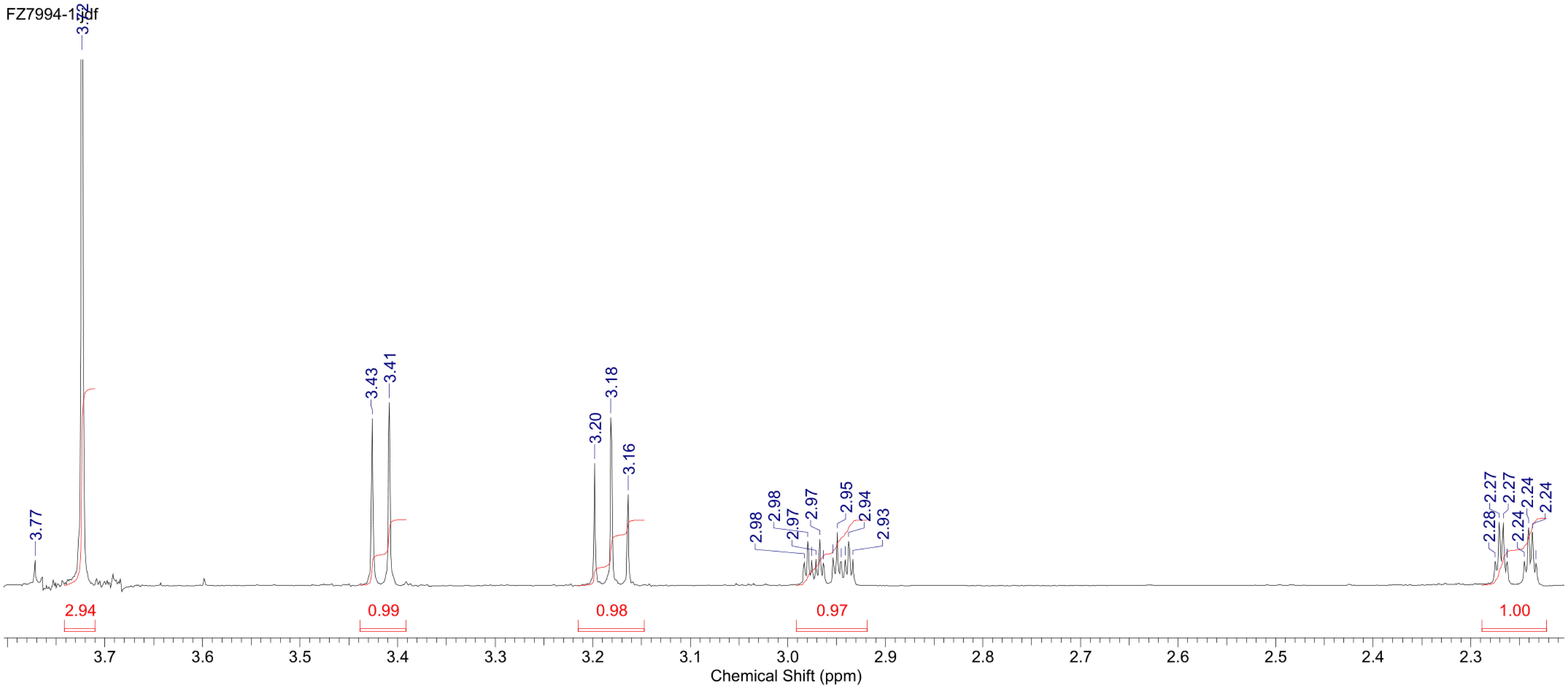

Acquisition Time (sec)	0.6921	***Comment***	single pulse decoupled gated NOE			***Date***	15 Aug 1990 07:21:36
Date Stamp	25 Oct 2019 13:24:11			***File Name***	C:\Users\Fedor\Desktop\25.10.19\FZ8038-1.jdf		
Frequency (MHz)	150.91	***Nucleus***	13C	***Number of Transients***	1000	***Origin***	ECA 600
Original Points Count	32768	***Owner***	delta	***Points Count***	32768	***Pulse Sequence***	single_pulse_dec
Receiver Gain	54.00	***Solvent***	CHLOROFORM-d			***Spectrum Offset (Hz)***	15079.3525
Sweep Width (Hz)	47348.49	***Temperature (degree C)***	24.700				

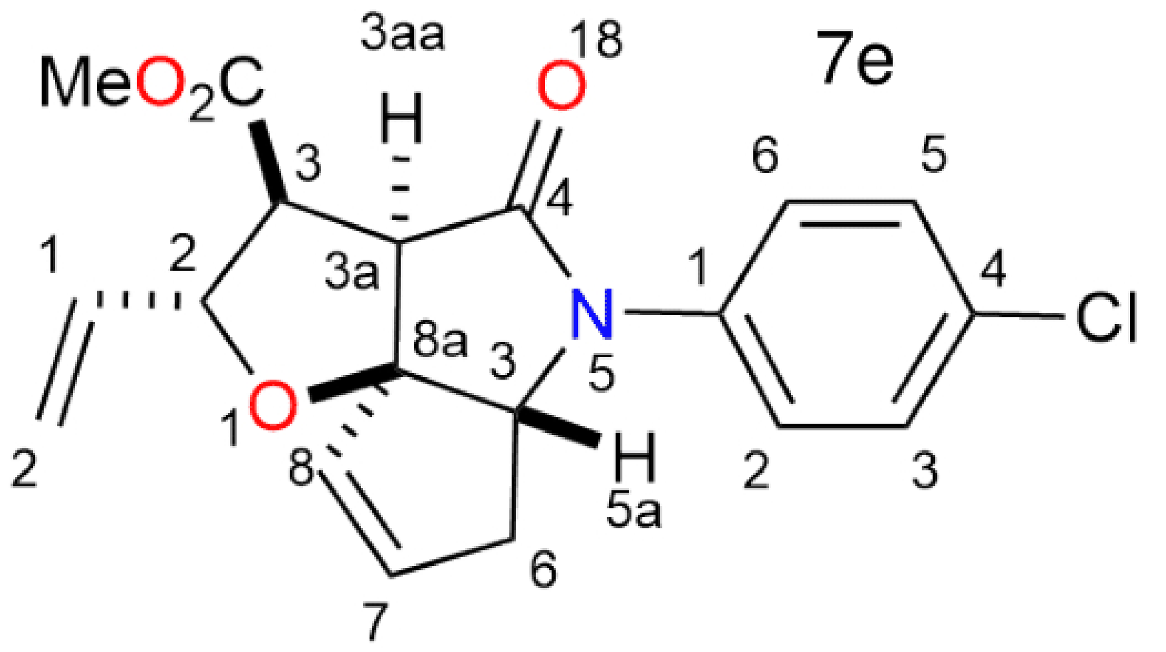

FZ8038-1.esp

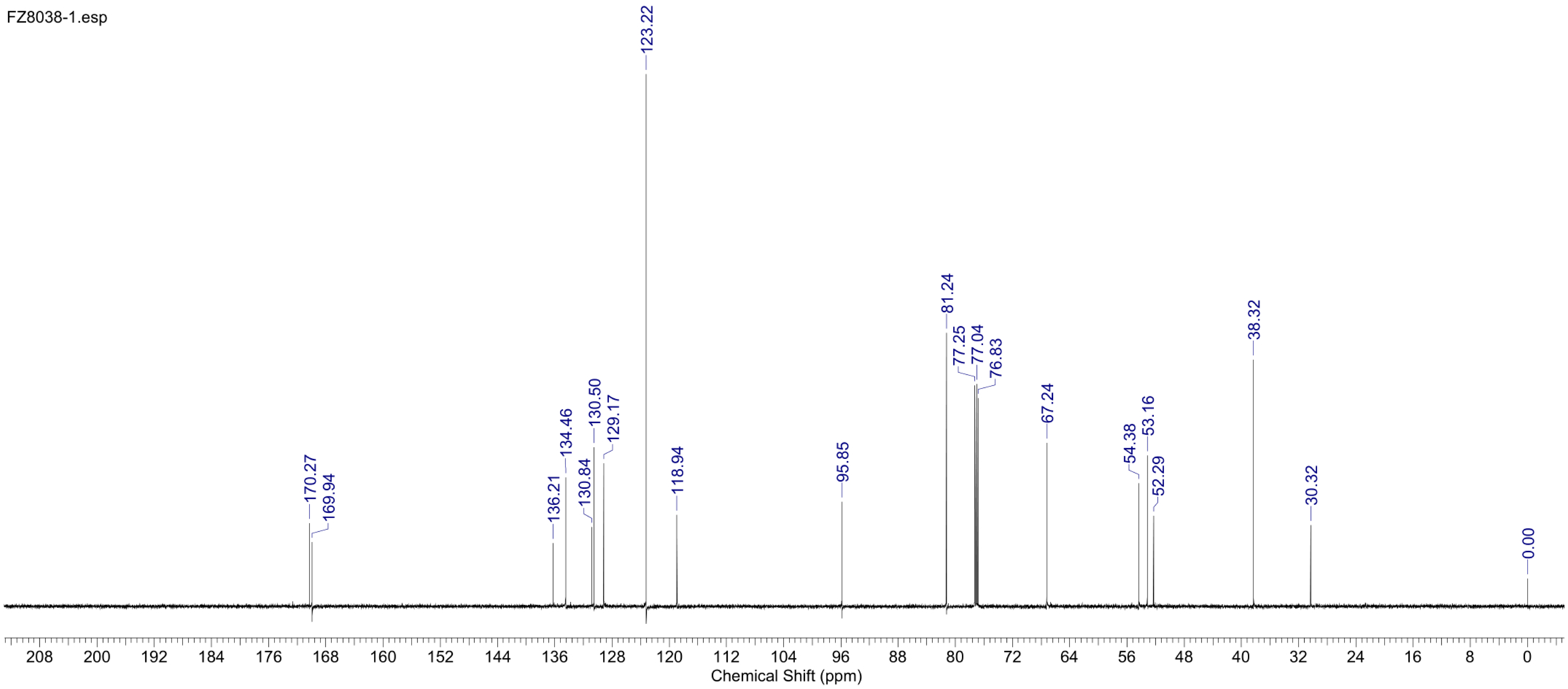

NMR spectrum data for compound 7f

Acquisition Time (sec)	1.9818	***Comment***	single_pulse	***Date***	28 May 1990 07:44:00				
Date Stamp	07 Aug 2019 13:47:25			***File Name***	C:\Users\Fedor\Desktop\07.08.19\FZ7652-1.jdf			***Frequency (MHz)***	600.17
Nucleus	1H	***Number of Transients***	8	***Origin***	ECA 600	***Original Points Count***	32768	***Owner***	delta
Points Count	32768	***Pulse Sequence***	single_pulse.ex2			***Receiver Gain***	32.00	***Solvent***	CHLOROFORM-d
Spectrum Offset (Hz)	5370.2588	***Sweep Width (Hz)***	16534.39	***Temperature (degree C)***	22.100				

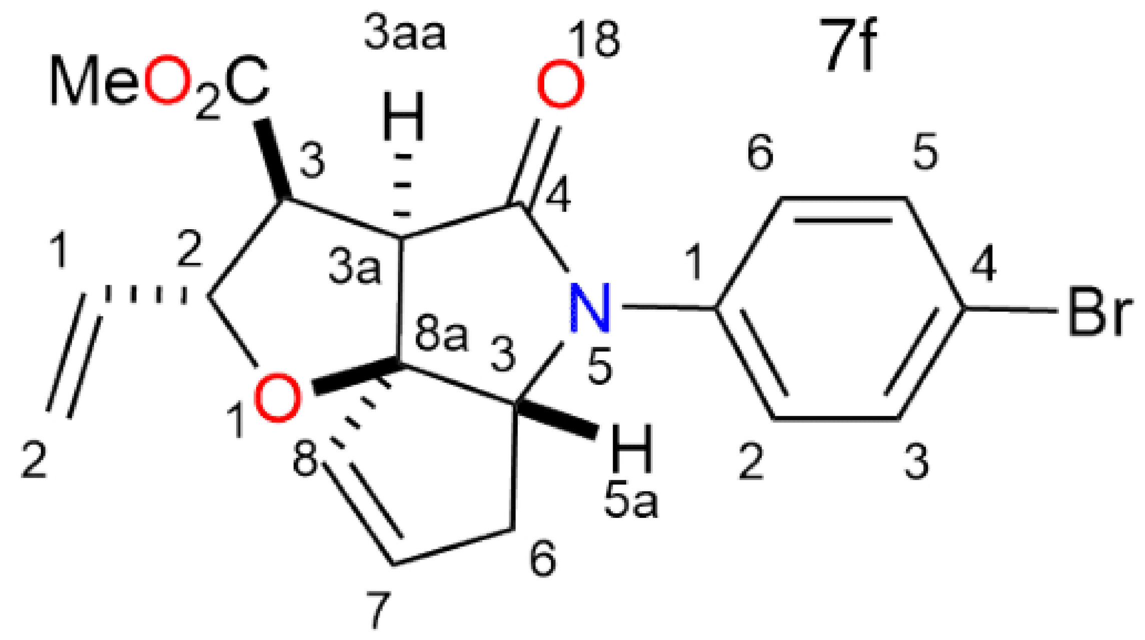

7f

FZ7652-1.jdf

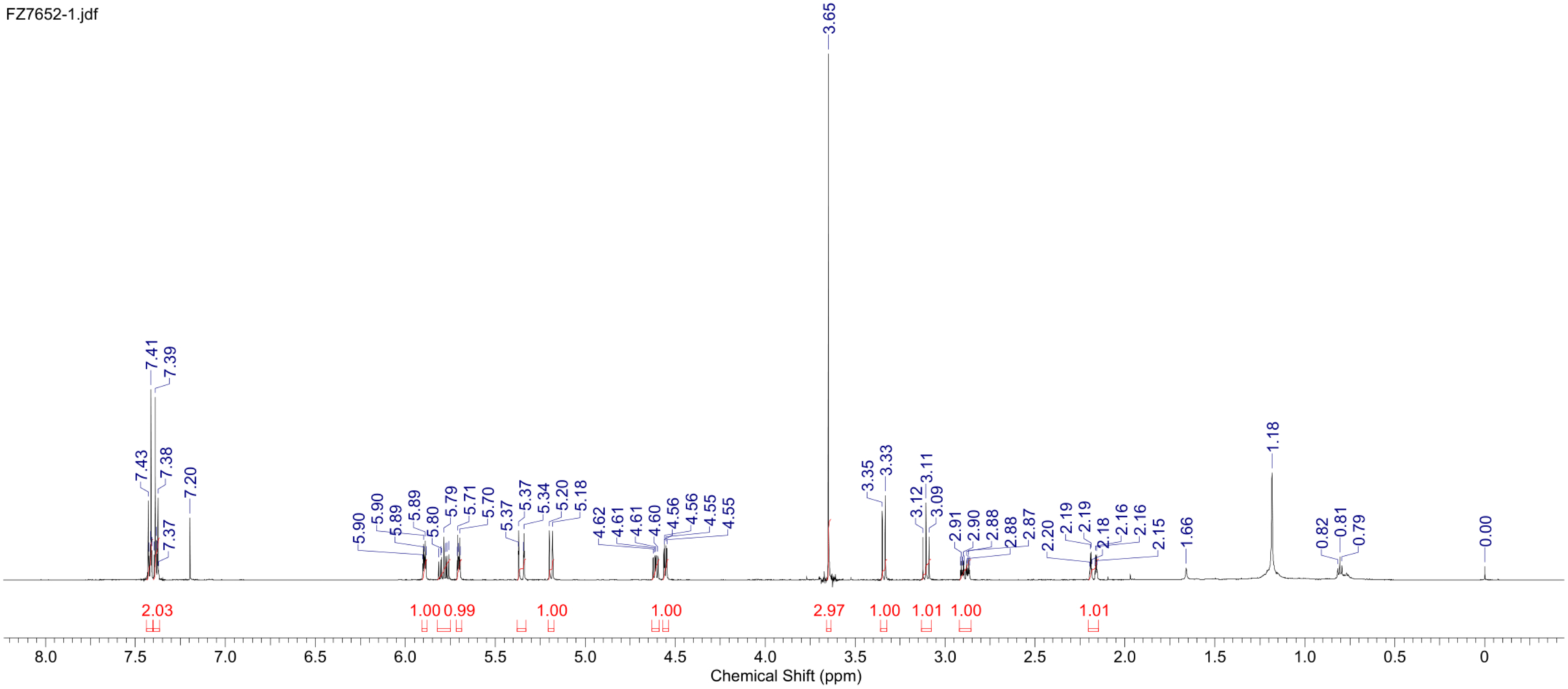

**Acquisition Time (sec)**	1.9818	**Comment**	single_pulse	**Date**	28 May 1990 07:44:00				
**Date Stamp**	07 Aug 2019 13:47:25			**File Name**	C:\Users\Fedor\Desktop\07.08.19\FZ7652-1.jdf			**Frequency (MHz)**	600.17
**Nucleus**	1H	**Number of Transients**	8	**Origin**	ECA 600	**Original Points Count**	32768	**Owner**	delta
**Points Count**	32768	**Pulse Sequence**	single_pulse.ex2			**Receiver Gain**	32.00	**Solvent**	CHLOROFORM-d
**Spectrum Offset (Hz)**	5370.2588	**Sweep Width (Hz)**	16534.39	**Temperature (degree C)**	22.100				

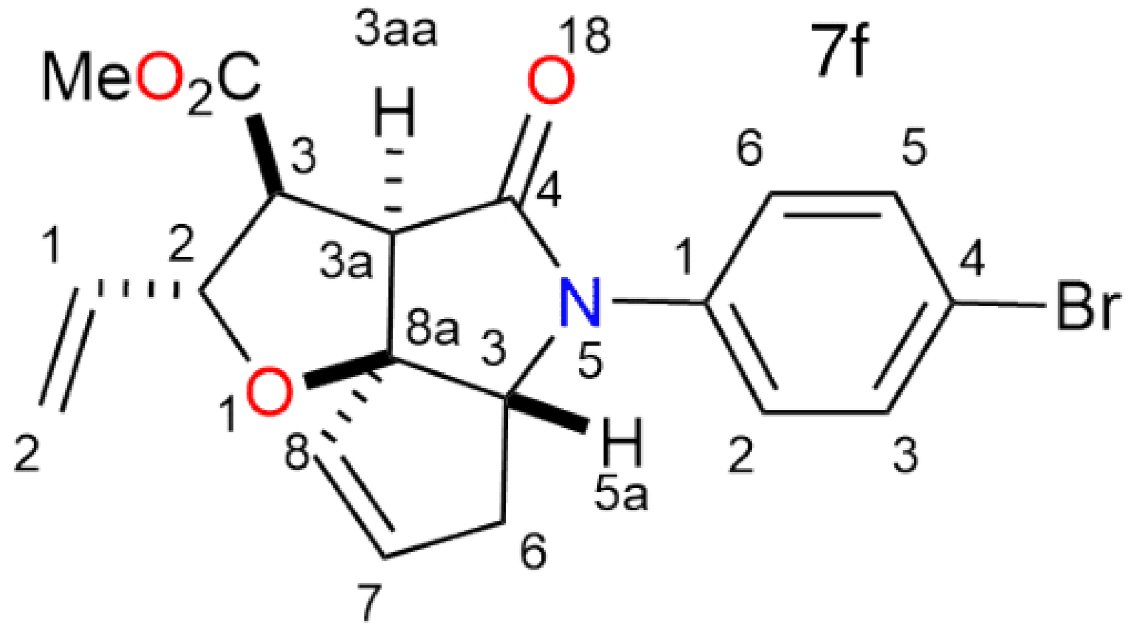

7f

FZ7652-1.jdf

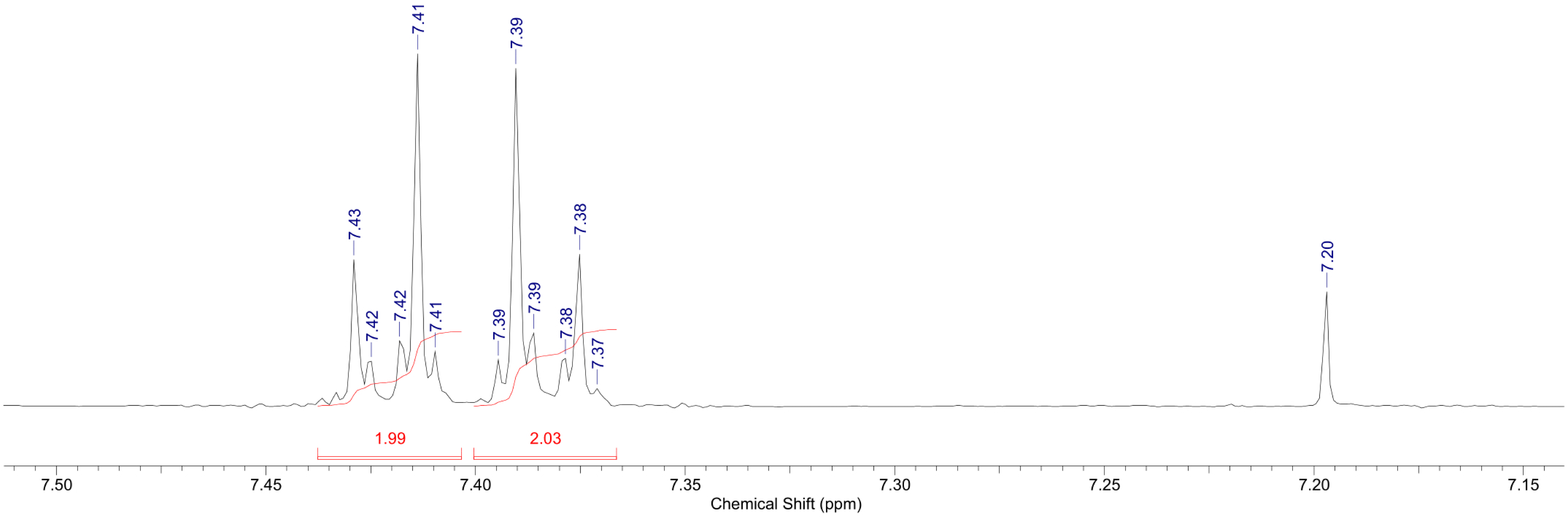

Acquisition Time (sec)	1.9818	***Comment***	single_pulse	***Date***	28 May 1990 07:44:00				
Date Stamp	07 Aug 2019 13:47:25			***File Name***	C:\Users\Fedor\Desktop\07.08.19\FZ7652-1.jdf			***Frequency (MHz)***	600.17
Nucleus	1H	***Number of Transients***	8	***Origin***	ECA 600	***Original Points Count***	32768	***Owner***	delta
Points Count	32768	***Pulse Sequence***	single_pulse.ex2			***Receiver Gain***	32.00	***Solvent***	CHLOROFORM-d
Spectrum Offset (Hz)	5370.2588	***Sweep Width (Hz)***	16534.39	***Temperature (degree C)***	22.100				

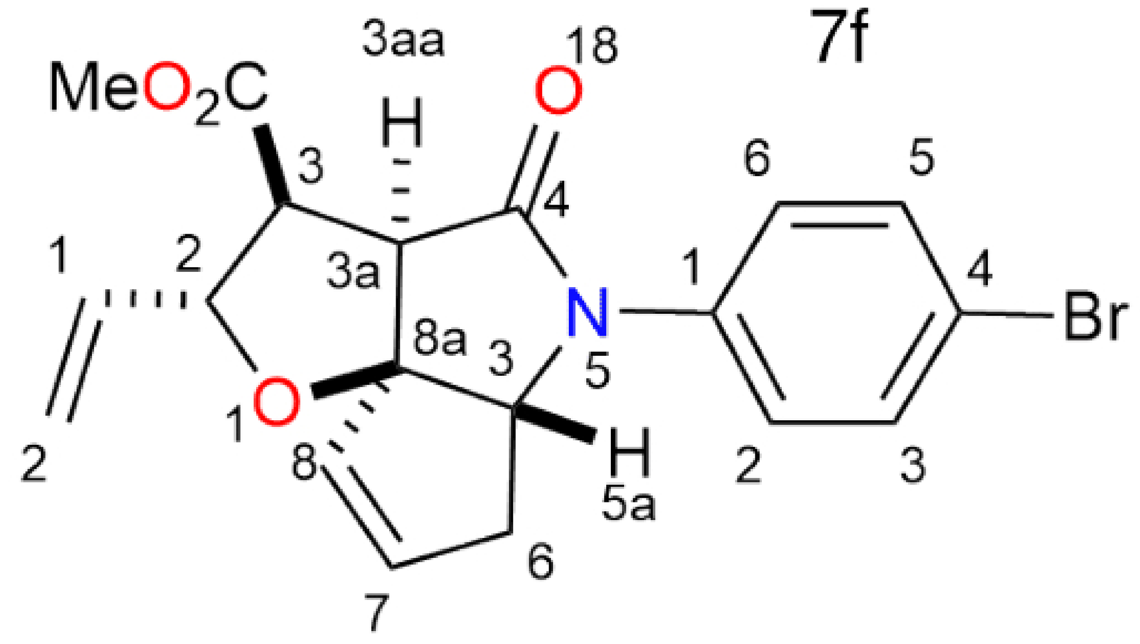

FZ7652-1.jdf

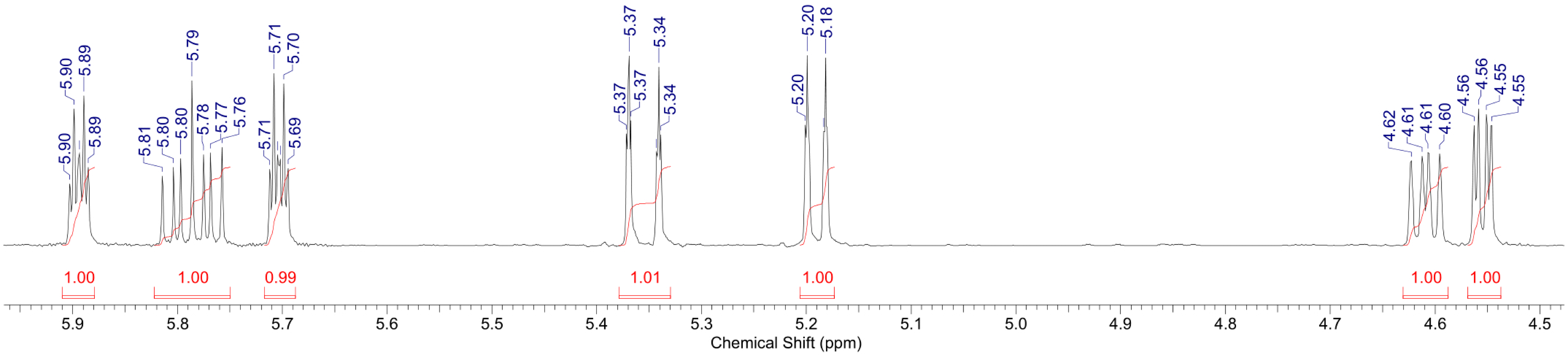

Acquisition Time (sec)	1.9818	***Comment***	single_pulse	***Date***	28 May 1990 07:44:00				
Date Stamp	07 Aug 2019 13:47:25			***File Name***	C:\Users\Fedor\Desktop\07.08.19\FZ7652-1.jdf			***Frequency (MHz)***	600.17
Nucleus	1H	***Number of Transients***	8	***Origin***	ECA 600	***Original Points Count***	32768	***Owner***	delta
Points Count	32768	***Pulse Sequence***	single_pulse.ex2			***Receiver Gain***	32.00	***Solvent***	CHLOROFORM-d
Spectrum Offset (Hz)	5370.2588	***Sweep Width (Hz)***	16534.39	***Temperature (degree C)***	22.100				

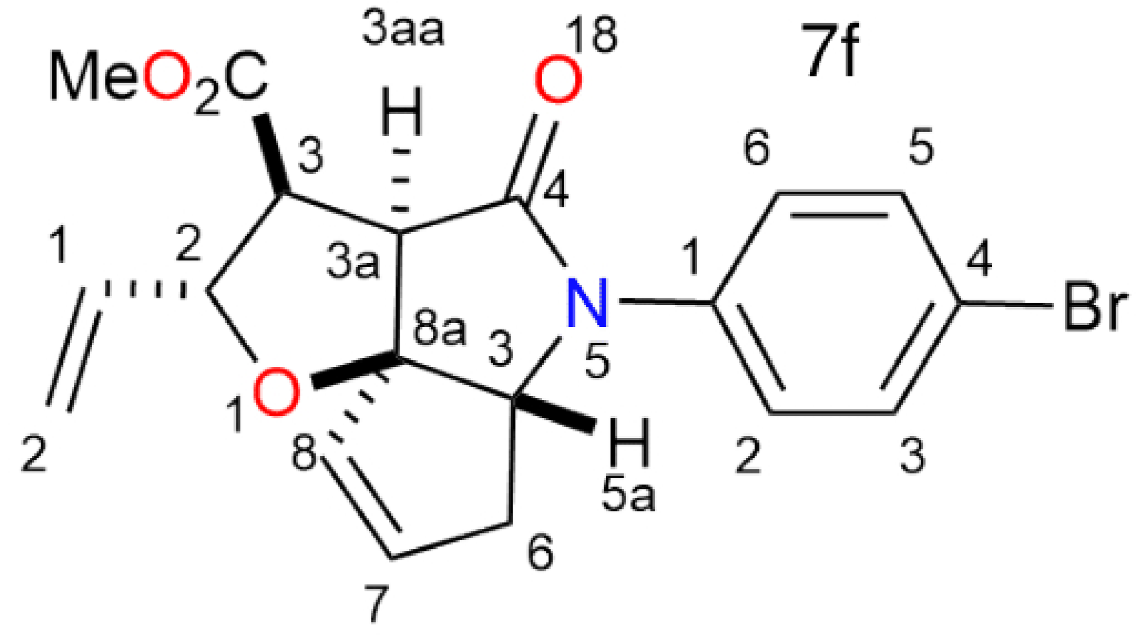

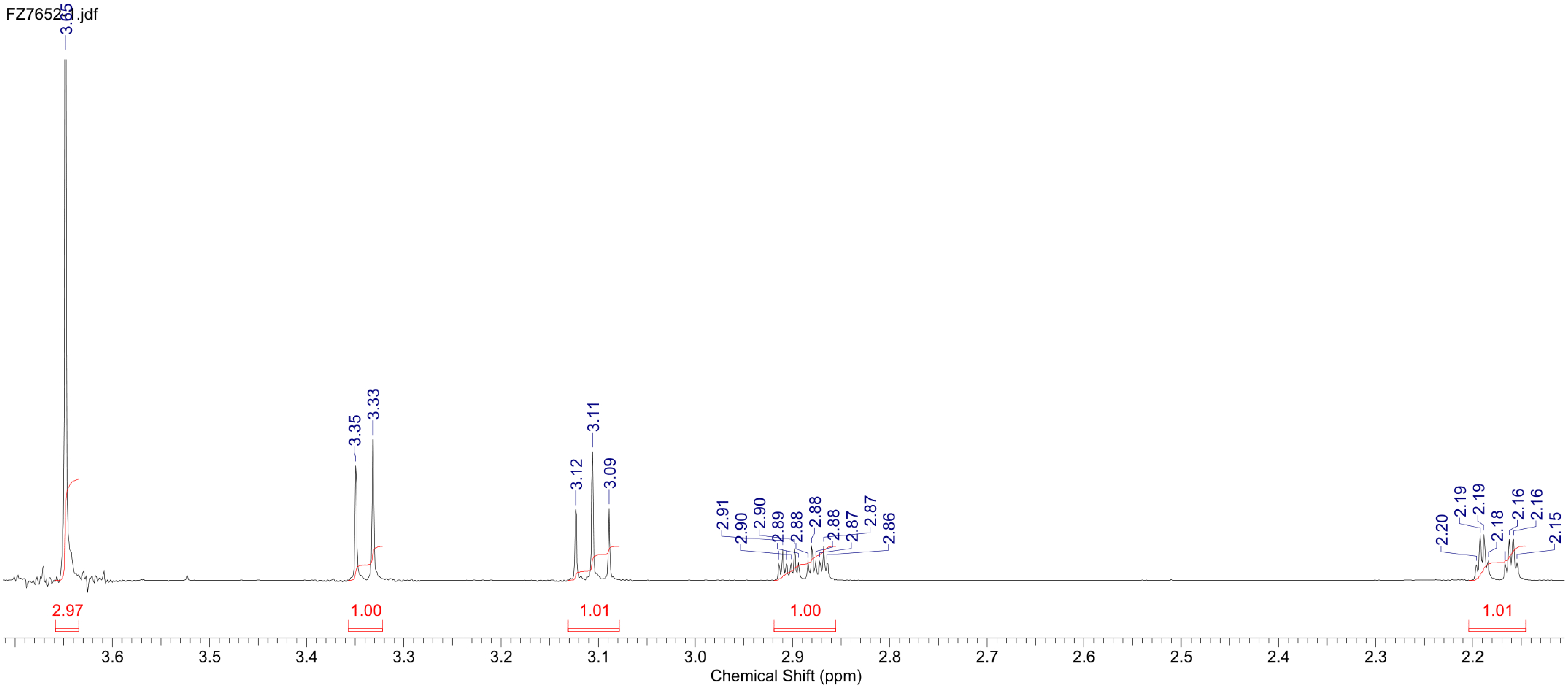

Acquisition Time (sec)	0.6921	***Comment***	single pulse decoupled gated NOE	***Date***	30 May 1990 06:09:56
Date Stamp	09 Aug 2019 12:13:24	***File Name***	C:\Users\Fedor\Desktop\09.08.19\FZ7669-1.jdf		
Frequency (MHz)	150.91	***Nucleus***	13C	***Number of Transients***	1000
Origin	ECA 600				
Original Points Count	32768	***Owner***	delta	***Points Count***	32768
Pulse Sequence	single_pulse_dec				
Receiver Gain	58.00	***Solvent***	CHLOROFORM-d	***Spectrum Offset (Hz)***	15091.3428
Sweep Width (Hz)	47348.49	***Temperature (degree C)***	23.400		

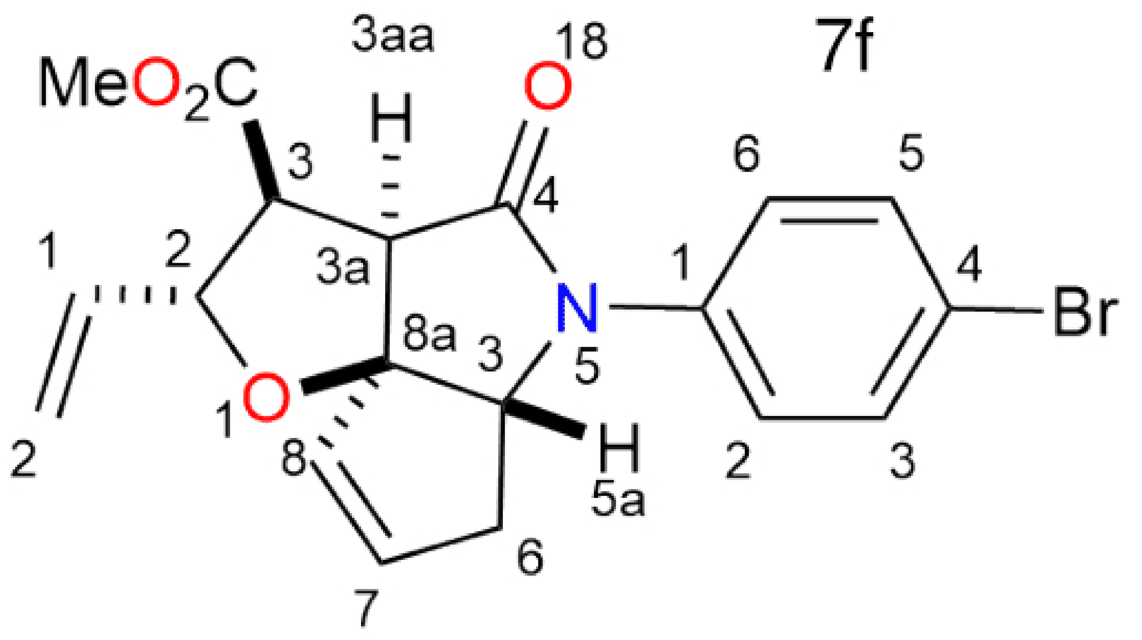

7f

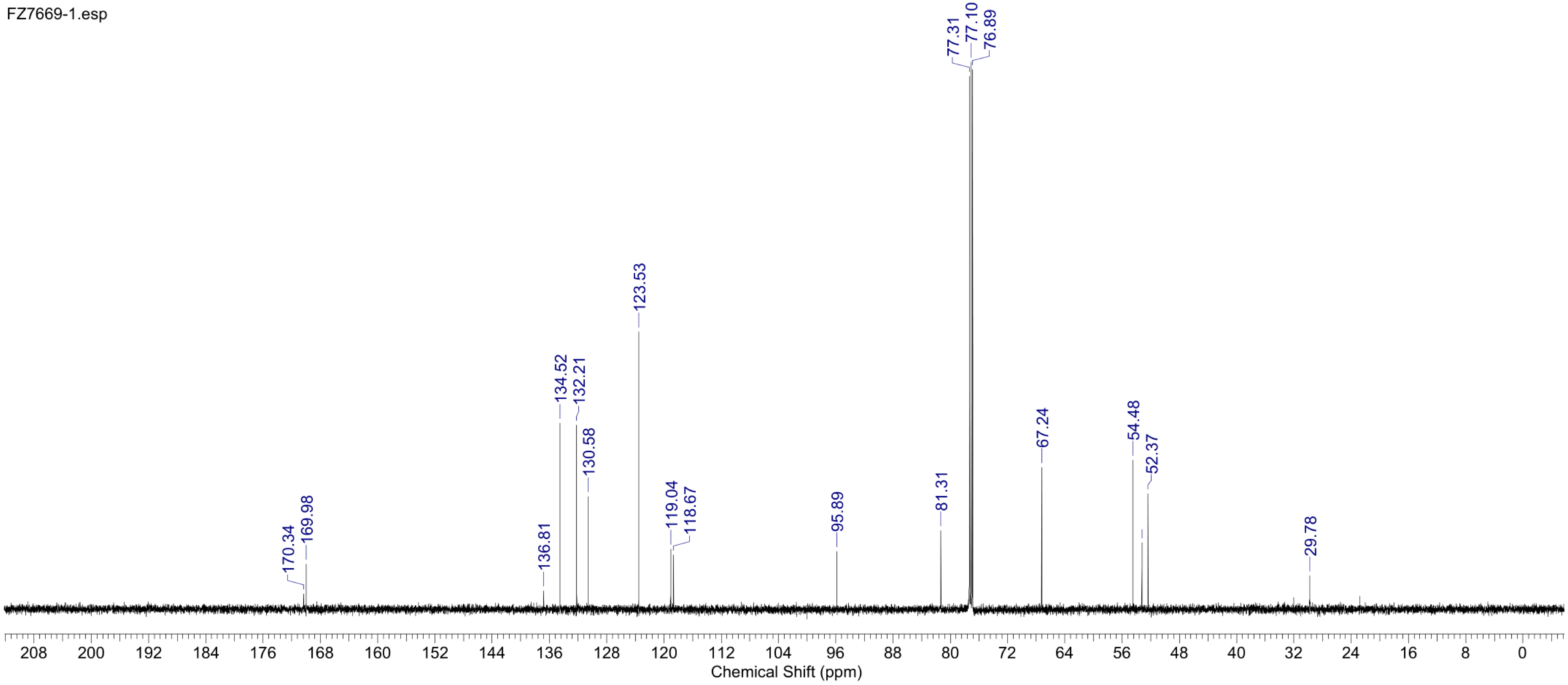

Acquisition Time (sec)	0.6921	***Comment***	single pulse decoupled gated NOE			***Date***	30 May 1990 06:09:56
Date Stamp	09 Aug 2019 12:13:24			***File Name***	C:\Users\Fedor\Desktop\09.08.19\FZ7669-1.jdf		
Frequency (MHz)	150.91	***Nucleus***	13C	***Number of Transients***	1000	***Origin***	ECA 600
Original Points Count	32768	***Owner***	delta	***Points Count***	32768	***Pulse Sequence***	single_pulse_dec
Receiver Gain	58.00	***Solvent***	CHLOROFORM-d			***Spectrum Offset (Hz)***	15091.3428
Sweep Width (Hz)	47348.49	***Temperature (degree C)***	23.400				

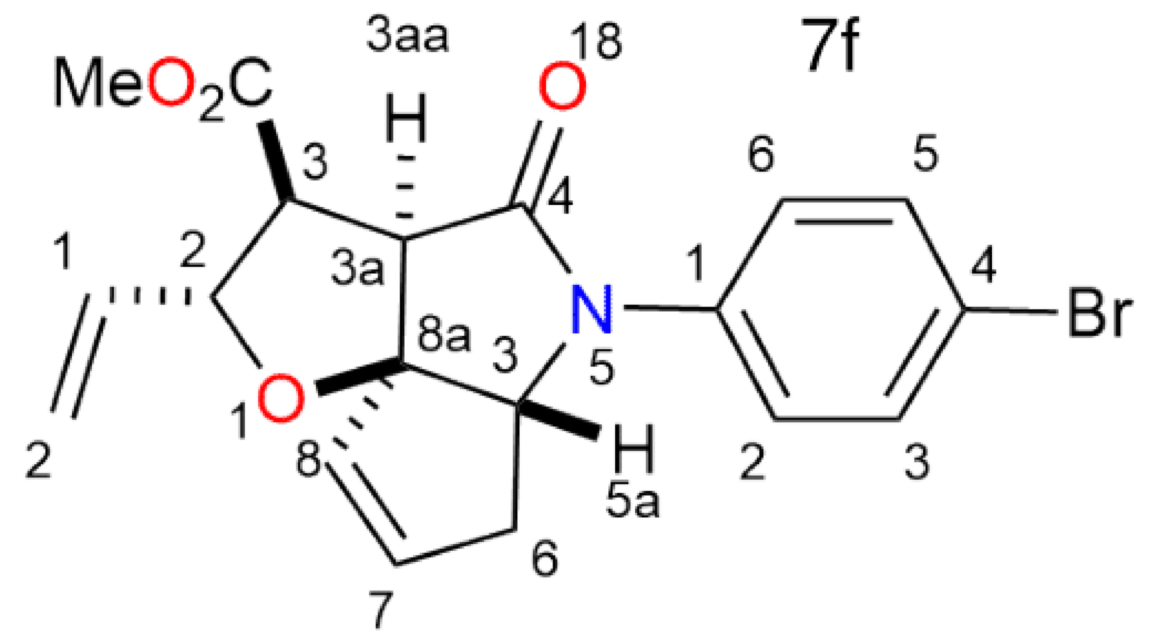

FZ7669-1.esp

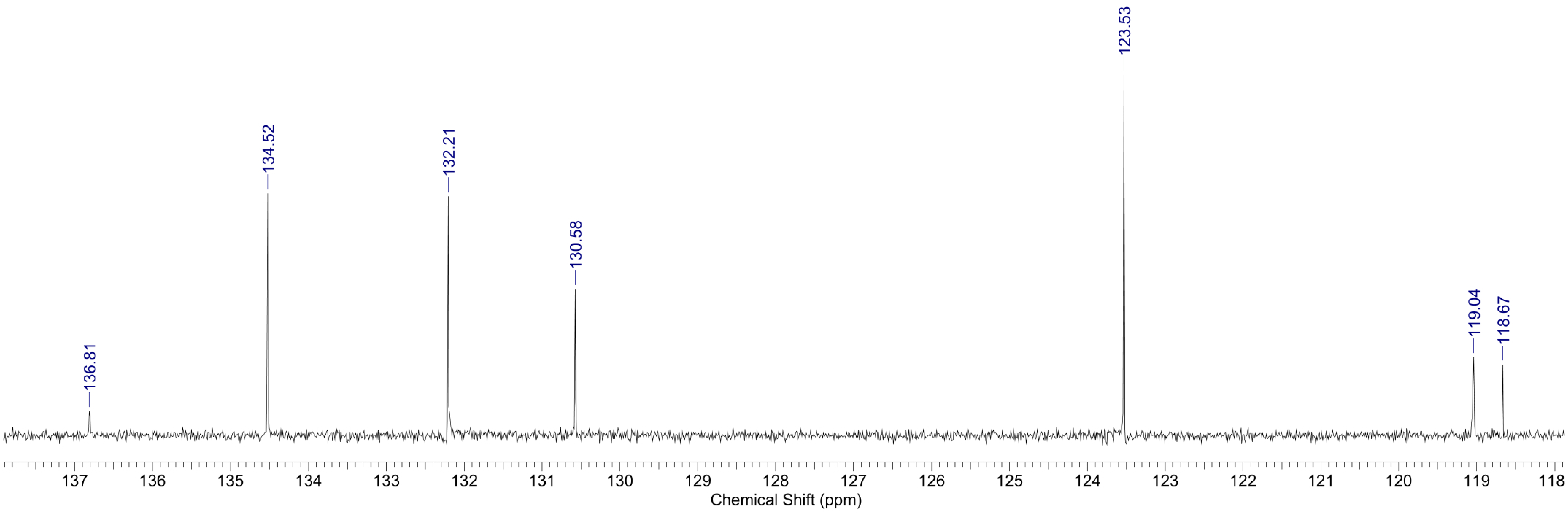

**Acquisition Time (sec)**	0.6921	**Comment**	single pulse decoupled gated NOE			**Date**	30 May 1990 06:09:56
**Date Stamp**	09 Aug 2019 12:13:24			**File Name**	C:\Users\Fedor\Desktop\09.08.19\FZ7669-1.jdf		
**Frequency (MHz)**	150.91	**Nucleus**	13C	**Number of Transients**	1000	**Origin**	ECA 600
**Original Points Count**	32768	**Owner**	delta	**Points Count**	32768	**Pulse Sequence**	single_pulse_dec
**Receiver Gain**	58.00	**Solvent**	CHLOROFORM-d			**Spectrum Offset (Hz)**	15091.3428
**Sweep Width (Hz)**	47348.49	**Temperature (degree C)**	23.400				

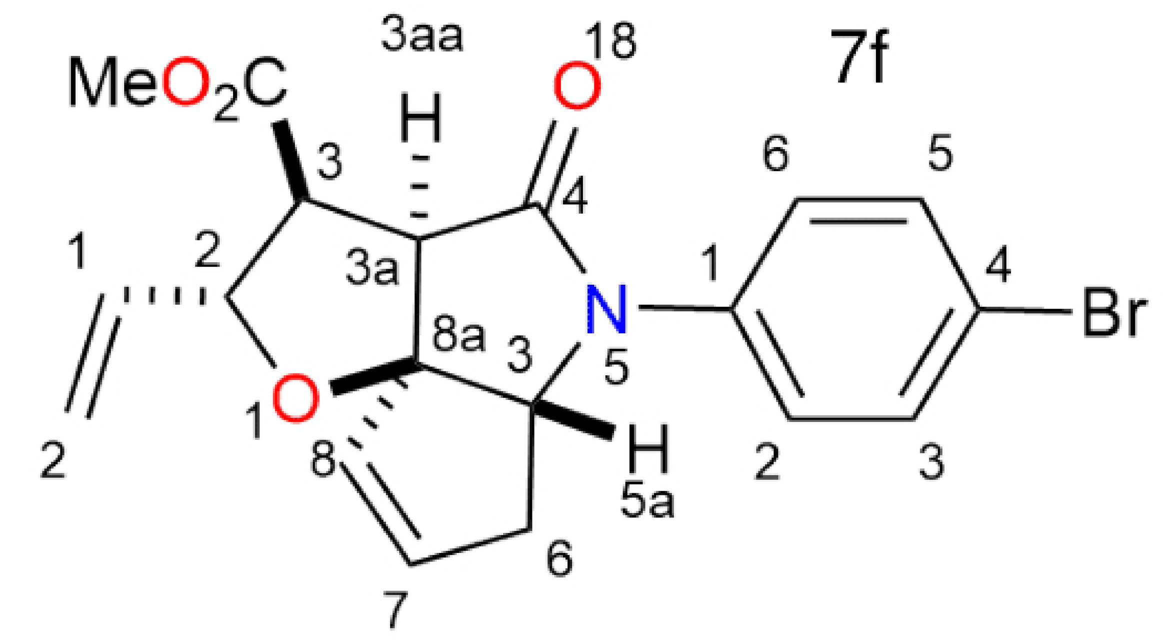

FZ7669-1.esp

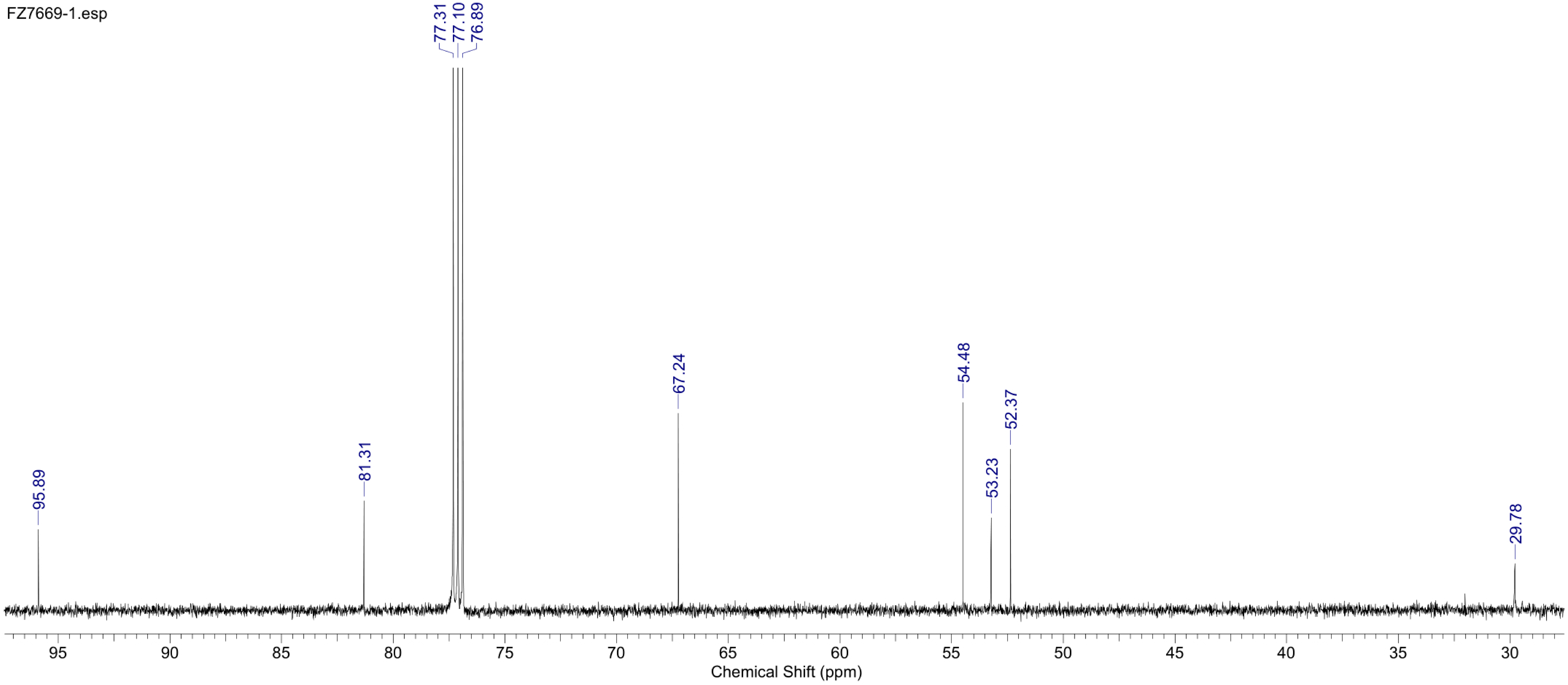

NMR spectrum data for compound 7g

26.11.2019 20:50:11

**Formula**	$C_{19}H_{18}INO_4$	**FW**	451.2550

**Acquisition Time (sec)**	1.9818	**Comment**	single_pulse	**Date**	06 Sep 1990 03:20:17		
**Date Stamp**	16 Nov 2019 08:37:27			**File Name**	C:\Users\User\Desktop\05.11.19\FZ8139-1.jdf	**Frequency (MHz)**	600.17
**Nucleus**	1H	**Number of Transients**	8	**Origin**	ECA 600	**Original Points Count** 32768	**Owner** delta
**Points Count**	32768	**Pulse Sequence**	single_pulse.ex2			**Receiver Gain** 36.00	**Solvent** CHLOROFORM-d
**Spectrum Offset (Hz)**	5410.6274	**Sweep Width (Hz)**	16534.39	**Temperature (degree C)**	21.700		

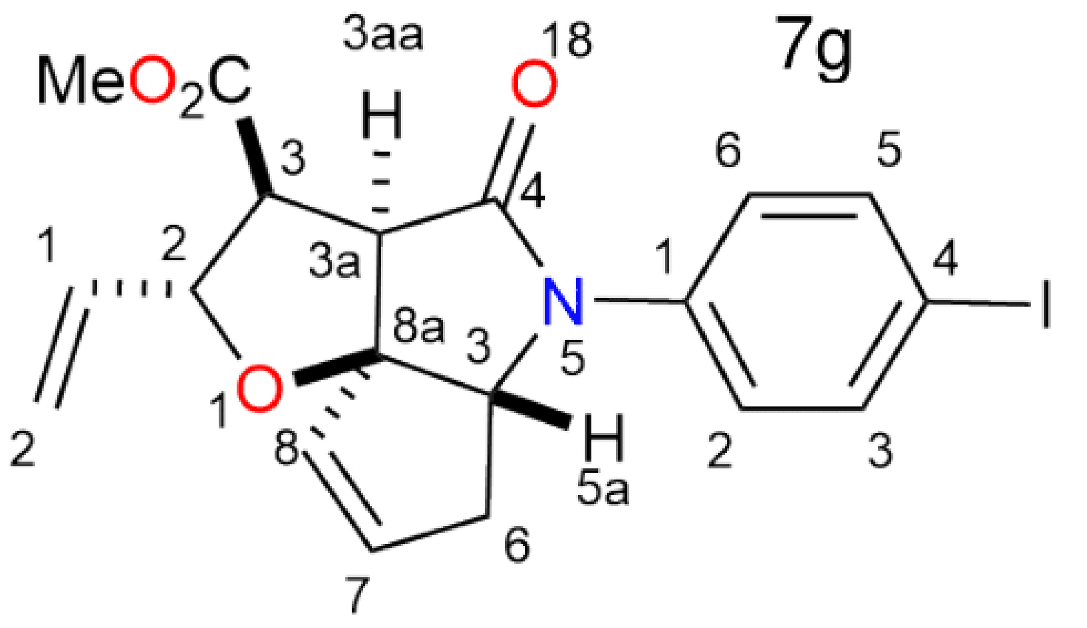

FZ8139-1.esp

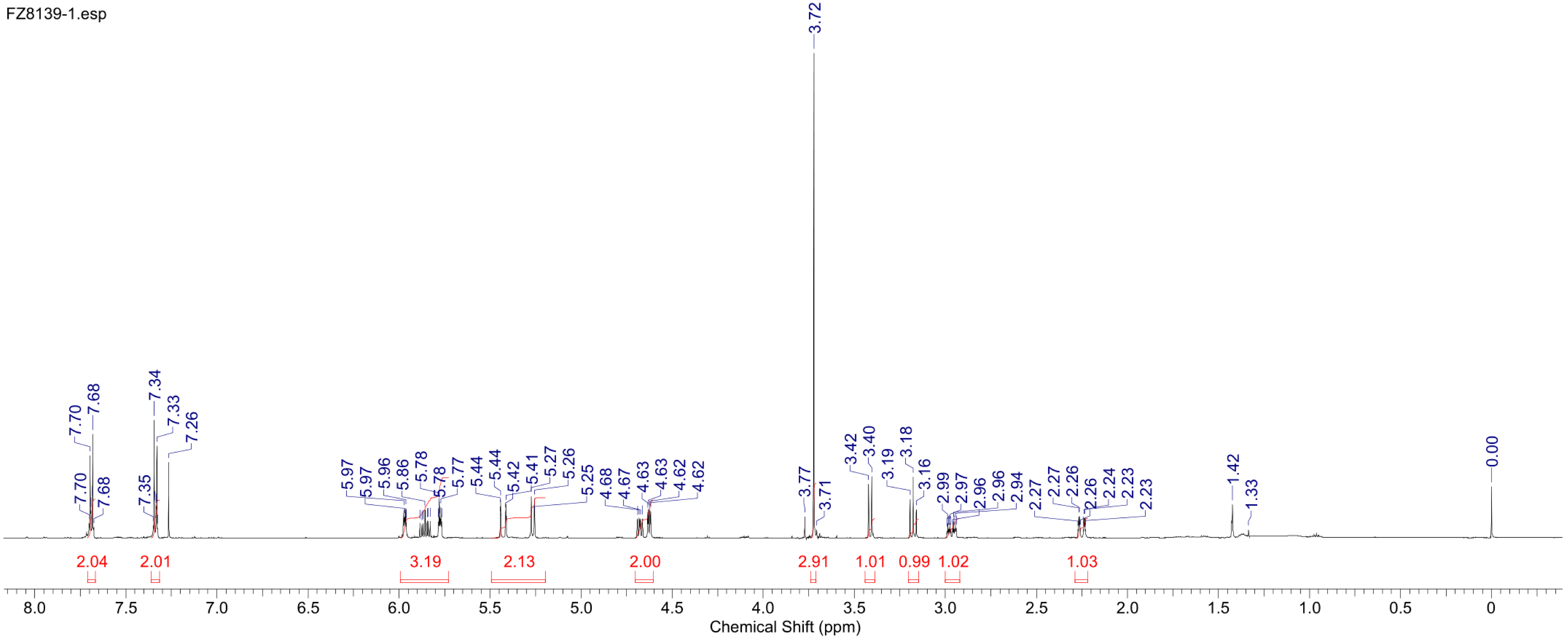

26.11.2019 20:50:35

Formula	$C_{19}H_{18}INO_4$	FW	451.2550

**Acquisition Time (sec)**	1.9818	**Comment**	single_pulse	**Date**	06 Sep 1990 03:20:17				
**Date Stamp**	16 Nov 2019 08:37:27			**File Name**	C:\Users\User\Desktop\05.11.19\FZ8139-1.jdf			**Frequency (MHz)**	600.17
**Nucleus**	1H	**Number of Transients**	8	**Origin**	ECA 600	**Original Points Count**	32768	**Owner**	delta
**Points Count**	32768	**Pulse Sequence**	single_pulse.ex2			**Receiver Gain**	36.00	**Solvent**	CHLOROFORM-d
**Spectrum Offset (Hz)**	5410.6274	**Sweep Width (Hz)**	16534.39	**Temperature (degree C)**	21.700				

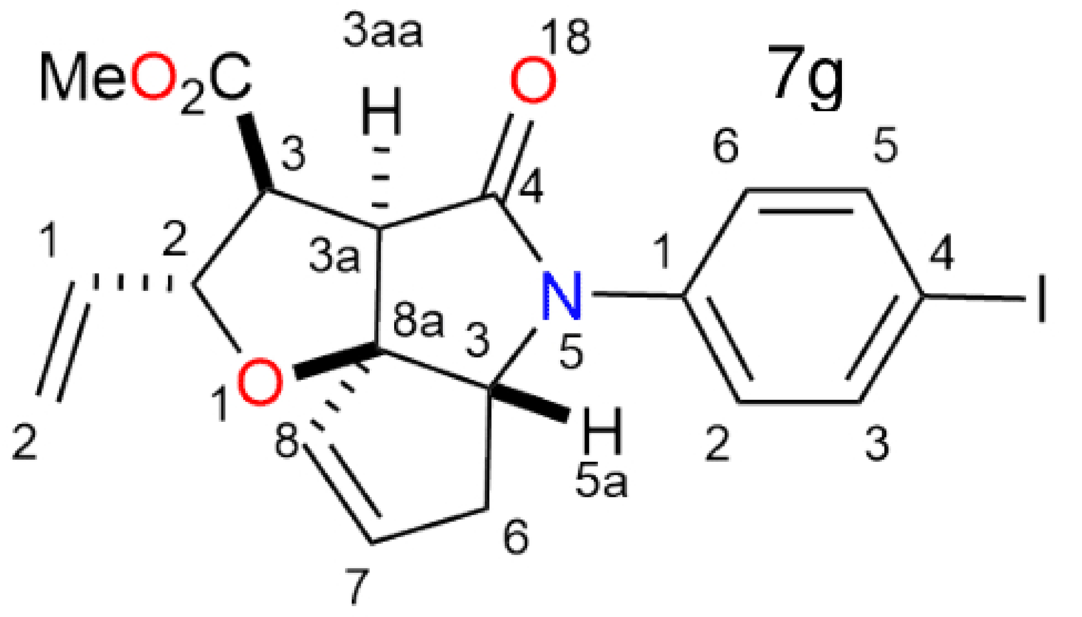

FZ8139-1.esp

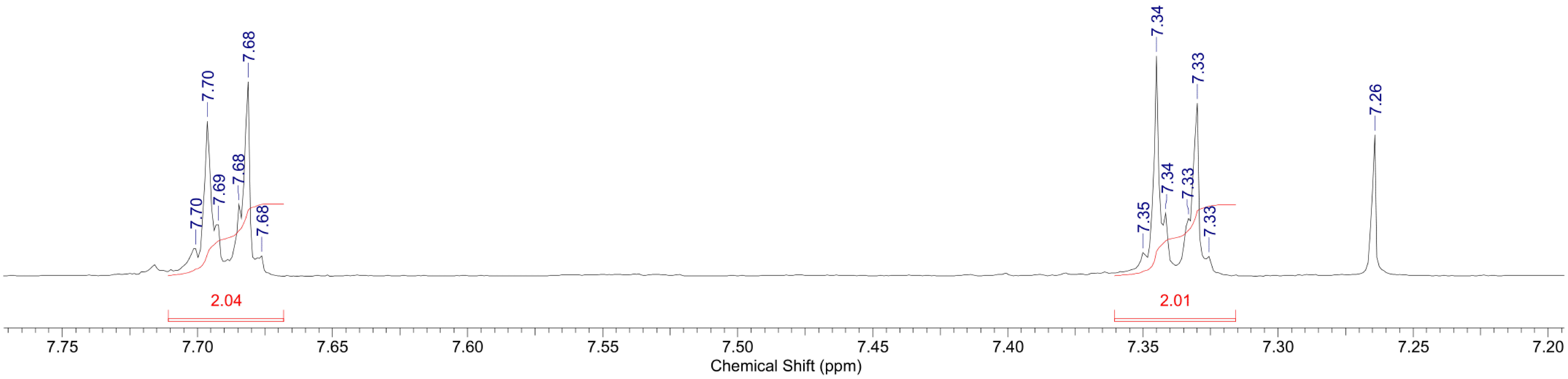

26.11.2019 20:51:02

**Formula**	$C_{19}H_{18}INO_4$	**FW**	451.2550

**Acquisition Time (sec)**	1.9818	**Comment**	single_pulse	**Date**	06 Sep 1990 03:20:17				
**Date Stamp**	16 Nov 2019 08:37:27			**File Name**	C:\Users\User\Desktop\05.11.19\FZ8139-1.jdf			**Frequency (MHz)**	600.17
**Nucleus**	1H	**Number of Transients**	8	**Origin**	ECA 600	**Original Points Count**	32768	**Owner**	delta
**Points Count**	32768	**Pulse Sequence**	single_pulse.ex2			**Receiver Gain**	36.00	**Solvent**	CHLOROFORM-d
**Spectrum Offset (Hz)**	5410.6274	**Sweep Width (Hz)**	16534.39	**Temperature (degree C)**	21.700				

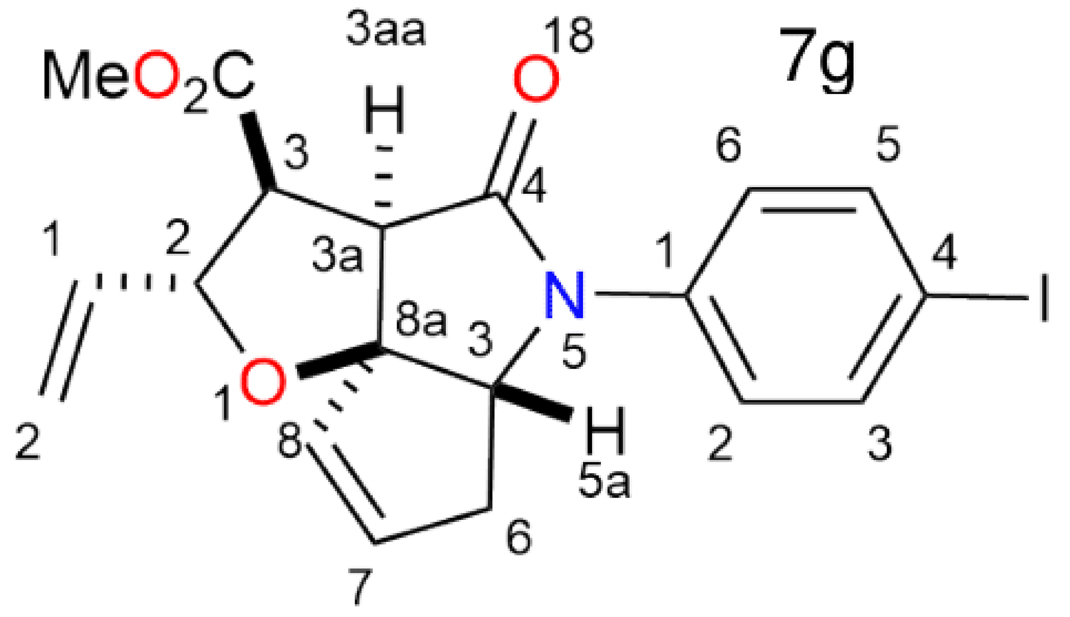

FZ8139-1.esp

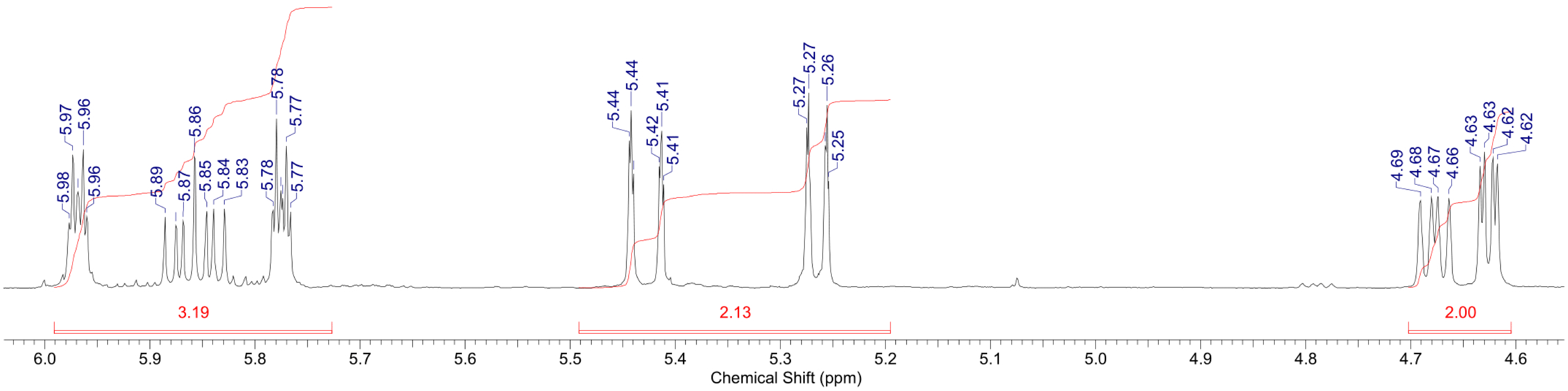

26.11.2019 20:51:28

Formula	$C_{19}H_{18}INO_4$	FW	451.2550

**Acquisition Time (sec)**	1.9818	**Comment**	single_pulse	**Date**	06 Sep 1990 03:20:17		
**Date Stamp**	16 Nov 2019 08:37:27			**File Name**	C:\Users\User\Desktop\05.11.19\FZ8139-1.jdf	**Frequency (MHz)**	600.17
**Nucleus**	1H	**Number of Transients**	8	**Origin**	ECA 600	**Original Points Count** 32768	**Owner** delta
**Points Count**	32768	**Pulse Sequence**	single_pulse.ex2			**Receiver Gain** 36.00	**Solvent** CHLOROFORM-d
**Spectrum Offset (Hz)**	5410.6274	**Sweep Width (Hz)**	16534.39	**Temperature (degree C)**	21.700		

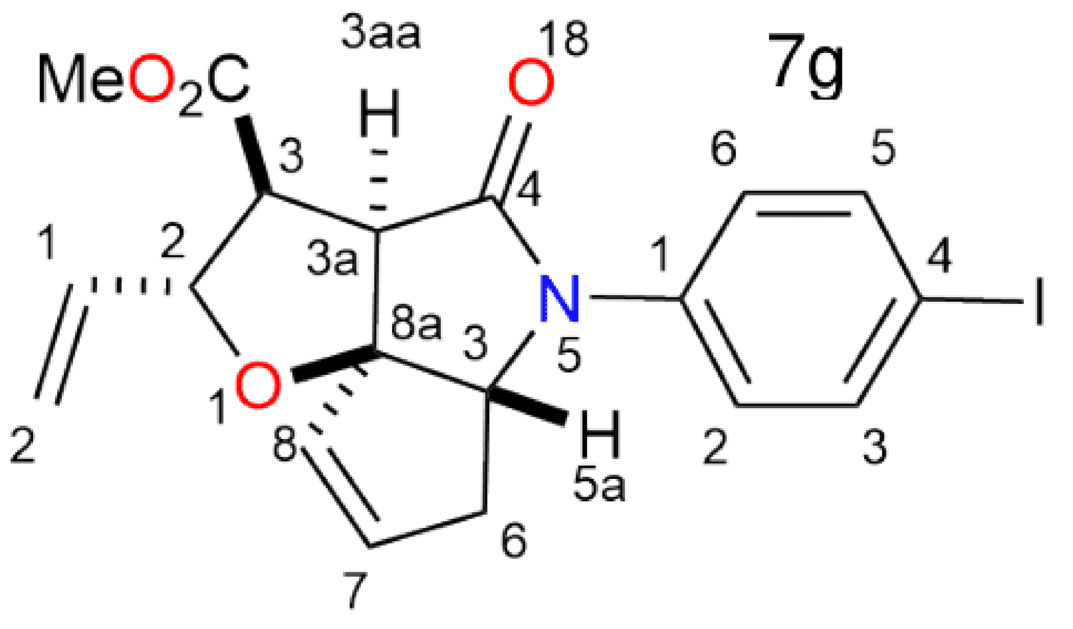

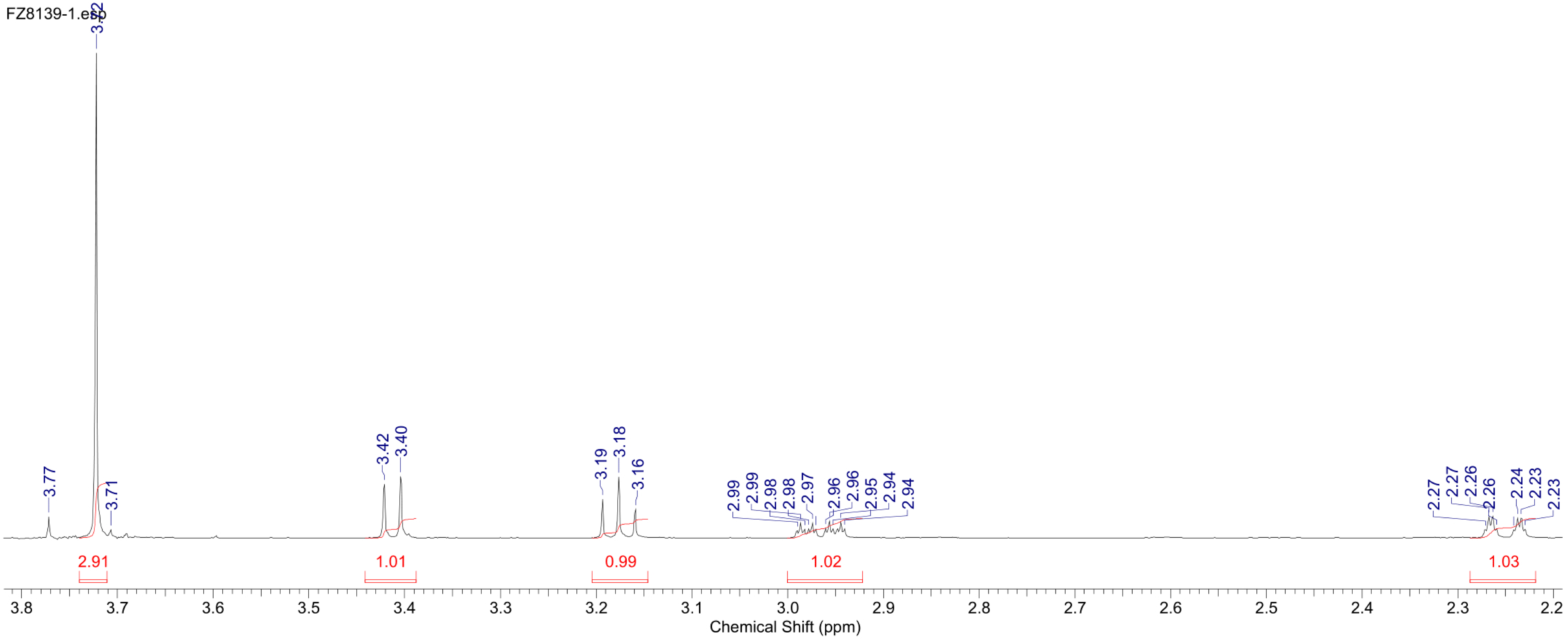

**Formula** $C_{19}H_{18}INO_4$	**FW** 451.2550

**Acquisition Time (sec)**	0.6921	**Comment**	single pulse decoupled gated NOE			**Date**	19 Sep 1990 09:43:12
**Date Stamp**	29 Nov 2019 15:00:37			**File Name**	C:\Users\User\Desktop\02.12.19\FZ8206-1.jdf		
**Frequency (MHz)**	150.91	**Nucleus**	13C	**Number of Transients**	1000	**Origin**	ECA 600
**Original Points Count**	32768	**Owner**	delta	**Points Count**	32768	**Pulse Sequence**	single_pulse_dec
**Receiver Gain**	54.00	**Solvent**	CHLOROFORM-d			**Spectrum Offset (Hz)**	15079.3525
**Sweep Width (Hz)**	47348.49	**Temperature (degree C)**	23.900				

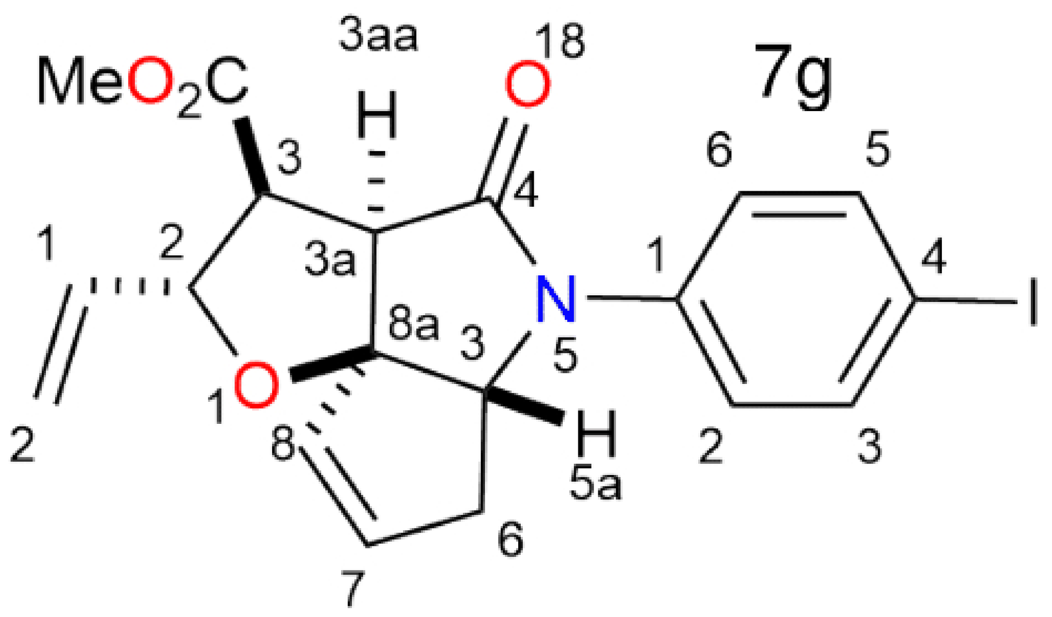

FZ8206-1.esp

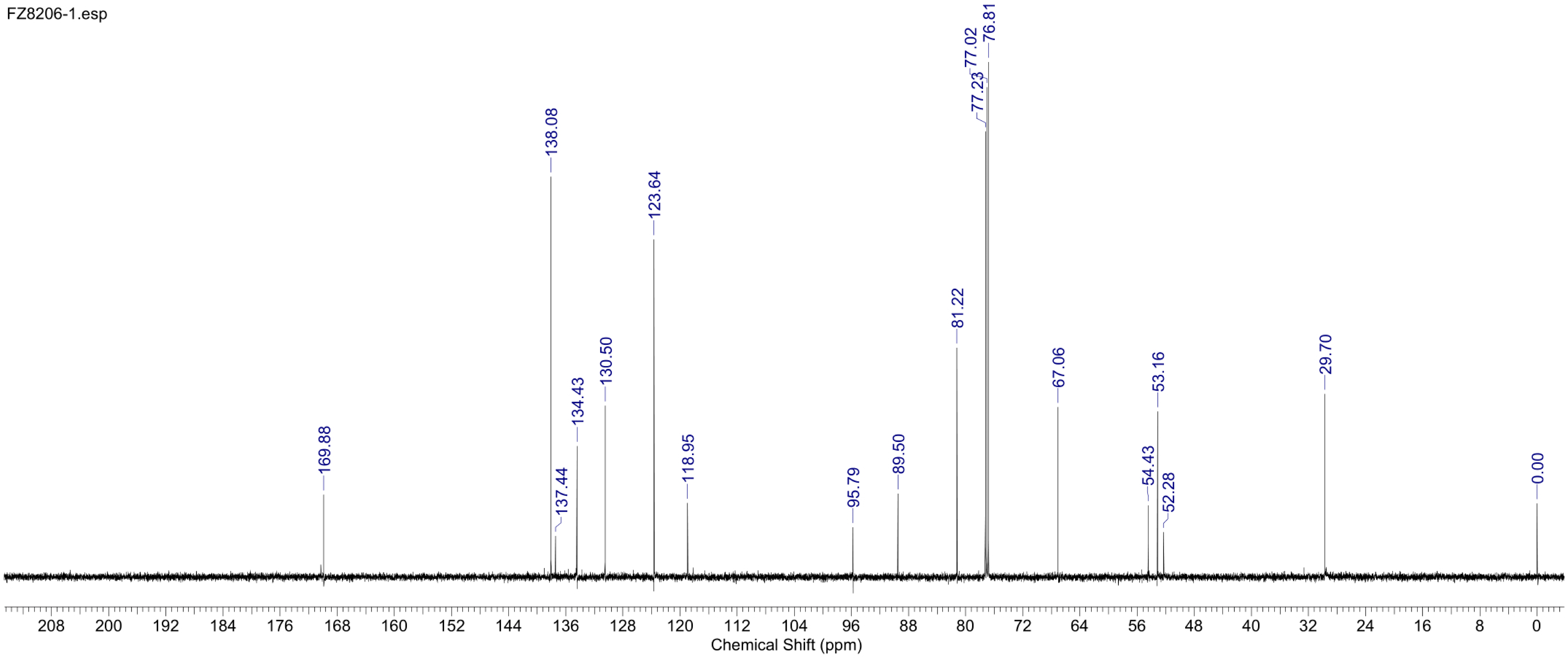

Formula $C_{19}H_{18}INO_4$	FW 451.2550

**Acquisition Time (sec)**	0.6921	**Comment**	single pulse decoupled gated NOE			**Date**	19 Sep 1990 09:43:12
**Date Stamp**	29 Nov 2019 15:00:37			**File Name**	C:\Users\User\Desktop\02.12.19\FZ8206-1.jdf		
**Frequency (MHz)**	150.91	**Nucleus**	13C	**Number of Transients**	1000	**Origin**	ECA 600
**Original Points Count**	32768	**Owner**	delta	**Points Count**	32768	**Pulse Sequence**	single_pulse_dec
**Receiver Gain**	54.00	**Solvent**	CHLOROFORM-d			**Spectrum Offset (Hz)**	15079.3525
**Sweep Width (Hz)**	47348.49	**Temperature (degree C)**	23.900				

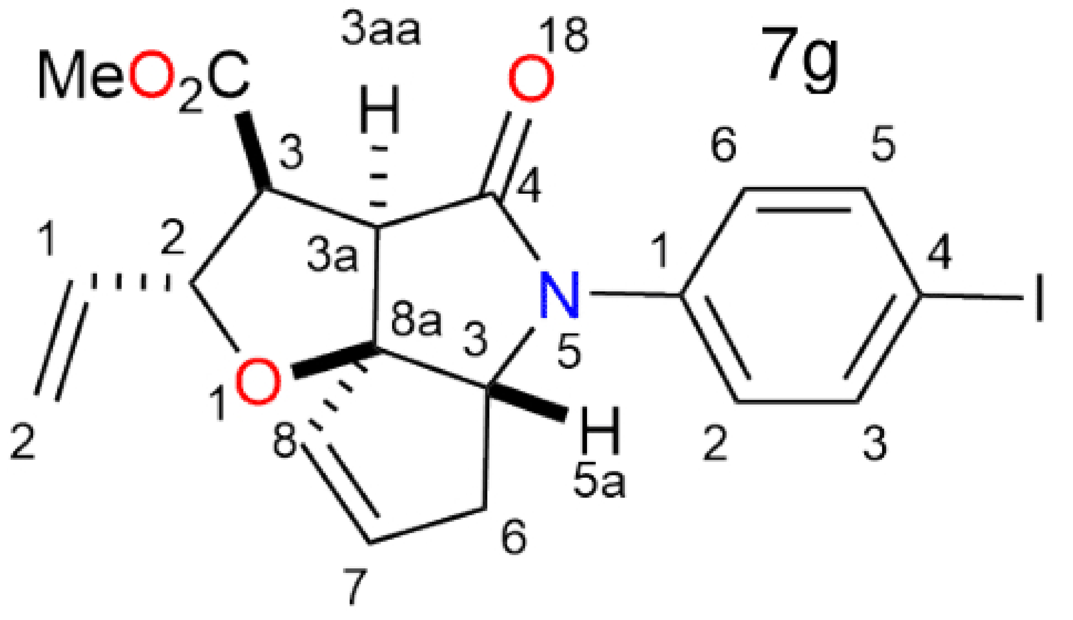

FZ8206-1.esp

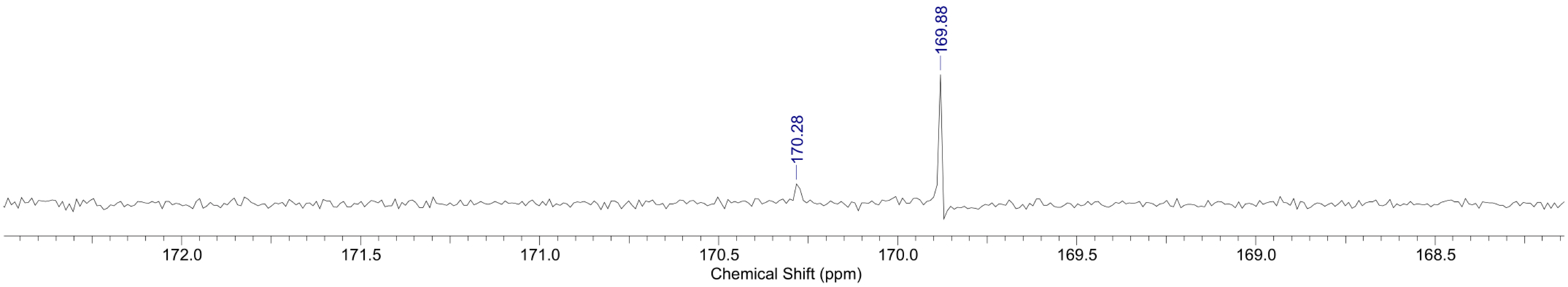

**Formula**	$C_{19}H_{18}INO_4$	**FW**	451.2550

**Acquisition Time (sec)**	0.6921	**Comment**	single pulse decoupled gated NOE			**Date**	19 Sep 1990 09:43:12
**Date Stamp**	29 Nov 2019 15:00:37			**File Name**	C:\Users\User\Desktop\02.12.19\FZ8206-1.jdf		
**Frequency (MHz)**	150.91	**Nucleus**	13C	**Number of Transients**	1000	**Origin**	ECA 600
**Original Points Count**	32768	**Owner**	delta	**Points Count**	32768	**Pulse Sequence**	single_pulse_dec
**Receiver Gain**	54.00	**Solvent**	CHLOROFORM-d			**Spectrum Offset (Hz)**	15079.3525
**Sweep Width (Hz)**	47348.49	**Temperature (degree C)**	23.900				

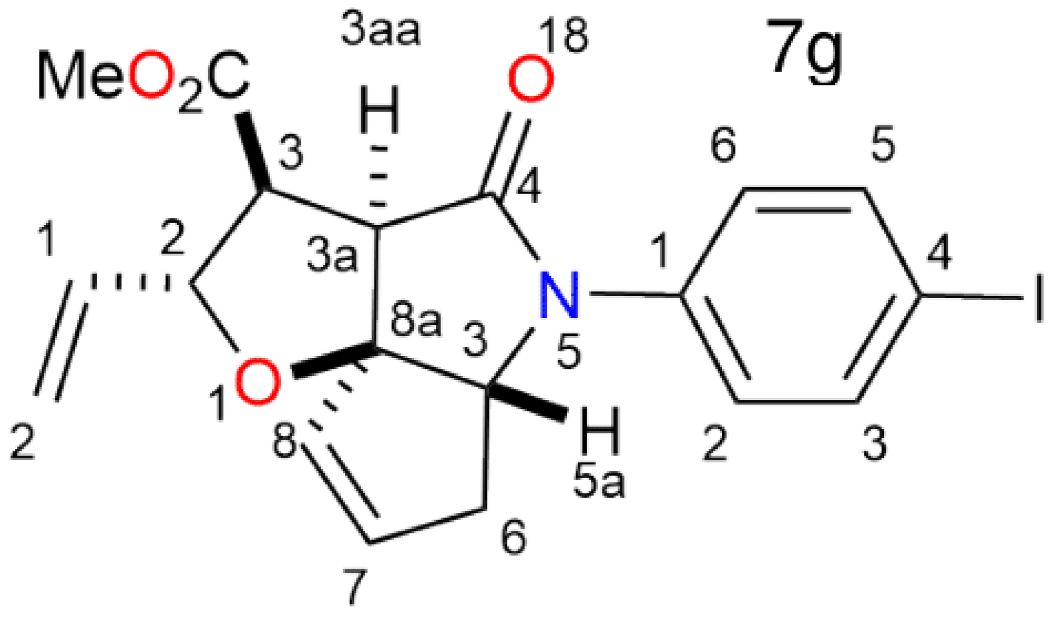

7g

FZ8206-1.esp

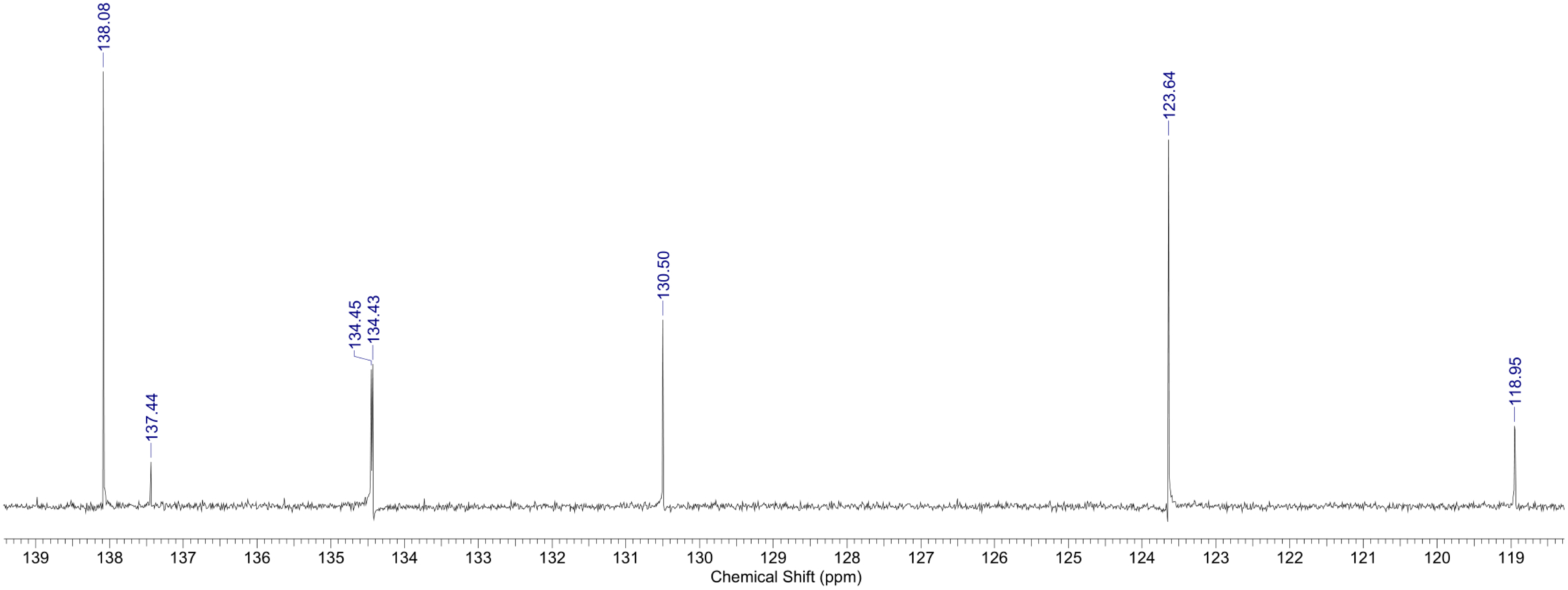

Formula	$C_{19}H_{18}INO_4$	FW	451.2550

Acquisition Time (sec)	0.6921	Comment	single pulse decoupled gated NOE			Date	19 Sep 1990 09:43:12
Date Stamp	29 Nov 2019 15:00:37			File Name	C:\Users\User\Desktop\02.12.19\FZ8206-1.jdf		
Frequency (MHz)	150.91	Nucleus	13C	Number of Transients	1000	Origin	ECA 600
Original Points Count	32768	Owner	delta	Points Count	32768	Pulse Sequence	single_pulse_dec
Receiver Gain	54.00	Solvent	CHLOROFORM-d			Spectrum Offset (Hz)	15079.3525
Sweep Width (Hz)	47348.49	Temperature (degree C)	23.900				

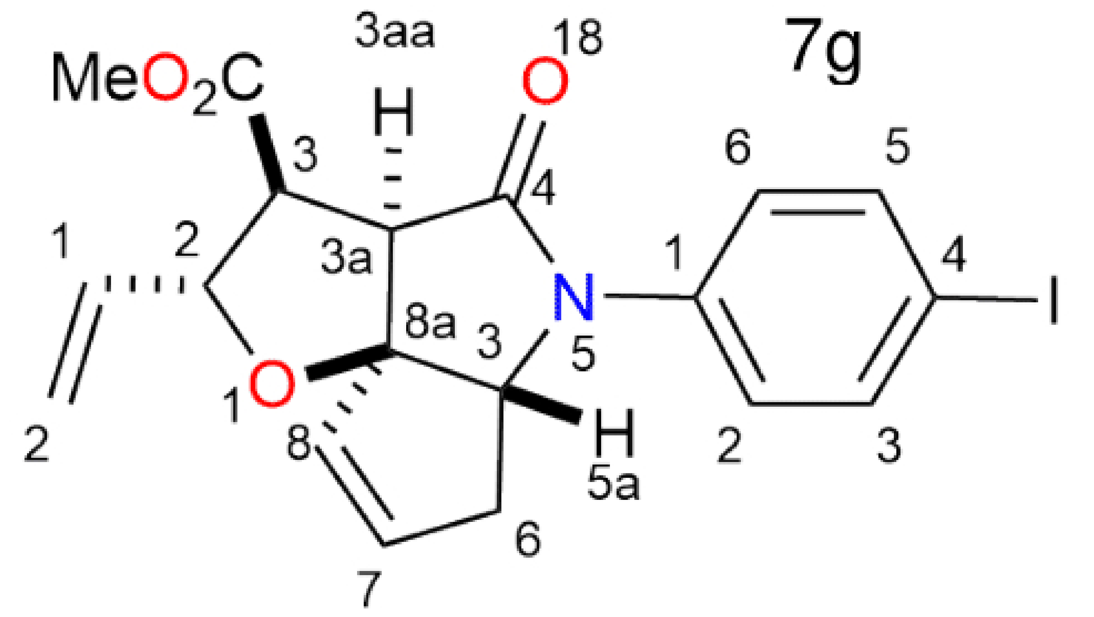

7g

FZ8206-1.esp

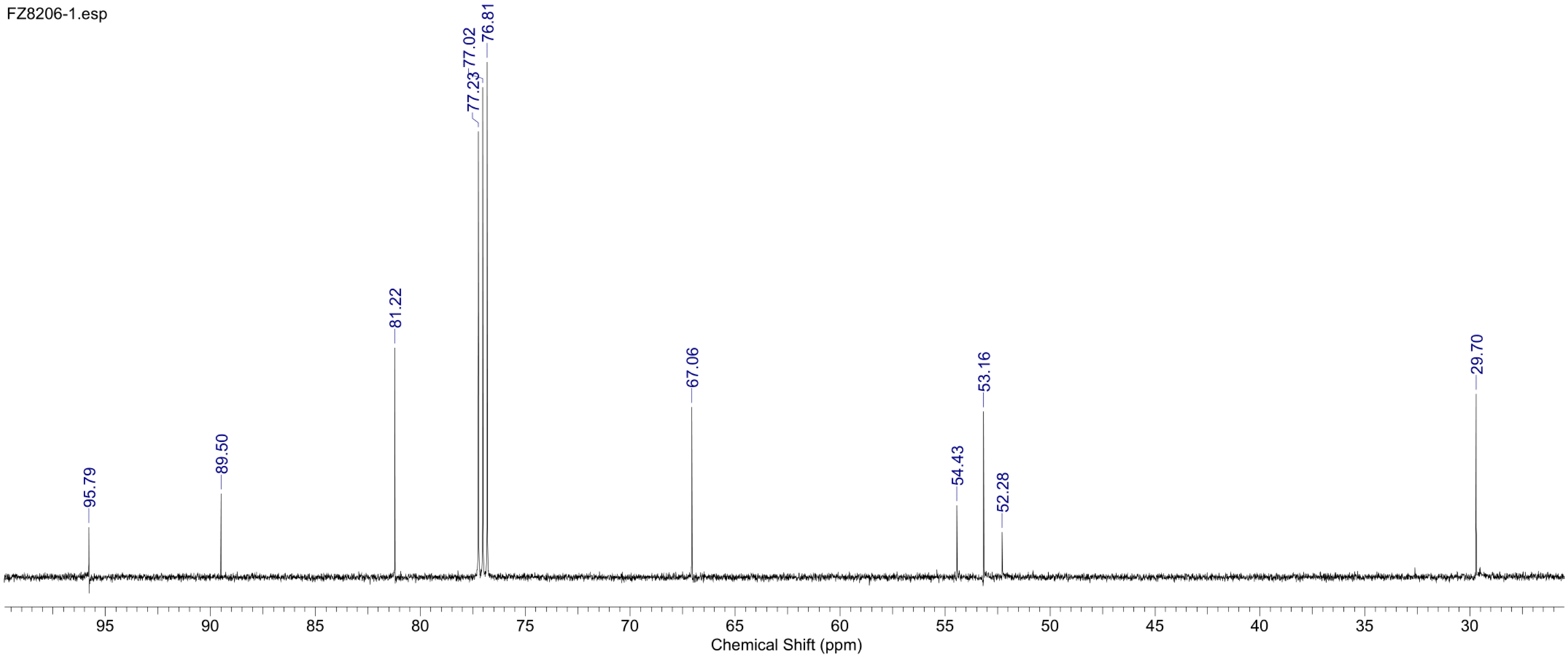

NMR spectrum data for compound 7h

14.07.2020 19:22:26

Formula	$C_{19}H_{17}ClFNO_4$	***FW***	377.7940

Acquisition Time (sec)	1.9818	***Comment***	single_pulse	***Date***	15 Aug 1990 03:19:37				
Date Stamp	25 Oct 2019 09:22:13			***File Name***	C:\USERS\Лаба534\DOWNLOADS\FZ8029-1.JDF			***Frequency (MHz)***	600.17
Nucleus	1H	***Number of Transients***	8	***Origin***	ECA 600	***Original Points Count***	32768	***Owner***	delta
Points Count	32768	***Pulse Sequence***	single_pulse.ex2			***Receiver Gain***	38.00	***Solvent***	CHLOROFORM-d
Spectrum Offset (Hz)	5410.1226	***Sweep Width (Hz)***	16534.39	***Temperature (degree C)***	23.700				

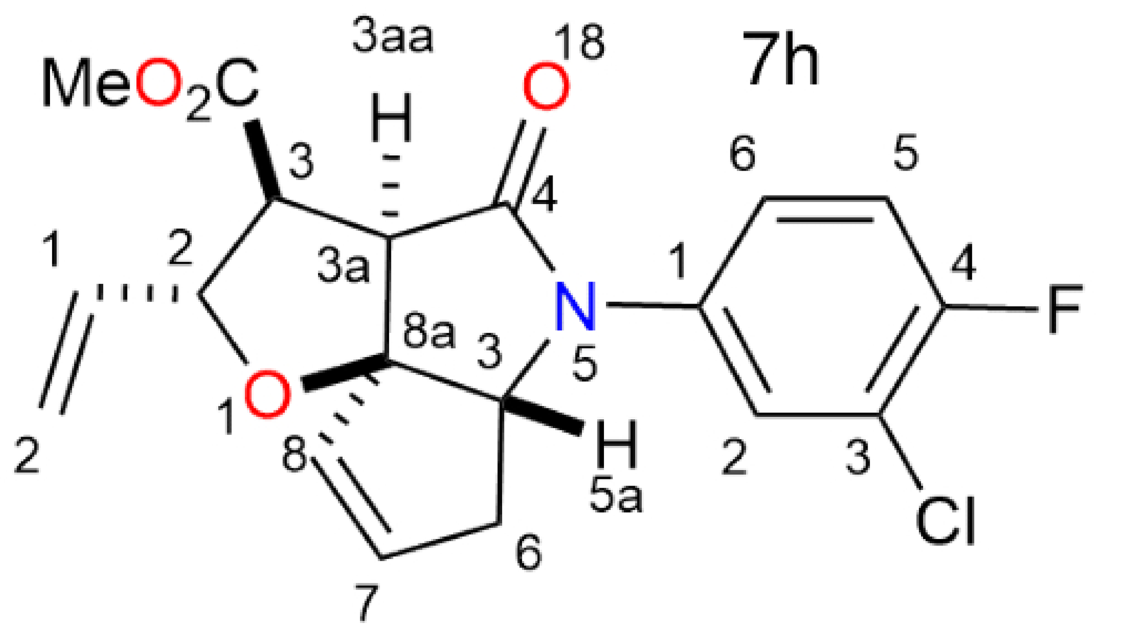

FZ8029-1.JDF

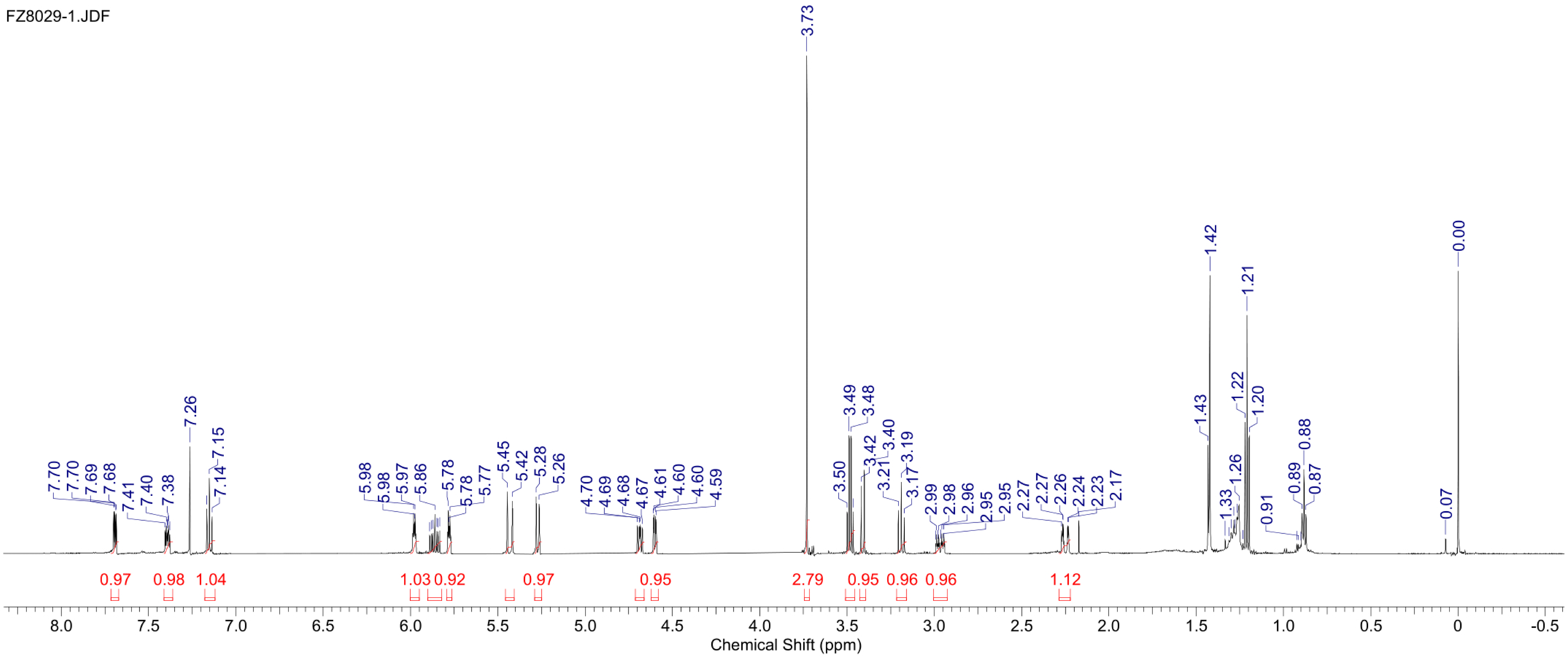

14.07.2020 19:22:43

**Formula** $C_{19}H_{17}ClFNO_4$	**FW** 377.7940

**Acquisition Time (sec)**	1.9818	**Comment**	single_pulse	**Date**	15 Aug 1990 03:19:37		
**Date Stamp**	25 Oct 2019 09:22:13			**File Name**	C:\USERS\Лаба534\DOWNLOADS\FZ8029-1.JDF	**Frequency (MHz)**	600.17
**Nucleus**	1H	**Number of Transients**	8	**Origin**	ECA 600	**Original Points Count** 32768	**Owner** delta
**Points Count**	32768	**Pulse Sequence**	single_pulse.ex2			**Receiver Gain** 38.00	**Solvent** CHLOROFORM-d
**Spectrum Offset (Hz)**	5410.1226	**Sweep Width (Hz)**	16534.39	**Temperature (degree C)**	23.700		

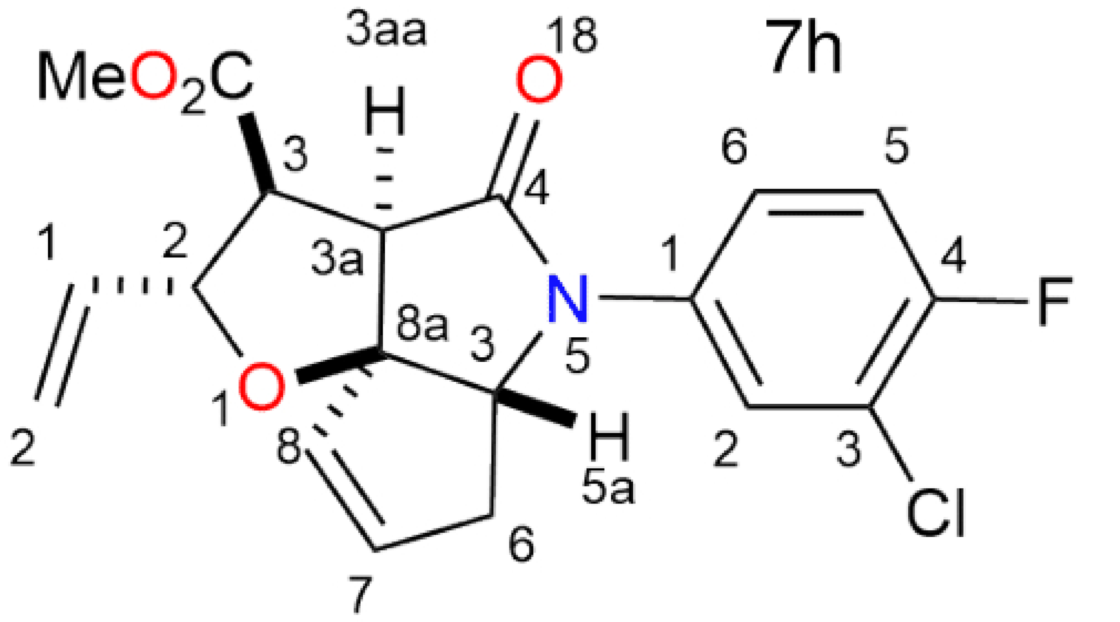

FZ8029-1.JDF

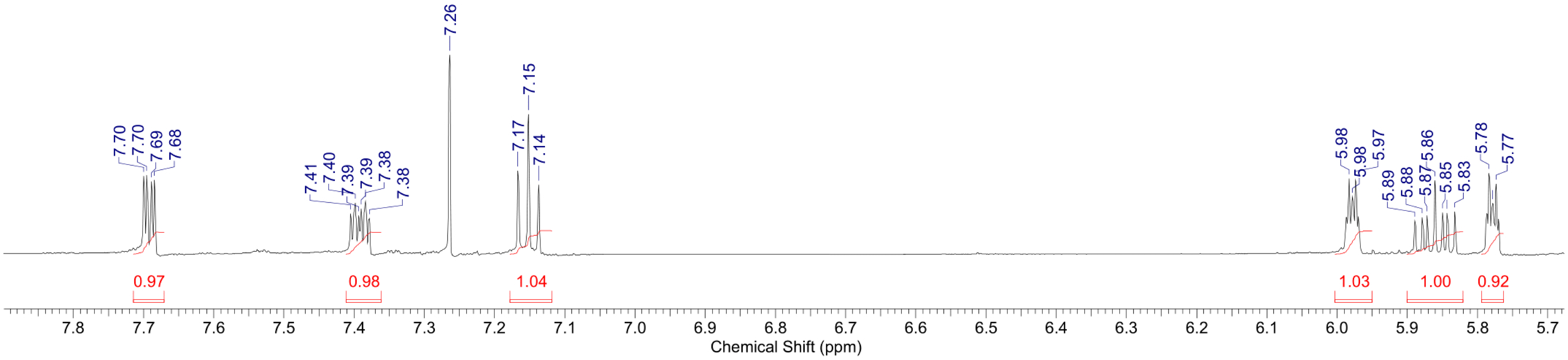

14.07.2020 19:23:06

**Formula** $C_{19}H_{17}ClFNO_4$	**FW** 377.7940

**Acquisition Time (sec)**	1.9818	**Comment**	single_pulse	**Date**	15 Aug 1990 03:19:37
**Date Stamp**	25 Oct 2019 09:22:13			**File Name**	C:\USERS\Лаба534\DOWNLOADS\FZ8029-1.JDF
**Nucleus**	1H	**Number of Transients**	8	**Origin**	ECA 600
**Points Count**	32768	**Pulse Sequence**	single_pulse.ex2		
**Spectrum Offset (Hz)**	5410.1226	**Sweep Width (Hz)**	16534.39	**Temperature (degree C)**	23.700

**Original Points Count**	32768	**Frequency (MHz)**	600.17
		**Owner**	delta
**Receiver Gain**	38.00	**Solvent**	CHLOROFORM-d

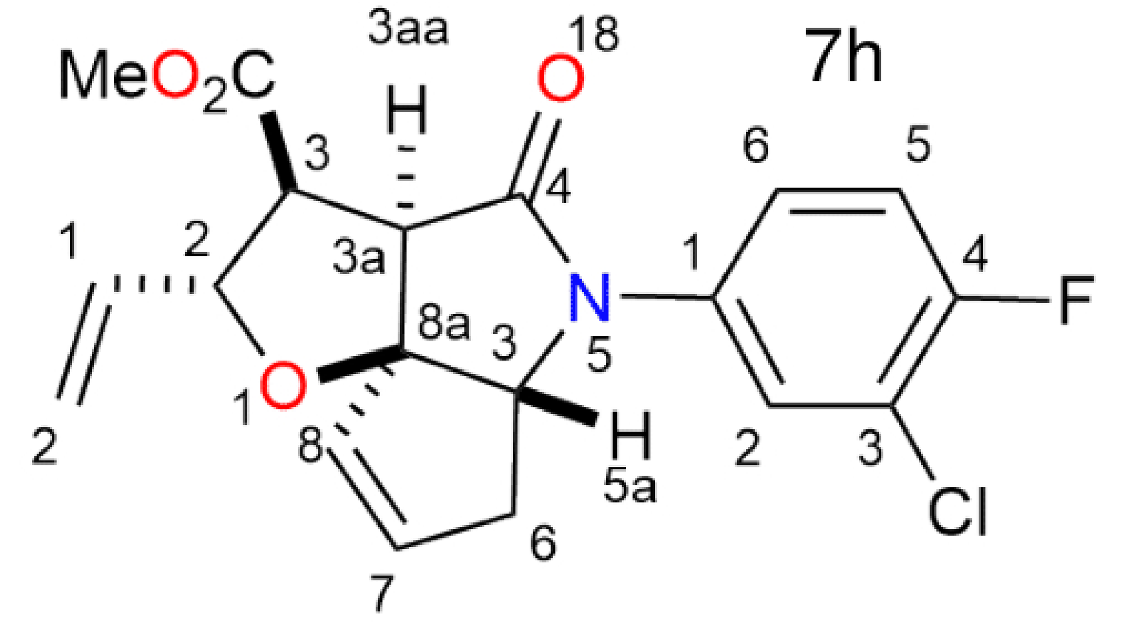

7h

FZ8029-1.JDF

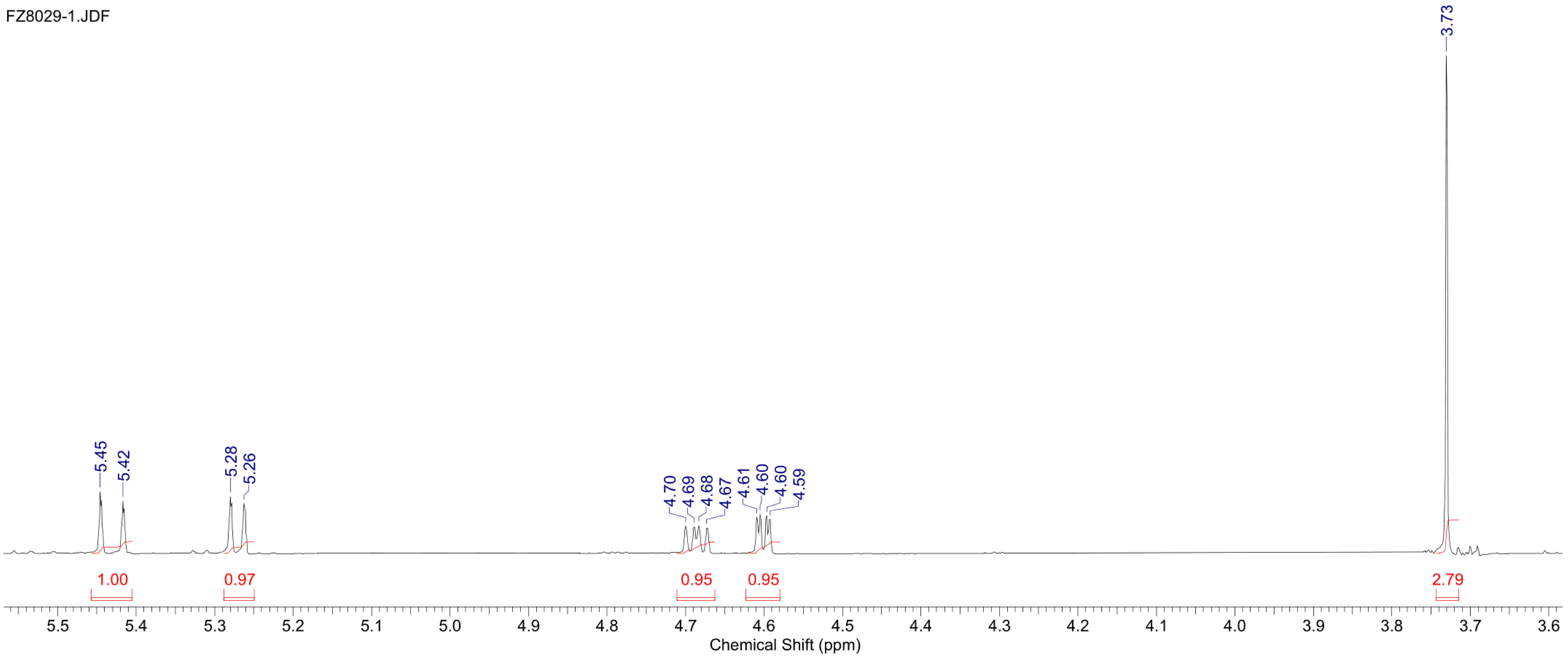

14.07.2020 19:23:20

**Formula**	$C_{19}H_{17}ClFNO_4$	**FW**	377.7940

**Acquisition Time (sec)**	1.9818	**Comment**	single_pulse	**Date**	15 Aug 1990 03:19:37		
**Date Stamp**	25 Oct 2019 09:22:13			**File Name**	C:\USERS\Лаба534\DOWNLOADS\FZ8029-1.JDF	**Frequency (MHz)**	600.17
**Nucleus**	1H	**Number of Transients**	8	**Origin**	ECA 600	**Original Points Count** 32768	**Owner** delta
**Points Count**	32768	**Pulse Sequence**	single_pulse.ex2			**Receiver Gain** 38.00	**Solvent** CHLOROFORM-d
**Spectrum Offset (Hz)**	5410.1226	**Sweep Width (Hz)**	16534.39	**Temperature (degree C)**	23.700		

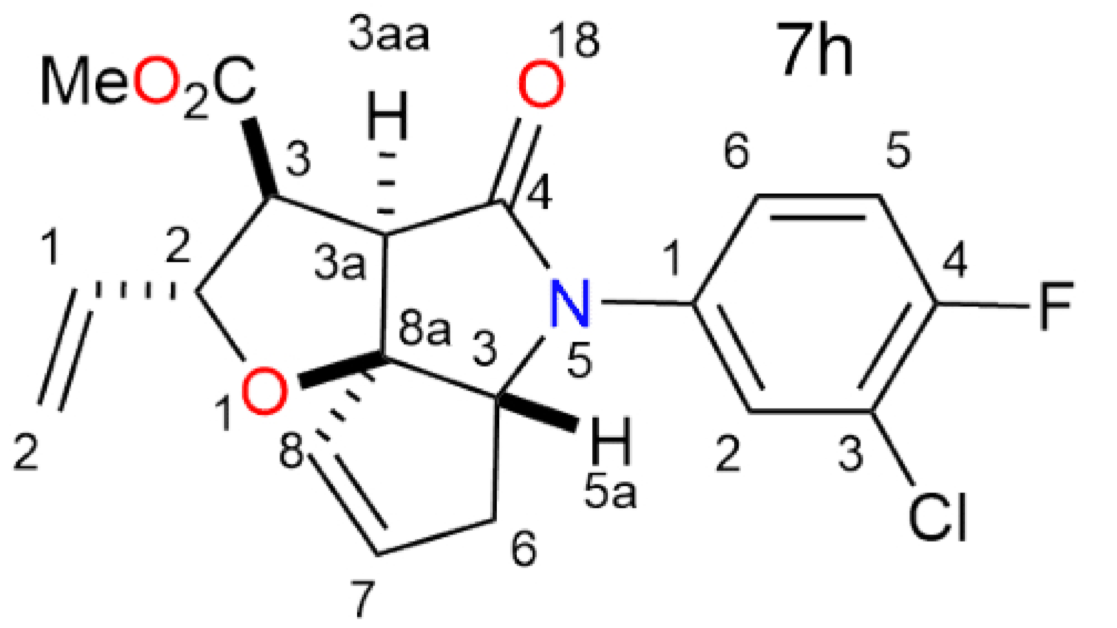

FZ8029-1.JDF

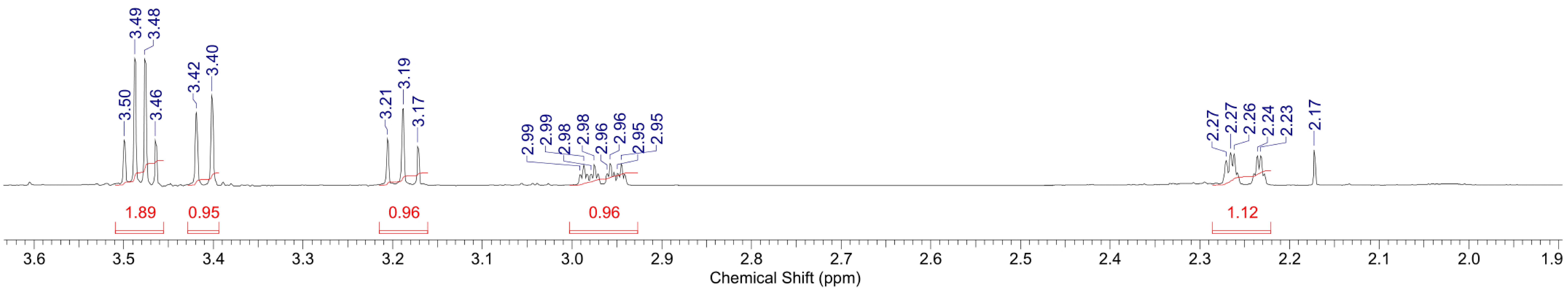

14.07.2020 19:24:47

**Formula** $C_{19}H_{17}ClFNO_4$	**FW** 377.7940

**Acquisition Time (sec)**	0.6921	**Comment**	single pulse decoupled gated NOE			**Date**	09 Feb 2020 15:08:29
**Date Stamp**	21 Feb 2020 16:54:12			**File Name**	C:\USERS\Лаба534\DOWNLOADS\FZ8500-1.JDF		
**Frequency (MHz)**	150.91	**Nucleus**	13C	**Number of Transients**	4000	**Origin**	ECA 600
**Original Points Count**	32768	**Owner**	CKP	**Points Count**	32768	**Pulse Sequence**	single_pulse_dec
**Receiver Gain**	56.00	**Solvent**	CHLOROFORM-d			**Spectrum Offset (Hz)**	15080.7979
**Sweep Width (Hz)**	47348.49	**Temperature (degree C)**	24.000				

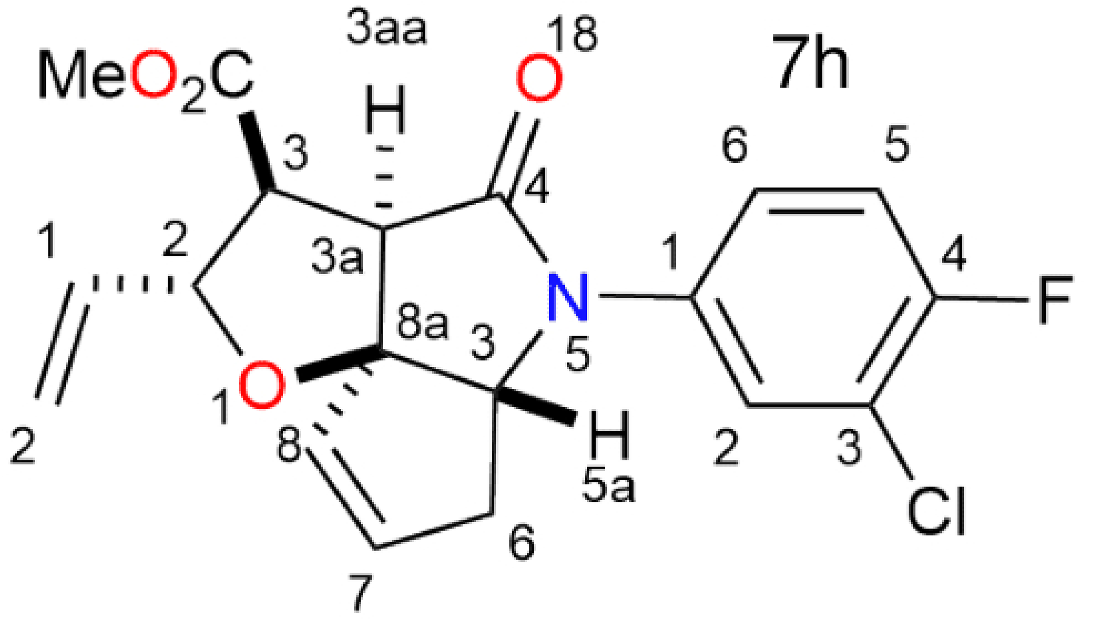

7h

FZ8500-1.JDF

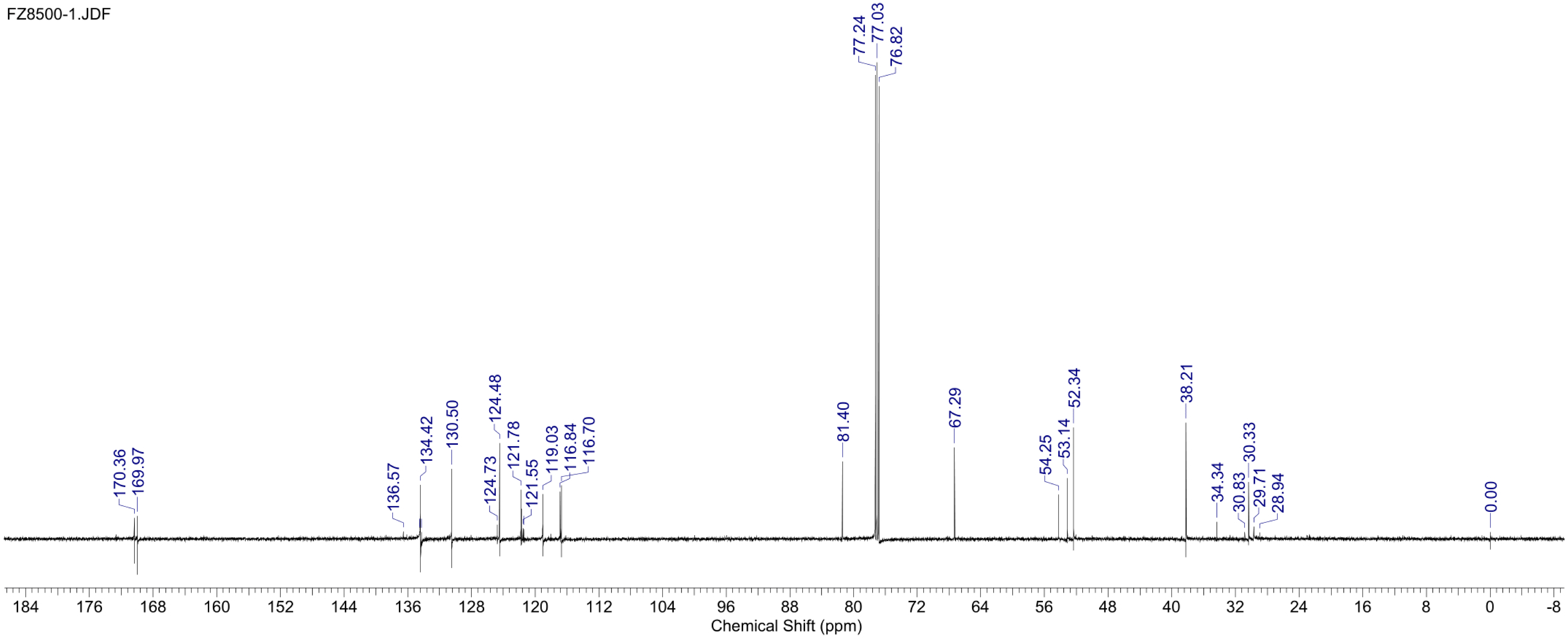

14.07.2020 19:24:57

Formula	$C_{19}H_{17}ClFNO_4$	***FW***	377.7940

Acquisition Time (sec)	0.6921	***Comment***	single pulse decoupled gated NOE			***Date***	09 Feb 2020 15:08:29
Date Stamp	21 Feb 2020 16:54:12			***File Name***	C:\USERS\Лаба534\DOWNLOADS\FZ8500-1.JDF		
Frequency (MHz)	150.91	***Nucleus***	13C	***Number of Transients***	4000	***Origin***	ECA 600
Original Points Count	32768	***Owner***	CKP	***Points Count***	32768	***Pulse Sequence***	single_pulse_dec
Receiver Gain	56.00	***Solvent***	CHLOROFORM-d			***Spectrum Offset (Hz)***	15080.7979
Sweep Width (Hz)	47348.49	***Temperature (degree C)***	24.000				

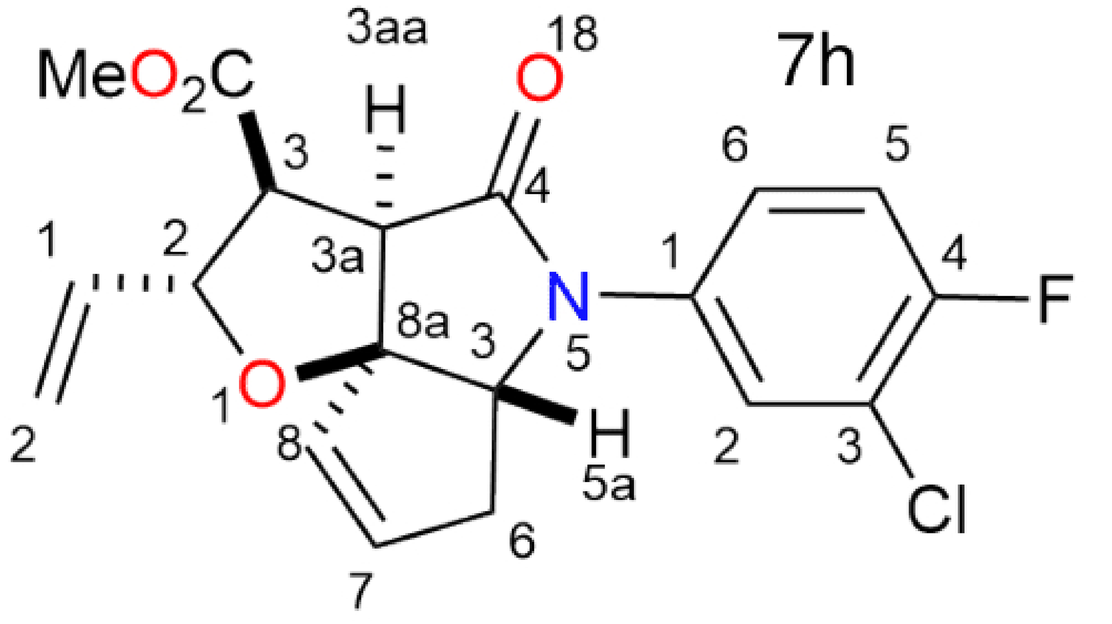

FZ8500-1.JDF

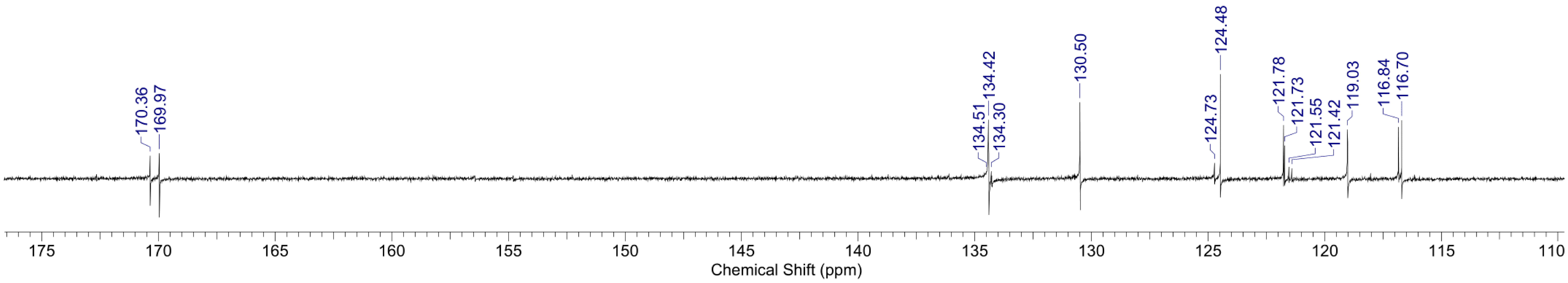

14.07.2020 19:25:05

Formula $C_{19}H_{17}ClFNO_4$	***FW*** 377.7940		

Acquisition Time (sec)	0.6921	***Comment***	single pulse decoupled gated NOE			***Date***	09 Feb 2020 15:08:29
Date Stamp	21 Feb 2020 16:54:12			***File Name***	C:\USERS\Лаба534\DOWNLOADS\FZ8500-1.JDF		
Frequency (MHz)	150.91	***Nucleus***	13C	***Number of Transients***	4000	***Origin***	ECA 600
Original Points Count	32768	***Owner***	CKP	***Points Count***	32768	***Pulse Sequence***	single_pulse_dec
Receiver Gain	56.00	***Solvent***	CHLOROFORM-d			***Spectrum Offset (Hz)***	15080.7979
Sweep Width (Hz)	47348.49	***Temperature (degree C)***	24.000				

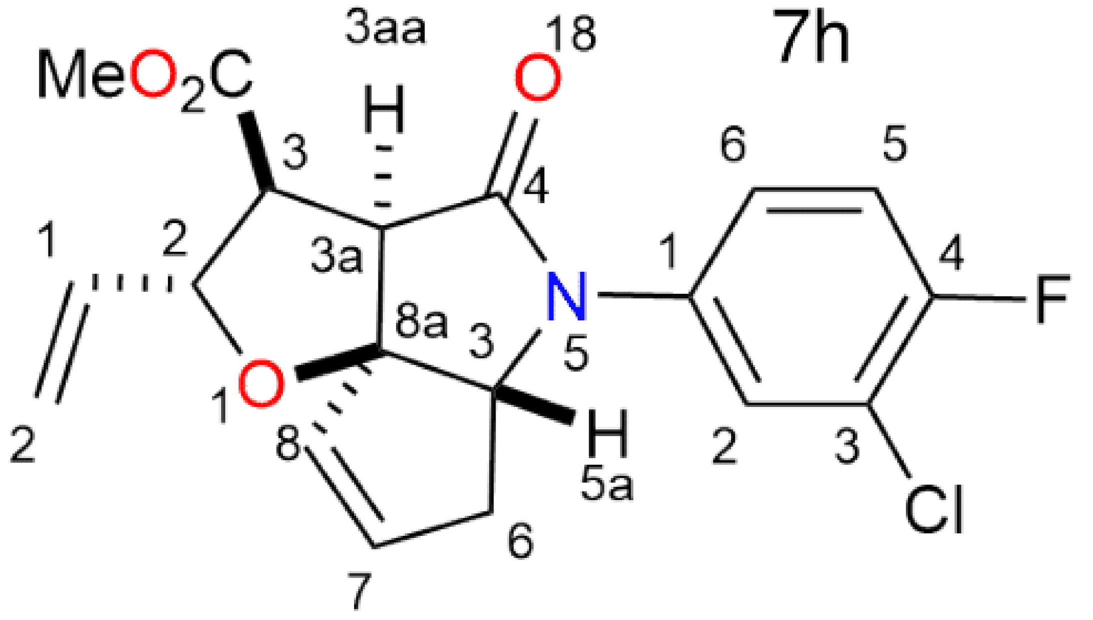

7h

FZ8500-1.JDF

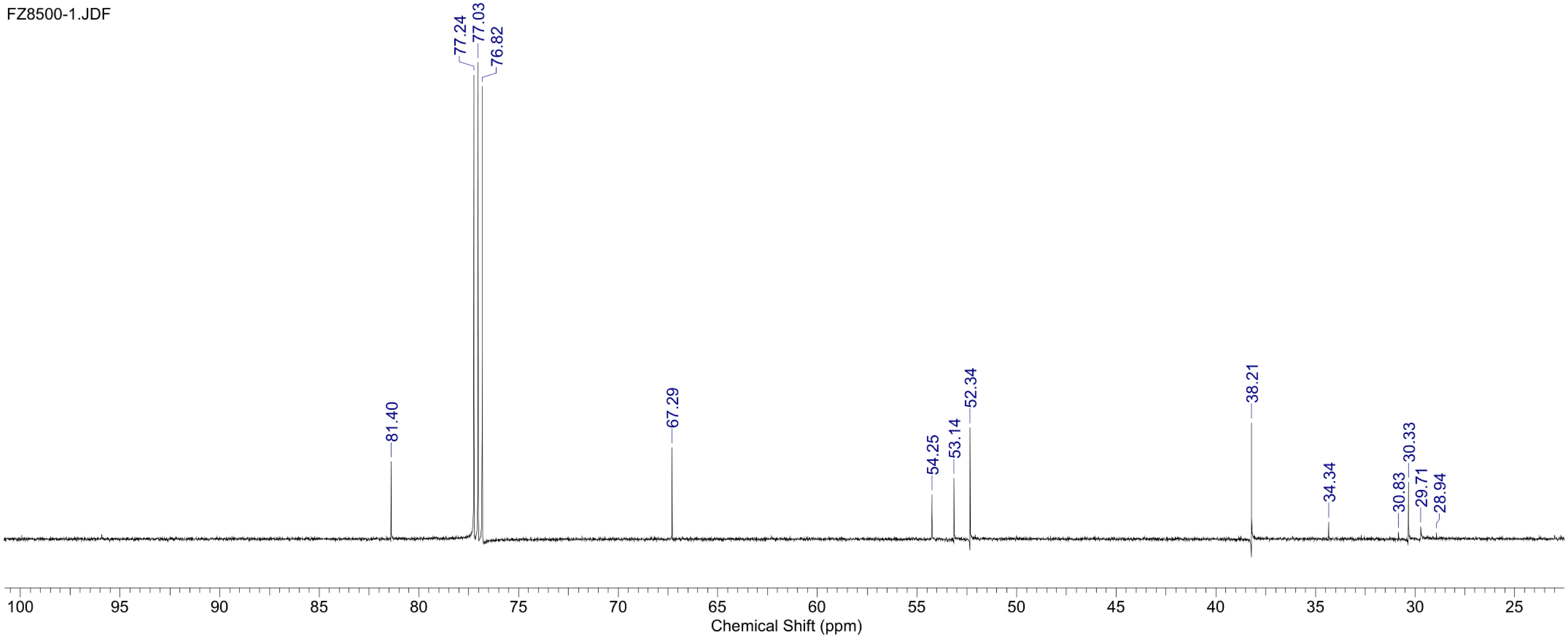

NMR spectrum data for compound 7i

Formula	$C_{20}H_{21}NO_4$	FW	339.3850

**Acquisition Time (sec)**	1.9818	**Comment**	single_pulse	**Date**	23 Jun 2020 11:24:58		
**Date Stamp**	23 Jun 2020 11:26:18			**File Name**	C:\USERS\лаба 534\DESKTOP\Саша\ЯМР\NMR FIDS\FZ8694-1.JDF		
**Frequency (MHz)**	600.17	**Nucleus**	1H	**Number of Transients**	8	**Origin**	ECA 600
**Original Points Count**	32768	**Owner**	CKP	**Points Count**	32768	**Pulse Sequence**	single_pulse.ex2
**Receiver Gain**	40.00	**Solvent**	CHLOROFORM-d			**Spectrum Offset (Hz)**	5409.1133
**Sweep Width (Hz)**	16534.39	**Temperature (degree C)**	21.800				

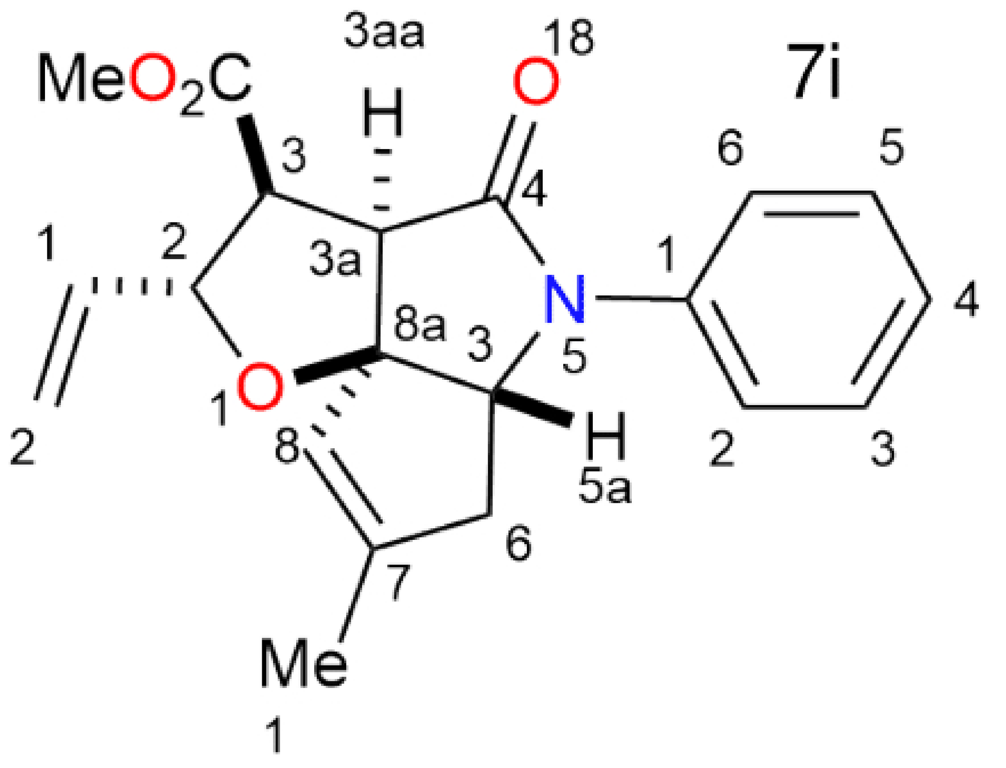

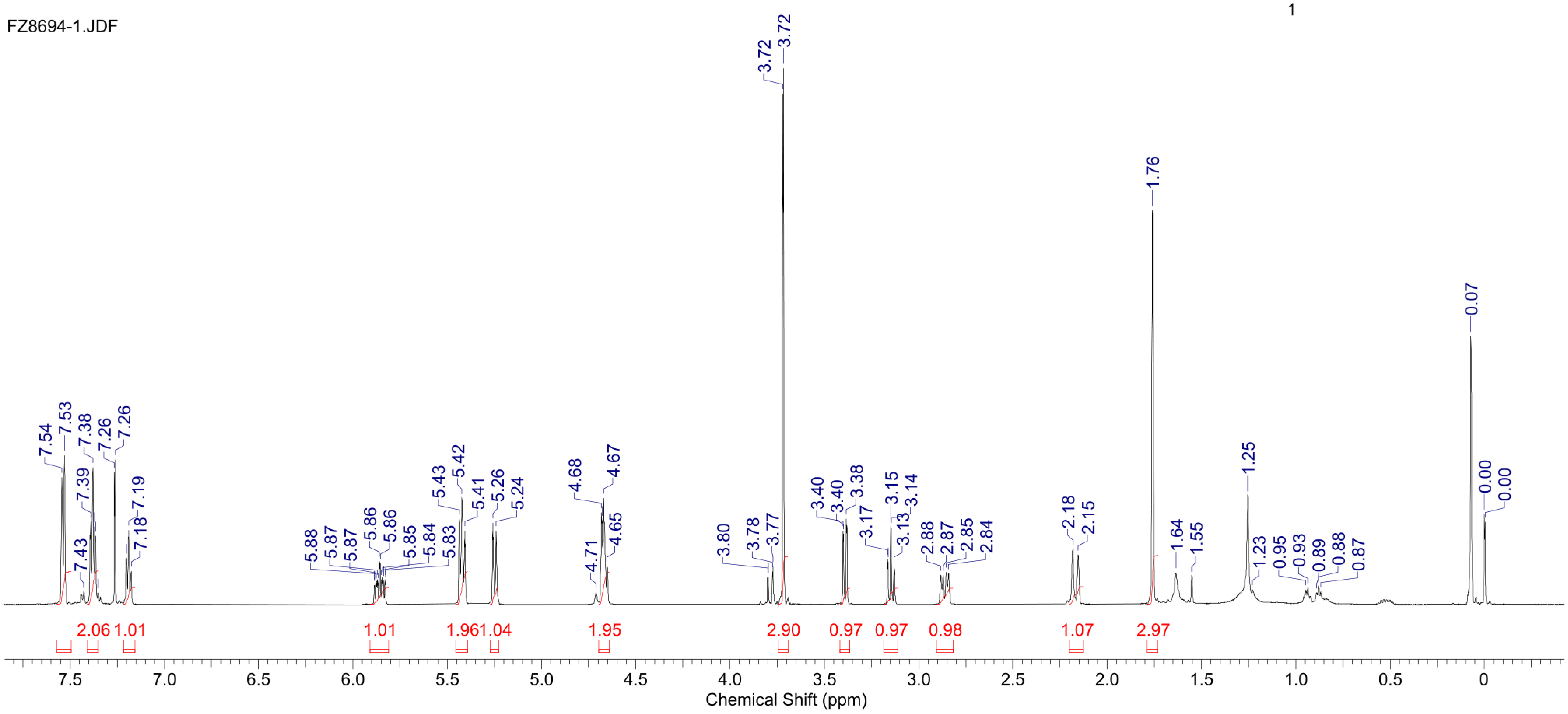

Formula $C_{20}H_{21}NO_4$	FW 339.3850

Acquisition Time (sec)	1.9818	Comment	single_pulse	Date	23 Jun 2020 11:24:58		
Date Stamp	23 Jun 2020 11:26:18			File Name	C:\USERS\лаба 534\DESKTOP\Саша\ЯМР\NMR FIDS\FZ8694-1.JDF		
Frequency (MHz)	600.17	Nucleus	1H	Number of Transients	8	Origin	ECA 600
Original Points Count	32768	Owner	CKP	Points Count	32768	Pulse Sequence	single_pulse.ex2
Receiver Gain	40.00	Solvent	CHLOROFORM-d			Spectrum Offset (Hz)	5409.1133
Sweep Width (Hz)	16534.39	Temperature (degree C)	21.800				

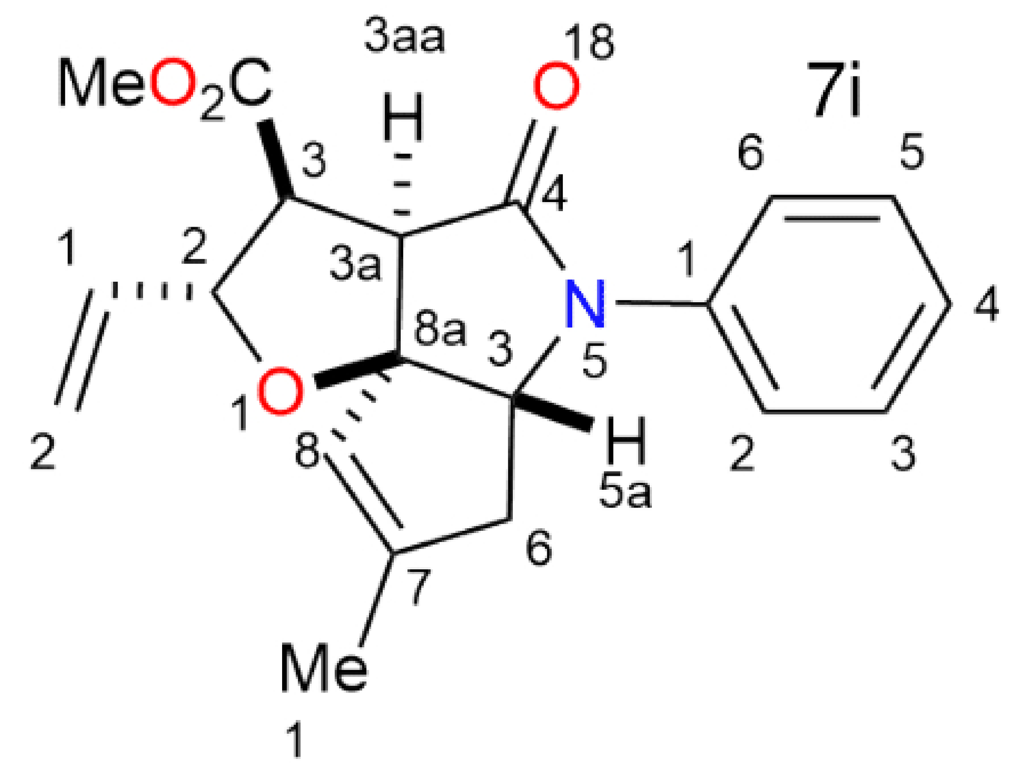

FZ8694-1.JDF

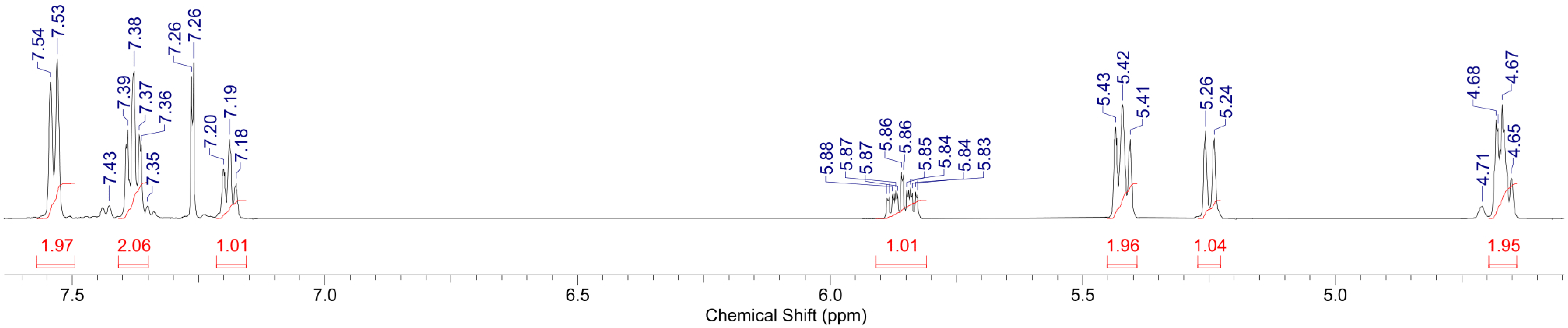

**Formula** $C_{20}H_{21}NO_4$	**FW** 339.3850

Acquisition Time (sec)	1.9818	***Comment***	single_pulse	***Date***	23 Jun 2020 11:24:58		
Date Stamp	23 Jun 2020 11:26:18			***File Name***	C:\USERS\лаба 534\DESKTOP\Саша\ЯМР\NMR FIDS\FZ8694-1.JDF		
Frequency (MHz)	600.17	***Nucleus***	1H	***Number of Transients***	8	***Origin***	ECA 600
Original Points Count	32768	***Owner***	CKP	***Points Count***	32768	***Pulse Sequence***	single_pulse.ex2
Receiver Gain	40.00	***Solvent***	CHLOROFORM-d			***Spectrum Offset (Hz)***	5409.1133
Sweep Width (Hz)	16534.39	***Temperature (degree C)***	21.800				

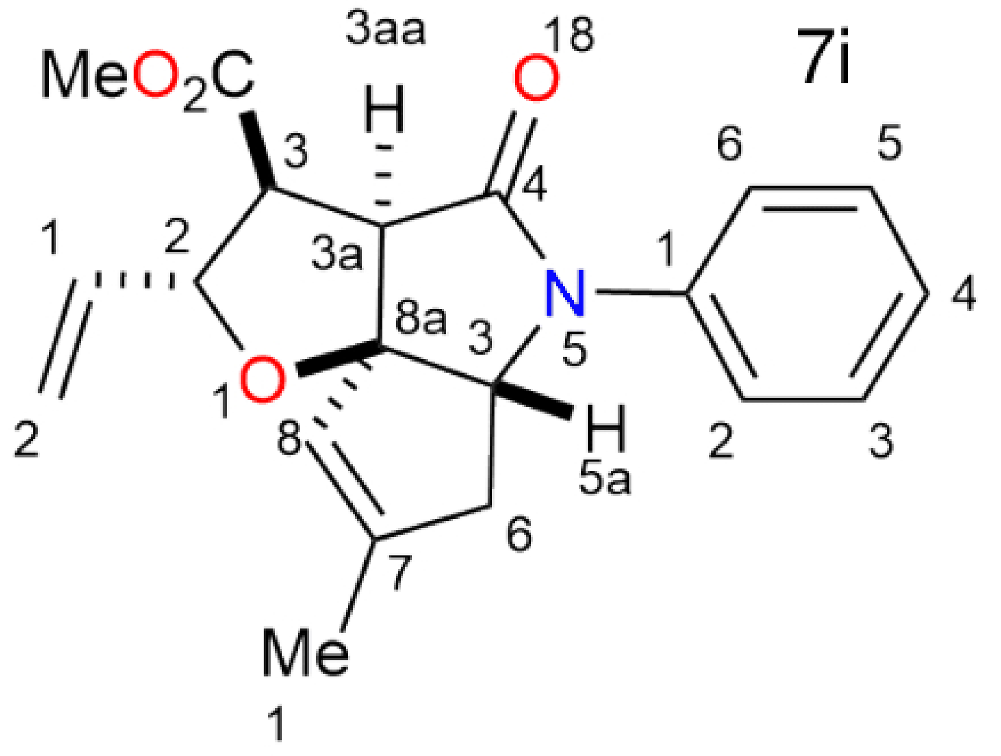

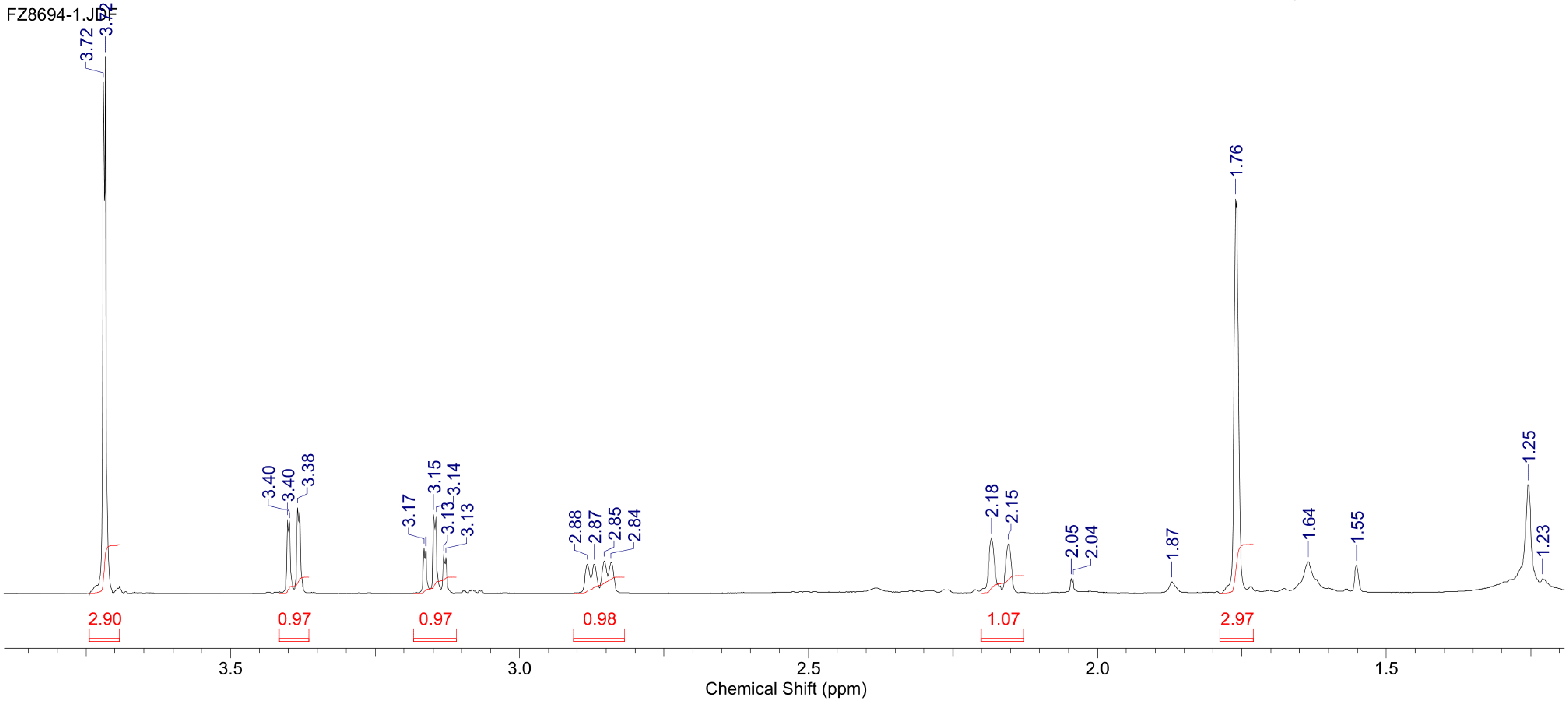

**Formula** $C_{20}H_{21}NO_4$	**FW** 339.3850

**Acquisition Time (sec)**	0.6921	**Comment**	single pulse decoupled gated NOE			**Date**	08 Jul 2020 02:49:00
**Date Stamp**	08 Jul 2020 02:50:33			**File Name**	C:\USERS\лаба 534\DESKTOP\Саша\ЯМР\NMR FIDS\FZ8747-1.JDF		
**Frequency (MHz)**	150.91	**Nucleus**	13C	**Number of Transients**	2000	**Origin**	ECA 600
**Original Points Count**	32768	**Owner**	CKP	**Points Count**	32768	**Pulse Sequence**	single_pulse_dec
**Receiver Gain**	58.00	**Solvent**	CHLOROFORM-d			**Spectrum Offset (Hz)**	15079.3525
**Sweep Width (Hz)**	47348.49	**Temperature (degree C)**	24.900				

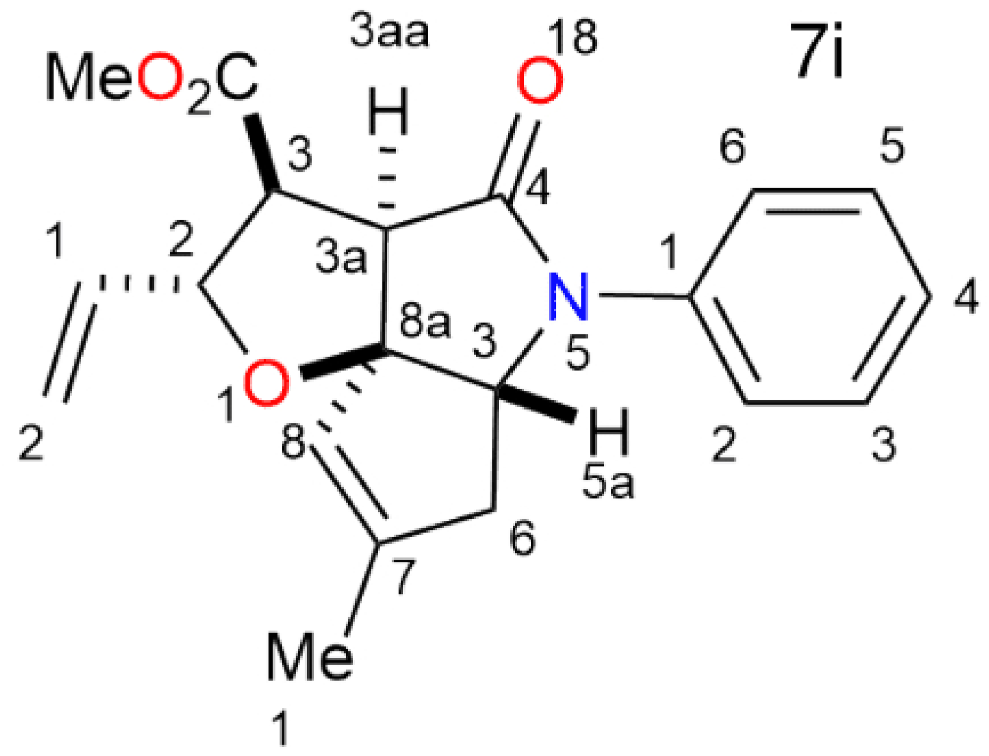

FZ8747-1.JDF

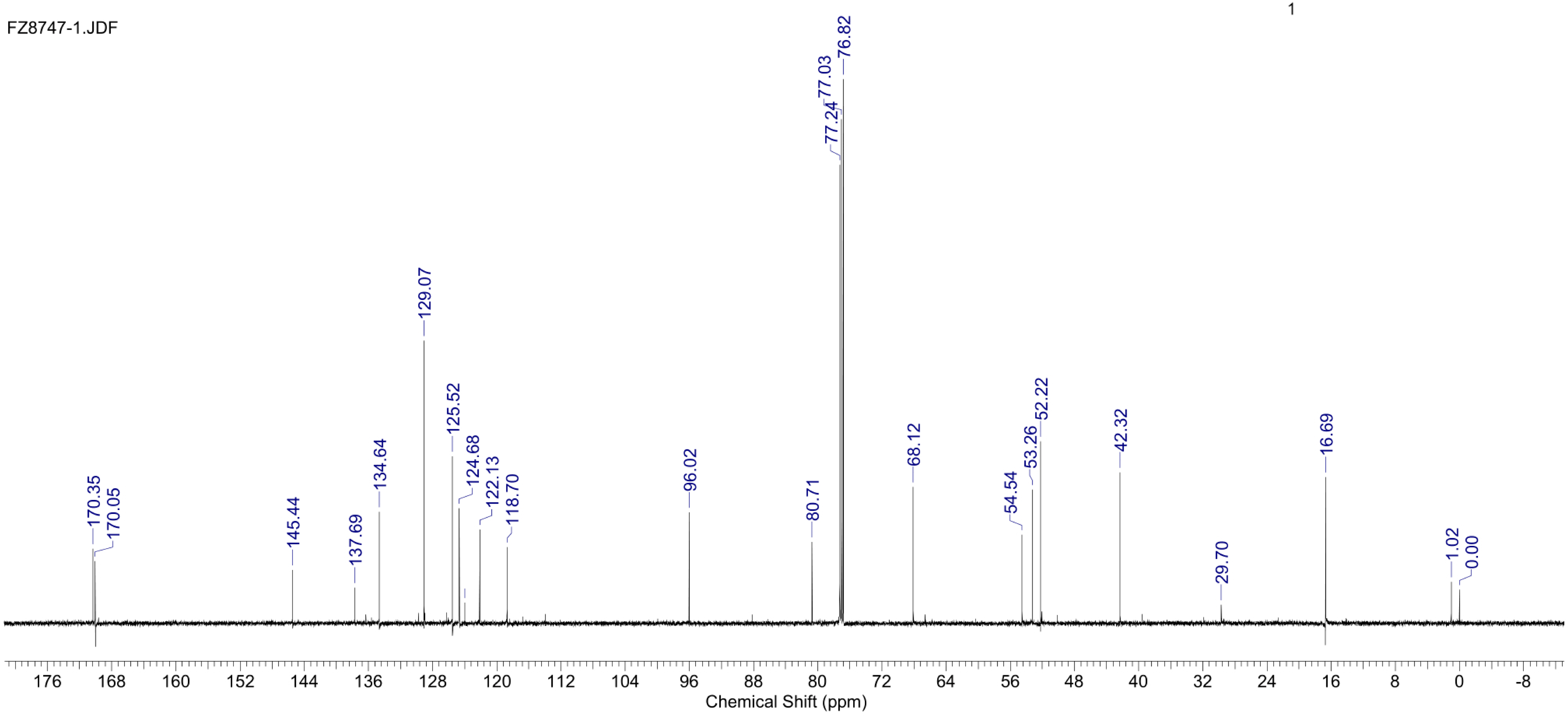

Formula $C_{20}H_{21}NO_4$	*FW* 339.3850

Acquisition Time (sec)	0.6921	***Comment***	single pulse decoupled gated NOE			***Date***	08 Jul 2020 02:49:00
Date Stamp	08 Jul 2020 02:50:33			***File Name***	C:\USERS\лаба 534\DESKTOP\Саша\ЯМР\NMR FIDS\FZ8747-1.JDF		
Frequency (MHz)	150.91	***Nucleus***	13C	***Number of Transients***	2000	***Origin***	ECA 600
Original Points Count	32768	***Owner***	CKP	***Points Count***	32768	***Pulse Sequence***	single_pulse_dec
Receiver Gain	58.00	***Solvent***	CHLOROFORM-d			***Spectrum Offset (Hz)***	15079.3525
Sweep Width (Hz)	47348.49	***Temperature (degree C)***	24.900				

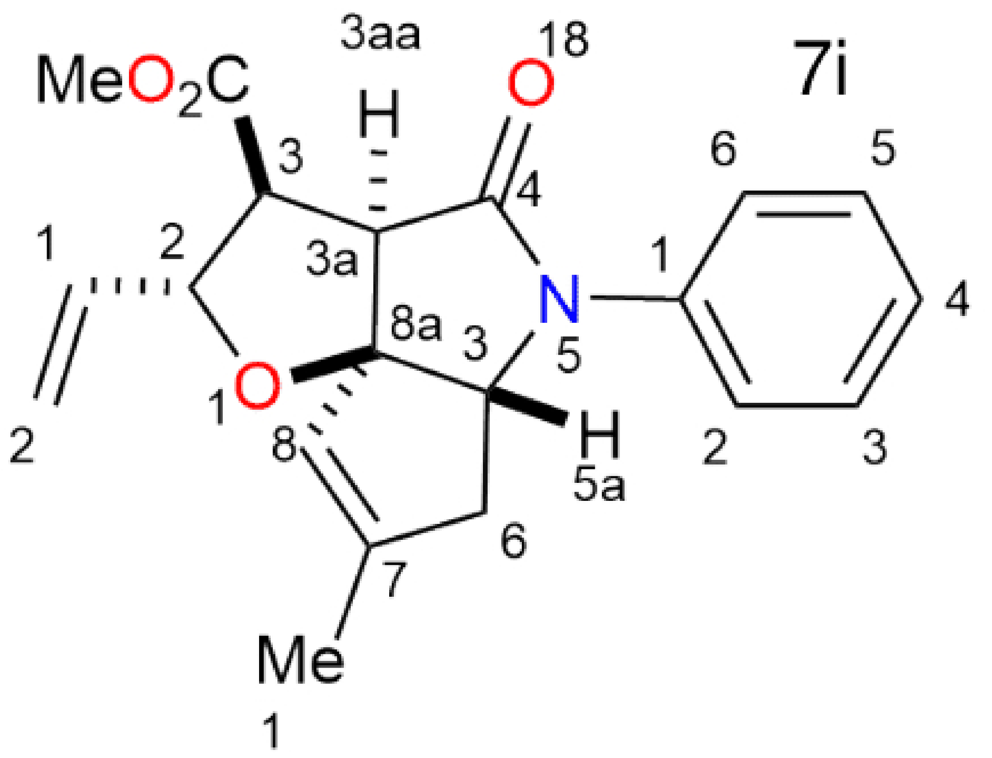

FZ8747-1.JDF

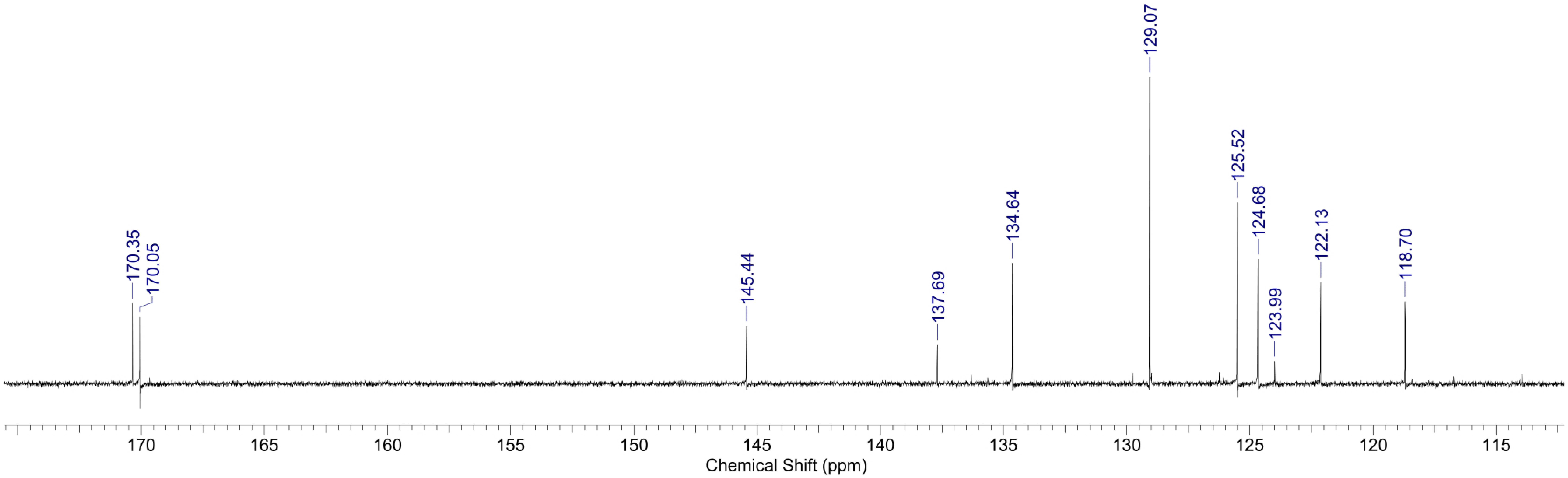

Formula $C_{20}H_{21}NO_4$	*FW* 339.3850

Acquisition Time (sec)	0.6921	***Comment***	single pulse decoupled gated NOE			***Date***	08 Jul 2020 02:49:00
Date Stamp	08 Jul 2020 02:50:33			***File Name***	C:\USERS\лаба 534\DESKTOP\Саша\ЯМР\NMR FIDS\FZ8747-1.JDF		
Frequency (MHz)	150.91	***Nucleus***	13C	***Number of Transients***	2000	***Origin***	ECA 600
Original Points Count	32768	***Owner***	CKP	***Points Count***	32768	***Pulse Sequence***	single_pulse_dec
Receiver Gain	58.00	***Solvent***	CHLOROFORM-d			***Spectrum Offset (Hz)***	15079.3525
Sweep Width (Hz)	47348.49	***Temperature (degree C)***	24.900				

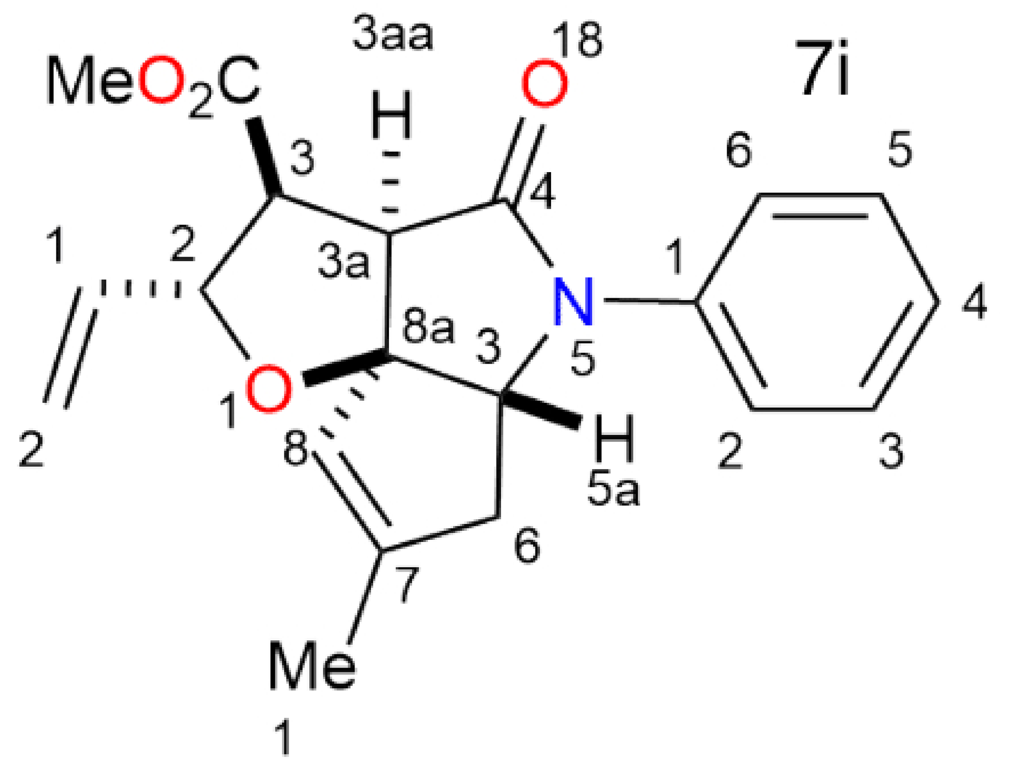

FZ8747-1.JDF

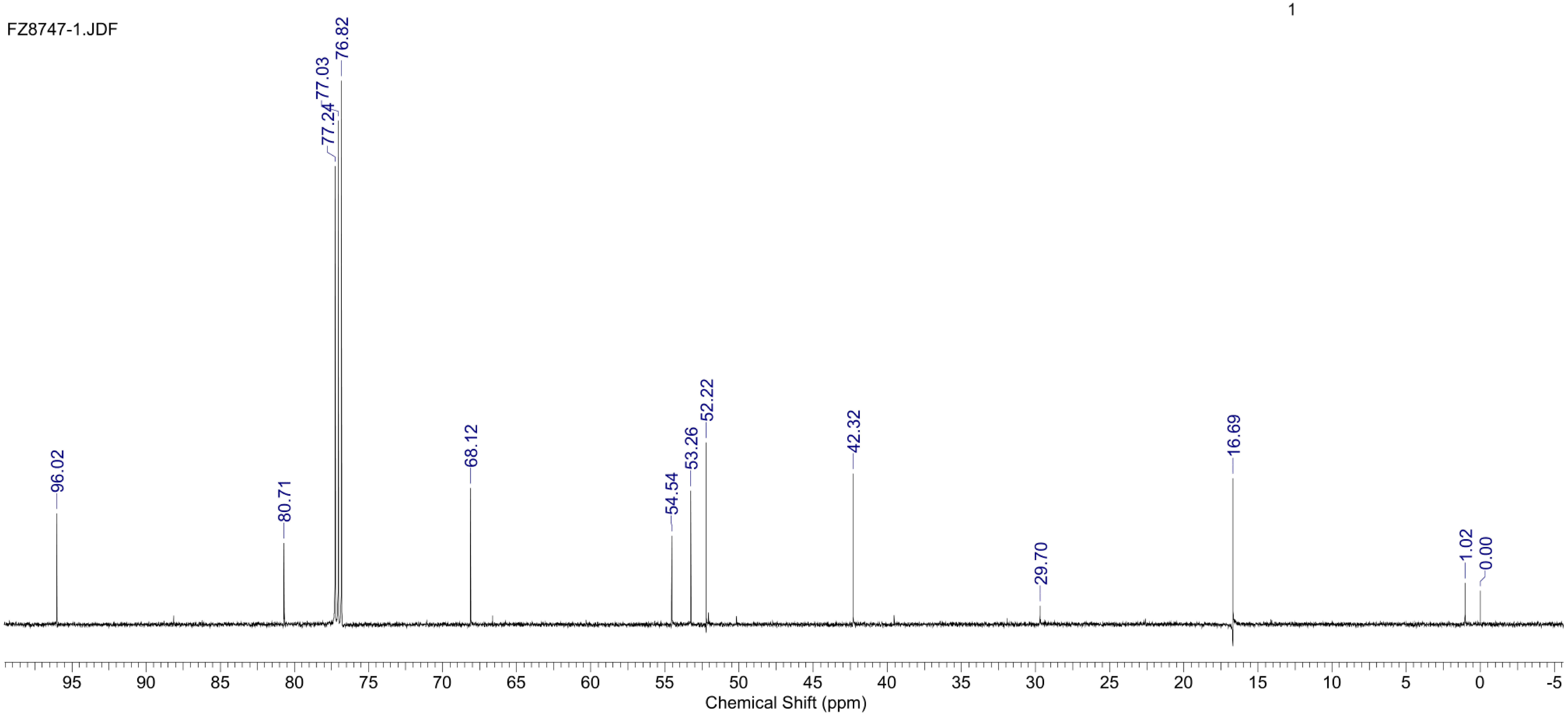

NMR spectrum data for compound 7j

**Formula**	**FW**
$C_{21}H_{23}NO_4$	353.4116

Acquisition Time (sec)	1.9818	***Comment***	single_pulse	***Date***	14 Jul 2020 13:36:14		
Date Stamp	14 Jul 2020 13:37:58			***File Name***	C:\USERS\паба 534\DOWNLOADS\FZ8799-1.JDF		
Frequency (MHz)	600.17	***Nucleus***	1H	***Number of Transients***	8	***Origin***	ECA 600
Original Points Count	32768	***Owner***	CKP	***Points Count***	32768	***Pulse Sequence***	single_pulse.ex2
Receiver Gain	36.00	***Solvent***	CHLOROFORM-d			***Spectrum Offset (Hz)***	5410.6274
Sweep Width (Hz)	16534.39	***Temperature (degree C)***	38.000				

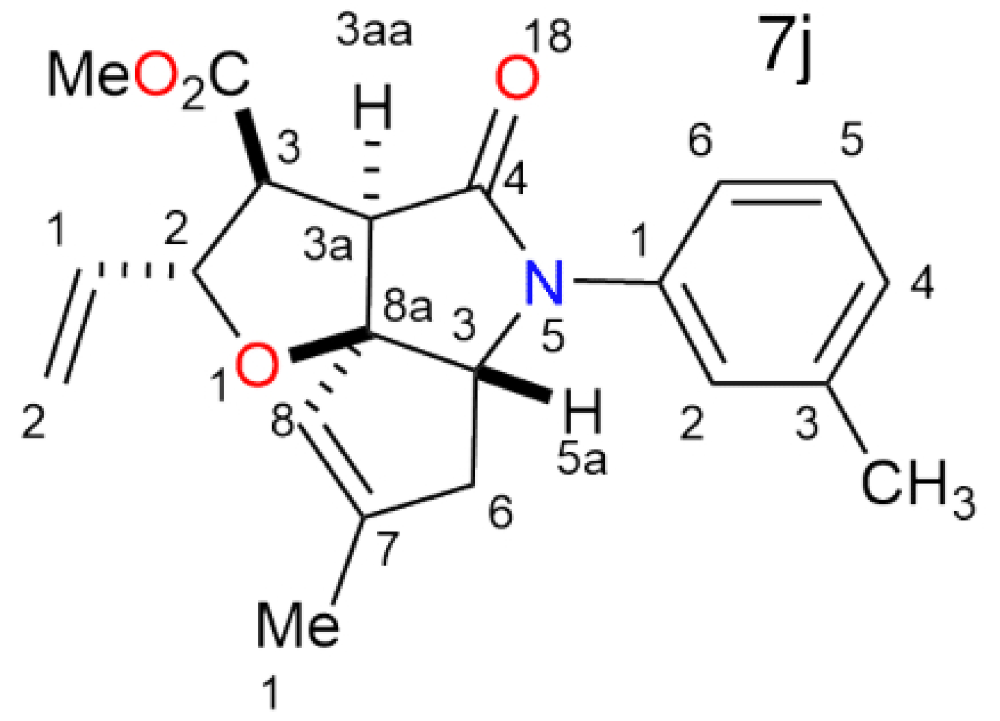

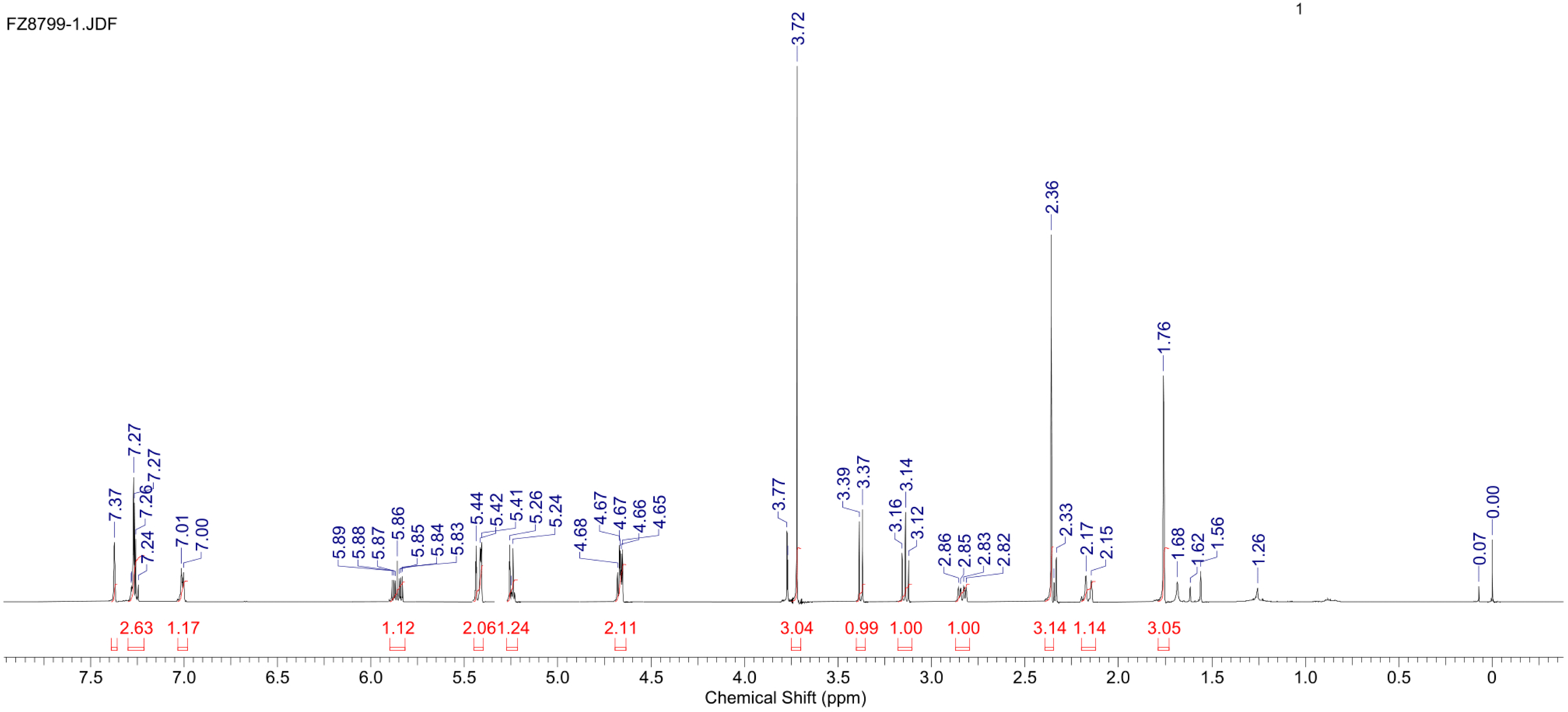

Formula $C_{21}H_{23}NO_4$	FW 353.4116

**Acquisition Time (sec)**	1.9818	**Comment**	single_pulse	**Date**	14 Jul 2020 13:36:14		
**Date Stamp**	14 Jul 2020 13:37:58			**File Name**	C:\USERS\па6а 534\DOWNLOADS\FZ8799-1.JDF		
**Frequency (MHz)**	600.17	**Nucleus**	1H	**Number of Transients**	8	**Origin**	ECA 600
**Original Points Count**	32768	**Owner**	CKP	**Points Count**	32768	**Pulse Sequence**	single_pulse.ex2
**Receiver Gain**	36.00	**Solvent**	CHLOROFORM-d			**Spectrum Offset (Hz)**	5410.6274
**Sweep Width (Hz)**	16534.39	**Temperature (degree C)**	38.000				

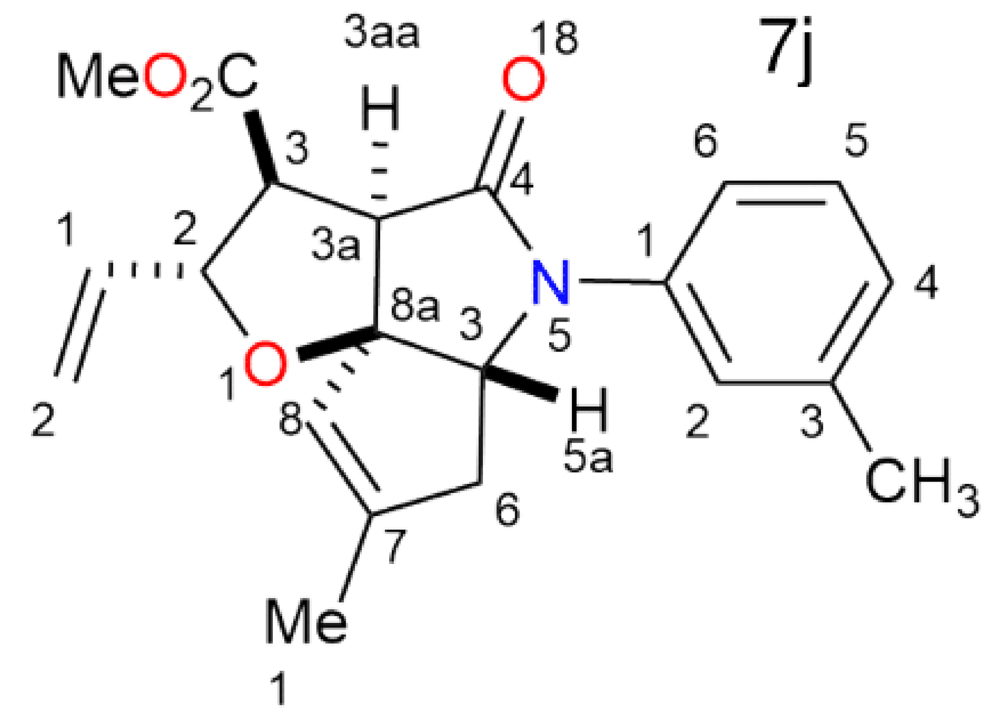

7j

FZ8799-1.JDF

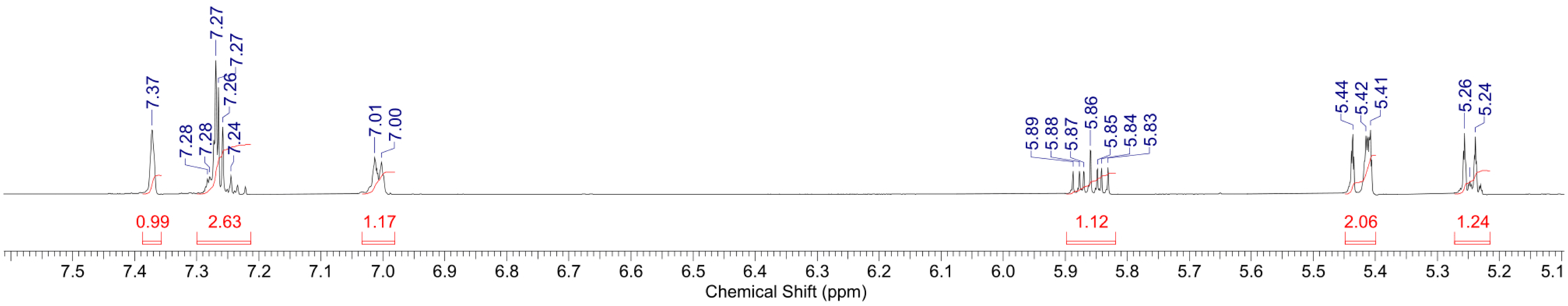

Formula $C_{21}H_{23}NO_4$	***FW*** 353.4116

Acquisition Time (sec)	1.9818	***Comment***	single_pulse	***Date***	14 Jul 2020 13:36:14		
Date Stamp	14 Jul 2020 13:37:58			***File Name***	C:\USERS\лaбa 534\DOWNLOADS\FZ8799-1.JDF		
Frequency (MHz)	600.17	***Nucleus***	1H	***Number of Transients***	8	***Origin***	ECA 600
Original Points Count	32768	***Owner***	CKP	***Points Count***	32768	***Pulse Sequence***	single_pulse.ex2
Receiver Gain	36.00	***Solvent***	CHLOROFORM-d			***Spectrum Offset (Hz)***	5410.6274
Sweep Width (Hz)	16534.39	***Temperature (degree C)***	38.000				

7j

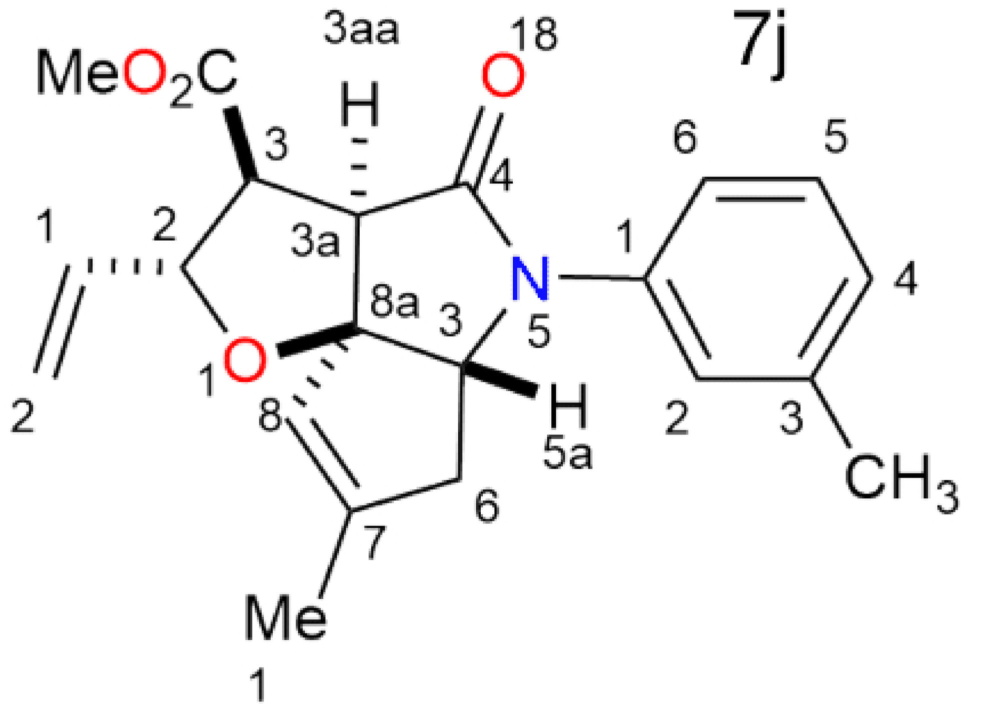

FZ8799-1.JDF

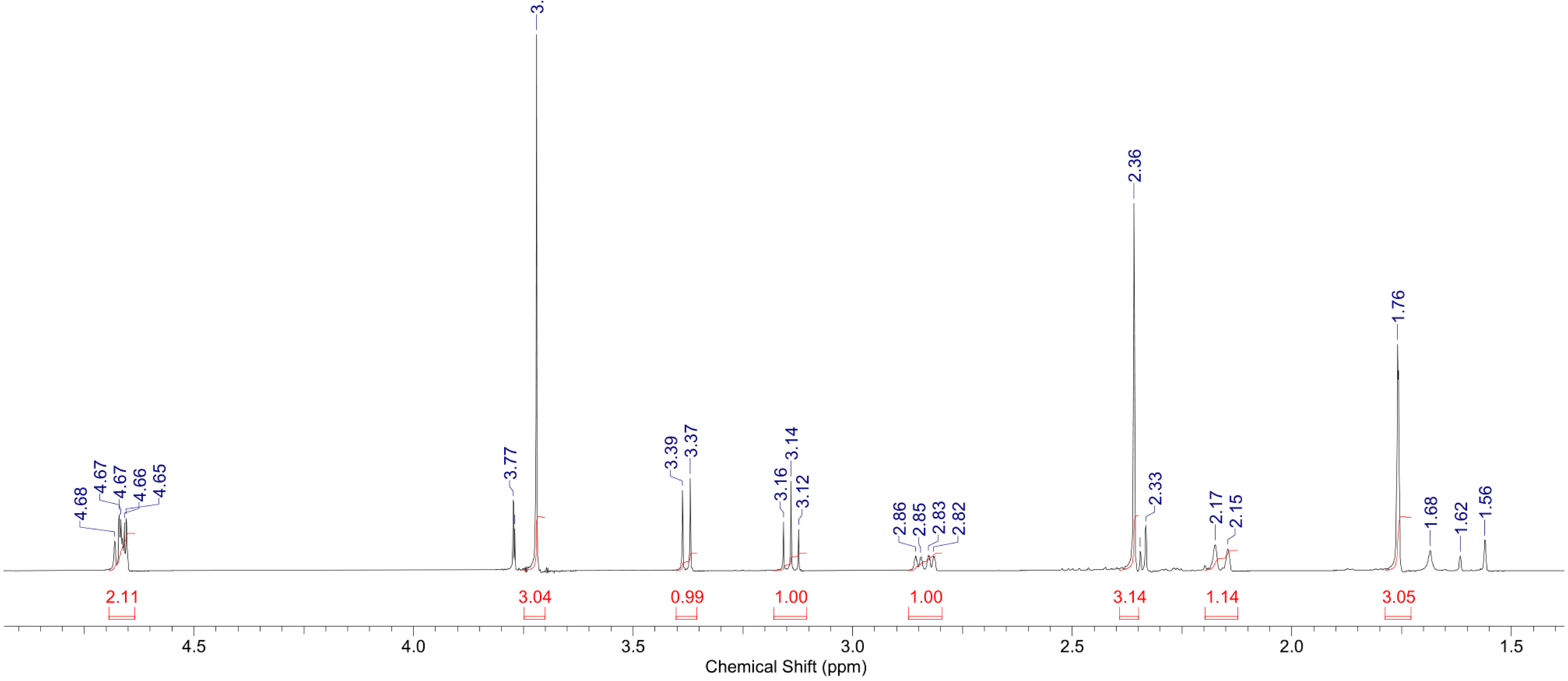

20.07.2020 18:01:20

Formula	$C_{22}H_{24}O_4$	FW	352.4236

Acquisition Time (sec)	0.6921	Comment	single pulse decoupled gated NOE			Date	17 Jul 2020 17:31:51
Date Stamp	17 Jul 2020 17:33:40			File Name	C:\USERS\Лаба534\DOWNLOADS\FZ8799-2.JDF		
Frequency (MHz)	150.91	Nucleus	13C	Number of Transients	2000	Origin	ECA 600
Original Points Count	32768	Owner	CKP	Points Count	32768	Pulse Sequence	single_pulse_dec
Receiver Gain	56.00	Solvent	CHLOROFORM-d			Spectrum Offset (Hz)	15091.3428
Sweep Width (Hz)	47348.49						

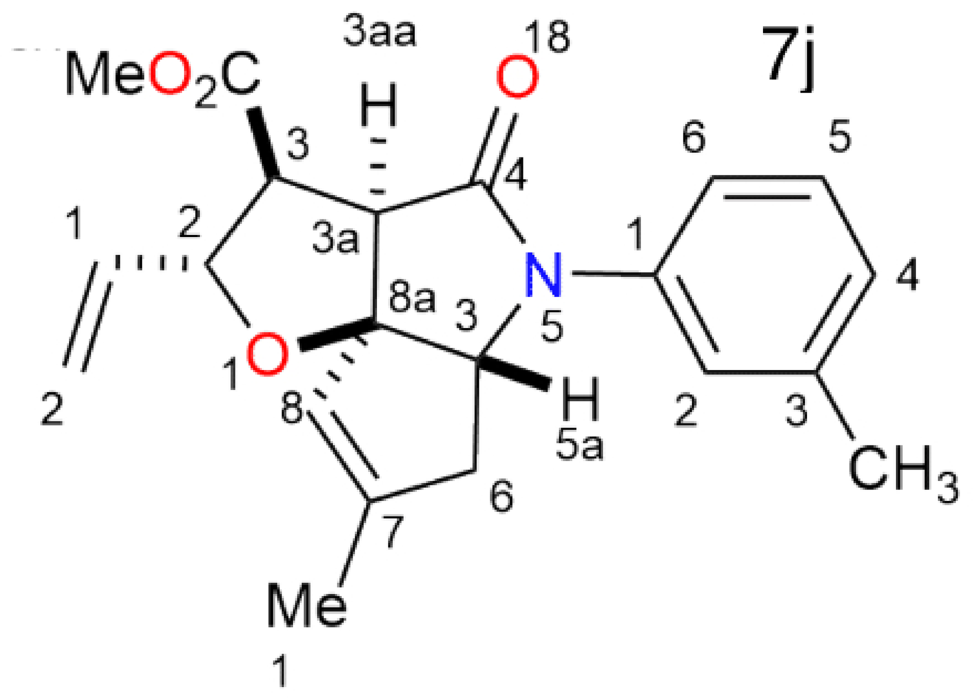

7j

FZ8799-2.JDF

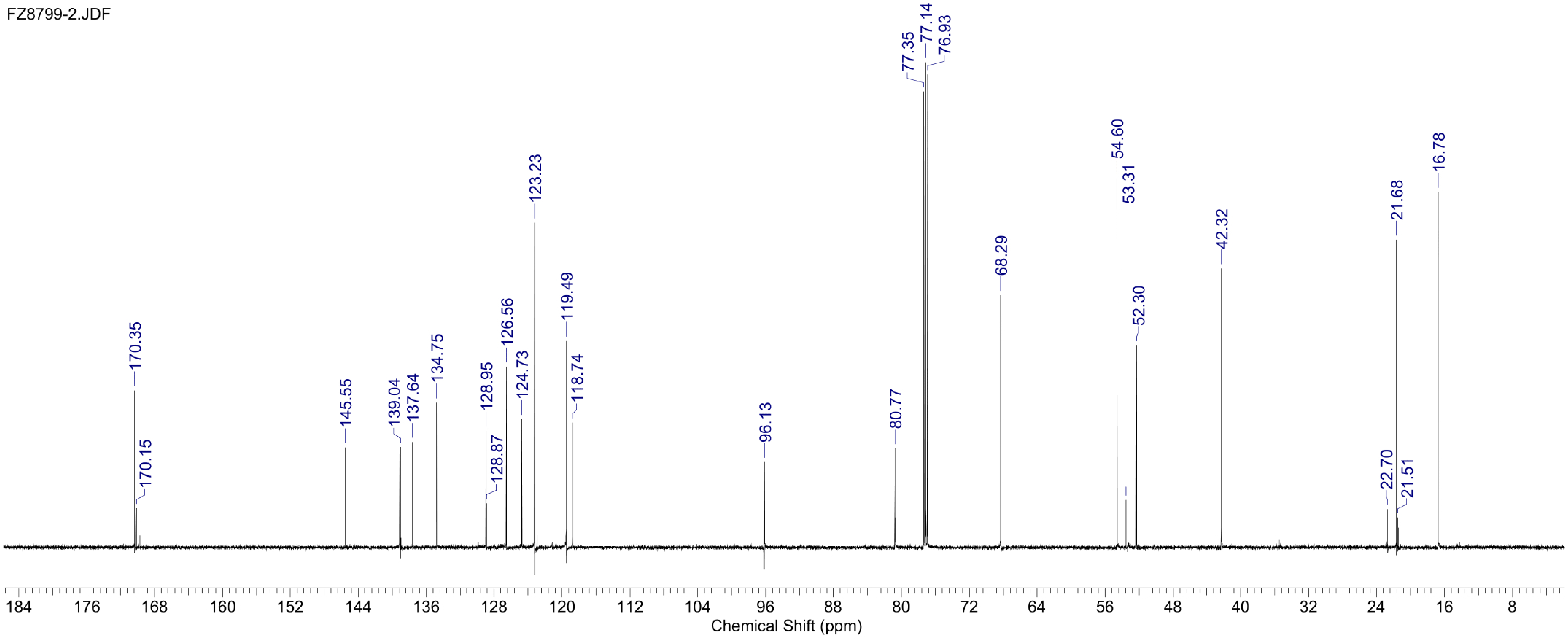

20.07.2020 18:01:51

Formula $C_{22}H_{24}O_4$	FW 352.4236

Acquisition Time (sec)	0.6921	***Comment***	single pulse decoupled gated NOE			***Date***	17 Jul 2020 17:31:51
Date Stamp	17 Jul 2020 17:33:40			***File Name***	C:\USERS\Ла6а534\DOWNLOADS\FZ8799-2.JDF		
Frequency (MHz)	150.91	***Nucleus***	13C	***Number of Transients***	2000	***Origin***	ECA 600
Original Points Count	32768	***Owner***	CKP	***Points Count***	32768	***Pulse Sequence***	single_pulse_dec
Receiver Gain	56.00	***Solvent***	CHLOROFORM-d			***Spectrum Offset (Hz)***	15091.3428
Sweep Width (Hz)	47348.49						

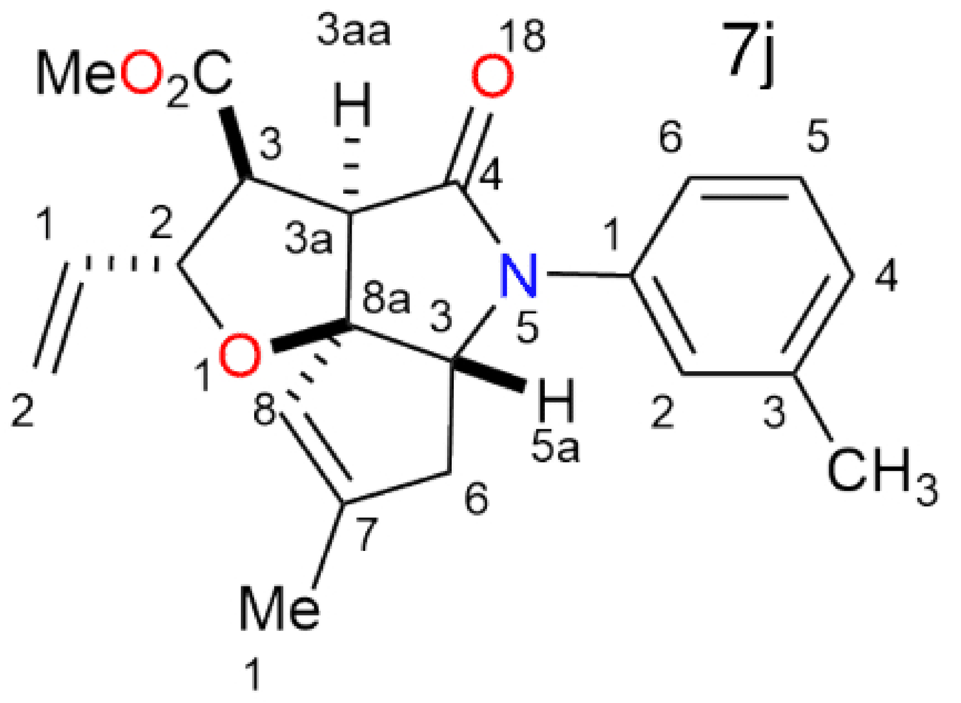

7j

FZ8799-2.JDF

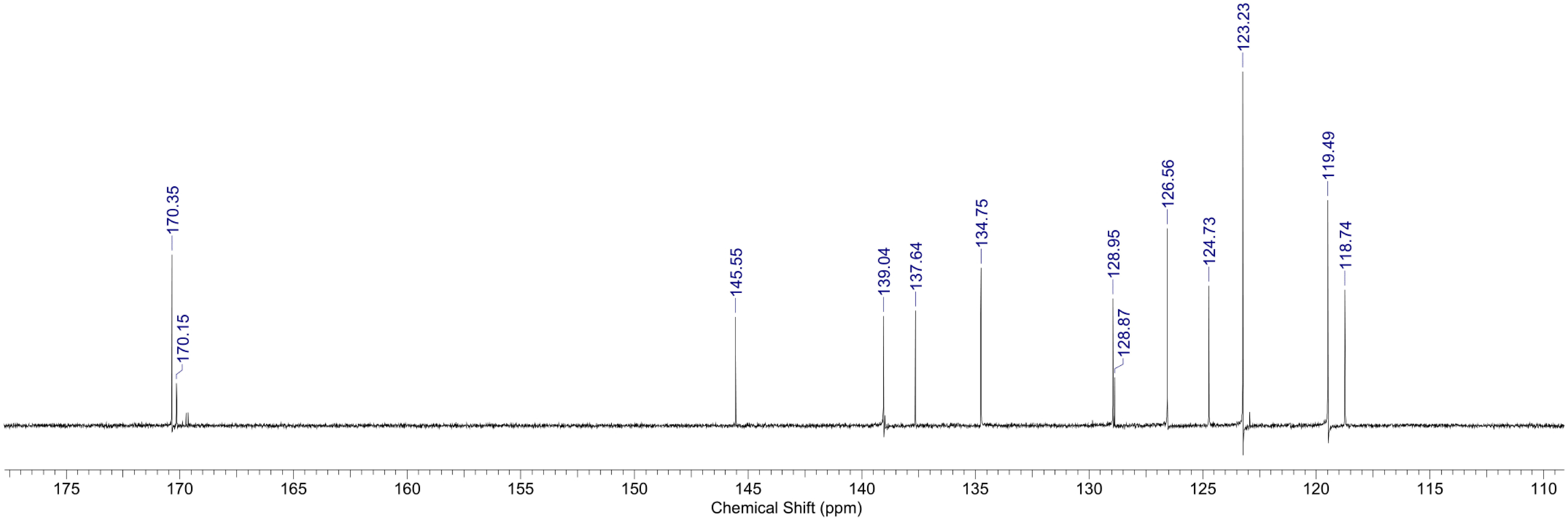

20.07.2020 18:01:59

Formula $C_{22}H_{24}O_4$	*FW* 352.4236

Acquisition Time (sec)	0.6921	***Comment***	single pulse decoupled gated NOE			***Date***	17 Jul 2020 17:31:51
Date Stamp	17 Jul 2020 17:33:40			***File Name***	C:\USERS\Лa6a534\DOWNLOADS\FZ8799-2.JDF		
Frequency (MHz)	150.91	***Nucleus***	13C	***Number of Transients***	2000	***Origin***	ECA 600
Original Points Count	32768	***Owner***	CKP	***Points Count***	32768	***Pulse Sequence***	single_pulse_dec
Receiver Gain	56.00	***Solvent***	CHLOROFORM-d			***Spectrum Offset (Hz)***	15091.3428
Sweep Width (Hz)	47348.49						

7j

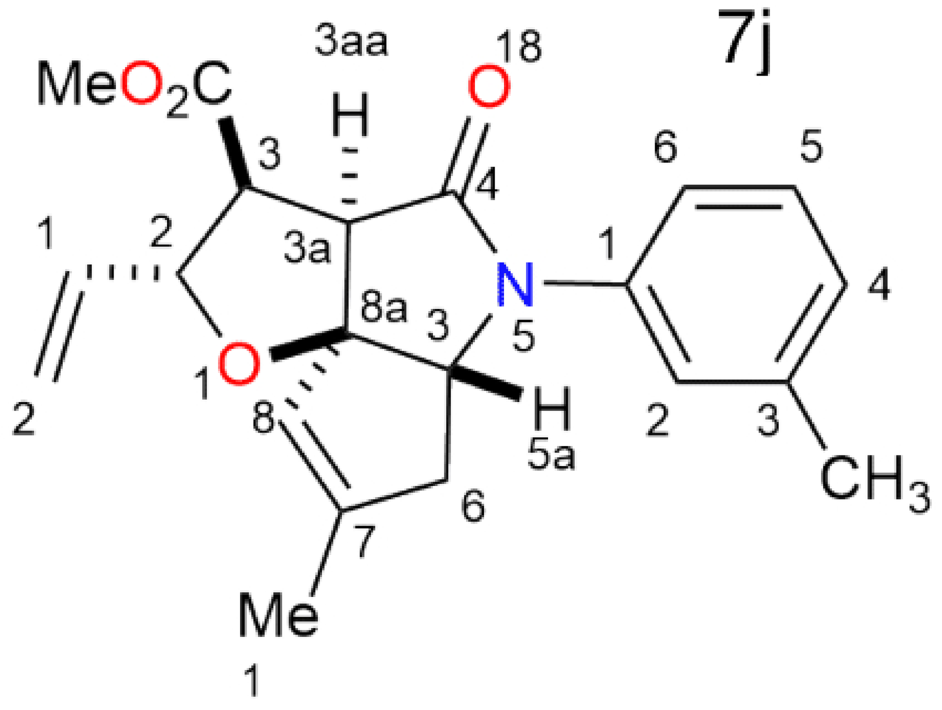

FZ8799-2.JDF

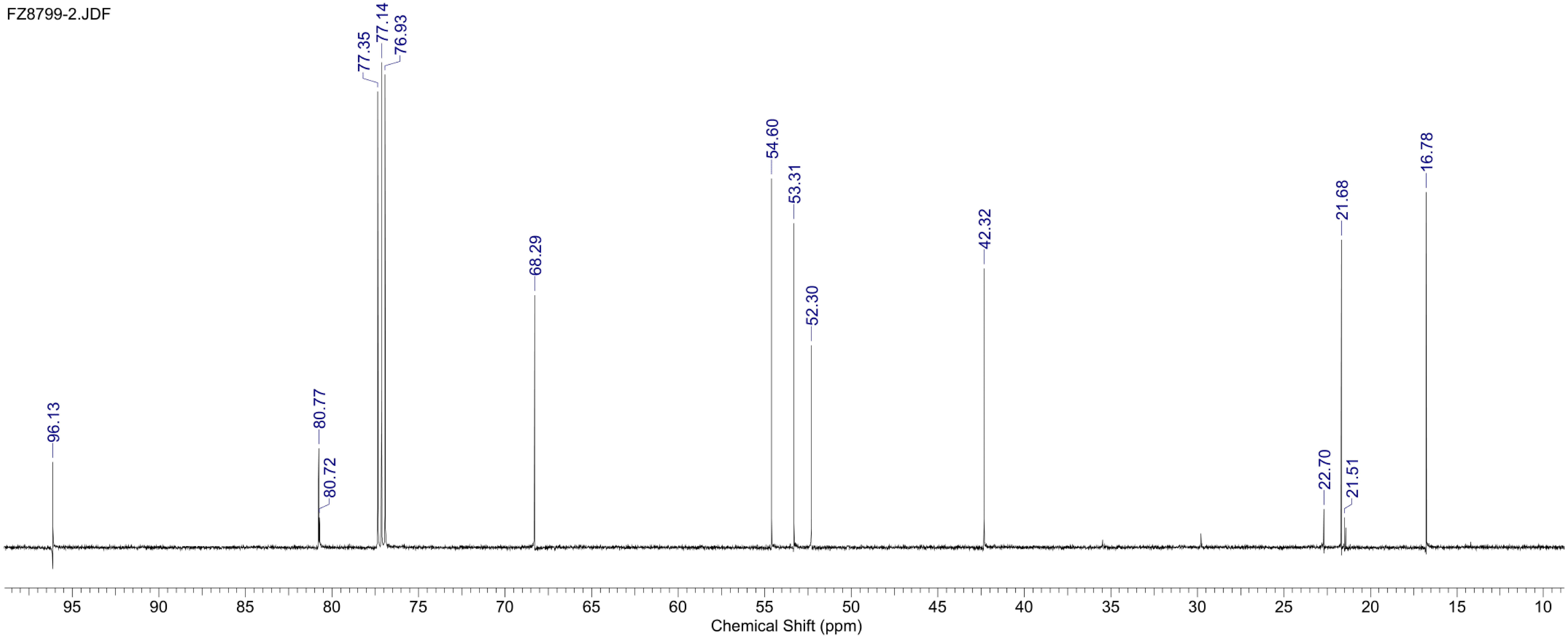

NMR spectrum data for compound 7k

Formula $C_{20}H_{21}NO_4$	FW 339.3850

**Acquisition Time (sec)**	0.9909	**Comment**	single_pulse	**Date**	27 Feb 2020 12:38:44		
**Date Stamp**	27 Feb 2020 11:58:25			**File Name**	F:\Саша\ЯМР\NMR FIDS\FZ8535-1.JDF		
**Frequency (MHz)**	600.17	**Nucleus**	1H	**Number of Transients**	8	**Origin**	ECA 600
**Original Points Count**	16384	**Owner**	CKP	**Points Count**	16384	**Pulse Sequence**	single_pulse.ex2
**Receiver Gain**	40.00	**Solvent**	CHLOROFORM-d			**Spectrum Offset (Hz)**	5410.0356
**Sweep Width (Hz)**	16534.39	**Temperature (degree C)**	23.100				

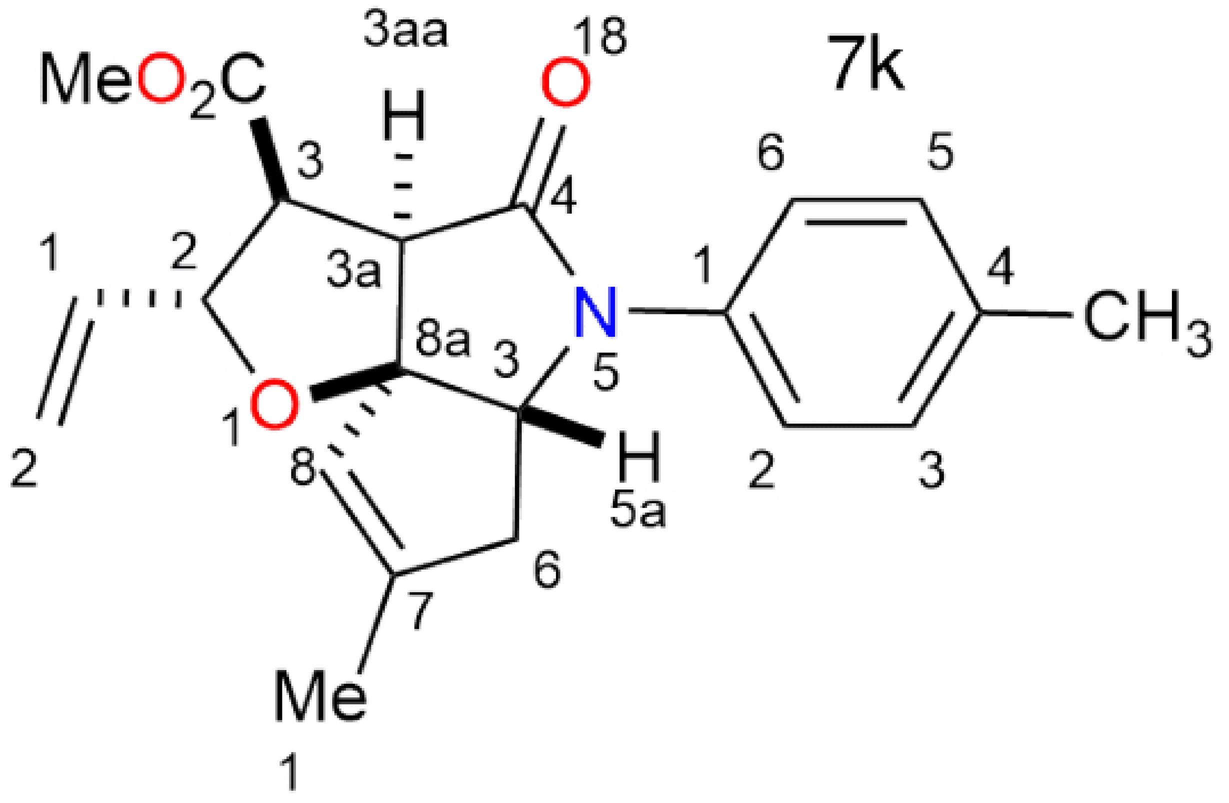

7k

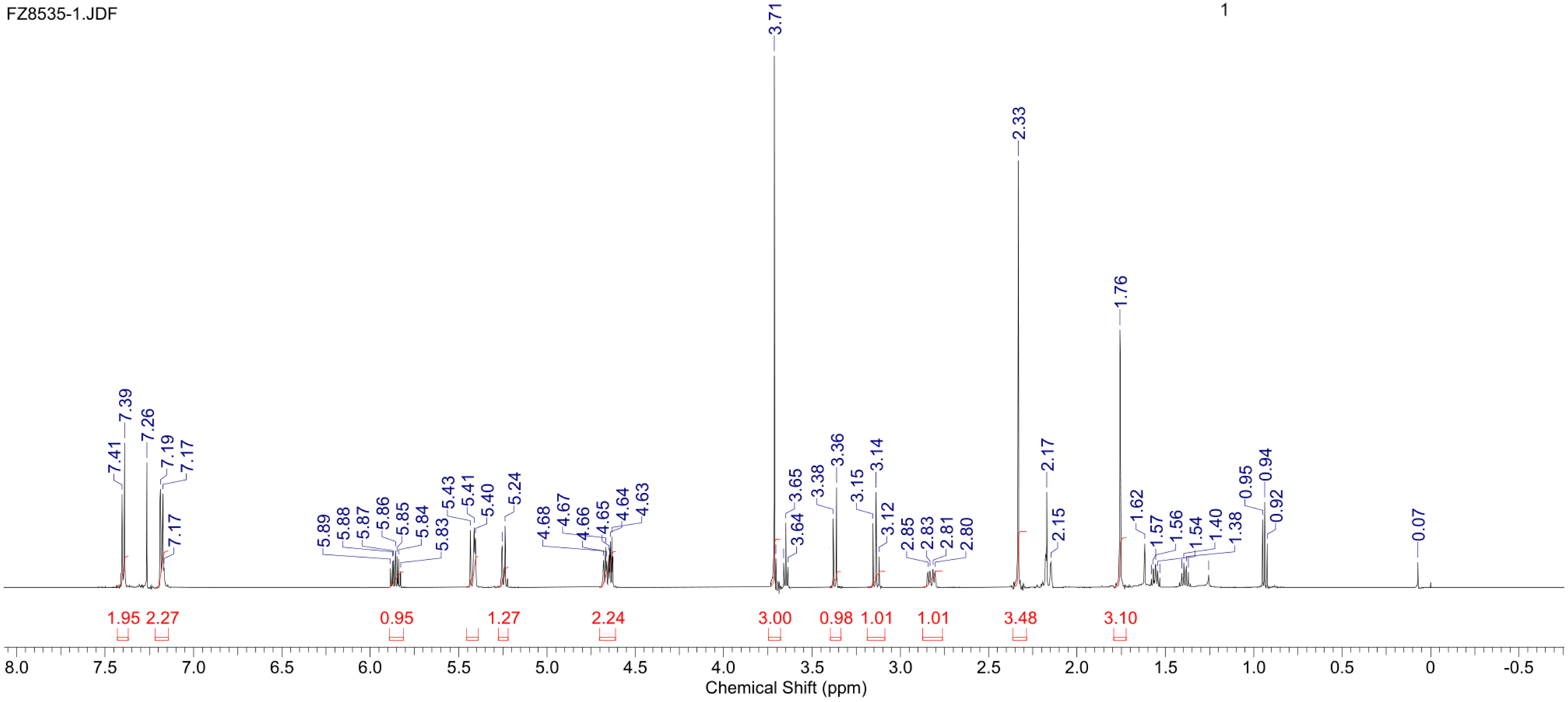

**Formula** $C_{20}H_{21}NO_4$	**FW** 339.3850

**Acquisition Time (sec)**	0.9909	**Comment**	single_pulse	**Date**	27 Feb 2020 12:38:44		
**Date Stamp**	27 Feb 2020 11:58:25			**File Name**	F:\Саша\ЯМР\NMR FIDS\FZ8535-1.JDF		
**Frequency (MHz)**	600.17	**Nucleus**	1H	**Number of Transients**	8	**Origin**	ECA 600
**Original Points Count**	16384	**Owner**	CKP	**Points Count**	16384	**Pulse Sequence**	single_pulse.ex2
**Receiver Gain**	40.00	**Solvent**	CHLOROFORM-d			**Spectrum Offset (Hz)**	5410.0356
**Sweep Width (Hz)**	16534.39	**Temperature (degree C)**	23.100				

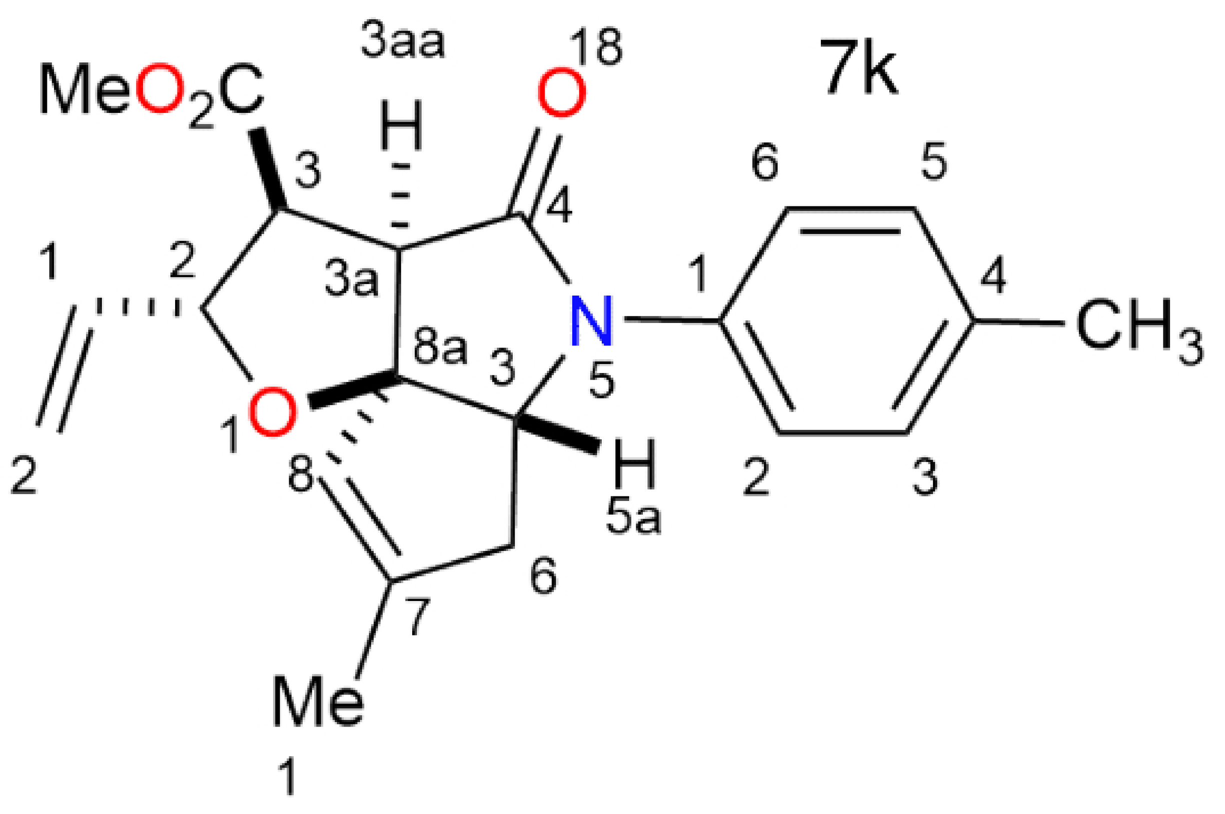

7k

FZ8535-1.JDF

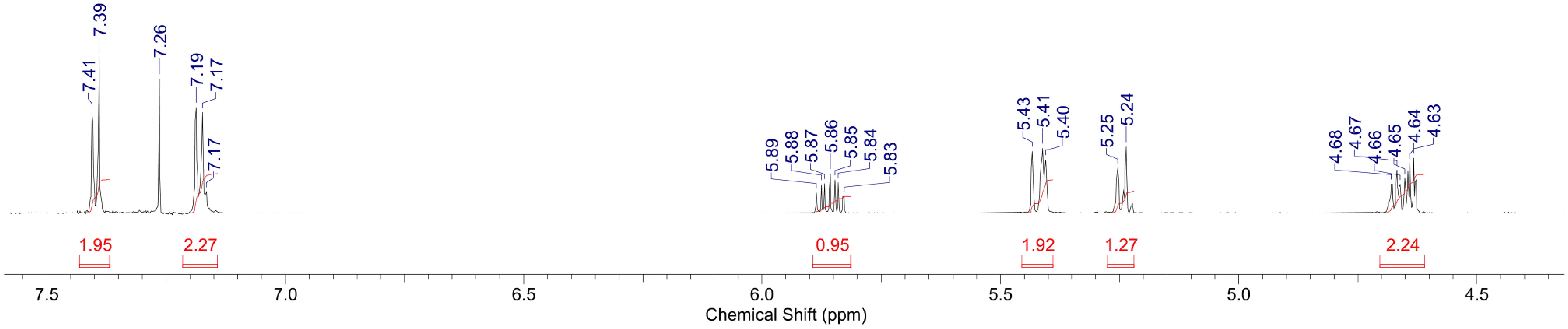

Formula $C_{20}H_{21}NO_4$	***FW*** 339.3850

Acquisition Time (sec)	0.9909	***Comment***	single_pulse	***Date***	27 Feb 2020 12:38:44		
Date Stamp	27 Feb 2020 11:58:25			***File Name***	F:\Саша\ЯМР\NMR FIDS\FZ8535-1.JDF		
Frequency (MHz)	600.17	***Nucleus***	1H	***Number of Transients***	8	***Origin***	ECA 600
Original Points Count	16384	***Owner***	CKP	***Points Count***	16384	***Pulse Sequence***	single_pulse.ex2
Receiver Gain	40.00	***Solvent***	CHLOROFORM-d			***Spectrum Offset (Hz)***	5410.0356
Sweep Width (Hz)	16534.39	***Temperature (degree C)***	23.100				

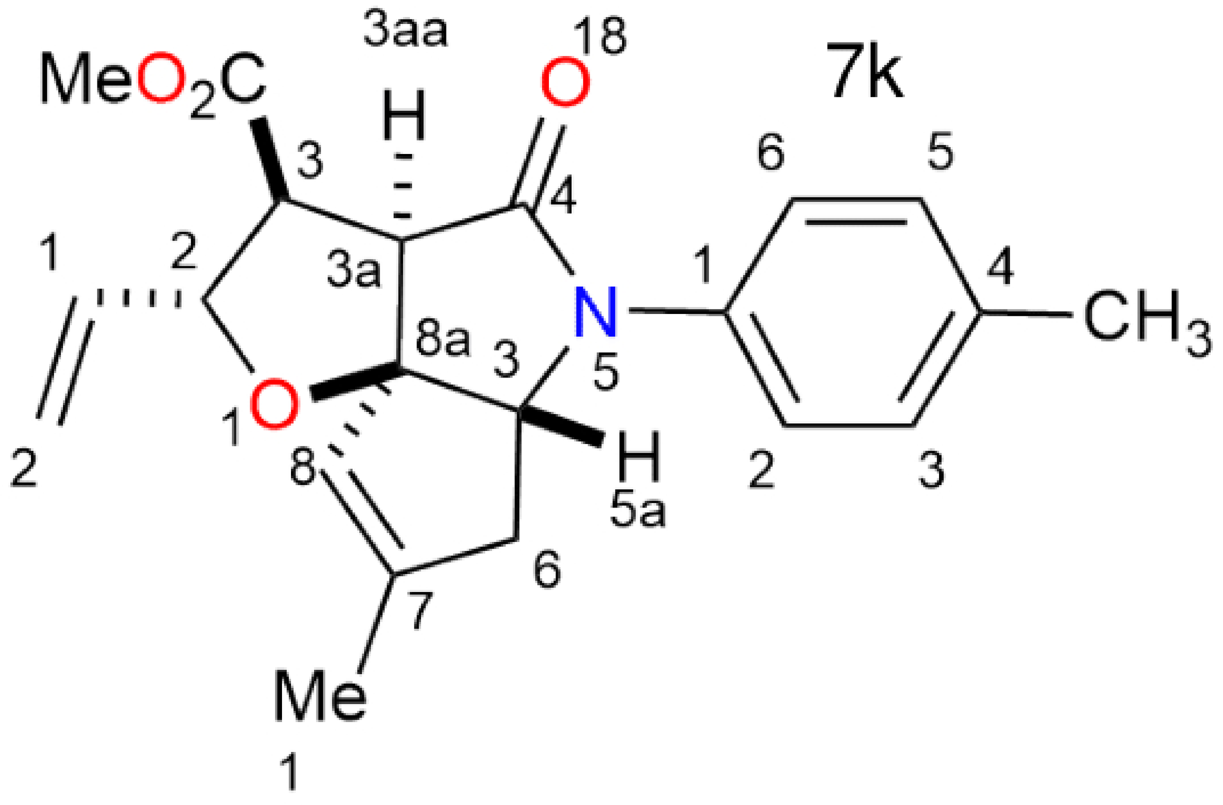

FZ8535-1.JDF

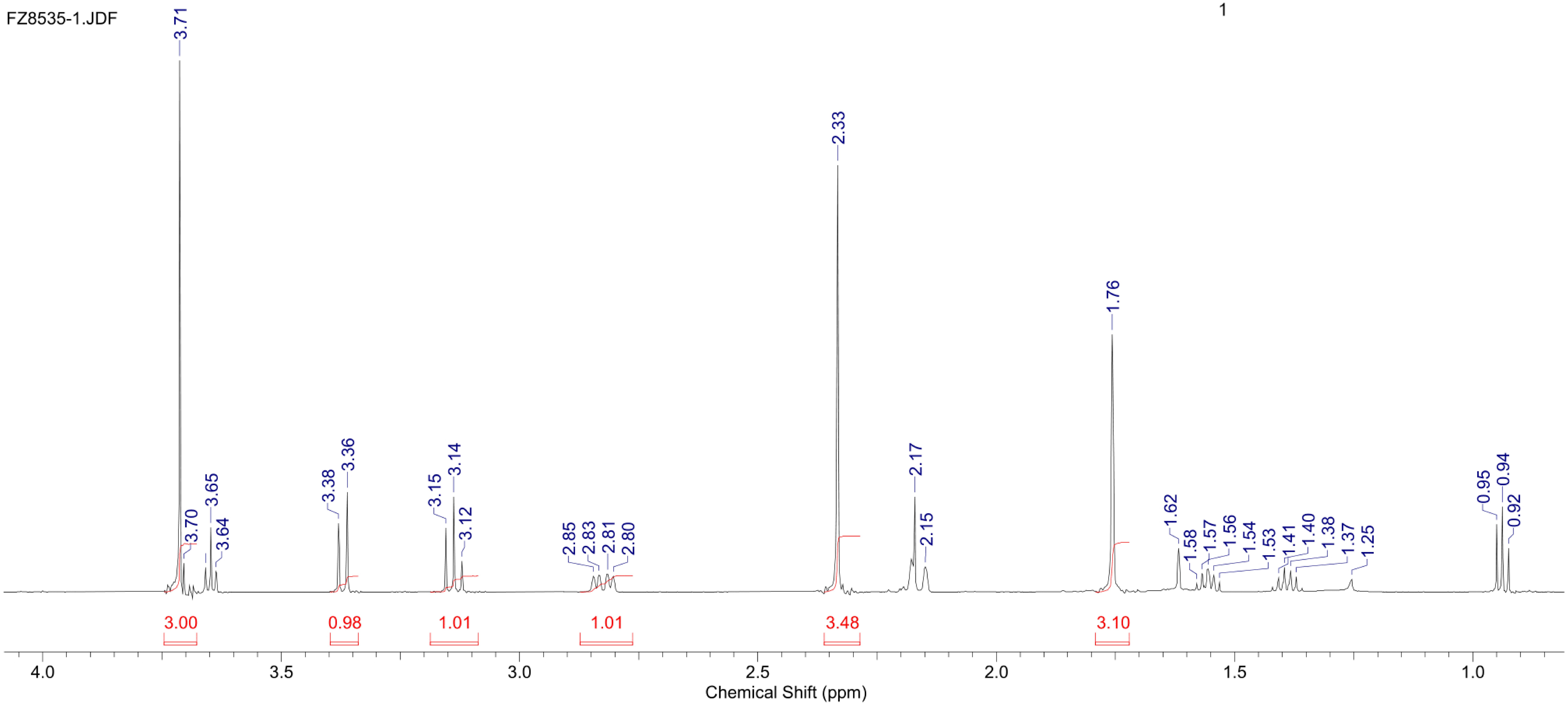

Formula $C_{21}H_{23}NO_4$	FW 353.4116

**Acquisition Time (sec)**	0.6921	**Comment**	single pulse decoupled gated NOE			**Date**	23 Jun 2020 12:25:47
**Date Stamp**	23 Jun 2020 12:27:07			**File Name**	C:\USERS\лаба 534\DESKTOP\Саша\ЯМР\NMR FIDS\FZ8695-1.JDF		
**Frequency (MHz)**	150.91	**Nucleus**	13C	**Number of Transients**	2000	**Origin**	ECA 600
**Original Points Count**	32768	**Owner**	CKP	**Points Count**	32768	**Pulse Sequence**	single_pulse_dec
**Receiver Gain**	58.00	**Solvent**	CHLOROFORM-d			**Spectrum Offset (Hz)**	15079.3525
**Sweep Width (Hz)**	47348.49	**Temperature (degree C)**	23.100				

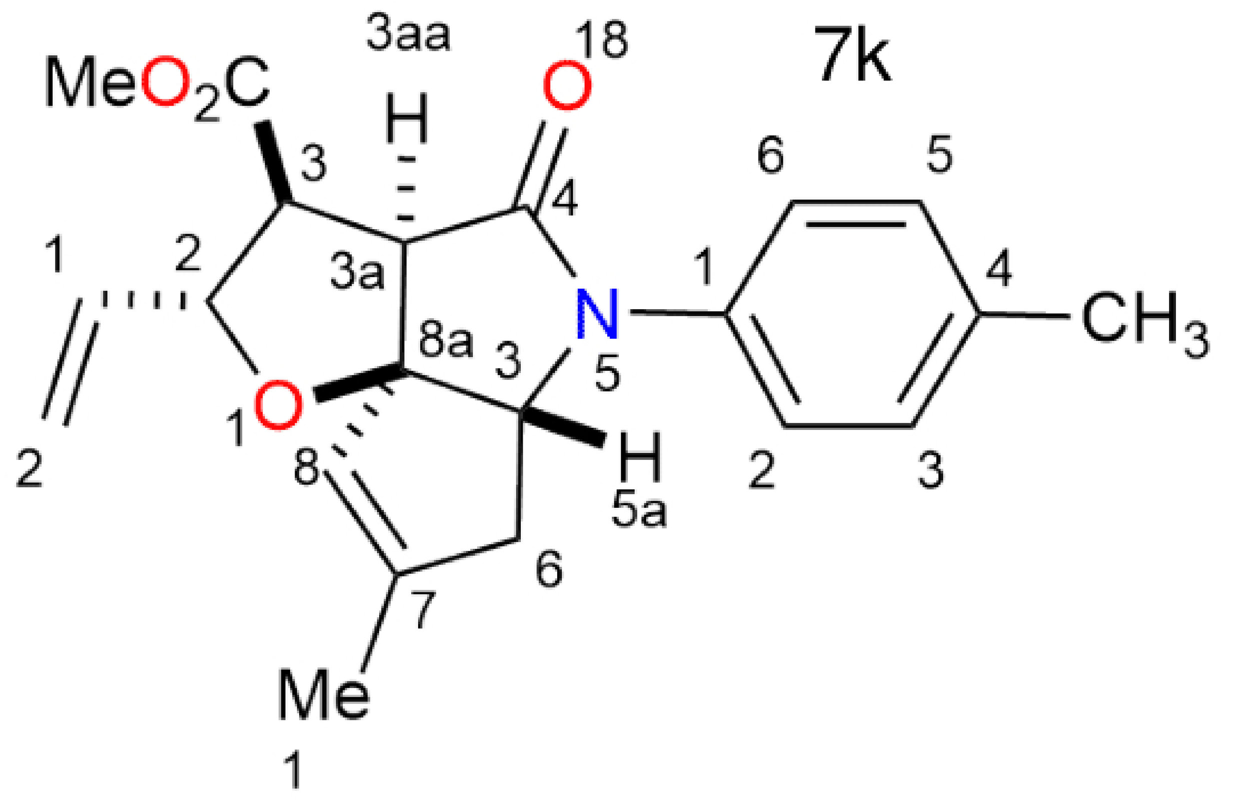

FZ8695-1.JDF

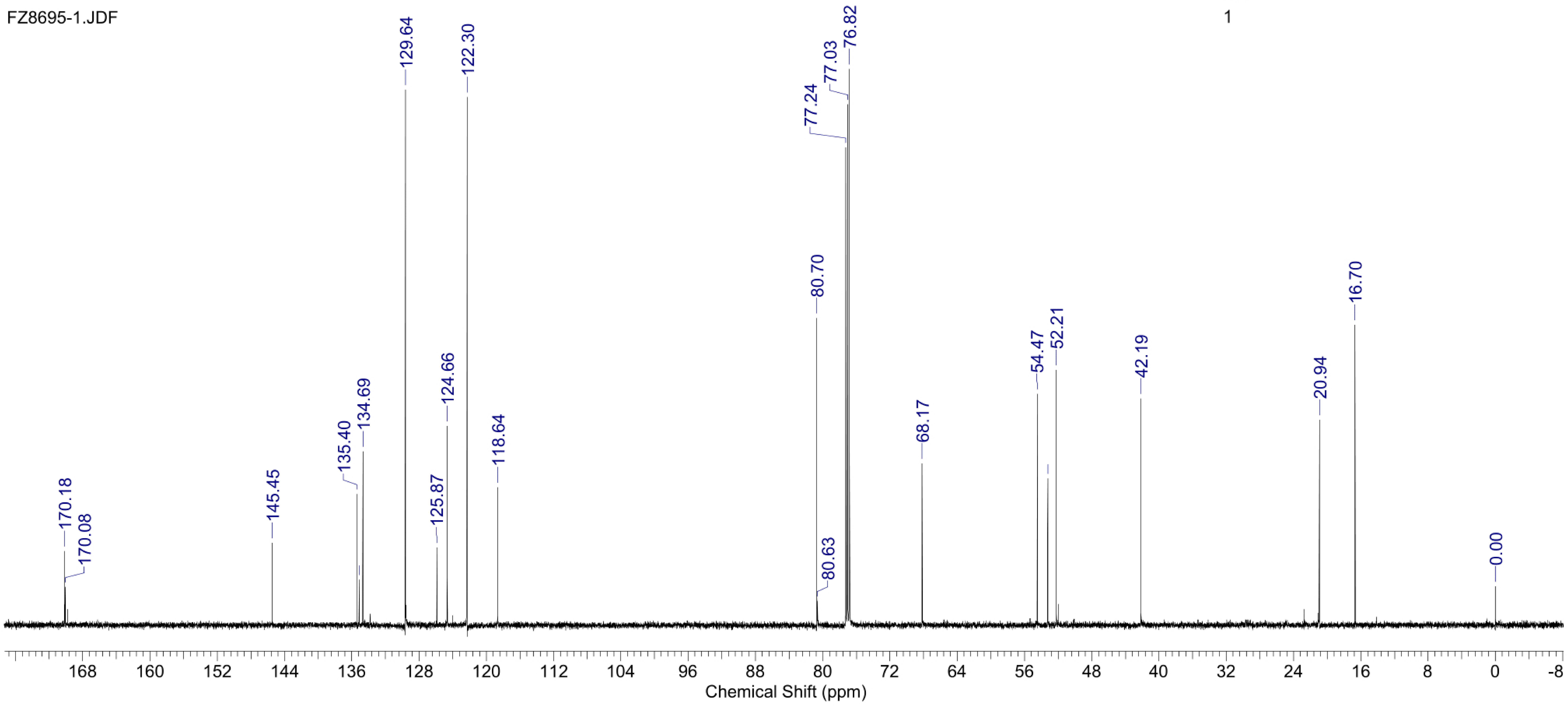

Formula $C_{21}H_{23}NO_4$	FW 353.4116

Acquisition Time (sec)	0.6921	***Comment***	single pulse decoupled gated NOE			***Date***	23 Jun 2020 12:25:47
Date Stamp	23 Jun 2020 12:27:07			***File Name***	C:\USERS\лаба 534\DESKTOP\Саша\ЯМР\NMR FIDS\FZ8695-1.JDF		
Frequency (MHz)	150.91	***Nucleus***	13C	***Number of Transients***	2000	***Origin***	ECA 600
Original Points Count	32768	***Owner***	CKP	***Points Count***	32768	***Pulse Sequence***	single_pulse_dec
Receiver Gain	58.00	***Solvent***	CHLOROFORM-d			***Spectrum Offset (Hz)***	15079.3525
Sweep Width (Hz)	47348.49	***Temperature (degree C)***	23.100				

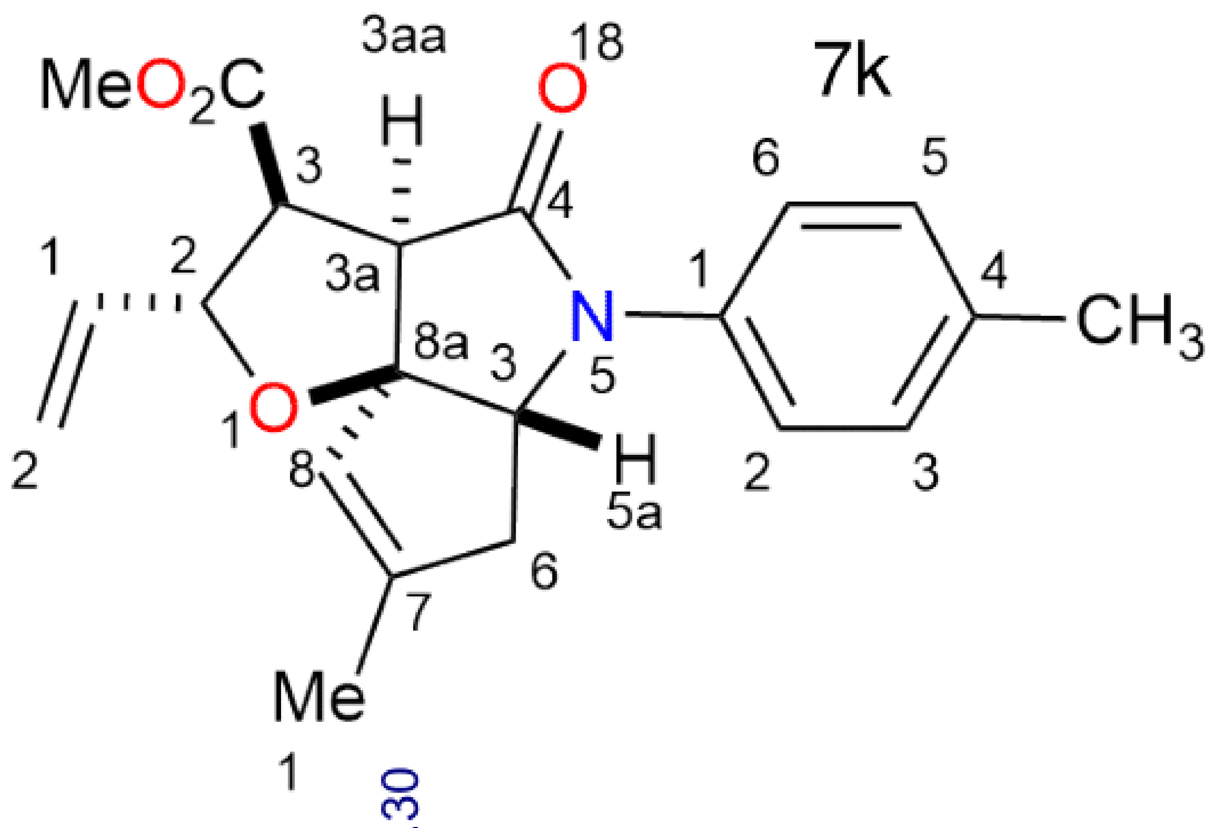

7k

FZ8695-1.JDF

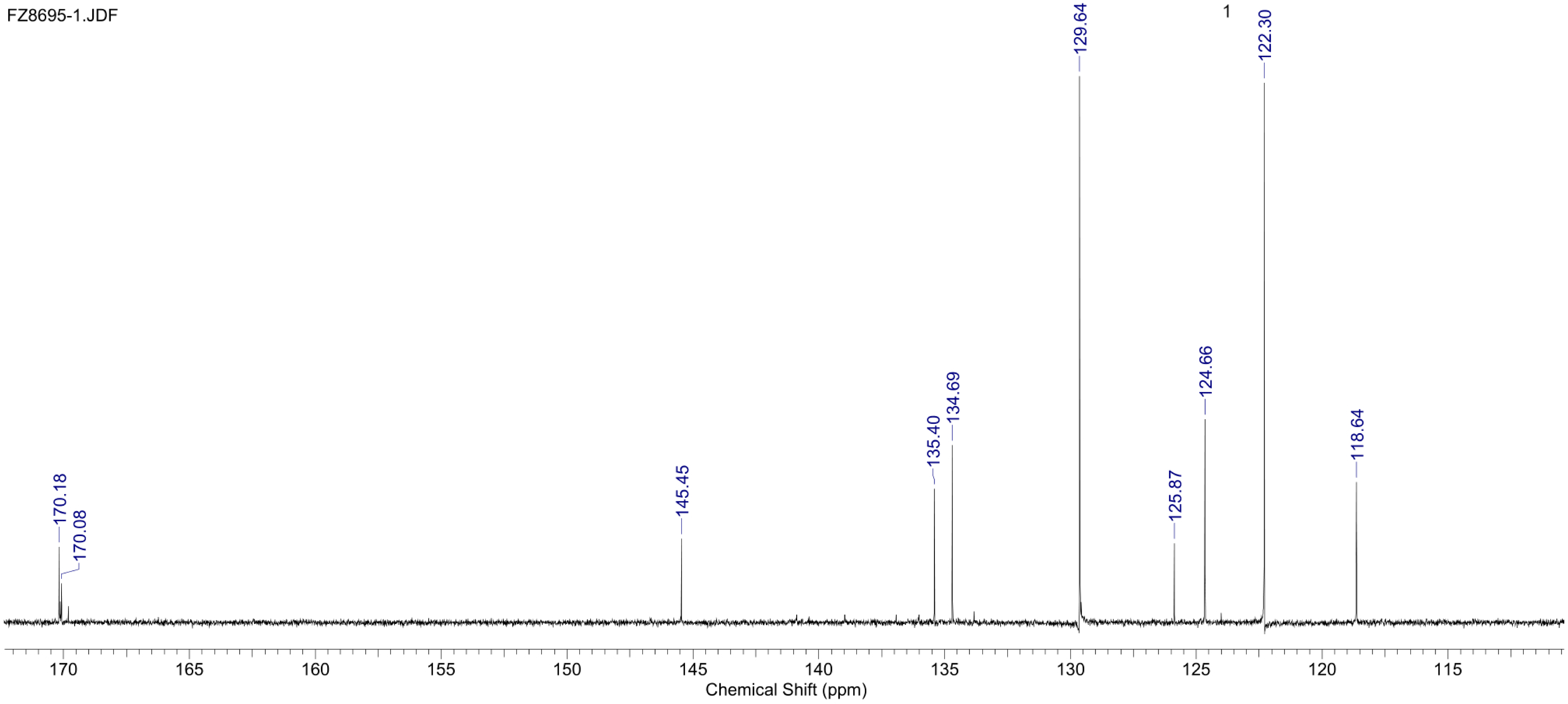

**Formula** $C_{21}H_{23}NO_4$	**FW** 353.4116

**Acquisition Time (sec)**	0.6921	**Comment**	single pulse decoupled gated NOE			**Date**	23 Jun 2020 12:25:47
**Date Stamp**	23 Jun 2020 12:27:07			**File Name**	C:\USERS\лаба 534\DESKTOP\Саша\ЯМР\NMR FIDS\FZ8695-1.JDF		
**Frequency (MHz)**	150.91	**Nucleus**	13C	**Number of Transients**	2000	**Origin**	ECA 600
**Original Points Count**	32768	**Owner**	CKP	**Points Count**	32768	**Pulse Sequence**	single_pulse_dec
**Receiver Gain**	58.00	**Solvent**	CHLOROFORM-d			**Spectrum Offset (Hz)**	15079.3525
**Sweep Width (Hz)**	47348.49	**Temperature (degree C)**	23.100				

7k

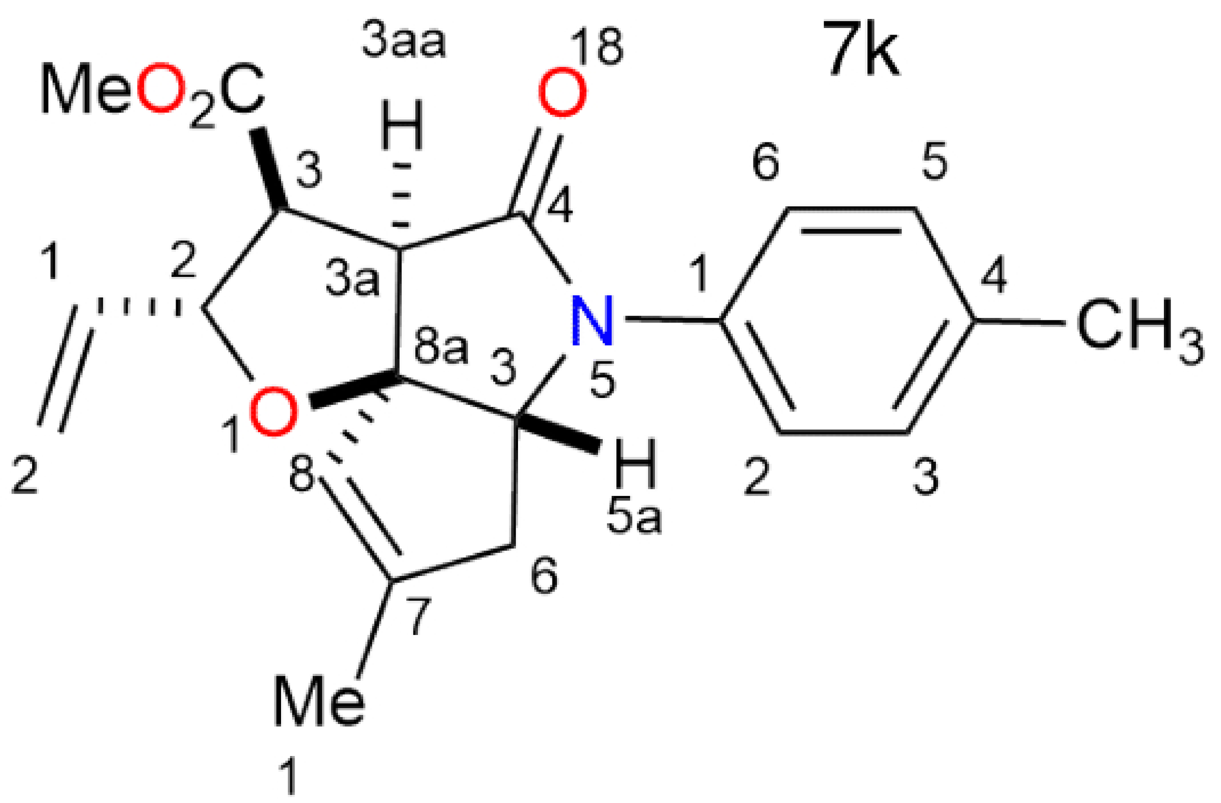

FZ8695-1.JDF

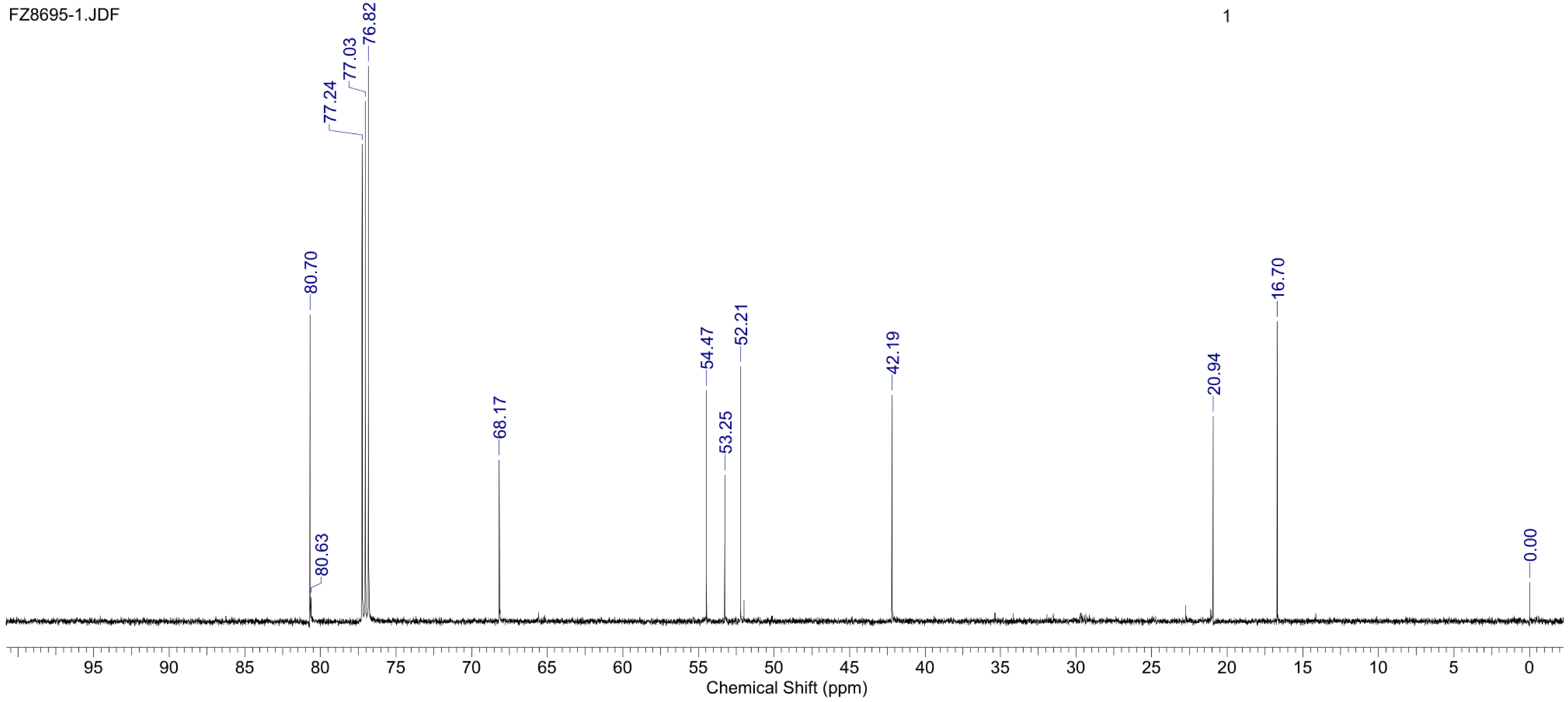

NMR spectrum data for compound 7l

**Formula**	$C_{23}H_{27}NO_4$	**FW**	381.4648

**Acquisition Time (sec)**	1.9818	**Comment**	single_pulse	**Date**	07 Jul 2020 13:22:11		
**Date Stamp**	07 Jul 2020 13:23:43			**File Name**	C:\USERS\лаба 534\DESKTOP\Саша\ЯМР\NMR FIDS\FZ8752-1.JDF		
**Frequency (MHz)**	600.17	**Nucleus**	1H	**Number of Transients**	8	**Origin**	ECA 600
**Original Points Count**	32768	**Owner**	CKP	**Points Count**	32768	**Pulse Sequence**	single_pulse.ex2
**Receiver Gain**	38.00	**Solvent**	CHLOROFORM-d			**Spectrum Offset (Hz)**	5411.6367
**Sweep Width (Hz)**	16534.39	**Temperature (degree C)**	23.300				

7l

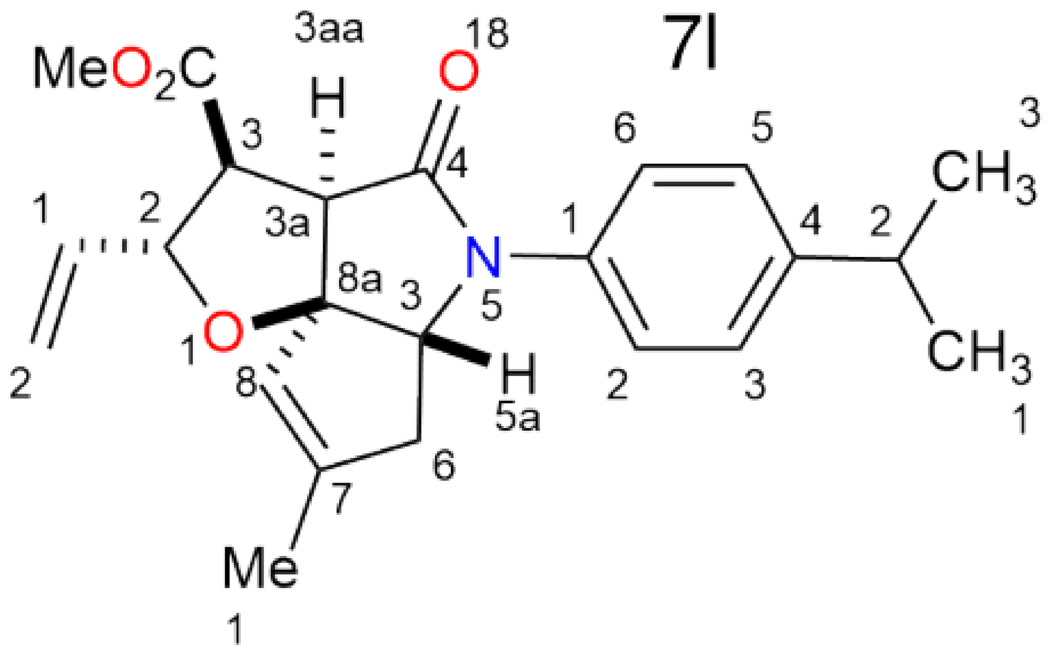

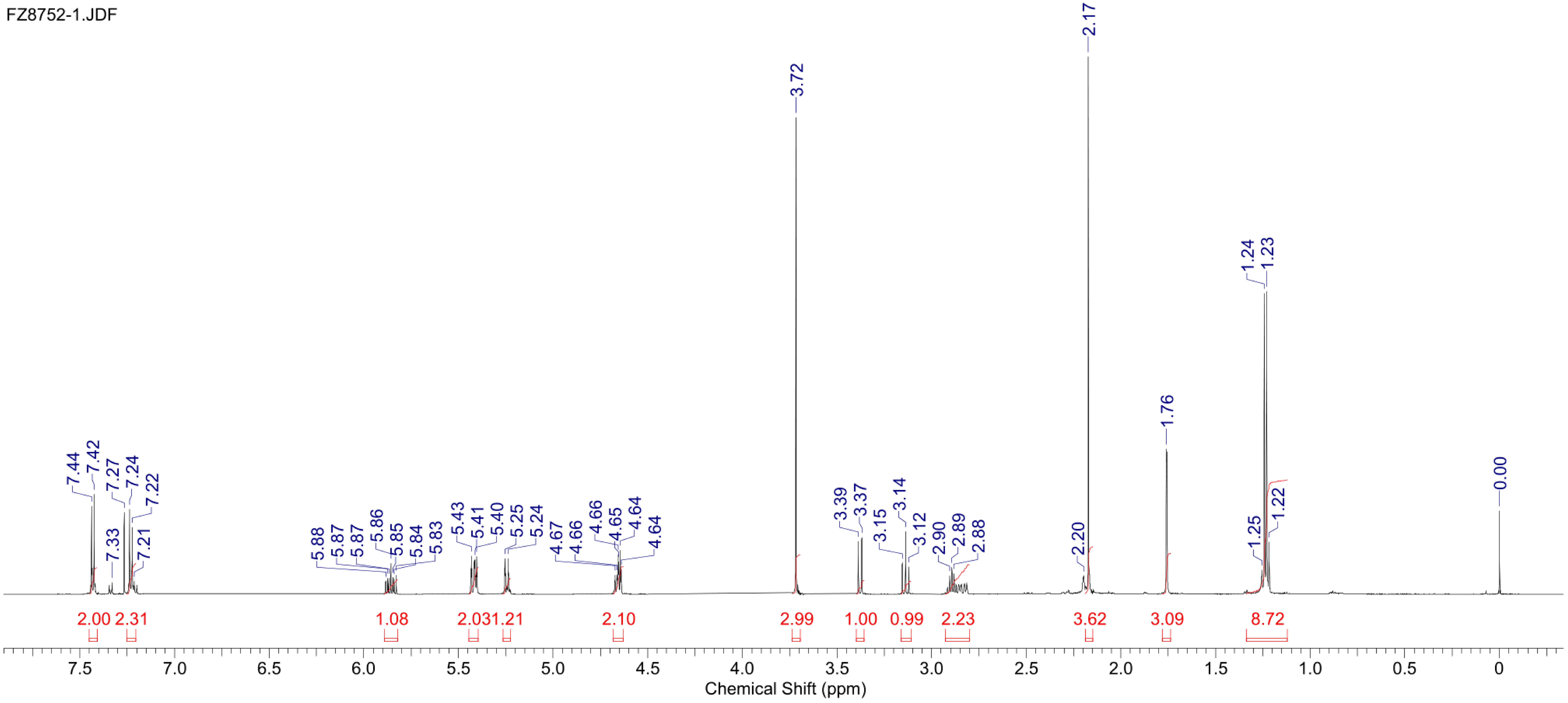

Formula $C_{23}H_{27}NO_4$	FW 381.4648

Acquisition Time (sec)	1.9818	***Comment***	single_pulse	***Date***	07 Jul 2020 13:22:11		
Date Stamp	07 Jul 2020 13:23:43			***File Name***	C:\USERS\лаба 534\DESKTOP\Саша\ЯМР\NMR FIDS\FZ8752-1.JDF		
Frequency (MHz)	600.17	***Nucleus***	1H	***Number of Transients***	8	***Origin***	ECA 600
Original Points Count	32768	***Owner***	CKP	***Points Count***	32768	***Pulse Sequence***	single_pulse.ex2
Receiver Gain	38.00	***Solvent***	CHLOROFORM-d			***Spectrum Offset (Hz)***	5411.6367
Sweep Width (Hz)	16534.39	***Temperature (degree C)***	23.300				

7l

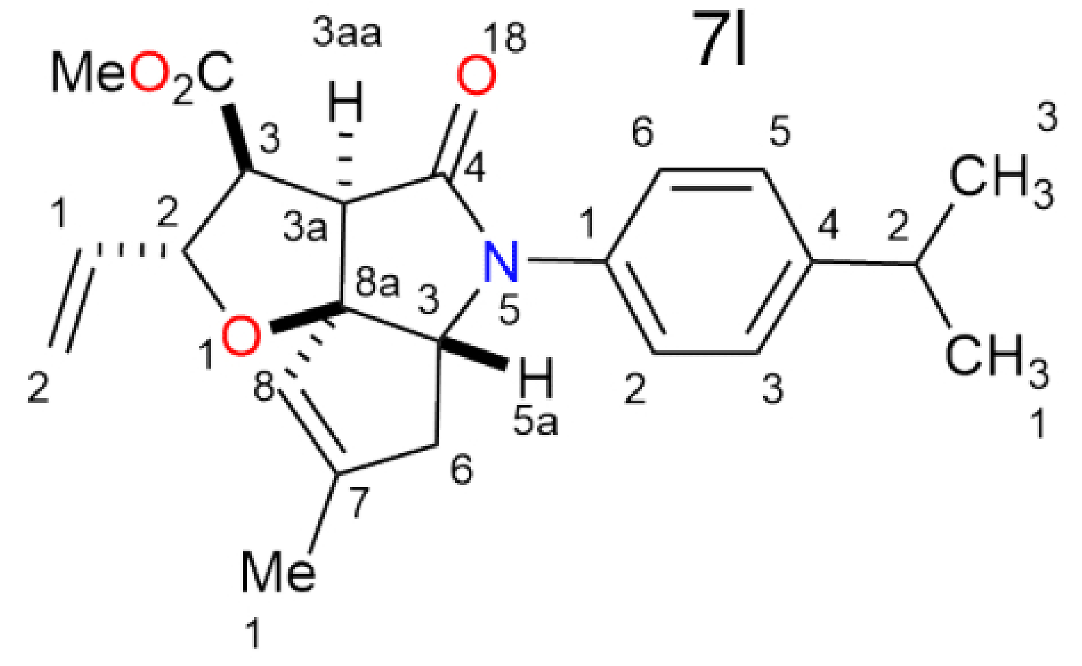

FZ8752-1.JDF

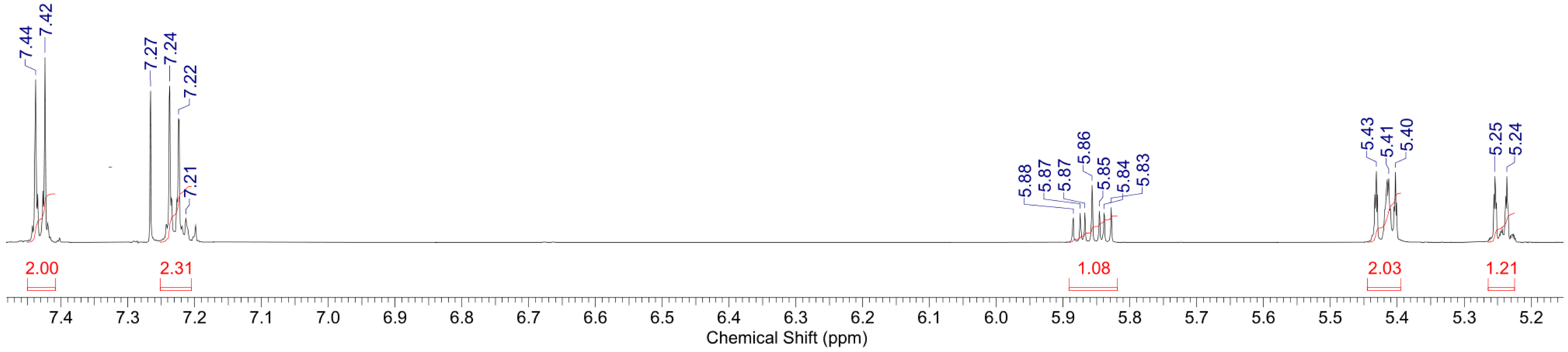

**Formula** $C_{23}H_{27}NO_4$	**FW** 381.4648

**Acquisition Time (sec)**	1.9818	**Comment**	single_pulse	**Date**	07 Jul 2020 13:22:11		
**Date Stamp**	07 Jul 2020 13:23:43			**File Name**	C:\USERS\лаба 534\DESKTOP\Саша\ЯМР\NMR FIDS\FZ8752-1.JDF		
**Frequency (MHz)**	600.17	**Nucleus**	1H	**Number of Transients**	8	**Origin**	ECA 600
**Original Points Count**	32768	**Owner**	CKP	**Points Count**	32768	**Pulse Sequence**	single_pulse.ex2
**Receiver Gain**	38.00	**Solvent**	CHLOROFORM-d			**Spectrum Offset (Hz)**	5411.6367
**Sweep Width (Hz)**	16534.39	**Temperature (degree C)**	23.300				

7l

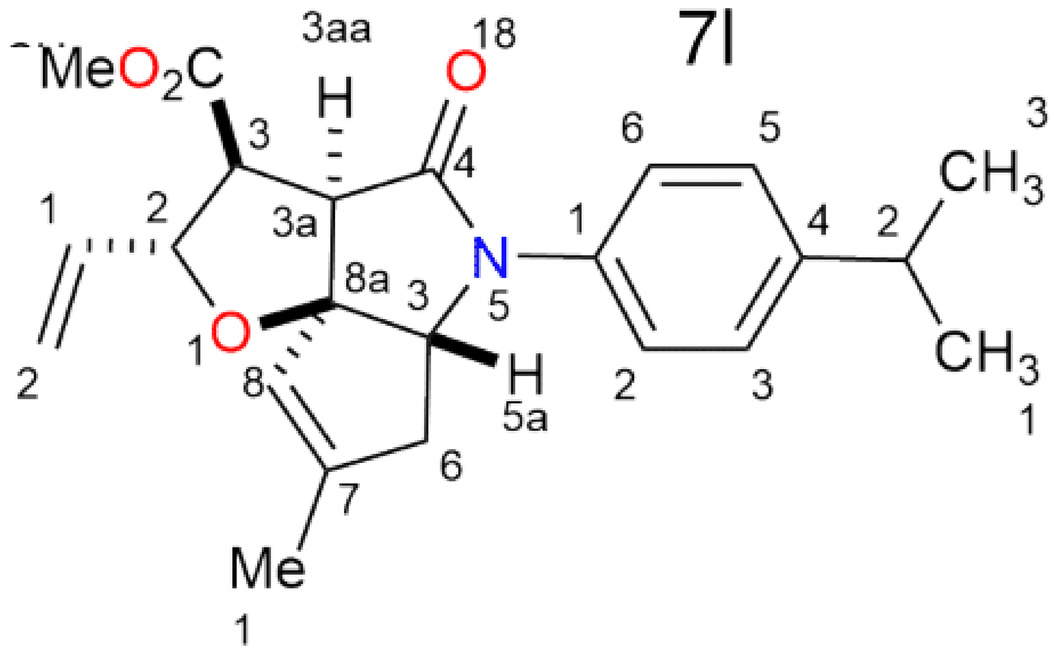

FZ8752-1.JDF

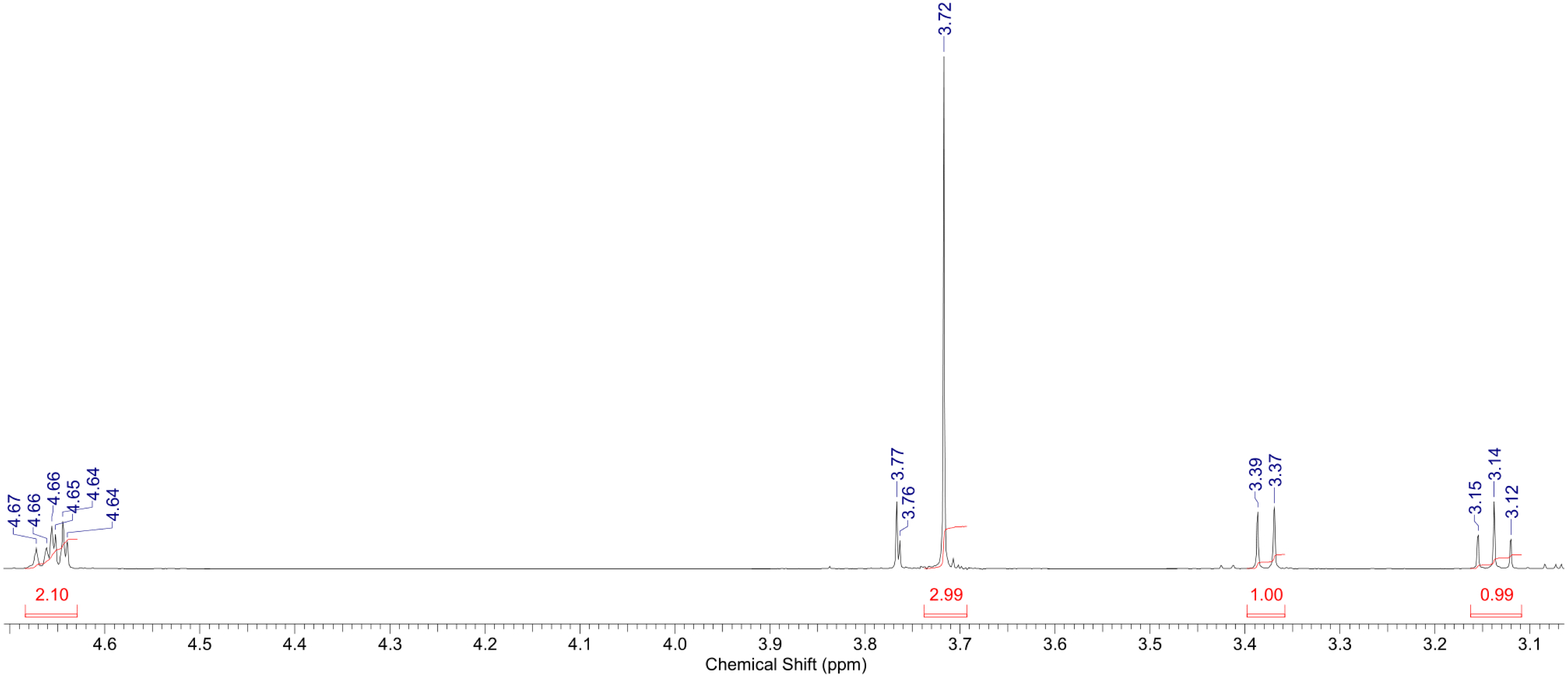

Formula $C_{23}H_{27}NO_4$	***FW*** 381.4648		

Acquisition Time (sec)	1.9818	***Comment***	single_pulse	***Date***	07 Jul 2020 13:22:11		
Date Stamp	07 Jul 2020 13:23:43			***File Name***	C:\USERS\лаба 534\DESKTOP\Саша\ЯМР\NMR FIDS\FZ8752-1.JDF		
Frequency (MHz)	600.17	***Nucleus***	1H	***Number of Transients***	8	***Origin***	ECA 600
Original Points Count	32768	***Owner***	CKP	***Points Count***	32768	***Pulse Sequence***	single_pulse.ex2
Receiver Gain	38.00	***Solvent***	CHLOROFORM-d			***Spectrum Offset (Hz)***	5411.6367
Sweep Width (Hz)	16534.39	***Temperature (degree C)***	23.300				

7l

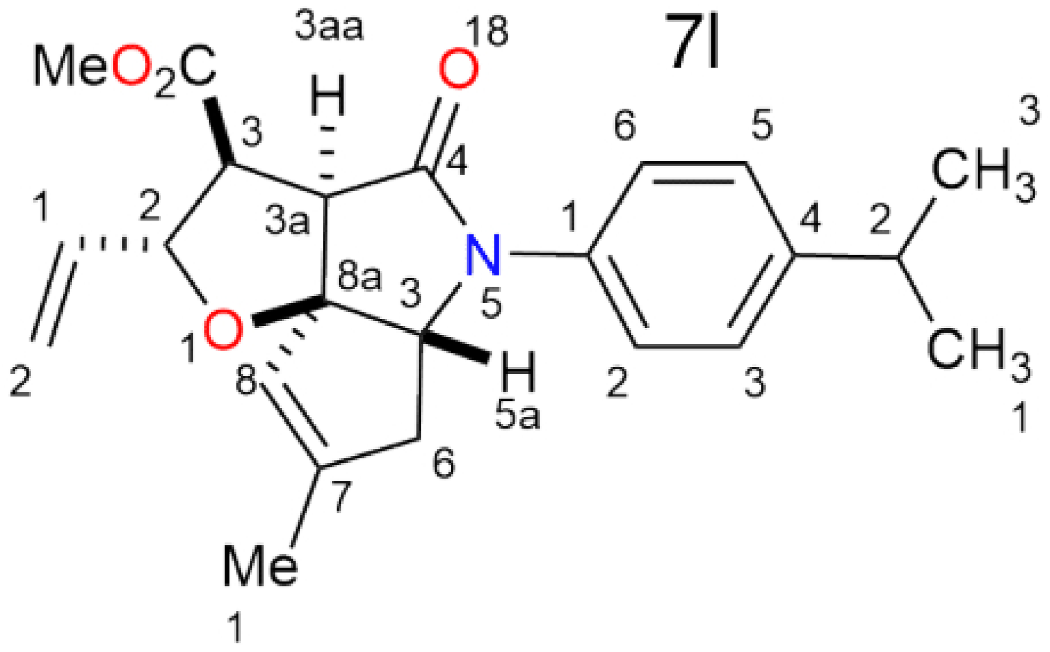

FZ8752-1.JDF

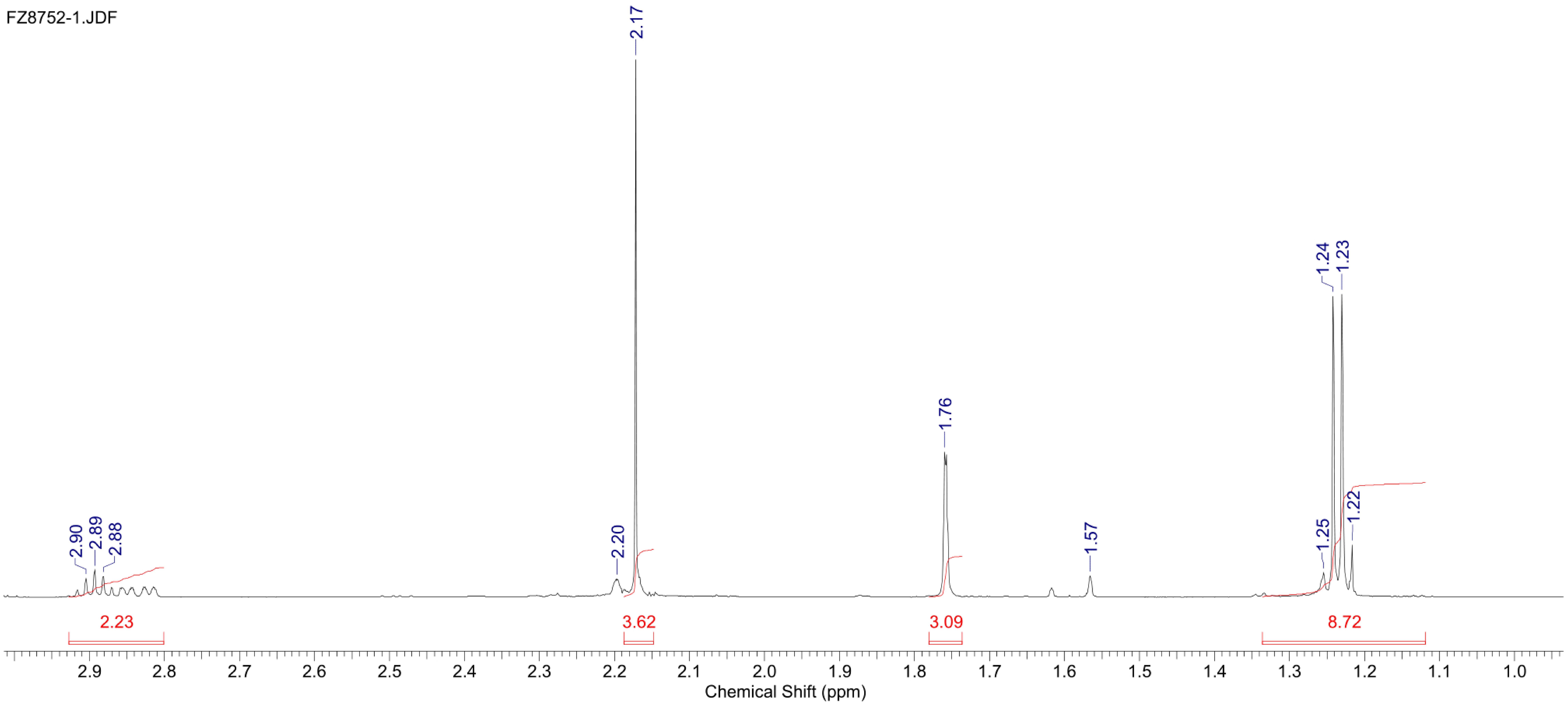

13.07.2020 14:56:34

**Formula** $C_{23}H_{27}NO_4$	**FW** 381.4648

**Acquisition Time (sec)**	0.6921	**Comment**	single pulse decoupled gated NOE			**Date**	11 Jul 2020 07:44:22
**Date Stamp**	11 Jul 2020 07:46:00			**File Name**	C:\USERS\Лa6a534\DOWNLOADS\FZ8775-1.JDF		
**Frequency (MHz)**	150.91	**Nucleus**	13C	**Number of Transients**	2000	**Origin**	ECA 600
**Original Points Count**	32768	**Owner**	CKP	**Points Count**	32768	**Pulse Sequence**	single_pulse_dec
**Receiver Gain**	58.00	**Solvent**	CHLOROFORM-d			**Spectrum Offset (Hz)**	15079.3525
**Sweep Width (Hz)**	47348.49	**Temperature (degree C)**	29.000				

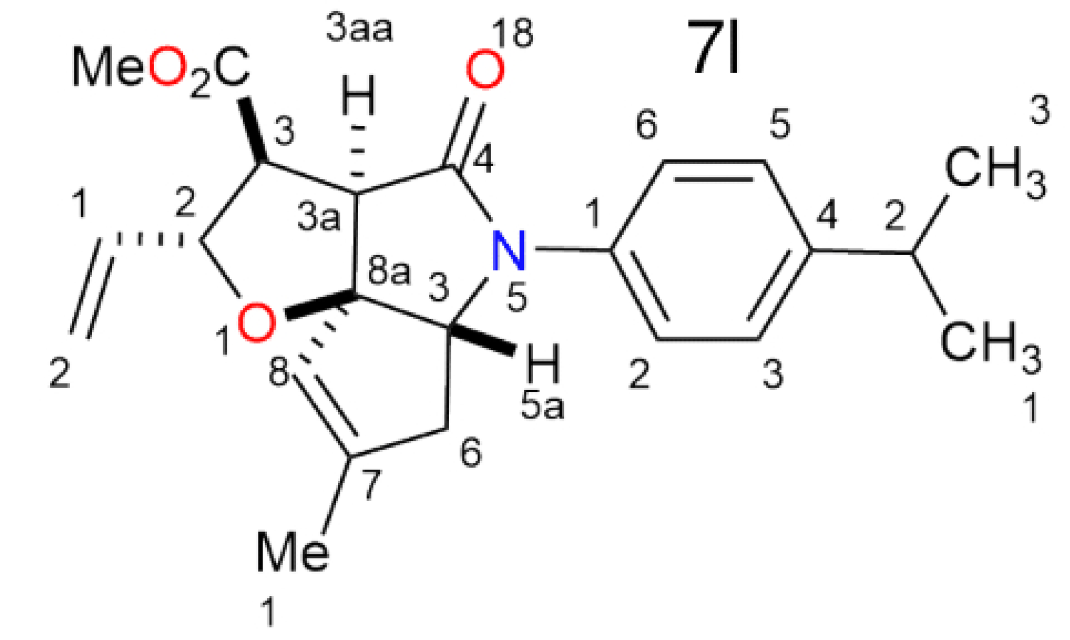

7l

FZ8775-1.JDF

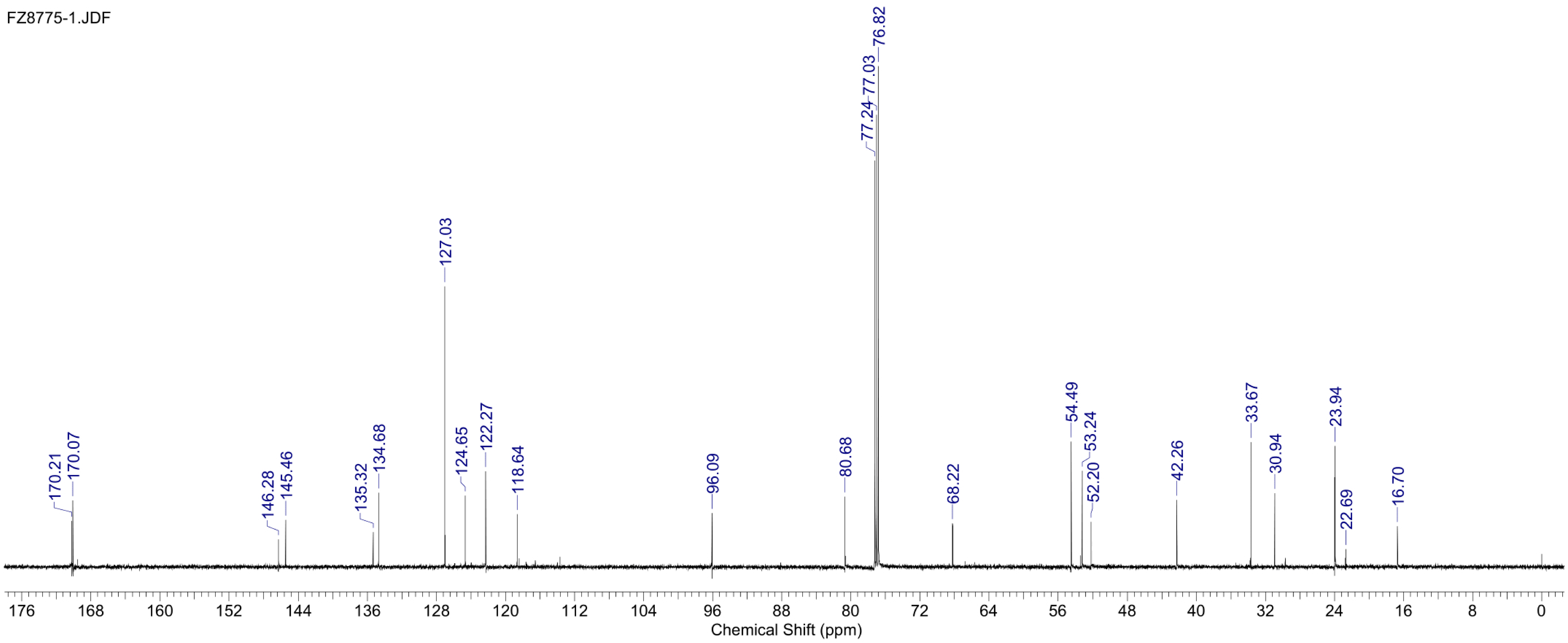

13.07.2020 14:59:19

Formula	$C_{23}H_{27}NO_4$	FW	381.4648

Acquisition Time (sec)	0.6921	Comment	single pulse decoupled gated NOE	Date	11 Jul 2020 07:44:22
Date Stamp	11 Jul 2020 07:46:00	File Name	C:\USERS\Лa6a534\DOWNLOADS\FZ8775-1.JDF		
Frequency (MHz)	150.91	Nucleus	13C	Number of Transients	2000
Origin	ECA 600				
Original Points Count	32768	Owner	CKP	Points Count	32768
Pulse Sequence	single_pulse_dec				
Receiver Gain	58.00	Solvent	CHLOROFORM-d	Spectrum Offset (Hz)	15079.3525
Sweep Width (Hz)	47348.49	Temperature (degree C)	29.000		

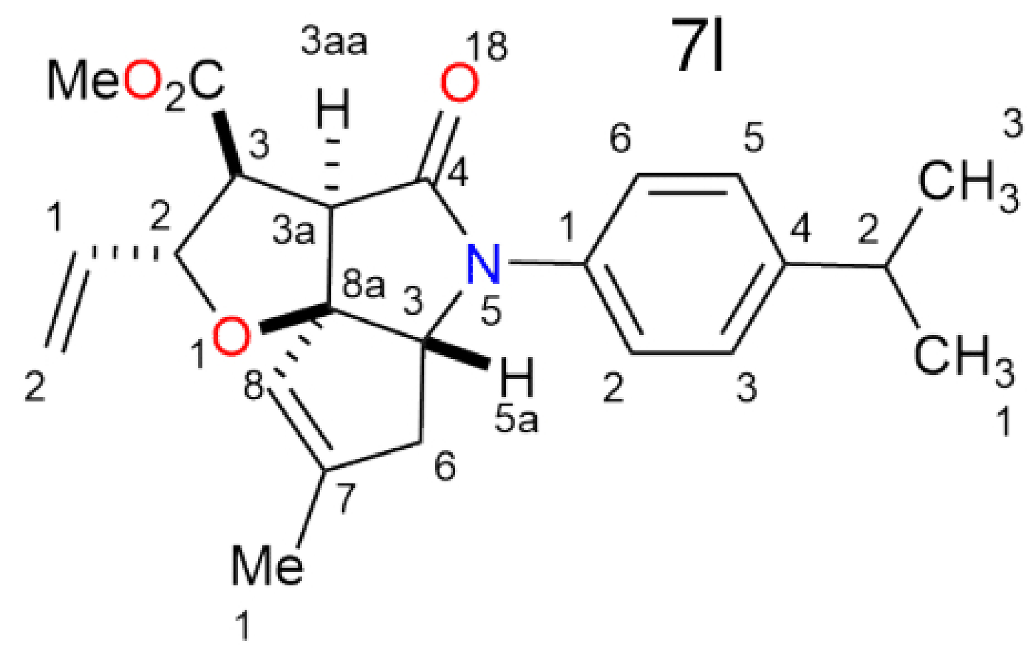

FZ8775-1.JDF

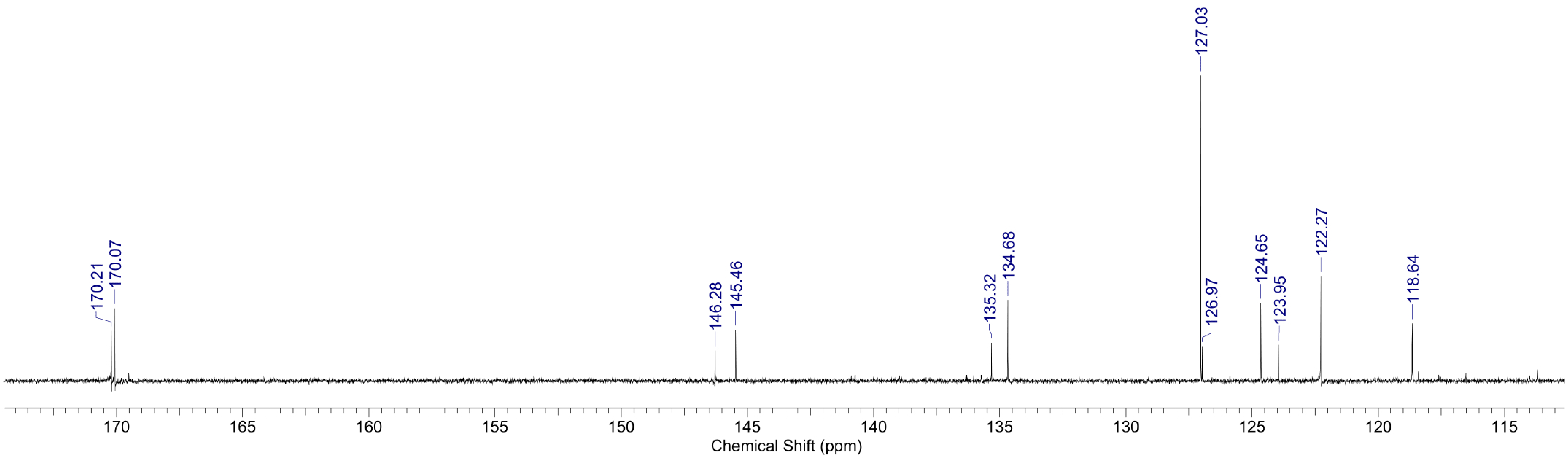

13.07.2020 14:59:36

**Formula** $C_{23}H_{27}NO_4$	**FW** 381.4648

**Acquisition Time (sec)**	0.6921	**Comment**	single pulse decoupled gated NOE			**Date**	11 Jul 2020 07:44:22
**Date Stamp**	11 Jul 2020 07:46:00			**File Name**	C:\USERS\Лаба534\DOWNLOADS\FZ8775-1.JDF		
**Frequency (MHz)**	150.91	**Nucleus**	13C	**Number of Transients**	2000	**Origin**	ECA 600
**Original Points Count**	32768	**Owner**	CKP	**Points Count**	32768	**Pulse Sequence**	single_pulse_dec
**Receiver Gain**	58.00	**Solvent**	CHLOROFORM-d			**Spectrum Offset (Hz)**	15079.3525
**Sweep Width (Hz)**	47348.49	**Temperature (degree C)**	29.000				

7l

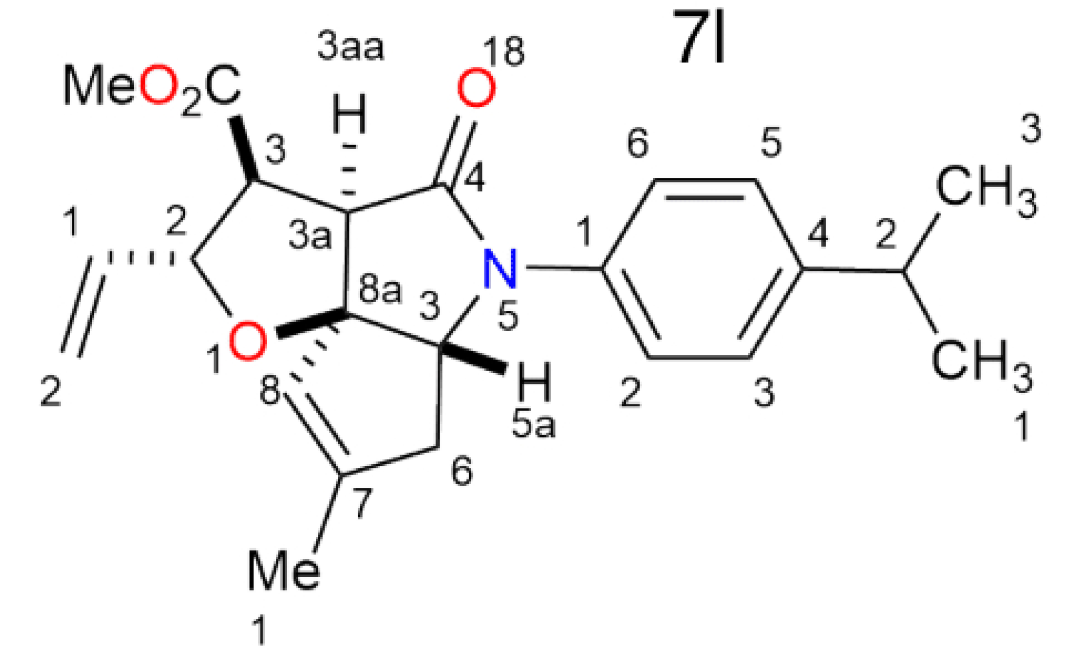

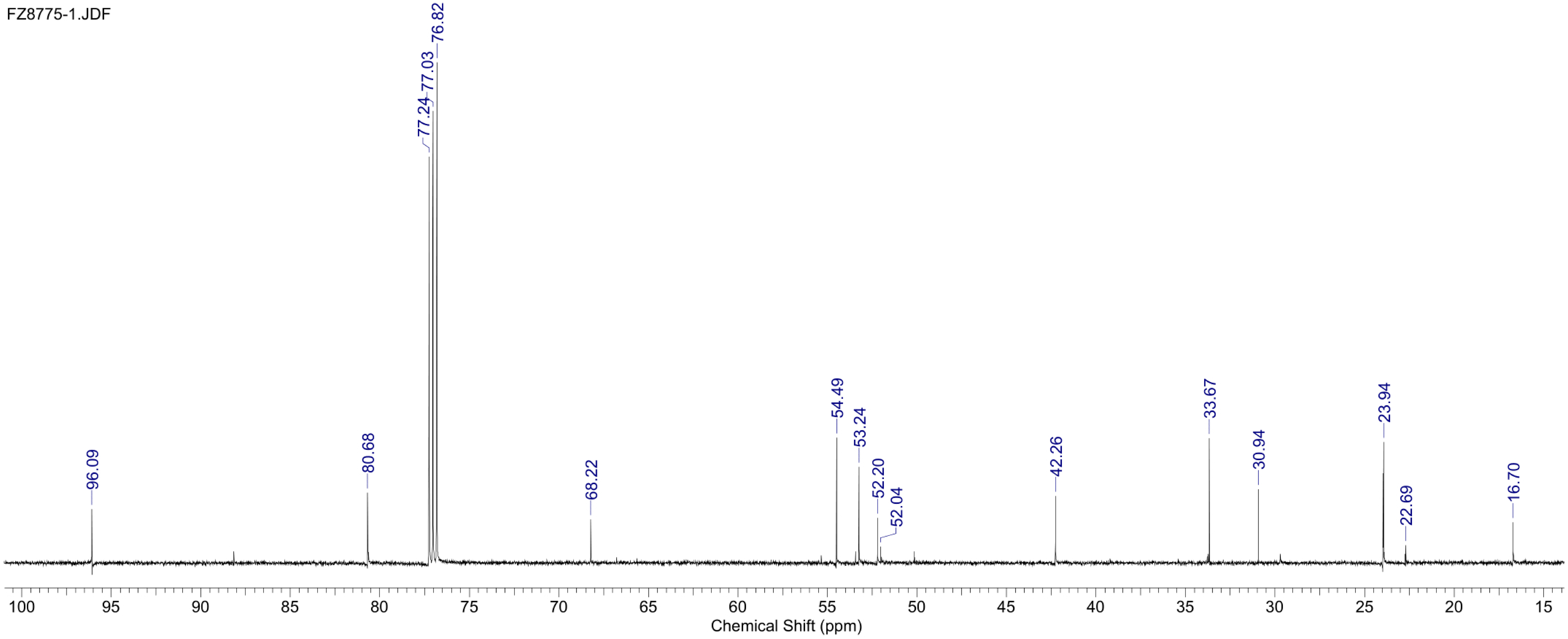

NMR spectrum data for compound 7m

**Formula** $C_{21}H_{23}NO_4$	**FW** 353.4116

Acquisition Time (sec)	1.9818	***Comment***	single_pulse	***Date***	07 Jul 2020 12:24:06		
Date Stamp	07 Jul 2020 12:25:38			***File Name***	C:\USERS\лаба 534\DESKTOP\Саша\ЯМР\NMR FIDS\FZ8748-1.JDF		
Frequency (MHz)	600.17	***Nucleus***	1H	***Number of Transients***	8	***Origin***	ECA 600
Original Points Count	32768	***Owner***	CKP	***Points Count***	32768	***Pulse Sequence***	single_pulse.ex2
Receiver Gain	40.00	***Solvent***	CHLOROFORM-d			***Spectrum Offset (Hz)***	5412.1411
Sweep Width (Hz)	16534.39	***Temperature (degree C)***	23.100				

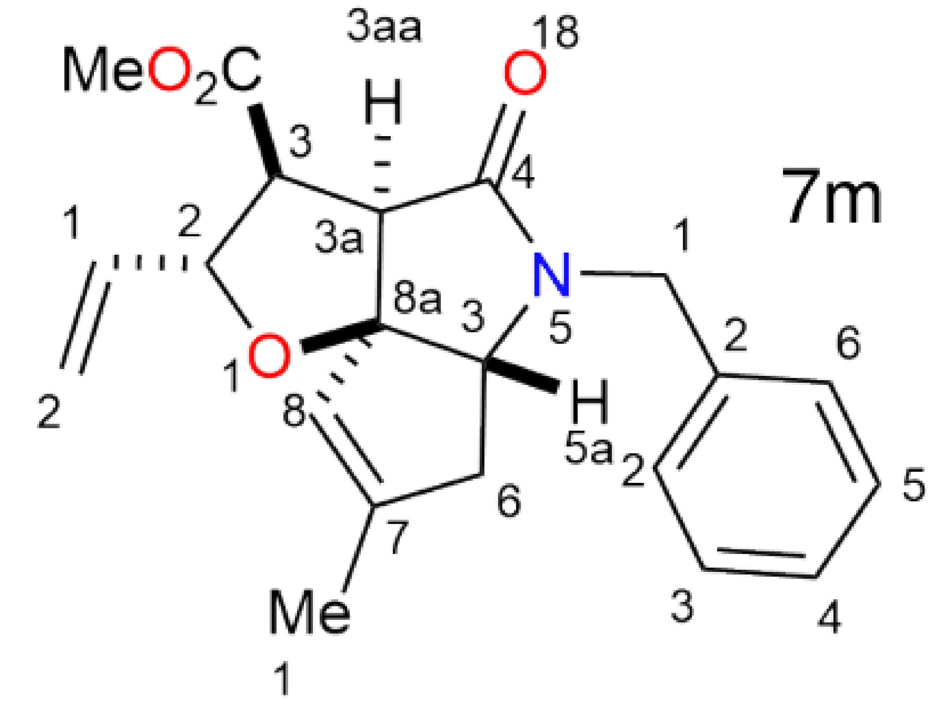

7m

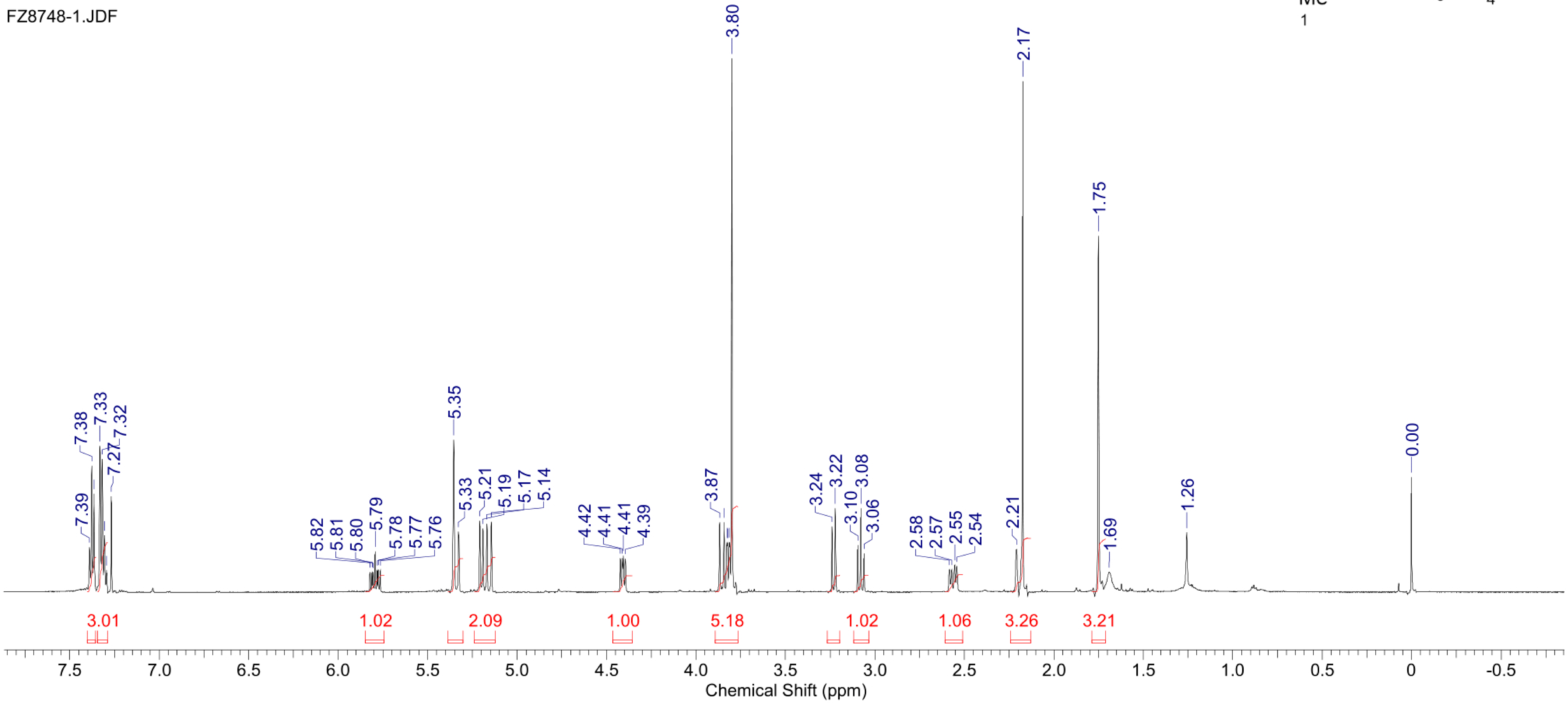

**Formula** $C_{21}H_{23}NO_4$	**FW** 353.4116

**Acquisition Time (sec)**	1.9818	**Comment**	single_pulse	**Date**	07 Jul 2020 12:24:06		
**Date Stamp**	07 Jul 2020 12:25:38			**File Name**	C:\USERS\лаба 534\DESKTOP\Саша\ЯМР\NMR FIDS\FZ8748-1.JDF		
**Frequency (MHz)**	600.17	**Nucleus**	1H	**Number of Transients**	8	**Origin**	ECA 600
**Original Points Count**	32768	**Owner**	CKP	**Points Count**	32768	**Pulse Sequence**	single_pulse.ex2
**Receiver Gain**	40.00	**Solvent**	CHLOROFORM-d			**Spectrum Offset (Hz)**	5412.1411
**Sweep Width (Hz)**	16534.39	**Temperature (degree C)**	23.100				

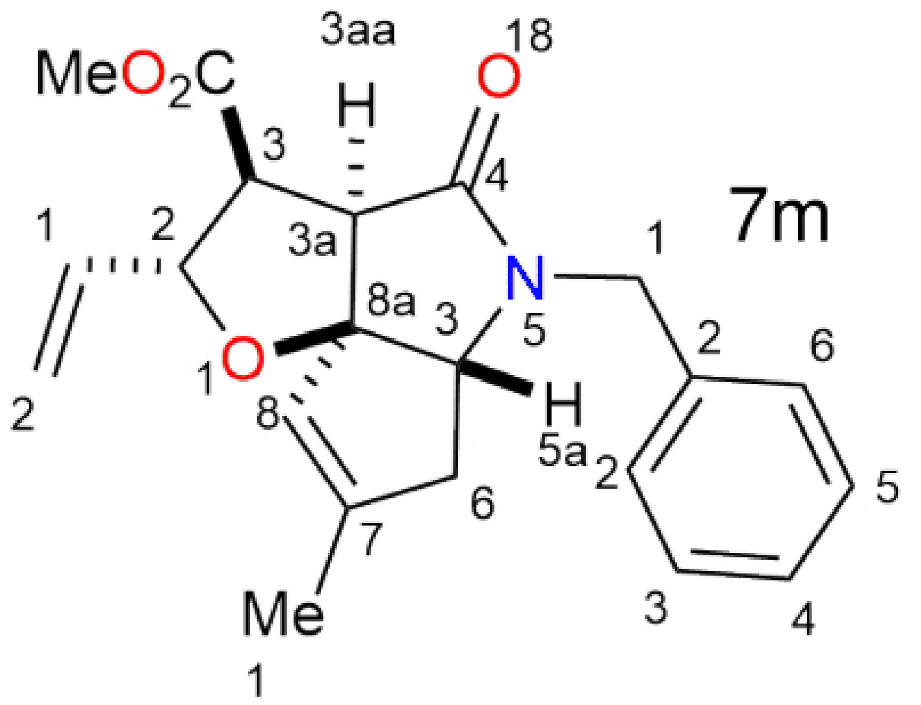

7m

FZ8748-1.JDF

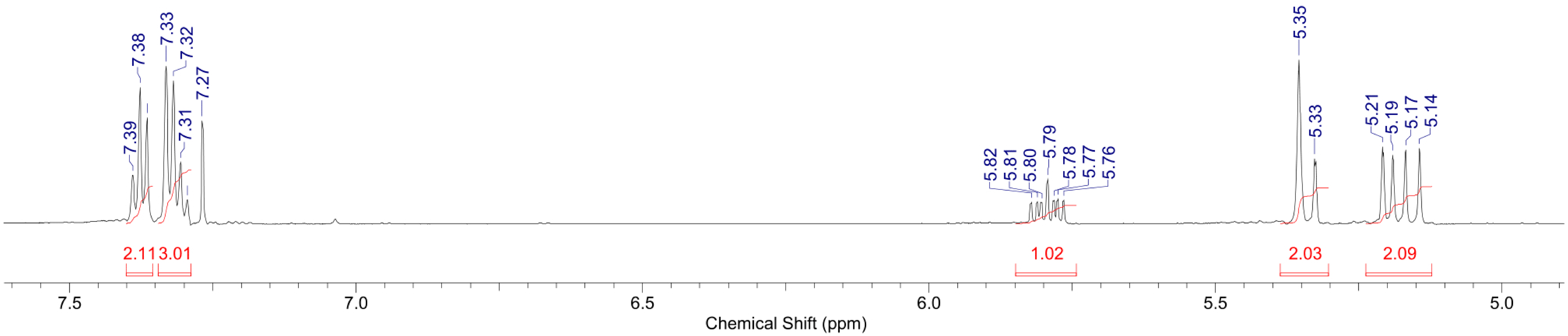

**Formula** $C_{21}H_{23}NO_4$	**FW** 353.4116

**Acquisition Time (sec)**	1.9818	**Comment**	single_pulse	**Date**	07 Jul 2020 12:24:06		
**Date Stamp**	07 Jul 2020 12:25:38			**File Name**	C:\USERS\лаба 534\DESKTOP\Саша\ЯМР\NMR FIDS\FZ8748-1.JDF		
**Frequency (MHz)**	600.17	**Nucleus**	1H	**Number of Transients**	8	**Origin**	ECA 600
**Original Points Count**	32768	**Owner**	CKP	**Points Count**	32768	**Pulse Sequence**	single_pulse.ex2
**Receiver Gain**	40.00	**Solvent**	CHLOROFORM-d			**Spectrum Offset (Hz)**	5412.1411
**Sweep Width (Hz)**	16534.39	**Temperature (degree C)**	23.100				

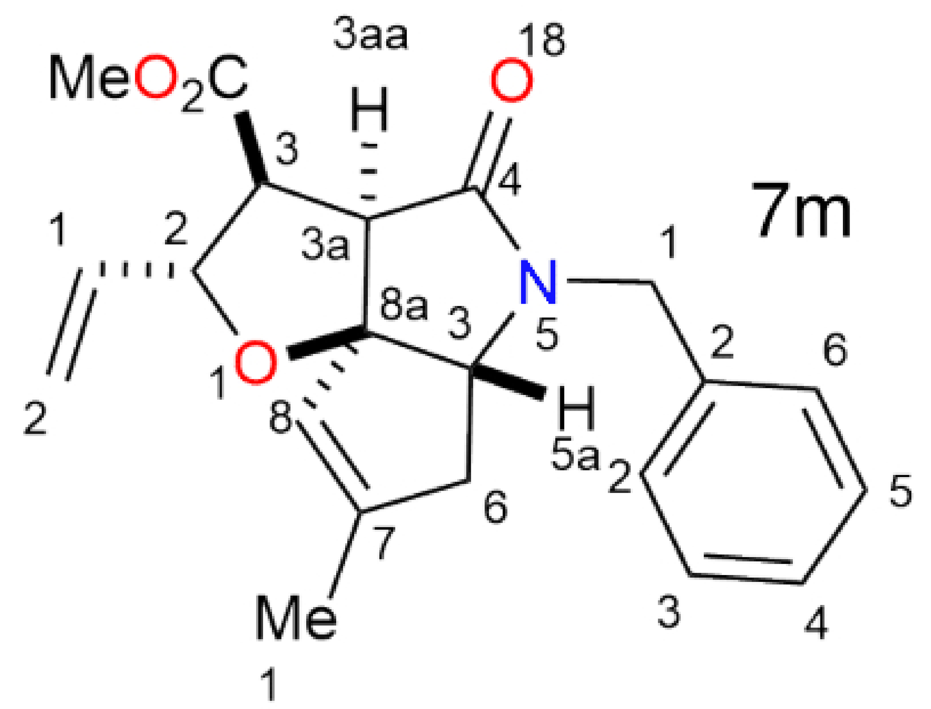

7m

FZ8748-1.JDF

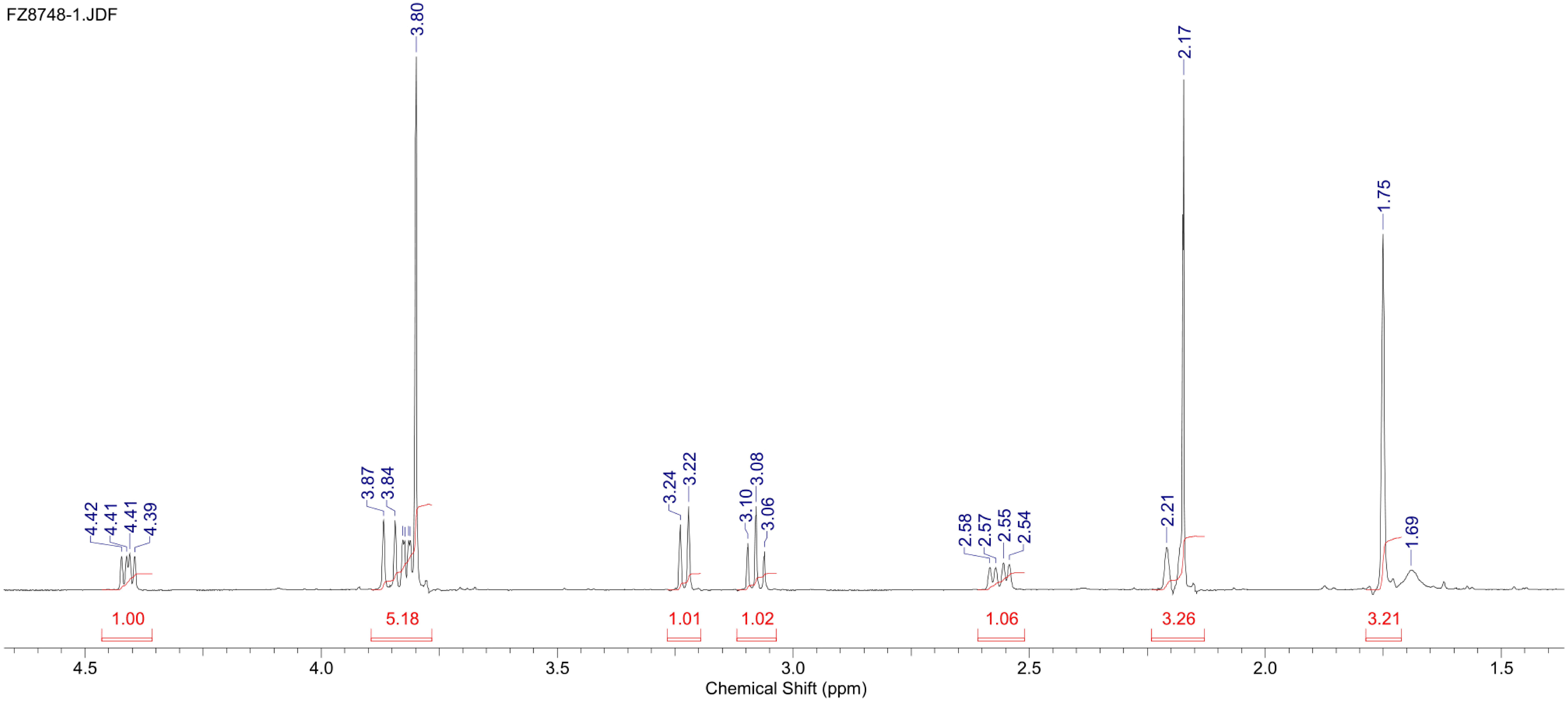

13.07.2020 15:01:09

**Formula** $C_{21}H_{23}NO_4$	**FW** 353.4116

**Acquisition Time (sec)**	0.6921	**Comment**	single pulse decoupled gated NOE			**Date**	11 Jul 2020 08:32:09
**Date Stamp**	11 Jul 2020 08:33:47			**File Name**	C:\USERS\Ла6а534\DOWNLOADS\FZ8776-1.JDF		
**Frequency (MHz)**	150.91	**Nucleus**	13C	**Number of Transients**	2000	**Origin**	ECA 600
**Original Points Count**	32768	**Owner**	CKP	**Points Count**	32768	**Pulse Sequence**	single_pulse_dec
**Receiver Gain**	58.00	**Solvent**	CHLOROFORM-d			**Spectrum Offset (Hz)**	15079.3525
**Sweep Width (Hz)**	47348.49	**Temperature (degree C)**	29.000				

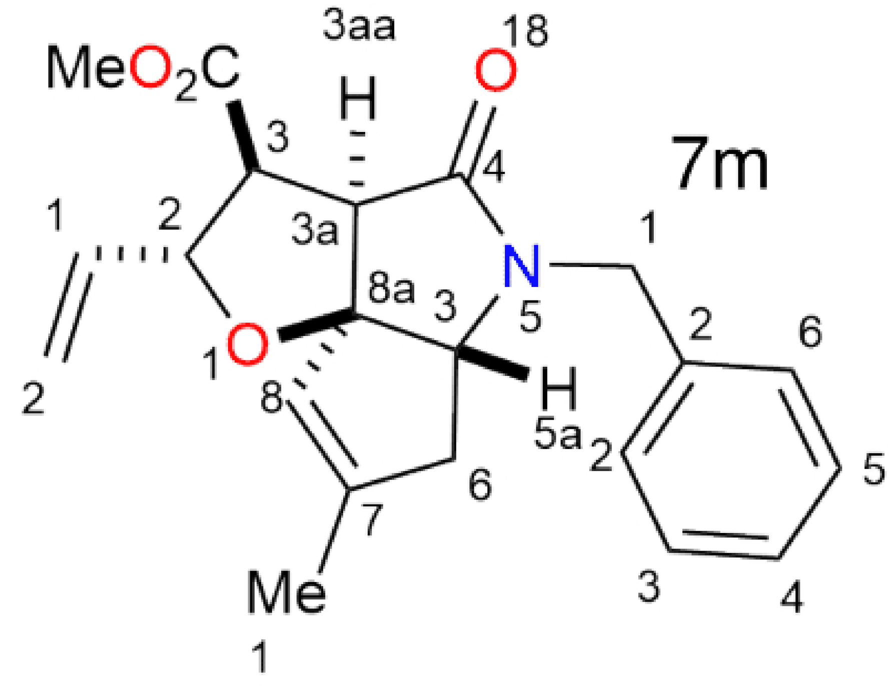

FZ8776-1.JDF

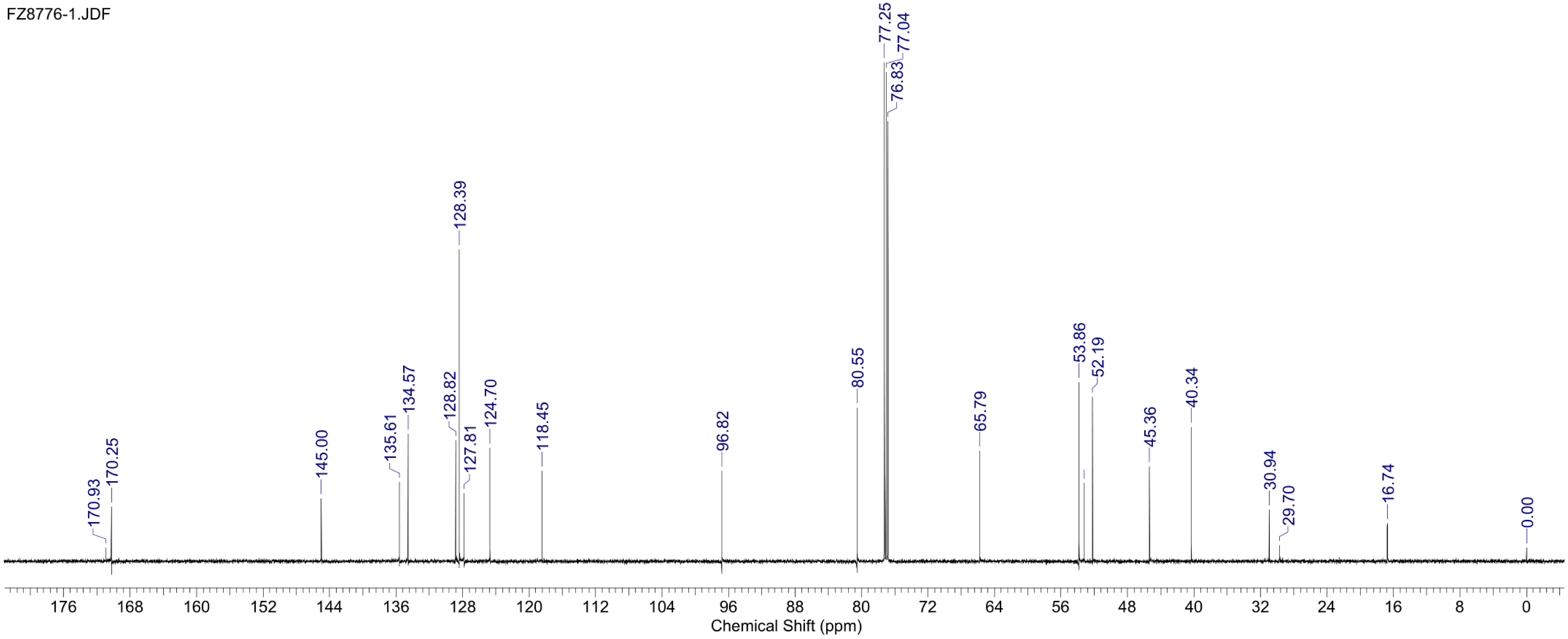

13.07.2020 15:01:46

Formula $C_{21}H_{23}NO_4$	*FW* 353.4116

Acquisition Time (sec)	0.6921	***Comment***	single pulse decoupled gated NOE			***Date***	11 Jul 2020 08:32:09
Date Stamp	11 Jul 2020 08:33:47			***File Name***	C:\USERS\Лаба534\DOWNLOADS\FZ8776-1.JDF		
Frequency (MHz)	150.91	***Nucleus***	13C	***Number of Transients***	2000	***Origin***	ECA 600
Original Points Count	32768	***Owner***	CKP	***Points Count***	32768	***Pulse Sequence***	single_pulse_dec
Receiver Gain	58.00	***Solvent***	CHLOROFORM-d			***Spectrum Offset (Hz)***	15079.3525
Sweep Width (Hz)	47348.49	***Temperature (degree C)***	29.000				

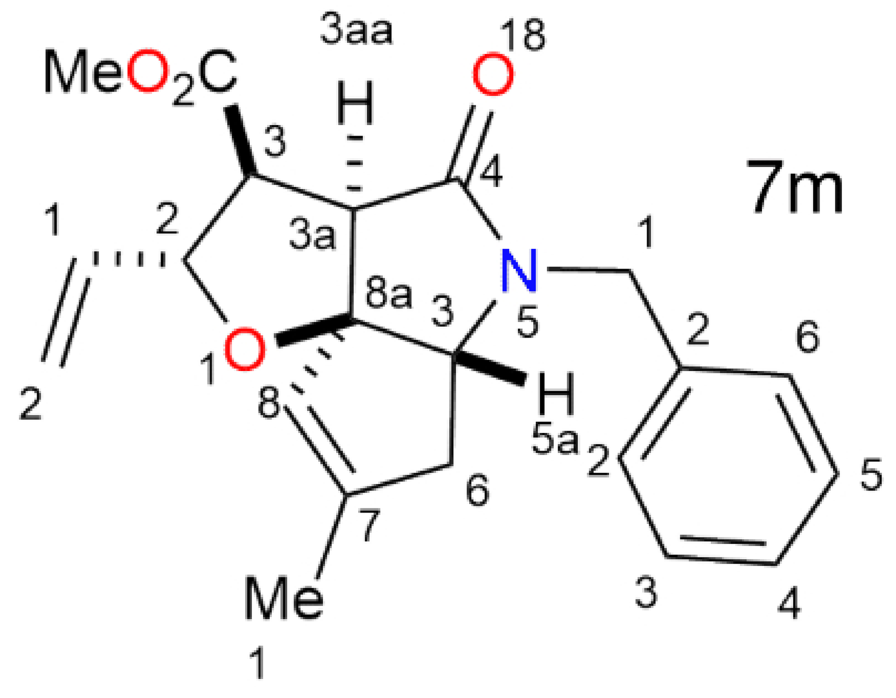

7m

FZ8776-1.JDF

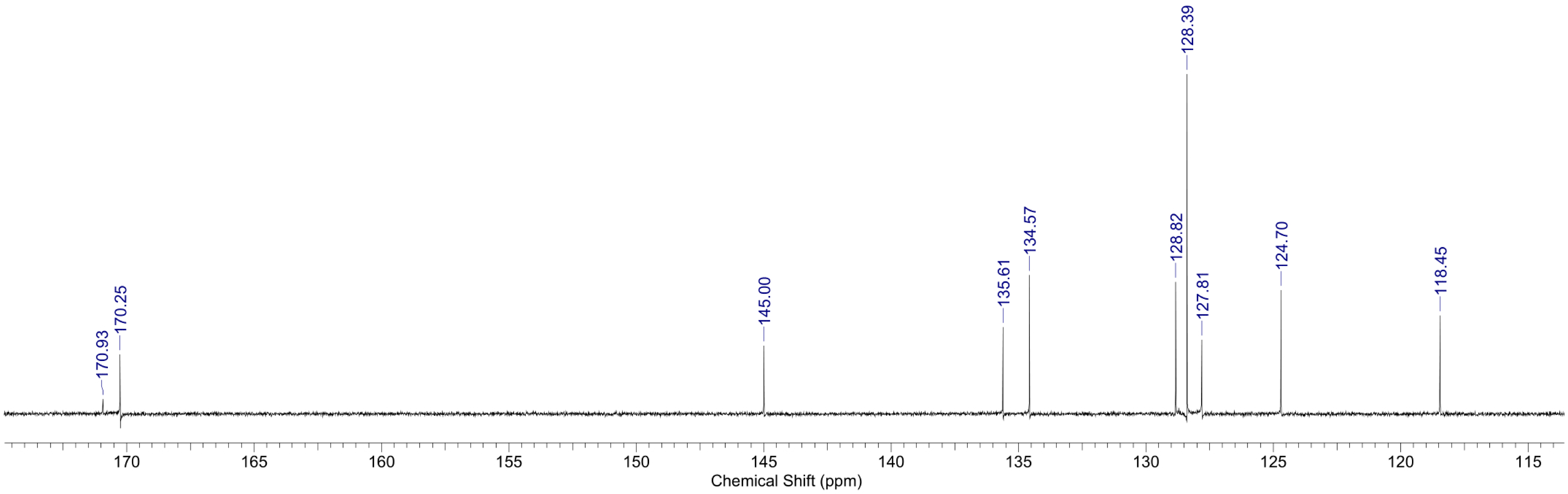

13.07.2020 15:02:00

**Formula** $C_{21}H_{23}NO_4$	**FW** 353.4116

Acquisition Time (sec)	0.6921	***Comment***	single pulse decoupled gated NOE			***Date***	11 Jul 2020 08:32:09
Date Stamp	11 Jul 2020 08:33:47			***File Name***	C:\USERS\Ла6а534\DOWNLOADS\FZ8776-1.JDF		
Frequency (MHz)	150.91	***Nucleus***	13C	***Number of Transients***	2000	***Origin***	ECA 600
Original Points Count	32768	***Owner***	CKP	***Points Count***	32768	***Pulse Sequence***	single_pulse_dec
Receiver Gain	58.00	***Solvent***	CHLOROFORM-d			***Spectrum Offset (Hz)***	15079.3525
Sweep Width (Hz)	47348.49	***Temperature (degree C)***	29.000				

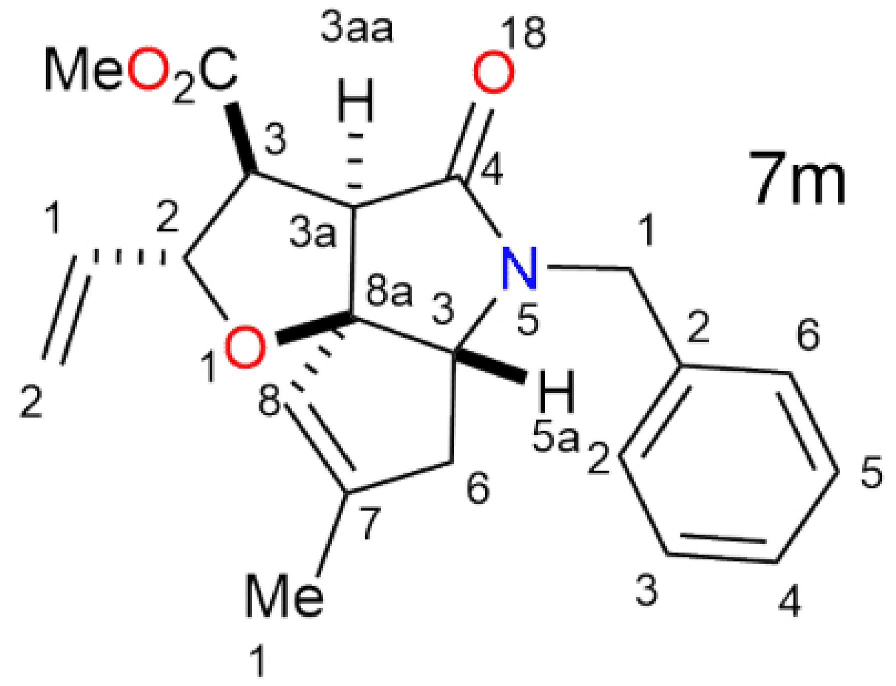

7m

FZ8776-1.JDF

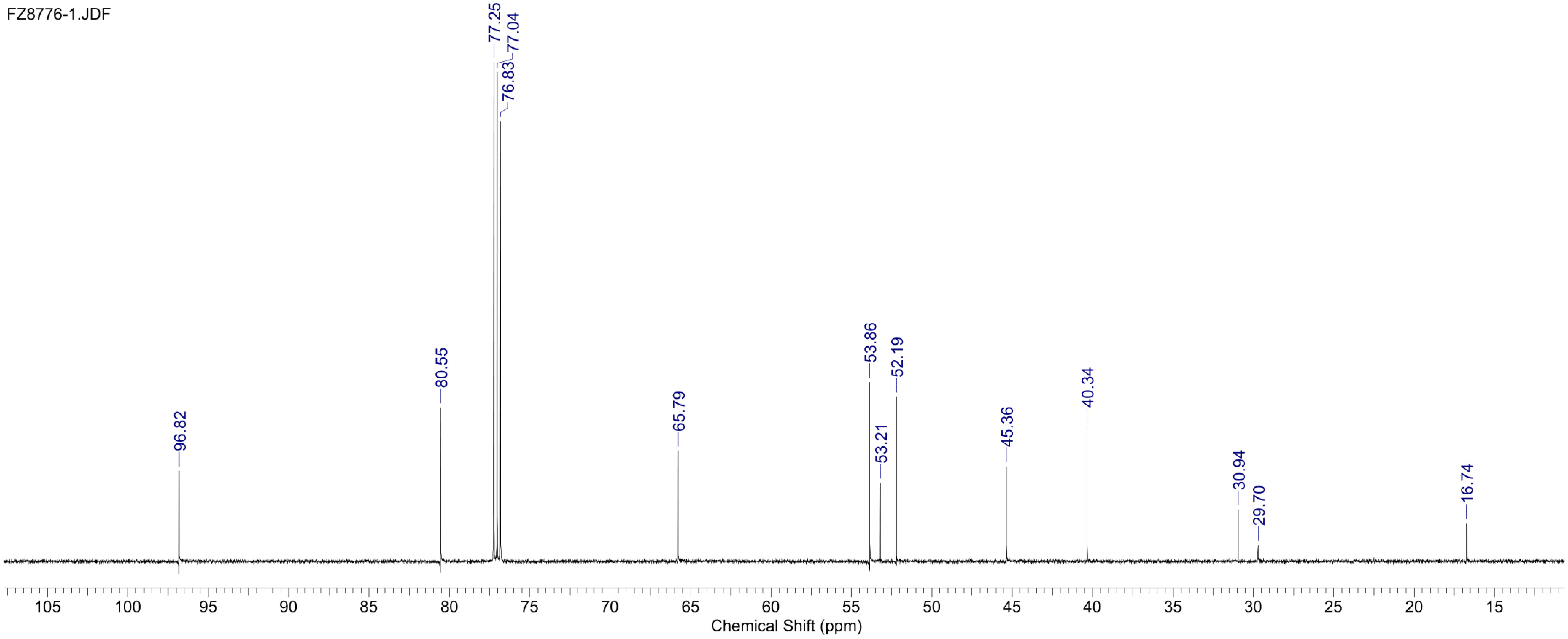

NMR spectrum data for compound 7n

Formula $C_{19}H_{18}ClNO_4$	FW 359.8035

Acquisition Time (sec)	1.9818	Comment	single_pulse	Date	12 Mar 2020 13:32:24		
Date Stamp	12 Mar 2020 12:50:27			File Name	C:\USERS\лаба 534\DESKTOP\Саша\ЯМР\NMR FIDS\FZ8593-1.JDF		
Frequency (MHz)	600.17	Nucleus	1H	Number of Transients	8	Origin	ECA 600
Original Points Count	32768	Owner	CKP	Points Count	32768	Pulse Sequence	single_pulse.ex2
Receiver Gain	46.00	Solvent	CHLOROFORM-d			Spectrum Offset (Hz)	5409.6182
Sweep Width (Hz)	16534.39	Temperature (degree C)	23.300				

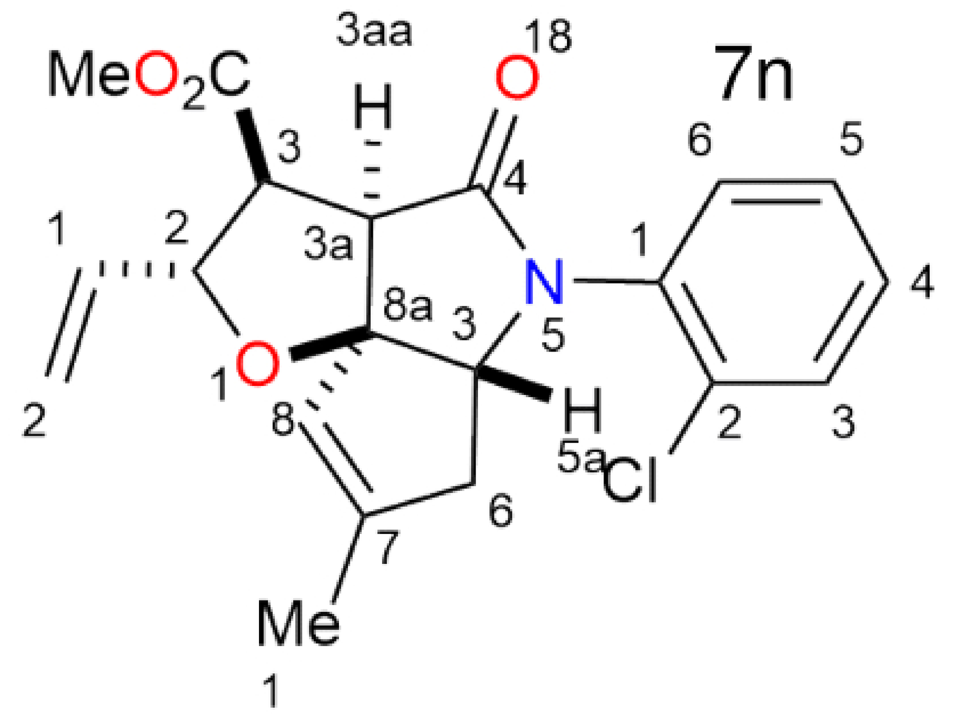

FZ8593-1.JDF

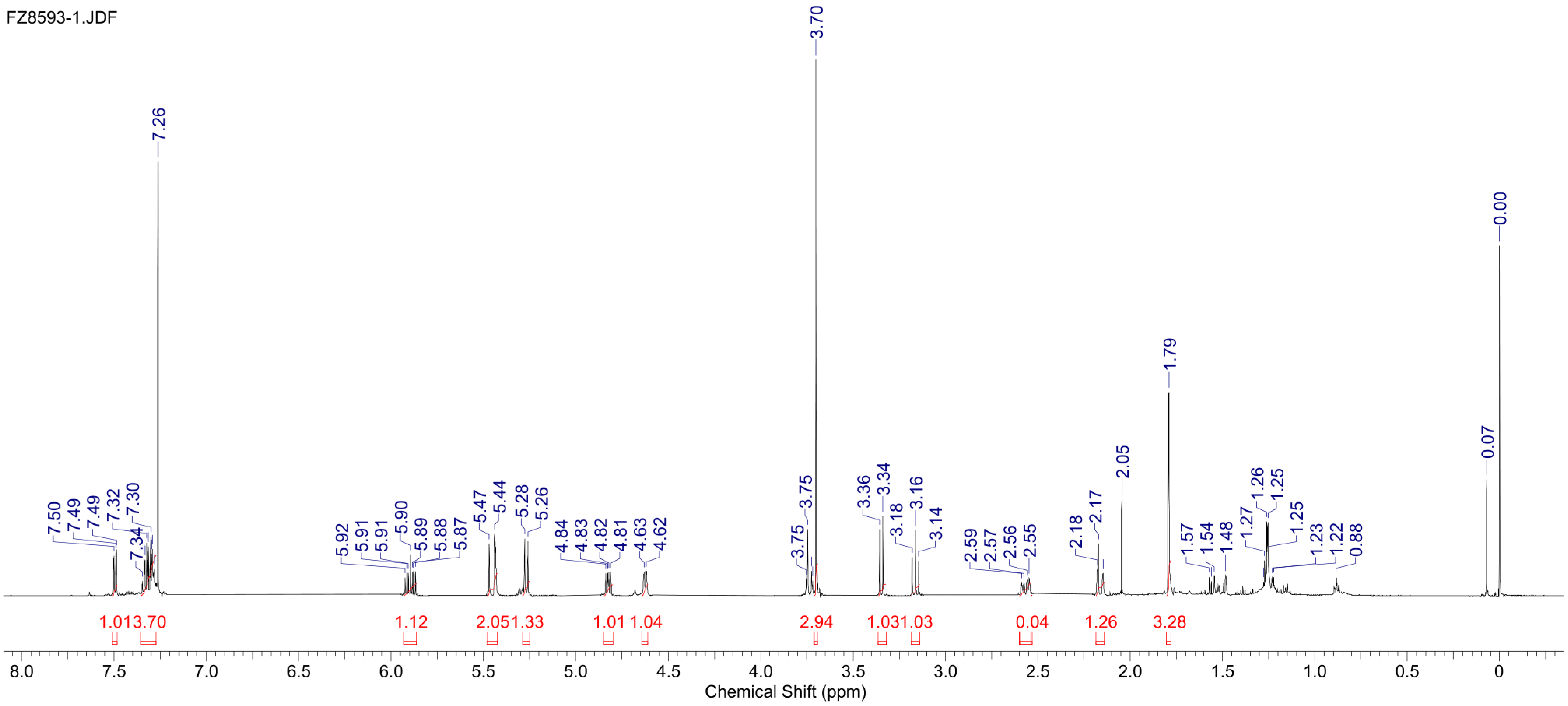

Formula	$C_{19}H_{18}ClNO_4$	FW	359.8035

Acquisition Time (sec)	1.9818	Comment	single_pulse	Date	12 Mar 2020 13:32:24		
Date Stamp	12 Mar 2020 12:50:27			File Name	C:\USERS\лаба 534\DESKTOP\Саша\ЯМР\NMR FIDS\FZ8593-1.JDF		
Frequency (MHz)	600.17	Nucleus	1H	Number of Transients	8	Origin	ECA 600
Original Points Count	32768	Owner	CKP	Points Count	32768	Pulse Sequence	single_pulse.ex2
Receiver Gain	46.00	Solvent	CHLOROFORM-d			Spectrum Offset (Hz)	5409.6182
Sweep Width (Hz)	16534.39	Temperature (degree C)	23.300				

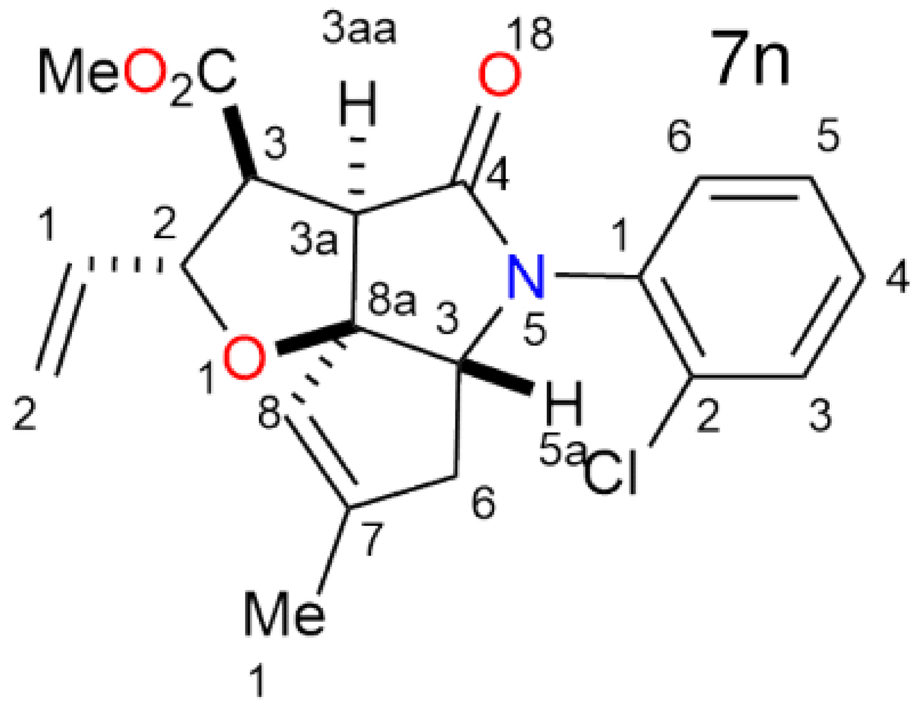

FZ8593-1.JDF

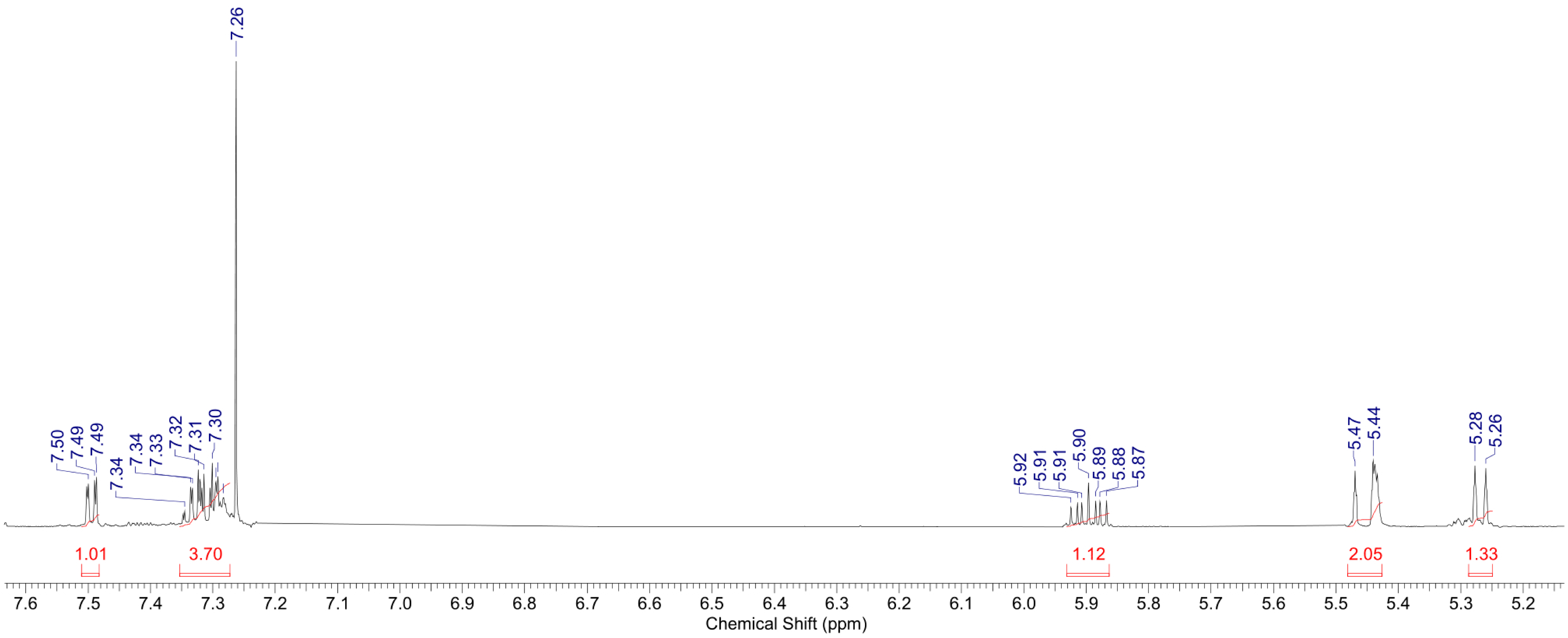

Formula $C_{19}H_{18}ClNO_4$	FW 359.8035

**Acquisition Time (sec)**	1.9818	**Comment**	single_pulse	**Date**	12 Mar 2020 13:32:24		
**Date Stamp**	12 Mar 2020 12:50:27			**File Name**	C:\USERS\лаба 534\DESKTOP\Саша\ЯМР\NMR FIDS\FZ8593-1.JDF		
**Frequency (MHz)**	600.17	**Nucleus**	1H	**Number of Transients**	8	**Origin**	ECA 600
**Original Points Count**	32768	**Owner**	CKP	**Points Count**	32768	**Pulse Sequence**	single_pulse.ex2
**Receiver Gain**	46.00	**Solvent**	CHLOROFORM-d			**Spectrum Offset (Hz)**	5409.6182
**Sweep Width (Hz)**	16534.39	**Temperature (degree C)**	23.300				

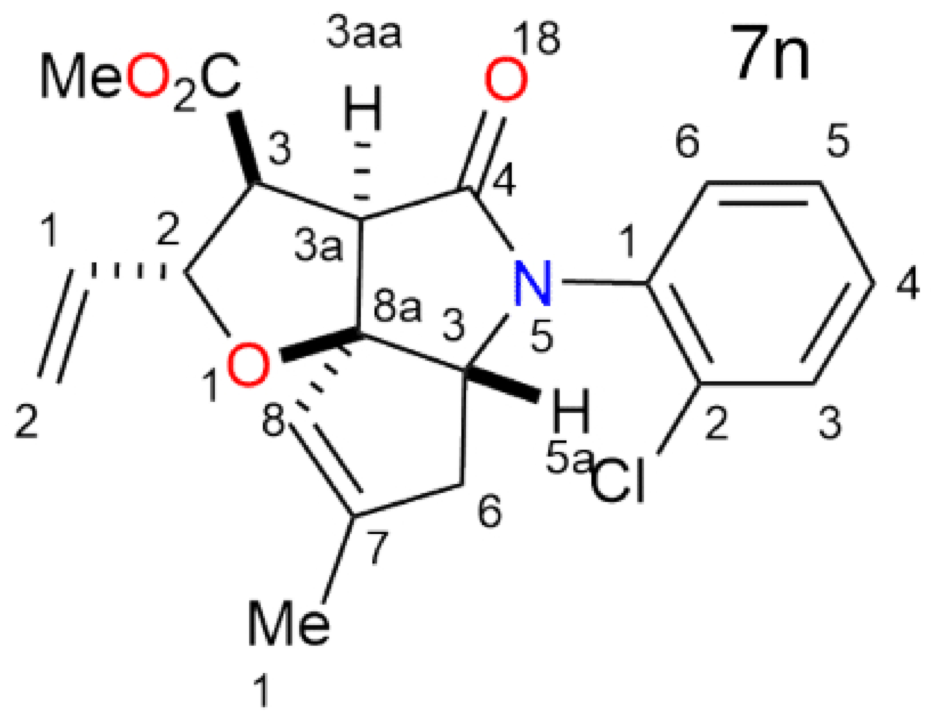

7n

FZ8593-1.JDF

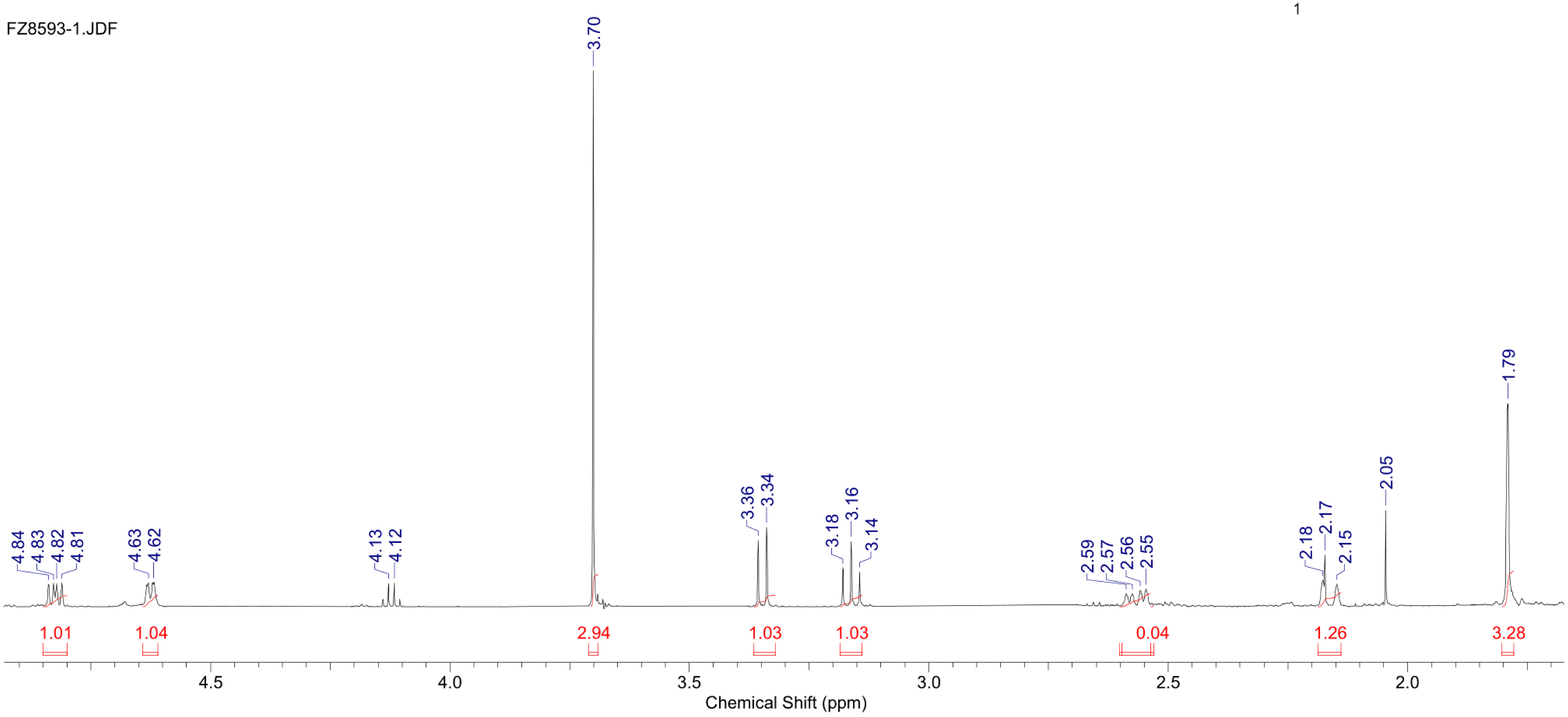

**Formula** $C_{20}H_{20}ClNO_4$	**FW** 373.8301

Acquisition Time (sec)	0.6921	***Comment***	single pulse decoupled gated NOE	***Date***	24 Jun 2020 06:23:34
Date Stamp	24 Jun 2020 06:24:55	***File Name***	C:\USERS\лаба 534\DESKTOP\Саша\ЯМР\NMR FIDS\FZ8696-1.JDF		
Frequency (MHz)	150.91	***Nucleus***	13C	***Number of Transients***	4000
Origin	ECA 600				
Original Points Count	32768	***Owner***	CKP	***Points Count***	32768
Pulse Sequence	single_pulse_dec				
Receiver Gain	54.00	***Solvent***	CHLOROFORM-d	***Spectrum Offset (Hz)***	15079.3525
Sweep Width (Hz)	47348.49	***Temperature (degree C)***	22.100		

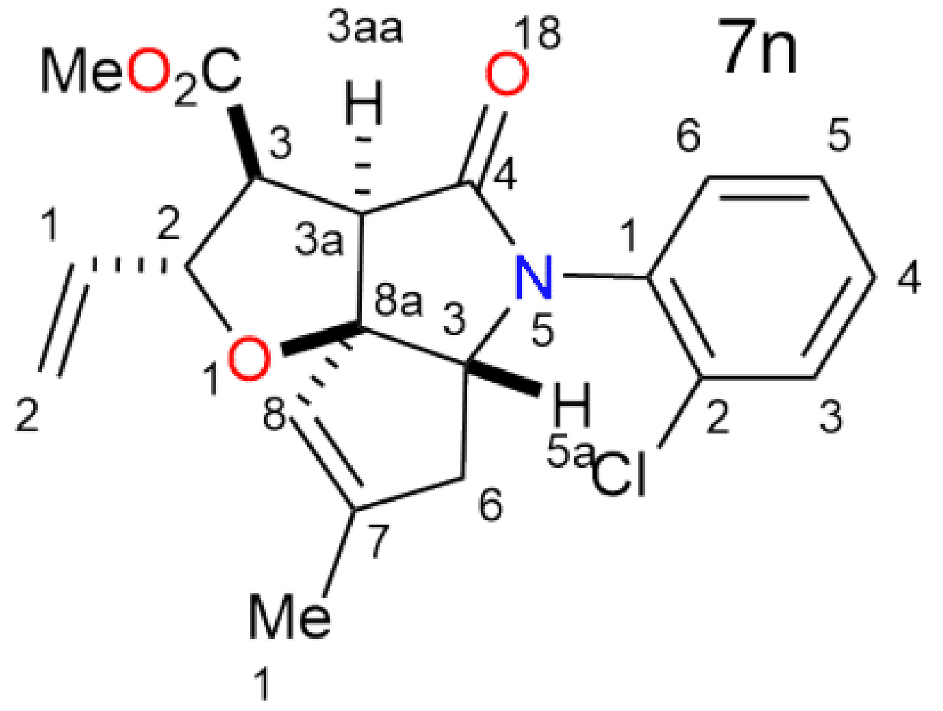

7n

FZ8696-1.JDF

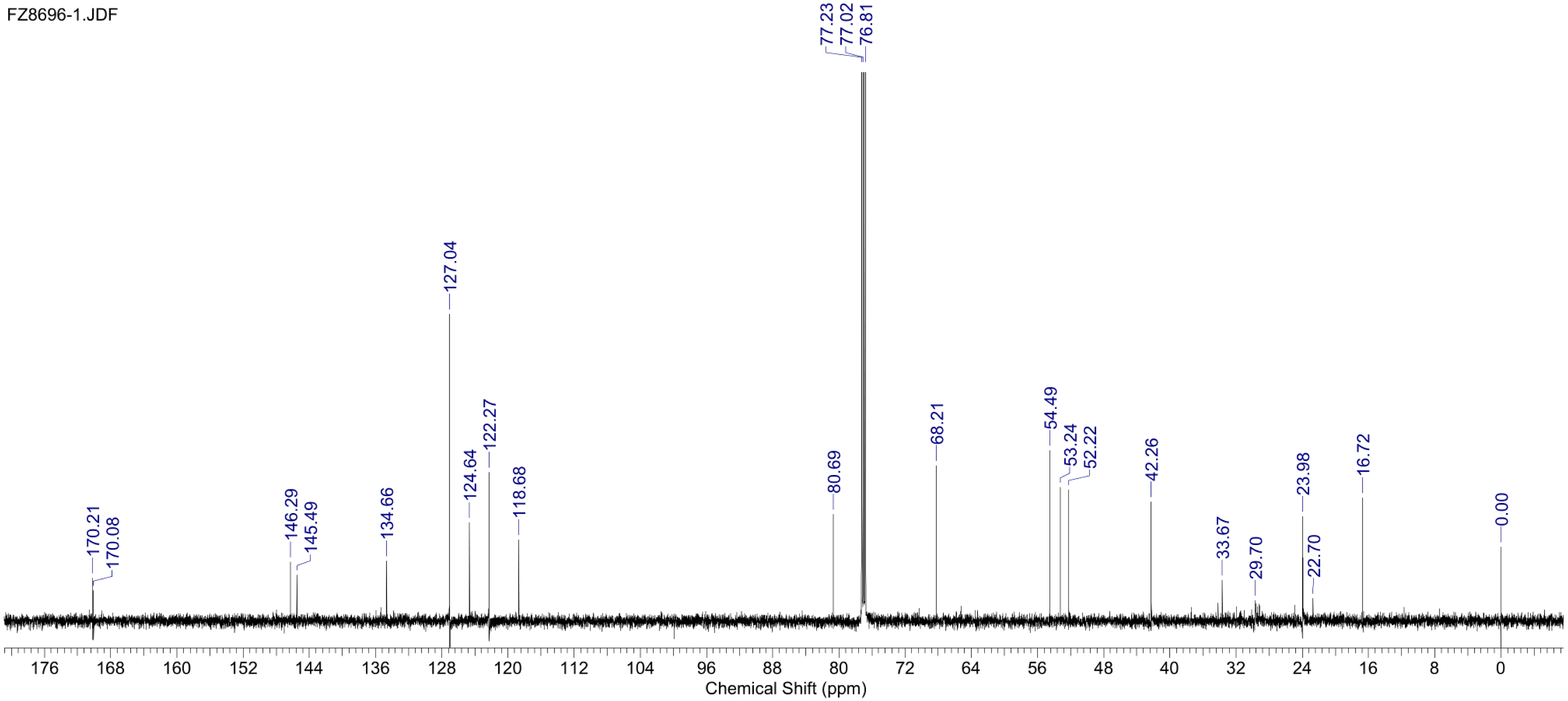

Formula $C_{20}H_{20}ClNO_4$	FW 373.8301

Acquisition Time (sec)	0.6921	***Comment***	single pulse decoupled gated NOE			***Date***	24 Jun 2020 06:23:34
Date Stamp	24 Jun 2020 06:24:55			***File Name***	C:\USERS\лаба 534\DESKTOP\Саша\ЯМР\NMR FIDS\FZ8696-1.JDF		
Frequency (MHz)	150.91	***Nucleus***	13C	***Number of Transients***	4000	***Origin***	ECA 600
Original Points Count	32768	***Owner***	CKP	***Points Count***	32768	***Pulse Sequence***	single_pulse_dec
Receiver Gain	54.00	***Solvent***	CHLOROFORM-d			***Spectrum Offset (Hz)***	15079.3525
Sweep Width (Hz)	47348.49	***Temperature (degree C)***	22.100				

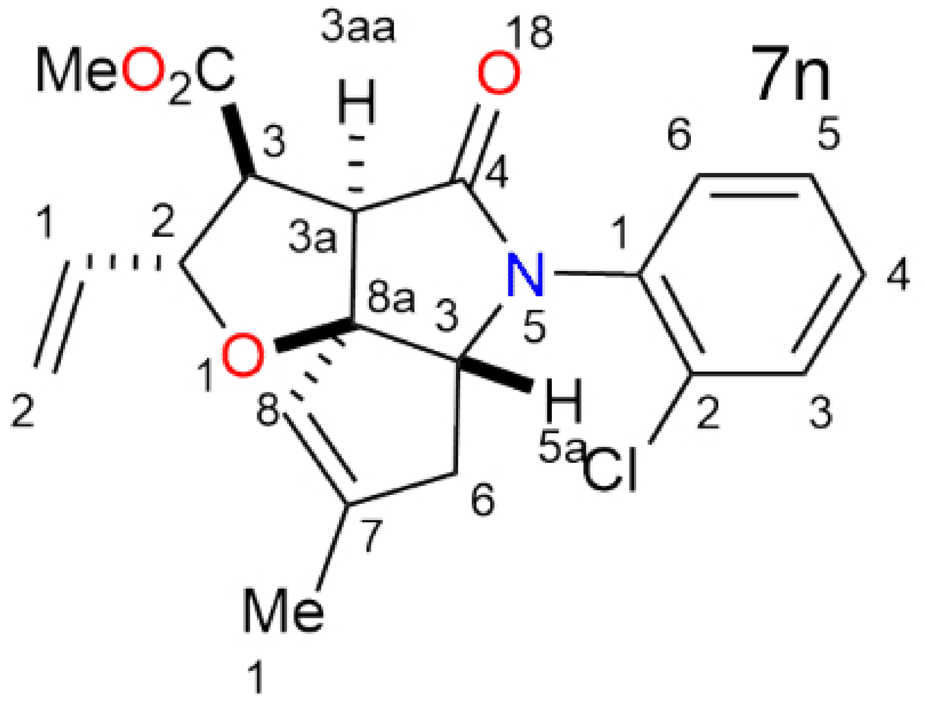

7n

FZ8696-1.JDF

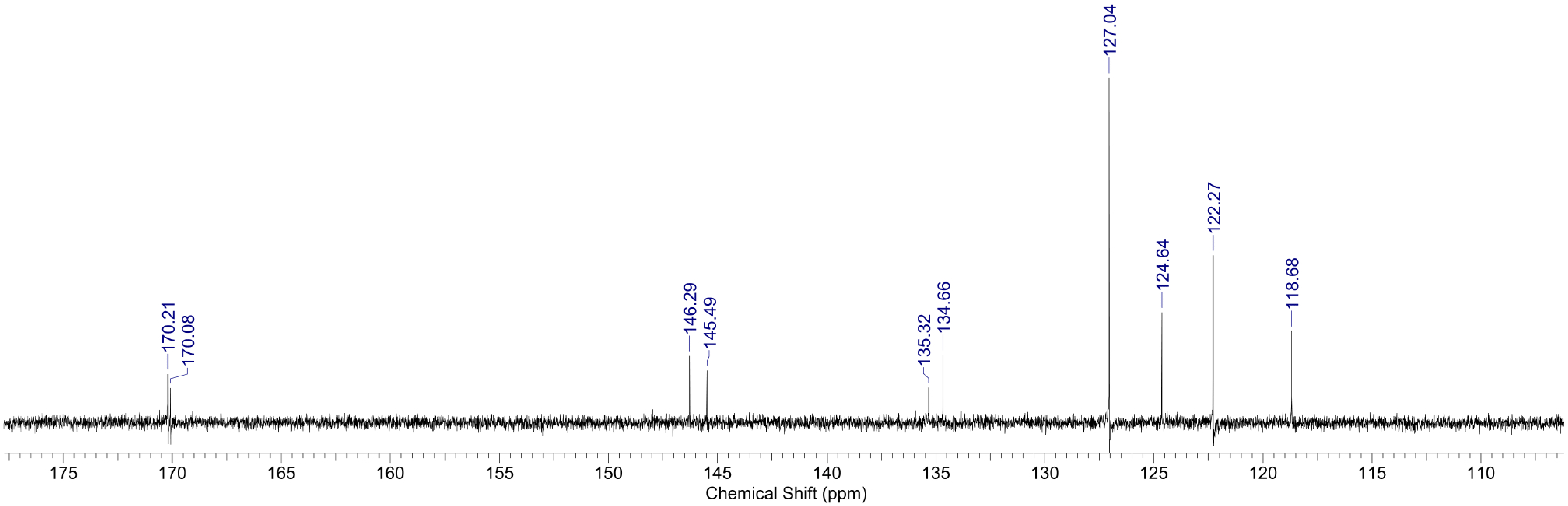

**Formula** $C_{20}H_{20}ClNO_4$	**FW** 373.8301

Acquisition Time (sec)	0.6921	***Comment***	single pulse decoupled gated NOE			***Date***	24 Jun 2020 06:23:34
Date Stamp	24 Jun 2020 06:24:55			***File Name***	C:\USERS\лаба 534\DESKTOP\Саша\ЯМР\NMR FIDS\FZ8696-1.JDF		
Frequency (MHz)	150.91	***Nucleus***	13C	***Number of Transients***	4000	***Origin***	ECA 600
Original Points Count	32768	***Owner***	CKP	***Points Count***	32768	***Pulse Sequence***	single_pulse_dec
Receiver Gain	54.00	***Solvent***	CHLOROFORM-d			***Spectrum Offset (Hz)***	15079.3525
Sweep Width (Hz)	47348.49	***Temperature (degree C)***	22.100				

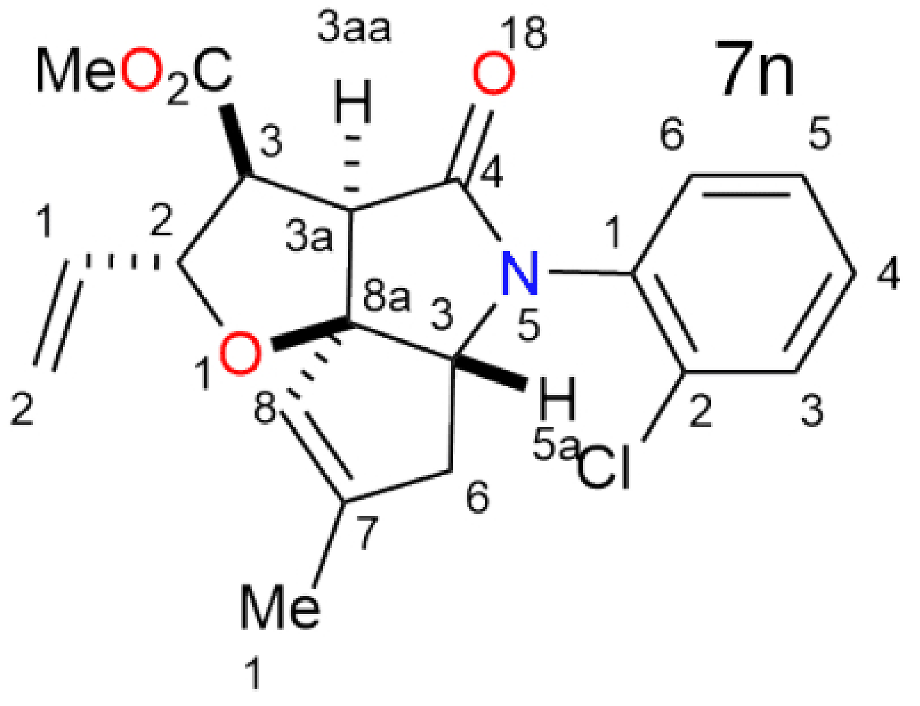

7n

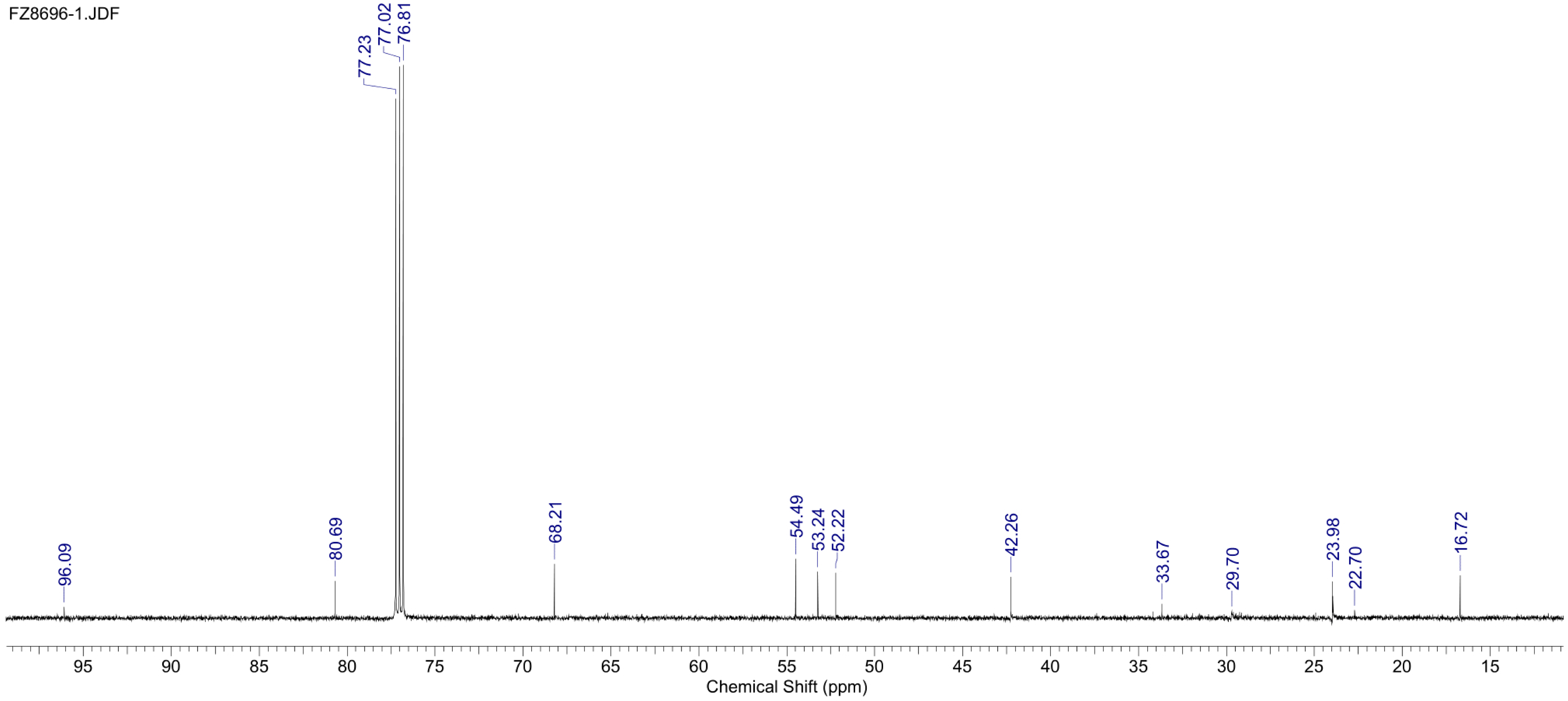

NMR spectrum data for compound 7o

15.07.2020 20:45:21

**Formula** $C_{20}H_{20}ClNO_4$	**FW** 373.8301

**Acquisition Time (sec)**	0.9909	**Comment**	single_pulse	**Date**	27 Feb 2020 12:44:31		
**Date Stamp**	27 Feb 2020 12:04:13			**File Name**	C:\USERS\Лаба534\DOWNLOADS\FZ8536-1.JDF	**Frequency (MHz)**	600.17
**Nucleus**	1H	**Number of Transients**	8	**Origin**	ECA 600	**Original Points Count** 16384	**Owner** CKP
**Points Count**	16384	**Pulse Sequence**	single_pulse.ex2			**Receiver Gain** 48.00	**Solvent** CHLOROFORM-d
**Spectrum Offset (Hz)**	5409.0264	**Sweep Width (Hz)**	16534.39	**Temperature (degree C)**	23.200		

7o

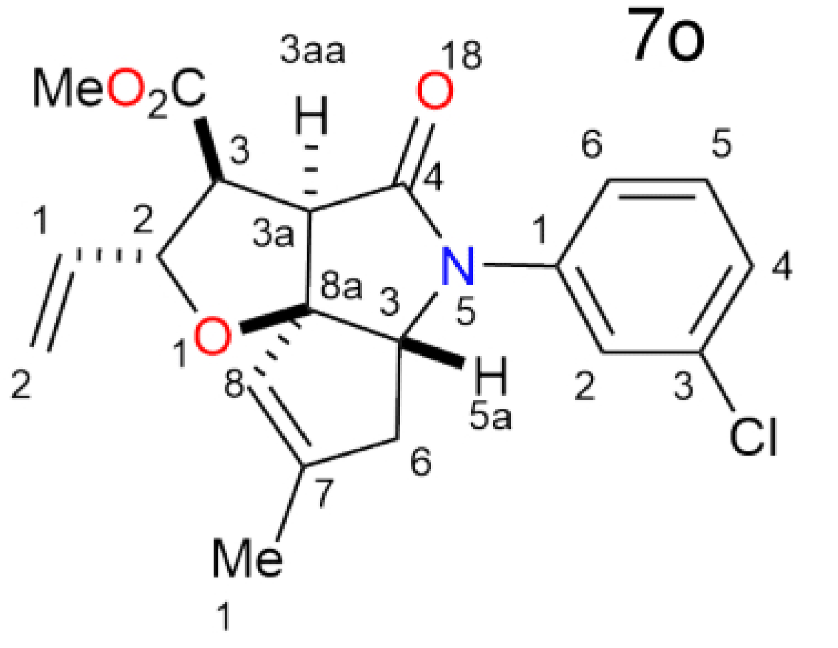

FZ8536-1.JDF

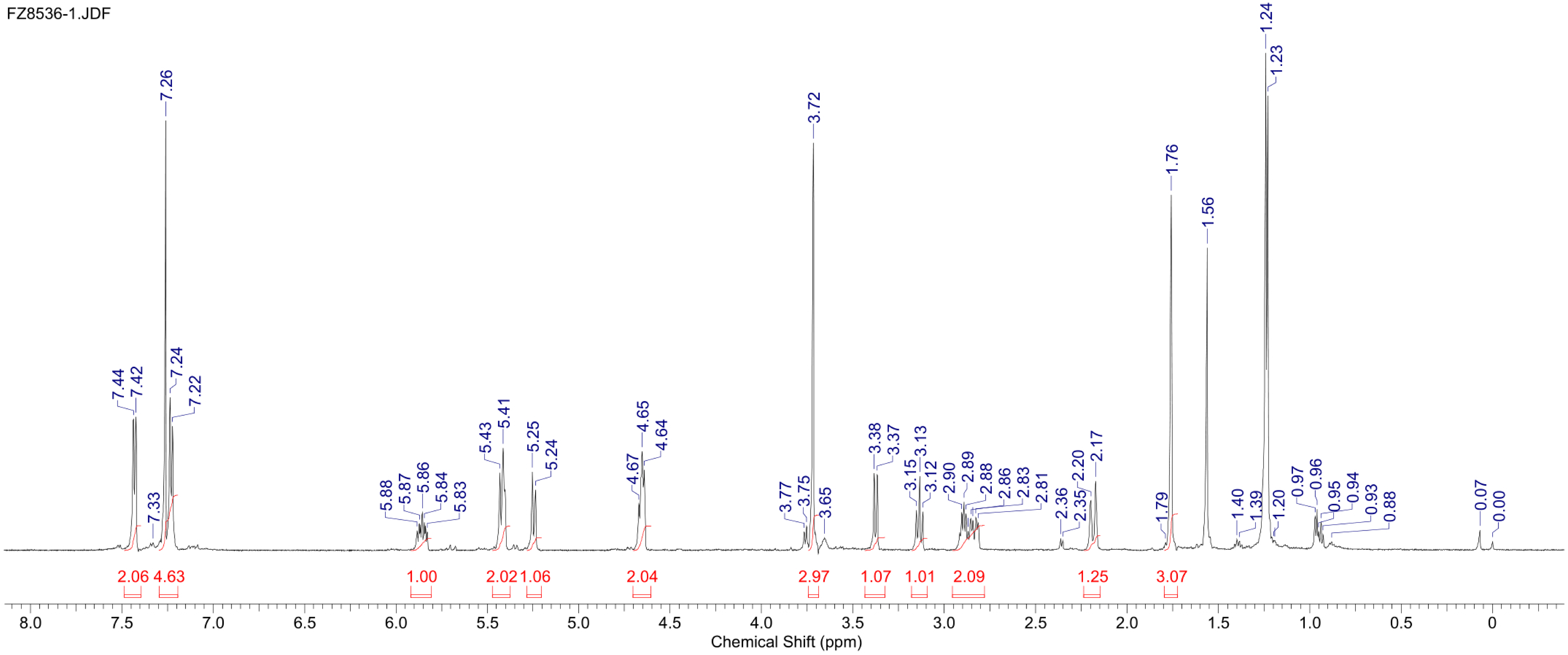

15.07.2020 20:46:15

Formula	$C_{20}H_{20}ClNO_4$	FW	373.8301

**Acquisition Time (sec)**	0.9909	**Comment**	single_pulse	**Date**	27 Feb 2020 12:44:31		
**Date Stamp**	27 Feb 2020 12:04:13			**File Name**	C:\USERS\Лаба534\DOWNLOADS\FZ8536-1.JDF	**Frequency (MHz)**	600.17
**Nucleus**	1H	**Number of Transients**	8	**Origin**	ECA 600	**Original Points Count**	16384
**Owner**	CKP						
**Points Count**	16384	**Pulse Sequence**	single_pulse.ex2	**Receiver Gain**	48.00	**Solvent**	CHLOROFORM-d
**Spectrum Offset (Hz)**	5409.0264	**Sweep Width (Hz)**	16534.39	**Temperature (degree C)**	23.200		

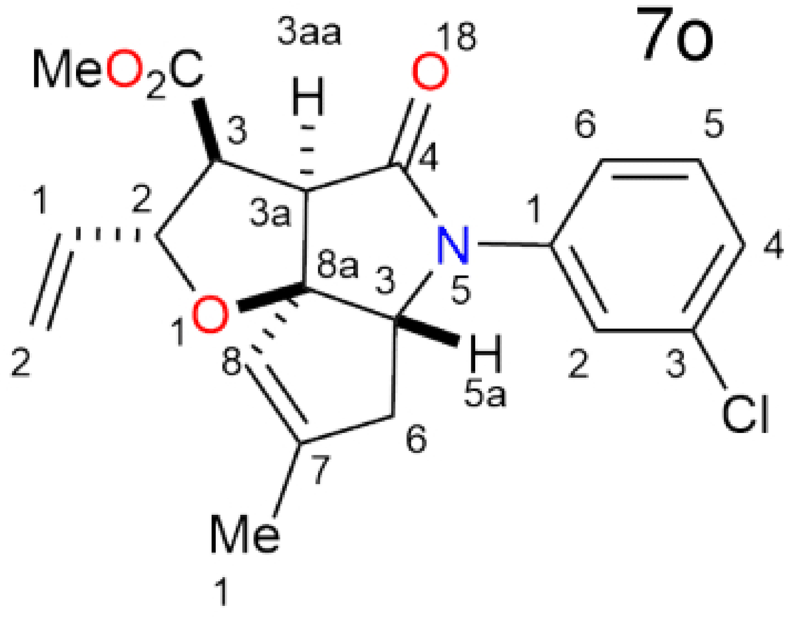

7o

FZ8536-1.JDF

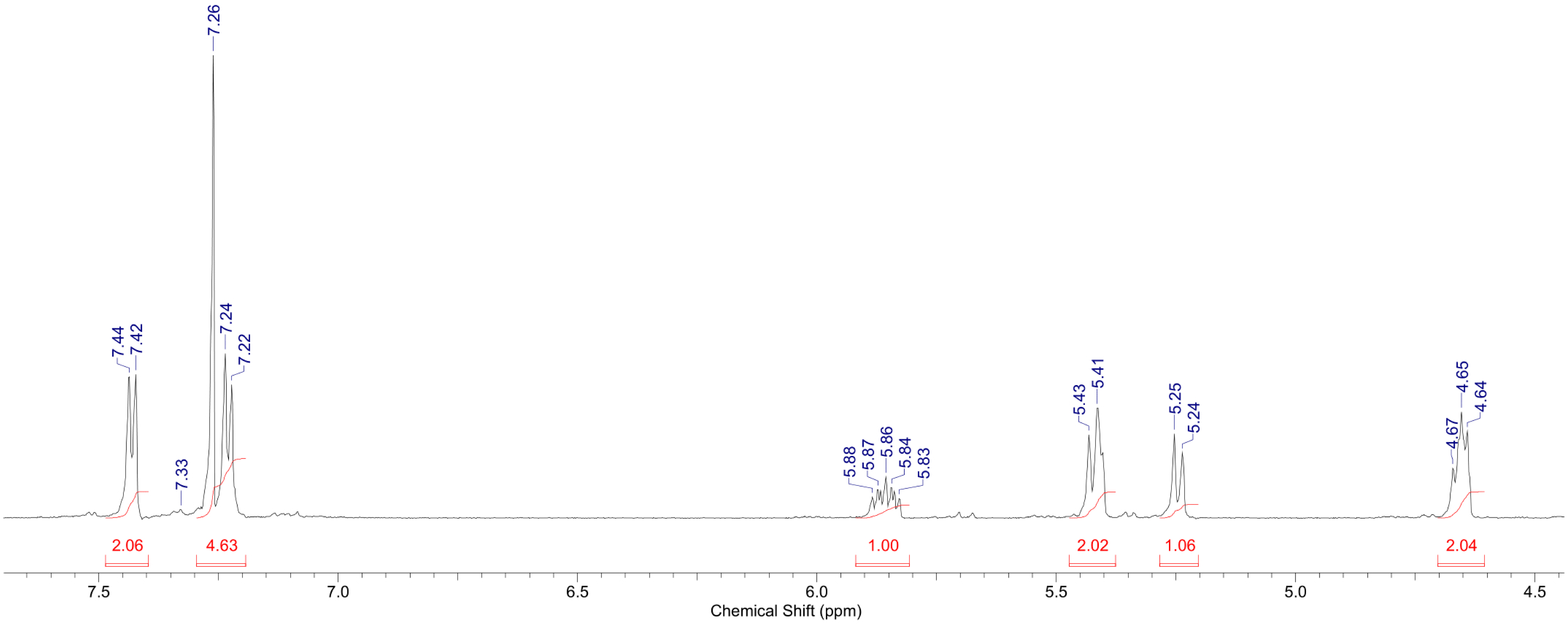

15.07.2020 20:46:25

Formula	$C_{20}H_{20}ClNO_4$	***FW***	373.8301

Acquisition Time (sec)	0.9909	***Comment***	single_pulse	***Date***	27 Feb 2020 12:44:31		
Date Stamp	27 Feb 2020 12:04:13			***File Name***	C:\USERS\Лаб534\DOWNLOADS\FZ8536-1.JDF	***Frequency (MHz)***	600.17
Nucleus	1H	***Number of Transients***	8	***Origin***	ECA 600	***Original Points Count*** 16384	***Owner*** CKP
Points Count	16384	***Pulse Sequence***	single_pulse.ex2			***Receiver Gain*** 48.00	***Solvent*** CHLOROFORM-d
Spectrum Offset (Hz)	5409.0264	***Sweep Width (Hz)***	16534.39	***Temperature (degree C)***	23.200		

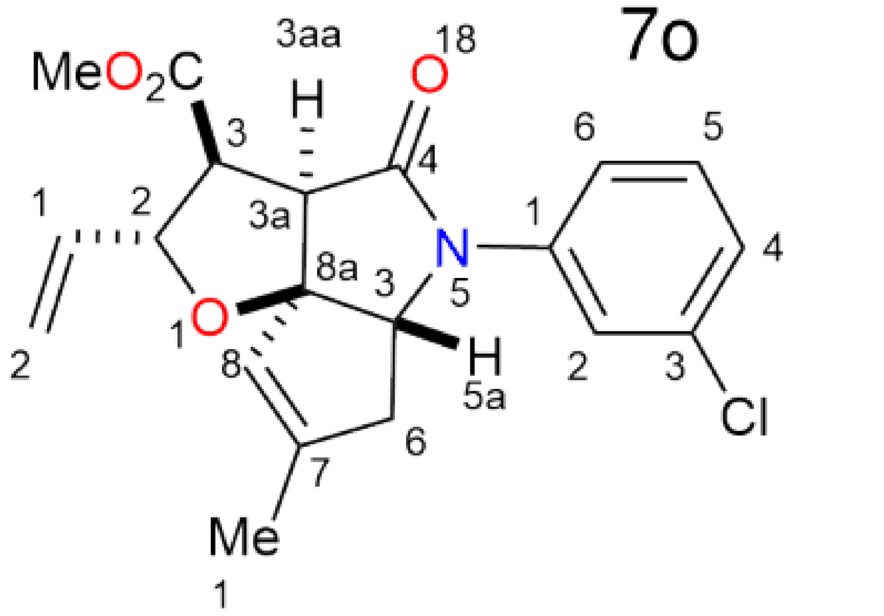

7o

FZ8536-1.JDF

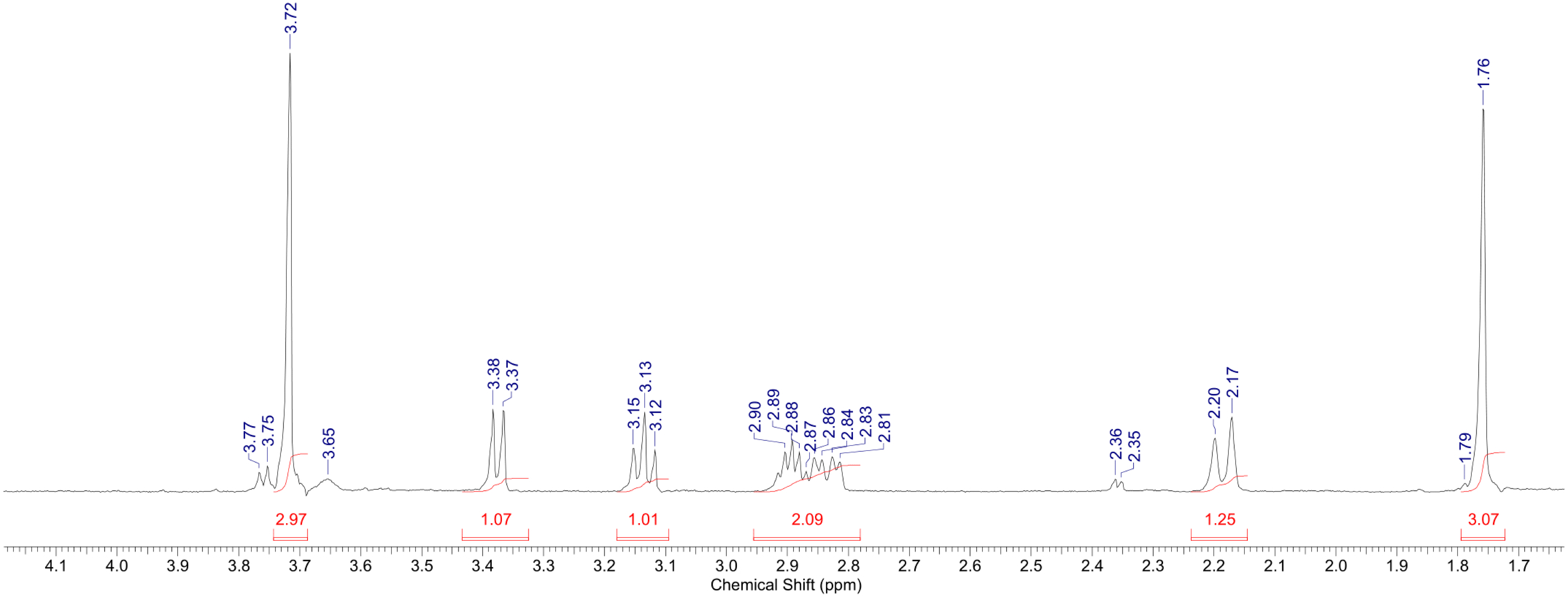

15.07.2020 20:46:54

**Formula** $C_{20}H_{20}ClNO_4$	**FW** 373.8301

**Acquisition Time (sec)**	0.6921	**Comment**	single pulse decoupled gated NOE			**Date**	24 Jun 2020 06:23:34
**Date Stamp**	24 Jun 2020 06:24:55			**File Name**	C:\USERS\Лаба534\DOWNLOADS\FZ8696-1.JDF		
**Frequency (MHz)**	150.91	**Nucleus**	13C	**Number of Transients**	4000	**Origin**	ECA 600
**Original Points Count**	32768	**Owner**	CKP	**Points Count**	32768	**Pulse Sequence**	single_pulse_dec
**Receiver Gain**	54.00	**Solvent**	CHLOROFORM-d			**Spectrum Offset (Hz)**	15079.3525
**Sweep Width (Hz)**	47348.49	**Temperature (degree C)**	22.100				

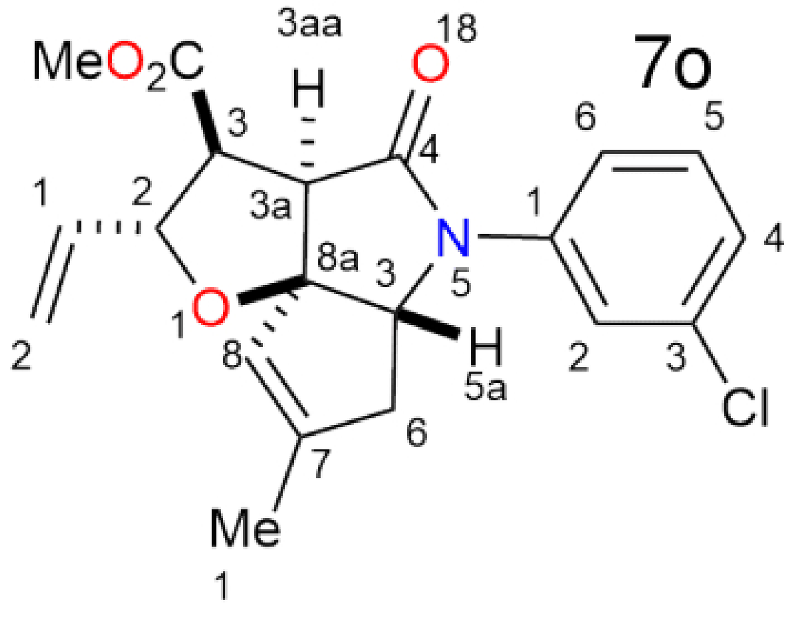

7o

FZ8696-1.JDF

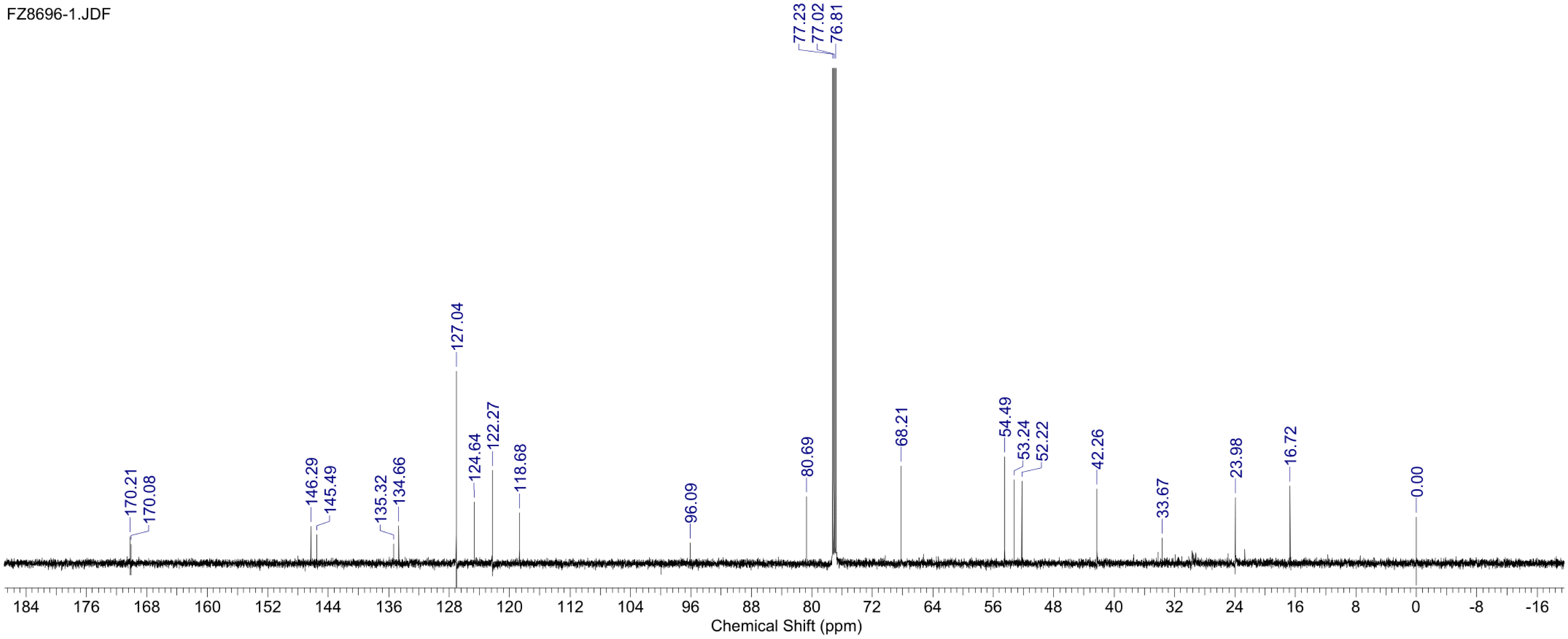

15.07.2020 20:47:01

Formula	$C_{20}H_{20}ClNO_4$	FW	373.8301

**Acquisition Time (sec)**	0.6921	**Comment**	single pulse decoupled gated NOE			**Date**	24 Jun 2020 06:23:34
**Date Stamp**	24 Jun 2020 06:24:55			**File Name**	C:\USERS\Лаба534\DOWNLOADS\FZ8696-1.JDF		
**Frequency (MHz)**	150.91	**Nucleus**	13C	**Number of Transients**	4000	**Origin**	ECA 600
**Original Points Count**	32768	**Owner**	CKP	**Points Count**	32768	**Pulse Sequence**	single_pulse_dec
**Receiver Gain**	54.00	**Solvent**	CHLOROFORM-d			**Spectrum Offset (Hz)**	15079.3525
**Sweep Width (Hz)**	47348.49	**Temperature (degree C)**	22.100				

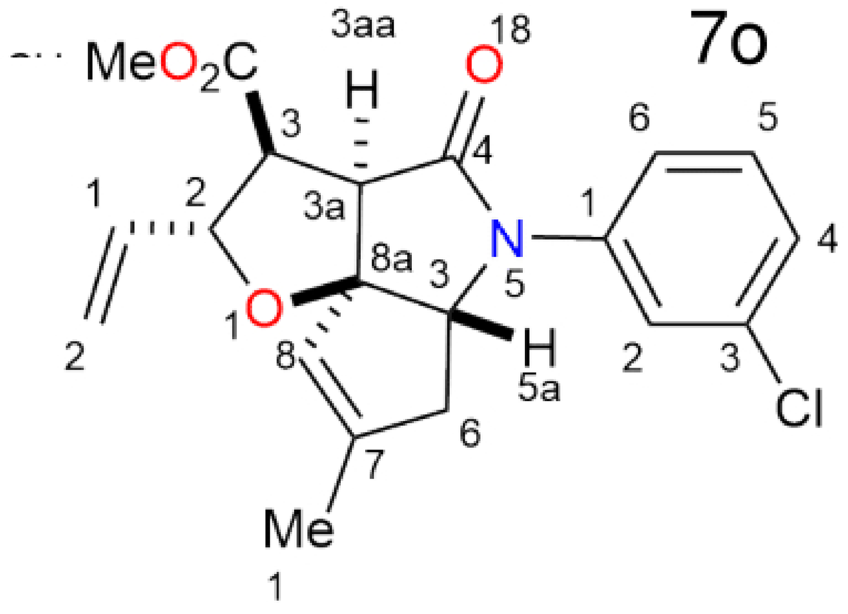

7o

FZ8696-1.JDF

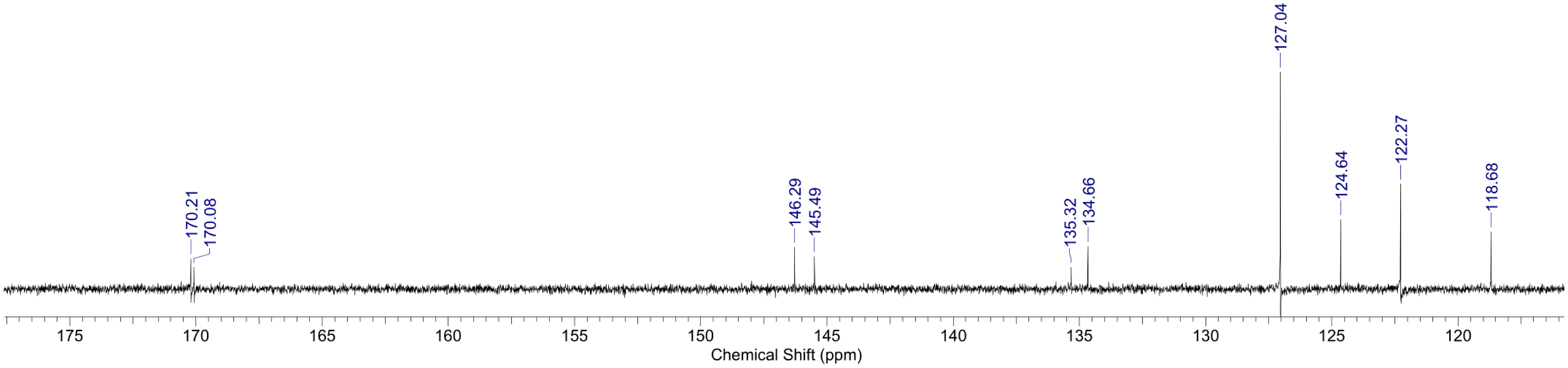

15.07.2020 20:47:15

Formula	$C_{20}H_{20}ClNO_4$	FW	373.8301

Acquisition Time (sec)	0.6921	***Comment***	single pulse decoupled gated NOE			***Date***	24 Jun 2020 06:23:34
Date Stamp	24 Jun 2020 06:24:55			***File Name***	C:\USERS\Лаба534\DOWNLOADS\FZ8696-1.JDF		
Frequency (MHz)	150.91	***Nucleus***	13C	***Number of Transients***	4000	***Origin***	ECA 600
Original Points Count	32768	***Owner***	CKP	***Points Count***	32768	***Pulse Sequence***	single_pulse_dec
Receiver Gain	54.00	***Solvent***	CHLOROFORM-d			***Spectrum Offset (Hz)***	15079.3525
Sweep Width (Hz)	47348.49	***Temperature (degree C)***	22.100				

7o

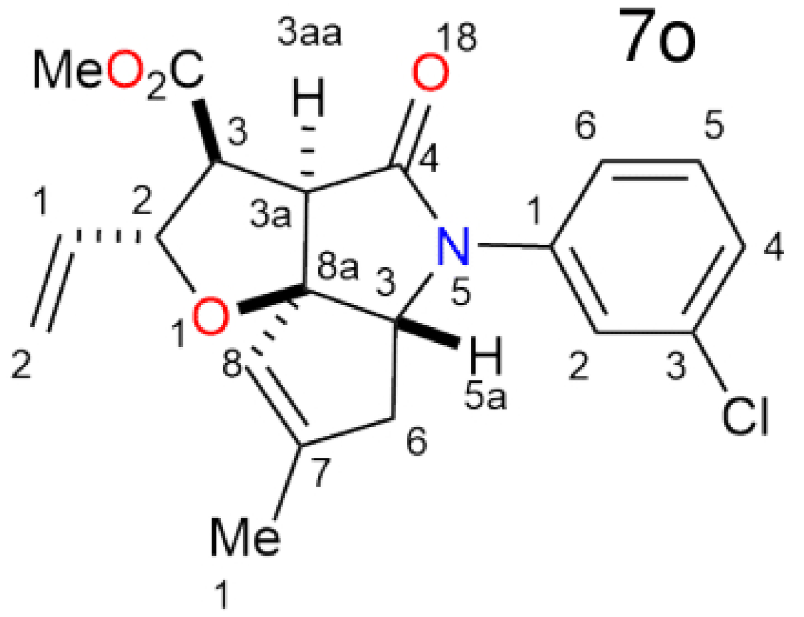

FZ8696-1.JDF

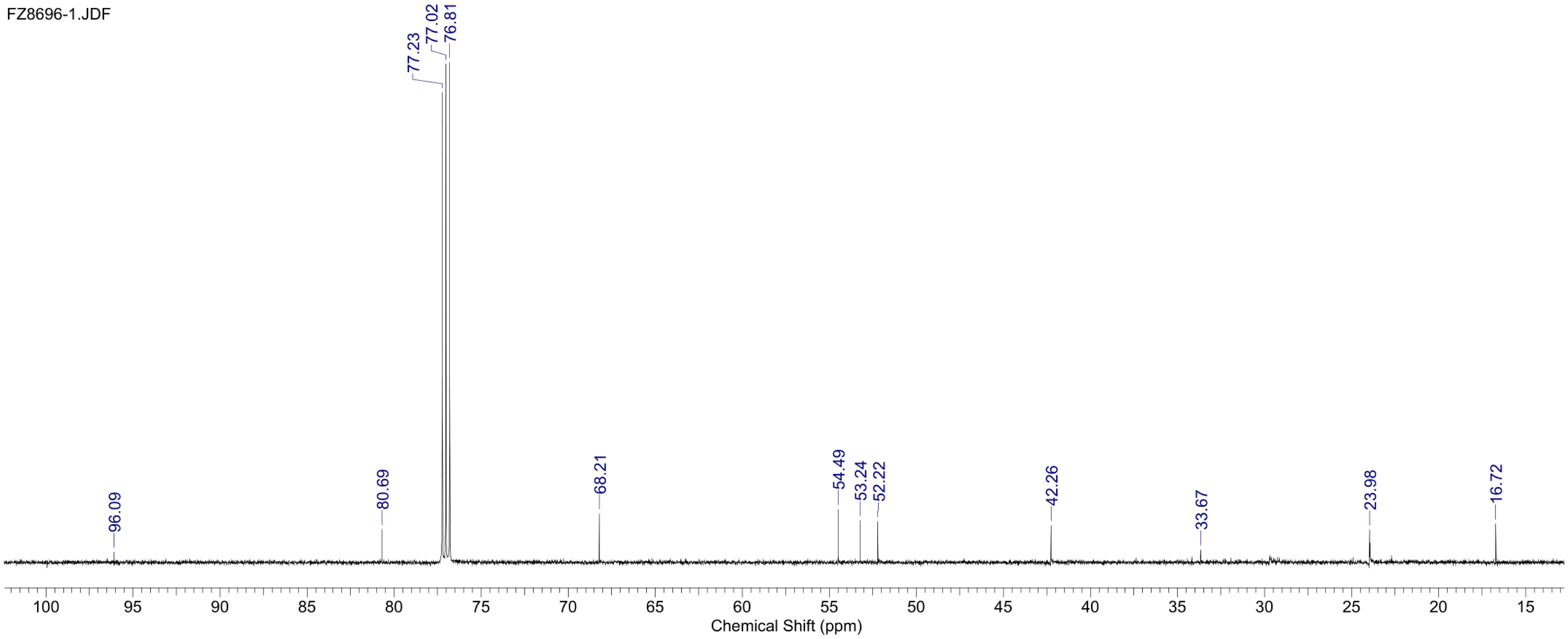

NMR spectrum data for compound 7p

Formula	$C_{20}H_{20}BrNO_4$	FW	418.2811

Acquisition Time (sec)	1.9818	Comment	single_pulse	Date	16 Jan 2020 12:22:01				
Date Stamp	16 Jan 2020 11:41:22			File Name	C:\Users\User\Desktop\17.01.20\FZ8345-1.jdf			Frequency (MHz)	600.17
Nucleus	1H	Number of Transients	8	Origin	ECA 600	Original Points Count	32768	Owner	delta
Points Count	32768	Pulse Sequence	single_pulse.ex2			Receiver Gain	54.00	Solvent	CHLOROFORM-d
Spectrum Offset (Hz)	5409.1133	Sweep Width (Hz)	16534.39	Temperature (degree C)	22.300				

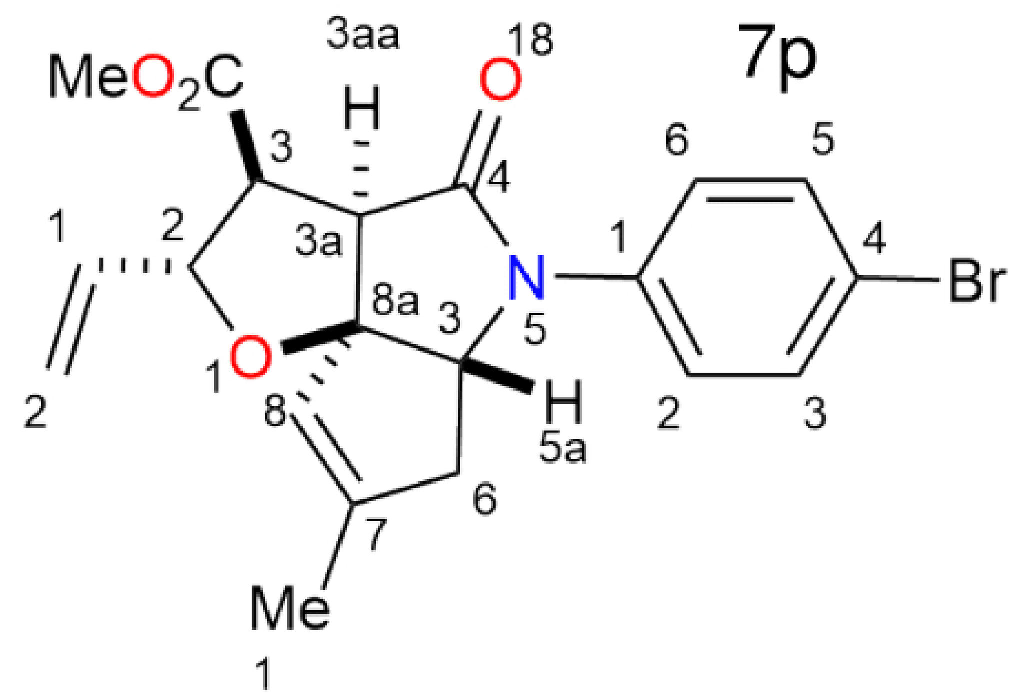

7p

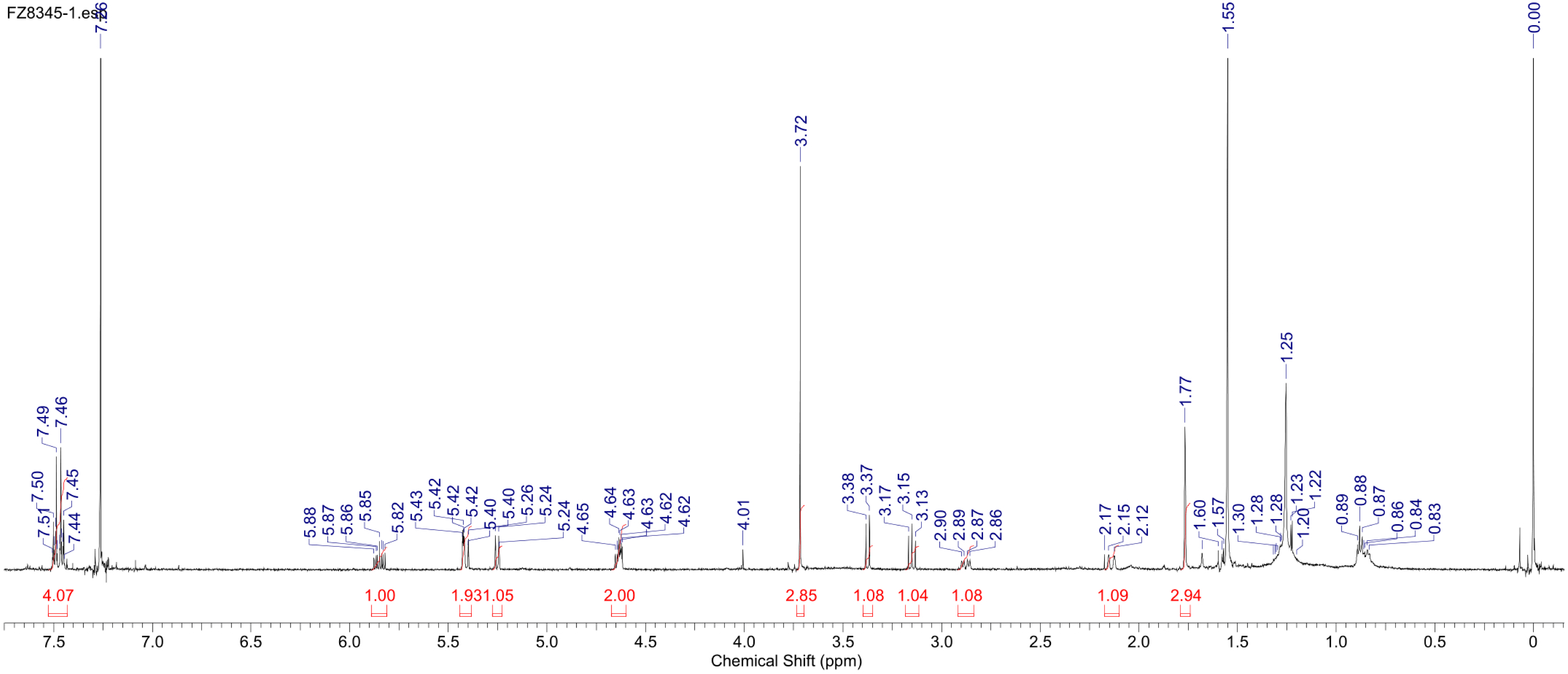

Formula	$C_{20}H_{20}BrNO_4$	FW	418.2811

**Acquisition Time (sec)**	1.9818	**Comment**	single_pulse	**Date**	16 Jan 2020 12:22:01		
**Date Stamp**	16 Jan 2020 11:41:22			**File Name**	C:\Users\User\Desktop\17.01.20\FZ8345-1.jdf	**Frequency (MHz)**	600.17
**Nucleus**	1H	**Number of Transients**	8	**Origin**	ECA 600	**Original Points Count**	32768
**Owner**	delta						
**Points Count**	32768	**Pulse Sequence**	single_pulse.ex2			**Receiver Gain**	54.00
**Solvent**	CHLOROFORM-d						
**Spectrum Offset (Hz)**	5409.1133	**Sweep Width (Hz)**	16534.39	**Temperature (degree C)**	22.300		

7p

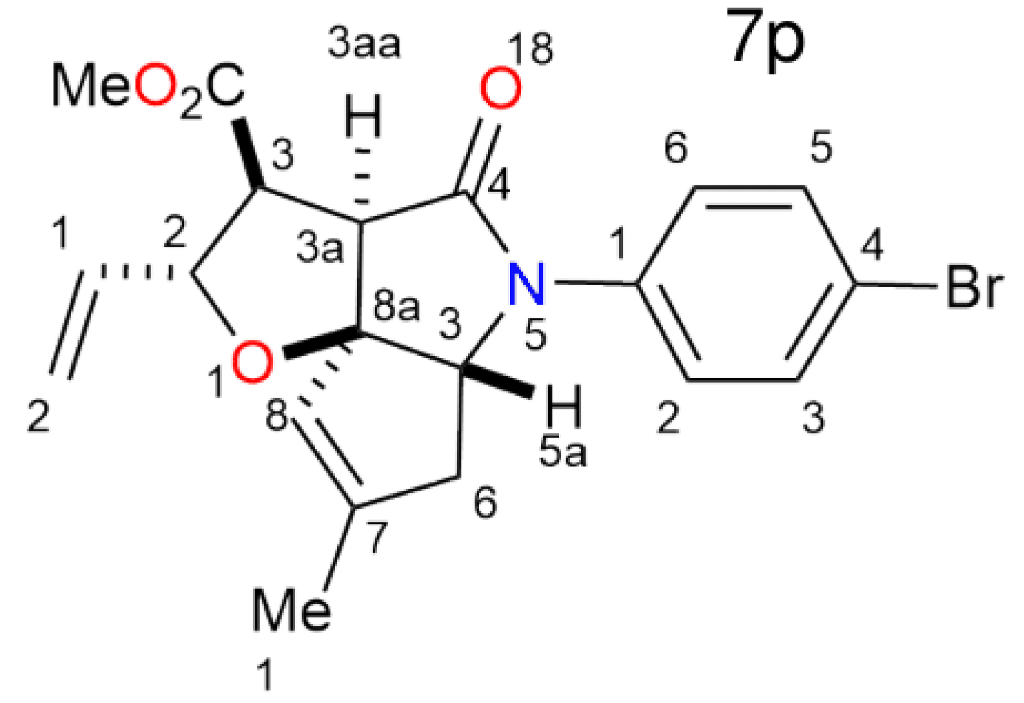

FZ8345-1.esp

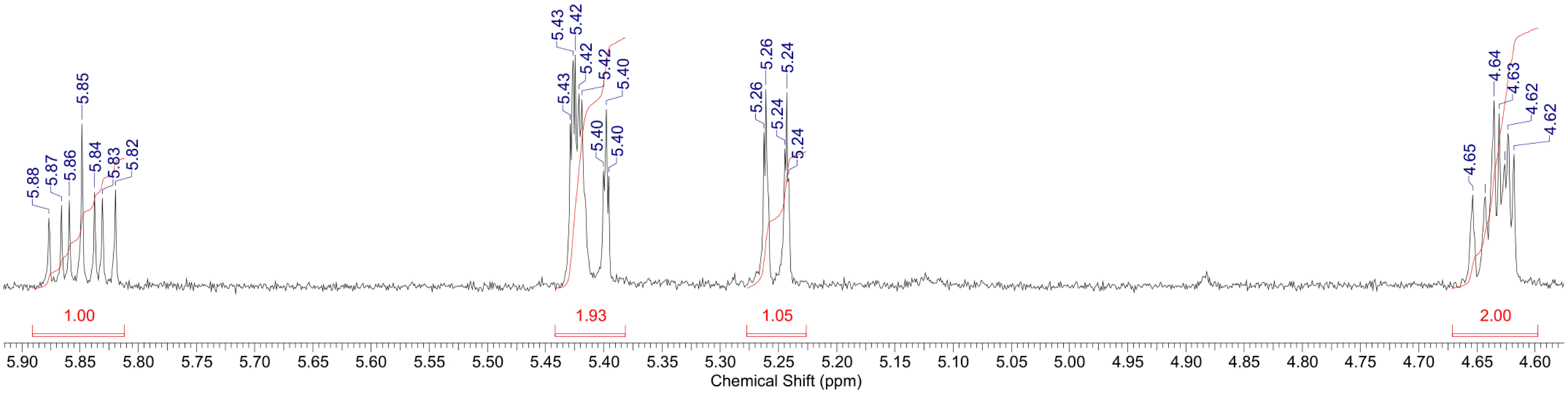

Formula $C_{20}H_{20}BrNO_4$	FW 418.2811

Acquisition Time (sec)	1.9818	Comment	single_pulse	Date	16 Jan 2020 12:22:01			
Date Stamp	16 Jan 2020 11:41:22			File Name	C:\Users\User\Desktop\17.01.20\FZ8345-1.jdf		Frequency (MHz)	600.17
Nucleus	1H	Number of Transients	8	Origin	ECA 600	Original Points Count 32768	Owner	delta
Points Count	32768	Pulse Sequence	single_pulse.ex2			Receiver Gain 54.00	Solvent	CHLOROFORM-d
Spectrum Offset (Hz)	5409.1133	Sweep Width (Hz)	16534.39	Temperature (degree C)	22.300			

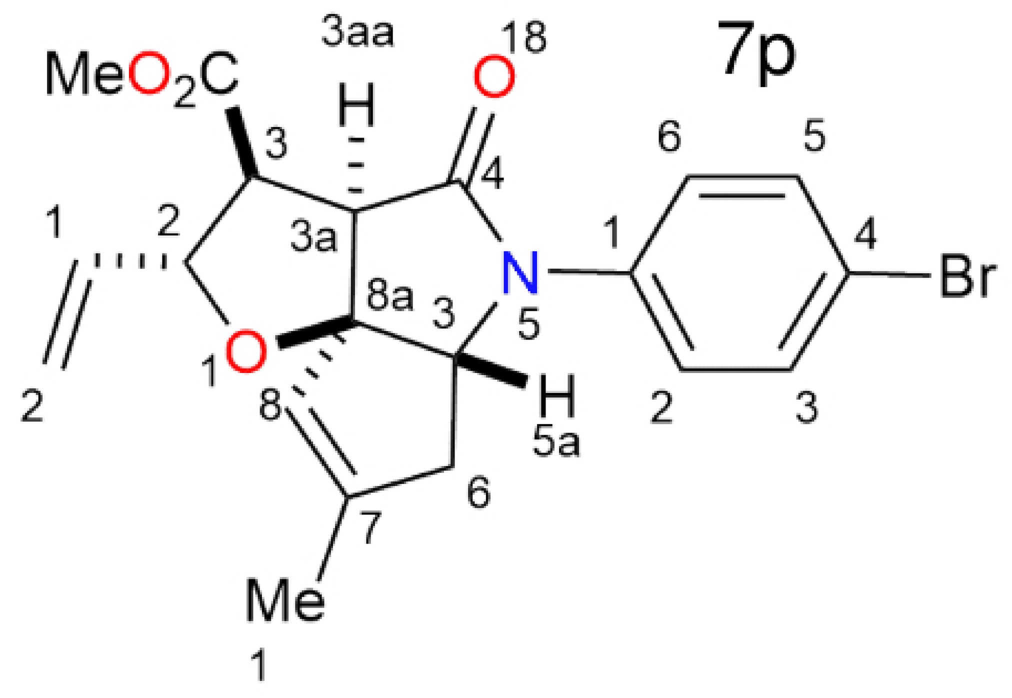

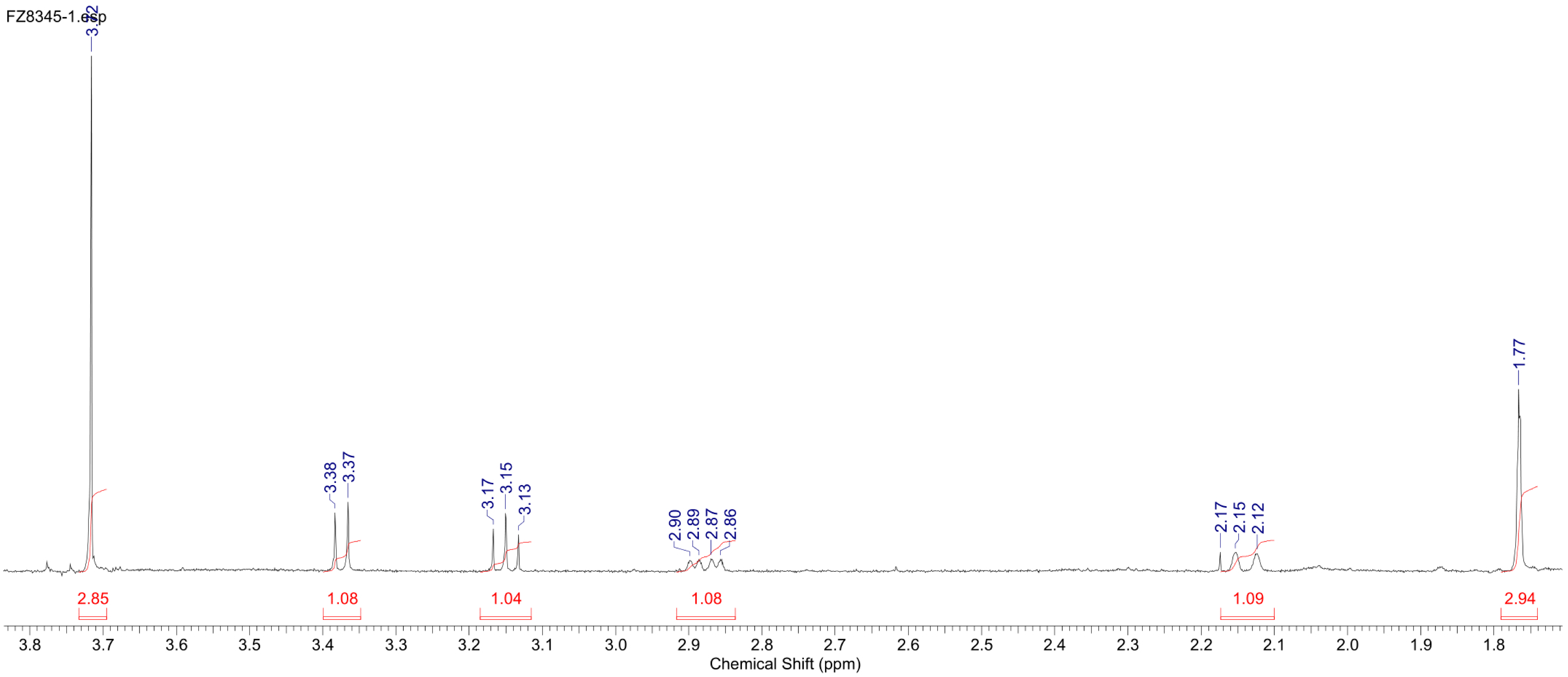

**Formula** $C_{20}H_{20}BrNO_4$	**FW** 418.2811

**Acquisition Time (sec)**	0.6921	**Comment**	single pulse decoupled gated NOE	**Date**	27 Feb 2020 17:06:14
**Date Stamp**	27 Feb 2020 16:25:56	**File Name**	C:\USERS\лаба 534\DESKTOP\Саша\ЯМР\NMR FIDS\FZ8533-1.JDF		
**Frequency (MHz)**	150.91	**Nucleus**	13C	**Number of Transients**	4000
**Origin**	ECA 600	**Original Points Count**	32768	**Owner**	CKP
**Points Count**	32768	**Pulse Sequence**	single_pulse_dec	**Receiver Gain**	54.00
**Solvent**	CHLOROFORM-d	**Spectrum Offset (Hz)**	15091.3428	**Sweep Width (Hz)**	47348.49
**Temperature (degree C)**	23.800				

7p

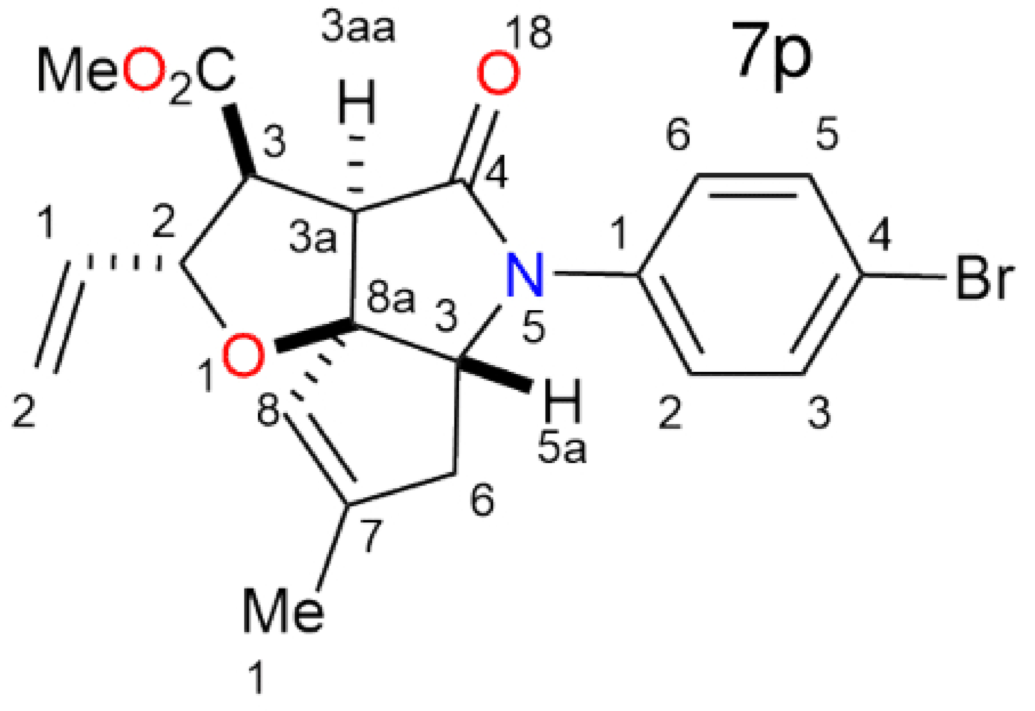

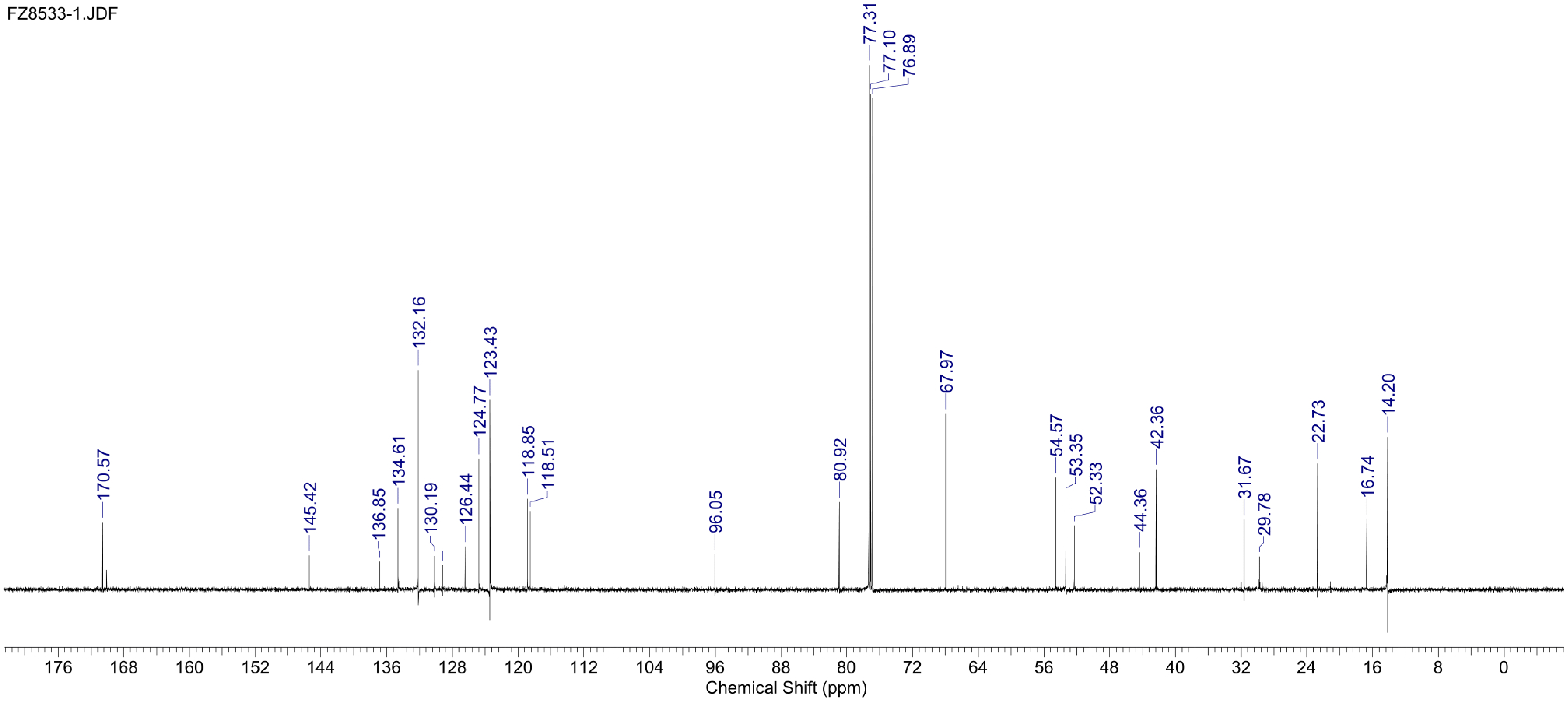

**Formula** $C_{20}H_{20}BrNO_4$	**FW** 418.2811

**Acquisition Time (sec)**	0.6921	**Comment**	single pulse decoupled gated NOE	**Date**	27 Feb 2020 17:06:14
**Date Stamp**	27 Feb 2020 16:25:56	**File Name**	C:\USERS\лаба 534\DESKTOP\Саша\ЯМР\NMR FIDS\FZ8533-1.JDF		
**Frequency (MHz)**	150.91	**Nucleus**	13C	**Number of Transients**	4000
**Origin**	ECA 600				
**Original Points Count**	32768	**Owner**	CKP	**Points Count**	32768
**Pulse Sequence**	single_pulse_dec				
**Receiver Gain**	54.00	**Solvent**	CHLOROFORM-d	**Spectrum Offset (Hz)**	15091.3428
**Sweep Width (Hz)**	47348.49	**Temperature (degree C)**	23.800		

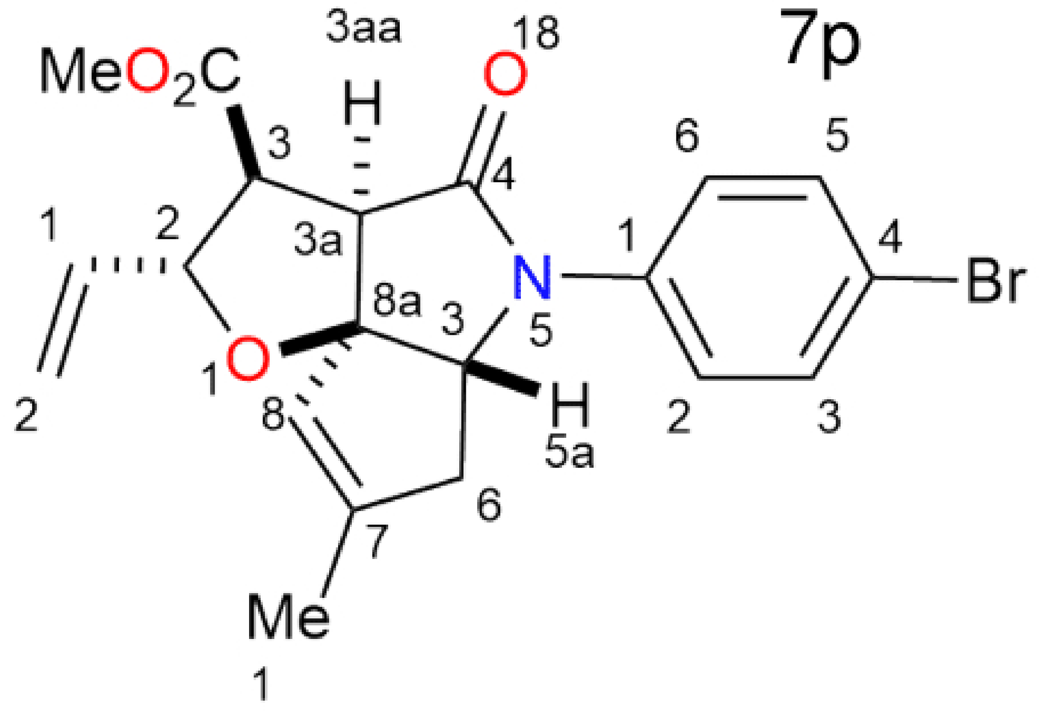

FZ8533-1.JDF

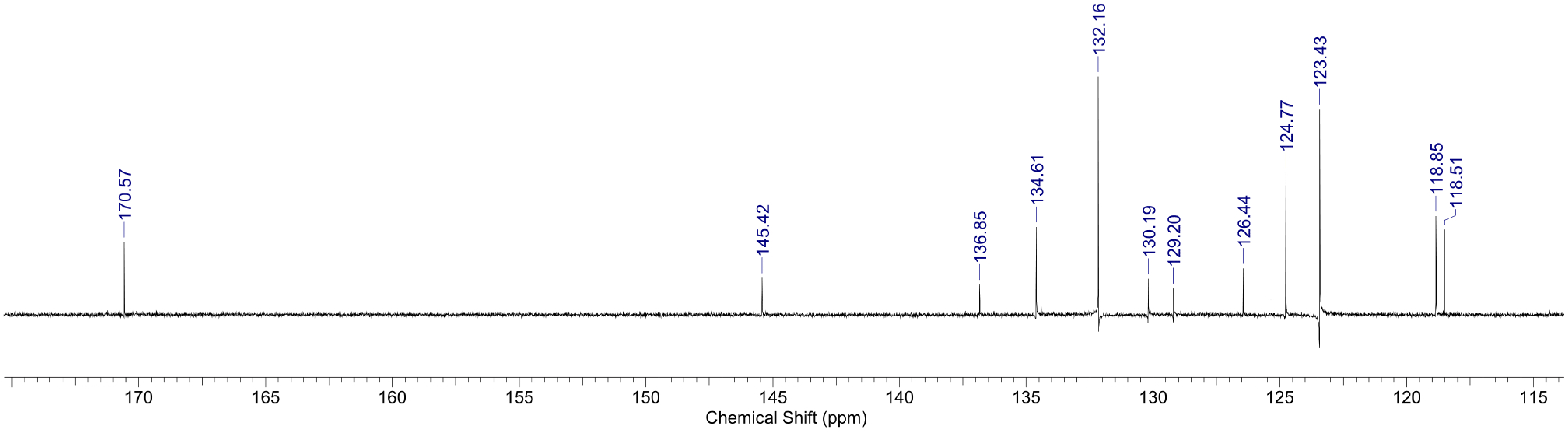

**Formula** $C_{20}H_{20}BrNO_4$	**FW** 418.2811

Acquisition Time (sec)	0.6921	***Comment***	single pulse decoupled gated NOE	***Date***	27 Feb 2020 17:06:14
Date Stamp	27 Feb 2020 16:25:56	***File Name***	C:\USERS\лаба 534\DESKTOP\Саша\ЯМР\NMR FIDS\FZ8533-1.JDF		
Frequency (MHz)	150.91	***Nucleus***	13C	***Number of Transients***	4000
Origin	ECA 600				
Original Points Count	32768	***Owner***	CKP	***Points Count***	32768
Pulse Sequence	single_pulse_dec				
Receiver Gain	54.00	***Solvent***	CHLOROFORM-d	***Spectrum Offset (Hz)***	15091.3428
Sweep Width (Hz)	47348.49	***Temperature (degree C)***	23.800		

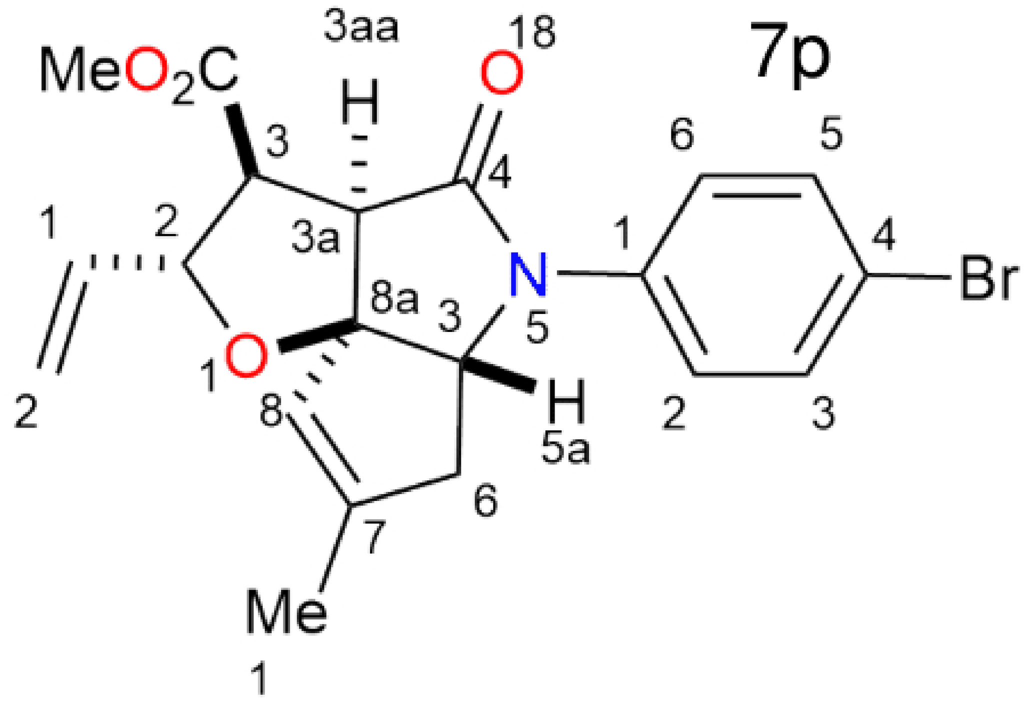

7p

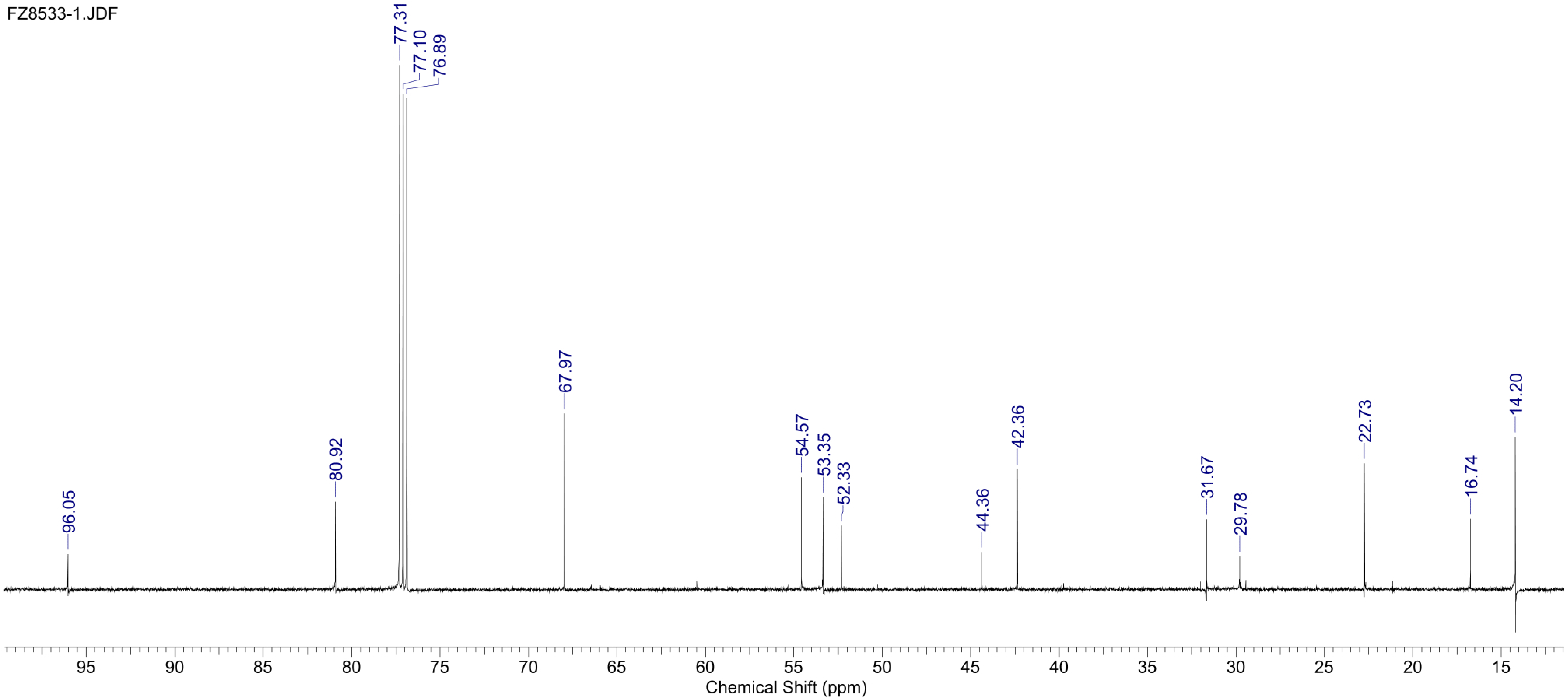

NMR spectrum data for compound 8a

Formula	$C_{21}H_{22}ClNO_4$	FW	387.8567

Acquisition Time (sec)	1.9818	***Comment***	single_pulse	***Date***	23 Oct 2020 10:04:05	***Date Stamp***	23 Oct 2020 10:03:11
File Name	C:\Users\User\Desktop\22.10.20\FZ9208-1.jdf	***Frequency (MHz)***	600.17	***Nucleus***	1H	***Number of Transients***	8
Origin	ECA 600	***Original Points Count***	32768	***Owner***	CKP	***Points Count***	32768
Pulse Sequence	single_pulse.ex2	***Receiver Gain***	54.00	***Solvent***	CHLOROFORM-d	***Spectrum Offset (Hz)***	5401.5503
Sweep Width (Hz)	16534.39						

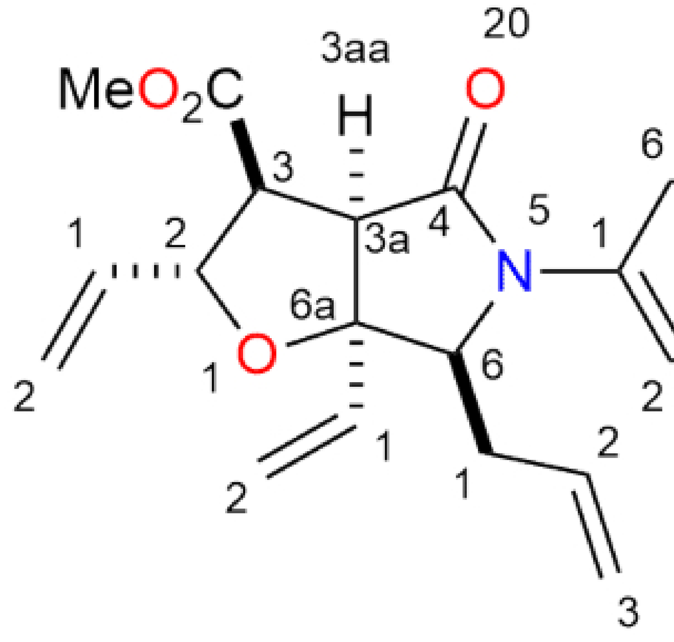

8a

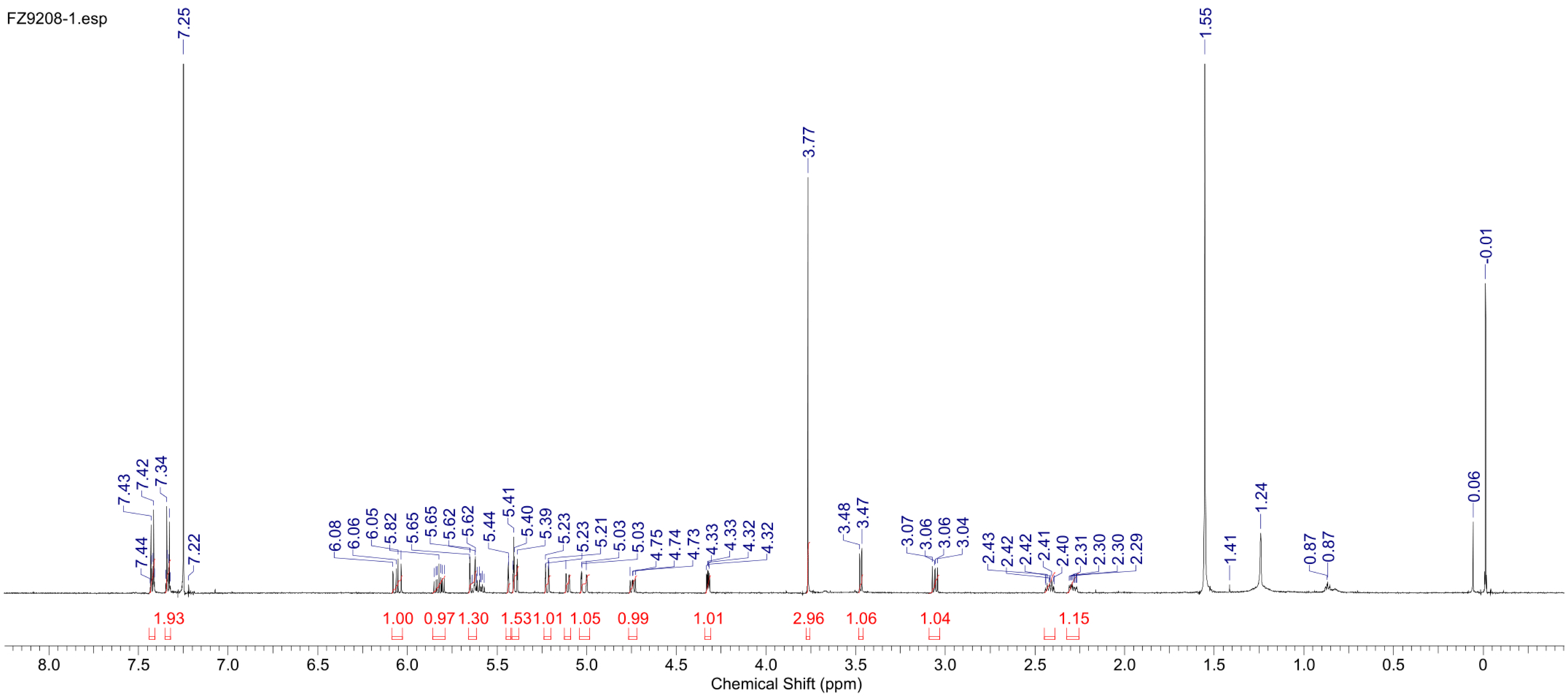

Formula	$C_{21}H_{22}ClNO_4$	FW	387.8567

Acquisition Time (sec)	1.9818	Comment	single_pulse	Date	23 Oct 2020 10:04:05			Date Stamp	23 Oct 2020 10:03:11
File Name	C:\Users\User\Desktop\22.10.20\FZ9208-1.jdf			Frequency (MHz)	600.17	Nucleus	1H	Number of Transients	8
Origin	ECA 600	Original Points Count	32768	Owner	CKP	Points Count	32768	Pulse Sequence	single_pulse.ex2
Receiver Gain	54.00	Solvent	CHLOROFORM-d			Spectrum Offset (Hz)	5401.5503	Sweep Width (Hz)	16534.39

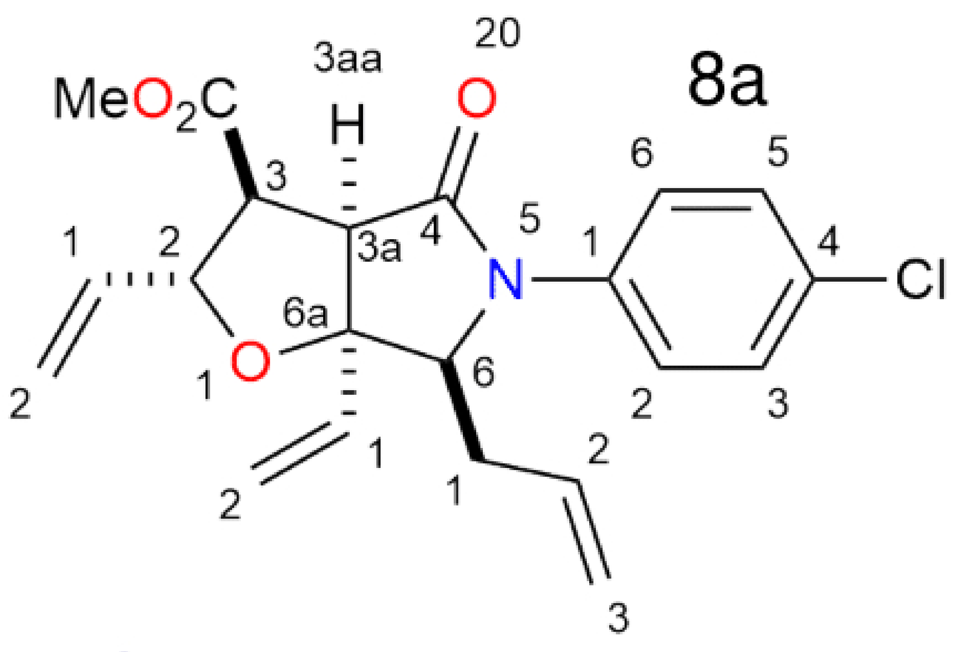

FZ9208-1.esp

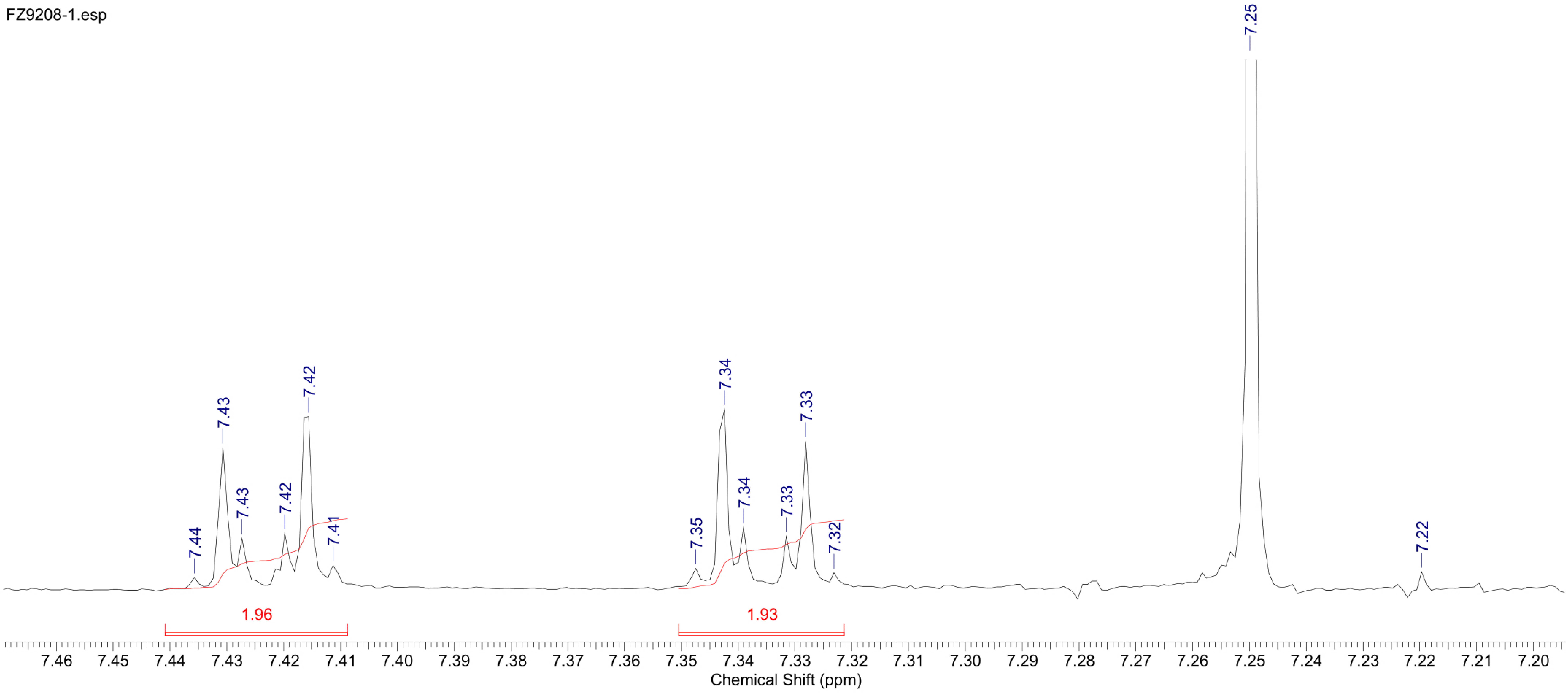

Formula $C_{21}H_{22}ClNO_4$	FW 387.8567

Acquisition Time (sec)	1.9818	Comment	single_pulse	Date	23 Oct 2020 10:04:05			Date Stamp	23 Oct 2020 10:03:11
File Name	C:\Users\User\Desktop\22.10.20\FZ9208-1.jdf			Frequency (MHz)	600.17	Nucleus	1H	Number of Transients	8
Origin	ECA 600	Original Points Count	32768	Owner	CKP	Points Count	32768	Pulse Sequence	single_pulse.ex2
Receiver Gain	54.00	Solvent	CHLOROFORM-d			Spectrum Offset (Hz)	5401.5503	Sweep Width (Hz)	16534.39

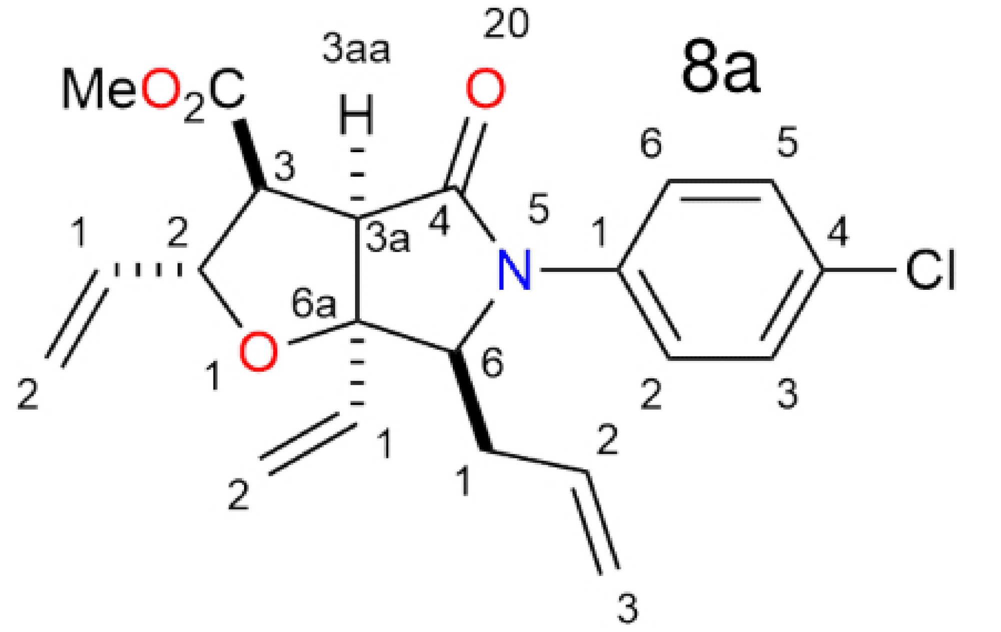

8a

FZ9208-1.esp

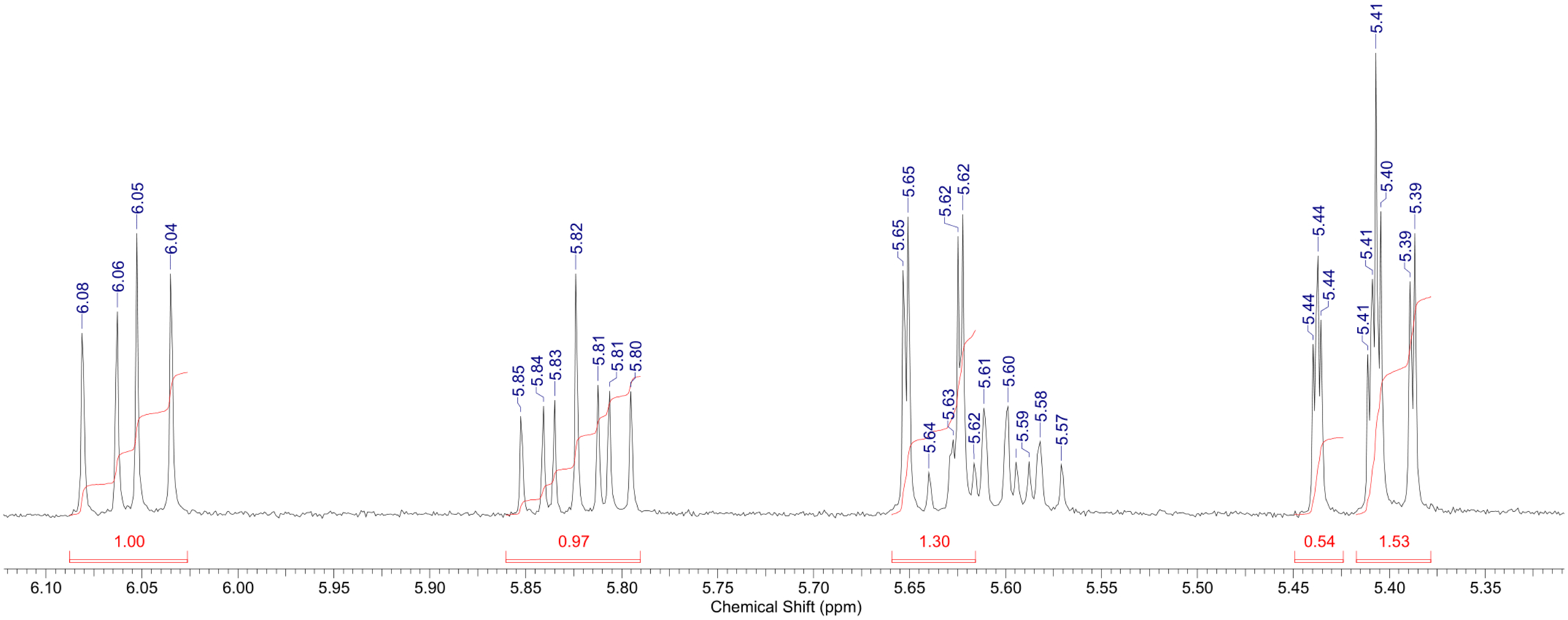

Formula $C_{21}H_{22}ClNO_4$	FW 387.8567

Acquisition Time (sec)	1.9818	Comment	single_pulse	Date	23 Oct 2020 10:04:05			Date Stamp	23 Oct 2020 10:03:11
File Name	C:\Users\User\Desktop\22.10.20\FZ9208-1.jdf			Frequency (MHz)	600.17	Nucleus	1H	Number of Transients	8
Origin	ECA 600	Original Points Count	32768	Owner	CKP	Points Count	32768	Pulse Sequence	single_pulse.ex2
Receiver Gain	54.00	Solvent	CHLOROFORM-d			Spectrum Offset (Hz)	5401.5503	Sweep Width (Hz)	16534.39

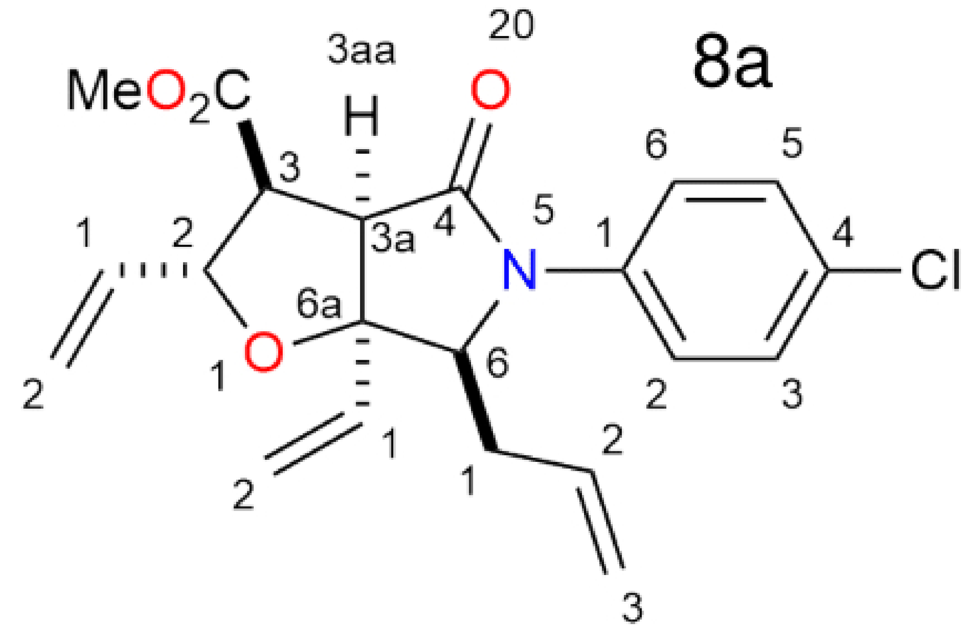

8a

FZ9208-1.esp

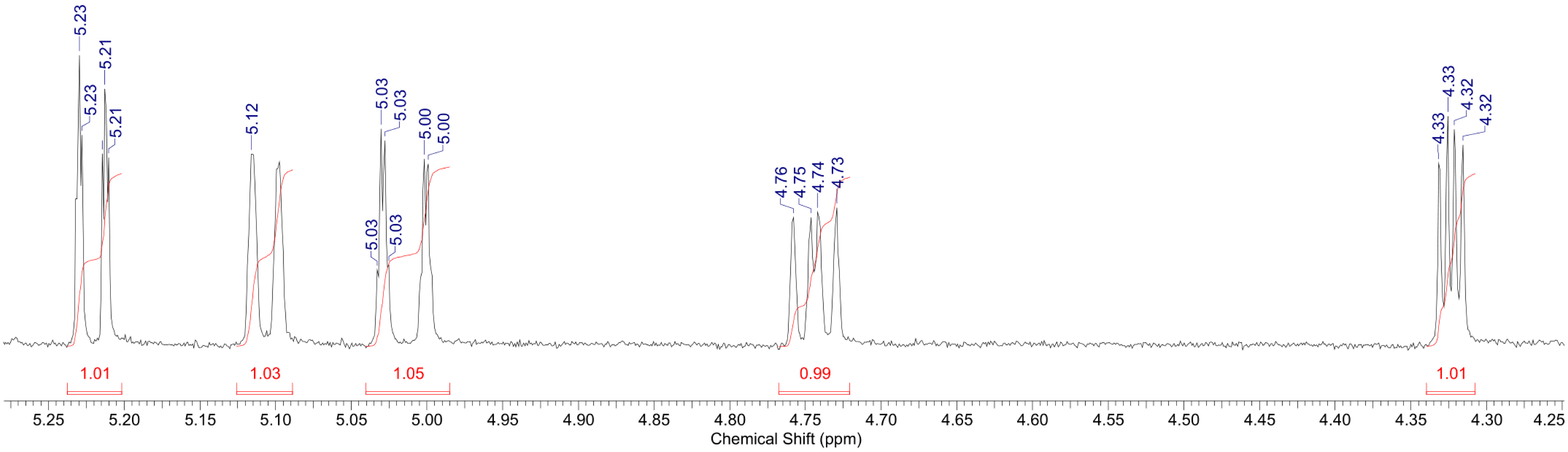

Formula $C_{21}H_{22}ClNO_4$	FW 387.8567

**Acquisition Time (sec)**	1.9818	**Comment**	single_pulse	**Date**	23 Oct 2020 10:04:05			**Date Stamp**	23 Oct 2020 10:03:11
**File Name**	C:\Users\User\Desktop\22.10.20\FZ9208-1.jdf			**Frequency (MHz)**	600.17	**Nucleus**	1H	**Number of Transients**	8
**Origin**	ECA 600	**Original Points Count**	32768	**Owner**	CKP	**Points Count**	32768	**Pulse Sequence**	single_pulse.ex2
**Receiver Gain**	54.00	**Solvent**	CHLOROFORM-d			**Spectrum Offset (Hz)**	5401.5503	**Sweep Width (Hz)**	16534.39

8a

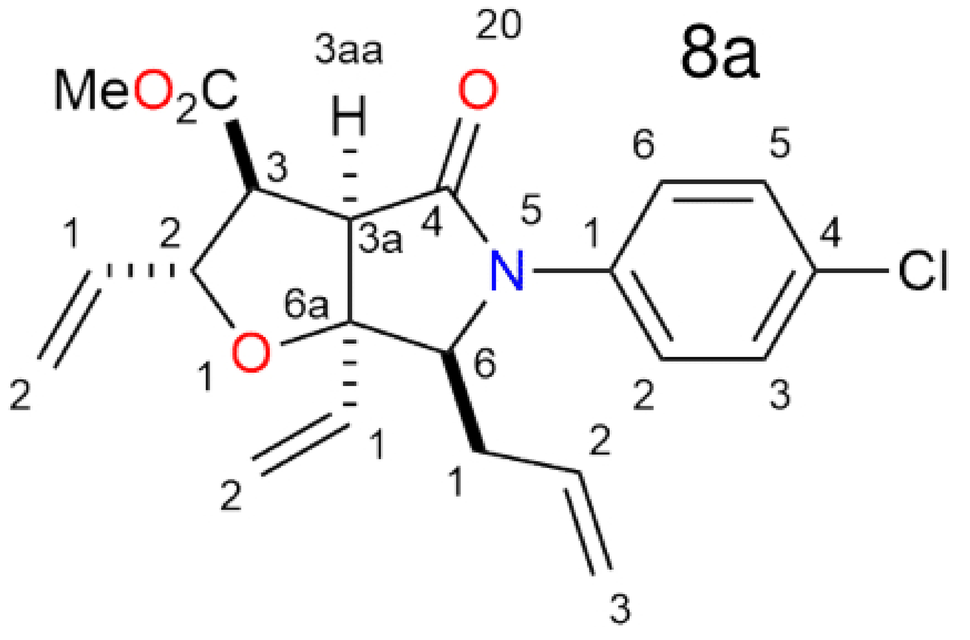

FZ9208-1.esp

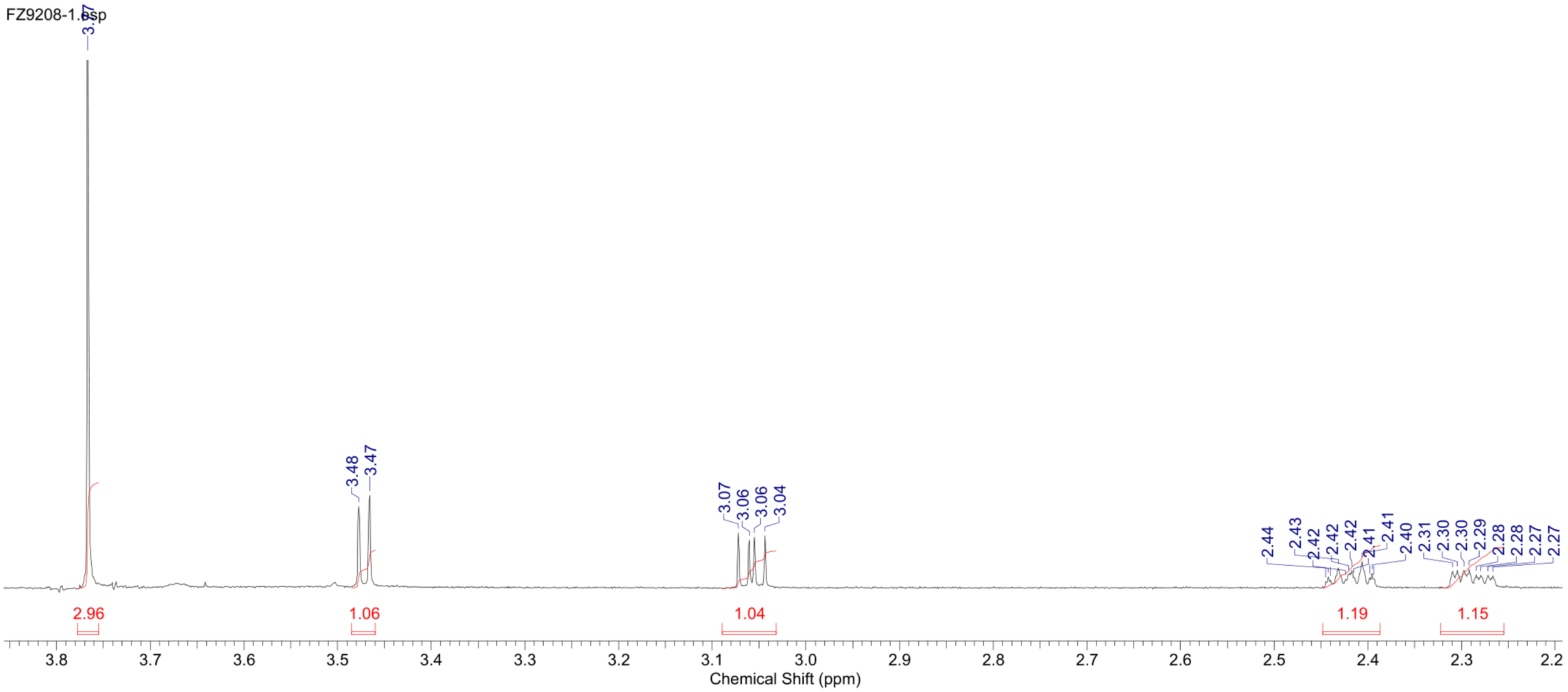

Formula	$C_{21}H_{22}ClNO_4$	FW	387.8567

**Acquisition Time (sec)**	0.6921	**Comment**	single pulse decoupled gated NOE			**Date**	27 Oct 2020 05:18:24
**Date Stamp**	27 Oct 2020 04:32:06	**File Name**	C:\Users\User\Desktop\22.10.20\FZ9208-2.jdf	**Frequency (MHz)**	150.91		
**Nucleus**	13C	**Number of Transients**	40000	**Origin**	ECA 600	**Original Points Count**	32768
**Owner**	CKP						
**Points Count**	32768	**Pulse Sequence**	single_pulse_dec	**Receiver Gain**	56.00	**Solvent**	CHLOROFORM-d
**Spectrum Offset (Hz)**	15079.3525	**Sweep Width (Hz)**	47348.49				

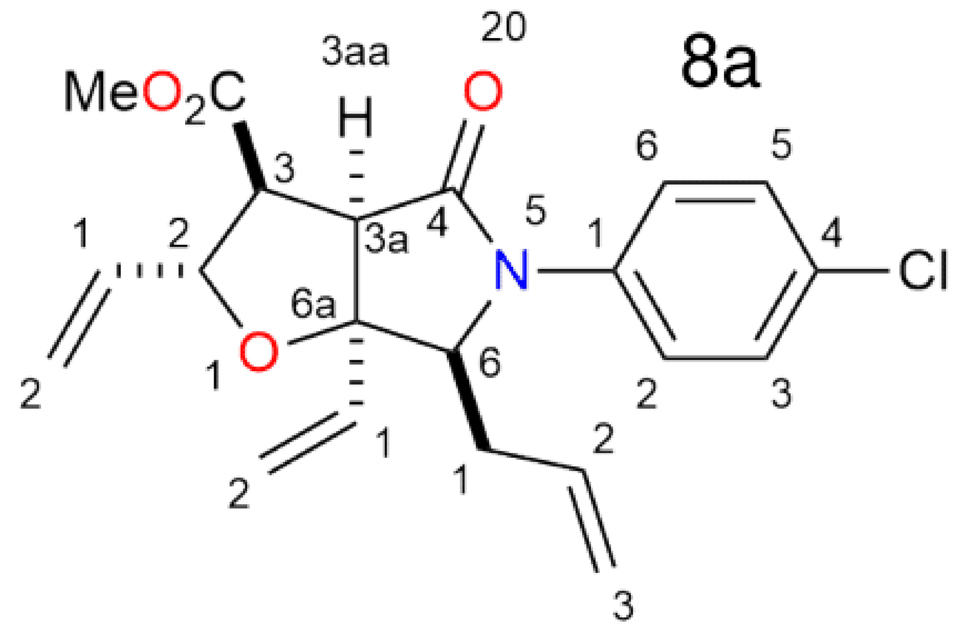

8a

FZ9208-2.esp

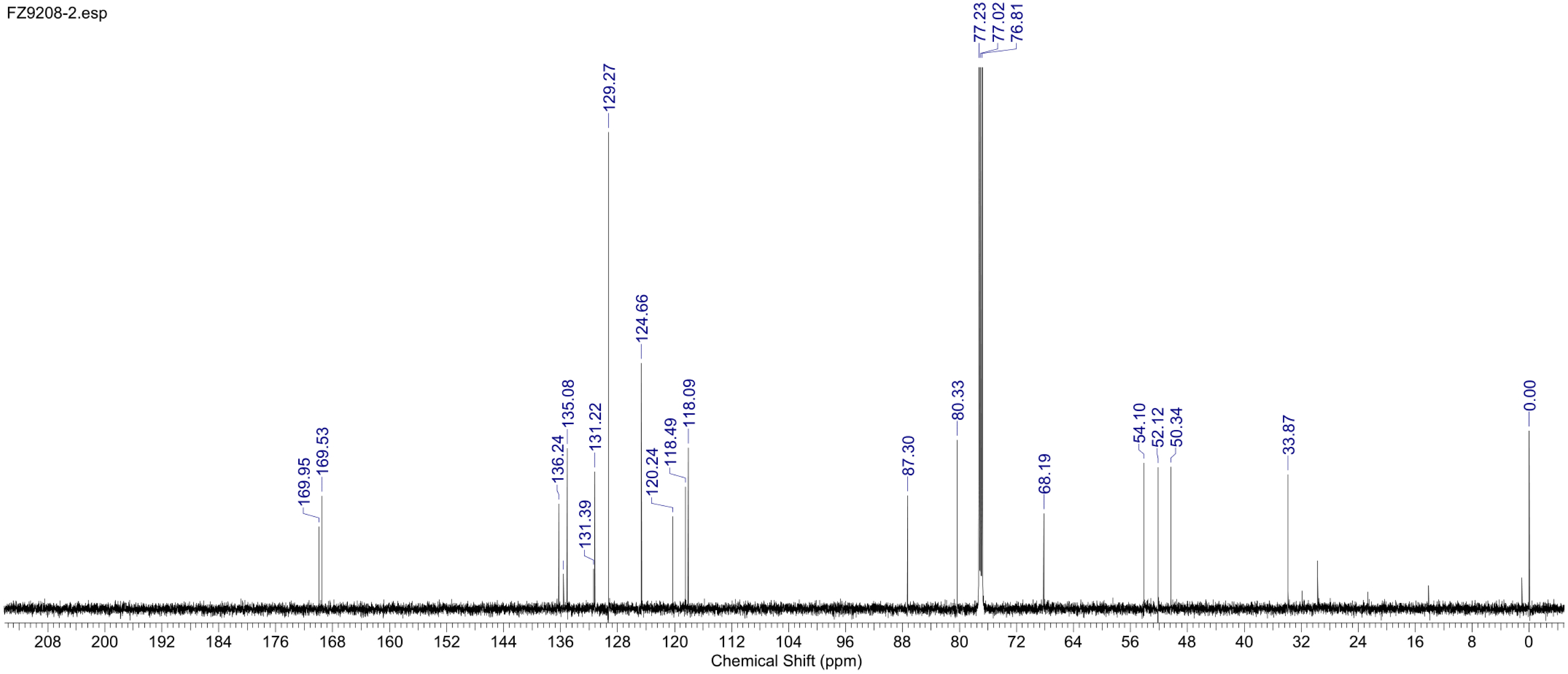

Formula	$C_{21}H_{22}ClNO_4$	FW	387.8567

**Acquisition Time (sec)**	0.6921	**Comment**	single pulse decoupled gated NOE			**Date**	27 Oct 2020 05:18:24
**Date Stamp**	27 Oct 2020 04:32:06	**File Name**	C:\Users\User\Desktop\22.10.20\FZ9208-2.jdf	**Frequency (MHz)**	150.91		
**Nucleus**	13C	**Number of Transients**	40000	**Origin**	ECA 600	**Original Points Count**	32768
**Owner**	CKP						
**Points Count**	32768	**Pulse Sequence**	single_pulse_dec	**Receiver Gain**	56.00	**Solvent**	CHLOROFORM-d
**Spectrum Offset (Hz)**	15079.3525	**Sweep Width (Hz)**	47348.49				

8a

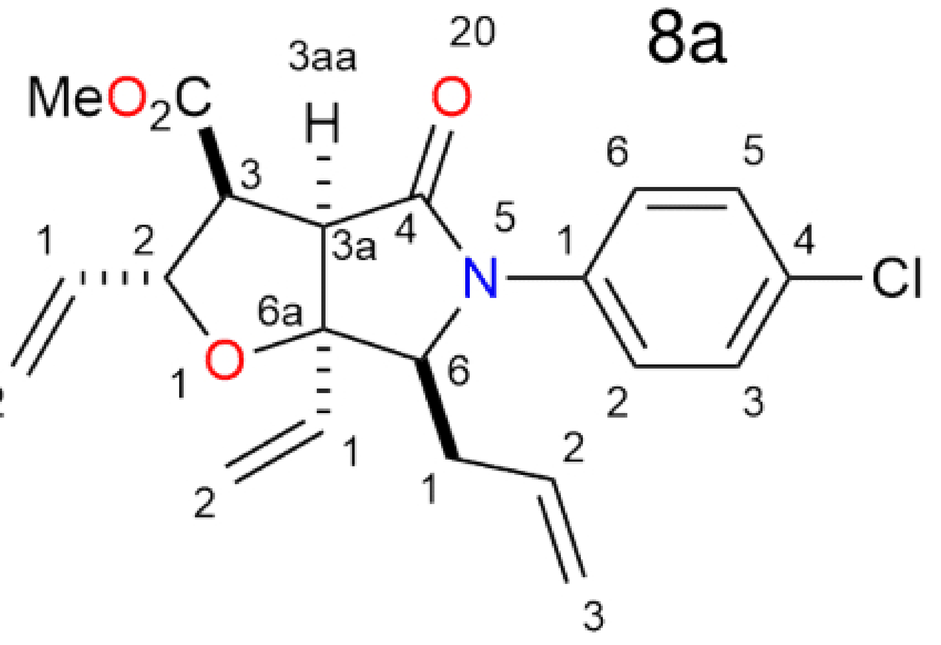

FZ9208-2.esp

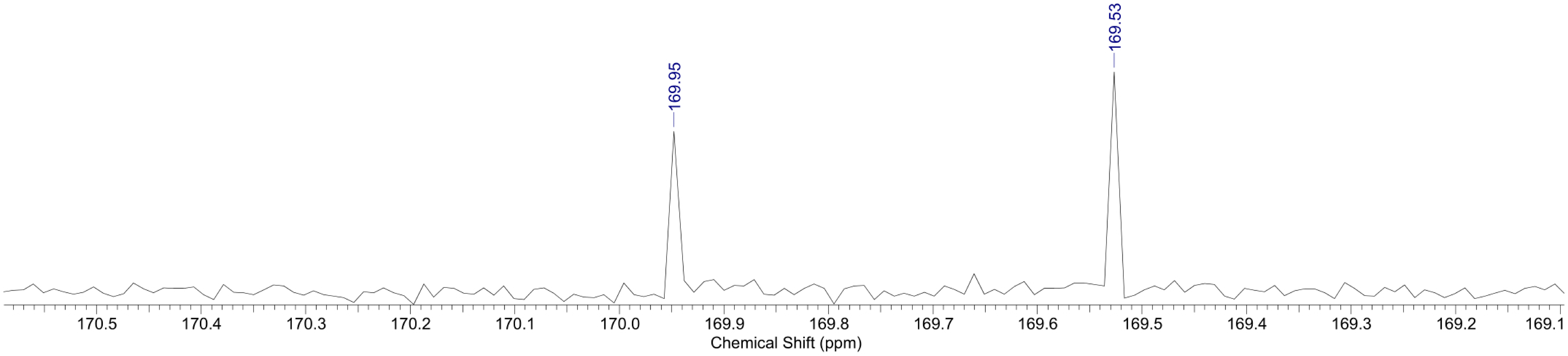

Formula $C_{21}H_{22}ClNO_4$	*FW* 387.8567

Acquisition Time (sec)	0.6921	***Comment***	single pulse decoupled gated NOE			***Date***	27 Oct 2020 05:18:24		
Date Stamp	27 Oct 2020 04:32:06			***File Name***	C:\Users\User\Desktop\22.10.20\FZ9208-2.jdf			***Frequency (MHz)***	150.91
Nucleus	13C	***Number of Transients***	40000	***Origin***	ECA 600	***Original Points Count***	32768	***Owner***	CKP
Points Count	32768	***Pulse Sequence***	single_pulse_dec			***Receiver Gain***	56.00	***Solvent***	CHLOROFORM-d
Spectrum Offset (Hz)	15079.3525	***Sweep Width (Hz)***	47348.49						

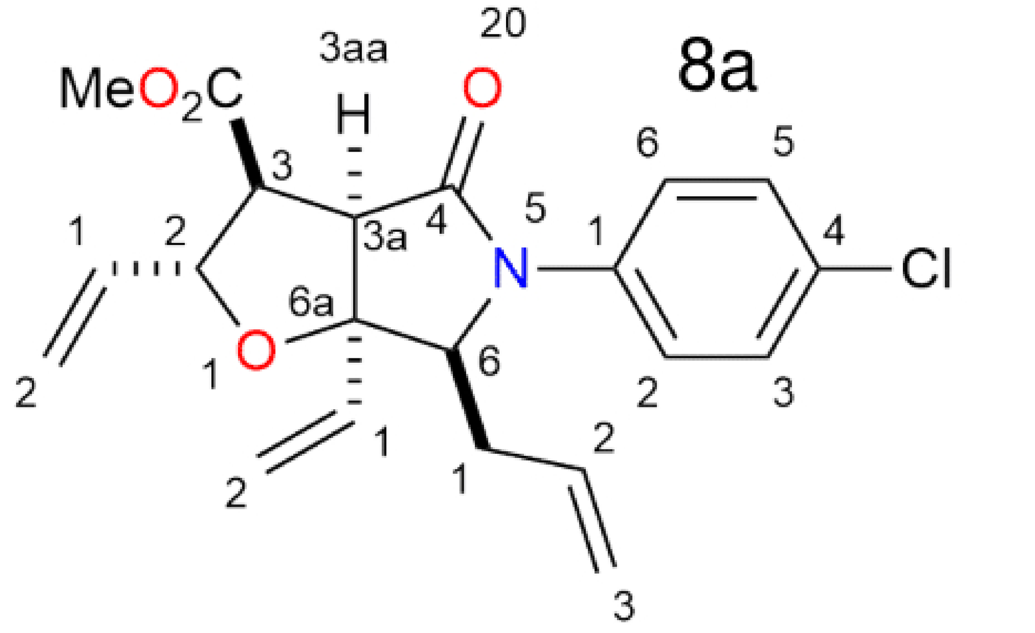

8a

FZ9208-2.esp

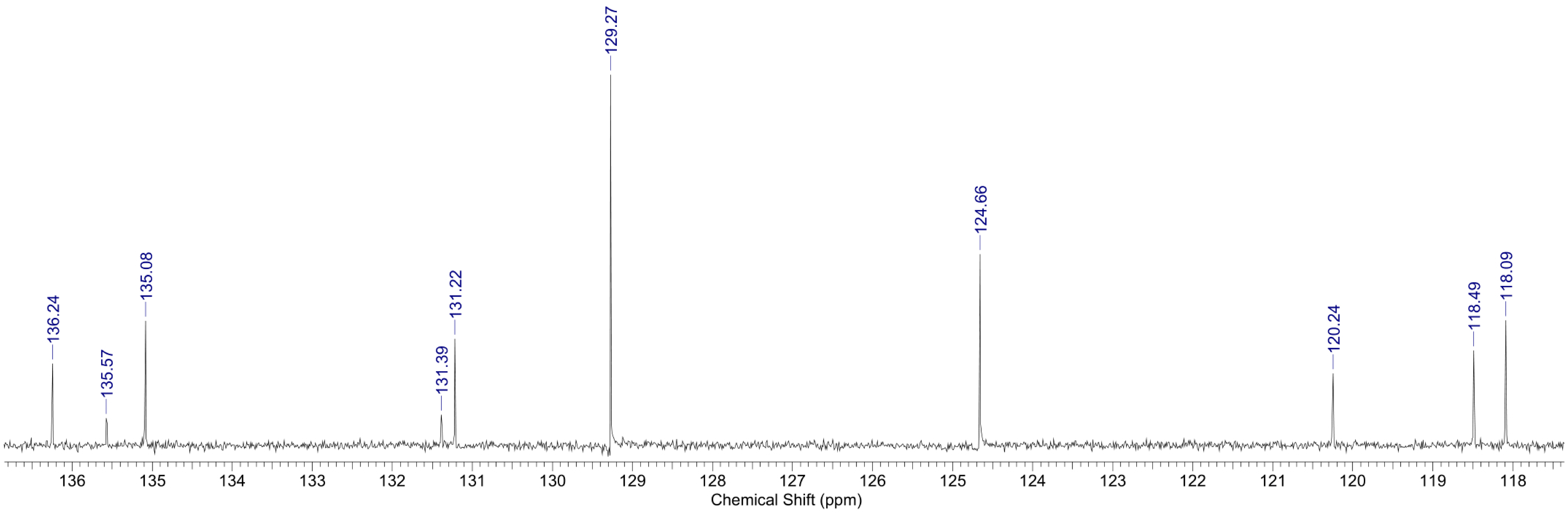

**Formula** $C_{21}H_{22}ClNO_4$	**FW** 387.8567

**Acquisition Time (sec)**	0.6921	**Comment**	single pulse decoupled gated NOE			**Date**	27 Oct 2020 05:18:24
**Date Stamp**	27 Oct 2020 04:32:06	**File Name**	C:\Users\User\Desktop\22.10.20\FZ9208-2.jdf	**Frequency (MHz)**	150.91		
**Nucleus**	13C	**Number of Transients**	40000	**Origin**	ECA 600	**Original Points Count**	32768
**Owner**	CKP	**Points Count**	32768	**Pulse Sequence**	single_pulse_dec	**Receiver Gain**	56.00
**Solvent**	CHLOROFORM-d	**Spectrum Offset (Hz)**	15079.3525	**Sweep Width (Hz)**	47348.49		

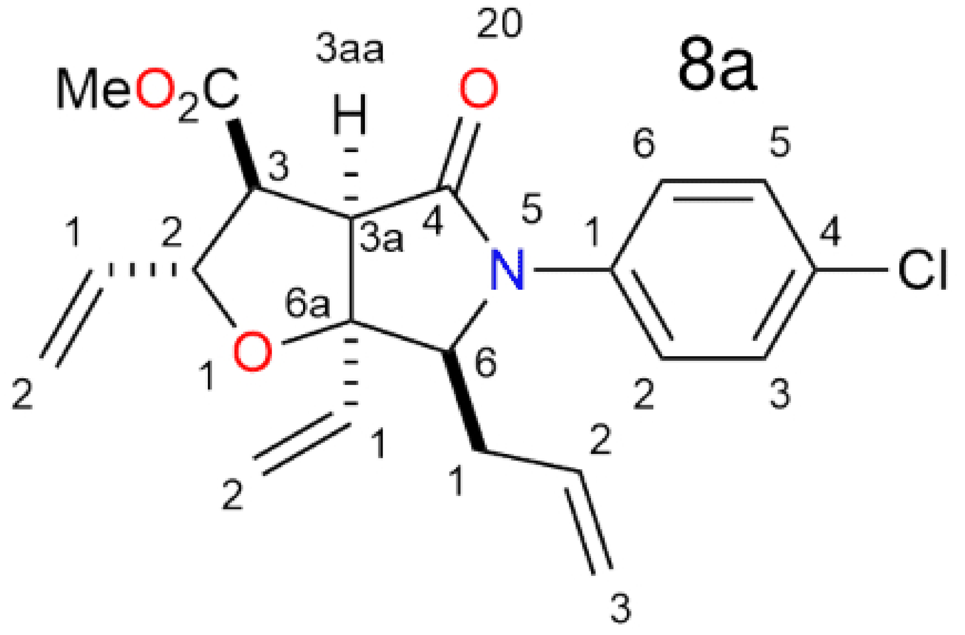

8a

FZ9208-2.esp

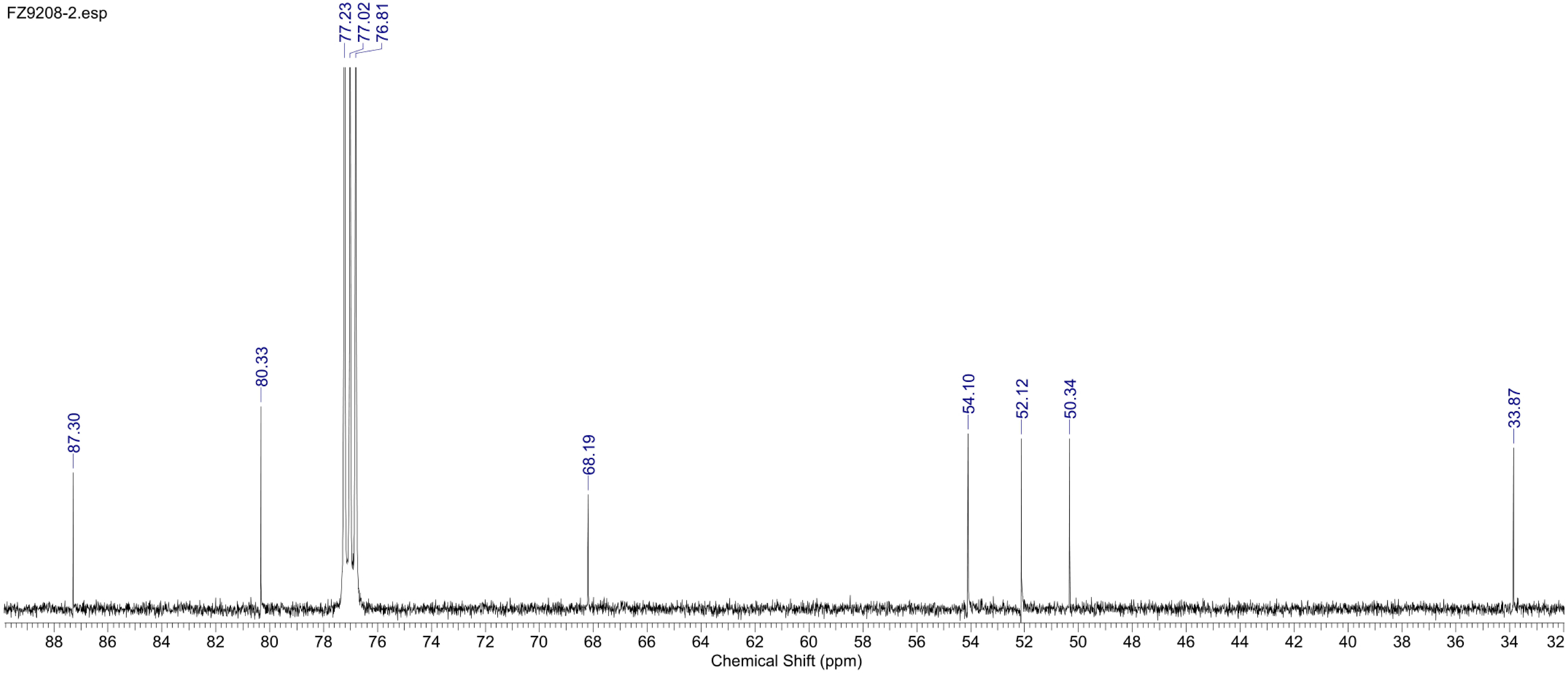

NMR spectrum data for compound 8b

Formula	$C_{21}H_{22}BrNO_4$	***FW***	432.3077

Acquisition Time (sec)	1.9818	***Comment***	single_pulse	***Date***	13 Oct 2020 12:10:52			***Date Stamp***	13 Oct 2020 12:10:22
File Name	C:\USERS\лаба 534\DOWNLOADS\FZ9177-1.JDF			***Frequency (MHz)***	600.17	***Nucleus***	1H	***Number of Transients***	8
Origin	ECA 600	***Original Points Count***	32768	***Owner***	CKP	***Points Count***	32768	***Pulse Sequence***	single_pulse.ex2
Receiver Gain	54.00	***Solvent***	CHLOROFORM-d			***Spectrum Offset (Hz)***	5401.5503	***Sweep Width (Hz)***	16534.39

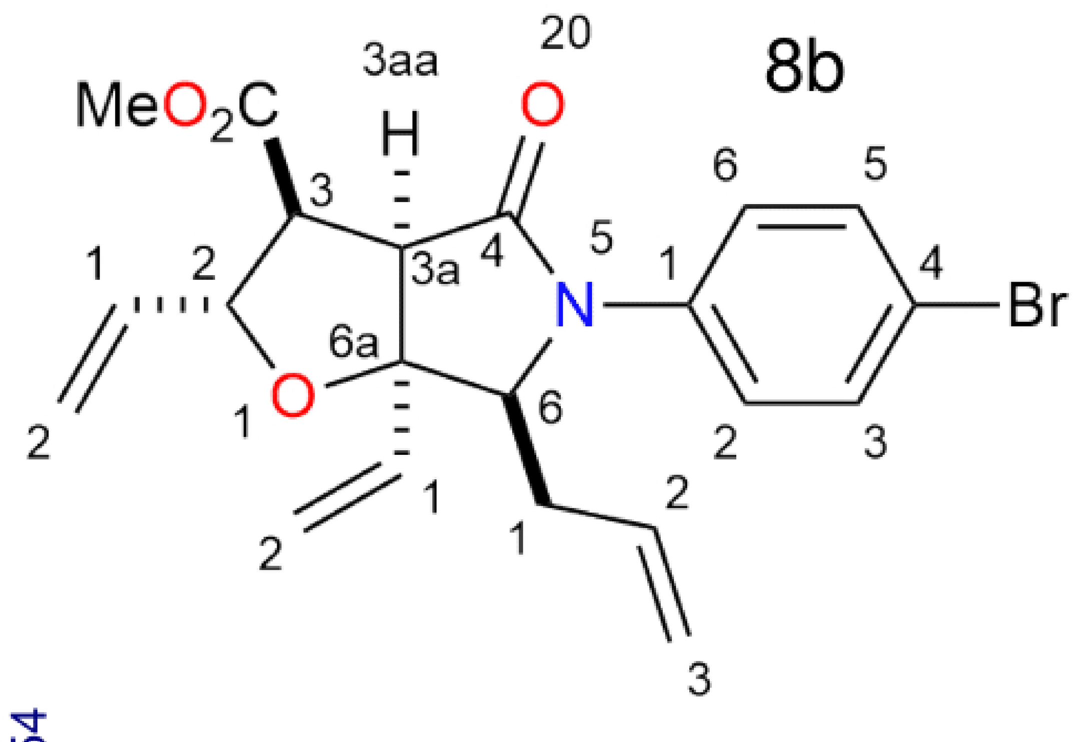

8b

FZ9177-1 вещество Саши от Ли.esp

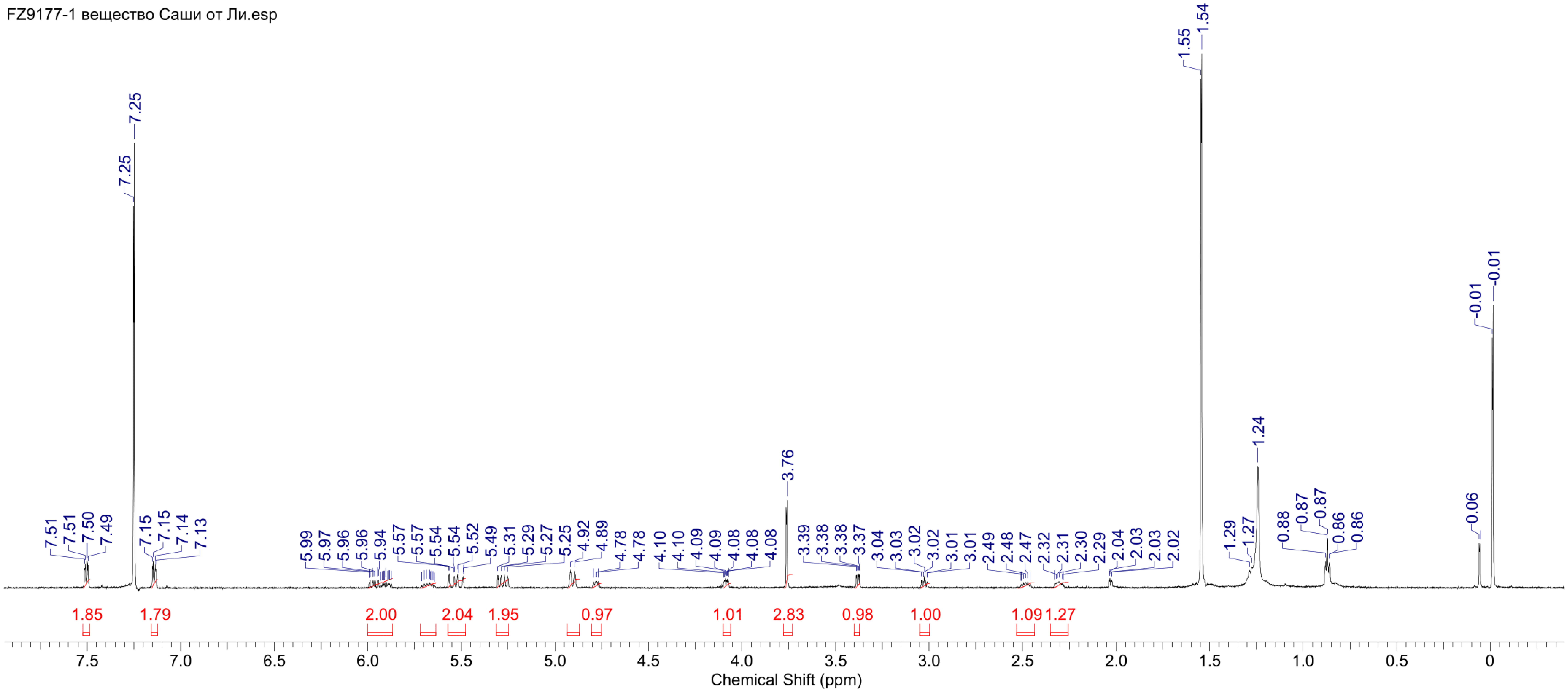

**Formula** $C_{21}H_{22}BrNO_4$	**FW** 432.3077

**Acquisition Time (sec)**	1.9818	**Comment**	single_pulse	**Date**	13 Oct 2020 12:10:52			**Date Stamp**	13 Oct 2020 12:10:22
**File Name**	C:\USERS\лаба 534\DOWNLOADS\FZ9177-1.JDF			**Frequency (MHz)**	600.17	**Nucleus**	1H	**Number of Transients**	8
**Origin**	ECA 600	**Original Points Count**	32768	**Owner**	CKP	**Points Count**	32768	**Pulse Sequence**	single_pulse.ex2
**Receiver Gain**	54.00	**Solvent**	CHLOROFORM-d			**Spectrum Offset (Hz)**	5401.5503	**Sweep Width (Hz)**	16534.39

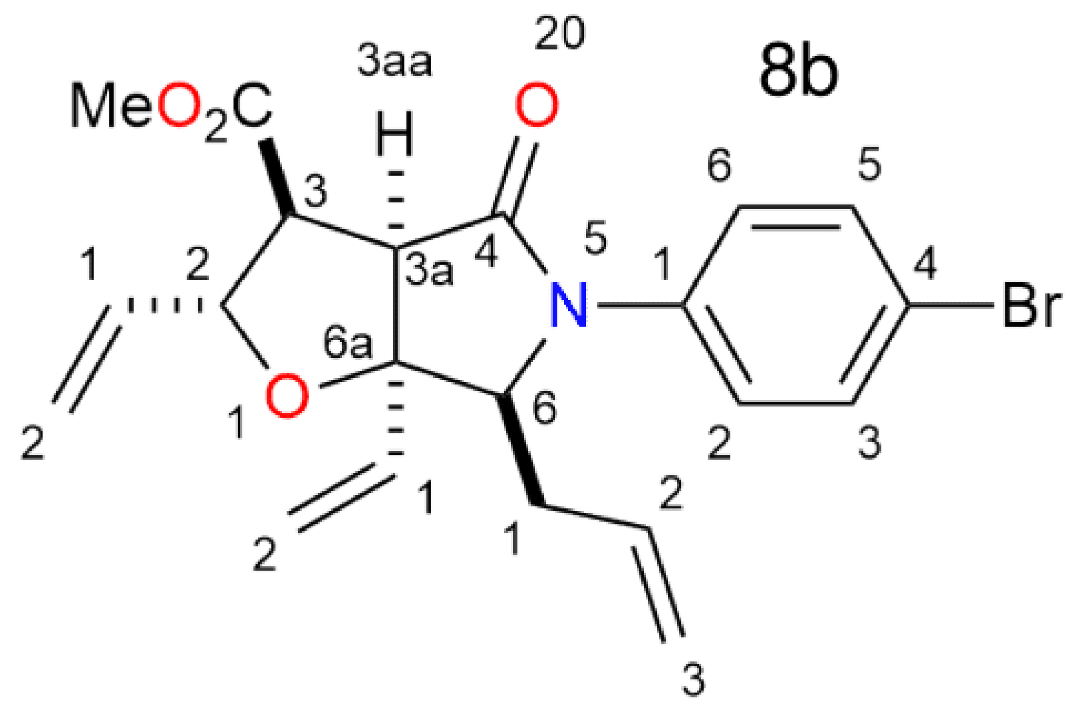

8b

FZ9177-1 вещество Саши от Ли.esp

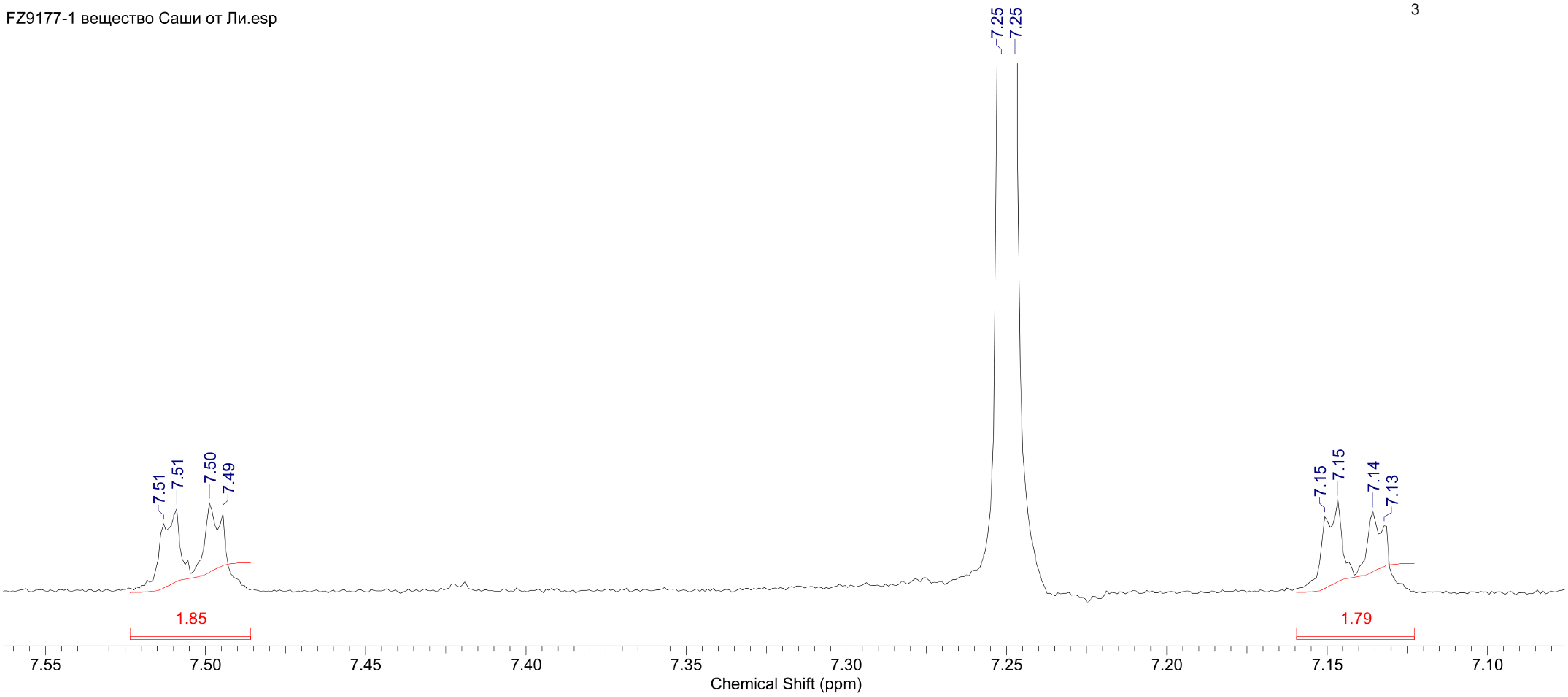

**Formula** $C_{21}H_{22}BrNO_4$	**FW** 432.3077

**Acquisition Time (sec)**	1.9818	**Comment**	single_pulse	**Date**	13 Oct 2020 12:10:52	**Date Stamp**	13 Oct 2020 12:10:22
**File Name**	C:\USERS\лаба 534\DOWNLOADS\FZ9177-1.JDF	**Frequency (MHz)**	600.17	**Nucleus**	1H	**Number of Transients**	8
**Origin**	ECA 600	**Original Points Count**	32768	**Owner**	CKP	**Points Count**	32768
**Pulse Sequence**	single_pulse.ex2	**Receiver Gain**	54.00	**Solvent**	CHLOROFORM-d	**Spectrum Offset (Hz)**	5401.5503
**Sweep Width (Hz)**	16534.39						

8b

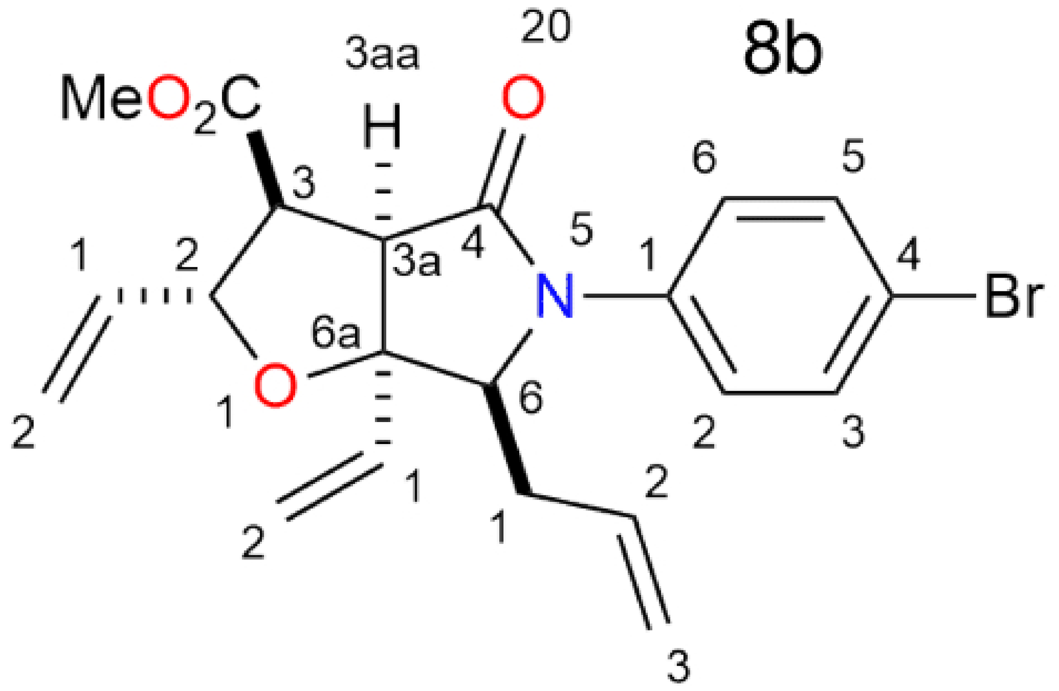

FZ9177-1 вещество Саши от Ли.esp

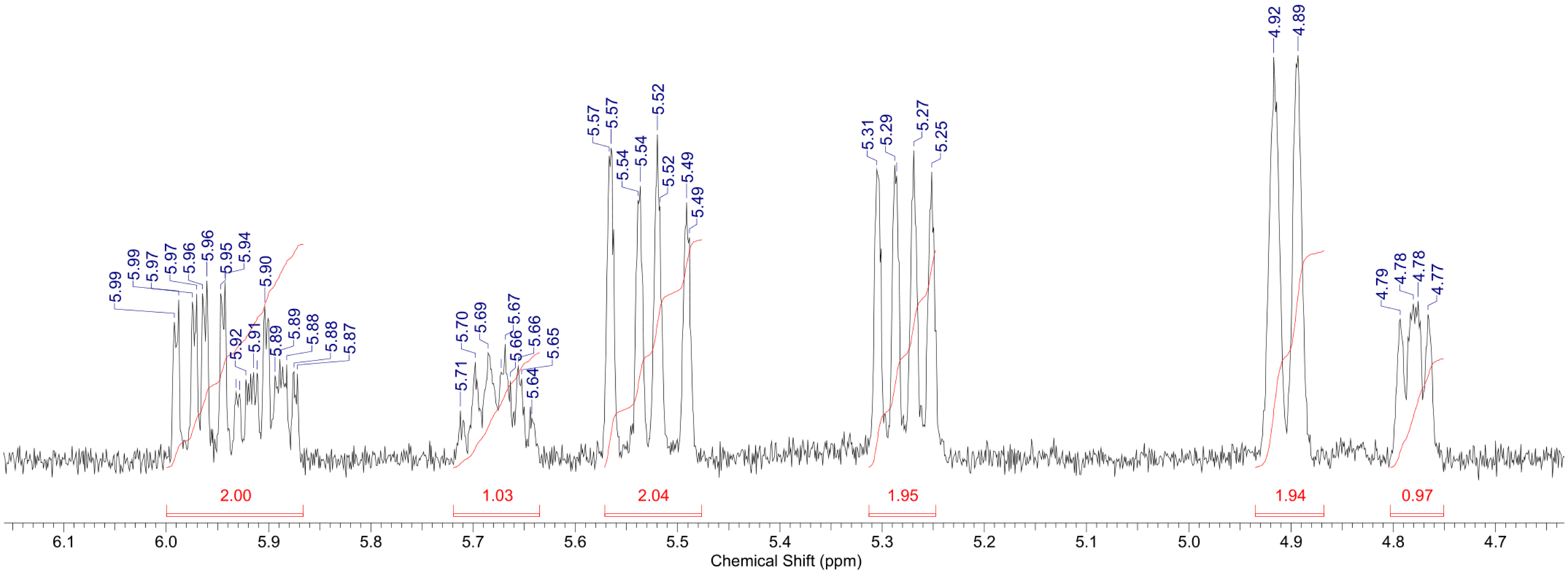

**Formula** $C_{21}H_{22}BrNO_4$	**FW** 432.3077

Acquisition Time (sec)	1.9818	***Comment***	single_pulse	***Date***	13 Oct 2020 12:10:52			***Date Stamp***	13 Oct 2020 12:10:22
File Name	C:\USERS\лаба 534\DOWNLOADS\FZ9177-1.JDF			***Frequency (MHz)***	600.17	***Nucleus***	1H	***Number of Transients***	8
Origin	ECA 600	***Original Points Count***	32768	***Owner***	CKP	***Points Count***	32768	***Pulse Sequence***	single_pulse.ex2
Receiver Gain	54.00	***Solvent***	CHLOROFORM-d			***Spectrum Offset (Hz)***	5401.5503	***Sweep Width (Hz)***	16534.39

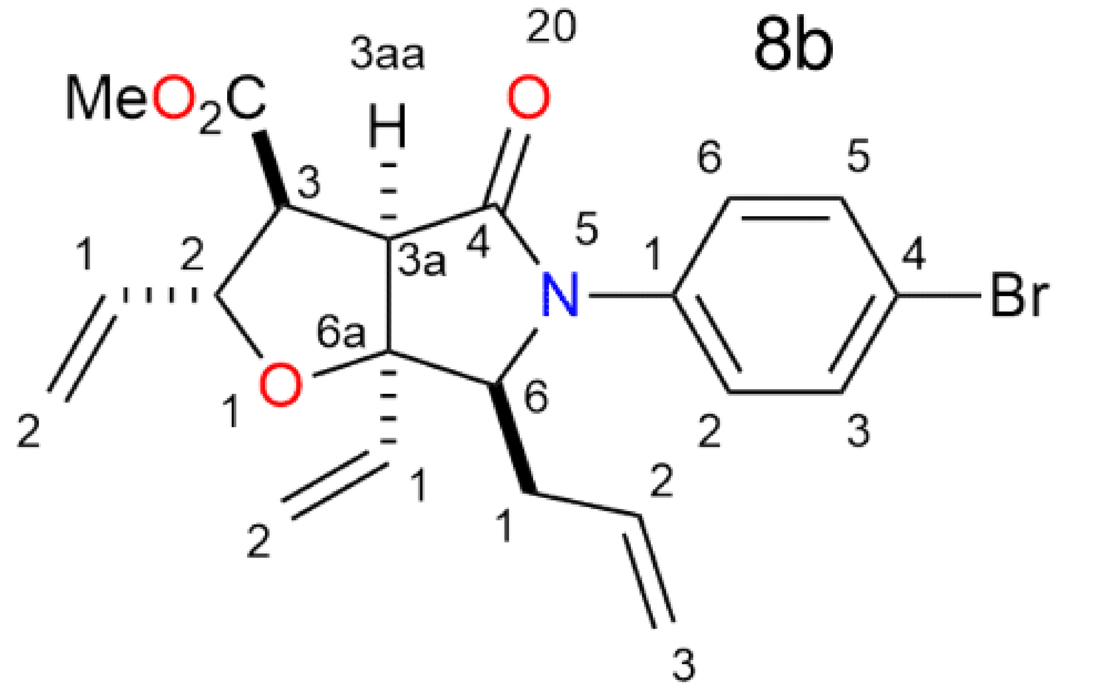

8b

FZ9177-1 вещество Саши от Ли.esp

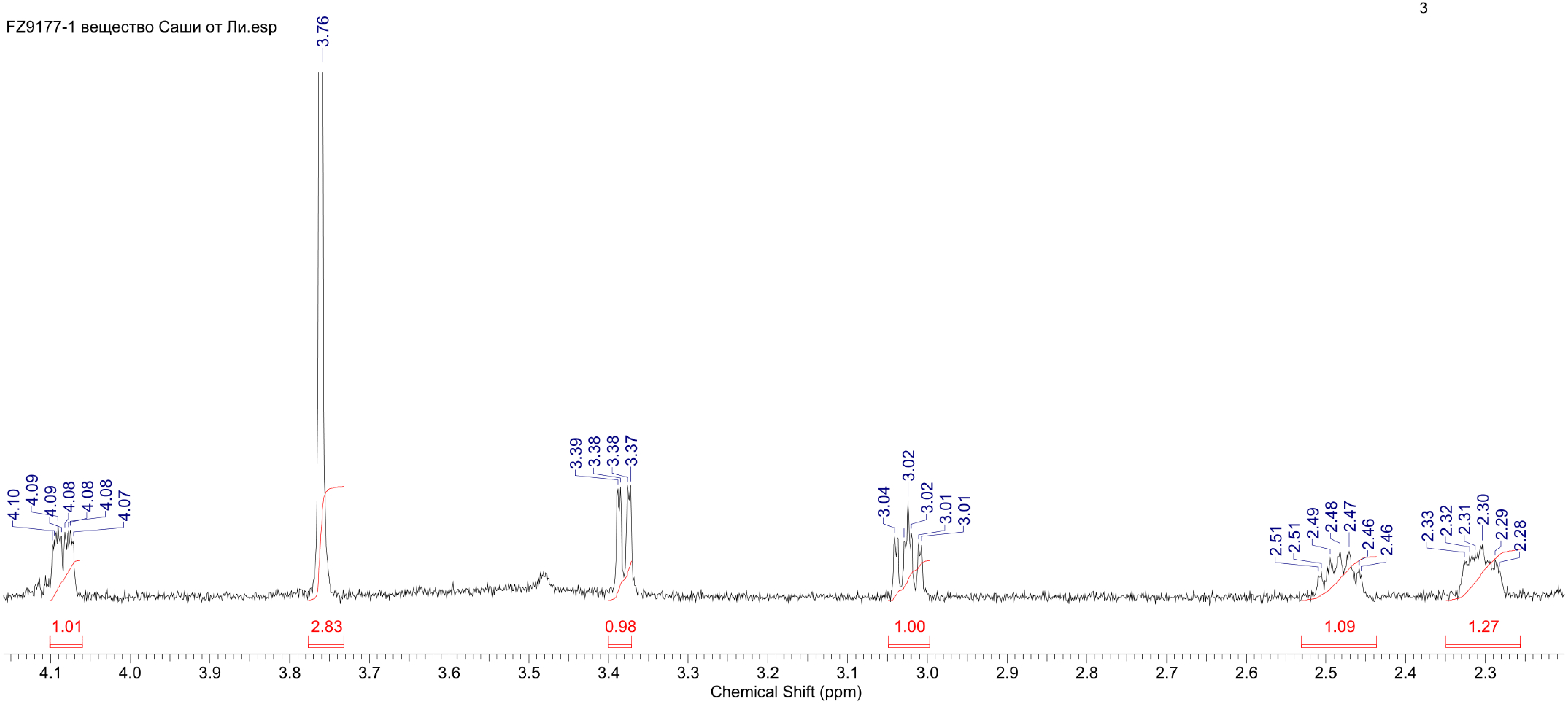

Formula	*FW*
$C_{21}H_{22}BrNO_4$	432.3077

Acquisition Time (sec)	0.6921	***Comment***	single pulse decoupled gated NOE			***Date***	15 Oct 2020 06:11:33
Date Stamp	15 Oct 2020 06:11:06			***File Name***	C:\USERS\лаба 534\DOWNLOADS\FZ9177-2.JDF		
Frequency (MHz)	150.91	***Nucleus***	13C	***Origin***	ECA 600	***Original Points Count***	32768
Owner	CKP	***Points Count***	32768	***Pulse Sequence***	single_pulse_dec	***Receiver Gain***	54.00
Solvent	CHLOROFORM-d			***Spectrum Offset (Hz)***	15080.7979	***Sweep Width (Hz)***	47348.49

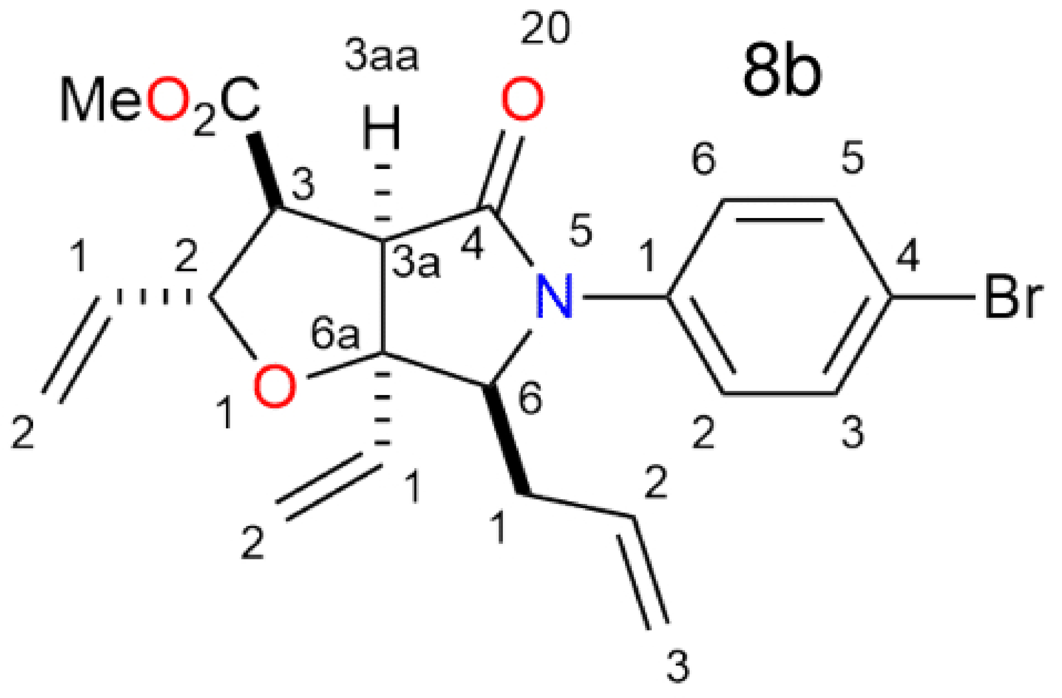

8b

FZ9177-2 вещество Саши от Ли углерод.esp

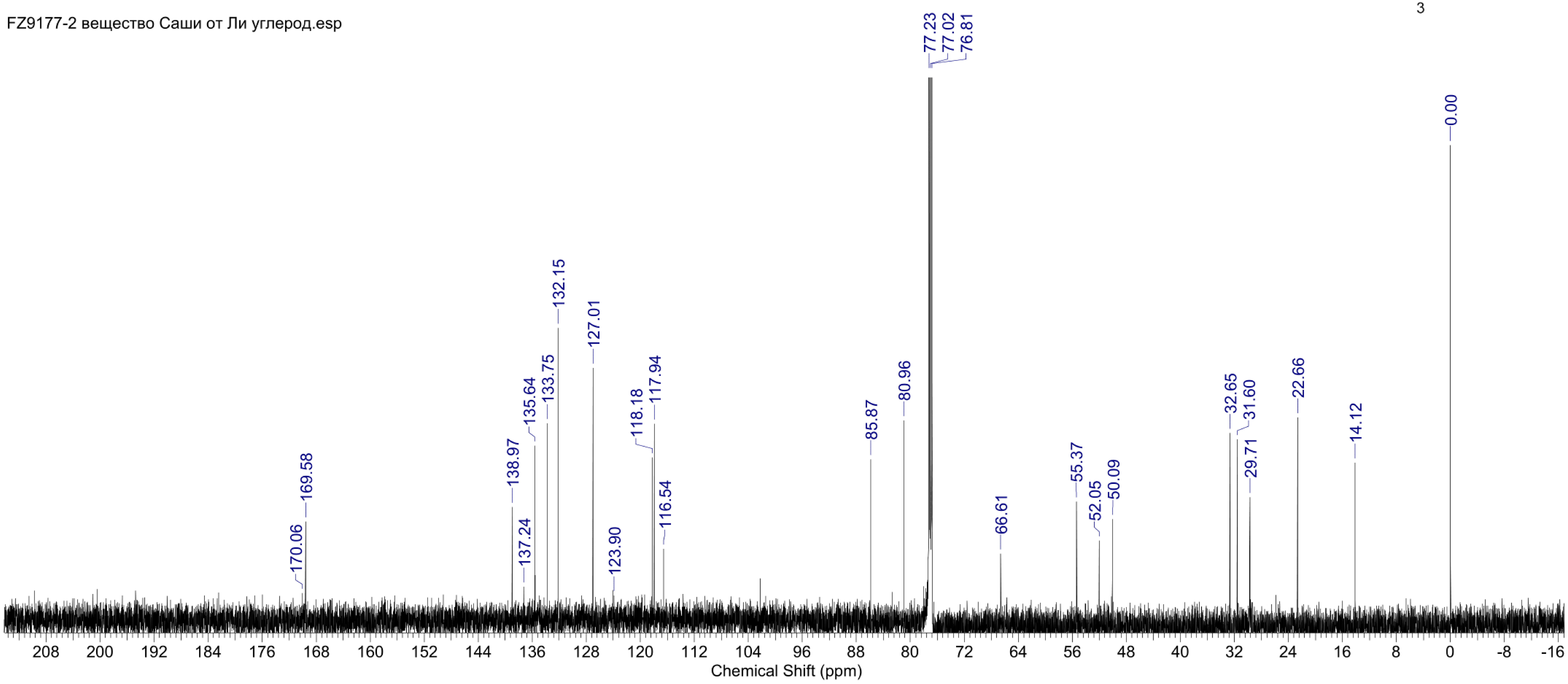

Formula	$C_{21}H_{22}BrNO_4$	FW	432.3077

**Acquisition Time (sec)**	0.6921	**Comment**	single pulse decoupled gated NOE			**Date**	15 Oct 2020 06:11:33
**Date Stamp**	15 Oct 2020 06:11:06			**File Name**	C:\USERS\лаба 534\DOWNLOADS\FZ9177-2.JDF		
**Frequency (MHz)**	150.91	**Nucleus**	13C	**Origin**	ECA 600	**Original Points Count**	32768
**Owner**	CKP	**Points Count**	32768	**Pulse Sequence**	single_pulse_dec	**Receiver Gain**	54.00
**Solvent**	CHLOROFORM-d			**Spectrum Offset (Hz)**	15080.7979	**Sweep Width (Hz)**	47348.49

8b

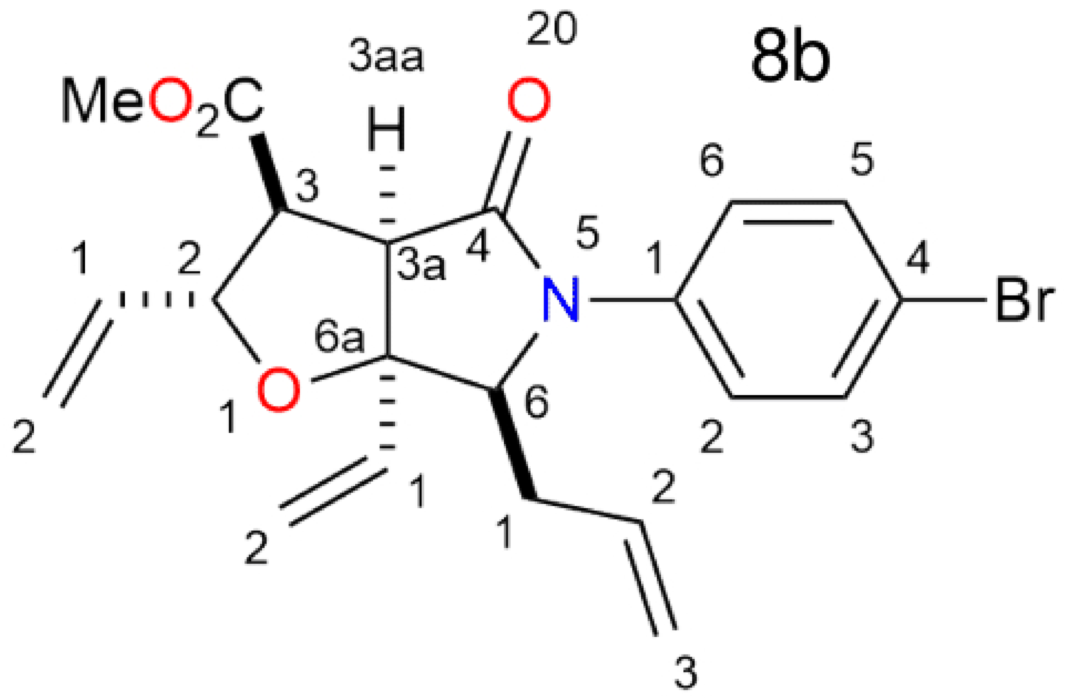

FZ9177-2 вещество Саши от Ли углерод.esp

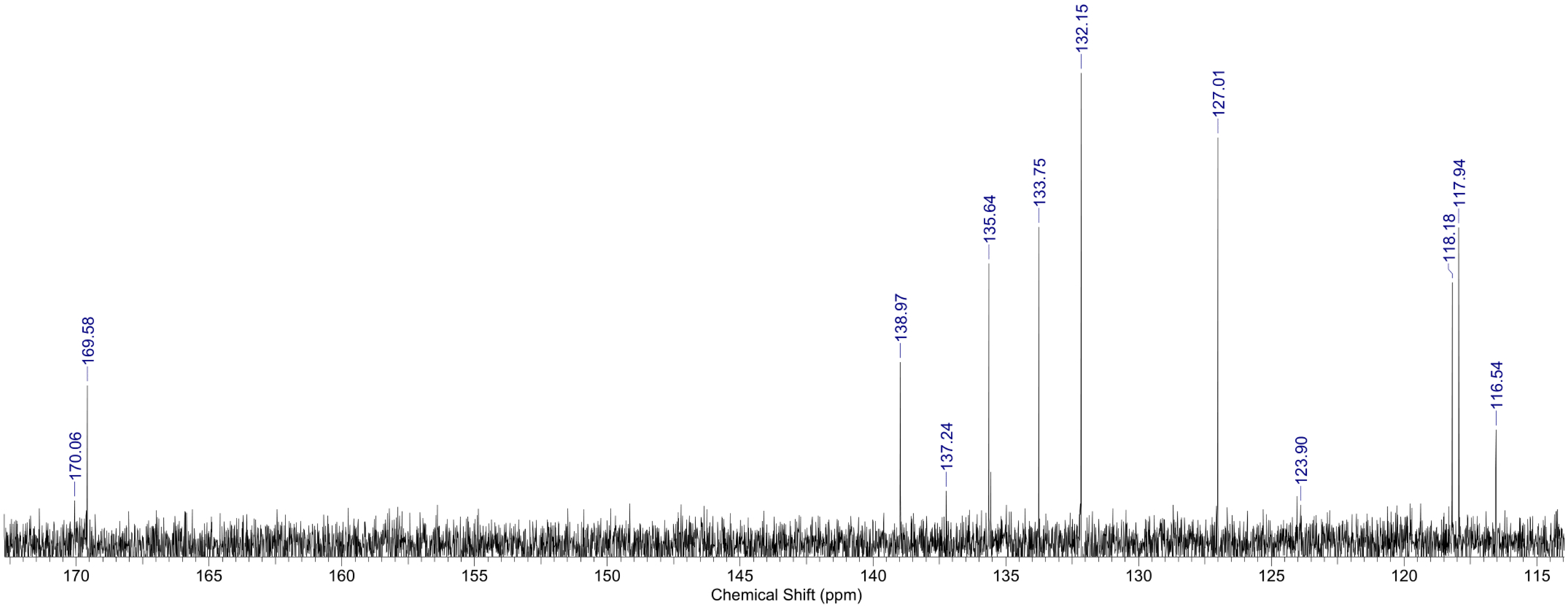

Formula	$C_{21}H_{22}BrNO_4$	FW	432.3077

Acquisition Time (sec)	0.6921	Comment	single pulse decoupled gated NOE	Date	15 Oct 2020 06:11:33
Date Stamp	15 Oct 2020 06:11:06	File Name	C:\USERS\лаба 534\DOWNLOADS\FZ9177-2.JDF		
Frequency (MHz)	150.91	Nucleus	13C	Origin	ECA 600
Original Points Count	32768	Owner	CKP	Points Count	32768
Pulse Sequence	single_pulse_dec	Receiver Gain	54.00	Solvent	CHLOROFORM-d
Spectrum Offset (Hz)	15080.7979	Sweep Width (Hz)	47348.49		

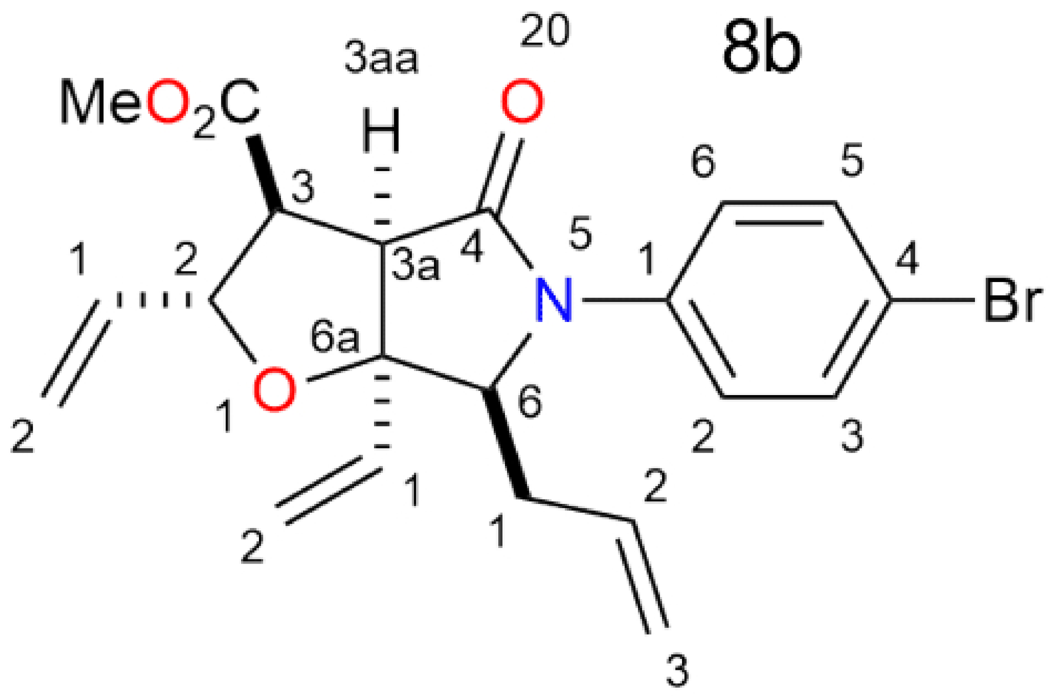

FZ9177-2 вещество Саши от Ли углерод.esp

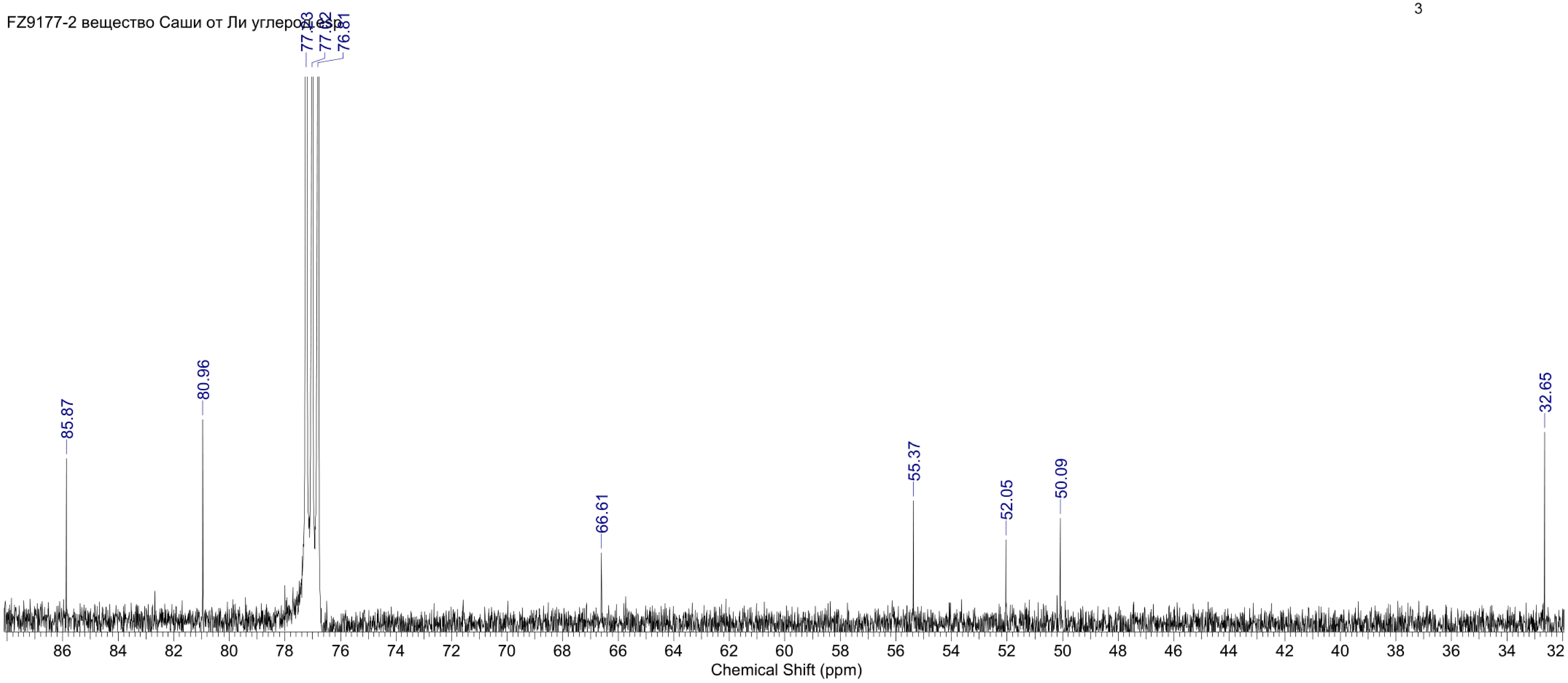

Supplement: Supplementary file 1 [file molecules-25-05379-s001.zip › o│opies of NMR spectra.pdf]
